# Whole-Genome Sequencing of Invasion-Resistant Cells Identifies Laminin α2 as a Host Factor for Bacterial Invasion

**DOI:** 10.1128/mBio.02128-16

**Published:** 2017-01-10

**Authors:** Xander M. van Wijk, Simon Döhrmann, Björn M. Hallström, Shangzhong Li, Bjørn G. Voldborg, Brandon X. Meng, Karen K. McKee, Toin H. van Kuppevelt, Peter D. Yurchenco, Bernhard O. Palsson, Nathan E. Lewis, Victor Nizet, Jeffrey D. Esko

**Affiliations:** a Department of Cellular and Molecular Medicine, University of California, San Diego, La Jolla, California, USA; b Department of Pediatrics, University of California, San Diego, La Jolla, California, USA; c Novo Nordisk Foundation Center for Biosustainability, Technical University of Denmark, Hørsholm, Denmark; d Royal Institute of Technology, Stockholm, Sweden; e Department of Pathology and Laboratory Medicine, Robert Wood Johnson Medical School, Piscataway, New Jersey, USA; f Department of Biochemistry, Radboud Institute for Molecular Life Sciences, Radboud University Medical Centre, Nijmegen, The Netherlands; g Department of Bioengineering, University of California, San Diego, La Jolla, California, USA; h Novo Nordisk Foundation Center for Biosustainability, University of California, San Diego, La Jolla, California, USA; Childrens Hospital, Boston; Harvard Medical School

## Abstract

To understand the role of glycosaminoglycans in bacterial cellular invasion, xylosyltransferase-deficient mutants of Chinese hamster ovary (CHO) cells were created using clustered regularly interspaced short palindromic repeat (CRISPR) and CRISPR-associated gene 9 (CRISPR-*cas9*) gene targeting. When these mutants were compared to the pgsA745 cell line, a CHO xylosyltransferase mutant generated previously using chemical mutagenesis, an unexpected result was obtained. Bacterial invasion of pgsA745 cells by group B Streptococcus (GBS), group A *Streptococcus*, and *Staphylococcus aureus* was markedly reduced compared to the invasion of wild-type cells, but newly generated CRISPR-*cas9* mutants were only resistant to GBS. Invasion of pgsA745 cells was not restored by transfection with xylosyltransferase, suggesting that an additional mutation conferring panresistance to multiple bacteria was present in pgsA745 cells. Whole-genome sequencing and transcriptome sequencing (RNA-Seq) uncovered a deletion in the gene encoding the laminin subunit α2 (*Lama2*) that eliminated much of domain L4a. Silencing of the long *Lama2* isoform in wild-type cells strongly reduced bacterial invasion, whereas transfection with human *LAMA2* cDNA significantly enhanced invasion in pgsA745 cells. The addition of exogenous laminin-α2β1γ1/laminin-α2β2γ1 strongly increased bacterial invasion in CHO cells, as well as in human alveolar basal epithelial and human brain microvascular endothelial cells. Thus, the L4a domain in laminin α2 is important for cellular invasion by a number of bacterial pathogens.

## IMPORTANCE

Pathogenic bacteria penetrate host cellular barriers by attachment to extracellular matrix molecules, such as proteoglycans, laminins, and collagens, leading to invasion of epithelial and endothelial cells. Here, we show that cellular invasion by the human pathogens group B *Streptococcus*, group A *Streptococcus*, and *Staphylococcus aureus* depends on a specific domain of the laminin α2 subunit. This finding may provide new leads for the molecular pathogenesis of these bacteria and the development of novel antimicrobial drugs.

## INTRODUCTION

Glycosaminoglycans (GAGs) are long, polyanionic polysaccharides present on the surface of virtually all animal cells and in the extracellular matrix. GAGs, and in particular heparan sulfate (HS) and chondroitin sulfate/dermatan sulfate (CS/DS), are involved in cellular adhesion and invasion by multiple pathogens (1). This long list of pathogens includes viruses like herpes simplex virus, human immunodeficiency virus, and hepatitis C virus and bacteria like *Listeria monocytogenes* and *Neisseria gonorrhoeae*. We reported previously a role for GAGs in endothelial cell invasion by group B *Streptococcus* (GBS) during its penetration of the blood-brain barrier (2).

The biosynthesis of HS and CS/DS starts with the formation of a linkage tetrasaccharide (xylose-galactose-galactose-glucuronic acid) attached to specific serine residues in a small number of proteoglycan core proteins. Chinese hamster ovary (CHO) cell mutants deficient in xylosyltransferase 2 (*Xylt2*), galactosyltransferase I (*β4galt7*), and glucuronosyltransferase I (*β*3*gat3*) were generated previously by chemical mutagenesis (3–5). The pgsA745 cell line harbors a nonsense mutation in *Xylt2* (6), completely lacks HS and CS/DS, and has been used by many laboratories to assess the role of GAGs in various processes, including adhesion and invasion by pathogens (7).

Genome editing has been simplified greatly by the introduction of the clustered regularly interspaced short palindromic repeat (CRISPR) and CRISPR-associated gene 9 (CRISPR-*cas9*) system (8). Here, we created new *Xylt2*-deficient CHO mutants using this system and examined invasion by multiple bacterial pathogens. A discrepancy in infectivity became apparent when comparing bacterial invasion in pgsA745 cells to that in the new *Xylt2*-deficient mutants. Whole-genome sequencing and transcriptome sequencing (RNA-Seq) revealed that pgsA745 cells also contain a deletion in the gene encoding laminin subunit α2 (*Lama2*), which diminished bacterial invasion. The deletion removes much of domain L4a in the laminin 2 subunit, demonstrating the importance of this region in invasion by multiple bacterial species.

## RESULTS

### Bacterial invasion in *XylT2* mutants generated by CRISPR-*cas9* and in pgsA745 cells differs.

**FIG 1:**
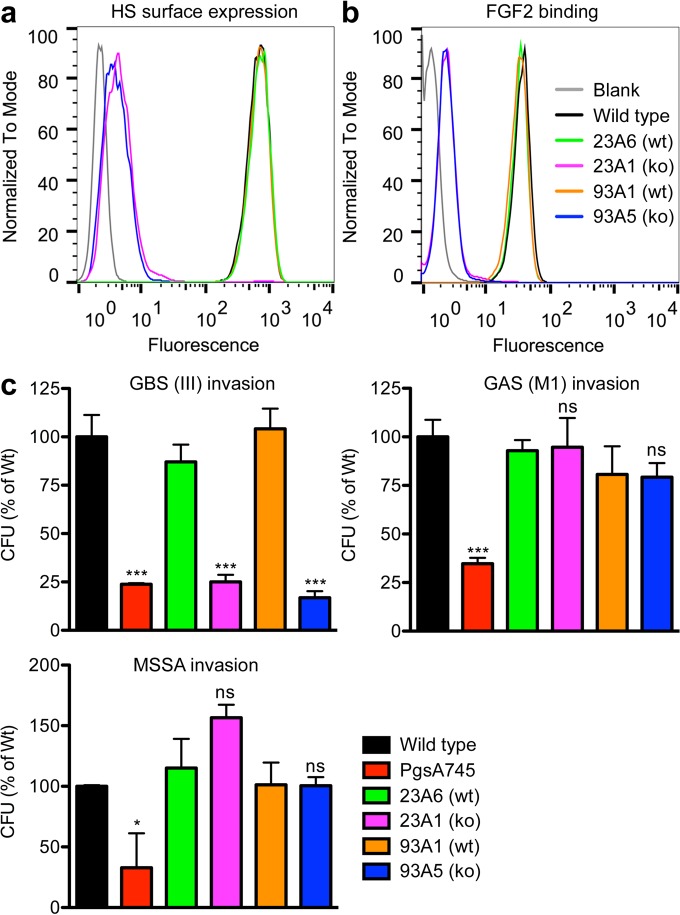
Resistance of pgsA745 cells to GAS and MSSA invasion does not correlate with lack of heparan sulfate expression. (a) Cell surface expression of heparan sulfate was reduced in *Xylt2* knockout clonal lines 23A1 and 93A5 generated by CRISPR-*cas9* targeting, as measured by flow cytometry using scFv antibody HS4C3. (b) Binding of biotinylated FGF2 to cell surface heparan sulfate was affected similarly in the mutants. (c) Invasion by GBS was reduced in pgsA745 cells and *Xylt* knockout clonal lines 23A1 and 93A5. In contrast, GAS and MSSA invasion was altered in pgsA745 cells but normal in 23A1 and 93A5 cells compared to the levels of invasion in control clones 23A6 and 93A1. *, *P* < 0.05, ***, *P* < 0.001, and ns, not significant versus results for wild-type cells using the two-tailed *t* test. Error bars indicate standard deviations (SD); *n* = 3.

Bacterial invasion of cells contributes to penetration of host barriers, a hallmark of pathogenicity, and provides an intracellular niche for bacterial survival and proliferation. To examine the role of GAGs in bacterial invasion, we inactivated *Xylt2* in CHO-K1 cells using the CRISPR-*cas9* system. Sequencing showed *Xylt2* frameshift mutations in clonal lines 23A1 and 93A5, respectively, but not in control clonal lines 23A6 and 93A1 isolated from the same targeted cell pool (see Fig. S1 in the supplemental material). Inactivation of *Xylt2* markedly reduced cell surface expression of HS as determined by flow cytometry using the single-chain variable-fragment (scFv) antibody HS4C3 (Fig. 1a) and by the binding of an HS-dependent growth factor, fibroblast growth factor 2 (FGF2) (Fig. 1b). Invasion of GBS was much lower in the *Xylt2* mutants (Fig. 1c), in agreement with previous studies of mutant pgsA745 cells (9), which also carry a loss-of-function allele of *Xylt2* (6). Group A Streptococcus (GAS) and *Staphylococcus aureus* can also bind to GAGs (10, 11), but their invasion was not compromised in the new *XylT*2 knockouts, suggesting that interaction with GAGs is not required for invasion (Fig. 1c). In contrast, invasion by all three pathogens was clearly reduced in strain pgsA745 (Fig. 1c), but there was no difference in invasion by methicillin-resistant *S. aureus* (MRSA) in wild-type and pgsA745 cells or CRISPR-*cas9* control and knockout cells (data not shown). Stable transfection of pgsA745 cells with *Xylt1* or *Xylt2* cDNAs restored cell surface expression of HS (see Fig. S2a) but did not restore bacterial invasion (Table 1; see also Fig. S2b). Based on the resistance of XylT2 mutants derived by CRISPR-*cas9*, we concluded that GAGs are necessary for invasion by GBS but not by GAS or *S. aureus*. This is consistent with our previous observation that HS is important for the invasion of GBS in brain microvascular endothelial cells (2). The lack of restoration of GBS invasion in pgsA745 cells by transfection with xylosyltransferase led us to conclude that pgsA745 cells harbor an additional defect that alters susceptibility to infection by multiple bacterial species.

10.1128/mBio.02128-16.2 FIG S1  Sequencing results for CRISPR-*cas9*-generated *Xylt2* knockout clones. Sequences are shown for mutant clones 23A1 and 93A5 and wild-type control clones 23A6 and 93A1. The CRISPR-*cas9* target sequence is shown in boldface. Insertions and deletions are highlighted in yellow. Note that the inserted DNA sequence in 93A5 is part of the *cas9* expression vector that was used. No mutations in the analyzed *Xylt2* region were found for the control clones. Download FIG S1, PDF file, 0.02 MB. Copyright © 2017 van Wijk et al. 2017 van Wijk et al. This content is distributed under the terms of the Creative Commons Attribution 4.0 International license.

10.1128/mBio.02128-16.3 FIG S2  Restoration of glycosaminoglycan biosynthesis by xylosyltransferase does not restore invasion of GBS. (a) HS cell surface expression as measured by flow cytometry using scFv antibody HS4C3. Bimodal distribution of pgsA745-Xylt2 cells was reported previously (6). (b) GBS invasion of wild-type, pgsA745, and Xylt1- or Xylt2-corrected pgsA745 cells, as measured by the antibiotic protection assay. ***, *P* < 0.001 versus the results for wild-type cells using the two-tailed *t* test. (c) Analysis of FITC-labeled GBS invasion using flow cytometry (~10^6^ CFU per well in a 24-well plate). An arbitrary uptake index (UI) was obtained by multiplying the percentage of FITC-positive cells by the mean fluorescence intensity (MFI) of these cells, as described elsewhere (44). (d) Uptake of heat-killed GBS analyzed using flow cytometry, as described for panel c. Download FIG S2, TIF file, 1.2 MB. Copyright © 2017 van Wijk et al. 2017 van Wijk et al. This content is distributed under the terms of the Creative Commons Attribution 4.0 International license.

**TABLE 1:** Xylosyltransferase transfection does not restore bacterial invasion

Bacterium (serotype)	Mean CFU ± SD (%)^a^					
	Wild-type cells		pgsA745 cells		pgsA745-XylT cells	
	Adhesion	Invasion	Adhesion	Invasion	Adhesion	Invasion
GBS (III)	100 ± 31	100 ± 24	100 ± 36	22 ± 2^b^	122 ± 30	17 ± 3^b^
GBS (Ia)	100 ± 23	100 ± 19	80 ± 20	28 ± 12^c^	107 ± 12	22 ± 14^c^
GAS (M1T1)	100 ± 19	100 ± 14	109 ± 10	16 ± 5^c^	98 ± 16	16 ± 1^c^
MSSA	100 ± 29	100 ± 6	104 ± 25	27 ± 8^c^	99 ± 20	28 ± 9^c^

a Percentage of the results for wild-type cells; *n* = 4 to 6.

b *P* < 0.001 versus wild type (two-tailed t test).

c *P* < 0.0001 versus Wild type (two-tailed *t* test).

### Adhesion and endocytosis is normal in pgs745 cells.

**FIG 2:**
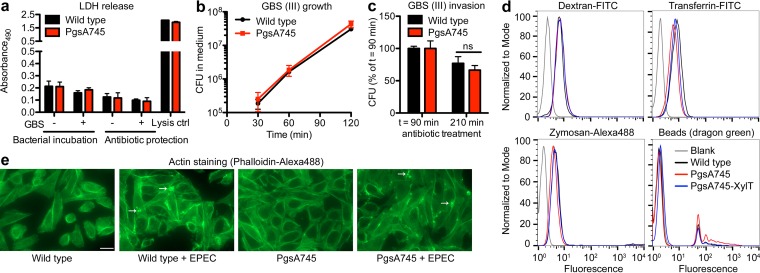
Viability, bacterial growth rates, endocytosis/phagocytosis, and actin relocalization are normal in pgsA745 cells. (a) LDH release was not affected by infection or treatment with antibiotics in pgsA745 cells. Error bars indicate SD; *n* = 4. (b) Bacterial growth rate in pgsA745 cells was unaffected. Error bars indicate SD; *n* = 3. (c) Intracellular bacterial growth/survival was unaltered in pgsA745 cells as measured by CFU count after 90 and 210 min of antibiotic protection. Bacterial counts at *t* = 90 min were set to 100%. Error bars indicate SD; *n* = 3. (d) Endocytosis and phagocytosis of fluorochrome-labeled dextran, transferrin, zymosan, or beads were unaffected in pgsA745 cells as measured by flow cytometry. (e) Actin relocalization probed using EPEC infection was unaltered in pgsA745 cells. Arrows point to typical actin “pedestals” induced by EPEC. Scale bar = 20 μm.

The first event in bacterial invasion of cells requires attachment of the bacteria to adhesins on host cells. Adhesion was unaffected in pgsA745 cells (Table 1) before and after transfection with *Xylt*, suggesting that bacterial resistance was due to a downstream factor involved in bacterial entry. A higher susceptibly of pgsA745 cells to cell death due to infection could falsely lower bacterial invasion levels, as the assay relies on the recovery of intracellular bacteria protected from membrane-impermeant antibiotics. However, the release of lactate dehydrogenase (LDH), a marker of cell lysis, did not differ between wild-type and pgsA745 cells, before or after infection and in the presence or absence of antibiotics (Fig. 2a). In addition, an alternative bacterial invasion assay based on flow cytometry also showed reduced bacterial invasion in pgsA745 cells with either live (see Fig. S2c in the supplemental material) or heat-killed GBS, indicating a cell-autonomous defect in the host (see Fig. S2d). pgsA745 and wild-type cells also did not differ in extracellular and intracellular bacterial growth/survival (Fig. 2b and c). A general defect of pgsA745 cells in endocytosis/phagocytosis was also excluded, as uptake of fluorochrome-labeled markers of macropinocytosis (dextran), clathrin-mediated endocytosis (transferrin), and phagocytosis (yeast cell wall zymosan and latex beads) was unaffected (Fig. 2d). To assess actin remodeling, a final step in bacterial invasion (12), we infected the cells with enteropathogenic *Escherichia coli* (EPEC), which causes easily distinguishable actin pedestals (13). Similar numbers of actin pedestals were observed in response to EPEC infection of both wild-type and pgsA745 cells (Fig. 2e). Bacteria also exploit integrins for host invasion (14), but cell surface integrin expression as determined by flow cytometry appeared normal (see Fig. S3), with the exception of integrin α6 and α7 expression, which depended on GAG expression (i.e., integrin α6 and α7 expression is lost in pgsA745 cells but is restored in pgsA745-XylT cells). The reexpression of these integrins in pgsA745-XylT cells did not restore susceptibility to infection, indicating that these integrins do not play a role in infection.

10.1128/mBio.02128-16.4 FIG S3  Cell surface expression of various integrins and α-dystroglycan (α-DG). Surface expression was measured by flow cytometry. With the exception of α6 and α7 integrins, there was no clear difference in integrin/α-DG expression between wild-type and pgsA745 cells. Expression of α6 and α7 was dependent on glycosaminoglycan expression, based on the observation that expression of these integrins was restored in pgsA745-Xylt cells. α-DG expression was examined using two antibodies (IIH6C4 and V IA4.1). Download FIG S3, TIF file, 1.6 MB. Copyright © 2017 van Wijk et al. 2017 van Wijk et al. This content is distributed under the terms of the Creative Commons Attribution 4.0 International license.

### pgsA745 cells contain a large deletion in *Lama2*.

**FIG 3:**
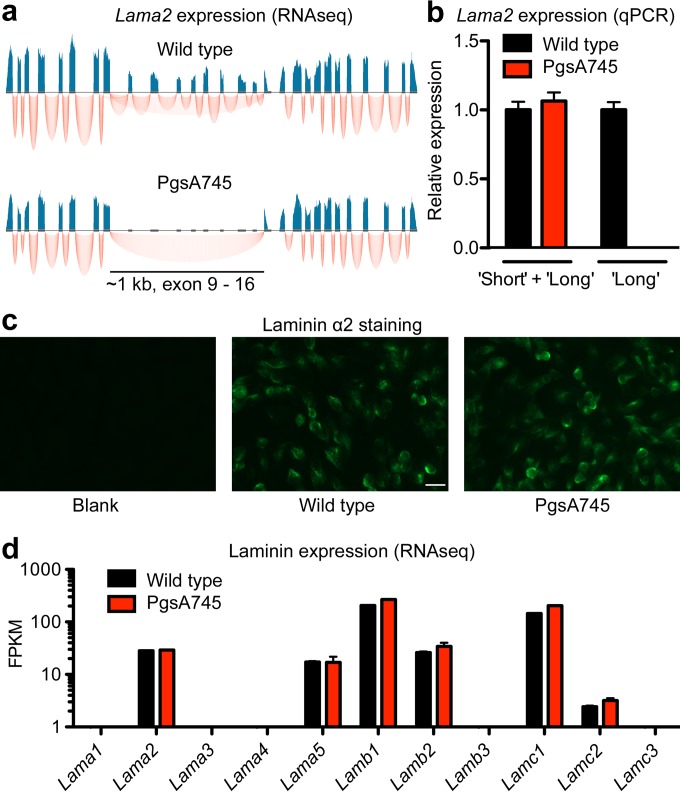
RNA-Seq reveals a loss of 8 exons in *Lama2* in pgsA745 cells. (a) RNA-Seq profile of *Lama2* showing exon reads in blue and exon-intron junctions in red. Note that only the first 28 of 61 exons are shown. (b) pgsA745 cells express wild-type amounts of total (short + long) *Lama2* transcripts but lack expression of long *Lama2* transcripts. Error bars indicate SD; *n* = 3. (c) Immunocytochemistry using antibody 5H2 shows laminin α2 expression in both wild-type and pgsA745 cells. Scale bar = 20 μm. (d) Expression of the different laminin α (*Lama*), β (*Lamb*), and γ (*Lamc*) units as expressed in fragments per kilobase of transcript per million mapped reads (FPKM) measured by RNA-Seq. Error bars indicate SD; *n* = 2.

To identify the cause of reduced bacterial invasion in pgsA745 cells, we sequenced the entire genome and compared it to the sequence of a reference wild-type CHO genome. We also analyzed the entire transcriptome of pgsA745 cells by RNA-Seq and compared it to the transcriptomes of wild-type and pgsA745-XylT cells (see Data set S1 in the supplemental material). Numerous heterozygous mutations were present, most likely due to chemical mutagenesis and accumulation of mutations over time between the reference genome and the mutant, but none of these resulted in genetic changes deemed likely to affect infection. However, a large deletion in the gene encoding the extracellular matrix protein laminin subunit α2 (*Lama2*) was identified by both methods. The deleted region consisted of 60 kbp at the genomic level, covering exons 9 to 16 (Fig. 3a; note that only the first 28 of 61 exons are shown). This genomic deletion resulted in a corresponding loss of 1,074 bases (out of 9,558 bp) in the mRNA and 358 amino acids in a region that includes domains L4a and LEb of the laminin α2 chain (15). Interestingly, the number of RNA reads in the RNA-Seq data was reduced by approximately 50% in this region in the wild-type cells compared to the number of reads for other exons (Fig. 3a). Therefore, we concluded that parental CHO-K1 cells are heterozygous with respect to the deletion, or short allele, and heterozygosity was lost in pgsA745 cells. In other words, parental cells contain a long and a short form of *Lama2*, whereas pgsA745 cells contain only the short form. By calculating the read depth and percentage of homozygosity for all DNA scaffolds of >100 kbp and by comparing the results to those of six other CHO genomes (16), including CHO-K1, we found that 45 of approximately 300 scaffolds with >80% homozygosity appeared to be uniquely monosomic in pgsA745 cells. All 45 scaffolds mapped to either chromosome 2 or X of the Chinese hamster genome, suggesting partial monosomy of chromosome 2 and X for pgsA745.

10.1128/mBio.02128-16.5 DATA SET S1  RNA-Seq data analysis of wild-type, pgsA745, and pgsA745-XylT cells. Download DATA SET S1, PDF file, 6.5 MB. Copyright © 2017 van Wijk et al. 2017 van Wijk et al. This content is distributed under the terms of the Creative Commons Attribution 4.0 International license.

To confirm the *Lama2* deletion in pgsA745, we developed quantitative PCR (qPCR) primers specifically recognizing the long form of *Lama2* and primers recognizing both the short and the long form. As expected, we did not observe expression of the long form in pgsA745 cells (Fig. 3b). Nevertheless, the short form of laminin α2 protein was expressed normally in the mutant, and immunocytochemistry showed extracellular localization similar to that in wild-type cells (Fig. 3c).

### Laminin α2 is important for bacterial invasion.

**FIG 4:**
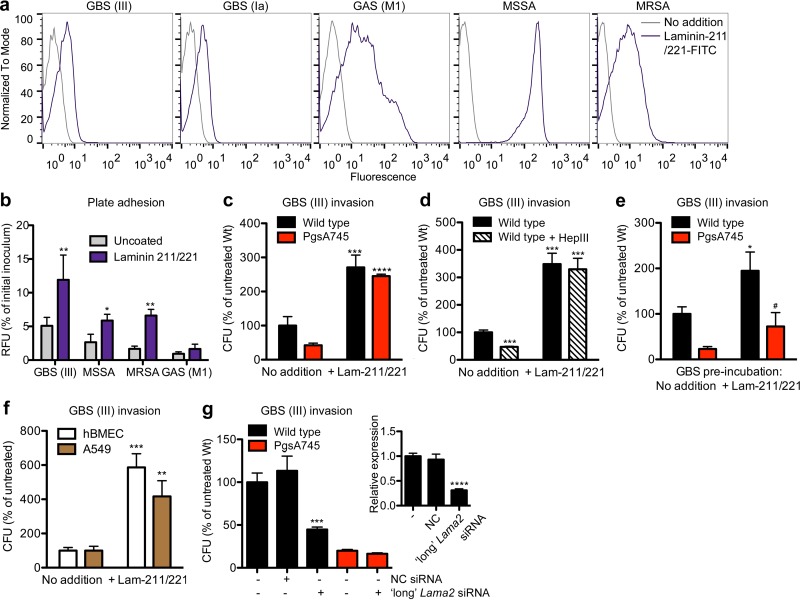
Laminin-211/221 facilitates bacterial invasion. (a) Flow cytometry results show that FITC-labeled laminin-211/221 binds to different bacteria. (b) FITC-labeled bacteria adhere to laminin-211/221-coated plates. RFU, relative fluorescent units. *, *P* < 0.05, and **, *P* < 0.01 versus the results for uncoated plates using a two-tailed *t* test. Error bars indicate SD; *n* = 3 to 6. (c and d) Bacterial invasion is strongly enhanced in the presence of 5-μg/ml laminin-211/221 in wild-type and pgsA745 cells (c), and this increase is independent of removal of HS by heparinase III (HepIII) (d). Removal of HS by heparinase III (HepIII) in wild-type cells reduced bacterial invasion without addition of laminin-211/221. (c) ***, *P* < 0.001, and ****, *P* < 0.0001 versus the results for no addition of laminin-211/221 using the two-tailed *t* test. (d) ***, *P* < 0.001 versus the results for untreated wild-type cells using a two-tailed *t* test. Error bars indicate SD; *n* = 3 or 4. (e) Preincubation of GBS with 50-μg/ml laminin-211/221 enhanced invasion in wild-type and pgsA745 cells. *, *P* < 0.05, and #, *P* = 0.05 versus the results for no addition of laminin-211/221 using the two-tailed *t* test. Error bars indicate SD; *n* = 3. (f) Bacterial invasion is strongly enhanced in the presence of 10-μg/ml laminin-211/221 in human brain microvascular endothelial cells (hBMEC) and human alveolar epithelial cells (A549). **, *P* < 0.01, and ***, *P* < 0.001 versus the results for no addition of laminin-211/221 using the two-tailed *t* test. Error bars indicate SD; *n* = 3. (g) siRNA knockdown of the expression of long *Lama2* reduces bacterial invasion. Inset, silencing of long *Lama2* by siRNA reduces expression of long *Lama2* transcripts relative to their expression when using negative-control siRNA (NC) or the buffer control (leftmost bar). ***, *P* < 0.001, and ****, *P* < 0.0001 versus the results for buffer-treated cells using the two-tailed *t* test. Error bars indicate SD; *n* = 3.

Laminin consists of a trimer of different isoforms of α, β, and γ subunits. Inspection of the expression data from the RNA-Seq analysis showed that CHO cells express primarily subunits α2, α5, β1, β2, γ1, and to a lesser extent, γ2 (Fig. 3d). To assess whether bacteria bind to laminin, we mixed fluorescently labeled laminin-211/221 (laminin-α2β1γ1/laminin-α2β2γ1) with different bacteria: two serotypes of GBS, GAS, *S. aureus* (methicillin-sensitive *S. aureus* [MSSA] and MRSA). Binding was measured using flow cytometry gated on the bacteria. In all cases, there was a clear shift in fluorescence after the addition of the labeled laminin, indicating laminin binding (Fig. 4a). In the second method, we noted significantly enhanced binding of bacteria to a microtiter plate coated with laminin-211/221 (Fig. 4b). The addition of laminin-211/221 to a cellular assay also strongly enhanced the invasion of GBS in both wild-type and pgsA745 cells (Fig. 4c). This increase was independent of HS, since the removal of HS by heparinase III in the presence of laminin-211/221 had no effect on invasion (Fig. 4d). In the absence of added laminin-211/221, treatment with heparinase III reduced GBS invasion, consistent with our observation in CRISPR-*cas9* mutants (Fig. 1c). Finally, preincubation of bacteria with laminin-211/221, followed by washing to remove unbound laminin, increased invasion as well (Fig. 4e). Laminin-211/221 also enhanced the invasion of GBS in human brain microvascular endothelial cells (hBMEC) and alveolar basal epithelial cells (A549) (Fig. 4f), both of which are susceptible to GBS invasion during experimental infection *in vitro* and *in vivo* (17).

### Long Lama2 isoform is important for bacterial invasion.

**FIG 5:**
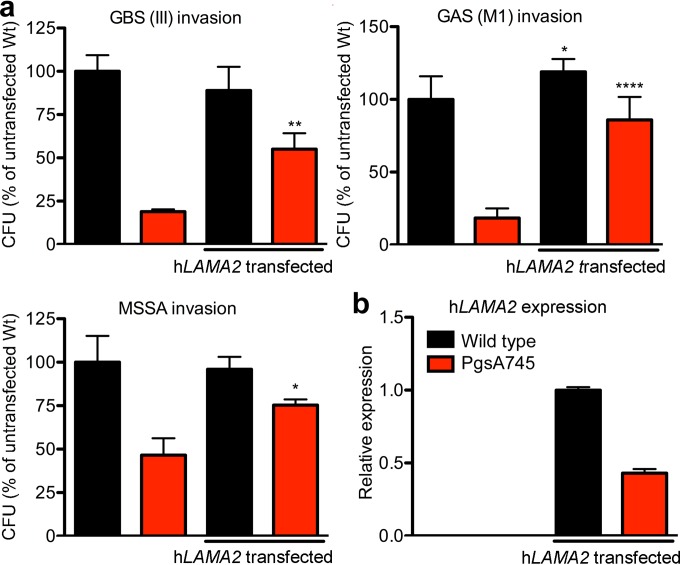
Transfection of pgsA745 cells with human *LAMA2* enhances bacterial invasion. (a) Bacterial invasion was restored in pgsA745 cells after transfection with human *LAMA2* cDNA. Untransfected, left 2 bars; transfected, right 2 bars. *, *P* < 0.05, **, *P* < 0.01, and ****, *P* < 0.0001 versus the results for untransfected cells using the two-tailed *t* test. Error bars indicate SD; *n* = 2 to 6. (b) Relative expression of human *LAMA2* determined by qPCR with primers specific for the human form of *LAMA2*, amplifying a region corresponding to CHO long *Lama2*. Error bars indicate SD; *n* = 3. Note that human *LAMA2* was not detected in untransfected cells.

To investigate the effect of the long *Lama2* isoform in bacterial invasion, we specifically reduced its expression by using small interfering RNA (siRNA) directed to the long sequence (Fig. 4g, inset). Knockdown of the long form of *Lama2* significantly decreased bacterial invasion in wild-type cells but not in pgsA745 cells, as they lack the long form (Fig. 4g). Finally, transfection of pgsA745 cells with the human form of full-length *LAMA2* (h*LAMA2*) cDNA significantly increased the invasion of GBS, GAS, and *S. aureus*, whereas transfection of wild-type cells had little if any effect (Fig. 5a). PCR analysis using primers specific for the human form of *LAMA2* showed robust expression in both mutant and wild-type cells (Fig. 5b).

## DISCUSSION

Laminins are major components of basement membranes, a specialized extracellular matrix that underlies endothelium and epithelium (15). Different laminins are composed of different combinations of α, β, and γ subunits. Although theoretically more than 50 heterotrimers can exist, only a third of the possible combinations have been described (18). Laminin subunit α2 is found as part of laminin-211 (laminin-α2β1γ1), laminin-221 (laminin-α2β2γ1), and the less-abundant laminin-213 (laminin-α2β1γ3). Laminin-211 and -221 are primarily known for their function in basement membrane architecture. In this paper, we show that laminin α2 is also important for the cellular invasion of the bacterial pathogens GBS, GAS, and *S. aureus*. Consistent with this observation, proteins with laminin-binding properties have been described for these bacteria (19–24). However, the studies whose results are shown here are the first to implicate a specific laminin isoform in the invasion of these bacteria. Interestingly, this isoform is also relevant for host-pathogen interactions of mycobacteria like *Mycobacterium leprae* (25, 26). *M. leprae*, the causative agent of leprosy, can attach to Schwann cells via the interaction of laminin α2 with cellular laminin receptors, specifically integrin β4 (26) and α-dystroglycan (27). In this context, laminin α2 acts as a bridge between the host cell and the pathogen, which may also pertain to the interaction of GBS, GAS, and *S. aureus* with host cells. The observation that incubation of host cells or GBS with laminin α2 enhances bacterial invasion supports a bridging function for laminins in infection. Other extracellular matrix proteins, such as fibronectin, can function in a similar way, as has been reported for the invasion of *S. aureus* (28).

The availability of pgsA745 cells lacking a specific part of laminin α2 maps a functional site for invasion by GBS, GAS, and *S. aureus*. This deleted region corresponds to an ~90% loss of the N-terminal globular domain L4a, as well as a loss of half of the rodlike LEb domain. The function of these domains is largely unknown. One report indicated that the L4a domain, although only tested for the laminin α1 chain, binds to the extracellular matrix protein fibulin-2 (29). The LE domains seem to function as spacers between the globular domains (30). Whether the L4a-LEb region serves as an attachment site for the bacteria or as a coreceptor for invasion remains unknown. However, given that attachment of the bacteria evaluated here occurs normally in pgsA745 cells, it is conceivable that an interaction of laminin α2 with integrins or other receptors for internalization is impaired in pgsA745 cells. It is also possible that the deletion described above results in a global change in the laminin trimer, with a consequent loss in functionality as a portal of entry for bacteria. Thus, laminin α2 may contribute to colonization and the penetration of host cell barriers by providing an initial foothold for bacterial pathogens.

The mutant pgsA745 cell line was identified by a forward screen seeking mutants that failed to incorporate radioactive sulfate into GAGs (3). It belongs to a collection of strains deficient in specific biochemical steps involved in GAG biosynthesis (31). This particular mutant cell line has been used in a large number of studies that generally established the importance of GAGs in various systems. In most cases, the mutant phenotype correlates with a loss of GAGs, based on similar phenotypic changes in other GAG-deficient CHO cell mutants and on correction of pgsA745 cells by transfection with xylosyltransferase isozymes (6, 32). However, as this paper illustrates, (chemically) mutagenized strains, such as pgsA745, may contain additional mutations that can influence experimental outcomes and can lead to incorrect interpretation of the data. Nevertheless, careful experiments to reconfirm initial findings using gene-targeting methods can help uncover interesting covert features of these original mutants.

In conclusion, the combination of whole-genome sequencing and RNA-Seq led to the discovery that the pgsA745 cell line harbors an additional mutation in *Lama2* that confers panresistance to bacterial invasion. pgsA745 cells are also resistant to infection by multiple viruses due to the lack of GAG receptors (e.g., see references 33 and 34). As CHO cells are used extensively for recombinant protein production, the mutant pgsA745 cell line is an excellent candidate for an infection-resistant expression system.

## MATERIALS AND METHODS

Detailed materials and methods are provided in Text S1 in the supplemental material.

10.1128/mBio.02128-16.1 TEXT S1  Supplemental materials and methods. Detailed materials and methods are provided. Download TEXT S1, DOCX file, 0.04 MB. Copyright © 2017 van Wijk et al. 2017 van Wijk et al. This content is distributed under the terms of the Creative Commons Attribution 4.0 International license.

### Animal cells.

CHO-K1 cells were obtained from the American Type Culture Collection (ATCC) (CCL-61). The mutant pgsA745 cell line was described previously (3) and subsequently shown to contain a point mutation in *XylT2*. Simian virus 40 (SV40) large T antigen-immortalized human brain microvascular endothelial cells (hBMEC) were obtained from Kwang Sik Kim (Johns Hopkins University, Baltimore, MD). A549 human alveolar basal epithelial cells were obtained from the ATCC (CCL-185). The culture conditions can be found in the supplemental materials and methods in Text S1 in the supplemental material.

### Bacterial strains.

GBS strain COH1 is a serotype III isolate from an infant with bacteremia (35). GBS strain A909 is a serotype Ia neonatal isolate (36). GAS strain 5448 is an M1T1 serotype isolate from a patient with necrotizing fasciitis and toxic shock syndrome (37). Methicillin-resistant *Staphylococcus aureus* (MRSA) strain USA300 TCH1516 is an isolate from an adolescent patient with severe sepsis syndrome (38). *Staphylococcus aureus* Newman (39) is a methicillin-sensitive (MSSA) strain.

### Generation of *XylT2* knockouts using the CRISPR-*cas9* system.

*Xylosyltransferase 2* (*Xylt2*) mutant cell lines CHO-23A1 and CHO-93A5 were generated using the Edit-R CRISPR-*cas9* gene engineering system (Dharmacon GE Healthcare) according to the manufacturer’s instructions. In short, CHO-K1 cells were cotransfected with a *cas9* expression vector containing a blasticidin resistance marker (pHCSVBlast-Cas9, catalog number U-001000-120), *trans*-activating CRISPR RNA (tracrRNA) (catalog number U-002000-120), and CRISPR RNA (crRNA) specific for CHO *Xylt2* (crRNA-107962; 5′ GAGGCACUAAUGGGCGCUGCGUUUUAGAGCUAUGCUGUUUUG 3′). The target sequence (GAGGCACTAATGGGCGCTGCTGG, target identifier (ID) 791342), found in exon 1, was determined using CRISPy, a web-based target-finding tool for CHO-K1 cells (40). Two days after transfection, cells were selected by incubation with 10-μg/ml blasticidin S (Thermo Fisher Scientific) for 5 days, and cells were then seeded in 96-well plates to obtain single-cell clones. The clonal lines obtained were screened for heparan sulfate expression by flow cytometry using the anti-heparan sulfate single-chain antibody HS4C3 (41) as described below (see “Flow cytometry”).

### Bacterial adherence and invasion assays.

Adherence and invasion assays were performed essentially as described previously (2). In one experiment, wild-type cells were pretreated with or without 10 mIU/ml heparinase III in Ham’s F-12 growth medium supplemented with 0.5% (vol/vol) FBS for 30 min at 37°C before the addition of laminin-211/221 and GBS. In another experiment, GBS was preincubated with or without 50-μg/ml laminin-211/221 in phosphate-buffered saline (PBS) for 30 min at room temperature and washed with PBS before being added to CHO cells. To ensure that differences in bacterial invasion were not due to differences in cell viability, the release of lactate dehydrogenase (LDH) into the medium was quantified during bacterial incubation and antibiotic protection. The LDH in 50 μl of culture supernatant or in 50 μl of cells lysed with 0.025% (vol/vol) Triton X-100 was quantified using the CytoTox 96 nonradioactive cytotoxicity assay (Promega) according to the manufacturer’s instructions.

### Bacterial adhesion to laminin.

Laminin-211/221 (10 µg/ml, human merosin CC085; EMD Millipore) in 50 mM carbonate-bicarbonate buffer (product number C3041; Sigma-Aldrich) was used to coat a 96-well plate (Costar 9018, enzyme immunoassay/radioimmunoassay [EIA/RIA] high binding). The plate was covered with Parafilm and incubated overnight at 4°C. The plate was washed 4 times with PBS and blocked with PBS containing 1% (wt/vol) bovine serum albumin (BSA) for 1 h at room temperature. Bacteria were labeled with fluorescein isothiocyanate (FITC) as described in Text S1 in the supplemental material (see “Invasion assay with FITC-labeled bacteria”). The plate was washed 4 times with PBS, and ~10^7^ CFU of bacteria in 100 μl was added per well. The plate was centrifuged at 500 × *g* for 10 min and incubated for 30 min at 37°C. Fluorescence (excitement/emission, 485/538) was measured on a plate reader (SpectraMax M3; Molecular Devices) before and after washing 5 times with PBS containing 1% (wt/vol) BSA.

### Flow cytometry.

Flow cytometry was performed essentially as described previously (42). CHO cells were detached by using a Versene solution (Life Technologies, Inc.), incubated with the antibodies/proteins described in Text S1 in the supplemental material, and analyzed on a FACSCalibur instrument (BD Biosciences). For detection of HS, cells were incubated sequentially with the vesicular stomatitis virus (VSV)-tagged single-chain variable-fragment (scFv) antibody HS4C3 (1:100 [41]), mouse anti-VSV IgG (1:10; P5D4), and Alexa Fluor 488 fluorochrome-conjugated anti-mouse IgG (20 μg/ml; Life Technologies, Inc.). For detection of FGF2 binding, cells were incubated sequentially with biotinylated FGF2 (1:250 [43]) and DyLight 488 fluorochrome-conjugated streptavidin (10 μg/ml; Thermo Scientific). Endocytic/phagocytic assays are described in Text S1.

To determine the laminin-binding capacities of different bacteria, laminin-211/221 was concentrated using a 100-kDa-cutoff Amicon centrifugal filter unit (EMD Millipore) and FITC labeled using the FluoReporter FITC protein labeling kit (Life Technologies, Inc.) according to the manufacturer’s instructions. Bacteria (3.75 × 10^7^ CFU) were incubated with 50 μg/ml of the labeled laminin for 30 min at 37°C, washed twice with PBS, and analyzed by flow cytometry on a FACSCalibur instrument.

### Immunocytochemistry.

For detection of laminin α2, 4.0 × 10^4^ CHO cells per well were seeded in 8-well glass chamber slides (Lab-Tek). Two days later, cells were washed with PBS, fixed with 4% paraformaldehyde (PFA) in phosphate buffer (PB-PFA) for 10 min, blocked with PBS containing 1% (wt/vol) BSA, and incubated with anti-laminin α2 antibody (1:500, clone 5H2; EMD Millipore), followed by Alexa Fluor 488-conjugated anti-mouse IgG (10 μg/ml; Life Technologies, Inc.). For actin staining after incubation with EPEC, CHO cells (1.0 × 10^5^ per well) were seeded in 4-well glass chamber slides (Lab-Tek). The next day, cells were incubated with or without ~2 × 10^5^ CFU of log-phase-grown EPEC for 2.5 h at 37°C. Cells were washed twice with PBS, fixed with 4% PB-PFA for 10 min, washed twice with PBS, permeabilized using 0.2% (vol/vol) Triton X-100 for 8 min, blocked with PBS containing 1% (wt/vol) BSA, and stained for actin using Alexa Fluor 488-conjugated phalloidin (1:100; Life Technologies, Inc.). The slides were mounted in SlowFade (Life Technologies, Inc.). Images were acquired using a Zeiss Axio Observer D1 microscope and Zen 2012 Blue software.

### siRNA and cDNA transfections.

For siRNA transfections, 5.0 × 10^4^ CHO cells per well were seeded into 24-well plates. About 1 h after seeding, cells were transfected with a final concentration of 5 nM siRNA using HiPerFect transfection reagent (Qiagen) according to the manufacturer’s instructions. Laminin α2 was targeted using the following duplex: sense, GGAACAAACUUACCAGUCAdTdT, and antisense, UGACUGGUAAGUUUGUUCCdTdT (Sigma-Aldrich). Universal negative control #1 siRNA (Sigma-Aldrich) was used as a negative control. Two days after transfection, RNA was isolated as described below (see “DNA and RNA isolation”), and a bacterial invasion assay was performed as described above (see “Bacterial adherence and invasion assays”).

For h*LAMA2* cDNA transfection, CHO cells (3.0 × 10^5^ cells per well) were seeded into 6-well plates. The following day, cells were transfected with 2 μg human *LAMA2* cDNA in a pcDNA3.1 vector that was modified to contain a puromycin resistance gene instead of the zeocin resistance gene. Cells were transfected using Lipofectamine 3000 transfection reagent (Life Technologies, Inc.) according to the manufacturer’s instructions. Cells were reseeded the next day, and selection using 10 μg/ml puromycin was started one day later. Selection pressure was maintained for two weeks, after which cells were used for bacterial invasion assays and isolation of RNA as described in the corresponding sections herein. pgsA745-XylT1 cells were generated by stable transfection with human XylT1 cDNA in pcDNA3.1, and a single-cell population was generated. pgsA745-XylT2 cells were a gift from Cuellar et al. (6).

### DNA and RNA isolation.

For DNA isolation, cells were detached using 0.05% trypsin–0.53 mM EDTA and pelleted. DNA was isolated using the DNeasy blood and tissue kit (Qiagen) according to the manufacturer’s instructions. For RNA isolation, cells were lysed directly using Trizol, after which chloroform was added. Following centrifugation at 12,000 × *g* for 15 min at 4°C, the transparent upper phase was transferred and an equal volume of 70% ethanol was added. This mixture was applied to an RNeasy mini-spin column (Qiagen), and the manufacturer’s instructions were followed.

### Quantitative PCR analysis.

cDNA was synthesized from total RNA using the SuperScript III first-strand synthesis system (Life Technologies, Inc.) according to the manufacturer’s instructions. Quantitative PCR (qPCR) was performed using the Power SYBR green PCR master mix (2×; Life Technologies, Inc.) on a CFX96-C1000 real-time PCR detection system (Bio-Rad) according to the manufacturers’ instructions. Primer sequences can be found in Text S1 in the supplemental material.

### Whole-genome/RNA sequencing.

Whole-genome sequencing libraries were prepared using the TruSeq DNA sample prep kit (Illumina, San Diego, CA, USA), and RNA libraries were prepared for sequencing using the TruSeq stranded mRNA sample preparation kit (Illumina) according to the manufacturer’s instructions, with the following change: poly(A) enrichment was used to eliminate rRNA transcripts from RNA-Seq libraries. The libraries were clustered using cBot and sequenced on a HiSeq 2500 system (HiSeq Control Software version 2.2.38/RTA version 1.18.61) with a 2 × 101 setup. Bcl-to-Fastq conversion was performed using bcl2Fastq version 1.8.3 from the CASAVA software suite. The data analysis methods are described in Text S1 in the supplemental material. All sequence data were deposited in NCBI GenBank (accession number PRJNA304606). DNA sequencing data can be obtained from the NCBI Bioproject PRJNA305442, Biosample SAMN04325241.
