# Supplementary material for: Whole-Genome Sequencing of Invasion-Resistant Cells Identifies Laminin α2 as a Host Factor for Bacterial Invasion
Source: mBio. 2017 Jan 10;8(1):e02128-16. doi: 10.1128/mBio.02128-16 (PMC5225314; doi:10.1128/mBio.02128-16)
Supplement: FIG S1 [file mbo006163131sf1.pdf]

```
CHO-K1            gcacactacgttggcgatctcctgctggcattgcttggtgctggcccgggccaatgctga
23A6WT-XYLT2_F9 gcacactacgttggcgatctcctgctggcattgcttggtgctggcccgggccaatgctga
93A1WT-XYLT2_F9 gcacactacgttggcgatctcctgctggcattgcttggtgctggcccgggccaatgctga
23A1KO-XYLT2_F9 gcacactacgttggcgatctcctgctggcattgcttggtgctggcccgggccaatgctga
93A5KO-XYLT2_F9 gcacactacgttggcgatctcctgctggcattgcttggtgctggcccgggccaatgctga
                ************************************************************

CHO-K1          cagtgcatctttgcccacaatctcgcactttggagtgaagctgttgtccgtgggctgggg
23A6WT-XYLT2_F9 cagtgcatctttgcccacaatctcgcactttggagtgaagctgttgtccgtgggctgggg
93A1WT-XYLT2_F9 cagtgcatctttgcccacaatctcgcactttggagtgaagctgttgtccgtgggctgggg
23A1KO-XYLT2_F9 cagtgcatctttgcccacaatctcgcactttggagtgaagctgttgtccgtgggctgggg
93A5KO-XYLT2_F9 cagtgcatctttgcccacaatctcgcactttggagtgaagctgttgtccgtgggctgggg
                ************************************************************

CHO-K1          ggcaccttccacgcttcctgtgtctccatgtggtgggaagccagca--------------
23A6WT-XYLT2_F9 ggcaccttccacgcttcctgtgtctccatgtggtgggaagccagca--------------
93A1WT-XYLT2_F9 ggcaccttccacgcttcctgtgtctccatgtggtgggaagccagca--------------
23A1KO-XYLT2_F9 ggcaccttccacgcttcctgtgtctccatgtggtgggaagcca-----------------
93A5KO-XYLT2_F9 ggcaccttccacgcttcctgtgtctccatgtggtgggaagccagcacgtacacgcctacc
                *******************************************

CHO-K1          ------------------------------------------------------------
23A6WT-XYLT2_F9 ------------------------------------------------------------
93A1WT-XYLT2_F9 ------------------------------------------------------------
23A1KO-XYLT2_F9 ------------------------------------------------------------
93A5KO-XYLT2_F9 gcccatttgcgtcaatggggcggagttgttacgacattttggaaagtcccgttgattttg

CHO-K1          ------------------------------------------------------------
23A6WT-XYLT2_F9 ------------------------------------------------------------
93A1WT-XYLT2_F9 ------------------------------------------------------------
23A1KO-XYLT2_F9 ------------------------------------------------------------
93A5KO-XYLT2_F9 gtgccaaaacaaactcccattgacgtcaatggggtggagacttggaaatccccgtgagtc

CHO-K1          ------------------------------------------------------------
23A6WT-XYLT2_F9 ------------------------------------------------------------
93A1WT-XYLT2_F9 ------------------------------------------------------------
23A1KO-XYLT2_F9 ------------------------------------------------------------
93A5KO-XYLT2_F9 aaaccgctatccacgcccattgatgtactgccaaaaccgcatcaccatggtaatagcgat

CHO-K1          ------------------------------------------------------------
23A6WT-XYLT2_F9 ------------------------------------------------------------
93A1WT-XYLT2_F9 ------------------------------------------------------------
23A1KO-XYLT2_F9 ------------------------------------------------------------
93A5KO-XYLT2_F9 gactaatacgtagatgtactgccaagtaggaaagtcccataaggtcatgtactgggctac

CHO-K1          --gcgcccattagtgcctccccagctgctgctccactcaggttctggcgacctggtgcct
23A6WT-XYLT2_F9 --gcgcccattagtgcctccccagctgctgctccactcaggttctggcgacctggtgcct
93A1WT-XYLT2_F9 --gcgcccattagtgcctccccagctgctgctccactcaggttctggcgacctggtgcct
23A1KO-XYLT2_F9 ----gcccattagtgcctccccagctgctgctccactcaggttctggcgacctggtgcct
93A5KO-XYLT2_F9 ccgcgcccattagtgcctccccagctgctgctccactcaggttctggcgacctggtgcct
                    ********************************************************

CHO-K1          ccggaggtggggcaggtgggacccgtcggctggctctttggcggctggttactgcccgca
23A6WT-XYLT2_F9 ccggaggtggggcaggtgggacccgtcggctggctctttggcggctggttactgcccgca
93A1WT-XYLT2_F9 ccggaggtggggcaggtgggacccgtcggctggctctttggcggctggttactgcccgca
23A1KO-XYLT2_F9 ccggaggtggggcaggtgggacccgtcggctggctctttggcggctggttactgcccgca
93A5KO-XYLT2_F9 ccggaggtggggcaggtgggacccgtcggctggctctttggcggctggttactgcccgca
                ************************************************************

CHO-K1          ccaccttggccacaggcacacctggactctcggcacggccccgccagcgcccatgccttt
23A6WT-XYLT2_F9 ccaccttggccacaggcacacctggactctcggcacggccccgccagcgcccatgccttt
93A1WT-XYLT2_F9 ccaccttggccacaggcacacctggactctcggcacggccccgccagcgcccatgccttt
23A1KO-XYLT2_F9 ccaccttggccacaggcacacctggactctcggcacggccccgccagcgcccatgccttt
93A5KO-XYLT2_F9 ccaccttggccacaggcacacctggactctcggcacggccccgccagcgcccatgccttt
                ************************************************************
```
