# Supplementary figures and images for: Whole-Genome Sequencing of Invasion-Resistant Cells Identifies Laminin α2 as a Host Factor for Bacterial Invasion
Source: mBio. 2017 Jan 10;8(1):e02128-16. doi: 10.1128/mBio.02128-16 (PMC5225314; doi:10.1128/mBio.02128-16)

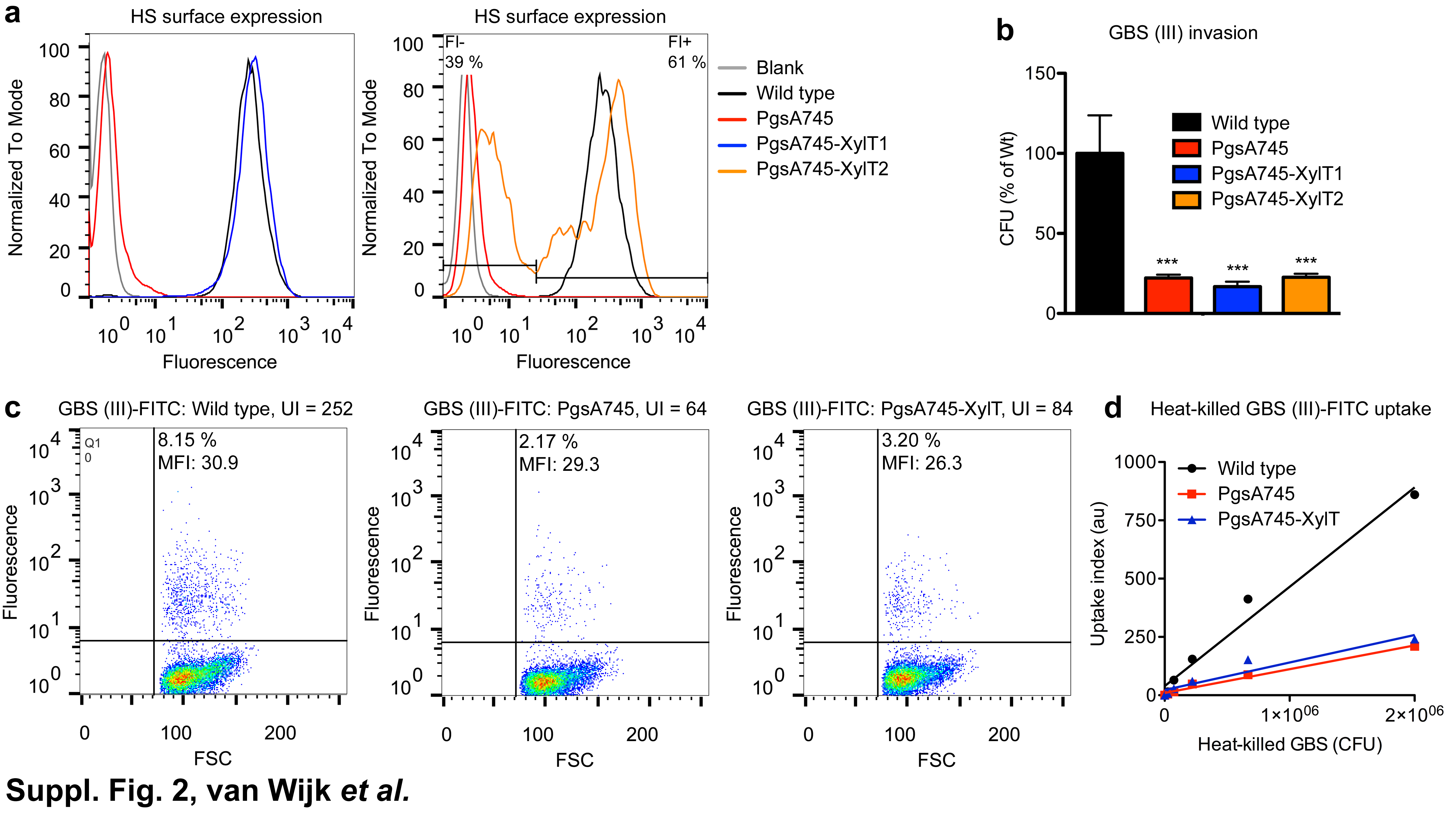

Supplement: FIG S2 [file mbo006163131sf2.tif]

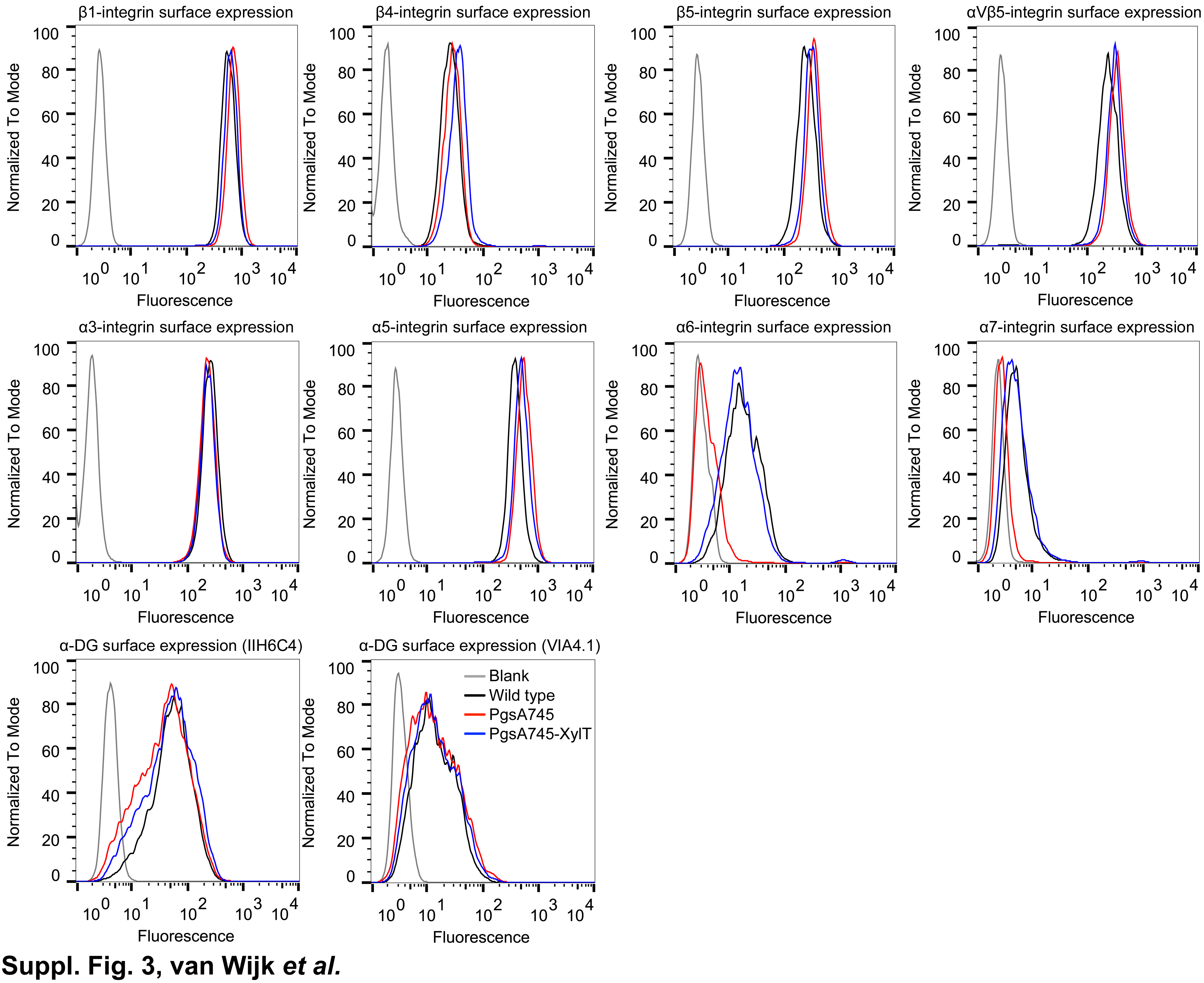

Supplement: FIG S3 [file mbo006163131sf3.tif]
