# Supplementary material for: Whole-Genome Sequencing of Invasion-Resistant Cells Identifies Laminin α2 as a Host Factor for Bacterial Invasion
Source: mBio. 2017 Jan 10;8(1):e02128-16. doi: 10.1128/mBio.02128-16 (PMC5225314; doi:10.1128/mBio.02128-16)
Supplement: TEXT S1 [file mbo006163131s1.docx]

**S5. Supporting Information for:**

**Whole Genome Sequencing of Invasion-Resistant Cells Identifies Laminin α2 as a Host Factor for Bacterial Invasion**

Xander M. van Wijk^1^, Simon Döhrmann^2^, Björn M. Hallström^3, 4^, Shangzhong Li^2, 7, 8^, Bjørn G. Voldborg^3^, Brandon X. Meng^1^, Karen K. McKee^5^, Toin H. van Kuppevelt^6^, Peter D. Yurchenco^5^, Bernhard O. Palsson^3, 7, 8^, Nathan E. Lewis^2, 8^, Victor Nizet^2^, Jeffrey D. Esko^1^

^1^Department of Cellular and Molecular Medicine, University of California, San Diego, La Jolla, California, USA; ^2^Department of Pediatrics, University of California, San Diego, La Jolla, California, USA; ^3^Novo Nordisk Foundation Center for Biosustainability, Technical University of Denmark, Hørsholm, Denmark; ^4^Royal Institute of Technology, Stockholm, Sweden; ^5^Department of Pathology and Laboratory Medicine, Robert Wood Johnson Medical School, Piscataway, NJ 08854, USA; ^6^Department of Biochemistry, Radboud Institute for Molecular Life Sciences, Radboud University Medical Centre, Nijmegen, The Netherlands; ^7^Department of Bioengineering, University of California, San Diego, La Jolla, California, USA; ^8^Novo Nordisk Foundation Center for Biosustainability, University of California, San Diego, La Jolla, California, USA.

**Supplemental materials and methods**

**Animal cell culture conditions.** CHO cells were cultured in Ham’s F-12 growth medium (Life Technologies) supplemented with 10% (v/v) fetal bovine serum (FBS; Gemini Bioproducts), 100 µg/ml of streptomycin, and 100 Units/ml of penicillin (Life Technologies). hBMEC and A549 cells were cultured in RPMI 1640 medium (Life Technologies) supplemented with 20% (v/v) FBS, 100 µg/ml of streptomycin, and 100 Units/ml of penicillin.

**Growth of bacterial strains.** GBS strains COH1 and A909, and GAS strain 5448, were grown in Todd-Hewitt broth (THB) at 37 °C in stationary culture. *Staphylococcus aureus* strains Newman (MSSA) and USA300 TCH1516 (MRSA) were grown in THB at 37° C with shaking at 200 rpm. Enteropathogenic *Escherichia coli* (EPEC) strain 2348/69 (serotype O127: H6) was grown in Luria-Bertani broth (LB) at 37 °C with shaking at 200 rpm.

**Sequencing of CRISPR-Cas9 generated *Xylt2* knockout clones.** *Xylt2*-targeted clones 23A1 and 93A5 and wild-type control clones 23A6 and 93A1, were analyzed for genetic aberrations in *XylT2*. DNA was isolated using the DNeasy Blood & Tissue Kit (Qiagen, Venlo, The Netherlands) and a region of 472 bp that includes the *Xylt2* target sequence was amplified by PCR (F 5'-GATCAGAGTACCCTAGTCCCTC-3', R 5'-TCTAAGGACACAGACAGCTCT-3'). PCR products were purified by agarose gel extraction using the QIAquick Gel Extraction Kit (Qiagen) and were sequenced by Sanger DNA Sequencing (Genewiz, South Plainfield, NJ). The sequences were compared to the CHO-K1 cDNA sequence in the NCBI database.

**Bacterial adherence and invasion assays.** Adherence and invasion assays were essentially performed as described (1). CHO cells (1.5 to 2.0 x 10^5^), A549 cells (1.0 x 10^5^) or hBMECs (1.0 x 10^5^) were seeded into each well of 24-well plates one day prior to assay. In some experiments, 5.0 x 10^4^ CHO cells per well were seeded into 24-well plates two days prior to assay. On the day of the assay, medium was replaced with 0.4 ml fresh medium, with or without 6.25/12.5 μg/ml laminin 211/221 (human merosin CC085; EMD Millipore) as indicated. After ~1 h, 100 μl of log-phase grown bacteria (~10^6^ CFU) was added and contact was initiated by 5 min centrifugation at 500 x *g*. After 30-45 min of incubation, cells were washed with PBS and treated with gentamicin and penicillin G for 1.5-2 h. Cells were disrupted with 0.025% Triton X-100, and the number of invasive bacteria was quantified by serial dilution plating. The level of surface-adherent (total cell-associated) bacteria was quantified after 15 min of incubation without the addition of antibiotics. Bacterial counts were normalized to the cellular protein content of a parallel plate incubated without bacteria, using the Pierce™ BCA protein assay kit (Thermo Scientific).

**Invasion assay with FITC-labeled bacteria.** Bacterial invasion was also measured by flow cytometry, using bacteria labeled with fluorescein isothiocyanate (FITC). Log-phase bacteria were sedimented by centrifugation, washed with PBS, resuspended at ~10^9^ CFU / ml in 0.1% FITC (F7250; Sigma Aldrich) in PBS, and incubated at 37 °C for 30 min. In some experiments, bacteria were heat-killed by incubation at 56 °C for 40 min. Bacteria were extensively washed (at least 4-5 times) with PBS to remove trace amounts of free FITC and then resuspended in medium for the invasion assay. Approximately 10^6^ CFU were added to CHO cells in a 24-well plate, after which contact was initiated by 5 min centrifugation at 500 x *g*. After 2 h of incubation, cells were detached using 0.05% trypsin-0.53 mM EDTA, washed with PBS, and analyzed by flow cytometry. CHO cells were gated to avoid ‘free’ bacteria. To obtain an estimate of the amount of internalized bacteria, the percentage of FITC-positive cells was multiplied by the mean fluorescence intensity (MFI) of these cells to obtain an arbitrary uptake index, as described elsewhere (2).

**Flow cytometry.** Flow cytometry was essentially performed as described previously (3). CHO cells were detached with Versene (Life Technologies), incubated with the following antibodies/proteins, and analyzed on a FACSCalibur (BD Biosciences). For endocytosis/phagocytosis assays, CHO cells were incubated with (i) 1 mg/ml dextran-FITC (3 - 5 kDa; FD4, Sigma Aldrich) for 30 min, (ii) 5 µg/ml fluorescein conjugated transferrin (T-2871, Life Technologies) for 60 min at 4 ºC, PBS washed, and 5 min at 37 ºC, (iii) 17 µg/ml Alexa488 fluorochrome conjugated Zymosan A (*S. cerevisiae*) BioParticles® (Z-23373, Life Technologies) for 1 h, or (iv) 6 × 10^8^/ml 0.52 µm dragon green fluorescent polymer microspheres (latex beads; FS03F/5069 Bangs Laboratories) for 1 h. Cells were washed twice with PBS, detached using 0.05% trypsin-0.53 mM EDTA, washed with PBS, and analyzed by flow cytometry.

For detection of integrins, the following antibodies were used: anti-α3 (15 μg/ml; Ralph3.1, Developmental Studies Hybridoma Bank: DSHB), anti-α5 (10 μg/ml; PB1, DSHB), anti-α6 (20 μg/ml; LS-A8770, LifeSpan BioSciences), anti-α7 (25 μg/ml; Santa Cruz Biotechnology), anti-αVβ5 (20 μg/ml; P5H9, DSHB), anti-β1 (10 μg/ml; 7E2, DSHB), anti-β4 (100 μg/ml; H101, Santa Cruz Biotechnology), and anti-β5 (10 μg/ml; KN52, DSHB), followed by the appropriate Alexa488 fluorochrome conjugated secondary antibody: anti-mouse IgG, anti-rabbit IgG, anti-rat IgG, or anti-goat IgG (20 μg/ml; Life Technologies). Dystroglycan was detected using antibodies IIH6C4 (10 μg/ml; DSHB) and VIA4.1 (20 μg/ml; DSHB), followed by Alexa488 fluorochrome conjugated anti-mouse IgM or anti-mouse IgG (20 μg/ml; Life Technologies), respectively.

**Quantitative PCR primers.** A region specific for ‘long’ form of *Lama2* was amplified using the following primers: F 5’-CAGGAGAGTGTCTGAACTGTAAG-3’; R 5’- CCTCGAGTAGGATCACCATAGA-3’. Total or ‘short + long’ Lama2 was amplified using: F 5’-GCCAGAACTGTCAGCATCATA-3’; R 5’-CTGGGAGAAATCAGAGGACAAG-3’. Expression was normalized to the ‘house-keeping’ gene *TBP*: F 5’-GGGATTCAGGAAGACCACATAG-3’; R 5’-GTACCATGAGCGGTACCAAA-3’. Human *LAMA2* cDNA was detected using: F 5’-TTTCTATGGCGAGCCTACTAAAG-3’; R 5’-GCACGTTGGGCTAAAGTTATTG-3’, by amplifying a region corresponding to CHO ‘long’ form of *Lama2*.

**Whole genome/RNA-Seq** **data analysis**. Raw whole genome sequencing data of pgsA745 was mapped to the Chinese hamster reference genome (cgr_alt_C_griseus_v1.0) using BWA version 0.7.8, with the BWA-MEM algorithm (4, 5). Sites with potential indels were detected and realigned using the RealignerTargetCreator/IndelRealigner modules of GATK version 3.3.0 (6). PCR duplicates were detected and eliminated using the tool MarkDuplicates from Picard version 1.100 (http://broadinstitute.github.io/picard/). Raw RNA-sequencing reads from wild-type CHO-K1, pgsA745, and pgsA745-Xylt1 (all in duplicate) were mapped to the Chinese hamster reference genome using TopHat version 2.0.8b (7) with Bowtie 2.2.3 (8). Gene expression values (FPKM) were estimated using Cufflinks version 2.1.1 (9).

To detect larger deletions/duplications in protein-coding genes of pgsA745 the mapped coverage of each annotated exon was calculated using the genomecov module from bedtools version 2.16.2 for the pgsA745 genome and for six other CHO genomes (10). The coverages for the exons were normalized to the average coverage across the whole genome. Exons with a deviating coverage in the pgsA745 strain (2-fold higher/lower than the median of the other strains) were marked as potential deletions or duplications. The alignments were manually inspected in order to detect obvious false positives from alignment problems and to detect soft-clipped reads at the borders of the indel events. Additionally, the RNA-Seq alignments were used for additional verification of potential deletions. This was done using two criteria, (i) the expression of deleted exon should be lower than other exons of the same gene, and (ii) if the mutated allele is expressed it should have splicing events spanning over the deleted exons. A script was created that plotted the mapped coverage of all exons for the gene together with arcs showing individual spliced reads. The resulting plots were manually inspected to validate the expression and proper splicing of the “deletion allele”.

**Supplemental references**

1. Chang YC, Wang Z, Flax LA, Xu D, Esko JD, Nizet V, et al. Glycosaminoglycan binding facilitates entry of a bacterial pathogen into central nervous systems. PLoS Pathog. 2011;7(6):e1002082.

2. Pils S, Schmitter T, Neske F, Hauck CR. Quantification of bacterial invasion into adherent cells by flow cytometry. J Microbiol Methods. 2006;65(2):301-10.

3. van Wijk XM, Thijssen VL, Lawrence R, van den Broek SA, Dona M, Naidu N, et al. Interfering with UDP-GlcNAc metabolism and heparan sulfate expression using a sugar analogue reduces angiogenesis. ACS chemical biology. 2013;8(10):2331-8.

4. Li H, Durbin R. Fast and accurate short read alignment with Burrows-Wheeler transform. Bioinformatics (Oxford, England). 2009;25(14):1754-60.

5. Li H. Aligning sequence reads, clone sequences and assembly contigs with BWA-MEM. arXiv:13033997 [q-bioGN]. 2013.

6. McKenna A, Hanna M, Banks E, Sivachenko A, Cibulskis K, Kernytsky A, et al. The Genome Analysis Toolkit: a MapReduce framework for analyzing next-generation DNA sequencing data. Genome Res. 2010;20(9):1297-303.

7. Kim D, Pertea G, Trapnell C, Pimentel H, Kelley R, Salzberg SL. TopHat2: accurate alignment of transcriptomes in the presence of insertions, deletions and gene fusions. Genome Biol. 2013;14(4):R36.

8. Langmead B, Salzberg SL. Fast gapped-read alignment with Bowtie 2. Nat Methods. 2012;9(4):357-9.

9. Trapnell C, Roberts A, Goff L, Pertea G, Kim D, Kelley DR, et al. Differential gene and transcript expression analysis of RNA-seq experiments with TopHat and Cufflinks. Nat Protoc. 2012;7(3):562-78.

10. Lewis NE, Liu X, Li Y, Nagarajan H, Yerganian G, O'Brien E, et al. Genomic landscapes of Chinese hamster ovary cell lines as revealed by the Cricetulus griseus draft genome. Nat Biotechnol. 2013;31(8):759-65.
