# Supplementary material for: Whole-Genome Sequencing of Invasion-Resistant Cells Identifies Laminin α2 as a Host Factor for Bacterial Invasion
Source: mBio. 2017 Jan 10;8(1):e02128-16. doi: 10.1128/mBio.02128-16 (PMC5225314; doi:10.1128/mBio.02128-16)
Supplement: DATA SET S1 [file mbo006163131sd1.pdf]

Product	gene_short_name	Entrez_GeneID	CHOK1_1_FPKM	CHOK1_2_FPKM	PgsA745_1_FPKM	PgsA745_2_FPKM	Pgsa745.XYLT1_1_FPKM	Pgsa745.XYLT1_2_FPKM	CHOK1_1_NormCount	CHOK1_2_NormCount	PgsA745_1_NormCount	PgsA745_2_NormCount	Pgsa745.XYLT1_1_NormCount	Pgsa745.XYLT1_2_NormCount
APOBEC1 complementation factor	A1cf	100767014	0.00	0.00	0.00	0.00	0.00	0.00	0.00	0.00	0.00	0.00	0.00	0.00
alpha-2-macroglobulin-like 1	A2ml1	100762067	0.00	0.00	0.00	0.00	0.00	0.00	0.00	0.00	0.00	0.00	0.00	0.00
alpha 1,3-galactosyltransferase 2	A3galt2	100774283	0.00	0.00	0.00	0.00	0.00	0.00	0.00	0.00	0.00	0.00	0.00	0.00
alpha 1,4-galactosyltransferase	A4galt	100770462	0.00	0.00	0.00	0.02	0.04	0.00	0.00	0.00	1.24	1.16	2.07	1.87
alpha-1,4-N-acetylglucosaminyltransferase	A4gnt	100771969	0.00	0.00	0.00	0.00	0.00	0.00	0.00	0.00	0.00	0.00	0.00	0.00
ATP synthase F0 subunit 6	ATP6	3979188	0.00	0.00	0.00	0.00	0.00	0.00	0.00	0.00	0.00	0.00	0.00	0.00
ATP synthase F0 subunit 8	ATP8	3979187	0.00	0.00	0.00	0.00	0.00	0.00	0.00	0.00	0.00	0.00	0.00	0.00
achalasia, adrenocortical insufficiency, alacrimia	Aaas	100767976	22.67	22.81	28.71	28.67	29.70	29.22	1269.95	1270.98	1570.00	1530.93	1609.40	1584.27
acetoacetyl-CoA synthetase	Aacs	100750893	9.16	9.04	10.16	8.70	7.75	8.25	859.04	845.49	956.41	830.95	729.67	777.65
arylacetamide deacetylase	Aadac	100771815	0.00	0.00	0.00	0.00	0.00	0.00	0.00	0.00	0.00	0.00	0.00	0.00
arylacetamide deacetylase-like 3	Aadacl3	100751803	0.00	0.00	0.00	0.00	0.00	0.00	0.00	0.00	0.00	0.00	0.00	0.00
aminoadipate aminotransferase	Aadat	100771752	0.03	0.04	0.00	0.00	0.00	0.00	1.52	0.92	0.00	0.00	0.00	0.00
AhpC/TSA antioxidant enzyme domain containing 1	Aaed1	100760049	8.98	9.09	8.41	10.07	10.93	11.12	342.55	348.63	337.85	385.92	395.36	426.21
alpha- and gamma-adaptin binding protein	Aagab	100756630	26.67	26.86	25.68	27.14	29.92	31.39	1095.26	1150.20	1121.60	1175.14	1311.33	1309.47
AP2 associated kinase 1	Aak1	100771995	7.39	7.58	7.85	7.39	12.60	12.61	1190.96	1231.64	1233.39	1222.66	1796.74	1813.26
adipogenesis associated, Mth938 domain containing	Aamdc	100767599	11.97	9.93	10.88	14.94	15.44	15.54	101.78	97.91	93.16	115.89	117.99	121.51
angio-associated, migratory cell protein	Aamp	100775009	104.78	101.62	104.85	89.55	64.68	74.06	4988.65	4910.07	5165.84	4436.35	3329.55	3668.58
aralkylamine N-acetyltransferase	Aanat	100755392	0.00	0.00	0.00	0.09	0.00	0.00	0.76	0.00	2.48	1.16	0.00	0.00
AAR2 splicing factor homolog (S. cerevisiae)	Aar2	100752282	41.72	35.85	36.45	36.08	32.60	41.51	2386.47	2127.45	2156.26	2072.15	1997.52	2346.96
alanyl-tRNA synthetase	Aars	100766178	168.01	161.99	189.80	187.18	157.46	164.23	17000.77	16681.98	19575.29	19149.99	16130.26	16628.71
alanyl-tRNA synthetase 2, mitochondrial	Aars2	100754221	6.82	6.64	5.71	4.11	2.82	3.14	940.31	923.27	797.42	563.24	397.43	438.36
	Aasdh	100770305	13.56	12.98	10.96	10.62	11.54	13.20	603.07	591.11	494.35	514.56	541.30	596.32
aminoadipate-semialdehyde dehydrogenase-phosphopantetheinyl transferase	Aasdhppt	100754811	46.93	48.82	59.83	63.18	66.37	63.82	3527.30	3704.97	4554.73	4770.11	4978.28	4809.81
aminoadipate-semialdehyde synthase	Aass	100751161	0.01	0.01	0.00	0.00	0.00	0.00	0.76	0.92	0.00	0.00	0.00	0.00
apoptosis antagonizing transcription factor	Aatf	100758164	102.38	99.19	56.74	63.20	59.41	56.87	6519.88	6373.21	3691.48	4029.56	3768.39	3595.67
apoptosis-associated tyrosine kinase	Aatk	100753989	0.35	0.25	0.13	0.09	0.06	0.09	60.00	48.50	26.08	13.91	12.42	14.95
4-aminobutyrate aminotransferase	Abat	100765485	0.00	0.02	0.00	0.03	0.00	0.03	1.52	1.83	0.00	5.79	2.07	7.48
ATP-binding cassette, sub-family A (ABC1), member 1	Abca1	100761226	27.39	28.17	48.59	55.26	53.61	53.13	8550.89	8821.84	15386.97	17295.72	16690.19	16465.14
ATP-binding cassette, sub-family A (ABC1), member 13	Abca13	100753262	0.01	0.01	0.00	0.00	0.00	0.00	2.28	1.83	0.00	0.00	0.00	0.00
ATP-binding cassette, sub-family A (ABC1), member 2	Abca2	100772799	5.26	4.97	5.80	4.52	3.88	4.34	1306.41	1230.72	1452.00	1116.04	975.99	1083.28

ATP-binding cassette, sub-family A (ABC1), member 3	Abca3	100770749	0.00	0.00	0.00	0.01	0.00	0.00	0.00	0.00	3.73	2.32	0.00	0.00
ATP-binding cassette, sub-family A (ABC1), member 5	Abca5	100772052	2.21	2.24	2.91	3.44	4.89	4.44	352.43	361.44	462.06	542.37	768.99	702.87
ATP-binding cassette, sub-family A (ABC1), member 6	Abca6	100772341	0.01	0.03	0.01	0.01	0.00	0.00	3.04	5.49	2.48	4.64	2.07	0.00
ATP-binding cassette, sub-family A (ABC1), member 7	Abca7	100764464	1.30	1.16	1.68	1.37	1.12	1.21	307.61	281.83	387.53	327.97	252.54	282.27
ATP-binding cassette, sub-family A (ABC1), member 8	Abca8	100773221	0.01	0.00	0.00	0.00	0.00	0.00	0.76	0.00	0.00	0.00	0.00	0.00
ATP-binding cassette, sub-family A (ABC1), member 9	Abca9	100772634	0.18	0.19	0.00	0.01	0.00	0.01	32.66	39.35	0.00	1.16	0.00	0.93
ATP-binding cassette, sub-family B (MDR/TAP), member 10	Abcb10	100774734	2.79	2.39	2.75	2.63	3.10	2.72	305.33	261.70	305.55	288.57	332.23	299.09
ATP-binding cassette, sub-family B (MDR/TAP), member 11	Abcb11	100762480	0.00	0.02	0.00	0.01	0.00	0.00	0.00	1.83	1.24	1.16	0.00	0.00
ATP-binding cassette, sub-family B (MDR/TAP), member 1A	Abcb1a	100682536	26.99	27.77	30.62	33.06	35.32	33.55	3158.16	3317.00	3671.61	3841.82	4050.94	3910.66
ATP-binding cassette, sub-family B (MDR/TAP), member 1B	Abcb1b	100682537	5.62	5.26	5.86	7.99	8.20	7.60	353.95	346.80	448.39	527.31	560.96	537.44
ATP-binding cassette, sub-family B (MDR/TAP), member 4	Abcb4	100682535	0.26	0.24	0.19	0.20	0.14	0.19	0.76	0.00	0.00	0.00	0.00	0.00
ATP-binding cassette, sub-family B (MDR/TAP), member 6	Abcb6	100768549	12.84	11.57	13.09	13.48	13.12	12.49	1167.41	1086.14	1213.52	1234.25	1238.88	1161.79
ATP-binding cassette, sub-family B (MDR/TAP), member 7	Abcb7	100766714	12.04	11.84	10.73	11.69	13.69	12.87	2081.90	2043.27	1855.68	2036.22	2365.98	2239.47
ATP-binding cassette, sub-family B (MDR/TAP), member 8	Abcb8	100763667	7.25	7.40	6.31	5.26	4.01	4.63	1247.92	1290.20	1119.12	967.70	728.63	847.75
ATP-binding cassette, sub-family B (MDR/TAP), member 9	Abcb9	100772877	0.03	0.02	0.06	0.00	0.03	0.00	4.56	1.83	6.21	1.16	3.10	2.80
ATP-binding cassette, sub-family C (CFTR/MRP), member 1	Abcc1	100689208	14.21	14.06	14.81	14.28	12.15	13.54	2616.61	2609.68	2746.25	2649.29	2240.75	2513.33
ATP-binding cassette, sub-family C (CFTR/MRP), member 10	Abcc10	100757623	2.14	2.12	1.67	1.50	1.24	1.28	332.68	332.16	259.60	229.47	196.65	212.17
ATP-binding cassette, sub-family C (CFTR/MRP), member 2	Abcc2	100689220	0.10	0.10	0.07	0.08	0.09	0.11	15.95	20.13	12.42	15.07	18.63	21.50
ATP-binding cassette, sub-family C (CFTR/MRP), member 3	Abcc3	100767558	2.70	2.52	0.86	0.83	0.86	0.99	423.06	414.51	145.32	137.91	136.62	160.76
ATP-binding cassette, sub-family C (CFTR/MRP), member 4	Abcc4	100766754	16.96	16.60	17.04	18.73	19.86	18.40	2809.54	2769.81	2810.84	3053.75	3225.02	2983.47
ATP-binding cassette, sub-family C (CFTR/MRP), member 5	Abcc5	100774121	10.50	10.48	14.11	15.42	13.69	15.05	1495.53	1577.52	2219.61	2356.08	2134.14	2220.78
ATP-binding cassette, sub-family C (CFTR/MRP), member 6	Abcc6	100752070	0.06	0.12	0.01	0.00	0.01	0.00	12.15	16.47	2.48	1.16	1.03	0.00
ATP-binding cassette, sub-family C (CFTR/MRP), member 8	Abcc8	100764394	0.00	0.00	0.00	0.00	0.00	0.00	0.76	0.00	0.00	0.00	0.00	0.00
ATP-binding cassette, sub-family C (CFTR/MRP), member 9	Abcc9	100769198	0.00	0.00	0.00	0.00	0.00	0.00	0.00	0.00	0.00	0.00	0.00	0.00
ATP-binding cassette, sub-family D (ALD), member 1	Abcd1	100769988	2.33	2.26	2.99	2.38	2.51	2.52	246.09	241.57	320.46	244.53	274.27	259.84
ATP-binding cassette, sub-family D (ALD), member 2	Abcd2	100758232	2.70	2.24	2.47	2.98	3.37	3.25	252.17	216.86	236.00	288.57	317.74	301.90
ATP-binding cassette, sub-family D (ALD), member 3	Abcd3	100765314	19.40	20.57	15.57	17.33	17.33	16.53	1869.99	1990.20	1500.44	1638.71	1660.12	1582.40

ATP-binding cassette, sub-family D (ALD), member 4	Abcd4	100768387	5.74	5.11	5.24	6.06	4.28	5.37	424.58	395.29	416.10	471.68	371.56	393.50
ATP-binding cassette, sub-family E (OABP), member 1	Abce1	100773014	73.88	72.32	70.14	77.17	79.73	74.03	7953.89	7790.60	7568.04	8198.20	8446.52	7883.00
ATP-binding cassette, sub-family F (GCN20), member 1	Abcf1	100765933	101.40	99.86	105.74	96.01	71.67	75.84	10040.35	10078.18	10640.96	9471.85	7259.39	7540.92
ATP-binding cassette, sub-family F (GCN20), member 2	Abcf2	100760577	79.31	77.54	82.31	78.82	65.53	68.77	5578.06	5507.59	5893.70	5592.95	4659.51	4909.82
ATP-binding cassette, sub-family F (GCN20), member 3	Abcf3	100772769	19.03	17.92	20.25	20.52	17.81	19.24	1670.23	1632.42	1812.20	1825.30	1611.47	1716.99
ATP-binding cassette, sub-family G (WHITE), member 1	Abcg1	100759619	0.14	0.18	0.06	0.03	0.11	0.12	9.87	13.73	4.97	3.48	8.28	13.09
ATP-binding cassette, sub-family G (WHITE), member 2	Abcg2	100761503	0.00	0.00	0.02	0.00	0.00	0.00	0.00	0.00	1.24	0.00	0.00	0.00
ATP-binding cassette, sub-family G (WHITE), member 4	Abcg4	100756677	0.46	0.55	0.55	0.32	0.46	0.37	41.02	47.58	50.93	28.97	39.33	29.91
ATP-binding cassette, sub-family G (WHITE), member 5	Abcg5	100761230	0.00	0.02	0.00	0.02	0.00	0.02	0.00	0.92	0.00	1.16	0.00	0.93
ATP-binding cassette, sub-family G (WHITE), member 8	Abcg8	100765601	0.00	0.00	0.00	0.00	0.00	0.00	0.00	0.00	0.00	0.00	0.00	0.00
abhydrolase domain containing 1	Abhd1	100763847	2.42	2.37	2.73	3.14	3.03	3.28	549.15	532.55	621.04	710.42	712.07	732.78
abhydrolase domain containing 10	Abhd10	100770127	9.25	9.90	9.98	9.78	10.33	10.19	404.07	430.07	422.31	420.69	448.15	439.29
abhydrolase domain containing 11	Abhd11	100766777	22.75	21.88	25.25	21.76	20.28	22.58	938.79	915.95	1048.32	888.89	836.27	919.71
abhydrolase domain containing 12	Abhd12	100765125	13.11	13.38	17.98	17.45	16.66	15.67	690.42	711.90	950.20	920.18	863.18	814.10
abhydrolase domain containing 12B	Abhd12b	100752990	0.15	0.09	0.05	0.05	0.10	0.15	12.15	13.73	7.45	3.48	10.35	10.28
abhydrolase domain containing 13	Abhd13	100754934	9.59	9.25	10.80	10.21	12.20	10.82	729.92	694.51	818.54	790.38	925.28	819.71
abhydrolase domain containing 14A	Abhd14a	100765996	33.39	28.54	32.33	29.60	18.64	20.40	932.71	790.59	927.84	798.50	523.70	566.41
abhydrolase domain containing 14B	Abhd14b	100759415	9.33	8.72	7.43	6.44	6.50	8.21	296.22	284.58	249.66	225.99	230.80	282.27
abhydrolase domain containing 15	Abhd15	100758482	0.76	0.96	0.96	0.75	0.69	0.93	51.65	64.05	63.35	46.36	44.50	71.03
abhydrolase domain containing 16A	Abhd16a	100771419	13.54	12.97	15.15	10.49	7.06	7.83	1010.95	984.58	1145.20	773.00	547.51	604.73
abhydrolase domain containing 16B	Abhd16b	100762917	0.36	0.18	0.20	0.16	0.20	0.21	25.06	10.98	12.42	11.59	14.49	14.95
abhydrolase domain containing 17A	Abhd17a	100769196	5.55	5.81	5.58	3.00	2.18	2.37	707.89	746.67	719.17	375.49	263.92	302.83
abhydrolase domain containing 17B	Abhd17b	100773175	11.99	12.08	11.74	12.64	13.69	13.44	728.40	730.20	709.23	756.77	824.88	811.29
abhydrolase domain containing 17C	Abhd17c	100759841	35.44	35.75	43.98	45.85	47.45	46.99	1748.46	1778.83	2182.35	2245.99	2311.12	2298.35
abhydrolase domain containing 2	Abhd2	100755212	44.56	43.55	36.52	41.06	43.82	43.87	5719.33	5646.67	4741.05	5315.97	5648.96	5707.09
abhydrolase domain containing 3	Abhd3	100766111	0.00	0.00	0.00	0.00	0.00	0.00	0.00	0.00	0.00	0.00	0.00	0.00
abhydrolase domain containing 4	Abhd4	100767680	18.81	18.85	18.47	21.33	21.11	23.31	1235.77	1236.21	1222.21	1391.86	1373.43	1526.32
abhydrolase domain containing 5	Abhd5	100769851	18.48	19.34	18.69	21.48	22.84	21.37	1588.96	1701.96	1630.86	1846.16	1946.81	1841.30
abhydrolase domain containing 6	Abhd6	100769391	27.33	25.99	11.37	11.07	10.83	11.04	1806.94	1741.31	771.34	734.76	724.49	726.24
abhydrolase domain containing 8	Abhd8	100752213	7.72	7.14	7.71	4.34	3.09	3.41	582.57	521.57	573.84	319.86	224.59	255.16

abl-interactor 1	Abi1	100772463	21.00	21.90	24.59	25.69	26.39	25.69	1670.23	1771.50	1952.56	2022.32	1948.88	1955.33
abl-interactor 2	Abi2	100768975	6.96	6.61	6.07	7.11	7.90	7.88	1160.58	1112.68	1044.60	1209.91	1320.64	1340.32
ABI family, member 3	Abi3	100754696	0.00	0.00	0.00	0.00	0.00	0.00	0.76	0.92	0.00	0.00	1.03	0.93
ABI family, member 3 (NESH) binding protein	Abi3bp	100754709	0.02	0.02	0.04	0.00	0.01	0.00	1.52	4.58	6.21	1.16	1.03	4.67
c-abl oncogene 1, non-receptor tyrosine kinase	Abl1	100768098	11.93	11.53	11.44	9.89	7.80	8.32	1751.50	1729.41	1720.29	1472.99	1184.02	1281.43
c-abl oncogene 2, non-receptor tyrosine kinase	Abl2	100772469	10.12	9.66	7.92	8.41	7.97	9.33	3076.13	2847.58	2474.24	2633.07	2477.76	2908.69
actin binding LIM protein 1	Ablim1	100767228	0.02	0.00	0.00	0.00	0.00	0.00	0.76	0.00	0.00	0.00	0.00	0.00
actin binding LIM protein family, member 2	Ablim2	100774533	0.41	0.41	0.11	0.13	0.05	0.09	44.81	46.67	13.66	11.59	5.17	8.41
actin binding LIM protein family, member 3	Ablim3	100759449	0.00	0.00	0.00	0.00	0.00	0.00	0.00	0.00	0.00	0.00	0.00	0.00
ABO blood group (transferase A, alpha 1-3-N-acetylgalactosaminyltransferase; transferase B, alpha 1-3-galactosyltransferase)	Abo	100772592	0.07	0.05	0.09	0.09	0.04	0.07	3.04	1.83	4.97	4.64	2.07	5.61
active BCR-related	Abr	100762348	29.23	28.92	35.20	36.99	37.29	38.93	4240.51	4305.23	5200.62	5416.79	5474.04	5656.62
actin-binding Rho activating protein	Abra	100767760	0.00	0.00	0.00	0.00	0.00	0.00	0.00	0.00	0.00	0.00	1.03	0.00
ABRA C-terminal like	Abracl	100769731	52.28	53.97	52.74	52.15	58.70	56.75	1121.08	1188.63	1146.45	1133.42	1187.13	1163.66
activator of basal transcription 1	Abt1	100771876	20.79	19.82	18.19	16.06	14.57	13.50	1727.19	1659.87	1520.31	1332.76	1201.62	1117.86
ankyrin repeat and BTB (POZ) domain containing 1	Abtb1	100767751	2.13	1.86	2.23	1.77	1.12	1.30	152.67	132.68	155.26	129.80	79.69	94.40
ankyrin repeat and BTB (POZ) domain containing 2	Abtb2	100756314	0.25	0.28	0.02	0.02	0.00	0.01	45.57	43.01	14.91	19.70	14.49	12.15
acetyl-CoA acyltransferase 2	Acaa2	100765829	32.42	33.65	32.03	31.69	32.44	32.54	1405.15	1451.24	1384.93	1340.87	1333.06	1400.14
acetyl-CoA carboxylase alpha	Acaca	100689225	14.95	15.42	8.66	8.70	10.03	9.87						
acetyl-CoA carboxylase alpha	Acaca	100762904	23.74	23.63	12.73	13.67	13.28	13.71	6117.33	6183.80	3374.75	3534.71	3547.93	3599.41
acetyl-CoA carboxylase beta	Acacb	100753124	0.75	0.69	0.80	0.83	0.69	0.65	190.64	181.18	203.70	209.76	180.09	171.04
acyl-CoA dehydrogenase family, member 10	Acad10	100763570	6.21	6.13	7.15	6.54	5.40	6.07	672.95	679.87	793.69	723.17	596.15	666.42
acyl-CoA dehydrogenase family, member 11	Acad11	100763073	3.88	3.90	2.17	2.75	3.26	3.15	464.84	464.84	262.08	323.34	400.54	374.80
acyl-CoA dehydrogenase family, member 8	Acad8	100751213	7.74	8.16	8.51	8.46	8.37	8.78	468.64	489.54	542.79	540.06	526.81	548.65
acyl-CoA dehydrogenase family, member 9	Acad9	100770943	19.54	20.26	24.48	25.82	25.72	26.87	1604.15	1658.96	1992.31	2067.51	2086.53	2108.61
acyl-CoA dehydrogenase, long chain	Acadl	100759026	65.22	67.00	69.88	77.06	84.67	85.42	3395.14	3504.58	3652.98	3936.85	4346.94	4385.47
acyl-CoA dehydrogenase, C-4 to C-12 straight chain	Acadm	100763159	10.62	10.65	11.52	11.88	13.65	13.41	592.44	605.75	633.46	671.01	762.78	752.41
acyl-CoA dehydrogenase, C-2 to C-3 short chain	Acads	100774097	9.53	8.52	10.34	8.03	5.59	5.77	543.07	490.46	589.99	451.98	324.99	329.94
acyl-CoA dehydrogenase, short/branched chain	Acadsb	100766828	14.36	14.63	16.93	19.86	21.28	20.28	1606.43	1622.35	1883.00	2177.61	2301.81	2218.91
acyl-CoA dehydrogenase, very long chain	Acadvl	103158536	11.66	12.28	13.97	15.04	15.22	13.84	837.77	883.92	1017.27	1065.05	1097.09	955.23
aggrecan	Acan	100764201	0.00	0.00	0.00	0.00	0.00	0.00	0.00	0.00	0.00	0.00	0.00	0.00
ArfGAP with coiled-coil, ankyrin repeat and PH domains 1	Acap1	100765780	0.07	0.13	0.13	0.02	0.02	0.06	109.37	125.36	106.82	74.17	61.06	85.05

ArfGAP with coiled-coil, ankyrin repeat and PH domains 2	Acap2	100760438	15.21	15.79	16.97	19.45	20.70	19.66	1453.76	1530.85	1645.77	1857.75	2022.36	1874.95
ArfGAP with coiled-coil, ankyrin repeat and PH domains 3	Acap3	100768652	5.55	5.57	4.37	3.85	4.99	5.27	702.57	721.05	558.94	516.88	653.08	659.88
acetyl-CoA acetyltransferase 1	Acat1	100770936	92.85	89.18	84.70	96.87	104.23	102.88	4050.62	3962.10	3707.63	4160.52	4457.69	4444.35
acetyl-CoA acetyltransferase 2	Acat2	100760000	18.82	20.24	23.80	24.80	23.55	22.64	995.76	1077.91	1286.80	1364.05	1244.05	1208.53
acyl-CoA binding domain containing 3	Acbd3	100773815	24.37	24.58	23.10	23.74	26.32	24.82	2123.67	2149.41	2038.26	2055.92	2263.52	2135.72
acyl-CoA binding domain containing 4	Acbd4	100759909	12.61	11.55	9.21	7.61	6.62	6.81	976.01	881.18	698.05	581.78	506.11	530.89
acyl-CoA binding domain containing 5	Acbd5	100771023	16.27	15.99	13.35	15.76	17.48	17.14	1766.69	1773.33	1480.57	1694.34	1854.70	1801.11
acyl-CoA binding domain containing 6	Acbd6	100750555	59.37	60.57	66.37	68.41	62.01	61.88	1546.42	1607.71	1737.68	1663.05	1571.11	1535.66
acyl-CoA binding domain containing 7	Acbd7	100752791	0.00	0.00	0.00	0.00	0.00	0.00	0.00	0.00	0.00	0.00	1.03	0.00
1-aminocyclopropane-1-carboxylate synthase homolog (Arabidopsis)(non-functional)	Accs	100774985	0.03	0.00	0.02	0.07	0.00	0.02	1.52	0.00	1.24	3.48	2.07	0.93
adrenocortical dysplasia homolog (mouse)	Acd	100763518	47.22	46.60	56.33	50.48	43.77	50.43	1917.84	1948.11	2363.69	2073.31	1832.96	2053.47
angiotensin I converting enzyme (peptidyl-dipeptidase A) 1	Ace	100772844	0.00	0.02	0.00	0.02	0.00	0.00	0.00	0.92	0.00	1.16	0.00	0.00
angiotensin I converting enzyme 2	Ace2	100772552	0.49	0.53	0.23	0.24	0.36	0.53	51.65	53.99	27.33	25.50	38.29	53.28
angiotensin I converting enzyme (peptidyl-dipeptidase A) 3	Ace3	100753209	0.00	0.02	0.00	0.00	0.00	0.02	0.00	1.83	4.97	2.32	4.14	2.80
alkaline ceramidase 1	Acer1	100773997	0.13	0.11	0.28	0.17	0.29	0.35	29.62	24.71	28.57	18.54	23.80	35.52
alkaline ceramidase 2	Acer2	100758912	0.00	0.00	0.00	0.00	0.00	0.00	0.00	0.00	0.00	0.00	0.00	0.00
alkaline ceramidase 3	Acer3	100766923	6.24	6.27	7.70	8.83	9.43	9.47	385.09	387.06	519.19	569.03	603.40	606.60
acetylcholinesterase (Yt blood group)	Ache	100770679	0.02	0.00	0.00	0.00	0.00	0.00	1.52	0.00	1.24	0.00	0.00	0.00
apoptotic chromatin condensation inducer 1	Acin1	100769875	44.68	45.58	55.66	54.30	53.25	54.58	6537.35	6731.90	8333.16	8085.78	7827.60	7997.97
atypical chemokine receptor 1 (Duffy blood group)	Ackr1	100757110	0.00	0.00	0.00	0.00	0.00	0.00	0.00	0.00	0.00	0.00	0.00	0.00
atypical chemokine receptor 2	Ackr2	100766393	0.00	0.00	0.00	0.00	0.00	0.00	0.00	0.00	0.00	0.00	0.00	0.00
atypical chemokine receptor 3	Ackr3	100774656	18.86	18.61	11.57	12.42	10.75	11.89	1030.69	1024.84	642.16	677.97	586.84	654.27
atypical chemokine receptor 4	Ackr4	100762778	0.43	0.33	0.48	0.30	0.46	0.58	16.71	13.73	19.87	15.07	20.70	23.37
ATP citrate lyase	Acly	100773325	221.56	195.94	257.90	272.44	260.31	269.19	8311.64	8433.86	10314.29	9321.19	8807.73	9065.36
aminocarboxymuconate semialdehyde decarboxylase	Acmsd	100771266	0.00	0.00	0.00	0.00	0.00	0.00	0.00	0.00	0.00	0.00	0.00	0.00
ACN9 homolog (S. cerevisiae)	Acn9	100767267	27.05	27.39	34.73	36.06	36.46	30.84	297.74	301.96	398.71	399.83	436.76	352.37
aconitase 1, soluble	Aco1	100766024	7.96	8.11	9.12	8.95	9.32	9.86	905.37	940.65	1059.50	1034.92	1076.39	1127.21
aconitase 2, mitochondrial	Aco2	100759203	116.57	114.31	117.96	112.25	97.56	106.59	9657.54	9490.73	9811.24	9247.02	8054.26	8802.72
acyl-CoA thioesterase 11	Acot11	100752371	0.42	0.38	0.39	0.48	0.31	0.46	27.34	26.54	28.57	31.29	22.77	23.37
acyl-CoA thioesterase 12	Acot12	100752768	0.02	0.00	0.00	0.00	0.00	0.00	3.80	4.58	0.00	1.16	4.14	1.87
acyl-CoA thioesterase 13	Acot13	100758652	15.85	17.09	10.56	10.06	13.58	11.40	240.01	266.27	165.20	156.45	203.89	171.98
acyl-CoA thioesterase 4	Acot4	100773433	0.00	0.00	0.00	0.00	0.00	0.00	0.00	1.83	0.00	0.00	0.00	0.00
acyl-CoA thioesterase 7	Acot7	100755154	62.54	57.47	51.01	42.00	30.49	31.13	2469.26	2301.31	2045.72	1631.76	1200.58	1232.83
acyl-CoA thioesterase 9	Acot9	100761882	32.93	34.71	31.05	34.57	40.41	40.08	1674.78	1798.96	1591.11	1759.24	2086.53	2035.71
acyl-CoA oxidase 1, palmitoyl	Acox1	100689199	9.56	10.37	11.26	14.10	15.18	14.00	975.25	1057.78	1150.17	1386.07	1477.96	1417.89
acyl-CoA oxidase 2, branched chain	Acox2	100766043	0.06	0.06	0.05	0.08	0.07	0.02	9.11	5.49	4.97	6.95	8.28	4.67

acyl-CoA oxidase 3, pristanoyl	Acox3	100767060	17.71	17.83	21.13	20.05	17.86	18.54	1542.62	1510.72	1849.47	1765.03	1632.17	1696.43
acyl-CoA oxidase-like	Acoxl	100772220	0.00	0.00	0.00	0.00	0.00	0.03	0.00	0.00	0.00	1.16	0.00	2.80
acid phosphatase 1, soluble	Acp1	100773599	102.13	102.03	101.33	106.86	100.09	103.69	3818.96	3812.94	3784.64	3935.69	3696.97	3827.47
acid phosphatase 2, lysosomal	Acp2	100756404	30.31	30.03	29.53	29.77	29.08	30.99	1646.68	1609.54	1634.59	1615.53	1547.30	1615.11
acid phosphatase 5, tartrate resistant	Acp5	100768228	14.23	13.15	15.68	14.10	12.01	11.81	675.99	617.65	744.01	645.52	541.30	550.52
acid phosphatase 6, lysophosphatidic	Acp6	100751531	13.04	11.58	13.17	11.83	10.95	11.50	663.84	600.26	671.97	613.07	553.72	595.38
acid phosphatase, prostate	Acpp	100763644	0.13	0.08	0.03	0.05	0.02	0.00	8.35	5.49	2.48	4.64	2.07	0.93
acid phosphatase, testicular	Acpt	100757346	0.00	0.00	0.00	0.00	0.00	0.00	0.76	0.00	0.00	0.00	0.00	0.93
acrosin	Acr	100754856	2.45	2.73	1.19	1.55	1.01	1.22	80.51	82.35	27.33	37.09	36.22	37.39
acrosin binding protein	Acrbp	100754463	0.24	0.20	0.19	0.30	0.10	0.11	19.75	16.47	14.91	25.50	16.56	15.89
acrosomal vesicle protein 1	Acrv1	100752850	0.00	0.00	0.00	0.00	0.00	0.00	0.76	0.00	0.00	2.32	1.03	0.93
acyl-CoA synthetase bubblegum family member 1	Acsbg1	100754767	0.00	0.03	0.06	0.01	0.01	0.06	6.84	8.24	8.69	6.95	13.45	15.89
acyl-CoA synthetase bubblegum family member 2	Acsbg2	100773126	0.00	0.00	0.00	0.00	0.00	0.00	0.00	0.00	0.00	0.00	0.00	0.00
acyl-CoA synthetase family member 2	Acsf2	100768417	4.45	4.59	2.92	2.69	2.80	2.70	311.41	324.84	209.91	183.11	199.75	182.26
acyl-CoA synthetase family member 3	Acsf3	100764604	9.22	8.81	12.66	11.34	10.10	11.25	629.66	614.90	886.85	805.45	685.16	758.95
acyl-CoA synthetase long-chain family member 1	Acsl1	100751492	7.97	7.94	7.48	7.81	7.94	7.55	899.29	893.07	850.83	869.19	888.02	836.53
acyl-CoA synthetase long-chain family member 3	Acsl3	100752583	4.93	5.22	7.91	8.48	9.28	8.52	426.86	464.84	690.60	728.96	808.32	732.78
acyl-CoA synthetase long-chain family member 4	Acsl4	100760496	26.92	28.16	29.57	31.50	32.45	31.97	3149.81	3333.47	3482.81	3701.59	3805.64	3811.58
acyl-CoA synthetase long-chain family member 5	Acsl5	100773264	163.67	164.91	188.97	201.29	187.56	191.14	14023.37	14157.40	16235.32	17029.17	15888.07	16139.88
acyl-CoA synthetase long-chain family member 6	Acsl6	100762894	1.94	1.85	1.03	1.10	1.72	1.70	351.67	341.31	202.46	220.19	320.85	319.66
acyl-CoA synthetase medium-chain family member 3	Acsm3	100751305	0.00	0.00	0.00	0.00	0.00	0.00	0.00	0.00	0.00	0.00	0.00	0.93
acyl-CoA synthetase medium-chain family member 5	Acsm5	100755162	0.00	0.00	0.00	0.00	0.00	0.00	0.00	0.00	0.00	0.00	0.00	0.00
acyl-CoA synthetase short-chain family member 1	Acss1	100769754	0.00	0.00	0.00	0.00	0.00	0.02	0.00	0.92	0.00	0.00	0.00	0.93
acyl-CoA synthetase short-chain family member 2	Acss2	100752902	5.45	5.52	5.82	6.04	6.08	5.86	566.62	582.88	599.93	645.52	638.59	653.33
acyl-CoA synthetase short-chain family member 3	Acss3	100772576	0.10	0.04	0.05	0.03	0.00	0.00	6.84	3.66	3.73	2.32	0.00	0.00
actin, alpha 1, skeletal muscle	Acta1	100768972	0.13	0.21	0.07	0.23	0.15	0.16	4.56	11.90	8.69	11.59	8.28	7.48
actin, alpha 2, smooth muscle, aorta	Acta2	100758575	0.18	0.30	0.18	0.03	0.19	0.17	7.60	13.73	6.21	3.48	7.24	5.61
actin, beta	Actb	100689477	1016.39	1018.05	1063.28	923.58	709.29	780.80	44003.90	43970.11	47470.07	42200.91	35481.40	39057.98
actin, alpha, cardiac muscle 1	Actc1	100766388	0.25	0.29	0.00	0.00	0.00	0.00	10.63	12.81	0.00	0.00	0.00	0.00
actin, gamma 1	Actg1	100764022	1195.18	1116.64	1101.58	881.78	643.89	739.69	19495.85	18448.91	18283.52	14314.98	9534.29	11240.34
actin, gamma 2, smooth muscle, enteric	Actg2	100768744	0.07	0.11	0.15	0.11	0.10	0.03	25.82	36.60	21.12	23.18	30.01	32.71
actin-like 10	Actl10	103160901	0.26	0.15	0.47	0.13	0.19	0.27	14.43	9.15	27.33	10.43	16.56	19.63
actin-like 6A	Actl6a	100757342	34.90	35.16	35.54	37.88	36.84	34.48	1932.27	1929.81	1988.58	2057.08	2009.94	1936.64
actin-like 6B	Actl6b	100753067	0.11	0.03	0.11	0.11	0.04	0.03	9.87	8.24	8.69	11.59	5.17	5.61
actin-like 7A	Actl7a	100767459	0.02	0.03	0.00	0.00	0.03	0.00	0.76	0.92	1.24	0.00	1.03	0.00
actin-like 7B	Actl7b	100767172	0.00	0.00	0.00	0.04	0.03	0.03	0.00	1.83	0.00	1.16	1.03	0.93

actin-like 9	Actl9	100752462	0.00	0.00	0.00	0.00	0.00	0.00	0.00	0.92	0.00	0.00	0.00	0.00
actinin, alpha 1	Actn1	100755831	57.65	55.27	52.34	46.38	37.94	42.03	5451.21	5170.85	4868.98	4303.07	3666.96	4007.86
actinin, alpha 2	Actn2	100765161	0.04	0.01	0.00	0.00	0.00	0.00	3.04	0.92	0.00	0.00	0.00	0.00
actinin, alpha 4	Actn4	100766890	27.28	27.66	25.10	20.69	15.60	17.12	1974.80	1960.92	1845.74	1491.53	1151.94	1265.54
actin-related protein 10 homolog (S. cerevisiae)	Actr10	100759261	46.28	46.26	44.03	46.81	55.32	54.19	2134.31	2177.78	2068.07	2139.37	2545.03	2451.64
ARP1 actin-related protein 1 homolog A, centractin alpha (yeast)	Actr1a	100750704	68.89	66.93	66.77	67.39	63.19	69.61	5224.11	5176.34	5127.33	5152.56	4845.81	5298.64
ARP1 actin-related protein 1 homolog B, centractin beta (yeast)	Actr1b	100768031	9.46	9.37	11.97	11.73	9.42	9.80	672.19	667.97	850.83	810.08	657.22	698.20
ARP2 actin-related protein 2 homolog (yeast)	Actr2	100752591	85.31	88.46	84.02	97.73	101.04	103.66	3183.99	3304.19	3231.91	3652.92	3859.46	3912.53
ARP3 actin-related protein 3 homolog (yeast)	Actr3	100759010	157.42	161.81	158.76	167.14	174.97	170.43	11599.68	12049.16	11896.71	12348.29	12835.90	12444.19
ARP5 actin-related protein 5 homolog (yeast)	Actr5	100765489	20.22	20.94	18.90	18.09	17.68	18.30	1256.28	1306.67	1165.08	1110.25	1139.52	1187.03
ARP6 actin-related protein 6 homolog (yeast)	Actr6	100762739	17.70	19.08	18.51	18.96	22.05	21.78	1015.50	1096.21	1074.41	1082.43	1268.89	1259.93
ARP8 actin-related protein 8 homolog (yeast)	Actr8	100771927	13.87	12.44	13.97	15.04	15.85	15.10	882.58	810.72	880.64	958.43	991.52	942.15
actin-related protein T1	Actrt1	100772817	0.00	0.00	0.00	0.00	0.00	0.00	0.00	0.00	0.00	0.00	0.00	0.00
actin-related protein T3	Actrt3	100768343	0.55	0.53	0.53	0.43	0.50	0.45	46.33	45.75	44.72	37.09	41.40	36.45
activin A receptor, type I	Acvr1	100758565	17.28	17.75	18.76	19.86	20.77	19.87	1328.43	1369.81	1419.71	1507.75	1563.86	1504.82
activin A receptor, type IB	Acvr1b	100764812	14.35	11.99	10.75	13.11	10.10	10.77	568.14	489.54	435.97	513.40	408.82	445.84
activin A receptor, type IC	Acvr1c	100756930	0.00	0.00	0.04	0.00	0.00	0.00	0.76	0.00	1.24	0.00	0.00	0.00
activin A receptor, type IIA	Acvr2a	100774605	3.02	2.55	2.54	2.68	3.10	3.16	547.63	472.16	470.75	487.91	562.00	587.91
activin A receptor, type IIB	Acvr2b	100755103	1.86	1.68	1.33	1.12	0.71	0.72	110.89	100.65	79.49	55.63	34.15	32.71
activin A receptor type II-like 1	Acvrl1	100764896	0.00	0.00	0.00	0.00	0.00	0.00	0.00	0.00	0.00	0.00	0.00	0.00
aminoacylase 1	Acy1	100765707	26.54	27.69	33.85	29.83	23.54	26.27	1223.62	1267.32	1530.25	1274.81	1054.65	1136.56
aspartoacylase (aminocyclase) 3	Acy3	100763612	1.92	2.16	0.61	0.57	0.26	0.40	109.37	103.40	40.99	31.29	18.63	16.82
acylphosphatase 1, erythrocyte (common) type	Acyp1	100755378	24.50	23.84	11.97	14.61	14.92	15.07	328.88	330.33	177.62	194.70	187.33	195.35
acylphosphatase 2, muscle type	Acyp2	100758982	1.80	2.61	2.97	3.12	2.95	3.20	69.12	91.50	114.27	117.05	109.71	115.90
adenosine deaminase	Ada	100756254	16.94	17.78	18.08	18.44	16.56	17.97	1068.67	1149.28	1175.01	1183.26	1059.83	1158.06
adenosine deaminase domain containing 1 (testis-specific)	Adad1	100754029	0.00	0.00	0.00	0.00	0.00	0.00	0.00	0.00	0.00	0.00	0.00	0.00
adenosine deaminase domain containing 2	Adad2	100773843	0.00	0.00	0.00	0.00	0.00	0.00	0.00	0.00	0.00	0.00	0.00	0.00
adenosine deaminase-like	Adal	100755738	14.70	14.76	15.48	15.04	18.01	16.74	973.73	916.86	945.23	1012.90	1202.65	1154.32
ADAM metallopeptidase domain 10	Adam10	100772891	25.08	25.20	24.50	25.92	29.16	27.08	3252.35	3270.33	3204.58	3342.32	3762.18	3475.10
ADAM metallopeptidase domain 11	Adam11	100764563	0.00	0.00	0.00	0.00	0.00	0.00	0.00	0.00	0.00	0.00	0.00	0.00
ADAM metallopeptidase domain 12	Adam12	100759770	2.67	1.71	0.01	0.00	0.00	0.00	201.28	180.26	2.48	0.00	0.00	0.00
ADAM metallopeptidase domain 15	Adam15	100773702	29.81	27.52	36.50	32.82	26.37	30.08	2750.29	2599.61	3438.10	2971.47	2358.73	2709.61
ADAM metallopeptidase domain 17	Adam17	100684971	26.63	25.29	30.20	31.24	32.58	31.00	2578.64	2501.70	2953.68	3002.76	3215.70	3037.68
ADAM metallopeptidase domain 19	Adam19	100762809	28.46	27.79	39.19	39.96	34.99	36.67	3402.74	3406.67	4805.63	4876.73	4276.56	4388.27

ADAM metallopeptidase domain 2	Adam2	100756746	0.00	0.00	0.00	0.00	0.00	0.00	0.00	0.00	0.00	0.00	0.00	0.00
ADAM metallopeptidase domain 21	Adam21	100763081	0.01	0.02	0.00	0.00	0.02	0.00	0.76	0.92	0.00	0.00	1.03	0.00
ADAM metallopeptidase domain 22	Adam22	100751750	8.93	9.02	12.34	13.96	13.63	13.53	1860.87	1890.46	2599.69	2941.34	2853.46	2799.34
ADAM metallopeptidase domain 23	Adam23	100756536	12.72	13.39	17.75	19.36	20.78	20.27	2275.58	2423.92	3197.13	3462.85	3669.03	3558.29
ADAM metallopeptidase domain 28	Adam28	100773280	0.00	0.00	0.00	0.00	0.00	0.00	0.00	0.00	0.00	0.00	0.00	0.00
ADAM metallopeptidase domain 29	Adam29	100759070	0.00	0.00	0.00	0.00	0.00	0.00	0.00	0.00	0.00	0.00	0.00	0.00
ADAM metallopeptidase domain 30	Adam30	100771496	0.00	0.00	0.00	0.02	0.00	0.00	0.76	0.00	0.00	1.16	0.00	0.00
ADAM metallopeptidase domain 32	Adam32	100754956	0.00	0.01	0.00	0.02	0.01	0.00	0.00	0.92	1.24	1.16	1.03	0.00
ADAM metallopeptidase domain 33	Adam33	100773977	0.01	0.02	0.00	0.00	0.00	0.03	4.56	8.24	0.00	3.48	1.03	4.67
ADAM metallopeptidase domain 7	Adam7	100773564	0.00	0.00	0.00	0.00	0.00	0.00	0.00	0.00	0.00	0.00	0.00	0.00
ADAM metallopeptidase domain 8	Adam8	100770949	0.02	0.06	0.00	0.01	0.01	0.01	3.04	5.49	3.73	1.16	1.03	1.87
ADAM metallopeptidase domain 9	Adam9	100754662	48.41	48.30	42.77	44.65	50.49	48.47	4854.22	4861.57	4312.53	4486.18	5067.29	4903.28
ADAM-like, decysin 1	Adamdec1	100752574	0.00	0.00	0.00	0.00	0.00	0.00	0.00	0.00	0.00	0.00	0.00	0.00
ADAM metallopeptidase with thrombospondin type 1 motif, 1	Adamts 1	100761390	3.20	3.29	2.56	2.80	2.68	2.74	493.70	506.01	396.23	443.87	406.75	429.01
ADAM metallopeptidase with thrombospondin type 1 motif, 10	Adamts 10	100752765	0.00	0.00	0.00	0.00	0.00	0.00	2.28	0.92	1.24	0.00	0.00	0.00
ADAM metallopeptidase with thrombospondin type 1 motif, 12	Adamts 12	100772449	0.00	0.00	0.00	0.00	0.00	0.00	0.00	0.00	0.00	1.16	0.00	0.00
ADAM metallopeptidase with thrombospondin type 1 motif, 13	Adamts 13	100770010	0.02	0.02	0.00	0.00	0.00	0.02	3.04	3.66	0.00	0.00	0.00	3.74
ADAM metallopeptidase with thrombospondin type 1 motif, 14	Adamts 14	100768329	0.00	0.00	0.00	0.00	0.00	0.00	0.76	0.00	0.00	0.00	0.00	0.00
ADAM metallopeptidase with thrombospondin type 1 motif, 15	Adamts 15	100754358	0.01	0.01	0.00	0.00	0.00	0.00	0.76	1.83	0.00	0.00	1.03	0.00
ADAM metallopeptidase with thrombospondin type 1 motif, 16	Adamts 16	100761157	0.00	0.00	0.00	0.00	0.00	0.00	0.00	0.00	0.00	0.00	0.00	0.00
ADAM metallopeptidase with thrombospondin type 1 motif, 17	Adamts 17	100770235	0.01	0.00	0.00	0.00	0.00	0.00	0.76	0.00	1.24	0.00	0.00	0.00
ADAM metallopeptidase with thrombospondin type 1 motif, 18	Adamts 18	100763192	0.00	0.00	0.00	0.00	0.00	0.00	0.00	0.00	0.00	0.00	0.00	0.00
ADAM metallopeptidase with thrombospondin type 1 motif, 19	Adamts 19	100773163	0.00	0.00	0.00	0.00	0.00	0.00	0.00	0.00	0.00	0.00	0.00	0.00
ADAM metallopeptidase with thrombospondin type 1 motif, 2	Adamts 2	100769264	0.00	0.00	0.00	0.00	0.00	0.00	0.00	0.00	0.00	0.00	0.00	0.00
ADAM metallopeptidase with thrombospondin type 1 motif, 20	Adamts 20	100750834	0.01	0.03	0.00	0.00	0.00	0.00	0.76	2.75	0.00	0.00	0.00	0.00
ADAM metallopeptidase with thrombospondin type 1 motif, 3	Adamts 3	100758325	0.30	0.26	0.58	0.75	0.78	0.74	51.65	43.01	104.34	127.48	129.37	126.18
ADAM metallopeptidase with thrombospondin type 1 motif, 4	Adamts 4	100773045	0.43	0.42	0.36	0.16	0.25	0.34	58.48	62.22	53.41	27.81	42.43	56.08
ADAM metallopeptidase with thrombospondin type 1 motif, 5	Adamts 5	100761876	0.00	0.00	0.00	0.01	0.00	0.00	0.00	0.00	0.00	3.48	0.00	0.00
ADAM metallopeptidase with thrombospondin type 1 motif, 6	Adamts 6	100760416	4.51	4.47	6.78	7.92	8.51	7.99	988.92	999.22	1506.65	1739.54	1885.75	1750.64

ADAM metallopeptidase with thrombospondin type 1 motif, 7	Adamts 7	100756390	3.64	3.57	3.21	2.73	1.97	2.09	602.31	598.43	537.82	446.18	321.88	343.96
ADAM metallopeptidase with thrombospondin type 1 motif, 8	Adamts 8	100754641	0.00	0.00	0.00	0.00	0.00	0.01	2.28	0.00	1.24	1.16	0.00	1.87
ADAM metallopeptidase with thrombospondin type 1 motif, 9	Adamts 9	100768091	0.00	0.00	0.00	0.00	0.00	0.00	0.00	0.00	0.00	0.00	1.03	0.00
ADAMTS-like 1	Adamtsl 1	100757121	0.06	0.08	0.03	0.02	0.01	0.01	9.11	10.98	4.97	3.48	2.07	0.93
ADAMTS-like 2	Adamtsl 2	100761699	0.00	0.00	0.00	0.00	0.00	0.00	0.00	0.00	0.00	0.00	0.00	0.00
ADAMTS-like 4	Adamtsl 4	100763942	6.77	5.93	4.10	3.34	2.49	2.60	827.90	732.94	503.05	433.44	302.22	334.61
ADAMTS-like 5	Adamtsl 5	100767851	8.58	8.26	7.65	7.88	6.17	6.04	987.40	927.84	871.94	839.06	629.27	627.16
ArfGAP with dual PH domains 1	Adap1	100770927	0.03	0.05	0.00	0.02	0.00	0.02	2.28	3.66	1.24	2.32	1.03	1.87
ArfGAP with dual PH domains 2	Adap2	100772203	0.00	0.04	0.00	0.00	0.00	0.00	0.00	0.92	0.00	0.00	0.00	0.00
hydrogen peroxide-induced mRNA	Adapt15	100689050	1486.68	1471.03	737.25	734.48	715.60	681.23	31806.46	31223.69	15548.44	15209.66	15065.26	14363.07
adenosine deaminase, RNA-specific	Adar	100760578	11.28	10.78	11.78	12.38	11.61	12.24	1576.80	1513.47	1676.82	1734.90	1633.21	1742.22
adenosine deaminase, RNA-specific, B1	Adarb1	100755658	4.03	3.74	4.67	4.04	3.40	3.60	761.06	720.13	875.67	778.79	640.66	672.96
adenosine deaminase, RNA-specific, B2 (non-functional)	Adarb2	100759087	0.00	0.00	0.00	0.00	0.00	0.00	0.00	0.00	0.00	0.00	0.00	0.00
adenosine deaminase, tRNA-specific 1	Adat1	100767121	9.23	10.58	12.18	13.07	11.15	10.68	516.49	581.05	635.95	690.72	593.05	574.82
adenosine deaminase, tRNA-specific 2	Adat2	100759031	6.36	6.25	9.63	9.45	9.38	9.88	598.52	597.52	1130.30	1095.18	1090.88	1170.21
adenosine deaminase, tRNA-specific 3	Adat3	100769481	3.57	3.22	2.90	1.62	1.45	1.58	272.67	247.97	224.82	141.39	122.13	134.59
	Adc	100774865	0.00	0.00	0.00	0.00	0.00	0.00	0.00	0.00	0.00	0.00	0.00	0.00
aarF domain containing kinase 1	Adck1	100759124	2.67	2.56	2.18	1.71	1.56	1.72	176.97	169.28	144.08	110.10	99.36	114.96
aarF domain containing kinase 2	Adck2	100771195	14.37	14.28	15.79	14.34	13.45	13.81	883.34	885.75	978.76	864.55	832.13	869.24
aarF domain containing kinase 3	Adck3	100752533	1.75	1.67	0.98	1.41	0.55	0.82	135.20	126.27	77.01	103.14	43.47	68.23
aarF domain containing kinase 4	Adck4	100770066	3.07	2.90	3.63	3.30	1.46	1.67	152.67	131.76	186.31	150.66	79.69	104.68
aarF domain containing kinase 5	Adck5	100762357	13.52	13.06	14.53	12.66	9.67	9.82	865.87	878.43	994.91	841.38	625.13	634.64
adenylate cyclase 1 (brain)	Adcy1	100764561	0.00	0.00	0.00	0.00	0.00	0.00	0.00	0.00	0.00	0.00	0.00	0.00
adenylate cyclase 10 (soluble)	Adcy10	100767272	0.02	0.00	0.03	0.01	0.00	0.00	5.32	0.92	3.73	3.48	1.03	0.00
adenylate cyclase 2 (brain)	Adcy2	100773811	0.00	0.00	0.00	0.00	0.00	0.00	0.00	0.00	0.00	0.00	0.00	0.00
adenylate cyclase 3	Adcy3	100770498	0.13	0.02	0.05	0.02	0.08	0.10	12.15	9.15	6.21	6.95	14.49	18.69
adenylate cyclase 4	Adcy4	100768998	0.02	0.00	0.00	0.00	0.00	0.00	1.52	0.00	0.00	0.00	1.03	0.00
adenylate cyclase 5	Adcy5	100765958	0.00	0.00	0.00	0.01	0.00	0.00	2.28	4.58	0.00	1.16	2.07	1.87
adenylate cyclase 6	Adcy6	100768368	7.99	8.06	9.99	9.11	8.09	8.61	1383.12	1397.26	1752.58	1556.43	1392.06	1484.26
adenylate cyclase 7	Adcy7	100756132	8.06	7.88	5.41	5.28	5.15	6.15	1192.48	1134.64	821.02	799.65	701.72	849.61
adenylate cyclase 9	Adcy9	100751372	1.52	1.44	1.13	1.43	1.58	1.57	312.17	299.22	229.79	289.73	330.16	330.87
adenylate cyclase activating polypeptide 1 (pituitary)	Adcyap 1	100760233	0.00	0.00	0.00	0.00	0.00	0.00	0.00	0.00	0.00	0.00	0.00	0.00
adenylate cyclase activating polypeptide 1 (pituitary) receptor type I	Adcyap 1r1	100772260	0.00	0.00	0.00	0.00	0.00	0.00	0.00	0.92	0.00	0.00	0.00	0.00
adducin 1 (alpha)	Add1	100768168	59.46	57.58	58.95	62.95	68.74	71.01	6565.46	6418.05	6566.91	6747.23	6917.85	7212.85

adducin 2 (beta)	Add2	100753858	1.91	2.07	0.31	0.36	0.43	0.56	216.47	231.50	36.02	46.36	51.75	67.30
adducin 3 (gamma)	Add3	100754769	56.49	56.47	78.30	85.79	91.55	92.09	3661.74	3747.98	4789.49	5280.04	5680.00	5549.13
alcohol dehydrogenase 5 (class III), chi polypeptide	Adh5	100767929	83.54	79.88	77.69	78.69	77.10	80.45	3225.76	3116.60	2990.95	2994.65	2972.48	3066.65
alcohol dehydrogenase 7 (class IV), mu or sigma polypeptide	Adh7	100759210	0.02	0.02	0.00	0.00	0.00	0.00	2.28	1.83	0.00	0.00	0.00	0.00
alcohol dehydrogenase, iron containing, 1	Adhfe1	100764101	0.42	0.40	0.30	0.44	0.33	0.37	44.05	37.52	27.33	45.20	36.22	37.39
acireductone dioxygenase 1	Adi1	100765075	23.64	23.95	17.36	16.62	16.33	14.17	415.47	423.66	327.91	294.37	296.01	270.12
adipogenin	Adig	100765200	0.00	0.00	0.00	0.00	0.00	0.00	0.00	0.00	0.00	0.00	0.00	0.00
adiponectin, C1Q and collagen domain containing	Adipoq	100763032	0.00	0.00	0.00	0.00	0.00	0.06	0.00	0.00	0.00	0.00	0.00	0.93
adiponectin receptor 1	Adipor1	100752505	82.22	86.63	73.50	77.83	80.56	80.17	4996.25	5260.53	4472.75	4686.67	4815.79	4814.48
adiponectin receptor 2	Adipor2	100752717	39.13	39.04	40.14	39.19	39.61	40.40	4300.51	4321.70	4475.24	4328.57	4358.33	4471.46
adenosine kinase	Adk	100736551	63.48	63.72	58.36	63.41	67.82	64.50	2665.22	2634.38	2388.53	2480.09	2658.88	2572.21
adrenomedullin	Adm	100760129	0.32	0.44	0.89	1.09	0.69	0.71	12.15	16.47	33.54	42.88	25.87	28.04
adrenomedullin 2	Adm2	100755992	0.00	0.00	0.00	0.00	0.00	0.00	0.00	0.00	0.00	0.00	0.00	0.00
activity-dependent neuroprotector homeobox	Adnp	100757883	40.60	39.53	35.10	38.00	40.71	39.39	5652.49	5536.87	4903.76	5309.01	5659.31	5517.36
ADNP homeobox 2	Adnp2	100760580	11.42	11.04	11.01	11.22	9.39	10.59	1453.00	1413.73	1419.71	1450.97	1210.93	1384.25
adenosine A1 receptor	Adora1	100751346	0.00	0.04	0.00	0.00	0.00	0.00	0.00	0.92	0.00	0.00	0.00	0.00
adenosine A2a receptor	Adora2a	100762652	0.16	0.15	0.32	0.30	0.15	0.22	6.84	7.32	13.66	13.91	6.21	9.35
adenosine A2b receptor	Adora2b	100759954	0.42	0.36	0.18	0.13	0.14	0.09	30.38	23.79	13.66	11.59	10.35	7.48
adenosine A3 receptor	Adora3	100773958	0.00	0.00	0.00	0.00	0.00	0.00	0.76	0.00	0.00	0.00	2.07	0.93
ADP-dependent glucokinase	Adpgk	100754978	30.00	30.01	31.51	32.69	31.10	32.28	2098.61	2129.28	2220.85	2296.98	2187.96	2283.40
ADP-ribosylarginine hydrolase	Adprh	100761196	167.59	163.02	187.21	166.70	145.93	152.69	7580.96	7421.84	8557.98	7493.58	6564.92	6904.41
ADP-ribosylhydrolase like 1	Adprhl1	100757894	0.00	0.00	0.00	0.00	0.00	0.00	0.00	0.00	0.00	0.00	0.00	0.00
ADP-ribosylhydrolase like 2	Adprhl2	100762798	29.72	27.27	31.15	26.55	17.59	21.47	1401.35	1280.13	1473.11	1247.00	814.53	995.42
ADP-ribose/CDP-alcohol diphosphatase, manganese-dependent	Adprm	100767531	3.32	3.54	4.02	4.16	5.12	3.91	132.16	135.42	168.92	166.88	195.61	153.29
adrenoceptor alpha 1A	Adra1a	100758859	0.00	0.00	0.00	0.00	0.00	0.00	0.00	0.00	0.00	0.00	0.00	0.00
adrenoceptor alpha 1B	Adra1b	103160164	0.00	0.00	0.00	0.00	0.00	0.00	0.00	0.00	0.00	0.00	0.00	0.00
adrenoceptor alpha 1D	Adra1d	100772819	0.01	0.02	0.00	0.00	0.01	0.00	3.04	4.58	0.00	0.00	1.03	2.80
adrenoceptor alpha 2A	Adra2a	100768369	0.00	0.00	0.00	0.00	0.00	0.09	0.00	0.00	0.00	0.00	0.00	0.93
adrenoceptor alpha 2B	Adra2b	100757384	0.00	0.00	0.00	0.00	0.00	0.00	0.00	0.00	0.00	0.00	0.00	0.00
adrenoceptor alpha 2C	Adra2c	103161652	0.00	0.00	0.00	0.00	0.00	0.00	0.00	0.00	0.00	0.00	0.00	0.00
adrenoceptor beta 2, surface	Adrb2	100758902	0.00	0.00	0.00	0.00	0.00	0.00	0.00	0.00	0.00	0.00	0.00	0.00
adrenoceptor beta 3	Adrb3	100775030	0.00	0.00	0.00	0.00	0.00	0.00	0.00	0.00	0.00	0.00	0.00	0.00
adrenergic, beta, receptor kinase 1	Adrbk1	100762552	47.40	45.03	53.46	47.94	38.11	43.97	4728.13	4619.09	5562.06	4974.08	3992.98	4535.02
adhesion regulating molecule 1	Adrm1	100754801	152.16	151.58	128.45	98.93	85.23	88.75	5931.24	5857.13	4923.63	3706.23	3233.30	3416.22
adenylosuccinate lyase	Adsl	100689060	59.63	60.63	68.00	58.97	48.95	54.34	3185.51	3232.81	3577.21	3064.18	2583.33	2880.65
adenylosuccinate synthase	Adss	100767083	47.21	51.82	51.86	56.09	54.20	52.38	3225.76	3521.05	3531.25	3751.42	3686.62	3482.58
adenylosuccinate synthase like 1	Adssl1	100751825	0.76	0.45	0.89	0.81	0.54	0.36	34.94	21.96	40.99	34.77	22.77	16.82

androgen-dependent TFPI-regulating protein	Adtrp	100763412	0.00	0.00	0.08	0.00	0.00	0.00	0.76	0.92	1.24	0.00	0.00	0.00
AE binding protein 1	Aebp1	100762528	0.03	0.02	0.00	0.01	0.01	0.01	3.80	4.58	0.00	1.16	1.03	0.93
AE binding protein 2	Aebp2	100765454	50.92	51.59	42.69	45.14	46.38	44.64	3706.55	3718.70	3097.76	3335.37	3347.15	3336.77
apoptosis enhancing nuclease	Aen	100765585	48.13	49.88	29.76	26.68	20.80	24.10	3079.17	3164.19	1915.30	1709.41	1299.94	1530.99
amino-terminal enhancer of split	Aes	100769877	86.97	85.66	88.78	73.65	50.53	55.58	4194.18	4178.04	4351.03	3585.70	2537.79	2702.13
actin filament associated protein 1	Afap1	100767632	23.61	23.50	26.15	26.20	23.90	25.19	4521.54	4523.01	5060.26	5040.14	4570.50	4841.59
actin filament associated protein 1-like 1	Afap1l1	100759163	0.03	0.02	0.02	0.00	0.02	0.00	1.52	1.83	1.24	0.00	1.03	0.00
actin filament associated protein 1-like 2	Afap1l2	100771462	0.00	0.00	0.00	0.00	0.00	0.00	0.00	0.00	0.00	0.00	0.00	0.00
AF4/FMR2 family, member 1	Aff1	100751914	8.20	8.08	7.62	7.80	7.62	7.17	1014.74	1014.77	966.34	983.92	998.76	928.13
AF4/FMR2 family, member 2	Aff2	100756662	1.94	2.11	3.72	4.24	4.05	3.96	242.29	255.29	469.51	523.83	490.58	475.75
AF4/FMR2 family, member 3	Aff3	100758079	0.00	0.02	0.00	0.00	0.00	0.00	0.00	0.92	0.00	0.00	0.00	0.00
AF4/FMR2 family, member 4	Aff4	100756909	16.57	16.75	17.16	19.79	21.10	20.88	4605.09	4662.10	4799.42	5494.44	5822.83	5762.24
AFG3-like AAA ATPase 2	Afg3l2	100763687	19.89	20.26	20.28	20.39	19.75	19.57	1576.04	1602.22	1613.47	1599.31	1542.13	1531.92
afamin	Afm	100757632	0.00	0.00	0.00	0.00	0.00	0.00	0.00	0.00	0.00	0.00	0.00	0.00
arylformamidase	Afmid	100773211	3.69	3.89	3.56	4.11	3.26	3.37	243.81	242.48	238.48	264.23	205.96	200.95
alpha-fetoprotein	Afp	100759078	0.00	0.00	0.00	0.00	0.00	0.00	1.52	0.00	1.24	2.32	0.00	0.00
aftiphilin	Aftph	100754955	42.36	42.05	42.16	47.20	45.55	43.79	3758.96	3795.56	3824.39	4213.83	4056.11	3929.35
aspartylglucosaminidase	Aga	100754017	219.21	213.29	216.99	220.98	239.83	235.98	7780.72	7678.05	7799.06	7793.74	8570.72	8245.66
ArfGAP with GTPase domain, ankyrin repeat and PH domain 1	Agap1	100770318	87.53	87.90	111.78	96.08	75.77	81.33	4614.20	4635.56	5902.40	5028.55	4072.67	4346.21
ArfGAP with GTPase domain, ankyrin repeat and PH domain 2	Agap2	100764978	0.04	0.02	0.02	0.00	0.01	0.00	6.08	7.32	2.48	2.32	3.10	0.93
ArfGAP with GTPase domain, ankyrin repeat and PH domain 3	Agap3	100769383	21.34	20.17	21.82	15.47	10.10	10.99	1655.80	1589.41	1747.62	1240.04	852.83	898.22
ATP/GTP binding protein-like 1	Agbl1	100750752	0.00	0.00	0.00	0.00	0.00	0.00	0.00	0.00	0.00	0.00	0.00	0.00
ATP/GTP binding protein-like 2	Agbl2	100761623	0.01	0.00	0.00	0.00	0.00	0.00	1.52	0.00	0.00	0.00	0.00	0.00
ATP/GTP binding protein-like 3	Agbl3	100764584	1.65	1.79	1.63	1.55	2.31	1.94	268.12	281.83	269.53	279.30	377.77	332.74
ATP/GTP binding protein-like 4	Agbl4	100759283	0.00	0.00	0.00	0.00	0.00	0.00	0.00	0.00	0.00	0.00	0.00	0.00
ATP/GTP binding protein-like 5	Agbl5	100762486	4.28	4.64	4.52	4.75	4.61	4.62	528.64	582.88	583.78	601.48	565.10	555.19
advanced glycosylation end product-specific receptor	Ager	100757624	0.28	0.14	0.32	0.10	0.18	0.14	22.03	13.73	17.39	13.91	9.31	15.89
ArfGAP with FG repeats 1	Agfg1	100757749	49.48	47.89	48.53	51.96	50.10	49.46	4268.61	4220.13	4287.68	4560.35	4376.96	4317.24
ArfGAP with FG repeats 2	Agfg2	100755409	10.21	10.06	14.12	12.69	12.84	12.93	816.50	825.36	1137.75	1007.10	969.78	987.01
angiogenic factor with G patch and FHA domains 1	Aggf1	100757002	21.44	21.22	23.40	27.08	29.45	27.21	1830.49	1830.98	2048.20	2322.48	2507.77	2318.92
acylglycerol kinase	Agk	100771779	24.62	24.56	24.95	26.80	27.51	28.18	1528.19	1540.00	1566.27	1644.51	1732.57	1745.03
amylo-alpha-1, 6-glucosidase, 4-alpha-glucanotransferase	Agl	100772306	6.73	7.17	6.00	6.73	7.94	7.72	985.12	1071.50	870.70	982.76	1162.29	1113.19
agmatine ureohydrolase (agmatinase)	Agmat	100752066	0.00	0.00	0.00	0.00	0.00	0.00	0.76	0.00	0.00	0.00	0.00	0.00
alkylglycerol monooxygenase	Agmo	100752746	0.00	0.05	0.00	0.03	0.00	0.05	0.00	1.83	0.00	1.16	0.00	1.87
argonaute RISC catalytic component 1	Ago1	100768807	0.09	0.05	0.02	0.04	0.04	0.02	14.43	10.07	8.69	9.27	8.28	2.80
argonaute RISC catalytic component 2	Ago2	100760040	14.12	13.78	12.77	13.10	10.95	11.45	1477.30	1429.28	1321.58	1384.91	1152.97	1211.33
argonaute RISC catalytic component 3	Ago3	100769090	2.30	2.18	1.97	2.13	2.41	2.28	303.82	290.07	262.08	301.32	304.29	294.42

argonaute RISC catalytic component 4	Ago4	100762217	5.47	5.45	6.60	6.48	7.83	7.45	629.66	633.20	770.09	759.09	909.75	893.54
1-acylglycerol-3-phosphate O-acyltransferase 1	Agpat1	100758206	42.12	40.04	38.46	33.81	28.46	29.48	1753.02	1646.15	1526.52	1266.70	974.96	1058.05
	Agpat2	100754963	31.67	34.13	31.42	27.64	21.82	23.85	555.22	560.92	496.83	455.46	410.89	430.88
1-acylglycerol-3-phosphate O-acyltransferase 3	Agpat3	100763383	27.69	27.88	29.93	25.33	21.01	21.28	2691.81	2747.85	2925.11	2453.43	2029.61	2051.60
1-acylglycerol-3-phosphate O-acyltransferase 4	Agpat4	100761743	26.05	27.47	22.83	23.28	23.95	21.96	1331.47	1446.67	1206.07	1179.78	1249.23	1116.93
1-acylglycerol-3-phosphate O-acyltransferase 5	Agpat5	100757875	20.26	20.22	22.14	24.61	28.75	29.44	1677.82	1681.83	1842.01	2040.86	2371.15	2407.71
1-acylglycerol-3-phosphate O-acyltransferase 6	Agpat6	100751939	42.36	41.38	51.83	54.49	53.25	52.99	3952.64	3913.60	4913.70	5045.94	4893.42	4870.56
1-acylglycerol-3-phosphate O-acyltransferase 9	Agpat9	100758424	15.75	16.75	21.31	22.28	24.44	23.77	1325.40	1410.98	1798.54	1833.41	2010.98	1940.37
alkylglycerone phosphate synthase	Agps	100756809	12.43	12.62	14.80	16.57	18.19	17.70	1588.96	1665.36	1960.01	2172.97	2356.66	2272.18
anterior gradient 2	Agr2	100769015	0.00	0.00	0.00	0.00	0.00	0.00	0.00	0.00	0.00	0.00	0.00	0.00
anterior gradient 3	Agr3	100768729	0.08	0.00	0.00	0.00	0.11	0.00	1.52	1.83	0.00	0.00	1.03	0.00
agrin	Agrn	100765949	8.41	8.36	11.27	10.42	8.95	9.84	1862.39	1850.20	2460.57	2312.05	2108.27	2277.79
agouti related protein homolog (mouse)	Agrp	100762653	0.11	0.04	0.00	0.00	0.11	0.10	7.60	5.49	3.73	1.16	6.21	2.80
angiotensinogen (serpin peptidase inhibitor, clade A, member 8)	Agt	100773858	0.02	0.04	0.14	0.13	0.10	0.11	3.04	2.75	9.94	9.27	8.28	7.48
ATP/GTP binding protein 1	Agtpbp1	100772683	6.13	6.08	6.14	6.86	6.94	6.55	776.25	786.93	763.88	877.30	860.07	823.44
angiotensin II receptor, type 1	Agtr1	100750798	0.00	0.00	0.00	0.00	0.00	0.00	0.00	0.00	0.00	0.00	0.00	0.00
angiotensin II receptor, type 2	Agtr2	100750901	0.26	0.27	0.20	0.18	0.23	0.20	63.80	68.63	60.86	49.83	74.52	61.69
angiotensin II receptor-associated protein	Agtrap	100774061	7.60	7.30	6.50	5.30	3.90	4.03	221.79	210.46	192.52	152.98	115.92	119.64
alanine-glyoxylate aminotransferase	Agxt	100771278	0.00	0.00	0.00	0.00	0.00	0.00	0.00	0.00	0.00	0.00	0.00	0.00
alanine--glyoxylate aminotransferase 2	Agxt2	100759906	0.00	0.00	0.00	0.00	0.00	0.00	0.00	0.00	1.24	0.00	0.00	0.00
AT hook containing transcription factor 1	Ahctf1	100768805	64.63	63.15	59.44	72.86	68.61	63.89	7499.69	7325.76	7158.15	8396.37	7857.62	7379.22
adenosylhomocysteinase	Ahcy	100750969	103.55	106.89	112.35	95.79	74.95	81.35	6131.00	6343.01	6684.91	5674.07	4424.57	4798.59
adenosylhomocysteinase-like 1	Ahcyl1	100767631	77.94	77.21	60.10	64.40	69.14	71.67	3875.17	3970.33	3023.24	3220.64	3439.26	3505.95
adenosylhomocysteinase-like 2	Ahcyl2	100754476	7.80	7.63	10.32	10.86	11.29	12.85	1038.29	1031.24	1404.80	1435.90	1531.78	1731.01
AT hook, DNA binding motif, containing 1	Ahdc1	100758977	2.77	2.34	2.19	1.34	0.86	1.05	431.42	366.93	341.57	216.72	137.65	175.72
Abelson helper integration site 1	Ahi1	100773665	9.02	8.61	12.85	13.87	17.02	15.67	1222.86	1179.48	1740.16	1871.66	2292.49	2118.90
AHNAK nucleoprotein	Ahnak	100753847	68.14	74.19	57.40	70.68	99.38	94.12	24885.54	27381.46	21183.79	26452.35	37183.95	35483.80
AHNAK nucleoprotein 2	Ahnak2	100752400	0.94	1.03	0.31	0.43	0.52	0.57	233.94	257.12	68.31	115.89	138.69	156.09
aryl hydrocarbon receptor	Ahr	100682538	19.40	18.78	19.07	21.83	20.37	20.26	1699.85	1655.30	1720.29	1962.05	1839.17	1786.15
AHA1, activator of heat shock 90kDa protein ATPase homolog 1 (yeast)	Ahsa1	100760684	198.79	197.74	213.38	206.70	188.77	197.44	7674.38	7788.77	8376.63	8009.30	7370.14	7567.09
AHA1, activator of heat shock 90kDa protein ATPase homolog 2 (yeast)	Ahsa2	100756374	36.99	38.23	39.20	37.75	40.13	37.83	775.49	771.37	799.90	740.55	777.27	776.71
alpha-2-HS-glycoprotein	Ahsg	100765165	0.00	0.00	0.00	0.00	0.00	0.00	0.00	0.00	0.00	0.00	0.00	0.00
alpha hemoglobin stabilizing protein	Ahsp	100770599	2.38	2.34	2.27	2.96	3.92	2.92	44.81	54.90	65.83	57.95	69.34	61.69
activation-induced cytidine deaminase	Aicda	100689250	0.00	0.00	0.00	0.00	0.00	0.00	0.00	0.00	0.00	0.00	0.00	0.00

axin interactor, dorsalization associated	Aida	100755322	27.21	27.52	29.05	32.06	40.84	37.90	3200.70	3248.37	3423.19	3797.78	4794.06	4446.22
allograft inflammatory factor 1	Aif1	100764184	0.00	0.00	0.00	0.00	0.00	0.00	0.00	0.92	0.00	1.16	1.03	1.87
allograft inflammatory factor 1-like	Aif1l	100752261	0.00	0.00	0.00	0.00	0.00	0.00	0.00	0.00	0.00	0.00	0.00	0.00
apoptosis-inducing factor, mitochondrion-associated, 1	Aifm1	100771367	52.48	50.94	51.69	55.05	55.31	53.85	3338.17	3274.91	3257.99	3428.09	3446.50	3390.98
apoptosis-inducing factor, mitochondrion-associated, 2	Aifm2	100763473	10.50	9.63	9.68	9.15	8.04	8.59	925.88	854.64	857.04	806.61	693.44	766.43
apoptosis-inducing factor, mitochondrion-associated, 3	Aifm3	100752310	0.07	0.05	0.10	0.21	0.09	0.15	7.60	3.66	8.69	17.38	10.35	14.95
androgen-induced 1	Aig1	100757869	13.53	13.56	12.14	13.81	15.33	14.42	405.59	421.83	371.38	403.30	463.67	428.08
absent in melanoma 1	Aim1	100774444	0.70	0.62	0.40	0.58	0.33	0.33	163.30	138.17	90.67	125.16	80.73	81.32
absent in melanoma 1-like	Aim1l	100767991	0.00	0.01	0.03	0.00	0.00	0.01	0.00	0.92	3.73	1.16	1.03	0.93
aminoacyl tRNA synthetase complex-interacting multifunctional protein 1	Aimp1	100689238	188.26	188.75	193.21	203.23	206.10	201.34	2653.07	2734.12	2835.68	3041.01	2989.04	2915.23
aminoacyl tRNA synthetase complex-interacting multifunctional protein 2	Aimp2	100682533	50.60	48.13	48.21	42.01	36.73	36.91	1623.89	1577.52	1561.30	1325.80	1147.80	1161.79
aryl hydrocarbon receptor interacting protein	Aip	100759077	41.62	41.53	41.82	39.15	35.56	34.66	1456.80	1486.93	1504.17	1383.75	1281.31	1225.35
aryl hydrocarbon receptor interacting protein-like 1	Aipl1	100760409	0.00	0.00	0.00	0.00	0.00	0.00	0.00	0.00	0.00	0.00	0.00	0.00
autoimmune regulator	Aire	100768045	0.00	0.00	0.00	0.00	0.00	0.00	0.76	1.83	0.00	0.00	0.00	0.00
adherens junctions associated protein 1	Ajap1	100762681	0.00	0.00	0.00	0.00	0.00	0.00	0.00	0.00	0.00	0.00	0.00	0.00
ajuba LIM protein	Ajuba	103158564	101.31	100.07	77.00	76.80	71.27	74.70	4140.25	4091.11	3159.87	3075.77	2848.28	3004.03
adenylate kinase 1	Ak1	100759346	0.13	0.17	0.23	0.24	0.16	0.18	8.35	11.90	14.91	15.07	11.38	12.15
adenylate kinase 2	Ak2	100756723	237.42	228.89	234.49	192.41	162.87	175.48	3341.97	3228.24	3236.88	2738.53	2393.92	2535.76
adenylate kinase 3	Ak3	100760312	77.46	83.14	79.94	81.36	82.64	83.21	5743.64	6216.74	5979.41	6051.88	6129.19	6186.58
adenylate kinase 4	Ak4	100755987	7.18	7.73	6.52	6.11	6.88	7.61	268.12	284.58	258.35	234.10	268.06	293.49
adenylate kinase 5	Ak5	100761703	0.05	0.07	0.02	0.04	0.02	0.00	3.80	3.66	1.24	1.16	1.03	1.87
adenylate kinase 6	Ak6	100768838	59.11	57.89	49.67	54.84	58.42	59.24	799.04	733.86	613.59	711.58	783.48	789.80
adenylate kinase 7	Ak7	100760489	0.01	0.00	0.04	0.04	0.05	0.02	0.76	1.83	2.48	2.32	3.10	0.93
adenylate kinase 8	Ak8	100767050	0.05	0.30	0.04	0.21	0.08	0.14	3.04	8.24	2.48	5.79	2.07	4.67
adenylate kinase 9	Ak9	100752880	0.02	0.01	0.01	0.00	0.01	0.00	3.80	0.92	2.48	1.16	3.10	0.93
A kinase (PRKA) anchor protein 1	Akap1	100760847	25.66	25.31	13.72	13.70	12.26	12.60	2735.10	2677.39	1465.66	1439.38	1296.84	1344.99
A kinase (PRKA) anchor protein 10	Akap10	100759380	6.43	6.17	7.24	7.94	8.37	7.88	586.36	583.79	675.70	738.23	765.89	721.57
A kinase (PRKA) anchor protein 11	Akap11	100769001	15.54	15.14	14.38	18.28	19.24	18.24	3168.04	3095.56	2987.22	3745.63	3918.46	3734.01
A kinase (PRKA) anchor protein 12	Akap12	100772605	67.95	70.68	140.59	171.84	209.32	199.21	11927.80	12439.88	24910.05	30293.01	36627.13	35215.55
A kinase (PRKA) anchor protein 13	Akap13	100768171	8.12	8.58	14.38	15.45	14.50	14.33	2813.33	2920.79	4592.00	5001.90	4734.03	4722.89
A kinase (PRKA) anchor protein 14	Akap14	100763759	0.03	0.00	0.00	0.00	0.00	0.00	0.76	0.00	0.00	0.00	1.03	0.00
A kinase (PRKA) anchor protein 17A	Akap17a	100769666	0.95	0.99	0.62	1.16	1.03	1.06	158.74	170.20	106.82	199.33	173.88	183.20
A kinase (PRKA) anchor protein 2	Akap2	100767422	23.07	22.45	19.59	20.65	21.30	19.53	3819.72	3826.67	3430.64	3737.52	4020.92	3617.17
A kinase (PRKA) anchor protein 3	Akap3	100773279	1.89	2.25	1.00	1.27	1.68	1.38	183.05	228.76	104.34	114.73	153.18	129.92
A kinase (PRKA) anchor protein 4	Akap4	100754812	0.00	0.00	0.00	0.00	0.00	0.00	0.00	0.00	0.00	0.00	0.00	0.00
A kinase (PRKA) anchor protein 5	Akap5	100768039	0.05	0.03	0.04	0.07	0.03	0.00	2.28	1.83	1.24	4.64	1.03	0.00

A kinase (PRKA) anchor protein 6	Akap6	100756287	0.00	0.00	0.00	0.01	0.00	0.00	0.00	0.00	0.00	1.16	0.00	0.00
A kinase (PRKA) anchor protein 7	Akap7	100757731	18.20	17.99	9.90	11.02	13.40	14.34	1039.81	1034.90	577.57	632.77	764.85	820.64
A kinase (PRKA) anchor protein 8	Akap8	100771696	24.76	25.12	25.09	25.97	23.25	24.34	2678.89	2745.10	2741.29	2819.65	2499.49	2624.55
A kinase (PRKA) anchor protein 8-like	Akap8l	100754234	15.49	14.92	15.58	15.91	14.61	14.75	966.89	980.92	1023.48	1032.60	935.63	936.54
A kinase (PRKA) anchor protein 9	Akap9	100767118	8.66	8.17	7.43	10.18	11.25	10.46	2864.22	2692.03	2458.09	3348.12	3675.24	3435.85
A kinase (PRKA) interacting protein 1	Akip1	100751192	29.02	30.23	26.06	26.51	32.25	29.73	785.36	806.14	701.78	672.17	829.02	736.52
akirin 1	Akirin1	100751284	43.39	41.98	37.64	40.67	41.73	41.46	2306.72	2237.26	1997.28	2144.00	2193.14	2205.82
akirin 2	Akirin2	100769005	48.70	49.90	48.27	49.37	53.31	51.63	1402.87	1445.75	1393.62	1413.88	1521.43	1457.15
AT-hook transcription factor	Akna	100774585	1.22	1.26	0.97	0.91	0.72	0.67	257.48	272.68	212.40	200.49	162.49	157.02
aldo-keto reductase family 1, member A1 (aldehyde reductase)	Akr1a1	100689106	422.36	386.11	393.29	381.33	345.33	394.16	16740.24	15636.09	15827.91	15159.83	13855.36	15491.22
aldo-keto reductase family 1, member B1 (aldose reductase)	Akr1b1	100751272	138.32	137.81	123.03	125.63	119.73	126.76	5456.53	5511.25	4947.23	4937.00	4719.54	4933.19
aldo-keto reductase family 1, member B8	Akr1b8	100689318	37.26	39.31	45.36	50.44	52.78	52.51	1323.12	1400.00	1568.76	1723.31	1779.14	1802.98
aldo-keto reductase family 1, member D1	Akr1d1	100770348	0.00	0.00	0.00	0.00	0.04	0.00	0.00	0.92	0.00	0.00	1.03	0.00
aldo-keto reductase family 1, member E2	Akr1e2	100750439	32.11	31.32	21.83	25.83	35.61	33.96	1343.62	1326.80	934.05	1036.07	1525.57	1465.56
v-akt murine thymoma viral oncogene homolog 1	Akt1	100769460	132.76	137.18	144.11	138.62	114.83	122.95	8865.34	9266.54	9788.89	9253.98	7706.51	8269.96
AKT1 substrate 1 (proline-rich)	Akt1s1	100768286	27.76	25.69	29.88	18.41	14.32	15.72	1816.82	1688.24	1986.10	1263.22	968.75	1090.76
v-akt murine thymoma viral oncogene homolog 2	Akt2	100768351	28.21	28.35	27.79	23.96	21.95	23.06	2428.25	2487.98	2446.91	2093.01	1892.99	1988.98
v-akt murine thymoma viral oncogene homolog 3	Akt3	100752454	20.42	20.98	25.44	24.18	26.41	27.33	1107.41	1160.26	1371.26	1310.74	1381.71	1437.52
AKT interacting protein	Aktip	100770507	52.18	55.59	49.65	55.67	57.75	56.54	2511.80	2681.05	2419.59	2680.58	2786.18	2717.08
aminolevulinate dehydratase	Alad	100752314	23.02	21.85	24.45	19.56	17.53	19.54	791.44	786.01	849.59	680.29	624.10	678.57
aminolevulinate, delta-, synthase 1	Alas1	100766370	10.34	11.25	14.13	12.72	10.93	11.80	637.25	700.00	862.01	774.16	670.67	733.72
aminolevulinate, delta-, synthase 2	Alas2	100753315	0.00	0.00	0.00	0.00	0.00	0.00	2.28	0.92	0.00	3.48	0.00	1.87
albumin	Alb	100768954	0.48	0.11	1.14	1.46	1.10	1.53	13.67	5.49	23.60	37.09	33.12	41.13
activated leukocyte cell adhesion molecule	Alcam	100774312	84.52	86.27	95.62	114.76	133.82	123.95	7575.64	7994.65	9237.40	11044.51	12869.02	11717.95
aldehyde dehydrogenase 16 family, member A1	Aldh16a 1	100772664	35.48	33.35	37.75	24.01	14.65	16.36	2542.94	2396.47	2745.01	1736.06	1039.13	1158.06
aldehyde dehydrogenase 18 family, member A1	Aldh18a 1	100755485	0.20	0.16	0.25	0.25	0.39	0.31	22.03	17.39	23.60	28.97	43.47	32.71
aldehyde dehydrogenase 1 family, member A2	Aldh1a2	100772304	0.00	0.00	0.00	0.00	0.00	0.00	0.00	0.00	0.00	0.00	0.00	0.00
aldehyde dehydrogenase 1 family, member A3	Aldh1a3	100768227	0.04	0.05	0.06	0.00	0.03	0.00	2.28	2.75	2.48	0.00	4.14	1.87
aldehyde dehydrogenase 1 family, member B1	Aldh1b1	100765516	0.00	0.00	0.00	0.00	0.00	0.00	0.00	0.00	0.00	0.00	0.00	0.00
aldehyde dehydrogenase 1 family, member L1	Aldh1l1	100762089	0.00	0.00	0.00	0.00	0.00	0.00	0.00	0.00	0.00	0.00	0.00	0.00
aldehyde dehydrogenase 1 family, member L2	Aldh1l2	100772371	0.06	0.03	0.05	0.01	0.00	0.00	5.32	4.58	7.45	1.16	0.00	0.00
aldehyde dehydrogenase 2 family (mitochondrial)	Aldh2	100772225	43.53	41.28	53.50	47.27	38.26	46.01	2235.32	2164.05	2818.30	2436.05	1997.52	2343.22
aldehyde dehydrogenase 3 family, member A1	Aldh3a1	100757371	0.00	0.03	0.00	0.00	0.00	0.00	0.00	0.92	0.00	0.00	0.00	0.00

aldehyde dehydrogenase 3 family, member A2	Aldh3a2	100757658	6.20	6.21	6.93	7.34	8.47	7.92	543.83	552.68	623.53	643.20	734.84	689.79
aldehyde dehydrogenase 3 family, member B2	Aldh3b2	100763332	0.01	0.00	0.00	0.00	0.00	0.00	0.00	0.00	1.24	0.00	0.00	0.00
aldehyde dehydrogenase 4 family, member A1	Aldh4a1	100764994	3.32	2.87	3.77	3.18	2.64	3.26	349.39	312.03	409.89	341.88	283.59	340.22
aldehyde dehydrogenase 5 family, member A1	Aldh5a1	100764462	4.91	4.64	6.09	6.81	6.14	6.15	268.88	270.85	345.30	375.49	363.28	341.15
aldehyde dehydrogenase 6 family, member A1	Aldh6a1	100754996	14.56	13.73	13.81	15.30	19.14	18.67	1023.10	993.73	999.88	1074.32	1353.76	1304.80
aldehyde dehydrogenase 7 family, member A1	Aldh7a1	100756509	23.13	22.93	22.75	24.83	21.76	24.55	1121.08	1152.03	1163.84	1224.98	1092.95	1151.51
aldehyde dehydrogenase 8 family, member A1	Aldh8a1	100751303	0.02	0.04	0.04	0.07	0.02	0.08	1.52	2.75	2.48	4.64	1.03	4.67
aldehyde dehydrogenase 9 family, member A1	Aldh9a1	100758951	15.64	15.22	16.60	16.28	18.00	18.70	952.46	936.08	1017.27	985.08	1091.91	1133.75
aldolase A, fructose-bisphosphate	Aldoa	100771717	396.34	383.35	414.28	405.99	371.26	405.71	16295.91	16087.20	17140.80	15732.34	14102.72	15277.18
aldolase B, fructose-bisphosphate	Aldob	100755179	0.00	0.00	0.07	0.07	0.06	0.00	0.76	0.00	2.48	2.32	2.07	0.00
aldolase C, fructose-bisphosphate	Aldoc	100760749	0.16	0.12	0.21	0.07	0.11	0.15	18.99	15.56	19.87	18.54	15.52	19.63
ALG1, chitobiosyldiphosphodolichol beta-mannosyltransferase	Alg1	100773731	5.84	6.33	5.30	5.37	4.35	4.85	518.01	551.77	449.64	457.77	360.18	380.41
ALG11, alpha-1,2-mannosyltransferase	Alg11	100771009	21.59	22.16	22.70	24.90	26.67	26.53	1085.38	1109.02	1163.84	1263.22	1334.10	1330.97
ALG12, alpha-1,6-mannosyltransferase	Alg12	100770096	20.92	21.58	26.72	27.13	25.03	26.20	1432.49	1486.01	1854.44	1838.05	1729.46	1798.30
ALG13, UDP-N-acetylglucosaminyltransferase subunit	Alg13	100754023	7.63	8.00	7.15	7.44	8.68	8.28	1103.61	1166.67	1060.74	1143.85	1300.98	1226.29
ALG14, UDP-N-acetylglucosaminyltransferase subunit	Alg14	100773644	21.70	21.40	18.04	18.74	15.22	17.58	335.72	323.92	276.99	288.57	240.12	283.20
ALG2, alpha-1,3/1,6-mannosyltransferase	Alg2	100768412	12.85	13.30	12.17	12.84	14.65	15.48	1102.09	1144.71	1053.29	1107.93	1250.26	1334.71
ALG3, alpha-1,3- mannosyltransferase	Alg3	100772003	19.08	18.63	20.94	18.64	14.18	16.56	780.05	785.10	868.22	788.07	606.50	678.57
ALG5, dolichyl-phosphate beta-glucosyltransferase	Alg5	100769679	23.52	23.64	21.31	27.78	28.66	24.48	833.97	858.30	770.09	997.83	1026.71	875.79
ALG6, alpha-1,3-glucosyltransferase	Alg6	100753783	6.43	6.93	6.08	6.64	9.19	7.97	518.01	561.83	496.83	535.42	729.67	641.18
ALG8, alpha-1,3-glucosyltransferase	Alg8	100766150	11.94	12.48	12.07	13.42	14.79	12.93	767.89	815.29	766.37	840.22	958.40	842.14
ALG9, alpha-1,2-mannosyltransferase	Alg9	100755062	20.36	19.98	20.17	22.74	23.98	22.68	2362.93	2397.39	2350.03	2606.41	2778.94	2710.54
anaplastic lymphoma receptor tyrosine kinase	Alk	100760366	0.04	0.04	0.01	0.01	0.01	0.02	9.11	11.90	3.73	2.32	2.07	2.80
alkB, alkylation repair homolog 1 (E. coli)	Alkbh1	100760177	16.39	16.27	16.56	17.94	18.54	18.44	1830.49	1820.00	1861.89	1999.14	2038.92	2047.86
alkB, alkylation repair homolog 2 (E. coli)	Alkbh2	100753724	19.58	20.87	16.79	14.83	10.29	11.97	483.07	512.42	421.07	345.36	247.36	278.53
alkB, alkylation repair homolog 3 (E. coli)	Alkbh3	100774403	37.50	38.73	39.66	39.62	36.97	38.19	1589.72	1658.96	1678.06	1636.39	1556.62	1601.09
alkB, alkylation repair homolog 4 (E. coli)	Alkbh4	100766685	7.63	8.04	10.77	9.96	9.13	11.00	415.47	425.49	576.33	538.90	512.32	594.45
alkB, alkylation repair homolog 5 (E. coli)	Alkbh5	100755624	4.38	4.53	5.22	5.80	5.96	5.11	143.55	149.15	182.59	194.70	193.54	162.63

alkB, alkylation repair homolog 6 (E. coli)	Alkbh6	100771885	28.35	23.91	30.84	27.15	21.52	19.93	354.70	309.28	387.53	327.97	255.64	260.77
alkB, alkylation repair homolog 7 (E. coli)	Alkbh7	100774290	16.53	15.50	15.19	12.06	10.90	12.92	270.40	248.89	258.35	190.06	160.42	191.61
alkB, alkylation repair homolog 8 (E. coli)	Alkbh8	100760738	17.13	16.87	15.53	18.03	20.53	19.16	1412.74	1422.88	1340.21	1532.09	1768.79	1654.37
allantoicase	Allc	100766149	0.00	0.00	0.00	0.00	0.00	0.00	0.00	0.00	0.00	0.00	0.00	0.00
Alstrom syndrome 1	Alms1	103162359	4.40	4.03	4.36	5.64	5.75	5.12	1430.97	1293.86	1458.21	1834.57	1841.24	1693.62
arachidonate 12-lipoxygenase	Alox12	100764620	0.01	0.01	0.00	0.02	0.00	0.01	3.80	2.75	4.97	4.64	13.45	1.87
arachidonate 12-lipoxygenase, 12R type	Alox12b	100761707	0.05	0.02	0.00	0.00	0.00	0.02	3.04	0.92	0.00	0.00	0.00	0.93
arachidonate 15-lipoxygenase	Alox15	100764916	0.00	0.00	0.00	0.00	0.00	0.00	0.00	0.00	0.00	0.00	0.00	0.00
arachidonate 15-lipoxygenase, type B	Alox15b	100754473	0.00	0.00	0.00	0.00	0.00	0.00	0.00	0.00	0.00	0.00	0.00	0.00
arachidonate 5-lipoxygenase	Alox5	100772492	0.00	0.00	0.00	0.00	0.00	0.00	0.76	0.00	0.00	0.00	0.00	0.00
arachidonate 5-lipoxygenase-activating protein	Alox5ap	100760744	0.00	0.00	0.10	0.00	0.00	0.15	0.00	0.00	1.24	1.16	0.00	1.87
arachidonate lipoxygenase 3	Aloxe3	100754187	1.45	1.23	1.38	1.52	1.90	2.13	170.14	154.64	177.62	197.02	251.50	275.73
alpha-kinase 1	Alpk1	100755133	1.62	1.35	1.41	1.47	1.65	1.30	213.43	191.24	191.28	206.29	212.17	172.91
alpha-kinase 3	Alpk3	100751103	0.00	0.01	0.00	0.00	0.00	0.00	2.28	0.92	0.00	0.00	0.00	0.00
alkaline phosphatase, liver/bone/kidney	Alpl	100771587	0.04	0.02	0.00	0.00	0.00	0.00	1.52	0.92	0.00	0.00	0.00	0.00
amyotrophic lateral sclerosis 2 (juvenile)	Als2	100763641	10.66	10.58	11.05	11.36	11.63	11.87	2085.69	2112.81	2189.80	2265.69	2284.21	2292.74
ALS2 C-terminal like	Als2cl	100767195	5.99	6.02	5.75	5.36	7.00	6.74	922.84	953.46	880.64	817.04	1031.88	1036.55
amyotrophic lateral sclerosis 2 (juvenile) chromosome region, candidate 11	Als2cr11	100759628	0.46	0.48	0.52	0.58	0.74	0.69	124.56	123.53	147.81	161.09	189.40	174.78
amyotrophic lateral sclerosis 2 (juvenile) chromosome region, candidate 12	Als2cr12	100759917	0.07	0.06	0.04	0.00	0.03	0.03	6.08	3.66	1.24	2.32	3.10	1.87
ALX homeobox 1	Alx1	100759154	0.00	0.00	0.00	0.00	0.00	0.00	0.00	0.00	0.00	0.00	0.00	0.00
ALX homeobox 3	Alx3	100770020	0.00	0.00	0.00	0.00	0.00	0.00	0.00	0.00	0.00	1.16	0.00	0.00
ALX homeobox 4	Alx4	100750505	0.00	0.00	0.00	0.00	0.00	0.00	0.00	0.00	0.00	0.00	0.00	0.00
Aly/REF export factor	Alyref	100756310	270.98	286.72	111.40	104.22	77.32	83.07	6439.37	6871.90	2660.55	2436.05	1853.66	1907.66
ameloblastin (enamel matrix protein)	Ambn	100773253	0.00	0.03	0.00	0.00	0.00	0.00	0.00	0.92	0.00	0.00	0.00	0.00
alpha-1-microglobulin/bikunin precursor	Ambp	100750686	0.00	0.00	0.00	0.00	0.00	0.00	0.00	0.00	0.00	0.00	0.00	0.00
autophagy/beclin-1 regulator 1	Ambra1	100753101	9.45	9.40	9.82	9.29	8.09	8.29	1775.04	1766.93	1855.68	1737.22	1505.91	1524.45
adenosylmethionine decarboxylase 1	Amd1	100774596	65.00	65.37	77.97	80.30	91.64	86.02	5303.86	5448.11	6506.05	6712.46	7590.59	7274.53
amidohydrolase domain containing 1	Amdhd1	100754101	0.00	0.00	0.00	0.00	0.00	0.00	0.00	0.00	0.00	0.00	0.00	0.00
amidohydrolase domain containing 2	Amdhd2	100771907	10.54	9.58	11.72	9.98	9.30	9.25	476.23	433.73	522.92	445.03	410.89	421.54
APC membrane recruitment protein 1	Amer1	100751609	6.10	6.67	7.01	6.76	7.04	7.59	912.97	1003.79	1059.50	1021.01	1052.58	1139.36
APC membrane recruitment protein 2	Amer2	100766392	0.00	0.00	0.00	0.00	0.00	0.00	0.00	0.00	0.00	0.00	0.00	0.00
APC membrane recruitment protein 3	Amer3	100750361	0.00	0.00	0.00	0.00	0.00	0.00	0.00	0.00	0.00	0.00	0.00	0.00
autocrine motility factor receptor, E3 ubiquitin protein ligase	Amfr	100762626	43.50	41.68	49.66	50.04	46.06	49.30	3812.89	3711.38	4469.03	4474.59	4080.95	4364.91
anti-Mullerian hormone	Amh	100759997	0.54	0.35	0.13	0.22	0.11	0.14	29.62	21.96	7.45	10.43	6.21	7.48

anti-Mullerian hormone receptor, type II	Amhr2	100772657	0.06	0.17	0.10	0.17	0.12	0.11	28.10	34.77	40.99	53.31	27.94	37.39
adhesion molecule, interacts with CXADR antigen 1	Amica1	100767539	0.00	0.00	0.00	0.00	0.00	0.00	0.00	0.00	0.00	0.00	0.00	0.00
adhesion molecule with Ig-like domain 1	Amigo1	100772963	0.00	0.00	0.00	0.03	0.00	0.00	0.00	0.00	0.00	1.16	0.00	0.00
adhesion molecule with Ig-like domain 2	Amigo2	100761994	0.00	0.00	0.00	0.00	0.00	0.00	0.00	0.00	0.00	0.00	0.00	0.00
adhesion molecule with Ig-like domain 3	Amigo3	100761154	0.80	0.75	0.53	0.76	0.58	0.54	81.27	75.95	53.41	77.65	58.99	53.28
Alport syndrome, mental retardation, midface hypoplasia and elliptocytosis chromosomal region gene 1	Ammecr1	100759636	3.75	4.15	4.30	5.71	5.19	5.03	218.75	225.10	245.93	310.59	294.97	284.14
AMMECR1-like	Ammecr1l	100754731	9.11	8.37	8.36	8.60	9.33	8.94	981.32	917.78	906.72	948.00	1042.23	1002.90
antagonist of mitotic exit network 1 homolog (S. cerevisiae)	Amn1	100761574	8.27	8.59	9.61	8.41	12.89	11.60	259.00	270.85	296.86	260.76	410.89	351.44
angiomotin	Amot	100769669	4.04	4.33	3.43	3.95	3.47	3.57	676.75	745.75	582.54	671.01	583.73	607.54
angiomotin like 1	Amotl1	100754241	70.84	71.14	78.60	78.67	73.34	76.94	17189.89	17422.24	19395.19	19249.66	17643.41	18672.83
angiomotin like 2	Amotl2	100771000	56.80	59.28	48.32	43.09	44.10	46.56	6762.18	7069.55	5791.85	5122.43	5200.81	5523.90
adenosine monophosphate deaminase 1	Ampd1	100757901	0.00	0.00	0.00	0.00	0.00	0.00	0.00	0.00	0.00	0.00	0.00	0.00
adenosine monophosphate deaminase 2	Ampd2	100774910	28.40	26.88	31.90	29.38	24.89	25.41	3041.95	2890.59	3480.33	3064.18	2511.91	2636.70
adenosine monophosphate deaminase 3	Ampd3	100758386	0.10	0.16	0.15	0.11	0.17	0.10	10.63	15.56	18.63	18.54	18.63	11.22
amphiphysin	Amph	100767913	0.00	0.00	0.00	0.00	0.00	0.00	0.00	0.00	0.00	0.00	0.00	0.00
aminomethyltransferase	Amt	100763091	3.61	3.55	3.27	4.14	5.53	4.90	254.45	257.12	240.96	296.68	381.91	339.29
amelotin	Amtn	100768466	0.00	0.00	0.00	0.00	0.00	0.00	0.00	0.00	0.00	0.00	0.00	0.00
archaelysin family metallopeptidase 1	Amz1	100761231	0.05	0.04	0.02	0.03	0.01	0.04	3.80	2.75	2.48	5.79	1.03	4.67
archaelysin family metallopeptidase 2	Amz2	100774378	21.08	21.96	21.23	21.54	25.11	25.03	835.49	897.65	825.99	798.50	960.47	956.17
anaphase promoting complex subunit 1	Anapc1	100770891	55.22	55.33	59.11	64.96	69.51	68.70	14131.99	14209.56	15244.13	16547.06	17619.61	17548.42
anaphase promoting complex subunit 10	Anapc10	100772439	27.38	27.75	26.37	30.27	34.80	33.71	537.75	574.64	522.92	594.53	716.21	668.29
anaphase promoting complex subunit 11	Anapc11	100767345	122.74	110.56	43.70	26.74	12.47	16.40	1506.17	1370.72	558.94	340.72	190.44	255.16
anaphase promoting complex subunit 13	Anapc13	100770713	65.14	73.56	34.15	35.54	29.99	33.82	652.44	737.52	335.36	336.09	290.83	339.29
anaphase promoting complex subunit 15	Anapc15	100774442	23.20	25.67	25.27	25.25	26.69	27.45	597.00	620.39	623.53	650.15	647.90	687.92
anaphase promoting complex subunit 16	Anapc16	100759455	18.46	17.27	14.60	14.55	12.94	15.51	903.09	850.07	726.62	711.58	627.20	758.95
anaphase promoting complex subunit 2	Anapc2	100774636	29.68	28.75	33.73	32.59	28.63	32.99	2378.88	2311.37	2718.93	2670.15	2363.91	2640.44
anaphase promoting complex subunit 4	Anapc4	100767504	29.08	30.15	30.91	34.03	37.65	32.78	2406.98	2470.59	2525.16	2765.18	3068.73	2692.78
anaphase promoting complex subunit 5	Anapc5	100766376	138.75	137.54	142.11	138.25	124.48	128.22	10183.14	10303.28	10741.57	10315.55	9340.75	9508.39
anaphase promoting complex subunit 7	Anapc7	100765036	27.35	27.21	28.37	29.51	26.48	28.39	1791.75	1798.04	1885.49	1926.12	1735.67	1860.93
angiogenin, ribonuclease, RNase A family, 5	Ang	100755402	6.03	7.36	6.49	8.67	10.47	10.00	56.97	65.88	57.14	57.95	84.87	73.84

angel homolog 1 (Drosophila)	Angel1	100761072	7.96	7.97	8.07	7.89	6.80	7.59	923.60	934.25	963.86	936.41	793.83	896.35
angel homolog 2 (Drosophila)	Angel2	100767019	30.73	30.82	30.37	34.06	35.17	32.32	1661.11	1670.85	1615.95	1809.07	1819.51	1738.49
angiopoietin 1	Angpt1	100774625	0.00	0.00	0.00	0.00	0.00	0.00	0.00	0.00	0.00	0.00	0.00	0.00
angiopoietin 2	Angpt2	100756240	0.01	0.00	0.00	0.02	0.01	0.01	0.76	0.00	1.24	1.16	1.03	0.93
angiopoietin 4	Angpt4	100761260	0.11	0.01	0.04	0.00	0.02	0.00	8.35	0.92	2.48	0.00	1.03	0.00
angiopoietin-like 1	Angptl1	100773728	0.00	0.02	0.03	0.00	0.00	0.00	0.00	0.92	1.24	0.00	0.00	0.00
angiopoietin-like 2	Angptl2	100758488	0.00	0.00	0.00	0.00	0.00	0.00	0.00	0.00	0.00	0.00	0.00	0.00
angiopoietin-like 3	Angptl3	100754950	0.00	0.00	0.00	0.00	0.03	0.00	1.52	0.00	1.24	2.32	6.21	0.00
angiopoietin-like 4	Angptl4	100754832	0.04	0.05	0.00	0.00	0.00	0.00	3.80	3.66	0.00	0.00	1.03	0.00
angiopoietin-like 5	Angptl5	100762578	0.02	0.06	0.00	0.03	0.05	0.04	1.52	2.75	0.00	1.16	3.10	1.87
angiopoietin-like 6	Angptl6	100772824	0.86	0.84	0.74	0.54	0.41	0.40	28.86	31.11	23.60	16.22	14.49	11.22
angiopoietin-like 7	Angptl7	100755713	0.00	0.00	0.00	0.00	0.00	0.04	0.00	0.00	1.24	0.00	0.00	1.87
anomalous homeobox	Anhx	100751696	0.00	0.00	0.00	0.00	0.00	0.00	0.00	0.00	0.00	0.00	0.00	0.00
ankyrin 2, neuronal	Ank2	100757830	5.55	5.26	5.74	6.66	5.99	5.67	2560.41	2413.86	2670.49	3052.59	2746.85	2649.79
ankyrin 3, node of Ranvier (ankyrin G)	Ank3	100767295	1.38	1.29	1.25	1.36	1.40	1.30	741.31	719.22	674.45	757.93	742.09	714.09
ankyrin and armadillo repeat containing	Ankar	100767421	0.31	0.31	0.52	0.43	0.62	0.62	30.38	30.20	45.96	37.09	52.78	53.28
ankyrin repeat and death domain containing 1A	Ankdd1a	100754818	0.07	0.10	0.04	0.04	0.02	0.00	6.84	10.98	6.21	5.79	4.14	1.87
ankyrin repeat and death domain containing 1B	Ankdd1b	100773274	0.87	1.00	1.12	1.12	1.26	1.18	105.58	118.95	126.69	141.39	157.32	137.40
ankyrin repeat and EF-hand domain containing 1	Ankef1	100750817	0.00	0.00	0.00	0.00	0.00	0.00	0.00	0.00	0.00	0.00	0.00	0.00
ankyrin-repeat and fibronectin type III domain containing 1	Ankfn1	100764232	0.00	0.00	0.00	0.00	0.00	0.00	0.00	0.00	0.00	0.00	0.00	0.00
ankyrin repeat and FYVE domain containing 1	Ankfy1	100757230	10.39	10.00	9.81	10.83	11.75	12.04	1548.70	1496.08	1471.87	1586.56	1709.80	1816.06
ANKH inorganic pyrophosphate transport regulator	Ankh	100771094	13.97	14.02	11.84	12.27	11.73	11.59	1323.12	1322.22	1148.93	1179.78	1124.00	1109.45
ankyrin repeat and KH domain containing 1	Ankhd1	100756474	4.60	4.43	4.64	5.29	5.23	4.99	1222.86	1196.86	1260.72	1412.72	1375.50	1335.64
ankyrin repeat and IBR domain containing 1	Ankib1	100759287	18.62	18.10	20.43	21.99	21.99	22.57	2930.30	2881.44	3255.51	3462.85	3476.52	3580.72
ankyrin repeat and kinase domain containing 1	Ankk1	100751636	0.00	0.00	0.00	0.00	0.00	0.00	0.00	0.00	0.00	0.00	0.00	0.00
ankyrin repeat and LEM domain containing 1	Ankle1	100753605	9.82	9.20	7.65	7.31	6.06	6.23	968.41	904.97	767.61	712.74	589.94	608.47
ankyrin repeat and LEM domain containing 2	Ankle2	100750769	13.37	13.97	16.07	17.33	18.47	19.64	1631.49	1750.46	1984.85	2117.35	2260.41	2384.34
ankyrin repeat and MYND domain containing 1	Ankmy1	100751434	0.55	0.52	0.52	0.59	0.53	0.53	59.24	55.82	62.10	61.42	53.82	50.47
ankyrin repeat and MYND domain containing 2	Ankmy2	100758778	9.72	9.97	11.48	12.10	12.90	12.71	663.84	698.17	808.60	836.74	900.44	883.26
ankyrin repeat, family A (RFXANK-like), 2	Ankra2	100755782	6.09	7.18	6.83	7.74	8.21	8.01	422.30	485.88	480.69	529.63	607.54	600.99

ankyrin repeat domain 1 (cardiac muscle)	Ankrd1	100689447	29.89	32.90	5.25	6.07	10.66	9.50	1465.15	1596.73	286.92	330.29	594.08	528.09
ankyrin repeat domain 10	Ankrd10	100766146	13.70	12.54	11.36	12.37	11.50	10.83	1231.97	1154.77	1165.08	1249.32	1310.29	1257.13
ankyrin repeat domain 11	Ankrd11	100764009	44.01	41.83	41.78	45.83	40.75	41.00	11453.85	10898.05	10963.90	11914.86	10537.20	10706.64
ankyrin repeat domain 12	Ankrd12	100755296	11.13	10.79	10.83	12.64	13.74	12.90	2619.65	2563.92	2578.57	3007.40	3248.82	3057.30
ankyrin repeat domain 13A	Ankrd13a	100756344	19.42	20.51	22.98	24.03	24.73	24.44	2025.69	2127.45	2456.85	2476.61	2555.38	2534.82
ankyrin repeat domain 13B	Ankrd13b	100757903	0.60	0.62	0.28	0.28	0.20	0.26	45.57	45.75	23.60	22.02	14.49	19.63
ankyrin repeat domain 13C	Ankrd13c	100751847	32.58	32.20	34.83	36.48	40.24	40.73	1665.67	1667.19	1809.72	1869.34	2081.36	2065.62
ankyrin repeat domain 13 family, member D	Ankrd13d	100762256	0.17	0.12	0.14	0.04	0.06	0.12	16.71	15.56	19.87	12.75	6.21	20.56
ankyrin repeat domain 16	Ankrd16	100765375	2.65	2.69	2.50	2.17	2.30	2.24	183.05	198.56	187.56	154.14	171.81	181.33
ankyrin repeat domain 17	Ankrd17	100768373	24.81	24.38	23.95	26.97	25.54	23.96	6080.11	5983.40	5888.73	6530.51	6137.47	5874.40
ankyrin repeat domain 2 (stretch responsive muscle)	Ankrd2	100758530	0.03	0.03	0.00	0.04	0.00	0.00	3.04	1.83	0.00	1.16	1.03	2.80
ankyrin repeat domain 22	Ankrd22	100762241	0.03	0.03	0.00	0.00	0.00	0.00	1.52	1.83	0.00	0.00	1.03	0.00
ankyrin repeat domain 23	Ankrd23	100769457	1.78	1.72	1.84	2.16	1.98	1.81	133.68	123.53	131.66	154.14	141.79	127.12
ankyrin repeat domain 24	Ankrd24	100769301	0.02	0.04	0.08	0.01	0.01	0.07	4.56	6.41	7.45	2.32	5.17	6.54
ankyrin repeat domain 27 (VPS9 domain)	Ankrd27	100758465	6.25	5.65	7.32	7.47	7.02	7.84	786.12	743.01	952.68	959.59	889.05	978.60
ankyrin repeat domain 28	Ankrd28	100767224	13.56	13.18	15.47	19.95	21.66	20.69	1596.55	1602.22	1830.84	2374.63	2641.29	2522.67
ankyrin repeat domain 29	Ankrd29	100755582	0.40	0.21	0.24	0.14	0.10	0.16	39.50	31.11	22.36	19.70	10.35	14.95
ankyrin repeat domain 30A	Ankrd30a	100769687	4.27	4.31	4.94	6.01	7.09	6.20	738.27	726.54	839.65	1022.17	1200.58	1046.83
ankyrin repeat domain 31	Ankrd31	100757808	0.00	0.00	0.00	0.00	0.00	0.00	0.00	0.00	0.00	0.00	0.00	0.00
ankyrin repeat domain 32	Ankrd32	100753941	6.98	6.84	7.02	9.00	10.30	9.09	819.54	813.46	835.92	1032.60	1190.23	1063.65
ankyrin repeat domain 33	Ankrd33	100765182	0.00	0.00	0.00	0.00	0.00	0.00	0.00	0.00	0.00	0.00	0.00	0.00
ankyrin repeat domain 33B	Ankrd33b	100760181	0.00	0.00	0.00	0.00	0.00	0.00	0.00	0.00	0.00	0.00	0.00	0.00
ankyrin repeat domain 34A	Ankrd34a	100755248	1.04	1.20	2.77	1.73	1.40	1.54	69.12	76.86	161.47	88.08	62.10	79.45
ankyrin repeat domain 34B	Ankrd34b	100761769	0.00	0.00	0.00	0.00	0.00	0.00	0.00	0.00	0.00	0.00	0.00	0.00
ankyrin repeat domain 34C	Ankrd34c	100757468	0.00	0.00	0.00	0.00	0.00	0.00	0.00	0.00	0.00	0.00	0.00	0.00
ankyrin repeat domain 35	Ankrd35	100753898	0.12	0.12	0.04	0.06	0.05	0.02	16.71	11.90	7.45	5.79	6.21	1.87
ankyrin repeat domain 37	Ankrd37	100771039	3.33	3.61	4.34	5.05	4.49	4.04	60.00	64.05	79.49	86.92	81.76	68.23

ankyrin repeat domain 39	Ankrd39	100769174	8.09	8.50	6.29	5.54	3.84	5.46	134.44	133.59	100.61	84.60	73.48	87.86
ankyrin repeat domain 40	Ankrd40	100761647	47.98	49.62	29.33	28.65	27.91	26.48	2038.60	2111.90	1232.15	1211.07	1170.57	1150.58
ankyrin repeat domain 42	Ankrd42	100753052	1.62	1.35	1.78	1.70	2.28	2.24	129.88	111.63	144.08	135.59	193.54	186.00
ankyrin repeat domain 45	Ankrd45	100755441	0.00	0.00	0.00	0.00	0.06	0.00	0.76	0.92	2.48	2.32	3.10	0.00
ankyrin repeat domain 46	Ankrd46	100756375	13.09	13.06	16.98	15.46	14.62	14.81	796.00	786.93	1025.96	923.66	865.25	888.87
ankyrin repeat domain 49	Ankrd49	100750582	13.84	14.75	13.61	14.81	15.77	15.01	843.09	905.88	848.35	874.98	953.22	913.17
ankyrin repeat domain 50	Ankrd50	100756565	6.27	6.02	5.69	5.17	4.72	5.60	995.00	960.79	912.93	820.52	747.26	900.09
ankyrin repeat domain 52	Ankrd52	100751169	18.84	18.48	15.00	13.37	9.10	10.39	2505.72	2461.44	2066.83	1892.52	1347.55	1488.93
ankyrin repeat domain 53	Ankrd53	100757823	0.02	0.00	0.00	0.00	0.00	0.03	2.28	0.00	1.24	2.32	0.00	1.87
ankyrin repeat domain 54	Ankrd54	100756181	11.63	10.50	13.67	11.96	11.95	12.32	634.97	570.07	755.19	654.79	641.69	674.83
ankyrin repeat domain 55	Ankrd55	100766888	0.00	0.00	0.00	0.00	0.00	0.00	0.00	0.92	0.00	0.00	1.03	0.00
ankyrin repeat domain 6	Ankrd6	100772602	0.03	0.00	0.00	0.00	0.00	0.00	3.04	0.92	0.00	0.00	0.00	0.00
ankyrin repeat domain 60	Ankrd60	100773959	0.00	0.00	0.00	0.00	0.00	0.00	0.00	0.00	0.00	0.00	0.00	0.00
ankyrin repeat domain 61	Ankrd61	100756204	0.97	0.84	0.87	1.02	0.96	1.09	64.56	51.24	58.38	64.90	71.41	71.03
ankyrin repeat domain 63	Ankrd63	100760434	0.00	0.00	0.00	0.00	0.00	0.00	0.00	0.00	0.00	0.00	0.00	0.00
ankyrin repeat domain 65	Ankrd65	100773738	0.29	0.49	0.24	0.11	0.00	0.06	13.67	21.05	13.66	11.59	7.24	5.61
ankyrin repeat domain 66	Ankrd66	100771794	0.00	0.00	0.00	0.00	0.00	0.00	0.00	0.00	0.00	0.00	0.00	0.00
ankyrin repeat domain 7	Ankrd7	100755680	0.00	0.00	0.00	0.06	0.00	0.05	0.00	0.00	0.00	1.16	0.00	0.93
ankyrin repeat domain 9	Ankrd9	100773105	1.95	1.67	1.86	0.81	0.45	0.60	79.75	65.88	74.53	34.77	21.73	30.84
ankyrin repeat and sterile alpha motif domain containing 1A	Anks1a	100755580	4.51	4.40	4.52	3.59	3.03	3.17	577.25	595.69	591.23	472.84	402.61	409.39
ankyrin repeat and sterile alpha motif domain containing 1B	Anks1b	100766915	0.11	0.03	0.00	0.00	0.00	0.00	3.80	0.92	0.00	0.00	0.00	0.00
ankyrin repeat and sterile alpha motif domain containing 3	Anks3	100772275	22.14	20.90	19.99	15.09	11.57	11.54	1775.80	1676.34	1618.44	1252.79	995.66	1009.44
ankyrin repeat and sterile alpha motif domain containing 4B	Anks4b	100770300	0.02	0.03	0.00	0.00	0.00	0.00	0.76	0.92	0.00	1.16	0.00	0.93
ankyrin repeat and sterile alpha motif domain containing 6	Anks6	100771869	3.05	3.05	3.03	2.45	1.48	1.94	310.65	317.52	332.88	270.03	198.72	237.41
ankyrin repeat and ubiquitin domain containing 1	Ankub1	100769801	0.08	0.06	0.05	0.12	0.04	0.02	10.63	3.66	2.48	10.43	5.17	4.67
ankyrin repeat and zinc finger domain containing 1	Ankzf1	100773729	10.09	10.45	11.77	12.57	10.11	9.98	881.07	928.76	1017.27	1106.77	910.79	872.98
anillin, actin binding protein	Anln	100768074	50.45	50.60	52.95	58.50	70.78	66.74	7418.42	7473.08	7828.87	8571.37	10314.67	9801.88
anoctamin 1, calcium activated chloride channel	Ano1	100774893	0.00	0.00	0.03	0.00	0.02	0.00	0.00	0.92	1.24	0.00	1.03	0.00
anoctamin 10	Ano10	100770142	19.38	21.31	37.39	39.74	36.64	36.95	1465.15	1581.18	2759.92	2908.89	2695.10	2732.04

anoctamin 2, calcium activated chloride channel	Ano2	100775014	0.00	0.00	0.00	0.00	0.00	0.00	0.00	0.00	0.00	0.00	0.00	0.00
anoctamin 3	Ano3	100760607	0.01	0.02	0.02	0.02	0.06	0.05	1.52	2.75	2.48	2.32	7.24	6.54
anoctamin 4	Ano4	100773499	0.01	0.01	0.00	0.00	0.00	0.00	0.76	1.83	0.00	0.00	0.00	0.00
anoctamin 5	Ano5	100774784	0.00	0.00	0.00	0.00	0.00	0.00	0.00	0.00	0.00	0.00	0.00	0.00
anoctamin 7	Ano7	100753794	0.02	0.03	0.00	0.00	0.00	0.00	2.28	2.75	0.00	2.32	0.00	0.00
anoctamin 8	Ano8	100751640	5.02	4.71	4.93	3.47	2.94	2.96	601.56	562.75	596.20	421.85	342.58	357.98
anoctamin 9	Ano9	100758985	0.00	0.01	0.00	0.02	0.00	0.00	0.00	0.92	2.48	1.16	1.03	0.00
acidic (leucine-rich) nuclear phosphoprotein 32 family, member A	Anp32a	100758072	43.58	43.97	53.08	55.14	53.19	54.69	2681.93	2753.34	3315.13	3418.81	3245.72	3261.06
acidic (leucine-rich) nuclear phosphoprotein 32 family, member B	Anp32b	100767552	495.07	537.16	591.93	576.74	550.27	581.12	15660.18	17081.85	18638.76	17783.63	17013.10	17859.67
acidic (leucine-rich) nuclear phosphoprotein 32 family, member E	Anp32e	100751239	96.78	96.74	93.09	94.41	98.97	101.23	3106.52	3250.20	3108.94	3126.77	3362.67	3399.39
alanyl (membrane) aminopeptidase	Anpep	100751632	0.00	0.00	0.00	0.00	0.00	0.00	0.00	0.00	0.00	0.00	0.00	0.00
anthrax toxin receptor 1	Antxr1	100769395	0.43	0.24	0.00	0.00	0.00	0.00	29.62	18.30	0.00	0.00	0.00	0.00
anthrax toxin receptor 2	Antxr2	100689461	21.96	23.06	25.08	28.80	30.41	29.55	3718.70	3882.49	4269.05	4868.62	5086.96	4960.29
anthrax toxin receptor-like	Antxrl	100759372	0.00	0.00	0.00	0.00	0.00	0.00	0.00	0.00	0.00	0.00	0.00	0.00
annexin A1	Anxa1	100768079	384.40	376.49	386.80	451.25	541.01	541.30	16982.54	17209.04	17549.44	20037.72	24064.47	23298.51
annexin A10	Anxa10	100761636	0.00	0.00	0.00	0.00	0.00	0.00	0.00	0.00	0.00	0.00	0.00	0.00
annexin A11	Anxa11	100752736	32.32	31.80	33.16	32.07	28.76	29.17	2198.11	2172.29	2269.29	2186.88	1948.88	2006.74
annexin A2	Anxa2	100760539	892.56	839.44	834.47	891.22	1008.07	1071.97	18603.39	18025.25	17427.72	18037.43	20887.06	21839.49
annexin A3	Anxa3	100762572	3.69	4.23	5.05	5.23	5.09	4.15	189.13	215.95	242.21	246.85	248.40	206.56
annexin A4	Anxa4	100771507	49.80	47.53	46.77	50.70	56.66	61.17	2902.96	2848.50	2787.24	2954.09	3275.73	3535.86
annexin A5	Anxa5	100759361	101.59	105.26	103.49	105.02	108.82	111.33	4690.91	4914.65	4785.76	4713.33	4976.21	5018.24
annexin A6	Anxa6	100759027	37.20	36.85	34.11	37.48	41.62	42.15	3150.57	3245.62	3030.69	3265.84	3613.14	3584.46
annexin A7	Anxa7	100763423	56.60	59.92	59.58	63.87	72.24	71.18	2914.35	3095.56	3056.78	3279.74	3699.04	3605.02
annexin A9	Anxa9	100766282	0.40	0.39	0.37	0.40	0.19	0.21	31.14	34.77	29.81	31.29	15.52	14.95
acyloxyacyl hydrolase (neutrophil)	Aoah	100768833	0.00	0.00	0.00	0.00	0.00	0.00	0.00	0.00	0.00	0.00	0.00	0.00
amine oxidase, copper containing 2 (retina-specific)	Aoc2	100762635	0.28	0.28	0.27	0.35	0.35	0.34	33.42	32.94	28.57	44.04	45.54	44.86
adaptor-related protein complex 1 associated regulatory protein	Ap1ar	100755236	10.63	12.84	11.06	11.55	13.63	10.95	110.89	127.19	129.18	120.53	144.90	103.75
adaptor-related protein complex 1, beta 1 subunit	Ap1b1	100752826	126.68	132.31	151.17	147.08	171.19	186.15	7933.39	7892.16	8765.41	8770.71	8858.45	10058.92
adaptor-related protein complex 1, gamma 1 subunit	Ap1g1	100755260	14.12	14.20	13.54	15.30	16.05	15.08	2836.12	2867.72	2715.20	3010.87	3153.60	2957.29
adaptor-related protein complex 1, gamma 2 subunit	Ap1g2	100764053	5.93	6.16	7.91	7.74	7.18	7.91	532.44	563.66	674.45	665.22	646.87	709.41
adaptor-related protein complex 1, mu 1 subunit	Ap1m1	100759505	29.10	28.38	28.32	23.59	16.66	19.11	1656.56	1638.83	1645.77	1352.46	963.57	1086.09
adaptor-related protein complex 1, mu 2 subunit	Ap1m2	100758260	0.00	0.00	0.04	0.04	0.00	0.00	0.00	0.00	1.24	1.16	0.00	0.00
adaptor-related protein complex 1, sigma 1 subunit	Ap1s1	100759417	45.38	46.02	47.95	46.08	41.81	48.01	1670.99	1724.84	1792.33	1630.60	1484.17	1684.27
adaptor-related protein complex 1, sigma 2 subunit	Ap1s2	100768456	18.37	21.09	15.40	16.69	21.15	22.08	639.53	660.65	583.78	607.27	772.10	746.80
adaptor-related protein complex 1, sigma 3 subunit	Ap1s3	100764202	0.00	0.00	0.00	0.00	0.00	0.00	0.00	0.00	0.00	0.00	0.00	0.00

adaptor-related protein complex 2, alpha 1 subunit	Ap2a1	100772481	54.47	54.58	58.18	46.70	33.54	37.72	5356.27	5349.29	5744.65	4561.51	3326.45	3681.66
adaptor-related protein complex 2, alpha 2 subunit	Ap2a2	100755406	53.26	52.06	53.66	51.23	43.39	45.96	5202.84	5100.40	5271.42	4965.97	4209.29	4477.07
adaptor-related protein complex 2, beta 1 subunit	Ap2b1	100750609	40.07	39.04	21.47	22.66	23.40	25.22	6192.52	6105.10	3398.35	3600.76	3641.08	3890.10
adaptor-related protein complex 2, mu 1 subunit	Ap2m1	100773058	162.43	155.39	167.78	168.83	174.78	190.49	8967.88	8761.45	9442.34	9433.61	9788.90	10432.78
adaptor-related protein complex 2, sigma 1 subunit	Ap2s1	100764194	35.18	32.94	39.04	34.00	29.01	31.68	724.60	706.41	807.36	660.58	537.16	578.56
adaptor-related protein complex 3, beta 1 subunit	Ap3b1	100755352	13.48	14.10	14.35	14.56	17.81	17.41	2161.65	2286.67	2292.89	2294.66	2806.88	2756.34
adaptor-related protein complex 3, beta 2 subunit	Ap3b2	100759367	0.00	0.01	0.00	0.01	0.00	0.01	0.00	0.92	0.00	1.16	0.00	0.93
adaptor-related protein complex 3, delta 1 subunit	Ap3d1	100770344	36.10	34.16	39.61	39.32	34.54	37.59	5177.78	4953.08	5791.85	5643.94	4917.22	5332.29
adaptor-related protein complex 3, mu 1 subunit	Ap3m1	100689277	39.04	41.08	39.79	44.79	46.56	44.10	3663.26	3723.27	3589.63	4050.42	4419.39	4220.97
adaptor-related protein complex 3, mu 2 subunit	Ap3m2	100751069	0.02	0.03	0.01	0.00	0.01	0.02	1.52	0.92	1.24	1.16	1.03	0.93
adaptor-related protein complex 3, sigma 1 subunit	Ap3s1	100770551	24.78	24.52	25.52	26.27	31.58	29.50	858.28	865.62	905.48	910.91	1083.63	1022.53
adaptor-related protein complex 4, beta 1 subunit	Ap4b1	100759820	7.65	7.21	6.08	5.90	7.36	7.40	580.29	549.02	473.24	465.89	541.30	565.48
adaptor-related protein complex 4, epsilon 1 subunit	Ap4e1	100751812	2.64	2.41	2.40	3.00	3.30	3.08	365.34	345.88	320.46	429.96	462.64	416.86
adaptor-related protein complex 4, mu 1 subunit	Ap4m1	100774803	4.06	4.23	5.62	5.10	4.92	5.04	268.12	250.72	342.82	310.59	293.94	300.96
adaptor-related protein complex 4, sigma 1 subunit	Ap4s1	100763289	7.14	6.95	8.65	6.56	10.70	9.32	144.31	137.26	192.52	141.39	215.28	196.28
adaptor-related protein complex 5, beta 1 subunit	Ap5b1	100761023	14.12	13.18	13.12	9.30	6.15	6.80	1149.18	1082.48	1070.68	764.89	519.56	569.21
adaptor-related protein complex 5, sigma 1 subunit	Ap5s1	100765217	11.17	9.29	13.53	13.38	10.06	11.68	442.05	405.36	556.45	536.58	446.08	502.85
adaptor-related protein complex 5, zeta 1 subunit	Ap5z1	100760656	4.50	5.01	4.98	3.03	2.24	2.58	404.07	446.54	449.64	293.21	203.89	242.08
apoptotic peptidase activating factor 1	Apaf1	100758500	5.39	5.51	5.36	6.18	7.38	6.84	511.93	520.65	500.56	594.53	696.55	634.64
amyloid beta (A4) precursor protein-binding, family A, member 1	Apba1	100774931	2.16	2.49	2.69	3.00	3.02	3.12	359.26	419.09	437.21	505.29	500.93	530.89
amyloid beta (A4) precursor protein-binding, family A, member 2	Apba2	100763312	0.11	0.10	0.04	0.05	0.03	0.04	8.35	10.07	3.73	3.48	2.07	2.80
amyloid beta (A4) precursor protein-binding, family A, member 3	Apba3	100763507	4.39	4.23	4.59	1.72	1.01	1.26	325.84	329.41	319.22	129.80	75.55	94.40
amyloid beta (A4) precursor protein-binding, family B, member 1 (Fe65)	Apbb1	100760848	21.80	22.71	24.92	21.24	15.48	17.52	1673.26	1767.84	1968.71	1661.89	1228.53	1344.06
amyloid beta (A4) precursor protein-binding, family B, member 1 interacting protein	Apbb1ip	100773337	34.86	35.39	39.72	36.75	31.50	32.52	2240.64	2259.22	2576.09	2337.54	2024.43	2082.44
amyloid beta (A4) precursor protein-binding, family B, member 2	Apbb2	100755787	8.92	9.57	8.01	7.90	8.81	8.48	720.80	717.39	635.95	646.68	736.91	750.54
amyloid beta (A4) precursor protein-binding, family B, member 3	Apbb3	100756770	2.15	2.59	3.43	3.18	3.11	2.98	161.78	188.50	237.24	251.49	253.57	232.73
adenomatous polyposis coli	Apc	100750563	6.29	5.64	4.50	5.96	6.17	5.65	1690.73	1537.26	1224.70	1612.06	1659.08	1535.66
adenomatosis polyposis coli 2	Apc2	100767273	0.11	0.16	0.30	0.28	0.20	0.17	28.86	28.37	75.77	54.47	45.54	43.93

adenomatosis polyposis coli down-regulated 1	Apcdd1	100766679	0.00	0.00	0.00	0.00	0.00	0.00	0.00	0.00	0.00	0.00	0.00	0.00
amyloid P component, serum	Apcs	100772827	0.00	0.00	0.00	0.00	0.00	0.00	0.00	0.00	0.00	0.00	0.00	0.00
acylaminoacyl-peptide hydrolase	Apeh	100761732	29.50	26.49	32.52	33.05	29.10	33.08	2214.06	2037.78	2532.62	2476.61	2189.00	2425.47
APEX nuclease (multifunctional DNA repair enzyme) 1	Apex1	100689281	186.07	188.85	198.23	188.51	178.20	190.17	6736.35	6895.69	7232.67	6572.23	6038.11	6493.15
APEX nuclease (apurinic/apyrimidinic endonuclease) 2	Apex2	100753797	11.62	11.21	12.70	13.39	12.10	12.48	625.10	613.99	695.57	718.53	661.36	678.57
APH1A gamma secretase subunit	Aph1a	100760965	46.12	44.71	48.84	48.60	47.70	50.31	1993.79	1920.66	1994.79	1749.97	1530.74	1552.49
APH1B gamma secretase subunit	Aph1b	100772143	1.97	2.14	2.06	2.16	2.56	2.34	146.59	161.96	154.02	162.25	196.65	177.59
apoptosis inhibitor 5	Api5	100758910	62.62	67.17	63.68	67.21	74.01	73.95	5884.91	6409.81	6070.08	6375.22	6966.49	6959.55
APAF1 interacting protein	Apip	100774834	32.55	32.46	24.91	25.38	28.89	27.42	821.82	822.62	635.95	647.84	731.74	682.31
aprataxin and PNKP like factor	Aplf	100765532	6.93	7.77	10.09	10.89	12.21	11.31	382.05	420.00	547.76	625.82	687.23	644.92
apelin	Apln	103161730	0.99	0.86	0.77	0.85	0.88	0.90	82.03	72.29	67.07	71.85	72.45	76.64
apelin receptor	Aplnr	100759527	0.00	0.00	0.00	0.00	0.00	0.00	0.00	0.00	0.00	0.00	0.00	0.00
amyloid beta (A4) precursor-like protein 1	Aplp1	100773034	0.00	0.00	0.00	0.00	0.00	0.00	0.00	0.92	1.24	0.00	0.00	0.00
amyloid beta (A4) precursor-like protein 2	Aplp2	100758329	121.84	119.79	116.49	113.88	117.52	121.36	12448.09	12374.91	12075.57	11598.47	11969.62	12233.89
adipocyte plasma membrane associated protein	Apmap	100765704	46.83	47.72	45.05	47.77	50.36	49.42	2994.10	3016.87	2895.30	3001.60	3156.71	3132.08
apolipoprotein A-I	Apoa1	100772590	0.04	0.00	0.00	0.13	0.00	0.10	0.76	0.92	0.00	4.64	2.07	3.74
apolipoprotein A-I binding protein	Apoa1bp	100762326	48.05	46.12	58.21	50.34	45.69	50.03	1591.23	1565.62	1946.35	1680.43	1496.59	1630.06
apolipoprotein A-II	Apoa2	100768641	1.63	1.88	0.86	1.01	1.03	1.09	47.85	52.16	33.54	41.72	36.22	42.06
apolipoprotein A-IV	Apoa4	100774995	0.04	0.02	0.02	0.02	0.00	0.02	3.80	0.92	6.21	1.16	0.00	0.93
apolipoprotein A-V	Apoa5	100772884	0.00	0.00	0.00	0.00	0.00	0.00	0.00	0.00	0.00	0.00	0.00	0.00
apolipoprotein B	Apob	100762698	0.00	0.01	0.00	0.00	0.00	0.00	1.52	4.58	1.24	0.00	0.00	0.93
apolipoprotein B mRNA editing enzyme, catalytic polypeptide 1	Apobec1	100760326	0.00	0.00	0.00	0.00	0.00	0.00	0.00	0.00	0.00	0.00	0.00	0.00
apolipoprotein B mRNA editing enzyme, catalytic polypeptide-like 2	Apobec2	100756679	0.00	0.00	0.00	0.00	0.00	0.00	0.00	0.00	0.00	0.00	0.00	0.00
apolipoprotein B mRNA editing enzyme, catalytic polypeptide-like 4 (putative)	Apobec4	100764761	0.00	0.00	0.00	0.00	0.00	0.00	0.00	0.00	0.00	0.00	0.00	0.00
apolipoprotein B receptor	Apobr	100753181	0.01	0.04	0.00	0.00	0.02	0.02	0.76	4.58	0.00	0.00	2.07	1.87
apolipoprotein C-I	Apoc1	103159291	0.00	0.00	0.00	0.00	0.00	0.00	0.00	0.00	0.00	0.00	0.00	0.00
apolipoprotein C-II	Apoc2	100774395	0.00	0.00	0.00	0.00	0.00	0.00	0.00	0.92	0.00	0.00	0.00	0.00
apolipoprotein C-III	Apoc3	100774705	0.00	0.00	0.00	0.00	0.00	0.00	0.00	0.00	0.00	0.00	0.00	0.00
apolipoprotein C-IV	Apoc4	100774105	0.00	0.00	0.00	0.00	0.00	0.00	0.00	0.00	0.00	0.00	0.00	0.00
apolipoprotein D	Apod	100774049	0.05	0.00	0.00	0.00	0.00	0.00	0.76	0.00	0.00	0.00	0.00	0.00
apolipoprotein E	Apoe	100773810	0.00	0.08	0.00	0.00	0.00	0.00	0.00	1.83	1.24	0.00	1.03	0.00
apolipoprotein F	Apof	100775063	0.00	0.00	0.00	0.00	0.00	0.00	0.76	0.00	0.00	0.00	0.00	0.00
apolipoprotein H (beta-2-glycoprotein I)	Apoh	100762364	0.08	0.05	0.00	0.03	0.03	0.03	4.56	1.83	1.24	3.48	4.14	6.54
apolipoprotein L domain containing 1	Apold1	100773016	0.00	0.00	0.00	0.06	0.05	0.00	0.00	0.00	1.24	1.16	1.03	0.93
apolipoprotein M	Apom	100763606	1.11	1.28	1.13	1.76	1.20	1.50	51.65	52.16	53.41	81.12	61.06	56.08
apolipoprotein O	Apoo	100768153	37.72	38.35	33.53	33.73	41.88	37.02	1020.82	1074.25	889.33	915.55	1122.96	948.69
apolipoprotein O-like	Apool	100755508	12.86	13.16	12.38	13.00	14.05	12.74	468.64	499.61	459.57	477.47	515.42	461.73
apoptogenic 1, mitochondrial	Apopt1	100750367	28.28	28.29	31.05	32.57	33.11	29.81	637.25	638.69	700.54	719.69	746.23	679.50

amyloid beta (A4) precursor protein	App	100689222	283.16	284.96	285.22	273.55	241.21	248.67	27326.70	27549.83	27715.93	26161.46	22963.24	23846.23
amyloid beta precursor protein (cytoplasmic tail) binding protein 2	Appbp2	100764050	24.09	24.95	12.95	14.49	13.99	13.54	2654.59	2769.81	1484.29	1625.96	1527.64	1483.32
adaptor protein, phosphotyrosine interaction, PH domain and leucine zipper containing 1	Appl1	100751324	17.33	17.71	15.58	18.03	20.80	19.06	1253.24	1259.09	1140.24	1285.24	1467.61	1383.31
adaptor protein, phosphotyrosine interaction, PH domain and leucine zipper containing 2	Appl2	100771802	7.05	8.03	6.39	7.57	9.12	8.19	634.22	712.81	593.72	695.35	812.46	744.93
adenine phosphoribosyltransferase	Aprt	100765665	185.07	179.16	148.68	107.79	74.02	84.34	4045.31	3926.41	3238.12	2287.71	1578.35	1803.91
aprataxin	Aptx	100774231	7.13	7.14	7.01	7.33	8.11	7.81	461.80	462.09	458.33	470.52	506.11	501.92
aquaporin 1 (Colton blood group)	Aqp1	100773231	1.09	1.20	0.87	1.28	1.49	1.43	73.68	83.27	57.14	84.60	98.32	93.47
aquaporin 10	Aqp10	100751558	0.00	0.00	0.00	0.00	0.00	0.00	0.00	0.92	0.00	0.00	0.00	0.00
aquaporin 11	Aqp11	100768567	0.41	0.49	0.30	0.44	0.63	0.54	25.82	24.71	14.91	20.86	36.22	34.58
aquaporin 2 (collecting duct)	Aqp2	100773344	0.00	0.00	0.00	0.00	0.00	0.00	0.00	0.00	0.00	0.00	0.00	0.00
aquaporin 3 (Gill blood group)	Aqp3	100758981	1.61	1.73	0.68	0.56	0.60	1.05	75.95	78.69	32.29	26.66	26.91	49.54
aquaporin 4	Aqp4	100770342	0.00	0.00	0.00	0.00	0.00	0.00	0.00	0.00	0.00	0.00	0.00	0.00
aquaporin 5	Aqp5	100773918	0.00	0.05	0.00	0.00	0.00	0.00	0.00	0.92	0.00	0.00	0.00	0.00
aquaporin 6, kidney specific	Aqp6	100750886	0.00	0.00	0.00	0.00	0.00	0.00	0.00	0.00	0.00	0.00	0.00	0.00
aquaporin 7	Aqp7	100760137	0.00	0.00	0.00	0.00	0.00	0.00	0.00	0.00	0.00	0.00	0.00	0.00
aquaporin 8	Aqp8	100762692	0.00	0.00	0.00	0.00	0.00	0.00	0.00	0.00	0.00	0.00	0.00	0.00
aquaporin 9	Aqp9	100772598	0.00	0.00	0.00	0.00	0.00	0.00	0.00	0.00	0.00	0.00	0.00	0.00
aquarius intron-binding spliceosomal factor	Aqr	100766095	26.56	29.18	27.79	30.73	32.43	30.48	3845.55	4244.84	4045.48	4399.26	4680.21	4370.52
androgen receptor	Ar	100752675	0.00	0.00	0.00	0.00	0.00	0.00	0.00	0.00	0.00	0.00	0.00	0.00
v-raf murine sarcoma 3611 viral oncogene homolog	Araf	100751432	39.53	39.44	35.00	34.03	32.80	33.77	1179.56	1216.08	1112.91	1002.47	909.75	963.64
ArfGAP with RhoGAP domain, ankyrin repeat and PH domain 1	Arap1	100774696	9.82	9.62	11.88	9.35	7.91	8.51	1518.32	1491.50	1817.17	1434.74	1213.00	1304.80
ArfGAP with RhoGAP domain, ankyrin repeat and PH domain 2	Arap2	100751597	0.01	0.02	0.00	0.00	0.00	0.00	0.76	2.75	0.00	0.00	0.00	0.00
ArfGAP with RhoGAP domain, ankyrin repeat and PH domain 3	Arap3	100752046	7.05	6.94	6.13	5.89	5.29	5.24	1270.71	1255.43	1105.46	1059.25	933.56	938.41
activity-regulated cytoskeleton-associated protein	Arc	100766738	0.00	0.00	0.04	0.00	0.00	0.00	0.00	0.00	1.24	0.00	0.00	0.00
archain 1	Arcn1	100758971	74.76	76.90	73.79	82.68	87.97	84.08	7457.92	7700.01	7409.05	8205.15	8712.51	8421.37
amphiregulin	Areg	100772017	0.47	0.25	0.00	0.00	0.00	0.00	21.27	11.90	0.00	0.00	0.00	0.00
apoptosis resistant E3 ubiquitin protein ligase 1	Arel1	100771045	19.24	18.05	21.02	22.34	24.03	23.87	2271.78	2170.46	2550.00	2709.55	2877.26	2913.37
ADP-ribosylation factor 1	Arf1	100757864	239.96	249.73	228.50	182.66	166.37	222.67	1455.28	1551.90	1384.93	1237.73	1136.41	1303.86
ADP-ribosylation factor 3	Arf3	100766631	68.38	66.63	72.21	69.66	69.20	74.39	1762.89	1774.25	1915.30	1812.55	1825.72	1982.43
ADP-ribosylation factor 4	Arf4	100769922	103.40	106.94	118.43	120.19	122.85	123.82	4496.47	4692.29	5186.95	5161.83	5316.72	5331.36
ADP-ribosylation factor 5	Arf5	100759676	53.87	50.51	55.03	39.86	37.81	38.44	1317.04	1272.81	1392.38	1066.21	1016.36	1038.42
ADP-ribosylation factor 6	Arf6	100774654	65.87	67.76	76.50	81.18	83.41	75.11	5917.57	6099.61	6927.12	7311.63	7457.08	6778.22
ADP-ribosylation factor GTPase activating protein 1	Arfgap1	100759225	50.21	48.00	49.69	43.77	36.51	39.85	4011.89	3878.83	3968.47	3508.05	2956.96	3224.61
ADP-ribosylation factor GTPase activating protein 2	Arfgap2	100755528	35.49	36.68	34.54	36.49	35.36	36.08	2362.93	2434.90	2330.15	2488.20	2428.08	2473.14
ADP-ribosylation factor GTPase activating protein 3	Arfgap3	100767017	14.43	14.47	15.14	16.22	16.99	19.02	1149.94	1168.50	1206.07	1295.67	1347.55	1486.12

ADP-ribosylation factor guanine nucleotide-exchange factor 1 (brefeldin A-inhibited)	Arfgef1	100750748	26.35	25.34	24.29	29.64	28.55	27.11	2590.79	2573.99	2500.32	2948.29	2910.38	2698.39
ADP-ribosylation factor guanine nucleotide-exchange factor 2 (brefeldin A-inhibited)	Arfgef2	100773989	16.25	16.35	17.33	18.79	21.61	21.96	4325.58	4417.78	4705.02	5033.19	5712.09	5830.47
ADP-ribosylation factor interacting protein 1	Arfip1	100761181	18.75	19.13	20.07	21.16	25.07	24.96	1538.83	1586.67	1623.41	1679.27	1983.03	1969.35
ADP-ribosylation factor interacting protein 2	Arfip2	100760054	15.17	14.06	15.92	13.51	10.82	12.00	1468.19	1389.02	1541.43	1332.76	1056.72	1173.01
ADP-ribosylation factor related protein 1	Arfrp1	100762329	53.35	53.67	63.17	61.22	68.78	71.20	2432.04	2283.01	2685.39	2572.80	2801.71	2998.42
arginase 1	Arg1	100750727	0.00	0.00	0.00	0.00	0.00	0.00	0.00	0.00	0.00	2.32	0.00	0.00
arginase 2	Arg2	100760459	0.00	0.03	0.00	0.00	0.00	0.00	0.00	3.66	1.24	1.16	0.00	2.80
arginine and glutamate rich 1	Arglu1	100756104	79.20	80.21	78.53	78.99	85.50	91.93	2341.66	2424.84	2330.15	2462.70	2675.44	2791.86
Rho GTPase activating protein 1	Arhgap1	100754369	40.66	38.07	42.79	44.96	47.56	50.15	2642.44	2523.66	2870.46	2963.36	3128.76	3290.97
Rho GTPase activating protein 10	Arhgap10	100775038	11.33	11.34	13.32	13.90	13.99	14.19	1088.42	1132.81	1352.63	1399.98	1389.99	1404.81
Rho GTPase activating protein 11A	Arhgap11a	100757746	24.61	24.03	22.95	26.21	26.72	24.31	3109.55	3035.17	2906.48	3295.97	3341.97	3061.04
Rho GTPase activating protein 12	Arhgap12	100770897	12.59	11.51	12.55	13.74	15.59	14.61	1746.94	1608.63	1743.89	1919.17	2140.35	2005.80
Rho GTPase activating protein 15	Arhgap15	100765262	0.19	0.07	0.06	0.12	0.03	0.02	6.08	1.83	2.48	2.32	2.07	0.93
Rho GTPase activating protein 17	Arhgap17	100759183	7.37	6.97	6.15	6.61	6.63	6.74	750.42	738.43	654.58	713.89	702.76	707.54
Rho GTPase activating protein 18	Arhgap18	100774646	16.35	16.08	7.63	8.30	9.90	9.77	768.65	777.78	380.08	385.92	476.09	451.45
Rho GTPase activating protein 19	Arhgap19	100765788	12.20	12.56	12.49	12.97	15.18	15.92	1836.57	1895.03	1884.25	1955.10	2274.90	2400.23
Rho GTPase activating protein 20	Arhgap20	100753596	0.65	0.66	0.39	0.37	0.36	0.31	117.73	118.95	68.31	66.06	66.24	55.15
Rho GTPase activating protein 21	Arhgap21	100766620	30.32	29.02	30.90	36.63	34.92	32.94	6325.44	6091.38	6535.86	7667.41	7304.93	6910.01
Rho GTPase activating protein 22	Arhgap22	100771443	24.60	24.70	22.32	17.25	11.66	13.44	1958.09	1959.09	1799.78	1379.11	961.50	1092.63
Rho GTPase activating protein 23	Arhgap23	100755359	10.94	10.51	7.02	5.79	4.96	5.04	1882.90	1806.28	1193.65	973.49	844.55	874.85
Rho GTPase activating protein 24	Arhgap24	100761525	29.06	31.33	42.14	41.32	34.95	34.47	1877.58	2030.46	2712.72	2726.94	2380.47	2371.26
Rho GTPase activating protein 25	Arhgap25	100766511	23.36	20.66	13.14	15.01	14.36	14.72	2037.84	1844.71	1136.51	1291.04	1262.68	1331.90
Rho GTPase activating protein 26	Arhgap26	100765139	0.08	0.10	0.06	0.06	0.00	0.04	9.11	13.73	7.45	5.79	0.00	3.74
Rho GTPase activating protein 27	Arhgap27	100765871	0.12	0.16	0.06	0.07	0.07	0.03	18.23	24.71	9.94	11.59	13.45	5.61
Rho GTPase activating protein 28	Arhgap28	100765840	1.06	1.06	0.00	0.00	0.00	0.00	169.38	164.71	0.00	0.00	0.00	0.00
Rho GTPase activating protein 29	Arhgap29	100773090	18.12	18.11	13.12	15.47	14.75	14.74	2744.97	2804.58	2038.26	2376.94	2257.31	2216.10
Rho GTPase activating protein 30	Arhgap30	100765257	0.01	0.02	0.00	0.00	0.01	0.00	0.76	1.83	0.00	0.00	1.03	0.00

Rho GTPase activating protein 31	Arhgap31	100758592	13.76	13.87	12.28	13.78	13.54	13.41	3432.36	3474.38	3110.19	3452.42	3376.13	3382.57
Rho GTPase activating protein 32	Arhgap32	100767169	5.71	6.30	6.23	6.89	6.84	6.92	1678.58	1841.96	1823.38	1999.14	1978.89	2017.02
Rho GTPase activating protein 33	Arhgap33	100774198	1.03	0.91	0.75	0.49	0.61	0.65	83.55	82.35	74.53	61.42	44.50	55.15
Rho GTPase activating protein 35	Arhgap35	100752481	13.58	13.41	14.08	15.07	15.14	15.03	1981.64	1952.68	2066.83	2182.25	2176.58	2171.24
Rho GTPase activating protein 36	Arhgap36	100761307	0.02	0.00	0.00	0.00	0.00	0.00	0.76	0.00	0.00	1.16	0.00	0.00
Rho GTPase activating protein 39	Arhgap39	100759161	8.80	8.73	9.47	7.95	5.70	6.24	846.89	848.24	937.78	750.98	539.23	594.45
Rho GTPase activating protein 4	Arhgap4	100775066	0.03	0.04	0.06	0.07	0.08	0.07	3.04	6.41	7.45	5.79	6.21	6.54
Rho GTPase activating protein 40	Arhgap40	100760450	0.00	0.02	0.00	0.00	0.04	0.00	0.00	0.92	0.00	0.00	2.07	0.00
Rho GTPase activating protein 42	Arhgap42	100757941	0.00	0.00	0.00	0.00	0.00	0.00	0.00	0.00	0.00	0.00	0.00	0.00
Rho GTPase activating protein 44	Arhgap44	100772129	0.00	0.00	0.00	0.00	0.00	0.00	0.00	0.00	3.73	4.64	0.00	1.87
Rho GTPase activating protein 5	Arhgap5	100765474	11.75	11.60	10.54	12.94	14.25	12.30	2400.90	2377.26	2177.38	2665.52	2899.00	2526.41
Rho GTPase activating protein 6	Arhgap6	100763825	4.69	5.14	5.07	6.18	5.61	4.89	369.90	397.12	388.77	469.36	428.48	377.61
Rho GTPase activating protein 8	Arhgap8	100760345	2.52	4.39	6.29	3.30	2.83	2.88	48.61	51.24	75.77	81.12	60.03	50.47
Rho GTPase activating protein 9	Arhgap9	100756457	0.29	0.19	0.16	0.13	0.12	0.14	20.51	12.81	17.39	16.22	10.35	12.15
Rho GDP dissociation inhibitor (GDI) alpha	Arhgdia	100767064	396.74	366.40	152.06	122.44	69.32	79.03	20332.11	19062.89	7787.88	6112.14	3543.79	3936.83
Rho GDP dissociation inhibitor (GDI) beta	Arhgdib	100753905	0.04	0.05	0.00	0.00	0.00	0.00	1.52	0.92	0.00	0.00	0.00	0.00
Rho GDP dissociation inhibitor (GDI) gamma	Arhgdig	100757877	0.03	0.00	0.16	0.05	0.00	0.24	1.52	0.92	3.73	2.32	1.03	6.54
Rho guanine nucleotide exchange factor (GEF) 1	Arhgef1	100758784	15.70	14.26	16.47	12.87	7.76	8.64	1579.84	1479.61	1715.32	1315.37	843.51	913.17
Rho guanine nucleotide exchange factor (GEF) 10	Arhgef10	100757483	15.23	15.78	13.51	13.49	12.81	13.69	2580.15	2660.00	2330.15	2324.79	2186.93	2305.83
Rho guanine nucleotide exchange factor (GEF) 11	Arhgef11	100760875	6.12	6.25	6.34	6.03	5.86	6.28	1174.25	1213.33	1229.67	1149.65	1125.03	1199.18
Rho guanine nucleotide exchange factor (GEF) 12	Arhgef12	100765006	41.02	40.76	43.96	49.88	51.10	50.31	10066.17	10011.38	10832.24	12223.13	12411.56	12327.36
Rho guanine nucleotide exchange factor (GEF) 15	Arhgef15	100752685	0.19	0.19	0.06	0.01	0.02	0.03	23.55	21.05	6.21	1.16	2.07	3.74
Rho guanine nucleotide exchange factor (GEF) 16	Arhgef16	100768964	0.14	0.13	0.04	0.00	0.01	0.03	12.15	11.90	3.73	0.00	1.03	2.80
Rho guanine nucleotide exchange factor (GEF) 17	Arhgef17	100753083	5.46	5.18	4.90	4.01	2.86	3.53	1778.08	1647.06	1601.05	1284.08	919.07	1198.25
Rho/Rac guanine nucleotide exchange factor (GEF) 18	Arhgef18	100770929	10.94	10.39	12.58	11.73	10.39	10.75	1539.59	1462.22	1786.12	1664.21	1473.82	1522.58
Rho guanine nucleotide exchange factor (GEF) 19	Arhgef19	100752548	1.17	1.11	0.44	0.23	0.07	0.21	89.63	89.67	36.02	16.22	8.28	18.69
Rho/Rac guanine nucleotide exchange factor (GEF) 2	Arhgef2	100758849	37.51	37.42	38.75	36.68	33.60	34.37	4775.98	4801.18	5005.61	4670.45	4284.84	4413.51

Rho guanine nucleotide exchange factor (GEF) 25	Arhgef25	100758200	0.02	0.04	0.04	0.04	0.02	0.05	2.28	2.75	3.73	3.48	2.07	2.80
Rho guanine nucleotide exchange factor (GEF) 26	Arhgef26	100772368	0.02	0.00	0.00	0.00	0.00	0.00	2.28	0.00	0.00	0.00	0.00	0.00
Rho guanine nucleotide exchange factor (GEF) 28	Arhgef28	100752236	19.66	19.43	29.19	29.86	30.13	30.99	1958.85	1965.49	2938.78	2985.38	3036.65	3029.26
Rho guanine nucleotide exchange factor (GEF) 3	Arhgef3	100751908	0.03	0.01	0.00	0.03	0.00	0.01	2.28	0.92	0.00	2.32	0.00	0.93
Rho guanine nucleotide exchange factor (GEF) 33	Arhgef33	100757802	0.04	0.08	0.03	0.00	0.01	0.04	4.56	7.32	3.73	0.00	1.03	4.67
Rho guanine nucleotide exchange factor (GEF) 37	Arhgef37	100756858	0.30	0.15	0.04	0.07	0.09	0.05	26.58	18.30	2.48	5.79	6.21	6.54
Rho guanine nucleotide exchange factor (GEF) 39	Arhgef39	100773515	0.11	0.02	0.24	0.14	0.08	0.02	9.11	6.41	11.18	12.75	13.45	12.15
Rho guanine nucleotide exchange factor (GEF) 4	Arhgef4	100774841	2.18	2.16	2.80	2.17	2.19	1.62	161.02	163.79	180.10	146.02	142.83	109.36
Rho guanine nucleotide exchange factor (GEF) 40	Arhgef40	100758097	11.87	11.39	11.86	10.50	8.93	10.02	1665.67	1608.63	1678.06	1470.67	1249.23	1419.76
Rho guanine nucleotide exchange factor (GEF) 5	Arhgef5	100756445	21.37	20.39	28.16	29.02	29.83	29.58	3317.67	3155.95	4415.62	4501.24	4566.36	4594.84
Rac/Cdc42 guanine nucleotide exchange factor (GEF) 6	Arhgef6	100762795	13.31	13.33	14.47	15.44	14.79	15.26	1823.65	1889.54	2035.78	2153.27	2025.47	2081.51
Rho guanine nucleotide exchange factor (GEF) 7	Arhgef7	100766623	24.80	24.84	27.56	27.49	24.40	24.98	3302.48	3340.79	3651.74	3629.74	3189.83	3275.08
Cdc42 guanine nucleotide exchange factor (GEF) 9	Arhgef9	100768180	0.00	0.00	0.00	0.00	0.00	0.00	0.00	0.00	0.00	0.00	0.00	0.00
AT rich interactive domain 1A (SWI-like)	Arid1a	100771997	18.90	18.40	18.11	18.27	14.07	14.84	3442.99	3378.30	3390.90	3388.68	2600.92	2736.71
AT rich interactive domain 1B (SWI1-like)	Arid1b	100773316	9.50	9.09	9.78	9.22	8.05	7.65	1879.10	1795.30	1945.11	1835.73	1595.95	1544.07
AT rich interactive domain 2 (ARID, RFX-like)	Arid2	100754162	7.06	7.08	7.09	7.99	7.81	7.56	1526.67	1502.49	1532.74	1721.00	1650.80	1643.15
AT rich interactive domain 3A (BRIGHT-like)	Arid3a	100764162	2.10	2.13	1.43	0.98	0.98	0.82	144.31	140.00	99.37	67.22	68.31	57.95
AT rich interactive domain 3B (BRIGHT-like)	Arid3b	100751851	0.48	0.38	0.35	0.39	0.44	0.47	62.28	56.73	47.20	50.99	60.03	64.49
AT rich interactive domain 3C (BRIGHT-like)	Arid3c	100753126	0.03	0.00	0.00	0.00	0.05	0.00	6.84	3.66	0.00	1.16	3.10	0.93
AT rich interactive domain 4A (RBP1-like)	Arid4a	100760045	7.41	7.09	8.35	9.30	10.04	8.73	830.18	793.33	936.53	1019.85	1103.30	977.66
AT rich interactive domain 4B (RBP1-like)	Arid4b	100752333	7.07	6.90	7.47	8.37	9.26	8.33	1203.87	1180.39	1278.11	1413.88	1568.00	1410.42
AT rich interactive domain 5A (MRF1-like)	Arid5a	100773678	7.57	7.28	6.31	5.62	4.11	4.62	998.03	952.55	832.20	726.64	538.19	612.21
AT rich interactive domain 5B (MRF1-like)	Arid5b	100754803	21.39	21.15	29.86	32.32	29.96	29.77	3528.82	3556.74	4954.68	5482.85	4885.14	4881.78
ariadne RBR E3 ubiquitin protein ligase 1	Arih1	100755555	31.05	31.40	33.43	38.18	38.61	36.88	4309.63	4367.46	4680.18	5292.79	5327.07	5079.00
ariadne RBR E3 ubiquitin protein ligase 2	Arih2	100764705	24.06	23.08	26.64	28.19	27.82	28.60	1813.78	1739.48	2019.63	2042.02	1971.65	2027.30
ADP-ribosylation factor-like 1	Arl1	100751351	104.30	100.98	109.74	121.76	121.14	123.11	1799.35	1733.07	1892.94	2073.31	2031.68	2097.40
ADP-ribosylation factor-like 10	Arl10	100768218	33.63	32.60	35.02	34.85	34.10	36.10	2188.99	2130.20	2297.86	2298.14	2234.54	2367.52
ADP-ribosylation factor-like 11	Arl11	100764642	0.63	0.48	0.17	0.26	0.28	0.26	31.14	24.71	9.94	11.59	13.45	13.09
ADP-ribosylation factor-like 13A	Arl13a	100750956	0.09	0.08	0.14	0.07	0.06	0.08	9.87	7.32	12.42	8.11	13.45	7.48

ADP-ribosylation factor-like 13B	Arl13b	100768683	14.08	14.25	14.11	15.65	18.56	16.14	1342.87	1367.98	1353.87	1482.26	1750.16	1531.92
ADP-ribosylation factor-like 14	Arl14	100757028	0.00	0.00	0.00	0.12	0.16	0.10	0.00	0.00	0.00	2.32	4.14	1.87
ADP-ribosylation factor-like 14 effector protein	Arl14ep	100765495	61.89	63.45	62.66	63.09	65.65	64.72	4321.02	4511.12	4369.66	4351.74	4548.76	4505.11
ADP-ribosylation factor-like 14 effector protein-like	Arl14epl	100769406	0.00	0.00	0.00	0.00	0.00	0.00	0.76	0.00	0.00	0.00	0.00	0.00
ADP-ribosylation factor-like 15	Arl15	100750883	10.22	10.16	9.06	11.12	11.28	11.09	375.97	394.38	349.03	442.71	428.48	419.67
ADP-ribosylation factor-like 16	Arl16	100765389	34.20	31.39	24.36	24.32	19.08	21.33	1076.27	1055.95	725.38	761.41	610.64	672.96
ADP-ribosylation factor-like 2	Arl2	100760039	74.79	73.06	76.75	57.74	47.46	50.82	1836.57	1840.13	1900.39	1382.59	1170.57	1221.61
ADP-ribosylation factor-like 2 binding protein	Arl2bp	100763207	66.94	64.89	65.12	67.17	73.33	73.59	3384.51	3275.82	3320.10	3372.46	3663.85	3691.01
ADP-ribosylation factor-like 3	Arl3	100757638	114.16	110.33	109.88	100.19	98.61	99.22	2757.13	2670.07	2634.47	2390.85	2339.07	2355.37
ADP-ribosylation factor-like 4A	Arl4a	100771484	8.35	7.85	4.55	4.09	4.48	4.99	310.65	288.24	162.71	155.30	158.35	185.06
ADP-ribosylation factor-like 4C	Arl4c	100772909	16.55	16.07	20.60	19.45	18.92	18.74	1654.28	1599.48	2080.50	1949.30	1866.08	1868.40
ADP-ribosylation factor-like 5A	Arl5a	100765602	27.64	27.49	30.81	36.23	36.07	34.15	4016.44	4004.19	4511.26	5255.70	5205.98	4973.38
ADP-ribosylation factor-like 5B	Arl5b	100773320	1.79	1.96	2.12	2.09	2.46	2.11	205.84	222.35	244.69	241.06	278.41	238.34
ADP-ribosylation factor-like 5C	Arl5c	100766973	0.00	0.00	0.00	0.00	0.00	0.00	0.00	0.92	0.00	0.00	0.00	0.00
ADP-ribosylation factor-like 6	Arl6	100773053	9.42	10.72	10.22	10.60	13.31	13.34	388.88	440.13	394.98	426.48	526.81	515.94
ADP-ribosylation-like factor 6 interacting protein 4	Arl6ip4	100772007	44.51	45.14	49.48	38.25	27.94	29.65	2119.11	2145.75	2384.81	1841.52	1372.39	1432.85
ADP-ribosylation-like factor 6 interacting protein 5	Arl6ip5	100756195	48.78	49.06	38.47	39.25	48.77	44.16	1738.59	1750.46	1379.96	1384.91	1713.94	1576.79
ADP-ribosylation-like factor 6 interacting protein 6	Arl6ip6	100774425	8.93	9.39	8.73	9.61	10.84	9.75	775.49	814.38	853.31	931.77	1117.79	1025.33
ADP-ribosylation factor-like 8A	Arl8a	100762890	33.73	32.61	38.90	36.74	32.64	32.64	1421.10	1397.26	1643.28	1571.50	1349.62	1389.85
ADP-ribosylation factor-like 8B	Arl8b	100765619	33.75	35.33	33.59	34.45	41.25	42.07	4305.07	4511.12	4307.56	4384.19	5217.37	5373.42
ADP-ribosylation factor-like 9	Arl9	100759276	11.45	11.76	16.13	20.02	17.24	17.84	550.67	564.58	781.27	964.22	836.27	860.83
armadillo repeat containing 1	Armc1	100772139	51.59	51.92	51.78	53.33	62.05	63.11	2336.34	2355.30	2440.70	2543.83	2767.55	2710.54
armadillo repeat containing 10	Armc10	100761091	33.64	35.53	32.07	33.93	40.21	37.99	1610.98	1719.35	1548.88	1615.53	1897.13	1797.37
armadillo repeat containing 12	Armc12	100760615	0.00	0.00	0.00	0.00	0.00	0.00	0.00	0.00	1.24	0.00	0.00	0.00
armadillo repeat containing 2	Armc2	100751417	0.56	0.35	0.43	0.44	0.53	0.62	63.04	36.60	48.44	50.99	55.89	66.36
armadillo repeat containing 3	Armc3	100751830	0.00	0.00	0.00	0.00	0.00	0.00	0.00	0.00	0.00	0.00	0.00	0.00
armadillo repeat containing 4	Armc4	100773009	0.00	0.00	0.00	0.00	0.00	0.00	0.00	0.00	0.00	0.00	0.00	0.00
armadillo repeat containing 5	Armc5	100772037	10.43	10.01	9.14	6.83	4.09	4.23	949.42	897.65	842.13	618.86	388.12	392.56
armadillo repeat containing 6	Armc6	100752507	16.91	16.11	15.58	9.90	7.04	8.07	1093.74	1057.78	1045.84	702.31	529.91	642.12
armadillo repeat containing 7	Armc7	100770961	12.27	11.27	15.69	13.41	12.73	13.92	865.12	796.99	1084.34	952.63	875.60	978.60
armadillo repeat containing 8	Armc8	100768239	5.54	6.24	2.86	2.82	3.42	3.17	919.80	1008.37	449.64	438.07	548.54	504.72
armadillo repeat containing 9	Armc9	100754576	8.02	7.99	10.27	9.38	9.55	9.84	420.78	452.94	547.76	512.24	543.37	545.85
armadillo repeat containing, X-linked 1	Armcx1	100773690	11.41	12.86	16.42	15.50	15.82	16.04	661.56	725.62	934.05	874.98	905.61	920.65
armadillo repeat containing, X-linked 2	Armcx2	100765185	0.00	0.00	0.00	0.00	0.00	0.00	0.00	0.00	0.00	0.00	0.00	0.00
armadillo repeat containing, X-linked 3	Armcx3	100765475	10.35	10.78	10.53	10.57	13.53	12.28	642.57	684.45	667.00	669.86	849.72	775.78
armadillo repeat containing, X-linked 4	Armcx4	100753309	0.03	0.03	0.05	0.04	0.06	0.03	7.60	7.32	11.18	9.27	12.42	7.48
armadillo repeat containing, X-linked 5	Armcx5	100769784	17.39	17.83	20.21	25.27	23.10	24.62	854.48	864.71	982.49	1098.66	1055.69	1046.83

aryl hydrocarbon receptor nuclear translocator	Arnt	100752106	20.97	20.61	20.72	21.88	20.96	21.39	3450.59	3476.21	3531.25	3648.28	3515.85	3551.74
aryl-hydrocarbon receptor nuclear translocator 2	Arnt2	100773566	1.30	1.30	1.49	1.31	2.65	2.69	192.16	180.26	207.43	188.90	374.67	387.89
aryl hydrocarbon receptor nuclear translocator-like	Arntl	100755141	14.35	13.50	13.83	13.91	15.06	15.70	738.27	707.32	710.47	712.74	767.96	786.06
aryl hydrocarbon receptor nuclear translocator-like 2	Arntl2	100758300	0.92	1.19	0.56	0.42	0.86	0.68	60.00	85.10	42.23	28.97	62.10	47.67
actin related protein 2/3 complex, subunit 1A, 41kDa	Arpc1a	100775083	67.91	67.80	69.76	67.22	60.10	62.38	3045.75	3108.37	3177.26	3050.28	2711.66	2781.58
actin related protein 2/3 complex, subunit 1B, 41kDa	Arpc1b	100774796	417.70	403.89	441.55	331.45	266.73	290.53	17663.84	17497.27	19126.90	14172.43	11443.84	12215.20
actin related protein 2/3 complex, subunit 2, 34kDa	Arpc2	100774430	223.04	223.72	234.44	254.28	264.33	258.61	8229.61	8346.02	8633.75	9063.91	9482.54	9251.36
actin related protein 2/3 complex, subunit 3, 21kDa	Arpc3	100764746	154.42	150.69	155.37	159.46	152.57	158.19	4005.81	3888.89	3995.79	4023.77	3850.15	4034.03
actin related protein 2/3 complex, subunit 4, 20kDa	Arpc4	100756023	75.16	72.21	81.50	69.04	65.78	74.13	3249.31	3123.01	3541.19	2930.91	2746.85	3111.52
actin related protein 2/3 complex, subunit 5, 16kDa	Arpc5	100766799	58.96	64.70	54.57	61.79	69.54	68.26	2922.71	3155.04	2740.04	3050.28	3436.15	3349.86
actin related protein 2/3 complex, subunit 5-like	Arpc5l	100751476	54.39	52.40	83.90	93.85	91.83	98.99	1139.31	1099.87	1745.13	1892.52	1852.63	1997.39
cAMP-regulated phosphoprotein, 19kDa	Arpp19	100765476	21.71	22.08	23.30	26.01	27.77	26.41	2315.84	2347.98	2510.26	2780.25	2953.85	2883.46
cAMP-regulated phosphoprotein, 21kDa	Arpp21	100769175	0.06	0.04	3.70	3.81	4.33	3.74	6.08	7.32	391.26	394.03	478.16	421.54
arrestin 3, retinal (X-arrestin)	Arr3	100770938	0.00	0.00	0.00	0.00	0.00	0.00	0.00	0.00	0.00	0.00	0.00	0.00
arrestin, beta 1	Arrb1	100754574	6.71	6.48	4.71	4.53	4.59	5.65	439.77	420.92	313.01	296.68	311.53	370.13
arrestin, beta 2	Arrb2	100750830	7.73	7.79	7.59	7.46	7.80	8.08	360.78	375.16	358.96	355.79	366.39	378.54
arrestin domain containing 1	Arrdc1	100754257	10.40	10.95	10.04	9.26	6.88	7.79	490.66	516.08	474.48	441.55	330.16	370.13
arrestin domain containing 2	Arrdc2	100771761	2.52	2.59	2.48	2.14	1.73	2.09	149.63	173.86	145.32	122.85	102.46	115.90
arrestin domain containing 3	Arrdc3	100755882	2.49	2.97	3.61	4.91	5.16	4.98	281.79	329.41	401.19	535.42	577.52	559.87
arrestin domain containing 4	Arrdc4	100770261	0.00	0.00	0.00	0.00	0.00	0.00	0.00	0.00	0.00	0.00	0.00	0.00
arrestin domain containing 5	Arrdc5	100769962	0.00	0.08	0.00	0.00	0.23	0.00	1.52	4.58	0.00	0.00	6.21	1.87
arylsulfatase A	Arsa	100754567	38.82	34.98	46.89	40.18	32.38	36.87	2397.11	2147.58	2926.36	2521.81	2152.77	2395.56
arylsulfatase B	Arsb	100759794	0.02	0.02	0.00	0.00	0.00	0.00	1.52	1.83	0.00	0.00	0.00	0.00
arylsulfatase E (chondrodysplasia punctata 1)	Arse	100773060	0.00	0.02	0.00	0.00	0.00	0.00	0.00	1.83	0.00	0.00	0.00	0.00
arylsulfatase G	Arsg	100751256	20.56	19.74	4.98	5.34	4.26	4.39	1244.13	1247.19	442.18	464.73	378.80	381.35
arylsulfatase family, member H	Arsh	100773352	0.00	0.00	0.00	0.00	0.00	0.00	0.00	0.00	0.00	0.00	0.00	0.00
arylsulfatase family, member I	Arsi	100765546	0.00	0.00	0.00	0.00	0.00	0.00	0.00	0.00	0.00	1.16	0.00	0.00
arylsulfatase family, member J	Arsj	100752184	5.75	5.99	7.77	7.67	8.69	8.24	356.22	388.89	500.56	479.79	539.23	512.20
arylsulfatase family, member K	Arsk	100762898	7.86	9.00	9.62	9.44	11.10	11.61	328.88	326.67	335.36	339.56	431.59	405.65
ADP-ribosyltransferase 1	Art1	100759929	0.00	0.00	0.00	0.00	0.00	0.00	0.00	0.00	0.00	0.00	0.00	0.00
ADP-ribosyltransferase 3	Art3	100771336	0.30	0.22	0.04	0.04	0.06	0.09	10.63	8.24	2.48	2.32	2.07	3.74
ADP-ribosyltransferase 4 (Dombrock blood group)	Art4	100757103	0.00	0.00	0.00	0.00	0.00	0.00	0.00	0.00	0.00	0.00	0.00	0.00
ADP-ribosyltransferase 5	Art5	100759641	0.00	0.00	0.00	0.00	0.00	0.00	0.76	1.83	0.00	1.16	0.00	0.93
artemin	Artn	100755266	0.99	1.03	0.53	0.19	0.34	0.46	57.72	61.31	31.05	12.75	20.70	28.04
ARV1 homolog (S. cerevisiae)	Arv1	100773285	11.55	11.01	14.78	14.10	16.26	14.78	407.87	402.61	511.74	470.52	543.37	492.57

armadillo repeat gene deleted in velocardiofacial syndrome	Arvcf	100773799	2.27	2.16	2.29	1.74	1.61	1.65	499.02	564.58	626.01	542.37	518.53	549.59
aristaless related homeobox	Arx	100759833	0.02	0.05	0.03	0.00	0.03	0.03	3.04	4.58	4.97	2.32	4.14	4.67
arsenic (+3 oxidation state) methyltransferase	As3mt	100758973	0.03	0.06	0.14	0.05	0.02	0.17	1.52	2.75	6.21	2.32	1.03	7.48
N-acylsphingosine amidohydrolase (acid ceramidase) 1	Asah1	100771068	96.40	99.44	98.52	103.32	116.32	118.65	5226.39	5479.22	5470.15	5573.25	6312.38	6437.07
N-acylsphingosine amidohydrolase (non-lysosomal ceramidase) 2	Asah2	100765841	0.00	0.01	0.00	0.02	0.00	0.00	0.00	0.92	0.00	1.16	0.00	0.00
ArfGAP with SH3 domain, ankyrin repeat and PH domain 2	Asap2	100759382	13.27	12.40	9.43	9.03	12.61	10.87	1374.01	1292.03	939.02	908.59	1195.41	1105.71
ArfGAP with SH3 domain, ankyrin repeat and PH domain 3	Asap3	100751283	1.04	1.16	0.35	0.37	0.25	0.32	131.40	146.41	48.44	48.67	32.08	40.19
ankyrin repeat and SOCS box containing 1	Asb1	100771136	15.38	15.72	13.03	12.97	12.96	11.61	662.32	670.72	562.67	584.10	583.73	570.15
ankyrin repeat and SOCS box containing 10	Asb10	100761357	0.00	0.00	0.00	0.00	0.00	0.00	0.00	0.00	0.00	2.32	0.00	0.00
ankyrin repeat and SOCS box containing 11, E3 ubiquitin protein ligase	Asb11	100774006	0.05	0.03	0.04	0.04	0.07	0.06	2.28	0.92	2.48	2.32	4.14	3.74
ankyrin repeat and SOCS box containing 12	Asb12	100751313	0.00	0.00	0.00	0.00	0.00	0.00	0.00	0.92	0.00	0.00	0.00	0.00
ankyrin repeat and SOCS box containing 13	Asb13	100766461	3.94	3.98	3.58	3.34	3.28	3.50	202.04	213.20	185.07	188.90	176.98	178.52
ankyrin repeat and SOCS box containing 14	Asb14	100751033	0.34	0.43	0.33	0.47	0.62	0.47	84.31	96.08	89.43	124.00	143.86	122.44
ankyrin repeat and SOCS box containing 15	Asb15	100759425	0.72	0.86	0.60	0.54	0.70	0.52	97.22	109.80	64.59	64.90	95.22	75.71
ankyrin repeat and SOCS box containing 16	Asb16	100753821	0.00	0.00	0.00	0.00	0.00	0.05	0.76	0.92	0.00	0.00	1.03	2.80
ankyrin repeat and SOCS box containing 17	Asb17	100759666	0.00	0.00	0.00	0.00	0.00	0.00	0.00	0.00	0.00	0.00	0.00	0.00
ankyrin repeat and SOCS box containing 18	Asb18	100768096	0.00	0.00	0.00	0.00	0.00	0.00	0.00	0.00	0.00	0.00	0.00	0.00
ankyrin repeat and SOCS box containing 2	Asb2	100764972	0.00	0.00	0.00	0.00	0.00	0.02	0.76	0.00	0.00	0.00	0.00	0.93
ankyrin repeat and SOCS box containing 3	Asb3	100773709	19.28	19.76	23.78	22.68	22.16	22.39	1314.76	1340.52	1598.57	1530.93	1499.70	1516.03
ankyrin repeat and SOCS box containing 4	Asb4	100763121	0.00	0.00	0.00	0.00	0.00	0.00	0.00	0.00	0.00	0.00	0.00	0.00
ankyrin repeat and SOCS box containing 5	Asb5	100752543	0.39	0.47	0.17	0.18	0.46	0.45	32.66	36.60	8.69	10.43	35.19	28.97
ankyrin repeat and SOCS box containing 7	Asb7	100767938	6.88	6.10	7.22	7.53	8.00	7.70	339.51	306.54	351.51	346.52	373.63	366.39
ankyrin repeat and SOCS box containing 8	Asb8	100753972	22.17	21.68	24.99	25.14	27.24	28.02	1298.81	1277.39	1474.36	1467.19	1583.53	1648.76
ankyrin repeat and SOCS box containing 9	Asb9	100774675	0.00	0.00	0.00	0.00	0.00	0.00	0.00	0.00	0.00	0.00	0.00	0.00
activating signal cointegrator 1 complex subunit 1	Ascc1	100759170	27.18	27.73	26.81	30.29	34.89	33.88	982.08	1012.03	973.80	1068.52	1241.98	1187.97
activating signal cointegrator 1 complex subunit 2	Ascc2	100775056	29.66	29.54	29.28	27.68	23.56	27.37	2400.90	2434.90	2433.25	2243.67	1919.90	2233.86
activating signal cointegrator 1 complex subunit 3	Ascc3	100762460	14.04	13.80	7.24	8.24	9.42	8.22	3037.40	2982.09	1553.85	1783.58	1972.68	1767.46

achaete-scute family bHLH transcription factor 1	Ascl1	100772145	0.00	0.00	0.00	0.00	0.00	0.00	0.00	0.00	0.00	0.00	0.00	0.00
achaete-scute family bHLH transcription factor 2	Ascl2	100762061	1.31	1.21	1.10	0.90	0.86	0.99	87.35	85.10	73.28	57.95	56.92	65.43
achaete-scute family bHLH transcription factor 3	Ascl3	100751483	0.00	0.00	0.00	0.00	0.00	0.09	0.00	0.00	0.00	0.00	0.00	0.93
achaete-scute family bHLH transcription factor 5	Ascl5	103163069	0.00	0.00	0.00	0.00	0.00	0.00	0.00	0.00	0.00	0.00	0.00	0.00
anti-silencing function 1A histone chaperone	Asf1a	103163586	54.96	56.32	23.71	31.33	32.69	31.42	543.07	533.46	248.42	304.80	324.99	311.25
anti-silencing function 1B histone chaperone	Asf1b	100769479	25.95	24.69	30.76	29.22	27.20	27.29	1162.85	1117.26	1378.72	1301.47	1203.69	1221.61
asialoglycoprotein receptor 1	Asgr1	100763164	0.00	0.06	0.00	0.00	0.00	0.07	1.52	0.92	0.00	0.00	0.00	1.87
asialoglycoprotein receptor 2	Asgr2	100768020	0.00	0.00	0.00	0.00	0.00	0.00	0.00	0.00	0.00	0.00	0.00	0.00
ash1 (absent, small, or homeotic)-like (Drosophila)	Ash1l	100754103	12.20	11.14	10.72	13.02	13.81	13.20	4197.97	3860.53	3706.39	4471.11	4708.15	4564.93
ash2 (absent, small, or homeotic)-like (Drosophila)	Ash2l	100771359	61.17	61.13	67.85	71.28	76.18	75.03	4315.70	4313.47	4723.66	4877.89	5165.62	5129.47
acid-sensing (proton-gated) ion channel 1	Asic1	100751471	0.05	0.01	0.00	0.03	0.01	0.01	4.56	1.83	0.00	3.48	2.07	2.80
acid-sensing (proton-gated) ion channel 2	Asic2	100762972	8.03	7.86	7.61	7.62	5.64	5.97	569.65	577.39	539.07	514.56	391.22	418.73
acid-sensing (proton-gated) ion channel 3	Asic3	100763386	0.00	0.00	0.00	0.03	0.00	0.00	0.00	1.83	0.00	1.16	0.00	0.00
acid-sensing (proton-gated) ion channel family member 4	Asic4	100765570	0.00	0.00	0.00	0.00	0.00	0.02	3.04	2.75	3.73	2.32	2.07	4.67
acid-sensing (proton-gated) ion channel family member 5	Asic5	100768408	0.00	0.00	0.00	0.00	0.00	0.00	0.76	0.00	0.00	0.00	0.00	0.00
agouti signaling protein	Asip	100750676	0.06	0.05	0.10	0.00	0.00	0.00	2.28	1.83	0.00	0.00	0.00	0.00
argininosuccinate lyase	Asl	100756687	5.01	4.78	4.01	4.53	2.94	3.23	284.83	260.78	236.00	243.37	168.70	184.13
arsA arsenite transporter, ATP-binding, homolog 1 (bacterial)	Asna1	100761936	123.91	120.19	139.04	135.74	113.51	116.82	4409.13	4259.48	4974.56	4795.61	3998.15	4168.63
asparagine synthetase (glutamine-hydrolyzing)	Asns	100760225	69.88	68.62	63.62	62.81	74.75	76.73	4504.07	4447.98	4090.19	3970.46	4699.87	4838.78
asparagine synthetase domain containing 1	Asnsd1	100765791	133.82	131.31	126.85	133.42	140.88	136.14	8004.78	7887.59	7613.99	7938.60	8409.26	8183.97
aspartoacylase	Aspa	100767695	0.13	0.00	0.00	0.00	0.00	0.00	5.32	0.00	0.00	0.00	0.00	0.00
aspartate dehydrogenase domain containing	Aspdh	100755598	0.00	0.00	0.00	0.00	0.00	0.00	0.76	0.00	0.00	2.32	2.07	0.00
asparaginase	Aspg	100750655	6.51	5.91	6.12	4.81	4.14	4.24	540.03	486.80	508.01	398.67	353.97	352.37
aspartate beta-hydroxylase	Asph	100755768	17.80	18.28	20.58	22.18	20.42	19.49	1331.47	1378.96	1517.83	1636.39	1505.91	1445.00
aspartate beta-hydroxylase domain containing 1	Asphd1	100774801	0.00	0.00	0.00	0.00	0.00	0.00	0.00	0.00	0.00	0.00	0.00	1.87
aspartate beta-hydroxylase domain containing 2	Asphd2	100773627	0.00	0.00	0.00	0.00	0.00	0.00	0.00	0.00	0.00	1.16	0.00	0.00
asp (abnormal spindle) homolog, microcephaly associated (Drosophila)	Aspm	100764564	7.98	7.43	5.42	6.82	7.24	6.48	2261.91	2125.62	1558.82	1951.62	2043.06	1845.04
asporin	Aspn	100754602	0.04	0.02	0.03	0.05	0.00	0.00	1.52	0.92	1.24	2.32	0.00	0.00
aspartic peptidase, retroviral-like 1	Asprv1	100773346	0.00	0.00	0.05	0.00	0.00	0.00	0.00	0.00	0.00	0.00	0.00	0.00
alveolar soft part sarcoma chromosome region, candidate 1	Aspscr1	100768779	34.41	32.18	17.19	16.10	10.44	11.10	1926.19	1827.32	956.41	909.75	579.59	612.21
asparaginase like 1	Asrgl1	100754434	35.60	34.86	38.22	39.27	37.39	38.73	2199.63	2118.30	2418.34	2427.94	2259.38	2389.02
argininosuccinate synthase 1	Ass1	100762274	0.00	0.00	0.08	0.00	0.00	0.00	0.00	0.00	1.24	0.00	0.00	0.00

asteroid homolog 1 (Drosophila)	Aste1	100765786	1.77	1.66	1.78	1.87	2.29	2.07	230.14	215.03	238.48	250.33	288.76	269.18
astacin-like metallo-endopeptidase (M12 family)	Astl	100750643	0.30	0.16	0.00	0.07	0.16	0.06	16.71	12.81	2.48	2.32	7.24	6.54
astrotactin 1	Astn1	100753322	0.00	0.00	0.00	0.00	0.00	0.00	0.00	0.00	0.00	0.00	0.00	0.00
astrotactin 2	Astn2	100750695	0.39	0.14	0.00	0.07	0.04	0.02	13.67	7.32	0.00	1.16	3.10	3.74
asunder spermatogenesis regulator	Asun	100757147	49.76	55.29	59.23	60.80	64.41	60.60	3755.16	4185.36	4447.91	4473.43	4788.88	4525.67
additional sex combs like 1 (Drosophila)	Asxl1	100761070	18.99	18.41	18.84	19.06	16.84	17.05	3871.37	3776.34	3890.22	3893.97	3418.56	3465.76
additional sex combs like 2 (Drosophila)	Asxl2	100756895	11.67	11.76	12.37	13.08	12.82	11.99	1565.41	1562.88	1673.09	1761.56	1689.10	1626.33
additional sex combs like 3 (Drosophila)	Asxl3	100759073	0.00	0.00	0.00	0.00	0.00	0.00	0.00	0.00	0.00	0.00	0.00	0.00
ankyrin repeat, SAM and basic leucine zipper domain containing 1	Asz1	100754218	0.00	0.00	0.03	0.03	0.00	0.00	0.00	0.00	1.24	1.16	0.00	0.00
ATPase family, AAA domain containing 1	Atad1	100764591	57.06	59.24	63.24	67.02	74.00	69.66	4574.70	4730.72	5008.09	5288.15	5769.01	5545.40
ATPase family, AAA domain containing 2B	Atad2b	100763563	2.41	2.67	2.30	2.84	3.11	2.68	453.44	496.86	429.76	540.06	587.87	509.39
ATPase family, AAA domain containing 5	Atad5	100771634	2.99	3.03	3.34	4.10	4.77	4.40	507.37	509.67	577.57	698.83	796.94	735.59
alpha tubulin acetyltransferase 1	Atat1	100764669	4.65	4.72	6.78	4.69	5.26	4.46	251.41	248.89	392.50	272.35	291.87	268.25
ataxia, cerebellar, Cayman type	Atcay	100773040	0.00	0.03	0.04	0.08	0.00	0.00	0.00	0.92	1.24	2.32	0.00	0.00
arginyltransferase 1	Ate1	100774144	9.75	9.35	10.36	11.23	11.11	11.54	982.84	973.60	1068.19	1112.56	1113.65	1140.30
activating transcription factor 1	Atf1	100773050	49.18	47.04	44.35	47.60	45.34	47.91	1675.54	1627.84	1548.88	1612.06	1528.67	1641.28
activating transcription factor 2	Atf2	100754663	21.09	21.15	18.38	20.73	22.21	21.65	2462.43	2479.74	2161.23	2380.42	2589.54	2507.72
activating transcription factor 3	Atf3	100763246	27.23	27.20	27.76	23.45	16.23	19.26	1496.29	1500.65	1530.25	1294.51	882.84	1056.18
activating transcription factor 4	Atf4	100689386	587.20	628.62	604.02	576.97	554.62	511.16	19135.07	20059.37	18918.22	17818.39	16761.60	15842.65
activating transcription factor 5	Atf5	100769320	89.86	83.71	48.91	35.87	32.33	32.83	3725.54	3486.28	2039.51	1469.51	1321.68	1348.73
activating transcription factor 6	Atf6	100772173	83.97	83.95	75.93	82.75	73.37	75.16	7500.45	7499.61	6792.97	7282.65	6482.12	6625.87
activating transcription factor 6 beta	Atf6b	100767112	63.20	60.13	63.18	47.96	33.03	37.10	4534.45	4338.17	4558.46	3399.11	2326.65	2627.36
activating transcription factor 7	Atf7	100764874	10.94	10.62	10.08	11.08	9.93	11.59	722.32	714.64	691.84	725.48	676.88	767.36
activating transcription factor 7 interacting protein	Atf7ip	100753637	16.11	15.99	15.54	15.15	15.48	15.38	2537.62	2522.75	2481.69	2392.01	2440.50	2422.66
activating transcription factor 7 interacting protein 2	Atf7ip2	100767143	0.00	0.00	0.00	0.00	0.00	0.00	0.00	0.00	0.00	0.00	0.00	0.00
autophagy related 10	Atg10	100773027	11.49	10.66	9.70	10.56	11.66	11.35	361.54	339.48	314.25	319.86	359.14	372.00
autophagy related 101	Atg101	100763923	50.56	46.16	47.77	39.44	25.90	29.80	1498.57	1389.02	1419.71	1151.97	761.75	885.13
autophagy related 12	Atg12	100760609	19.67	23.55	20.02	22.51	29.79	22.70	236.98	269.02	236.00	266.55	310.50	270.12
autophagy related 13	Atg13	100753698	34.28	32.61	35.28	37.87	33.52	36.63	3644.27	3500.00	3758.55	3992.48	3525.16	3877.94
autophagy related 14	Atg14	100753411	8.63	7.92	8.83	8.83	9.24	9.40	804.35	751.24	839.65	827.47	868.35	871.11
autophagy related 16-like 1 (S. cerevisiae)	Atg16l1	100771530	13.80	13.27	16.41	16.50	15.19	15.09	1108.17	1076.08	1332.76	1318.85	1208.86	1203.85
autophagy related 16-like 2 (S. cerevisiae)	Atg16l2	100751003	6.31	6.25	6.50	7.34	5.47	5.31	481.55	484.97	514.22	531.94	425.38	429.01
autophagy related 2A	Atg2a	100758391	6.35	5.84	6.25	5.38	3.59	3.88	1293.50	1221.57	1306.68	1102.13	759.68	807.55
autophagy related 2B	Atg2b	100760198	6.75	6.56	7.49	8.02	9.01	8.51	1512.24	1497.91	1717.81	1790.53	2013.05	1910.46
autophagy related 3	Atg3	100765208	56.22	56.38	56.33	60.64	62.87	62.70	1867.71	1925.23	1865.61	1993.34	2096.88	1982.43
autophagy related 4A, cysteine peptidase	Atg4a	100750721	10.17	10.41	8.52	9.50	11.88	11.35	661.56	695.43	582.54	625.82	799.01	754.28

autophagy related 4B, cysteine peptidase	Atg4b	100754964	28.51	27.32	26.71	25.46	23.56	26.17	2402.42	2291.24	2300.34	2135.89	1984.07	2195.54
autophagy related 4C, cysteine peptidase	Atg4c	100754372	12.59	12.69	14.98	16.92	16.15	16.27	999.55	1010.20	1194.89	1357.10	1286.49	1300.13
autophagy related 4D, cysteine peptidase	Atg4d	100758833	5.33	4.68	5.19	4.44	3.55	3.72	325.08	283.66	306.80	275.82	245.29	256.10
autophagy related 5	Atg5	100759159	27.96	27.39	29.41	30.48	34.02	33.22	1354.26	1321.31	1414.74	1438.22	1612.51	1567.44
autophagy related 9A	Atg9a	100774022	16.53	16.65	18.32	16.67	14.90	16.04	1892.01	1912.42	2120.24	1911.06	1706.69	1845.04
autophagy related 9B	Atg9b	100769671	3.60	3.35	2.53	2.46	1.95	1.89	315.97	303.79	228.54	221.35	185.26	170.11
5-aminoimidazole-4-carboxamide ribonucleotide formyltransferase/IMP cyclohydrolase	Atic	100750895	188.25	185.15	173.43	167.47	151.94	164.55	14694.05	14624.07	13802.07	13085.36	11883.71	12709.64
atlastin GTPase 1	Atl1	100765875	1.69	1.64	1.65	2.12	2.06	1.86	144.31	142.75	139.11	170.36	168.70	152.35
atlastin GTPase 2	Atl2	100758956	18.59	19.37	17.91	19.96	21.73	20.85	1101.33	1158.43	1094.28	1189.05	1295.80	1235.63
atlastin GTPase 3	Atl3	100752365	13.94	13.50	13.54	15.03	16.20	15.82	2514.07	2456.87	2495.35	2752.43	2943.50	2878.78
ataxia telangiectasia mutated	Atm	100754226	21.45	21.44	27.92	34.71	35.48	33.31	4802.57	4809.42	6159.51	7656.98	7648.55	7146.48
ATM interactor	Atmin	103162505	10.26	10.91	11.66	12.63	11.52	11.92	781.57	843.66	903.00	982.76	885.95	932.80
atrophin 1	Atn1	100767627	26.19	26.38	24.42	18.10	14.34	14.65	4298.23	4307.98	3995.79	3005.08	2401.17	2445.10
atonal homolog 1 (Drosophila)	Atoh1	100769461	0.00	0.00	0.00	0.00	0.00	0.00	0.00	0.00	0.00	0.00	0.00	0.00
atonal homolog 7 (Drosophila)	Atoh7	100763008	0.00	0.00	0.00	0.00	0.00	0.00	0.00	0.00	0.00	0.00	0.00	0.00
atonal homolog 8 (Drosophila)	Atoh8	100751745	0.37	0.53	0.12	0.23	0.00	0.09	4.56	6.41	1.24	3.48	1.03	0.93
antioxidant 1 copper chaperone	Atox1	100760386	28.65	27.41	26.90	27.68	21.68	20.18	301.54	278.17	259.60	261.92	215.28	214.97
ATPase, class V, type 10A	Atp10a	100774607	48.46	46.62	46.51	48.88	56.99	57.50	7299.93	7012.82	7033.94	7346.39	8579.00	8667.19
ATPase, class V, type 10B	Atp10b	100767948	0.00	0.00	0.00	0.00	0.00	0.00	0.00	0.00	0.00	0.00	0.00	0.00
ATPase, class V, type 10D	Atp10d	100755398	5.95	6.31	7.54	7.33	8.18	7.83	893.22	949.80	1136.51	1106.77	1237.84	1190.77
ATPase, class VI, type 11A	Atp11a	100761170	29.10	28.69	37.34	39.05	34.76	36.12	5909.97	5822.36	7739.44	8067.24	7169.35	7453.99
ATPase, class VI, type 11B	Atp11b	100773257	35.39	36.25	34.81	38.75	41.58	38.36	2155.57	2237.26	2150.05	2352.61	2558.49	2338.54
ATPase, class VI, type 11C	Atp11c	100756336	6.54	6.26	7.27	9.06	9.63	8.65	1102.09	1064.18	1249.54	1566.86	1637.35	1466.50
ATPase, H+/K+ transporting, nongastric, alpha polypeptide	Atp12a	100765522	0.15	0.14	0.01	0.04	0.05	0.01	17.47	19.22	2.48	4.64	7.24	0.93
ATPase type 13A1	Atp13a1	100762605	15.91	14.63	15.83	13.81	11.26	11.04	1900.37	1766.93	1902.88	1663.05	1322.71	1314.15
ATPase type 13A2	Atp13a2	100770673	7.98	7.53	8.14	6.17	4.05	4.49	940.31	891.24	978.76	737.07	472.99	518.74
ATPase type 13A3	Atp13a3	100769908	42.38	41.91	37.73	41.89	47.56	45.76	8283.53	8319.48	7472.39	8246.87	9263.13	8972.83
ATPase type 13A4	Atp13a4	100752452	0.01	0.00	0.00	0.00	0.00	0.00	0.76	0.00	0.00	0.00	0.00	0.00
ATPase, Na+/K+ transporting, alpha 1 polypeptide	Atp1a1	100771378	91.99	91.34	83.49	82.11	68.58	73.23	9207.13	9178.70	8411.41	8118.23	6843.33	7258.64
ATPase, Na+/K+ transporting, alpha 2 polypeptide	Atp1a2	100763478	0.00	0.01	0.01	0.00	0.02	0.03	0.76	0.92	2.48	0.00	2.07	2.80
ATPase, Na+/K+ transporting, alpha 3 polypeptide	Atp1a3	100758209	0.00	0.00	0.02	0.00	0.00	0.00	0.00	0.00	1.24	1.16	0.00	0.00
ATPase, Na+/K+ transporting, alpha 4 polypeptide	Atp1a4	100763191	0.00	0.00	0.00	0.00	0.00	0.00	0.00	0.92	0.00	0.00	0.00	0.00
ATPase, Na+/K+ transporting, beta 1 polypeptide	Atp1b1	100760718	0.16	0.17	0.03	0.03	0.03	0.00	9.11	8.24	1.24	1.16	2.07	0.00
ATPase, Na+/K+ transporting, beta 2 polypeptide	Atp1b2	100757379	0.00	0.00	0.06	0.00	0.00	0.00	0.00	0.00	1.24	0.00	0.00	0.00

ATPase, Na+/K+ transporting, beta 3 polypeptide	Atp1b3	100765669	96.69	96.06	57.83	57.47	62.91	59.86	3504.51	3525.62	2123.97	2083.74	2311.12	2160.96
ATPase, Na+/K+ transporting, beta 4 polypeptide	Atp1b4	100762833	0.00	0.00	0.00	0.00	0.00	0.00	0.00	0.00	0.00	0.00	0.00	0.00
ATPase, Ca++ transporting, cardiac muscle, fast twitch 1	Atp2a1	100763714	0.11	0.18	0.13	0.12	0.16	0.15	15.19	21.05	16.15	18.54	23.80	19.63
ATPase, Ca++ transporting, cardiac muscle, slow twitch 2	Atp2a2	100751820	89.57	88.34	94.98	99.54	94.04	98.31	13351.18	13343.93	14318.78	14922.25	14000.26	14532.24
ATPase, Ca++ transporting, ubiquitous	Atp2a3	100756356	0.02	0.00	0.02	0.00	0.00	0.00	1.52	0.00	1.24	0.00	0.00	0.00
ATPase, Ca++ transporting, plasma membrane 1	Atp2b1	100764066	28.46	27.49	26.77	31.06	35.83	33.28	5880.35	5686.02	5579.45	6412.30	7340.12	6869.82
ATPase, Ca++ transporting, plasma membrane 2	Atp2b2	100759670	0.01	0.00	0.00	0.00	0.00	0.01	0.76	0.92	0.00	0.00	0.00	0.93
ATPase, Ca++ transporting, plasma membrane 3	Atp2b3	100753233	0.01	0.00	0.00	0.00	0.00	0.00	0.76	0.00	0.00	0.00	0.00	0.00
ATPase, Ca++ transporting, plasma membrane 4	Atp2b4	100764868	4.71	4.79	7.95	9.12	9.30	9.51	729.92	764.05	1240.84	1391.86	1463.47	1474.91
ATPase, Ca++ transporting, type 2C, member 1	Atp2c1	100765106	14.25	14.93	15.67	17.78	19.80	19.39	2047.72	2149.41	2253.15	2506.74	2809.99	2726.43
ATPase, Ca++ transporting, type 2C, member 2	Atp2c2	100772976	0.05	0.04	0.03	0.03	0.00	0.06	3.80	4.58	3.73	2.32	2.07	5.61
ATPase, H+/K+ exchanging, alpha polypeptide	Atp4a	100767384	0.00	0.00	0.00	0.00	0.00	0.00	0.00	0.00	0.00	0.00	0.00	0.00
ATPase, H+/K+ exchanging, beta polypeptide	Atp4b	100750430	0.00	0.00	0.00	0.00	0.00	0.00	0.00	0.00	0.00	0.00	0.00	0.00
ATP synthase, H+ transporting, mitochondrial F1 complex, alpha subunit 1, cardiac muscle	Atp5a1	100770431	161.99	162.90	159.77	152.28	145.09	157.78	8157.45	8296.61	8090.95	7625.69	7284.23	7897.96
ATP synthase, H+ transporting, mitochondrial F1 complex, beta polypeptide	Atp5b	100763024	456.04	447.96	508.46	504.92	502.11	536.12	22199.81	21962.63	24854.16	24382.52	24268.36	25892.22
ATP synthase, H+ transporting, mitochondrial F1 complex, delta subunit	Atp5d	100766108	107.15	93.44	91.67	58.47	36.71	45.21	2385.71	2080.79	2014.66	1267.86	824.88	990.75
ATP synthase, H+ transporting, mitochondrial F1 complex, epsilon subunit	Atp5e	103162776	293.98	274.55	250.54	231.40	262.97	266.31	3098.92	2915.30	2598.45	2321.32	2749.96	2699.33
ATP synthase, H+ transporting, mitochondrial Fo complex, subunit B1	Atp5f1	100774535	204.36	209.85	194.02	200.59	213.71	209.77	5725.41	5926.67	5416.74	5427.22	5831.11	5695.88
ATP synthase, H+ transporting, mitochondrial Fo complex, subunit C1 (subunit 9)	Atp5g1	100752739	519.55	480.57	329.21	297.03	253.20	308.20	7448.04	6897.52	4619.32	4075.92	3596.58	4274.24
ATP synthase, H+ transporting, mitochondrial Fo complex, subunit C2 (subunit 9)	Atp5g2	100772366	306.83	283.00	295.46	252.37	193.23	226.19	7075.11	6587.33	6850.11	5685.66	4409.04	5043.48
ATP synthase, H+ transporting, mitochondrial Fo complex, subunit C3 (subunit 9)	Atp5g3	100754380	316.55	292.24	284.94	276.33	263.69	307.94	4997.77	4727.98	4392.02	3903.24	3731.13	4253.68
ATP synthase, H+ transporting, mitochondrial Fo complex, subunit d	Atp5h	100771541	261.17	261.97	288.90	301.65	308.80	305.18	3155.13	3169.68	3444.31	3528.91	3720.78	3607.83
ATP synthase, H+ transporting, mitochondrial Fo complex, subunit E	Atp5i	100766302	251.53	257.14	213.79	188.84	122.18	139.99	1308.69	1378.96	1135.27	890.05	633.41	660.81
ATP synthase, H+ transporting, mitochondrial Fo complex, subunit F6	Atp5j	100756936	365.82	375.40	368.61	369.79	392.26	415.11	3966.31	4058.17	3897.67	3791.99	4144.09	4322.85

ATP synthase, H+ transporting, mitochondrial Fo complex, subunit F2	Atp5j2	103160361	563.68	532.73	478.73	504.25	519.06	531.68	5385.13	4995.17	4420.59	4459.52	4756.80	4792.99
ATP synthase, H+ transporting, mitochondrial Fo complex, subunit G	Atp5l	100757815	370.80	386.95	357.75	282.36	226.75	256.34	5316.77	5534.12	5008.09	3757.22	2940.40	3341.44
ATP synthase, H+ transporting, mitochondrial F1 complex, O subunit	Atp5o	100770018	231.19	216.79	230.16	231.27	217.17	237.17	7142.71	6839.88	7253.79	7076.36	6698.43	7124.05
ATP synthase, H+ transporting, mitochondrial Fo complex, subunit s (factor B)	Atp5s	100768179	8.56	7.95	6.35	5.87	7.73	6.43	215.71	199.48	155.26	161.09	190.44	170.11
ATP5S-like	Atp5sl	100774519	3.20	3.24	3.00	2.06	0.97	1.21	162.54	163.79	144.08	141.39	105.57	100.94
ATPase, H+ transporting, lysosomal accessory protein 1	Atp6ap1	100760638	93.61	90.95	103.47	103.69	112.78	118.74	4947.64	4793.86	5483.81	5455.04	5868.37	6229.57
ATPase, H+ transporting, lysosomal accessory protein 1-like	Atp6ap1l	100772450	0.06	0.04	0.00	0.00	0.00	0.08	1.52	0.92	1.24	1.16	0.00	1.87
ATPase, H+ transporting, lysosomal accessory protein 2	Atp6ap2	100768578	72.96	68.56	62.30	66.28	76.90	71.58	3834.15	3626.28	3277.87	3460.53	4022.99	3808.78
ATPase, H+ transporting, lysosomal V0 subunit a1	Atp6v0a1	100756648	10.21	10.31	6.38	6.73	7.81	7.46	1216.78	1231.64	762.64	801.97	942.87	899.15
ATPase, H+ transporting, lysosomal V0 subunit a2	Atp6v0a2	100766909	7.63	6.96	9.44	10.29	10.51	10.60	1241.85	1125.49	1545.16	1651.46	1667.36	1710.45
ATPase, H+ transporting, lysosomal V0 subunit a4	Atp6v0a4	100769769	0.00	0.00	0.00	0.00	0.02	0.00	0.00	0.00	0.00	0.00	1.03	0.00
ATPase, H+ transporting, lysosomal 21kDa, V0 subunit b	Atp6v0b	100754687	65.73	57.75	74.33	74.86	62.20	73.75	1822.13	1651.64	2073.04	2000.30	1659.08	1917.01
ATPase, H+ transporting, lysosomal 16kDa, V0 subunit c	Atp6v0c	100772184	100.38	99.84	104.80	64.50	51.48	52.13	3070.82	3065.36	3218.25	1969.00	1572.14	1614.17
ATPase, H+ transporting, lysosomal 38kDa, V0 subunit d1	Atp6v0d1	100762360	37.57	36.55	46.40	43.59	41.22	46.65	1779.60	1761.44	2246.94	2099.96	1965.44	2205.82
ATPase, H+ transporting, lysosomal 38kDa, V0 subunit d2	Atp6v0d2	100758035	0.05	0.06	0.00	0.00	0.00	0.00	1.52	3.66	0.00	0.00	0.00	0.00
ATPase, H+ transporting, lysosomal 9kDa, V0 subunit e1	Atp6v0e1	100769759	69.53	66.62	73.94	72.70	78.10	77.94	1406.67	1354.25	1468.15	1415.04	1550.41	1559.96
ATPase, H+ transporting V0 subunit e2	Atp6v0e2	100766003	1.66	1.30	1.93	1.91	1.63	1.77	98.74	73.20	111.79	111.26	87.97	95.34
ATPase, H+ transporting, lysosomal 70kDa, V1 subunit A	Atp6v1a	100768315	15.93	16.10	16.87	19.44	21.15	22.41	1609.46	1654.38	1733.95	1957.42	2125.86	2245.08
ATPase, H+ transporting, lysosomal 56/58kDa, V1 subunit B1	Atp6v1b1	100756094	0.00	0.00	0.00	0.00	0.00	0.00	0.00	0.00	0.00	0.00	1.03	0.00
ATPase, H+ transporting, lysosomal 56/58kDa, V1 subunit B2	Atp6v1b2	100774665	34.66	33.08	36.12	37.67	40.56	43.88	2584.71	2473.34	2685.39	2775.61	2983.87	3249.85
ATPase, H+ transporting, lysosomal 42kDa, V1 subunit C1	Atp6v1c1	100773930	26.71	26.98	32.08	35.09	33.18	35.00	1555.54	1598.56	1902.88	2003.77	1921.97	1980.57
ATPase, H+ transporting, lysosomal 42kDa, V1 subunit C2	Atp6v1c2	100763093	12.91	13.37	13.93	14.41	18.06	17.23	464.84	488.63	485.66	487.91	610.64	622.49
ATPase, H+ transporting, lysosomal 34kDa, V1 subunit D	Atp6v1d	100762500	80.08	76.68	84.36	85.46	87.60	88.60	2994.10	2879.61	3173.53	3141.83	3214.67	3306.86
ATPase, H+ transporting, lysosomal 31kDa, V1 subunit E1	Atp6v1e1	100755187	99.38	99.79	104.79	96.89	87.88	98.36	3416.41	3480.79	3618.20	3276.27	3023.20	3330.23
ATPase, H+ transporting, lysosomal 31kDa, V1 subunit E2	Atp6v1e2	100765179	0.92	0.88	0.85	0.69	0.75	0.62	71.40	65.88	67.07	59.10	55.89	53.28
ATPase, H+ transporting, lysosomal 14kDa, V1 subunit F	Atp6v1f	100752884	84.97	84.65	104.81	81.99	65.69	65.71	1287.42	1289.28	1592.36	1222.66	998.76	991.68
ATPase, H+ transporting, lysosomal 13kDa, V1 subunit G1	Atp6v1g1	100774299	183.39	171.53	177.06	154.16	157.02	164.79	4989.41	4652.03	4779.55	4119.96	4169.96	4351.82

ATPase, H+ transporting, lysosomal 13kDa, V1 subunit G2	Atp6v1g2	100765359	9.00	8.64	8.94	9.48	9.63	10.58	380.53	366.01	391.26	394.03	415.03	466.40
ATPase, H+ transporting, lysosomal 13kDa, V1 subunit G3	Atp6v1g3	100763970	0.00	0.00	0.00	0.00	0.00	0.00	0.00	0.00	1.24	0.00	0.00	0.93
ATPase, H+ transporting, lysosomal 50/57kDa, V1 subunit H	Atp6v1h	100754134	16.17	16.53	16.91	18.86	20.49	20.58	830.18	849.15	854.56	949.16	1027.74	1036.55
ATPase, Cu++ transporting, alpha polypeptide	Atp7a	100689242	3.79	4.03	4.31	4.50	5.05	5.04	878.79	937.91	1006.09	1038.39	1164.36	1164.60
ATPase, Cu++ transporting, beta polypeptide	Atp7b	100770719	3.02	3.02	3.15	3.21	3.75	3.43	575.73	588.37	616.08	620.02	725.53	672.03
ATPase, aminophospholipid transporter (APLT), class I, type 8A, member 1	Atp8a1	100756975	10.90	11.17	9.55	10.98	13.75	12.41	817.26	760.39	786.24	895.85	1029.81	930.00
ATPase, aminophospholipid transporter, class I, type 8A, member 2	Atp8a2	100763581	0.01	0.02	0.00	0.00	0.00	0.00	6.84	8.24	1.24	0.00	1.03	0.00
ATPase, aminophospholipid transporter, class I, type 8B, member 1	Atp8b1	100763292	0.01	0.00	0.01	0.00	0.00	0.00	0.76	0.00	1.24	0.00	0.00	0.93
ATPase, aminophospholipid transporter, class I, type 8B, member 2	Atp8b2	100751265	32.75	30.32	29.67	29.68	22.15	24.73	5323.61	5033.60	4943.51	4940.48	3636.94	4021.88
ATPase, aminophospholipid transporter, class I, type 8B, member 3	Atp8b3	100757984	0.05	0.08	0.03	0.02	0.04	0.02	13.67	12.81	4.97	3.48	5.17	3.74
ATPase, class I, type 8B, member 4	Atp8b4	100759968	0.01	0.01	0.01	0.00	0.00	0.00	0.76	1.83	1.24	0.00	0.00	0.00
ATPase, class II, type 9B	Atp9b	100765138	4.31	4.14	4.70	4.66	4.88	4.41	587.12	566.41	658.31	654.79	688.27	633.71
ATP synthase mitochondrial F1 complex assembly factor 1	Atpaf1	100770122	22.30	24.15	24.12	27.20	27.46	26.42	1275.27	1387.19	1413.49	1562.22	1591.81	1528.18
ATP synthase mitochondrial F1 complex assembly factor 2	Atpaf2	100754752	17.48	16.44	15.87	16.58	14.76	15.15	694.22	683.53	674.45	699.99	635.48	636.51
ATPase inhibitory factor 1	Atpif1	100771468	105.78	103.91	102.84	89.05	93.37	89.85	1417.30	1404.58	1361.33	1135.74	1200.58	1151.51
ataxia telangiectasia and Rad3 related	Atr	100766256	4.35	4.58	2.35	2.89	3.24	3.02	1093.74	1160.26	621.04	727.80	842.48	778.58
all-trans retinoic acid-induced differentiation factor	Atraid	100772507	40.79	39.32	31.75	35.78	34.80	36.60	1002.59	980.00	786.24	868.03	855.93	889.81
ATR interacting protein	Atrip	100762140	5.06	5.19	5.99	5.73	5.65	6.51	412.43	420.92	512.98	483.27	476.09	529.02
attractin	Atrn	100774269	6.86	6.61	7.13	7.77	7.75	8.34	1345.14	1313.07	1447.03	1562.22	1548.34	1651.56
alpha thalassemia/mental retardation syndrome X-linked	Atrx	100767383	10.46	10.49	10.93	13.29	15.13	14.13	3165.76	3165.10	3371.02	4057.38	4564.29	4277.05
ataxin 1	Atxn1	100765598	0.52	0.54	0.40	0.30	0.24	0.27	47.85	50.33	36.02	28.97	25.87	24.30
ataxin 10	Atxn10	100756095	63.15	61.18	64.08	65.51	74.66	77.08	4062.02	4043.53	4170.93	4184.86	4800.27	4901.41
ataxin 1-like	Atxn1l	100755753	8.74	8.50	8.05	8.77	7.90	8.45	1566.93	1536.34	1463.18	1576.13	1412.76	1550.62
ataxin 2	Atxn2	100772811	19.09	18.78	17.76	18.42	16.22	16.93	2249.00	2214.38	2140.12	2162.54	1941.63	2066.55
ataxin 2-like	Atxn2l	100765372	87.59	87.01	84.62	76.14	73.70	75.78	9691.72	9552.04	9356.64	8305.98	7986.99	8290.52
ataxin 3	Atxn3	100754332	17.21	18.03	18.07	20.49	21.94	22.41	1181.84	1164.84	1280.59	1459.08	1523.50	1464.63
ataxin 7	Atxn7	100753336	3.23	2.74	2.49	3.15	2.81	2.69	293.18	244.31	231.03	297.84	268.06	260.77
ataxin 7-like 1	Atxn7l1	100758796	0.51	0.66	0.35	0.55	0.82	0.67	118.49	124.44	119.24	141.39	156.28	171.98
ataxin 7-like 2	Atxn7l2	100773539	5.47	4.28	4.83	3.70	2.84	3.07	499.78	389.80	460.81	360.42	285.66	294.42
ataxin 7-like 3	Atxn7l3	100754412	25.26	24.25	17.84	18.34	18.10	19.53	1339.83	1356.08	982.49	1055.78	1116.75	1173.95

ataxin 7-like 3B	Atxn7l3b	100759327	49.95	51.33	63.36	55.27	59.49	62.90	4790.41	4928.37	6123.49	5314.81	5672.76	6066.94
AU RNA binding protein/enoyl-CoA hydratase	Auh	100774025	6.77	6.38	7.63	8.27	6.90	8.77	277.99	268.10	310.52	334.93	290.83	354.24
aurora kinase A and ninein interacting protein	Aunip	100763527	11.83	12.32	15.52	17.34	17.70	17.99	1101.33	1157.52	1490.50	1636.39	1665.29	1698.29
ancient ubiquitous protein 1	Aup1	100760920	66.57	63.87	73.98	68.20	65.20	67.83	2874.10	2774.38	3205.83	2884.55	2645.43	2779.71
aurora kinase A	Aurka	100761359	63.56	64.22	54.23	53.47	50.59	54.60	3427.80	3493.60	2956.17	2883.39	2726.15	2943.27
aurora kinase A interacting protein 1	Aurkaip1	100770569	238.01	226.30	236.19	242.24	203.90	217.64	5034.99	4754.51	4947.23	4617.14	3731.13	3967.67
aurora kinase B	Aurkb	100753283	10.52	9.97	10.57	11.61	10.00	11.09	545.35	503.27	529.13	587.57	512.32	567.34
aurora kinase C	Aurkc	100762186	32.59	32.47	39.25	39.15	39.26	42.14	574.21	561.83	695.57	649.00	661.36	706.61
autism susceptibility candidate 2	Auts2	100750545	0.00	0.00	0.00	0.00	0.00	0.00	0.00	0.00	0.00	0.00	0.00	0.00
apoptosis, caspase activation inhibitor	Aven	100751273	51.81	51.31	62.00	61.21	58.97	61.20	1164.37	1146.54	1378.72	1320.01	1310.29	1321.62
advillin	Avil	100761585	0.03	0.01	0.02	0.03	0.03	0.02	4.56	2.75	2.48	3.48	8.28	5.61
AVL9 homolog (S. cerevisiase)	Avl9	100768061	14.12	13.23	12.96	14.73	13.49	12.81	2853.59	2686.54	2653.10	2994.65	2716.84	2611.47
arginine vasopressin-induced 1	Avpi1	100759679	20.89	22.36	17.24	13.33	11.14	10.06	632.70	674.38	525.40	398.67	331.20	306.57
arginine vasopressin receptor 1A	Avpr1a	100756428	0.00	0.00	0.00	0.00	0.00	0.00	0.00	0.00	0.00	0.00	0.00	0.00
arginine vasopressin receptor 1B	Avpr1b	100757693	0.00	0.00	0.00	0.00	0.00	0.00	0.00	0.00	0.00	0.00	0.00	0.00
arginine vasopressin receptor 2	Avpr2	100768840	0.00	0.00	0.00	0.00	0.00	0.00	0.00	0.92	0.00	0.00	0.00	0.00
acyl-CoA wax alcohol acyltransferase 1	Awat1	100769131	0.00	0.00	0.00	0.00	0.00	0.00	0.00	0.00	0.00	0.00	0.00	0.00
acyl-CoA wax alcohol acyltransferase 2	Awat2	100768365	0.00	0.00	0.00	0.00	0.00	0.00	0.00	0.00	0.00	0.00	0.00	0.00
axin 1	Axin1	100758169	32.47	30.72	35.26	34.45	28.25	32.42	3090.57	2956.47	3415.74	3301.76	2695.10	3071.32
axin 2	Axin2	100750382	1.06	1.25	0.30	0.46	0.67	0.59	132.16	157.39	36.02	59.10	82.80	75.71
AXL receptor tyrosine kinase	Axl	100772486	125.30	123.66	135.63	132.60	128.13	132.61	14078.82	13961.58	15231.71	14705.53	14114.11	14664.97
alpha-2-glycoprotein 1, zinc-binding	Azgp1	100773168	0.00	0.00	0.00	0.00	0.00	0.00	0.00	0.00	2.48	0.00	0.00	0.00
5-azacytidine induced 2	Azi2	100750738	36.19	39.22	39.96	42.93	44.80	43.50	2818.65	3040.66	3123.85	3308.72	3440.29	3352.66
antizyme inhibitor 1	Azin1	100774224	55.47	56.00	57.98	64.78	60.56	60.63	6276.83	6369.55	6601.69	7303.51	6782.27	6842.72
beta-2-microglobulin	B2m	100689409	234.17	250.00	259.01	272.40	328.77	330.39	4384.06	4724.32	4891.34	5118.95	6233.72	6203.40
beta-1,3-N-acetylgalactosaminyltransferase 1 (globoside blood group)	B3galnt1	100756438	14.71	14.92	18.70	18.17	20.44	18.87	1170.45	1187.71	1471.87	1430.11	1586.63	1486.12
beta-1,3-N-acetylgalactosaminyltransferase 2	B3galnt2	100768564	13.08	13.20	14.27	14.93	16.28	16.16	1104.37	1140.13	1261.96	1257.43	1319.61	1366.49
UDP-Gal:betaGlcNAc beta 1,3-galactosyltransferase, polypeptide 1	B3galt1	100761765	0.65	0.50	0.43	0.52	0.64	0.65	65.32	46.67	42.23	48.67	53.82	60.75
UDP-Gal:betaGlcNAc beta 1,3-galactosyltransferase, polypeptide 2	B3galt2	100766715	0.00	0.00	0.00	0.00	0.00	0.00	0.00	0.00	0.00	0.00	0.00	0.00
UDP-Gal:betaGlcNAc beta 1,3-galactosyltransferase, polypeptide 4	B3galt4	100751197	2.67	2.49	2.53	2.01	0.92	1.31	125.32	118.04	124.21	96.19	49.68	60.75
UDP-Gal:betaGlcNAc beta 1,3-galactosyltransferase, polypeptide 5	B3galt5	100755714	0.00	0.00	0.00	0.00	0.00	0.00	0.00	0.00	0.00	0.00	0.00	0.00
UDP-Gal:betaGal beta 1,3-galactosyltransferase polypeptide 6	B3galt6	100767598	13.69	13.89	15.95	15.92	15.77	16.46	1281.34	1281.96	1484.29	1498.48	1468.65	1544.07
beta 1,3-galactosyltransferase-like	B3galtl	100759864	16.56	16.97	17.11	19.59	21.41	20.01	1374.01	1417.39	1470.63	1622.49	1763.62	1639.41
beta-1,3-glucuronyltransferase 1 (glucuronosyltransferase P)	B3gat1	100750701	0.00	0.00	0.00	0.00	0.00	0.00	0.00	0.00	0.00	0.00	0.00	0.00

beta-1,3-glucuronyltransferase 2 (glucuronosyltransferase S)	B3gat2	100756696	0.08	0.00	0.00	0.00	0.00	0.00	2.28	0.92	0.00	0.00	0.00	1.87
beta-1,3-glucuronyltransferase 3 (glucuronosyltransferase I)	B3gat3	100689419	18.82	16.64	17.26	11.51	7.30	7.24	686.62	593.86	626.01	424.16	288.76	283.20
UDP-GlcNAc:betaGal beta-1,3-N-acetylglucosaminyltransferase 1	B3gnt1	100762253	19.94	20.81	26.11	20.52	21.08	20.65	985.12	1016.60	1300.46	1015.21	1029.81	1025.33
UDP-GlcNAc:betaGal beta-1,3-N-acetylglucosaminyltransferase 2	B3gnt2	100766691	22.80	26.05	25.18	26.33	30.55	29.61	1680.86	1918.83	1874.31	1942.35	2236.61	2187.13
UDP-GlcNAc:betaGal beta-1,3-N-acetylglucosaminyltransferase 3	B3gnt3	100773213	0.07	0.09	0.03	0.03	0.00	0.02	4.56	4.58	1.24	1.16	1.03	0.93
UDP-GlcNAc:betaGal beta-1,3-N-acetylglucosaminyltransferase 4	B3gnt4	100769077	0.25	0.16	0.25	0.35	0.21	0.17	9.87	6.41	8.69	13.91	7.24	5.61
UDP-GlcNAc:betaGal beta-1,3-N-acetylglucosaminyltransferase 5	B3gnt5	100757919	0.00	0.00	0.00	0.00	0.00	0.00	0.00	0.00	0.00	0.00	0.00	0.00
UDP-GlcNAc:betaGal beta-1,3-N-acetylglucosaminyltransferase 6 (core 3 synthase)	B3gnt6	100768656	0.00	0.00	0.00	0.00	0.00	0.00	0.00	0.00	0.00	0.00	0.00	0.00
UDP-GlcNAc:betaGal beta-1,3-N-acetylglucosaminyltransferase 7	B3gnt7	100760456	0.00	0.03	0.00	0.03	0.00	0.00	0.00	0.92	0.00	1.16	0.00	0.00
UDP-GlcNAc:betaGal beta-1,3-N-acetylglucosaminyltransferase 8	B3gnt8	103160327	0.00	0.04	0.00	0.00	0.00	0.00	0.00	0.92	0.00	0.00	0.00	0.00
UDP-GlcNAc:betaGal beta-1,3-N-acetylglucosaminyltransferase 9	B3gnt9	103159649	3.59	3.68	4.55	4.61	3.66	4.41	185.33	189.41	245.93	227.15	180.09	230.86
UDP-GlcNAc:betaGal beta-1,3-N-acetylglucosaminyltransferase-like 1	B3gntl1	100751193	16.33	17.20	6.00	4.68	3.87	4.47	684.34	713.73	234.75	200.49	165.60	186.00
beta-1,4-N-acetyl-galactosaminyl transferase 1	B4galnt 1	100764682	0.00	0.00	0.03	0.00	0.00	0.00	0.00	0.00	1.24	0.00	0.00	0.00
beta-1,4-N-acetyl-galactosaminyl transferase 2	B4galnt 2	100760696	0.00	0.00	0.00	0.00	0.00	0.00	0.76	0.00	0.00	1.16	0.00	0.00
beta-1,4-N-acetyl-galactosaminyl transferase 3	B4galnt 3	100756528	0.00	0.00	0.00	0.00	0.00	0.00	0.00	0.00	0.00	1.16	0.00	0.00
beta-1,4-N-acetyl-galactosaminyl transferase 4	B4galnt 4	100758404	0.02	0.02	0.19	0.07	0.09	0.31	9.87	4.58	27.33	12.75	13.45	39.26
UDP-Gal:betaGlcNAc beta 1,4-galactosyltransferase, polypeptide 1	B4galt1	100689430	9.24	8.99	11.57	12.13	13.30	12.95	238.50	237.91	296.86	308.27	348.79	366.39
UDP-Gal:betaGlcNAc beta 1,4-galactosyltransferase, polypeptide 2	B4galt2	100689434	32.13	32.96	35.51	32.05	23.88	26.04	1597.31	1678.17	1864.37	1689.71	1209.90	1340.32
UDP-Gal:betaGlcNAc beta 1,4-galactosyltransferase, polypeptide 3	B4galt3	100689346	19.29	18.96	23.01	20.05	16.67	18.69	944.11	950.72	1147.69	1002.47	861.11	962.71
UDP-Gal:betaGlcNAc beta 1,4-galactosyltransferase, polypeptide 4	B4galt4	100689435	16.12	17.40	15.74	17.03	18.13	18.25	981.32	1020.26	977.52	1017.53	1103.30	1074.87
UDP-Gal:betaGlcNAc beta 1,4-galactosyltransferase, polypeptide 5	B4galt5	100689347	8.52	8.17	6.18	5.92	8.01	7.52	334.20	338.56	255.87	252.64	315.67	279.47
UDP-Gal:betaGlcNAc beta 1,4-galactosyltransferase, polypeptide 6	B4galt6	100689438	5.58	5.30	5.31	4.48	6.50	5.41	198.24	206.80	190.04	190.06	239.08	196.28
xylosylprotein beta 1,4-galactosyltransferase, polypeptide 7	B4galt7	100769652	42.78	42.62	46.61	37.52	23.93	27.56	2074.30	2066.15	2261.84	1833.41	1199.55	1356.21
B9 protein domain 1	B9d1	100774531	5.66	6.47	6.87	6.58	5.25	6.83	139.76	155.56	157.75	172.68	141.79	156.09
B9 protein domain 2	B9d2	103160326	8.19	8.32	8.15	7.07	3.89	5.65	108.61	107.97	99.37	93.87	57.96	74.77
brain and acute leukemia, cytoplasmic	Baalc	100774798	0.00	0.00	0.00	0.00	0.00	0.00	0.00	0.00	0.00	0.00	0.00	0.00
BRISC and BRCA1 A complex member 1	Babam1	100755943	55.31	53.99	64.18	49.82	33.91	40.32	2215.58	2164.97	2624.53	1994.50	1421.04	1641.28
beta-site APP-cleaving enzyme 1	Bace1	100762079	20.57	20.88	25.20	25.94	26.59	28.93	2319.63	2376.34	2858.04	2965.68	3021.13	3292.84

beta-site APP-cleaving enzyme 2	Bace2	100764950	0.00	0.03	0.00	0.00	0.00	0.00	0.00	0.92	0.00	0.00	0.00	0.00
BTB and CNC homology 1, basic leucine zipper transcription factor 1	Bach1	100770017	6.65	7.10	7.85	8.61	8.00	8.00	1100.57	1187.71	1329.03	1441.70	1336.17	1350.60
BTB and CNC homology 1, basic leucine zipper transcription factor 2	Bach2	100774050	0.04	0.00	0.00	0.00	0.00	0.00	3.80	0.00	0.00	0.00	0.00	0.00
BCL2-associated agonist of cell death	Bad	100760426	7.89	6.78	7.02	4.89	3.82	3.59	388.88	346.80	417.34	276.98	215.28	214.97
BCL2-associated athanogene	Bag1	100759550	21.77	20.79	28.20	30.24	34.67	34.34	467.88	465.75	577.57	606.12	712.07	714.09
BCL2-associated athanogene 2	Bag2	100750795	17.90	18.42	24.18	28.45	23.62	22.15	443.57	454.77	583.78	667.54	555.79	558.00
BCL2-associated athanogene 3	Bag3	100758034	28.07	28.87	24.85	22.20	18.64	18.12	1978.60	2032.29	1762.52	1577.29	1298.91	1278.63
BCL2-associated athanogene 4	Bag4	100753192	15.12	14.53	17.56	17.75	18.74	20.10	1092.22	1064.18	1299.22	1293.35	1380.67	1483.32
BCL2-associated athanogene 5	Bag5	100757492	38.12	40.88	39.27	43.57	44.12	43.62	1888.97	2025.88	1958.77	2150.96	2165.19	2148.81
BCL2-associated athanogene 6	Bag6	100763893	82.98	78.54	74.87	56.22	39.60	44.63	9651.46	9030.47	8586.55	6233.83	4369.71	4928.51
BAH domain and coiled-coil containing 1	Bahcc1	100754288	7.75	7.77	3.12	2.32	1.61	1.28	2020.37	2023.14	816.05	620.02	450.22	370.13
bromo adjacent homology domain containing 1	Bahd1	100753540	10.19	10.07	9.37	7.49	5.89	6.10	979.05	971.77	956.41	718.53	586.84	604.73
brain-specific angiogenesis inhibitor 1	Bai1	100766156	0.00	0.01	0.00	0.00	0.00	0.00	0.00	0.92	0.00	0.00	0.00	0.00
brain-specific angiogenesis inhibitor 2	Bai2	100774076	0.37	0.35	0.00	0.00	0.00	0.01	52.41	54.90	0.00	0.00	0.00	0.93
brain-specific angiogenesis inhibitor 3	Bai3	100753086	0.00	0.00	0.00	0.00	0.00	0.00	0.00	0.00	0.00	0.00	0.00	0.00
BAI1-associated protein 2	Baiap2	100761815	26.65	26.92	18.97	14.77	11.07	11.98	2856.63	2906.15	2046.96	1692.02	1229.56	1307.60
BAI1-associated protein 2-like 1	Baiap2l 1	100763126	12.95	13.79	15.32	17.66	13.81	13.37	1143.87	1238.96	1356.36	1563.38	1217.14	1176.75
BAI1-associated protein 2-like 2	Baiap2l 2	100757056	0.00	0.00	0.00	0.12	0.00	0.00	0.00	0.00	4.97	5.79	0.00	0.00
BAI1-associated protein 3	Baiap3	100751355	0.04	0.02	0.04	0.04	0.03	0.03	4.56	3.66	6.21	6.95	5.17	3.74
BCL2-antagonist/killer 1	Bak1	100689368	49.92	43.99	50.83	47.21	36.17	35.39	2767.76	2752.42	2911.45	2562.37	1868.15	1945.98
BMP and activin membrane-bound inhibitor	Bambi	100771599	3.91	3.69	3.04	3.20	3.55	3.68	120.01	107.97	93.16	98.51	101.43	114.96
barrier to autointegration factor 1	Banf1	100765074	563.10	528.64	561.95	549.15	527.26	597.18	8463.55	7969.94	8505.81	8183.13	7937.31	8935.44
barrier to autointegration factor 2	Banf2	100768587	0.00	0.00	0.00	0.00	0.00	0.00	0.00	0.00	0.00	0.00	0.00	0.00
B-cell scaffold protein with ankyrin repeats 1	Bank1	100762463	0.51	0.54	0.48	0.51	0.28	0.13	66.84	70.46	63.35	62.58	31.05	18.69
BTG3 associated nuclear protein	Banp	100768192	21.74	20.17	18.93	17.93	15.44	16.23	1476.54	1411.90	1319.10	1231.93	1073.28	1100.11
BRCA1 associated protein-1 (ubiquitin carboxy-terminal hydrolase)	Bap1	100765297	44.24	42.79	42.91	42.62	38.70	41.79	4486.60	4401.31	4408.17	4340.15	3929.84	4263.96
BRCA1 associated RING domain 1	Bard1	100750589	6.07	5.97	5.98	6.04	7.09	7.34	615.99	610.33	616.08	628.13	729.67	759.89
BarH-like homeobox 1	Barhl1	100774052	0.00	0.00	0.00	0.00	0.00	0.00	0.00	0.92	0.00	0.00	0.00	0.00
BarH-like homeobox 2	Barhl2	100762455	0.00	0.00	0.00	0.00	0.00	0.00	0.00	0.00	0.00	0.00	0.00	0.00
BARX homeobox 1	Barx1	100763836	0.00	0.00	0.00	0.00	0.00	0.00	0.00	0.00	0.00	0.00	0.00	0.00
BARX homeobox 2	Barx2	100765416	0.00	0.00	0.00	0.00	0.00	0.00	0.00	0.00	0.00	0.00	0.00	0.00
basic leucine zipper transcription factor, ATF-like	Batf	100755965	12.71	12.25	10.00	6.64	5.09	6.28	289.38	280.00	229.79	155.30	125.23	148.61
basic leucine zipper transcription factor, ATF-like 2	Batf2	100759752	2.65	2.76	3.02	2.15	1.31	1.56	168.62	188.50	185.07	135.59	96.25	101.88
basic leucine zipper transcription factor, ATF-like 3	Batf3	100765560	0.00	0.00	0.00	0.24	0.00	0.00	0.76	0.00	0.00	1.16	0.00	0.00
BCL2-associated X protein	Bax	100689032	49.04	46.98	46.22	44.47	33.44	34.12	932.71	904.97	875.67	807.77	582.70	617.82
bromodomain adjacent to zinc finger domain, 1A	Baz1a	100757354	20.57	19.52	18.76	22.46	19.75	18.92	3011.57	2875.04	2799.66	3263.52	2917.63	2795.60

bromodomain adjacent to zinc finger domain, 1B	Baz1b	100760864	58.12	58.83	69.50	70.08	69.86	72.60	9881.61	10054.39	11941.42	11935.72	11844.38	12349.79
bromodomain adjacent to zinc finger domain, 2A	Baz2a	100763314	12.58	12.53	11.70	11.92	11.88	12.19	3123.98	3120.26	2946.23	2949.45	2945.57	3054.50
bromodomain adjacent to zinc finger domain, 2B	Baz2b	100770047	4.04	3.89	3.53	4.17	4.32	3.78	1040.57	1010.20	939.02	1080.11	1115.72	991.68
BCL2 binding component 3	Bbc3	103164214	19.90	19.01	13.53	6.50	3.36	3.60	143.55	127.19	95.64	40.56	19.66	23.37
BBSome interacting protein 1	Bbip1	100767213	23.45	25.19	20.17	23.16	27.64	23.95	683.59	699.09	582.54	640.88	741.05	678.57
butyrobetaine (gamma), 2-oxoglutarate dioxygenase (gamma-butyrobetaine hydroxylase) 1	Bbox1	100759730	0.00	0.00	0.00	0.00	0.00	0.00	0.00	0.00	0.00	0.00	0.00	0.00
Bardet-Biedl syndrome 1	Bbs1	100767595	12.66	11.97	14.63	16.19	15.36	17.71	704.85	679.87	828.47	920.18	883.88	972.06
Bardet-Biedl syndrome 10	Bbs10	100763026	5.87	5.36	5.90	5.93	7.99	7.41	451.93	415.43	459.57	461.25	605.47	563.61
Bardet-Biedl syndrome 12	Bbs12	100755190	4.33	4.99	4.36	4.71	5.10	5.38	406.35	463.92	407.40	434.59	464.71	483.22
Bardet-Biedl syndrome 2	Bbs2	100756643	0.02	0.04	0.02	0.00	0.03	0.01	2.28	4.58	1.24	1.16	2.07	2.80
Bardet-Biedl syndrome 4	Bbs4	100755264	21.37	23.18	21.07	23.21	25.57	23.98	2131.27	2301.31	2133.91	2280.75	2535.72	2370.32
Bardet-Biedl syndrome 5	Bbs5	100762765	0.00	0.00	0.00	0.00	0.00	0.00	0.00	0.00	0.00	0.00	0.00	0.00
Bardet-Biedl syndrome 7	Bbs7	100758793	9.53	9.68	10.39	12.85	14.91	13.42	761.06	787.84	824.75	1014.05	1213.00	1085.15
Bardet-Biedl syndrome 9	Bbs9	100768362	7.84	6.98	6.76	8.00	8.38	8.71	737.51	686.28	655.82	723.17	808.32	795.40
bobby sox homolog (Drosophila)	Bbx	100769386	24.18	23.87	28.61	33.71	35.42	33.44	2245.20	2265.62	2701.54	3222.96	3290.22	3115.25
basal cell adhesion molecule (Lutheran blood group)	Bcam	100751659	0.00	0.00	0.00	0.00	0.00	0.00	1.52	0.00	0.00	0.00	0.00	0.00
brevican	Bcan	100753507	0.00	0.00	0.02	0.00	0.00	0.00	0.00	0.00	1.24	0.00	0.00	0.00
B-cell receptor-associated protein 29	Bcap29	100754548	91.51	96.96	83.35	91.30	90.50	82.39	2193.55	2278.43	2027.09	2132.41	2175.54	2005.80
B-cell receptor-associated protein 31	Bcap31	100770274	61.54	63.34	62.74	66.27	68.60	75.19	2050.76	2109.15	2140.12	2237.87	2330.79	2508.65
breast cancer anti-estrogen resistance 1	Bcar1	100768861	31.15	29.97	28.14	21.60	16.74	17.92	2815.61	2713.07	2558.70	1971.32	1524.53	1641.28
breast cancer anti-estrogen resistance 3	Bcar3	100758812	38.75	40.82	46.44	47.91	60.54	55.78	1184.12	1207.84	1398.59	1489.21	1780.18	1741.29
breast carcinoma amplified sequence 1	Bcas1	100756615	0.09	0.00	0.00	0.00	0.00	0.00	2.28	0.00	0.00	0.00	0.00	0.00
breast carcinoma amplified sequence 2	Bcas2	100758667	61.16	62.26	60.82	62.81	64.25	62.81	1730.23	1776.08	1686.75	1739.54	1773.97	1729.14
breast carcinoma amplified sequence 3	Bcas3	100761557	5.56	5.62	6.81	7.34	7.60	7.43	619.78	622.22	755.19	789.22	859.04	829.05
branched chain amino-acid transaminase 1, cytosolic	Bcat1	100763658	52.90	54.56	58.14	60.08	55.35	58.83	5924.41	6065.76	6539.59	6720.58	6150.92	6647.37
branched chain amino-acid transaminase 2, mitochondrial	Bcat2	100752344	1.13	0.97	0.69	0.97	0.45	0.57	58.48	50.33	40.99	53.31	23.80	30.84
BRCA2 and CDKN1A interacting protein	Bccip	100757180	69.24	66.96	67.21	71.43	70.83	68.33	2258.87	2180.52	2228.30	2265.69	2216.94	2165.63
BCDIN3 domain containing	Bcdin3d	100757267	17.05	18.75	17.35	15.12	13.47	13.69	583.33	642.35	603.65	508.77	472.99	484.16
butyrylcholinesterase	Bche	100767789	0.00	0.00	0.00	0.00	0.00	0.00	0.00	0.00	0.00	0.00	0.00	0.00
branched chain keto acid dehydrogenase E1, alpha polypeptide	Bckdha	100774234	21.14	27.00	27.98	22.14	16.38	14.86	301.54	345.88	353.99	248.01	183.19	174.78
branched chain keto acid dehydrogenase E1, beta polypeptide	Bckdhb	100774277	26.65	23.91	14.02	16.13	16.00	15.30	985.88	940.65	561.42	599.16	596.15	566.41

branched chain ketoacid dehydrogenase kinase	Bckdk	100755998	39.72	38.55	48.10	44.28	40.22	39.62	2130.51	2077.13	2536.34	2339.86	2125.86	2138.52
B-cell CLL/lymphoma 10	Bcl10	100750466	47.54	46.04	44.36	46.60	48.48	46.22	2279.38	2207.98	2128.94	2226.29	2337.00	2254.42
B-cell CLL/lymphoma 11A (zinc finger protein)	Bcl11a	100760174	0.00	0.00	0.00	0.00	0.00	0.00	0.00	0.00	0.00	0.00	0.00	0.00
B-cell CLL/lymphoma 11B (zinc finger protein)	Bcl11b	100768427	0.00	0.00	0.00	0.00	0.00	0.00	0.00	0.00	0.00	0.00	0.00	0.00
B-cell CLL/lymphoma 2	Bcl2	100774873	2.62	3.14	3.60	3.78	3.76	4.01	141.27	164.71	188.80	193.54	190.44	209.37
BCL2-like 1	Bcl2l1	100689371	25.10	24.27	34.33	31.20	27.19	28.22	1576.80	1615.03	2245.69	2157.91	1915.76	1991.78
BCL2-like 10 (apoptosis facilitator)	Bcl2l10	100752860	0.00	0.00	0.00	0.00	0.00	0.00	0.00	0.00	0.00	0.00	0.00	0.00
BCL2-like 11 (apoptosis facilitator)	Bcl2l11	100771754	5.37	5.31	7.06	6.34	5.99	6.42	805.11	803.40	1064.47	959.59	893.19	965.51
BCL2-like 12 (proline rich)	Bcl2l12	100765966	25.46	23.74	30.25	22.00	15.68	17.48	873.47	813.46	1065.71	748.66	537.16	598.19
BCL2-like 14 (apoptosis facilitator)	Bcl2l14	100774179	0.00	0.00	0.00	0.00	0.00	0.00	0.00	0.00	0.00	0.00	0.00	0.00
BCL2-like 15	Bcl2l15	100760311	0.00	0.00	0.00	0.00	0.11	0.00	0.76	0.00	0.00	0.00	1.03	0.00
BCL2-like 2	Bcl2l2	103159537	27.24	26.83	26.66	27.94	26.21	26.85	2945.49	2906.15	2892.82	2996.97	2774.80	2879.72
B-cell CLL/lymphoma 3	Bcl3	100751077	0.22	0.18	0.08	0.00	0.00	0.00	7.60	5.49	1.24	0.00	0.00	0.00
B-cell CLL/lymphoma 6	Bcl6	100773755	3.70	3.84	4.05	4.10	3.30	3.53	350.91	360.52	388.77	395.19	323.95	344.89
B-cell CLL/lymphoma 6, member B	Bcl6b	100764025	0.14	0.06	0.03	0.19	0.10	0.20	6.08	3.66	1.24	9.27	6.21	8.41
B-cell CLL/lymphoma 7A	Bcl7a	100753895	6.92	5.83	5.00	6.35	5.62	4.65	239.25	262.61	219.85	259.60	229.77	187.87
B-cell CLL/lymphoma 7B	Bcl7b	100760574	21.03	21.89	19.99	18.42	11.02	14.46	784.60	792.42	740.28	674.49	413.99	554.26
B-cell CLL/lymphoma 7C	Bcl7c	100753452	44.01	44.25	41.41	28.93	19.15	17.04	986.64	1002.88	957.65	646.68	444.01	401.91
B-cell CLL/lymphoma 9	Bcl9	100751238	10.20	9.97	9.48	8.74	6.81	7.19	1820.62	1785.23	1692.96	1557.59	1210.93	1301.99
B-cell CLL/lymphoma 9-like	Bcl9l	100770128	13.64	13.31	13.75	10.76	8.69	8.76	2656.11	2578.56	2656.82	2068.67	1652.87	1703.90
BCL2-associated transcription factor 1	Bclaf1	100773081	28.00	29.16	28.18	33.04	35.37	31.54	2827.00	2900.66	2901.51	3401.43	3653.50	3271.34
beta-carotene 15,15'-monooxygenase 1	Bcmo1	100760350	0.00	0.05	0.00	0.02	0.03	0.06	1.52	4.58	1.24	1.16	3.10	6.54
beta-carotene oxygenase 2	Bco2	100774947	4.32	3.72	4.64	5.53	5.43	5.78	303.06	268.10	324.18	389.40	377.77	416.86
BCL6 corepressor	Bcor	100767711	4.78	4.90	4.85	4.60	4.02	4.00	770.93	785.10	792.45	741.71	644.80	652.40
BCL6 corepressor-like 1	Bcorl1	100773975	3.41	2.83	3.05	2.54	2.85	2.90	548.39	468.50	499.32	411.42	458.50	464.53
breakpoint cluster region	Bcr	100768252	17.33	16.66	19.55	19.01	17.02	16.92	2653.83	2545.62	3017.03	2894.98	2591.61	2591.84
3-hydroxybutyrate dehydrogenase, type 1	Bdh1	100774340	16.48	17.41	21.40	19.64	17.61	18.13	796.76	832.68	1018.51	925.98	852.83	866.44
bradykinin receptor B1	Bdkrb1	100759905	0.03	0.07	0.05	0.00	0.00	0.07	2.28	1.83	1.24	0.00	2.07	1.87
bradykinin receptor B2	Bdkrb2	100759615	0.05	0.09	0.00	0.00	0.00	0.00	1.52	2.75	0.00	0.00	0.00	0.00
brain-derived neurotrophic factor	Bdnf	100768664	16.18	16.90	20.64	21.73	23.03	21.48	1023.10	1076.08	1284.32	1382.59	1467.61	1357.14
B double prime 1, subunit of RNA polymerase III transcription initiation factor IIIB	Bdp1	100759229	10.62	9.81	9.30	11.61	11.91	10.86	3224.24	2977.52	2810.84	3505.73	3577.95	3324.62
brain expressed, associated with NEDD4, 1	Bean1	100768989	1.42	1.35	0.02	0.08	0.07	0.06	109.37	104.31	3.73	9.27	6.21	5.61
beclin 1, autophagy related	Becn1	100762045	64.81	65.74	56.46	59.49	69.84	65.02	3818.96	3890.72	3257.99	3384.05	3770.46	3605.96
brain-enriched guanylate kinase-associated	Begain	100766701	0.00	0.00	0.00	0.00	0.00	0.00	1.52	0.00	0.00	0.00	0.00	0.00
BEN domain containing 3	Bend3	100768308	8.38	7.60	7.87	7.22	5.96	6.09	1367.93	1256.34	1295.50	1187.89	963.57	1005.70
BEN domain containing 4	Bend4	100766260	12.52	12.84	13.02	14.30	13.86	13.41	2539.90	2614.25	2669.24	2920.48	2785.15	2731.10
BEN domain containing 5	Bend5	103162627	0.00	0.00	0.00	0.00	0.00	0.00	0.00	0.00	0.00	0.00	0.00	0.00
BEN domain containing 6	Bend6	100774404	8.25	8.08	5.73	8.51	9.92	8.67	533.96	533.46	365.17	557.44	638.59	567.34
BEN domain containing 7	Bend7	100754282	0.00	0.03	0.00	0.00	0.00	0.00	0.00	0.92	0.00	0.00	0.00	0.00

bestrophin 1	Best1	100758040	0.19	0.23	0.11	0.25	0.21	0.20	8.35	15.56	9.94	15.07	14.49	11.22
bestrophin 2	Best2	100762224	0.04	0.02	0.09	0.00	0.00	0.03	3.80	1.83	6.21	1.16	0.00	3.74
bestrophin 3	Best3	100759541	0.01	0.05	0.00	0.00	0.00	0.00	0.76	2.75	0.00	0.00	0.00	0.00
bestrophin 4	Best4	100754760	0.00	0.00	0.03	0.00	0.06	0.00	3.80	4.58	4.97	1.16	4.14	0.00
Bet1 golgi vesicular membrane trafficking protein	Bet1	100761331	46.36	54.53	50.52	45.68	45.37	43.84	824.86	939.74	920.39	805.45	905.61	899.15
Bet1 golgi vesicular membrane trafficking protein-like	Bet1l	100770224	16.86	15.97	17.58	17.56	17.65	17.98	1468.19	1374.38	1510.38	1504.28	1496.59	1538.47
brain expressed, X-linked 4	Bex4	100765842	0.00	0.00	0.00	0.00	0.00	0.00	0.00	0.00	0.00	0.00	0.00	0.00
bifunctional apoptosis regulator	Bfar	100772795	14.83	14.95	17.54	18.06	19.80	18.14	1032.97	1012.03	1206.07	1250.47	1320.64	1247.78
beaded filament structural protein 1, filensin	Bfsp1	100772902	0.51	0.45	0.21	0.12	0.09	0.32	29.62	28.37	11.18	8.11	9.31	20.56
beaded filament structural protein 2, phakinin	Bfsp2	100767821	0.00	0.00	0.00	0.00	0.00	0.00	0.00	0.00	0.00	0.00	0.00	0.00
bone gamma-carboxyglutamate (gla) protein	Bglap	100763202	0.17	0.53	0.44	0.84	0.49	0.89	11.39	9.15	7.45	16.22	8.28	10.28
biglycan	Bgn	100771022	0.02	0.00	0.00	0.00	0.00	0.00	0.76	0.00	0.00	0.00	0.00	0.00
basic helix-loop-helix family, member a15	Bhlha15	100768574	0.00	0.08	0.00	0.00	0.00	0.00	0.00	0.92	0.00	0.00	0.00	0.00
basic helix-loop-helix family, member a9	Bhlha9	100753830	0.00	0.00	0.00	0.00	0.00	0.00	0.00	0.00	0.00	0.00	0.00	0.00
basic helix-loop-helix domain containing, class B, 9	Bhlhb9	100767015	15.62	16.34	17.45	17.83	20.77	20.63	1263.87	1292.94	1403.56	1441.70	1621.82	1644.08
basic helix-loop-helix family, member e40	Bhlhe40	100765325	3.68	3.93	1.43	1.86	1.60	1.71	291.66	309.28	115.51	143.71	125.23	132.72
basic helix-loop-helix family, member e41	Bhlhe41	100759250	11.53	12.85	9.91	10.49	12.68	12.14	239.25	272.68	214.88	201.65	243.22	249.56
betaine--homocysteine S-methyltransferase	Bhmt	100754488	0.00	0.00	0.00	0.00	0.00	0.00	0.00	0.00	0.00	0.00	0.00	0.00
betaine--homocysteine S-methyltransferase 2	Bhmt2	100754773	0.00	0.00	0.00	0.00	0.00	0.00	0.00	0.00	0.00	0.00	0.00	0.00
BicC family RNA binding protein 1	Bicc1	100771763	0.00	0.00	0.00	0.00	0.00	0.00	0.00	0.00	0.00	0.00	0.00	0.00
bicaudal D homolog 1 (Drosophila)	Bicd1	100762966	0.74	0.76	0.63	0.74	0.86	1.05	40.26	42.09	33.54	41.72	45.54	60.75
bicaudal D homolog 2 (Drosophila)	Bicd2	100758573	12.67	13.63	9.78	9.71	8.47	9.46	1599.59	1743.14	1254.51	1215.71	1053.62	1186.10
BH3 interacting domain death agonist	Bid	100754606	27.27	28.23	49.83	45.88	56.45	56.80	1704.41	1790.72	3141.24	2850.94	3480.66	3511.55
BCL2-interacting killer (apoptosis-inducing)	Bik	100768268	0.20	0.21	0.11	0.12	0.22	0.12	9.87	9.15	3.73	11.59	8.28	6.54
bridging integrator 1	Bin1	100772852	12.66	11.27	15.42	14.37	11.18	12.44	865.87	764.05	1008.57	917.86	722.42	784.19
bridging integrator 2	Bin2	100750538	0.02	0.00	0.00	0.00	0.00	0.00	0.76	0.92	0.00	0.00	0.00	0.93
bridging integrator 3	Bin3	100769924	21.46	19.85	23.06	22.75	19.21	21.32	943.35	886.67	1023.48	992.04	850.76	948.69
baculoviral IAP repeat containing 2	Birc2	100756596	6.88	6.80	7.59	8.89	9.44	9.25	670.67	654.25	736.56	854.12	921.14	893.54
baculoviral IAP repeat containing 3	Birc3	100761700	6.85	7.29	8.90	10.32	11.52	10.90	615.23	669.80	803.63	941.04	1045.34	999.16
baculoviral IAP repeat containing 5	Birc5	100689366	153.25	150.57	156.31	158.19	144.13	152.96	2759.41	2730.46	2762.40	2798.79	2538.82	2696.52
baculoviral IAP repeat containing 6	Birc6	100689237	8.95	8.52	7.77	9.65	10.31	9.97	4223.80	4066.41	3751.10	4590.48	4872.72	4676.15
baculoviral IAP repeat containing 7	Birc7	100758467	0.00	0.05	0.00	0.00	0.05	0.00	0.00	0.92	0.00	0.00	1.03	0.00
bladder cancer associated protein	Blcap	100757663	58.73	57.53	57.78	55.62	54.61	56.21	3171.84	3115.69	3137.51	2988.85	2912.45	3033.94
B lymphoid tyrosine kinase	Blk	100755534	0.00	0.00	0.00	0.00	0.00	0.00	0.00	0.92	0.00	0.00	0.00	0.00
Bloom syndrome, RecQ helicase-like	Blm	100762862	29.56	28.88	33.73	41.95	44.46	40.35	1953.53	1903.27	2219.61	2750.12	2955.92	2654.46
bleomycin hydrolase	Blmh	100754419	89.59	87.97	83.44	83.33	82.15	86.07	5865.16	5769.29	5447.79	5376.23	5303.27	5576.24
B-cell linker	Blnk	100755442	7.59	8.10	9.39	9.09	8.94	9.75	478.51	516.08	618.56	594.53	553.72	614.08

biogenesis of lysosomal organelles complex-1, subunit 1	Bloc1s1	100772750	82.13	74.99	78.33	63.39	35.85	41.64	991.20	942.48	980.01	754.46	431.59	495.37
biogenesis of lysosomal organelles complex-1, subunit 2	Bloc1s2	100754144	86.60	86.21	87.37	89.01	93.94	99.37	1787.96	1798.04	1812.20	1807.92	1895.06	1995.52
biogenesis of lysosomal organelles complex-1, subunit 4, cappuccino	Bloc1s4	100769537	17.79	17.95	15.63	15.50	17.03	17.35	584.09	605.75	539.07	538.90	612.71	627.16
biogenesis of lysosomal organelles complex-1, subunit 5, muted	Bloc1s5	100766796	20.02	19.91	19.61	21.41	23.86	23.14	953.98	953.46	941.50	1008.26	1118.82	1095.43
biogenesis of lysosomal organelles complex-1, subunit 6, pallidin	Bloc1s6	100771201	20.97	21.42	20.64	22.71	23.76	24.20	916.00	959.87	960.13	1059.25	1125.03	1133.75
biliverdin reductase A	Blvra	100751522	27.54	28.94	27.71	29.57	33.74	32.74	750.42	811.63	777.55	810.08	902.51	872.05
biliverdin reductase B (flavin reductase (NADPH))	Blvrb	100768929	147.71	142.50	187.09	177.79	153.02	168.23	1127.16	1090.72	1371.26	1157.76	951.15	1044.03
basic leucine zipper nuclear factor 1	Blzf1	100761007	16.93	17.56	14.78	16.29	19.73	17.67	1363.37	1400.00	1192.40	1307.26	1555.58	1430.98
Bcl2 modifying factor	Bmf	100761166	1.53	1.79	1.76	1.83	1.15	1.31	145.83	116.21	119.24	129.80	111.78	110.29
BMI1 polycomb ring finger oncogene	Bmi1	100754672	33.85	37.35	28.50	28.58	27.94	27.58	1127.92	1216.08	951.44	938.73	939.77	930.00
bone morphogenetic protein 1	Bmp1	100751120	21.51	20.01	18.75	16.77	12.61	14.26	2255.07	2122.88	1992.31	1748.81	1328.92	1512.30
bone morphogenetic protein 10	Bmp10	100766801	0.00	0.00	0.00	0.00	0.00	0.00	0.00	0.00	0.00	0.00	0.00	0.00
bone morphogenetic protein 15	Bmp15	100756159	0.05	0.00	0.04	0.00	0.04	0.06	1.52	0.00	1.24	0.00	2.07	1.87
bone morphogenetic protein 2	Bmp2	100752564	0.00	0.00	0.00	0.00	0.00	0.00	0.00	0.00	0.00	0.00	0.00	0.00
BMP2 inducible kinase	Bmp2k	100769791	14.79	14.33	14.39	15.05	16.04	15.48	3181.71	3092.81	3086.59	3200.94	3386.48	3300.32
bone morphogenetic protein 3	Bmp3	100766849	0.00	0.00	0.00	0.00	0.00	0.00	0.00	0.00	0.00	0.00	0.00	0.00
bone morphogenetic protein 4	Bmp4	100754303	0.00	0.00	0.00	0.00	0.00	0.00	0.00	0.00	0.00	0.00	0.00	0.00
bone morphogenetic protein 5	Bmp5	100763439	0.02	0.00	0.00	0.00	0.00	0.00	2.28	0.00	0.00	0.00	0.00	0.00
bone morphogenetic protein 6	Bmp6	100768517	0.79	0.65	0.07	0.13	0.16	0.22	42.53	34.77	3.73	6.95	8.28	11.22
bone morphogenetic protein 7	Bmp7	100759897	0.00	0.00	0.00	0.00	0.00	0.00	0.00	0.00	0.00	0.00	0.00	0.00
BMP binding endothelial regulator	Bmper	100766307	0.13	0.08	0.03	0.03	0.06	0.08	10.63	5.49	3.73	2.32	5.17	5.61
bone morphogenetic protein receptor, type IA	Bmpr1a	100750332	16.51	17.06	19.41	20.20	20.98	20.19	2603.70	2696.60	3077.89	3189.35	3295.40	3179.75
bone morphogenetic protein receptor, type II (serine/threonine kinase)	Bmpr2	100764519	8.75	8.42	8.41	9.64	10.35	10.83	1364.13	1332.29	1338.97	1508.91	1607.33	1694.56
BMS1 ribosome biogenesis factor	Bms1	100770546	82.53	81.74	85.35	94.92	91.09	85.64	10439.11	10343.54	10860.81	11893.99	11411.76	10766.46
BMX non-receptor tyrosine kinase	Bmx	100751568	0.00	0.00	0.00	0.00	0.00	0.00	0.00	0.00	0.00	0.00	0.00	0.00
basonuclin 1	Bnc1	100768562	0.01	0.00	0.00	0.00	0.01	0.02	1.52	0.00	0.00	0.00	1.03	1.87
basonuclin 2	Bnc2	100754985	5.11	5.05	5.88	7.24	7.13	6.72	862.08	853.73	991.19	1135.74	1180.92	1081.41
BCL2/adenovirus E1B 19kDa interacting protein 1	Bnip1	100770337	56.11	52.23	58.65	61.06	53.65	56.05	1761.37	1660.79	1842.01	1871.66	1660.12	1734.75
BCL2/adenovirus E1B 19kDa interacting protein 2	Bnip2	100750620	31.25	30.56	32.13	34.98	36.09	35.80	3507.55	3473.47	3631.86	3948.44	4089.23	4036.84
BCL2/adenovirus E1B 19kDa interacting protein 3	Bnip3	100774634	17.34	17.38	16.65	18.51	20.67	20.95	720.04	735.69	715.44	756.77	848.69	855.22
BCL2/adenovirus E1B 19kDa interacting protein 3-like	Bnip3l	100759633	38.84	38.68	38.10	39.78	53.30	51.71	3502.24	3519.22	3467.91	3581.06	4725.75	4619.14
BCL2/adenovirus E1B 19kD interacting protein like	Bnipl	100767161	0.22	0.25	0.20	0.22	0.38	0.27	26.58	29.28	23.60	25.50	41.40	34.58
BOC cell adhesion associated, oncogene regulated	Boc	100766664	6.87	6.66	8.06	7.59	5.96	6.30	856.00	824.45	1013.54	959.59	740.02	771.10
biorientation of chromosomes in cell division 1	Bod1	100750386	56.72	58.14	69.13	70.37	68.81	68.72	1673.26	1737.65	2066.83	2043.18	2019.26	2009.54
biorientation of chromosomes in cell division 1-like 1	Bod1l1	100769598	14.44	14.05	13.89	16.95	17.60	16.57	4609.64	4500.13	4488.90	5430.70	5588.93	5302.38

BCL2-related ovarian killer	Bok	100773011	0.00	0.00	0.00	0.00	0.00	0.00	0.00	1.83	1.24	0.00	0.00	0.93
bolA family member 1	Bola1	100758926	9.31	8.59	7.74	3.15	3.54	2.41	131.40	109.80	103.09	48.67	50.71	42.06
bolA family member 2B	Bola2b	100770374	26.04	26.74	26.56	17.20	13.36	15.39	570.41	580.13	555.21	354.63	287.73	350.50
bolA family member 3	Bola3	100769793	38.04	35.76	37.32	41.87	41.55	44.44	524.08	507.84	524.16	540.06	542.33	579.50
boule-like RNA-binding protein	Boll	100772853	0.00	0.00	0.00	0.00	0.00	0.00	0.00	0.00	0.00	0.00	1.03	0.00
block of proliferation 1	Bop1	100763888	58.89	56.49	59.66	49.37	36.69	42.01	4352.16	4221.96	4385.81	3621.62	2700.28	3096.56
bora, aurora kinase A activator	Bora	100774852	11.70	11.77	11.81	12.67	13.98	13.39	951.70	938.82	955.16	1037.23	1135.38	1091.69
2,3-bisphosphoglycerate mutase	Bpgm	100757548	10.22	10.00	9.10	10.92	9.67	10.64	444.33	441.96	399.95	482.11	425.38	471.07
biphenyl hydrolase-like (serine hydrolase)	Bphl	100761605	16.84	15.37	13.68	14.18	18.32	16.46	571.17	513.33	455.85	465.89	607.54	541.17
bactericidal/permeability-increasing protein	Bpi	100759574	0.00	0.00	0.00	0.03	0.00	0.00	0.00	0.00	0.00	2.32	0.00	0.00
BPI fold containing family A, member 1	Bpifa1	100753755	0.00	0.00	0.00	0.00	0.00	0.00	0.00	0.00	0.00	0.00	0.00	0.00
BPI fold containing family A, member 2	Bpifa2	100759193	0.00	0.00	0.00	0.00	0.00	0.00	0.00	0.00	0.00	0.00	0.00	0.00
BPI fold containing family A, member 3	Bpifa3	100754343	0.00	0.00	0.00	0.00	0.00	0.00	0.00	0.00	0.00	0.00	0.00	0.00
BPI fold containing family B, member 1	Bpifb1	100753690	0.06	0.02	0.00	0.00	0.00	0.02	4.56	2.75	0.00	0.00	0.00	0.93
BPI fold containing family B, member 2	Bpifb2	100759900	0.00	0.00	0.00	0.00	0.00	0.00	0.00	0.00	0.00	0.00	0.00	0.00
BPI fold containing family B, member 3	Bpifb3	100756247	0.00	0.00	0.00	0.00	0.00	0.00	0.00	0.00	0.00	0.00	0.00	0.00
BPI fold containing family B, member 4	Bpifb4	100754016	0.00	0.00	0.00	0.00	0.00	0.00	0.00	0.00	0.00	0.00	0.00	0.00
BPI fold containing family B, member 6	Bpifb6	100758462	0.00	0.00	0.00	0.00	0.00	0.00	0.00	0.00	0.00	0.00	0.00	0.00
BPI fold containing family C	Bpifc	100752136	0.00	0.02	0.00	0.03	0.00	0.02	3.80	2.75	1.24	3.48	2.07	2.80
3'(2'), 5'-bisphosphate nucleotidase 1	Bpnt1	100774047	7.90	8.91	11.58	11.75	9.68	10.46	487.62	551.77	710.47	709.26	588.91	636.51
bromodomain PHD finger transcription factor	Bptf	100772838	11.99	12.22	11.20	12.15	11.96	11.67	3373.87	3445.10	3228.18	3408.38	3358.53	3252.65
v-raf murine sarcoma viral oncogene homolog B	Braf	100771486	10.89	10.74	9.70	10.53	11.59	10.83	1336.03	1402.75	1219.73	1301.47	1335.13	1301.06
BRCA1 associated protein	Brap	100763856	23.50	22.83	28.80	27.88	26.86	30.33	2217.85	2143.92	2685.39	2621.48	2378.40	2729.24
BRCA1-associated ATM activator 1	Brat1	100761522	5.32	4.53	5.30	4.51	3.77	4.08	566.62	501.44	565.15	489.06	427.45	446.77
breast cancer 1, early onset	Brca1	100770724	46.08	44.42	12.21	16.01	18.23	17.54	8577.48	8452.16	2425.80	3030.58	3451.68	3311.53
breast cancer 2, early onset	Brca2	100689197	5.89	5.58	5.59	7.21	8.44	7.86	1835.81	1779.74	1767.49	2254.10	2634.04	2430.14
BRCA1/BRCA2-containing complex, subunit 3	Brcc3	100756268	12.10	11.43	10.74	11.77	13.46	12.86	909.93	837.26	799.90	897.00	995.66	927.19
bromodomain containing 1	Brd1	100770868	10.67	10.72	9.80	9.46	8.66	8.75	1513.76	1517.13	1408.53	1333.92	1239.91	1273.02
bromodomain containing 2	Brd2	100767103	153.64	152.84	141.92	136.58	104.02	108.71	13733.99	13831.65	12916.46	12428.26	9456.67	9956.10
bromodomain containing 3	Brd3	100768189	20.52	19.94	22.91	20.59	19.54	21.05	3147.53	3080.92	3551.13	3154.58	2997.32	3215.26
bromodomain containing 4	Brd4	100771403	25.62	25.24	22.97	22.30	19.32	19.02	3518.95	3524.71	3207.07	3098.95	2664.05	2598.38
bromodomain containing 7	Brd7	100758832	73.20	72.80	72.77	77.19	70.89	72.84	4577.74	4587.98	4516.23	4690.15	4311.75	4400.42
bromodomain containing 8	Brd8	100774001	6.33	6.65	6.25	7.46	7.58	7.70	762.58	786.93	762.64	849.49	863.18	902.89
bromodomain containing 9	Brd9	100761427	13.29	13.51	14.21	17.28	16.46	15.65	939.55	964.45	1006.09	1204.12	1165.39	1118.80
bromodomain, testis-specific	Brdt	100768519	0.71	0.74	0.68	0.66	0.74	0.58	74.43	88.76	72.04	74.17	80.73	81.32
brain and reproductive organ-expressed (TNFRSF1A modulator)	Bre	100756206	36.59	37.84	35.91	37.81	33.71	36.64	1368.69	1443.92	1334.00	1366.37	1233.70	1284.24

BRF1, RNA polymerase III transcription initiation factor 90 kDa subunit	Brf1	100753600	26.83	26.74	29.73	28.77	21.87	24.34	1804.67	1810.85	2055.65	1948.14	1497.63	1596.42
BRF2, RNA polymerase III transcription initiation factor 50 kDa subunit	Brf2	100769943	9.13	8.05	8.95	8.76	7.70	8.76	508.89	441.05	488.14	478.63	431.59	474.81
brain protein I3	Bri3	100764284	93.01	93.16	132.42	129.33	125.19	131.77	735.23	727.45	988.70	941.04	918.03	977.66
BRI3 binding protein	Bri3bp	100752052	31.23	33.19	38.07	33.71	27.09	28.49	1131.71	1191.37	1367.54	1242.36	1045.34	1101.04
BRICHOS domain containing 5	Bricd5	100757307	2.62	2.79	3.78	4.01	3.20	3.34	281.03	297.39	388.77	398.67	321.88	339.29
bone morphogenetic protein/retinoic acid inducible neural-specific 1	Brinp1	100774741	0.00	0.00	0.00	0.00	0.00	0.00	0.00	0.00	0.00	0.00	0.00	0.00
bone morphogenetic protein/retinoic acid inducible neural-specific 2	Brinp2	100753022	0.00	0.00	0.00	0.00	0.00	0.00	0.00	0.00	0.00	0.00	0.00	0.00
bone morphogenetic protein/retinoic acid inducible neural-specific 3	Brinp3	100774253	0.13	0.21	0.11	0.10	0.12	0.09	11.39	15.56	7.45	6.95	6.21	8.41
BRCA1 interacting protein C-terminal helicase 1	Brip1	100774617	5.21	5.50	2.68	3.46	4.12	3.69	878.03	928.76	465.78	589.89	690.34	628.10
BRX1, biogenesis of ribosomes, homolog (S. cerevisiae)	Brix1	100757120	47.57	48.10	41.10	46.45	48.05	43.30	1726.43	1758.69	1494.23	1639.87	1746.02	1542.20
BRICK1, SCAR/WAVE actin-nucleating complex subunit	Brk1	100758338	87.08	88.86	97.78	95.08	93.98	99.20	3057.15	3131.24	3424.43	3312.19	3261.24	3478.84
breast cancer metastasis suppressor 1	Brms1	100762549	27.87	29.02	31.56	28.24	23.95	27.60	1073.23	1130.07	1214.76	1026.80	906.65	1030.01
breast cancer metastasis-suppressor 1-like	Brms1l	100774936	13.75	14.76	14.10	15.07	15.00	13.13	560.54	565.49	558.94	631.61	669.64	587.91
BRO1 domain and CAAX motif containing	Brox	100772686	37.92	38.65	38.02	41.62	49.92	46.64	3815.93	3928.24	3901.39	4183.70	4978.28	4712.60
bromodomain and PHD finger containing, 1	Brpf1	100754861	10.26	10.47	10.12	9.45	8.17	9.17	1440.85	1452.16	1420.95	1307.26	1163.32	1289.84
bromodomain and PHD finger containing, 3	Brpf3	100759740	16.16	15.91	14.69	12.46	9.55	10.28	2672.82	2647.19	2456.85	2068.67	1568.00	1698.29
bombesin-like receptor 3	Brs3	100763382	0.00	0.00	0.00	0.00	0.00	0.00	0.00	0.00	0.00	0.00	0.00	0.00
BR serine/threonine kinase 1	Brsk1	100772369	0.01	0.02	0.00	0.02	0.00	0.00	0.76	0.92	0.00	2.32	1.03	0.00
BR serine/threonine kinase 2	Brsk2	100769140	0.00	0.00	0.00	0.00	0.00	0.00	0.00	0.00	0.00	0.00	0.00	0.00
bromodomain and WD repeat domain containing 1	Brwd1	100750709	4.22	4.07	4.25	4.52	4.81	4.75	965.37	925.10	981.25	1067.37	1112.61	1108.52
bromodomain and WD repeat domain containing 3	Brwd3	100751005	9.17	8.73	8.02	9.36	10.22	9.31	874.23	831.77	780.03	903.96	965.64	887.00
Berardinelli-Seip congenital lipodystrophy 2 (seipin)	Bscl2	100755147	4.72	4.25	6.25	6.98	5.35	5.05	317.49	294.64	428.52	449.66	357.07	355.17
BSD domain containing 1	Bsdc1	100752123	26.07	25.44	25.54	25.47	23.32	24.91	1822.13	1782.49	1803.51	1763.88	1614.58	1744.09
basigin (Ok blood group)	Bsg	100689246	296.22	290.93	301.87	272.94	224.59	243.10	10516.58	10306.94	10652.14	9470.69	7828.64	8508.30
bassoon presynaptic cytomatrix protein	Bsn	100770905	0.01	0.01	0.00	0.01	0.00	0.00	3.04	1.83	2.48	3.48	2.07	0.00
Bartter syndrome, infantile, with sensorineural deafness (Barttin)	Bsnd	100750920	0.00	0.00	0.00	0.00	0.00	0.00	0.00	0.00	0.00	0.00	0.00	0.00
B-box and SPRY domain containing	Bspry	100756148	0.02	0.01	0.02	0.00	0.02	0.01	1.52	0.92	1.24	0.00	1.03	0.93
bone marrow stromal cell antigen 1	Bst1	100773468	0.00	0.00	0.00	0.00	0.00	0.00	0.00	0.00	0.00	0.00	0.00	0.00
bone marrow stromal cell antigen 2	Bst2	100689092	162.82	156.98	160.81	141.35	121.07	140.21	5780.09	5519.48	5670.13	4934.68	4263.11	4931.32
brain-specific homeobox	Bsx	100762865	0.04	0.00	0.00	0.00	0.05	0.05	3.80	6.41	2.48	1.16	2.07	3.74
BTAF1 RNA polymerase II, B-TFIID transcription factor-associated, 170kDa	Btaf1	100769949	13.93	13.51	13.56	15.94	15.29	14.06	2643.20	2557.52	2586.02	2976.11	2882.44	2666.61

BTB (POZ) domain containing 1	Btbd1	100770759	58.69	59.63	61.34	68.74	67.18	64.53	4536.73	4653.86	4782.03	5335.67	5157.34	4953.75
BTB (POZ) domain containing 10	Btbd10	100751295	16.48	16.81	14.99	16.20	17.99	17.32	1114.24	1184.05	1075.65	1151.97	1299.94	1244.05
BTB (POZ) domain containing 11	Btbd11	100761949	0.00	0.00	0.00	0.00	0.00	0.00	0.00	0.00	0.00	0.00	0.00	0.00
BTB (POZ) domain containing 16	Btbd16	100762672	0.51	0.54	0.06	0.21	0.28	0.13	14.43	15.56	2.48	5.79	6.21	3.74
BTB (POZ) domain containing 17	Btbd17	100761410	0.00	0.00	0.00	0.00	0.00	0.00	0.00	0.00	0.00	0.00	0.00	0.00
BTB (POZ) domain containing 18	Btbd18	100754420	0.15	0.28	0.16	0.20	0.19	0.29	15.19	24.71	14.91	22.02	19.66	26.17
BTB (POZ) domain containing 19	Btbd19	100754188	1.10	1.48	0.83	1.11	1.53	1.49	70.64	86.93	48.44	59.10	81.76	82.25
BTB (POZ) domain containing 2	Btbd2	100759139	13.60	14.26	18.44	14.22	9.72	9.50	872.71	918.69	1176.26	924.82	620.99	607.54
BTB (POZ) domain containing 3	Btbd3	100768764	18.15	17.72	18.91	19.98	19.91	19.36	2337.10	2276.60	2459.33	2584.39	2541.93	2496.50
BTB (POZ) domain containing 6	Btbd6	103161198	25.08	24.01	26.15	26.74	21.89	23.81	1304.13	1249.02	1358.84	1371.00	1131.24	1224.42
BTB (POZ) domain containing 7	Btbd7	100765835	12.59	13.15	12.71	13.41	15.13	14.72	2283.93	2334.25	2280.47	2485.88	2698.21	2650.72
	Btbd9	100756253	15.45	12.69	16.69	15.66	15.84	13.32	539.27	471.24	553.97	531.94	509.21	472.94
betacellulin	Btc	100763277	0.00	0.00	0.00	0.00	0.00	0.00	0.00	0.00	0.00	0.00	0.00	0.00
biotinidase	Btd	100755324	5.17	4.97	6.55	6.65	7.30	6.77	299.26	279.09	361.45	375.49	406.75	389.76
basic transcription factor 3	Btf3	100756079	639.13	642.74	632.50	628.78	619.04	635.39	14384.91	14677.14	14377.16	13894.29	13767.39	13958.36
basic transcription factor 3-like 4	Btf3l4	100753985	76.32	75.35	67.83	68.96	74.90	72.99	1950.50	1960.92	1755.07	1748.81	1923.00	1832.89
B-cell translocation gene 1, anti-proliferative	Btg1	100755272	41.03	40.36	49.23	53.05	53.10	50.30	1427.93	1409.15	1727.74	1842.68	1821.58	1745.96
BTG family, member 2	Btg2	100766229	0.64	1.32	0.38	0.00	0.08	0.22	10.63	21.96	6.21	3.48	3.10	3.74
BTG family, member 3	Btg3	100753614	45.91	42.79	32.46	37.05	38.80	37.70	1497.81	1386.28	1058.26	1186.73	1249.23	1194.51
B-cell translocation gene 4	Btg4	100770985	0.04	0.10	0.00	0.00	0.12	0.00	0.76	3.66	1.24	0.00	2.07	0.00
Bruton agammaglobulinemia tyrosine kinase	Btk	100772144	0.02	0.00	0.00	0.00	0.00	0.00	0.76	0.00	0.00	0.00	0.00	0.00
B and T lymphocyte associated	Btla	100771853	0.00	0.00	0.00	0.00	0.00	0.00	0.00	0.00	0.00	0.00	0.00	0.00
butyrophilin, subfamily 1, member A1	Btn1a1	100760393	0.00	0.02	0.02	0.02	0.00	0.00	0.76	2.75	1.24	3.48	1.03	0.93
butyrophilin-like 10	Btnl10	100762433	0.00	0.02	0.11	0.05	0.09	0.02	1.52	3.66	6.21	6.95	4.14	3.74
butyrophilin-like 2 (MHC class II associated)	Btnl2	100767924	0.00	0.00	0.00	0.00	0.00	0.00	0.00	0.00	0.00	0.00	0.00	0.00
butyrophilin-like 9	Btnl9	100770259	0.00	0.00	0.00	0.00	0.00	0.00	0.00	0.00	0.00	0.00	0.00	0.00
beta-transducin repeat containing E3 ubiquitin protein ligase	Btrc	100750426	10.21	10.60	11.64	10.61	12.16	12.32	651.68	681.70	746.49	667.54	751.40	767.36
BUB1 mitotic checkpoint serine/threonine kinase	Bub1	100772514	14.61	15.70	13.27	16.50	18.13	17.41	1483.38	1590.33	1343.94	1665.37	1840.21	1758.11
BUB1 mitotic checkpoint serine/threonine kinase B	Bub1b	100754771	43.78	46.98	44.16	53.11	57.74	55.61	2207.22	2350.72	2225.82	2621.48	2858.63	2783.45
BUB3 mitotic checkpoint protein	Bub3	100767122	76.34	75.67	69.91	74.36	69.23	73.20	5811.23	5772.03	5345.94	5624.24	5219.44	5549.13
BUD13 homolog (S. cerevisiae)	Bud13	100773647	26.48	26.28	28.72	28.90	30.17	28.90	2182.92	2183.27	2377.35	2378.10	2482.93	2412.38
BUD31 homolog (S. cerevisiae)	Bud31	100774223	119.97	113.36	123.97	128.38	139.29	133.91	2811.05	2721.31	2972.31	2983.06	3233.30	3056.37
blood vessel epicardial substance	Bves	100770468	0.00	0.00	0.00	0.00	0.00	0.00	0.76	0.00	0.00	0.00	0.00	0.00
bystin-like	Bysl	100765734	6.90	7.05	6.44	4.78	3.65	3.71	316.73	285.49	285.68	250.33	179.05	216.84
benzodiazepine receptor (peripheral) associated protein 1	Bzrap1	100752521	5.39	5.32	2.19	1.88	1.61	1.78	1149.94	1169.41	504.29	443.87	382.94	413.12
basic leucine zipper and W2 domains 1	Bzw1	100758868	100.94	100.23	89.44	95.37	105.49	97.07	8301.76	8337.79	7432.65	7860.95	8631.79	8041.90
basic leucine zipper and W2 domains 2	Bzw2	100759248	151.58	147.92	144.15	150.58	140.75	146.75	7417.66	7272.69	7071.20	7182.99	6737.76	7052.08
C1D nuclear receptor corepressor	C1d	100689353	41.87	44.66	38.44	42.47	44.61	47.01	1137.03	1241.70	1054.53	1109.09	1190.23	1223.48

core 1 synthase, glycoprotein-N-acetylgalactosamine 3-beta-galactosyltransferase, 1	C1galt1	100761169	6.28	5.63	5.80	6.28	6.61	6.41	312.93	278.17	299.34	310.59	327.06	340.22
C1GALT1-specific chaperone 1	C1galt1c1	100751243	22.14	22.13	18.77	20.07	25.68	23.42	865.87	861.96	745.25	777.64	999.80	908.50
complement component 1, q subcomponent, A chain	C1qa	100766409	0.00	0.00	0.00	0.00	0.00	0.00	0.00	0.00	0.00	0.00	0.00	0.00
complement component 1, q subcomponent, B chain	C1qb	100766700	0.00	0.00	0.00	0.00	0.00	0.00	0.00	0.00	0.00	0.00	0.00	0.00
complement component 1, q subcomponent binding protein	C1qbp	100754945	169.30	174.67	184.94	178.25	171.93	172.46	3660.98	3762.62	3951.08	3750.26	3601.75	3595.67
complement component 1, q subcomponent, C chain	C1qc	100769576	0.00	0.00	0.00	0.00	0.00	0.00	0.00	0.00	0.00	0.00	0.00	0.00
complement component 1, q subcomponent-like 1	C1ql1	100765437	0.00	0.00	0.00	0.00	0.00	0.00	0.00	0.92	0.00	0.00	0.00	0.00
complement component 1, q subcomponent-like 2	C1ql2	100760767	0.00	0.00	0.00	0.00	0.00	0.00	0.00	0.00	0.00	0.00	0.00	0.00
complement component 1, q subcomponent-like 3	C1ql3	100767576	0.82	0.95	1.85	1.84	1.63	1.47	59.24	72.29	136.63	139.07	115.92	111.23
complement component 1, q subcomponent-like 4	C1ql4	100769166	0.09	0.14	0.14	0.23	0.08	0.11	4.56	7.32	4.97	6.95	6.21	4.67
C1q and tumor necrosis factor related protein 1	C1qtnf1	100766965	3.38	3.51	4.20	2.74	2.68	3.05	151.15	157.39	195.01	134.43	127.30	126.18
C1q and tumor necrosis factor related protein 2	C1qtnf2	100765047	1.05	1.05	0.77	0.61	0.80	0.75	35.70	34.77	32.29	19.70	31.05	25.24
C1q and tumor necrosis factor related protein 3	C1qtnf3	100758853	0.02	0.00	0.03	0.03	0.03	0.02	2.28	0.92	1.24	3.48	2.07	3.74
C1q and tumor necrosis factor related protein 4	C1qtnf4	100764222	0.00	0.00	0.00	0.00	0.00	0.00	0.00	0.00	0.00	0.00	0.00	0.00
C1q and tumor necrosis factor related protein 5	C1qtnf5	100760072	8.61	9.54	10.05	9.37	6.36	7.16	342.55	366.01	407.40	355.79	227.70	263.58
C1q and tumor necrosis factor related protein 6	C1qtnf6	100750938	0.05	0.06	0.00	0.00	0.00	0.01	4.56	4.58	0.00	0.00	0.00	0.93
C1q and tumor necrosis factor related protein 7	C1qtnf7	100768477	0.00	0.00	0.00	0.00	0.00	0.00	0.00	0.00	0.00	0.00	0.00	0.00
C1QTNF9B antisense RNA 1	C1qtnf9b-as1	103161527	0.00	0.00	0.00	0.00	0.00	0.00	0.76	0.00	0.00	0.00	0.00	0.00
complement component 1, r subcomponent	C1r	100768476	80.83	82.78	84.45	83.55	73.53	74.90	6466.72	6651.38	6837.69	6680.01	5863.20	5987.49
complement component 1, r subcomponent-like	C1rl	100760066	5.62	5.88	5.50	5.58	4.36	5.52	446.61	479.48	447.15	448.50	360.18	450.51
complement component 1, s subcomponent	C1s	100759777	60.24	62.84	65.25	68.29	72.97	75.88	5275.00	5494.78	5686.27	5873.41	6129.19	6440.81
complement component 2	C2	100759069	0.03	0.00	0.00	0.02	0.00	0.01	2.28	0.00	1.24	1.16	1.03	1.87
C2 calcium-dependent domain containing 2	C2cd2	100762912	6.82	6.86	7.20	7.72	9.35	9.50	1216.78	1221.57	1274.38	1371.00	1646.66	1701.10
C2CD2-like	C2cd2l	100757251	8.29	7.89	9.50	9.54	8.34	8.82	829.42	789.67	953.92	951.47	837.30	868.31
C2 calcium-dependent domain containing 3	C2cd3	100760339	7.40	7.40	8.85	9.09	10.28	10.43	1728.71	1761.44	2087.95	2097.64	2393.92	2459.12
C2 calcium-dependent domain containing 4A	C2cd4a	103159384	0.00	0.00	0.00	0.00	0.00	0.00	0.00	0.00	0.00	0.00	0.00	0.00
C2 calcium-dependent domain containing 4C	C2cd4c	100774877	0.00	0.00	0.00	0.00	0.00	0.00	0.00	0.00	0.00	0.00	0.00	0.00
C2 calcium-dependent domain containing 5	C2cd5	100766087	12.11	12.53	10.27	11.71	13.28	11.71	1450.72	1518.96	1230.91	1397.66	1606.30	1447.80

complement component 3	C3	100763958	0.10	0.09	0.01	0.06	0.05	0.03	14.43	14.64	2.48	8.11	7.24	4.67
complement component 3a receptor 1	C3ar1	100771140	7.29	7.68	13.26	14.97	15.48	15.78	837.77	882.09	1568.76	1716.36	1798.81	1852.52
complement component 4A (Rodgers blood group)	C4a	100767983	0.32	0.22	0.11	0.11	0.08	0.11	58.48	38.43	23.60	22.02	16.56	19.63
complement component 4 binding protein, beta	C4bpb	100773000	0.20	0.24	0.07	0.25	0.22	0.20	6.08	9.15	1.24	9.27	9.31	8.41
complement component 5a receptor 1	C5ar1	100766822	0.00	0.00	0.00	0.00	0.00	0.00	0.00	0.00	0.00	0.00	0.00	0.00
complement component 5a receptor 2	C5ar2	100766530	0.24	0.19	0.19	0.10	0.02	0.04	11.39	10.07	8.69	4.64	2.07	1.87
complement component 6	C6	100766699	0.00	0.00	0.00	0.00	0.02	0.01	0.00	0.00	0.00	0.00	2.07	0.93
complement component 7	C7	100769002	0.14	0.08	0.14	0.14	0.23	0.21	14.43	7.32	16.15	18.54	23.80	18.69
complement component 8, alpha polypeptide	C8a	100774244	0.00	0.00	0.00	0.00	0.00	0.00	0.00	0.00	0.00	0.00	0.00	0.00
complement component 8, beta polypeptide	C8b	100773949	0.00	0.00	0.00	0.00	0.00	0.00	0.00	0.00	0.00	0.00	0.00	0.00
complement component 8, gamma polypeptide	C8g	100763555	0.22	0.29	0.33	0.34	0.51	0.35	18.99	21.96	31.05	26.66	38.29	26.17
complement component 9	C9	100774096	0.02	0.00	0.00	0.00	0.00	0.00	0.76	0.00	0.00	0.00	0.00	0.00
cytochrome c oxidase subunit I	COX1	3979185	0.00	0.00	0.00	0.00	0.00	0.00	0.00	0.00	0.00	0.00	0.00	0.00
cytochrome c oxidase subunit II	COX2	3979186	0.00	0.00	0.00	0.00	0.00	0.00						
cytochrome c oxidase subunit III	COX3	3979189	0.00	0.00	0.00	0.00	0.00	0.00						
cytochrome b	CYTB	3979182	0.00	0.00	0.00	0.00	0.00	0.00						
carbonic anhydrase I	Ca1	100772227	0.00	0.00	0.00	0.00	0.00	0.00	0.00	0.00	0.00	0.00	0.00	0.00
carbonic anhydrase X	Ca10	100760776	0.00	0.00	0.00	0.00	0.00	0.00	0.00	0.00	0.00	0.00	0.00	0.00
carbonic anhydrase XI	Ca11	100750594	0.07	0.06	0.04	0.00	0.03	0.06	3.80	2.75	3.73	0.00	3.10	1.87
carbonic anhydrase XII	Ca12	100772435	0.00	0.04	0.00	0.00	0.04	0.00	0.00	0.92	0.00	1.16	1.03	0.00
carbonic anhydrase XIII	Ca13	100771949	0.00	0.00	0.00	0.00	0.00	0.00	0.00	0.00	0.00	0.00	0.00	0.00
carbonic anhydrase XIV	Ca14	100760677	1.07	0.81	1.25	1.04	1.30	1.18	55.45	38.43	68.31	56.79	64.17	73.84
carbonic anhydrase II	Ca2	100774844	0.00	0.00	0.00	0.00	0.00	0.00	0.00	0.00	0.00	0.00	0.00	0.00
carbonic anhydrase III, muscle specific	Ca3	100774549	0.02	0.00	0.00	0.00	0.00	0.00	0.76	0.00	0.00	0.00	0.00	0.00
carbonic anhydrase IV	Ca4	100760390	0.23	0.03	0.05	0.04	0.00	0.04	6.08	0.92	1.24	1.16	0.00	0.93
carbonic anhydrase VA, mitochondrial	Ca5a	100755419	0.02	0.00	0.00	0.00	0.00	0.00	5.32	6.41	1.24	6.95	1.03	1.87
carbonic anhydrase VB, mitochondrial	Ca5b	100768750	6.42	5.76	4.44	5.11	7.68	6.66	262.04	250.72	171.41	199.33	299.11	259.84
carbonic anhydrase VI	Ca6	100754196	0.00	0.00	0.00	0.00	0.00	0.00	0.00	0.00	0.00	0.00	0.00	0.00
carbonic anhydrase VII	Ca7	100769849	0.00	0.00	0.00	0.00	0.00	0.00	0.00	0.00	0.00	0.00	0.00	0.00
carbonic anhydrase IX	Ca9	100756257	2.35	2.16	2.29	2.48	2.63	2.15	170.90	165.62	156.50	170.36	173.88	143.00
caspase activity and apoptosis inhibitor 1	Caap1	100766887	14.58	14.66	16.75	17.04	18.74	18.08	745.11	768.63	871.94	880.78	952.19	953.36
calcium binding protein 39	Cab39	100772119	38.55	39.59	40.50	45.04	44.73	42.06	3791.62	3937.39	4031.81	4418.96	4352.12	4132.17
calcium binding protein 39-like	Cab39l	100764345	22.89	22.94	21.65	23.24	22.00	23.69	982.84	987.32	953.92	998.99	941.84	1008.51
calcineurin binding protein 1	Cabin1	100764877	13.56	12.59	14.68	13.41	11.17	11.64	2975.87	2804.58	3277.87	2966.83	2504.67	2550.71
Cdk5 and Abl enzyme substrate 1	Cables1	100752271	2.06	2.05	2.57	2.73	2.66	2.66	112.41	109.80	137.87	158.77	151.11	143.94
Cdk5 and Abl enzyme substrate 2	Cables2	100765141	21.90	21.21	22.75	22.48	22.35	23.27	1734.79	1705.62	1827.11	1791.69	1744.99	1833.82

calcium binding protein 1	Cabp1	100756642	0.20	0.21	0.22	0.13	0.20	0.04	6.84	10.07	8.69	4.64	7.24	3.74
calcium binding protein 2	Cabp2	100757813	0.00	0.00	0.00	0.00	0.00	0.00	0.00	0.00	0.00	0.00	1.03	0.00
calcium binding protein 4	Cabp4	100759357	0.30	0.29	0.29	0.22	0.24	0.31	27.34	21.96	22.36	16.22	15.52	25.24
calcium binding protein 5	Cabp5	100753677	0.00	0.00	0.00	0.00	0.00	0.00	0.00	0.00	0.00	0.00	0.00	0.00
calcium binding protein 7	Cabp7	100753424	0.00	0.00	0.00	0.00	0.00	0.00	2.28	2.75	0.00	1.16	0.00	0.00
calcium-binding protein, spermatid-specific 1	Cabs1	100768190	0.00	0.00	0.00	0.00	0.00	0.00	0.00	0.00	0.00	0.00	0.00	0.00
calcium binding tyrosine-(Y)-phosphorylation regulated	Cabyr	100753647	0.35	0.40	0.56	0.33	0.54	0.48	24.31	25.62	32.29	28.97	36.22	38.32
calcium channel flower domain containing 1	Cacfd1	100769716	11.53	11.65	11.31	10.86	10.75	12.35	767.13	770.46	746.49	719.69	711.04	810.36
cache domain containing 1	Cachd1	100756408	19.34	18.87	19.79	21.36	20.12	21.03	2649.27	2640.79	2798.42	2942.50	2833.79	2884.39
calcium channel, voltage-dependent, P/Q type, alpha 1A subunit	Cacna1a	100758171	0.00	0.00	0.02	0.00	0.01	0.00	3.80	5.49	8.69	5.79	8.28	4.67
calcium channel, voltage-dependent, N type, alpha 1B subunit	Cacna1b	100770495	0.11	0.04	0.00	0.01	0.00	0.08	8.35	10.07	0.00	1.16	0.00	2.80
calcium channel, voltage-dependent, L type, alpha 1C subunit	Cacna1c	100754788	3.85	2.92	3.40	3.57	3.01	3.20	492.18	398.95	460.81	502.97	439.87	430.88
calcium channel, voltage-dependent, L type, alpha 1D subunit	Cacna1d	100772497	0.01	0.01	0.00	0.00	0.01	0.01	1.52	1.83	0.00	1.16	1.03	1.87
calcium channel, voltage-dependent, R type, alpha 1E subunit	Cacna1e	100773594	0.00	0.00	0.00	0.00	0.00	0.00	0.00	0.00	0.00	0.00	0.00	0.00
calcium channel, voltage-dependent, L type, alpha 1F subunit	Cacna1f	100757994	0.08	0.03	0.02	0.03	0.01	0.05	98.74	84.18	80.74	91.55	82.80	85.99
calcium channel, voltage-dependent, T type, alpha 1G subunit	Cacna1g	100761932	0.89	1.00	0.18	0.18	0.15	0.15	208.11	240.65	45.96	45.20	36.22	33.65
calcium channel, voltage-dependent, T type, alpha 1H subunit	Cacna1h	100762221	0.00	0.00	0.00	0.00	0.00	0.00	0.76	0.00	0.00	0.00	0.00	0.00
calcium channel, voltage-dependent, T type, alpha 1I subunit	Cacna1i	100769169	0.10	0.08	0.02	0.00	0.00	0.03	12.15	9.15	4.97	0.00	1.03	1.87
calcium channel, voltage-dependent, L type, alpha 1S subunit	Cacna1s	100765508	0.03	0.04	0.00	0.01	0.00	0.02	1.52	4.58	0.00	1.16	2.07	0.93
calcium channel, voltage-dependent, alpha 2/delta subunit 1	Cacna2d1	100764769	15.87	16.01	17.55	19.59	20.85	20.31	3139.94	3182.49	3520.07	3868.47	4107.86	4019.08
calcium channel, voltage-dependent, alpha 2/delta subunit 2	Cacna2d2	100769472	0.55	0.43	0.67	0.75	0.77	0.73	87.35	66.80	104.34	119.37	117.99	111.23
calcium channel, voltage-dependent, alpha 2/delta subunit 3	Cacna2d3	100753384	0.00	0.00	0.00	0.01	0.00	0.00	33.42	32.03	14.91	25.50	1.03	1.87
calcium channel, voltage-dependent, alpha 2/delta subunit 4	Cacna2d4	100755076	0.01	0.01	0.00	0.00	0.00	0.00	2.28	1.83	0.00	1.16	3.10	0.93
calcium channel, voltage-dependent, beta 1 subunit	Cacnb1	100757399	0.00	0.00	0.00	0.00	0.00	0.00	1.52	0.00	0.00	0.00	0.00	0.00
calcium channel, voltage-dependent, beta 2 subunit	Cacnb2	100774766	0.00	0.00	0.00	0.00	0.00	0.00	2.28	1.83	3.73	2.32	3.10	4.67
calcium channel, voltage-dependent, beta 3 subunit	Cacnb3	100768080	6.71	5.75	10.90	9.90	9.24	9.28	505.85	448.37	811.08	742.87	681.02	705.68
calcium channel, voltage-dependent, beta 4 subunit	Cacnb4	100765888	0.37	0.22	0.13	0.09	0.11	0.12	18.23	16.47	9.94	8.11	8.28	7.48
calcium channel, voltage-dependent, gamma subunit 1	Cacng1	100766592	0.00	0.00	0.00	0.00	0.00	0.00	0.76	0.00	0.00	0.00	0.00	0.00
calcium channel, voltage-dependent, gamma subunit 2	Cacng2	100769826	0.00	0.00	0.00	0.00	0.00	0.00	0.00	0.00	0.00	0.00	0.00	0.00

calcium channel, voltage-dependent, gamma subunit 3	Cacng3	100760631	0.00	0.00	0.00	0.00	0.00	0.00	0.00	0.00	0.00	0.00	0.00	0.00
calcium channel, voltage-dependent, gamma subunit 4	Cacng4	100771968	0.42	0.41	0.15	0.14	0.16	0.09	169.38	135.42	136.63	124.00	75.55	92.53
calcium channel, voltage-dependent, gamma subunit 5	Cacng5	100766301	0.00	0.05	0.00	0.00	0.00	0.00	0.76	0.92	0.00	0.00	0.00	0.00
calcium channel, voltage-dependent, gamma subunit 6	Cacng6	100763524	0.00	0.00	0.00	0.00	0.00	0.00	0.00	0.00	0.00	0.00	0.00	0.00
calcium channel, voltage-dependent, gamma subunit 7	Cacng7	100763239	0.05	0.00	0.00	0.00	0.00	0.00	0.00	0.00	0.00	0.00	0.00	0.00
cactin, spliceosome C complex subunit	Cactin	100771890	19.57	19.29	15.50	8.95	6.13	5.96	1505.41	1499.74	1228.42	698.83	485.41	482.29
CDK2-associated, cullin domain 1	Cacul1	100751838	13.38	12.88	13.09	12.68	15.04	14.65	1042.85	1001.05	1030.93	993.19	1184.02	1149.64
calcyclin binding protein	Cacybp	100752498	172.58	177.08	211.70	217.38	209.98	196.74	5824.15	5938.57	7173.05	7243.25	7074.13	6589.42
carbamoyl-phosphate synthetase 2, aspartate transcarbamylase, and dihydroorotase	Cad	100689232	26.19	25.29	26.75	22.40	16.27	17.05	5703.38	5491.12	5840.29	4808.36	3503.43	3691.01
cell adhesion molecule 1	Cadm1	100766050	0.00	0.01	0.00	0.00	0.00	0.00	0.00	0.92	0.00	0.00	0.00	0.00
cell adhesion molecule 2	Cadm2	100773220	0.09	0.15	0.05	0.02	0.09	0.08	3.80	8.24	1.24	1.16	7.24	2.80
cell adhesion molecule 3	Cadm3	100757404	0.00	0.00	0.00	0.00	0.00	0.00	0.00	0.00	0.00	0.00	0.00	0.00
Ca++-dependent secretion activator	Cadps	100761643	1.35	1.24	3.63	4.23	3.74	3.65	183.05	176.60	499.32	592.21	515.42	504.72
Ca++-dependent secretion activator 2	Cadps2	100752027	0.00	0.00	0.00	0.00	0.00	0.00	0.00	0.00	0.00	0.00	0.00	0.00
cancer antigen 1	Cage1	100765629	0.29	0.25	0.39	0.40	0.41	0.36	82.03	75.03	91.91	84.60	83.83	83.19
calbindin 1, 28kDa	Calb1	100751076	0.00	0.00	0.00	0.00	0.00	0.00	0.76	0.00	0.00	0.00	0.00	0.00
calbindin 2	Calb2	100754392	0.00	0.00	0.00	0.00	0.00	0.00	0.00	0.00	0.00	0.00	0.00	0.00
calcium binding and coiled-coil domain 1	Calcoco 1	100768405	32.00	29.73	38.47	35.49	35.55	39.66	1588.96	1487.84	1947.59	1774.31	1790.53	2003.00
calcium binding and coiled-coil domain 2	Calcoco 2	100752435	64.05	63.81	51.38	55.32	61.04	61.02	4615.72	4577.91	3698.93	3943.80	4361.43	4342.48
calcitonin receptor	Calcr	100765683	1.06	1.33	0.13	0.08	0.07	0.14	129.88	169.28	19.87	15.07	11.38	22.43
calcitonin receptor-like	Calcrl	100753229	0.05	0.01	0.00	0.07	0.05	0.12	6.84	1.83	0.00	9.27	7.24	16.82
caldesmon 1	Cald1	100763421	39.67	41.14	36.99	37.63	38.52	36.56	4670.41	4867.98	4426.80	4460.68	4562.22	4357.43
calcium homeostasis modulator 1	Calhm1	100753669	0.00	0.00	0.00	0.00	0.00	0.00	0.00	0.00	0.00	0.00	0.00	0.93
calcium homeostasis modulator 2	Calhm2	100753368	5.08	4.95	5.37	3.81	3.67	3.73	560.54	547.19	598.69	478.63	431.59	443.03
calcium homeostasis modulator 3	Calhm3	100753966	0.76	0.75	0.60	0.88	0.72	0.74	67.60	63.14	52.17	77.65	69.34	68.23
calmodulin 1 (phosphorylase kinase, delta)	Calm1	100771772	92.08	96.47	97.70	107.57	111.02	101.95	6135.56	6424.45	6452.64	7055.50	7270.78	6724.95
calmodulin 2 (phosphorylase kinase, delta)	Calm2	100765281	367.75	377.89	327.37	362.56	401.84	390.91	9798.82	10074.52	8754.23	9536.75	10578.60	10319.69
calmodulin 3 (phosphorylase kinase, delta)	Calm3	100762749	39.57	43.65	41.74	36.02	32.03	32.70	893.22	1002.88	941.50	735.91	592.01	622.49
calmodulin-like 3	Calml3	100766751	0.00	0.00	0.00	0.00	0.00	0.00	0.00	0.00	0.00	0.00	0.00	0.00
calmodulin-like 4	Calml4	100757500	0.00	0.13	0.18	0.00	0.00	0.07	0.76	3.66	3.73	1.16	0.00	0.93
calmodulin-like 5	Calml5	100767046	0.00	0.00	0.00	0.00	0.00	0.00	0.00	0.92	0.00	1.16	0.00	0.00
calmodulin	Calmod ulin	100751524	33.32	31.02	29.27	29.96	25.90	30.94						

calmodulin	Calmodulin	100759184	0.00	0.00	0.00	0.00	0.00	0.00	50.89	44.84	57.14	61.42	62.10	54.21
calneuron 1	Caln1	100751134	0.00	0.00	0.00	0.00	0.00	0.00	0.00	0.00	0.00	0.00	0.00	0.00
calreticulin	Calr	100689096	782.44	832.21	977.39	998.90	1016.45	1002.67	30877.55	32555.98	38464.95	38345.18	37613.47	38013.02
calreticulin 3	Calr3	100759217	1.96	2.11	1.06	1.29	1.08	1.41	88.11	95.16	47.20	60.26	45.54	64.49
calumenin	Calu	100750933	164.39	174.08	174.56	192.29	211.21	209.25	13447.64	14333.09	14428.08	15792.60	17180.77	17096.98
calcyon neuron-specific vesicular protein	Caly	100772099	0.00	0.00	0.00	0.00	0.00	0.00	0.00	0.00	0.00	0.00	0.00	0.00
calcium/calmodulin-dependent protein kinase I	Camk1	100755149	32.24	30.84	38.52	36.13	33.11	35.43	1939.86	1902.35	2399.71	2170.66	2022.36	2077.77
calcium/calmodulin-dependent protein kinase ID	Camk1d	100772409	0.00	0.00	0.00	0.00	0.00	0.00	0.00	0.92	0.00	0.00	0.00	0.00
calcium/calmodulin-dependent protein kinase IG	Camk1g	100763374	0.00	0.00	0.00	0.00	0.00	0.00	0.00	0.00	0.00	0.00	0.00	0.00
calcium/calmodulin-dependent protein kinase II alpha	Camk2a	100765253	0.02	0.00	0.03	0.00	0.00	0.00	0.00	0.00	0.00	0.00	0.00	0.00
calcium/calmodulin-dependent protein kinase II beta	Camk2b	100769205	0.04	0.02	0.06	0.05	0.00	0.06	2.28	1.83	2.48	2.32	0.00	1.87
calcium/calmodulin-dependent protein kinase II gamma	Camk2g	100763272	21.06	20.63	21.74	22.55	21.83	22.44	2228.49	2242.75	2362.45	2416.35	2339.07	2403.04
calcium/calmodulin-dependent protein kinase II inhibitor 2	Camk2n2	100750713	13.25	12.04	9.74	1.05	0.83	1.63	39.50	49.41	36.02	12.75	4.14	8.41
calcium/calmodulin-dependent protein kinase IV	Camk4	100774010	0.08	0.02	0.03	0.00	0.02	0.00	5.32	0.92	1.24	0.00	1.03	0.00
calcium/calmodulin-dependent protein kinase kinase 1, alpha	Camkk1	100768559	0.92	0.89	1.41	0.85	0.81	0.91	46.33	45.75	73.28	49.83	42.43	49.54
calcium/calmodulin-dependent protein kinase kinase 2, beta	Camkk2	100765904	10.79	11.41	11.42	10.50	8.91	9.01	1247.16	1304.84	1412.25	1347.82	1248.19	1273.02
calmodulin-lysine N-methyltransferase	Camkmt	100766475	6.29	6.49	6.55	7.59	8.66	7.59	324.32	336.73	356.48	385.92	438.83	386.95
CaM kinase-like vesicle-associated	Camkv	100770335	0.00	0.00	0.00	0.00	0.00	0.00	0.00	0.00	0.00	0.00	0.00	0.00
calcium modulating ligand	Camlg	100769365	163.66	165.29	180.46	173.53	165.43	164.68	5654.01	5763.80	6349.55	6017.11	5725.54	5700.55
cathelicidin antimicrobial peptide	Camp	100767672	0.66	0.41	0.44	0.43	0.41	0.55	27.34	24.71	24.84	28.97	23.80	29.91
calmodulin regulated spectrin-associated protein 1	Camsap1	100760053	11.70	11.70	11.41	11.65	10.10	10.51	2074.30	2090.85	2043.23	2102.28	1827.79	1905.79
calmodulin regulated spectrin-associated protein family, member 2	Camsap2	100758440	13.78	14.84	13.81	15.74	17.51	17.02	2445.72	2646.28	2515.23	2816.18	3056.31	2960.10
calmodulin regulated spectrin-associated protein family, member 3	Camsap3	100772080	3.98	4.08	2.47	2.37	0.95	0.86	596.24	618.56	380.08	360.42	149.04	135.53
calmodulin binding transcription activator 1	Camta1	103164264	3.84	3.59	3.55	3.70	3.86	3.50	413.19	390.72	366.42	389.40	400.54	384.15
calmodulin binding transcription activator 2	Camta2	100772126	1.95	1.55	1.84	2.24	1.62	1.82	232.42	195.82	229.79	264.23	201.82	228.99
cullin-associated and neddylation-dissociated 1	Cand1	100768210	31.02	30.39	34.29	37.12	39.32	38.06	6463.68	6342.10	7221.49	7698.70	8131.89	7940.95
cullin-associated and neddylation-dissociated 2 (putative)	Cand2	100770365	3.68	3.74	4.63	3.34	2.63	3.06	439.01	444.71	555.21	407.94	308.43	379.48
calcium activated nucleotidase 1	Cant1	100767250	35.52	34.28	42.39	33.23	29.20	31.08	2788.27	2698.43	3341.21	2595.98	2268.69	2438.55
calnexin	Canx	100689345	219.14	228.34	238.27	255.36	256.27	256.31	19425.22	20039.23	21315.45	22554.90	23343.08	23219.06
CAP, adenylate cyclase-associated protein 1 (yeast)	Cap1	100774682	115.42	116.42	113.44	104.85	109.33	114.76	7338.67	7473.99	7240.12	6634.82	6905.43	7292.29
CAP, adenylate cyclase-associated protein, 2 (yeast)	Cap2	100766472	0.00	0.00	0.02	0.00	0.00	0.00	0.00	0.00	1.24	0.00	0.00	0.00

capping protein (actin filament), gelsolin-like	Capg	100774775	183.83	184.27	174.62	146.87	119.54	123.65	6921.68	6940.53	6523.44	5508.35	4566.36	4759.34
calpain 1, (mu/I) large subunit	Capn1	100758636	12.13	13.30	17.21	17.36	15.14	16.21	1136.27	1232.55	1607.26	1623.65	1406.55	1555.29
calpain 10	Capn10	100752300	10.50	9.66	11.31	10.39	8.74	10.29	946.39	851.90	1029.69	946.84	817.64	984.21
calpain 11	Capn11	100755371	0.04	0.07	0.00	0.02	0.07	0.00	3.80	8.24	2.48	3.48	5.17	0.93
calpain 12	Capn12	100767183	0.15	0.15	0.04	0.02	0.12	0.05	6.84	5.49	1.24	1.16	3.10	0.93
calpain 13	Capn13	100760070	0.00	0.00	0.00	0.00	0.00	0.00	0.00	0.00	0.00	0.00	0.00	0.00
calpain 15	Capn15	100763096	3.27	3.49	3.92	3.04	1.51	1.94	381.29	399.87	448.39	326.82	166.63	214.97
calpain 2, (m/II) large subunit	Capn2	100771239	62.73	59.59	55.80	56.70	59.78	62.38	6265.44	6136.21	5625.41	5648.58	6036.04	6169.75
calpain 3, (p94)	Capn3	100758723	0.00	0.00	0.00	0.00	0.00	0.00	0.00	0.00	0.00	0.00	0.00	0.00
calpain 5	Capn5	100768370	7.95	7.50	8.91	8.67	9.67	9.21	822.58	799.74	950.20	919.02	1026.71	979.53
calpain 6	Capn6	100773921	0.03	0.04	0.00	0.02	0.06	0.04	2.28	3.66	0.00	1.16	4.14	2.80
calpain 7	Capn7	100766061	15.99	16.71	15.52	17.29	17.38	15.74	1783.40	1859.35	1706.63	1916.85	1915.76	1732.88
calpain 8	Capn8	100771531	1.17	1.05	0.60	0.86	0.77	0.64	120.01	123.53	57.14	69.54	80.73	68.23
calpain 9	Capn9	100766480	0.00	0.00	0.00	0.00	0.00	0.02	0.00	0.00	0.00	0.00	0.00	0.93
calpain, small subunit 1	Capns1	100770160	162.74	156.39	147.34	151.99	157.01	170.02	4322.54	4272.29	3972.19	4026.09	4280.70	4557.45
calpain, small subunit 2	Capns2	100772425	0.00	0.00	0.00	0.00	0.00	0.00	0.00	0.00	0.00	0.00	0.00	0.00
cell cycle associated protein 1	Caprin1	100756029	197.98	199.98	207.00	213.60	219.66	214.72	22033.47	22224.33	23038.23	23502.90	24034.45	23700.42
caprin family member 2	Caprin2	100762439	3.60	3.60	4.47	5.15	5.31	4.76	226.34	223.27	273.26	302.48	320.85	274.79
calcyphosine	Caps	100759707	0.21	0.11	0.18	0.13	0.11	0.09	10.63	7.32	11.18	6.95	2.07	3.74
calcyphosine 2	Caps2	100760290	0.74	0.69	0.25	0.48	0.37	0.42	64.56	62.22	19.87	44.04	33.12	36.45
calcyphosine-like	Capsl	100757989	0.07	0.21	0.00	0.15	0.05	0.04	1.52	4.58	0.00	3.48	1.03	0.93
capping protein (actin filament) muscle Z-line, alpha 1	Capza1	100750522	96.23	101.23	97.20	98.14	115.87	114.42	8399.74	8797.13	8433.77	8477.50	9930.69	9832.73
capping protein (actin filament) muscle Z-line, alpha 2	Capza2	100753621	101.48	101.90	80.98	89.62	103.47	97.89	7704.01	7688.11	6168.20	6668.42	7734.45	7347.44
capping protein (actin filament) muscle Z-line, alpha 3	Capza3	100753734	0.00	0.00	0.00	0.00	0.00	0.00	0.00	0.00	0.00	0.00	0.00	0.00
capping protein (actin filament) muscle Z-line, beta	Capzb	100755876	180.26	172.93	176.96	181.96	184.33	185.15	6533.56	6412.55	6527.17	6533.99	6727.41	6673.54
caspase recruitment domain family, member 10	Card10	100762588	13.08	11.87	6.48	5.18	5.89	6.32	1581.36	1457.65	781.27	629.29	698.62	751.47
caspase recruitment domain family, member 11	Card11	100769055	0.00	0.00	0.01	0.00	0.00	0.01	0.00	0.00	1.24	0.00	0.00	0.93
caspase recruitment domain family, member 14	Card14	100757428	3.04	3.14	3.78	3.75	3.72	4.14	301.54	322.09	372.63	323.34	301.18	354.24
caspase recruitment domain family, member 6	Card6	100769285	2.82	2.92	3.40	4.03	4.33	4.31	284.83	291.90	344.06	403.30	436.76	434.62
caspase recruitment domain family, member 9	Card9	100759255	0.09	0.05	0.07	0.14	0.10	0.07	13.67	10.07	7.45	16.22	11.38	10.28
calcium responsive transcription factor	Carf	100768109	2.23	2.31	2.25	2.67	2.86	2.05	268.12	283.66	280.71	330.29	337.41	259.84
calcium regulated heat stable protein 1, 24kDa	Carhsp1	100764910	8.86	8.86	8.08	7.42	7.97	8.19	751.94	750.33	688.12	626.98	668.60	696.33

carbohydrate kinase domain containing	Carkd	100767494	23.98	23.62	23.80	20.59	18.02	17.96	950.94	937.91	955.16	841.38	735.88	742.13
coactivator-associated arginine methyltransferase 1	Carm1	100771088	89.92	85.15	80.29	75.22	65.09	71.48	5737.56	5481.97	5219.25	4968.29	4383.17	4691.11
carnosine synthase 1	Carns1	100765077	0.64	0.73	0.89	0.53	0.33	0.53	113.93	120.78	124.21	114.73	65.20	97.21
cysteinyl-tRNA synthetase	Cars	100761418	45.76	45.13	49.83	51.00	46.25	46.37	3954.92	3969.42	4347.30	4296.12	3891.55	3866.73
cysteinyl-tRNA synthetase 2, mitochondrial (putative)	Cars2	100765566	4.26	4.14	4.14	3.19	3.62	3.43	262.80	257.12	265.81	223.67	230.80	233.67
CART prepropeptide	Cartpt	100757030	0.00	0.00	0.00	0.00	0.00	0.00	0.00	0.00	0.00	0.00	0.00	0.00
cancer susceptibility candidate 1	Casc1	100765214	2.42	2.64	2.64	3.01	3.62	3.22	236.22	245.23	233.51	261.92	315.67	288.81
cancer susceptibility candidate 3	Casc3	100773741	52.39	52.85	33.78	36.95	35.03	37.21	5670.72	5772.95	3725.02	4024.93	3781.84	4071.42
cancer susceptibility candidate 4	Casc4	100765980	11.22	11.70	11.59	13.87	15.01	13.96	1150.70	1215.16	1186.19	1412.72	1527.64	1427.24
cancer susceptibility candidate 5	Casc5	100755881	6.75	6.49	5.92	6.93	8.76	7.72	1306.41	1245.36	1140.24	1371.00	1690.13	1480.52
CAS1 domain containing 1	Casd1	100766850	7.95	8.67	8.52	10.08	11.19	10.57	852.96	942.48	924.11	1076.64	1191.27	1130.02
calcium/calmodulin-dependent serine protein kinase (MAGUK family)	Cask	100764902	11.16	11.08	11.55	12.90	12.08	11.65	1310.96	1330.46	1371.26	1495.01	1437.60	1373.03
CASK interacting protein 1	Caskin1	100769762	0.01	0.01	0.00	0.00	0.01	0.00	1.52	0.92	0.00	0.00	2.07	0.00
CASK interacting protein 2	Caskin2	100769048	5.29	5.65	5.29	4.16	2.96	3.50	865.87	930.59	854.56	653.63	482.30	567.34
caspase 1, apoptosis-related cysteine peptidase	Casp1	100759171	0.12	0.05	0.05	0.02	0.09	0.17	9.11	5.49	3.73	1.16	6.21	14.02
caspase 14, apoptosis-related cysteine peptidase	Casp14	100771116	0.00	0.00	0.00	0.00	0.00	0.00	0.00	0.00	0.00	0.00	0.00	0.00
caspase 16, apoptosis-related cysteine peptidase (putative)	Casp16	100773249	0.02	0.10	0.07	0.03	0.03	0.02	2.28	10.07	8.69	6.95	5.17	5.61
caspase 2, apoptosis-related cysteine peptidase	Casp2	100689445	7.86	7.11	8.30	8.51	8.52	8.55	306.85	284.58	327.91	315.23	314.64	336.48
caspase 3, apoptosis-related cysteine peptidase	Casp3	100689061	15.22	15.96	16.25	19.56	20.63	21.46	994.24	1037.65	1153.90	1220.34	1369.29	1317.88
caspase 6, apoptosis-related cysteine peptidase	Casp6	100766458	8.60	7.50	5.49	5.59	6.06	6.01	484.59	430.98	308.04	321.02	339.48	322.46
caspase 7, apoptosis-related cysteine peptidase	Casp7	100766857	23.65	24.72	26.62	25.49	27.95	28.56	1599.59	1687.32	1789.85	1708.25	1819.51	1878.69
caspase 8, apoptosis-related cysteine peptidase	Casp8	100689369	35.74	38.29	44.38	46.32	44.05	44.30	1929.23	2093.60	2405.92	2569.33	2409.45	2412.38
caspase 8 associated protein 2	Casp8ap2	100773754	7.61	6.28	6.35	9.08	10.24	9.86	657.76	709.15	689.36	930.61	973.92	921.58
calsequestrin 1 (fast-twitch, skeletal muscle)	Casq1	100762901	0.22	0.14	0.21	0.20	0.26	0.14	15.95	10.98	16.15	15.07	18.63	11.22
calsequestrin 2 (cardiac muscle)	Casq2	100752020	0.02	0.00	0.00	0.00	0.00	0.00	0.76	0.00	0.00	0.00	1.03	0.00
calcium-sensing receptor	Casr	100766791	0.00	0.00	0.00	0.00	0.00	0.00	0.00	0.00	0.00	0.00	0.00	0.00
Cas scaffolding protein family member 4	Cass4	100764252	0.00	0.00	0.00	0.00	0.00	0.00	0.00	0.00	0.00	0.00	0.00	0.00
calpastatin	Cast	100761152	37.99	37.72	31.73	37.57	43.02	40.09	4655.98	4732.55	3962.26	4606.71	5116.97	4751.86
castor zinc finger 1	Casz1	100770088	0.01	0.01	0.00	0.00	0.00	0.00	0.76	1.83	0.00	0.00	0.00	0.00
catalase	Cat	100773774	33.84	31.88	33.36	34.28	34.43	34.62	1994.55	1878.56	1956.29	2028.11	2094.81	2061.88
ciliogenesis associated TTC17 interacting protein	Catip	100751611	0.03	0.02	0.03	0.05	0.05	0.08	2.28	1.83	2.48	2.32	2.07	6.54
cation channel, sperm associated 1	Catsper1	100769314	0.09	0.07	0.10	0.00	0.14	0.05	4.56	8.24	9.94	3.48	9.31	5.61

cation channel, sperm associated 2	Catsper 2	100766859	2.31	2.25	2.27	1.93	1.83	1.77	257.48	258.04	280.71	290.89	276.34	268.25
cation channel, sperm associated 3	Catsper 3	100762411	0.02	0.00	0.00	0.00	0.00	0.00	0.76	0.00	0.00	0.00	0.00	0.00
cation channel, sperm associated 4	Catsper 4	100771423	0.00	0.00	0.00	0.00	0.00	0.00	0.00	0.00	0.00	0.00	0.00	0.00
catsper channel auxiliary subunit beta	Catsper b	100751204	0.00	0.00	0.00	0.00	0.00	0.00	0.00	0.00	0.00	0.00	0.00	0.00
catsper channel auxiliary subunit delta	Catsper d	100771676	0.27	0.28	0.25	0.38	0.35	0.41	47.09	45.75	55.89	69.54	68.31	66.36
catsper channel auxiliary subunit gamma	Catsper g	100764558	0.52	0.42	0.43	0.60	0.56	0.47	89.63	70.46	70.80	93.87	81.76	79.45
caveolin 1, caveolae protein, 22kDa	Cav1	100753021	170.46	177.22	171.94	196.07	239.93	233.66	11772.86	12284.33	11969.99	13559.36	16466.63	16194.09
caveolin 2	Cav2	100751645	16.27	16.90	15.92	16.64	22.94	19.23	875.75	926.93	855.80	901.64	1278.21	1069.26
caveolin 3	Cav3	100773488	0.00	0.00	0.00	0.00	0.00	0.00	0.00	0.00	0.00	0.00	0.00	0.00
core-binding factor, runt domain, alpha subunit 2; translocated to, 2	Cbfa2t2	100773698	11.28	11.56	12.76	13.20	13.17	13.21	2003.66	2053.34	2275.50	2350.29	2328.72	2341.35
core-binding factor, runt domain, alpha subunit 2; translocated to, 3	Cbfa2t3	100764900	0.00	0.00	0.00	0.02	0.00	0.00	0.00	0.00	0.00	1.16	0.00	0.00
core-binding factor, beta subunit	Cbfb	100765267	34.57	38.05	36.89	36.11	40.21	40.34	2564.96	2796.34	2732.59	2671.31	2960.06	2972.25
Cbl proto-oncogene, E3 ubiquitin protein ligase	Cbl	100754737	17.10	16.33	14.59	16.74	17.56	18.71	2040.12	1975.56	1772.46	2024.63	2127.93	2266.57
Cbl proto-oncogene B, E3 ubiquitin protein ligase	Cblb	100774600	4.50	4.19	4.04	4.27	4.76	4.85	565.10	531.63	514.22	531.94	581.66	607.54
Cbl proto-oncogene C, E3 ubiquitin protein ligase	Cblc	100751369	0.02	0.00	0.00	0.04	0.03	0.00	0.76	0.00	1.24	1.16	1.03	0.00
Cbl proto-oncogene-like 1, E3 ubiquitin protein ligase	Cbll1	100758211	6.28	5.91	5.34	5.10	5.41	5.60	720.80	686.28	614.83	594.53	623.06	649.60
cerebellin 1 precursor	Cbln1	100760568	0.00	0.00	0.00	0.00	0.00	0.00	0.00	0.00	0.00	0.00	0.00	0.00
cerebellin 2 precursor	Cbln2	100753954	0.00	0.00	0.00	0.00	0.00	0.00	0.00	0.00	0.00	0.00	0.00	0.00
cerebellin 3 precursor	Cbln3	100756636	0.06	0.00	0.20	0.00	0.00	0.16	1.52	0.00	2.48	0.00	0.00	1.87
cerebellin 4 precursor	Cbln4	100761933	0.00	0.00	0.00	0.03	0.00	0.00	0.00	0.00	0.00	1.16	0.00	0.00
carbonyl reductase 3	Cbr3	100689021	15.70	16.63	19.15	16.56	13.67	14.69	494.46	522.48	606.14	519.20	426.41	475.75
carbonyl reductase 4	Cbr4	100758439	10.93	13.12	9.45	10.29	11.17	10.52	356.22	424.58	311.76	336.09	378.80	340.22
cystathionine-beta-synthase	Cbs	100762141	0.01	0.01	0.00	0.00	0.02	0.03	4.56	5.49	3.73	4.64	8.28	11.22
chromobox homolog 1	Cbx1	100689359	55.91	59.59	42.16	45.69	46.54	46.75	2201.14	2385.49	1659.43	1798.64	1837.10	1847.84
chromobox homolog 2	Cbx2	100755979	9.29	9.22	15.66	15.85	14.62	15.18	991.96	975.43	1674.33	1683.91	1542.13	1600.15
chromobox homolog 3	Cbx3	100689443	67.80	68.19	88.24	91.09	109.15	108.15	2457.87	2486.15	3267.93	3377.09	3967.10	3891.96
chromobox homolog 4	Cbx4	100756554	3.23	3.45	3.54	2.30	2.13	1.88	310.65	326.67	337.85	228.31	200.79	182.26
chromobox homolog 5	Cbx5	100689287	121.72	123.03	124.57	125.10	135.09	140.59	2628.01	2653.60	2696.57	2641.18	2848.28	2965.71
chromobox homolog 6	Cbx6	103161880	49.14	48.97	55.84	49.45	46.44	50.87	7274.11	7281.84	8341.86	7354.51	6885.76	7605.41
chromobox homolog 7	Cbx7	100750509	2.56	2.47	2.22	2.38	2.30	2.16	225.58	214.12	188.80	203.97	202.86	186.93
chromobox homolog 8	Cbx8	100756264	3.24	3.19	3.69	2.97	2.72	3.29	287.87	281.83	327.91	267.71	252.54	297.22
chibby homolog 1 (Drosophila)	Cby1	100774410	10.95	11.84	13.69	13.84	15.92	17.23	591.68	653.33	742.77	760.25	858.00	939.34
coiled-coil and C2 domain containing 1A	Cc2d1a	100767087	5.46	5.56	7.87	6.07	4.72	5.36	637.25	641.44	859.52	694.19	535.09	607.54
coiled-coil and C2 domain containing 1B	Cc2d1b	100754941	15.01	14.91	15.33	14.44	13.31	14.35	2577.88	2571.24	2653.10	2487.04	2272.83	2432.01
coiled-coil and C2 domain containing 2A	Cc2d2a	100767719	6.56	6.32	5.51	6.02	6.91	7.60	1101.33	1094.38	927.84	1010.58	1161.25	1247.78

coiled-coil and C2 domain containing 2B	Cc2d2b	100763170	0.00	0.00	0.00	0.00	0.00	0.00	0.00	0.00	0.00	0.00	0.00	0.00
cell division cycle and apoptosis regulator 1	Ccar1	100767167	45.31	43.08	35.93	40.57	40.19	40.29	5715.53	5557.91	4654.10	5254.54	5223.58	5118.25
cell cycle and apoptosis regulator 2	Ccar2	100769631	25.67	24.70	26.46	22.95	19.08	20.79	2727.51	2639.87	2887.85	2522.97	2171.40	2397.43
collagen and calcium binding EGF domains 1	Ccbe1	100754816	41.85	41.48	33.72	35.23	33.26	33.23	4762.31	4768.24	3910.09	4060.86	3846.01	3832.15
cysteine conjugate-beta lyase, cytoplasmic	Ccbl1	100769821	11.36	9.79	20.59	19.03	14.30	14.88	648.65	581.05	1152.66	1043.03	814.53	844.01
cysteine conjugate-beta lyase 2	Ccbl2	100771070	45.89	42.14	55.40	54.79	70.82	61.66	1089.18	1084.31	1413.49	1288.72	1713.94	1466.50
coiled-coil domain containing 101	Ccdc101	100769355	13.92	12.29	15.83	15.65	12.01	11.41	525.60	487.71	612.35	577.14	466.78	454.25
coiled-coil domain containing 102A	Ccdc102a	100766334	20.80	19.70	18.81	15.46	11.18	12.28	916.76	870.20	817.29	673.33	488.51	528.09
coiled-coil domain containing 103	Ccdc103	100758187	7.04	7.69	6.90	6.25	7.12	8.07	182.29	195.82	176.38	164.57	202.86	214.04
coiled-coil domain containing 104	Ccdc104	100766513	66.24	68.43	73.67	80.00	90.41	79.02	2501.16	2568.50	2728.86	2958.72	3331.62	2947.95
coiled-coil domain containing 105	Ccdc105	100751575	0.00	0.00	0.00	0.00	0.00	0.00	0.00	0.00	0.00	0.00	0.00	0.00
coiled-coil domain containing 107	Ccdc107	100773807	6.76	7.41	6.36	6.78	6.23	6.63	123.05	136.34	115.51	135.59	129.37	121.51
coiled-coil domain containing 108	Ccdc108	100750413	0.00	0.00	0.00	0.00	0.00	0.00	0.00	0.00	0.00	0.00	0.00	0.00
coiled-coil domain containing 109B	Ccdc109b	100765870	35.58	40.36	39.33	40.99	49.75	46.57	1088.42	1194.12	1160.11	1190.21	1476.93	1382.38
coiled-coil domain containing 11	Ccdc11	100763503	0.04	0.02	0.03	0.00	0.00	0.00	3.04	1.83	1.24	0.00	3.10	0.00
coiled-coil domain containing 110	Ccdc110	100771325	0.00	0.00	0.00	0.00	0.00	0.00	0.00	0.92	0.00	0.00	0.00	0.93
coiled-coil domain containing 112	Ccdc112	100759442	6.39	5.85	5.39	6.31	6.91	5.92	408.63	370.59	349.03	391.71	442.97	372.93
coiled-coil domain containing 113	Ccdc113	100760328	0.04	0.00	0.00	0.00	0.00	0.00	2.28	0.00	0.00	0.00	0.00	0.00
coiled-coil domain containing 114	Ccdc114	100751784	2.16	2.74	2.28	2.08	1.89	1.67	155.71	192.16	160.23	148.34	163.53	142.07
coiled-coil domain containing 115	Ccdc115	100773635	21.61	21.91	21.17	17.14	13.94	14.79	738.27	727.45	752.70	665.22	611.68	649.60
coiled-coil domain containing 116	Ccdc116	100754886	0.00	0.00	0.00	0.00	0.00	0.00	0.00	0.00	0.00	0.00	0.00	0.00
coiled-coil domain containing 117	Ccdc117	100751075	21.38	22.04	24.77	26.49	30.28	28.93	1981.64	2041.44	2333.88	2455.75	2795.50	2686.24
coiled-coil domain containing 12	Ccdc12	100766224	21.45	21.70	21.43	21.80	14.48	17.61	446.61	443.79	443.43	428.80	290.83	353.31
coiled-coil domain containing 120	Ccdc120	100760495	5.96	6.07	4.98	4.58	5.01	5.23	719.28	737.52	602.41	536.58	595.12	616.88
coiled-coil domain containing 121	Ccdc121	100762691	0.00	0.00	0.00	0.00	0.11	0.00	0.76	0.92	0.00	0.00	4.14	0.00
coiled-coil domain containing 122	Ccdc122	100770045	9.29	9.63	8.40	10.80	11.42	10.31	452.69	480.39	423.55	526.15	553.72	500.05
coiled-coil domain containing 124	Ccdc124	100753303	36.56	34.51	35.66	21.59	14.87	15.84	1737.83	1643.40	1716.56	992.04	682.06	707.54
coiled-coil domain containing 125	Ccdc125	100769496	1.79	1.86	2.07	2.35	2.37	2.76	186.85	184.84	201.22	231.78	230.80	278.53

coiled-coil domain containing 126	Ccdc126	100762153	9.46	8.78	8.89	9.28	10.09	11.12	568.14	524.31	535.34	562.08	610.64	666.42
coiled-coil domain containing 127	Ccdc127	100751028	14.29	14.25	14.18	15.10	16.92	16.31	1007.15	1018.43	1006.09	1067.37	1194.37	1159.93
coiled-coil domain containing 129	Ccdc129	100771395	0.03	0.00	0.00	0.00	0.00	0.00	2.28	0.00	0.00	0.00	0.00	0.00
coiled-coil domain containing 13	Ccdc13	100766977	2.59	2.80	3.72	3.78	3.61	4.17	336.48	357.78	447.15	478.63	398.47	484.16
coiled-coil domain containing 130	Ccdc130	100765628	13.22	12.48	13.27	12.34	10.07	10.18	619.02	566.41	597.44	544.69	425.38	431.82
coiled-coil domain containing 132	Ccdc132	100760449	16.59	17.10	15.79	18.02	20.54	19.68	1722.63	1775.17	1635.83	1848.48	2086.53	2010.47
coiled-coil domain containing 134	Ccdc134	100762269	20.80	20.76	21.09	19.78	19.64	20.19	1218.30	1223.40	1304.19	1178.62	1189.20	1237.50
coiled-coil domain containing 135	Ccdc135	100764290	0.30	0.19	0.54	0.54	0.03	0.04	28.86	18.30	50.93	45.20	5.17	2.80
coiled-coil domain containing 136	Ccdc136	100751519	4.68	4.26	5.40	5.06	4.58	4.17	570.41	513.33	660.79	638.56	553.72	533.70
coiled-coil domain containing 137	Ccdc137	100765099	275.20	278.12	222.83	210.49	161.71	161.20	7696.41	7694.52	6149.57	5815.46	4301.40	4478.00
coiled-coil domain containing 138	Ccdc138	100761168	1.25	1.20	1.39	1.45	2.14	1.80	83.55	87.84	100.61	99.67	149.04	122.44
coiled-coil domain containing 14	Ccdc14	100757937	3.75	4.13	4.97	4.86	5.88	5.42	391.16	437.39	517.95	523.83	634.45	600.06
coiled-coil domain containing 141	Ccdc141	100768994	0.00	0.01	0.02	0.01	0.00	0.00	0.00	0.92	2.48	1.16	0.00	0.00
coiled-coil domain containing 142	Ccdc142	100764197	1.18	1.20	1.06	0.89	0.69	0.57	173.17	179.35	173.89	162.25	129.37	136.46
coiled-coil domain containing 146	Ccdc146	100760217	1.48	1.46	1.35	1.80	1.86	1.77	240.01	243.40	219.85	286.25	296.01	275.73
coiled-coil domain containing 147	Ccdc147	100762393	0.03	0.05	0.03	0.01	0.01	0.00	3.80	4.58	2.48	1.16	1.03	0.00
coiled-coil domain containing 148	Ccdc148	100757988	0.46	0.53	0.06	0.21	0.07	0.09	28.10	31.11	3.73	11.59	6.21	5.61
coiled-coil domain containing 149	Ccdc149	100765586	0.00	0.00	0.00	0.00	0.00	0.00	0.00	0.00	0.00	0.00	0.00	0.00
coiled-coil domain containing 15	Ccdc15	100755146	1.60	1.24	1.34	1.84	1.98	1.97	217.23	172.94	188.80	243.37	274.27	263.58
coiled-coil domain containing 151	Ccdc151	100754389	6.38	6.85	5.46	5.05	5.14	5.43	398.00	428.24	341.57	301.32	318.78	331.81
coiled-coil domain containing 152	Ccdc152	100763254	0.00	0.00	0.00	0.00	0.00	0.06	0.00	0.00	0.00	1.16	0.00	0.93
coiled-coil domain containing 153	Ccdc153	100755793	0.77	0.40	0.36	0.17	0.15	0.14	17.47	9.15	7.45	6.95	8.28	4.67
coiled-coil domain containing 154	Ccdc154	100764550	0.12	0.06	0.02	0.08	0.02	0.10	15.95	5.49	6.21	9.27	10.35	9.35
coiled-coil domain containing 155	Ccdc155	100755883	0.00	0.00	0.00	0.00	0.00	0.00	0.00	0.00	0.00	0.00	0.00	0.00
coiled-coil domain containing 157	Ccdc157	100754911	1.45	1.30	1.06	0.88	0.87	1.00	186.85	164.71	150.29	141.39	129.37	157.96
coiled-coil domain containing 158	Ccdc158	100769646	0.53	0.61	0.69	0.83	0.36	0.49	85.07	97.91	105.58	137.91	63.13	77.58
coiled-coil domain containing 159	Ccdc159	100769664	4.36	3.55	7.53	7.60	7.26	8.89	208.87	177.52	360.21	355.79	351.90	404.71

coiled-coil domain containing 160	Ccdc160	100766990	0.00	0.00	0.00	0.00	0.00	0.00	0.00	0.00	0.00	0.00	0.00	0.00
coiled-coil domain containing 166	Ccdc166	100763932	0.02	0.03	0.00	0.00	0.03	0.03	0.76	0.92	0.00	0.00	1.03	0.93
coiled-coil domain containing 167	Ccdc167	100755674	38.51	35.26	36.71	30.38	35.05	38.36	461.80	441.96	438.46	373.17	418.13	462.66
coiled-coil domain containing 168	Ccdc168	103162099	0.00	0.00	0.01	0.00	0.00	0.00	0.00	0.92	1.24	0.00	1.03	0.93
coiled-coil domain containing 169	Ccdc169	100767466	0.00	0.00	0.00	0.00	0.00	0.00	0.76	2.75	1.24	1.16	0.00	0.00
coiled-coil domain containing 17	Ccdc17	100761042	1.43	1.45	1.41	1.62	1.59	1.64	270.40	280.92	267.05	295.52	279.45	285.07
coiled-coil domain containing 170	Ccdc170	100773188	2.06	2.45	1.73	2.19	2.70	2.76	294.70	351.37	245.93	316.39	382.94	392.56
coiled-coil domain containing 172	Ccdc172	100767515	0.00	0.00	0.00	0.00	0.00	0.00	0.00	0.00	3.73	1.16	0.00	0.00
coiled-coil domain containing 173	Ccdc173	100764424	2.71	3.06	3.61	4.05	4.57	4.19	154.19	172.94	201.22	219.04	239.08	223.39
coiled-coil domain containing 174	Ccdc174	100765768	22.72	23.24	22.80	24.23	25.13	23.84	1150.70	1153.86	1142.72	1183.26	1231.63	1172.08
coiled-coil domain containing 175	Ccdc175	100764413	0.44	0.50	0.32	0.28	0.23	0.27	42.53	52.16	21.12	34.77	20.70	25.24
coiled-coil domain containing 176	Ccdc176	100754703	2.72	3.14	3.29	3.77	3.90	4.10	287.11	303.79	322.94	370.85	432.62	420.60
coiled-coil domain containing 178	Ccdc178	100757741	0.00	0.00	0.00	0.00	0.00	0.00	0.00	0.00	0.00	0.00	0.00	0.00
coiled-coil domain containing 179	Ccdc179	100766121	0.00	0.00	0.00	0.00	0.00	0.00	0.00	0.00	0.00	0.00	0.00	0.00
coiled-coil domain containing 18	Ccdc18	100770535	2.21	2.53	2.21	2.44	3.16	2.88	473.19	534.38	493.11	557.44	688.27	636.51
coiled-coil domain containing 180	Ccdc180	100769850	0.00	0.00	0.00	0.00	0.00	0.00	0.76	0.00	0.00	0.00	0.00	0.00
coiled-coil domain containing 181	Ccdc181	100761297	0.32	0.37	0.27	0.21	0.34	0.23	31.90	34.77	17.39	18.54	28.98	19.63
coiled-coil domain containing 182	Ccdc182	100761428	0.00	0.00	0.00	0.00	0.00	0.00	0.76	0.00	1.24	0.00	0.00	0.00
coiled-coil domain containing 183	Ccdc183	100761232	0.14	0.15	0.11	0.12	0.20	0.21	42.53	46.67	49.68	73.01	87.97	102.81
coiled-coil domain containing 184	Ccdc184	100753374	0.00	0.00	0.00	0.00	0.00	0.00	0.00	0.00	0.00	0.00	0.00	0.00
coiled-coil domain containing 185	Ccdc185	100754743	0.00	0.00	0.00	0.00	0.00	0.00	0.00	0.00	0.00	0.00	0.00	0.00
coiled-coil domain containing 19	Ccdc19	100755656	2.34	2.11	2.09	2.29	2.38	1.93	150.39	129.93	122.97	146.02	160.42	139.27
coiled-coil domain containing 22	Ccdc22	100757706	36.48	34.01	37.31	31.78	23.84	28.63	3045.75	2879.61	3212.04	2718.83	2074.11	2418.93
coiled-coil domain containing 23	Ccdc23	100773604	42.20	41.73	45.73	47.11	46.23	49.68	648.65	634.12	694.33	712.74	686.20	749.61
coiled-coil domain containing 24	Ccdc24	100754400	0.67	0.87	0.75	1.00	0.69	0.52	50.13	58.56	52.17	63.74	54.85	34.58
coiled-coil domain containing 25	Ccdc25	100756157	31.05	31.37	27.63	35.22	38.25	32.75	862.08	840.92	763.88	953.79	1050.51	898.22
coiled-coil domain containing 27	Ccdc27	100774639	0.00	0.00	0.00	0.00	0.00	0.00	0.00	0.00	0.00	0.00	0.00	0.00

coiled-coil domain containing 28A	Ccdc28a	100770774	22.79	21.89	22.48	25.27	27.13	30.60	751.94	728.37	765.13	783.43	855.93	940.28
coiled-coil domain containing 28B	Ccdc28b	100753619	0.70	1.02	0.47	0.87	0.56	0.45	37.22	44.84	31.05	39.40	37.26	25.24
coiled-coil domain containing 3	Ccdc3	100772989	0.00	0.00	0.00	0.00	0.00	0.00	0.00	0.00	0.00	0.00	0.00	0.00
coiled-coil domain containing 30	Ccdc30	100770106	0.03	0.01	0.00	0.00	0.05	0.00	2.28	1.83	3.73	2.32	5.17	0.93
coiled-coil domain containing 33	Ccdc33	100756733	0.00	0.00	0.00	0.00	0.00	0.00	0.00	0.00	0.00	0.00	0.00	0.00
coiled-coil domain containing 34	Ccdc34	100752060	38.10	36.60	34.56	37.04	39.33	35.02	1490.22	1453.99	1362.57	1483.42	1543.16	1378.64
coiled-coil domain containing 36	Ccdc36	100771768	0.00	0.00	0.00	0.00	0.00	0.00	0.00	0.00	0.00	0.00	1.03	0.00
coiled-coil domain containing 37	Ccdc37	100771826	0.00	0.00	0.00	0.00	0.00	0.00	1.52	0.92	2.48	3.48	2.07	0.00
coiled-coil domain containing 38	Ccdc38	100757876	0.00	0.00	0.00	0.00	0.00	0.00	1.52	4.58	0.00	2.32	2.07	0.93
coiled-coil domain containing 39	Ccdc39	100750499	0.95	1.02	1.29	1.35	1.49	1.23	247.61	251.63	298.10	310.59	364.32	298.16
coiled-coil domain containing 40	Ccdc40	100756847	0.19	0.16	0.17	0.15	0.16	0.16	29.62	24.71	26.08	23.18	22.77	23.37
coiled-coil domain containing 42	Ccdc42	100752092	0.00	0.00	0.00	0.00	0.00	0.00	0.00	0.00	0.00	0.00	0.00	0.00
coiled-coil domain containing 42B	Ccdc42b	100770861	0.00	0.00	0.00	0.00	0.00	0.00	0.76	0.00	0.00	0.00	0.00	0.00
coiled-coil domain containing 43	Ccdc43	100763684	93.62	99.86	67.44	69.82	77.67	71.72	5528.69	5929.42	4035.54	4173.27	4577.74	4304.15
coiled-coil domain containing 50	Ccdc50	100750624	36.13	34.06	37.56	40.53	39.23	38.82	2190.51	2174.12	2352.51	2578.60	2530.54	2484.35
coiled-coil domain containing 51	Ccdc51	100762437	15.29	14.69	16.95	15.84	13.44	14.61	637.25	613.07	716.68	658.27	557.86	610.34
coiled-coil domain containing 53	Ccdc53	100771573	73.38	71.24	75.15	80.70	81.16	85.91	1605.67	1626.01	1683.03	1758.08	1820.54	1847.84
coiled-coil domain containing 54	Ccdc54	100769857	0.00	0.00	0.00	0.00	0.00	0.00	0.00	0.00	0.00	0.00	0.00	0.00
coiled-coil domain containing 57	Ccdc57	100771842	4.62	4.36	2.24	1.81	1.07	1.39	489.14	477.65	242.21	184.27	111.78	150.48
coiled-coil domain containing 58	Ccdc58	100767366	67.30	68.12	59.98	60.79	65.37	60.33	1869.23	1952.68	1706.63	1672.32	1808.12	1601.09
coiled-coil domain containing 6	Ccdc6	100761094	15.90	14.98	13.51	14.42	12.67	14.21	732.20	680.78	604.90	679.13	605.47	637.44
coiled-coil domain containing 60	Ccdc60	100759279	0.00	0.00	0.00	0.00	0.00	0.00	0.00	0.00	0.00	0.00	0.00	0.00
coiled-coil domain containing 61	Ccdc61	100758950	1.86	2.05	1.51	1.96	1.53	1.15	55.45	63.14	60.86	53.31	43.47	32.71
coiled-coil domain containing 62	Ccdc62	100766639	0.07	0.00	0.00	0.05	0.05	0.02	3.04	0.00	1.24	5.79	2.07	0.93
coiled-coil domain containing 63	Ccdc63	100774262	0.00	0.02	0.03	0.00	0.03	0.00	0.00	0.92	1.24	0.00	1.03	0.00
coiled-coil domain containing 64	Ccdc64	100759857	0.10	0.05	0.04	0.01	0.04	0.02	9.11	5.49	4.97	1.16	5.17	2.80
coiled-coil domain containing 64B	Ccdc64b	100767692	0.10	0.10	0.08	0.03	0.00	0.02	12.15	9.15	13.66	8.11	3.10	2.80
coiled-coil domain containing 65	Ccdc65	100767212	2.95	3.31	3.53	4.15	3.93	4.45	155.71	176.60	188.80	224.83	214.24	243.01

coiled-coil domain containing 66	Ccdc66	100752485	4.12	3.77	4.13	5.38	5.53	4.88	391.16	355.03	394.98	493.70	520.60	455.18
coiled-coil domain containing 67	Ccdc67	100753613	0.29	0.25	0.21	0.16	0.26	0.23	38.74	35.69	29.81	32.45	34.15	31.78
coiled-coil domain containing 68	Ccdc68	100761099	0.00	0.00	0.00	0.03	0.00	0.00	0.00	0.00	0.00	1.16	0.00	0.00
coiled-coil domain containing 69	Ccdc69	100765513	0.04	0.19	0.20	0.12	0.17	0.00	1.52	4.58	4.97	2.32	4.14	2.80
coiled-coil domain containing 7	Ccdc7	100764183	0.00	0.00	0.00	0.00	0.00	0.00	0.00	0.00	0.00	0.00	0.00	0.00
coiled-coil domain containing 70	Ccdc70	100770438	0.00	0.00	0.00	0.00	0.00	0.00	0.00	0.00	0.00	0.00	0.00	0.00
coiled-coil domain containing 71	Ccdc71	100762268	32.90	32.13	36.86	32.33	30.79	34.65	2215.58	2177.78	2502.80	2220.49	2102.06	2441.36
coiled-coil domain containing 73	Ccdc73	100770503	0.06	0.06	0.05	0.05	0.08	0.03	10.63	12.81	14.91	15.07	15.52	10.28
coiled-coil domain containing 74B	Ccdc74b	100757786	0.12	0.07	0.09	0.04	0.04	0.04	6.08	7.32	3.73	4.64	2.07	1.87
coiled-coil domain containing 77	Ccdc77	100753915	30.01	27.61	31.42	37.37	34.20	30.11	2697.88	2489.81	2884.13	3404.91	3066.66	2742.32
coiled-coil domain containing 78	Ccdc78	100761067	0.00	0.04	0.00	0.00	0.02	0.02	0.76	2.75	2.48	1.16	1.03	1.87
coiled-coil domain containing 79	Ccdc79	100767836	0.01	0.00	0.00	0.00	0.00	0.00	0.76	0.00	0.00	1.16	0.00	0.00
coiled-coil domain containing 8	Ccdc8	103164277	1.91	1.87	1.93	1.50	1.26	0.93	169.38	159.22	172.65	147.18	115.92	98.14
coiled-coil domain containing 80	Ccdc80	100765789	128.22	130.25	165.89	176.06	137.40	140.80	17298.51	17676.62	22574.93	23949.08	18633.89	19125.21
coiled-coil domain containing 81	Ccdc81	100762831	0.25	0.31	0.08	0.09	0.15	0.18	18.23	22.88	8.69	8.11	17.59	14.95
coiled-coil domain containing 82	Ccdc82	100762899	4.20	4.23	3.25	3.76	5.41	4.83	390.40	398.04	321.70	370.85	519.56	473.88
coiled-coil domain containing 83	Ccdc83	100763604	0.60	0.30	0.19	0.32	0.45	0.26	31.90	21.96	13.66	19.70	24.84	20.56
coiled-coil domain containing 84	Ccdc84	100759181	8.37	9.09	12.06	9.62	10.80	12.39	379.77	396.21	510.50	429.96	483.34	521.55
coiled-coil domain containing 85A	Ccdc85a	100750517	0.00	0.00	0.00	0.00	0.00	0.00	0.00	0.00	0.00	0.00	0.00	0.00
coiled-coil domain containing 85B	Ccdc85b	100766533	35.83	30.45	27.65	13.25	10.30	10.10	1606.43	1374.38	1187.44	584.10	440.90	458.92
coiled-coil domain containing 85C	Ccdc85c	100769004	12.80	12.62	15.02	12.99	9.33	10.80	1240.33	1202.35	1453.24	1223.82	873.53	1025.33
coiled-coil domain containing 86	Ccdc86	100774139	77.51	79.46	73.02	63.32	41.81	46.60	2866.50	2931.77	2715.20	2345.65	1575.25	1789.89
coiled-coil domain containing 87	Ccdc87	100761101	0.02	0.02	0.07	0.05	0.04	0.05	1.52	1.83	6.21	3.48	4.14	4.67
coiled-coil domain containing 88A	Ccdc88a	100765924	6.11	5.98	4.96	6.33	7.16	6.16	1409.70	1367.98	1162.59	1493.85	1671.50	1466.50
coiled-coil domain containing 88B	Ccdc88b	100774835	0.02	0.01	0.03	0.00	0.02	0.01	41.02	37.52	24.84	26.66	12.42	16.82
coiled-coil domain containing 88C	Ccdc88c	100753852	0.37	0.33	0.14	0.14	0.04	0.09	80.51	71.37	32.29	31.29	9.31	18.69
coiled-coil domain containing 89	Ccdc89	100761005	0.00	0.00	0.00	0.00	0.00	0.00	0.00	0.00	0.00	0.00	0.00	0.00
coiled-coil domain containing 9	Ccdc9	100767115	6.78	5.94	7.18	4.09	2.84	3.11	341.03	319.35	367.66	213.24	163.53	171.98

coiled-coil domain containing 90B	Ccdc90b	100751582	21.77	23.01	24.57	26.99	27.99	26.08	911.45	959.87	1017.27	1087.07	1151.94	1074.87
coiled-coil domain containing 92	Ccdc92	100767201	8.08	8.21	7.51	6.36	6.63	6.51	505.09	507.84	478.20	403.30	406.75	413.12
coiled-coil domain containing 93	Ccdc93	100760176	8.59	8.72	9.37	9.70	10.73	11.80	1396.79	1449.41	1545.16	1580.77	1712.90	1858.12
coiled-coil domain containing 94	Ccdc94	100755173	5.72	5.79	2.76	2.65	2.16	2.58	615.99	627.71	308.04	289.73	235.98	289.75
coiled-coil domain containing 96	Ccdc96	100750342	0.22	0.26	0.23	0.19	0.28	0.25	12.15	13.73	13.66	10.43	14.49	13.09
coiled-coil domain containing 97	Ccdc97	100773067	7.24	7.11	7.64	6.70	5.18	4.82	476.99	480.39	506.77	441.55	345.69	315.92
coiled-coil glutamate-rich protein 1	Ccer1	100754117	0.00	0.00	0.00	0.00	0.00	0.00	0.00	0.00	0.00	0.00	0.00	0.00
coiled-coil glutamate-rich protein 2	Ccer2	100769486	0.10	0.04	0.11	0.10	0.05	0.00	20.51	10.98	26.08	19.70	8.28	9.35
coiled-coil alpha-helical rod protein 1	Cchcr1	100754418	6.63	6.32	7.62	5.25	3.93	4.43	592.44	570.98	665.76	482.11	357.07	408.45
calicin	Ccin	100757067	0.14	0.17	0.11	0.14	0.14	0.12	11.39	14.64	8.69	12.75	11.38	9.35
cholecystokinin	Cck	100772438	0.00	0.00	0.00	0.00	0.00	0.00	0.00	0.00	0.00	0.00	0.00	0.00
cholecystokinin A receptor	Cckar	100774917	0.10	0.11	0.31	0.39	0.19	0.19	11.39	10.98	29.81	41.72	19.66	21.50
cholecystokinin B receptor	Cckbr	100754060	0.02	0.00	0.00	0.00	0.00	0.00	1.52	0.00	0.00	0.00	0.00	0.00
chemokine (C-C motif) ligand 1	Ccl1	103159565	0.00	0.00	0.00	0.00	0.00	0.00	0.00	0.00	0.00	0.00	0.00	0.00
chemokine (C-C motif) ligand 11	Ccl11	100763547	0.00	0.13	0.00	0.00	0.00	0.00	0.00	1.83	0.00	0.00	0.00	0.00
chemokine (C-C motif) ligand 16	Ccl16	100751682	0.00	0.00	0.00	0.00	0.00	0.00	0.76	0.92	0.00	0.00	0.00	0.93
chemokine (C-C motif) ligand 17	Ccl17	100764062	1.19	1.82	0.19	0.30	0.24	0.49	34.18	45.75	3.73	9.27	9.31	12.15
chemokine (C-C motif) ligand 19	Ccl19	100751942	0.00	0.00	0.00	0.00	0.00	0.00	0.00	0.00	0.00	0.00	0.00	0.00
chemokine (C-C motif) ligand 20	Ccl20	100756991	0.04	0.00	0.00	0.00	0.00	0.00	0.76	0.00	0.00	0.00	0.00	0.00
chemokine (C-C motif) ligand 21	Ccl21	100770444	0.00	0.00	0.00	0.00	0.00	0.00	0.00	0.92	0.00	1.16	0.00	0.93
chemokine (C-C motif) ligand 22	Ccl22	100760298	0.00	0.00	0.00	0.00	0.00	0.00	0.00	0.00	0.00	0.00	0.00	0.00
chemokine (C-C motif) ligand 24	Ccl24	100762320	0.00	0.00	0.00	0.00	0.00	0.00	0.00	0.00	0.00	0.00	0.00	0.00
chemokine (C-C motif) ligand 25	Ccl25	100758484	0.04	0.00	0.00	0.14	0.18	0.06	0.76	0.00	1.24	2.32	3.10	3.74
chemokine (C-C motif) ligand 26	Ccl26	103161431	0.00	0.00	0.00	0.00	0.00	0.00	0.00	0.00	0.00	0.00	0.00	0.00
chemokine (C-C motif) ligand 27	Ccl27	100770725	2.28	2.52	3.40	3.56	3.96	4.39	70.64	75.03	120.48	129.80	129.37	141.14
chemokine (C-C motif) ligand 28	Ccl28	100765151	0.00	0.00	0.00	0.00	0.00	0.00	0.76	0.00	0.00	0.00	0.00	0.00
chemokine (C-C motif) ligand 5	Ccl5	100773745	0.26	0.00	0.00	0.00	0.00	0.00	1.52	0.00	0.00	0.00	1.03	0.00
cerebral cavernous malformation 2	Ccm2	100766896	16.21	15.59	15.32	13.76	8.83	10.89	794.48	757.65	751.46	691.88	449.18	553.32
cerebral cavernous malformation 2-like	Ccm2l	100773603	0.00	0.00	0.00	0.00	0.00	0.00	0.00	0.00	0.00	0.00	0.00	0.00
cyclin A1	Ccna1	100769969	0.00	0.00	0.00	0.00	0.00	0.00	0.00	0.00	0.00	0.00	0.00	0.00
cyclin A2	Ccna2	100759649	79.41	83.53	71.95	80.65	91.22	84.34	3942.77	4158.83	3547.40	3810.53	4217.57	3908.79
cyclin B1	Ccnb1	100770842	84.85	83.43	83.00	88.63	96.55	91.51	5836.30	5847.06	5908.61	6210.65	6745.01	6426.79
cyclin B1 interacting protein 1, E3 ubiquitin protein ligase	Ccnb1ip1	100753354	0.00	0.00	0.00	0.00	0.00	0.00	0.00	0.00	0.00	0.00	0.00	0.00
cyclin B2	Ccnb2	100773656	50.70	52.46	50.40	57.01	92.59	89.17	2031.77	2148.50	2023.36	2234.40	3636.94	3552.68
cyclin B3	Ccnb3	100755282	0.01	0.01	0.00	0.00	0.00	0.00	0.76	0.92	0.00	0.00	1.03	0.00
cyclin C	Ccnc	100764362	16.45	16.14	14.67	15.61	15.83	15.56	728.40	755.82	667.00	718.53	838.34	747.74
cyclin D1	Ccnd1	100689063	51.90	53.19	41.86	36.30	29.22	31.72	1530.47	1579.35	1204.82	958.43	715.18	779.51
cyclin D2	Ccnd2	100771544	68.13	67.57	80.86	82.69	71.50	75.93	12124.52	12017.14	14524.96	14762.32	12671.34	13587.29
cyclin D3	Ccnd3	100766025	44.96	45.48	48.87	41.70	35.88	40.36	2147.98	2122.88	2350.03	1914.54	1631.14	1818.87
cyclin D-type binding-protein 1	Ccndbp1	100756614	23.67	23.60	25.26	25.55	28.20	29.76	1056.52	1048.63	1094.28	1109.09	1234.74	1301.06

cyclin E1	Ccne1	100753358	7.95	7.05	5.68	5.43	3.41	3.67	382.05	355.95	285.68	261.92	163.53	184.13
cyclin E2	Ccne2	100760464	3.67	5.20	5.43	6.56	6.88	6.75	134.44	170.20	149.05	157.61	192.51	178.52
cyclin F	Ccnf	100774506	18.29	16.27	14.53	14.33	12.50	13.26	1936.83	1744.97	1568.76	1530.93	1336.17	1407.61
cyclin G1	Ccng1	100771963	11.33	11.19	6.42	7.62	7.61	8.03	1004.11	960.79	555.21	660.58	661.36	701.00
cyclin G2	Ccng2	100753075	7.05	7.14	8.12	8.68	8.79	9.33	515.73	527.97	589.99	623.50	648.94	681.37
cyclin H	Ccnh	100762144	53.82	54.74	50.67	54.06	56.30	55.22	1847.20	1914.25	1750.10	1843.84	1979.93	1902.99
cyclin I	Ccni	100752774	72.37	72.59	85.12	87.77	108.41	112.56	4572.43	4628.24	5416.74	5585.99	6804.00	7102.55
cyclin J	Ccnj	100763458	2.55	2.57	2.42	2.75	2.79	2.75	271.92	270.85	258.35	285.09	284.62	284.14
cyclin J-like	Ccnjl	100767662	2.47	2.44	2.58	2.48	2.59	2.65	234.70	216.86	242.21	228.31	239.08	241.14
cyclin K	Ccnk	100769288	26.62	26.79	24.25	22.66	19.78	21.31	1972.52	1996.60	1809.72	1696.66	1482.10	1577.72
cyclin L1	Ccnl1	100758148	42.53	44.39	30.57	35.48	37.63	33.42	2093.29	2164.97	1517.83	1811.39	1881.61	1688.95
cyclin L2	Ccnl2	100770856	39.74	39.40	41.61	43.61	47.16	43.21	3326.02	3354.51	3629.38	3694.64	4090.27	3753.63
cyclin O	Ccno	100751971	0.00	0.00	0.00	0.00	0.00	0.00	0.00	0.00	0.00	0.00	0.00	0.00
cyclin T1	Ccnt1	100758059	8.54	9.59	7.75	8.58	8.57	8.17	600.04	588.37	562.67	574.82	578.56	556.13
cyclin T2	Ccnt2	100764441	8.30	8.14	8.28	9.37	9.23	9.26	817.26	842.75	894.30	1027.96	1002.90	1000.10
cyclin Y	Ccny	100771569	43.19	44.72	47.50	48.01	49.03	52.25	2391.79	2513.60	2655.58	2701.44	2735.47	2896.54
cyclin Y-like 1	Ccnyl1	100760997	9.95	9.94	9.41	10.18	11.98	11.53	870.43	862.88	847.10	902.80	1041.20	1005.70
centriolar coiled coil protein 110kDa	Ccp110	100775002	7.85	7.97	7.08	8.53	9.54	9.09	830.18	851.90	789.97	884.26	1008.08	931.87
cell cycle progression 1	Ccpg1	100768292	27.14	27.24	33.67	40.76	44.61	41.39	2571.80	2620.66	3194.65	3728.25	4030.24	3738.68
chemokine (C-C motif) receptor 10	Ccr10	100760302	2.76	2.41	1.78	0.95	1.05	1.10	248.37	241.57	190.04	115.89	133.51	144.87
chemokine (C-C motif) receptor 2	Ccr2	100762449	0.00	0.00	0.00	0.00	0.00	0.00	0.00	0.00	0.00	0.00	0.00	0.00
chemokine (C-C motif) receptor 3	Ccr3	100762154	0.00	0.00	0.05	0.00	0.00	0.00	0.00	0.00	1.24	0.00	0.00	0.00
chemokine (C-C motif) receptor 4	Ccr4	100770515	0.13	0.00	0.00	0.03	0.08	0.02	5.32	0.00	0.00	1.16	3.10	0.93
chemokine (C-C motif) receptor 5	Ccr5	100762740	0.03	0.00	0.00	0.00	0.00	0.00	0.76	0.00	0.00	0.00	0.00	0.00
chemokine (C-C motif) receptor 6	Ccr6	100756572	0.00	0.00	0.00	0.00	0.03	0.00	0.00	0.00	0.00	0.00	1.03	0.00
chemokine (C-C motif) receptor 7	Ccr7	100773169	2.28	2.02	0.19	0.23	0.77	0.99	152.67	138.17	13.66	17.38	51.75	67.30
chemokine (C-C motif) receptor 8	Ccr8	100753346	0.05	0.07	0.00	0.00	0.00	0.00	1.52	1.83	0.00	0.00	0.00	0.00
chemokine (C-C motif) receptor 9	Ccr9	100763320	0.02	0.02	0.03	0.03	0.05	0.00	1.52	0.92	1.24	1.16	2.07	0.00
chemokine (C-C motif) receptor-like 2	Ccrl2	100768546	11.06	11.44	8.21	10.05	8.70	8.77	438.25	444.71	330.40	387.08	337.41	359.85
CCR4 carbon catabolite repression 4-like (S. cerevisiae)	Ccrn4l	100766002	29.37	30.64	38.82	38.35	31.97	33.23	1838.84	1914.25	2432.01	2380.42	1970.61	2073.10
copper chaperone for superoxide dismutase	Ccs	100757246	42.48	43.30	39.71	38.13	29.41	31.76	1240.33	1232.55	1165.08	1087.07	851.79	930.93
centriole, cilia and spindle-associated protein	Ccsap	100769539	13.25	14.33	17.49	18.16	20.43	18.65	1105.89	1197.78	1478.08	1517.03	1685.99	1561.83
coiled-coil serine-rich protein 1	Ccser1	100775049	0.00	0.00	0.00	0.00	0.00	0.00	0.00	0.00	0.00	0.00	0.00	0.00
coiled-coil serine-rich protein 2	Ccser2	100752321	7.95	7.52	7.76	9.12	10.19	8.54	735.99	720.13	712.96	813.56	932.52	811.29
chaperonin containing TCP1, subunit 2 (beta)	Cct2	100759253	273.95	270.31	257.67	272.14	275.74	291.87	15777.15	15800.80	14989.50	15521.41	15778.37	16439.91
chaperonin containing TCP1, subunit 3 (gamma)	Cct3	100755267	412.85	420.95	428.44	420.03	376.11	408.56	22875.80	23334.27	23696.53	22841.15	20671.78	22344.21
chaperonin containing TCP1, subunit 4 (delta)	Cct4	100767849	204.13	207.70	207.70	217.42	219.67	215.24	11309.54	11516.61	11484.33	11897.47	12004.81	11754.41
chaperonin containing TCP1, subunit 5 (epsilon)	Cct5	100757108	196.97	203.06	204.14	201.72	178.45	180.50	9596.02	9864.06	10034.82	9885.59	8937.11	9012.09
chaperonin containing TCP1, subunit 6A (zeta 1)	Cct6a	100754913	260.61	255.48	242.05	258.56	250.26	248.29	22025.88	22083.42	20894.38	21953.42	21270.00	20781.45

chaperonin containing TCP1, subunit 6B (zeta 2)	Cct6b	100764422	0.22	0.16	0.11	0.18	0.21	0.27	15.95	14.64	8.69	11.59	13.45	15.89
chaperonin containing TCP1, subunit 7 (eta)	Cct7	100770561	314.26	313.97	327.96	325.25	291.24	302.63	15041.91	15164.85	15829.15	15435.65	13832.59	14357.46
chaperonin containing TCP1, subunit 8 (theta)	Cct8	100761027	631.61	618.42	611.48	646.82	681.20	679.38	14088.69	13917.66	13685.31	14177.07	15035.25	14705.16
chaperonin containing TCP1, subunit 8 (theta)-like 2	Cct8l2	100767085	0.00	0.00	0.00	0.00	0.00	0.00	0.00	0.00	0.00	0.00	0.00	0.00
CD101 molecule	Cd101	100771961	0.01	0.01	0.01	0.01	0.02	0.02	1.52	0.92	2.48	1.16	4.14	2.80
CD109 molecule	Cd109	100757414	16.63	17.24	26.10	30.64	29.22	28.00	2325.71	2417.52	3667.88	4239.33	4019.89	3816.26
CD14 molecule	Cd14	100757057	0.00	0.00	0.00	0.03	0.00	0.02	0.00	0.00	0.00	1.16	0.00	0.93
CD151 molecule (Raph blood group)	Cd151	100754541	126.69	121.70	148.97	158.86	159.05	168.09	6287.47	6061.18	7370.54	7775.19	7826.57	8415.77
CD160 molecule	Cd160	100772814	0.00	0.00	0.00	0.00	0.00	0.00	0.76	0.92	0.00	0.00	0.00	0.00
CD164 molecule, sialomucin	Cd164	100751988	101.72	103.02	101.21	106.21	113.49	113.10	7690.33	7804.32	7710.88	7978.00	8398.91	8466.24
CD164 sialomucin-like 2	Cd164l2	100759840	0.00	0.00	0.00	0.00	0.00	0.00	0.00	0.00	0.00	0.00	0.00	0.00
CD177 molecule	Cd177	100770932	0.00	0.00	0.00	0.00	0.00	0.00	0.00	0.00	0.00	0.00	0.00	0.00
CD180 molecule	Cd180	100774952	0.00	0.00	0.00	0.00	0.00	0.00	0.00	0.00	0.00	0.00	0.00	0.00
CD19 molecule	Cd19	100765953	0.00	0.04	0.00	0.00	0.00	0.00	0.00	1.83	0.00	1.16	1.03	0.00
CD2 molecule	Cd2	100769473	0.06	0.00	0.00	0.00	0.00	0.00	2.28	0.00	0.00	0.00	0.00	0.00
CD209 molecule	Cd209	100770076	0.00	0.00	0.00	0.00	0.00	0.00	0.00	0.00	0.00	0.00	0.00	0.00
CD22 molecule	Cd22	100752625	0.00	0.00	0.00	0.00	0.00	0.00	0.00	0.00	0.00	0.00	0.00	0.00
CD226 molecule	Cd226	100762155	0.00	0.00	0.00	0.00	0.00	0.00	0.00	0.00	0.00	0.00	0.00	0.00
CD24 molecule	Cd24	100767726	0.57	0.17	1.20	1.18	0.00	0.37	5.32	5.49	14.91	6.95	3.10	4.67
CD244 molecule, natural killer cell receptor 2B4	Cd244	100753316	0.00	0.00	0.00	0.00	0.00	0.00	0.00	0.00	0.00	0.00	0.00	0.00
CD247 molecule	Cd247	100771291	0.00	0.00	0.00	0.00	0.00	0.00	0.00	0.00	0.00	0.00	0.00	0.00
CD248 molecule, endosialin	Cd248	100768169	0.00	0.05	0.02	0.00	0.00	0.00	0.00	2.75	1.24	0.00	0.00	0.00
CD27 molecule	Cd27	100751906	0.02	0.07	0.06	0.06	0.03	0.05	4.56	5.49	6.21	6.95	6.21	7.48
CD274 molecule	Cd274	100759180	0.01	0.03	0.07	0.02	0.02	0.02	1.52	2.75	3.73	1.16	1.03	0.93
CD276 molecule	Cd276	100754397	17.43	17.51	24.06	22.09	17.87	20.83	1348.18	1336.86	1839.53	1673.48	1344.45	1578.66
CD28 molecule	Cd28	100769830	1.23	1.03	0.73	1.09	2.18	2.06	64.56	49.41	32.29	54.47	106.60	105.62
CD2-associated protein	Cd2ap	100770360	39.98	42.16	34.03	38.57	39.31	36.17	2829.28	2991.24	2450.64	2735.05	2764.45	2510.52
CD2 (cytoplasmic tail) binding protein 2	Cd2bp2	100774263	61.46	58.66	69.43	67.90	65.57	72.06	5184.61	4969.55	5860.17	5681.03	5483.36	6046.38
CD300a molecule	Cd300a	100750625	0.00	0.00	0.00	0.00	0.00	0.00	0.00	0.00	0.00	0.00	0.00	0.00
CD300e molecule	Cd300e	100759375	0.00	0.00	0.00	0.00	0.00	0.00	0.00	0.00	0.00	0.00	0.00	0.00
CD300 molecule-like family member b	Cd300lb	100750336	0.00	0.00	0.00	0.00	0.00	0.00	0.00	0.00	0.00	0.00	0.00	0.00
CD300 molecule-like family member f	Cd300lf	100759092	0.03	0.00	0.11	0.00	0.05	0.00	0.76	0.00	2.48	0.00	1.03	0.00
CD300 molecule-like family member g	Cd300lg	100761368	0.00	0.00	0.00	0.00	0.00	0.00	0.00	0.00	0.00	0.00	0.00	0.00
CD320 molecule	Cd320	100756475	17.33	16.00	21.75	21.05	17.85	17.40	555.98	520.65	674.45	654.79	538.19	521.55
CD33 molecule	Cd33	100753674	0.00	0.00	0.00	0.00	0.00	0.00	0.00	0.00	0.00	0.00	0.00	0.00
CD34 molecule	Cd34	100771922	0.00	0.00	0.00	0.00	0.00	0.00	0.00	0.00	0.00	0.00	0.00	0.00
CD36 molecule (thrombospondin receptor)	Cd36	100765055	1.31	1.11	1.74	2.31	1.66	1.48	100.26	86.01	135.39	175.00	129.37	118.70

CD37 molecule	Cd37	100771511	0.00	0.04	0.00	0.05	0.00	0.04	0.00	0.92	1.24	1.16	0.00	2.80
CD38 molecule	Cd38	100773187	0.00	0.00	0.00	0.00	0.00	0.00	0.00	0.00	0.00	0.00	0.00	0.00
CD3d molecule, delta (CD3-TCR complex)	Cd3d	100756906	0.00	0.00	0.00	0.00	0.00	0.00	0.76	0.00	0.00	0.00	0.00	0.00
CD3e molecule, epsilon (CD3-TCR complex)	Cd3e	100757488	0.00	0.00	0.00	0.00	0.00	0.00	0.00	0.00	0.00	0.00	0.00	0.00
CD3e molecule, epsilon associated protein	Cd3eap	100755473	80.58	84.26	92.08	79.82	61.57	60.00	2968.28	3110.20	3364.81	2892.66	2263.52	2205.82
CD3g molecule, gamma (CD3-TCR complex)	Cd3g	100757196	0.10	0.00	0.00	0.00	0.00	0.00	0.76	0.00	0.00	0.00	0.00	0.00
CD4 molecule	Cd4	100766761	0.00	0.00	0.00	0.00	0.00	0.03	0.00	1.83	0.00	0.00	2.07	5.61
CD40 molecule, TNF receptor superfamily member 5	Cd40	100770242	0.04	0.00	0.00	0.00	0.00	0.00	0.76	0.00	0.00	0.00	0.00	0.00
CD40 ligand	Cd40lg	100763089	0.00	0.00	0.00	0.00	0.00	0.00	0.00	0.00	0.00	0.00	0.00	0.00
CD44 molecule (Indian blood group)	Cd44	100750640	212.89	218.10	162.46	176.13	173.68	171.07	24853.64	25453.49	18842.46	20328.61	19897.61	19779.48
CD46 molecule, complement regulatory protein	Cd46	100772491	0.34	0.19	0.28	0.36	0.43	0.48	36.46	23.79	26.08	39.40	50.71	53.28
CD47 molecule	Cd47	100770433	8.93	9.01	5.37	5.76	6.29	6.20	1227.42	1307.58	780.03	829.79	905.61	908.50
CD48 molecule	Cd48	100753914	0.00	0.00	0.00	0.00	0.00	0.00	0.00	0.00	0.00	0.00	0.00	0.00
CD5 molecule	Cd5	100764231	0.00	0.00	0.00	0.00	0.00	0.00	0.00	0.00	0.00	0.00	0.00	0.00
CD53 molecule	Cd53	100772300	0.02	0.03	0.00	0.00	0.00	0.00	2.28	0.92	0.00	0.00	0.00	0.00
CD55 molecule, decay accelerating factor for complement (Cromer blood group)	Cd55	100772131	0.02	0.01	0.05	0.00	0.01	0.10	1.52	0.92	2.48	0.00	1.03	4.67
CD59 molecule, complement regulatory protein	Cd59	100772910	20.74	21.11	18.05	18.13	21.19	22.51	432.18	425.49	353.99	345.36	410.89	434.62
CD5 molecule-like	Cd5l	100759713	0.00	0.00	0.00	0.00	0.00	0.00	0.00	0.00	0.00	0.00	0.00	0.00
CD6 molecule	Cd6	100751977	0.00	0.00	0.00	0.00	0.00	0.00	0.00	0.00	0.00	0.00	0.00	0.00
CD63 molecule	Cd63	100772167	593.08	567.28	684.11	664.66	611.12	653.35	10545.44	9824.72	11709.15	10786.07	9623.30	10419.70
CD68 molecule	Cd68	100762586	1.85	2.02	1.71	1.97	2.16	2.49	156.47	165.62	157.75	179.63	202.86	221.52
CD7 molecule	Cd7	100751416	0.00	0.00	0.00	0.00	0.00	0.00	0.76	0.00	0.00	0.00	0.00	0.00
CD70 molecule	Cd70	100750972	0.00	0.00	0.00	0.00	0.00	0.00	0.00	0.00	0.00	0.00	0.00	0.00
CD72 molecule	Cd72	100756547	0.12	0.17	0.20	0.09	0.14	0.10	8.35	7.32	8.69	12.75	12.42	12.15
CD74 molecule, major histocompatibility complex, class II invariant chain	Cd74	100766126	0.06	0.07	0.03	0.03	0.06	0.02	6.84	5.49	3.73	2.32	5.17	6.54
CD79a molecule, immunoglobulin-associated alpha	Cd79a	100759072	0.00	0.00	0.00	0.00	0.00	0.00	0.00	0.92	0.00	0.00	0.00	1.87
CD79b molecule, immunoglobulin-associated beta	Cd79b	100770248	0.00	0.06	0.00	0.00	0.28	0.13	0.00	1.83	0.00	0.00	19.66	14.02
CD80 molecule	Cd80	100760030	0.12	0.13	0.06	0.14	0.12	0.18	8.35	5.49	3.73	6.95	5.17	10.28
CD81 molecule	Cd81	100759632	83.10	78.49	47.59	44.92	14.54	16.53	3323.74	3191.64	1951.32	1829.93	598.22	671.09
CD82 molecule	Cd82	100772417	0.51	0.61	0.28	0.16	0.37	0.63	25.82	33.86	14.91	13.91	17.59	35.52
CD83 molecule	Cd83	100769362	0.00	0.00	0.00	0.00	0.00	0.00	0.00	0.00	0.00	0.00	0.00	0.00
CD84 molecule	Cd84	100760770	0.00	0.00	0.00	0.00	0.00	0.00	0.00	0.92	0.00	0.00	1.03	0.93
CD86 molecule	Cd86	100769382	0.00	0.00	0.00	0.00	0.00	0.00	0.00	0.00	0.00	0.00	0.00	0.00
CD8a molecule	Cd8a	100766512	0.00	0.00	0.00	0.00	0.00	0.00	0.00	0.92	0.00	0.00	0.00	0.00
CD8b molecule	Cd8b	100769972	0.00	0.00	0.00	0.00	0.00	0.00	0.00	0.00	0.00	0.00	0.00	0.00
CD9 molecule	Cd9	100764427	0.64	0.77	2.03	2.50	2.51	2.90	16.71	25.62	54.65	71.85	73.48	85.05
CD93 molecule	Cd93	100764788	0.00	0.00	0.00	0.00	0.00	0.00	0.00	0.00	0.00	0.00	0.00	0.00

CD96 molecule	Cd96	100769835	0.01	0.00	0.06	0.06	0.09	0.02	10.63	9.15	9.94	13.91	14.49	15.89
CD97 molecule	Cd97	100761362	4.93	5.48	3.99	4.56	3.42	3.60	504.33	569.15	423.55	465.89	357.07	368.26
CD99 molecule-like 2	Cd99l2	100770391	12.36	13.09	11.32	12.89	14.07	14.93	439.01	441.05	404.92	439.23	505.07	525.28
cytidine deaminase	Cda	100754972	2.20	2.48	1.36	1.28	1.11	1.78	108.61	112.55	63.35	73.01	69.34	80.38
cytidine and dCMP deaminase domain containing 1	Cdadc1	100763867	3.51	3.57	4.20	4.76	5.03	4.99	243.81	247.06	281.95	297.84	310.50	317.79
codanin 1	Cdan1	100756905	3.14	3.11	3.36	2.99	2.84	2.61	380.53	376.08	401.19	380.13	350.86	333.68
cell division cycle 123	Cdc123	100753983	40.62	41.83	40.49	46.52	48.77	50.38	1713.52	1783.40	1715.32	1921.49	2044.10	2084.31
cell division cycle 14A	Cdc14a	100772604	7.04	7.77	6.74	7.88	8.60	8.57	378.25	408.10	371.38	424.16	459.53	448.64
cell division cycle 14B	Cdc14b	100759761	5.71	5.10	5.01	5.86	6.61	7.22	613.71	550.85	522.92	628.13	682.06	738.39
cell division cycle 16	Cdc16	100759495	109.71	109.42	104.91	111.17	122.18	124.16	7367.53	7438.31	7124.61	7454.17	8224.00	8199.86
cell division cycle 20	Cdc20	100767761	107.41	101.03	116.62	118.81	100.02	111.23	5770.98	5507.59	6339.61	6287.14	5358.12	5820.19
cell division cycle 20B	Cdc20b	100751686	0.00	0.00	0.00	0.00	0.00	0.00	0.00	0.00	0.00	0.00	0.00	0.00
cell division cycle 23	Cdc23	100752608	31.42	32.50	39.95	38.02	41.68	43.76	2531.54	2634.38	3218.25	3024.78	3322.31	3509.68
cell division cycle 25A	Cdc25a	100767961	28.50	28.18	31.17	32.39	31.42	31.86	2830.80	2842.09	3149.93	3258.88	3143.25	3196.57
cell division cycle 25B	Cdc25b	100765509	18.60	19.41	22.97	21.77	17.63	17.90	1778.08	1868.50	2218.37	2112.71	1731.53	1724.47
cell division cycle 25C	Cdc25c	100773417	3.51	3.83	4.11	4.04	4.40	4.78	250.65	273.60	280.71	290.89	337.41	359.85
cell division cycle 26	Cdc26	100753813	41.58	41.04	40.24	43.84	48.86	47.87	1927.71	1886.80	1849.47	2003.77	2227.29	2208.62
cell division cycle 27	Cdc27	100770539	28.07	28.00	28.58	32.46	34.81	32.03	4273.93	4355.56	4460.33	5028.55	5404.70	4908.89
cell division cycle 34	Cdc34	100750683	63.11	63.40	57.25	43.55	24.09	28.55	2001.39	2034.12	1822.14	1380.27	773.13	882.33
cell division cycle 37	Cdc37	100760178	174.81	168.92	180.35	136.29	101.64	110.17	8720.27	8475.04	9013.82	6785.48	5125.25	5548.20
cell division cycle 37-like 1	Cdc37l1	100761473	18.16	18.83	18.80	21.24	20.76	20.26	1377.04	1422.88	1420.95	1583.08	1536.95	1529.12
cell division cycle 40	Cdc40	100772994	20.37	21.67	22.64	22.29	24.03	22.10	1907.96	2034.12	2131.42	2045.49	2210.73	2059.08
cell division cycle 42	Cdc42	100765433	241.85	261.41	268.36	274.02	292.99	287.28	7455.64	7991.90	8335.64	8343.06	8990.93	8950.40
CDC42 binding protein kinase alpha (DMPK-like)	Cdc42bpa	100750503	25.62	24.40	28.27	31.40	32.01	33.78	3490.84	3248.37	3757.31	4182.54	4145.12	4336.87
CDC42 binding protein kinase beta (DMPK-like)	Cdc42bpb	100773684	32.67	32.12	21.53	19.97	17.93	18.67	6094.54	6152.69	4141.12	3769.97	3442.36	3549.88
CDC42 binding protein kinase gamma (DMPK-like)	Cdc42bpg	100758107	0.02	0.02	0.00	0.01	0.00	0.01	7.60	9.15	3.73	4.64	2.07	7.48
CDC42 effector protein (Rho GTPase binding) 1	Cdc42ep1	100751388	67.48	62.42	53.41	42.88	30.38	34.16	4602.81	4255.82	3672.85	2935.54	2101.02	2364.71
CDC42 effector protein (Rho GTPase binding) 2	Cdc42ep2	100768016	1.08	1.11	0.89	0.46	0.70	0.72	45.57	44.84	36.02	20.86	28.98	31.78
CDC42 effector protein (Rho GTPase binding) 3	Cdc42ep3	100755114	52.48	53.17	57.40	56.54	69.43	68.11	2589.27	2626.15	2870.46	2783.73	3387.51	3374.16
CDC42 effector protein (Rho GTPase binding) 4	Cdc42ep4	100752272	5.13	4.96	5.47	5.85	4.60	5.12	420.78	416.34	448.39	479.79	362.25	419.67
CDC42 small effector 1	Cdc42se1	100767648	26.41	26.67	23.28	24.42	22.67	23.26	2075.06	2109.15	1858.16	1912.22	1776.04	1833.82
CDC42 small effector 2	Cdc42se2	100770481	1.05	0.71	0.78	1.14	0.25	0.60	15.19	10.07	18.63	11.59	4.14	7.48
cell division cycle 45	Cdc45	100689362	27.18	28.34	31.21	33.06	36.06	37.23	1519.08	1608.63	1784.88	1828.78	1995.45	2080.57
cell division cycle 5-like	Cdc5l	100756825	49.19	48.59	46.59	51.61	53.41	51.11	4909.66	4929.29	4811.84	5168.78	5391.24	5141.62

cell division cycle 6	Cdc6	100689243	11.44	12.41	11.95	12.48	13.29	13.31	800.55	862.88	866.98	859.92	866.28	898.22
cell division cycle 7	Cdc7	100689341	19.99	19.83	22.60	24.02	25.19	24.18	1652.76	1636.99	1866.86	1991.02	2098.95	1997.39
cell division cycle 73	Cdc73	100767294	20.40	20.68	18.19	20.92	21.83	20.46	2390.27	2460.53	2114.03	2407.08	2511.91	2353.50
cell division cycle associated 2	Cdca2	100775015	21.44	21.47	24.90	28.99	27.51	27.04	2267.98	2328.76	2677.94	3082.73	2911.42	2899.35
cell division cycle associated 3	Cdca3	100757076	75.40	80.26	84.06	85.63	90.54	88.50	2216.34	2373.60	2512.74	2437.21	2560.56	2503.05
cell division cycle associated 4	Cdca4	100770517	51.40	49.22	48.74	42.05	38.50	41.36	3116.39	2983.01	2974.80	2547.31	2324.58	2516.13
cell division cycle associated 5	Cdca5	103159496	26.72	26.53	33.72	35.13	36.02	39.41	1436.29	1422.88	1809.72	1868.18	1938.53	2131.98
cell division cycle associated 7	Cdca7	100774637	66.43	67.80	76.37	74.27	74.28	75.88	4924.09	5067.46	5714.84	5488.64	5422.29	5570.63
cell division cycle associated 7-like	Cdca7l	100770155	0.00	0.00	0.00	0.00	0.00	0.00	0.00	0.00	0.00	0.00	0.00	0.00
cell division cycle associated 8	Cdca8	100753031	49.05	45.53	41.27	39.38	37.71	41.91	2362.93	2240.00	2028.33	1908.74	1834.00	1987.11
CUB domain containing protein 1	Cdcp1	100760890	0.01	0.00	0.00	0.00	0.00	0.00	0.76	0.00	0.00	0.00	0.00	0.00
CUB domain containing protein 2	Cdcp2	100770767	0.00	0.00	0.00	0.00	0.00	0.00	0.00	0.00	0.00	0.00	0.00	0.00
cadherin 1, type 1, E-cadherin (epithelial)	Cdh1	100758185	0.44	0.59	0.65	0.81	0.99	1.09	54.69	71.37	83.22	101.98	122.13	130.85
cadherin 10, type 2 (T2-cadherin)	Cdh10	100768913	0.00	0.00	0.00	0.00	0.00	0.00	0.00	0.00	0.00	0.00	0.00	0.00
cadherin 11, type 2, OB-cadherin (osteoblast)	Cdh11	100767551	0.00	0.00	0.00	0.00	0.00	0.00	0.00	0.00	0.00	0.00	0.00	0.00
cadherin 12, type 2 (N-cadherin 2)	Cdh12	100756950	0.01	0.00	0.00	0.00	0.00	0.00	0.76	0.00	0.00	0.00	2.07	0.00
cadherin 13	Cdh13	100751312	0.43	0.54	0.04	0.01	0.00	0.03	36.46	53.07	3.73	1.16	0.00	2.80
cadherin 15, type 1, M-cadherin (myotubule)	Cdh15	100764305	0.00	0.00	0.00	0.00	0.00	0.00	0.00	0.00	0.00	0.00	0.00	0.00
cadherin 16, KSP-cadherin	Cdh16	100761107	0.00	0.00	0.00	0.00	0.00	0.00	0.00	0.00	0.00	0.00	0.00	0.00
cadherin 17, LI cadherin (liver-intestine)	Cdh17	100761958	0.00	0.03	0.00	0.01	0.01	0.00	1.52	2.75	0.00	1.16	1.03	0.00
cadherin 18, type 2	Cdh18	100751447	0.00	0.01	0.00	0.00	0.00	0.01	0.00	0.92	0.00	0.00	0.00	0.93
cadherin 19, type 2	Cdh19	100765140	0.00	0.00	0.00	0.00	0.00	0.00	0.00	0.00	0.00	0.00	0.00	0.00
cadherin 2, type 1, N-cadherin (neuronal)	Cdh2	100689204	0.06	0.00	0.02	0.00	0.02	0.03	4.56	0.00	1.24	0.00	2.07	3.74
cadherin 20, type 2	Cdh20	100756854	0.00	0.00	0.00	0.00	0.00	0.00	0.00	0.00	0.00	0.00	0.00	0.00
cadherin 22, type 2	Cdh22	100767839	0.00	0.00	0.00	0.00	0.00	0.00	0.00	0.00	0.00	0.00	0.00	0.00
cadherin-related 23	Cdh23	100760032	0.00	0.00	0.00	0.01	0.00	0.01	0.00	0.92	1.24	2.32	0.00	1.87
cadherin 24, type 2	Cdh24	100766231	0.40	0.64	0.71	0.30	0.53	0.48	29.62	39.35	43.47	18.54	36.22	29.91
cadherin 26	Cdh26	100770778	0.00	0.00	0.00	0.00	0.00	0.00	0.00	0.00	0.00	0.00	0.00	0.00
cadherin 3, type 1, P-cadherin (placental)	Cdh3	100758473	0.00	0.00	0.00	0.00	0.00	0.00	0.00	0.00	0.00	0.00	0.00	0.00
cadherin 5, type 2 (vascular endothelium)	Cdh5	100767079	0.02	0.00	0.00	0.02	0.00	0.00	3.04	0.00	0.00	1.16	0.00	0.00
cadherin 6, type 2, K-cadherin (fetal kidney)	Cdh6	100767269	0.00	0.00	0.00	0.00	0.00	0.00	0.00	0.00	0.00	0.00	0.00	0.00
cadherin 7, type 2	Cdh7	100764852	0.00	0.01	0.00	0.00	0.00	0.00	0.00	0.92	0.00	0.00	0.00	0.00
cadherin 8, type 2	Cdh8	100758353	0.00	0.00	0.00	0.00	0.00	0.00	0.00	0.00	0.00	0.00	0.00	0.00
cadherin 9, type 2 (T1-cadherin)	Cdh9	100758572	1.40	1.37	0.76	1.11	1.05	1.21	132.16	132.68	78.25	105.46	105.57	119.64
cadherin-related family member 1	Cdhr1	100755383	0.00	0.00	0.00	0.00	0.00	0.00	0.00	0.00	0.00	0.00	0.00	0.00
cadherin-related family member 2	Cdhr2	100774943	0.01	0.02	0.00	0.00	0.01	0.00	2.28	2.75	0.00	0.00	1.03	0.00
cadherin-related family member 3	Cdhr3	100760935	0.03	0.03	0.04	0.02	0.05	0.08	3.80	4.58	2.48	2.32	3.10	6.54
cadherin-related family member 4	Cdhr4	100759988	0.00	0.00	0.00	0.00	0.00	0.00	0.76	1.83	2.48	2.32	0.00	0.00
cadherin-related family member 5	Cdhr5	100756177	0.00	0.00	0.00	0.00	0.00	0.00	6.84	11.90	12.42	9.27	8.28	8.41
cell death-inducing p53 target 1	Cdip1	100773437	19.05	17.57	20.72	20.85	22.07	22.20	1381.60	1281.05	1531.49	1529.77	1581.46	1608.57

CDP-diacylglycerol--inositol 3-phosphatidyltransferase	Cdipt	100750801	46.62	45.29	48.10	39.74	33.59	34.76	2495.85	2454.12	2586.02	2131.25	1806.05	1880.56
cyclin-dependent kinase 1	Cdk1	100766716	87.14	87.74	94.18	101.51	105.44	104.89	3178.67	3220.00	3445.55	3640.17	3835.66	3738.68
cyclin-dependent kinase 10	Cdk10	100762088	22.48	20.50	28.83	30.41	27.29	29.15	1214.50	1144.71	1642.04	1670.00	1504.87	1587.07
cyclin-dependent kinase 12	Cdk12	100758448	32.60	33.01	24.65	26.21	25.57	24.55	4769.91	4803.93	3592.12	3840.66	3692.83	3620.91
cyclin-dependent kinase 13	Cdk13	100772207	14.70	14.17	15.87	16.69	16.77	15.92	2288.49	2236.34	2497.84	2597.14	2619.55	2503.98
cyclin-dependent kinase 14	Cdk14	100769772	33.18	34.86	44.07	52.10	59.99	57.20	4210.13	4455.30	5640.32	6502.70	7474.67	7124.99
cyclin-dependent kinase 15	Cdk15	100763928	0.15	0.16	0.16	0.06	0.02	0.08	22.03	21.05	23.60	9.27	7.24	13.09
cyclin-dependent kinase 16	Cdk16	100769331	78.01	76.33	88.79	86.11	73.19	79.65	4149.36	4051.77	4708.75	4525.58	3841.87	4178.91
cyclin-dependent kinase 17	Cdk17	100753201	26.03	25.38	22.29	23.63	25.75	24.52	2542.18	2498.04	2207.19	2339.86	2522.26	2472.20
cyclin-dependent kinase 18	Cdk18	100755170	0.00	0.00	0.00	0.00	0.00	0.00	0.00	0.92	0.00	0.00	0.00	0.00
cyclin-dependent kinase 19	Cdk19	100754073	13.74	14.51	16.06	18.60	20.55	19.65	2228.49	2376.34	2627.01	3020.15	3316.10	3192.83
cyclin-dependent kinase 2	Cdk2	100689039	22.01	21.28	20.56	23.39	25.32	24.96	918.28	925.10	890.58	1001.31	1016.36	1050.57
cyclin-dependent kinase 20	Cdk20	100758610	6.23	5.73	8.22	5.56	4.22	4.99	374.45	349.54	488.14	345.36	253.57	303.77
cyclin-dependent kinase 2 associated protein 1	Cdk2ap1	100773827	146.17	148.58	158.94	152.66	155.44	162.93	3606.29	3643.66	3908.85	3670.30	3716.64	3923.74
cyclin-dependent kinase 2 associated protein 2	Cdk2ap2	100758502	22.85	19.72	21.43	18.33	14.79	16.35	513.45	446.54	479.45	405.62	330.16	366.39
cyclin-dependent kinase 3	Cdk3	100759733	0.21	0.15	0.23	0.29	0.26	0.23	7.60	5.49	9.94	11.59	9.31	12.15
cyclin-dependent kinase 4	Cdk4	100760122	288.84	278.87	293.48	269.32	244.64	261.09	10954.07	10765.37	11417.26	10315.55	9447.36	9996.29
cyclin-dependent kinase 5	Cdk5	100762907	20.97	18.86	25.84	22.52	19.74	21.36	717.76	678.95	927.84	793.86	699.65	789.80
cyclin-dependent kinase 5, regulatory subunit 1 (p35)	Cdk5r1	100773660	0.56	0.54	0.32	0.46	0.40	0.48	56.97	53.07	33.54	44.04	40.36	48.60
cyclin-dependent kinase 5, regulatory subunit 2 (p39)	Cdk5r2	100750997	0.00	0.00	0.00	0.00	0.00	0.00	0.00	0.00	0.00	0.00	0.00	0.00
CDK5 regulatory subunit associated protein 1	Cdk5rap1	100773409	16.24	16.33	14.60	17.44	16.54	15.83	952.46	961.70	874.43	1008.26	957.36	915.04
CDK5 regulatory subunit associated protein 2	Cdk5rap2	100764684	17.37	16.99	18.31	22.55	24.57	23.88	2915.87	2931.77	3127.57	3751.42	4105.79	4006.93
CDK5 regulatory subunit associated protein 3	Cdk5rap3	100752708	50.90	50.32	36.23	35.27	29.66	36.00	3022.97	3017.78	2173.65	2088.37	1770.86	2117.03
cyclin-dependent kinase 6	Cdk6	100764515	81.67	82.27	83.24	93.37	108.12	120.34	6168.98	6267.06	6411.65	7069.41	8263.33	9149.48
cyclin-dependent kinase 7	Cdk7	100769780	21.90	21.65	19.92	22.42	21.64	21.33	1010.95	1012.94	906.72	1046.50	1019.46	1004.77
cyclin-dependent kinase 8	Cdk8	100759285	29.16	30.55	28.31	29.98	28.74	30.59	2265.71	2395.56	2237.00	2376.94	2251.10	2352.56
cyclin-dependent kinase 9	Cdk9	100757719	48.03	43.10	47.01	45.47	38.18	36.01	2306.72	2095.43	2304.07	2159.07	1824.68	1753.44
CDK5 regulatory subunit associated protein 1-like 1	Cdkal1	100758711	42.94	41.51	42.77	44.19	48.25	48.87	1386.92	1379.87	1445.79	1466.03	1607.33	1591.74
cyclin-dependent kinase-like 1 (CDC2-related kinase)	Cdkl1	100767891	0.16	0.17	0.16	0.13	0.19	0.17	15.95	13.73	13.66	12.75	21.73	17.76
cyclin-dependent kinase-like 2 (CDC2-related kinase)	Cdkl2	100753865	4.07	3.84	2.83	4.04	5.74	4.94	470.15	469.41	347.78	483.27	688.27	579.50
cyclin-dependent kinase-like 3	Cdkl3	100757962	0.32	0.36	0.42	0.26	0.33	0.38	38.74	46.67	50.93	40.56	41.40	44.86
cyclin-dependent kinase-like 4	Cdkl4	100756171	0.00	0.00	0.00	0.03	0.00	0.00	0.00	0.00	1.24	1.16	0.00	0.00
cyclin-dependent kinase-like 5	Cdkl5	100751248	0.17	0.12	0.00	0.00	0.00	0.00	15.19	9.15	0.00	0.00	0.00	0.00
cyclin-dependent kinase inhibitor 1A (p21, Cip1)	Cdkn1a	100689175	36.39	40.32	15.53	13.23	12.96	16.13	1807.70	2000.26	772.58	653.63	635.48	792.60
cyclin-dependent kinase inhibitor 1B (p27, Kip1)	Cdkn1b	100689401	43.91	45.28	55.76	41.95	27.34	29.99	1111.97	1108.11	1471.87	1178.62	970.82	950.56
cyclin-dependent kinase inhibitor 1C (p57, Kip2)	Cdkn1c	103164177	0.00	0.00	0.00	0.00	0.00	0.00	0.00	0.00	0.00	0.00	0.00	0.00

CDKN2A interacting protein	Cdkn2aip	100755619	26.75	28.65	28.61	29.61	35.28	34.12	2643.96	2861.31	2864.25	2970.31	3481.69	3395.65
CDKN2A interacting protein N-terminal like	Cdkn2aipnl	103159798	29.42	30.86	34.08	35.57	42.72	42.87	1278.30	1350.59	1488.02	1539.05	1847.45	1866.54
cyclin-dependent kinase inhibitor 2C (p18, inhibits CDK4)	Cdkn2c	100774733	11.26	10.99	12.03	12.28	14.23	12.83	361.54	357.78	386.29	388.24	445.04	420.60
cyclin-dependent kinase inhibitor 2D (p19, inhibits CDK4)	Cdkn2d	100758546	8.10	8.32	7.72	7.97	7.20	8.04	219.51	229.67	207.43	206.29	189.40	211.24
cyclin-dependent kinase inhibitor 3	Cdkn3	100752216	118.11	123.32	86.67	94.20	107.14	101.26	2555.85	2690.20	1902.88	1992.18	2270.76	2131.05
cerebral dopamine neurotrophic factor	Cdnf	100751621	3.61	2.98	20.65	22.94	24.22	24.61	104.06	96.08	686.87	762.57	869.39	806.62
cysteine dioxygenase type 1	Cdo1	100760315	0.16	0.10	0.22	0.25	0.19	0.17	6.08	3.66	6.21	10.43	5.17	5.61
cell adhesion associated, oncogene regulated	Cdon	100759140	2.21	2.13	2.04	2.23	3.10	3.01	476.23	479.48	453.36	501.81	675.85	655.20
cysteine-rich, DPF motif domain containing 1	Cdpf1	100759562	5.76	6.55	4.66	4.36	3.85	3.66	390.40	409.94	326.67	294.37	267.03	263.58
cerebellar degeneration-related protein 1, 34kDa	Cdr1	100769322	0.00	0.00	0.00	0.00	0.00	0.00	0.00	0.00	0.00	0.00	0.00	0.00
cerebellar degeneration-related protein 2, 62kDa	Cdr2	100765677	10.49	10.02	10.21	10.36	10.01	9.73	615.23	588.37	612.35	603.80	578.56	573.89
cerebellar degeneration-related protein 2-like	Cdr2l	100758231	8.89	9.22	10.65	9.36	6.70	7.49	695.74	741.18	827.23	740.55	497.83	560.80
CMT1A duplicated region transcript 1	Cdrt1	100761995	0.00	0.00	0.00	0.00	0.00	0.00	0.00	0.00	0.00	1.16	0.00	1.87
CMT1A duplicated region transcript 4	Cdrt4	100762581	0.00	0.00	0.00	0.00	0.00	0.07	0.00	0.00	0.00	0.00	0.00	0.93
CDP-diacylglycerol synthase (phosphatidate cytidylyltransferase) 1	Cds1	100761973	6.82	6.83	5.05	5.75	7.16	6.96	792.96	803.40	587.51	676.81	831.09	826.25
CDP-diacylglycerol synthase (phosphatidate cytidylyltransferase) 2	Cds2	100761349	28.65	29.19	29.83	31.21	33.53	34.39	3022.21	3123.92	3223.22	3336.53	3526.20	3636.80
corneodesmosin	Cdsn	100761666	3.49	3.61	1.92	1.32	1.67	1.58	209.63	227.84	129.18	86.92	108.67	107.49
chromatin licensing and DNA replication factor 1	Cdt1	100689460	14.80	15.02	17.86	14.06	9.86	10.35	784.60	776.86	962.62	756.77	581.66	569.21
CDV3 homolog (mouse)	Cdv3	100767533	169.50	161.39	153.25	164.00	159.70	161.38	12828.62	12318.18	11742.69	12576.60	12143.49	12309.60
caudal type homeobox 1	Cdx1	100764671	0.00	0.00	0.00	0.00	0.00	0.00	0.00	0.00	0.00	0.00	0.00	0.00
caudal type homeobox 2	Cdx2	100762482	0.00	0.00	0.00	0.00	0.00	0.00	0.00	0.00	0.00	0.00	0.00	0.00
caudal type homeobox 4	Cdx4	100764476	0.00	0.00	0.00	0.00	0.00	0.00	0.00	0.00	0.00	0.00	0.00	0.00
chromodomain protein, Y-like	Cdyl	100763740	13.32	13.58	15.16	15.92	14.63	16.01	1258.56	1290.20	1440.82	1504.28	1377.57	1511.36
chromodomain protein, Y-like 2	Cdyl2	100766529	0.00	0.00	0.00	0.00	0.00	0.00	0.00	0.00	0.00	0.00	0.00	0.00
carcinoembryonic antigen-related cell adhesion molecule 16	Ceacam16	100750783	0.00	0.00	0.00	0.00	0.00	0.00	0.76	0.00	0.00	0.00	0.00	0.00
carcinoembryonic antigen-related cell adhesion molecule 19	Ceacam19	100750495	0.00	0.00	0.00	0.00	0.00	0.00	0.00	0.00	0.00	0.00	0.00	0.00
carcinoembryonic antigen-related cell adhesion molecule 21	Ceacam21	100775099	0.00	0.00	0.00	0.00	0.00	0.00	0.00	0.00	0.00	0.00	0.00	0.00
CCAAT/enhancer binding protein (C/EBP), delta	Cebpd	100771036	0.34	0.29	0.10	0.00	0.06	0.07	20.51	20.13	12.42	4.64	5.17	5.61
CCAAT/enhancer binding protein (C/EBP), epsilon	Cebpe	100770450	0.46	0.47	0.34	0.35	0.41	0.26	20.51	21.05	19.87	16.22	18.63	12.15
CCAAT/enhancer binding protein (C/EBP), gamma	Cebpg	100770190	29.98	29.94	28.35	27.15	24.89	24.97	1479.58	1464.05	1430.88	1354.78	1253.37	1274.89
CCAAT/enhancer binding protein (C/EBP), zeta	Cebpz	100756756	66.59	63.02	61.64	73.39	70.46	65.45	6542.67	6192.03	6048.96	7082.16	6886.80	6386.60
cat eye syndrome chromosome region, candidate 2	Cecr2	100757228	0.00	0.00	0.00	0.00	0.00	0.00	0.00	1.83	0.00	0.00	0.00	0.00

cat eye syndrome chromosome region, candidate 5	Cecr5	100757691	11.72	11.14	12.75	11.58	10.35	11.95	598.52	569.15	658.31	629.29	554.75	619.69
cat eye syndrome chromosome region, candidate 6	Cecr6	100757405	0.00	0.00	0.00	0.00	0.00	0.00	0.00	0.00	0.00	0.00	0.00	0.00
carboxyl ester lipase	Cel	100771549	0.03	0.00	0.02	0.02	0.00	0.00	2.28	0.92	1.24	1.16	0.00	0.00
chymotrypsin-like elastase family, member 1	Cela1	100750829	0.23	0.09	0.06	0.00	0.00	0.10	5.32	1.83	1.24	0.00	0.00	1.87
chymotrypsin-like elastase family, member 2A	Cela2a	100753261	0.03	0.00	0.00	0.00	0.00	0.00	0.76	0.92	1.24	2.32	2.07	0.93
CUGBP, Elav-like family member 1	Celf1	100760547	58.84	59.85	54.44	53.33	51.56	54.77	10081.36	10235.57	9213.80	8983.95	8722.86	9216.78
CUGBP, Elav-like family member 2	Celf2	100769634	0.01	0.01	0.01	0.01	0.00	0.00	1.52	0.92	1.24	1.16	0.00	0.00
CUGBP, Elav-like family member 3	Celf3	100752975	0.00	0.00	0.00	0.00	0.00	0.00	0.00	1.83	1.24	1.16	0.00	1.87
CUGBP, Elav-like family member 4	Celf4	100763521	0.00	0.00	0.00	0.00	0.00	0.00	0.00	0.92	0.00	2.32	0.00	0.00
CUGBP, Elav-like family member 5	Celf5	100770452	0.00	0.08	0.09	0.00	0.00	0.03	0.76	5.49	7.45	0.00	1.03	0.93
CUGBP, Elav-like family member 6	Celf6	100755645	0.00	0.00	0.00	0.00	0.00	0.00	0.00	0.00	0.00	0.00	0.00	0.00
cadherin, EGF LAG seven-pass G-type receptor 1	Celsr1	103160949	0.07	0.08	0.06	0.05	0.08	0.08	10.63	12.81	13.66	9.27	13.45	14.95
cadherin, EGF LAG seven-pass G-type receptor 2	Celsr2	100751011	0.04	0.03	0.00	0.01	0.01	0.00	10.63	10.98	3.73	5.79	3.10	1.87
cadherin, EGF LAG seven-pass G-type receptor 3	Celsr3	100766515	0.08	0.05	0.09	0.09	0.09	0.09	39.50	32.94	91.91	71.85	68.31	78.51
cell migration inducing protein, hyaluronan binding	Cemip	100760429	0.01	0.00	0.01	0.01	0.00	0.01	2.28	0.00	3.73	2.32	2.07	2.80
cell cycle exit and neuronal differentiation 1	Cend1	100752544	0.00	0.00	0.00	0.00	0.00	0.00	0.00	0.00	0.00	0.00	0.00	0.00
centromere protein A	Cenpa	100689459	68.95	70.92	77.90	79.16	87.22	81.16	3223.48	3298.70	3594.60	3639.01	3933.98	3716.25
centromere protein B, 80kDa	Cenpb	100689274	19.15	18.70	15.68	12.38	12.13	11.19	1108.93	1050.46	886.85	686.08	633.41	618.75
centromere protein C	Cenpc	100761015	11.19	9.78	9.54	12.25	12.89	10.73	1030.69	923.27	881.88	1102.13	1175.74	968.32
centromere protein E, 312kDa	Cenpe	100771167	22.11	20.43	14.33	19.65	21.88	19.40	5288.67	4992.42	3500.20	4784.02	5259.80	4634.09
centromere protein F, 350/400kDa	Cenpf	100752075	12.24	11.54	10.26	13.16	15.46	13.76	3515.91	3346.28	3008.33	3818.64	4430.78	3985.43
centromere protein H	Cenph	100771123	20.68	20.73	21.07	26.74	28.06	27.19	708.65	727.45	744.01	905.12	969.78	937.47
centromere protein I	Cenpi	100771572	10.95	11.46	10.17	11.49	13.70	14.05	882.58	883.92	827.23	921.34	1065.00	1092.63
centromere protein J	Cenpj	100752517	4.15	4.23	4.10	4.94	6.13	5.77	535.48	549.02	532.86	643.20	785.55	750.54
centromere protein K	Cenpk	100760714	15.78	16.42	18.06	21.64	27.18	22.25	502.06	541.70	597.44	690.72	872.49	715.96
centromere protein L	Cenpl	100774360	16.31	16.75	16.96	17.50	19.86	18.11	796.00	820.79	835.92	862.24	960.47	898.22
centromere protein M	Cenpm	100762853	21.29	20.28	22.08	15.94	12.95	13.91	495.22	482.22	464.54	334.93	254.61	290.68
centromere protein N	Cenpn	100765070	16.19	15.44	18.25	19.59	23.24	22.68	550.67	556.34	617.32	603.80	724.49	672.03
centromere protein O	Cenpo	100755730	15.44	16.95	15.94	19.82	20.28	20.21	634.97	678.95	649.61	799.65	813.50	804.75
centromere protein P	Cenpp	100758284	5.43	5.37	5.12	5.77	6.71	6.06	394.20	379.74	353.99	382.44	484.37	413.12
centromere protein Q	Cenpq	100764991	24.81	25.21	26.91	29.66	33.76	29.09	1028.42	1050.46	1151.41	1266.70	1438.63	1235.63
centromere protein T	Cenpt	100753819	31.97	32.07	35.68	35.31	34.57	35.79	1579.84	1583.92	1779.91	1724.47	1734.64	1774.94
centromere protein U	Cenpu	100753571	9.72	9.34	10.81	12.99	12.79	14.04	386.61	375.16	440.94	501.81	541.30	544.91
centromere protein V	Cenpv	100761123	31.98	30.51	46.57	45.14	34.42	38.40	811.95	796.08	1212.28	1153.13	895.26	971.12
centromere protein W	Cenpw	100761050	83.13	87.43	54.10	53.70	64.96	63.70	3536.41	3690.33	2291.65	2243.67	2682.68	2696.52
centrin-1	Centrin-1	100757090	0.00	0.00	0.00	0.00	0.00	0.00						
centrin-1	Centrin-1	100768500	0.00	0.00	0.00	0.00	0.00	0.00	0.00	0.00	0.00	0.00	0.00	0.00
centrin-4	Centrin-2	100757715	2.88	2.72	1.21	1.85	1.96	1.64	72.92	69.54	32.29	49.83	50.71	42.99

centrosomal protein 112kDa	Cep112	100762658	1.96	2.37	2.68	3.11	2.93	3.01	132.92	146.41	175.13	199.33	200.79	184.13
centrosomal protein 120kDa	Cep120	100774588	8.73	8.22	9.22	10.30	12.42	11.00	1236.53	1162.09	1290.53	1437.06	1721.18	1543.14
centrosomal protein 128kDa	Cep128	100752420	4.29	4.09	4.46	5.21	6.42	5.38	626.62	623.14	638.43	786.91	967.71	805.69
centrosomal protein 131kDa	Cep131	100762484	16.51	16.42	18.11	12.74	8.78	8.71	1900.37	1900.52	2122.73	1455.60	1047.41	1042.16
centrosomal protein 135kDa	Cep135	100689465	19.27	19.55	21.17	21.07	25.38	24.84	1430.97	1497.91	1355.12	1583.08	1709.80	1690.82
centrosomal protein 152kDa	Cep152	100760797	6.99	7.32	9.98	11.85	11.39	11.00	1165.89	1224.31	1700.42	1985.23	1904.37	1840.36
centrosomal protein 162kDa	Cep162	100759950	4.48	4.30	2.20	2.88	2.93	2.96	777.01	765.88	380.08	496.02	496.79	503.79
centrosomal protein 164kDa	Cep164	100762565	9.66	9.83	9.92	9.10	8.22	8.89	1739.34	1767.84	1832.08	1649.14	1451.05	1581.46
centrosomal protein 170kDa	Cep170	100771665	31.94	31.12	41.82	46.72	43.99	39.82	4750.92	4638.30	6311.04	6929.18	6425.20	5911.79
centrosomal protein 170B	Cep170 b	100769745	8.46	8.16	8.00	5.68	3.95	4.19	1637.57	1576.60	1577.45	1135.74	777.27	839.33
centrosomal protein 19kDa	Cep19	100751790	8.73	9.53	9.35	9.30	10.37	10.37	410.91	455.69	452.12	451.98	498.86	492.57
centrosomal protein 192kDa	Cep192	100756846	5.09	4.87	4.77	6.20	6.74	6.36	1228.17	1179.48	1189.92	1521.66	1654.94	1541.27
centrosomal protein 250kDa	Cep250	100762321	3.42	3.19	2.66	2.46	2.49	2.78	818.02	767.71	623.53	585.25	592.01	658.94
centrosomal protein 290kDa	Cep290	100753037	3.20	3.13	2.96	3.68	4.60	4.12	740.55	734.77	689.36	847.17	1054.65	953.36
centrosomal protein 350kDa	Cep350	100754786	4.50	4.30	3.87	5.27	5.51	5.42	1360.33	1311.24	1183.71	1595.83	1653.91	1645.95
centrosomal protein 41kDa	Cep41	100757951	9.64	9.50	9.53	9.64	9.40	8.63	1001.07	1018.43	1022.24	1021.01	1007.04	931.87
centrosomal protein 44kDa	Cep44	100755401	13.43	13.06	16.53	16.84	20.26	18.58	695.74	661.57	794.94	822.83	1010.15	901.96
centrosomal protein 55kDa	Cep55	100752217	28.73	28.69	30.08	33.42	39.52	35.04	1916.32	1897.78	1955.04	2170.66	2527.44	2274.99
centrosomal protein 57kDa	Cep57	100762024	23.95	24.13	21.18	25.03	28.78	25.59	2012.78	2037.78	1840.77	2128.94	2426.01	2190.87
centrosomal protein 57kDa-like 1	Cep57l1	100771063	27.58	25.70	25.19	27.84	29.25	27.78	2078.10	1974.64	1940.14	2098.80	2197.28	2113.29
centrosomal protein 63kDa	Cep63	100771874	22.91	23.20	14.19	14.80	13.83	14.36	1370.21	1428.37	839.65	905.12	831.09	857.09
centrosomal protein 68kDa	Cep68	100754661	12.13	11.72	11.97	11.29	10.14	9.71	792.96	768.63	791.21	741.71	675.85	658.01
centrosomal protein 70kDa	Cep70	100767664	5.64	5.36	2.06	2.66	3.17	2.94	429.14	397.12	162.71	194.70	247.36	222.45
centrosomal protein 72kDa	Cep72	100761899	22.73	22.03	22.26	23.21	22.38	23.98	1582.12	1565.62	1570.00	1642.19	1575.25	1701.10
centrosomal protein 76kDa	Cep76	100760887	3.45	3.67	3.57	3.93	4.58	4.28	328.12	350.46	339.09	366.22	426.41	400.04
centrosomal protein 78kDa	Cep78	100761244	5.05	5.51	7.44	6.87	6.76	6.75	327.36	351.37	471.99	436.91	425.38	426.21
centrosomal protein 83kDa	Cep83	100765559	26.44	26.06	26.68	34.64	30.91	28.09	1995.31	1972.81	2049.44	2630.75	2340.10	2093.66
centrosomal protein 85kDa	Cep85	100768859	19.84	20.72	9.96	10.51	11.25	11.55	2279.38	2393.73	1165.08	1230.77	1307.19	1344.06
centrosomal protein 85kDa-like	Cep85l	100763810	1.31	1.55	0.86	0.83	1.00	0.90	189.88	219.61	121.72	122.85	143.86	128.98
centrosomal protein 89kDa	Cep89	100757884	4.72	4.89	5.91	4.94	4.07	4.16	362.30	377.91	435.97	380.13	316.71	325.27
centrosomal protein 95kDa	Cep95	100773998	10.61	10.14	8.53	9.55	10.64	10.20	892.46	874.77	748.98	850.65	938.73	881.39
centrosomal protein 97kDa	Cep97	100757520	4.98	5.08	5.57	6.98	7.12	6.91	435.98	441.96	498.08	596.84	618.92	588.84
choline/ethanolamine phosphotransferase 1	Cept1	100773070	26.45	24.79	25.15	26.04	29.34	26.89	1536.55	1434.77	1458.21	1497.32	1676.68	1562.77
cerberus 1, DAN family BMP antagonist	Cer1	100764648	0.00	0.00	0.00	0.00	0.00	0.00	0.00	0.00	0.00	0.00	0.00	0.00

cerebral endothelial cell adhesion molecule	Cercam	100765284	6.71	7.03	9.15	8.49	6.33	6.57	503.57	511.50	695.57	636.25	470.92	493.51
ceramide kinase	Cerk	100763715	32.70	31.44	23.89	23.56	22.65	25.67	3992.14	3866.93	2957.41	2852.10	2740.64	3124.60
ceramide kinase-like	Cerkl	100762029	0.33	0.42	0.12	0.11	0.32	0.36	21.27	25.62	13.66	12.75	26.91	33.65
ceramide synthase 1	Cers1	100765511	0.43	0.37	0.47	0.44	0.33	0.44	14.43	15.56	19.87	13.91	11.38	15.89
ceramide synthase 2	Cers2	100765986	33.38	32.72	37.06	37.52	35.85	36.48	1841.88	1826.41	2075.53	2096.49	1989.24	1994.59
ceramide synthase 3	Cers3	100770521	0.00	0.00	0.00	0.00	0.00	0.00	0.00	0.00	0.00	0.00	0.00	0.00
ceramide synthase 4	Cers4	100758196	0.00	0.00	0.00	0.00	0.00	0.00	0.00	0.00	0.00	0.00	0.00	0.00
ceramide synthase 5	Cers5	100774213	25.90	25.95	23.36	23.98	25.43	27.65	1241.09	1249.02	1173.77	1190.21	1229.56	1337.51
ceramide synthase 6	Cers6	100761613	19.31	19.10	22.68	24.11	25.19	24.45	3563.00	3539.35	4256.63	4485.02	4652.26	4556.51
carboxylesterase 1	Ces1	100761459	0.00	0.00	0.00	0.00	0.00	0.00	0.00	0.00	0.00	0.00	0.00	0.00
carboxylesterase 3	Ces3	100757439	0.02	0.02	0.00	0.00	0.00	0.00	0.76	0.92	0.00	0.00	0.00	0.00
carboxylesterase 4A	Ces4a	100764981	0.00	0.00	0.00	0.03	0.00	0.00	0.00	0.00	0.00	1.16	0.00	0.93
carboxylesterase 5A	Ces5a	100761747	0.00	0.00	0.00	0.00	0.00	0.00	0.00	0.00	0.00	0.00	0.00	0.00
centrin, EF-hand protein, 2	Cetn2	100768482	33.55	35.37	33.63	34.19	45.06	41.93	972.21	1056.86	999.88	986.24	1295.80	1190.77
centrin, EF-hand protein, 3	Cetn3	100765724	137.68	146.58	126.44	144.76	170.36	154.96	2964.48	3135.82	2726.38	3003.92	3571.74	3269.47
cholesteryl ester transfer protein, plasma	Cetp	100758852	0.00	0.00	0.00	0.00	0.00	0.00	0.00	0.00	0.00	0.00	0.00	0.00
complement factor B	Cfb	100758781	0.04	0.04	0.00	0.02	0.03	0.03	25.82	25.62	26.08	31.29	34.15	40.19
complement factor D (adipsin)	Cfd	100763302	0.00	0.00	0.00	0.00	0.00	0.00	3.80	2.75	1.24	0.00	0.00	0.93
craniofacial development protein 1	Cfdp1	100768565	130.85	128.26	123.03	137.03	126.02	121.06	4258.74	4209.15	3946.11	4347.11	3974.35	3846.17
complement factor H	Cfh	100768853	19.36	19.86	15.54	16.86	20.18	20.37	448.13	458.43	371.38	397.51	475.06	469.20
complement factor I	Cfi	100767614	0.24	0.19	0.23	0.19	0.26	0.25	29.62	31.11	23.60	20.86	24.84	38.32
cofilin 1 (non-muscle)	Cfl1	100760435	1033.41	1005.76	1014.91	911.97	838.64	938.56	31539.87	30830.22	31075.77	27573.02	25596.25	28642.02
cofilin 2 (muscle)	Cfl2	100757641	109.41	120.70	169.53	166.84	177.63	169.87	3792.38	4175.30	5907.37	5839.80	6225.44	5909.92
CASP8 and FADD-like apoptosis regulator	Cflar	100757136	41.94	42.84	34.43	40.48	50.05	51.04	4254.18	4404.06	3686.51	4214.99	5229.79	5280.88
complement factor properdin	Cfp	100763603	0.08	0.07	0.03	0.05	0.02	0.02	3.80	3.66	1.24	2.32	1.03	0.93
cystic fibrosis transmembrane conductance regulator (ATP-binding cassette sub-family C, member 7)	Cftr	100754506	0.00	0.00	0.00	0.00	0.00	0.00	0.00	0.92	0.00	0.00	0.00	0.00
glycoprotein hormones, alpha polypeptide	Cga	100689452	0.00	0.00	0.08	0.08	0.00	0.00	0.00	0.00	1.24	2.32	0.00	0.00
CGG triplet repeat binding protein 1	Cggbp1	100767237	45.17	47.14	53.62	57.33	62.59	65.41	2036.32	2152.16	2432.01	2577.44	2792.39	2958.23
cingulin	Cgn	100754199	0.22	0.20	0.21	0.15	0.19	0.15	33.42	25.62	28.57	24.34	27.94	28.97
cingulin-like 1	Cgnl1	100771153	0.04	0.03	0.00	0.02	0.03	0.01	6.84	6.41	0.00	4.64	5.17	1.87
cell growth regulator with EF-hand domain 1	Cgref1	100771935	0.00	0.04	0.12	0.00	0.05	0.05	0.76	1.83	2.48	0.00	1.03	0.93
cell growth regulator with ring finger domain 1	Cgrrf1	100751644	16.89	16.21	16.12	17.06	16.23	15.77	896.26	860.13	865.73	920.18	905.61	874.85
cholesterol 25-hydroxylase	Ch25h	100762824	0.06	0.07	0.05	0.00	0.00	0.00	1.52	1.83	2.48	0.00	0.00	0.00
ChaC, cation transport regulator homolog 1 (E. coli)	Chac1	100762174	56.83	51.44	39.13	30.37	18.25	19.87	2050.76	1884.97	1417.22	1083.59	654.11	710.35
ChaC, cation transport regulator homolog 2 (E. coli)	Chac2	100768240	7.58	7.95	8.52	10.67	11.41	9.82	306.85	320.26	371.38	427.64	455.39	384.15
chondroadherin	Chad	100758456	0.04	0.01	0.03	0.06	0.04	0.02	6.08	1.83	2.48	4.64	6.21	4.67
chondroadherin-like	Chadl	100763136	0.00	0.00	0.03	0.02	0.04	0.00	1.52	0.92	3.73	3.48	4.14	2.80
chromatin assembly factor 1, subunit A (p150)	Chaf1a	100756533	27.42	27.79	30.36	27.62	22.55	22.93	2586.99	2662.75	2851.83	2573.96	2156.91	2175.91

chromatin assembly factor 1, subunit B (p60)	Chaf1b	100751472	25.36	25.08	32.80	30.21	25.48	28.63	1402.87	1431.11	1891.70	1729.11	1496.59	1590.81
chromosome alignment maintaining phosphoprotein 1	Champ1	100758920	24.17	23.96	25.57	24.98	23.68	25.79	2507.24	2490.72	2674.21	2593.66	2437.39	2690.91
choline O-acetyltransferase	Chat	100756785	0.00	0.00	0.00	0.00	0.00	0.00	0.00	0.00	0.00	0.00	0.00	0.00
coiled-coil-helix-coiled-coil-helix domain containing 1	Chchd1	100758627	74.01	75.73	89.81	83.35	73.52	69.48	1941.38	1956.34	2281.71	2089.53	1752.23	1666.52
coiled-coil-helix-coiled-coil-helix domain containing 10	Chchd10	100766619	0.06	0.07	0.19	0.09	0.00	0.00	1.52	0.92	3.73	3.48	0.00	0.00
coiled-coil-helix-coiled-coil-helix domain containing 2	Chchd2	100754041	330.25	328.87	321.73	249.31	236.32	238.65	6175.05	6139.87	5989.34	4555.71	4398.69	4450.90
coiled-coil-helix-coiled-coil-helix domain containing 3	Chchd3	100750398	77.08	80.45	89.45	92.25	95.23	98.31	2974.36	3083.66	3423.19	3416.50	3653.50	3682.60
coiled-coil-helix-coiled-coil-helix domain containing 4	Chchd4	100764116	36.06	36.08	35.97	36.65	28.64	30.39	655.48	656.08	698.05	664.06	516.46	572.02
coiled-coil-helix-coiled-coil-helix domain containing 5	Chchd5	100754765	21.08	21.56	23.70	21.67	21.51	22.53	2309.76	2364.45	2620.80	2380.42	2295.60	2427.34
coiled-coil-helix-coiled-coil-helix domain containing 6	Chchd6	100761221	82.85	77.70	75.23	62.18	41.57	47.21	2096.33	1996.60	1865.61	1586.56	1137.45	1282.37
coiled-coil-helix-coiled-coil-helix domain containing 7	Chchd7	100755404	12.88	10.84	16.04	14.48	14.34	16.58	151.15	124.44	185.07	172.68	175.95	200.95
carbonyl reductase 1	Chcr1	100689019	3.00	2.50	3.10	1.98	2.05	2.05	91.90	78.69	78.25	42.88	38.29	42.06
carbonyl reductase 2	Chcr2	100689020	27.93	29.63	31.49	31.68	36.63	34.43	667.63	721.96	760.16	753.30	898.37	851.48
chromodomain helicase DNA binding protein 1	Chd1	100751866	26.31	25.45	24.25	29.07	28.16	25.23	5208.16	5036.34	4849.11	5726.22	5508.20	4956.55
chromodomain helicase DNA binding protein 1-like	Chd1l	100772942	8.67	9.30	11.34	11.74	12.16	10.83	775.49	833.60	996.15	1031.44	1061.90	954.30
chromodomain helicase DNA binding protein 2	Chd2	100756099	18.97	18.24	17.15	19.77	22.60	22.10	5027.39	4933.86	4661.55	5306.69	6039.15	5944.50
chromodomain helicase DNA binding protein 3	Chd3	100755630	17.54	17.58	17.18	16.48	14.82	15.14	3711.11	3801.05	3716.32	3545.14	3130.83	3180.68
chromodomain helicase DNA binding protein 4	Chd4	100754178	62.84	62.95	63.32	64.65	61.03	62.84	12763.30	13003.54	13193.45	13233.71	12449.85	12690.01
chromodomain helicase DNA binding protein 5	Chd5	100766055	8.40	8.54	7.50	6.57	5.81	6.39	2373.56	2411.11	2158.75	1860.07	1661.15	1869.34
chromodomain helicase DNA binding protein 6	Chd6	100770276	10.14	10.07	9.73	10.79	12.03	11.60	3223.48	3192.55	3111.43	3441.99	3819.10	3696.62
chromodomain helicase DNA binding protein 7	Chd7	100755474	2.17	2.27	1.64	1.97	1.52	1.46	655.48	702.75	495.59	623.50	458.50	443.03
chromodomain helicase DNA binding protein 8	Chd8	100750414	25.87	24.86	25.89	28.02	28.13	27.93	6592.04	6365.89	6637.71	7129.68	7137.27	7110.03
chromodomain helicase DNA binding protein 9	Chd9	100769940	3.99	3.68	2.72	3.88	4.06	3.43	1303.37	1234.38	891.82	1286.40	1311.33	1164.60
calponin homology domain containing 2	Chdc2	100762077	0.00	0.00	0.00	0.00	0.00	0.00	0.00	0.92	0.00	0.00	0.00	0.00
choline dehydrogenase	Chdh	100772204	0.00	0.00	0.00	0.00	0.00	0.00	0.00	0.00	0.00	0.00	0.00	0.00
checkpoint kinase 1	Chek1	100751099	33.76	31.72	39.27	41.27	45.74	43.19	2533.82	2451.37	2988.46	3108.22	3441.33	3221.81
checkpoint kinase 2	Chek2	100769280	5.15	5.33	6.55	6.90	8.69	7.73	306.09	319.35	385.05	394.03	494.72	442.10
calcium homeostasis endoplasmic reticulum protein	Cherp	100755648	29.31	27.32	24.66	18.07	12.70	12.32	2719.15	2568.50	2326.43	1733.74	1218.18	1194.51
checkpoint with forkhead and ring finger domains, E3 ubiquitin protein ligase	Chfr	100771287	10.37	10.23	8.25	10.21	10.51	10.99	933.47	959.87	785.00	917.86	951.15	993.55

chromogranin A (parathyroid secretory protein 1)	Chga	100766424	0.00	0.00	0.00	0.00	0.00	0.00	0.00	0.00	0.00	0.00	0.00	0.00
chromogranin B (secretogranin 1)	Chgb	100773273	0.00	0.00	0.00	0.00	0.00	0.02	0.00	0.00	0.00	0.00	0.00	0.93
chitinase 3-like 1 (cartilage glycoprotein-39)	Chi3l1	100760271	0.00	0.00	0.00	0.00	0.00	0.00	0.76	0.00	0.00	0.00	0.00	0.00
chitinase, acidic	Chia	100750633	0.00	0.00	0.00	0.00	0.00	0.00	0.00	0.00	0.00	0.00	0.00	0.00
cysteine-rich hydrophobic domain 1	Chic1	100764176	1.74	1.64	1.41	1.85	2.13	2.13	104.82	101.57	85.70	104.30	131.44	122.44
cysteine-rich hydrophobic domain 2	Chic2	100765671	29.15	31.97	35.03	33.38	37.13	32.47	331.92	344.97	428.52	394.03	429.52	342.09
chitinase domain containing 1	Chid1	100755120	94.56	95.07	93.56	90.34	74.14	90.13	3840.99	3947.45	3896.43	3674.93	3040.79	3610.63
chitinase 1 (chitotriosidase)	Chit1	100750757	0.00	0.00	0.00	0.00	0.00	0.00	0.00	0.00	0.00	0.00	0.00	0.00
	Chka	100767436	29.86	29.28	51.01	49.77	44.50	47.88	907.65	894.90	1563.79	1522.82	1357.90	1447.80
choline kinase beta	Chkb	100770695	5.46	4.88	5.63	6.87	6.27	6.82	390.40	370.59	376.35	461.25	432.62	458.92
cell adhesion molecule L1-like	Chl1	100760694	0.30	0.30	0.16	0.19	0.11	0.10	51.65	51.24	28.57	35.93	18.63	19.63
choroideremia (Rab escort protein 1)	Chm	100763141	4.91	4.99	5.93	7.13	8.36	8.04	706.37	710.07	829.71	997.83	1175.74	1117.86
choroideremia-like (Rab escort protein 2)	Chml	100772631	5.69	5.27	7.26	8.55	9.88	8.53	890.18	835.43	1147.69	1357.10	1557.65	1362.75
charged multivesicular body protein 1A	Chmp1a	100762678	51.30	52.56	57.16	48.16	42.51	46.10	3164.24	3259.35	3573.48	2970.31	2620.59	2866.63
charged multivesicular body protein 1B	Chmp1b	103164052	20.66	21.25	23.15	19.89	21.15	21.34	1162.10	1197.78	1335.24	1145.01	1220.25	1240.31
charged multivesicular body protein 2A	Chmp2a	100767596	12.00	11.58	15.19	14.49	11.54	12.68	186.85	183.01	226.06	187.75	164.56	198.15
charged multivesicular body protein 2B	Chmp2b	100773840	40.57	42.86	47.65	50.29	57.83	56.17	2293.05	2482.49	2741.29	2825.45	3228.12	3165.73
charged multivesicular body protein 3	Chmp3	100770826	37.47	38.16	41.44	41.18	42.14	42.76	2662.18	2724.05	2959.89	2900.78	2980.76	3025.53
charged multivesicular body protein 4B	Chmp4b	100759419	212.68	222.80	233.07	209.44	174.39	177.87	3638.95	3651.90	3676.58	3185.87	2678.54	2651.66
charged multivesicular body protein 4C	Chmp4c	100759934	0.06	0.03	0.00	0.00	0.06	0.03	2.28	0.92	0.00	0.00	2.07	0.93
charged multivesicular body protein 5	Chmp5	100758690	41.46	41.80	45.31	51.52	59.80	51.42	1625.41	1631.50	1736.44	1936.56	2213.84	1911.40
charged multivesicular body protein 6	Chmp6	100753694	50.22	48.99	52.64	49.55	39.56	44.89	2252.03	2217.13	2372.39	2218.17	1776.04	2008.61
charged multivesicular body protein 7	Chmp7	100770771	23.73	22.37	28.24	29.02	29.88	31.08	1643.64	1570.20	1935.17	1995.66	2084.46	2142.26
chimerin 1	Chn1	100755161	0.01	0.00	0.00	0.00	0.00	0.00	0.76	0.00	0.00	0.00	0.00	0.00
chimerin 2	Chn2	100757437	0.00	0.00	0.00	0.00	0.00	0.00	0.00	0.00	0.00	0.00	0.00	0.00
chondrolectin	Chodl	100754785	0.00	0.05	0.00	0.00	0.03	0.03	0.76	1.83	0.00	0.00	1.03	0.93
cysteine and histidine-rich domain (CHORD) containing 1	Chordc1	100768748	30.38	33.39	34.71	36.74	40.06	36.25	2001.39	2230.85	2327.67	2410.55	2635.08	2389.95
calcineurin-like EF-hand protein 1	Chp1	100763935	49.23	49.47	60.22	64.75	71.48	71.76	3544.77	3627.19	4347.30	4692.47	5076.61	5153.77
calcineurin-like EF-hand protein 2	Chp2	100759970	0.00	0.00	0.00	0.00	0.00	0.00	0.00	0.00	0.00	0.00	0.00	0.00
chondroitin polymerizing factor	Chpf	100765856	77.18	69.47	87.29	53.96	31.04	35.85	5252.97	4752.68	6004.25	3679.57	2124.83	2465.66
choline phosphotransferase 1	Chpt1	100750762	43.89	44.69	67.32	72.75	85.12	80.20	1752.26	1775.17	2676.70	2835.88	3353.36	3030.20
chromatin accessibility complex 1	Chrac1	100759753	28.34	28.55	31.26	24.73	22.53	23.13	621.30	646.01	694.33	547.01	492.65	525.28
chordin	Chrd	100774612	0.00	0.01	0.00	0.00	0.00	0.00	0.00	0.92	1.24	0.00	0.00	0.00
chordin-like 1	Chrdl1	100750419	0.15	0.14	0.05	0.03	0.01	0.01	12.91	10.98	4.97	3.48	1.03	0.93
chordin-like 2	Chrdl2	100756377	0.00	0.00	0.00	0.00	0.00	0.00	0.00	0.00	1.24	0.00	0.00	0.00
cholinergic receptor, muscarinic 1	Chrm1	100755522	0.00	0.05	0.00	0.00	0.00	0.00	0.00	3.66	0.00	0.00	1.03	0.00
cholinergic receptor, muscarinic 2	Chrm2	100770997	0.00	0.00	0.00	0.00	0.00	0.00	0.00	0.00	0.00	0.00	0.00	0.00
cholinergic receptor, muscarinic 3	Chrm3	100753955	0.00	0.00	0.00	0.00	0.00	0.00	0.76	0.00	0.00	0.00	0.00	0.00
cholinergic receptor, muscarinic 4	Chrm4	100752496	0.06	0.02	0.05	0.07	0.04	0.08	3.80	0.92	2.48	3.48	2.07	3.74

cholinergic receptor, muscarinic 5	Chrm5	100773423	0.00	0.00	0.00	0.00	0.00	0.00	0.00	0.00	0.00	0.00	0.00	0.00
cholinergic receptor, nicotinic, alpha 1 (muscle)	Chrna1	100755935	0.00	0.03	0.00	0.00	0.00	0.00	0.00	0.92	0.00	0.00	0.00	0.00
cholinergic receptor, nicotinic, alpha 10 (neuronal)	Chrna10	100760224	0.00	0.00	0.00	0.00	0.00	0.00	0.00	1.83	1.24	0.00	0.00	0.00
cholinergic receptor, nicotinic, alpha 2 (neuronal)	Chrna2	100757795	0.00	0.00	0.00	0.00	0.00	0.00	0.00	0.00	0.00	0.00	0.00	0.00
cholinergic receptor, nicotinic, alpha 3 (neuronal)	Chrna3	100763280	0.00	0.00	0.00	0.00	0.00	0.00	0.00	0.00	0.00	0.00	0.00	0.00
cholinergic receptor, nicotinic, alpha 4 (neuronal)	Chrna4	100760001	0.00	0.00	0.00	0.02	0.00	0.00	0.00	0.00	0.00	1.16	0.00	0.00
cholinergic receptor, nicotinic, alpha 5 (neuronal)	Chrna5	100762991	0.00	0.00	0.00	0.00	0.00	0.00	0.00	0.00	0.00	0.00	0.00	0.00
cholinergic receptor, nicotinic, alpha 6 (neuronal)	Chrna6	100769172	0.00	0.00	0.00	0.00	0.03	0.00	0.00	0.00	0.00	0.00	1.03	0.00
cholinergic receptor, nicotinic, alpha 9 (neuronal)	Chrna9	100756671	0.00	0.00	0.00	0.00	0.00	0.00	0.00	0.00	0.00	0.00	0.00	0.00
cholinergic receptor, nicotinic, beta 1 (muscle)	Chrnb1	100758816	2.42	2.44	1.75	1.40	1.81	1.87	157.22	161.05	110.55	90.40	122.13	128.05
cholinergic receptor, nicotinic, beta 2 (neuronal)	Chrnb2	100761068	0.02	0.06	0.12	0.12	0.03	0.09	2.28	6.41	11.18	11.59	5.17	8.41
cholinergic receptor, nicotinic, beta 3 (neuronal)	Chrnb3	100774746	0.03	0.02	0.10	0.02	0.00	0.04	2.28	0.92	4.97	1.16	0.00	1.87
cholinergic receptor, nicotinic, beta 4 (neuronal)	Chrnb4	100763565	0.00	0.00	0.00	0.00	0.00	0.00	0.00	0.00	0.00	0.00	0.00	0.00
cholinergic receptor, nicotinic, delta (muscle)	Chrnd	100763831	0.00	0.00	0.00	0.00	0.00	0.00	0.00	0.00	0.00	0.00	0.00	0.00
cholinergic receptor, nicotinic, epsilon (muscle)	Chrne	100769735	0.00	0.00	0.00	0.03	0.00	0.02	3.04	1.83	1.24	3.48	0.00	2.80
cholinergic receptor, nicotinic, gamma (muscle)	Chrng	100764120	0.09	0.00	0.03	0.00	0.00	0.02	6.08	0.00	2.48	0.00	1.03	4.67
carbohydrate (keratan sulfate Gal-6) sulfotransferase 1	Chst1	100774066	0.00	0.00	0.00	0.00	0.00	0.00	0.00	0.00	0.00	0.00	0.00	0.00
carbohydrate sulfotransferase 10	Chst10	100755464	0.00	0.00	0.05	0.00	0.12	0.11	0.00	0.00	1.24	0.00	3.10	5.61
carbohydrate (chondroitin 4) sulfotransferase 11	Chst11	100771304	12.37	12.74	14.45	14.44	14.46	15.10	1754.54	1805.36	2059.38	2038.54	2040.99	2136.65
carbohydrate (chondroitin 4) sulfotransferase 12	Chst12	100762689	6.38	6.22	4.07	3.50	2.63	3.12	288.62	284.58	187.56	158.77	119.02	143.00
carbohydrate (chondroitin 4) sulfotransferase 13	Chst13	100771243	0.00	0.00	0.00	0.00	0.00	0.00	0.00	0.00	0.00	0.00	0.00	0.00
carbohydrate (N-acetylgalactosamine 4-0) sulfotransferase 14	Chst14	100753239	7.88	7.59	6.11	4.99	4.26	4.27	341.79	324.84	280.71	225.99	191.47	206.56
carbohydrate (N-acetylgalactosamine 4-sulfate 6-O) sulfotransferase 15	Chst15	100768085	0.00	0.00	0.00	0.00	0.00	0.00	0.00	0.00	0.00	0.00	0.00	0.00
carbohydrate (N-acetylglucosamine-6-O) sulfotransferase 2	Chst2	100767419	2.57	3.19	1.58	1.18	1.08	0.81	123.05	149.15	77.01	59.10	51.75	40.19
carbohydrate (chondroitin 6) sulfotransferase 3	Chst3	100758880	0.02	0.01	0.03	0.00	0.00	0.00	0.76	0.00	0.00	0.00	0.00	0.00
carbohydrate (N-acetylglucosamine 6-O) sulfotransferase 4	Chst4	100759418	0.00	0.00	0.00	0.00	0.00	0.00	0.00	0.00	0.00	0.00	0.00	0.00
carbohydrate (N-acetylglucosamine 6-O) sulfotransferase 7	Chst7	103163077	0.00	0.00	0.00	0.00	0.00	0.00	0.00	0.00	0.00	0.00	0.00	0.00
carbohydrate (N-acetylgalactosamine 4-0) sulfotransferase 9	Chst9	100770625	0.05	0.00	0.00	0.04	0.03	0.00	2.28	1.83	0.00	1.16	1.03	0.00

chondroitin sulfate synthase 1	Chsy1	100770803	17.99	18.31	24.16	24.02	26.10	25.14	1290.46	1343.27	1768.73	1743.02	1876.43	1813.26
CTF18, chromosome transmission fidelity factor 18 homolog (S. cerevisiae)	Chtf18	100761649	8.16	8.60	10.72	7.82	5.66	6.53	782.33	817.12	1016.03	755.62	542.33	621.56
CTF8, chromosome transmission fidelity factor 8 homolog (S. cerevisiae)	Chtf8	100757608	78.47	74.46	78.39	72.88	65.47	68.88	2084.94	1922.49	1919.02	1821.82	1797.77	1894.58
chromatin target of PRMT1	Chtop	100769194	89.67	89.92	92.90	92.02	89.01	93.93	4464.57	4512.03	4656.58	4508.20	4260.00	4492.96
conserved helix-loop-helix ubiquitous kinase	Chuk	100754432	27.10	28.32	34.01	37.52	37.86	37.68	2815.61	2933.60	3523.80	3838.34	3873.95	3886.36
churchill domain containing 1	Churc1	103160311	30.61	31.62	34.39	32.02	41.81	39.19	687.38	713.73	770.09	706.94	909.75	847.75
chymosin	Chymosin	100759177	0.08	0.06	0.04	0.04	0.00	0.03	3.80	2.75	1.24	4.64	0.00	1.87
cytosolic iron-sulfur assembly component 1	Ciao1	100758531	58.41	56.89	75.35	73.85	77.41	75.54	1599.59	1591.24	2086.71	2001.45	2056.52	2060.95
cytokine induced apoptosis inhibitor 1	Ciapin1	100760880	45.62	45.22	59.60	60.81	60.91	65.20	2387.23	2402.88	3172.29	3199.78	3205.35	3387.24
circadian associated repressor of transcription	Ciart	100761544	5.22	4.85	3.85	3.88	3.62	4.25	352.43	335.82	272.02	248.01	225.63	256.10
calcium and integrin binding 1 (calmyrin)	Cib1	100772612	51.18	51.36	60.55	51.69	52.85	53.63	1126.40	1119.09	1306.68	1084.75	1130.21	1151.51
calcium and integrin binding family member 2	Cib2	100753997	4.00	3.75	5.37	4.43	4.24	4.84	243.05	240.65	295.62	232.94	220.45	266.38
calcium and integrin binding family member 3	Cib3	100760089	0.00	0.00	0.00	0.00	0.00	0.00	1.52	0.00	0.00	0.00	0.00	0.00
calcium and integrin binding family member 4	Cib4	100760371	2.29	2.41	1.26	1.37	2.17	1.93	223.30	210.46	144.08	112.42	170.77	175.72
capicua transcriptional repressor	Cic	100756465	5.55	5.70	5.34	3.40	2.40	2.63	486.11	497.78	470.75	299.00	207.00	232.73
cell death-inducing DFFA-like effector a	Cidea	100763404	0.04	0.08	0.17	0.11	0.10	0.17	0.76	1.83	4.97	2.32	2.07	4.67
cell death-inducing DFFA-like effector b	Cideb	100769570	0.29	0.46	0.25	0.27	0.31	0.25	15.19	21.96	13.66	16.22	17.59	13.09
cell death-inducing DFFA-like effector c	Cidec	100750457	47.31	44.80	29.07	26.73	26.10	27.21	2340.90	2227.19	1456.97	1335.08	1311.33	1373.96
class II, major histocompatibility complex, transactivator	Ciita	100768306	0.00	0.00	0.00	0.00	0.00	0.00	0.00	0.00	0.00	0.00	0.00	0.00
cartilage intermediate layer protein, nucleotide pyrophosphohydrolase	Cilp	100774209	0.01	0.00	0.00	0.00	0.00	0.00	0.76	0.00	0.00	0.00	0.00	0.00
cartilage intermediate layer protein 2	Cilp2	103160703	0.22	0.29	0.36	0.29	0.11	0.23	20.51	30.20	23.60	18.54	15.52	15.89
cyclin-dependent kinase 2 interacting protein	Cinp	100755743	29.27	27.23	26.71	27.65	26.05	28.31	638.01	613.07	561.42	567.87	526.81	572.95
CLOCK-interacting pacemaker	Cipc	100762121	13.64	13.50	15.95	17.05	16.28	15.79	1617.82	1579.35	1901.63	1951.62	1860.91	1814.19
corepressor interacting with RBPJ, 1	Cir1	100771162	54.86	53.73	57.56	64.67	63.41	59.52	2854.35	2816.47	3051.81	3328.42	3262.28	3084.41
cold inducible RNA binding protein	Cirbp	100689075	59.93	66.50	64.32	55.18	55.07	58.87	2103.92	2293.99	2230.79	1895.99	1869.19	1976.83
cirrhosis, autosomal recessive 1A (cirhin)	Cirh1a	100757324	76.06	78.26	86.62	93.13	86.38	86.39	5164.11	5445.36	5984.37	6304.52	5823.87	5831.40
CDGSH iron sulfur domain 1	Cisd1	100772922	29.55	30.81	31.92	30.74	31.88	32.78	710.17	733.86	761.40	725.48	756.57	785.12
CDGSH iron sulfur domain 3	Cisd3	100755651	75.62	72.81	50.09	46.36	41.05	46.80	1304.89	1265.49	862.01	782.27	700.69	799.14
cytokine inducible SH2-containing protein	Cish	100774955	2.25	2.15	2.37	2.08	1.96	1.50	123.05	115.29	130.42	112.42	107.64	85.05
citron rho-interacting serine/threonine kinase	Cit	100755517	8.74	8.67	8.68	8.94	8.18	8.44	2463.19	2491.64	2527.65	2522.97	2316.30	2380.60

Cbp/p300-interacting transactivator, with Glu/Asp-rich carboxy-terminal domain, 1	Cited1	100759164	5.71	5.82	12.03	8.94	6.23	6.18	304.58	318.43	675.70	509.92	360.18	367.33
Cbp/p300-interacting transactivator, with Glu/Asp-rich carboxy-terminal domain, 2	Cited2	100773955	21.86	23.03	25.66	26.59	29.07	27.25	1069.43	1077.91	1264.44	1275.97	1540.06	1455.28
CDKN1A interacting zinc finger protein 1	Ciz1	100753565	11.47	10.57	12.98	11.32	8.30	8.34	950.94	926.01	1110.43	985.08	764.85	738.39
cytoskeleton associated protein 2	Ckap2	100771975	24.58	25.71	19.77	24.22	27.63	24.05	1545.66	1647.98	1259.48	1511.23	1716.01	1494.54
cytoskeleton associated protein 2-like	Ckap2l	100754478	8.78	8.48	8.87	10.90	12.83	11.30	872.71	834.51	901.76	1078.95	1229.56	1111.32
cytoskeleton-associated protein 4	Ckap4	100766532	79.34	84.78	103.23	101.05	90.85	91.24	6386.21	6834.39	8376.63	8140.25	7254.22	7357.72
cytoskeleton associated protein 5	Ckap5	100755237	42.67	42.52	36.59	43.63	48.07	45.53	8784.83	8876.74	7638.83	8973.52	9844.79	9316.79
creatine kinase, brain	Ckb	100774847	0.15	0.12	0.16	0.36	0.19	0.19	11.39	9.15	18.63	22.02	13.45	19.63
chemokine-like factor	Cklf	100766496	92.84	87.98	101.19	98.81	111.42	118.73	1449.20	1372.55	1573.72	1542.52	1726.36	1814.19
creatine kinase, muscle	Ckm	100754895	0.00	0.00	0.00	0.00	0.00	0.00	0.00	0.00	0.00	0.00	0.00	0.00
creatine kinase, mitochondrial 1B	Ckmt1b	100754659	0.27	0.25	0.03	0.03	0.13	0.14	10.63	10.07	2.48	2.32	5.17	5.61
creatine kinase, mitochondrial 2 (sarcomeric)	Ckmt2	100752172	0.00	0.00	0.00	0.00	0.00	0.00	0.00	0.00	0.00	0.00	0.00	0.93
CDC28 protein kinase regulatory subunit 2	Cks2	100760104	132.88	136.93	146.31	140.40	172.33	169.48	1475.78	1515.30	1552.61	1508.91	1851.59	1775.87
cytoplasmic linker associated protein 1	Clasp1	100756713	9.60	10.11	11.03	12.10	12.10	11.99	1750.74	1840.13	2018.39	2194.99	2208.66	2185.26
cytoplasmic linker associated protein 2	Clasp2	100769743	12.11	12.76	14.02	15.36	17.83	16.73	2163.93	2304.97	2535.10	2757.07	3168.09	3019.92
claudin-1	Claudin-18	100755597	0.00	0.00	0.00	0.00	0.00	0.00	0.00	0.00	0.00	0.00	0.00	0.00
claudin-4	Claudin-4	100770792	0.00	0.00	0.00	0.00	0.00	0.00	0.00	0.00	0.00	0.00	0.00	0.00
chloride channel accessory 1	Clca1	100754570	0.00	0.00	0.00	0.02	0.00	0.00	0.00	0.92	0.00	1.16	0.00	0.00
chloride channel accessory 2	Clca2	100754285	0.02	0.00	0.00	0.00	0.00	0.00	2.28	0.92	0.00	1.16	0.00	0.00
chloride channel CLIC-like 1	Clcc1	100768001	29.54	29.43	30.78	34.79	34.73	34.13	2789.03	2846.67	2989.70	3362.03	3307.82	3252.65
cardiotrophin-like cytokine factor 1	Clcf1	100761392	7.80	6.67	9.62	8.54	7.45	7.75	407.11	348.63	504.29	443.87	379.84	408.45
chloride channel, voltage-sensitive 1	Clcn1	100751277	0.02	0.02	0.00	0.01	0.03	0.01	2.28	3.66	0.00	1.16	3.10	4.67
chloride channel, voltage-sensitive 2	Clcn2	100769999	1.83	1.77	1.42	1.94	1.79	1.78	195.96	197.65	158.99	219.04	183.19	186.93
chloride channel, voltage-sensitive 3	Clcn3	100759588	20.87	20.49	22.70	23.60	24.15	25.44	3199.18	3192.55	3510.14	3584.54	3687.66	3935.89
chloride channel, voltage-sensitive 4	Clcn4	100762547	3.81	3.35	3.48	3.51	4.01	3.89	543.83	483.14	506.77	508.77	571.31	564.54
chloride channel, voltage-sensitive 5	Clcn5	100754025	0.10	0.12	0.01	0.01	0.03	0.03	25.82	30.20	2.48	3.48	8.28	8.41
chloride channel, voltage-sensitive 6	Clcn6	100774640	6.99	7.20	6.58	6.62	6.14	5.79	1122.60	1162.09	1055.77	1074.32	979.10	937.47
chloride channel, voltage-sensitive 7	Clcn7	100773701	13.45	12.43	12.99	12.79	10.99	11.78	1214.50	1139.22	1197.37	1201.80	1023.60	1106.65
claudin 1	Cldn1	100771737	0.00	0.00	0.00	0.00	0.00	0.00	0.00	0.00	0.00	0.00	0.00	0.00
claudin 10	Cldn10	100762571	0.00	0.00	0.00	0.00	0.00	0.00	0.76	1.83	0.00	0.00	0.00	0.93
claudin 11	Cldn11	100765050	0.00	0.00	0.00	0.00	0.00	0.00	0.00	1.83	0.00	0.00	0.00	0.00
claudin 12	Cldn12	100769202	8.99	8.88	7.82	8.83	9.09	9.32	937.27	934.25	833.44	927.14	950.12	978.60
claudin 14	Cldn14	100751181	0.00	0.00	0.00	0.00	0.00	0.00	0.00	0.00	0.00	0.00	0.00	0.00
claudin 15	Cldn15	100758840	0.14	0.12	0.07	0.12	0.08	0.10	5.32	4.58	2.48	8.11	4.14	5.61
claudin 16	Cldn16	100751792	0.00	0.12	0.00	0.15	0.07	0.06	1.52	1.83	0.00	3.48	1.03	0.93
claudin 17	Cldn17	100771924	0.00	0.00	0.00	0.00	0.00	0.00	0.00	0.00	0.00	0.00	0.00	0.00
claudin 18	Cldn18	100769098	0.00	0.00	0.00	0.00	0.00	0.00	0.00	0.00	0.00	0.00	0.00	0.00

claudin 19	Cldn19	100768379	0.00	0.13	0.00	0.00	0.00	0.00	0.76	1.83	0.00	0.00	0.00	0.00
claudin 2	Cldn2	100762065	0.00	0.00	0.00	0.00	0.00	0.00	0.00	0.00	0.00	0.00	0.00	0.00
claudin 20	Cldn20	100771740	0.14	0.17	0.14	0.11	0.18	0.21	23.55	28.37	24.84	18.54	31.05	34.58
claudin 22	Cldn22	100752077	5.84	5.19	5.79	6.38	7.81	8.44	274.95	251.63	263.32	275.82	318.78	369.19
claudin 23	Cldn23	100770787	0.00	0.00	0.00	0.00	0.00	0.00	0.00	0.00	0.00	0.00	0.00	0.00
claudin 25	Cldn25	100756403	0.00	0.00	0.00	0.00	0.00	0.00	0.76	0.00	0.00	0.00	0.00	0.93
claudin 3	Cldn3	100766489	0.00	0.00	0.00	0.00	0.00	0.00	0.00	0.00	0.00	0.00	0.00	0.00
claudin 4	Cldn4	100768680	0.00	0.00	0.00	0.00	0.00	0.00	0.00	0.00	0.00	0.00	0.00	0.00
claudin 5	Cldn5	100769480	0.00	0.00	0.00	0.00	0.00	0.00	0.00	0.00	0.00	0.00	0.00	0.00
claudin 6	Cldn6	100769217	0.70	0.72	0.68	0.49	0.50	0.79	41.77	44.84	38.50	28.97	30.01	45.80
claudin 7	Cldn7	100766949	0.00	0.00	0.00	0.00	0.00	0.00	0.00	0.92	0.00	0.00	1.03	0.00
claudin 8	Cldn8	100772201	0.00	0.00	0.00	0.00	0.00	0.00	0.00	0.00	0.00	0.00	0.00	0.00
claudin 9	Cldn9	100769504	0.00	0.00	0.00	0.00	0.00	0.00	0.00	0.00	0.00	0.00	0.00	0.00
claudin domain containing 1	Cldnd1	100770002	81.12	83.94	89.43	94.89	105.97	108.88	3060.94	3160.53	3400.83	3548.61	3947.44	4054.60
claudin domain containing 2	Cldnd2	100752776	0.00	0.00	0.00	0.00	0.00	0.00	0.00	0.00	0.00	0.00	0.00	0.00
C-type lectin domain family 11, member A	Clec11a	100755020	0.07	0.00	0.00	0.00	0.00	0.00	0.76	0.92	0.00	0.00	0.00	0.00
C-type lectin domain family 12, member A	Clec12a	100769792	0.00	0.00	0.00	0.00	0.00	0.00	0.00	0.00	0.00	0.00	0.00	0.00
C-type lectin domain family 12, member B	Clec12b	100772182	0.05	0.00	0.00	0.00	0.04	0.00	1.52	0.00	0.00	0.00	1.03	0.00
C-type lectin domain family 14, member A	Clec14a	100766342	0.00	0.00	0.00	0.00	0.00	0.00	0.00	0.00	0.00	0.00	0.00	0.00
C-type lectin domain family 16, member A	Clec16a	100768590	6.23	6.00	8.48	8.68	7.68	7.77	1158.30	1109.94	1496.71	1550.63	1382.74	1393.59
C-type lectin domain family 19, member A	Clec19a	103158746	0.00	0.00	0.00	0.00	0.00	0.00	0.00	0.00	0.00	0.00	0.00	0.00
C-type lectin domain family 1, member A	Clec1a	100772475	0.00	0.00	0.00	0.00	0.00	0.00	0.00	0.00	0.00	0.00	0.00	0.00
C-type lectin domain family 1, member B	Clec1b	100770085	0.00	0.00	0.00	0.00	0.00	0.00	0.00	0.00	0.00	0.00	0.00	0.00
C-type lectin domain family 2, member L	Clec2l	100770823	0.48	0.48	0.80	0.67	0.43	0.43	37.98	30.20	60.86	54.47	45.54	46.73
C-type lectin domain family 3, member A	Clec3a	100762318	0.00	0.00	0.00	0.00	0.00	0.00	0.00	0.00	0.00	0.00	0.00	0.00
C-type lectin domain family 3, member B	Clec3b	100760600	0.00	0.00	0.00	0.00	0.00	0.00	0.00	0.00	0.00	1.16	1.03	0.00
C-type lectin domain family 4, member D	Clec4d	100775089	0.00	0.00	0.00	0.00	0.00	0.00	0.00	0.00	0.00	0.00	0.00	0.00
C-type lectin domain family 4, member E	Clec4e	100773355	0.00	0.00	0.00	0.00	0.00	0.00	0.00	0.00	0.00	0.00	0.00	0.00
C-type lectin domain family 4, member F	Clec4f	100754159	0.00	0.02	0.00	0.00	0.00	0.00	0.00	0.92	0.00	0.00	0.00	0.00
C-type lectin domain family 4, member G	Clec4g	100772659	0.00	0.00	0.00	0.00	0.00	0.00	0.00	0.00	0.00	0.00	0.00	0.00
C-type lectin domain family 5, member A	Clec5a	100754713	0.00	0.00	0.00	0.03	0.00	0.00	0.00	0.00	0.00	1.16	0.00	0.00
C-type lectin domain family 6, member A	Clec6a	100772671	0.00	0.00	0.00	0.00	0.00	0.00	0.00	0.00	0.00	0.00	0.00	0.00
C-type lectin domain family 7, member A	Clec7a	100772766	0.00	0.00	0.00	0.00	0.00	0.00	0.00	0.00	0.00	0.00	0.00	0.00

C-type lectin domain family 9, member A	Clec9a	100770747	0.00	0.00	0.00	0.00	0.00	0.00	0.00	0.00	0.00	0.00	0.00	0.00
calmegin	Clgn	100773546	0.03	0.02	0.00	0.00	0.00	0.00	1.52	0.92	1.24	0.00	0.00	0.00
clathrin heavy chain linker domain containing 1	Clhc1	100764559	3.14	3.11	2.67	2.69	2.81	3.40	203.56	194.90	175.13	184.27	186.30	208.43
chloride intracellular channel 1	Clic1	100761384	261.15	257.22	250.99	257.53	253.50	272.26	7595.39	7532.56	7291.05	7323.21	7213.85	7814.77
chloride intracellular channel 3	Clic3	100763283	0.00	0.06	0.00	0.08	0.00	0.00	4.56	4.58	2.48	2.32	1.03	2.80
chloride intracellular channel 4	Clic4	100758280	56.16	55.92	50.25	53.31	55.61	57.35	6447.73	6437.26	5825.39	6133.00	6344.47	6614.66
chloride intracellular channel 6	Clic6	100757995	0.00	0.00	0.00	0.00	0.00	0.00	1.52	0.92	1.24	0.00	0.00	0.00
clathrin interactor 1	Clint1	100761656	27.31	26.00	25.16	26.67	26.09	25.45	2590.79	2471.51	2424.55	2539.19	2462.23	2415.19
CAP-GLY domain containing linker protein 1	Clip1	100769657	20.58	19.52	20.88	24.60	22.70	22.57	3694.40	3543.92	3784.64	4447.93	4050.94	4049.92
CAP-GLY domain containing linker protein 2	Clip2	100769773	11.26	10.26	12.43	11.15	8.16	8.39	1391.48	1308.50	1570.00	1379.11	1025.67	1060.85
CAP-GLY domain containing linker protein 3	Clip3	100771592	0.00	0.00	0.00	0.00	0.00	0.00	3.04	3.66	7.45	3.48	4.14	5.61
CAP-GLY domain containing linker protein family, member 4	Clip4	100754754	0.00	0.00	0.00	0.00	0.00	0.00	0.00	0.00	0.00	0.00	0.00	0.00
CDC-like kinase 1	Clk1	100758582	33.97	32.47	29.44	33.90	36.21	33.28	1901.13	1793.47	1624.65	1884.40	2013.05	1866.54
CDC-like kinase 2	Clk2	100755756	19.07	17.81	22.05	21.09	20.02	20.47	1107.41	1034.90	1278.11	1226.14	1201.62	1199.18
CDC-like kinase 3	Clk3	100756143	35.34	35.17	33.83	30.36	24.78	26.36	1947.46	1988.37	1923.99	1729.11	1374.46	1437.52
CDC-like kinase 4	Clk4	100759687	8.84	8.77	7.92	8.53	11.54	10.29	558.26	539.87	471.99	530.79	694.48	624.36
calmin (calponin-like, transmembrane)	Clmn	100768555	0.02	0.03	0.00	0.01	0.00	0.00	1.52	3.66	0.00	1.16	0.00	1.87
CXADR-like membrane protein	Clmp	100762282	4.76	5.37	6.74	6.58	6.35	6.68	382.81	420.92	534.10	548.17	515.42	534.63
ceroid-lipofuscinosis, neuronal 3	Cln3	100754370	9.55	8.68	5.63	5.61	5.66	4.98	526.36	496.86	322.94	290.89	301.18	261.71
ceroid-lipofuscinosis, neuronal 5	Cln5	100763877	19.65	20.11	15.43	19.17	20.09	20.03	1154.50	1183.14	909.21	1117.20	1164.36	1166.47
ceroid-lipofuscinosis, neuronal 6, late infantile, variant	Cln6	100760975	8.78	9.21	8.24	9.32	9.19	9.22	512.69	534.38	474.48	531.94	521.63	524.35
ceroid-lipofuscinosis, neuronal 8 (epilepsy, progressive with mental retardation)	Cln8	100757767	6.26	6.59	7.77	6.41	5.92	6.20	410.91	430.98	511.74	427.64	383.98	410.32
cytokine-dependent hematopoietic cell linker	Clnk	100767591	0.00	0.00	0.00	0.00	0.00	0.00	0.00	0.00	0.00	0.00	0.00	0.00
chloride channel, nucleotide-sensitive, 1A	Clns1a	100768284	110.18	112.16	90.49	93.90	98.68	98.87	4426.59	4514.78	3630.62	3706.23	3937.09	3939.63
clock circadian regulator	Clock	100770092	14.53	13.99	13.97	15.90	16.38	15.99	3197.66	3058.96	3096.52	3546.29	3534.48	3561.09
cleavage and polyadenylation factor I subunit 1	Clp1	100755872	20.70	19.04	19.93	19.00	22.20	19.60	1014.74	940.65	983.73	932.93	1088.81	968.32
ClpB caseinolytic peptidase B homolog (E. coli)	Clpb	100772391	40.77	41.16	44.38	44.17	38.28	39.19	2589.27	2593.21	2787.24	2783.73	2434.29	2457.25
caseinolytic mitochondrial matrix peptidase proteolytic subunit	Clpp	100761651	101.16	92.54	98.93	90.05	67.03	75.16	2667.50	2459.61	2594.72	2403.60	1875.40	2033.84
colipase, pancreatic	Clps	100760027	0.00	0.00	0.00	0.00	0.00	0.00	0.00	0.00	0.00	0.00	0.00	0.00
colipase-like 2	Clpsl2	100760322	0.00	0.00	0.00	0.00	0.00	0.00	0.00	0.00	0.00	0.00	0.00	0.00
cleft lip and palate associated transmembrane protein 1	Clptm1	100752525	17.39	16.63	15.27	13.81	9.80	9.99	1342.11	1297.52	1184.95	1069.68	764.85	775.78
CLPTM1-like	Clptm1l	100773964	96.00	92.56	95.03	89.92	89.97	93.53	5991.25	5861.70	5966.99	5595.27	5672.76	5837.95
caseinolytic mitochondrial matrix peptidase chaperone subunit	Clpx	100774494	18.01	19.63	23.40	24.34	28.41	26.91	1287.42	1402.75	1659.43	1710.57	1999.59	1915.14
clarin 1	Clrn1	100768820	0.17	0.45	0.14	0.18	0.17	0.26	15.19	17.39	14.91	11.59	12.42	12.15

clarin 2	Clrn2	100769210	0.00	0.00	0.00	0.00	0.00	0.00	0.00	0.00	0.00	0.00	0.00	0.00
clarin 3	Clrn3	100755717	0.00	0.00	0.00	0.00	0.00	0.00	0.00	0.00	0.00	0.00	0.00	0.00
claspin	Clspn	100768047	9.60	9.36	10.22	11.43	12.82	11.89	1474.27	1462.22	1606.02	1763.88	1970.61	1836.63
calsyntenin 1	Clstn1	100771175	47.97	45.93	53.39	49.61	40.72	45.15	6319.37	6115.17	7190.44	6556.01	5446.10	6017.40
calsyntenin 2	Clstn2	100763054	0.00	0.00	0.00	0.00	0.00	0.00	0.00	0.00	0.00	0.00	0.00	0.00
calsyntenin 3	Clstn3	100760360	0.10	0.11	0.18	0.14	0.14	0.12	9.87	14.64	19.87	17.38	13.45	13.09
clathrin, light chain A	Clta	100758987	103.99	105.10	112.42	107.59	110.97	117.35	1888.21	1913.34	2023.36	1879.77	1955.09	2074.03
clathrin, light chain B	Cltb	100767928	34.37	33.46	48.14	40.65	36.88	40.52	732.20	708.24	1008.57	830.95	759.68	847.75
clathrin, heavy chain (Hc)	Cltc	100756644	62.91	62.96	32.95	35.99	38.82	39.11	9846.67	9843.02	5177.02	5582.52	5978.08	6088.44
clusterin	Clu	100756447	127.37	124.19	212.55	223.35	169.11	179.55	5866.68	5759.22	9817.45	10035.09	7563.68	8037.22
clusterin associated protein 1	Cluap1	100764485	15.31	15.67	16.61	18.74	21.17	19.84	754.98	799.74	821.02	920.18	1013.25	955.23
clustered mitochondria (cluA/CLU1) homolog	Cluh	100766235	17.09	15.66	16.24	15.56	11.56	12.70	2685.73	2509.02	2567.39	2454.59	1816.40	2016.08
clusterin-like 1 (retinal)	Clul1	100754851	0.02	0.00	0.00	0.00	0.00	0.00	0.76	0.00	0.00	0.00	0.00	0.00
clavesin 2	Clvs2	100752437	0.00	0.00	0.00	0.00	0.00	0.00	0.00	0.00	0.00	0.00	0.00	0.00
citrate lyase beta like	Clybl	100767276	17.22	16.38	16.39	18.55	18.33	18.94	515.73	501.44	479.45	534.26	539.23	541.17
cytidine monophospho-N-acetylneuraminic acid hydroxylase	Cmah	100689391	0.53	0.56	0.55	0.74	0.84	0.55	47.09	42.09	32.29	55.63	63.13	39.26
cytidine monophosphate N-acetylneuraminic acid synthetase	Cmas	100773134	119.34	121.66	129.48	136.85	142.51	143.81	4745.60	4880.79	5232.91	5488.64	5591.00	5645.40
carboxymethylenebutenolidase homolog (Pseudomonas)	Cmbl	100757587	0.79	0.73	1.46	1.30	1.16	1.20	59.24	45.75	68.31	77.65	57.96	72.90
C-x(9)-C motif containing 1	Cmc1	100753575	28.43	31.58	31.53	32.51	39.89	41.61	390.40	415.43	442.18	435.75	550.61	551.46
C-x(9)-C motif containing 2	Cmc2	100764782	20.09	20.80	20.50	23.73	28.77	27.35	638.77	665.23	653.34	747.50	876.63	855.22
C-x(9)-C motif containing 4	Cmc4	100754531	5.54	8.89	4.25	4.90	7.00	5.69	6.84	5.49	4.97	3.48	0.00	1.87
c-Maf inducing protein	Cmip	100759767	11.56	10.36	11.10	9.21	6.28	7.22	709.41	653.33	684.39	557.44	418.13	457.99
chemokine-like receptor 1	Cmklr1	100760709	0.00	0.00	0.00	0.00	0.00	0.00	0.00	0.00	0.00	0.00	0.00	0.00
cytidine monophosphate (UMP-CMP) kinase 1, cytosolic	Cmpk1	100758717	11.90	13.03	12.67	13.41	16.38	14.51	947.15	1031.24	1007.33	1061.57	1291.66	1150.58
cytidine monophosphate (UMP-CMP) kinase 2, mitochondrial	Cmpk2	100754791	0.00	0.00	0.00	0.00	0.00	0.00	0.00	0.00	0.00	0.00	0.00	0.00
cms1 ribosomal small subunit homolog (yeast)	Cmss1	100757899	42.10	41.26	45.51	47.50	45.90	46.14	1246.40	1202.35	1332.76	1351.30	1324.78	1331.90
CKLF-like MARVEL transmembrane domain containing 1	Cmtm1	103163017	0.66	0.39	0.93	1.01	0.93	0.83	30.38	27.45	45.96	46.36	45.54	53.28
CKLF-like MARVEL transmembrane domain containing 3	Cmtm3	100765909	29.53	27.66	32.46	31.21	32.41	32.74	1352.74	1282.88	1501.68	1445.17	1460.37	1484.26
CKLF-like MARVEL transmembrane domain containing 4	Cmtm4	100768129	8.24	8.66	7.99	8.93	8.88	9.13	330.40	344.97	337.85	366.22	366.39	366.39
CKLF-like MARVEL transmembrane domain containing 5	Cmtm5	100767104	0.00	0.00	0.00	0.00	0.00	0.00	0.00	0.00	0.00	0.00	0.00	0.00
CKLF-like MARVEL transmembrane domain containing 6	Cmtm6	100768322	37.78	37.29	44.30	44.72	46.50	47.99	3684.52	3643.66	4359.72	4371.45	4514.61	4707.00
CKLF-like MARVEL transmembrane domain containing 7	Cmtm7	100768605	68.58	68.16	72.04	66.44	53.75	55.56	1530.47	1518.96	1640.80	1491.53	1180.92	1223.48
CKLF-like MARVEL transmembrane domain containing 8	Cmtm8	100768898	0.04	0.05	0.13	0.06	0.17	0.05	2.28	2.75	4.97	2.32	4.14	0.93
cap methyltransferase 1	Cmtr1	100755381	25.57	25.57	26.33	28.19	28.01	28.91	2902.96	2931.77	3035.66	3203.25	3168.09	3312.47
cap methyltransferase 2	Cmtr2	100754099	11.44	11.17	13.44	13.57	15.84	15.57	1047.40	1024.84	1239.60	1248.16	1448.98	1432.85
cardiomyopathy associated 5	Cmya5	100761385	0.05	0.04	0.08	0.06	0.08	0.11	16.71	12.81	27.33	22.02	24.84	37.39

cyclic nucleotide binding domain containing 1	Cnbd1	100763226	0.10	0.07	0.10	0.03	0.03	0.02	5.32	4.58	2.48	1.16	1.03	0.93
cyclic nucleotide binding domain containing 2	Cnbd2	100751515	0.00	0.00	0.00	0.00	0.00	0.00	0.76	0.00	0.00	0.00	0.00	0.00
CCHC-type zinc finger, nucleic acid binding protein	Cnbp	100689247	510.30	523.86	570.67	599.43	586.07	568.76	16207.05	16610.60	18043.80	19065.39	18011.87	18295.22
carnosine dipeptidase 1 (metallopeptidase M20 family)	Cndp1	100761203	0.00	0.00	0.00	0.00	0.00	0.00	0.00	0.00	0.00	0.00	1.03	0.93
CNDP dipeptidase 2 (metallopeptidase M20 family)	Cndp2	100760626	81.85	77.05	89.34	88.90	89.00	96.94	3562.24	3375.56	3937.42	3907.88	4013.68	4255.55
CTD nuclear envelope phosphatase 1 regulatory subunit 1	Cnep1r1	100759409	9.13	7.55	10.36	7.02	10.07	11.39	233.94	223.27	264.56	242.21	294.97	288.81
cyclic nucleotide gated channel alpha 1	Cnga1	100768599	0.12	0.05	0.10	0.03	0.08	0.05	7.60	2.75	3.73	2.32	4.14	1.87
cyclic nucleotide gated channel alpha 2	Cnga2	100771809	0.00	0.00	0.00	0.00	0.00	0.00	0.00	0.00	0.00	0.00	0.00	0.00
cyclic nucleotide gated channel alpha 3	Cnga3	100771361	0.00	0.00	0.00	0.00	0.00	0.00	0.00	0.00	0.00	0.00	0.00	0.00
cyclic nucleotide gated channel alpha 4	Cnga4	100753462	0.07	0.02	0.03	0.03	0.00	0.00	5.32	2.75	1.24	2.32	0.00	0.93
cyclic nucleotide gated channel beta 3	Cngb3	100757356	0.00	0.00	0.00	0.00	0.00	0.00	0.00	0.00	0.00	0.00	0.00	0.00
cornichon family AMPA receptor auxiliary protein 1	Cnih1	103160710	204.78	214.61	232.35	238.30	271.61	262.80	5655.53	6002.62	6590.51	6689.29	7595.76	7352.11
cornichon family AMPA receptor auxiliary protein 2	Cnih2	100768448	0.57	0.88	0.46	0.34	0.46	0.62	22.79	41.18	17.39	19.70	22.77	26.17
cornichon family AMPA receptor auxiliary protein 3	Cnih3	100759925	0.03	0.03	0.05	0.00	0.07	0.04	1.52	0.92	1.24	2.32	3.10	0.93
cornichon family AMPA receptor auxiliary protein 4	Cnih4	100760720	60.81	59.59	88.86	90.25	115.84	114.92	1753.78	1697.39	2482.93	2485.88	3256.07	3256.39
connector enhancer of kinase suppressor of Ras 1	Cnksr1	100769136	4.94	4.82	2.09	2.04	2.12	2.23	454.96	445.62	226.06	198.18	191.47	216.84
connector enhancer of kinase suppressor of Ras 2	Cnksr2	100752301	1.22	1.15	1.59	1.66	1.88	1.92	173.93	165.62	231.03	232.94	270.13	280.40
CNKSR family member 3	Cnksr3	100774635	4.76	4.71	5.39	6.03	5.02	5.05	376.73	370.59	447.15	485.59	415.03	416.86
calponin 1, basic, smooth muscle	Cnn1	100753310	0.23	0.20	0.05	0.05	0.05	0.00	7.60	6.41	1.24	1.16	2.07	0.00
calponin 2	Cnn2	100754225	110.75	110.65	127.29	119.61	110.28	113.91	6301.14	6310.07	7298.50	6783.16	6227.51	6419.31
calponin 3, acidic	Cnn3	100773931	168.74	178.45	169.84	172.44	173.19	171.31	7245.24	7652.43	7174.29	7373.05	7256.29	7330.61
cyclin M1	Cnnm1	100768398	0.00	0.00	0.00	0.00	0.00	0.00	0.00	0.00	0.00	0.00	0.00	1.87
cyclin M3	Cnnm3	100772518	8.25	8.21	11.09	10.16	9.56	9.54	730.68	725.62	1014.78	939.88	900.44	900.09
cyclin M4	Cnnm4	100772810	7.74	7.84	10.94	10.69	11.53	12.13	798.28	804.31	1146.45	1098.66	1196.44	1265.54
CCR4-NOT transcription complex, subunit 1	Cnot1	100761778	40.61	40.43	38.53	43.00	43.95	43.66	9603.61	9722.23	9326.83	10242.53	10409.89	10277.63
CCR4-NOT transcription complex, subunit 10	Cnot10	100770796	33.76	30.44	37.26	42.30	40.62	42.26	2612.06	2434.90	2935.05	3270.47	3133.94	3188.16
CCR4-NOT transcription complex, subunit 11	Cnot11	100760179	26.41	26.64	29.36	32.53	35.93	34.26	1439.33	1475.03	1643.28	1811.39	1960.26	1868.40
CCR4-NOT transcription complex, subunit 3	Cnot3	100765854	3.68	3.44	3.86	2.73	2.34	1.99	511.17	476.73	581.30	403.30	359.14	300.03
CCR4-NOT transcription complex, subunit 4	Cnot4	100761964	12.13	12.37	12.40	12.92	12.60	11.41	1149.18	1165.75	1193.65	1219.18	1181.95	1075.80
CCR4-NOT transcription complex, subunit 6	Cnot6	100764369	8.37	8.43	8.52	9.49	10.34	9.80	1209.19	1225.23	1235.88	1362.89	1489.35	1419.76

CCR4-NOT transcription complex, subunit 6-like	Cnot6l	100769928	8.81	8.86	10.12	12.09	13.11	12.21	2277.86	2285.75	2643.16	3153.42	3370.95	3155.44
CCR4-NOT transcription complex, subunit 7	Cnot7	100773091	36.91	40.15	40.94	42.82	47.78	43.98	2294.57	2544.71	2501.56	2701.44	2988.01	2803.07
CCR4-NOT transcription complex, subunit 8	Cnot8	100770253	15.02	15.29	17.38	16.71	16.44	17.35	920.56	949.80	1080.62	1040.71	1009.11	1052.44
2',3'-cyclic nucleotide 3' phosphodiesterase	Cnp	100753220	0.06	0.07	0.02	0.02	0.02	0.06	6.08	4.58	2.48	2.32	1.03	4.67
cyclin Pas1/PHO80 domain containing 1	Cnppd1	100768844	49.40	49.47	54.97	52.48	51.59	57.34	3985.30	3962.10	4440.46	4137.34	4047.83	4509.78
canopy FGF signaling regulator 1	Cnpy1	100772238	0.00	0.00	0.00	0.00	0.00	0.00	0.00	0.00	0.00	0.00	0.00	0.00
canopy FGF signaling regulator 2	Cnpy2	100765645	118.30	113.21	132.40	132.25	118.69	127.98	2865.74	2723.14	3117.64	2958.72	2640.25	2849.81
canopy FGF signaling regulator 3	Cnpy3	100769298	39.62	40.85	36.23	29.86	23.72	26.84	1896.57	1973.73	1758.79	1419.68	1152.97	1289.84
canopy FGF signaling regulator 4	Cnpy4	100750605	10.90	10.74	11.09	13.12	14.86	14.22	501.30	492.29	501.80	599.16	700.69	655.20
cannabinoid receptor 1 (brain)	Cnr1	100768005	0.00	0.00	0.00	0.00	0.00	0.00	0.00	0.00	0.00	0.00	0.00	0.00
cannabinoid receptor 2 (macrophage)	Cnr2	100752745	0.03	0.00	0.00	0.00	0.00	0.00	1.52	0.00	0.00	0.00	0.00	0.00
cannabinoid receptor interacting protein 1	Cnrip1	100773416	33.17	32.20	30.11	30.71	33.41	35.60	1520.60	1471.37	1377.47	1373.32	1487.28	1588.94
consortin, connexin sorting protein	Cnst	100765705	5.47	5.59	6.14	6.70	7.64	8.26	602.31	616.73	688.12	748.66	855.93	922.52
cyclin N-terminal domain containing 1	Cntd1	100761754	3.56	3.56	2.96	2.92	3.63	3.60	227.86	216.86	157.75	169.20	215.28	206.56
ciliary neurotrophic factor	Cntf	100774933	0.20	0.12	0.29	0.57	0.61	0.35	22.79	12.81	23.60	46.36	50.71	28.04
ciliary neurotrophic factor receptor	Cntfr	100753725	0.00	0.00	0.00	0.00	0.00	0.00	0.00	0.00	0.00	0.00	0.00	0.00
centlein, centrosomal protein	Cntln	100755760	7.11	6.90	6.13	8.25	9.26	7.87	609.91	581.96	531.61	693.03	766.92	650.53
contactin 2 (axonal)	Cntn2	100753717	0.03	0.03	0.01	0.00	0.00	0.00	3.80	2.75	1.24	0.00	0.00	0.00
contactin 3 (plasmacytoma associated)	Cntn3	100757650	0.10	0.15	0.00	0.00	0.00	0.00	9.11	7.32	0.00	0.00	0.00	0.00
contactin 4	Cntn4	100765906	0.00	0.00	0.00	0.00	0.00	0.00	0.00	0.00	0.00	0.00	0.00	0.00
contactin 5	Cntn5	100762864	0.00	0.00	0.00	0.00	0.00	0.00	0.00	0.00	0.00	0.00	0.00	0.00
contactin 6	Cntn6	100772078	0.00	0.00	0.00	0.00	0.00	0.00	0.00	0.00	0.00	0.00	0.00	0.00
contactin associated protein 1	Cntnap1	100751740	7.25	7.19	6.03	5.64	4.73	5.15	934.99	931.50	767.61	732.44	625.13	653.33
contactin associated protein-like 2	Cntnap2	100761620	0.00	0.00	0.00	0.00	0.00	0.00	0.00	4.58	0.00	0.00	0.00	0.00
contactin associated protein-like 4	Cntnap4	100760772	0.00	0.00	0.00	0.00	0.00	0.00	0.00	0.00	0.00	0.00	0.00	0.00
contactin associated protein-like 5	Cntnap5	100766518	0.00	0.00	0.00	0.00	0.00	0.00	0.00	0.00	0.00	0.00	0.00	0.00
centriolin	Cntrl	100753057	17.83	17.17	15.02	18.48	20.13	18.89	3455.14	3337.13	3019.51	3663.35	3964.00	3707.83
centrobin, centrosomal BRCA2 interacting protein	Cntrob	100755050	5.91	6.05	5.01	4.69	3.49	3.80	604.59	653.33	541.55	507.61	405.71	421.54
CoA synthase	Coasy	100757898	48.95	47.54	30.64	26.17	21.23	22.99	3100.44	3015.04	1930.20	1656.10	1351.69	1487.06
cordon-bleu WH2 repeat protein	Cobl	100761073	0.04	0.04	0.04	0.05	0.08	0.04	6.84	6.41	6.21	6.95	12.42	10.28
cordon-bleu WH2 repeat protein-like 1	Cobll1	100753532	20.99	20.69	26.35	29.68	32.44	30.86	2899.16	2852.16	3684.03	4099.10	4436.99	4267.70
cochlin	Coch	100765512	0.00	0.00	0.00	0.00	0.00	0.00	0.76	0.00	0.00	0.00	1.03	0.00
component of oligomeric golgi complex 1	Cog1	100752570	18.23	18.40	19.68	19.57	16.90	17.37	2201.14	2218.04	2417.10	2386.22	2009.94	2102.07
component of oligomeric golgi complex 2	Cog2	100767234	13.85	14.09	15.36	16.61	15.34	15.97	1206.15	1243.53	1377.47	1447.49	1342.38	1397.33
component of oligomeric golgi complex 3	Cog3	100770618	8.87	8.32	8.84	9.43	10.50	10.26	1091.46	1064.18	1095.52	1170.51	1267.86	1243.11

component of oligomeric golgi complex 4	Cog4	100752814	11.21	11.02	10.43	11.68	12.55	14.00	959.30	935.16	904.24	1018.69	1111.58	1188.90
component of oligomeric golgi complex 6	Cog6	100771724	21.50	23.01	22.80	24.81	24.05	25.07	1853.28	1949.02	1906.60	2082.58	2023.40	2105.81
component of oligomeric golgi complex 7	Cog7	100754278	15.11	14.82	15.60	14.13	14.24	13.80	1259.32	1262.75	1324.06	1189.05	1187.13	1154.32
component of oligomeric golgi complex 8	Cog8	100759350	9.19	8.80	8.45	8.93	8.02	8.23	545.35	531.63	506.77	524.99	465.74	488.83
coilin	Coil	100760263	9.33	9.83	5.03	4.82	4.99	4.75	701.81	741.18	373.87	363.90	371.56	355.17
collagen, type X, alpha 1	Col10a1	100750863	0.00	0.00	0.00	0.00	0.02	0.00	0.00	0.00	0.00	0.00	1.03	0.00
collagen, type XI, alpha 1	Col11a1	100768858	0.02	0.02	0.00	0.00	0.00	0.00	4.56	3.66	0.00	0.00	0.00	0.00
collagen, type XI, alpha 2	Col11a2	100766812	0.06	0.08	0.02	0.02	0.01	0.00	11.39	12.81	4.97	8.11	2.07	0.00
collagen, type XII, alpha 1	Col12a1	100752735	12.92	13.51	11.83	13.19	8.92	8.70	2031.77	2091.77	1726.50	1892.52	1295.80	1347.79
collagen, type XIII, alpha 1	Col13a1	100769753	0.00	0.00	0.00	0.00	0.00	0.00	0.76	0.00	0.00	0.00	0.00	0.00
collagen, type XIV, alpha 1	Col14a1	103158800	0.03	0.40	0.00	0.68	0.04	0.04	0.76	5.49	0.00	3.48	1.03	0.93
collagen, type XV, alpha 1	Col15a1	100772437	0.02	0.01	0.00	0.00	0.01	0.01	3.04	0.92	1.24	0.00	1.03	0.93
collagen, type XVI, alpha 1	Col16a1	100768792	7.33	7.70	8.03	5.60	3.05	3.43	1263.87	1341.44	1406.04	986.24	568.21	630.90
collagen, type XVII, alpha 1	Col17a1	100766261	0.66	0.63	0.36	0.27	0.45	0.45	116.21	113.46	70.80	52.15	72.45	79.45
collagen, type XVIII, alpha 1	Col18a1	100764844	0.47	0.44	0.07	0.12	0.05	0.06	87.35	80.52	16.15	20.86	11.38	14.02
collagen, type XIX, alpha 1	Col19a1	100755518	0.00	0.00	0.00	0.00	0.00	0.00	0.00	0.00	0.00	0.00	0.00	0.00
collagen, type I, alpha 1	Col1a1	100759814	0.04	0.05	0.00	0.00	0.00	0.00	5.32	6.41	0.00	0.00	0.00	0.00
collagen, type I, alpha 2	Col1a2	100766269	0.01	0.01	0.00	0.00	0.01	0.00	2.28	1.83	0.00	0.00	1.03	0.93
collagen, type XX, alpha 1	Col20a1	100759714	0.01	0.00	0.01	0.00	0.02	0.00	4.56	3.66	2.48	6.95	5.17	5.61
collagen, type XXII, alpha 1	Col22a1	100766816	0.00	0.00	0.00	0.00	0.00	0.00	0.00	0.00	0.00	0.00	0.00	0.00
collagen, type XXIII, alpha 1	Col23a1	100768117	0.00	0.00	0.00	0.00	0.00	0.00	1.52	0.92	1.24	1.16	1.03	0.93
collagen, type XXV, alpha 1	Col25a1	100758217	0.00	0.00	0.00	0.00	0.00	0.00	0.00	0.00	0.00	0.00	0.00	0.00
collagen, type XXVI, alpha 1	Col26a1	100768133	0.00	0.00	0.00	0.00	0.00	0.00	0.00	0.00	0.00	0.00	0.00	0.00
collagen, type XXVII, alpha 1	Col27a1	100750399	0.92	0.88	0.72	0.43	0.38	0.34	178.49	172.94	151.53	103.14	81.76	69.17
collagen, type XXVIII, alpha 1	Col28a1	100760882	0.00	0.00	0.00	0.00	0.00	0.00	0.00	0.00	0.00	0.00	0.00	0.00
collagen, type II, alpha 1	Col2a1	100755037	0.00	0.00	0.00	0.00	0.00	0.00	5.32	4.58	1.24	5.79	2.07	2.80
collagen, type III, alpha 1	Col3a1	100753589	27.10	26.15	34.24	28.75	16.95	18.88	4217.72	4139.61	5403.08	4482.70	2604.03	2872.24
collagen, type IV, alpha 1	Col4a1	100757740	0.38	0.41	0.40	0.29	0.13	0.24	82.03	86.93	79.49	60.26	33.12	61.69
collagen, type IV, alpha 2	Col4a2	100766914	0.00	0.02	0.00	0.00	0.00	0.00	0.00	1.83	0.00	0.00	1.03	1.87
collagen, type IV, alpha 3 (Goodpasture antigen) binding protein	Col4a3bp	100689038	12.01	11.98	9.84	11.59	12.90	13.28	1425.66	1474.12	1300.46	1402.29	1593.88	1583.33

collagen, type IV, alpha 6	Col4a6	100772869	0.02	0.02	0.00	0.03	0.03	0.04	1.52	1.83	0.00	1.16	3.10	3.74
collagen, type V, alpha 2	Col5a2	100759871	99.09	96.25	81.50	77.70	51.79	54.24	18099.82	17859.62	15153.46	14083.19	9323.16	9605.60
collagen, type V, alpha 3	Col5a3	100763294	0.04	0.08	0.02	0.01	0.01	0.00	7.60	15.56	3.73	2.32	1.03	0.00
collagen, type VI, alpha 1	Col6a1	100755078	1.61	1.68	0.49	0.33	0.01	0.01	199.00	208.63	59.62	41.72	4.14	0.93
collagen, type VI, alpha 2	Col6a2	100754390	0.00	0.01	0.00	0.00	0.00	0.00	0.00	0.92	0.00	0.00	0.00	0.00
collagen, type VI, alpha 3	Col6a3	100771857	0.00	0.00	0.00	0.00	0.01	0.00	0.76	0.00	0.00	0.00	0.00	0.00
collagen, type VI, alpha 5	Col6a5	100770126	0.00	0.00	0.00	0.00	0.00	0.00	0.00	0.00	1.24	0.00	1.03	0.00
collagen, type VI, alpha 6	Col6a6	100769833	0.03	0.02	0.02	0.03	0.03	0.01	9.87	6.41	4.97	10.43	10.35	6.54
collagen, type VII, alpha 1	Col7a1	100689027	4.55	4.00	2.39	1.53	0.83	0.96	1472.75	1303.01	786.24	513.40	289.80	352.37
collagen, type VIII, alpha 1	Col8a1	100755279	2.84	3.12	16.62	18.09	11.33	12.19	454.20	495.03	2654.34	2868.33	1789.49	1943.18
collagen, type VIII, alpha 2	Col8a2	100758166	0.01	0.07	0.04	0.02	0.00	0.01	2.28	4.58	2.48	1.16	1.03	1.87
collagen, type IX, alpha 1	Col9a1	100755811	0.00	0.00	0.00	0.00	0.00	0.00	0.00	0.00	0.00	0.00	0.00	0.00
collagen, type IX, alpha 2	Col9a2	100753027	0.00	0.00	0.00	0.00	0.00	0.02	0.00	0.92	0.00	0.00	0.00	0.93
collagen, type IX, alpha 3	Col9a3	100757317	0.01	0.01	0.00	0.03	0.00	0.01	0.76	1.83	0.00	2.32	0.00	0.93
colorectal cancer associated 2	Colca2	100754197	0.54	0.54	0.46	0.87	0.60	0.23	22.79	19.22	16.15	25.50	15.52	7.48
collectin sub-family member 10 (C-type lectin)	Colec10	100753023	0.00	0.00	0.00	0.00	0.00	0.00	0.00	0.00	0.00	0.00	0.00	0.00
collectin sub-family member 11	Colec11	100765657	0.00	0.00	0.00	0.00	0.00	0.11	0.00	0.00	0.00	0.00	0.00	0.00
collectin sub-family member 12	Colec12	100771704	0.03	0.02	0.02	0.01	0.01	0.05	11.39	10.07	7.45	10.43	20.70	23.37
collagen beta(1-O)galactosyltransferase 1	Colgalt1	100774081	143.33	132.98	150.79	116.55	89.40	97.96	10948.76	10285.89	11719.09	9007.13	6898.18	7566.15
collagen beta(1-O)galactosyltransferase 2	Colgalt2	100764465	0.00	0.00	0.00	0.00	0.00	0.00	0.00	0.00	0.00	0.00	0.00	0.00
collagen-like tail subunit (single strand of homotrimer) of asymmetric acetylcholinesterase	Colq	100767802	0.00	0.03	0.00	0.00	0.00	0.00	0.00	0.92	0.00	0.00	3.10	0.93
copper metabolism (Murr1) domain containing 1	Commd 1	100767564	37.78	37.46	51.66	46.76	39.57	38.38	595.48	613.99	801.15	731.28	628.24	602.86
COMM domain containing 10	Commd 10	100769692	5.40	5.05	6.20	6.93	7.99	6.81	194.44	195.82	222.33	244.53	258.75	229.93
COMM domain containing 2	Commd 2	100770093	24.41	25.08	26.71	25.98	27.50	26.94	859.04	870.20	929.08	886.57	934.59	924.39
COMM domain containing 3	Commd 3	103159468	59.88	62.01	56.54	63.71	64.68	60.05	1151.46	1163.01	1078.13	1163.56	1158.15	1069.26
COMM domain containing 4	Commd 4	100762408	72.40	65.96	65.83	55.44	43.20	54.53	1807.70	1656.21	1656.94	1329.28	1086.74	1330.97
COMM domain containing 6	Commd 6	100754809	42.18	39.65	49.49	48.61	54.26	60.69	1053.48	1003.79	1230.91	1193.69	1330.99	1494.54
COMM domain containing 7	Commd 7	100752819	24.17	22.58	25.51	23.19	12.66	11.77	609.91	592.94	655.82	567.87	315.67	296.29
COMM domain containing 8	Commd 8	100754822	47.21	48.12	41.36	40.37	52.77	46.28	1825.93	1876.73	1614.71	1558.75	2006.84	1775.87
COMM domain containing 9	Commd 9	100753103	29.63	31.18	28.76	28.11	27.30	26.66	938.79	1010.20	912.93	878.46	861.11	841.20
cartilage oligomeric matrix protein	Comp	100766091	0.01	0.00	0.00	0.00	0.00	0.00	0.76	0.00	0.00	0.00	0.00	0.00
catechol-O-methyltransferase	Comt	100770626	161.98	159.08	182.24	173.78	144.70	153.86	5871.24	5866.28	6856.32	6605.84	5761.77	5976.28
catechol-O-methyltransferase domain containing 1	Comtd1	100762102	8.84	8.41	10.07	4.15	1.27	1.96	382.05	384.31	416.10	166.88	63.13	93.47
coatomer protein complex, subunit alpha	Copa	100762124	57.49	55.80	56.31	58.91	61.45	65.12	7557.42	7324.84	7396.63	7626.85	7946.62	8411.09

coatomer protein complex, subunit beta 1	Copb1	100774203	44.98	46.16	44.33	48.17	53.46	52.69	4428.11	4566.93	4392.02	4670.45	5205.98	5148.16
coatomer protein complex, subunit beta 2 (beta prime)	Copb2	100758514	55.64	56.20	29.02	32.36	35.37	34.66	5224.87	5395.95	2767.37	3068.82	3330.59	3217.13
coatomer protein complex, subunit epsilon	Cope	100689412	71.77	71.83	85.24	53.85	34.77	40.68	2061.39	2091.77	2440.70	1513.55	1067.07	1181.42
coatomer protein complex, subunit gamma 1	Copg1	100763679	57.90	55.58	62.99	64.11	59.46	63.51	5291.71	5238.57	5907.37	5948.74	5575.47	5796.82
coatomer protein complex, subunit gamma 2	Copg2	100758528	30.67	28.34	29.32	31.51	35.35	35.94	2560.41	2433.07	2507.77	2642.34	2992.15	2982.53
coordinator of PRMT5, differentiation stimulator	Coprs	100756315	27.90	26.37	26.58	29.65	29.61	33.22	629.66	593.86	603.65	638.56	646.87	708.48
COP9 signalosome subunit 2	Cops2	100760211	31.96	33.06	30.28	35.80	38.35	32.21	3081.45	3171.51	2944.99	3436.20	3637.98	3088.15
COP9 signalosome subunit 3	Cops3	100760692	33.29	33.25	33.57	35.54	36.18	38.24	1391.48	1412.81	1396.11	1419.68	1525.57	1531.92
COP9 signalosome subunit 4	Cops4	100755032	55.12	56.39	54.87	57.63	66.70	62.55	2676.62	2777.13	2756.19	2799.95	3229.16	3024.59
COP9 signalosome subunit 5	Cops5	100767044	65.74	67.16	69.96	73.62	78.49	75.68	2242.16	2260.13	2350.03	2403.60	2591.61	2507.72
COP9 signalosome subunit 6	Cops6	100774513	70.21	71.19	84.53	83.59	75.92	79.70	3114.11	3164.19	3695.21	3563.68	3352.32	3501.27
COP9 signalosome subunit 7A	Cops7a	100756202	86.32	83.01	76.55	65.68	53.16	57.24	5112.46	4909.15	4519.95	3841.82	3175.34	3425.56
COP9 signalosome subunit 7B	Cops7b	100762866	29.54	27.67	23.40	20.97	20.99	21.45	1705.93	1628.76	1383.68	1245.84	1245.09	1271.15
COP9 signalosome subunit 8	Cops8	100767812	131.91	130.10	142.47	143.46	136.82	144.18	3676.93	3790.07	4108.82	3943.80	3738.37	3933.09
coatomer protein complex, subunit zeta 1	Copz1	100769319	87.56	84.44	86.76	86.81	85.18	89.13	4366.59	4191.77	4288.93	4255.55	4213.43	4501.37
coatomer protein complex, subunit zeta 2	Copz2	100761728	0.15	0.05	0.06	0.06	0.21	0.38	3.80	1.83	1.24	1.16	6.21	8.41
coenzyme Q10 homolog A (S. cerevisiae)	Coq10a	100750875	12.51	11.72	19.57	19.02	17.15	17.44	509.65	483.14	788.73	763.73	708.97	713.15
coenzyme Q10 homolog B (S. cerevisiae)	Coq10b	100773426	30.32	26.94	22.06	24.62	25.87	26.03	1494.77	1339.61	1088.07	1209.91	1272.00	1280.50
coenzyme Q2 4-hydroxybenzoate polyprenyltransferase	Coq2	100755903	18.86	20.38	20.92	21.67	24.50	24.94	781.57	856.47	888.09	879.62	985.31	1020.66
coenzyme Q3 methyltransferase	Coq3	100763494	33.29	32.23	11.20	13.33	14.08	12.70	1350.46	1317.65	408.65	496.02	516.46	457.99
coenzyme Q4	Coq4	100766160	12.60	11.42	13.52	12.13	12.68	12.02	439.77	398.95	481.93	425.32	448.15	424.34
coenzyme Q5 homolog, methyltransferase (S. cerevisiae)	Coq5	100750776	30.59	27.90	33.06	34.14	29.15	30.24	1588.96	1494.25	1761.28	1796.33	1560.76	1610.44
coenzyme Q6 monooxygenase	Coq6	100751162	12.65	12.24	9.25	8.30	8.40	9.60	570.41	557.26	455.85	398.67	410.89	465.47
coenzyme Q7 homolog, ubiquinone (yeast)	Coq7	100762279	36.03	34.37	25.37	28.93	25.70	26.63	552.19	538.95	380.08	427.64	380.87	399.10
coenzyme Q9	Coq9	100765748	11.27	10.65	9.90	10.47	10.77	11.87	833.97	803.40	736.56	767.20	778.31	858.03
corin, serine peptidase	Corin	103162946	1.09	0.86	0.52	0.68	0.22	0.28	63.80	55.82	40.99	46.36	16.56	23.37
	Cornifin-A	100760951	51.85	50.22	190.17	130.11	85.32	98.93	992.72	954.38	3607.02	2412.87	1611.47	1850.65
coronin, actin binding protein, 1B	Coro1b	100759932	147.28	142.27	186.39	172.54	151.79	161.98	8085.30	7777.78	10191.32	9323.51	8203.30	8854.13
coronin, actin binding protein, 1C	Coro1c	100754313	47.70	49.65	46.19	50.02	49.56	49.78	4747.88	4967.72	4651.62	4983.36	4909.98	4967.77
coronin, actin binding protein, 2A	Coro2a	100768130	0.00	0.01	0.00	0.01	0.00	0.01	0.00	0.92	0.00	1.16	1.03	0.93
coronin, actin binding protein, 2B	Coro2b	100757780	0.02	0.04	0.00	0.00	0.00	0.00	0.76	2.75	0.00	0.00	0.00	0.00
coronin 6	Coro6	100756161	0.00	0.00	0.00	0.00	0.00	0.00	0.00	0.00	0.00	0.00	0.00	0.00
coactosin-like F-actin binding protein 1	Cotl1	100772395	16.39	16.34	13.46	12.87	9.73	11.10	690.42	689.94	575.09	540.06	416.06	470.14
ceruloplasmin (ferroxidase)	Cp	100753849	0.07	0.05	0.06	0.06	0.05	0.07	7.60	4.58	8.69	6.95	6.21	9.35

carboxypeptidase A1 (pancreatic)	Cpa1	100757667	0.00	0.00	0.00	0.00	0.00	0.00	0.00	0.00	0.00	0.00	0.00	0.00
carboxypeptidase A2 (pancreatic)	Cpa2	100756609	0.00	0.00	0.00	0.00	0.00	0.00	0.00	0.00	0.00	0.00	0.00	0.00
carboxypeptidase A3 (mast cell)	Cpa3	100751382	0.00	0.00	0.00	0.00	0.00	0.00	0.00	0.00	0.00	0.00	1.03	0.00
carboxypeptidase A4	Cpa4	100756900	0.00	0.00	0.00	0.00	0.00	0.00	0.00	0.00	0.00	0.00	0.00	0.00
carboxypeptidase A5	Cpa5	100757381	0.00	0.00	0.00	0.00	0.00	0.00	0.00	0.00	0.00	0.00	0.00	0.00
carboxypeptidase A6	Cpa6	100761681	0.00	0.00	0.00	0.00	0.00	0.00	0.00	0.00	0.00	0.00	0.00	0.00
carboxypeptidase B1 (tissue)	Cpb1	100751091	0.00	0.00	0.00	0.00	0.00	0.00	0.00	0.00	0.00	0.00	0.00	0.00
carboxypeptidase B2 (plasma)	Cpb2	100754304	0.52	0.54	0.54	0.55	0.44	0.57	38.74	38.43	39.75	38.24	43.47	36.45
carboxypeptidase D	Cpd	100757331	45.36	46.97	44.17	49.22	57.58	55.88	10006.93	10443.28	9862.17	10937.89	12664.09	12362.88
carboxypeptidase E	Cpe	100757401	0.00	0.00	0.00	0.00	0.00	0.00	0.00	0.00	0.00	0.00	1.03	0.00
cytoplasmic polyadenylation element binding protein 1	Cpeb1	100759655	28.45	28.88	32.29	32.38	32.10	31.98	2470.78	2534.64	2800.91	2764.02	2747.89	2751.67
cytoplasmic polyadenylation element binding protein 2	Cpeb2	100761763	1.76	1.53	1.19	1.41	1.76	1.82	277.99	232.42	199.98	230.63	285.66	298.16
cytoplasmic polyadenylation element binding protein 3	Cpeb3	100770232	1.33	1.56	1.50	1.52	1.67	1.72	183.05	209.54	203.70	208.61	224.59	227.12
cytoplasmic polyadenylation element binding protein 4	Cpeb4	103158539	2.32	2.61	2.00	2.53	2.73	2.61	365.34	415.43	331.64	395.19	437.80	429.01
cadherin-like and PC-esterase domain containing 1	Cped1	100766134	8.77	9.02	14.64	15.80	15.01	14.41	1285.90	1347.84	2157.50	2301.61	2189.00	2095.53
complexin 1	Cplx1	100771100	0.00	0.00	0.00	0.00	0.00	0.00	0.00	0.00	0.00	0.00	0.00	0.00
complexin 2	Cplx2	100768790	0.00	0.00	0.00	0.00	0.00	0.00	0.00	0.00	0.00	0.00	0.00	0.00
complexin 3	Cplx3	100755334	0.00	0.00	0.00	0.14	0.00	0.00	0.00	0.00	0.00	1.16	0.00	0.00
complexin 4	Cplx4	100755686	0.00	0.00	0.00	0.00	0.00	0.00	0.00	0.00	0.00	0.00	0.00	0.00
carboxypeptidase M	Cpm	100770407	0.29	0.19	0.13	0.18	0.15	0.20	24.31	16.47	11.18	13.91	12.42	14.95
carboxypeptidase N, polypeptide 1	Cpn1	100752048	1.70	1.50	0.65	0.57	0.34	0.35	101.02	96.08	39.75	33.61	19.66	19.63
carboxypeptidase N, polypeptide 2	Cpn2	100770664	0.00	0.00	0.00	0.00	0.00	0.00	0.00	0.00	0.00	0.00	0.00	0.00
copine I	Cpne1	100756430	64.51	63.99	59.22	60.29	66.12	71.00	5616.79	5546.02	5266.45	5274.24	5773.15	6168.82
copine II	Cpne2	100759144	12.19	12.09	13.00	12.38	10.96	11.85	857.52	861.96	917.90	839.06	753.47	822.51
copine III	Cpne3	100757065	9.00	10.63	11.21	12.48	14.28	14.85	603.07	696.34	751.46	819.36	963.57	975.80
copine IV	Cpne4	100763931	0.00	0.00	0.00	0.00	0.02	0.00	0.76	0.00	1.24	0.00	1.03	0.00
copine V	Cpne5	100750402	0.11	0.13	0.03	0.01	0.01	0.05	10.63	12.81	3.73	1.16	1.03	4.67
copine VI (neuronal)	Cpne6	100762909	0.00	0.00	0.00	0.00	0.00	0.00	0.00	0.00	0.00	0.00	0.00	0.00
copine VII	Cpne7	100763257	0.00	0.00	0.00	0.00	0.00	0.00	0.00	0.00	0.00	0.00	0.00	0.00
copine family member IX	Cpne9	100754571	0.00	0.00	0.03	0.03	0.00	0.00	30.38	25.62	18.63	20.86	27.94	31.78
carboxypeptidase O	Cpo	100755961	0.00	0.00	0.00	0.00	0.00	0.00	0.00	0.00	0.00	1.16	0.00	0.00
coproporphyrinogen oxidase	Cpox	100772187	35.20	35.12	34.81	36.10	41.47	41.71	2269.50	2317.78	2254.39	2332.91	2618.52	2669.42
calcineurin-like phosphoesterase domain containing 1	Cpped1	100754184	19.77	19.50	24.29	27.20	26.37	27.49	656.24	646.93	859.52	895.85	922.17	924.39
carboxypeptidase Q	Cpq	100753216	1.50	1.10	6.90	7.85	0.15	0.14	66.08	55.82	315.49	321.02	6.21	5.61
carbamoyl-phosphate synthase 1, mitochondrial	Cps1	100760467	0.04	0.00	0.00	0.00	0.00	0.00	5.32	0.00	0.00	0.00	0.00	0.00
cleavage and polyadenylation specific factor 1, 160kDa	Cpsf1	100761673	24.29	23.56	31.27	28.14	22.20	23.94	3457.42	3327.98	4416.86	3919.47	3116.34	3345.18
cleavage and polyadenylation specific factor 2, 100kDa	Cpsf2	100753441	41.75	43.67	45.61	52.13	51.24	48.56	6800.15	7167.46	7508.41	8491.41	8292.31	7938.15
cleavage and polyadenylation specific factor 3, 73kDa	Cpsf3	100758811	43.76	43.10	49.67	52.56	52.93	54.43	2940.18	2929.94	3377.23	3498.78	3518.95	3570.44

cleavage and polyadenylation specific factor 3-like	Cpsf3l	100769426	76.59	71.35	78.47	76.67	65.84	74.67	4922.57	4725.23	5132.30	4950.91	4314.86	4753.73
cleavage and polyadenylation specific factor 4, 30kDa	Cpsf4	100773929	30.98	29.61	37.19	35.72	24.55	29.80	1477.30	1437.52	1827.11	1715.20	1222.32	1454.35
cleavage and polyadenylation specific factor 4-like	Cpsf4l	100762278	0.00	0.00	0.00	0.00	0.00	0.00	0.76	0.92	0.00	0.00	0.00	2.80
cleavage and polyadenylation specific factor 6, 68kDa	Cpsf6	100773290	21.41	20.99	22.82	21.15	21.24	21.59	2498.88	2503.53	2747.50	2507.90	2500.53	2572.21
cleavage and polyadenylation specific factor 7, 59kDa	Cpsf7	100770766	26.37	27.25	27.75	28.16	28.12	26.84	2232.29	2333.34	2362.45	2455.75	2453.95	2385.28
carnitine palmitoyltransferase 1A (liver)	Cpt1a	100762166	14.87	15.83	10.64	11.31	12.68	14.36	1026.90	1114.51	737.80	813.56	900.44	964.58
carnitine palmitoyltransferase 1B (muscle)	Cpt1b	100770034	0.96	1.06	0.79	1.02	0.87	0.91	105.58	117.12	98.12	115.89	108.67	96.27
carnitine palmitoyltransferase 1C	Cpt1c	100767138	2.41	1.87	1.97	2.39	1.94	1.98	400.28	319.35	358.96	366.22	274.27	262.64
carnitine palmitoyltransferase 2	Cpt2	100766757	26.81	26.68	31.92	31.49	33.98	34.92	1778.08	1772.42	2127.69	2080.26	2230.40	2309.57
carboxypeptidase, vitellogenic-like	Cpvl	100761019	0.00	0.00	0.00	0.00	0.00	0.00	0.00	0.00	0.00	0.00	0.00	0.00
CPX chromosome region, candidate 1	Cpxcr1	100768537	0.00	0.04	0.06	0.00	0.05	0.00	0.76	0.92	1.24	0.00	1.03	0.93
carboxypeptidase X (M14 family), member 1	Cpxm1	100771369	0.01	0.02	0.00	0.02	0.00	0.00	0.76	0.92	0.00	2.32	0.00	0.00
carboxypeptidase X (M14 family), member 2	Cpxm2	100759007	0.00	0.00	0.00	0.00	0.00	0.00	0.00	0.00	0.00	0.00	0.00	0.00
carboxypeptidase Z	Cpz	100766478	0.00	0.00	0.00	0.00	0.00	0.00	0.00	0.00	0.00	0.00	0.00	0.00
complement component (3b/4b) receptor 1-like	Cr1l	100772783	92.19	87.61	97.75	107.02	126.35	124.91	4425.84	4286.02	4652.86	5054.05	6037.08	5994.04
complement component (3d/Epstein Barr virus) receptor 2	Cr2	100773072	0.00	0.00	0.03	0.06	0.01	0.01	1.52	0.92	7.45	9.27	2.07	3.74
cellular retinoic acid binding protein 1	Crabp1	100755827	0.00	0.00	0.00	0.00	0.00	0.00	0.00	0.00	0.00	0.00	0.00	0.00
cellular retinoic acid binding protein 2	Crabp2	100753208	0.00	0.00	0.00	0.00	0.00	0.00	0.00	0.00	1.24	0.00	1.03	1.87
CASP2 and RIPK1 domain containing adaptor with death domain	Cradd	100751661	18.97	21.25	20.98	18.79	15.92	16.53	632.70	647.84	653.34	559.76	447.11	472.94
Crm, cramped-like (Drosophila)	Cramp1l	100764850	3.63	3.29	3.13	3.31	3.42	3.72	722.32	643.27	621.04	660.58	672.74	730.91
carnitine O-acetyltransferase	Crat	100762198	11.36	10.57	13.86	13.31	11.97	12.84	1006.39	948.89	1244.57	1167.03	1056.72	1116.00
crumbs family member 1, photoreceptor morphogenesis associated	Crb1	100765149	0.00	0.01	0.01	0.00	0.00	0.01	0.00	0.92	1.24	0.00	0.00	0.93
crumbs family member 2	Crb2	100774304	0.00	0.00	0.00	0.00	0.00	0.00	0.00	0.00	1.24	0.00	0.00	0.00
crumbs family member 3	Crb3	100762804	0.07	0.18	0.16	0.08	0.00	0.00	3.04	4.58	4.97	1.16	1.03	0.00
cereblon	Crbn	100762708	14.71	16.01	13.86	16.77	17.78	16.77	1058.04	1183.14	1035.90	1209.91	1300.98	1208.53
CGRP receptor component	Crcp	100756386	11.58	10.14	11.52	11.89	12.00	11.44	567.38	500.52	573.84	555.12	597.19	570.15
cAMP responsive element binding protein 1	Creb1	100761582	13.70	13.53	14.31	16.14	17.80	17.00	2675.86	2682.88	2782.27	3117.49	3422.70	3324.62
cAMP responsive element binding protein 3	Creb3	100772940	8.27	7.28	10.45	9.98	10.45	9.64	350.15	322.09	462.06	425.32	456.43	416.86
cAMP responsive element binding protein 3-like 1	Creb3l1	100762192	33.61	31.02	21.33	18.51	14.83	15.80	1923.91	1800.79	1293.01	1148.49	969.78	1009.44
cAMP responsive element binding protein 3-like 2	Creb3l2	100771872	0.00	0.00	0.00	0.00	0.00	0.00	0.00	0.00	0.00	0.00	0.00	0.00
cAMP responsive element binding protein 3-like 3	Creb3l3	100774488	0.08	0.12	0.03	0.18	0.00	0.04	4.56	5.49	3.73	8.11	2.07	2.80

cAMP responsive element binding protein 3-like 4	Creb3l4	100766300	0.00	0.02	0.00	0.00	0.03	0.00	1.52	0.92	0.00	0.00	1.03	1.87
cAMP responsive element binding protein 5	Creb5	100764159	0.00	0.00	0.00	0.00	0.00	0.00	0.00	0.00	0.00	0.00	0.00	0.00
CREB binding protein	Crebbp	100770451	4.80	4.47	4.12	4.42	3.55	3.38	603.83	570.07	526.64	555.12	450.22	434.62
cAMP responsive element binding protein-like 2	Crebl2	100772731	4.24	4.71	5.22	6.81	6.53	6.28	179.25	198.56	227.30	286.25	279.45	275.73
CREB3 regulatory factor	Crebrf	100770049	3.34	3.79	4.78	6.56	5.80	5.44	543.07	560.00	801.15	972.33	886.98	901.02
CREB/ATF bZIP transcription factor	Crebzf	100763322	17.33	18.16	14.75	13.20	14.54	15.71	2394.07	2485.23	2059.38	1827.62	2015.12	2179.65
cellular repressor of E1A-stimulated genes 1	Creg1	100767565	38.03	38.97	44.19	43.71	49.75	51.25	1488.70	1516.21	1711.60	1672.32	1945.77	1977.76
cellular repressor of E1A-stimulated genes 2	Creg2	100759597	0.00	0.00	0.00	0.00	0.00	0.00	0.00	0.00	0.00	0.00	0.00	0.00
cysteine-rich with EGF-like domains 1	Creld1	100757184	40.25	40.46	45.13	39.93	32.68	35.01	2384.19	2375.43	2654.34	2354.93	1917.83	2062.82
cysteine-rich with EGF-like domains 2	Creld2	100689097	81.38	78.95	89.50	84.07	86.89	93.39	2740.42	2751.51	3125.09	2825.45	2932.12	3058.24
cAMP responsive element modulator	Crem	100767867	5.26	4.26	5.61	6.35	6.91	6.07	266.60	239.74	299.34	331.45	368.46	318.72
corticotropin releasing hormone binding protein	Crhbp	100757291	0.16	0.13	0.17	0.20	0.16	0.31	28.10	28.37	32.29	39.40	42.43	50.47
corticotropin releasing hormone receptor 2	Crhr2	100774100	0.00	0.00	0.00	0.00	0.00	0.00	0.00	0.00	0.00	0.00	0.00	0.00
cysteine rich transmembrane BMP regulator 1 (chordin-like)	Crim1	100765449	75.60	73.83	87.09	99.02	112.85	106.91	11279.16	10997.79	13036.94	14680.04	16659.14	15929.58
cysteine-rich protein 1 (intestinal)	Crip1	100754628	48.55	50.51	27.94	26.01	19.88	16.87	485.35	511.50	264.56	246.85	196.65	160.76
cysteine-rich protein 2	Crip2	100771081	132.11	129.09	110.97	82.68	65.44	64.92	3672.37	3497.26	3218.25	2367.67	1699.45	1920.75
cysteine-rich protein 3	Crip3	100754142	0.00	0.00	0.00	0.00	0.00	0.00	0.00	0.00	0.00	0.00	0.00	0.00
cysteine-rich PDZ-binding protein	Cript	100764597	46.53	46.36	43.62	44.38	50.96	55.01	1492.49	1510.72	1404.80	1410.41	1615.61	1726.33
cysteine-rich secretory protein 1	Crisp1	100756652	0.00	0.00	0.00	0.00	0.00	0.00	0.00	0.00	0.00	0.00	0.00	0.00
cysteine-rich secretory protein 2	Crisp2	100764099	0.03	0.00	0.00	0.00	0.00	0.00	1.52	0.00	0.00	0.00	0.00	0.00
cysteine-rich secretory protein LCCL domain containing 1	Crispld1	100764078	0.00	0.00	0.00	0.00	0.00	0.00	0.76	0.00	0.00	0.00	0.00	0.00
cysteine-rich secretory protein LCCL domain containing 2	Crispld2	100758322	0.00	0.07	0.00	0.00	0.00	0.00	0.00	3.66	0.00	0.00	1.03	0.93
v-crk avian sarcoma virus CT10 oncogene homolog	Crk	100762827	132.40	132.68	120.72	124.02	136.10	131.51	3376.15	3600.66	3173.53	3021.30	2835.86	2824.57
v-crk avian sarcoma virus CT10 oncogene homolog-like	Crkl	100752014	23.07	21.76	24.87	26.00	26.35	26.55	3238.68	3069.94	3487.78	3645.96	3739.41	3780.74
cytokine receptor-like factor 1	Crlf1	100767254	0.00	0.00	0.00	0.00	0.00	0.00	0.76	0.00	0.00	1.16	0.00	0.00
cytokine receptor-like factor 3	Crlf3	100771341	8.14	8.63	8.36	10.07	10.00	9.64	634.97	666.14	647.13	774.16	765.89	747.74
crooked neck pre-mRNA splicing factor 1	Crnkl1	100771455	29.17	30.60	32.33	34.72	35.64	33.49	2013.54	2091.77	2238.24	2369.99	2434.29	2292.74
cornulin	Crnn	100758583	0.01	0.01	0.00	0.00	0.00	0.00	2.28	1.83	0.00	0.00	0.00	0.00
ciliary rootlet coiled-coil, rootletin	Crocc	100770958	2.44	2.05	1.79	1.30	0.88	0.91	249.89	257.12	209.91	136.75	94.18	100.94
carnitine O-octanoyltransferase	Crot	100750995	20.57	20.96	19.56	22.03	22.80	21.82	1677.82	1731.24	1624.65	1799.80	1872.29	1792.70
C-reactive protein, pentraxin-related	Crp	100772240	0.00	0.00	0.00	0.00	0.00	0.00	0.00	0.00	0.00	0.00	0.00	0.00
cartilage acidic protein 1	Crtac1	100760959	0.00	0.00	0.00	0.00	0.00	0.00	0.00	0.00	0.00	0.00	0.00	0.00
cytotoxic and regulatory T cell molecule	Crtam	100774440	0.04	0.03	0.04	0.03	0.00	0.05	2.28	1.83	1.24	1.16	0.00	1.87
cartilage associated protein	Crtap	100767159	100.41	99.44	110.32	110.26	111.79	123.77	3304.76	3316.08	3644.28	3592.65	3652.47	3978.89

CREB regulated transcription coactivator 1	Crtc1	100766676	5.32	5.44	5.69	4.04	2.50	3.08	903.85	923.27	972.55	683.76	416.06	510.33
CREB regulated transcription coactivator 2	Crtc2	100751848	24.82	23.74	24.75	20.78	17.04	18.35	1952.02	1857.52	1919.02	1619.01	1345.48	1427.24
CREB regulated transcription coactivator 3	Crtc3	100751622	26.43	26.04	22.99	23.19	21.68	21.19	2275.58	2197.00	2086.71	2031.59	1855.73	1897.38
cone-rod homeobox	Crx	100770134	0.00	0.00	0.00	0.00	0.00	0.00	0.00	0.00	0.00	0.00	0.00	0.00
cryptochrome circadian clock 1	Cry1	100761664	16.92	16.18	15.83	15.62	15.48	17.27	1275.27	1239.87	1216.00	1184.42	1170.57	1274.89
cryptochrome circadian clock 2	Cry2	100774356	8.54	8.37	7.72	6.70	4.70	5.00	708.65	704.58	659.55	570.19	392.26	429.01
crystallin, alpha A	Cryaa	100762633	0.00	0.00	0.00	0.00	0.00	0.00	0.00	0.00	0.00	0.00	0.00	0.93
crystallin, alpha B	Cryab	100772623	0.35	0.29	0.31	0.38	0.47	0.35	13.67	17.39	8.69	22.02	21.73	17.76
crystallin, beta A2	Cryba2	100769887	0.00	0.00	0.00	0.00	0.00	0.00	0.00	0.00	0.00	0.00	0.00	0.00
crystallin, beta A4	Cryba4	100772464	0.00	0.00	0.00	0.00	0.00	0.00	0.76	0.00	1.24	2.32	0.00	0.00
crystallin, beta B1	Crybb1	100772171	0.05	0.16	0.07	0.00	0.00	0.11	0.76	2.75	3.73	0.00	0.00	1.87
crystallin, beta B2	Crybb2	100759533	0.00	0.00	0.00	0.00	0.00	0.00	0.00	0.00	0.00	0.00	0.00	0.00
crystallin, beta B3	Crybb3	100759827	0.00	0.00	0.00	0.00	0.00	0.00	0.00	0.00	0.00	0.00	0.00	0.00
beta-gamma crystallin domain containing 3	Crybg3	100752646	6.86	7.04	7.53	9.20	9.94	9.15	1949.74	2007.58	2166.20	2604.09	2812.06	2608.66
crystallin, gamma A	Cryga	100759243	0.00	0.00	0.00	0.00	0.00	0.00	0.00	0.00	0.00	0.00	0.00	0.00
crystallin, gamma B	Crygb	100759531	0.00	0.00	0.00	0.00	0.00	0.00	0.00	0.00	0.00	0.00	0.00	0.00
crystallin, gamma C	Crygc	100759825	0.00	0.00	0.00	0.00	0.00	0.00	0.00	0.00	0.00	0.00	0.00	0.00
crystallin, gamma N	Crygn	100759221	0.00	0.00	0.00	0.00	0.00	0.18	0.76	1.83	0.00	0.00	0.00	1.87
crystallin, gamma S	Crygs	100765744	0.20	0.08	0.00	0.00	0.10	0.17	2.28	1.83	0.00	0.00	5.17	5.61
crystallin, lambda 1	Cryl1	100763866	9.31	9.24	11.02	11.15	11.95	11.63	446.61	447.45	549.00	527.31	589.94	567.34
crystallin, mu	Crym	100771156	0.00	0.00	0.00	0.00	0.00	0.00	0.76	0.92	0.00	0.00	0.00	0.00
crystallin, zeta (quinone reductase)	Cryz	100768247	14.66	14.72	18.16	17.82	17.43	16.92	593.20	593.86	715.44	701.15	682.06	675.77
crystallin, zeta (quinone reductase)-like 1	Cryzl1	100758122	12.91	13.08	13.08	13.57	14.19	15.12	644.09	670.72	681.91	672.17	719.32	730.91
citrate synthase	Cs	100750580	105.53	107.67	115.27	112.51	118.95	121.55	5650.21	5787.59	6296.14	6087.81	6354.82	6577.27
cold shock domain containing C2, RNA binding	Csdc2	100759782	0.04	0.05	0.00	0.00	0.06	0.00	3.04	0.92	0.00	0.00	1.03	0.00
cold shock domain containing E1, RNA-binding	Csde1	100760894	95.74	95.77	102.17	119.30	168.08	156.74	7752.62	7758.57	8312.05	9510.10	13453.79	12646.08
CSE1 chromosome segregation 1-like (yeast)	Cse1l	100756434	108.79	111.85	107.36	115.59	133.91	128.76	11375.62	11750.86	11280.63	11902.11	13723.92	13295.68
colony stimulating factor 1 (macrophage)	Csf1	100767142	18.55	18.98	14.22	14.81	14.71	15.16	2043.16	2107.32	1536.46	1616.69	1641.49	1686.14
colony stimulating factor 1 receptor	Csf1r	100763221	0.03	0.04	0.01	0.02	0.01	0.00	3.80	4.58	1.24	2.32	2.07	0.00
colony stimulating factor 2 (granulocyte-macrophage)	Csf2	100765221	1.42	1.58	0.36	0.26	0.23	0.82	22.79	25.62	6.21	3.48	4.14	12.15
colony stimulating factor 2 receptor, beta, low-affinity (granulocyte-macrophage)	Csf2rb	100764512	0.00	0.00	0.00	0.00	0.00	0.00	0.00	0.00	0.00	0.00	0.00	0.00
colony stimulating factor 3 (granulocyte)	Csf3	100755744	0.68	0.72	0.17	0.08	0.16	0.16	55.45	58.56	12.42	5.79	12.42	14.02
colony stimulating factor 3 receptor (granulocyte)	Csf3r	100760185	0.00	0.00	0.00	0.00	0.00	0.00	0.00	0.00	0.00	0.00	0.00	0.00
chondroitin sulfate N-acetylgalactosaminyltransferase 1	Csgalnact1	100754969	0.01	0.02	0.00	0.00	0.00	0.00	0.76	1.83	0.00	0.00	0.00	0.00
chondroitin sulfate N-acetylgalactosaminyltransferase 2	Csgalnact2	100770830	7.79	7.74	7.42	9.05	10.04	9.26	365.34	361.44	358.96	423.01	472.99	432.75

c-src tyrosine kinase	Csk	100755922	26.29	25.78	27.00	24.36	20.37	21.37	1618.58	1584.84	1643.28	1520.50	1263.72	1308.54
CUB and Sushi multiple domains 1	Csmd1	100759788	0.00	0.00	0.00	0.00	0.00	0.00	0.00	0.00	0.00	0.00	0.00	0.00
CUB and Sushi multiple domains 2	Csmd2	100755548	0.00	0.00	0.00	0.00	0.00	0.00	0.00	0.00	0.00	0.00	0.00	0.00
CUB and Sushi multiple domains 3	Csmd3	100774887	0.01	0.00	0.00	0.00	0.00	0.00	2.28	0.00	0.00	1.16	0.00	0.93
casein beta	Csn2	100755914	0.00	0.00	0.00	0.00	0.00	0.00	0.00	0.00	0.00	0.00	0.00	0.00
casein kappa	Csn3	100752988	0.00	0.00	0.00	0.00	0.00	0.00	0.00	0.00	0.00	0.00	0.00	0.00
casein kinase 1, alpha 1	Csnk1a 1	100758298	50.53	49.17	44.46	48.84	48.80	49.70	1989.99	1985.62	1769.97	1893.68	1871.26	1828.21
casein kinase 1, delta	Csnk1d	100751124	92.22	86.33	37.13	35.58	25.45	27.87	9354.49	8799.88	3834.32	3606.56	2515.02	2824.57
casein kinase 1, epsilon	Csnk1e	100766643	30.73	29.04	32.85	30.51	23.18	25.57	2561.17	2469.68	2773.58	2554.26	1911.62	2123.57
casein kinase 1, gamma 1	Csnk1g 1	100774663	15.45	15.81	14.26	15.92	15.38	16.13	1592.75	1650.72	1491.75	1652.62	1614.58	1678.67
casein kinase 1, gamma 2	Csnk1g 2	100758847	21.83	23.12	26.96	16.73	12.78	12.39	1175.01	1275.56	1438.34	908.59	671.71	681.37
casein kinase 1, gamma 3	Csnk1g 3	100773714	6.01	5.90	6.10	6.63	7.38	7.10	739.03	728.37	765.13	812.40	908.72	829.99
casein kinase 2, alpha 1 polypeptide	Csnk2a 1	100759365	118.13	118.86	110.65	113.05	124.66	121.02	14429.73	14585.63	13620.72	13744.79	15002.13	14567.76
casein kinase 2, alpha prime polypeptide	Csnk2a 2	100763424	54.54	54.34	58.06	59.51	59.57	59.15	5158.03	5177.26	5594.36	5599.90	5526.83	5511.75
casein kinase 2, beta polypeptide	Csnk2b	100763034	101.59	103.59	149.02	140.50	112.12	116.89	2147.98	2196.08	3173.53	2940.18	2368.05	2447.90
chondroitin sulfate proteoglycan 4	Cspg4	100760376	16.01	14.82	21.48	17.93	13.30	13.47	3781.75	3524.71	5137.27	4262.51	3153.60	3209.66
chondroitin sulfate proteoglycan 5 (neuroglycan C)	Cspg5	100768532	0.04	0.02	0.11	0.06	0.09	0.07	3.04	0.92	6.21	8.11	5.17	5.61
centrosome and spindle pole associated protein 1	Cspp1	100751044	2.25	2.50	1.80	2.02	2.80	2.94	308.37	335.82	265.81	283.94	406.75	410.32
uncharacterized LOC100689233	Csr1	100689233	0.24	0.10	0.26	0.00	0.11	0.10	2.28	0.92	0.00	0.00	0.00	0.93
cysteine-serine-rich nuclear protein 1	Csrnp1	100752750	2.99	3.21	1.91	1.32	1.28	1.11	265.08	287.32	173.89	118.21	112.81	100.01
cysteine-serine-rich nuclear protein 2	Csrnp2	100756497	1.80	2.14	2.22	1.75	2.16	2.04	224.06	266.27	283.20	225.99	264.96	264.51
cysteine-serine-rich nuclear protein 3	Csrnp3	100753527	0.02	0.00	0.00	0.00	0.00	0.00	0.76	0.00	0.00	0.00	0.00	0.00
cysteine and glycine-rich protein 1	Csrp1	100756325	175.22	173.67	218.79	215.34	198.34	217.50	8906.36	8817.26	11131.58	10823.15	9948.29	10994.52
cysteine and glycine-rich protein 2	Csrp2	100764077	32.54	31.59	21.97	19.91	28.07	26.58	736.75	732.94	498.08	465.89	626.17	622.49
CSRP2 binding protein	Csrp2bp	100767721	11.84	11.15	13.84	14.78	13.00	13.28	1168.93	1152.03	1434.61	1500.80	1373.43	1360.88
cysteine and glycine-rich protein 3 (cardiac LIM protein)	Csrp3	100761159	0.00	0.08	0.11	0.00	0.00	0.00	0.76	0.92	1.24	0.00	0.00	0.00
cystatin 11	Cst11	100763132	0.00	0.00	0.00	0.00	0.00	0.00	0.00	0.00	0.00	0.00	0.00	0.00
cystatin E/M	Cst6	100764786	0.09	0.09	0.19	0.22	0.28	0.27	6.08	6.41	9.94	12.75	14.49	16.82
cystatin F (leukocystatin)	Cst7	100765994	0.00	0.08	0.00	0.00	0.00	0.00	2.28	0.92	0.00	0.00	0.00	0.00
cystatin 8 (cystatin-related epididymal specific)	Cst8	100766873	0.00	0.00	0.00	0.00	0.00	0.00	0.00	0.00	0.00	0.00	0.00	0.00
cystatin A (stefin A)	Csta	100767084	0.00	0.00	0.00	0.00	0.00	0.00	0.00	0.00	0.00	0.00	0.00	0.00
cystatin B (stefin B)	Cstb	100768687	153.87	146.98	140.54	125.41	139.86	138.40	2135.06	2067.98	1936.41	1697.82	1901.27	1851.58
cleavage stimulation factor, 3' pre-RNA, subunit 1, 50kDa	Cstf1	100761066	38.72	36.68	32.86	32.55	29.32	31.88	2205.70	2108.24	1874.31	1876.29	1682.89	1854.38
cleavage stimulation factor, 3' pre-RNA, subunit 2, 64kDa	Cstf2	100770425	30.24	31.67	28.05	29.92	30.76	30.07	3206.77	3367.32	2974.80	3119.81	3196.04	3140.49

cleavage stimulation factor, 3' pre-RNA, subunit 2, 64kDa, tau variant	Cstf2t	100768909	22.21	21.89	17.98	18.41	18.38	18.23	2274.82	2245.49	1861.89	1898.31	1877.47	1883.36
cleavage stimulation factor, 3' pre-RNA, subunit 3, 77kDa	Cstf3	100772127	16.07	16.41	16.24	16.98	18.13	18.28	1441.61	1542.75	1531.49	1556.43	1610.44	1605.76
cystatin-like 1	Cstl1	100766245	0.00	0.00	0.00	0.00	0.00	0.00	0.00	0.00	0.00	0.00	0.00	0.00
cancer/testis antigen 83	Ct83	103159767	0.00	0.00	0.00	0.00	0.00	0.00	0.00	0.00	0.00	0.00	0.00	0.00
C-terminal binding protein 1	Ctbp1	100766703	42.39	43.09	37.47	28.21	23.32	25.66	2842.96	2947.32	2562.43	1929.60	1624.93	1762.79
C-terminal binding protein 2	Ctbp2	100769412	37.67	36.93	40.45	40.13	32.51	33.88	2285.45	2264.71	2477.96	2476.61	2014.08	2103.94
chitobiase, di-N-acetyl-	Ctbs	103162615	10.17	9.81	10.81	11.40	16.50	15.14	448.13	430.98	489.38	514.56	714.14	672.03
CTS telomere maintenance complex component 1	Ctc1	100752986	4.25	4.18	4.72	4.80	4.05	4.26	578.77	577.39	652.10	658.27	566.14	583.23
CCCTC-binding factor (zinc finger protein)	Ctcf	100763233	48.13	47.85	43.98	44.93	46.64	44.13	5072.20	5038.17	4649.13	4727.23	4888.24	4645.31
CCCTC-binding factor (zinc finger protein)-like	Ctcfl	100759040	0.00	0.00	0.00	0.00	0.04	0.00	0.00	0.00	0.00	0.00	2.07	0.00
CTD nuclear envelope phosphatase 1	Ctdnep1	100762001	55.25	53.57	69.22	71.99	72.95	74.45	1895.81	1819.09	2372.39	2429.10	2504.67	2543.24
CTD (carboxy-terminal domain, RNA polymerase II, polypeptide A) phosphatase, subunit 1	Ctdp1	100764255	13.57	13.64	14.01	12.86	11.21	13.13	1558.57	1574.77	1644.52	1515.87	1254.40	1460.89
CTD (carboxy-terminal domain, RNA polymerase II, polypeptide A) small phosphatase 1	Ctdsp1	100751900	34.38	33.94	38.75	32.35	26.59	29.52	2138.86	2122.88	2453.12	1981.75	1581.46	1782.42
CTD (carboxy-terminal domain, RNA polymerase II, polypeptide A) small phosphatase 2	Ctdsp2	100765556	27.61	27.84	25.87	27.38	26.51	29.36	2980.43	2972.94	2835.68	3014.35	2852.42	3193.77
CTD (carboxy-terminal domain, RNA polymerase II, polypeptide A) small phosphatase-like	Ctdspl	100754751	6.00	6.19	10.10	10.63	10.31	10.11	824.10	846.41	1398.59	1448.65	1379.64	1380.51
CTD (carboxy-terminal domain, RNA polymerase II, polypeptide A) small phosphatase like 2	Ctdspl2	100752292	7.75	7.89	8.82	9.84	10.85	11.01	886.38	913.20	1002.36	1104.45	1239.91	1250.59
connective tissue growth factor	Ctgf	100750810	14.02	13.05	2.98	2.51	3.68	4.48	906.89	852.81	198.73	162.25	235.98	289.75
cystathionase (cystathionine gamma-lyase)	Cth	100752127	0.05	0.00	0.00	0.02	0.09	0.00	2.28	0.92	0.00	1.16	4.14	0.00
collagen triple helix repeat containing 1	Cthrc1	103163281	0.00	0.00	0.00	0.00	0.00	0.00	0.00	0.00	1.24	0.00	1.03	0.00
CBP80/20-dependent translation initiation factor	Ctif	100754827	3.16	3.99	3.96	3.09	2.73	2.30	170.90	221.44	202.46	170.36	151.11	139.27
cytotoxic T-lymphocyte-associated protein 4	Ctla4	100765782	0.00	0.00	0.00	0.00	0.00	0.00	0.00	0.00	0.00	0.00	0.00	0.00
catenin (cadherin-associated protein), alpha 1, 102kDa	Ctnna1	100754399	43.75	45.02	44.30	45.52	49.05	50.40	4602.81	4753.60	4743.53	4778.23	5122.15	5290.23
catenin (cadherin-associated protein), alpha 2	Ctnna2	100766706	3.83	3.91	6.47	6.73	8.63	8.72	281.79	289.15	473.24	487.91	620.99	636.51
catenin (cadherin-associated protein), alpha 3	Ctnna3	100763869	0.04	0.04	0.00	0.06	0.00	0.00	3.80	7.32	0.00	3.48	0.00	0.00
catenin (cadherin-associated protein), alpha-like 1	Ctnnal1	100766294	5.72	6.55	6.41	6.40	10.09	9.94	568.14	653.33	632.22	632.77	999.80	981.40
catenin (cadherin-associated protein), beta 1, 88kDa	Ctnnb1	100773307	98.94	92.73	89.87	88.08	83.95	90.28	9690.96	9170.47	8774.10	8528.49	8154.66	8692.43
catenin, beta interacting protein 1	Ctnnbip1	100760454	11.29	12.15	11.30	8.63	8.24	7.46	239.25	257.12	244.69	184.27	163.53	152.35

catenin, beta like 1	Ctnnbl1	100764320	71.27	69.64	66.66	68.77	68.07	71.72	3956.44	3881.57	3718.81	3736.36	3719.74	3929.35
catenin (cadherin-associated protein), delta 1	Ctnnd1	100753529	29.22	29.76	31.97	34.27	37.55	36.46	4553.44	4671.25	5117.40	5401.73	5942.89	5902.44
catenin (cadherin-associated protein), delta 2	Ctnnd2	100752408	0.00	0.00	0.00	0.00	0.00	0.00	0.00	0.00	0.00	0.00	0.00	0.00
cystinosin, lysosomal cystine transporter	Ctns	100764451	4.29	5.04	5.38	6.70	6.66	4.41	161.78	189.41	188.80	220.19	231.84	192.54
CTP synthase 1	Ctps1	100689101	99.73	101.49	106.95	112.52	107.65	105.95	8049.60	8215.17	8708.27	8994.38	8647.31	8543.82
CTP synthase 2	Ctps2	100758964	31.23	33.76	20.76	25.34	24.28	23.50	960.06	1040.39	654.58	760.25	712.07	685.11
CTR9, Paf1/RNA polymerase II complex component	Ctr9	100757530	45.45	46.01	52.67	54.50	54.73	56.84	6142.39	6315.56	7241.37	7376.53	7382.56	7602.60
chymotrypsin C (caldecrin)	Ctrc	100752962	0.00	0.00	0.00	0.00	0.00	0.00	0.00	0.00	0.00	0.00	0.00	0.00
chymotrypsin-like	Ctrl	100756444	0.06	0.10	0.14	0.24	0.19	0.24	12.15	8.24	21.12	28.97	16.56	30.84
cathepsin A	Ctsa	100772055	180.95	170.47	195.19	182.81	161.83	179.87	9343.09	8927.98	10154.06	9300.33	8317.15	9164.44
cathepsin B	Ctsb	100756995	156.56	148.50	164.03	170.82	173.73	188.71	8506.84	8152.03	9043.63	9272.52	9400.78	10180.42
cathepsin C	Ctsc	100769144	0.00	0.00	0.00	0.00	0.00	0.00	0.00	0.00	0.00	0.00	0.00	0.00
cathepsin D	Ctsd	100766628	128.85	122.98	165.99	161.24	146.70	169.49	7108.53	6867.33	9287.08	8895.87	8128.78	9346.70
cathepsin E	Ctse	100757212	0.00	0.00	0.00	0.00	0.00	0.00	0.76	0.00	0.00	1.16	1.03	0.00
cathepsin F	Ctsf	100756958	22.12	21.49	19.94	17.42	13.47	15.44	1386.92	1409.15	1290.53	1185.58	877.67	972.99
cathepsin H	Ctsh	100756691	26.71	27.59	28.87	29.55	31.34	30.76	1082.34	1120.00	1166.32	1175.14	1239.91	1217.87
cathepsin K	Ctsk	100765407	0.44	0.41	0.30	0.30	0.38	0.27	32.66	35.69	26.08	23.18	35.19	28.04
cathepsin O	Ctso	100767833	2.41	2.62	2.27	2.62	3.39	3.21	157.22	167.45	147.81	163.41	212.17	206.56
cathepsin S	Ctss	100751823	0.05	0.00	0.00	0.00	0.00	0.00	1.52	0.00	0.00	0.00	0.00	0.00
cathepsin W	Ctsw	100767305	0.11	0.31	0.10	0.28	0.22	0.22	9.11	20.13	9.94	20.86	16.56	13.09
cathepsin Z	Ctsz	100689392	380.31	357.52	349.31	312.93	302.05	324.71	13466.63	12695.18	12381.12	10976.13	10657.25	11480.55
cortactin	Cttn	100772651	116.23	113.49	56.30	57.16	52.39	57.73	10875.08	10843.15	5422.95	5408.68	4921.36	5377.15
cortactin binding protein 2	Cttnbp2	100754789	0.56	0.64	0.40	0.41	0.53	0.50	97.98	112.55	69.56	75.33	100.39	90.66
CTTNBP2 N-terminal like	Cttnbp2 nl	100757568	16.39	15.61	15.91	17.16	16.55	16.77	2331.79	2251.90	2290.41	2459.23	2352.52	2403.04
cytosolic thiouridylase subunit 1	Ctu1	100764314	9.19	8.82	8.32	8.15	6.93	7.72	801.31	771.37	730.35	710.42	606.50	668.29
cytosolic thiouridylase subunit 2 homolog (S. pombe)	Ctu2	100765955	21.64	19.49	25.35	22.40	15.39	18.29	1154.50	1050.46	1317.85	1168.19	810.39	940.28
cortexin 2	Ctxn2	100762346	0.00	0.00	0.00	0.00	0.00	0.00	0.00	0.00	0.00	0.00	0.00	0.00
cortexin 3	Ctxn3	100753891	0.00	0.00	0.00	0.00	0.00	0.00	0.00	0.00	0.00	0.00	0.00	0.00
cubilin (intrinsic factor-cobalamin receptor)	Cubn	100774474	0.20	0.26	0.18	0.24	0.26	0.26	47.09	60.39	42.23	64.90	66.24	72.90
CUE domain containing 1	Cuedc1	100765982	7.87	7.35	5.15	3.90	3.19	3.02	401.04	399.87	269.53	215.56	184.23	174.78
CUE domain containing 2	Cuedc2	100750415	88.09	88.50	105.84	98.78	80.60	90.69	2691.05	2714.90	3249.30	2969.15	2443.60	2738.58
cullin 1	Cul1	100757763	52.34	53.03	54.08	61.87	61.18	58.25	4559.51	4677.65	4727.38	5326.40	5246.35	5036.00
cullin 2	Cul2	100767582	41.11	42.54	39.86	44.95	47.87	44.71	3525.78	3701.31	3512.62	3830.23	4099.58	3799.43
cullin 3	Cul3	100760346	42.24	41.53	32.32	38.47	38.44	35.60	4862.57	4820.40	3756.07	4406.21	4394.55	4045.25
cullin 4A	Cul4a	100760883	38.30	38.38	40.52	42.59	44.49	46.85	3777.95	3796.47	4105.10	4235.85	4428.71	4598.57
cullin 4B	Cul4b	100763989	19.34	20.00	20.15	23.93	25.22	23.04	1514.52	1581.18	1597.32	1861.23	1961.30	1831.02
cullin 5	Cul5	100770463	42.26	41.94	44.18	50.58	51.47	46.59	7253.60	7209.55	7612.75	8595.71	8642.14	7874.59
cullin 7	Cul7	100771595	19.40	18.44	27.19	23.29	18.89	20.92	3428.56	3310.59	4859.04	4186.02	3404.07	3762.05

cullin 9	Cul9	100753842	0.06	0.06	0.02	0.01	0.01	0.00	14.43	15.56	3.73	3.48	4.14	0.93
cutA divalent cation tolerance homolog (E. coli)	Cuta	100774038	51.73	44.53	57.57	51.23	42.54	47.13	924.36	821.70	937.78	820.52	729.67	817.84
cutC copper transporter	Cutc	100752859	13.30	12.29	9.95	11.42	12.94	11.54	496.74	463.01	387.53	433.44	476.09	429.95
cut-like homeobox 1	Cux1	100767555	1.94	1.86	1.28	1.12	1.00	0.78	330.40	299.22	231.03	206.29	186.30	161.70
cut-like homeobox 2	Cux2	100773679	0.01	0.00	0.00	0.00	0.00	0.00	1.52	0.00	0.00	0.00	1.03	0.00
CUB and zona pellucida-like domains 1	Cuzd1	100765370	0.00	0.00	0.00	0.02	0.00	0.00	0.00	0.92	0.00	1.16	0.00	0.00
CWC15 spliceosome-associated protein homolog (S. cerevisiae)	Cwc15	100775065	120.96	130.42	132.35	141.46	135.89	129.66	2538.38	2714.90	2731.35	2845.15	2785.15	2668.48
CWC22 spliceosome-associated protein homolog (S. cerevisiae)	Cwc22	100768134	47.54	45.93	40.27	46.51	44.85	42.50	4450.90	4323.53	3856.68	4402.74	4254.83	3973.28
CWC25 spliceosome-associated protein homolog (S. cerevisiae)	Cwc25	100756522	35.50	36.00	20.09	21.57	19.97	21.23	2466.98	2574.90	1500.44	1546.00	1450.02	1491.73
CWC27 spliceosome-associated protein homolog (S. cerevisiae)	Cwc27	100760125	21.93	22.30	21.65	23.11	24.64	20.95	1974.04	2024.97	1972.43	2059.40	2185.89	1889.90
CWF19-like 1, cell cycle control (S. pombe)	Cwf19l1	100751475	10.87	11.07	12.75	13.59	13.43	13.58	644.09	656.99	762.64	810.08	816.60	834.66
CWF19-like 2, cell cycle control (S. pombe)	Cwf19l2	100761030	6.52	6.35	6.44	7.30	8.35	8.03	822.58	802.48	813.57	925.98	1052.58	1022.53
cell wall biogenesis 43 C-terminal homolog (S. cerevisiae)	Cwh43	100764522	0.02	0.04	0.37	0.00	0.04	0.31	1.52	1.83	1.24	0.00	5.17	1.87
chemokine (C-X3-C motif) ligand 1	Cx3cl1	100760591	0.00	0.00	0.04	0.00	0.00	0.00	0.00	0.00	1.24	0.00	0.00	0.93
chemokine (C-X3-C motif) receptor 1	Cx3cr1	100753050	0.00	0.00	0.00	0.00	0.00	0.00	0.00	0.00	0.00	0.00	0.00	0.00
coxsackie virus and adenovirus receptor	Cxadr	100755073	0.00	0.02	0.00	0.00	0.00	0.00	0.00	0.92	0.00	0.00	1.03	0.00
chemokine (C-X-C motif) ligand 1	Cxcl1	100689059	6.94	8.24	11.84	10.89	20.95	17.83	172.42	204.05	289.41	272.35	501.97	432.75
chemokine (C-X-C motif) ligand 10	Cxcl10	100770195	0.20	0.09	0.20	0.23	0.24	0.09	6.84	3.66	7.45	6.95	8.28	3.74
chemokine (C-X-C motif) ligand 11	Cxcl11	100771051	0.00	0.11	0.00	0.00	0.00	0.00	1.52	2.75	0.00	0.00	0.00	0.00
chemokine (C-X-C motif) ligand 12	Cxcl12	100759082	0.23	0.22	0.00	0.00	0.00	0.00	12.15	11.90	0.00	0.00	0.00	0.00
chemokine (C-X-C motif) ligand 13	Cxcl13	100773550	0.15	0.18	0.00	0.00	0.00	0.00	3.04	1.83	0.00	0.00	0.00	0.00
chemokine (C-X-C motif) ligand 14	Cxcl14	100754406	0.00	0.00	0.00	0.00	0.00	0.00	0.00	0.00	0.00	0.00	0.00	0.00
chemokine (C-X-C motif) ligand 16	Cxcl16	100751127	7.31	7.21	6.71	7.02	8.44	7.33	442.81	427.32	407.40	419.53	488.51	435.56
chemokine (C-X-C motif) ligand 17	Cxcl17	103161592	0.00	0.00	0.00	0.00	0.00	0.00	0.00	0.00	0.00	0.00	0.00	0.00
chemokine (C-X-C motif) ligand 3	Cxcl3	100689100	0.96	1.28	1.07	1.17	0.42	0.25	31.90	38.43	32.29	35.93	12.42	7.48
chemokine (C-X-C motif) ligand 6	Cxcl6	100759933	0.00	0.00	0.00	0.00	0.00	0.00	0.00	0.00	0.00	0.00	0.00	0.00
chemokine (C-X-C motif) ligand 9	Cxcl9	100770763	0.09	0.06	0.00	0.00	0.00	0.00	1.52	1.83	0.00	1.16	0.00	1.87
chemokine (C-X-C motif) receptor 2	Cxcr2	100755640	0.00	0.00	0.00	0.00	0.00	0.00	0.00	0.00	0.00	0.00	0.00	0.00
chemokine (C-X-C motif) receptor 3	Cxcr3	100769241	0.00	0.03	0.00	0.00	0.00	0.00	0.76	0.92	0.00	0.00	0.00	0.00
chemokine (C-X-C motif) receptor 4	Cxcr4	100767733	0.00	0.00	0.00	0.00	0.00	0.00	0.00	0.00	0.00	0.00	0.00	0.00
chemokine (C-X-C motif) receptor 5	Cxcr5	100770604	0.00	0.03	0.00	0.00	0.00	0.00	0.76	1.83	0.00	0.00	0.00	0.00
chemokine (C-X-C motif) receptor 6	Cxcr6	103160301	0.00	0.00	0.00	0.00	0.00	0.00	0.00	0.92	0.00	0.00	0.00	1.87
CXXC finger protein 1	Cxxc1	100770455	14.52	13.66	15.89	15.67	13.99	15.46	765.62	741.18	848.35	826.31	749.33	811.29
CXXC finger protein 4	Cxxc4	100750942	0.37	0.39	0.07	0.13	0.11	0.04	31.90	37.52	4.97	9.27	10.35	4.67
CXXC finger protein 5	Cxxc5	100758671	2.04	1.96	2.12	1.51	1.69	1.96	95.70	96.99	110.55	82.28	79.69	100.94
	Cyclin-K	100753370	0.00	0.06	0.00	0.00	0.00	0.00	0.00	2.75	0.00	0.00	0.00	0.00
cytoplasmic FMR1 interacting protein 1	Cyfip1	100759530	31.78	30.86	29.48	32.42	32.61	32.88	5042.58	4966.80	4796.94	5258.02	5203.91	5162.18

cytoplasmic FMR1 interacting protein 2	Cyfip2	100767787	0.01	0.00	0.00	0.00	0.00	0.01	1.52	0.00	0.00	0.00	0.00	2.80
cytoglobin	Cygb	100762644	0.04	0.00	0.00	0.00	0.00	0.05	0.76	0.92	0.00	1.16	1.03	0.93
cysteine/histidine-rich 1	Cyhr1	100755112	19.43	18.10	22.31	20.03	14.15	16.45	1065.63	958.96	1170.05	1110.25	781.41	921.58
cylicin, basic protein of sperm head cytoskeleton 1	Cylc1	100755781	0.00	0.00	0.02	0.02	0.00	0.00	0.00	0.00	1.24	1.16	0.00	0.00
cylicin, basic protein of sperm head cytoskeleton 2	Cylc2	100763548	0.00	0.00	0.00	0.00	0.00	0.00	0.00	0.00	0.00	0.00	0.00	0.00
cylindromatosis (turban tumor syndrome)	Cyld	100758070	8.84	9.67	9.31	10.91	13.16	12.82	1105.13	1216.99	1170.05	1362.89	1646.66	1596.42
Cytochrome P-450 2A14	Cyp2a14	100689033	0.00	0.00	0.00	0.00	0.00	0.00	0.00	0.00	0.00	0.00	0.00	0.00
cytochrome P450 2A15	Cyp2a15	100689035	0.00	0.00	0.00	0.00	0.00	0.00	0.00	0.00	0.00	1.16	0.00	0.00
cytochrome P450 2A17	Cyp2a17	100689034	0.00	0.00	0.00	0.00	0.00	0.00	0.00	0.00	0.00	0.00	0.00	0.00
cytochrome P450, family 7, subfamily a, polypeptide 1	Cyp7a1	100689275	0.00	0.00	0.00	0.00	0.00	0.00	0.00	0.00	0.00	0.00	0.00	0.00
cysteine-rich, angiogenic inducer, 61	Cyr61	100766753	21.86	22.12	15.13	12.56	11.37	12.78	1265.39	1284.71	880.64	715.05	667.57	755.21
cysteinyl leukotriene receptor 1	Cysltr1	100768254	0.00	0.00	0.00	0.00	0.00	0.00	0.00	0.00	0.00	0.00	0.00	0.00
cysteinyl leukotriene receptor 2	Cysltr2	100752628	0.03	0.00	0.00	0.00	0.00	0.00	1.52	0.00	0.00	0.00	0.00	0.00
cysteine-rich transmembrane module containing 1	Cystm1	103162545	10.45	11.23	12.77	12.38	12.28	11.02	160.26	173.86	195.01	186.59	186.30	172.91
cytohesin 1	Cyth1	100768404	5.43	5.35	5.16	3.81	3.71	3.92	483.83	484.97	468.27	338.40	335.34	346.76
cytohesin 2	Cyth2	100774811	10.57	10.10	11.05	10.81	10.47	10.66	426.86	420.92	440.94	432.28	436.76	418.73
cytohesin 3	Cyth3	100756495	8.33	8.60	9.22	10.41	10.11	10.58	922.08	959.87	1049.56	1179.78	1120.89	1173.01
cytohesin 4	Cyth4	100766937	0.00	0.00	0.00	0.00	0.00	0.00	0.00	0.00	0.00	1.16	0.00	0.93
cytohesin 1 interacting protein	Cytip	100758851	0.00	0.00	0.00	0.04	0.04	0.04	0.00	0.00	0.00	1.16	1.03	0.93
cytokine-like 1	Cytl1	100772210	0.00	0.00	0.00	0.00	0.00	0.00	0.00	0.00	0.00	0.00	0.00	0.00
cysteine/tyrosine-rich 1	Cyyr1	100757225	0.00	0.00	0.00	0.00	0.00	0.00	0.00	0.00	0.00	0.00	0.00	0.00
D-2-hydroxyglutarate dehydrogenase	D2hgdh	100773592	13.10	12.82	12.89	12.47	9.98	10.51	988.92	974.51	998.64	941.04	749.33	795.40
dishevelled associated activator of morphogenesis 1	Daam1	100765004	15.44	14.54	11.61	11.26	14.86	15.42	584.85	596.60	498.08	511.08	527.84	529.02
dishevelled associated activator of morphogenesis 2	Daam2	100764704	0.00	0.00	0.00	0.00	0.00	0.00	0.00	0.00	0.00	0.00	0.00	0.00
Dab, reelin signal transducer, homolog 1 (Drosophila)	Dab1	100773659	0.00	0.00	0.00	0.00	0.00	0.00	0.00	0.00	0.00	0.00	0.00	0.00
Dab, mitogen-responsive phosphoprotein, homolog 2 (Drosophila)	Dab2	100774674	20.99	21.39	20.94	24.47	34.10	31.88	2035.57	2074.38	2068.07	2379.26	3295.40	3106.84
DAB2 interacting protein	Dab2ip	100752536	11.24	11.08	12.01	9.87	7.45	7.92	2115.32	2120.13	2321.46	1916.85	1436.56	1540.34
dachshund family transcription factor 2	Dach2	100752459	0.00	0.03	0.00	0.00	0.00	0.00	0.00	0.92	0.00	0.00	0.00	0.00
dishevelled-binding antagonist of beta-catenin 1	Dact1	100768384	0.05	0.02	0.03	0.00	0.04	0.02	2.28	1.83	1.24	0.00	3.10	0.93
dishevelled-binding antagonist of beta-catenin 2	Dact2	100774769	0.05	0.06	0.00	0.00	0.00	0.00	4.56	5.49	0.00	0.00	0.00	0.93
defender against cell death 1	Dad1	100767387	131.20	126.15	120.82	116.42	107.33	109.62	2078.86	1992.94	1917.78	1792.85	1700.48	1700.16
dystroglycan 1 (dystrophin-associated glycoprotein 1)	Dag1	100762216	25.92	26.77	32.49	32.12	30.45	31.49	3906.31	4036.21	4941.02	4863.99	4560.15	4772.42
diacylglycerol lipase, alpha	Dagla	100756392	1.83	1.96	1.49	1.00	0.93	0.99	293.18	311.11	238.48	158.77	144.90	154.22

diacylglycerol lipase, beta	Daglb	100765603	2.94	3.16	3.33	3.23	3.17	3.68	255.21	259.87	275.74	263.07	265.99	310.31
dihydroxyacetone kinase 2 homolog (S. cerevisiae)	Dak	100765605	11.51	10.09	10.14	9.58	8.47	8.99	780.81	681.70	707.99	654.79	574.42	620.62
DALR anticodon binding domain containing 3	Dalrd3	100764409	12.92	11.02	14.82	13.52	12.93	13.25	527.88	479.48	648.37	580.62	578.56	602.86
DAN domain family member 5, BMP antagonist	Dand5	100764256	0.29	0.33	0.17	0.10	0.14	0.03	13.67	15.56	12.42	5.79	5.17	1.87
D-amino-acid oxidase	Dao	100689280	0.00	0.00	0.00	0.00	0.00	0.00	0.00	0.00	0.00	0.00	0.00	0.00
death associated protein 3	Dap3	100771966	80.92	77.83	80.10	80.43	78.20	78.13	3518.95	3510.98	3534.98	3468.65	3338.87	3400.33
death-associated protein kinase 1	Dapk1	100766355	0.14	0.16	0.06	0.04	0.06	0.10	40.26	44.84	24.84	30.13	19.66	24.30
death-associated protein kinase 2	Dapk2	100773012	0.02	0.06	0.03	0.03	0.02	0.02	0.76	2.75	1.24	1.16	1.03	0.93
death-associated protein kinase 3	Dapk3	100773624	27.29	26.00	28.33	16.82	11.93	11.71	1020.06	978.17	1048.32	643.20	456.43	441.16
death associated protein-like 1	Dapl1	100771189	0.31	0.19	0.00	0.00	0.00	0.00	2.28	2.75	0.00	0.00	0.00	0.00
dual adaptor of phosphotyrosine and 3-phosphoinositides	Dapp1	100751628	0.03	0.15	0.06	0.08	0.00	0.00	2.28	7.32	2.48	2.32	0.00	0.00
aspartyl-tRNA synthetase	Dars	100767438	113.90	112.33	113.74	122.54	131.12	120.00	7410.82	7403.54	7448.79	7896.88	8441.35	7679.25
aspartyl-tRNA synthetase 2, mitochondrial	Dars2	100774650	8.89	9.35	9.15	10.19	12.50	12.50	710.93	736.60	753.95	818.20	996.69	979.53
dynein assembly factor with WDR repeat domains 1	Daw1	100757280	0.00	0.00	0.00	0.00	0.00	0.00	0.00	0.00	0.00	0.00	0.00	0.00
death-domain associated protein	Daxx	100774618	104.43	97.78	105.14	90.63	79.44	86.15	7095.62	6678.83	7137.03	6091.28	5391.24	5841.69
DAZ associated protein 1	Dazap1	100756248	40.26	42.38	40.68	35.22	32.50	30.45	2405.46	2435.82	2422.07	2086.06	1676.68	1679.60
DAZ associated protein 2	Dazap2	100775021	56.21	55.58	59.00	54.38	59.91	60.90	2602.94	2556.60	2753.71	2511.38	2714.77	2836.72
deleted in azoospermia-like	Dazl	100771380	0.00	0.00	0.00	0.00	0.00	0.00	0.00	0.00	0.00	0.00	0.00	0.00
DBF4 zinc finger	Dbf4	100689339	30.31	29.97	33.35	35.50	37.30	34.79	1982.40	1993.86	2220.85	2342.18	2446.71	2260.03
dopamine beta-hydroxylase (dopamine beta-monooxygenase)	Dbh	100761411	0.00	0.00	0.00	0.00	0.00	0.00	0.00	0.00	0.00	0.00	0.00	0.00
diazepam binding inhibitor (GABA receptor modulator, acyl-CoA binding protein)	Dbi	100761641	59.84	64.18	48.99	53.24	63.00	59.87	888.66	945.23	711.72	755.62	909.75	850.55
drebrin 1	Dbn1	100763853	59.83	60.04	53.66	42.84	29.26	29.87	4774.46	4757.26	4242.97	3414.18	2394.96	2426.40
dysbindin (dystrobrevin binding protein 1) domain containing 1	Dbndd1	100759269	0.15	0.04	0.18	0.15	0.42	0.16	9.87	1.83	4.97	5.79	11.38	8.41
dysbindin (dystrobrevin binding protein 1) domain containing 2	Dbndd2	100765248	81.08	80.14	76.48	79.33	86.26	95.03	2143.42	2102.75	2015.91	1991.02	2229.36	2418.93
drebrin-like	Dbnl	100761466	33.13	32.16	35.77	31.66	27.53	28.51	1760.61	1692.81	1830.84	1579.61	1371.36	1399.20
D site of albumin promoter (albumin D-box) binding protein	Dbp	100775076	4.20	3.94	5.66	6.15	5.69	4.73	106.34	95.16	160.23	155.30	135.58	119.64
debranching RNA lariats 1	Dbr1	100768520	9.53	10.27	5.40	5.58	6.09	5.60	550.67	603.01	309.28	324.50	348.79	322.46
dihydrolipoamide branched chain transacylase E2	Dbt	100759869	18.26	17.82	17.67	18.44	21.50	20.04	2149.50	2109.15	2100.37	2206.58	2507.77	2351.63
developing brain homeobox 1	Dbx1	100760100	0.00	0.00	0.00	0.00	0.00	0.00	0.00	0.00	0.00	0.00	0.00	0.00
developing brain homeobox 2	Dbx2	100761209	0.00	0.00	0.00	0.00	0.00	0.00	0.00	0.00	0.00	0.00	0.00	0.00
DDB1 and CUL4 associated factor 10	Dcaf10	100754235	5.09	4.94	5.70	5.74	6.48	6.91	278.75	265.36	314.25	311.75	344.65	372.00
DDB1 and CUL4 associated factor 11	Dcaf11	100762322	22.67	22.86	28.32	29.97	29.62	32.37	1809.22	1843.79	2309.04	2417.51	2381.50	2559.13
DDB1 and CUL4 associated factor 12	Dcaf12	100771230	23.83	23.70	25.18	26.64	27.27	28.40	1212.98	1212.42	1294.25	1350.14	1383.78	1452.48
DDB1 and CUL4 associated factor 12-like 2	Dcaf12l2	100764386	18.97	18.34	22.14	20.15	22.68	20.75	1201.59	1174.90	1448.27	1353.62	1487.28	1416.02
DDB1 and CUL4 associated factor 13	Dcaf13	100751757	25.02	24.66	29.02	31.53	27.40	27.46	973.73	963.53	1148.93	1220.34	1041.20	1051.50

DDB1 and CUL4 associated factor 15	Dcaf15	100759899	7.63	7.55	9.29	7.98	6.26	7.04	569.65	585.62	690.60	596.84	448.15	534.63
DDB1 and CUL4 associated factor 17	Dcaf17	100767135	4.30	4.52	3.72	4.03	4.36	3.98	313.69	327.58	258.35	290.89	318.78	291.62
DDB1 and CUL4 associated factor 4	Dcaf4	100752324	15.40	15.84	18.20	18.88	15.90	16.40	1295.77	1311.24	1541.43	1572.65	1309.26	1368.36
DDB1 and CUL4 associated factor 5	Dcaf5	100765902	19.08	18.70	21.10	21.82	20.20	20.78	2880.17	2834.77	3217.00	3314.51	3048.03	3164.79
DDB1 and CUL4 associated factor 6	Dcaf6	100770150	6.17	6.05	7.41	7.33	8.52	7.61	593.96	615.82	739.04	771.84	869.39	796.34
DDB1 and CUL4 associated factor 7	Dcaf7	100773606	29.25	29.27	36.68	37.75	38.13	39.76	4930.93	4962.23	6226.58	6381.01	6399.32	6739.90
DDB1 and CUL4 associated factor 8	Dcaf8	100762613	42.25	40.42	45.18	45.24	43.38	46.34	4770.67	4579.74	5132.30	5103.88	4872.72	5215.46
dephospho-CoA kinase domain containing	Dcakd	100759055	124.59	116.76	87.66	76.61	65.02	68.60	5701.10	5392.29	4029.33	3501.10	2934.19	3101.23
discoidin, CUB and LCCL domain containing 1	Dcbld1	100760899	2.96	2.83	2.09	1.67	1.97	1.48	252.93	237.91	173.89	139.07	163.53	125.25
discoidin, CUB and LCCL domain containing 2	Dcbld2	100772480	46.10	47.89	58.62	65.17	67.40	66.30	7521.72	7831.77	9679.58	10738.55	10985.34	10853.38
deleted in colorectal carcinoma	Dcc	100758377	0.00	0.00	0.00	0.00	0.00	0.00	0.76	0.00	0.00	0.00	0.00	0.00
doublecortin domain containing 1	Dcdc1	100769645	0.47	0.40	0.17	0.28	0.56	0.24	41.02	27.45	12.42	22.02	41.40	25.24
doublecortin domain containing 2	Dcdc2	100770382	2.00	2.39	1.91	2.04	2.10	2.21	195.20	225.10	182.59	195.86	194.58	214.04
doublecortin domain containing 2B	Dcdc2b	100759892	4.01	3.91	6.37	6.42	6.02	6.59	348.63	331.24	558.94	551.65	536.12	583.23
doublecortin domain containing 2C	Dcdc2c	100766730	0.00	0.00	0.00	0.00	0.00	0.00	0.00	0.00	0.00	0.00	0.00	0.00
doublecortin domain containing 5	Dcdc5	100769357	0.02	0.00	0.00	0.00	0.00	0.01	2.28	0.00	0.00	0.00	0.00	0.93
dachsous cadherin-related 1	Dchs1	100760642	0.03	0.02	0.00	0.01	0.00	0.01	9.11	8.24	1.24	3.48	0.00	4.67
dachsous cadherin-related 2	Dchs2	100774272	0.01	0.00	0.00	0.00	0.00	0.00	3.04	0.00	1.24	1.16	0.00	0.00
deoxycytidine kinase	Dck	100751299	9.37	10.49	13.78	14.93	17.81	17.29	523.32	574.64	760.16	808.93	989.45	970.19
doublecortin-like kinase 1	Dclk1	100768340	8.04	8.32	6.24	6.42	8.16	7.98	946.39	963.53	614.83	657.11	850.76	838.40
doublecortin-like kinase 2	Dclk2	100764661	3.76	3.52	2.88	2.92	2.33	2.12	494.46	494.12	377.59	404.46	312.57	284.14
doublecortin-like kinase 3	Dclk3	100765735	0.00	0.00	0.00	0.00	0.00	0.00	0.00	0.00	0.00	0.00	0.00	0.00
DNA cross-link repair 1A	Dclre1a	100750521	8.35	7.99	9.11	8.74	9.47	9.12	1010.95	973.60	1106.70	1058.09	1135.38	1101.98
DNA cross-link repair 1B	Dclre1b	100759525	6.33	6.09	6.50	7.10	7.42	7.43	485.35	469.41	506.77	544.69	564.07	566.41
DNA cross-link repair 1C	Dclre1c	100752196	4.70	4.83	5.71	5.78	6.80	6.29	315.21	337.65	382.56	372.01	442.97	422.47
decorin	Dcn	100756152	30.90	32.78	57.76	53.40	27.08	27.70	1481.86	1593.07	2799.66	2526.45	1286.49	1373.03
decapping mRNA 1A	Dcp1a	100773763	20.07	18.80	20.52	21.70	30.31	32.11	2570.28	2411.11	2676.70	2815.02	3860.50	4157.41
decapping mRNA 1B	Dcp1b	103160459	10.69	10.28	9.57	8.46	6.69	8.09	536.99	529.80	478.20	428.80	365.35	437.43
decapping mRNA 2	Dcp2	100773631	5.64	5.40	5.98	6.09	6.98	6.67	609.15	602.09	649.61	671.01	754.51	700.07
decapping enzyme, scavenger	Dcps	100761162	42.34	42.99	46.14	46.06	39.81	42.19	1440.09	1488.76	1612.23	1561.07	1370.32	1446.87
DC-STAMP domain containing 1	Dcst1	100773996	0.03	0.02	0.02	0.02	0.00	0.00	12.91	7.32	7.45	9.27	8.28	6.54
DC-STAMP domain containing 2	Dcst2	100774289	3.93	3.62	5.36	4.08	3.01	3.50	446.61	419.09	613.59	463.57	344.65	410.32
dendrocyte expressed seven transmembrane protein	Dcstamp	100752337	0.00	0.00	0.00	0.00	0.00	0.00	0.00	0.00	0.00	0.00	0.00	0.00
dopachrome tautomerase	Dct	100764118	0.00	0.00	0.00	0.00	0.00	0.00	0.00	0.00	0.00	0.00	0.00	0.00
dCMP deaminase	Dctd	100751791	0.00	0.02	0.00	0.00	0.00	0.00	0.00	0.92	0.00	0.00	0.00	0.00
dynactin 1	Dctn1	100765083	42.70	42.33	41.12	32.18	25.22	27.12	5334.24	5329.16	5179.50	4021.45	3245.72	3476.97
dynactin 2 (p50)	Dctn2	100757525	86.88	82.36	84.64	87.18	70.39	83.14	4557.24	4466.28	4580.82	4634.52	3792.19	4345.28
dynactin 3 (p22)	Dctn3	100753423	52.81	52.23	56.86	57.79	54.36	60.72	1176.53	1122.75	1188.68	1082.43	1046.37	1158.99
dynactin 4 (p62)	Dctn4	100767865	17.83	17.97	20.30	22.63	25.12	25.33	1828.21	1853.86	2119.00	2317.84	2582.29	2598.38
dynactin 5 (p25)	Dctn5	100773966	69.01	67.96	88.52	90.62	95.33	92.89	1298.81	1333.20	1678.06	1723.31	1805.02	1826.34

dynactin 6	Dctn6	100754783	60.52	61.16	59.48	62.37	68.82	66.49	1597.31	1625.10	1568.76	1591.20	1781.21	1712.31
dCTP pyrophosphatase 1	Dctpp1	100769832	79.76	85.97	108.33	102.54	99.67	94.05	1335.27	1432.03	1797.30	1668.84	1636.31	1535.66
DCN1, defective in cullin neddylation 1, domain containing 1	Dcun1d 1	100771521	10.33	10.10	10.66	11.91	11.99	11.04	1160.58	1163.01	1211.03	1360.57	1338.24	1265.54
DCN1, defective in cullin neddylation 1, domain containing 2	Dcun1d 2	100757607	7.52	7.83	8.27	8.87	8.18	9.21	559.02	551.77	544.03	569.03	601.33	642.12
DCN1, defective in cullin neddylation 1, domain containing 3	Dcun1d 3	100752469	4.74	3.74	3.14	3.17	3.08	3.04	77.47	63.14	50.93	62.58	50.71	50.47
DCN1, defective in cullin neddylation 1, domain containing 4	Dcun1d 4	100756000	8.42	8.94	9.79	10.28	10.20	11.49	650.17	696.34	756.43	806.61	812.46	871.11
DCN1, defective in cullin neddylation 1, domain containing 5	Dcun1d 5	100759094	38.22	37.20	39.86	41.55	38.63	44.47	902.33	908.63	967.59	934.09	930.45	1013.18
doublecortin	Dcx	100773444	0.00	0.00	0.00	0.00	0.00	0.00	0.00	0.00	0.00	0.00	0.00	0.00
dicarbonyl/L-xylulose reductase	Dcxr	100769825	38.33	37.32	21.93	16.08	8.56	9.30	939.55	929.67	525.40	396.35	221.49	224.32
DET1 and DDB1 associated 1	Dda1	100751929	31.26	32.90	33.13	21.65	19.32	18.72	972.97	1040.39	1019.75	688.40	599.26	559.87
dimethylarginine dimethylaminohydrolase 1	Ddah1	100767722	0.00	0.00	0.02	0.00	0.00	0.02	0.00	0.00	1.24	0.00	0.00	0.93
dimethylarginine dimethylaminohydrolase 2	Ddah2	100761676	0.09	0.14	0.05	0.05	0.00	0.04	2.28	3.66	2.48	1.16	0.00	2.80
damage-specific DNA binding protein 1, 127kDa	Ddb1	100764133	105.15	104.69	86.73	89.75	92.29	95.14	13603.35	13818.84	11495.51	11753.77	12055.52	12315.21
damage-specific DNA binding protein 2, 48kDa	Ddb2	100756705	3.58	3.37	4.00	4.35	4.77	4.74	164.06	179.35	203.70	220.19	250.47	226.19
dopa decarboxylase (aromatic L-amino acid decarboxylase)	Ddc	100761742	0.02	0.02	0.00	0.03	0.03	0.02	0.76	1.83	0.00	2.32	1.03	2.80
DDHD domain containing 1	Ddhd1	100761687	6.42	6.98	7.11	8.35	9.14	8.77	795.24	853.73	886.85	1026.80	1100.19	1088.89
DDHD domain containing 2	Ddhd2	100772516	24.34	23.24	26.97	29.69	33.88	30.57	2732.06	2695.69	3077.89	3363.19	3882.23	3576.98
DNA-damage inducible 1 homolog 1 (S. cerevisiae)	Ddi1	100755779	0.00	0.00	0.00	0.00	0.00	0.00	0.00	0.00	0.00	0.00	0.00	0.00
DNA-damage inducible 1 homolog 2 (S. cerevisiae)	Ddi2	100753562	18.38	18.52	17.21	18.46	14.10	15.98	530.92	537.12	499.32	522.67	408.82	462.66
DNA-damage-inducible transcript 3	Ddit3	100763514	124.99	120.92	140.67	145.36	129.05	133.09	2556.61	2500.79	2905.24	2920.48	2594.71	2697.46
DNA-damage-inducible transcript 4	Ddit4	100759745	7.96	8.22	4.90	4.06	2.92	3.77	344.07	362.35	216.12	175.00	124.20	160.76
DNA-damage-inducible transcript 4-like	Ddit4l	100751717	0.00	0.00	0.00	0.00	0.00	0.00	0.00	0.92	1.24	0.00	0.00	0.00
dendrin	Ddn	100769143	0.00	0.00	0.00	0.00	0.00	0.00	0.00	1.83	0.00	0.00	0.00	0.00
D-aspartate oxidase	Ddo	100773572	0.00	0.04	0.00	0.00	0.00	0.00	0.00	0.92	0.00	0.00	0.00	0.00
dolichyl-diphosphooligosaccharide--protein glycosyltransferase subunit (non-catalytic)	Ddost	100755259	196.68	185.68	207.49	197.33	187.24	192.46	9364.36	8934.39	9965.26	9394.20	9010.59	9117.70
discoidin domain receptor tyrosine kinase 1	Ddr1	100762536	9.39	8.55	10.31	7.62	4.61	5.47	992.72	917.78	1124.09	823.99	497.83	578.56
discoidin domain receptor tyrosine kinase 2	Ddr2	100689338	97.46	94.33	119.35	138.49	146.73	143.50	12991.16	12615.57	16117.32	18423.35	19444.29	19214.94
DDRGK domain containing 1	Ddrgk1	100689201	52.74	54.60	47.42	44.55	33.47	36.71	1751.50	1840.13	1577.45	1459.08	1140.55	1247.78
D-dopachrome tautomerase	Ddt	100765163	39.26	36.06	37.80	28.06	23.71	25.44	581.81	541.70	561.42	395.19	341.55	361.72
DEAD (Asp-Glu-Ala-Asp) box helicase 1	Ddx1	100756637	71.93	71.11	70.73	80.33	80.94	82.45	5588.69	5706.15	5612.99	6225.72	6352.75	6322.11
DEAD (Asp-Glu-Ala-Asp) box polypeptide 10	Ddx10	100752733	84.19	83.82	81.11	85.97	80.77	74.87	7653.12	7653.34	7451.28	7922.38	7319.42	6819.35
DEAD/H (Asp-Glu-Ala-Asp/His) box helicase 11	Ddx11	100756953	11.33	10.67	12.19	12.29	11.82	12.00	1303.37	1270.07	1471.87	1478.78	1435.53	1442.20

DEAD (Asp-Glu-Ala-Asp) box helicase 17	Ddx17	100774831	50.22	47.54	49.47	49.42	47.81	51.39	3648.07	3533.86	3681.55	3633.21	3515.85	3717.18
DEAD (Asp-Glu-Ala-Asp) box polypeptide 18	Ddx18	100759595	65.11	66.08	72.36	75.15	71.87	70.12	4251.90	4299.74	4711.24	4894.12	4666.75	4588.29
DEAD (Asp-Glu-Ala-Asp) box polypeptide 19B	Ddx19b	100766470	18.36	19.13	20.12	20.18	18.48	17.52	962.34	1014.77	1111.67	1049.98	1097.09	1011.31
DEAD (Asp-Glu-Ala-Asp) box polypeptide 20	Ddx20	100752364	18.80	20.82	24.37	25.24	24.61	25.07	1400.59	1573.86	1825.87	1861.23	1842.28	1878.69
DEAD (Asp-Glu-Ala-Asp) box helicase 21	Ddx21	100771185	144.12	139.82	135.04	140.56	129.59	130.22	11725.77	11479.10	11093.08	11528.93	10595.15	10641.21
DEAD (Asp-Glu-Ala-Asp) box polypeptide 23	Ddx23	100767790	62.69	62.97	62.51	65.54	58.58	61.53	5801.36	5899.22	5832.84	6054.20	5433.68	5694.01
DEAD (Asp-Glu-Ala-Asp) box helicase 24	Ddx24	100764379	35.29	34.86	34.36	33.53	29.95	32.35	2792.07	2720.39	2702.78	2636.54	2327.68	2543.24
DEAD (Asp-Glu-Ala-Asp) box helicase 25	Ddx25	100759427	0.15	0.07	0.06	0.09	0.08	0.09	7.60	5.49	3.73	5.79	3.10	4.67
DEAD (Asp-Glu-Ala-Asp) box polypeptide 27	Ddx27	100758755	93.17	94.30	94.56	84.90	77.10	77.90	6922.44	6952.42	7005.37	6108.67	5730.72	5707.09
DEAD (Asp-Glu-Ala-Asp) box polypeptide 28	Ddx28	100750865	10.60	11.67	11.10	12.54	12.08	11.28	519.52	576.47	545.28	614.23	588.91	552.39
DEAD (Asp-Glu-Ala-Asp) box polypeptide 31	Ddx31	100773757	11.52	11.27	10.95	11.28	10.21	11.11	1490.22	1472.29	1423.43	1449.81	1295.80	1449.67
DEAD (Asp-Glu-Ala-Asp) box polypeptide 39A	Ddx39a	100752425	60.08	56.71	65.55	60.76	54.93	60.53	2653.07	2553.86	2887.85	2659.72	2381.50	2560.99
DEAD (Asp-Glu-Ala-Asp) box polypeptide 39B	Ddx39b	100773161	83.61	80.83	111.16	112.41	94.29	101.19	2290.77	2225.36	3070.44	3081.57	2391.85	2657.27
DEAD (Asp-Glu-Ala-Asp) box helicase 3, X-linked	Ddx3x	100764307	226.90	228.27	182.79	194.16	195.11	184.38	30679.31	30922.64	24787.08	26137.12	26164.45	24888.38
DEAD (Asp-Glu-Ala-Asp) box polypeptide 4	Ddx4	100767469	0.71	0.96	1.22	1.54	1.60	1.51	77.47	98.82	135.39	165.73	167.67	152.35
DEAD (Asp-Glu-Ala-Asp) box polypeptide 41	Ddx41	100770508	38.22	35.82	48.96	46.09	35.78	39.18	2390.27	2237.26	3034.42	2798.79	2252.13	2415.19
DEAD (Asp-Glu-Ala-Asp) box helicase 42	Ddx42	100771386	30.01	29.44	33.50	34.92	35.38	36.18	3571.35	3519.22	4010.70	4154.73	4214.46	4291.07
DEAD (Asp-Glu-Ala-Asp) box polypeptide 43	Ddx43	100754802	0.01	0.00	0.02	0.00	0.00	0.00	0.76	0.00	0.00	0.00	0.00	0.00
DEAD (Asp-Glu-Ala-Asp) box polypeptide 46	Ddx46	100763282	37.65	36.47	31.00	34.33	31.20	30.81	4311.90	4252.16	3630.62	3936.85	3631.77	3532.12
DEAD (Asp-Glu-Ala-Asp) box polypeptide 47	Ddx47	100751146	86.34	88.09	100.97	103.47	91.56	98.14	4369.63	4439.74	5076.41	5128.22	4536.34	4907.02
DEAD (Asp-Glu-Ala-Asp) box polypeptide 49	Ddx49	100765219	19.79	17.05	19.11	13.69	9.40	10.05	1053.48	958.96	1002.36	735.91	504.04	521.55
DEAD (Asp-Glu-Ala-Asp) box helicase 5	Ddx5	100768812	234.23	243.97	259.85	270.56	274.42	274.30	20761.25	21600.28	23243.17	24034.84	24397.73	24332.26
DEAD (Asp-Glu-Ala-Asp) box polypeptide 50	Ddx50	100766872	34.01	32.77	35.48	39.28	39.16	36.73	2668.26	2610.59	2795.94	3078.09	3055.28	2871.31
DEAD (Asp-Glu-Ala-Asp) box polypeptide 51	Ddx51	100773225	17.27	17.26	14.12	12.82	11.86	13.51	1264.63	1283.79	1048.32	961.90	884.91	995.42
DEAD (Asp-Glu-Ala-Asp) box polypeptide 52	Ddx52	100771911	22.19	21.11	11.06	12.98	12.80	11.24	1459.83	1380.79	711.72	826.31	831.09	742.13
DEAD (Asp-Glu-Ala-Asp) box polypeptide 54	Ddx54	100770376	52.44	50.83	44.09	34.11	24.67	25.36	4642.30	4535.82	3933.69	2978.42	2221.08	2272.18
DEAD (Asp-Glu-Ala-Asp) box polypeptide 55	Ddx55	100767780	27.50	29.29	32.36	34.83	28.63	29.78	1740.10	1858.43	2064.35	2184.56	1820.54	1888.03

DEAD (Asp-Glu-Ala-Asp) box helicase 56	Ddx56	100764269	45.47	43.79	48.82	47.25	44.31	45.88	2522.43	2478.83	2843.14	2687.54	2489.14	2567.54
DEAD (Asp-Glu-Ala-Asp) box polypeptide 58	Ddx58	100766319	1.07	1.04	1.08	0.87	0.88	0.78	155.71	154.64	144.08	143.71	133.51	132.72
DEAD (Asp-Glu-Ala-Asp) box polypeptide 59	Ddx59	100758928	5.13	4.96	4.21	4.94	4.54	4.73	299.26	293.73	245.93	289.73	264.96	286.94
DEAD (Asp-Glu-Ala-Asp) box helicase 6	Ddx6	100759836	20.13	21.10	17.16	18.33	19.48	19.34	2716.11	2876.87	2337.61	2505.58	2612.31	2670.35
DEAD (Asp-Glu-Ala-Asp) box polypeptide 60	Ddx60	100760761	0.04	0.04	0.00	0.00	0.00	0.00	10.63	10.07	0.00	0.00	1.03	0.00
DEAF1 transcription factor	Deaf1	100756469	6.03	5.73	6.39	5.53	5.11	5.64	281.03	269.02	325.43	293.21	264.96	309.38
2,4-dienoyl CoA reductase 1, mitochondrial	Decr1	100753128	39.81	39.86	43.44	43.17	44.72	46.60	1272.23	1271.90	1361.33	1326.96	1396.20	1463.69
2,4-dienoyl CoA reductase 2, peroxisomal	Decr2	100759323	10.86	11.00	9.73	8.60	10.58	9.88	463.32	475.82	417.34	359.27	444.01	410.32
death effector domain containing	Dedd	100766615	8.18	9.24	9.12	7.28	5.34	5.88	268.88	321.18	316.73	206.29	135.58	137.40
death effector domain containing 2	Dedd2	103160330	0.16	0.11	0.17	0.16	0.07	0.11	12.91	8.24	13.66	13.91	6.21	10.28
differentially expressed in FDCP 6 homolog (mouse)	Def6	100757712	18.06	17.08	13.97	12.24	9.84	11.03	1566.93	1475.03	1214.76	1082.43	879.74	992.62
differentially expressed in FDCP 8 homolog (mouse)	Def8	100759557	26.66	25.36	30.85	30.57	30.31	32.94	2623.45	2544.71	3097.76	2985.38	2934.19	3191.90
defensin, beta 1	Defb1	103161655	0.00	0.00	0.00	0.00	0.00	0.00	0.00	0.00	0.00	0.00	0.00	0.00
defensin, beta 110 locus	Defb110	100756066	0.00	0.00	0.00	0.00	0.00	0.00	0.00	0.00	0.00	0.00	0.00	0.00
defensin, beta 115	Defb115	100775044	0.00	0.00	0.00	0.00	0.00	0.00	0.00	0.00	0.00	0.00	0.00	0.00
defensin, beta 123	Defb123	100758173	0.00	0.00	0.00	0.00	0.00	0.00	0.00	0.00	0.00	0.00	0.00	0.00
defensin, beta 125	Defb125	103160370	0.00	0.00	0.00	0.00	0.00	0.00	0.00	0.00	0.00	0.00	0.00	0.00
defensin, beta 128	Defb128	100759044	0.00	0.00	0.00	0.00	0.00	0.00	0.00	0.00	0.00	0.00	0.00	0.00
defensin, beta 129	Defb129	100750561	0.00	0.00	0.00	0.00	0.00	0.00	0.00	0.00	0.00	0.00	0.00	0.00
defensin, beta 135	Defb135	100757570	0.00	0.00	0.00	0.00	0.00	0.00	0.00	0.00	0.00	0.00	0.00	0.00
defensin, beta 136	Defb136	100757284	0.00	0.00	0.00	0.00	0.00	0.00	0.00	0.00	0.00	0.00	0.00	0.00
delta(4)-desaturase, sphingolipid 1	Degs1	100753569	165.09	178.31	178.66	187.73	241.86	219.76	5341.08	5817.78	5868.86	6090.12	7787.24	7152.09
delta(4)-desaturase, sphingolipid 2	Degs2	100771298	0.03	0.00	0.00	0.00	0.00	0.00	0.76	0.92	0.00	0.00	0.00	0.00
DEK oncogene	Dek	100767626	62.31	63.67	67.93	79.41	89.99	78.56	2193.55	2236.34	2332.64	2732.73	3079.08	2764.75
DENN/MADD domain containing 1A	Dennd1a	100774592	4.64	4.80	6.32	6.07	5.90	5.85	684.34	711.90	943.99	874.98	839.37	820.64
DENN/MADD domain containing 1B	Dennd1b	100765438	2.53	2.39	3.06	3.67	3.76	3.64	566.62	524.31	695.57	815.88	821.78	799.14
DENN/MADD domain containing 1C	Dennd1c	100763098	0.02	0.06	0.02	0.04	0.02	0.00	3.04	3.66	1.24	2.32	1.03	0.00
DENN/MADD domain containing 2A	Dennd2a	100770913	10.02	9.76	11.09	10.08	8.82	9.68	1305.65	1274.64	1458.21	1339.71	1161.25	1265.54
DENN/MADD domain containing 2C	Dennd2c	100758192	2.06	2.32	1.97	2.19	2.07	2.22	211.15	239.74	211.15	235.26	210.10	219.65
DENN/MADD domain containing 2D	Dennd2d	100759018	0.03	0.00	0.00	0.00	0.00	0.00	6.08	3.66	2.48	2.32	2.07	4.67

DENN/MADD domain containing 3	Dennd3	100774715	2.35	2.08	1.87	2.00	1.95	2.02	398.00	346.80	293.13	316.39	334.30	328.07
DENN/MADD domain containing 4A	Dennd4a	100772570	13.04	12.97	15.78	18.66	19.73	18.18	3159.68	3157.78	3881.52	4525.58	4764.04	4406.97
DENN/MADD domain containing 4B	Dennd4b	100767268	2.89	2.61	2.06	1.91	1.83	1.81	541.55	488.63	383.80	355.79	337.41	343.02
DENN/MADD domain containing 4C	Dennd4c	100754289	11.52	11.19	13.93	16.08	16.83	16.29	1901.13	1873.07	2351.27	2671.31	2818.27	2668.48
DENN/MADD domain containing 5A	Dennd5a	100767226	49.80	47.94	48.92	51.91	55.42	56.89	7002.19	6902.10	7017.79	7289.61	7854.51	7983.95
DENN/MADD domain containing 5B	Dennd5b	100762143	1.63	1.66	1.46	1.77	2.05	1.66	220.27	231.50	202.46	241.06	277.38	229.93
DENN/MADD domain containing 6A	Dennd6a	100769342	4.88	4.56	6.26	7.13	8.09	6.76	622.06	571.90	787.48	903.96	1006.01	849.61
DENN/MADD domain containing 6B	Dennd6b	100754914	3.97	3.75	5.93	5.43	4.25	4.56	381.29	371.50	525.40	486.75	395.36	453.31
density-regulated protein	Denr	100765761	98.11	100.98	90.46	91.56	85.61	93.85	2839.16	2844.84	2566.15	2520.65	2361.84	2560.99
DEP domain containing 1	Depdc1	100767779	16.02	15.57	14.12	16.11	18.69	15.34	1320.84	1249.02	1153.90	1302.63	1505.91	1234.70
DEP domain containing 1B	Depdc1b	100754645	12.24	12.44	14.26	15.40	17.56	18.15	874.23	895.82	1043.35	1091.70	1249.23	1282.37
DEP domain containing 5	Depdc5	100767573	8.76	8.41	10.03	10.72	10.54	11.20	1693.01	1647.98	1942.62	2069.83	2033.75	2160.02
DEP domain containing 7	Depdc7	100771553	4.17	4.91	5.05	4.66	4.91	4.36	228.62	266.27	275.74	249.17	263.92	231.80
deoxyribose-phosphate aldolase (putative)	Dera	100750675	21.77	21.71	23.55	22.41	20.56	22.69	805.11	818.96	860.77	811.24	765.89	823.44
derlin 1	Derl1	103158862	98.81	96.24	69.59	67.97	63.70	64.87	5735.28	5594.51	4093.92	3973.94	3730.09	3825.60
derlin 2	Derl2	100755526	13.88	13.72	14.88	15.63	16.36	16.86	391.16	415.43	424.79	453.14	492.65	489.77
derlin 3	Derl3	100768848	0.24	0.26	0.23	0.22	0.13	0.18	12.15	10.98	7.45	11.59	5.17	8.41
desmin	Des	100764990	0.19	0.35	0.09	0.08	0.09	0.10	12.91	21.96	6.21	6.95	6.21	9.35
desumoylating isopeptidase 1	Desi1	100770500	18.00	18.37	22.53	18.55	19.49	21.12	517.25	544.44	663.27	564.39	615.82	626.23
desumoylating isopeptidase 2	Desi2	100763663	32.59	30.30	33.04	27.54	25.91	25.74	700.30	678.04	755.19	635.09	627.20	613.14
de-etiolated homolog 1 (Arabidopsis)	Det1	100770116	19.57	18.77	19.99	19.12	15.46	16.99	1275.27	1249.94	1310.40	1244.68	1001.87	1116.00
Dexi homolog (mouse)	Dexi	100750632	10.24	10.44	12.66	10.94	10.98	10.91	266.60	267.19	327.91	278.14	278.41	276.66
DNA fragmentation factor, 45kDa, alpha polypeptide	Dffa	100762210	30.50	32.80	26.13	26.10	22.18	21.42	585.60	606.67	494.35	465.89	368.46	384.15
DNA fragmentation factor, 40kDa, beta polypeptide (caspase-activated DNase)	Dffb	100768492	8.37	7.33	10.31	10.72	10.68	10.72	555.22	477.65	686.87	681.44	713.11	741.19
deafness, autosomal dominant 5	Dfna5	100757679	5.29	5.24	2.92	2.58	2.57	2.91	499.02	501.44	281.95	264.23	269.10	313.11
deafness, autosomal recessive 31	Dfnb31	100754986	9.74	8.93	11.21	8.93	4.98	5.23	1005.63	945.23	1172.53	956.11	545.44	595.38
deafness, autosomal recessive 59	Dfnb59	100766395	0.12	0.09	0.04	0.19	0.07	0.24	6.08	6.41	1.24	6.95	9.31	8.41
diacylglycerol O-acyltransferase 1	Dgat1	100755985	6.12	6.64	8.23	9.49	8.73	8.60	473.19	512.42	638.43	725.48	645.83	627.16
diacylglycerol O-acyltransferase 2	Dgat2	100774981	25.83	23.60	22.65	21.28	16.24	18.89	2172.28	2015.82	1919.02	1784.74	1361.01	1575.85
diacylglycerol O-acyltransferase 2-like 6	Dgat2l6	100768854	0.28	0.44	0.30	0.31	0.23	0.16	18.99	31.11	19.87	19.70	15.52	20.56
DiGeorge syndrome critical region gene 14	Dgcr14	100751728	15.86	15.25	17.08	16.43	14.03	15.30	1675.54	1615.03	1817.17	1726.79	1468.65	1605.76
DiGeorge syndrome critical region gene 2	Dgcr2	100772062	33.24	31.66	35.09	34.71	28.24	30.42	3658.70	3499.09	3916.30	3823.28	3093.57	3376.03
diacylglycerol kinase, alpha 80kDa	Dgka	100769779	5.27	4.61	5.05	5.52	7.01	7.04	441.29	414.51	443.43	471.68	599.26	592.58

diacylglycerol kinase, beta 90kDa	Dgkb	100753343	0.00	0.00	0.00	0.00	0.00	0.00	0.00	0.00	0.00	0.00	0.00	0.00
diacylglycerol kinase, delta 130kDa	Dgkd	100771821	17.78	17.14	19.62	20.48	17.55	18.94	3152.09	3058.04	3461.70	3593.81	3085.29	3333.97
diacylglycerol kinase, epsilon 64kDa	Dgke	100765113	3.28	3.36	1.60	1.71	1.84	1.80	445.09	447.45	208.67	235.26	242.19	243.95
diacylglycerol kinase, gamma 90kDa	Dgkg	100757224	0.00	0.00	0.00	0.00	0.00	0.00	0.00	0.00	0.00	0.00	0.00	0.00
diacylglycerol kinase, eta	Dgkh	100769284	1.01	1.00	0.93	1.02	1.08	0.97	202.04	205.88	188.80	214.40	216.31	196.28
diacylglycerol kinase, iota	Dgki	100770430	0.00	0.00	0.00	0.00	0.00	0.00	0.00	0.00	0.00	0.00	0.00	0.00
diacylglycerol kinase, kappa	Dgkk	100755575	0.01	0.00	0.00	0.00	0.00	0.00	0.76	0.00	0.00	0.00	0.00	0.00
diacylglycerol kinase, theta 110kDa	Dgkq	100771387	6.06	5.61	7.54	6.53	6.97	7.34	896.26	840.00	1096.76	970.02	1004.97	1040.29
diacylglycerol kinase, zeta	Dgkz	100751991	16.46	16.32	13.59	10.64	6.63	7.83	2126.71	2126.54	1796.06	1397.66	920.10	1064.59
deoxyguanosine kinase	Dguok	100769222	9.47	10.63	9.43	11.15	10.78	9.80	268.12	286.41	257.11	285.09	276.34	263.58
24-dehydrocholesterol reductase	Dhcr24	100769920	2.70	2.84	3.93	3.13	2.53	2.97	302.30	312.94	439.70	346.52	280.48	331.81
7-dehydrocholesterol reductase	Dhcr7	100771700	19.15	17.89	19.16	19.22	16.26	17.86	1366.41	1288.37	1330.28	1364.05	1167.46	1288.91
dehydrodolichyl diphosphate synthase	Dhdds	100767025	74.41	75.34	74.28	73.17	70.78	77.59	4691.67	4849.68	4903.76	4734.19	4608.79	4945.34
dihydrodiol dehydrogenase (dimeric)	Dhdh	100753544	9.54	10.29	8.97	9.48	8.23	7.51	397.24	424.58	380.08	376.65	329.13	312.18
dihydrofolate reductase	Dhfr	100689028	20.07	22.84	22.52	25.90	29.88	26.87	840.81	981.83	1006.09	1077.80	1178.85	1062.72
desert hedgehog	Dhh	100768865	0.00	0.00	0.00	0.00	0.00	0.00	0.00	0.00	0.00	0.00	0.00	0.00
dihydroorotate dehydrogenase (quinone)	Dhodh	100756632	41.27	39.46	38.83	32.61	26.09	28.74	1794.03	1740.39	1705.39	1411.56	1155.04	1259.00
deoxyhypusine synthase	Dhps	100755665	42.94	41.01	38.24	35.23	35.36	39.87	1624.65	1591.24	1521.56	1355.94	1387.92	1526.32
dehydrogenase/reductase (SDR family) member 1	Dhrs1	100757215	37.84	36.27	35.75	37.53	33.61	36.66	1658.83	1587.58	1573.72	1625.96	1479.00	1578.66
dehydrogenase/reductase (SDR family) member 11	Dhrs11	100763193	13.13	12.49	8.15	6.95	7.84	8.11	471.67	456.60	293.13	250.33	275.31	287.88
dehydrogenase/reductase (SDR family) member 13	Dhrs13	100767705	10.22	9.79	12.62	11.90	9.14	10.03	495.98	485.88	623.53	573.67	435.73	481.35
dehydrogenase/reductase (SDR family) member 2	Dhrs2	100771582	0.00	0.00	0.00	0.00	0.00	0.00	0.00	0.00	0.00	0.00	0.00	0.00
dehydrogenase/reductase (SDR family) member 3	Dhrs3	100756186	1.75	1.02	0.83	1.12	0.50	0.66	62.28	42.09	36.02	46.36	18.63	27.11
dehydrogenase/reductase (SDR family) member 7B	Dhrs7b	100773373	12.76	13.12	14.05	14.72	15.15	15.66	511.93	533.46	582.54	564.39	627.20	634.64
dehydrogenase/reductase (SDR family) member 7C	Dhrs7c	100765494	0.00	0.00	0.00	0.00	0.00	0.00	0.00	0.00	0.00	0.00	0.00	0.00
dehydrogenase/reductase (SDR family) member 9	Dhrs9	100771741	1.29	1.23	0.20	0.29	0.57	0.33	112.41	103.40	21.12	25.50	46.57	27.11
dehydrogenase E1 and transketolase domain containing 1	Dhtkd1	100753689	2.14	2.40	3.02	3.30	3.47	3.34	236.98	268.10	332.88	365.06	391.22	365.46
DEAH (Asp-Glu-Ala-His) box helicase 15	Dhx15	100764898	72.91	71.76	82.21	85.78	91.02	89.03	6023.15	5979.74	6795.46	7026.53	7431.20	7209.11
DEAH (Asp-Glu-Ala-His) box polypeptide 16	Dhx16	100756749	33.50	31.36	33.34	26.26	19.42	20.83	3193.86	2996.73	3194.65	2534.56	1936.46	2031.97
DEAH (Asp-Glu-Ala-His) box polypeptide 29	Dhx29	100768526	9.27	8.80	8.05	10.29	11.02	10.07	1314.76	1259.09	1176.26	1491.53	1595.95	1473.04
DEAH (Asp-Glu-Ala-His) box helicase 30	Dhx30	100763879	66.61	64.05	67.42	56.11	42.96	45.93	8073.14	7875.69	8285.96	7017.26	5504.06	5801.49
DEAH (Asp-Glu-Ala-His) box polypeptide 32	Dhx32	100757757	16.84	16.32	20.22	21.08	23.98	23.68	1342.87	1320.39	1638.31	1715.20	1905.41	1911.40
DEAH (Asp-Glu-Ala-His) box polypeptide 33	Dhx33	100755235	12.24	11.61	10.61	10.97	9.63	11.02	902.33	879.35	791.21	837.90	734.84	848.68
DEAH (Asp-Glu-Ala-His) box polypeptide 34	Dhx34	100766240	3.08	3.14	3.82	1.89	1.54	1.31	276.47	290.98	324.18	164.57	125.23	99.07

DEAH (Asp-Glu-Ala-His) box polypeptide 35	Dhx35	100761819	11.36	11.87	9.88	10.20	9.68	9.78	1102.09	1152.03	945.23	994.35	918.03	947.76
DEAH (Asp-Glu-Ala-His) box polypeptide 36	Dhx36	100771317	17.87	18.44	16.28	20.21	19.85	16.86	1836.57	1883.14	1663.15	2035.06	2014.08	1734.75
DEAH (Asp-Glu-Ala-His) box polypeptide 37	Dhx37	100751766	14.26	13.66	14.62	12.49	9.87	10.97	1917.84	1856.60	1977.40	1693.18	1372.39	1481.45
DEAH (Asp-Glu-Ala-His) box polypeptide 38	Dhx38	100757501	18.73	19.13	17.90	18.30	16.27	17.18	2403.18	2489.81	2337.61	2376.94	2080.32	2184.32
DEAH (Asp-Glu-Ala-His) box polypeptide 40	Dhx40	100756345	10.86	11.59	13.83	14.92	16.83	15.87	1134.75	1202.35	1432.13	1539.05	1730.50	1644.08
DEAH (Asp-Glu-Ala-Asp/His) box polypeptide 57	Dhx57	100759824	6.59	6.01	6.99	8.07	8.57	8.23	954.74	870.20	1008.57	1155.44	1244.05	1176.75
DEXH (Asp-Glu-X-His) box polypeptide 58	Dhx58	100754123	0.09	0.04	0.08	0.02	0.03	0.01	9.11	4.58	6.21	2.32	2.07	1.87
DEAH (Asp-Glu-Ala-His) box helicase 9	Dhx9	100765632	72.97	73.46	57.36	61.77	61.05	62.20	9842.87	10047.98	7889.74	8401.01	8272.65	8329.78
diablo, IAP-binding mitochondrial protein	Diablo	100754770	42.02	42.18	41.87	44.57	47.33	49.59	1171.21	1221.57	1141.48	1238.89	1308.22	1359.94
diaphanous-related formin 1	Diaph1	100752938	12.95	13.19	13.88	14.96	15.56	16.74	1473.51	1540.00	1638.31	1717.52	1753.27	1893.64
diaphanous-related formin 3	Diaph3	100772633	13.41	13.02	13.73	15.80	19.25	16.76	1669.47	1662.62	1733.95	1930.76	2350.45	2095.53
dicer 1, ribonuclease type III	Dicer1	100689239	10.60	10.68	9.93	11.74	11.20	11.61	1979.36	2024.05	1885.49	2230.92	2121.72	2172.17
death inducer-obliterator 1	Dido1	100757601	16.09	16.87	15.61	16.56	15.82	16.50	3003.22	3066.28	3551.13	3356.23	3239.51	3409.68
digestive organ expansion factor homolog (zebrafish)	Diexf	100765405	30.48	30.03	29.75	28.66	27.12	29.26	2273.30	2236.34	2269.29	2124.30	2048.24	2133.85
DIM1 dimethyladenosine transferase 1 homolog (S. cerevisiae)	Dimt1	100756397	6.34	6.02	6.61	7.51	8.04	7.74	614.47	586.54	674.45	712.74	764.85	696.33
deiodinase, iodothyronine, type I	Dio1	100765192	0.00	0.06	0.00	0.00	0.00	0.04	0.00	2.75	1.24	1.16	2.07	2.80
deiodinase, iodothyronine, type II	Dio2	100774757	0.00	0.00	0.00	0.00	0.00	0.00	0.00	0.00	0.00	0.00	0.00	0.00
deiodinase, iodothyronine, type III	Dio3	100753739	0.00	0.00	0.00	0.00	0.00	0.00	0.00	0.00	0.00	0.00	0.00	0.00
DIP2 disco-interacting protein 2 homolog A (Drosophila)	Dip2a	100763951	12.39	11.55	12.39	12.55	11.55	12.32	2357.61	2221.70	2407.16	2424.46	2175.54	2354.43
DIP2 disco-interacting protein 2 homolog B (Drosophila)	Dip2b	100750591	6.75	7.04	7.27	7.88	8.02	8.10	1058.04	1109.02	1120.36	1207.59	1239.91	1277.69
DIP2 disco-interacting protein 2 homolog C (Drosophila)	Dip2c	100754252	6.79	7.23	5.57	5.43	4.79	5.29	871.95	843.66	849.59	880.78	756.57	817.84
DIRAS family, GTP-binding RAS-like 1	Diras1	100765547	0.03	0.00	0.00	0.00	0.00	0.00	0.00	0.92	0.00	0.00	0.00	0.00
DIRAS family, GTP-binding RAS-like 2	Diras2	100758943	0.00	0.00	0.00	0.00	0.00	0.00	0.00	0.00	0.00	0.00	0.00	0.00
disrupted in renal carcinoma 2	Dirc2	100767130	7.60	7.68	8.75	8.72	8.94	8.66	383.57	391.63	435.97	431.12	451.25	433.69
DIS3 exosome endoribonuclease and 3'-5' exoribonuclease	Dis3	100750372	13.67	13.08	13.36	15.15	14.99	13.78	1912.52	1854.77	1919.02	2135.89	2141.39	1931.96
DIS3 like exosome 3'-5' exoribonuclease	Dis3l	100767879	6.16	5.97	7.64	8.28	8.02	8.36	603.07	596.60	761.40	811.24	803.15	832.79
DIS3 like 3'-5' exoribonuclease 2	Dis3l2	100774616	5.54	5.96	5.56	5.36	5.10	5.54	491.42	519.74	481.93	469.36	446.08	476.68
disrupted in schizophrenia 1	Disc1	100772120	0.00	0.00	0.00	0.00	0.00	0.00	0.00	0.00	0.00	0.00	0.00	0.00
dispatched homolog 1 (Drosophila)	Disp1	100772392	8.33	8.18	8.91	8.84	7.89	7.88	1172.73	1150.20	1247.06	1238.89	1051.55	1085.15
dispatched homolog 2 (Drosophila)	Disp2	100759267	0.00	0.01	0.00	0.01	0.02	0.00	0.00	0.92	0.00	1.16	2.07	0.00
DIX domain containing 1	Dixdc1	100773209	8.24	9.03	8.30	9.13	10.97	10.28	999.55	1091.64	1016.03	1084.75	1261.65	1203.85
dyskeratosis congenita 1, dyskerin	Dkc1	100755395	145.38	145.33	138.28	141.89	139.07	137.22	10331.25	10493.60	10009.98	10152.14	9893.43	9779.45
dickkopf WNT signaling pathway inhibitor 1	Dkk1	100769380	0.00	0.00	0.00	0.00	0.00	0.00	0.00	0.00	0.00	0.00	0.00	0.00

dickkopf WNT signaling pathway inhibitor 2	Dkk2	100774823	0.00	0.08	0.00	0.00	0.00	0.00	0.00	0.92	0.00	0.00	0.00	0.00
dickkopf WNT signaling pathway inhibitor 3	Dkk3	100767109	97.56	100.20	145.00	144.22	139.96	141.56	6626.22	6886.54	9951.60	9810.26	9510.49	9612.14
dickkopf WNT signaling pathway inhibitor 4	Dkk4	100774676	0.00	0.00	0.00	0.00	0.07	0.00	0.00	0.00	1.24	2.32	1.03	0.93
dickkopf-like 1	Dkkl1	100771803	0.00	0.00	0.00	0.00	0.00	0.00	0.00	0.92	0.00	0.00	0.00	0.00
dihydrolipoamide S-acetyltransferase	Dlat	100773493	40.79	37.91	45.93	44.23	42.99	44.58	2630.28	2475.17	2979.77	2852.10	2841.04	2898.41
deleted in liver cancer 1	Dlc1	100771649	15.09	15.12	11.15	11.94	10.95	10.12	2710.04	2733.21	2028.33	2146.32	1950.95	1827.28
dihydrolipoamide dehydrogenase	Dld	100689437	65.09	70.36	61.27	69.65	75.95	72.25	4094.68	4272.29	3778.43	4130.39	4584.99	4458.37
deleted in lung and esophageal cancer 1	Dlec1	100753645	0.00	0.00	0.01	0.00	0.00	0.00	0.00	0.00	1.24	1.16	0.00	0.00
deleted in lymphocytic leukemia, 7	Dleu7	100753133	0.00	0.00	0.00	0.00	0.00	0.00	0.00	0.00	0.00	0.00	0.00	0.00
discs, large homolog 1 (Drosophila)	Dlg1	100774630	20.95	20.70	19.52	21.36	22.68	21.10	2791.31	2778.04	2612.11	2833.56	2990.08	2818.03
discs, large homolog 2 (Drosophila)	Dlg2	100752752	0.00	0.02	0.00	0.00	0.00	0.00	0.76	1.83	0.00	0.00	0.00	0.00
discs, large homolog 3 (Drosophila)	Dlg3	100770281	9.09	8.50	9.18	9.93	10.49	9.95	950.94	881.18	1017.27	1075.48	1129.17	1082.35
discs, large homolog 4 (Drosophila)	Dlg4	100762874	2.88	2.74	3.16	3.22	2.98	2.94	265.08	256.21	283.20	272.35	262.89	242.08
discs, large homolog 5 (Drosophila)	Dlg5	100762401	13.77	13.53	12.74	13.39	11.52	12.34	3047.27	3018.70	2851.83	2952.93	2528.47	2676.89
discs, large (Drosophila) homolog-associated protein 1	Dlgap1	100774912	0.26	0.23	0.21	0.11	0.11	0.10	19.75	17.39	14.91	8.11	10.35	9.35
discs, large (Drosophila) homolog-associated protein 2	Dlgap2	100758058	0.00	0.00	0.02	0.00	0.00	0.00	0.00	0.00	1.24	0.00	0.00	0.00
discs, large (Drosophila) homolog-associated protein 3	Dlgap3	100760476	0.00	0.00	0.00	0.00	0.00	0.00	0.76	0.92	0.00	0.00	0.00	3.74
discs, large (Drosophila) homolog-associated protein 4	Dlgap4	100752581	13.39	13.13	13.66	11.15	8.23	9.38	1483.38	1444.84	1500.44	1228.46	905.61	1058.05
discs, large (Drosophila) homolog-associated protein 5	Dlgap5	100755750	38.82	42.16	37.29	44.52	53.17	51.59	3277.41	3565.89	3167.32	3740.99	4440.09	4325.65
delta-like 1 homolog (Drosophila)	Dlk1	100762551	0.00	0.00	0.00	0.00	0.00	0.00	0.00	0.00	0.00	0.00	0.00	0.00
delta-like 2 homolog (Drosophila)	Dlk2	100754430	0.09	0.11	0.12	0.06	0.03	0.07	4.56	4.58	4.97	2.32	1.03	2.80
delta-like 1 (Drosophila)	Dll1	100750697	0.00	0.00	0.00	0.00	0.00	0.00	0.00	0.00	0.00	0.00	0.00	0.00
delta-like 3 (Drosophila)	Dll3	100773804	0.00	0.03	0.00	0.00	0.00	0.00	0.00	0.92	0.00	0.00	1.03	0.00
delta-like 4 (Drosophila)	Dll4	100763048	0.01	0.00	0.00	0.00	0.00	0.00	1.52	0.00	0.00	0.00	0.00	0.93
dihydrolipoamide S-succinyltransferase (E2 component of 2-oxo-glutarate complex)	Dlst	100758466	135.16	135.44	137.79	138.09	138.64	143.98	10446.70	10510.08	10775.10	10579.78	10653.11	11113.22
distal-less homeobox 1	Dlx1	100768966	4.22	3.97	3.84	3.43	2.93	3.36	298.50	286.41	275.74	245.69	212.17	240.21
distal-less homeobox 2	Dlx2	100769245	3.00	3.05	2.89	2.54	2.93	3.13	208.87	217.78	211.15	178.47	202.86	223.39
distal-less homeobox 3	Dlx3	100758183	0.00	0.00	0.00	0.00	0.00	0.00	0.00	0.00	0.00	0.00	0.00	0.00
distal-less homeobox 4	Dlx4	100757892	6.70	6.64	2.21	2.23	3.88	3.76	642.57	630.46	202.46	207.45	340.51	332.74
distal-less homeobox 5	Dlx5	100766793	0.00	0.00	0.00	0.00	0.00	0.00	0.00	0.00	0.00	0.00	0.00	0.00
distal-less homeobox 6	Dlx6	100770813	0.00	0.00	0.00	0.00	0.00	0.00	0.00	0.00	0.00	0.00	0.00	0.00
DNA methyltransferase 1 associated protein 1	Dmap1	100756406	33.46	33.34	35.80	32.83	27.46	31.02	1531.99	1562.88	1675.58	1496.17	1293.73	1416.02
deleted in malignant brain tumors 1	Dmbt1	100763251	0.00	0.00	0.00	0.01	0.00	0.00	0.00	0.00	0.00	1.16	0.00	0.00
diencephalon/mesencephalon homeobox 1	Dmbx1	100770688	0.00	0.00	0.00	0.00	0.00	0.00	0.00	0.00	0.00	0.00	0.00	0.00
DNA meiotic recombinase 1	Dmc1	100750348	0.00	0.04	0.10	0.09	0.04	0.00	0.00	0.92	2.48	3.48	3.10	0.93
dystrophin	Dmd	100774409	7.50	7.47	7.79	8.79	9.55	9.45	914.49	912.29	1027.21	1077.80	1199.55	1166.47
dimethylglycine dehydrogenase	Dmgdh	100759503	0.00	0.00	0.00	0.00	0.00	0.00	0.00	0.00	0.00	0.00	0.00	0.00

dermokine	Dmkn	100752030	0.03	0.00	0.13	0.17	0.04	0.04	3.04	0.00	7.45	9.27	2.07	1.87
dentin matrix acidic phosphoprotein 1	Dmp1	100773494	0.00	0.00	0.00	0.00	0.00	0.00	0.00	0.00	0.00	0.00	0.00	0.00
dystrophia myotonica-protein kinase	Dmpk	103164072	0.05	0.18	0.00	0.24	0.07	0.06	4.56	8.24	3.73	3.48	1.03	2.80
doublesex and mab-3 related transcription factor 1	Dmrt1	100757765	0.43	0.36	0.00	0.04	0.00	0.03	15.19	13.73	0.00	1.16	0.00	0.93
doublesex and mab-3 related transcription factor 2	Dmrt2	100756035	0.03	0.02	0.00	0.00	0.00	0.00	1.52	0.92	0.00	0.00	0.00	0.00
doublesex and mab-3 related transcription factor 3	Dmrt3	100759006	0.02	0.00	0.00	0.00	0.00	0.00	1.52	0.00	0.00	0.00	0.00	0.00
DMRT-like family B with proline-rich C-terminal, 1	Dmrtb1	100772202	0.00	0.00	0.00	0.00	0.00	0.05	0.00	0.00	0.00	0.00	0.00	1.87
DMRT-like family C2	Dmrtc2	100759114	0.00	0.00	0.00	0.00	0.00	0.00	0.00	0.00	0.00	0.00	0.00	0.00
cyclin D binding myb-like transcription factor 1	Dmtf1	100760330	30.36	31.28	27.90	31.34	34.70	31.56	3086.77	3189.81	2855.56	3197.46	3504.46	3180.68
dematin actin binding protein	Dmtn	100764907	3.81	3.76	2.72	3.03	2.69	3.29	285.59	277.26	211.15	210.92	208.03	243.01
dystrophia myotonica, WD repeat containing	Dmwd	100757796	13.18	11.81	11.97	7.62	4.95	4.30	622.06	572.81	541.55	356.95	227.70	214.04
Dmx-like 1	Dmxl1	100773136	2.75	2.75	2.95	3.71	3.84	3.44	916.00	919.61	1013.54	1231.93	1269.93	1134.69
Dmx-like 2	Dmxl2	100753187	0.01	0.00	0.00	0.00	0.00	0.00	3.04	0.00	1.24	0.00	0.00	0.93
DNA replication helicase/nuclease 2	Dna2	100761842	1.46	1.33	1.60	2.41	2.39	2.14	120.77	112.55	144.08	187.75	193.54	162.63
dynein, axonemal, assembly factor 1	Dnaaf1	100774628	2.51	2.75	2.25	2.15	2.37	2.22	163.30	175.69	145.32	139.07	155.25	147.68
dynein, axonemal, assembly factor 2	Dnaaf2	100753981	11.33	11.70	15.69	17.01	17.91	17.54	483.07	504.18	665.76	717.37	765.89	742.13
dynein, axonemal, heavy chain 1	Dnah1	100765769	0.00	0.00	0.01	0.00	0.00	0.00	4.56	3.66	7.45	4.64	8.28	10.28
dynein, axonemal, heavy chain 10	Dnah10	103161549	0.00	0.00	0.00	0.00	0.00	0.00	0.76	1.83	0.00	1.16	1.03	0.93
dynein, axonemal, heavy chain 11	Dnah11	100770442	0.00	0.00	0.00	0.00	0.00	0.00	0.00	0.00	0.00	0.00	0.00	0.00
dynein, axonemal, heavy chain 12	Dnah12	100750737	0.66	0.67	0.65	0.72	0.72	0.67	304.58	296.47	288.16	343.04	337.41	299.09
dynein, axonemal, heavy chain 14	Dnah14	100752669	0.04	0.04	0.02	0.01	0.04	0.03	10.63	13.73	3.73	5.79	16.56	6.54
dynein, axonemal, heavy chain 17	Dnah17	100768695	0.11	0.08	0.10	0.07	0.06	0.08	47.09	32.94	44.72	34.77	25.87	39.26
dynein, axonemal, heavy chain 2	Dnah2	100756503	0.00	0.00	0.00	0.00	0.00	0.00	0.76	3.66	0.00	1.16	0.00	0.00
dynein, axonemal, heavy chain 3	Dnah3	100752771	0.00	0.00	0.00	0.00	0.00	0.00	0.00	0.92	1.24	0.00	0.00	0.93
dynein, axonemal, heavy chain 5	Dnah5	100771964	0.01	0.02	0.00	0.00	0.02	0.02	5.32	10.07	1.24	0.00	10.35	7.48
dynein, axonemal, heavy chain 6	Dnah6	100754128	0.00	0.00	0.00	0.00	0.00	0.00	1.52	2.75	0.00	0.00	0.00	1.87
dynein, axonemal, heavy chain 7	Dnah7	100752773	11.12	10.80	12.59	13.37	13.74	11.96	582.57	597.52	696.81	744.03	765.89	651.46
dynein, axonemal, heavy chain 8	Dnah8	100763108	1.80	1.88	2.14	2.27	2.30	2.19	857.52	892.16	994.91	1044.19	1074.32	1029.07
dynein, axonemal, heavy chain 9	Dnah9	100770690	0.01	0.01	0.03	0.01	0.02	0.01	4.56	6.41	16.15	3.48	10.35	11.22
dynein, axonemal, intermediate chain 1	Dnai1	100754021	0.00	0.00	0.00	0.00	0.00	0.03	0.00	0.00	0.00	0.00	0.00	0.93
dynein, axonemal, intermediate chain 2	Dnai2	100761698	0.27	0.10	0.05	0.19	0.06	0.05	21.27	10.98	7.45	16.22	7.24	6.54
DnaJ (Hsp40) homolog, subfamily A, member 1	Dnaja1	100689381	36.94	38.02	44.24	51.49	51.09	49.63	1879.86	1900.52	2212.16	2569.33	2564.70	2488.09
DnaJ (Hsp40) homolog, subfamily A, member 2	Dnaja2	100766736	74.59	73.98	75.51	80.48	84.07	84.12	4549.64	4596.21	4729.87	4937.00	5145.95	5112.64
DnaJ (Hsp40) homolog, subfamily A, member 3	Dnaja3	100774313	51.91	49.86	58.57	60.86	56.59	58.98	3822.76	3761.70	4380.84	4465.32	4154.44	4328.46

DnaJ (Hsp40) homolog, subfamily A, member 4	Dnaja4	100755057	0.36	0.40	0.33	0.46	0.44	0.59	36.46	45.75	37.26	52.15	43.47	62.62
DnaJ (Hsp40) homolog, subfamily B, member 1	Dnajb1	100753924	8.10	8.32	10.35	10.41	9.25	10.38	496.74	505.10	632.22	632.77	556.82	642.12
DnaJ (Hsp40) homolog, subfamily B, member 11	Dnajb11	100765455	63.77	64.80	66.78	71.34	73.28	72.28	3032.08	3090.98	3225.70	3394.48	3484.80	3418.09
DnaJ (Hsp40) homolog, subfamily B, member 12	Dnajb12	100759717	27.13	26.52	25.80	23.32	19.50	20.51	2435.08	2394.64	2357.48	2123.14	1769.83	1859.06
DnaJ (Hsp40) homolog, subfamily B, member 13	Dnajb13	100759263	0.97	1.11	0.25	0.53	0.51	0.70	48.61	55.82	9.94	28.97	26.91	34.58
DnaJ (Hsp40) homolog, subfamily B, member 14	Dnajb14	100752201	7.17	4.83	6.16	9.27	2.23	3.96	196.72	159.22	170.17	173.84	162.49	156.09
DnaJ (Hsp40) homolog, subfamily B, member 2	Dnajb2	100773438	41.76	39.08	48.52	46.58	39.92	45.10	2474.58	2349.81	2904.00	2798.79	2420.83	2635.77
DnaJ (Hsp40) homolog, subfamily B, member 3	Dnajb3	100755138	1.95	1.97	1.25	1.73	1.01	1.62	47.85	46.67	31.05	42.88	23.80	41.13
DnaJ (Hsp40) homolog, subfamily B, member 4	Dnajb4	100757372	9.45	10.16	11.93	14.13	15.53	15.17	524.84	549.94	663.27	797.34	850.76	844.01
DnaJ (Hsp40) homolog, subfamily B, member 5	Dnajb5	100750493	1.17	1.39	0.91	1.26	1.01	0.81	107.10	128.10	83.22	117.05	92.11	77.58
DnaJ (Hsp40) homolog, subfamily B, member 6	Dnajb6	100758507	93.08	98.44	86.48	89.79	95.81	94.99	2771.56	2972.94	2501.56	2586.71	2675.44	2693.72
DnaJ (Hsp40) homolog, subfamily B, member 7	Dnajb7	100762842	0.00	0.00	0.05	0.00	0.04	0.00	0.00	0.00	1.24	0.00	1.03	0.00
DnaJ (Hsp40) homolog, subfamily B, member 8	Dnajb8	100762038	0.00	0.00	0.00	0.00	0.00	0.00	0.00	0.00	0.00	0.00	0.00	0.00
DnaJ (Hsp40) homolog, subfamily B, member 9	Dnajb9	100763648	9.63	9.77	15.00	15.58	15.92	15.40	634.22	652.42	1004.85	1046.50	1045.34	1029.07
DnaJ (Hsp40) homolog, subfamily C, member 1	Dnajc1	100751540	5.05	4.79	5.21	6.03	5.47	5.23	285.59	301.96	286.92	321.02	324.99	288.81
DnaJ (Hsp40) homolog, subfamily C, member 10	Dnajc10	100689230	50.71	52.73	63.59	72.41	78.34	72.90	5745.15	6057.52	7278.63	8110.12	8826.36	8146.58
DnaJ (Hsp40) homolog, subfamily C, member 11	Dnajc11	100760814	40.29	40.99	40.36	38.75	29.57	33.35	4143.29	4200.00	4157.26	4002.91	3133.94	3490.99
DnaJ (Hsp40) homolog, subfamily C, member 12	Dnajc12	100758554	0.93	1.19	0.65	0.91	1.21	1.50	44.81	45.75	27.33	41.72	39.33	62.62
DnaJ (Hsp40) homolog, subfamily C, member 13	Dnajc13	100763365	20.25	20.14	19.14	22.68	24.54	23.58	4724.33	4805.76	4604.42	5405.20	5775.22	5540.72
DnaJ (Hsp40) homolog, subfamily C, member 14	Dnajc14	100771122	21.71	20.60	24.25	23.78	32.16	32.74	2322.67	2219.87	2633.22	2584.39	3529.30	3599.41
DnaJ (Hsp40) homolog, subfamily C, member 15	Dnajc15	100770437	42.95	38.70	37.92	38.00	44.31	43.38	1168.17	1122.75	1049.56	1032.60	1181.95	1173.01
DnaJ (Hsp40) homolog, subfamily C, member 16	Dnajc16	100751780	8.63	8.42	9.43	8.60	8.86	9.23	1159.06	1141.05	1281.83	1169.35	1195.41	1271.15
DnaJ (Hsp40) homolog, subfamily C, member 17	Dnajc17	100752047	17.18	17.26	20.10	17.48	15.81	17.36	502.06	495.95	587.51	485.59	457.46	493.51
DnaJ (Hsp40) homolog, subfamily C, member 18	Dnajc18	100756658	5.61	6.42	7.39	7.20	9.16	8.56	202.04	243.40	262.08	270.03	324.99	315.92
DnaJ (Hsp40) homolog, subfamily C, member 19	Dnajc19	100751082	24.73	30.23	26.08	27.91	30.78	28.75	587.88	679.87	597.44	646.68	734.84	653.33
DnaJ (Hsp40) homolog, subfamily C, member 2	Dnajc2	100757311	82.26	82.28	84.48	95.47	87.43	80.31	5477.04	5613.73	5755.83	6401.87	5826.97	5254.71
DnaJ (Hsp40) homolog, subfamily C, member 21	Dnajc21	100759616	30.58	29.62	28.41	31.43	30.70	27.35	1755.30	1723.01	1639.55	1775.47	1746.02	1544.07

DnaJ (Hsp40) homolog, subfamily C, member 22	Dnajc22	100769451	29.90	28.64	25.56	27.47	23.91	27.66	1423.38	1360.65	1245.81	1309.58	1132.28	1284.24
DnaJ (Hsp40) homolog, subfamily C, member 24	Dnajc24	100766077	17.03	17.53	12.62	15.94	19.53	17.55	1003.35	1032.16	724.14	925.98	1085.70	1007.57
DnaJ (Hsp40) homolog, subfamily C , member 25	Dnajc25	100756280	21.75	21.37	24.24	24.14	27.44	27.92	623.58	629.54	686.87	693.03	793.83	788.86
DnaJ (Hsp40) homolog, subfamily C, member 27	Dnajc27	100756026	2.34	1.97	2.22	2.44	2.31	2.01	307.61	260.78	296.86	319.86	300.15	268.25
DnaJ (Hsp40) homolog, subfamily C, member 28	Dnajc28	100769155	2.74	3.02	2.60	3.50	4.58	3.29	113.93	120.78	105.58	137.91	179.05	142.07
DnaJ (Hsp40) homolog, subfamily C, member 3	Dnajc3	100766172	15.85	16.73	17.18	19.54	20.57	18.74	2273.30	2411.11	2501.56	2780.25	2924.87	2644.18
DnaJ (Hsp40) homolog, subfamily C, member 30	Dnajc30	103162154	4.85	5.52	4.92	5.42	5.91	5.66	212.67	238.82	214.88	237.58	254.61	245.82
DnaJ (Hsp40) homolog, subfamily C, member 4	Dnajc4	100756870	9.34	9.47	13.43	12.38	9.87	8.33	290.14	304.71	397.47	354.63	302.22	269.18
DnaJ (Hsp40) homolog, subfamily C, member 5	Dnajc5	100763676	63.98	64.81	61.22	63.73	68.02	71.23	7655.40	7767.72	7394.14	7634.96	8057.37	8569.99
DnaJ (Hsp40) homolog, subfamily C, member 5 beta	Dnajc5b	100774239	0.23	0.00	0.19	0.00	0.32	0.00	3.04	0.00	2.48	0.00	4.14	0.00
DnaJ (Hsp40) homolog, subfamily C, member 6	Dnajc6	100756272	1.19	1.30	1.71	1.65	1.62	1.72	195.20	212.29	254.63	254.96	244.26	268.25
DnaJ (Hsp40) homolog, subfamily C, member 7	Dnajc7	100753520	201.80	199.29	141.18	140.18	123.31	128.94	6380.89	6512.29	4329.91	4504.72	4209.29	4340.61
DnaJ (Hsp40) homolog, subfamily C, member 8	Dnajc8	100771757	143.05	149.08	145.90	142.21	137.03	138.12	4907.38	5108.63	4855.32	4698.26	4553.94	4585.49
DnaJ (Hsp40) homolog, subfamily C, member 9	Dnajc9	100762162	59.77	62.09	81.64	82.86	90.84	87.26	2071.26	2162.22	2794.70	2843.99	3075.98	2963.84
dynein, axonemal, light chain 1	Dnal1	100774309	4.04	4.46	4.45	4.46	6.39	6.18	183.05	225.10	213.64	217.88	291.87	277.60
dynein, axonemal, light chain 4	Dnal4	100772767	11.85	11.63	13.54	13.78	12.04	12.24	461.80	453.86	522.92	528.47	460.57	472.01
dynein, axonemal, light intermediate chain 1	Dnali1	100754224	0.30	0.22	0.50	0.45	0.37	0.48	23.55	19.22	36.02	34.77	23.80	29.91
deoxyribonuclease I	Dnase1	100764185	0.26	0.36	0.13	0.17	0.39	0.16	23.55	25.62	14.91	11.59	25.87	16.82
deoxyribonuclease I-like 1	Dnase1l1	100689095	4.02	4.20	5.56	5.35	5.93	6.02	241.53	257.12	336.61	327.97	357.07	365.46
deoxyribonuclease I-like 2	Dnase1l2	100770467	0.52	0.33	0.45	0.42	0.32	0.66	2.28	1.83	3.73	2.32	0.00	7.48
deoxyribonuclease I-like 3	Dnase1l3	100769677	4.88	5.12	3.42	2.82	4.25	4.48	207.35	201.31	137.87	130.96	197.68	193.48
deoxyribonuclease II, lysosomal	Dnase2	100763100	49.95	46.87	65.66	63.50	64.68	69.75	2160.13	2064.32	2855.56	2744.32	2793.43	2990.01
deoxyribonuclease II beta	Dnase2b	100767962	0.00	0.00	0.00	0.00	0.00	0.00	0.00	0.00	0.00	0.00	0.00	0.00
DND microRNA-mediated repression inhibitor 1	Dnd1	100755307	0.28	0.28	0.03	0.20	0.09	0.16	17.47	15.56	6.21	13.91	6.21	7.48
delta/notch-like EGF repeat containing	Dner	100755447	0.52	0.47	0.00	0.00	0.00	0.00	3.04	3.66	0.00	0.00	0.00	0.00
dynein heavy chain domain 1	Dnhd1	100761219	0.10	0.08	0.08	0.11	0.08	0.09	66.08	53.99	50.93	75.33	57.96	62.62
DNL-type zinc finger	Dnlz	100761013	20.44	17.29	21.86	15.58	10.69	13.70	364.58	309.28	376.35	280.46	194.58	248.62
dynamin 1	Dnm1	100753867	0.46	0.48	0.23	0.04	0.04	0.08	56.97	62.22	36.02	13.91	6.21	13.09
dynamin 1-like	Dnm1l	100750819	30.47	29.73	32.01	37.07	39.82	35.35	3610.09	3601.57	3845.50	4410.85	4709.19	4229.38
dynamin 2	Dnm2	100771373	17.04	16.76	18.68	18.81	16.95	17.45	1952.02	1979.22	2254.39	2198.47	2024.43	2118.90

dynamin 3	Dnm3	100772324	0.04	0.03	0.02	0.05	0.00	0.00	3.80	2.75	1.24	2.32	0.00	0.00
dynamin binding protein	Dnmbp	100752556	11.16	10.54	12.27	11.76	9.74	10.56	972.97	927.84	1084.34	1026.80	859.04	922.52
DNA (cytosine-5-)-methyltransferase 1	Dnmt1	100762713	37.58	38.72	45.37	47.06	44.93	45.92	6272.27	6525.10	7633.87	7804.17	7400.15	7606.34
DNA (cytosine-5-)-methyltransferase 3 alpha	Dnmt3a	100771064	5.27	5.17	4.88	4.86	4.29	4.39	1414.26	1400.92	1351.39	1336.23	1164.36	1199.18
DNA (cytosine-5-)-methyltransferase 3-like	Dnmt3l	100768334	0.00	0.00	0.00	0.00	0.00	0.00	0.00	0.00	0.00	0.00	0.00	0.00
aspartyl aminopeptidase	Dnpep	100764694	37.10	36.51	38.87	34.91	27.45	30.81	1917.08	1918.83	2084.22	1803.28	1494.52	1584.27
2'-deoxynucleoside 5'-phosphate N-hydrolase 1	Dnph1	100756461	29.09	22.57	23.47	19.84	16.21	14.43	363.82	296.47	290.65	236.42	195.61	177.59
DNA nucleotidylexotransferase	Dntt	100755735	0.00	0.00	0.00	0.00	0.02	0.00	0.76	0.92	0.00	0.00	1.03	0.00
deoxynucleotidyltransferase, terminal, interacting protein 1	Dnttip1	100769006	44.08	44.24	47.71	48.65	48.96	52.61	1453.76	1450.33	1565.03	1542.52	1594.91	1693.62
deoxynucleotidyltransferase, terminal, interacting protein 2	Dnttip2	100774727	113.79	111.15	111.48	133.39	121.88	110.10	7870.35	7723.80	7764.28	9223.84	8406.16	7593.26
double C2-like domains, alpha	Doc2a	100773354	3.41	3.16	2.69	1.55	0.99	1.32	391.92	349.54	428.52	361.58	284.62	310.31
double C2-like domains, beta	Doc2b	100759731	0.00	0.00	0.04	0.00	0.00	0.00	0.00	0.00	0.00	0.00	0.00	0.00
dedicator of cytokinesis 10	Dock10	100768884	0.44	0.51	0.27	0.27	0.19	0.17	34.94	32.03	17.39	17.38	13.45	12.15
dedicator of cytokinesis 11	Dock11	100771666	6.69	6.72	7.35	8.87	10.43	9.78	1432.49	1452.16	1602.29	1889.04	2223.15	2054.40
dedicator of cytokinesis 2	Dock2	100766266	0.00	0.00	0.00	0.00	0.00	0.00	0.00	0.00	0.00	0.00	0.00	0.00
dedicator of cytokinesis 3	Dock3	100768911	2.60	2.40	2.73	3.13	3.00	2.76	755.74	705.49	787.48	917.86	839.37	801.95
dedicator of cytokinesis 4	Dock4	100772385	4.88	4.69	10.93	13.06	11.67	11.88	1249.44	1214.25	2823.26	3323.78	2918.66	3030.20
dedicator of cytokinesis 5	Dock5	100752876	21.89	22.18	20.52	23.75	26.96	26.13	3184.75	3220.00	3017.03	3382.89	3849.11	3757.37
dedicator of cytokinesis 6	Dock6	100769954	0.58	0.60	0.51	0.47	0.37	0.35	139.76	147.32	116.76	112.42	83.83	83.19
dedicator of cytokinesis 7	Dock7	100758142	27.85	28.21	28.04	32.07	36.25	34.11	5504.38	5597.26	5581.94	6311.48	7103.11	6734.30
dedicator of cytokinesis 8	Dock8	100757085	9.70	9.50	8.62	8.53	9.11	9.46	1667.95	1663.53	1515.35	1467.19	1544.20	1623.52
dedicator of cytokinesis 9	Dock9	100768424	4.42	4.28	4.15	4.64	5.02	4.63	1036.77	1060.52	1019.75	1109.09	1213.00	1088.89
deoxyhypusine hydroxylase/monooxygenase	Dohh	100771021	29.58	27.99	24.68	11.55	6.33	6.93	1311.72	1246.28	1093.04	522.67	315.67	307.51
docking protein 1, 62kDa (downstream of tyrosine kinase 1)	Dok1	100760042	15.57	15.00	17.44	14.78	13.33	13.50	1014.74	984.58	1096.76	927.14	831.09	915.04
docking protein 2, 56kDa	Dok2	100764015	0.03	0.10	0.00	0.00	0.02	0.00	2.28	4.58	0.00	0.00	1.03	0.00
docking protein 3	Dok3	100763567	0.31	0.13	0.08	0.15	0.07	0.08	18.99	9.15	4.97	10.43	5.17	6.54
docking protein 4	Dok4	100766625	4.71	4.46	3.43	4.51	4.07	4.37	285.59	282.75	221.09	292.05	249.43	262.64
docking protein 5	Dok5	100751739	15.48	16.06	15.34	14.97	19.31	20.60	607.63	640.52	622.29	553.96	721.39	786.99
docking protein 6	Dok6	100763321	0.00	0.00	0.00	0.00	0.00	0.00	0.00	0.00	0.00	0.00	0.00	0.00
docking protein 7	Dok7	100765890	0.00	0.00	0.00	0.00	0.00	0.00	0.00	0.00	0.00	0.00	0.00	0.00
dolichol kinase	Dolk	100768385	6.48	6.30	5.84	5.98	5.52	5.79	362.30	354.12	329.15	336.09	305.32	320.59
dolichyldiphosphatase 1	Dolpp1	100761712	10.21	10.92	9.19	9.35	9.40	8.50	464.84	486.80	412.37	389.40	387.08	362.65
downstream neighbor of SON	Donson	100769724	15.18	14.73	17.56	19.51	22.66	21.83	1065.63	1037.65	1235.88	1371.00	1601.12	1542.20
dopey family member 1	Dopey1	100763056	5.40	5.44	2.68	3.22	3.53	3.31	1275.27	1296.60	647.13	766.05	830.06	793.53
dopey family member 2	Dopey2	100751759	2.15	2.46	2.20	2.27	2.33	2.03	505.09	579.22	512.98	527.31	541.30	486.03
DOT1-like histone H3K79 methyltransferase	Dot1l	100771005	12.95	12.40	10.31	7.63	5.01	5.61	2195.07	2094.51	1724.02	1321.17	905.61	1008.51

dolichyl-phosphate (UDP-N-acetylglucosamine) N-acetylglucosaminephosphotransferase 1 (GlcNAc-1-P transferase)	Dpagt1	100689054	95.59	110.27	107.11	113.77	136.71	127.23	2779.15	2949.15	3413.25	3487.19	3725.95	3621.85
deleted in primary ciliary dyskinesia homolog (mouse)	Dpcd	100751595	13.47	13.89	16.06	14.49	13.74	14.18	416.23	438.30	479.45	441.55	410.89	425.27
diffuse panbronchiolitis critical region 1	Dpcr1	100755283	0.00	0.02	0.00	0.06	0.00	0.02	0.00	0.92	0.00	2.32	1.03	1.87
dipeptidase 1 (renal)	Dpep1	100762968	0.04	0.13	0.00	0.03	0.00	0.03	2.28	8.24	0.00	1.16	0.00	0.93
dipeptidase 3	Dpep3	100756154	0.04	0.07	0.00	0.00	0.00	0.00	1.52	2.75	0.00	0.00	0.00	0.00
D4, zinc and double PHD fingers family 2	Dpf2	100767725	41.02	37.10	42.83	42.68	40.28	43.36	2820.17	2556.60	3012.06	2940.18	2790.32	2956.36
D4, zinc and double PHD fingers, family 3	Dpf3	100760706	2.10	1.92	1.46	1.03	1.77	2.00	70.64	75.95	50.93	40.56	60.03	61.69
diphthamide biosynthesis 1	Dph1	100765648	73.66	69.49	74.37	68.12	48.75	50.90	3120.19	2983.01	3228.18	2941.34	2189.00	2235.73
DPH2 homolog (S. cerevisiae)	Dph2	100689365	20.87	21.77	22.59	19.43	12.87	14.84	1060.32	1102.62	1138.99	998.99	704.83	764.56
diphthamide biosynthesis 3	Dph3	100689295	3.06	2.90	2.07	3.36	2.25	5.97						
diphthamide biosynthesis 3	Dph3	100768092	84.29	78.59	69.50	72.27	70.43	71.88	736.75	629.54	585.02	614.23	597.19	665.48
diphthamide biosynthesis 5	Dph5	100755934	30.56	30.75	23.86	26.18	22.39	18.76	513.45	537.12	417.34	441.55	371.56	327.13
diphthamine biosynthesis 6	Dph6	100765802	16.50	17.31	14.76	16.91	16.56	19.41	418.51	436.47	375.11	391.71	413.99	468.27
diphthamide biosynthesis 7	Dph7	100753959	7.90	7.88	9.22	9.06	8.49	8.56	789.92	786.93	911.69	895.85	863.18	877.65
dolichyl-phosphate mannosyltransferase polypeptide 1, catalytic subunit	Dpm1	100689420	52.82	53.45	49.95	53.74	66.14	64.97	1555.54	1562.88	1473.11	1522.82	1909.55	1817.93
dolichyl-phosphate mannosyltransferase polypeptide 2, regulatory subunit	Dpm2	100689294	52.13	50.07	56.17	55.51	48.01	57.87	977.53	962.62	1029.69	954.95	820.74	931.87
dolichyl-phosphate mannosyltransferase polypeptide 3	Dpm3	100689451	12.64	12.19	14.52	3.81	0.83	1.97	88.87	81.44	100.61	27.81	7.24	14.02
dipeptidyl-peptidase 10 (non-functional)	Dpp10	100761750	0.00	0.00	0.00	0.00	0.00	0.00	0.00	0.00	0.00	0.00	0.00	0.00
dipeptidyl-peptidase 3	Dpp3	100760621	49.79	49.95	54.90	52.42	46.04	50.77	3484.77	3541.18	3879.04	3676.09	3134.97	3458.28
dipeptidyl-peptidase 4	Dpp4	100768361	0.00	0.00	0.00	0.00	0.00	0.01	0.76	0.00	0.00	0.00	0.00	0.93
dipeptidyl-peptidase 6	Dpp6	100774564	0.00	0.00	0.00	0.00	0.00	0.00	0.00	0.00	0.00	0.00	0.00	0.00
dipeptidyl-peptidase 7	Dpp7	100756223	77.43	75.80	89.85	84.45	67.11	75.85	3967.07	3888.89	4557.22	4198.77	3370.95	3815.32
dipeptidyl-peptidase 8	Dpp8	100773914	31.40	32.04	35.50	38.48	41.80	40.68	4978.78	5104.06	5631.62	6090.12	6546.29	6448.29
dipeptidyl-peptidase 9	Dpp9	100757878	20.66	20.23	18.31	15.81	11.89	13.33	2365.21	2365.36	2110.31	1747.65	1379.64	1493.60
developmental pluripotency associated 2	Dppa2	100760093	0.00	0.04	0.00	0.00	0.00	0.00	0.00	0.92	0.00	0.00	0.00	0.00
developmental pluripotency associated 4	Dppa4	100760387	0.00	0.00	0.00	0.00	0.00	0.00	0.00	0.00	0.00	0.00	0.00	0.00
developmental pluripotency associated 5	Dppa5	100755380	0.00	0.00	0.23	0.00	0.00	0.00	0.00	0.00	1.24	0.00	0.00	0.00
dermatopontin	Dpt	100765812	0.00	0.00	0.00	0.00	0.00	0.00	0.00	0.00	0.00	0.00	0.00	0.00
dpy-19-like 1 (C. elegans)	Dpy19l1	100765722	12.97	12.92	12.74	13.92	16.45	16.48	1776.56	1778.83	1755.07	1921.49	2247.99	2252.55
dpy-19-like 2 (C. elegans)	Dpy19l2	100752353	0.00	0.00	0.00	0.00	0.00	0.00	0.00	0.00	0.00	0.00	0.00	0.00
dpy-19-like 3 (C. elegans)	Dpy19l3	100756435	19.07	19.38	17.17	16.06	16.66	17.05	3180.19	3250.20	2944.99	2673.63	2803.78	2885.33
dpy-19-like 4 (C. elegans)	Dpy19l4	100759810	3.86	3.58	4.57	4.66	5.15	4.68	398.00	378.82	481.93	475.16	522.67	485.09

dpy-30 homolog (C. elegans)	Dpy30	100763394	91.66	90.63	95.51	93.57	106.01	97.32	1087.66	1035.82	1112.91	1131.11	1374.46	1225.35
dihydropyrimidine dehydrogenase	Dpyd	100765169	16.82	17.47	23.00	27.25	20.68	25.43	280.27	326.67	481.93	501.81	466.78	485.09
dihydropyrimidinase	Dpys	100752636	0.00	0.00	0.00	0.00	0.00	0.00	0.00	0.00	0.00	0.00	0.00	0.00
dihydropyrimidinase-like 2	Dpysl2	100759148	10.69	10.55	13.90	14.65	16.05	16.87	1485.66	1490.59	1988.58	2064.04	2254.20	2370.32
dihydropyrimidinase-like 3	Dpysl3	100755197	0.16	0.09	0.00	0.03	0.02	0.01	25.06	17.39	1.24	4.64	1.03	0.93
dihydropyrimidinase-like 4	Dpysl4	100774927	0.00	0.00	0.00	0.00	0.00	0.00	0.00	0.00	0.00	0.00	0.00	0.00
dihydropyrimidinase-like 5	Dpysl5	100761240	0.00	0.00	0.00	0.00	0.00	0.00	0.76	0.00	0.00	2.32	0.00	1.87
DEAQ box RNA-dependent ATPase 1	Dqx1	100761206	1.66	1.32	1.75	1.80	1.35	1.58	166.34	144.58	182.59	187.75	165.60	174.78
down-regulator of transcription 1, TBP-binding (negative cofactor 2)	Dr1	100765234	66.75	70.00	76.03	77.39	81.03	77.83	2989.55	3101.05	3436.85	3519.64	3696.97	3673.25
DNA-damage regulated autophagy modulator 1	Dram1	100774664	6.03	5.66	5.04	6.16	6.84	5.99	516.49	482.22	447.15	526.15	582.70	523.42
DNA-damage regulated autophagy modulator 2	Dram2	100772594	8.46	7.44	6.95	8.99	10.01	9.29	495.98	465.75	419.83	526.15	572.35	551.46
DR1-associated protein 1 (negative cofactor 2 alpha)	Drap1	100765946	253.91	254.34	273.46	218.78	186.25	198.70	5931.24	6070.33	6451.40	5198.91	4307.61	4451.83
dynein regulatory complex subunit 1 homolog (Chlamydomonas)	Drc1	100759288	0.04	0.03	0.02	0.02	0.02	0.01	3.80	2.75	1.24	1.16	4.14	0.93
dopamine receptor D1	Drd1	100760963	0.00	0.00	0.00	0.03	0.00	0.00	0.00	0.00	0.00	1.16	0.00	0.00
dopamine receptor D2	Drd2	100751925	0.00	0.00	0.00	0.00	0.00	0.00	0.00	0.00	0.00	0.00	0.00	0.00
dopamine receptor D3	Drd3	100769070	0.00	0.03	0.00	0.00	0.00	0.00	0.00	0.92	0.00	0.00	0.00	0.00
dopamine receptor D4	Drd4	100751093	0.00	0.00	0.00	0.00	0.00	0.00	0.00	0.00	0.00	1.16	0.00	0.00
dopamine receptor D5	Drd5	100752680	0.00	0.00	0.00	0.00	0.00	0.00	0.00	0.00	0.00	0.00	0.00	0.00
developmentally regulated GTP binding protein 1	Drg1	100764489	39.75	38.83	41.96	37.54	40.37	41.17	1680.86	1673.60	1837.05	1585.40	1726.36	1741.29
developmentally regulated GTP binding protein 2	Drg2	100771930	28.50	28.19	31.56	29.74	26.39	28.29	1458.32	1436.60	1612.23	1498.48	1338.24	1446.87
dorsal root ganglia homeobox	Drgx	100770585	0.04	0.01	0.00	0.00	0.03	0.03	2.28	0.92	0.00	0.00	2.07	2.80
drosha, ribonuclease type III	Drosha	100766983	37.48	37.16	43.50	46.48	47.30	47.72	5182.34	5160.79	6067.59	6325.38	6427.26	6546.43
dystrophin related protein 2	Drp2	100771867	0.02	0.00	0.08	0.00	0.04	0.01	2.28	0.00	6.21	1.16	5.17	1.87
desmocollin 1	Dsc1	100762663	0.00	0.00	0.00	0.00	0.00	0.00	0.00	0.00	0.00	0.00	0.00	0.00
desmocollin 2	Dsc2	100762954	0.00	0.00	0.00	0.00	0.00	0.00	0.00	0.00	0.00	0.00	0.00	0.00
desmocollin 3	Dsc3	100771819	0.00	0.00	0.00	0.00	0.00	0.00	0.00	0.00	0.00	0.00	0.00	0.00
Down syndrome cell adhesion molecule	Dscam	100765236	0.03	0.02	0.01	0.02	0.00	0.00	4.56	2.75	1.24	3.48	0.00	0.00
Down syndrome cell adhesion molecule like 1	Dscaml 1	100763436	0.00	0.00	0.01	0.00	0.00	0.00	0.00	0.00	1.24	0.00	0.00	0.00
DNA replication and sister chromatid cohesion 1	Dscc1	103159024	33.99	27.64	18.32	28.28	27.69	23.02	141.27	140.00	118.00	124.00	156.28	157.96
Down syndrome critical region gene 3	Dscr3	100763887	44.31	43.41	52.36	48.87	45.29	48.61	2735.10	2696.60	3267.93	3023.62	2829.65	3052.63
dermatan sulfate epimerase	Dse	100775050	14.45	14.36	8.02	8.80	9.34	8.07	1635.29	1627.84	910.45	989.72	1051.55	915.98
dermatan sulfate epimerase-like	Dsel	100756462	1.44	1.60	1.67	1.97	1.97	2.11	353.19	404.44	408.65	467.04	464.71	500.05
desmoglein 1	Dsg1	100762370	0.00	0.00	0.00	0.00	0.00	0.00	0.00	0.00	0.00	0.00	0.00	0.00
desmoglein 3	Dsg3	100761782	0.00	0.00	0.00	0.00	0.00	0.00	0.00	0.00	0.00	0.00	0.00	0.00
desmoglein 4	Dsg4	100762072	0.00	0.00	0.00	0.00	0.00	0.00	0.00	0.00	0.00	0.00	0.00	0.00
DSN1, MIS12 kinetochore complex component	Dsn1	100754649	43.94	47.03	47.86	55.07	70.47	68.65	4043.79	4356.47	4500.08	5033.19	6328.94	6121.15
desmoplakin	Dsp	100767947	5.17	5.39	4.72	4.98	6.84	6.90	1407.43	1465.88	1296.74	1364.05	1859.87	1884.29

dentin sialophosphoprotein	Dspp	100754381	0.00	0.00	0.00	0.00	0.00	0.00	0.00	0.00	0.00	0.00	0.00	0.00
dystonin	Dst	100773919	13.02	12.01	11.07	13.31	14.07	13.72	6895.10	6507.72	6020.39	7145.90	7663.04	7349.31
destrin (actin depolymerizing factor)	Dstn	100769161	72.25	77.64	69.10	71.21	83.06	77.57	3370.84	3613.47	3230.67	3261.20	3781.84	3556.42
dual serine/threonine and tyrosine protein kinase	Dstyk	100759895	2.12	2.07	2.21	2.28	2.53	2.65	378.25	376.08	402.44	411.42	457.46	474.81
D-tyrosyl-tRNA deacylase 1	Dtd1	100765967	29.18	27.30	25.59	22.72	19.08	20.95	783.08	756.73	680.66	589.89	525.77	536.50
D-tyrosyl-tRNA deacylase 2 (putative)	Dtd2	100752381	5.90	6.24	6.33	6.08	5.98	6.17	422.30	445.62	457.09	436.91	426.41	439.29
death domain containing 1	Dthd1	100751886	0.00	0.00	0.00	0.00	0.00	0.00	0.00	0.00	0.00	1.16	0.00	0.00
denticleless E3 ubiquitin protein ligase homolog (Drosophila)	Dtl	100750427	23.43	23.16	27.68	29.04	29.70	31.41	2373.56	2373.60	2874.19	2996.97	3072.87	3172.27
dystrobrevin, alpha	Dtna	100761929	0.02	0.02	0.00	0.02	0.00	0.00	3.04	1.83	0.00	1.16	0.00	0.00
dystrobrevin, beta	Dtnb	100756605	12.55	12.58	11.15	10.62	8.91	9.03	907.65	915.03	817.29	749.82	634.45	629.97
dystrobrevin binding protein 1	Dtnbp1	100760065	23.30	26.74	30.66	29.32	25.35	26.05	735.23	807.97	951.44	891.21	755.54	821.57
DTW domain containing 1	Dtwd1	100761309	8.26	8.72	8.36	8.79	10.37	9.79	897.78	938.82	912.93	965.38	1106.40	1070.20
DTW domain containing 2	Dtwd2	100773422	0.00	0.00	0.00	0.00	0.00	0.00	0.00	0.00	0.00	0.00	0.00	0.00
deltex 1, E3 ubiquitin ligase	Dtx1	100772295	0.00	0.00	0.00	0.00	0.00	0.00	0.76	0.00	0.00	0.00	0.00	0.00
deltex 2, E3 ubiquitin ligase	Dtx2	100764941	7.38	7.18	7.49	5.17	3.61	3.84	532.44	516.99	565.15	377.81	267.03	298.16
deltex 3, E3 ubiquitin ligase	Dtx3	100764385	18.42	17.41	19.51	16.48	12.90	13.62	1238.05	1172.16	1352.63	1129.95	900.44	943.08
deltex 3 like, E3 ubiquitin ligase	Dtx3l	100759272	2.08	2.69	2.49	2.48	2.62	2.86	194.44	244.31	226.06	221.35	228.73	255.16
deltex 4, E3 ubiquitin ligase	Dtx4	100755527	0.36	0.19	0.47	0.35	0.12	0.04	34.18	21.05	42.23	30.13	13.45	5.61
deoxythymidylate kinase (thymidylate kinase)	Dtymk	100755252	37.47	37.36	44.87	44.29	48.87	53.15	1381.60	1395.43	1683.03	1586.56	1822.61	1944.11
dual oxidase 1	Duox1	100774391	0.54	0.59	0.35	0.33	0.20	0.23	87.35	94.25	65.83	54.47	34.15	36.45
dual oxidase 2	Duox2	100764526	0.03	0.00	0.00	0.05	0.02	0.00	12.91	9.15	12.42	12.75	10.35	11.22
dual oxidase maturation factor 1	Duoxa1	100750838	0.04	0.00	0.03	0.00	0.03	0.00	2.28	0.00	1.24	1.16	1.03	0.00
dual oxidase maturation factor 2	Duoxa2	100751135	0.00	0.00	0.00	0.00	0.00	0.00	0.00	0.00	0.00	0.00	0.00	0.00
dual specificity phosphatase and pro isomerase domain containing 1	Dupd1	100763558	0.00	0.00	0.00	0.00	0.00	0.00	0.00	0.00	0.00	0.00	0.00	0.93
dihydrouridine synthase 1-like (S. cerevisiae)	Dus1l	100770971	26.64	28.42	12.77	12.87	7.13	9.39	1608.70	1761.44	772.58	747.50	429.52	555.19
dihydrouridine synthase 2	Dus2	100750570	16.73	14.83	17.25	17.92	17.43	17.52	974.49	895.82	1030.93	1027.96	1012.22	981.40
dihydrouridine synthase 3-like (S. cerevisiae)	Dus3l	100760481	16.52	16.70	13.77	9.75	4.88	4.62	1084.62	1086.14	901.76	665.22	359.14	342.09
dihydrouridine synthase 4-like (S. cerevisiae)	Dus4l	100754261	7.95	7.37	7.35	8.02	8.35	8.07	432.94	389.80	392.50	436.91	452.29	443.97
dual specificity phosphatase 1	Dusp1	100769474	9.84	11.01	9.99	10.65	12.79	13.99	524.84	581.96	532.86	556.28	673.78	738.39
dual specificity phosphatase 10	Dusp10	100773751	11.34	11.35	13.28	11.25	11.25	12.39	633.46	622.22	732.83	625.82	623.06	698.20
dual specificity phosphatase 11 (RNA/RNP complex 1-interacting)	Dusp11	100770939	14.94	14.69	17.08	18.92	18.20	19.08	1169.69	1199.61	1368.78	1492.69	1466.58	1527.25
dual specificity phosphatase 12	Dusp12	100775069	20.90	22.67	21.54	22.50	25.21	23.87	883.34	935.16	885.61	895.85	1030.85	978.60
	Dusp13	100760949	0.47	0.46	0.57	0.25	0.03	0.24	13.67	18.30	16.15	16.22	6.21	6.54
dual specificity phosphatase 14	Dusp14	100768086	8.16	9.09	4.27	4.72	5.84	5.71	353.95	393.46	195.01	197.02	249.43	247.69
dual specificity phosphatase 15	Dusp15	100755376	13.96	15.18	11.04	11.13	8.30	9.69	499.78	560.00	409.89	404.46	292.90	337.42

dual specificity phosphatase 16	Dusp16	100774758	25.09	24.38	27.95	28.36	24.21	23.47	3513.63	3426.80	3931.20	3976.25	3353.36	3299.38
dual specificity phosphatase 18	Dusp18	100757443	6.15	5.50	6.25	6.46	6.73	6.36	240.01	204.05	245.93	265.39	258.75	269.18
dual specificity phosphatase 19	Dusp19	100757499	17.28	17.26	14.79	14.32	16.92	16.66	458.00	447.45	399.95	370.85	448.15	437.43
dual specificity phosphatase 2	Dusp2	100757670	0.00	0.00	0.00	0.00	0.08	0.00	0.00	0.00	0.00	0.00	1.03	0.00
dual specificity phosphatase 21	Dusp21	100755361	0.00	0.00	0.00	0.00	0.00	0.00	0.00	0.00	0.00	0.00	0.00	0.00
dual specificity phosphatase 22	Dusp22	100759286	0.00	0.00	0.00	0.00	0.00	0.00	0.00	0.00	0.00	0.00	0.00	0.00
dual specificity phosphatase 23	Dusp23	100771960	0.00	0.00	0.00	0.00	0.00	0.00	0.00	0.92	0.00	0.00	0.00	0.00
dual specificity phosphatase 26 (putative)	Dusp26	100761425	0.00	0.00	0.00	0.00	0.00	0.00	0.00	0.00	0.00	0.00	0.00	0.00
dual specificity phosphatase 27 (putative)	Dusp27	100768141	0.00	0.00	0.00	0.00	0.00	0.00	0.00	0.00	0.00	0.00	0.00	0.00
dual specificity phosphatase 3	Dusp3	100761077	21.39	21.34	20.84	17.05	15.36	17.79	897.02	904.05	895.54	749.82	664.46	774.84
dual specificity phosphatase 4	Dusp4	100756233	7.50	8.02	11.60	12.93	21.61	21.74	969.17	1051.37	1533.98	1692.02	2788.25	2820.83
dual specificity phosphatase 5	Dusp5	100758694	5.09	5.20	4.18	3.90	4.68	4.92	608.39	620.39	509.26	465.89	560.96	599.12
dual specificity phosphatase 6	Dusp6	100764365	3.18	3.17	3.07	3.13	2.79	2.90	369.14	368.76	360.21	363.90	320.85	336.48
dual specificity phosphatase 7	Dusp7	100765417	8.30	8.36	7.29	7.22	5.01	5.99	777.77	786.93	685.63	666.38	466.78	565.48
dual specificity phosphatase 8	Dusp8	100768862	4.10	4.73	4.00	3.70	2.09	2.15	221.79	243.40	217.37	208.61	109.71	123.38
dual specificity phosphatase 9	Dusp9	100752633	0.00	0.00	0.00	0.00	0.00	0.00	0.00	0.00	0.00	0.00	0.00	0.00
deoxyuridine triphosphatase	Dut	100761578	31.36	35.31	36.09	36.85	45.62	44.98	825.62	915.03	935.29	935.25	1128.14	1122.54
dishevelled segment polarity protein 1	Dvl1	100770288	28.99	27.20	29.65	24.64	19.31	19.73	1996.83	1900.52	1881.76	1329.28	918.03	981.40
dishevelled segment polarity protein 2	Dvl2	100762587	8.38	7.89	8.34	8.50	6.53	6.74	334.96	312.94	317.97	332.61	269.10	268.25
dishevelled segment polarity protein 3	Dvl3	100773535	16.27	16.35	16.50	14.94	12.88	13.69	2138.86	2157.65	2229.55	2014.20	1726.36	1817.00
decapping exoribonuclease	Dxo	100768556	7.11	6.61	10.87	8.76	5.09	5.64	217.99	200.39	320.46	256.12	148.00	169.18
DPY30 domain containing 1	Dydc1	100758376	0.00	0.00	0.00	0.00	0.00	0.00	0.00	0.92	0.00	0.00	0.00	0.00
DPY30 domain containing 2	Dydc2	100759438	0.00	0.00	0.00	0.00	0.00	0.00	0.00	0.00	0.00	0.00	0.00	0.00
dymeclin	Dym	100764371	12.17	11.46	13.82	15.22	16.66	15.61	882.58	820.79	967.59	1045.35	1161.25	1092.63
dynein, cytoplasmic 1, heavy chain 1	Dync1h 1	100771366	46.25	45.05	48.58	49.83	46.63	48.17	19916.64	19344.72	21001.20	21277.77	19934.87	20706.67
dynein, cytoplasmic 1, intermediate chain 1	Dync1i1	100765135	0.07	0.00	0.00	0.00	0.02	0.00	4.56	0.92	0.00	0.00	1.03	0.00
dynein, cytoplasmic 1, intermediate chain 2	Dync1i2	100773761	47.59	49.91	51.11	59.04	63.17	60.46	3451.35	3653.73	3711.36	4174.43	4508.40	4298.55
dynein, cytoplasmic 1, light intermediate chain 1	Dync1li1	100768032	19.19	19.77	20.88	22.38	24.23	23.75	1183.36	1220.65	1264.44	1365.21	1474.86	1426.31
dynein, cytoplasmic 1, light intermediate chain 2	Dync1li2	100770140	26.48	26.51	28.85	29.65	30.00	29.32	2588.51	2626.15	2879.16	2932.07	2957.99	2926.45
dynein, cytoplasmic 2, heavy chain 1	Dync2h 1	100758806	2.15	2.22	2.34	2.95	3.02	2.94	931.20	974.51	1007.33	1277.13	1332.03	1260.87
dynein, cytoplasmic 2, light intermediate chain 1	Dync2li1	100760943	43.11	44.98	35.93	38.14	37.54	36.17	1712.76	1787.98	1413.49	1475.31	1445.88	1416.02
dynein, light chain, LC8-type 2	Dynll2	100762300	50.11	50.39	45.08	46.19	46.97	46.65	3060.18	3071.77	2731.35	2760.55	2788.25	2797.47
dynein, light chain, roadblock-type 1	Dynlrb1	100760283	95.65	99.72	90.28	94.69	96.01	94.83	1643.64	1675.43	1491.75	1530.93	1506.94	1482.39

dynein, light chain, roadblock-type 2	Dynlrb2	100766239	0.00	0.00	0.00	0.00	0.00	0.00	0.00	0.00	0.00	0.00	0.00	0.00
dynein, light chain, Tctex-type 1	Dynlt1	100761379	47.12	46.43	39.57	40.03	38.38	37.99	853.72	848.24	716.68	713.89	678.95	688.85
dynein, light chain, Tctex-type 3	Dynlt3	100761090	12.67	12.06	7.91	8.65	11.88	10.34	747.39	705.49	471.99	508.77	688.27	607.54
dual-specificity tyrosine-(Y)-phosphorylation regulated kinase 1A	Dyrk1a	100764478	15.40	15.03	16.08	17.76	17.13	17.46	2253.55	2199.74	2404.68	2615.68	2506.74	2560.06
dual-specificity tyrosine-(Y)-phosphorylation regulated kinase 1B	Dyrk1b	100762532	1.13	1.00	1.29	0.56	0.47	0.59	103.30	93.33	119.24	57.95	47.61	57.95
dual-specificity tyrosine-(Y)-phosphorylation regulated kinase 2	Dyrk2	100771845	16.93	16.66	18.11	19.42	20.94	21.20	2397.87	2397.39	2625.77	2809.22	3000.43	3062.91
dual-specificity tyrosine-(Y)-phosphorylation regulated kinase 3	Dyrk3	100761931	9.43	8.69	6.35	6.92	6.67	6.63	507.37	481.31	357.72	369.70	357.07	361.72
dual-specificity tyrosine-(Y)-phosphorylation regulated kinase 4	Dyrk4	100772985	0.00	0.02	0.00	0.02	0.00	0.00	0.00	0.92	0.00	1.16	1.03	0.93
dysferlin	Dysf	100756449	0.00	0.01	0.00	0.01	0.00	0.00	0.76	2.75	0.00	1.16	0.00	0.00
dystrotelin	Dytn	100753329	0.00	0.00	0.00	0.00	0.00	0.00	0.00	0.00	0.00	0.00	0.00	0.00
dyslexia susceptibility 1 candidate 1	Dyx1c1	100769043	0.05	0.04	0.00	0.00	0.07	0.02	5.32	4.58	1.24	2.32	4.14	1.87
double zinc ribbon and ankyrin repeat domains 1	Dzank1	100767139	0.38	0.46	0.35	0.29	0.38	0.49	37.22	40.26	34.78	28.97	38.29	43.93
DAZ interacting zinc finger protein 1	Dzip1	100766464	7.05	7.26	6.16	7.08	6.57	6.40	692.70	752.16	630.98	658.27	652.04	649.60
DAZ interacting zinc finger protein 1-like	Dzip1l	100768816	5.89	6.09	0.00	0.00	0.00	0.01	686.62	707.32	0.00	0.00	0.00	0.93
DAZ interacting zinc finger protein 3	Dzip3	100761551	7.64	8.48	6.29	7.59	8.07	8.00	1221.34	1302.09	998.64	1208.75	1265.79	1245.91
E2F transcription factor 1	E2f1	100758556	14.14	13.49	14.32	14.05	11.05	12.39	970.69	943.40	1007.33	964.22	764.85	828.12
E2F transcription factor 2	E2f2	100754522	1.79	1.60	1.93	1.69	1.55	1.42	216.47	194.90	238.48	203.97	186.30	172.91
E2F transcription factor 3	E2f3	100754647	16.29	16.31	15.79	16.45	16.77	17.68	2321.91	2321.44	2250.66	2349.13	2382.54	2517.07
E2F transcription factor 4, p107/p130-binding	E2f4	100765561	76.90	73.28	78.74	72.93	57.68	63.26	4286.84	4097.52	4349.79	4008.70	3242.61	3526.51
E2F transcription factor 6	E2f6	100767557	13.94	15.04	14.06	14.66	14.47	15.17	920.56	1028.50	1002.36	1048.82	1081.56	1117.86
E2F transcription factor 7	E2f7	100762445	9.48	9.77	8.77	9.03	9.07	9.16	1088.42	1142.88	1002.36	1054.62	1045.34	1067.39
E2F transcription factor 8	E2f8	100760871	4.89	4.79	4.99	4.81	5.75	5.08	524.08	511.50	539.07	521.51	609.61	546.78
ELL associated factor 1	Eaf1	100754458	36.46	35.49	27.99	28.56	28.07	27.76	2695.60	2671.90	2097.88	2116.19	2065.83	2146.94
ELL associated factor 2	Eaf2	100765712	0.75	0.67	0.16	0.31	0.21	0.05	16.71	14.64	2.48	5.79	4.14	0.93
E2F-associated phosphoprotein	Eapp	100763566	36.47	37.20	35.58	42.68	43.43	42.51	1222.86	1257.26	1213.52	1425.47	1463.47	1415.09
eosinophil-associated, ribonuclease A family, member 10	Ear10	100689288	0.00	0.00	0.00	0.00	0.00	0.00	0.00	0.00	0.00	0.00	0.00	0.00
eosinophil-associated, ribonuclease A family, member 11	Ear11	100689289	0.00	0.00	0.00	0.00	0.00	0.00	0.00	0.00	0.00	0.00	0.00	0.00
Ear7 pseudogene	Ear30	100689484	0.00	0.00	0.00	0.00	0.00	0.00	0.00	0.00	0.00	0.00	0.00	0.00
eosinophil-associated, ribonuclease A family, member 7	Ear7	100689291	0.00	0.00	0.00	0.00	0.00	0.00	0.00	0.00	0.00	0.00	0.00	0.00
glutamyl-tRNA synthetase 2, mitochondrial	Ears2	100765390	3.88	3.81	4.66	4.04	3.97	4.24	242.29	247.06	319.22	253.80	269.10	292.55
estrogen receptor binding site associated, antigen, 9	Ebag9	100768914	7.11	7.81	8.00	8.22	8.51	7.84	593.96	632.29	680.66	679.13	697.58	651.46
early B-cell factor 1	Ebf1	100763962	0.00	0.00	0.00	0.00	0.00	0.00	0.00	0.00	0.00	0.00	0.00	0.00
early B-cell factor 2	Ebf2	100759162	14.26	13.93	16.16	17.49	19.51	17.93	2056.83	2016.73	2333.88	2494.00	2743.75	2546.04
early B-cell factor 3	Ebf3	100767609	2.54	2.31	3.15	3.35	2.97	2.97	309.13	283.66	386.29	405.62	363.28	356.11
early B-cell factor 4	Ebf4	100750951	1.64	1.58	2.20	2.07	1.76	2.05	153.43	145.49	198.73	185.43	141.79	172.91
Epstein-Barr virus induced 3	Ebi3	100754884	0.29	0.51	0.21	0.10	0.04	0.04	9.87	19.22	11.18	5.79	1.03	0.93

endogenous Bornavirus-like nucleoprotein 2	Ebln2	103159492	0.00	0.00	0.00	0.00	0.00	0.00	0.00	0.00	0.00	0.00	0.00	0.00
EBNA1 binding protein 2	Ebna1bp2	100769390	141.11	142.25	151.47	150.64	134.86	136.51	4946.88	4991.51	5283.84	5145.60	4688.49	4721.02
emopamil binding protein (sterol isomerase)	Ebp	100765639	28.91	28.76	33.10	33.19	37.82	36.51	824.10	827.19	957.65	941.04	1069.14	1031.88
emopamil binding protein-like	Ebpl	100762126	9.03	9.16	8.78	8.97	9.06	8.04	240.77	245.23	234.75	237.58	237.01	214.04
ecdysoneless homolog (Drosophila)	Ecd	100761389	36.41	38.63	46.23	47.35	48.34	48.28	2434.32	2607.85	3118.88	3132.56	3240.54	3193.77
endothelin converting enzyme 1	Ece1	100765950	0.17	0.22	0.06	0.10	0.14	0.13	23.55	27.45	7.45	13.91	16.56	15.89
endothelin converting enzyme 2	Ece2	100771038	39.15	33.80	37.52	30.38	21.94	24.37	1146.90	1036.73	1111.67	951.47	752.44	834.66
endothelin converting enzyme-like 1	Ecel1	100763545	0.00	0.00	0.00	0.00	0.00	0.00	0.00	0.00	0.00	0.00	0.00	0.00
enoyl CoA hydratase 1, peroxisomal	Ech1	100768055	18.25	16.10	16.31	14.57	10.82	13.50	531.68	503.27	490.62	397.51	301.18	365.46
enoyl CoA hydratase domain containing 1	Echdc1	100764713	27.30	26.47	11.16	13.43	14.03	11.44	850.68	840.92	360.21	400.99	416.06	357.04
enoyl CoA hydratase domain containing 2	Echdc2	100757273	0.17	0.09	0.00	0.00	0.00	0.00	13.67	7.32	0.00	0.00	0.00	0.00
enoyl CoA hydratase domain containing 3	Echdc3	100770967	0.00	0.00	0.00	0.00	0.00	0.00	1.52	0.00	1.24	0.00	0.00	0.00
enoyl CoA hydratase, short chain, 1, mitochondrial	Echs1	100754654	22.72	23.03	29.34	25.60	25.89	27.41	748.91	772.29	991.19	892.37	904.58	965.51
enoyl-CoA delta isomerase 1	Eci1	100774222	17.04	15.96	14.82	14.45	9.72	12.24	286.35	287.32	276.99	254.96	145.93	194.41
enoyl-CoA delta isomerase 2	Eci2	100763456	25.19	24.37	23.75	26.34	29.55	28.36	1063.35	1028.50	1012.30	1084.75	1170.57	1158.99
extracellular matrix protein 1	Ecm1	100763376	6.08	5.85	4.04	3.73	4.13	3.98	362.30	345.88	233.51	216.72	241.15	227.12
extracellular matrix protein 2, female organ and adipocyte specific	Ecm2	100754893	2.29	2.13	2.10	2.31	2.03	2.14	162.54	148.24	152.78	166.88	143.86	157.96
ECSIT signalling integrator	Ecsit	100753611	81.17	78.88	82.98	76.10	55.99	64.97	3512.87	3422.23	3578.45	3193.98	2400.13	2745.12
epithelial cell transforming 2	Ect2	100754674	29.36	29.57	34.56	38.93	43.36	40.69	3492.36	3599.74	4193.29	4680.88	5172.86	4790.18
epithelial cell transforming 2 like	Ect2l	100770493	0.05	0.03	0.06	0.01	0.00	0.01	6.84	4.58	4.97	2.32	1.03	2.80
ectodysplasin A	Eda	100768945	0.00	0.00	0.00	0.00	0.00	0.00	0.00	0.00	0.00	0.00	0.00	0.00
ectodysplasin A2 receptor	Eda2r	100752833	0.04	0.04	0.00	0.00	0.00	0.00	2.28	2.75	0.00	0.00	0.00	0.00
ectodysplasin A receptor	Edar	100763209	0.00	0.00	0.00	0.00	0.00	0.00	0.00	0.00	0.00	1.16	0.00	0.00
EDAR-associated death domain	Edaradd	100765451	13.49	13.21	18.29	19.38	13.72	14.32	1626.93	1606.80	2248.18	2321.32	1596.98	1716.99
enhancer of mRNA decapping 3	Edc3	100751562	18.70	18.55	22.39	22.64	19.95	21.44	1684.66	1677.26	2051.93	2032.75	1785.35	1935.70
enhancer of mRNA decapping 4	Edc4	100752917	34.57	33.02	36.11	32.12	27.22	30.60	5096.51	4963.14	5425.44	4779.39	4131.67	4547.17
epididymal protein 3B	Eddm3b	100758879	0.00	0.00	0.00	0.00	0.00	0.00	0.00	0.00	0.00	0.00	0.00	0.93
ER degradation enhancer, mannosidase alpha-like 1	Edem1	100689456	15.12	15.40	18.52	18.99	20.93	20.74	707.13	710.98	881.88	895.85	960.47	979.53
ER degradation enhancer, mannosidase alpha-like 2	Edem2	100753801	33.80	34.38	45.30	41.60	38.32	41.11	1897.33	1958.17	2594.72	2321.32	2150.70	2274.99
ER degradation enhancer, mannosidase alpha-like 3	Edem3	100755846	34.45	35.46	26.51	28.75	31.20	30.15	6314.81	6584.58	4923.63	5331.03	5730.72	5593.06
endothelial differentiation-related factor 1	Edf1	100763269	193.86	187.63	174.01	131.79	82.07	91.40	2590.79	2550.20	2352.51	1760.40	1173.67	1253.39
EGF-like repeats and discoidin I-like domains 3	Edil3	100766118	0.04	0.00	0.00	0.03	0.00	0.02	1.52	0.00	0.00	1.16	0.00	0.93
endothelin 1	Edn1	100768493	0.01	0.03	0.00	0.02	0.00	0.00	0.76	1.83	0.00	1.16	0.00	0.00
endothelin 2	Edn2	100754793	0.07	0.00	0.00	0.00	0.00	0.00	3.04	1.83	0.00	0.00	0.00	0.00
endothelin 3	Edn3	100772506	0.00	0.00	0.00	0.00	0.00	0.00	0.00	0.00	0.00	0.00	0.00	0.00
endothelin receptor type A	Ednra	100774176	0.00	0.04	0.00	0.00	0.00	0.00	0.00	0.92	0.00	0.00	0.00	0.00

endothelin receptor type B	Ednrb	100762726	0.00	0.00	0.00	0.00	0.00	0.00	0.00	0.00	0.00	0.00	0.00	0.00
erythroid differentiation regulatory factor 1	Edrf1	100756396	5.88	6.25	7.46	8.55	8.44	8.67	805.11	873.86	1019.75	1165.87	1140.55	1185.16
early endosome antigen 1	Eea1	100758601	15.24	14.55	12.25	15.78	18.08	16.69	1396.79	1245.36	1034.66	1328.12	1437.60	1339.38
embryonic ectoderm development	Eed	100764182	24.42	26.56	24.33	25.80	28.32	28.45	1006.39	1077.91	973.80	1054.62	1138.48	1151.51
eukaryotic translation elongation factor 1 alpha 1	Eef1a1	100689276	4685.36	4733.24	4848.25	5045.58	5173.42	5435.99	221586.45	227208.32	232005.67	239840.79	248031.36	259435.33
eukaryotic translation elongation factor 1 alpha 2	Eef1a2	100760295	4.12	3.88	5.14	4.55	4.37	4.43	166.34	154.64	211.15	180.79	176.98	191.61
eukaryotic translation elongation factor 1 beta 2	Eef1b2	100753925	595.13	595.09	741.24	676.03	663.31	660.79	17228.63	17360.02	21485.62	19234.59	18916.44	18643.86
eukaryotic translation elongation factor 1 epsilon 1	Eef1e1	100689310	92.03	95.58	96.32	95.72	84.34	94.27	2066.71	2178.69	2260.60	2190.36	1988.21	2113.29
eukaryotic translation elongation factor 1 gamma	Eef1g	100753545	1029.72	1069.17	1186.52	1079.75	929.39	1017.38	42596.48	44277.56	49032.62	44222.06	38330.72	41918.07
eukaryotic translation elongation factor 2	Eef2	100689051	928.09	904.36	957.32	782.35	551.27	627.20	77966.70	76736.54	81915.63	66224.16	46672.71	52853.70
eukaryotic elongation factor-2 kinase	Eef2k	100765385	13.51	13.47	14.59	14.60	14.16	14.53	1389.96	1441.18	1526.52	1482.26	1377.57	1465.56
eukaryotic elongation factor, selenocysteine-tRNA-specific	Eefsec	100761746	14.32	13.48	19.02	18.29	14.77	14.69	786.12	727.45	1032.17	930.61	757.61	773.91
endonuclease/exonuclease/phosphatase family domain containing 1	Eepd1	100772363	3.49	3.39	1.04	0.83	0.89	0.79	428.38	411.77	125.45	103.14	107.64	95.34
EF-hand calcium binding domain 1	Efcab1	100768257	0.00	0.00	0.08	0.00	0.00	0.00	0.00	0.00	1.24	0.00	0.00	0.00
EF-hand calcium binding domain 11	Efcab11	100769192	14.68	14.42	15.13	14.04	14.98	15.30	292.42	295.56	293.13	283.94	283.59	304.70
EF-hand calcium binding domain 12	Efcab12	100772176	0.00	0.02	0.03	0.00	0.02	0.08	4.56	2.75	1.24	3.48	3.10	3.74
EF-hand calcium binding domain 13	Efcab13	100750566	0.00	0.00	0.00	0.00	0.00	0.00	0.00	0.00	0.00	0.00	0.00	0.00
EF-hand calcium binding domain 14	Efcab14	100769544	13.80	13.96	14.96	15.69	17.56	17.23	2069.74	2121.05	2265.57	2364.20	2606.10	2577.82
EF-hand calcium binding domain 2	Efcab2	100768231	1.46	1.10	0.75	0.99	0.76	0.57	27.34	26.54	17.39	16.22	13.45	14.95
EF-hand calcium binding domain 3	Efcab3	100751732	0.00	0.00	0.00	0.00	0.00	0.00	0.00	0.00	0.00	0.00	0.00	0.00
EF-hand calcium binding domain 4A	Efcab4a	100754254	1.88	2.01	2.55	2.44	2.13	2.08	306.85	323.92	382.56	332.61	249.43	271.05
EF-hand calcium binding domain 4B	Efcab4b	100763551	0.01	0.00	0.00	0.00	0.00	0.00	0.76	0.00	0.00	0.00	0.00	0.00
EF-hand calcium binding domain 5	Efcab5	100757616	0.01	0.01	0.00	0.00	0.00	0.00	3.04	1.83	0.00	1.16	0.00	0.00
EF-hand calcium binding domain 6	Efcab6	100770176	0.00	0.00	0.00	0.00	0.00	0.00	0.00	0.00	0.00	0.00	0.00	0.00
EF-hand calcium binding domain 7	Efcab7	100753483	9.86	9.46	8.79	10.31	12.41	10.47	642.57	612.16	580.05	671.01	795.90	665.48
EF-hand calcium binding domain 9	Efcab9	100772537	0.00	0.00	0.00	0.00	0.00	0.00	0.00	0.00	0.00	0.00	0.00	0.00
EGF containing fibulin-like extracellular matrix protein 1	Efemp1	100750809	43.00	42.48	31.80	36.97	16.95	17.09	2039.36	2027.71	1495.47	1674.64	782.45	783.25
EGF containing fibulin-like extracellular matrix protein 2	Efemp2	100736553	35.25	32.43	42.60	41.47	28.77	32.97	1706.68	1582.09	2066.83	1940.03	1362.04	1536.60
EF-hand domain family, member B	Efhb	100772930	0.00	0.00	0.00	0.00	0.00	0.00	0.00	0.00	0.00	0.00	0.00	0.00
EF-hand domain (C-terminal) containing 1	Efhc1	100774119	0.06	0.05	0.02	0.04	0.09	0.00	4.56	3.66	2.48	3.48	5.17	0.00
EF-hand domain (C-terminal) containing 2	Efhc2	100770173	0.02	0.03	0.00	0.04	0.00	0.00	3.04	2.75	0.00	2.32	1.03	0.00
EF-hand domain family, member D1	Efhd1	100765009	0.00	0.00	0.00	0.00	0.00	0.00	0.00	0.00	0.00	0.00	0.00	0.00
EF-hand domain family, member D2	Efhd2	100752661	89.55	90.10	81.67	73.75	62.41	66.69	4652.94	4715.17	4306.32	3844.14	3240.54	3497.53

ephrin-A1	Efna1	100758074	5.57	5.00	5.86	6.09	4.95	5.42	221.03	208.63	236.00	239.90	189.40	205.63
ephrin-A2	Efna2	100755670	1.27	1.94	1.27	0.69	1.14	0.78	56.97	85.10	55.89	30.13	49.68	35.52
ephrin-A3	Efna3	100773125	7.74	8.60	7.98	7.03	5.67	5.26	335.72	381.57	339.09	297.84	244.26	230.86
ephrin-A4	Efna4	100773413	3.23	3.23	4.77	4.68	2.87	3.63	107.85	105.23	150.29	155.30	101.43	112.16
ephrin-A5	Efna5	100756587	0.02	0.05	0.00	0.01	0.00	0.00	2.28	6.41	0.00	1.16	0.00	0.00
ephrin-B1	Efnb1	100755216	0.00	0.00	0.00	0.00	0.00	0.00	0.00	0.00	0.00	0.00	0.00	0.00
ephrin-B2	Efnb2	100753573	0.99	1.13	1.30	1.32	1.11	1.19	131.40	148.24	180.10	186.59	161.46	170.11
ephrin-B3	Efnb3	100756800	0.00	0.00	0.00	0.00	0.00	0.00	0.00	0.92	1.24	1.16	0.00	0.93
EFR3 homolog B (S. cerevisiae)	Efr3b	100770780	0.00	0.00	0.00	0.00	0.00	0.00	0.00	0.00	0.00	0.00	0.00	0.00
embryonal Fyn-associated substrate	Efs	100771792	22.82	20.81	21.87	17.94	10.12	11.63	1962.65	1798.96	1927.72	1559.91	881.81	1028.14
elongation factor Tu GTP binding domain containing 1	Eftud1	100763625	31.75	31.63	36.03	36.60	34.51	36.76	3420.96	3472.55	3924.99	3918.31	3722.85	3937.76
elongation factor Tu GTP binding domain containing 2	Eftud2	100757897	111.41	108.32	79.56	76.24	70.06	73.95	11271.56	11317.13	8199.02	7774.03	7220.06	7495.12
epidermal growth factor	Egf	100763146	0.08	0.02	0.02	0.01	0.05	0.02	14.43	6.41	3.73	6.95	7.24	6.54
EGF-like-domain, multiple 6	Egfl6	100769506	0.00	0.00	0.00	0.00	0.00	0.00	0.00	0.00	0.00	0.00	0.00	0.00
EGF-like-domain, multiple 7	Egfl7	100753992	0.03	0.00	0.00	0.00	0.00	0.00	3.04	0.92	0.00	2.32	0.00	1.87
EGF-like-domain, multiple 8	Egfl8	100766819	0.20	0.14	0.26	0.16	0.09	0.16	4.56	5.49	6.21	4.64	3.10	2.80
EGF-like, fibronectin type III and laminin G domains	Egflam	100773318	0.00	0.00	0.00	0.00	0.00	0.00	0.00	0.00	0.00	0.00	0.00	0.00
epidermal growth factor receptor	Egfr	100774580	0.00	0.01	0.00	0.00	0.00	0.00	0.00	0.92	0.00	0.00	0.00	0.00
egl-9 family hypoxia-inducible factor 1	Egln1	100772704	23.62	24.56	23.90	24.36	23.44	22.40	785.36	841.83	806.11	796.18	794.87	770.17
egl-9 family hypoxia-inducible factor 2	Egln2	100771495	3.99	4.68	4.06	2.50	1.45	1.56	173.93	203.14	192.52	115.89	72.45	71.03
early growth response 1	Egr1	100755852	11.36	13.41	11.49	10.25	11.42	10.73	821.06	971.77	840.89	746.34	819.71	776.71
early growth response 2	Egr2	100770422	0.49	0.59	0.47	0.53	0.37	0.28	33.42	39.35	31.05	34.77	24.84	20.56
early growth response 3	Egr3	100751698	0.59	0.78	0.19	0.09	0.45	0.15	17.47	25.62	6.21	2.32	12.42	3.74
EH domain binding protein 1	Ehbp1	100765232	8.98	8.46	9.75	11.04	12.14	11.41	1269.19	1218.82	1399.83	1594.67	1718.08	1600.15
EH-domain containing 1	Ehd1	100761397	40.74	39.89	38.83	32.78	31.55	32.99	3602.49	3530.20	3453.00	2908.89	2770.66	2939.54
EH-domain containing 2	Ehd2	100764187	6.09	5.19	4.79	3.41	2.94	3.31	273.43	244.31	202.46	156.45	136.62	147.68
EH-domain containing 3	Ehd3	100766335	3.71	3.50	3.22	3.16	3.30	3.80	377.49	359.61	335.36	321.02	335.34	388.82
EH-domain containing 4	Ehd4	100771177	29.80	31.15	34.19	33.53	31.60	31.03	2776.88	2891.51	3199.62	3105.91	2918.66	2890.93
ets homologous factor	Ehf	100774359	0.00	0.00	0.00	0.00	0.00	0.00	0.00	0.00	0.00	0.00	0.00	0.00
enoyl-CoA, hydratase/3-hydroxyacyl CoA dehydrogenase	Ehhadh	100754698	0.13	0.09	0.11	0.07	0.31	0.11	11.39	9.15	8.69	5.79	24.84	8.41
euchromatic histone-lysine N-methyltransferase 1	Ehmt1	100754544	12.91	12.74	14.20	13.55	12.53	12.40	2192.03	2177.78	2403.44	2292.34	2119.65	2107.68
euchromatic histone-lysine N-methyltransferase 2	Ehmt2	100769417	56.08	54.46	64.64	46.27	30.22	32.48	6794.08	6641.31	7681.07	5567.45	3712.50	3988.24
etoposide induced 2.4	Ei24	100753150	11.99	11.74	11.90	13.36	14.77	17.52	770.17	779.61	781.27	854.12	958.40	1122.54
EP300 interacting inhibitor of differentiation 1	Eid1	100760502	91.71	99.62	107.40	102.63	106.25	111.39	4387.10	4770.98	5177.02	4914.98	5057.98	5339.77
EP300 interacting inhibitor of differentiation 3	Eid3	100771887	0.97	0.95	0.72	0.94	1.08	1.03	156.47	154.64	124.21	154.14	178.02	171.98
eukaryotic translation initiation factor 1	Eif1	100773613	978.27	959.76	750.96	717.84	746.24	763.58	18021.59	17895.31	13877.84	13485.19	13935.06	14087.34
eukaryotic translation initiation factor 1A domain containing	Eif1ad	100765365	154.45	150.69	150.33	147.48	135.66	143.23	5897.06	5833.34	5779.43	5630.03	5186.32	5552.87

eukaryotic translation initiation factor 1A, X-linked	Eif1ax	100750661	126.82	133.78	117.73	118.23	115.19	112.37	6224.42	6771.25	5893.70	5806.19	5737.96	5508.94
eukaryotic translation initiation factor 1B	Eif1b	100774503	112.30	106.51	122.76	116.62	113.78	118.04	1879.10	1797.13	2002.24	1891.36	1845.38	1891.77
eukaryotic translation initiation factor 2A, 65kDa	Eif2a	100767762	41.89	43.34	42.50	47.96	48.38	42.42	2821.69	2959.22	2858.04	3176.60	3195.00	2815.23
eukaryotic translation initiation factor 2-alpha kinase 1	Eif2ak1	100764727	49.54	48.45	49.57	54.68	58.56	58.70	3834.91	3810.20	3931.20	4240.49	4573.60	4571.47
eukaryotic translation initiation factor 2-alpha kinase 2	Eif2ak2	100757909	3.28	3.79	4.29	4.85	5.26	4.86	156.47	166.54	177.62	194.70	218.38	209.37
eukaryotic translation initiation factor 2-alpha kinase 3	Eif2ak3	100765343	9.15	8.93	10.28	11.38	11.82	11.27	1143.87	1110.85	1280.59	1398.82	1468.65	1409.48
eukaryotic translation initiation factor 2 alpha kinase 4	Eif2ak4	100763206	6.68	6.26	7.77	8.05	8.25	8.40	1319.32	1298.43	1582.42	1619.01	1706.69	1674.93
eukaryotic translation initiation factor 2B, subunit 1 alpha, 26kDa	Eif2b1	100770460	37.39	37.23	44.64	45.70	48.88	48.67	1601.87	1616.86	1968.71	2035.06	2146.56	2144.13
eukaryotic translation initiation factor 2B, subunit 2 beta, 39kDa	Eif2b2	100759048	70.49	71.82	66.61	69.91	63.53	67.30	2274.82	2323.27	2158.75	2226.29	2032.71	2160.02
eukaryotic translation initiation factor 2B, subunit 3 gamma, 58kDa	Eif2b3	100753584	22.82	23.95	24.51	26.17	29.31	22.73	986.64	1053.20	1061.98	1113.72	1251.30	966.45
eukaryotic translation initiation factor 2B, subunit 4 delta, 67kDa	Eif2b4	100766948	31.91	30.14	37.34	31.76	25.47	28.56	1664.15	1623.27	1968.71	1658.41	1391.02	1491.73
eukaryotic translation initiation factor 2B, subunit 5 epsilon, 82kDa	Eif2b5	100773826	39.90	38.10	44.69	45.18	45.21	48.68	2962.20	2887.85	3382.20	3386.36	3356.46	3618.11
eukaryotic translation initiation factor 2D	Eif2d	100759038	60.49	56.97	62.79	65.45	58.03	61.14	3597.94	3446.93	3835.56	3976.25	3537.58	3641.47
eukaryotic translation initiation factor 2, subunit 1 alpha, 35kDa	Eif2s1	100761915	200.14	210.00	183.38	200.57	205.70	192.65	8144.54	8608.64	7502.20	8033.63	8031.49	7609.15
eukaryotic translation initiation factor 2, subunit 2 beta, 38kDa	Eif2s2	100750388	377.99	387.56	392.69	401.58	378.38	360.09	15025.96	15592.17	15851.51	15973.39	15160.48	14239.69
eukaryotic translation initiation factor 2, subunit 3 gamma, 52kDa	Eif2s3	100769010	106.64	115.06	108.60	117.90	125.45	120.68	8920.03	9548.37	9094.56	9797.51	10271.20	9974.79
eukaryotic translation initiation factor 3, subunit A	Eif3a	100752118	100.78	99.08	93.97	105.77	103.92	99.44	14429.73	14334.92	13731.27	15236.32	14985.57	14402.33
eukaryotic translation initiation factor 3, subunit B	Eif3b	100762979	192.49	188.59	196.01	192.55	162.88	176.81	16876.20	17018.71	17637.63	17128.84	14536.38	15484.67
eukaryotic translation initiation factor 3, subunit D	Eif3d	100769541	237.16	232.83	250.48	248.12	205.43	222.21	13538.03	13565.37	14474.04	14092.47	11642.56	12404.94
eukaryotic translation initiation factor 3, subunit E	Eif3e	100771190	110.46	110.97	134.57	140.92	133.69	130.34	4361.27	4428.76	5375.75	5474.74	5207.02	4996.74
eukaryotic translation initiation factor 3, subunit F	Eif3f	100769028	151.84	140.51	161.40	171.08	180.35	181.07	3499.96	3392.03	3839.29	3944.96	4163.75	4142.46
eukaryotic translation initiation factor 3, subunit G	Eif3g	100763004	221.23	217.68	217.67	189.62	137.23	144.41	6658.12	6560.79	6483.69	5531.52	4092.34	4321.91
eukaryotic translation initiation factor 3, subunit H	Eif3h	100751998	145.49	139.61	167.21	151.66	118.25	122.10	4640.78	4446.15	5282.60	4764.32	3775.63	3906.92
eukaryotic translation initiation factor 3, subunit I	Eif3i	100753319	516.69	496.56	554.50	555.12	519.32	530.14	16409.09	16119.23	17900.96	17440.59	16390.04	16393.17
eukaryotic translation initiation factor 3, subunit J	Eif3j	100765691	96.61	95.31	87.15	90.66	95.21	88.43	3253.11	3284.97	3005.85	3044.48	3196.04	2903.08
eukaryotic translation initiation factor 3, subunit K	Eif3k	100766600	201.56	194.48	223.07	220.21	211.00	221.64	3654.90	3578.70	4016.91	3948.44	3743.55	3865.79
eukaryotic translation initiation factor 3, subunit L	Eif3l	100753359	116.08	116.77	125.08	128.34	125.85	126.82	5942.63	5991.64	6436.49	6494.59	6329.98	6447.35

eukaryotic translation initiation factor 3, subunit M	Eif3m	100768213	281.15	280.84	281.80	298.36	301.86	284.17	9958.32	10014.13	9926.76	10264.55	10505.11	9835.53
eukaryotic translation initiation factor 4A1	Eif4a1	100762873	413.03	412.12	418.84	442.84	426.27	442.39	20329.83	20703.55	21157.71	22164.34	21208.94	21766.59
eukaryotic translation initiation factor 4A2	Eif4a2	100763323	181.44	175.42	202.47	219.54	253.14	229.53	8415.69	8367.98	9652.26	10437.23	12076.22	10838.43
eukaryotic translation initiation factor 4A3	Eif4a3	100757133	141.60	135.68	151.42	159.09	134.45	148.29	5062.33	4952.16	5499.96	5682.18	4841.67	5234.15
eukaryotic translation initiation factor 4B	Eif4b	100758911	89.80	83.47	77.26	85.10	86.86	87.87	10178.59	9589.55	8821.30	9711.75	9846.86	9932.73
eukaryotic translation initiation factor 4E	Eif4e	100773768	178.08	180.26	149.85	157.64	163.61	163.19	5599.32	5760.14	4759.68	5016.96	5153.20	5164.98
eukaryotic translation initiation factor 4E family member 1B	Eif4e1b	100766778	0.00	0.00	0.00	0.00	0.04	0.00	0.76	0.00	1.24	1.16	4.14	0.00
eukaryotic translation initiation factor 4E family member 2	Eif4e2	100764716	96.34	92.03	101.02	95.85	94.73	95.75	2341.66	2218.04	2463.06	2268.01	2229.36	2257.23
eukaryotic translation initiation factor 4E family member 3	Eif4e3	100759947	17.43	17.89	19.21	20.29	25.24	22.85	751.18	818.96	971.31	978.13	1191.27	1126.28
eukaryotic translation initiation factor 4E binding protein 1	Eif4ebp 1	100769654	379.59	366.17	380.25	343.77	295.16	316.52	12024.27	11662.10	12112.83	10820.83	9317.98	9985.08
eukaryotic translation initiation factor 4E binding protein 2	Eif4ebp 2	100768613	35.78	36.29	51.82	51.46	53.49	56.47	5882.63	5985.23	8591.51	8487.93	8752.88	9334.55
eukaryotic translation initiation factor 4E binding protein 3	Eif4ebp 3	100752954	2.23	2.00	2.20	2.35	2.02	1.86	129.12	111.63	134.15	151.82	126.27	129.92
eukaryotic translation initiation factor 4E nuclear import factor 1	Eif4enif 1	100762835	23.34	23.65	25.37	25.29	23.84	24.07	2391.03	2429.41	2598.45	2584.39	2415.66	2460.99
eukaryotic translation initiation factor 4 gamma, 1	Eif4g1	100770567	182.96	182.24	164.43	151.44	130.46	139.52	30674.75	30904.34	28083.58	25485.81	21733.68	23129.34
eukaryotic translation initiation factor 4 gamma, 2	Eif4g2	100758677	455.64	473.31	450.79	495.99	512.27	499.21	48932.55	51227.24	48698.50	52944.10	54590.35	53325.71
eukaryotic translation initiation factor 4 gamma, 3	Eif4g3	100765660	21.86	21.76	23.82	26.33	23.83	24.55	3591.86	3622.62	3953.56	4257.87	3889.48	3960.20
eukaryotic translation initiation factor 4H	Eif4h	100770352	112.16	107.49	103.65	93.35	78.06	83.82	7447.28	7231.51	6992.95	6152.71	5128.36	5482.77
eukaryotic translation initiation factor 5	Eif5	100756911	111.81	116.62	106.20	119.51	123.38	117.70	8815.21	9236.35	8550.53	9622.51	9870.66	9517.74
eukaryotic translation initiation factor 5A	Eif5a	100766364	1035.08	987.41	1046.42	947.22	770.43	914.70	33640.75	32437.94	34014.55	30294.17	24936.96	29327.13
eukaryotic translation initiation factor 5A2	Eif5a2	100775097	19.94	20.02	13.52	15.96	19.40	17.85	2861.94	2871.38	1961.26	2295.82	2762.38	2571.28
eukaryotic translation initiation factor 5B	Eif5b	100758365	33.59	30.94	28.20	34.68	32.84	30.02	4513.94	4343.66	3941.14	4752.73	4467.00	4048.99
eukaryotic translation initiation factor 6	Eif6	100689224	322.65	312.45	279.40	240.07	188.28	221.98	10284.92	10097.39	9333.04	7891.09	6183.01	7191.35
elaC ribonuclease Z 1	Elac1	100771025	1.60	1.70	1.46	1.71	2.20	2.12	155.71	165.62	137.87	170.36	217.35	201.89
elaC ribonuclease Z 2	Elac2	100772420	15.79	14.55	13.83	14.11	12.93	14.28	1600.35	1516.21	1463.18	1471.83	1341.34	1454.35
elastase, neutrophil expressed	Elane	100752732	0.00	0.00	0.00	0.00	0.00	0.00	0.00	0.00	0.00	0.00	0.00	0.00
ELAV like RNA binding protein 1	Elavl1	100754131	26.07	26.23	28.45	28.17	27.96	30.03	4295.95	4340.00	4732.35	4655.38	4590.16	4946.27
ELAV like neuron-specific RNA binding protein 2	Elavl2	100771799	6.06	5.70	4.01	4.97	5.53	5.14	695.74	662.48	473.24	572.51	637.55	601.93
ELAV like neuron-specific RNA binding protein 3	Elavl3	100768802	0.03	0.00	0.05	0.00	0.04	0.00	1.52	0.00	2.48	0.00	1.03	0.93
ELAV like neuron-specific RNA binding protein 4	Elavl4	100773765	0.00	0.00	0.00	0.00	0.00	0.00	0.00	0.00	0.00	0.00	0.00	0.00

E74-like factor 1 (ets domain transcription factor)	Elf1	100751907	19.86	20.29	19.44	23.11	22.46	20.41	1279.06	1326.80	1290.53	1499.64	1440.70	1355.27
E74-like factor 2 (ets domain transcription factor)	Elf2	100762906	26.82	27.90	31.97	32.76	33.03	33.02	2348.50	2425.75	2772.34	2855.58	2878.30	2857.29
E74-like factor 3 (ets domain transcription factor, epithelial-specific)	Elf3	100754961	0.03	0.03	0.04	0.00	0.00	0.00	0.76	2.75	1.24	0.00	0.00	2.80
E74-like factor 4 (ets domain transcription factor)	Elf4	100771659	17.22	17.45	19.03	20.38	18.07	17.89	3025.24	3059.87	3361.09	3577.59	3141.18	3165.73
E74-like factor 5 (ets domain transcription factor)	Elf5	100774069	0.00	0.00	0.00	0.00	0.00	0.00	0.00	0.00	0.00	0.00	0.00	0.00
extracellular leucine-rich repeat and fibronectin type III domain containing 1	Elfn1	100770773	0.05	0.07	0.00	0.00	0.01	0.00	3.80	5.49	0.00	1.16	1.03	0.00
extracellular leucine-rich repeat and fibronectin type III domain containing 2	Elfn2	100766646	0.00	0.01	0.00	0.00	0.00	0.00	0.00	0.92	0.00	0.00	0.00	0.00
ELK1, member of ETS oncogene family	Elk1	100763890	18.32	17.28	19.50	17.39	15.91	15.38	1393.75	1315.82	1483.05	1325.80	1198.51	1189.83
ELK3, ETS-domain protein (SRF accessory protein 2)	Elk3	100689475	95.65	93.38	88.34	86.44	79.58	80.42	5188.41	5095.82	4798.18	4638.00	4265.18	4360.23
ELK4, ETS-domain protein (SRF accessory protein 1)	Elk4	100755459	6.38	6.78	6.85	7.83	7.83	7.78	459.52	479.48	498.08	563.24	571.31	564.54
elongation factor RNA polymerase II	Ell	100767546	6.29	5.65	4.92	3.86	3.08	3.35	651.68	615.82	529.13	414.89	338.44	358.91
elongation factor, RNA polymerase II, 2	Ell2	100762315	8.68	9.51	8.43	8.30	9.46	8.94	947.90	1043.14	921.63	907.43	1018.43	983.27
elongation factor RNA polymerase II-like 3	Ell3	100754376	1.05	0.96	0.88	1.11	1.27	0.83	65.32	60.39	48.44	59.10	69.34	59.82
engulfment and cell motility 1	Elmo1	100759062	4.97	4.81	5.31	5.49	6.12	6.54	467.88	459.35	494.35	515.72	581.66	611.27
engulfment and cell motility 2	Elmo2	100769563	14.74	15.72	15.86	16.24	19.28	20.20	1513.76	1610.46	1658.19	1676.96	1984.07	2057.21
engulfment and cell motility 3	Elmo3	100765847	0.36	0.39	0.71	0.76	0.63	0.61	53.17	64.05	111.79	106.62	96.25	90.66
ELMO/CED-12 domain containing 1	Elmod1	100769312	0.00	0.00	0.00	0.00	0.00	0.00	0.00	0.00	0.00	0.00	0.00	0.00
ELMO/CED-12 domain containing 2	Elmod2	100773262	5.53	5.88	4.96	6.16	7.31	6.29	648.65	687.19	594.96	705.78	825.92	729.04
ELMO/CED-12 domain containing 3	Elmod3	100775058	11.57	12.37	14.04	14.82	15.07	16.44	741.31	798.82	914.18	950.31	960.47	1035.61
ELM2 and Myb/SANT-like domain containing 1	Elmsan 1	100774891	8.20	7.85	8.18	6.86	6.01	5.92	1592.75	1548.24	1623.41	1368.68	1194.37	1217.87
elongation factor 1 homolog (S. cerevisiae)	Elof1	100753010	75.04	69.56	85.45	85.81	71.18	77.51	1782.64	1674.51	2017.15	1985.23	1680.82	1828.21
ELOVL fatty acid elongase 1	Elovl1	100767465	39.22	36.22	41.70	43.12	42.39	41.04	1364.89	1226.14	1414.74	1459.08	1445.88	1401.07
ELOVL fatty acid elongase 2	Elovl2	100764278	0.00	0.00	0.00	0.00	0.00	0.00	0.00	0.00	0.00	0.00	0.00	0.00
ELOVL fatty acid elongase 3	Elovl3	100774317	0.00	0.00	0.00	0.00	0.00	0.00	0.00	0.00	0.00	0.00	0.00	0.00
ELOVL fatty acid elongase 4	Elovl4	100774858	5.04	5.41	1.16	1.12	1.07	1.29	259.76	273.60	59.62	59.10	57.96	66.36
ELOVL fatty acid elongase 5	Elovl5	100753555	92.98	94.75	95.91	98.39	109.77	111.45	4188.86	4290.59	4424.31	4446.78	5033.14	5080.86
ELOVL fatty acid elongase 7	Elovl7	100754940	5.87	6.05	8.30	8.56	10.23	10.42	571.17	576.47	801.15	813.56	969.78	1001.97
elongator acetyltransferase complex subunit 2	Elp2	100764459	57.30	59.89	61.30	62.85	63.77	65.45	5325.89	5621.05	5718.57	5796.92	5874.58	6045.44
elongator acetyltransferase complex subunit 3	Elp3	100771722	66.76	67.97	67.60	65.48	63.51	62.51	5518.05	5603.67	5580.70	5350.73	5202.88	5150.03
elongator acetyltransferase complex subunit 4	Elp4	100766660	11.59	10.96	11.98	12.16	13.07	12.70	790.68	756.73	838.41	818.20	906.65	897.28

elongator acetyltransferase complex subunit 5	Elp5	100761708	51.57	52.47	60.43	62.51	60.99	61.19	2021.89	2109.15	2413.37	2449.96	2391.85	2391.82
elongator acetyltransferase complex subunit 6	Elp6	100764169	25.74	23.26	24.39	22.10	15.56	18.03	998.03	904.05	936.53	832.10	602.36	690.72
EGF, latrophilin and seven transmembrane domain containing 1	Eltd1	100756599	0.00	0.02	0.00	0.00	0.00	0.00	0.00	1.83	0.00	0.00	0.00	0.00
embigin	Emb	100752805	0.00	0.00	0.00	0.00	0.00	0.00	0.00	0.00	0.00	0.00	0.00	0.00
ER membrane protein complex subunit 1	Emc1	100764102	103.83	101.35	119.92	122.54	110.56	116.40	7462.47	7454.78	8943.03	8832.13	8213.65	8616.72
ER membrane protein complex subunit 10	Emc10	100758503	80.10	73.07	78.43	57.97	42.91	49.19	3925.30	3640.00	3956.05	2864.85	2197.28	2435.75
ER membrane protein complex subunit 2	Emc2	100769760	29.35	28.27	29.44	33.20	30.47	29.76	1212.22	1203.27	1259.48	1377.96	1309.26	1231.89
ER membrane protein complex subunit 3	Emc3	100757477	48.13	47.92	48.62	49.32	51.12	53.02	2665.22	2656.34	2728.86	2753.59	2836.90	2943.27
ER membrane protein complex subunit 4	Emc4	100772555	85.25	83.82	91.21	92.47	95.07	99.71	2305.20	2272.03	2460.57	2449.96	2535.72	2636.70
ER membrane protein complex subunit 7	Emc7	100773137	84.62	83.26	78.01	82.69	87.91	86.91	2365.96	2338.83	2192.28	2277.28	2437.39	2390.89
ER membrane protein complex subunit 8	Emc8	100770955	19.33	20.28	22.89	24.90	21.00	23.10	716.25	733.86	858.28	954.95	780.38	878.59
ER membrane protein complex subunit 9	Emc9	100761448	6.66	5.80	7.26	5.22	3.98	3.89	160.26	140.00	177.62	118.21	96.25	94.40
emerin	Emd	100760344	50.87	49.50	63.62	58.97	64.68	64.23	1681.62	1624.18	2092.92	1922.65	2101.02	2091.79
essential meiotic structure-specific endonuclease 1	Eme1	100759039	4.22	4.62	2.10	2.60	2.96	3.36	228.62	264.44	105.58	142.55	149.04	159.83
essential meiotic structure-specific endonuclease subunit 2	Eme2	100765424	2.10	2.08	2.13	1.10	0.95	1.07	113.93	117.12	141.60	92.71	78.66	86.92
EMG1 N1-specific pseudouridine methyltransferase	Emg1	100759485	54.39	51.98	54.05	49.84	42.89	46.66	1768.97	1723.92	1756.31	1667.69	1457.26	1571.18
EMI domain containing 1	Emid1	100751943	0.00	0.00	0.00	0.00	0.00	0.00	0.00	0.00	0.00	0.00	0.00	0.00
elastin microfibril interfacer 1	Emilin1	100763067	5.70	5.49	4.57	2.64	1.68	1.67	645.61	615.82	526.64	305.95	187.33	194.41
elastin microfibril interfacer 2	Emilin2	100770473	0.24	0.19	0.14	0.22	0.17	0.22	49.37	38.43	29.81	41.72	34.15	40.19
elastin microfibril interfacer 3	Emilin3	100771706	0.07	0.06	0.04	0.06	0.03	0.05	10.63	7.32	6.21	10.43	4.14	5.61
echinoderm microtubule associated protein like 1	Eml1	100770154	0.03	0.03	0.00	0.00	0.01	0.00	3.04	2.75	0.00	0.00	1.03	0.00
echinoderm microtubule associated protein like 2	Eml2	100756651	63.69	60.68	63.81	54.11	40.80	44.20	3619.20	3487.19	3661.67	3046.80	2290.42	2463.79
echinoderm microtubule associated protein like 3	Eml3	100755884	27.25	27.82	37.58	33.13	31.21	31.93	2466.22	2476.08	3371.02	2958.72	2771.69	2845.13
echinoderm microtubule associated protein like 4	Eml4	100764725	12.12	11.84	11.26	12.15	11.44	11.03	2101.65	2055.17	1973.68	2108.08	1986.14	1926.35
echinoderm microtubule associated protein like 5	Eml5	100771482	1.04	1.06	0.97	1.11	1.27	1.25	238.50	248.89	228.54	257.28	296.01	284.14
echinoderm microtubule associated protein like 6	Eml6	100763779	2.60	2.30	3.03	3.10	3.78	3.71	584.09	527.06	685.63	696.51	842.48	839.33
epithelial membrane protein 1	Emp1	100753041	162.96	174.01	116.86	120.22	129.19	127.54	2735.86	2944.58	1961.26	1974.80	2130.00	2055.34
epithelial membrane protein 2	Emp2	100767430	11.49	11.95	11.22	11.93	12.89	14.00	985.12	1017.52	967.59	1016.37	1089.84	1189.83
epithelial membrane protein 3	Emp3	100751496	47.99	46.56	35.40	32.77	26.77	30.81	799.79	810.72	634.71	589.89	526.81	553.32
egf-like module containing, mucin-like, hormone receptor-like 1	Emr1	100751559	0.00	0.00	0.00	0.00	0.00	0.00	0.76	0.00	0.00	0.00	0.00	0.00
empty spiracles homeobox 1	Emx1	100768849	0.00	0.00	0.00	0.00	0.00	0.00	0.00	0.00	0.00	0.00	0.00	0.93
empty spiracles homeobox 2	Emx2	100750961	0.00	0.00	0.00	0.00	0.00	0.00	0.00	0.00	0.00	0.00	0.00	0.00

engrailed homeobox 1	En1	103163841	0.00	0.00	0.00	0.00	0.00	0.00	0.00	0.00	0.00	0.00	0.00	0.00
engrailed homeobox 2	En2	100773986	0.00	0.00	0.00	0.00	0.00	0.00	0.00	0.00	0.00	0.00	0.00	0.00
enabled homolog (Drosophila)	Enah	100752970	27.36	27.63	23.84	25.53	26.15	25.28	4767.63	4878.04	4224.34	4425.92	4472.18	4302.28
enamelin	Enam	100773739	0.00	0.00	0.00	0.00	0.00	0.00	0.00	0.00	0.00	0.00	0.00	0.00
ectodermal-neural cortex 1 (with BTB domain)	Enc1	100751664	8.25	8.43	6.93	7.77	10.18	9.87	1058.04	1080.65	896.79	1000.15	1302.01	1275.82
endonuclease domain containing 1	Endod1	100763760	20.32	20.56	23.06	26.07	33.46	32.44	2270.26	2308.63	2598.45	2923.95	3714.57	3648.02
endonuclease G	Endog	100770499	11.44	9.33	11.42	11.27	10.18	11.61	34.94	30.20	34.78	23.18	23.80	18.69
endonuclease, polyU-specific	Endou	100753999	0.05	0.00	0.05	0.12	0.13	0.18	2.28	0.00	2.48	5.79	7.24	6.54
endonuclease V	Endov	100760952	7.07	6.12	5.58	4.26	2.52	3.30	262.80	242.48	211.15	161.09	99.36	116.83
endoglin	Eng	100757433	199.48	188.69	177.78	151.00	94.38	105.46	17605.36	16662.76	15762.08	13263.84	8302.66	9319.59
endo-beta-N-acetylglucosaminidase	Engase	100754028	6.04	5.57	5.19	4.62	3.37	3.30	728.40	681.70	628.50	555.12	399.50	400.04
energy homeostasis associated	Enho	100771493	1.74	1.78	0.87	0.82	0.29	0.53	28.86	31.11	13.66	12.75	5.17	8.41
enkurin domain containing 1	Enkd1	100764090	3.22	3.94	4.33	3.44	3.05	3.35	132.16	147.32	170.17	132.12	110.74	117.77
enkurin, TRPC channel interacting protein	Enkur	100757646	0.06	0.13	0.09	0.04	0.00	0.03	1.52	4.58	2.48	1.16	0.00	0.93
enolase 1, (alpha)	Eno1	100752856	552.82	559.80	658.62	629.41	463.03	516.63	21393.94	21659.76	25420.55	23834.35	17670.32	19749.57
enolase 2 (gamma, neuronal)	Eno2	100758414	2.50	2.28	3.13	3.15	3.25	3.56	262.80	237.91	327.91	333.77	358.11	372.93
enolase 3 (beta, muscle)	Eno3	100771169	1.59	1.57	1.36	1.28	1.04	1.26	96.46	98.82	84.46	81.12	67.27	77.58
enolase family member 4	Eno4	100773987	0.00	0.00	0.00	0.00	0.00	0.00	0.00	0.00	0.00	1.16	0.00	0.00
enolase-phosphatase 1	Enoph1	100767312	43.22	43.02	42.21	38.06	31.48	36.01	1495.53	1490.59	1459.45	1332.76	1152.97	1274.89
enolase superfamily member 1	Enosf1	100755139	0.55	0.58	0.57	0.66	0.84	0.88	51.65	47.58	48.44	61.42	78.66	80.38
ecto-NOX disulfide-thiol exchanger 1	Enox1	100770334	21.50	22.49	25.32	26.84	26.94	26.16	1961.89	2037.78	2282.96	2352.61	2466.37	2346.96
ecto-NOX disulfide-thiol exchanger 2	Enox2	100751206	5.51	5.52	4.67	5.38	4.90	5.13	517.25	531.63	455.85	515.72	481.27	500.05
ectonucleotide pyrophosphatase/phosphodiesterase 1	Enpp1	100750518	3.20	3.35	0.99	1.24	0.36	0.40	360.78	369.67	111.79	136.75	42.43	42.99
ectonucleotide pyrophosphatase/phosphodiesterase 2	Enpp2	100755955	0.00	0.01	0.00	0.00	0.01	0.00	0.00	0.92	0.00	0.00	1.03	0.00
ectonucleotide pyrophosphatase/phosphodiesterase 3	Enpp3	100751104	0.04	0.04	0.00	0.00	0.00	0.00	1.52	1.83	0.00	0.00	0.00	0.00
ectonucleotide pyrophosphatase/phosphodiesterase 4 (putative)	Enpp4	100763274	0.00	0.00	0.00	0.00	0.00	0.00	0.00	0.00	0.00	0.00	0.00	0.00
ectonucleotide pyrophosphatase/phosphodiesterase 5 (putative)	Enpp5	100757812	0.02	0.00	0.00	0.00	0.00	0.00	0.76	0.00	0.00	0.00	0.00	0.00
ectonucleotide pyrophosphatase/phosphodiesterase 6	Enpp6	100757074	0.00	0.00	0.00	0.00	0.00	0.00	0.00	0.00	0.00	0.00	0.00	0.00
ectonucleotide pyrophosphatase/phosphodiesterase 7	Enpp7	100755684	0.00	0.00	0.00	0.00	0.00	0.00	0.00	0.00	0.00	0.00	0.00	0.00
endosulfine alpha	Ensa	100764533	67.84	67.67	68.28	68.82	67.83	71.24	4078.73	4083.79	4167.20	4196.45	4089.23	4368.65
ENTH domain containing 1	Enthd1	100769307	0.07	0.13	0.00	0.00	0.00	0.00	3.04	5.49	0.00	0.00	0.00	0.00
ENTH domain containing 2	Enthd2	100762986	9.53	8.35	11.10	7.58	5.34	6.24	689.66	645.10	792.45	578.30	403.64	456.12

ectonucleoside triphosphate diphosphohydrolase 1	Entpd1	100762593	0.00	0.00	0.00	0.00	0.00	0.03	0.00	0.92	0.00	1.16	0.00	0.93
ectonucleoside triphosphate diphosphohydrolase 2	Entpd2	100771642	0.00	0.02	0.03	0.00	0.00	0.02	0.00	0.92	1.24	0.00	0.00	0.93
ectonucleoside triphosphate diphosphohydrolase 3	Entpd3	100772668	0.02	0.00	0.00	0.00	0.00	0.00	1.52	0.00	0.00	0.00	0.00	0.00
ectonucleoside triphosphate diphosphohydrolase 4	Entpd4	100771637	13.70	13.44	10.96	11.43	12.82	13.00	1399.07	1400.00	1170.05	1194.85	1326.85	1352.47
ectonucleoside triphosphate diphosphohydrolase 5	Entpd5	100751454	5.07	4.97	5.54	6.01	7.31	6.18	277.99	288.24	330.40	332.61	409.85	353.31
ectonucleoside triphosphate diphosphohydrolase 6 (putative)	Entpd6	100765414	0.00	0.00	0.00	0.00	0.00	0.00	0.00	0.00	0.00	0.00	0.00	0.00
ectonucleoside triphosphate diphosphohydrolase 7	Entpd7	100751972	6.27	5.97	6.14	6.09	5.54	6.00	587.12	564.58	594.96	573.67	524.74	564.54
ectonucleoside triphosphate diphosphohydrolase 8	Entpd8	100755122	0.00	0.00	0.00	0.00	0.00	0.00	0.00	0.00	0.00	0.00	1.03	0.00
enhancer of yellow 2 homolog (Drosophila)	Eny2	100768337	65.52	54.00	68.17	84.92	72.94	63.56	738.27	675.29	783.76	887.73	768.99	690.72
EGF domain-specific O-linked N-acetylglucosamine (GlcNAc) transferase	Eogt	100757071	2.90	3.44	3.44	4.04	4.09	3.89	332.68	351.37	363.93	416.05	478.16	457.05
eomesodermin	Eomes	100753878	0.00	0.00	0.00	0.00	0.00	0.00	0.00	0.00	0.00	0.00	0.00	0.00
E1A binding protein p300	Ep300	100762260	12.15	10.93	10.98	11.61	11.18	11.03	3144.49	2850.33	2865.49	3050.28	2911.42	2876.91
E1A binding protein p400	Ep400	100770341	13.35	12.89	10.93	11.19	11.00	10.59	4437.23	4292.42	3644.28	3683.05	3612.10	3506.88
endothelial PAS domain protein 1	Epas1	100771160	1.88	1.82	0.05	0.02	0.03	0.06	254.45	243.40	6.21	2.32	5.17	8.41
erythrocyte membrane protein band 4.1 (elliptocytosis 1, RH-linked)	Epb41	100767741	7.45	7.29	8.42	8.63	8.55	8.92	1133.99	1101.70	1294.25	1309.58	1265.79	1351.53
erythrocyte membrane protein band 4.1-like 1	Epb41l1	100751805	9.54	9.87	6.76	6.64	6.16	5.95	935.75	987.32	671.97	654.79	597.19	582.30
erythrocyte membrane protein band 4.1-like 2	Epb41l2	100756569	34.33	34.68	23.24	25.48	21.14	19.86	3581.23	3672.94	2474.24	2651.61	2205.56	2084.31
erythrocyte membrane protein band 4.1-like 3	Epb41l3	100751173	0.00	0.00	0.00	0.00	0.00	0.00	0.00	0.00	0.00	0.00	0.00	0.00
erythrocyte membrane protein band 4.1 like 4A	Epb41l4a	100754107	0.14	0.12	0.07	0.07	0.13	0.08	26.58	25.62	17.39	25.50	27.94	12.15
erythrocyte membrane protein band 4.1 like 4B	Epb41l4b	100764940	0.00	0.00	0.00	0.00	0.00	0.00	0.00	0.00	0.00	0.00	0.00	0.00
erythrocyte membrane protein band 4.1 like 5	Epb41l5	100757297	10.77	11.39	15.00	15.29	17.08	16.71	1639.85	1709.28	2161.23	2264.53	2599.89	2577.82
erythrocyte membrane protein band 4.2	Epb42	100768600	0.01	0.00	0.00	0.00	0.00	0.00	3.04	0.00	0.00	1.16	0.00	0.00
enhancer of polycomb homolog 1 (Drosophila)	Epc1	100773878	13.67	13.85	15.23	15.52	15.09	14.18	1543.38	1549.15	1727.74	1722.15	1675.64	1582.40
enhancer of polycomb homolog 2 (Drosophila)	Epc2	100773541	15.03	14.84	13.73	14.83	15.63	14.58	1527.43	1518.04	1388.65	1526.30	1585.60	1459.02
epithelial cell adhesion molecule	Epcam	100770236	0.02	0.00	1.00	1.48	0.95	1.06	2.28	0.92	31.05	49.83	35.19	40.19
ependymin related 1	Epdr1	100770772	67.46	67.52	68.67	74.46	72.16	70.25	4364.31	4400.40	4434.25	4685.51	4495.98	4423.79
ectopic P-granules autophagy protein 5 homolog (C. elegans)	Epg5	100770998	3.19	3.11	3.01	3.47	3.72	3.96	917.52	897.65	876.91	995.51	1063.97	1138.43
epithelial mitogen	Epgn	100771437	0.00	0.00	0.00	0.20	0.18	0.00	0.00	0.00	1.24	1.16	2.07	0.00
EPH receptor A1	Epha1	100752024	0.00	0.00	0.00	0.00	0.00	0.00	2.28	0.00	1.24	0.00	0.00	0.93
EPH receptor A10	Epha10	100752731	0.00	0.00	0.00	0.00	0.00	0.00	0.00	0.00	0.00	0.00	0.00	0.00

EPH receptor A2	Epha2	100752851	13.50	13.85	8.43	7.97	6.67	6.41	1606.43	1672.68	1016.03	949.16	792.80	773.91
EPH receptor A3	Epha3	100768107	0.00	0.00	0.00	0.00	0.00	0.00	0.00	0.00	0.00	0.00	0.00	0.00
EPH receptor A4	Epha4	100754542	0.00	0.00	0.00	0.00	0.00	0.00	0.00	0.00	0.00	0.00	0.00	0.00
EPH receptor A5	Epha5	100770099	0.02	0.01	0.00	0.00	0.00	0.00	1.52	1.83	0.00	0.00	0.00	0.00
EPH receptor A7	Epha7	100750730	0.00	0.00	0.00	0.00	0.01	0.00	0.00	0.00	0.00	0.00	1.03	0.00
EPH receptor A8	Epha8	100769287	0.00	0.00	0.00	0.00	0.00	0.00	0.00	0.00	0.00	0.00	0.00	0.00
EPH receptor B1	Ephb1	100769764	0.00	0.00	0.00	0.00	0.00	0.00	0.00	0.00	0.00	0.00	0.00	0.00
EPH receptor B2	Ephb2	100766993	0.00	0.01	0.00	0.00	0.00	0.00	0.00	0.92	0.00	0.00	0.00	0.00
EPH receptor B3	Ephb3	100774807	0.68	0.46	0.35	0.27	0.28	0.33	74.43	49.41	40.99	30.13	28.98	42.06
EPH receptor B4	Ephb4	100769535	1.07	0.98	1.44	1.17	0.90	1.04	113.17	118.95	167.68	142.55	102.46	113.10
EPH receptor B6	Ephb6	100762813	0.00	0.00	0.00	0.00	0.00	0.00	0.00	0.00	0.00	0.00	0.00	0.00
epoxide hydrolase 1, microsomal (xenobiotic)	Ephx1	100750792	13.29	13.32	18.92	12.80	10.78	11.08	34.18	32.94	31.05	24.34	23.80	24.30
epoxide hydrolase 2, cytoplasmic	Ephx2	100757328	0.00	0.02	0.00	0.00	0.00	0.00	0.00	0.92	0.00	0.00	0.00	0.00
epoxide hydrolase 3	Ephx3	100753937	0.00	0.00	0.00	0.00	0.00	0.00	0.76	0.00	0.00	0.00	0.00	0.00
epoxide hydrolase 4	Ephx4	100761937	0.04	0.07	0.09	0.10	0.09	0.19	3.80	5.49	8.69	11.59	10.35	19.63
epilepsy, progressive myoclonus type 2A, Lafora disease (laforin)	Epm2a	100766433	0.59	0.49	0.57	0.77	0.97	0.47	16.71	11.90	16.15	19.70	22.77	16.82
EPM2A (laforin) interacting protein 1	Epm2aip1	100770168	3.72	3.60	3.59	3.76	4.73	4.06	420.03	410.85	413.61	440.39	549.58	465.47
epsin 1	Epn1	100769501	4.79	4.26	3.79	1.93	1.34	1.16	208.87	215.95	182.59	104.30	90.04	85.99
epsin 2	Epn2	100774824	6.80	6.77	6.90	6.37	6.19	6.61	634.22	643.27	637.19	594.53	604.43	624.36
epsin 3	Epn3	100762219	0.00	0.00	0.00	0.00	0.00	0.00	0.00	0.92	0.00	0.00	0.00	0.00
erythropoietin	Epo	100753960	0.00	0.10	0.00	0.00	0.00	0.00	0.00	3.66	1.24	1.16	0.00	0.00
erythropoietin receptor	Epor	100769376	0.06	0.06	0.00	0.00	0.02	0.00	3.04	2.75	0.00	1.16	1.03	0.00
epiplakin 1	Eppk1	100757722	0.01	0.01	0.01	0.00	0.00	0.01	0.00	0.00	0.00	0.00	0.00	0.00
glutamyl-prolyl-tRNA synthetase	Eprs	100774338	111.25	112.40	117.95	128.42	129.64	128.57	16250.34	16471.52	17225.26	18507.95	18687.71	18603.67
epidermal growth factor receptor pathway substrate 15	Eps15	100751122	25.11	26.34	24.47	27.00	30.97	31.04	2948.53	3132.16	2916.42	3193.98	3635.91	3652.69
epidermal growth factor receptor pathway substrate 15-like 1	Eps15l1	100755944	19.83	19.92	17.83	17.70	14.80	14.92	1681.62	1681.83	1517.83	1403.45	1162.29	1205.72
epidermal growth factor receptor pathway substrate 8	Eps8	100750968	63.97	64.73	44.85	45.30	47.63	47.17	7250.56	7236.09	5191.92	5204.71	5350.88	5321.07
EPS8-like 2	Eps8l2	100751674	0.71	0.58	0.57	0.44	0.61	0.76	66.84	53.99	52.17	41.72	62.10	69.17
EPS8-like 3	Eps8l3	100753693	0.02	0.00	0.00	0.00	0.00	0.02	2.28	2.75	0.00	0.00	1.03	3.74
ethanolaminephosphotransferase 1 (CDP-ethanolamine-specific)	Ept1	100759003	11.77	11.70	12.33	13.63	14.29	15.16	2245.20	2218.96	2378.60	2585.55	2693.03	2882.52
eosinophil peroxidase	Epx	100751071	0.00	0.00	0.00	0.00	0.00	0.00	1.52	0.00	0.00	0.00	1.03	0.00
epiphycan	Epyc	100754407	0.10	0.09	0.00	0.04	0.00	0.00	3.80	2.75	0.00	1.16	0.00	0.93
equatorin, sperm acrosome associated	Eqtn	100766597	0.00	0.00	0.00	0.08	0.00	0.00	0.00	0.00	0.00	4.64	0.00	0.00
Era-like 12S mitochondrial rRNA chaperone 1	Eral1	100766924	13.60	14.63	13.90	14.13	13.71	14.84	780.05	815.29	827.23	847.17	820.74	899.15
endoplasmic reticulum aminopeptidase 1	Erap1	100756235	18.98	19.62	23.62	24.42	27.02	25.70	2155.57	2242.75	2692.84	2729.26	3052.17	2898.41
ES cell expressed Ras	Eras	100763405	0.00	0.00	0.00	0.08	0.00	0.00	0.00	0.00	0.00	1.16	0.00	0.00
v-erb-b2 avian erythroblastic leukemia viral oncogene homolog 2	Erbb2	100759983	10.90	10.22	7.85	7.73	5.41	5.41	1442.36	1356.08	1035.90	1017.53	724.49	723.43
erbb2 interacting protein	Erbb2ip	100774374	20.38	20.52	19.90	23.99	25.31	23.60	3519.70	3531.11	3461.70	4173.27	4383.17	4140.59

v-erb-b2 avian erythroblastic leukemia viral oncogene homolog 3	Erbb3	100767681	0.02	0.00	0.01	0.01	0.02	0.02	3.80	1.83	1.24	1.16	5.17	3.74
ELKS/RAB6-interacting/CAST family member 1	Erc1	100753317	12.33	11.91	12.66	14.08	14.25	14.29	2931.82	2852.16	3082.86	3380.57	3386.48	3405.94
ELKS/RAB6-interacting/CAST family member 2	Erc2	100770769	0.00	0.00	0.00	0.00	0.00	0.00	0.00	0.00	0.00	0.00	0.00	0.00
excision repair cross-complementation group 1	Ercc1	100689377	17.61	17.51	13.37	7.84	6.67	6.73	355.46	316.60	284.44	215.56	219.42	224.32
excision repair cross-complementation group 2	Ercc2	100689272	4.39	4.24	5.90	4.65	3.23	3.41	404.83	398.04	514.22	413.73	268.06	295.36
excision repair cross-complementation group 3	Ercc3	100689367	16.36	16.74	18.33	15.90	18.32	18.67	1157.54	1152.03	1281.83	1107.93	1222.32	1256.20
excision repair cross-complementation group 4	Ercc4	100689044	5.58	5.75	5.95	5.95	6.63	6.46	477.75	487.71	489.38	502.97	549.58	541.17
excision repair cross-complementation group 5	Ercc5	100764306	24.88	25.89	28.45	30.50	33.26	30.57	2549.77	2649.02	2910.21	3085.04	3335.76	3114.32
excision repair cross-complementation group 6-like	Ercc6l	100757441	10.65	10.74	12.00	13.30	14.78	14.49	1233.49	1254.51	1401.07	1540.20	1699.45	1686.14
excision repair cross-complementation group 8	Ercc8	100755229	18.49	19.25	20.97	23.02	23.14	23.30	1127.92	1194.12	1330.28	1405.77	1422.07	1416.96
epiregulin	Ereg	100771728	0.03	0.02	0.00	0.01	0.00	0.00	3.80	1.83	0.00	1.16	0.00	0.00
v-ets avian erythroblastosis virus E26 oncogene homolog	Erg	100764178	0.00	0.00	0.00	0.00	0.00	0.00	0.00	0.00	0.00	0.00	0.00	0.00
endoplasmic reticulum-golgi intermediate compartment (ERGIC) 1	Ergic1	100774570	54.27	54.85	64.27	68.38	64.34	67.26	4324.06	4363.79	5124.85	5361.16	5046.59	5316.40
ERGIC and golgi 2	Ergic2	100763592	60.73	60.76	49.38	57.45	62.24	59.91	623.58	631.37	505.53	585.25	671.71	612.21
ERGIC and golgi 3	Ergic3	100755369	89.01	88.01	120.22	110.63	108.73	122.35	3404.25	3344.45	4439.22	4051.58	4059.22	4527.54
enhancer of rudimentary homolog (Drosophila)	Erh	100758434	214.16	209.98	210.63	202.83	209.44	215.63	4156.96	4073.73	4041.75	3845.30	4006.43	4107.87
exoribonuclease 1	Eri1	100773583	17.11	18.00	20.85	21.87	23.86	23.34	1795.55	1896.86	2208.43	2295.82	2476.72	2450.70
ERI1 exoribonuclease family member 2	Eri2	100751601	5.53	5.59	5.95	6.63	7.40	7.40	498.26	529.80	544.03	629.29	687.23	679.50
ERI1 exoribonuclease family member 3	Eri3	100756117	41.23	40.98	41.91	40.39	35.76	37.35	1481.10	1418.30	1435.85	1348.98	1213.00	1269.28
glutamate-rich 1	Erich1	100758343	19.58	21.49	18.48	19.53	18.70	20.69	755.74	829.02	703.02	767.20	733.81	797.27
glutamate-rich 2	Erich2	100773190	0.00	0.00	0.00	0.00	0.00	0.00	0.00	0.00	0.00	0.00	0.00	0.00
glutamate-rich 3	Erich3	100770258	0.00	0.00	0.00	0.00	0.00	0.00	0.00	0.00	0.00	0.00	0.00	0.00
glutamate-rich 4	Erich4	100774810	0.00	0.00	0.00	0.00	0.00	0.00	0.00	0.00	0.00	0.00	0.00	0.00
glutamate-rich 6	Erich6	100772843	0.14	0.14	0.11	0.10	0.12	0.08	9.11	7.32	8.69	5.79	7.24	6.54
endoplasmic reticulum lectin 1	Erlec1	100767274	36.80	37.51	36.02	39.59	38.66	36.88	2557.37	2681.05	2586.02	2813.86	2772.73	2586.23
ER lipid raft associated 1	Erlin1	100754719	57.13	54.00	65.98	68.33	74.03	74.58	4848.90	4621.83	5668.88	5751.72	6198.53	6257.61
ER lipid raft associated 2	Erlin2	100770510	17.12	17.84	16.26	17.38	22.96	21.14	1973.28	2069.81	1890.46	1984.07	2627.83	2430.14
erythroblast membrane-associated protein (Scianna blood group)	Ermap	100753808	0.06	0.02	0.05	0.03	0.01	0.05	5.32	3.66	4.97	2.32	2.07	3.74
ER membrane-associated RNA degradation	Ermard	100752150	2.73	2.76	2.26	2.85	2.97	3.13	327.36	322.09	260.84	321.02	347.76	367.33
ermin, ERM-like protein	Ermn	100759143	0.04	0.00	0.00	0.00	0.00	0.00	0.76	0.00	0.00	0.00	0.00	0.00
endoplasmic reticulum metallopeptidase 1	Ermp1	100760334	26.36	27.28	25.50	29.10	33.44	31.94	3659.46	3791.90	3582.18	4033.04	4623.28	4409.77
endoplasmic reticulum to nucleus signaling 1	Ern1	100750680	15.00	14.48	18.81	19.10	17.70	17.78	3001.70	2922.62	3813.21	3827.91	3561.39	3584.46

endoplasmic reticulum to nucleus signaling 2	Ern2	100761252	0.00	0.00	0.00	0.03	0.00	0.00	1.52	0.92	1.24	2.32	1.03	0.93
ERO1-like (S. cerevisiae)	Ero1l	100763138	24.84	26.35	27.92	28.92	32.12	32.37	2801.94	2938.17	3148.69	3198.62	3577.95	3615.30
ERO1-like beta (S. cerevisiae)	Ero1lb	100764579	2.99	3.24	3.33	3.80	4.55	4.54	389.64	418.17	437.21	497.18	572.35	586.97
endoplasmic reticulum protein 27	Erp27	100754205	0.37	0.29	0.11	0.10	0.03	0.05	18.23	10.98	3.73	4.64	3.10	1.87
endoplasmic reticulum protein 29	Erp29	100762501	48.28	49.20	56.14	48.92	42.86	43.46	1430.21	1458.56	1678.06	1437.06	1257.51	1274.89
endoplasmic reticulum protein 44	Erp44	100750874	44.75	46.46	50.00	55.45	58.96	54.20	1850.24	1946.28	2038.26	2222.81	2373.22	2174.98
ERBB receptor feedback inhibitor 1	Errfi1	100758904	41.15	39.29	37.56	41.58	53.90	52.83	3484.01	3335.30	3212.04	3528.91	4536.34	4487.35
endothelial cell adhesion molecule	Esam	100769354	0.03	0.00	0.05	0.07	0.06	0.02	1.52	0.92	2.48	3.48	3.10	1.87
establishment of sister chromatid cohesion N-acetyltransferase 1	Esco1	100764656	8.80	8.38	8.18	9.63	11.98	10.09	1039.81	1012.94	950.20	1129.95	1381.71	1145.91
establishment of sister chromatid cohesion N-acetyltransferase 2	Esco2	100755867	9.63	9.22	10.09	12.69	14.82	12.70	982.08	946.14	1078.13	1304.94	1523.50	1343.12
esterase D	Esd	100774799	71.66	73.87	64.16	68.20	72.21	70.39	2163.93	2216.21	1938.90	1973.64	2141.39	2061.88
ESF1, nucleolar pre-rRNA processing protein, homolog (S. cerevisiae)	Esf1	100771608	64.21	60.68	58.55	69.07	65.16	64.32	3079.93	2918.04	2834.44	3290.17	3122.55	3090.95
endothelial cell-specific molecule 1	Esm1	100773837	0.00	0.00	0.00	0.00	0.00	0.00	0.00	0.00	0.00	0.00	0.00	0.00
extra spindle pole bodies homolog 1 (S. cerevisiae)	Espl1	100768842	13.07	12.47	12.53	12.40	10.85	11.32	2656.11	2541.05	2576.09	2513.70	2230.40	2317.05
espin	Espn	100755239	0.07	0.07	0.00	0.00	0.00	0.00	7.60	6.41	0.00	0.00	1.03	0.00
espin-like	Espnl	100773927	0.01	0.00	0.00	0.00	0.00	0.00	0.76	0.00	0.00	0.00	1.03	0.00
estrogen receptor 1	Esr1	100768474	1.41	1.33	1.25	1.35	1.48	1.52	236.22	223.27	207.43	223.67	245.29	250.49
estrogen receptor 2 (ER beta)	Esr2	100763291	0.79	0.79	0.86	0.74	0.62	0.78	68.36	72.29	80.74	69.54	58.99	71.97
epithelial splicing regulatory protein 1	Esrp1	100759516	0.02	0.02	0.00	0.00	0.00	0.00	0.76	0.92	1.24	0.00	1.03	0.93
epithelial splicing regulatory protein 2	Esrp2	100755569	0.13	0.21	0.06	0.05	0.14	0.01	15.19	16.47	11.18	8.11	12.42	5.61
estrogen-related receptor alpha	Esrra	100761304	21.05	18.77	20.53	13.13	7.85	9.79	1228.17	1104.45	1212.28	782.27	469.88	582.30
estrogen-related receptor beta	Esrrb	100760486	0.02	0.08	0.04	0.00	0.00	0.00	3.04	2.75	1.24	0.00	0.00	0.00
estrogen-related receptor gamma	Esrrg	100760533	0.00	0.00	0.00	0.00	0.00	0.00	0.00	0.00	0.00	0.00	0.00	0.00
extended synaptotagmin-like protein 1	Esyt1	100767105	30.32	30.09	35.24	32.69	31.28	35.23	3474.13	3528.37	4167.20	3848.77	3644.19	4074.23
extended synaptotagmin-like protein 2	Esyt2	100761977	37.56	37.21	39.57	40.21	45.41	45.18	5808.20	5791.25	6158.27	6230.35	6978.91	6948.33
extended synaptotagmin-like protein 3	Esyt3	100771388	0.00	0.00	0.00	0.00	0.00	0.00	1.52	0.00	0.00	2.32	1.03	0.00
Ewing tumor-associated antigen 1	Etaa1	100750682	13.66	13.04	16.70	19.36	21.10	19.23	1175.01	1130.98	1445.79	1666.53	1808.12	1669.32
eukaryotic translation termination factor 1	Etf1	100755265	94.25	97.56	93.78	100.80	99.65	98.92	9321.07	9672.82	9313.17	9914.56	9750.61	9749.54
electron-transfer-flavoprotein, alpha polypeptide	Etfa	100758144	110.45	112.19	115.64	125.15	134.46	133.58	4229.11	4429.68	4506.29	4727.23	5114.90	5022.91
electron-transfer-flavoprotein, beta polypeptide	Etfb	100753078	0.00	0.00	0.00	0.00	0.00	0.00	0.00	0.00	0.00	0.00	0.00	0.00
electron-transferring-flavoprotein dehydrogenase	Etfdh	100763190	25.84	26.76	27.74	30.26	36.08	33.52	1677.06	1762.35	1834.56	1997.98	2371.15	2145.07
ethylmalonic encephalopathy 1	Ethe1	100769786	47.11	41.23	72.10	65.08	47.34	57.08	540.79	460.26	787.48	673.33	509.21	612.21
ethanolamine kinase 1	Etnk1	100765795	20.56	20.36	20.77	22.40	23.62	22.92	3644.27	3613.47	3712.60	3983.21	4166.85	4088.25
ethanolamine kinase 2	Etnk2	100766811	0.00	0.00	0.00	0.00	0.00	0.00	0.00	0.00	0.00	0.00	0.00	0.00
ethanolamine-phosphate phospho-lyase	Etnppl	100753688	0.00	0.00	0.00	0.00	0.00	0.00	0.00	0.00	0.00	0.00	0.00	0.00
v-ets avian erythroblastosis virus E26 oncogene homolog 1	Ets1	100767456	103.82	106.12	89.45	94.73	96.12	95.24	8973.96	9064.32	7797.82	8504.15	9664.70	9627.10

v-ets avian erythroblastosis virus E26 oncogene homolog 2	Ets2	100774407	23.90	22.87	34.02	32.26	28.90	29.61	2334.82	2243.66	3342.46	3129.08	2797.57	2882.52
ets variant 1	Etv1	100758498	4.79	5.42	3.76	4.09	5.43	5.65	454.20	517.91	360.21	389.40	515.42	527.15
ets variant 3	Etv3	100750397	6.15	5.60	5.31	5.67	5.35	5.30	382.05	359.61	340.33	347.68	329.13	326.20
ets variant 3-like	Etv3l	100760586	0.15	0.23	0.24	0.13	0.03	0.14	20.51	40.26	22.36	22.02	11.38	15.89
ets variant 4	Etv4	100774750	163.42	156.64	168.02	162.16	144.04	149.02	5869.72	5559.74	5988.10	5766.79	5154.23	5398.65
ets variant 5	Etv5	100756646	9.85	9.37	7.48	7.97	7.52	7.60	1099.81	1055.95	842.13	895.85	836.27	844.94
ets variant 6	Etv6	100771873	4.11	3.93	4.75	4.79	5.25	5.17	530.92	511.50	613.59	624.66	694.48	688.85
eva-1 homolog A (C. elegans)	Eva1a	100765979	4.61	5.08	3.90	4.23	3.13	2.90	221.79	245.23	191.28	197.02	143.86	138.33
eva-1 homolog B (C. elegans)	Eva1b	100759036	4.20	4.56	5.20	2.56	1.18	1.42	115.45	123.53	142.84	68.38	27.94	36.45
eva-1 homolog C (C. elegans)	Eva1c	100767132	0.00	0.00	0.00	0.00	0.00	0.00	0.00	0.00	0.00	0.00	0.00	0.00
Ellis van Creveld syndrome	Evc	100771932	7.23	7.47	9.95	9.78	9.33	9.53	951.70	986.41	1294.25	1277.13	1227.49	1248.72
Ellis van Creveld syndrome 2	Evc2	100751511	6.17	6.54	5.77	5.51	4.15	4.17	745.87	764.97	706.75	649.00	509.21	533.70
ecotropic viral integration site 2A	Evi2a	100761225	0.00	0.00	0.00	0.00	0.00	0.00	0.00	0.00	0.00	0.00	0.00	0.00
ecotropic viral integration site 2B	Evi2b	100760937	0.00	0.00	0.00	0.00	0.00	0.00	0.00	0.00	0.00	0.00	0.00	0.00
ecotropic viral integration site 5	Evi5	100769964	5.89	6.05	5.96	7.54	7.77	7.33	1045.13	1076.08	1058.26	1317.69	1356.87	1297.32
ecotropic viral integration site 5-like	Evi5l	100755001	8.47	7.53	9.09	8.74	7.66	7.82	868.15	877.52	1009.82	903.96	806.25	803.82
Enah/Vasp-like	Evl	100771012	15.99	15.31	16.61	13.85	12.74	13.00	791.44	784.18	848.35	696.51	633.41	653.33
envoplakin	Evpl	100760021	0.04	0.04	0.11	0.04	0.05	0.01	9.11	7.32	19.87	6.95	8.28	1.87
even-skipped homeobox 1	Evx1	100763300	0.00	0.00	0.00	0.00	0.00	0.00	0.00	0.00	0.00	0.00	0.00	0.00
even-skipped homeobox 2	Evx2	100759401	0.00	0.00	0.00	0.00	0.00	0.00	0.00	0.00	0.00	0.00	0.00	0.00
EWS RNA-binding protein 1	Ewsr1	100773033	217.41	218.49	203.12	185.38	183.69	187.04	16020.96	16162.24	15124.89	13740.15	13838.80	14028.46
exonuclease 3'-5' domain containing 1	Exd1	100764230	3.10	3.34	3.04	3.44	4.29	4.20	309.13	330.33	303.07	345.36	454.36	430.88
exonuclease 3'-5' domain containing 2	Exd2	100765115	32.10	31.96	19.36	18.62	18.78	20.16	2674.34	2715.82	1642.04	1563.38	1579.39	1688.01
exonuclease 1	Exo1	100772338	13.36	13.23	16.62	16.45	17.87	17.18	1322.36	1324.05	1655.70	1686.23	1779.14	1704.84
exonuclease 5	Exo5	100753327	7.48	7.75	7.74	9.48	8.45	7.56	392.68	407.19	411.13	500.65	444.01	398.17
exocyst complex component 1	Exoc1	100770306	23.62	24.30	15.47	17.25	18.49	18.40	2282.42	2394.64	1535.22	1697.82	1790.53	1763.72
exocyst complex component 2	Exoc2	100755817	40.64	42.44	40.79	43.70	47.38	46.27	4921.81	5202.88	5060.26	5357.69	5856.99	5632.32
exocyst complex component 3	Exoc3	100756215	21.58	21.77	19.38	18.65	20.77	21.97	1583.64	1603.14	1458.21	1387.23	1592.84	1660.91
exocyst complex component 3-like 2	Exoc3l2	100754318	0.00	0.00	0.00	0.00	0.00	0.00	0.00	0.92	0.00	0.00	1.03	0.00
exocyst complex component 3-like 4	Exoc3l4	100756620	0.87	1.01	0.57	0.59	0.39	0.52	197.48	217.78	136.63	143.71	98.32	128.05
exocyst complex component 4	Exoc4	100750685	16.62	17.77	13.50	14.29	15.48	16.90	2159.37	2303.14	1719.05	1795.17	2003.73	2167.50
exocyst complex component 5	Exoc5	100769231	15.33	15.42	16.40	19.33	21.18	18.48	1968.73	2002.09	2131.42	2481.25	2706.49	2364.71
exocyst complex component 6	Exoc6	100751436	12.42	11.73	7.50	7.61	8.59	7.96	1000.31	952.55	592.48	596.84	658.25	630.90
exocyst complex component 6B	Exoc6b	100757038	10.24	10.41	10.95	12.62	14.13	14.08	1564.65	1576.60	1681.79	1907.58	2099.99	2107.68
exocyst complex component 7	Exoc7	100754030	13.65	13.13	15.63	16.08	13.62	15.08	1699.09	1663.53	1998.52	2013.04	1679.78	1874.01
exocyst complex component 8	Exoc8	100764733	6.85	7.01	8.68	7.66	8.45	8.91	766.38	783.27	982.49	865.71	957.36	1007.57
endo/exonuclease (5'-3'), endonuclease G-like	Exog	100750872	5.36	4.83	6.43	6.26	6.46	6.62	647.13	599.35	791.21	757.93	782.45	799.14
exosome component 1	Exosc1	100757484	40.58	40.00	49.54	52.12	49.87	48.80	1204.63	1229.81	1456.97	1519.34	1469.68	1435.65
exosome component 10	Exosc10	100754929	28.84	29.79	36.40	39.18	34.63	37.12	2551.29	2634.38	3197.13	3382.89	3018.02	3207.79
exosome component 2	Exosc2	100761222	48.12	48.35	47.87	49.97	45.44	47.32	2113.80	2132.03	2106.58	2149.80	2001.66	2065.62

exosome component 3	Exosc3	100753640	21.71	18.47	17.06	18.89	21.08	21.57	363.06	323.01	293.13	307.11	342.58	343.02
exosome component 4	Exosc4	100766618	35.50	32.01	40.82	33.78	20.51	26.84	756.50	703.66	898.03	717.37	463.67	575.76
exosome component 5	Exosc5	100773940	13.43	12.29	12.35	6.17	5.21	4.99	277.99	279.09	294.37	148.34	132.48	104.68
exosome component 7	Exosc7	100763217	38.87	36.98	39.11	36.73	33.27	32.92	1156.78	1098.04	1085.58	1058.09	921.14	946.82
exosome component 8	Exosc8	100769802	66.19	65.73	73.17	78.97	82.14	81.47	1693.01	1690.07	1892.94	1971.32	2075.15	2038.51
exosome component 9	Exosc9	100759081	24.97	25.81	21.33	23.49	24.21	23.15	956.26	970.85	824.75	924.82	933.56	899.15
exophilin 5	Exph5	100753332	1.78	1.73	1.90	2.13	2.00	1.95	369.14	346.80	385.05	431.12	405.71	401.91
exostosin glycosyltransferase 1	Ext1	100689334	21.83	19.76	23.65	25.92	19.85	20.62	1567.69	1462.22	1751.34	1872.81	1455.19	1505.75
exostosin glycosyltransferase 2	Ext2	100751585	34.79	34.23	42.83	43.94	37.58	42.97	2861.94	2853.07	3566.03	3598.45	3109.10	3549.88
exostosin-like glycosyltransferase 1	Extl1	100770000	6.46	5.76	5.34	3.90	1.78	2.00	625.86	560.92	514.22	368.54	172.84	189.74
exostosin-like glycosyltransferase 2	Extl2	100761218	27.16	28.03	32.43	32.43	38.06	38.19	2271.02	2349.81	2691.60	2663.20	3100.82	3144.23
exostosin-like glycosyltransferase 3	Extl3	100751999	13.92	13.24	12.89	13.39	12.54	12.89	2533.06	2422.09	2362.45	2452.27	2256.27	2359.11
eyes absent homolog 1 (Drosophila)	Eya1	100772860	0.04	0.20	0.00	0.00	0.00	0.00	4.56	17.39	0.00	0.00	0.00	0.00
eyes absent homolog 2 (Drosophila)	Eya2	100768991	0.00	0.00	0.00	0.00	0.00	0.00	0.00	0.00	0.00	0.00	0.00	0.00
eyes absent homolog 3 (Drosophila)	Eya3	100772330	16.84	17.02	18.51	19.85	21.13	21.22	2140.38	2178.69	2364.93	2531.08	2668.19	2690.91
eyes absent homolog 4 (Drosophila)	Eya4	100754333	0.00	0.00	0.00	0.00	0.00	0.00	0.00	0.00	0.00	0.00	0.00	0.00
enhancer of zeste homolog 1 (Drosophila)	Ezh1	100760595	12.48	12.24	9.14	9.17	9.72	10.19	1607.94	1626.93	1222.21	1213.39	1277.17	1344.99
enhancer of zeste homolog 2 (Drosophila)	Ezh2	100758055	19.32	18.09	20.06	21.88	22.48	23.40	1459.07	1422.88	1578.69	1678.12	1766.72	1739.42
ezrin	Ezr	100762541	88.18	94.77	58.98	58.68	55.83	57.99	6750.78	7233.34	4517.47	4406.21	4194.80	4326.59
coagulation factor X	F10	100758763	0.00	0.00	0.00	0.00	0.00	0.00	0.00	0.00	0.00	0.00	0.00	0.00
coagulation factor XI	F11	100768163	0.00	0.00	0.00	0.00	0.00	0.00	0.76	0.00	0.00	0.00	0.00	0.00
F11 receptor	F11r	100764378	0.00	0.00	0.00	0.06	0.00	0.00	0.00	0.00	0.00	1.16	0.00	0.00
coagulation factor XII (Hageman factor)	F12	100764143	0.00	0.00	0.00	0.00	0.00	0.02	0.76	0.00	0.00	0.00	0.00	1.87
coagulation factor XIII, A1 polypeptide	F13a1	100761709	0.00	0.00	0.00	0.00	0.00	0.00	0.00	0.00	0.00	0.00	0.00	0.00
coagulation factor XIII, B polypeptide	F13b	100764268	0.01	0.00	0.00	0.00	0.00	0.00	0.76	0.00	0.00	0.00	0.00	0.00
coagulation factor II (thrombin)	F2	100754947	0.00	0.00	0.00	0.00	0.02	0.00	1.52	0.00	0.00	1.16	2.07	0.00
coagulation factor II (thrombin) receptor	F2r	100758351	56.03	54.69	93.47	101.95	88.77	91.46	3933.65	3887.98	6694.85	7306.99	6395.18	6539.88
coagulation factor II (thrombin) receptor-like 1	F2rl1	100757860	1.44	1.69	1.53	1.71	1.84	1.67	110.89	126.27	116.76	125.16	137.65	129.92
coagulation factor II (thrombin) receptor-like 2	F2rl2	100758642	0.72	0.67	0.35	0.41	0.80	0.69	64.56	58.56	31.05	38.24	72.45	65.43
coagulation factor II (thrombin) receptor-like 3	F2rl3	100757293	0.00	0.00	0.00	0.00	0.00	0.00	0.76	0.00	0.00	0.00	0.00	0.93
coagulation factor III (thromboplastin, tissue factor)	F3	100765608	0.89	0.92	0.09	0.11	0.08	0.02	41.02	40.26	3.73	6.95	3.10	0.93
coagulation factor V (proaccelerin, labile factor)	F5	100761588	0.00	0.01	0.00	0.00	0.00	0.00	0.00	0.92	0.00	0.00	0.00	0.00
coagulation factor VII (serum prothrombin conversion accelerator)	F7	100759052	0.05	0.00	0.03	0.02	0.00	0.02	2.28	0.00	1.24	1.16	0.00	0.93
coagulation factor VIII, procoagulant component	F8	100756560	0.45	0.46	0.39	0.44	0.51	0.51	136.72	139.09	119.24	132.12	150.07	151.42
coagulation factor IX	F9	100757213	0.00	0.00	0.00	0.00	0.00	0.00	0.00	0.00	0.00	0.00	0.00	0.00
fatty acid 2-hydroxylase	Fa2h	100771615	0.01	0.01	0.61	0.28	0.00	0.01	0.76	0.92	45.96	20.86	0.00	0.93
fatty acid amide hydrolase	Faah	100771171	7.62	7.72	8.54	7.36	5.68	7.02	557.50	584.71	632.22	565.55	426.41	516.87
fatty acid binding protein 1, liver	Fabp1	100769104	0.00	0.00	0.00	0.00	0.00	0.00	0.00	0.00	0.00	0.00	0.00	0.00

fatty acid binding protein 12	Fabp12	100761683	0.00	0.00	0.00	0.00	0.00	0.00	0.00	0.00	0.00	0.00	0.00	0.00
fatty acid binding protein 3, muscle and heart (mammary-derived growth inhibitor)	Fabp3	100761139	1.50	1.10	1.87	1.41	1.62	1.42	48.61	32.03	54.65	45.20	48.64	49.54
fatty acid binding protein 4, adipocyte	Fabp4	100760812	0.10	0.44	0.00	0.00	0.00	0.00	1.52	8.24	0.00	0.00	0.00	0.00
fatty acid binding protein 5 (psoriasis-associated)	Fabp5	100762553	0.00	0.00	0.00	0.00	0.00	0.00	0.00	0.00	0.00	0.00	0.00	0.00
fatty acid binding protein 6, ileal	Fabp6	100767370	0.00	0.00	0.00	0.00	0.00	0.00	0.00	0.00	0.00	0.00	0.00	0.00
fatty acid binding protein 7, brain	Fabp7	100752143	0.00	0.00	0.00	0.00	0.00	0.00	0.00	0.00	0.00	0.00	0.00	0.00
fatty acid binding protein 9, testis	Fabp9	100761969	0.00	0.00	0.00	0.00	0.00	0.00	0.00	0.00	0.00	0.00	0.00	0.00
Fas (TNFRSF6)-associated via death domain	Fadd	100774599	5.09	5.16	5.76	5.87	5.67	6.11	326.60	334.90	360.21	381.28	380.87	401.91
fatty acid desaturase 1	Fads1	100773095	3.35	3.61	3.63	3.98	3.60	3.96	139.76	154.64	141.60	159.93	152.14	172.91
fatty acid desaturase 2	Fads2	100757464	0.00	0.00	0.00	0.00	0.00	0.01	0.00	0.00	0.00	0.00	0.00	0.93
fatty acid desaturase 3	Fads3	100757171	19.74	19.13	15.54	14.64	12.26	13.07	1023.86	994.64	797.42	749.82	634.45	672.96
fatty acid desaturase 6	Fads6	100772984	0.00	0.00	0.00	0.00	0.00	0.00	0.00	0.00	0.00	0.00	0.00	0.00
Fas (TNFRSF6) associated factor 1	Faf1	100774441	29.51	28.14	34.01	35.35	35.42	36.28	3616.93	3516.47	4256.63	4341.31	4350.05	4338.74
Fas associated factor family member 2	Faf2	100767643	22.13	22.29	21.39	23.71	22.73	24.18	2182.16	2233.60	2171.17	2351.45	2244.89	2413.32
fumarylacetoacetate hydrolase (fumarylacetoacetase)	Fah	100757944	11.53	10.68	11.70	12.16	10.26	10.33	285.59	279.09	301.83	304.80	232.87	277.60
fumarylacetoacetate hydrolase domain containing 1	Fahd1	100766004	19.79	18.65	18.99	14.87	14.27	11.73	546.11	515.16	582.54	455.46	464.71	421.54
fumarylacetoacetate hydrolase domain containing 2A	Fahd2a	100774837	14.96	12.61	8.80	8.14	7.06	8.11	562.82	478.56	352.75	319.86	291.87	325.27
Fas apoptotic inhibitory molecule 2	Faim2	100756789	0.00	0.00	0.00	0.00	0.00	0.00	0.76	0.00	0.00	0.00	0.00	0.00
Fas apoptotic inhibitory molecule 3	Faim3	100770414	0.11	0.04	0.03	0.00	0.00	0.04	6.08	2.75	2.48	1.16	0.00	1.87
family with sequence similarity 101, member A	Fam101a	100767489	0.00	0.00	0.00	0.00	0.00	0.00	0.00	0.92	0.00	1.16	0.00	0.00
family with sequence similarity 101, member B	Fam101b	100763414	18.08	16.22	15.20	16.37	14.78	15.78	567.38	518.82	521.68	538.90	477.13	526.22
family with sequence similarity 102, member A	Fam102a	100756559	4.49	4.53	5.19	4.61	4.82	4.62	501.30	500.52	575.09	513.40	522.67	515.00
family with sequence similarity 102, member B	Fam102b	100754924	16.03	16.51	21.24	23.95	25.60	23.75	2428.25	2513.60	3271.66	3649.44	3866.71	3607.83
family with sequence similarity 103, member A1	Fam103a1	100770192	33.18	34.57	30.74	32.59	33.26	32.72	1296.53	1346.01	1201.10	1263.22	1287.52	1272.09
family with sequence similarity 104, member A	Fam104a	100762576	23.41	24.27	25.28	24.30	23.79	22.12	818.78	799.74	943.99	909.75	892.16	821.57
family with sequence similarity 105, member A	Fam105a	100772539	0.00	0.00	0.00	0.00	0.00	0.00	0.00	0.00	0.00	0.00	0.00	0.00
family with sequence similarity 107, member A	Fam107a	100763708	0.00	0.00	0.00	0.00	0.00	0.00	0.00	0.00	0.00	0.00	0.00	0.00
family with sequence similarity 107, member B	Fam107b	100751328	77.53	82.64	82.72	89.91	106.89	104.21	6538.11	6991.77	7063.75	7624.53	8966.09	8815.80
family with sequence similarity 109, member A	Fam109a	100773389	8.68	7.67	8.43	5.00	4.08	4.35	538.51	488.63	557.70	377.81	324.99	341.15
family with sequence similarity 109, member B	Fam109b	100751381	0.08	0.00	0.06	0.06	0.00	0.00	1.52	2.75	1.24	1.16	0.00	1.87
family with sequence similarity 110, member A	Fam110a	100760972	10.77	9.68	8.29	6.08	3.17	4.60	465.60	417.26	362.69	270.03	148.00	205.63

family with sequence similarity 110, member B	Fam110b	100754309	3.50	3.88	6.92	6.56	5.05	5.81	287.87	323.01	586.26	548.17	415.03	478.55
family with sequence similarity 110, member C	Fam110c	100773311	0.03	0.15	0.00	0.05	0.00	0.07	0.76	3.66	0.00	1.16	0.00	1.87
family with sequence similarity 110, member D	Fam110d	100771715	0.22	0.26	0.00	0.08	0.00	0.00	6.08	4.58	2.48	1.16	0.00	0.00
family with sequence similarity 114, member A1	Fam114a1	100762494	10.40	10.94	11.02	11.87	11.89	11.42	861.32	903.14	914.18	982.76	974.96	954.30
family with sequence similarity 114, member A2	Fam114a2	100768819	13.94	14.34	14.07	14.46	14.69	15.50	1045.89	1087.06	1057.02	1088.23	1082.60	1140.30
family with sequence similarity 115, member A	Fam115a	100753518	5.35	5.18	5.48	5.71	5.03	5.61	821.82	807.97	863.25	887.73	778.31	873.92
family with sequence similarity 117, member A	Fam117a	100761276	6.15	5.84	3.19	2.81	2.56	3.16	366.10	348.63	188.80	170.36	154.21	183.20
family with sequence similarity 117, member B	Fam117b	100767817	7.36	7.22	7.23	8.03	8.97	8.36	1012.47	1001.05	1007.33	1107.93	1217.14	1148.71
family with sequence similarity 118, member A	Fam118a	100764016	7.29	7.87	7.23	7.34	8.60	7.92	412.43	432.81	428.52	434.59	496.79	462.66
family with sequence similarity 118, member B	Fam118b	100761453	14.22	15.37	13.95	16.66	15.29	16.80	645.61	697.26	635.95	754.46	694.48	743.06
family with sequence similarity 120A	Fam120a	100759484	103.30	104.43	115.10	110.86	150.83	150.34	13921.59	14267.20	15717.37	14933.84	20144.97	20146.81
family with sequence similarity 120B	Fam120b	100750992	20.16	19.62	18.27	18.61	19.49	19.99	2109.24	2053.34	1961.26	1981.75	2088.60	2163.76
family with sequence similarity 120C	Fam120c	100756331	4.27	4.16	6.99	7.54	8.18	7.74	930.44	900.39	1529.01	1628.28	1746.02	1664.65
family with sequence similarity 122A	Fam122a	100762589	8.78	8.23	9.28	9.24	8.37	9.48	283.31	280.00	304.31	304.80	274.27	308.44
family with sequence similarity 122B	Fam122b	100767852	3.18	3.28	3.72	3.87	5.39	5.14	337.99	341.31	371.38	391.71	521.63	502.85
family with sequence similarity 124A	Fam124a	100761912	0.00	0.00	0.00	0.00	0.00	0.00	0.00	0.00	0.00	0.00	0.00	0.00
family with sequence similarity 124B	Fam124b	100759764	0.00	0.00	0.00	0.00	0.00	0.00	0.00	0.00	0.00	1.16	0.00	0.00
family with sequence similarity 126, member A	Fam126a	100761584	36.44	35.84	35.46	38.42	40.70	38.90	4431.91	4410.46	4488.90	5010.01	5231.86	5161.25
family with sequence similarity 126, member B	Fam126b	100757717	1.21	1.33	1.29	1.52	1.65	1.17	158.74	169.28	172.65	194.70	210.10	157.96
family with sequence similarity 129, member A	Fam129a	100751843	29.70	28.52	32.73	33.61	33.16	34.08	3837.19	3690.33	4302.59	4401.58	4316.93	4455.57
family with sequence similarity 129, member B	Fam129b	100761378	52.27	49.99	49.74	46.14	39.60	41.80	5350.95	5136.08	5124.85	4731.87	4043.69	4266.77
family with sequence similarity 129, member C	Fam129c	100774372	0.10	0.11	0.08	0.13	0.11	0.09	19.75	15.56	19.87	19.70	14.49	16.82
family with sequence similarity 131, member A	Fam131a	100770285	7.13	6.94	8.07	5.81	4.08	5.10	417.75	409.02	452.12	292.05	202.86	257.97
family with sequence similarity 131, member B	Fam131b	100751738	1.25	1.37	0.51	0.67	0.47	0.50	150.39	156.47	62.10	79.97	55.89	64.49
family with sequence similarity 131, member C	Fam131c	100753151	0.00	0.00	0.00	0.00	0.00	0.00	0.00	0.00	0.00	0.00	0.00	0.00
family with sequence similarity 132, member A	Fam132a	100767884	0.12	0.03	0.40	0.30	0.10	0.24	12.15	2.75	22.36	17.38	6.21	16.82
family with sequence similarity 132, member B	Fam132b	100772579	0.00	0.00	0.00	0.00	0.00	0.00	0.00	0.00	0.00	0.00	0.00	0.00

family with sequence similarity 133, member A	Fam133a	100750880	2.11	2.12	2.14	2.15	3.29	3.22	192.16	189.41	188.80	191.22	293.94	298.16
family with sequence similarity 133, member B	Fam133b	100764219	35.23	35.95	33.17	34.54	35.32	32.32	2855.87	2942.75	2705.27	2791.84	2817.23	2589.97
family with sequence similarity 134, member A	Fam134a	100774602	36.05	33.37	38.04	37.26	32.78	38.07	2653.07	2477.00	2858.04	2791.84	2433.25	2833.92
family with sequence similarity 134, member B	Fam134b	100766605	16.71	16.45	17.05	19.73	20.33	18.78	1594.27	1572.94	1643.28	1869.34	1920.93	1795.50
family with sequence similarity 134, member C	Fam134c	100759145	17.19	15.95	13.57	12.08	11.53	12.91	1577.56	1486.01	1270.65	1124.15	1057.76	1189.83
family with sequence similarity 135, member A	Fam135a	100753684	15.69	15.19	17.89	21.27	21.84	20.57	2760.93	2701.18	3161.11	3774.60	3835.66	3637.73
family with sequence similarity 135, member B	Fam135b	100766234	0.00	0.00	0.00	0.00	0.00	0.00	0.76	0.00	0.00	0.00	0.00	0.00
family with sequence similarity 136, member A	Fam136a	100773052	30.95	31.68	28.87	28.79	27.37	25.14	1332.99	1347.84	1208.55	1185.58	1073.28	1016.92
family with sequence similarity 13, member A	Fam13a	100753068	1.77	1.57	1.66	1.76	1.72	1.63	274.95	251.63	257.11	268.87	262.89	259.84
family with sequence similarity 13, member B	Fam13b	100775045	8.15	7.73	6.66	7.60	8.92	8.28	924.36	876.60	763.88	848.33	984.27	925.32
family with sequence similarity 13, member C	Fam13c	100761386	0.00	0.00	0.00	0.00	0.00	0.00	0.00	0.00	0.00	0.00	0.00	0.00
family with sequence similarity 149, member A	Fam149a	100751039	19.75	20.39	22.57	25.06	26.27	24.11	2636.36	2739.61	3036.90	3343.48	3495.15	3210.59
family with sequence similarity 149, member B1	Fam149b1	100761680	10.31	9.82	9.39	8.47	9.59	10.35	681.31	660.65	634.71	601.48	685.16	689.79
family with sequence similarity 150, member A	Fam150a	103160480	0.00	0.00	0.00	0.00	0.00	0.00	0.00	0.00	0.00	0.00	0.00	0.00
family with sequence similarity 150, member B	Fam150b	100774090	0.00	0.00	0.00	0.00	0.00	0.00	0.00	0.00	0.00	0.00	0.00	0.00
family with sequence similarity 151, member A	Fam151a	100752673	0.16	0.02	0.08	0.05	0.07	0.06	15.19	11.90	24.84	10.43	19.66	22.43
family with sequence similarity 151, member B	Fam151b	100761480	11.51	12.31	11.36	12.89	13.83	14.99	413.19	446.54	421.07	450.82	479.20	529.96
family with sequence similarity 154, member A	Fam154a	100761997	0.02	0.00	0.00	0.00	0.00	0.00	0.76	0.00	0.00	0.00	0.00	0.93
family with sequence similarity 154, member B	Fam154b	100763911	0.12	0.18	0.21	0.21	0.12	0.21	13.67	23.79	21.12	23.18	20.70	21.50
family with sequence similarity 155, member A	Fam155a	100754176	0.00	0.00	0.00	0.00	0.00	0.00	0.00	0.00	0.00	0.00	0.00	0.00
family with sequence similarity 155, member B	Fam155b	100768072	0.00	0.00	0.00	0.00	0.00	0.00	0.00	0.00	0.00	0.00	0.00	0.00
family with sequence similarity 159, member A	Fam159a	100760742	0.00	0.00	0.00	0.00	0.00	0.00	0.00	0.00	0.00	0.00	0.00	0.00
family with sequence similarity 159, member B	Fam159b	100759831	0.00	0.00	0.00	0.00	0.00	0.00	0.76	0.00	0.00	0.00	1.03	0.00
family with sequence similarity 160, member A1	Fam160a1	100751310	0.01	0.00	0.01	0.00	0.00	0.00	3.04	0.00	3.73	1.16	0.00	0.93
family with sequence similarity 160, member A2	Fam160a2	100752778	12.26	11.49	11.99	9.88	7.95	8.73	1209.19	1151.11	1203.58	982.76	784.52	874.85
family with sequence similarity 160, member B1	Fam160b1	100768890	8.68	9.10	9.78	10.59	10.41	9.99	734.47	743.92	824.75	894.69	870.42	862.70
family with sequence similarity 160, member B2	Fam160b2	100750824	10.57	10.49	10.87	10.01	8.23	8.85	1213.74	1178.56	1238.36	1122.99	919.07	1006.64

family with sequence similarity 161, member A	Fam161a	100770716	5.12	5.35	7.62	7.41	8.72	8.27	449.65	473.07	704.26	690.72	806.25	749.61
family with sequence similarity 161, member B	Fam161b	100754415	4.97	5.97	5.23	5.93	6.21	7.87	299.26	365.10	319.22	356.95	364.32	457.99
family with sequence similarity 162, member A	Fam162a	100770984	46.02	44.92	53.40	68.90	63.81	63.07	516.49	488.63	585.02	713.89	670.67	681.37
family with sequence similarity 162, member B	Fam162b	100773450	0.00	0.00	0.00	0.00	0.00	0.00	0.00	0.00	0.00	0.00	0.00	0.00
family with sequence similarity 163, member A	Fam163a	100752117	0.06	0.10	0.00	0.00	0.00	0.00	3.80	6.41	0.00	0.00	0.00	0.93
family with sequence similarity 163, member B	Fam163b	100760830	0.00	0.00	0.00	0.00	0.00	0.00	0.00	0.00	0.00	0.00	0.00	0.00
family with sequence similarity 166, member A	Fam166a	100751030	0.09	0.00	0.00	0.00	0.04	0.00	3.04	0.92	2.48	3.48	2.07	1.87
family with sequence similarity 166, member B	Fam166b	100757125	0.63	0.57	0.15	0.32	0.49	0.55	22.79	17.39	4.97	11.59	15.52	16.82
family with sequence similarity 167, member A	Fam167a	100755242	0.22	0.24	0.03	0.00	0.05	0.05	21.27	21.05	3.73	1.16	4.14	4.67
family with sequence similarity 167, member B	Fam167b	100753020	0.00	0.00	0.00	0.00	0.00	0.00	0.00	0.00	0.00	0.00	0.00	0.00
family with sequence similarity 168, member A	Fam168a	100752186	14.61	15.14	13.81	14.45	15.66	16.31	1262.35	1292.03	1203.58	1236.57	1314.43	1418.83
family with sequence similarity 168, member B	Fam168b	100771076	49.09	49.43	48.92	48.41	51.25	51.33	5996.56	6067.59	6051.45	5931.35	6240.97	6330.52
family with sequence similarity 169, member A	Fam169a	100756076	4.11	4.18	4.06	4.96	4.90	4.66	711.69	713.73	725.38	873.83	842.48	829.05
family with sequence similarity 169, member B	Fam169b	100769400	0.02	0.00	0.00	0.03	0.06	0.10	3.80	1.83	1.24	1.16	6.21	4.67
family with sequence similarity 170, member A	Fam170a	100772259	0.00	0.00	0.00	0.00	0.00	0.00	0.00	0.00	0.00	0.00	0.00	0.00
family with sequence similarity 170, member B	Fam170b	100757364	0.09	0.00	0.00	0.00	0.00	0.00	3.04	1.83	1.24	1.16	0.00	0.93
family with sequence similarity 171, member A1	Fam171a1	100756305	14.52	13.70	14.92	15.55	16.20	15.73	1330.71	1281.05	1394.86	1446.33	1518.32	1479.58
family with sequence similarity 171, member A2	Fam171a2	100762527	8.00	7.70	5.63	3.22	1.61	2.15	385.09	382.48	273.26	163.41	83.83	118.70
family with sequence similarity 171, member B	Fam171b	100752911	0.00	0.01	0.00	0.00	0.00	0.00	0.76	0.92	0.00	0.00	0.00	0.00
family with sequence similarity 172, member A	Fam172a	100752273	49.54	49.18	55.65	53.25	52.40	53.45	941.83	950.72	1037.14	983.92	1013.25	1015.05
family with sequence similarity 173, member A	Fam173a	100773224	7.24	6.29	11.28	6.16	4.50	5.94	292.42	271.76	396.23	280.46	180.09	221.52
family with sequence similarity 173, member B	Fam173b	100759315	7.70	7.59	6.20	5.84	6.27	6.44	277.99	279.09	221.09	225.99	230.80	228.06
family with sequence similarity 174, member B	Fam174b	103162425	0.00	0.00	0.00	0.00	0.00	0.00	0.00	0.00	0.00	0.00	0.00	0.00
family with sequence similarity 175, member B	Fam175b	100770738	22.91	23.49	23.38	25.01	25.79	23.98	1917.84	1981.05	1981.13	2105.76	2164.16	2039.45
family with sequence similarity 177, member A1	Fam177a1	100753175	14.32	14.00	13.17	13.87	17.09	15.04	1028.42	995.56	963.86	1002.47	1211.97	1089.82
family with sequence similarity 177, member B	Fam177b	100755036	0.00	0.00	0.00	0.00	0.00	0.00	0.76	0.00	0.00	0.00	0.00	0.00
family with sequence similarity 178, member A	Fam178a	100756312	8.23	9.10	9.51	10.28	12.57	11.12	1511.48	1644.32	1700.42	1854.27	2223.15	1972.15

family with sequence similarity 178, member B	Fam178b	100768604	0.05	0.06	0.03	0.00	0.02	0.00	2.28	2.75	1.24	2.32	2.07	0.93
family with sequence similarity 179, member A	Fam179a	100760659	0.04	0.02	0.00	0.00	0.02	0.01	3.04	3.66	0.00	1.16	2.07	0.93
family with sequence similarity 179, member B	Fam179b	100756381	6.51	6.26	6.75	8.24	9.18	8.36	1141.59	1114.51	1207.31	1447.49	1632.17	1512.30
family with sequence similarity 180, member A	Fam180a	100773015	0.00	0.00	0.00	0.00	0.00	0.00	0.00	0.00	0.00	0.00	0.00	0.00
family with sequence similarity 180, member B	Fam180b	100763927	0.00	0.00	0.00	0.00	0.00	0.00	0.00	0.00	0.00	0.00	0.00	0.00
family with sequence similarity 181, member A	Fam181a	100767008	0.00	0.00	0.00	0.00	0.00	0.00	0.00	0.00	0.00	0.00	0.00	0.00
family with sequence similarity 183, member A	Fam183a	100768821	0.00	0.00	0.00	0.00	0.00	0.00	0.00	0.00	0.00	0.00	0.00	0.00
family with sequence similarity 184, member A	Fam184a	100767811	0.00	0.00	0.00	0.00	0.00	0.00	0.00	0.00	0.00	0.00	0.00	0.00
family with sequence similarity 184, member B	Fam184b	100768354	0.01	0.01	0.02	0.00	0.00	0.00	1.52	0.92	2.48	1.16	3.10	0.93
family with sequence similarity 185, member A	Fam185a	100761675	1.39	1.95	1.49	1.83	2.44	2.13	85.83	129.93	83.22	112.42	141.79	136.46
family with sequence similarity 186, member A	Fam186a	100774788	0.26	0.29	0.24	0.29	0.31	0.35	175.45	198.56	180.10	250.33	207.00	255.16
family with sequence similarity 186, member B	Fam186b	100766940	0.01	0.01	0.00	0.01	0.00	0.00	0.76	0.92	0.00	1.16	0.00	0.00
family with sequence similarity 188, member A	Fam188a	100769292	9.56	9.87	9.97	10.64	11.90	11.98	689.66	707.32	727.86	771.84	838.34	801.95
family with sequence similarity 188, member B	Fam188b	100773513	3.21	3.82	3.65	3.85	2.95	3.59	271.16	322.09	314.25	332.61	263.92	308.44
family with sequence similarity 189, member A1	Fam189a1	100764965	0.18	0.14	0.14	0.16	0.22	0.22	21.27	18.30	17.39	18.54	27.94	25.24
family with sequence similarity 189, member A2	Fam189a2	100759969	0.67	0.58	0.35	0.51	0.55	0.64	44.05	39.35	23.60	31.29	37.26	42.99
family with sequence similarity 189, member B	Fam189b	100756337	0.28	0.23	0.15	0.15	0.14	0.10	28.10	20.13	14.91	13.91	11.38	14.02
family with sequence similarity 192, member A	Fam192a	100759431	41.30	41.14	51.52	47.99	47.98	52.41	1495.53	1477.78	1805.99	1693.18	1603.19	1777.74
family with sequence similarity 193, member A	Fam193a	100769601	7.72	7.52	6.90	7.57	6.33	6.34	1171.97	1155.69	1052.05	1154.28	962.54	966.45
family with sequence similarity 193, member B	Fam193b	100770223	6.62	6.67	6.96	5.40	4.54	4.49	745.87	768.63	789.97	611.91	501.97	525.28
family with sequence similarity 195, member A	Fam195a	100759898	14.91	13.78	19.23	13.86	6.55	9.36	286.35	261.70	360.21	258.44	116.95	165.44
family with sequence similarity 195, member B	Fam195b	100756025	24.66	22.53	13.20	10.31	5.58	5.95	2934.86	2746.02	1614.71	1243.52	666.53	715.02
family with sequence similarity 196, member A	Fam196a	100751885	0.02	0.00	0.00	0.00	0.00	0.00	1.52	0.00	0.00	0.00	0.00	0.00
family with sequence similarity 196, member B	Fam196b	100756585	0.00	0.00	0.00	0.00	0.00	0.00	0.00	0.00	0.00	0.00	0.00	0.00
family with sequence similarity 198, member A	Fam198a	100766100	0.00	0.00	0.00	0.00	0.00	0.00	0.00	0.00	0.00	0.00	0.00	0.00
family with sequence similarity 198, member B	Fam198b	100764048	7.17	7.98	9.42	11.84	10.84	9.63	804.35	895.82	1058.26	1322.33	1205.76	1080.48
family with sequence similarity 199, X-linked	Fam199x	100757918	15.45	16.33	16.75	18.10	16.45	16.96	751.18	787.84	848.35	916.71	854.90	864.57

family with sequence similarity 19 (chemokine (C-C motif)-like), member A1	Fam19a1	100757363	0.00	0.00	0.00	0.00	0.00	0.00	0.00	0.00	0.00	0.00	0.00	0.00
family with sequence similarity 19 (chemokine (C-C motif)-like), member A2	Fam19a2	100759893	0.01	0.04	0.00	0.02	0.01	0.01	0.76	4.58	0.00	1.16	1.03	0.93
family with sequence similarity 19 (chemokine (C-C motif)-like), member A3	Fam19a3	100751401	0.00	0.00	0.00	0.00	0.00	0.00	0.00	0.00	0.00	0.00	0.00	0.00
family with sequence similarity 19 (chemokine (C-C motif)-like), member A4	Fam19a4	100760241	0.00	0.00	0.00	0.00	0.00	0.00	0.00	0.00	0.00	0.00	0.00	0.00
family with sequence similarity 203, member A	Fam203a	100764479	20.68	17.64	23.71	21.36	19.40	20.82	960.82	854.64	1130.30	990.88	917.00	958.04
family with sequence similarity 204, member A	Fam204a	100751254	12.67	11.37	9.01	10.64	11.34	10.02	675.23	635.95	544.03	591.05	618.92	563.61
family with sequence similarity 206, member A	Fam206a	100768046	22.53	23.12	21.25	21.79	23.51	21.85	968.41	1009.28	939.02	927.14	1020.50	942.15
family with sequence similarity 207, member A	Fam207a	100765418	19.84	18.29	20.28	17.01	12.29	12.36	971.45	906.80	1021.00	823.99	626.17	640.25
family with sequence similarity 208, member A	Fam208a	100752192	2.08	2.14	1.92	2.01	2.49	1.97	309.89	317.52	283.20	299.00	357.07	299.09
family with sequence similarity 208, member B	Fam208b	100766168	13.40	13.08	14.15	17.66	17.58	16.50	3467.30	3403.92	3712.60	4613.66	4558.08	4329.39
family with sequence similarity 20, member A	Fam20a	100750670	0.00	0.00	0.00	0.00	0.00	0.00	1.52	0.92	0.00	0.00	0.00	0.93
family with sequence similarity 20, member B	Fam20b	100772950	23.97	24.41	22.32	23.51	26.40	25.09	2984.99	3074.51	2813.33	2965.68	3307.82	3117.12
family with sequence similarity 20, member C	Fam20c	100771796	2.10	2.20	2.34	2.45	2.06	2.33	143.55	150.98	166.44	162.25	145.93	164.50
family with sequence similarity 210, member A	Fam210a	100758002	3.96	3.83	3.74	4.03	4.94	4.21	644.85	635.95	613.59	666.38	789.69	686.05
family with sequence similarity 210, member B	Fam210b	100764549	12.19	13.31	12.95	12.92	17.03	15.03	1086.90	1195.03	1188.68	1142.70	1498.66	1338.45
family with sequence similarity 212, member A	Fam212a	100759700	1.26	1.35	1.75	1.12	0.81	0.97	34.18	36.60	49.68	31.29	21.73	26.17
family with sequence similarity 212, member B	Fam212b	100751785	1.25	1.64	0.31	0.46	0.57	0.50	150.39	193.99	37.26	55.63	67.27	58.88
family with sequence similarity 213, member A	Fam213a	100759727	0.00	0.00	0.00	0.00	0.00	0.00	0.76	0.00	0.00	0.00	0.00	0.00
family with sequence similarity 213, member B	Fam213b	100755514	0.00	0.00	0.00	0.00	0.00	0.00	0.00	0.00	0.00	0.00	0.00	0.00
family with sequence similarity 214, member A	Fam214a	100753759	1.89	1.71	1.88	2.03	2.77	2.83	210.39	194.90	221.09	234.10	305.32	322.46
family with sequence similarity 214, member B	Fam214b	100769112	1.42	1.46	0.94	0.91	0.91	0.86	144.31	141.83	91.91	98.51	104.53	96.27
family with sequence similarity 216, member A	Fam216a	100751529	36.60	37.80	33.61	37.75	39.43	40.26	1054.24	1148.37	980.01	1067.37	1157.11	1136.56
family with sequence similarity 216, member B	Fam216b	100771008	0.00	0.00	0.00	0.00	0.00	0.00	0.00	0.00	0.00	0.00	0.00	0.00
family with sequence similarity 217, member A	Fam217a	100760256	0.00	0.00	0.00	0.00	0.00	0.00	0.00	0.00	0.00	0.00	0.00	0.00
family with sequence similarity 217, member B	Fam217b	100771062	0.00	0.00	0.00	0.00	0.00	0.00	0.00	0.00	0.00	0.00	0.00	0.00

family with sequence similarity 219, member A	Fam219a	100770378	0.58	0.80	0.49	1.69	0.83	0.95	12.91	20.13	14.91	26.66	21.73	19.63
family with sequence similarity 219, member B	Fam219b	100766049	25.14	24.56	34.41	34.60	33.44	35.19	2077.34	2015.82	2807.12	2808.06	2709.59	2825.51
family with sequence similarity 21, member C	Fam21c	100689255	21.97	23.17	28.85	32.91	32.70	31.98	2937.14	3072.68	3847.99	4282.21	4231.02	4209.75
family with sequence similarity 220, member A	Fam220a	100765308	30.86	32.25	33.55	35.04	39.99	38.63	1574.52	1652.55	1696.69	1784.74	2027.54	1949.72
family with sequence similarity 221, member A	Fam221a	100762448	0.00	0.00	0.00	0.00	0.00	0.00	0.76	0.92	0.00	0.00	0.00	0.00
family with sequence similarity 221, member B	Fam221b	100772069	0.10	0.07	0.02	0.01	0.03	0.08	11.39	11.90	4.97	6.95	4.14	10.28
family with sequence similarity 222, member A	Fam222a	100751362	0.03	0.07	0.04	0.03	0.06	0.00	2.28	4.58	2.48	3.48	4.14	0.00
family with sequence similarity 222, member B	Fam222b	100763154	5.25	5.26	5.69	4.99	4.32	4.32	773.97	769.54	863.25	727.80	619.96	644.92
family with sequence similarity 227, member A	Fam227a	100751092	0.50	0.81	3.29	3.19	1.17	1.31	26.58	37.52	124.21	121.69	70.38	77.58
family with sequence similarity 227, member B	Fam227b	100761884	0.44	0.31	0.26	0.15	0.33	0.32	16.71	14.64	9.94	6.95	16.56	15.89
family with sequence similarity 228, member A	Fam228a	100770212	1.13	1.01	0.89	0.70	0.40	0.33	95.70	84.18	77.01	57.95	31.05	27.11
family with sequence similarity 229, member A	Fam229a	103163721	0.00	0.00	0.00	0.00	0.00	0.00	6.84	8.24	12.42	3.48	2.07	1.87
family with sequence similarity 229, member B	Fam229b	100757032	10.40	9.88	5.85	6.58	8.40	8.93	152.67	146.41	89.43	96.19	104.53	119.64
family with sequence similarity 25, member A	Fam25a	100774241	29.85	30.06	12.31	10.09	3.63	5.20	282.55	312.94	119.24	97.35	38.29	43.93
family with sequence similarity 26, member D	Fam26d	100770005	0.00	0.00	0.00	0.00	0.00	0.00	0.00	0.00	0.00	0.00	0.00	0.00
family with sequence similarity 26, member E	Fam26e	100774191	0.23	0.13	0.24	0.20	0.22	0.13	12.91	7.32	13.66	13.91	12.42	7.48
family with sequence similarity 26, member F	Fam26f	100774476	0.77	0.76	0.38	0.44	0.45	0.61	9.11	12.81	6.21	8.11	6.21	8.41
family with sequence similarity 32, member A	Fam32a	100759796	66.27	62.81	70.52	67.19	60.62	66.47	1939.86	1856.60	2045.72	1897.15	1725.32	1877.75
family with sequence similarity 35, member A	Fam35a	100773947	4.66	4.77	5.43	6.36	6.91	6.31	474.71	491.37	588.75	667.54	715.18	659.88
family with sequence similarity 3, member A	Fam3a	100762082	21.57	20.80	19.30	16.22	14.09	15.52	1231.21	1198.69	1090.55	912.07	778.31	860.83
family with sequence similarity 3, member B	Fam3b	100764355	0.00	0.00	0.00	0.00	0.00	0.00	2.28	0.92	0.00	0.00	0.00	0.00
family with sequence similarity 3, member C	Fam3c	100764415	29.22	27.55	25.67	26.51	27.48	27.58	1792.51	1711.11	1597.32	1584.24	1668.40	1693.62
family with sequence similarity 3, member D	Fam3d	100765173	0.06	0.14	0.05	0.00	0.00	0.00	3.04	3.66	1.24	0.00	0.00	0.00
family with sequence similarity 43, member A	Fam43a	100759560	3.54	3.60	1.80	1.31	1.03	1.01	201.28	206.80	104.34	73.01	58.99	57.01
family with sequence similarity 45, member A	Fam45a	100752411	8.71	9.79	8.49	10.53	11.54	10.98	578.01	654.25	585.02	703.46	752.44	717.83
family with sequence similarity 46, member A	Fam46a	100751836	2.11	2.89	1.09	1.23	1.34	1.13	159.50	212.29	84.46	95.03	103.50	86.92
family with sequence similarity 46, member B	Fam46b	100771132	0.03	0.03	0.00	0.02	0.00	0.00	1.52	1.83	0.00	1.16	0.00	0.00

family with sequence similarity 46, member C	Fam46c	100770809	0.05	0.06	0.08	0.04	0.03	0.13	1.52	1.83	2.48	2.32	2.07	3.74
family with sequence similarity 46, member D	Fam46d	100755293	2.07	2.04	1.98	2.22	2.44	2.30	486.11	482.22	475.72	521.51	570.28	541.17
family with sequence similarity 49, member A	Fam49a	100756339	0.00	0.00	0.00	0.00	0.00	0.00	0.00	0.00	0.00	0.00	0.00	0.00
family with sequence similarity 50, member A	Fam50a	100759186	7.27	6.86	6.73	6.57	7.42	6.73	542.31	516.08	504.29	504.13	557.86	509.39
family with sequence similarity 50, member B	Fam50b	100759390	0.00	0.00	0.00	0.00	0.00	0.00	0.00	0.00	0.00	0.00	0.00	0.00
family with sequence similarity 53, member A	Fam53a	100766117	19.56	19.28	22.71	17.87	14.83	15.74	1182.60	1147.45	1382.44	1124.15	947.01	1029.07
family with sequence similarity 53, member B	Fam53b	100771312	3.42	3.43	3.68	3.07	2.63	2.94	489.14	504.18	541.55	454.30	370.53	429.95
family with sequence similarity 53, member C	Fam53c	100773129	5.87	5.67	5.73	4.62	3.64	4.11	294.70	278.17	293.13	231.78	166.63	193.48
family with sequence similarity 57, member A	Fam57a	100763695	11.60	11.08	14.88	15.31	18.48	19.08	630.42	613.07	799.90	812.40	982.20	999.16
family with sequence similarity 57, member B	Fam57b	100772583	0.17	0.10	0.09	0.13	0.04	0.00	7.60	4.58	2.48	3.48	2.07	1.87
family with sequence similarity 58, member A	Fam58a	100769050	0.00	0.00	0.00	0.00	0.00	0.00	0.00	0.00	0.00	0.00	0.00	0.00
family with sequence similarity 60, member A	Fam60a	100760112	21.93	22.62	26.13	25.27	26.65	27.05	1589.72	1635.16	1890.46	1826.46	1898.16	1958.13
family with sequence similarity 63, member A	Fam63a	100766575	24.30	23.96	29.91	29.36	25.94	27.03	1204.63	1195.95	1505.41	1479.94	1373.43	1434.72
family with sequence similarity 63, member B	Fam63b	100757261	5.61	5.56	4.41	5.08	6.52	6.13	1315.52	1280.13	1037.14	1192.53	1491.42	1432.85
family with sequence similarity 64, member A	Fam64a	100755771	0.00	0.00	0.00	0.00	0.00	0.00	0.00	0.00	0.00	0.00	0.00	0.00
family with sequence similarity 65, member A	Fam65a	100762946	18.60	16.83	21.21	20.71	17.35	17.14	2195.07	1981.96	2506.53	2437.21	2018.22	2043.19
family with sequence similarity 65, member B	Fam65b	100763872	0.00	0.02	0.00	0.00	0.00	0.00	0.76	2.75	0.00	0.00	0.00	0.00
family with sequence similarity 65, member C	Fam65c	100757599	0.08	0.08	0.08	0.02	0.04	0.04	16.71	11.90	14.91	2.32	5.17	7.48
family with sequence similarity 69, member A	Fam69a	100764055	3.51	3.26	4.43	4.48	5.48	5.60	163.30	157.39	192.52	181.95	219.42	246.75
family with sequence similarity 69, member B	Fam69b	100754761	0.02	0.05	0.00	0.00	0.00	0.00	0.76	2.75	1.24	0.00	0.00	0.00
family with sequence similarity 69, member C	Fam69c	100775103	0.04	0.22	0.02	0.00	0.02	0.00	5.32	14.64	1.24	0.00	1.03	0.00
family with sequence similarity 71, member A	Fam71a	100763531	0.02	0.02	0.03	0.10	0.07	0.06	1.52	0.92	1.24	4.64	4.14	2.80
family with sequence similarity 71, member B	Fam71b	100767497	0.03	0.08	0.03	0.00	0.07	0.04	1.52	5.49	3.73	0.00	3.10	2.80
family with sequence similarity 71, member D	Fam71d	100756714	0.02	0.02	0.03	0.00	0.00	0.00	3.04	4.58	1.24	0.00	2.07	1.87
family with sequence similarity 71, member E1	Fam71e1	100756180	0.71	0.71	0.27	0.20	0.06	0.42	19.75	19.22	9.94	6.95	1.03	11.22
family with sequence similarity 71, member E2	Fam71e2	100773246	0.04	0.02	0.04	0.00	0.00	0.00	2.28	0.92	2.48	1.16	0.00	0.93
family with sequence similarity 71, member F1	Fam71f1	100760841	0.36	0.45	0.30	0.28	0.15	0.11	28.86	31.11	24.84	22.02	20.70	25.24

family with sequence similarity 71, member F2	Fam71f2	100750641	0.21	0.15	0.18	0.23	0.12	0.18	12.91	9.15	11.18	15.07	8.28	11.22
family with sequence similarity 72, member A	Fam72a	100757979	3.39	3.69	2.57	2.62	3.50	3.19	136.72	141.83	105.58	103.14	132.48	129.92
family with sequence similarity 73, member A	Fam73a	100758334	0.02	0.01	0.01	0.01	0.08	0.02	3.04	1.83	3.73	2.32	8.28	1.87
family with sequence similarity 73, member B	Fam73b	100753105	14.32	13.49	19.18	14.80	11.25	12.01	763.34	706.41	1014.78	756.77	575.45	668.29
family with sequence similarity 76, member B	Fam76b	100764937	18.28	18.74	15.93	18.86	20.71	20.04	1870.75	1937.13	1675.58	1941.19	2111.37	2045.99
family with sequence similarity 78, member A	Fam78a	100753079	0.79	0.47	0.34	0.49	0.64	0.65	34.94	21.96	19.87	20.86	28.98	28.97
family with sequence similarity 78, member B	Fam78b	100760702	7.19	7.31	11.30	11.85	11.02	10.23	825.62	835.43	1324.06	1377.96	1267.86	1194.51
family with sequence similarity 81, member A	Fam81a	100774523	0.00	0.00	0.00	0.00	0.00	0.00	0.00	0.00	0.00	0.00	0.00	0.00
family with sequence similarity 81, member B	Fam81b	100759412	0.00	0.00	0.00	0.00	0.00	0.00	0.00	0.00	0.00	0.00	0.00	0.00
family with sequence similarity 83, member A	Fam83a	100760967	0.00	0.00	0.00	0.00	0.00	0.00	0.00	0.00	0.00	0.00	0.00	0.00
family with sequence similarity 83, member B	Fam83b	100761983	0.00	0.00	0.00	0.00	0.00	0.00	0.00	0.00	0.00	0.00	0.00	0.00
family with sequence similarity 83, member C	Fam83c	100761734	4.55	4.59	7.83	7.67	6.64	7.54	547.63	552.68	937.78	923.66	784.52	911.30
family with sequence similarity 83, member D	Fam83d	100761533	11.95	11.61	12.45	13.73	14.75	13.74	748.91	734.77	786.24	873.83	939.77	873.92
family with sequence similarity 83, member E	Fam83e	100756463	0.02	0.00	0.00	0.00	0.00	0.00	1.52	0.00	0.00	0.00	0.00	0.93
family with sequence similarity 83, member F	Fam83f	100771028	0.00	0.00	0.00	0.00	0.00	0.00	0.00	0.00	0.00	0.00	0.00	0.00
family with sequence similarity 83, member H	Fam83h	100754245	0.34	0.30	0.02	0.01	0.02	0.02	41.77	39.35	2.48	2.32	3.10	2.80
family with sequence similarity 84, member B	Fam84b	100765324	0.00	0.00	0.00	0.00	0.00	0.00	0.00	0.00	0.00	0.00	0.00	0.00
family with sequence similarity 86, member A	Fam86a	100772867	26.49	25.10	26.48	26.40	20.17	21.43	1034.49	992.81	1023.48	1004.78	780.38	844.94
family with sequence similarity 89, member A	Fam89a	100772991	0.00	0.00	0.00	0.03	0.00	0.00	0.00	0.00	0.00	1.16	0.00	0.00
family with sequence similarity 89, member B	Fam89b	100771743	19.58	19.33	21.25	16.84	14.19	15.06	891.70	862.88	953.92	756.77	635.48	701.00
family with sequence similarity 8, member A1	Fam8a1	100761518	31.21	33.07	32.58	37.99	45.10	41.44	2927.26	3128.50	3084.10	3576.43	4202.04	3908.79
family with sequence similarity 90, member A1	Fam90a1	103164082	0.00	0.00	0.00	0.00	0.00	0.00	0.00	0.00	0.00	0.00	0.00	0.00
family with sequence similarity 91, member A1	Fam91a1	103158823	15.13	11.81	9.64	10.26	9.35	11.86	323.56	266.27	186.31	206.29	212.17	241.14
family with sequence similarity 92, member A1	Fam92a1	100762247	20.87	21.99	23.31	27.12	30.34	25.06	633.46	654.25	730.35	823.99	924.24	788.86
family with sequence similarity 92, member B	Fam92b	103159421	0.00	0.00	0.00	0.00	0.00	0.00	0.00	0.00	0.00	0.00	0.00	0.00
family with sequence similarity 96, member A	Fam96a	100773305	40.65	44.58	40.22	36.25	36.77	36.75	1409.70	1561.05	1418.46	1271.34	1281.31	1285.17
family with sequence similarity 96, member B	Fam96b	100760527	18.77	17.10	16.79	16.21	14.71	14.73	805.11	731.11	736.56	686.08	647.90	629.97

family with sequence similarity 98, member A	Fam98a	100764161	64.83	63.41	69.52	73.15	74.58	75.61	4946.12	4853.34	5311.16	5562.82	5651.03	5779.06
family with sequence similarity 98, member B	Fam98b	100762330	84.59	87.06	81.83	82.51	85.23	84.65	2230.77	2319.61	2163.72	2149.80	2186.93	2175.91
family with sequence similarity 98, member C	Fam98c	100765434	9.05	8.81	8.63	5.32	4.07	4.06	303.06	284.58	293.13	191.22	178.02	195.35
FANCD2/FANCI-associated nuclease 1	Fan1	100762441	7.63	7.40	8.42	9.15	8.99	9.21	853.72	831.77	942.74	1008.26	986.34	1016.92
Fanconi anemia, complementation group A	Fanca	100761220	5.21	5.29	6.22	6.54	7.07	7.37	784.60	799.74	943.99	974.65	1054.65	1108.52
Fanconi anemia, complementation group B	Fancb	100750774	3.50	3.69	3.82	4.39	5.69	4.50	330.40	350.46	363.93	428.80	542.33	443.03
Fanconi anemia, complementation group C	Fancc	100753982	8.64	6.99	9.21	11.28	11.12	9.57	171.66	146.41	204.94	197.02	210.10	186.93
Fanconi anemia, complementation group D2	Fancd2	100757761	19.26	19.98	18.81	21.02	25.63	23.93	3072.34	3229.15	2972.31	3351.60	4045.76	3755.50
FANCD2 opposite strand	Fancd2 os	100758053	0.15	0.09	0.14	0.18	0.20	0.25	5.32	4.58	3.73	4.64	5.17	9.35
Fanconi anemia, complementation group E	Fance	100765848	12.96	12.94	15.99	11.79	7.14	7.90	710.93	704.58	903.00	669.86	396.40	443.97
Fanconi anemia, complementation group F	Fancf	100765540	7.88	7.83	7.79	8.85	8.98	8.43	330.40	331.24	326.67	368.54	369.49	353.31
Fanconi anemia, complementation group G	Fancg	100689273	3.57	3.70	4.88	4.55	4.51	5.32	249.89	273.60	360.21	334.93	330.16	376.67
Fanconi anemia, complementation group I	Fanci	100755795	10.56	11.30	10.27	12.67	14.69	14.38	1328.43	1424.71	1275.62	1579.61	1834.00	1795.50
Fanconi anemia, complementation group L	Fancl	100759885	14.17	14.77	10.86	12.37	13.92	15.83	527.88	553.60	402.44	457.77	536.12	557.06
Fanconi anemia, complementation group M	Fancm	100768967	3.41	3.50	3.68	3.81	4.70	4.48	633.46	661.57	695.57	717.37	867.32	833.73
fibronectin type III and ankyrin repeat domains 1	Fank1	100760059	0.57	0.44	0.44	0.38	0.91	0.61	22.03	16.47	17.39	13.91	35.19	22.43
fibroblast activation protein, alpha	Fap	100767303	0.05	0.03	0.00	0.00	0.03	0.01	3.80	3.66	0.00	0.00	2.07	1.87
fatty acyl CoA reductase 1	Far1	100761128	23.05	23.09	19.71	21.95	23.43	22.35	2932.58	2923.53	2522.68	2774.45	2964.20	2830.18
FERM, RhoGEF (ARHGEF) and pleckstrin domain protein 1 (chondrocyte-derived)	Farp1	100761407	11.80	12.18	13.45	13.99	12.45	12.28	1598.07	1690.07	1885.49	1916.85	1690.13	1668.39
FERM, RhoGEF and pleckstrin domain protein 2	Farp2	100772725	1.25	1.21	1.19	1.08	1.54	1.38	150.39	140.92	111.79	98.51	154.21	150.48
phenylalanyl-tRNA synthetase 2, mitochondrial	Fars2	100761130	31.91	30.77	41.44	39.41	37.26	39.60	1218.30	1162.09	1538.95	1421.99	1380.67	1429.11
phenylalanyl-tRNA synthetase, alpha subunit	Farsa	100763673	72.23	65.81	74.89	67.82	53.15	61.41	3916.94	3650.98	4192.04	3691.16	2937.29	3274.15
phenylalanyl-tRNA synthetase, beta subunit	Farsb	100760139	74.16	74.39	71.26	77.13	86.80	81.52	4326.34	4379.35	4144.84	4387.67	4979.32	4623.81
Fas cell surface death receptor	Fas	100762537	7.55	7.56	4.74	6.09	6.11	5.99	585.60	577.39	366.42	460.09	454.36	449.58
Fas ligand (TNF superfamily, member 6)	Faslg	100773293	0.00	0.00	0.00	0.00	0.00	0.05	0.76	0.00	0.00	0.00	0.00	0.93
fatty acid synthase	Fasn	100689207	30.70	28.96	11.11	9.54	5.03	5.77	7894.65	7581.97	2935.05	2485.88	1346.52	1505.75
Fas-activated serine/threonine kinase	Fastk	100762130	14.07	14.05	17.55	15.14	9.35	10.72	903.09	902.22	1158.87	954.95	616.85	710.35
FAST kinase domains 1	Fastkd1	100763353	9.85	9.09	9.32	9.43	10.34	9.31	1010.19	972.68	1006.09	963.06	1107.44	963.64
FAST kinase domains 2	Fastkd2	100757409	24.54	25.34	27.24	29.45	30.14	29.53	1673.26	1753.20	1842.01	1977.12	2021.33	1970.28

FAST kinase domains 3	Fastkd3	100772361	9.41	8.38	9.84	10.43	10.15	10.45	645.61	570.98	663.27	683.76	668.60	695.39
FAST kinase domains 5	Fastkd5	103163403	9.48	8.78	9.75	10.66	10.56	9.88	767.13	719.22	804.87	878.46	863.18	812.23
FAT atypical cadherin 1	Fat1	100766718	24.55	24.97	25.33	29.12	32.28	31.12	10874.32	11111.25	11326.59	12970.63	14234.17	13838.72
FAT atypical cadherin 2	Fat2	100766386	0.00	0.00	0.00	0.00	0.00	0.00	0.00	0.00	0.00	1.16	0.00	0.00
FAT atypical cadherin 3	Fat3	100754211	0.00	0.00	0.00	0.00	0.00	0.00	0.76	0.00	0.00	0.00	0.00	0.00
FAT atypical cadherin 4	Fat4	100769423	0.11	0.11	0.01	0.01	0.01	0.01	57.72	57.65	3.73	4.64	5.17	3.74
fetal and adult testis expressed 1	Fate1	103162528	0.00	0.00	0.00	0.00	0.00	0.00	0.00	0.00	0.00	0.00	0.00	0.00
Finkel-Biskis-Reilly murine sarcoma virus (FBR-MuSV) ubiquitously expressed	Fau	100689400	1020.52	960.92	982.90	744.37	549.35	635.16	12985.08	12195.57	12055.69	8077.67	5592.03	6266.96
failed axon connections homolog (Drosophila)	Faxc	100765533	0.00	0.00	0.00	0.00	0.00	0.00	0.00	0.00	0.00	0.00	0.00	0.00
fatty acid hydroxylase domain containing 2	Faxdc2	100767373	1.18	1.06	1.14	1.19	1.53	1.17	69.12	74.12	79.49	75.33	104.53	77.58
Fas (TNFRSF6) binding factor 1	Fbf1	100758866	7.08	7.02	9.51	8.21	7.13	7.53	1180.32	1164.84	1582.42	1359.41	1149.87	1225.35
fibrillarin	Fbl	100765730	215.49	196.79	171.62	145.41	133.62	143.25	6613.31	6160.01	5369.54	4447.93	4160.64	4372.38
filamin binding LIM protein 1	Fblim1	100774941	4.13	4.14	3.55	2.82	2.70	2.92	210.39	225.10	195.01	158.77	148.00	166.37
fibulin 1	Fbln1	100757176	0.00	0.00	0.00	0.00	0.00	0.00	0.00	0.00	0.00	0.00	0.00	0.00
fibulin 2	Fbln2	100763343	0.05	0.05	0.02	0.00	0.00	0.01	4.56	4.58	2.48	0.00	0.00	0.93
fibulin 5	Fbln5	100759853	0.03	0.05	0.02	0.02	0.04	0.00	1.52	2.75	2.48	1.16	2.07	0.00
fibulin 7	Fbln7	100755635	0.00	0.00	0.00	0.00	0.00	0.00	0.00	0.00	0.00	0.00	0.00	0.00
fibrillin 1	Fbn1	100761283	0.10	0.10	0.03	0.05	0.05	0.04	31.14	28.37	7.45	17.38	14.49	14.02
fibrillin 2	Fbn2	100769316	0.00	0.00	0.00	0.00	0.00	0.00	0.76	0.00	0.00	0.00	0.00	0.00
fructose-1,6-bisphosphatase 2	Fbp2	100761248	0.00	0.03	0.00	0.00	0.00	0.00	0.00	0.92	0.00	0.00	0.00	0.00
fibrosin	Fbrs	100754050	10.12	9.05	6.93	3.44	1.40	1.79	546.11	489.54	453.36	281.62	175.95	214.97
fibrosin-like 1	Fbrsl1	100774092	2.18	1.68	2.03	1.42	0.68	0.91	233.18	183.92	203.70	150.66	75.55	93.47
F-box and leucine-rich repeat protein 12	Fbxl12	100764456	7.78	8.12	8.27	5.50	5.55	5.95	424.58	454.77	454.60	308.27	292.90	313.11
F-box and leucine-rich repeat protein 13	Fbxl13	100761383	0.08	0.08	0.12	0.05	0.15	0.07	10.63	7.32	8.69	6.95	19.66	13.09
F-box and leucine-rich repeat protein 14	Fbxl14	100755365	11.74	12.64	13.12	11.04	10.83	10.89	802.83	863.79	917.90	755.62	749.33	766.43
F-box and leucine-rich repeat protein 15	Fbxl15	100752453	6.68	4.81	5.45	3.74	2.00	2.36	199.76	148.24	154.02	107.78	54.85	67.30
F-box and leucine-rich repeat protein 16	Fbxl16	100772347	11.70	11.87	13.44	12.04	9.15	9.41	1116.52	1133.73	1278.11	1131.11	844.55	890.74
F-box and leucine-rich repeat protein 17	Fbxl17	100757296	32.57	31.56	34.03	36.42	37.20	37.73	1601.11	1507.06	1741.41	1715.20	1802.95	1769.33
F-box and leucine-rich repeat protein 18	Fbxl18	100767914	9.25	8.81	9.63	7.14	5.02	5.96	865.12	840.92	922.87	679.13	481.27	574.82
F-box and leucine-rich repeat protein 19	Fbxl19	100757739	14.09	14.02	15.70	11.90	8.30	9.29	1298.81	1314.90	1454.48	1100.97	767.96	872.05
F-box and leucine-rich repeat protein 2	Fbxl2	100766573	2.71	3.06	2.88	2.46	2.92	3.04	243.81	277.26	269.53	225.99	262.89	268.25
F-box and leucine-rich repeat protein 20	Fbxl20	100767550	14.42	14.00	11.97	12.35	12.22	14.27	1331.47	1303.01	1099.25	1141.54	1161.25	1330.03
F-box and leucine-rich repeat protein 21	Fbxl21	100753212	0.00	0.00	0.00	0.00	0.00	0.00	0.00	0.00	0.00	0.00	0.00	0.00
F-box and leucine-rich repeat protein 22	Fbxl22	103163837	1.40	1.43	1.68	1.55	1.71	1.98	158.74	161.96	193.77	180.79	196.65	228.06

F-box and leucine-rich repeat protein 3	Fbxl3	100761851	26.55	26.23	23.73	27.44	28.70	27.02	1782.64	1762.35	1604.78	1829.93	1907.48	1822.61
F-box and leucine-rich repeat protein 4	Fbxl4	100764957	17.62	16.54	18.02	19.15	20.85	19.53	609.15	573.73	603.65	665.22	711.04	674.83
F-box and leucine-rich repeat protein 5	Fbxl5	100768201	9.19	9.35	9.35	10.75	10.37	11.34	504.33	506.01	540.31	620.02	605.47	653.33
F-box and leucine-rich repeat protein 6	Fbxl6	100762650	9.79	9.30	14.42	11.44	8.82	9.58	591.68	567.32	847.10	664.06	517.49	565.48
F-box and leucine-rich repeat protein 7	Fbxl7	100755012	0.00	0.00	0.00	0.00	0.00	0.00	0.00	0.00	0.00	0.00	0.00	0.00
F-box and leucine-rich repeat protein 8	Fbxl8	100758388	2.85	2.97	2.73	1.59	1.56	1.09	157.98	158.30	141.60	90.40	87.97	63.56
F-box protein 10	Fbxo10	100751576	0.65	0.64	0.75	0.57	0.55	0.58	73.68	68.63	77.01	57.95	61.06	64.49
F-box protein 11	Fbxo11	100771372	29.24	28.09	29.10	31.40	37.01	32.67	2887.01	2787.19	2891.58	3107.06	3632.80	3236.76
F-box protein 15	Fbxo15	100750915	2.68	2.42	1.98	1.54	1.90	1.96	154.19	135.42	110.55	86.92	114.88	124.31
F-box protein 16	Fbxo16	100759298	0.04	0.03	0.00	0.00	0.00	0.00	2.28	0.92	0.00	1.16	0.00	0.93
F-box protein, helicase, 18	Fbxo18	100765086	29.95	30.57	31.53	33.10	35.60	36.68	3111.83	3205.36	3317.61	3380.57	3632.80	3662.97
F-box protein 2	Fbxo2	100762647	0.00	0.00	0.00	0.00	0.00	0.00	0.00	0.00	0.00	0.00	0.00	0.00
F-box protein 21	Fbxo21	100752874	28.03	27.40	30.05	29.62	28.58	31.42	3082.97	3047.98	3352.39	3284.38	3158.78	3443.32
F-box protein 22	Fbxo22	100756994	32.93	30.64	33.22	32.97	36.43	34.21	1111.21	1076.99	1150.17	1095.18	1222.32	1110.39
F-box protein 24	Fbxo24	100755996	0.38	0.55	0.32	0.41	0.26	0.32	41.02	53.99	37.26	48.67	36.22	41.13
F-box protein 25	Fbxo25	100758634	8.78	8.42	8.16	9.56	11.05	11.71	492.18	475.82	474.48	548.17	631.34	648.66
F-box protein 27	Fbxo27	100770350	10.40	9.33	9.94	9.43	9.86	10.22	398.76	385.23	404.92	376.65	390.19	395.37
F-box protein 28	Fbxo28	100754171	17.42	18.03	18.72	17.92	19.96	20.82	2238.36	2356.21	2448.15	2306.25	2587.47	2704.00
F-box protein 3	Fbxo3	100755440	26.44	26.54	28.16	30.65	35.96	33.38	1762.89	1798.04	1917.78	2105.76	2431.18	2238.53
F-box protein 30	Fbxo30	100765066	4.60	4.47	5.13	5.97	5.54	5.34	519.52	510.59	581.30	669.86	617.89	599.12
F-box protein 31	Fbxo31	100756007	7.85	7.59	8.15	7.75	6.93	7.08	1063.35	1027.58	1105.46	1043.03	911.82	951.49
F-box protein 32	Fbxo32	103158884	16.57	16.00	12.29	13.96	14.14	14.03	715.49	703.66	540.31	598.00	607.54	620.62
F-box protein 33	Fbxo33	103160846	17.87	17.31	20.03	19.60	21.59	21.10	1334.51	1290.20	1494.23	1463.72	1569.04	1612.31
F-box protein 34	Fbxo34	100755456	11.81	11.97	12.95	12.92	13.62	12.47	972.21	990.07	1075.65	1065.05	1112.61	1029.07
F-box protein 36	Fbxo36	100753561	3.15	2.77	3.76	4.07	3.85	3.34	75.19	64.97	100.61	113.57	97.29	100.94
F-box protein 38	Fbxo38	100763577	9.19	9.15	9.64	10.66	11.52	11.55	1236.53	1245.36	1294.25	1439.38	1521.43	1543.14
F-box protein 39	Fbxo39	100754321	0.02	0.00	0.00	0.00	0.00	0.00	1.52	0.00	0.00	0.00	0.00	0.00
F-box protein 4	Fbxo4	100768716	10.99	11.41	12.07	11.51	12.39	11.78	495.98	506.01	541.55	509.92	554.75	534.63
F-box protein 40	Fbxo40	100769091	0.00	0.00	0.00	0.00	0.00	0.00	0.00	0.00	0.00	0.00	0.00	0.00

F-box protein 41	Fbxo41	100770848	0.00	0.00	0.00	0.00	0.00	0.00	0.00	0.00	0.00	0.00	0.00	0.00
F-box protein 42	Fbxo42	100751392	12.01	11.84	11.68	10.64	10.26	10.11	890.94	865.62	863.25	788.07	775.20	797.27
F-box protein 43	Fbxo43	100762936	0.00	0.00	0.00	0.00	0.00	0.00	0.00	0.00	0.00	0.00	0.00	0.00
F-box protein 44	Fbxo44	100761288	0.00	0.00	0.00	0.00	0.00	0.00	0.76	0.00	1.24	0.00	0.00	0.00
F-box protein 45	Fbxo45	100761316	14.21	14.50	17.45	18.40	18.75	18.84	1487.94	1516.21	1835.80	1915.69	1939.56	1966.55
F-box protein 46	Fbxo46	103159278	4.78	3.67	4.06	2.14	1.21	1.44	193.68	165.62	191.28	107.78	66.24	92.53
F-box protein 47	Fbxo47	100757104	1.46	1.40	0.71	0.97	1.11	0.95	91.90	96.99	45.96	64.90	75.55	65.43
F-box protein 48	Fbxo48	100774875	0.75	0.78	0.70	0.44	0.40	0.58	31.14	33.86	36.02	27.81	33.12	33.65
F-box protein 5	Fbxo5	100773759	14.90	15.55	15.49	18.94	19.88	18.37	838.53	873.86	876.91	1075.48	1128.14	1036.55
F-box protein 6	Fbxo6	100762938	92.92	91.65	96.85	87.34	67.58	79.15	2650.03	2579.48	2728.86	2416.35	1901.27	2207.69
F-box protein 7	Fbxo7	100753632	21.92	20.34	21.81	22.69	21.22	21.69	1042.85	986.41	1037.14	1092.86	995.66	1021.59
F-box protein 8	Fbxo8	100754825	17.13	17.86	16.59	19.09	21.82	21.51	950.94	990.07	925.35	1034.92	1178.85	1182.36
F-box protein 9	Fbxo9	103159530	20.22	19.82	20.55	22.43	25.08	25.23	1105.13	1086.14	1130.30	1226.14	1345.48	1348.73
F-box and WD repeat domain containing 11	Fbxw11	100772242	26.06	25.63	27.05	28.12	29.05	31.29	2801.18	2796.34	2997.16	3118.65	3257.10	3491.93
F-box and WD repeat domain containing 12	Fbxw12	100763018	0.06	0.02	0.03	0.06	0.00	0.00	4.56	1.83	2.48	2.32	0.00	0.00
F-box and WD repeat domain containing 2	Fbxw2	100756088	37.19	35.06	43.38	45.97	46.27	45.25	1821.38	1667.19	2073.04	2153.27	2134.14	2215.17
F-box and WD repeat domain containing 4	Fbxw4	100751884	11.98	11.33	13.25	12.33	9.69	10.25	519.52	494.12	580.05	526.15	419.17	457.99
F-box and WD repeat domain containing 5	Fbxw5	100762398	26.46	25.19	29.10	29.44	22.68	27.09	1888.21	1778.83	2082.98	2081.42	1596.98	1893.64
F-box and WD repeat domain containing 7, E3 ubiquitin protein ligase	Fbxw7	100761759	6.01	5.36	5.32	6.25	6.33	5.96	704.85	640.52	633.46	732.44	745.19	708.48
F-box and WD repeat domain containing 8	Fbxw8	100752372	25.85	26.45	29.57	27.30	23.07	25.86	2078.86	2120.13	2434.49	2203.11	1901.27	2086.18
F-box and WD repeat domain containing 9	Fbxw9	100758460	7.39	7.38	8.27	6.59	5.50	5.81	429.14	440.13	500.56	395.19	329.13	348.63
Fc receptor, IgA, IgM, high affinity	Fcamr	100773580	0.02	0.00	0.03	0.00	0.00	0.00	1.52	0.00	1.24	0.00	1.03	0.93
Fc fragment of IgE, high affinity I, receptor for; gamma polypeptide	Fcer1g	100768356	0.17	0.27	0.09	0.00	0.23	0.21	7.60	9.15	1.24	4.64	7.24	5.61
Fc fragment of IgE, low affinity II, receptor for (CD23)	Fcer2	100769597	2.69	2.63	0.63	0.39	0.86	0.70	198.24	191.24	50.93	38.24	67.27	59.82
FCF1 rRNA-processing protein	Fcf1	100771331	56.92	55.38	56.31	56.60	62.17	62.16	1098.29	1117.26	1071.92	1085.91	1199.55	1159.93
Fc fragment of IgG, low affinity IIa, receptor (CD32)	Fcgr2a	103161891	12.51	12.34	12.93	14.23	14.71	15.12	766.38	747.58	787.48	871.51	892.16	918.78
Fc fragment of IgG, low affinity IIb, receptor (CD32)	Fcgr2b	103161893	0.31	0.28	0.12	0.04	0.00	0.03	11.39	6.41	1.24	1.16	0.00	0.00
Fc fragment of IgG, receptor, transporter, alpha	Fcgrt	100755637	19.95	18.32	9.60	7.46	5.18	5.49	826.38	775.03	404.92	326.82	212.17	242.08
FCH domain only 1	Fcho1	100772920	1.32	1.22	0.75	0.49	0.53	0.39	157.98	145.49	95.64	69.54	74.52	59.82
FCH domain only 2	Fcho2	100760398	5.99	5.79	7.43	9.71	10.22	9.26	856.76	840.00	1060.74	1362.89	1440.70	1312.28
FCH and double SH3 domains 1	Fchsd1	100752340	5.92	6.11	7.20	6.68	4.71	6.10	407.87	423.66	498.08	468.20	331.20	404.71

FCH and double SH3 domains 2	Fchsd2	100773820	4.58	4.48	4.41	5.04	5.97	5.62	546.11	540.78	510.50	602.64	694.48	647.73
Fc receptor-like 5	Fcrl5	100760293	0.00	0.00	0.00	0.00	0.00	0.00	0.00	0.00	0.00	0.00	0.00	0.00
Fc receptor-like 6	Fcrl6	100755952	0.00	0.00	0.00	0.00	0.00	0.00	0.00	0.00	0.00	0.00	0.00	0.00
Fc receptor-like A	Fcrla	100771125	0.03	0.11	0.10	0.05	0.17	0.04	1.52	2.75	3.73	3.48	8.28	2.80
Fc receptor-like B	Fcrlb	100771895	0.00	0.03	0.00	0.00	0.00	0.00	0.00	0.92	0.00	0.00	0.00	0.00
farnesyl-diphosphate farnesyltransferase 1	Fdft1	100689192	8.69	9.06	12.01	11.82	11.16	10.49	555.98	584.71	768.85	763.73	719.32	674.83
farnesyl diphosphate synthase	Fdps	100754883	48.65	45.38	72.14	72.64	72.13	72.17	2441.92	2318.69	3628.14	3613.51	3493.08	3553.61
ferredoxin 1	Fdx1	100751853	41.25	39.87	40.06	41.68	43.76	47.15	1027.66	985.49	980.01	978.13	1054.65	1131.88
ferredoxin 1-like	Fdx1l	100761259	35.36	37.86	36.87	28.04	15.39	17.54	1080.82	1155.69	1084.34	800.81	439.87	504.72
ferredoxin-fold anticodon binding domain containing 1	Fdxacb1	100771758	2.58	2.37	1.77	2.00	1.81	2.07	331.16	309.28	233.51	249.17	238.05	272.92
ferredoxin reductase	Fdxr	100773278	24.38	24.70	36.82	28.37	17.67	18.46	1345.14	1345.10	2025.84	1548.32	1001.87	1031.88
ferrochelatase	Fech	100764152	10.91	11.28	11.78	13.58	12.83	13.47	890.94	917.78	957.65	1113.72	1036.02	1101.04
fem-1 homolog a (C. elegans)	Fem1a	100769384	17.66	17.29	16.05	14.26	9.86	10.21	1490.22	1464.05	1376.23	1211.07	839.37	875.79
fem-1 homolog b (C. elegans)	Fem1b	100761263	28.05	28.53	31.47	35.89	36.51	34.67	4578.50	4661.18	5190.68	5946.42	5972.91	5691.20
fem-1 homolog c (C. elegans)	Fem1c	100760897	6.68	6.80	6.28	7.14	7.96	7.00	821.06	828.11	775.06	883.10	964.61	861.77
flap structure-specific endonuclease 1	Fen1	100756103	13.14	11.92	15.41	14.19	9.97	9.75	712.45	672.55	885.61	859.92	667.57	639.31
fer (fps/fes related) tyrosine kinase	Fer	100755838	7.72	8.25	7.30	8.45	10.10	9.39	1807.70	1909.67	1710.35	1964.37	2275.93	2139.46
fer-1-like 5 (C. elegans)	Fer1l5	100773388	0.40	0.38	0.32	0.39	0.24	0.27	148.11	140.92	127.93	147.18	76.59	91.60
Fer3-like bHLH transcription factor	Ferd3l	100758559	0.00	0.00	0.00	0.00	0.00	0.00	0.00	0.00	0.00	0.00	0.00	0.00
fermitin family member 1	Fermt1	100772109	0.00	0.00	0.00	0.00	0.00	0.00	0.00	0.00	0.00	0.00	0.00	0.00
fermitin family member 2	Fermt2	100762844	92.75	95.82	92.98	103.41	113.44	105.34	8730.14	9120.14	8909.49	9795.19	10752.47	10071.07
fermitin family member 3	Fermt3	100755790	0.01	0.00	0.02	0.02	0.00	0.02	4.56	0.00	1.24	3.48	1.03	0.93
feline sarcoma oncogene	Fes	100763153	0.58	0.59	0.79	0.93	1.60	1.36	54.69	53.99	94.40	83.44	156.28	136.46
fetuin B	Fetub	100764879	0.00	0.00	0.00	0.00	0.00	0.00	0.00	0.00	0.00	0.00	0.00	0.00
FEV (ETS oncogene family)	Fev	100750702	0.00	0.00	0.00	0.00	0.00	0.00	0.00	0.00	0.00	0.00	0.00	0.00
fasciculation and elongation protein zeta 1 (zygin I)	Fez1	100751680	0.00	0.00	0.00	0.00	0.00	0.00	0.00	0.00	0.00	0.00	0.00	0.00
fasciculation and elongation protein zeta 2 (zygin II)	Fez2	100765737	74.33	77.30	78.08	88.20	102.67	100.75	3954.16	4156.08	4183.35	4690.15	5471.97	5259.39
FEZ family zinc finger 1	Fezf1	100750867	0.00	0.00	0.00	0.00	0.00	0.00	0.00	0.00	0.00	0.00	0.00	0.00
FEZ family zinc finger 2	Fezf2	100762796	0.00	0.00	0.00	0.00	0.00	0.00	0.00	0.00	0.00	0.00	0.00	0.00
free fatty acid receptor 1	Ffar1	100752326	0.00	0.00	0.00	0.00	0.00	0.00	0.00	0.00	0.00	0.00	0.00	0.00
free fatty acid receptor 4	Ffar4	100752511	0.00	0.00	0.00	0.00	0.00	0.00	0.00	0.00	0.00	0.00	0.00	0.00
fibrinogen alpha chain	Fga	100770138	0.00	0.00	0.00	0.00	0.00	0.00	0.00	0.00	0.00	0.00	0.00	0.00
fibrinogen beta chain	Fgb	100770424	0.02	0.00	0.00	0.00	0.06	0.08	1.52	0.00	0.00	0.00	2.07	2.80
FYVE, RhoGEF and PH domain containing 1	Fgd1	100760862	8.37	8.61	10.45	9.45	7.69	7.80	952.46	938.82	1147.69	1045.35	822.81	861.77
FYVE, RhoGEF and PH domain containing 2	Fgd2	100754230	0.00	0.00	0.00	0.00	0.00	0.00	0.00	0.00	0.00	0.00	0.00	0.00
FYVE, RhoGEF and PH domain containing 3	Fgd3	100756063	3.38	3.39	3.73	3.51	2.76	3.49	448.89	439.22	495.59	464.73	360.18	450.51
FYVE, RhoGEF and PH domain containing 4	Fgd4	100756483	2.34	2.17	1.59	1.65	2.19	2.02	565.10	523.40	386.29	394.03	516.46	488.83
FYVE, RhoGEF and PH domain containing 5	Fgd5	100775006	0.29	0.25	0.18	0.16	0.11	0.16	60.00	53.07	43.47	31.29	19.66	30.84

FYVE, RhoGEF and PH domain containing 6	Fgd6	100756530	0.74	0.65	0.65	0.66	0.87	0.83	188.37	164.71	170.17	169.20	223.56	215.91
fibroblast growth factor 1 (acidic)	Fgf1	100764257	0.00	0.00	0.00	0.00	0.00	0.00	0.00	0.00	0.00	0.00	0.00	0.00
fibroblast growth factor 10	Fgf10	100751051	0.00	0.00	0.00	0.00	0.00	0.00	0.00	0.00	0.00	0.00	0.00	0.00
fibroblast growth factor 11	Fgf11	100759104	0.01	0.02	0.00	0.00	0.00	0.00	0.76	0.92	0.00	0.00	0.00	0.00
fibroblast growth factor 12	Fgf12	100770589	0.08	0.00	0.00	0.00	0.00	0.00	1.52	0.92	0.00	0.00	0.00	0.00
fibroblast growth factor 13	Fgf13	100756051	3.24	3.57	4.28	4.05	2.86	3.44	182.29	194.90	236.00	222.51	164.56	186.93
fibroblast growth factor 14	Fgf14	100762042	0.00	0.03	0.00	0.00	0.04	0.11	0.00	1.83	1.24	0.00	3.10	1.87
fibroblast growth factor 16	Fgf16	100767099	0.00	0.00	0.00	0.00	0.00	0.00	0.76	0.00	0.00	0.00	0.00	0.00
fibroblast growth factor 17	Fgf17	100764611	0.00	0.00	0.00	0.00	0.00	0.00	0.00	0.00	0.00	0.00	0.00	0.00
fibroblast growth factor 18	Fgf18	100761504	5.39	5.88	6.00	6.70	4.50	3.41	149.63	154.64	188.80	205.13	139.72	121.51
fibroblast growth factor 19	Fgf19	100758307	0.00	0.00	0.00	0.00	0.00	0.00	0.00	0.00	0.00	0.00	0.00	0.00
fibroblast growth factor 2 (basic)	Fgf2	100757429	0.05	0.06	0.00	0.00	0.07	0.00	0.00	0.00	0.00	0.00	2.07	0.93
fibroblast growth factor 20	Fgf20	100773578	0.00	0.00	0.00	0.00	0.00	0.00	0.00	0.00	0.00	0.00	0.00	0.00
fibroblast growth factor 21	Fgf21	100757339	0.04	0.21	0.07	0.07	0.00	0.00	0.76	4.58	1.24	1.16	2.07	0.00
fibroblast growth factor 22	Fgf22	103160353	0.56	0.62	0.92	0.75	0.59	0.43	31.14	25.62	47.20	28.97	28.98	22.43
fibroblast growth factor 23	Fgf23	100763837	0.00	0.00	0.00	0.00	0.00	0.00	0.00	0.00	0.00	0.00	0.00	0.00
fibroblast growth factor 3	Fgf3	100757734	0.00	0.00	0.00	0.00	0.00	0.00	0.00	0.00	0.00	0.00	0.00	0.00
fibroblast growth factor 4	Fgf4	100758023	0.00	0.00	0.00	0.00	0.00	0.00	0.00	0.00	0.00	0.00	0.00	0.00
fibroblast growth factor 5	Fgf5	100763237	0.00	0.00	0.00	0.00	0.00	0.00	0.00	0.00	0.00	0.00	0.00	0.00
fibroblast growth factor 6	Fgf6	100772115	0.00	0.00	0.00	0.00	0.00	0.00	0.00	0.00	0.00	0.00	0.00	0.00
fibroblast growth factor 7	Fgf7	100761599	0.31	0.37	0.11	0.14	0.01	0.00	29.62	35.69	9.94	12.75	1.03	0.00
fibroblast growth factor 8 (androgen-induced)	Fgf8	100750999	0.00	0.06	0.00	0.00	0.07	0.00	0.00	0.92	0.00	0.00	1.03	0.00
fibroblast growth factor 9	Fgf9	100759509	0.00	0.00	0.00	0.00	0.00	0.00	0.00	0.00	0.00	0.00	0.00	0.00
fibroblast growth factor binding protein 1	Fgfbp1	100762110	0.00	0.00	0.00	0.00	0.00	0.00	0.00	0.00	0.00	0.00	0.00	0.00
fibroblast growth factor binding protein 3	Fgfbp3	103163266	0.00	0.00	0.00	0.00	0.00	0.00	0.00	0.00	0.00	0.00	0.00	0.00
fibroblast growth factor receptor 1	Fgfr1	100773586	19.58	18.81	17.56	18.40	15.90	18.00	2551.29	2477.00	2316.49	2354.93	2032.71	2304.90
FGFR1 oncogene partner	Fgfr1op	100756864	2.93	2.85	2.44	2.73	3.66	3.47	292.42	278.17	258.35	271.19	358.11	337.42
FGFR1 oncogene partner 2	Fgfr1op 2	100756667	46.18	47.15	44.26	48.11	50.09	50.43	1452.24	1543.66	1439.58	1542.52	1624.93	1623.52
fibroblast growth factor receptor 2	Fgfr2	100763455	0.03	0.03	0.02	0.02	0.01	0.01	4.56	2.75	2.48	3.48	1.03	0.93
fibroblast growth factor receptor 3	Fgfr3	100764560	1.64	1.53	1.47	1.39	1.29	1.07	198.24	190.33	176.38	162.25	145.93	127.12
fibroblast growth factor receptor 4	Fgfr4	100765320	0.04	0.04	0.01	0.01	0.00	0.00	5.32	5.49	2.48	2.32	0.00	0.93
fibroblast growth factor receptor-like 1	Fgfrl1	100771678	3.87	3.97	4.70	3.83	2.90	3.41	858.28	879.35	1040.87	849.49	634.45	757.08
fibrinogen gamma chain	Fgg	100769847	0.00	0.03	0.00	0.00	0.00	0.00	1.52	0.92	0.00	0.00	0.00	0.00
FGGY carbohydrate kinase domain containing	Fggy	100763881	13.55	12.74	15.29	16.49	16.06	17.71	697.26	686.28	761.40	806.61	792.80	841.20
fibrinogen-like 1	Fgl1	100771647	0.00	0.00	0.00	0.00	0.00	0.00	0.00	0.00	0.00	0.00	0.00	0.00
fibrinogen-like 2	Fgl2	100760509	0.22	0.19	0.10	0.14	0.13	0.10	17.47	14.64	9.94	11.59	9.31	9.35
feline Gardner-Rasheed sarcoma viral oncogene homolog	Fgr	100758687	0.09	0.06	0.03	0.05	0.00	0.02	5.32	5.49	1.24	3.48	3.10	0.93
fumarate hydratase	Fh	100773218	76.73	80.31	81.90	80.95	76.31	80.38	3465.02	3658.30	3738.68	3618.15	3417.52	3602.22
forkhead-associated (FHA) phosphopeptide binding domain 1	Fhad1	100752360	0.05	0.04	0.02	0.03	0.02	0.04	6.08	5.49	6.21	5.79	2.07	5.61

FH2 domain containing 1	Fhdc1	100760893	1.49	1.65	1.84	1.70	1.79	1.82	176.97	198.56	221.09	199.33	220.45	220.58
fragile histidine triad	Fhit	100755382	0.00	0.00	0.00	0.00	0.00	0.00	0.00	0.00	0.00	0.00	0.00	0.00
four and a half LIM domains 1	Fhl1	100763662	36.46	36.02	28.53	30.49	31.23	34.61	2504.96	2505.36	2002.24	2087.21	2099.99	2342.28
four and a half LIM domains 2	Fhl2	100762607	113.65	101.98	72.15	70.27	72.81	80.99	4336.97	3939.22	2773.58	2575.12	2640.25	2959.16
four and a half LIM domains 3	Fhl3	100751268	21.14	18.70	22.13	17.28	22.99	23.12	871.95	780.52	907.97	702.31	937.70	957.10
four and a half LIM domains 5	Fhl5	100773098	0.00	0.00	0.00	0.00	0.00	0.00	0.00	0.00	0.00	0.00	0.00	0.00
formin homology 2 domain containing 1	Fhod1	100760903	4.03	3.93	3.57	3.12	2.26	2.87	504.33	493.20	444.67	395.19	283.59	357.98
formin homology 2 domain containing 3	Fhod3	100761354	0.07	0.01	0.06	0.03	0.03	0.03	9.11	0.92	8.69	3.48	5.17	5.61
fibrinogen C domain containing 1	Fibcd1	100767519	0.00	0.00	0.00	0.00	0.00	0.00	0.00	0.00	0.00	0.00	0.00	0.00
fin bud initiation factor homolog (zebrafish)	Fibin	100760017	0.42	0.28	0.15	0.07	0.11	0.19	20.51	15.56	8.69	4.64	5.17	9.35
fibroblast growth factor (acidic) intracellular binding protein	Fibp	100766825	44.30	42.46	96.10	91.10	86.06	89.27	1648.96	1571.11	3382.20	3132.56	2968.34	3055.44
FIC domain containing	Ficd	100760999	3.97	3.53	5.31	4.59	3.34	4.08	360.78	320.26	485.66	416.05	301.18	368.26
FIG4 homolog, SAC1 lipid phosphatase domain containing (S. cerevisiae)	Fig4	100772123	11.96	12.20	11.45	12.42	13.55	12.76	1135.51	1166.67	1081.86	1168.19	1269.93	1189.83
c-fos induced growth factor (vascular endothelial growth factor D)	Figf	100773421	0.27	0.18	0.21	0.11	0.10	0.12	19.75	13.73	17.39	8.11	10.35	8.41
folliculogenesis specific basic helix-loop-helix	Figla	100755024	0.00	0.00	0.00	0.00	0.00	0.00	0.00	0.00	0.00	0.00	0.00	0.00
fidgetin	Fign	100751083	0.99	0.85	1.20	0.95	0.97	1.10	110.13	91.50	134.15	101.98	105.57	115.90
fidgetin-like 1	Fignl1	100758940	7.68	7.97	8.35	8.57	10.70	9.39	665.36	677.12	715.44	740.55	894.23	810.36
filamin A interacting protein 1	Filip1	100755967	0.06	0.02	0.06	0.06	0.01	0.02	8.35	3.66	7.45	6.95	1.03	2.80
filamin A interacting protein 1-like	Filip1l	100755572	10.66	10.73	12.57	15.09	13.40	11.84	1003.35	1011.11	1202.34	1427.79	1262.68	1124.41
factor interacting with PAPOLA and CPSF1	Fip1l1	100756086	109.35	114.07	126.07	130.09	118.87	115.57	6456.84	6793.21	7445.07	7458.81	6872.31	6710.93
fission 1 (mitochondrial outer membrane) homolog (S. cerevisiae)	Fis1	100689455	55.56	58.65	57.14	57.63	53.18	60.57	923.60	971.77	911.69	865.71	697.58	783.25
fat storage-inducing transmembrane protein 1	Fitm1	100762028	0.00	0.00	0.00	0.06	0.00	0.05	0.76	0.00	0.00	0.00	0.00	0.93
fat storage-inducing transmembrane protein 2	Fitm2	100771223	22.14	21.97	18.79	19.50	21.17	21.22	2465.46	2453.20	2111.55	2178.77	2343.21	2381.54
FLT3-interacting zinc finger 1	Fiz1	100768644	1.57	1.59	1.28	0.24	0.30	0.19	41.77	43.92	37.26	12.75	13.45	8.41
four jointed box 1 (Drosophila)	Fjx1	100756899	1.97	2.30	0.55	0.26	0.00	0.00	58.48	62.22	16.15	6.95	0.00	0.00
FK506 binding protein 10, 65 kDa	Fkbp10	100771014	143.37	143.58	102.72	89.01	60.31	65.20	10425.44	10480.79	7527.05	6483.00	4404.90	4781.77
FK506 binding protein 11, 19 kDa	Fkbp11	100766921	138.65	128.80	155.50	143.78	140.92	147.36	2574.84	2421.18	2937.54	2502.11	2305.95	2460.05
FK506 binding protein 14, 22 kDa	Fkbp14	100758490	14.91	15.41	15.39	17.30	21.66	20.57	1394.51	1439.35	1455.73	1644.51	2018.22	1932.90
FK506 binding protein 15, 133kDa	Fkbp15	100756439	10.45	10.89	9.75	10.30	10.40	10.77	1359.58	1428.37	1273.14	1331.60	1380.67	1405.74
FK506 binding protein 1A, 12kDa	Fkbp1a	100763474	350.61	348.50	280.34	285.03	336.56	341.98	12756.46	12686.02	10252.18	10334.09	12025.51	12358.20
FK506 binding protein 1B, 12.6 kDa	Fkbp1b	100763278	0.21	0.18	0.10	0.05	0.00	0.15	6.08	7.32	3.73	1.16	0.00	4.67
FK506 binding protein 2, 13kDa	Fkbp2	100757160	51.51	50.89	56.17	50.30	41.24	45.04	753.46	738.43	806.11	696.51	582.70	621.56
FK506 binding protein 3, 25kDa	Fkbp3	100757543	199.28	215.71	198.78	197.50	224.44	209.02	4648.38	5078.44	4664.04	4563.83	5245.31	4772.42
FK506 binding protein 4, 59kDa	Fkbp4	100762395	208.30	209.76	228.93	214.49	179.19	193.57	11928.56	12061.97	13142.52	12096.81	10287.76	10981.43

FK506 binding protein 5	Fkbp5	100760904	23.41	24.26	27.11	26.89	22.94	22.28	1585.16	1652.55	1849.47	1828.78	1572.14	1554.36
FK506 binding protein 6, 36kDa	Fkbp6	100761445	0.03	0.07	0.05	0.04	0.15	0.07	0.76	2.75	4.97	4.64	4.14	3.74
FK506 binding protein 7	Fkbp7	100766686	8.05	7.65	8.20	8.56	9.57	9.37	322.04	314.77	329.15	330.29	385.01	361.72
FK506 binding protein 8, 38kDa	Fkbp8	100758154	119.54	114.72	131.36	80.47	42.68	51.86	6494.06	6224.97	7182.99	4363.33	2394.96	2881.59
FK506 binding protein 9, 63 kDa	Fkbp9	100768635	222.42	218.10	205.79	213.60	212.22	221.89	18686.94	18394.00	17455.05	17927.33	17744.84	18638.25
fukutin	Fktn	100761516	12.03	11.71	11.32	10.98	13.58	12.04	735.99	736.60	695.57	699.99	838.34	754.28
flavin adenine dinucleotide synthetase 1	Flad1	100774575	10.90	9.64	12.01	11.40	7.75	8.86	652.44	581.96	722.89	672.17	480.23	564.54
folliculin	Flcn	100760396	5.09	4.93	5.42	5.03	5.27	5.61	420.03	398.95	433.49	417.21	418.13	448.64
filaggrin family member 2	Flg2	100759445	0.00	0.00	0.00	0.00	0.00	0.00	0.00	0.00	0.00	0.00	0.00	0.00
Fli-1 proto-oncogene, ETS transcription factor	Fli1	100765706	0.00	0.00	0.00	0.00	0.00	0.00	0.00	0.00	0.00	0.00	0.00	0.00
flightless I homolog (Drosophila)	Flii	100756205	36.50	34.80	41.13	38.92	33.13	38.02	4333.93	4199.09	4950.96	4632.20	3965.03	4460.24
filamin A, alpha	Flna	100760050	151.20	150.04	131.17	131.47	117.35	126.70	36819.42	37093.63	32570.00	32370.95	28826.44	30888.03
filamin B, beta	Flnb	100769967	43.98	43.47	55.40	58.24	55.40	55.72	11674.12	11708.77	15037.95	15568.93	14647.13	14666.84
filamin C, gamma	Flnc	100751994	0.52	0.50	0.19	0.12	0.14	0.09	135.96	133.59	50.93	32.45	35.19	24.30
flotillin 1	Flot1	100763504	40.45	42.02	40.53	34.17	30.65	32.18	2063.67	2135.69	2068.07	1717.52	1581.46	1674.93
flotillin 2	Flot2	100766445	25.79	26.78	38.04	37.67	31.14	33.74	1971.00	2024.97	2891.58	2825.45	2322.51	2553.52
fibronectin leucine rich transmembrane protein 1	Flrt1	100755496	0.00	0.00	0.00	0.00	0.00	0.00	0.76	0.00	0.00	0.00	0.00	0.00
fibronectin leucine rich transmembrane protein 2	Flrt2	100751322	0.00	0.00	0.00	0.00	0.00	0.00	0.00	0.00	0.00	0.00	0.00	0.00
fibronectin leucine rich transmembrane protein 3	Flrt3	100771316	3.28	3.68	3.96	4.68	4.42	4.07	380.53	423.66	450.88	538.90	505.07	471.07
fms-related tyrosine kinase 1	Flt1	100761329	0.14	0.17	0.01	0.03	0.04	0.05	29.62	36.60	3.73	6.95	8.28	10.28
fms-related tyrosine kinase 3	Flt3	100761902	0.01	0.00	0.00	0.01	0.03	0.02	0.76	0.00	0.00	1.16	2.07	1.87
fms-related tyrosine kinase 3 ligand	Flt3lg	100773162	8.02	7.61	8.60	7.53	6.27	7.96	290.90	275.43	322.94	280.46	239.08	285.07
fms-related tyrosine kinase 4	Flt4	100766609	0.00	0.01	0.00	0.00	0.01	0.00	0.00	0.92	0.00	0.00	1.03	0.93
feline leukemia virus subgroup C cellular receptor 1	Flvcr1	100766724	3.89	3.88	5.06	5.05	5.01	5.23	450.41	456.60	580.05	569.03	568.21	633.71
feline leukemia virus subgroup C cellular receptor family, member 2	Flvcr2	100760195	0.25	0.28	0.22	0.23	0.06	0.18	26.58	29.28	23.60	24.34	8.28	18.69
FLYWCH-type zinc finger 1	Flywch1	100772983	23.95	24.54	22.26	18.49	15.70	16.86	2434.32	2490.72	2263.08	1885.56	1588.70	1730.07
formin 1	Fmn1	100751856	0.10	0.14	0.00	0.04	0.02	0.01	9.87	12.81	0.00	3.48	1.03	0.93
formin 2	Fmn2	100770806	0.00	0.00	0.00	0.00	0.00	0.00	0.00	0.00	0.00	0.00	0.00	0.00
formin-like 1	Fmnl1	100765726	0.06	0.18	0.07	0.03	0.07	0.02	8.35	17.39	7.45	3.48	6.21	1.87
formin-like 2	Fmnl2	100773842	6.60	6.91	6.53	7.78	8.04	7.34	1004.11	1055.03	982.49	1165.87	1195.41	1108.52
formin-like 3	Fmnl3	100765961	16.26	16.14	16.43	15.40	16.95	16.52	2113.80	2101.83	2132.66	1992.18	2175.54	2130.11
flavin containing monooxygenase 1	Fmo1	100750993	0.01	0.01	0.00	0.00	0.00	0.01	0.76	0.92	0.00	0.00	0.00	0.93
flavin containing monooxygenase 2 (non-functional)	Fmo2	100753345	0.43	0.45	0.08	0.06	0.12	0.29	37.98	45.75	6.21	6.95	10.35	22.43
flavin containing monooxygenase 4	Fmo4	100753048	1.84	2.11	2.47	2.84	2.10	2.44	110.89	127.19	145.32	157.61	126.27	152.35
flavin containing monooxygenase 5	Fmo5	100750494	2.05	2.22	1.94	2.22	2.46	2.61	237.74	255.29	224.82	261.92	277.38	297.22
fibromodulin	Fmod	100765932	0.03	0.03	0.04	0.17	0.15	0.03	0.76	2.75	2.48	8.11	4.14	2.80
fragile X mental retardation 1	Fmr1	100760305	14.96	14.84	13.71	15.40	17.00	15.45	1708.20	1725.75	1612.23	1774.31	1960.26	1808.59
fibronectin 1	Fn1	100751189	47.37	46.30	4.35	4.30	0.22	0.23	11588.29	11311.64	1073.16	1034.92	54.85	58.88
fructosamine 3 kinase	Fn3k	100751771	0.25	0.34	0.08	0.08	0.03	0.03	13.67	13.73	2.48	5.79	2.07	2.80
fructosamine 3 kinase related protein	Fn3krp	100752057	32.78	34.37	15.31	14.50	11.76	12.72	1864.67	1957.26	876.91	822.83	661.36	713.15

formin binding protein 1	Fnbp1	100759492	4.52	4.83	5.32	5.13	4.81	4.67	334.96	364.18	404.92	374.33	357.07	345.83
formin binding protein 1-like	Fnbp1l	100757842	10.61	9.80	10.59	13.50	13.94	12.88	1609.46	1506.15	1630.86	2060.56	2136.21	1964.68
formin binding protein 4	Fnbp4	100764816	19.70	19.89	20.63	23.68	24.18	22.15	2358.37	2371.77	2494.11	2842.83	2905.21	2711.48
fibronectin type III domain containing 1	Fndc1	100761672	0.00	0.00	0.00	0.00	0.00	0.01	0.76	0.00	0.00	0.00	0.00	1.87
fibronectin type III domain containing 3A	Fndc3a	100775061	8.92	9.13	8.18	9.64	9.94	9.34	1525.91	1572.94	1425.92	1637.55	1697.38	1602.96
fibronectin type III domain containing 3B	Fndc3b	100755546	12.08	11.85	14.00	16.11	14.02	13.17	2352.29	2345.23	2772.34	3132.56	2716.84	2576.88
fibronectin type III domain containing 4	Fndc4	100757660	2.22	2.22	1.98	1.91	1.34	1.77	121.53	120.78	106.82	99.67	72.45	93.47
fibronectin type III domain containing 5	Fndc5	100757300	0.00	0.00	0.00	0.00	0.00	0.00	0.76	0.92	1.24	1.16	0.00	0.00
fibronectin type III domain containing 7	Fndc7	100752797	0.12	0.03	0.08	0.04	0.00	0.09	7.60	0.92	4.97	1.16	0.00	3.74
fibronectin type III domain containing 8	Fndc8	100766468	0.07	0.04	0.00	0.00	0.00	0.00	3.80	0.92	0.00	2.32	2.07	0.93
fibronectin type III domain containing 9	Fndc9	100768078	0.00	0.00	0.00	0.00	0.00	0.00	0.00	0.00	0.00	0.00	0.00	0.00
folliculin interacting protein 1	Fnip1	100763470	5.22	5.29	5.95	6.62	7.39	7.34	856.00	861.96	981.25	1072.00	1196.44	1219.74
folliculin interacting protein 2	Fnip2	100753221	6.28	6.19	8.35	9.00	9.29	9.77	1339.83	1338.69	1813.45	1907.58	1955.09	2075.90
farnesyltransferase, CAAX box, alpha	Fnta	100752295	101.63	102.84	104.54	110.74	128.07	123.69	4038.47	4046.28	4122.49	4274.10	4996.91	4929.45
farnesyltransferase, CAAX box, beta	Fntb	100752221	11.88	11.03	12.01	10.96	9.32	9.71	912.97	872.94	943.99	851.81	729.67	767.36
folate receptor 1 (adult)	Folr1	100689330	0.15	0.00	0.00	0.05	0.00	0.04	3.80	0.00	0.00	1.16	0.00	0.93
folate receptor 4, delta (putative)	Folr4	100755390	0.00	0.00	0.00	0.00	0.00	0.00	0.00	0.00	0.00	0.00	0.00	0.00
FGFR1OP N-terminal like	Fopnl	100750977	25.94	25.52	25.17	25.83	30.69	30.15	1153.74	1124.58	1136.51	1146.17	1330.99	1317.88
FBJ murine osteosarcoma viral oncogene homolog	Fos	100689418	1.36	2.67	1.22	1.11	0.85	1.26	48.61	93.33	43.47	35.93	25.87	40.19
FBJ murine osteosarcoma viral oncogene homolog B	Fosb	100755767	0.62	0.60	0.63	0.48	0.27	0.29	28.10	38.43	39.75	31.29	23.80	29.91
FOS-like antigen 1	Fosl1	103159499	206.08	201.30	159.21	135.10	137.26	136.74	9737.29	9503.54	7482.33	6299.89	6381.73	6435.20
FOS-like antigen 2	Fosl2	100755919	13.80	13.24	10.89	8.82	6.73	7.73	2105.44	2019.48	1676.82	1346.66	1026.71	1187.03
forkhead box A1	Foxa1	100755899	0.28	0.19	0.39	0.29	0.29	0.41	22.03	14.64	32.29	22.02	22.77	31.78
forkhead box A2	Foxa2	100772660	0.00	0.00	0.00	0.00	0.00	0.00	0.00	0.00	0.00	0.00	0.00	0.00
forkhead box A3	Foxa3	100758663	0.03	0.00	0.00	0.00	0.00	0.00	2.28	0.00	0.00	0.00	2.07	0.93
forkhead box B1	Foxb1	100751108	0.00	0.00	0.00	0.00	0.00	0.00	0.00	0.00	0.00	0.00	0.00	0.00
forkhead box F1	Foxf1	100757169	9.71	10.40	11.71	9.72	13.18	13.43	908.41	965.36	1099.25	903.96	1216.11	1255.26
forkhead box F2	Foxf2	100760156	7.23	6.07	7.96	7.77	8.04	7.54	403.32	341.31	445.91	433.44	447.11	417.80
forkhead box G1	Foxg1	100762710	0.00	0.00	0.00	0.00	0.00	0.00	0.00	0.00	0.00	0.00	0.00	0.00
forkhead box H1	Foxh1	100760801	0.37	0.26	0.15	0.25	0.13	0.15	22.79	18.30	11.18	13.91	13.45	10.28
forkhead box I1	Foxi1	100767484	0.00	0.00	0.00	0.00	0.00	0.00	0.00	0.00	0.00	0.00	0.00	0.00
forkhead box I3	Foxi3	100765634	0.00	0.00	0.00	0.00	0.00	0.00	0.00	0.00	0.00	0.00	0.00	0.00
forkhead box J1	Foxj1	100755106	0.02	0.00	0.00	0.00	0.00	0.00	0.76	0.00	0.00	0.00	0.00	0.00
forkhead box J2	Foxj2	100770857	12.03	11.38	12.12	13.02	11.68	12.25	1038.29	988.24	1150.17	1172.83	1044.30	1103.84
forkhead box J3	Foxj3	100759136	39.53	39.71	37.75	38.27	36.33	38.01	5113.22	5170.85	4919.91	4981.04	4702.98	4913.56
forkhead box K1	Foxk1	100768479	8.72	7.97	6.96	6.49	4.65	5.33	920.56	850.07	740.28	690.72	498.86	564.54
forkhead box K2	Foxk2	100752578	34.35	33.29	13.29	14.49	8.99	9.15	2735.86	2692.03	1065.71	1161.24	742.09	730.91
forkhead box M1	Foxm1	100769341	28.50	28.10	28.24	29.70	27.66	28.37	2607.50	2569.41	2599.69	2706.08	2507.77	2579.69
forkhead box N2	Foxn2	100768700	18.33	18.27	17.07	19.52	21.40	20.36	2735.10	2730.46	2564.91	2930.91	3176.37	3047.96

forkhead box N3	Foxn3	100768709	9.03	9.25	11.77	11.51	12.09	12.12	1939.10	1992.03	2533.86	2473.14	2579.19	2603.06
forkhead box N4	Foxn4	100752823	0.00	0.00	0.00	0.00	0.00	0.00	0.00	0.00	0.00	0.00	0.00	0.00
forkhead box O1	Foxo1	100768647	12.44	12.67	13.31	15.49	15.35	15.15	1585.92	1615.95	1704.14	1979.44	1948.88	1939.44
forkhead box O3	Foxo3	100770315	13.97	13.30	14.14	13.21	10.71	10.96	1137.79	1048.63	1137.75	1096.34	893.19	923.45
forkhead box O4	Foxo4	100756577	14.51	14.27	14.34	12.38	10.74	11.98	1055.00	1066.93	1057.02	929.45	835.23	922.52
forkhead box O6	Foxo6	100758558	0.00	0.00	0.00	0.00	0.00	0.00	0.76	0.00	0.00	0.00	0.00	0.00
forkhead box P1	Foxp1	100753762	10.46	10.06	10.15	10.88	9.26	9.53	1286.66	1249.02	1278.11	1360.57	1190.23	1216.01
forkhead box P2	Foxp2	100762481	6.41	6.02	6.53	7.53	7.59	6.77	1217.54	1166.67	1265.69	1440.54	1442.77	1289.84
forkhead box P3	Foxp3	100757421	0.41	0.33	0.50	0.37	0.43	0.43	80.51	75.03	98.12	73.01	71.41	72.90
forkhead box P4	Foxp4	100769418	17.43	15.22	18.51	14.93	9.82	10.38	1548.70	1356.08	1675.58	1338.55	879.74	953.36
forkhead box R1	Foxr1	100755030	0.00	0.00	0.00	0.00	0.00	0.00	0.00	0.00	0.00	0.00	0.00	0.00
forkhead box R2	Foxr2	100764480	0.00	0.00	0.00	0.00	0.00	0.00	0.00	0.00	0.00	0.00	0.00	0.00
FAD-dependent oxidoreductase domain containing 1	Foxred1	100758274	9.27	9.28	12.93	12.77	12.37	12.61	630.42	635.03	884.37	870.35	847.65	877.65
FAD-dependent oxidoreductase domain containing 2	Foxred2	100769253	0.01	0.00	0.00	0.00	0.00	0.00	1.52	0.00	1.24	1.16	0.00	0.00
forkhead box S1	Foxs1	100755669	6.63	6.94	5.03	2.86	2.18	2.42	278.75	296.47	216.12	122.85	98.32	107.49
folylpolyglutamate synthase	Fpgs	100689022	30.57	30.43	32.09	26.82	17.93	19.74	1981.64	2007.58	2173.65	1789.37	1232.67	1329.10
fucose-1-phosphate guanylyltransferase	Fpgt	100769105	8.52	8.43	8.89	9.77	11.97	10.60	864.36	864.71	916.66	992.04	1198.51	1075.80
formyl peptide receptor 1	Fpr1	100758056	0.00	0.00	0.00	0.00	0.00	0.00	0.00	0.00	0.00	0.00	0.00	0.00
formyl peptide receptor 2	Fpr2	100758340	0.00	0.00	0.00	0.00	0.00	0.00	0.00	0.00	0.00	0.00	0.00	0.00
fragile site, folic acid type, rare, fra(10)(q23.3) or fra(10)(q24.2) candidate 1	Fra10ac1	100753320	24.80	23.78	22.46	24.25	25.44	24.94	1048.16	1063.27	989.94	1024.49	1103.30	1041.22
Fraser syndrome 1	Fras1	100753685	0.01	0.00	0.00	0.00	0.00	0.00	3.04	0.92	0.00	0.00	0.00	0.00
frequently rearranged in advanced T-cell lymphomas	Frat1	103160455	6.42	6.78	7.86	6.86	5.12	5.67	268.12	289.15	332.88	287.41	196.65	221.52
FRAS1 related extracellular matrix 1	Frem1	100764944	0.72	0.60	0.53	0.53	0.60	0.67	107.10	97.91	80.74	83.44	95.22	100.94
FRAS1 related extracellular matrix protein 2	Frem2	100754532	0.00	0.00	0.00	0.00	0.00	0.00	0.76	0.00	0.00	0.00	0.00	0.00
FRAS1 related extracellular matrix 3	Frem3	100754422	0.00	0.00	0.00	0.00	0.00	0.00	0.00	0.00	0.00	0.00	0.00	0.00
FSHD region gene 1	Frg1	100770784	73.54	79.70	86.13	78.03	80.77	78.83	2055.31	2232.68	2392.26	2105.76	2233.50	2143.20
fyn-related kinase	Frk	100751157	0.06	0.03	0.02	0.03	0.09	0.08	8.35	3.66	2.48	5.79	11.38	11.22
FERM domain containing 1	Frmd1	100750569	0.00	0.00	0.00	0.00	0.00	0.00	0.00	0.00	0.00	0.00	0.00	0.00
FERM domain containing 3	Frmd3	100770240	0.00	0.00	0.00	0.00	0.00	0.00	0.00	0.00	0.00	0.00	0.00	0.00
FERM domain containing 4A	Frmd4a	100750826	17.89	18.13	17.21	18.38	17.06	17.02	2463.95	2528.24	2414.62	2591.34	2386.68	2371.26
FERM domain containing 4B	Frmd4b	100755616	0.82	0.80	0.18	0.21	0.07	0.07	107.10	106.14	23.60	28.97	9.31	11.22
FERM domain containing 5	Frmd5	100752890	0.02	0.00	0.00	0.03	0.00	0.05	3.04	2.75	2.48	5.79	1.03	7.48
FERM domain containing 6	Frmd6	100759547	51.45	53.24	55.54	59.47	64.84	63.36	7091.06	7343.14	7727.02	8163.43	8836.71	8713.93
FERM domain containing 7	Frmd7	100762442	0.03	0.04	0.02	0.02	0.03	0.03	3.04	2.75	1.24	1.16	2.07	1.87
FERM and PDZ domain containing 1	Frmpd1	100753341	0.00	0.00	0.01	0.00	0.00	0.00	0.00	0.00	1.24	0.00	0.00	0.00
FERM and PDZ domain containing 3	Frmpd3	100760412	0.00	0.00	0.00	0.00	0.00	0.00	0.00	0.00	0.00	0.00	0.00	0.00
FERM and PDZ domain containing 4	Frmpd4	100768458	0.00	0.00	0.00	0.00	0.00	0.00	0.00	0.00	0.00	0.00	0.00	0.00
ferric-chelate reductase 1	Frrs1	100756307	2.63	2.66	1.97	2.22	2.09	1.94	289.38	275.43	211.15	217.88	222.52	204.69

ferric-chelate reductase 1-like	Frrs1l	100766877	0.00	0.00	0.00	0.00	0.00	0.00	0.00	0.00	0.00	0.00	0.00	0.00
fibroblast growth factor receptor substrate 2	Frs2	100758678	6.55	6.76	6.77	6.46	6.74	6.98	665.36	697.26	686.87	671.01	697.58	715.02
fibroblast growth factor receptor substrate 3	Frs3	100770465	2.38	2.20	2.12	1.88	0.76	0.96	148.87	143.66	134.15	127.48	60.03	76.64
furry homolog (Drosophila)	Fry	100759522	3.70	3.88	4.11	4.51	6.14	5.84	1208.43	1267.32	1346.42	1426.63	1958.19	1840.36
FRY-like	Fryl	100753564	4.58	4.30	4.52	5.10	5.74	5.57	1528.95	1464.05	1550.12	1725.63	1943.70	1882.42
frizzled-related protein	Frzb	100757779	0.00	0.03	0.00	0.00	0.03	0.06	0.00	0.92	0.00	0.00	1.03	1.87
fibrinogen silencer binding protein	Fsbp	103162160	2.43	2.43	2.28	2.58	2.80	2.58	319.01	316.60	310.52	343.04	368.46	343.96
fibrous sheath CABYR binding protein	Fscb	103161332	0.00	0.00	0.00	0.00	0.00	0.00	0.00	0.00	0.00	0.00	0.00	0.00
fascin actin-bundling protein 1	Fscn1	100758707	1.77	2.02	0.59	0.46	0.56	0.55	141.27	160.13	47.20	45.20	52.78	50.47
fascin actin-bundling protein 2, retinal	Fscn2	100764319	0.20	0.13	0.15	0.12	0.13	0.07	15.95	12.81	12.42	10.43	10.35	11.22
fascin actin-bundling protein 3, testicular	Fscn3	100773577	0.00	0.00	0.00	0.00	0.00	0.00	0.00	0.00	0.00	0.00	0.00	0.00
fibronectin type III and SPRY domain containing 1	Fsd1	100755663	7.21	7.20	7.79	5.98	4.49	4.98	366.86	379.74	403.68	310.59	240.12	268.25
fibronectin type III and SPRY domain containing 1-like	Fsd1l	100766469	1.21	1.13	1.10	1.53	1.74	1.69	265.84	255.29	252.14	345.36	386.05	387.89
fibronectin type III and SPRY domain containing 2	Fsd2	100769907	0.00	0.00	0.00	0.00	0.00	0.00	0.00	0.00	1.24	0.00	2.07	2.80
follicle stimulating hormone, beta polypeptide	Fshb	100689453	0.00	0.00	0.00	0.00	0.00	0.00	0.00	0.00	0.00	0.00	0.00	0.00
follicle stimulating hormone receptor	Fshr	100769953	0.00	0.00	0.00	0.00	0.00	0.00	0.00	0.00	0.00	0.00	0.00	0.00
fibrous sheath interacting protein 1	Fsip1	100761745	0.55	0.49	0.44	0.41	0.65	0.92	28.86	31.11	27.33	27.81	44.50	49.54
fibrous sheath interacting protein 2	Fsip2	100754113	0.00	0.00	0.00	0.00	0.00	0.00	0.00	0.00	0.00	0.00	0.00	0.00
follistatin	Fst	100774874	30.83	28.94	34.74	39.48	34.27	33.03	2216.34	2101.83	2480.45	2905.41	2478.79	2461.92
follistatin-like 1	Fstl1	100761843	103.55	101.35	86.26	95.06	76.77	75.64	10926.73	10797.40	9248.58	10084.92	8074.96	8001.71
follistatin-like 3 (secreted glycoprotein)	Fstl3	103160351	2.48	2.28	2.71	2.07	2.41	2.50	131.40	127.19	144.08	114.73	128.34	141.14
follistatin-like 4	Fstl4	100764925	0.00	0.00	0.00	0.00	0.00	0.01	0.00	0.00	0.00	0.00	0.00	0.93
follistatin-like 5	Fstl5	100752648	0.00	0.00	0.00	0.00	0.00	0.00	0.00	0.00	0.00	0.00	0.00	0.00
formimidoyltransferase cyclodeaminase	Ftcd	100754097	0.00	0.00	0.00	0.00	0.00	0.00	0.00	0.00	13.66	16.22	9.31	16.82
ferritin, heavy polypeptide 1	Fth1	100689102	584.64	571.08	702.41	699.80	646.37	669.91	10216.56	10134.91	12382.36	11910.22	10988.45	11195.47
ferritin, light polypeptide	Ftl	100753846	0.43	0.15	0.28	0.41	0.24	0.26	4.56	1.83	7.45	4.64	4.14	3.74
fat mass and obesity associated	Fto	100771560	36.62	38.61	52.14	51.55	46.99	53.61	1631.49	1751.37	2269.29	2279.60	2137.25	2374.06
FtsJ RNA methyltransferase homolog 1 (E. coli)	Ftsj1	100766807	53.79	50.96	63.61	62.42	52.99	58.28	3572.87	3469.81	4312.53	4128.07	3577.95	3879.81
FtsJ RNA methyltransferase homolog 2 (E. coli)	Ftsj2	100770491	10.82	9.92	12.47	9.94	10.36	11.15	503.57	463.92	576.33	462.41	474.02	521.55
FtsJ homolog 3 (E. coli)	Ftsj3	100771677	107.59	110.46	109.47	110.53	96.79	101.16	9937.05	10212.69	10072.08	10033.93	8817.05	9153.22
far upstream element (FUSE) binding protein 1	Fubp1	100761415	30.30	28.69	41.65	38.41	37.14	37.74	2356.09	2254.64	3243.09	2999.28	2902.10	2959.16
far upstream element (FUSE) binding protein 3	Fubp3	100761512	24.01	26.17	24.59	25.19	24.79	25.85	2233.80	2431.24	2342.58	2281.91	2228.33	2294.61
fucosidase, alpha-L- 1, tissue	Fuca1	100752441	105.60	102.28	131.20	134.87	169.86	173.83	5814.27	5692.42	7313.41	7407.82	9352.14	9470.07
fucosidase, alpha-L- 2, plasma	Fuca2	100756134	0.01	0.00	0.00	0.00	0.00	0.00	0.76	0.00	0.00	0.00	0.00	0.00
fucokinase	Fuk	100752219	9.73	9.54	10.69	8.79	7.07	8.14	1156.02	1138.30	1274.38	1065.05	852.83	999.16
FUN14 domain containing 1	Fundc1	100769595	33.99	34.24	33.17	34.20	37.45	38.29	1111.21	1145.62	1105.46	1102.13	1231.63	1232.83

FUN14 domain containing 2	Fundc2	100754819	53.26	51.33	38.00	43.21	46.96	47.52	1198.55	1174.90	896.79	968.86	1049.48	1040.29
fucose mutarotase	Fuom	100772971	0.27	0.28	0.50	0.18	0.53	0.24	6.84	9.15	14.91	5.79	15.52	12.15
furin (paired basic amino acid cleaving enzyme)	Furin	100682534	22.30	21.66	10.11	9.07	6.11	6.49	1572.25	1524.45	717.93	617.70	415.03	472.94
fused in sarcoma	Fus	100756868	284.17	285.43	260.27	255.40	225.96	223.64	14801.14	14918.71	13569.80	13064.50	11712.94	11536.63
fucosyltransferase 1 (galactoside 2-alpha-L-fucosyltransferase, H blood group)	Fut1	100757047	0.06	0.00	0.00	0.00	0.04	0.00	1.52	0.00	0.00	0.00	1.03	0.93
fucosyltransferase 10 (alpha (1,3) fucosyltransferase)	Fut10	100760260	0.03	0.02	0.00	0.02	0.02	0.09	1.52	0.92	0.00	1.16	1.03	7.48
fucosyltransferase 11 (alpha (1,3) fucosyltransferase)	Fut11	100758336	3.41	2.95	3.60	3.04	3.66	3.71	220.27	191.24	233.51	201.65	233.91	242.08
fucosyltransferase 2 (secretor status included)	Fut2	100751185	0.07	0.00	0.00	0.04	0.00	0.00	3.04	0.92	0.00	1.16	0.00	0.00
fucosyltransferase 4 (alpha (1,3) fucosyltransferase, myeloid-specific)	Fut4	100754814	0.00	0.00	0.00	0.00	0.00	0.00	0.00	0.00	0.00	0.00	0.00	0.00
alpha (1,3) fucosyltransferase 6A	Fut6a	100689084	0.01	0.03	0.08	0.00	0.00	0.03	0.76	0.00	2.48	0.00	0.00	0.93
alpha (1,3) fucosyltransferase 6B	Fut6b	100689083	0.01	0.02	0.03	0.00	0.00	0.00	0.00	0.00	1.24	0.00	0.00	0.00
fucosyltransferase 7 (alpha (1,3) fucosyltransferase)	Fut7	100772214	0.00	0.03	0.00	0.00	0.00	0.00	0.00	0.92	0.00	0.00	0.00	0.00
fucosyltransferase 8 (alpha (1,6) fucosyltransferase)	Fut8	100751648	11.07	11.72	9.95	10.28	11.90	11.19	893.22	949.80	807.36	817.04	952.19	901.02
fucosyltransferase 9 (alpha (1,3) fucosyltransferase)	Fut9	100689036	0.00	0.00	0.00	0.00	0.00	0.00	0.00	0.00	0.00	0.00	0.00	0.00
fuzzy planar cell polarity protein	Fuz	100772773	12.02	12.36	13.72	12.45	12.48	13.02	480.03	508.76	589.99	500.65	501.97	515.94
frataxin	Fxn	100755339	8.35	7.02	8.10	7.74	7.28	7.10	507.37	444.71	491.87	463.57	425.38	432.75
fragile X mental retardation, autosomal homolog 1	Fxr1	100689323	69.36	71.57	69.86	78.50	73.87	68.11	4720.54	4917.39	4785.76	5369.28	5020.72	4649.98
fragile X mental retardation, autosomal homolog 2	Fxr2	100757950	25.46	24.10	27.29	25.44	21.15	20.92	1948.98	1858.43	2116.52	1955.10	1638.38	1641.28
FXYD domain containing ion transport regulator 2	Fxyd2	100771061	0.00	0.00	0.00	0.00	0.00	0.00	0.00	0.00	0.00	0.00	0.00	0.00
FXYD domain containing ion transport regulator 4	Fxyd4	100773029	0.00	0.00	0.00	0.00	0.00	0.00	0.76	0.00	0.00	0.00	0.00	0.00
FXYD domain containing ion transport regulator 6	Fxyd6	100770777	0.00	0.00	0.00	0.00	0.00	0.00	0.00	0.00	0.00	0.00	0.00	0.00
FYN binding protein	Fyb	100774387	0.55	0.55	0.21	0.25	0.43	0.29	76.71	80.52	32.29	37.09	64.17	42.06
FYVE and coiled-coil domain containing 1	Fyco1	100760712	14.42	14.56	16.12	16.08	15.51	16.31	2957.65	3004.06	3316.37	3308.72	3185.69	3382.57
FYN oncogene related to SRC, FGR, YES	Fyn	100772355	52.31	51.82	62.04	65.14	59.29	61.00	4033.91	4038.04	4841.65	5044.78	4661.58	4759.34
forty-two-three domain containing 1	Fyttd1	100763912	17.40	16.78	15.32	17.68	18.60	17.58	1010.95	980.92	884.37	1033.76	1105.37	1024.40
frizzled class receptor 1	Fzd1	100770917	3.73	3.35	3.54	2.95	3.03	3.05	363.06	323.01	344.06	289.73	292.90	298.16
frizzled class receptor 10	Fzd10	100755785	0.00	0.00	0.00	0.00	0.00	0.00	0.00	0.00	0.00	0.00	0.00	0.00
frizzled class receptor 2	Fzd2	100763109	10.74	9.81	4.94	2.30	0.98	1.34	548.39	496.86	262.08	149.50	69.34	84.12
frizzled class receptor 3	Fzd3	100751712	2.91	2.91	3.27	3.44	3.40	3.49	262.04	254.38	299.34	310.59	307.39	310.31
frizzled class receptor 4	Fzd4	100756296	0.83	0.69	0.93	0.84	1.18	1.08	113.17	96.08	127.93	115.89	162.49	151.42
frizzled class receptor 5	Fzd5	100762351	1.38	1.01	1.08	0.76	0.41	0.50	109.37	74.12	88.19	60.26	45.54	48.60
frizzled class receptor 6	Fzd6	100774509	0.00	0.00	0.00	0.00	0.00	0.00	0.00	0.00	0.00	0.00	0.00	0.00
frizzled class receptor 7	Fzd7	100766658	14.47	14.40	13.46	12.51	11.65	12.00	1308.69	1330.46	1269.41	1185.58	1107.44	1175.81
frizzled class receptor 9	Fzd9	100761155	0.02	0.00	0.04	0.03	0.00	0.00	0.76	0.00	1.24	1.16	0.00	0.00

fizzy/cell division cycle 20 related 1 (Drosophila)	Fzr1	100762149	35.09	34.17	37.22	27.57	15.31	17.71	2106.20	2085.36	2258.11	1661.89	955.29	1062.72
G0/G1 switch 2	G0s2	100762788	0.00	0.00	0.00	0.00	0.00	0.00	0.00	0.00	0.00	0.00	0.00	0.00
G2/M-phase specific E3 ubiquitin protein ligase	G2e3	100763860	11.76	10.90	11.25	12.59	13.73	12.84	1197.03	1141.96	1183.71	1302.63	1405.51	1299.19
GTPase activating protein (SH3 domain) binding protein 1	G3bp1	100761148	82.58	83.52	80.95	74.76	69.69	73.45	6342.15	6483.01	6345.82	5854.86	5394.35	5720.18
GTPase activating protein (SH3 domain) binding protein 2	G3bp2	100752963	104.57	109.21	110.46	110.11	108.61	111.33	12177.69	12699.75	12877.96	12641.50	12384.65	12783.48
glucose-6-phosphatase, catalytic subunit	G6pc	100763214	0.00	0.00	0.00	0.00	0.00	0.00	0.00	0.00	0.00	0.00	0.00	0.00
glucose-6-phosphatase, catalytic, 2	G6pc2	100762184	0.09	0.00	0.00	0.04	0.00	0.00	0.76	0.00	0.00	1.16	0.00	0.00
glucose 6 phosphatase, catalytic, 3	G6pc3	100753219	48.80	48.48	39.15	36.54	34.10	38.29	1874.54	1867.58	1514.10	1387.23	1304.08	1484.26
glucose-6-phosphate dehydrogenase	G6pd	100689469	19.65	19.19	24.64	28.35	25.03	27.99	1440.09	1455.82	1874.31	2098.80	1864.01	2050.67
glucosidase, alpha; acid	Gaa	100755105	138.21	127.03	128.58	109.70	82.71	93.86	19611.30	18099.36	18364.25	15521.41	11704.66	13336.80
GRB2-associated binding protein 1	Gab1	100753832	2.62	2.39	3.17	3.39	3.17	3.26	309.13	282.75	373.87	410.26	368.46	387.89
GRB2-associated binding protein 2	Gab2	100771626	12.88	12.30	18.32	19.48	17.55	18.06	480.79	457.52	734.07	728.96	699.65	698.20
GRB2-associated binding protein 3	Gab3	100757434	1.41	0.84	1.89	3.12	3.11	2.20	46.33	44.84	32.29	49.83	48.64	57.01
GABA(A) receptor-associated protein	Gabara p	100767236	159.33	163.38	192.34	196.26	216.09	222.22	3621.48	3706.80	4363.45	4361.02	4853.05	4977.12
GABA(A) receptor-associated protein like 1	Gabara pl1	100771320	41.80	42.08	65.49	64.42	65.18	65.89	2053.03	2060.66	3221.97	3122.13	3139.11	3217.13
GABA(A) receptor-associated protein-like 2	Gabara pl2	100767407	64.24	68.74	63.71	58.32	71.11	75.25	1822.89	1951.77	1810.96	1637.55	1996.49	2109.55
gamma-aminobutyric acid (GABA) B receptor, 1	Gabbr1	100770643	3.37	3.69	3.81	4.51	3.86	3.99	421.54	447.45	475.72	530.79	486.44	469.20
gamma-aminobutyric acid (GABA) B receptor, 2	Gabbr2	100771574	0.00	0.03	0.00	0.00	0.00	0.00	0.00	1.83	0.00	1.16	0.00	0.00
GA binding protein transcription factor, alpha subunit 60kDa	Gabpa	100756649	35.28	33.19	31.12	35.85	35.55	34.59	2545.98	2455.03	2285.44	2594.82	2554.35	2526.41
GA binding protein transcription factor, beta subunit 1	Gabpb1	100753587	8.88	9.38	10.40	10.13	9.95	9.77	650.93	680.78	772.58	742.87	706.90	700.07
GA binding protein transcription factor, beta subunit 2	Gabpb2	100768222	15.65	15.63	17.15	19.63	19.16	21.78	1639.09	1605.88	1833.32	2028.11	2001.66	2218.91
gamma-aminobutyric acid (GABA) A receptor, alpha 1	Gabra1	100765947	0.00	0.00	0.21	0.43	0.50	0.34	0.00	0.00	99.37	127.48	153.18	157.02
gamma-aminobutyric acid (GABA) A receptor, alpha 2	Gabra2	100771532	0.00	0.00	0.00	0.00	0.00	0.00	0.00	0.00	1.24	0.00	0.00	0.93
gamma-aminobutyric acid (GABA) A receptor, alpha 3	Gabra3	100754835	0.00	0.00	0.04	0.00	0.00	0.00	0.00	0.00	1.24	0.00	0.00	0.00
gamma-aminobutyric acid (GABA) A receptor, alpha 4	Gabra4	100755113	0.00	0.00	0.00	0.00	0.00	0.00	0.00	0.00	0.00	0.00	0.00	0.00
gamma-aminobutyric acid (GABA) A receptor, alpha 5	Gabra5	100764074	0.00	0.00	0.00	0.00	0.00	0.00	0.00	0.00	0.00	0.00	0.00	0.00
gamma-aminobutyric acid (GABA) A receptor, alpha 6	Gabra6	100765459	0.00	0.00	0.00	0.00	0.00	0.00	0.00	0.00	0.00	0.00	0.00	0.00
gamma-aminobutyric acid (GABA) A receptor, beta 1	Gabrb1	100754246	0.00	0.00	0.00	0.00	0.00	0.00	0.00	0.00	0.00	0.00	0.00	0.00
gamma-aminobutyric acid (GABA) A receptor, beta 2	Gabrb2	100766244	0.00	0.00	0.00	0.09	0.04	0.04	0.00	0.00	0.00	2.32	1.03	0.93
gamma-aminobutyric acid (GABA) A receptor, delta	Gabrd	100756568	0.00	0.00	0.00	0.00	0.00	0.00	0.00	0.00	0.00	0.00	0.00	0.00

gamma-aminobutyric acid (GABA) A receptor, epsilon	Gabre	100755410	0.00	0.00	0.00	0.00	0.00	0.00	0.76	0.00	0.00	0.00	0.00	0.00
gamma-aminobutyric acid (GABA) A receptor, gamma 1	Gabrg1	100756681	0.00	0.00	0.00	0.00	0.00	0.00	0.00	0.92	0.00	0.00	0.00	0.00
gamma-aminobutyric acid (GABA) A receptor, gamma 2	Gabrg2	100768164	0.00	0.00	0.03	0.03	0.06	0.03	0.00	0.00	6.21	3.48	6.21	4.67
gamma-aminobutyric acid (GABA) A receptor, gamma 3	Gabrg3	100764373	0.00	0.00	0.04	0.00	0.00	0.00	0.00	0.00	1.24	0.00	0.00	0.00
gamma-aminobutyric acid (GABA) A receptor, pi	Gabrp	100766614	0.02	0.00	0.00	0.00	0.00	0.00	0.76	0.00	0.00	0.00	0.00	0.00
gamma-aminobutyric acid (GABA) A receptor, theta	Gabrq	100767917	0.01	0.00	0.00	0.00	0.02	0.00	0.76	0.92	1.24	0.00	1.03	0.00
gamma-aminobutyric acid (GABA) A receptor, rho 1	Gabrr1	100769153	0.07	0.07	0.02	0.00	0.02	0.00	5.32	3.66	1.24	0.00	1.03	0.00
gamma-aminobutyric acid (GABA) A receptor, rho 2	Gabrr2	100769438	0.00	0.00	0.00	0.00	0.00	0.00	0.00	0.00	0.00	0.00	0.00	0.00
gamma-aminobutyric acid (GABA) A receptor, rho 3	Gabrr3	100773638	0.15	0.08	0.00	0.02	0.04	0.04	10.63	4.58	1.24	1.16	3.10	3.74
glutamate decarboxylase 1 (brain, 67kDa)	Gad1	100765882	0.00	0.00	0.09	0.00	0.00	0.00	0.00	0.00	0.00	0.00	0.00	0.00
glutamate decarboxylase 2 (pancreatic islets and brain, 65kDa)	Gad2	100757642	3.43	3.44	3.68	3.89	3.72	3.40	497.50	503.27	537.82	566.71	540.26	499.11
growth arrest and DNA-damage-inducible, beta	Gadd45b	100766127	17.26	17.52	11.42	9.76	11.12	11.68	581.81	605.75	386.29	330.29	370.53	394.43
growth arrest and DNA-damage-inducible, gamma	Gadd45g	100759228	0.00	0.00	0.00	0.00	0.00	0.00	0.00	0.00	0.00	0.00	0.00	0.00
growth arrest and DNA-damage-inducible, gamma interacting protein 1	Gadd45gip1	100763960	45.20	39.75	45.40	32.98	17.63	18.68	804.35	727.45	833.44	603.80	361.21	386.95
glutamate decarboxylase-like 1	Gadl1	100752374	0.02	0.02	0.00	0.00	0.00	0.00	1.52	0.92	1.24	1.16	0.00	0.00
cyclin G associated kinase	Gak	100766883	37.29	38.74	41.30	39.21	37.53	38.91	4923.33	5163.53	5534.74	5278.88	5053.84	5268.73
galanin/GMAP prepropeptide	Gal	100760816	0.00	0.00	0.00	0.00	0.00	0.00	0.00	0.00	0.00	0.00	0.00	0.00
galactose-3-O-sulfotransferase 1	Gal3st1	100756367	0.00	0.00	0.00	0.00	0.00	0.00	0.00	0.00	0.00	0.00	0.00	0.00
galactose-3-O-sulfotransferase 2	Gal3st2	100755837	0.00	0.00	0.00	0.04	0.00	0.00	0.00	0.00	0.00	0.00	0.00	0.00
galactose-3-O-sulfotransferase 3	Gal3st3	100769025	0.23	0.43	0.03	0.12	0.18	0.14	11.39	20.13	4.97	11.59	12.42	7.48
galactose-3-O-sulfotransferase 4	Gal3st4	100771720	1.05	1.29	1.09	1.21	1.06	1.01	29.62	48.50	36.02	42.88	47.61	43.93
galactosylceramidase	Galc	100751905	9.57	10.29	2.92	2.94	0.98	0.73	799.04	853.73	239.72	243.37	82.80	62.62
galactokinase 1	Galk1	100757135	35.84	32.59	37.78	24.27	12.38	16.76	1439.33	1328.63	1529.01	993.19	516.46	674.83
galactokinase 2	Galk2	100752083	16.79	17.01	15.90	15.80	19.19	18.60	742.07	761.31	695.57	703.46	835.23	823.44
galactose mutarotase (aldose 1-epimerase)	Galm	100756945	8.30	8.72	7.46	7.50	7.19	7.77	606.11	634.12	546.52	544.69	528.88	562.67
galactosamine (N-acetyl)-6-sulfate sulfatase	Galns	100753973	12.14	12.25	15.92	16.38	17.07	17.28	840.81	872.94	1109.18	1133.42	1176.78	1200.12
polypeptide N-acetylgalactosaminyltransferase 1	Galnt1	100763868	25.61	25.79	22.61	23.96	25.69	24.07	2659.91	2692.94	2371.14	2502.11	2679.58	2530.15
polypeptide N-acetylgalactosaminyltransferase 10	Galnt10	100768523	8.31	7.62	9.20	9.58	8.00	8.89	1263.11	1167.58	1391.14	1454.44	1185.06	1334.71
:polypeptide N-acetylgalactosaminyltransferase 11	Galnt11	100758359	15.62	15.29	17.62	19.57	18.70	19.20	1091.46	1093.47	1252.02	1366.37	1317.54	1354.34

polypeptide N-acetylgalactosaminyltransferase 12	Galnt12	100772146	0.00	0.00	0.00	0.00	0.00	0.00	0.00	0.00	0.00	0.00	0.00	0.00
polypeptide N-acetylgalactosaminyltransferase 13	Galnt13	100768831	1.18	1.14	1.99	1.57	2.69	2.55	110.89	105.23	180.10	144.86	240.12	219.65
polypeptide N-acetylgalactosaminyltransferase 14	Galnt14	100759781	0.00	0.00	0.00	0.00	0.02	0.00	0.00	0.00	0.00	0.00	1.03	0.00
polypeptide N-acetylgalactosaminyltransferase 15	Galnt15	100766936	0.00	0.00	0.00	0.02	0.00	0.02	0.00	0.00	0.00	1.16	0.00	0.93
polypeptide N-acetylgalactosaminyltransferase 16	Galnt16	100764829	0.07	0.11	0.12	0.03	0.13	0.02	3.04	5.49	4.97	5.79	7.24	0.93
polypeptide N-acetylgalactosaminyltransferase 18	Galnt18	100767393	0.08	0.04	0.00	0.00	0.00	0.00	3.80	4.58	0.00	0.00	0.00	0.93
polypeptide N-acetylgalactosaminyltransferase 2	Galnt2	100767525	60.61	59.01	52.22	51.01	49.25	52.82	5938.08	5838.83	5287.56	5064.48	4854.09	5216.39
polypeptide N-acetylgalactosaminyltransferase 3	Galnt3	100753226	0.00	0.00	0.00	0.00	0.00	0.01	0.00	0.00	0.00	0.00	0.00	0.93
polypeptide N-acetylgalactosaminyltransferase 5	Galnt5	100757219	0.00	0.00	0.00	0.00	0.00	0.00	0.00	0.00	0.00	0.00	0.00	0.00
polypeptide N-acetylgalactosaminyltransferase 6	Galnt6	100751126	0.00	0.00	0.00	0.00	0.00	0.00	0.00	0.00	0.00	0.00	0.00	0.00
polypeptide N-acetylgalactosaminyltransferase 7	Galnt7	100762043	14.34	14.50	18.14	20.59	19.66	17.68	1610.22	1662.62	2071.80	2329.43	2235.57	1991.78
polypeptide N-acetylgalactosaminyltransferase 8	Galnt8	100764127	0.00	0.00	0.00	0.00	0.00	0.02	0.00	0.00	0.00	0.00	0.00	0.93
polypeptide N-acetylgalactosaminyltransferase 9	Galnt9	100773797	0.00	0.02	0.00	0.00	0.00	0.00	0.76	0.92	0.00	0.00	0.00	0.00
polypeptide N-acetylgalactosaminyltransferase-like 5	Galntl5	100767659	0.02	0.00	0.00	0.00	0.00	0.00	0.76	0.00	0.00	0.00	0.00	0.00
polypeptide N-acetylgalactosaminyltransferase-like 6	Galntl6	100761752	0.02	0.02	0.00	0.00	0.00	0.00	4.56	0.92	0.00	1.16	3.10	1.87
galanin-like peptide	Galp	100764710	0.29	0.26	0.12	0.23	0.40	0.09	6.84	2.75	4.97	1.16	6.21	2.80
galanin receptor 1	Galr1	100755896	0.04	0.00	0.00	0.03	0.05	0.02	1.52	0.00	0.00	1.16	2.07	0.93
galanin receptor 2	Galr2	100760610	0.02	0.05	0.00	0.07	0.03	0.00	1.52	1.83	0.00	2.32	3.10	0.00
galactose-1-phosphate uridylyltransferase	Galt	100752522	16.21	15.62	17.88	18.56	15.62	16.52	606.87	605.75	681.91	691.88	577.52	612.21
guanidinoacetate N-methyltransferase	Gamt	100755964	23.38	22.52	30.27	23.09	22.71	22.78	402.56	398.04	541.55	405.62	381.91	383.21
gigaxonin	Gan	100760056	3.47	3.55	3.05	3.18	3.28	3.44	458.76	467.58	413.61	423.01	432.62	447.71
glucosidase, alpha; neutral AB	Ganab	100752162	137.24	132.51	125.21	126.64	120.44	126.91	15156.61	14717.40	14004.53	14097.10	13491.05	14107.90
glucosidase, alpha; neutral C	Ganc	100770033	1.16	1.12	1.03	0.89	1.22	1.27	150.39	151.90	145.32	129.80	169.74	165.44
growth associated protein 43	Gap43	100751655	0.43	0.42	0.11	0.13	0.15	0.32	16.71	16.47	4.97	5.79	5.17	11.22
glyceraldehyde-3-phosphate dehydrogenase	Gapdh	100736557	3813.77	3775.77	3401.73	2869.64	2154.72	2442.17	118235.95	117003.51	105526.46	87909.87	66593.09	75783.95
glyceraldehyde-3-phosphate dehydrogenase, spermatogenic	Gapdhs	100767862	0.00	0.00	0.00	0.00	0.06	0.00	6.84	10.07	18.63	9.27	18.63	14.02
GRB2-binding adaptor protein, transmembrane	Gapt	100753774	0.00	0.00	0.00	0.00	0.00	0.00	0.00	0.00	0.00	0.00	0.00	0.00
GTPase activating protein and VPS9 domains 1	Gapvd1	100755209	32.66	32.12	39.68	43.11	43.41	44.17	6112.77	6061.18	7534.50	8047.54	8071.86	8241.92
GAR1 ribonucleoprotein	Gar1	103159924	67.61	68.33	67.38	66.01	70.68	69.43	132.92	124.44	125.45	125.16	130.41	114.03

GRB2 associated, regulator of MAPK1	Garem	100774206	1.26	1.13	1.41	1.70	1.17	1.17	85.07	79.61	106.82	119.37	83.83	75.71
GRB2 associated, regulator of MAPK1-like	Gareml	100771349	0.07	0.02	0.10	0.09	0.10	0.10	6.84	1.83	12.42	9.27	9.31	10.28
GTPase activating Rap/RanGAP domain-like 3	Garnl3	100762245	0.58	0.62	0.89	1.02	0.81	1.03	68.36	66.80	95.64	120.53	96.25	105.62
glycyl-tRNA synthetase	Gars	100751073	260.12	249.43	292.56	300.95	283.73	294.87	17110.90	16630.73	19538.03	19775.81	18566.62	19213.07
phosphoribosylglycinamide formyltransferase, phosphoribosylglycinamide synthetase, phosphoribosylaminoimidazole synthetase	Gart	100689446	99.48	97.91	99.28	100.49	93.68	100.03	9964.40	10037.92	10075.81	10016.54	9370.77	9910.30
growth arrest-specific 2	Gas2	100764664	7.66	7.60	7.94	8.56	8.63	9.90	306.85	290.07	310.52	330.29	383.98	387.89
growth arrest-specific 2 like 1	Gas2l1	100773328	18.57	16.75	17.60	10.79	11.40	10.82	1460.59	1319.48	1381.20	826.31	891.12	837.46
growth arrest-specific 2 like 2	Gas2l2	100774805	0.01	0.01	0.00	0.00	0.00	0.00	0.76	0.92	0.00	0.00	0.00	0.00
growth arrest-specific 2 like 3	Gas2l3	100764083	6.14	6.08	5.37	6.73	7.42	6.85	982.84	982.75	860.77	1082.43	1182.99	1101.98
growth arrest-specific 6	Gas6	100751777	0.00	0.00	0.00	0.00	0.00	0.00	0.00	0.00	0.00	0.00	0.00	0.00
growth arrest-specific 7	Gas7	100768686	0.00	0.00	0.00	0.00	0.00	0.00	0.00	0.00	0.00	0.00	0.00	0.00
growth arrest-specific 8	Gas8	100752777	2.82	3.01	3.19	3.57	3.68	3.73	179.25	194.90	214.88	227.15	243.22	235.54
gastrin	Gast	100772161	0.00	0.00	0.00	0.00	0.00	0.00	0.00	0.00	0.00	0.00	0.00	0.00
GATA binding protein 1 (globin transcription factor 1)	Gata1	100763974	0.02	0.00	0.00	0.00	0.00	0.00	1.52	0.00	1.24	0.00	0.00	0.00
GATA binding protein 2	Gata2	100762331	8.65	8.82	9.37	7.72	6.74	6.27	741.31	753.99	796.18	652.47	564.07	529.02
GATA binding protein 3	Gata3	100760238	3.58	3.34	2.91	2.18	1.27	1.45	293.94	276.34	252.14	186.59	108.67	120.57
GATA binding protein 4	Gata4	100756123	21.42	20.68	21.69	24.13	23.52	25.32	1355.78	1308.50	1383.68	1512.39	1471.75	1588.94
GATA binding protein 5	Gata5	100755855	0.00	0.00	0.00	0.00	0.00	0.00	0.00	0.00	1.24	0.00	0.00	0.00
GATA binding protein 6	Gata6	103161469	5.37	5.25	5.89	5.54	6.42	6.06	252.17	243.40	275.74	263.07	299.11	285.07
GATA zinc finger domain containing 1	Gatad1	100763066	113.70	123.27	131.82	139.28	144.48	134.55	1957.33	2120.13	2250.66	2268.01	2384.61	2241.34
GATA zinc finger domain containing 2A	Gatad2a	100757004	77.87	74.69	71.66	60.99	45.21	49.10	8026.81	7815.30	7539.47	6381.01	4634.67	5066.84
GATA zinc finger domain containing 2B	Gatad2b	100767845	28.19	28.45	28.87	30.72	33.54	33.15	4429.63	4497.39	4625.53	4831.54	5243.24	5248.17
glutamyl-tRNA(Gln) amidotransferase, subunit C	Gatc	100762845	24.36	22.17	27.47	21.97	20.38	20.62	545.35	542.61	639.67	513.40	488.51	490.70
glycine amidinotransferase (L-arginine:glycine amidinotransferase)	Gatm	100771783	0.41	0.57	0.08	0.08	0.12	0.05	28.86	40.26	6.21	6.95	8.28	3.74
GATS protein-like 2	Gatsl2	100768347	1.21	1.23	0.89	0.51	1.20	1.30	37.98	46.67	23.60	18.54	42.43	52.34
GATS protein-like 3	Gatsl3	100754150	4.72	4.64	5.61	5.28	3.03	4.13	265.84	253.46	316.73	271.19	184.23	216.84
glucosidase, beta, acid	Gba	100756635	40.80	38.93	53.76	53.42	53.48	58.16	2512.56	2401.96	3317.61	3253.09	3244.68	3561.09
glucosidase, beta (bile acid) 2	Gba2	100772646	2.48	2.77	2.96	2.53	2.01	2.17	241.53	265.36	283.20	239.90	195.61	209.37
glioblastoma amplified sequence	Gbas	100756369	19.28	20.40	20.30	20.15	21.20	20.39	1101.33	1183.14	1172.53	1149.65	1211.97	1159.93
glucan (1,4-alpha-), branching enzyme 1	Gbe1	100772279	4.79	4.55	4.44	4.86	5.80	5.74	387.37	377.91	373.87	397.51	466.78	450.51
golgi brefeldin A resistant guanine nucleotide exchange factor 1	Gbf1	100689421	25.64	25.62	25.62	26.40	25.48	25.80	4845.10	4814.91	4835.44	4816.47	4588.09	4601.38
globoside alpha-1,3-N-acetylgalactosaminyltransferase 1	Gbgt1	100771256	3.18	2.44	3.34	2.85	3.25	3.42	119.25	98.82	127.93	110.10	130.41	143.94
guanylate binding protein 5	Gbp5	100767428	0.08	0.02	0.00	0.00	0.00	0.04	5.32	2.75	0.00	0.00	0.00	1.87
gastrulation brain homeobox 1	Gbx1	100769092	0.00	0.00	0.00	0.00	0.00	0.00	0.00	0.00	0.00	0.00	0.00	0.00

gastrulation brain homeobox 2	Gbx2	100768672	0.00	0.00	0.00	0.00	0.00	0.00	0.00	0.00	0.00	0.00	0.00	0.00
group-specific component (vitamin D binding protein)	Gc	100763450	0.00	0.00	0.00	0.00	0.00	0.00	0.00	0.00	0.00	0.00	0.00	0.00
grancalcin, EF-hand calcium binding protein	Gca	100768073	0.00	0.00	0.00	0.00	0.00	0.00	0.00	0.00	0.00	0.00	0.00	0.00
glycine C-acetyltransferase	Gcat	100752849	0.00	0.02	0.00	0.00	0.00	0.00	0.00	1.83	0.00	0.00	0.00	0.00
GRIP and coiled-coil domain containing 1	Gcc1	100773292	11.77	11.68	12.07	12.79	12.04	11.86	1337.55	1332.29	1387.41	1454.44	1365.15	1355.27
GRIP and coiled-coil domain containing 2	Gcc2	100760881	7.42	6.58	6.82	8.70	9.17	8.82	1451.48	1270.98	1343.94	1680.43	1762.58	1699.23
glutaryl-CoA dehydrogenase	Gcdh	100757019	14.28	14.72	16.33	16.44	14.39	15.68	813.47	837.26	916.66	880.78	770.03	842.14
GC-rich sequence DNA-binding factor 2	Gcfc2	100766568	6.59	7.04	8.83	9.91	10.79	10.56	512.69	536.21	701.78	778.79	862.14	809.42
glucagon	Gcg	100767021	0.00	0.00	0.00	0.00	0.00	0.00	0.00	0.00	0.00	0.00	0.00	0.00
glucagon receptor	Gcgr	100766482	0.00	0.00	0.00	0.00	0.00	0.00	0.00	0.00	0.00	0.00	0.00	0.00
GTP cyclohydrolase 1	Gch1	100750667	17.92	19.34	16.83	18.99	23.01	23.06	271.92	283.66	259.60	246.85	305.32	312.18
GTP cyclohydrolase I feedback regulator	Gchfr	100755588	0.00	0.00	0.00	0.00	0.00	0.00	0.00	0.00	0.00	0.00	0.00	0.00
glucokinase (hexokinase 4)	Gck	100763110	0.00	0.00	0.00	0.00	0.00	0.00	0.00	0.00	0.00	0.00	0.00	0.00
glucokinase (hexokinase 4) regulator	Gckr	100763556	0.05	0.06	0.00	0.00	0.00	0.00	3.80	2.75	2.48	1.16	0.00	0.93
glutamate-cysteine ligase, catalytic subunit	Gclc	100752019	25.11	25.16	27.37	28.74	30.76	29.09	2232.29	2261.05	2444.43	2525.29	2695.10	2545.11
glutamate-cysteine ligase, modifier subunit	Gclm	100750530	44.36	47.49	44.10	50.21	57.56	56.25	2770.80	2987.58	2789.73	3153.42	3497.22	3454.54
glial cells missing homolog 1 (Drosophila)	Gcm1	100755308	0.00	0.00	0.00	0.00	0.00	0.00	0.00	0.00	0.00	0.00	2.07	0.93
glial cells missing homolog 2 (Drosophila)	Gcm2	100761954	0.00	0.00	0.00	0.00	0.00	0.00	0.00	0.00	0.00	0.00	0.00	0.00
GCN1 general control of amino-acid synthesis 1-like 1 (yeast)	Gcn1l1	100760442	24.00	22.76	22.54	20.73	16.37	18.54	6377.09	6139.87	6133.42	5597.58	4402.83	4952.81
glucosaminyl (N-acetyl) transferase 1, core 2	Gcnt1	100767124	0.00	0.00	0.00	0.00	0.00	0.06	0.76	0.00	0.00	0.00	0.00	1.87
glucosaminyl (N-acetyl) transferase 3, mucin type	Gcnt3	100774815	0.00	0.02	0.00	0.04	0.02	0.00	0.00	0.92	0.00	3.48	1.03	0.00
glucosaminyl (N-acetyl) transferase 4, core 2	Gcnt4	100757239	0.00	0.00	0.00	0.00	0.00	0.00	0.00	0.00	0.00	0.00	0.00	0.00
glucosaminyl (N-acetyl) transferase family member 7	Gcnt7	100760777	0.02	0.10	0.00	0.07	0.00	0.05	2.28	4.58	0.00	3.48	0.00	2.80
germinal center-associated, signaling and motility	Gcsam	100770981	0.00	0.00	0.00	0.00	0.00	0.00	0.00	0.00	0.00	0.00	0.00	0.00
glycine cleavage system protein H (aminomethyl carrier)	Gcsh	100765652	74.48	73.88	75.75	75.26	80.29	76.18	2451.03	2413.86	2489.14	2441.84	2589.54	2468.46
guanine deaminase	Gda	100760443	0.15	0.10	0.03	0.03	0.10	0.09	20.51	14.64	8.69	4.64	15.52	12.15
ganglioside induced differentiation associated protein 1	Gdap1	100765549	0.00	0.00	0.00	0.00	0.00	0.00	0.00	0.00	0.00	0.00	0.00	0.00
ganglioside induced differentiation associated protein 1-like 1	Gdap1l1	100772667	0.11	0.05	0.12	0.09	0.10	0.17	9.11	7.32	9.94	5.79	7.24	9.35
ganglioside induced differentiation associated protein 2	Gdap2	100771093	14.38	14.56	15.34	16.97	19.97	19.75	1029.94	1042.22	1126.57	1206.44	1412.76	1383.31
glycerophosphodiester phosphodiesterase 1	Gde1	100750723	21.75	20.79	19.28	23.17	25.65	26.78	921.32	889.41	833.44	982.76	1082.60	1136.56
growth differentiation factor 10	Gdf10	100758802	0.02	0.00	0.00	0.00	0.00	0.00	0.76	0.00	0.00	0.00	0.00	0.00
growth differentiation factor 11	Gdf11	100753230	6.49	6.36	8.06	7.42	5.53	5.82	665.36	653.33	830.96	760.25	562.00	598.19

growth differentiation factor 2	Gdf2	100772200	0.00	0.00	0.00	0.00	0.00	0.00	0.00	0.00	0.00	0.00	0.00	0.00
growth differentiation factor 3	Gdf3	100763238	0.00	0.00	0.00	0.00	0.00	0.00	0.00	0.00	0.00	0.00	0.00	0.00
growth differentiation factor 5	Gdf5	100762027	0.05	0.00	0.00	0.00	0.00	0.00	0.76	0.00	0.00	1.16	0.00	0.00
growth differentiation factor 7	Gdf7	100773313	0.00	0.00	0.00	0.00	0.00	0.00	0.00	0.00	0.00	0.00	0.00	0.00
growth differentiation factor 9	Gdf9	100756618	0.45	0.48	0.61	0.35	0.57	0.42	27.34	29.28	32.29	25.50	33.12	26.17
GDP dissociation inhibitor 1	Gdi1	100760927	30.41	29.86	33.45	35.59	40.54	41.53	1911.76	1892.29	2142.60	2239.03	2552.28	2613.34
GDP dissociation inhibitor 2	Gdi2	100765873	140.16	145.66	135.81	146.13	141.31	140.87	7813.38	8114.52	7565.55	8003.50	7660.97	7729.72
glial cell derived neurotrophic factor	Gdnf	100773138	0.00	0.02	0.01	0.00	0.00	0.00	0.00	1.83	1.24	0.00	0.00	0.00
glycerophosphodiester phosphodiesterase domain containing 1	Gdpd1	100758659	0.81	0.69	0.58	0.59	0.59	0.44	37.22	33.86	31.05	32.45	23.80	24.30
glycerophosphodiester phosphodiesterase domain containing 2	Gdpd2	100771225	0.02	0.00	0.00	0.00	0.00	0.00	0.76	0.00	0.00	0.00	1.03	0.00
glycerophosphodiester phosphodiesterase domain containing 3	Gdpd3	100753447	0.03	0.07	0.05	0.04	0.04	0.04	2.28	3.66	4.97	9.27	3.10	3.74
glycerophosphodiester phosphodiesterase domain containing 4	Gdpd4	100767792	0.00	0.00	0.00	0.02	0.00	0.00	0.00	0.00	0.00	1.16	0.00	0.00
glycerophosphodiester phosphodiesterase domain containing 5	Gdpd5	100774747	4.70	4.67	5.04	4.06	2.92	3.68	192.92	205.88	191.28	168.04	115.92	129.92
GDP-D-glucose phosphorylase 1	Gdpgp1	100772319	17.77	17.23	16.37	14.46	13.96	12.92	674.47	659.74	639.67	559.76	514.39	488.83
GTP binding protein overexpressed in skeletal muscle	Gem	100757029	4.61	4.27	3.23	2.99	3.01	3.38	251.41	230.59	175.13	159.93	156.28	182.26
gem (nuclear organelle) associated protein 2	Gemin2	100755559	41.88	40.84	41.05	40.41	45.27	45.65	1398.31	1429.28	1394.86	1387.23	1568.00	1532.86
gem (nuclear organelle) associated protein 4	Gemin4	100761184	21.83	20.11	21.24	22.49	21.86	23.63	2110.76	1954.51	2079.25	2188.04	2114.48	2317.05
gem (nuclear organelle) associated protein 5	Gemin5	100766595	16.49	16.10	17.10	16.49	14.14	14.23	2792.07	2730.46	2916.42	2816.18	2409.45	2460.05
gem (nuclear organelle) associated protein 6	Gemin6	100756163	25.47	23.59	23.95	23.31	25.09	24.56	732.20	689.02	689.36	669.86	737.95	705.68
gem (nuclear organelle) associated protein 7	Gemin7	100753426	3.40	3.23	3.34	2.94	2.74	3.10	135.20	125.36	132.90	115.89	106.60	124.31
gem (nuclear organelle) associated protein 8	Gemin8	100765564	16.91	18.06	15.53	15.38	14.62	16.02	667.63	711.90	594.96	585.25	575.45	617.82
GEN1 Holliday junction 5' flap endonuclease	Gen1	100754800	3.34	3.65	3.67	4.66	4.95	4.53	494.46	546.28	544.03	681.44	719.32	661.75
golgi to ER traffic protein 4 homolog (S. cerevisiae)	Get4	100769304	43.97	40.46	41.81	38.32	31.48	37.47	2357.61	2197.91	2314.01	2072.15	1722.22	2008.61
glial fibrillary acidic protein	Gfap	100758767	0.09	0.00	0.07	0.00	0.04	0.04	7.60	3.66	7.45	2.32	4.14	7.48
growth factor, augmenter of liver regeneration	Gfer	100767463	35.01	36.02	36.49	33.13	28.31	32.68	849.16	883.92	875.67	788.07	669.64	768.30
growth factor independent 1 transcription repressor	Gfi1	100769674	0.00	0.00	0.00	0.00	0.00	0.00	0.00	0.92	0.00	0.00	0.00	0.00
growth factor independent 1B transcription repressor	Gfi1b	100767332	0.03	0.04	0.00	0.00	0.00	0.00	1.52	2.75	0.00	0.00	0.00	0.00
G elongation factor, mitochondrial 1	Gfm1	100757634	62.00	62.35	72.38	75.79	78.38	77.67	4785.86	4903.66	5698.69	5835.16	5988.43	5885.62
G elongation factor, mitochondrial 2	Gfm2	100755195	12.36	11.91	13.66	15.45	15.78	16.28	1212.98	1197.78	1363.81	1523.98	1582.49	1606.70

glucose-fructose oxidoreductase domain containing 1	Gfod1	100767640	6.07	6.13	7.42	7.38	7.57	7.54	1002.59	1017.52	1218.49	1205.28	1221.28	1232.83
glucose-fructose oxidoreductase domain containing 2	Gfod2	100764685	5.41	5.10	5.35	4.90	4.35	4.31	432.18	409.02	432.25	405.62	352.93	347.70
glutamine-fructose-6-phosphate transaminase 2	Gfpt2	100767191	0.16	0.15	0.01	0.11	0.10	0.12	17.47	16.47	1.24	9.27	8.28	12.15
GDNF family receptor alpha 2	Gfra2	100763728	0.00	0.00	0.00	0.00	0.00	0.00	0.00	0.00	0.00	0.00	0.00	0.00
GDNF family receptor alpha 3	Gfra3	100752308	0.00	0.00	0.00	0.00	0.00	0.00	0.00	0.00	0.00	0.00	0.00	0.00
GDNF family receptor alpha like	Gfral	100751689	0.00	0.00	0.00	0.00	0.00	0.00	0.00	0.00	0.00	0.00	0.00	0.00
golgi-associated, olfactory signaling regulator	Gfy	100772084	0.00	0.00	0.00	0.00	0.00	0.05	0.00	0.00	0.00	0.00	0.00	1.87
golgi-associated, gamma adaptin ear containing, ARF binding protein 1	Gga1	100755306	18.98	18.15	19.58	15.32	11.52	13.26	1555.54	1485.10	1608.50	1236.57	921.14	1054.31
golgi-associated, gamma adaptin ear containing, ARF binding protein 3	Gga3	100756491	10.71	10.35	11.38	10.91	9.82	10.36	1222.10	1202.35	1312.89	1258.59	1135.38	1195.44
gamma-glutamylamine cyclotransferase	Ggact	100765817	14.18	16.01	14.01	12.58	12.14	11.93	776.25	872.03	750.22	675.65	637.55	642.12
gamma-glutamylcyclotransferase	Ggct	100774679	18.21	18.20	16.43	18.37	17.63	16.42	394.96	367.84	345.30	377.81	369.49	345.83
gamma-glutamyl carboxylase	Ggcx	100774482	5.75	5.78	7.11	6.53	6.72	6.80	804.35	796.99	998.64	917.86	945.98	956.17
gamma-glutamyl hydrolase (conjugase, folylpolygammaglutamyl hydrolase)	Ggh	100751581	16.08	18.10	14.97	17.94	19.74	18.79	663.08	752.16	630.98	727.80	805.22	781.38
gametogenetin binding protein 2	Ggnbp2	100759034	40.63	40.97	23.29	26.48	27.33	26.48	2992.58	3032.42	1743.89	1956.26	1986.14	1917.94
geranylgeranyl diphosphate synthase 1	Ggps1	100767405	18.83	19.11	22.69	22.55	26.42	23.17	997.27	1016.60	1187.44	1227.30	1374.46	1245.91
gamma-glutamyltransferase 1	Ggt1	100763802	0.77	0.68	0.75	0.50	0.41	0.37	54.69	47.58	50.93	33.61	28.98	28.04
gamma-glutamyltransferase 5	Ggt5	100764088	0.02	0.00	0.00	0.00	0.07	0.00	0.76	0.92	0.00	0.00	3.10	0.00
gamma-glutamyltransferase 6	Ggt6	100758668	0.14	0.14	0.05	0.10	0.07	0.04	8.35	9.15	3.73	4.64	4.14	4.67
gamma-glutamyltransferase 7	Ggt7	100752303	14.49	13.80	16.25	12.81	9.01	10.16	1146.14	1098.96	1280.59	1000.15	696.55	780.45
growth hormone 1	Gh1	100770534	0.00	0.00	0.00	0.00	33.27	38.01	0.00	0.00	0.00	1.16	584.77	701.94
GH3 domain containing	Ghdc	100754994	23.36	22.80	19.81	17.10	13.45	14.44	1599.59	1555.56	1366.30	1167.03	921.14	999.16
growth hormone inducible transmembrane protein	Ghitm	100753936	328.28	312.83	337.52	356.06	367.32	379.66	22176.27	21441.98	23120.20	24016.30	24765.15	25432.36
growth hormone receptor	Ghr	100763540	5.59	5.29	4.89	5.99	6.87	6.23	647.13	615.82	563.91	682.60	785.55	715.02
growth hormone releasing hormone	Ghrh	100756401	0.00	0.00	0.00	0.00	0.00	0.00	0.00	0.00	0.00	0.00	0.00	0.00
growth hormone releasing hormone receptor	Ghrhr	100772938	0.00	0.00	0.03	0.00	0.00	0.00	0.00	0.92	1.24	0.00	0.00	0.93
ghrelin/obestatin prepropeptide	Ghrl	100759101	0.08	0.10	0.32	0.28	0.31	0.14	4.56	8.24	22.36	24.34	19.66	15.89
growth hormone secretagogue receptor	Ghsr	100755255	0.00	0.00	0.00	0.00	0.00	0.00	0.00	0.00	0.00	0.00	0.00	0.00
GID complex subunit 4	Gid4	100755044	9.52	9.35	10.58	11.63	12.46	12.62	1036.77	1013.86	1150.17	1273.65	1351.69	1378.64
GID complex subunit 8	Gid8	100757886	45.80	47.01	47.49	47.81	52.11	52.75	1784.92	1865.75	1865.61	1863.54	2032.71	2062.82
gastric intrinsic factor (vitamin B synthesis)	Gif	100756309	0.00	0.00	0.00	0.00	0.00	0.00	0.00	0.00	0.00	0.00	0.00	0.00
GRB10 interacting GYF protein 1	Gigyf1	100753362	7.60	7.21	6.82	6.58	4.86	5.35	1446.16	1371.64	1314.13	1233.09	895.26	979.53
GRB10 interacting GYF protein 2	Gigyf2	100773834	17.66	17.57	14.82	15.63	15.31	15.88	2702.44	2746.02	2358.72	2460.39	2414.62	2480.61
GTPase, IMAP family member 4	Gimap4	100772834	0.00	0.00	0.00	0.00	0.00	0.00	0.00	0.00	0.00	0.00	0.00	0.00
GTPase, IMAP family member 6	Gimap6	100772540	0.18	0.26	0.00	0.11	0.05	0.00	5.32	7.32	0.00	2.32	1.03	0.00

GTPase, IMAP family member 7	Gimap7	100772245	0.00	0.00	0.00	0.00	0.00	0.00	0.00	0.00	0.00	0.00	0.00	0.00
GTPase, IMAP family member 8	Gimap8	100773700	0.00	0.00	0.00	0.00	0.00	0.00	0.00	0.92	0.00	0.00	0.00	0.00
GIMAP family P-loop NTPase domain containing 1	Gimd1	100760074	0.00	0.00	0.00	0.00	0.00	0.00	0.00	0.00	0.00	0.00	0.00	0.00
gypsy retrotransposon integrase 1	Gin1	100769872	15.38	15.45	13.03	14.45	16.25	15.92	957.02	951.63	840.89	913.23	1016.36	1001.03
glycoprotein integral membrane 1	Ginm1	100762603	17.13	16.75	17.95	21.52	21.86	22.23	757.26	778.69	776.30	985.08	986.34	970.19
GINS complex subunit 1 (Psf1 homolog)	Gins1	100768908	12.91	12.41	14.01	13.91	14.95	14.97	480.03	462.09	509.26	487.91	520.60	517.81
GINS complex subunit 2 (Psf2 homolog)	Gins2	100758038	14.13	14.05	16.02	15.95	15.58	16.50	794.48	753.99	873.19	800.81	746.23	790.73
GINS complex subunit 3 (Psf3 homolog)	Gins3	100760912	20.75	21.14	27.84	28.76	32.61	31.43	1127.92	1152.03	1510.38	1543.68	1741.88	1705.77
GINS complex subunit 4 (Sld5 homolog)	Gins4	100752225	19.55	21.24	22.60	22.75	26.32	26.05	514.21	527.06	581.30	534.26	637.55	653.33
gastric inhibitory polypeptide	Gip	100753817	0.00	0.09	0.00	0.00	0.00	0.00	1.52	2.75	1.24	0.00	1.03	0.93
GIPC PDZ domain containing family, member 1	Gipc1	100753328	21.72	21.99	26.54	20.03	18.16	17.72	1803.15	1825.49	2225.82	1665.37	1517.29	1492.67
GIPC PDZ domain containing family, member 2	Gipc2	100761124	0.04	0.05	0.00	0.00	0.06	0.00	0.76	2.75	0.00	0.00	1.03	0.00
GIPC PDZ domain containing family, member 3	Gipc3	100762933	0.00	0.00	0.00	0.00	0.00	0.00	0.00	0.00	0.00	0.00	0.00	0.00
gastric inhibitory polypeptide receptor	Gipr	100756938	0.00	0.00	0.00	0.00	0.00	0.00	0.00	0.00	1.24	1.16	0.00	0.00
G protein-coupled receptor kinase interacting ArfGAP 1	Git1	100758194	41.60	40.49	49.67	36.97	21.86	23.44	4191.90	4066.41	5019.27	3732.88	2195.21	2393.69
G protein-coupled receptor kinase interacting ArfGAP 2	Git2	100756931	19.15	18.86	19.94	22.39	21.76	22.52	1752.26	1736.73	1868.10	2025.79	2023.40	2051.60
gap junction protein, alpha 1, 43kDa	Gja1	100689077	398.07	399.92	177.27	172.04	166.42	177.92	25554.70	26169.96	11679.34	11626.28	11787.46	12469.43
gap junction protein, alpha 10, 62kDa	Gja10	100770016	0.00	0.00	0.00	0.00	0.00	0.00	0.00	0.00	0.00	0.00	0.00	0.00
gap junction protein, alpha 3, 46kDa	Gja3	100762422	0.00	0.03	0.00	0.00	0.00	0.00	0.00	0.92	0.00	0.00	0.00	0.00
gap junction protein, alpha 4, 37kDa	Gja4	100689037	0.02	0.00	0.00	0.00	0.00	0.00	0.76	0.92	0.00	0.00	0.00	0.00
gap junction protein, alpha 5, 40kDa	Gja5	100751822	0.00	0.00	0.00	0.04	0.00	0.00	0.00	0.00	0.00	1.16	1.03	0.00
gap junction protein, alpha 8, 50kDa	Gja8	100752105	0.05	0.18	0.08	0.05	0.02	0.04	3.04	10.07	3.73	2.32	1.03	1.87
gap junction protein, beta 1, 32kDa	Gjb1	100754733	0.00	0.00	0.00	0.00	0.00	0.00	0.00	0.00	0.00	0.00	0.00	0.00
gap junction protein, beta 2, 26kDa	Gjb2	100762125	0.00	0.00	0.00	0.00	0.00	0.00	0.00	0.00	0.00	0.00	0.00	0.00
gap junction protein, beta 3, 31kDa	Gjb3	100765420	32.58	33.13	23.96	20.69	21.54	23.31	1759.85	1794.38	1304.19	1114.88	1152.97	1260.87
gap junction protein, beta 4, 30.3kDa	Gjb4	100765131	5.59	5.65	3.15	3.12	2.75	3.26	296.98	299.22	172.65	173.84	153.18	171.98
gap junction protein, beta 5, 31.1kDa	Gjb5	100764845	50.39	50.57	39.59	38.02	37.29	39.74	1724.91	1706.54	1329.03	1241.20	1265.79	1365.55
gap junction protein, beta 6, 30kDa	Gjb6	100689286	0.00	0.00	0.00	0.00	0.00	0.00	0.00	0.00	0.00	0.00	0.00	0.00
gap junction protein, gamma 1, 45kDa	Gjc1	100689357	0.00	0.00	0.00	0.00	0.00	0.00	0.00	0.00	0.00	0.00	0.00	0.00
gap junction protein, gamma 3, 30.2kDa	Gjc3	100772879	0.00	0.00	0.00	0.00	0.00	0.00	0.00	0.00	0.00	0.00	0.00	0.00
gap junction protein, delta 2, 36kDa	Gjd2	100766680	0.00	0.00	0.00	0.00	0.00	0.00	0.00	0.00	0.00	0.00	0.00	0.00
gap junction protein, delta 4, 40.1kDa	Gjd4	100772140	0.00	0.00	0.05	0.00	0.00	0.00	0.00	0.00	1.24	0.00	0.00	0.00
glycerol kinase	Gk	100753140	0.02	0.03	0.01	0.01	0.02	0.03	1.52	2.75	1.24	1.16	4.14	2.80
glycerol kinase 2	Gk2	100770084	0.00	0.00	0.00	0.00	0.00	0.00	0.00	0.00	0.00	0.00	0.00	0.00
glycerol kinase 5 (putative)	Gk5	100765959	0.36	0.34	0.21	0.29	0.27	0.41	56.21	50.33	36.02	41.72	38.29	57.95
G kinase anchoring protein 1	Gkap1	100762171	11.59	12.22	18.01	19.38	19.94	20.09	540.03	586.54	839.65	913.23	924.24	924.39

gastrokine 1	Gkn1	100769103	0.00	0.00	0.00	0.00	0.00	0.00	0.00	0.00	0.00	0.00	0.00	0.00
galactosidase, alpha	Gla	100752709	24.17	22.02	20.26	21.91	24.69	23.94	1077.79	1019.35	916.66	960.74	1098.12	1056.18
galactosidase, beta 1	Glb1	100767446	30.65	29.50	30.23	29.48	27.45	28.19	2007.46	1967.32	1991.07	1921.49	1785.35	1845.97
galactosidase, beta 1-like	Glb1l	100768265	2.63	2.47	2.68	3.60	3.54	3.59	186.85	184.84	201.22	253.80	279.45	263.58
galactosidase, beta 1-like 2	Glb1l2	100757754	1.27	1.21	0.72	0.74	1.31	1.30	189.13	196.73	104.34	121.69	198.72	202.82
galactosidase, beta 1-like 3	Glb1l3	100757469	0.00	0.00	0.00	0.00	0.00	0.00	0.76	0.00	0.00	0.00	0.00	0.00
glucocorticoid induced transcript 1	Glcci1	100760004	2.25	2.10	2.19	2.36	2.67	2.94	249.13	243.40	245.93	265.39	290.83	323.40
glucuronic acid epimerase	Glce	100754874	16.48	17.42	16.90	16.75	18.64	18.55	1550.22	1651.64	1594.84	1561.07	1741.88	1770.26
glycine dehydrogenase (decarboxylating)	Gldc	100762555	1.11	1.31	0.18	0.52	1.10	1.18	87.35	96.99	14.91	37.09	85.90	93.47
gliomedin	Gldn	100752888	0.00	0.00	0.00	0.00	0.00	0.00	0.00	0.00	0.00	0.00	0.00	0.00
GLE1 RNA export mediator	Gle1	100767037	31.47	31.88	39.02	37.38	35.76	41.02	2037.08	2102.75	2551.25	2430.25	2361.84	2638.57
golgi glycoprotein 1	Glg1	100689240	43.06	43.13	46.74	48.94	51.91	52.15	5183.10	5267.85	5747.14	5846.75	6189.22	6185.64
GLI family zinc finger 1	Gli1	100763229	3.75	3.51	2.68	2.42	1.63	2.03	489.14	464.84	357.72	324.50	228.73	271.05
GLI family zinc finger 2	Gli2	100757572	2.98	2.96	2.42	2.04	1.48	1.68	556.74	560.92	449.64	381.28	267.03	307.51
GLI family zinc finger 3	Gli3	100762738	8.32	8.18	8.75	9.92	9.67	9.07	1741.62	1712.94	1835.80	2069.83	1935.42	1887.10
GLI family zinc finger 4	Gli4	100758024	2.07	1.89	2.09	1.57	0.99	1.24	95.70	88.76	95.64	73.01	44.50	60.75
GLI pathogenesis-related 1	Glipr1	100760583	17.19	16.06	15.45	17.71	19.48	17.54	713.21	688.11	635.95	762.57	795.90	736.52
GLI pathogenesis-related 2	Glipr2	100758797	23.14	23.88	24.74	22.69	24.57	25.82	1147.66	1184.05	1213.52	1102.13	1184.02	1262.74
GLIS family zinc finger 1	Glis1	100771925	3.10	2.72	2.71	2.22	1.15	1.31	230.14	200.39	192.52	166.88	84.87	100.01
GLIS family zinc finger 2	Glis2	100750786	20.28	20.02	17.72	17.64	15.28	16.66	2115.32	2100.92	1863.13	1829.93	1578.35	1710.45
GLIS family zinc finger 3	Glis3	100760016	3.71	3.67	6.68	7.05	4.97	5.11	751.94	757.65	1346.42	1421.99	992.55	1029.07
glomulin, FKBP associated protein	Glmn	100762721	13.00	13.22	12.86	14.83	15.76	13.65	739.79	767.71	758.92	870.35	907.68	790.73
glyoxalase I	Glo1	100764266	91.53	96.56	97.07	99.02	104.11	104.53	4893.71	5212.95	5254.03	5259.18	5533.04	5534.18
glyoxalase domain containing 4	Glod4	100761474	47.09	46.43	47.82	51.96	60.78	57.92	1573.01	1572.94	1624.65	1667.69	2023.40	1912.33
glyoxalase domain containing 5	Glod5	100768535	0.00	0.00	0.00	0.00	0.00	0.00	0.00	0.00	0.00	2.32	0.00	0.00
glucagon-like peptide 1 receptor	Glp1r	100763400	0.00	0.00	0.00	0.00	0.00	0.00	0.00	0.00	0.00	0.00	0.00	0.00
glucagon-like peptide 2 receptor	Glp2r	100765783	0.00	0.00	0.00	0.00	0.00	0.00	0.00	0.00	0.00	0.00	0.00	0.00
glycine receptor, alpha 1	Glra1	100761438	0.00	0.00	0.00	0.00	0.00	0.00	0.00	0.00	0.00	0.00	0.00	0.00
glycine receptor, alpha 2	Glra2	100751068	0.00	0.01	0.00	0.00	0.01	0.00	0.00	0.92	0.00	0.00	1.03	0.00
glycine receptor, alpha 3	Glra3	100758207	0.00	0.00	0.00	0.00	0.00	0.00	0.00	0.00	0.00	0.00	0.00	0.00
glycine receptor, alpha 4	Glra4	100766637	0.00	0.00	0.00	0.00	0.00	0.00	0.76	0.00	0.00	0.00	0.00	0.00
glycine receptor, beta	Glrb	100764841	0.02	0.02	0.02	0.00	0.00	0.00	2.28	1.83	1.24	0.00	0.00	0.00
glutaredoxin (thioltransferase)	Glrx	100757688	34.01	32.26	30.77	33.44	32.16	30.68	1200.07	1122.75	1073.16	1158.92	1108.47	1065.52
glutaredoxin 2	Glrx2	100766426	77.99	77.83	80.75	92.01	91.66	96.07	771.69	781.44	783.76	851.81	867.32	882.33
glutaredoxin 3	Glrx3	100754656	138.67	139.46	134.91	140.99	137.70	134.08	5671.48	5653.08	5466.42	5484.01	5428.50	5324.81
glutaredoxin 5	Glrx5	100768850	115.77	115.13	128.55	123.74	114.83	117.98	1979.36	1965.49	2194.77	2060.56	1933.35	1984.30
glutaminase	Gls	100689202	23.56	23.52	26.89	29.17	28.85	29.48	3283.49	3282.23	3725.02	4072.44	3969.17	4097.59
glutaminase 2 (liver, mitochondrial)	Gls2	100774486	2.08	2.40	2.35	2.67	2.46	2.71	157.22	186.67	175.13	199.33	174.91	196.28
glycosyltransferase 1 domain containing 1	Glt1d1	100761757	0.06	0.00	0.00	0.00	0.04	0.08	1.52	0.00	0.00	1.16	1.03	1.87
glycosyltransferase 8 domain containing 1	Glt8d1	100762757	26.82	26.60	26.99	29.98	35.80	35.94	1314.00	1305.75	1325.31	1453.29	1742.92	1725.40
glycosyltransferase 8 domain containing 2	Glt8d2	100772458	1.82	1.80	1.13	1.40	0.42	0.50	95.70	90.59	60.86	74.17	32.08	32.71
glycolipid transfer protein	Gltp	100757510	17.31	17.10	15.45	15.85	16.58	17.25	856.00	829.94	763.88	763.73	796.94	832.79

glycolipid transfer protein domain containing 1	Gltpd1	100769709	6.87	6.90	5.91	5.73	6.05	6.25	424.58	433.73	366.42	361.58	382.94	393.50
glycolipid transfer protein domain containing 2	Gltpd2	100752283	0.00	0.00	0.00	0.00	0.00	0.00	0.00	0.00	0.00	0.00	1.03	0.00
glioma tumor suppressor candidate region gene 1	Gltscr1	100764487	1.44	1.36	1.33	1.04	0.60	0.76	107.85	95.16	96.88	76.49	47.61	57.01
GLTSCR1-like	Gltscr1l	100768726	6.63	7.12	7.20	7.65	7.29	7.20	1216.78	1305.75	1341.45	1390.70	1337.20	1324.43
glioma tumor suppressor candidate region gene 2	Gltscr2	100763895	30.27	31.86	29.42	22.16	15.88	16.96	1255.52	1336.86	1275.62	888.89	656.18	698.20
glutamate dehydrogenase 1	Glud1	100759948	92.15	98.47	120.97	131.78	156.99	158.79	6607.23	7083.27	8741.81	9331.62	11068.14	11219.77
glutamate-ammonia ligase	Glul	100689337	0.11	0.08	0.20	0.06	0.13	0.00						
glutamate-ammonia ligase	Glul	100764163	13.23	13.22	14.12	13.02	13.34	13.20	1163.61	1165.75	1248.30	1126.47	1135.38	1130.02
glycine-N-acyltransferase	Glyat	100767729	0.23	0.23	0.46	0.40	0.42	0.60	24.31	24.71	48.44	41.72	45.54	59.82
glycine-N-acyltransferase-like 3	Glyatl3	100764695	0.15	0.11	0.12	0.17	0.10	0.14	7.60	5.49	4.97	8.11	6.21	10.28
glycerate kinase	Glyctk	100766079	0.78	0.75	0.65	0.45	0.38	0.34	91.14	87.84	77.01	57.95	49.68	42.06
glyoxylate reductase 1 homolog (Arabidopsis)	Glyr1	100771020	24.42	23.17	27.40	26.27	26.31	27.98	2521.67	2421.18	2853.07	2730.42	2701.31	2878.78
GM2 ganglioside activator	Gm2a	100759507	16.69	17.59	12.22	10.66	12.29	11.88	853.72	891.24	634.71	535.42	626.17	605.67
germ cell-less, spermatogenesis associated 1	Gmcl1	100774214	15.83	16.14	17.41	18.85	21.60	21.98	1386.16	1395.43	1525.28	1644.51	1836.07	1861.86
GDP-mannose 4,6-dehydratase	Gmds	100689436	13.35	13.42	14.72	16.57	14.56	14.97	446.61	455.69	478.20	520.36	455.39	471.07
glucocorticoid modulatory element binding protein 1	Gmeb1	100769076	6.68	6.38	7.02	7.14	6.76	6.98	761.06	741.18	822.26	818.20	771.06	797.27
glucocorticoid modulatory element binding protein 2	Gmeb2	100761456	19.75	19.84	18.33	17.78	17.27	18.49	2311.28	2379.09	2187.31	2108.08	2076.18	2209.56
glia maturation factor, beta	Gmfb	100754009	67.34	67.12	61.97	69.55	72.17	66.59	8233.41	8191.38	7595.36	8449.68	8698.02	8124.15
glia maturation factor, gamma	Gmfg	100760695	6.40	6.41	5.49	5.42	5.46	5.61	190.64	193.07	165.20	157.61	163.53	162.63
GEM interacting protein	Gmip	100758734	5.15	4.83	4.46	4.10	3.24	3.60	578.01	547.19	532.86	480.95	405.71	413.12
geminin coiled-coil domain containing	Gmnc	100750918	0.00	0.00	0.00	0.00	0.00	0.00	0.00	0.00	0.00	0.00	0.00	0.00
geminin, DNA replication inhibitor	Gmnn	100689393	48.38	50.33	48.52	56.09	62.84	59.28	1242.61	1256.34	1233.39	1359.41	1425.18	1335.64
GDP-mannose pyrophosphorylase A	Gmppa	100765276	8.82	8.23	9.26	8.45	7.61	8.86	489.14	475.82	520.43	482.11	423.31	498.18
GDP-mannose pyrophosphorylase B	Gmppb	100760573	9.72	10.55	9.71	9.10	7.91	8.19	561.30	593.86	553.97	522.67	471.95	486.96
guanosine monophosphate reductase	Gmpr	100765304	1.31	1.28	0.90	0.85	0.88	0.96	344.07	330.33	222.33	220.19	228.73	247.69
guanosine monophosphate reductase 2	Gmpr2	100758075	27.35	26.12	23.05	25.61	28.64	29.39	1120.32	1115.43	1016.03	1142.70	1290.63	1239.37
guanine monphosphate synthase	Gmps	100756126	21.46	22.00	26.23	29.54	29.48	27.84	5193.73	5409.68	6421.59	7143.58	7102.08	6751.12
guanine nucleotide binding protein (G protein), alpha 11 (Gq class)	Gna11	100689065	45.68	46.00	53.34	49.68	42.95	45.64	1203.87	1192.29	1347.66	1241.20	1036.02	1118.80
guanine nucleotide binding protein (G protein) alpha 12	Gna12	100760944	60.02	57.52	53.52	52.40	45.06	48.96	5341.08	5143.40	4806.88	4676.24	4001.26	4391.08
guanine nucleotide binding protein (G protein), alpha 13	Gna13	100689071	8.87	11.45	11.14	12.87	12.19	12.73	235.46	293.73	270.77	326.82	289.80	287.88
guanine nucleotide binding protein (G protein), alpha 14	Gna14	100762675	0.02	0.05	0.00	0.00	0.03	0.00	3.04	1.83	3.73	1.16	3.10	0.93
guanine nucleotide binding protein (G protein), alpha 15 (Gq class)	Gna15	100761285	0.20	0.21	0.28	0.13	0.08	0.04	9.87	5.49	9.94	8.11	2.07	1.87
guanine nucleotide binding protein (G protein), alpha inhibiting activity polypeptide 1	Gnai1	100689070	8.48	9.05	9.42	9.59	8.64	9.06	259.76	276.34	275.74	292.05	269.10	283.20

guanine nucleotide binding protein (G protein), alpha inhibiting activity polypeptide 2	Gnai2	100689068	147.24	143.85	163.01	154.98	122.45	133.51	4649.14	4598.96	5203.10	4607.87	3562.42	3828.41
guanine nucleotide binding protein (G protein), alpha inhibiting activity polypeptide 3	Gnai3	100689072	61.05	58.72	53.76	58.90	65.81	61.94	5329.69	5352.95	4921.15	5334.51	6023.62	5758.50
guanine nucleotide binding protein (G protein), alpha activating activity polypeptide, olfactory type	Gnal	100760304	0.08	0.03	0.09	0.07	0.07	0.06	14.43	10.98	18.63	15.07	12.42	14.95
guanine nucleotide binding protein (G protein), alpha activating activity polypeptide O	Gnao1	100689069	3.82	4.23	2.48	3.34	3.17	3.39	109.37	126.27	77.01	91.55	102.46	96.27
guanine nucleotide binding protein, alpha q polypeptide	Gnaq	100689066	48.64	47.89	47.45	55.97	52.43	55.09	590.16	584.71	571.36	653.63	635.48	646.79
GNAS complex locus	Gnas	100689067	632.49	621.17	613.17	554.18	481.27	491.63	25372.41	25394.01	25034.26	22102.92	19205.21	19481.32
guanine nucleotide binding protein (G protein), alpha transducing activity polypeptide 1	Gnat1	100689190	2.06	2.14	1.09	1.56	1.13	1.38	186.85	190.33	109.30	139.07	96.25	120.57
guanine nucleotide binding protein (G protein), alpha transducing activity polypeptide 2	Gnat2	100772380	0.69	0.64	0.26	0.27	0.35	0.46	29.62	32.94	11.18	12.75	21.73	24.30
guanine nucleotide binding protein, alpha transducing 3	Gnat3	100764767	0.06	0.05	0.07	0.03	0.00	0.00	2.28	1.83	2.48	3.48	1.03	0.93
guanine nucleotide binding protein (G protein), alpha z polypeptide	Gnaz	100767381	0.00	0.00	0.00	0.00	0.00	0.00	0.00	0.00	0.00	0.00	0.00	0.00
guanine nucleotide binding protein (G protein), beta polypeptide 1	Gnb1	100689442	507.97	490.09	511.27	549.63	541.09	570.90	20712.63	20504.07	21299.31	22413.51	22314.30	23074.19
guanine nucleotide binding protein (G protein), beta polypeptide 1-like	Gnb1l	100773508	7.26	6.82	7.63	6.58	5.44	5.54	285.59	268.10	329.15	273.51	218.38	220.58
guanine nucleotide binding protein (G protein), beta polypeptide 2	Gnb2	100756281	146.23	135.44	149.90	119.64	92.67	108.03	10676.08	9927.20	10975.08	8690.74	6768.81	7833.47
guanine nucleotide binding protein (G protein), beta polypeptide 2-like 1	Gnb2l1	100767670	729.83	678.74	870.44	742.89	679.56	855.30	17998.04	16814.66	21362.65	17906.47	16657.07	20732.84
guanine nucleotide binding protein (G protein), beta polypeptide 3	Gnb3	100756790	1.64	1.58	2.07	1.92	2.07	2.47	190.64	181.18	228.54	224.83	194.58	228.99
guanine nucleotide binding protein (G protein), beta polypeptide 4	Gnb4	100757050	34.22	35.84	34.69	36.79	39.42	38.91	3213.61	3383.79	3261.72	3450.10	3624.52	3594.74
guanine nucleotide binding protein (G protein), beta 5	Gnb5	100764899	13.17	13.06	13.15	13.67	12.63	13.97	807.39	789.67	809.84	811.24	752.44	840.27
glucosamine (UDP-N-acetyl)-2-epimerase/N-acetylmannosamine kinase	Gne	100689450	3.90	3.14	3.47	3.43	3.84	3.52	293.94	242.48	267.05	258.44	293.94	258.90
guanine nucleotide binding protein (G protein), gamma 10	Gng10	100755995	75.27	75.72	75.18	78.47	92.25	91.26	1844.92	1840.13	1840.77	1864.70	2109.30	2112.35
guanine nucleotide binding protein (G protein), gamma 11	Gng11	100761038	36.58	37.27	49.71	52.87	61.28	62.05	612.19	619.48	813.57	830.95	965.64	983.27
guanine nucleotide binding protein (G protein), gamma 12	Gng12	100757536	59.83	63.67	63.77	68.69	78.03	76.89	6933.83	7359.61	7428.92	7906.15	8905.02	8880.30
guanine nucleotide binding protein (G protein), gamma 13	Gng13	100774382	0.00	0.00	0.20	0.00	0.00	0.00	2.28	2.75	2.48	2.32	3.10	2.80
guanine nucleotide binding protein (G protein), gamma 2	Gng2	100768753	0.00	0.00	0.00	0.00	0.00	0.00	0.00	0.00	0.00	0.00	0.00	0.00
guanine nucleotide binding protein (G protein), gamma 3	Gng3	100755432	0.70	0.85	0.61	0.84	0.69	0.57	16.71	16.47	17.39	16.22	19.66	15.89

guanine nucleotide binding protein (G protein), gamma 4	Gng4	100767992	0.00	0.07	0.00	0.00	0.00	0.00	0.00	0.92	0.00	0.00	0.00	0.00
guanine nucleotide binding protein (G protein), gamma 7	Gng7	100765832	0.00	0.00	0.00	0.00	0.00	0.00	0.00	0.00	0.00	0.00	0.00	0.00
guanine nucleotide binding protein (G protein), gamma transducing activity polypeptide 1	Gngt1	100760746	0.00	0.00	0.00	0.00	0.00	0.00	0.00	0.00	1.24	0.00	0.00	0.00
guanine nucleotide binding protein (G protein), gamma transducing activity polypeptide 2	Gngt2	100754408	0.00	0.00	0.00	0.00	0.00	0.00	0.00	0.92	0.00	0.00	1.03	0.00
guanine nucleotide binding protein-like 1	Gnl1	100757330	44.29	43.76	50.35	39.65	27.65	33.04	2450.27	2410.20	2726.38	2109.23	1520.39	1824.48
guanine nucleotide binding protein-like 2 (nucleolar)	Gnl2	100753926	75.18	78.56	72.45	80.05	73.44	72.22	5122.33	5336.48	4893.82	5353.05	4861.33	4857.48
guanine nucleotide binding protein-like 3 (nucleolar)	Gnl3	100762471	121.96	125.28	131.53	140.13	131.86	128.60	7394.11	7723.80	8095.92	8560.94	8240.56	7760.56
guanine nucleotide binding protein-like 3 (nucleolar)-like	Gnl3l	100755751	38.39	37.56	46.18	49.20	46.79	47.84	5165.63	5111.38	6334.64	6622.07	6276.16	6446.42
glycine N-methyltransferase	Gnmt	100769585	0.10	0.06	0.17	0.16	0.21	0.19	12.15	6.41	18.63	13.91	19.66	21.50
glyceronephosphate O-acyltransferase	Gnpat	100765024	15.92	16.25	16.25	18.82	18.70	18.60	1206.91	1246.28	1265.69	1456.76	1423.11	1412.29
glucosamine-6-phosphate deaminase 1	Gnpda1	100689210	18.61	18.39	17.89	18.81	20.55	20.51	1231.97	1228.89	1204.82	1256.27	1353.76	1378.64
glucosamine-6-phosphate deaminase 2	Gnpda2	100761133	16.54	16.49	13.61	15.68	17.52	17.20	788.40	777.78	644.64	720.85	825.92	805.69
glucosamine-phosphate N-acetyltransferase 1	Gnpnat1	100761974	26.92	25.83	21.79	23.32	23.73	24.80	2078.86	2047.85	1722.77	1833.41	1820.54	1918.88
N-acetylglucosamine-1-phosphate transferase, alpha and beta subunits	Gnptab	100774953	26.76	27.64	26.83	32.33	36.00	33.25	4108.35	4269.55	4152.30	4955.54	5498.88	5116.38
N-acetylglucosamine-1-phosphate transferase, gamma subunit	Gnptg	100772246	28.22	26.31	31.49	32.36	30.47	33.47	977.53	938.82	1116.64	1092.86	1031.88	1136.56
gonadotropin-releasing hormone 1 (luteinizing-releasing hormone)	Gnrh1	100774729	0.16	0.09	0.05	0.11	0.06	0.11	10.63	6.41	6.21	6.95	6.21	10.28
gonadotropin-releasing hormone receptor	Gnrhr	100758518	0.00	0.00	0.00	0.00	0.00	0.00	0.76	0.00	2.48	0.00	1.03	2.80
glucosamine (N-acetyl)-6-sulfatase	Gns	100770659	17.18	18.02	19.88	20.59	20.05	19.33	1911.00	2024.97	2209.67	2257.58	2214.87	2129.18
golgin A1	Golga1	100751002	6.30	6.00	10.01	10.76	11.88	10.61	754.98	735.69	1202.34	1273.65	1402.41	1255.26
golgin A2	Golga2	100754167	10.10	9.86	8.56	8.32	8.17	8.02	1394.51	1377.13	1212.28	1187.89	1142.62	1159.93
golgin A3	Golga3	100771774	18.50	18.16	17.68	19.91	20.13	20.73	4593.69	4514.78	4477.72	4934.68	4983.46	5102.36
golgin A4	Golga4	100754180	13.90	13.28	14.13	16.13	15.82	14.09	2926.50	2828.37	2938.78	3377.09	3277.80	2975.99
golgin A5	Golga5	100751878	18.47	18.46	16.68	20.45	22.39	21.02	1424.90	1431.11	1335.24	1583.08	1738.78	1616.04
golgin A7	Golga7	100752519	62.49	65.48	64.67	68.76	75.10	69.46	3408.05	3599.74	3567.27	3763.01	4092.34	3836.82
golgin A7 family, member B	Golga7b	100767242	0.15	0.04	0.12	0.00	0.05	0.05	3.80	1.83	2.48	1.16	1.03	0.93
golgin B1	Golgb1	100764942	11.39	11.08	11.57	13.63	15.07	14.92	3342.73	3308.76	3416.98	4062.01	4453.55	4416.31
golgi integral membrane protein 4	Golim4	100758666	30.72	31.55	32.15	36.82	38.63	37.37	3390.58	3499.09	3571.00	4059.70	4223.78	4100.40
golgi membrane protein 1	Golm1	100772389	15.82	15.59	14.60	15.06	16.09	15.26	1341.35	1294.77	1197.37	1277.13	1358.94	1333.77
golgi phosphoprotein 3 (coat-protein)	Golph3	100750982	53.89	52.38	54.49	57.55	58.13	58.62	3477.93	3360.00	3537.46	3695.80	3723.88	3725.59
golgi phosphoprotein 3-like	Golph3l	100764832	15.36	15.53	16.03	17.84	18.38	18.72	1230.45	1238.96	1276.87	1427.79	1454.16	1486.12
golgi transport 1A	Golt1a	100768539	0.00	0.00	0.00	0.00	0.00	0.00	0.00	0.00	0.00	0.00	0.00	0.00
golgi transport 1B	Golt1b	100773711	37.19	38.39	36.52	37.39	41.68	40.04	3201.46	3329.81	3203.34	3226.43	3583.12	3438.65

golgi-associated PDZ and coiled-coil motif containing	Gopc	100761187	14.21	14.99	8.13	8.77	9.93	9.33	1805.42	1904.18	1030.93	1112.56	1249.23	1166.47
golgin, RAB6-interacting	Gorab	100751867	31.13	31.73	39.57	42.96	42.61	42.51	1922.39	1948.11	2450.64	2641.18	2644.39	2616.14
golgi reassembly stacking protein 1, 65kDa	Gorasp1	100755976	36.79	35.81	35.08	32.11	27.63	30.38	2437.36	2385.49	2346.30	2086.06	1778.11	1931.96
golgi reassembly stacking protein 2, 55kDa	Gorasp2	100773471	53.62	49.99	57.32	55.88	55.58	53.23	3168.04	2987.58	3397.11	3285.54	3265.38	3139.56
golgi SNAP receptor complex member 1	Gosr1	100689312	26.45	27.00	25.22	26.17	28.59	29.29	2072.02	2091.77	1978.64	2057.08	2245.92	2333.87
glutamic-oxaloacetic transaminase 1, soluble	Got1	100762297	24.34	23.95	27.48	28.52	26.62	27.97	1354.26	1357.91	1556.33	1601.63	1497.63	1552.49
glutamic-oxaloacetic transaminase 1-like 1	Got1l1	100750547	0.00	0.00	0.00	0.00	0.00	0.00	0.00	0.00	0.00	0.00	0.00	0.00
glutamic-oxaloacetic transaminase 2, mitochondrial	Got2	100762838	78.66	77.27	78.41	76.30	76.65	79.00	5293.99	5245.89	5348.43	5144.45	5130.43	5313.60
glycoprotein Ib (platelet), alpha polypeptide	Gp1ba	100770316	0.04	0.02	0.00	0.02	0.02	0.03	3.04	0.92	0.00	2.32	2.07	1.87
glycoprotein 2 (zymogen granule membrane)	Gp2	100767123	0.00	0.00	0.00	0.00	0.00	0.00	0.00	0.00	0.00	0.00	0.00	0.00
glycoprotein V (platelet)	Gp5	100770950	0.00	0.00	0.00	0.00	0.00	0.00	0.00	0.00	0.00	0.00	0.00	0.00
glycoprotein VI (platelet)	Gp6	100766737	0.00	0.00	0.00	0.00	0.00	0.00	0.00	0.00	0.00	0.00	0.00	0.00
glycoprotein IX (platelet)	Gp9	100767667	0.00	0.09	0.00	0.00	0.00	0.00	0.00	0.92	0.00	0.00	0.00	0.00
glycoprotein A33 (transmembrane)	Gpa33	100771877	0.00	0.00	0.00	0.00	0.04	0.00	0.00	0.92	0.00	1.16	1.03	0.00
glycosylphosphatidylinositol anchor attachment 1	Gpaa1	100766326	27.93	25.18	35.27	28.01	20.58	25.00	1991.51	1798.04	2568.64	2082.58	1618.72	1898.31
GPALPP motifs containing 1	Gpalpp1	100768616	33.62	33.83	32.26	33.85	37.62	38.05	1202.35	1228.89	1186.19	1257.43	1387.92	1373.03
glycerol-3-phosphate acyltransferase, mitochondrial	Gpam	100760138	42.76	42.69	53.72	54.57	53.41	55.10	7558.17	7638.70	9658.47	9708.27	9453.56	9734.58
G patch domain and ankyrin repeats 1	Gpank1	100763325	58.72	60.97	71.43	55.91	46.93	50.71	2354.57	2456.87	2921.39	2295.82	1960.26	2115.16
glycerol-3-phosphate acyltransferase 2, mitochondrial	Gpat2	100750356	0.00	0.00	0.02	0.00	0.00	0.05	20.51	22.88	19.87	16.22	14.49	18.69
G patch domain containing 1	Gpatch1	100757025	2.25	2.28	2.40	2.19	2.05	2.24	205.84	211.37	203.70	201.65	192.51	205.63
G patch domain containing 11	Gpatch11	100765159	36.93	36.08	37.27	39.52	44.70	39.50	3994.42	3914.51	4052.93	4257.87	4797.16	4260.22
G patch domain containing 2	Gpatch2	100760239	7.43	7.62	8.17	8.35	9.83	9.49	514.21	519.74	541.55	573.67	626.17	628.10
G patch domain containing 2-like	Gpatch2l	100757412	16.08	13.62	14.91	15.59	10.55	12.91	1961.13	1908.76	2029.57	2150.96	2063.76	2162.83
G patch domain containing 3	Gpatch3	100769311	7.64	7.45	7.45	4.95	3.51	3.65	504.33	489.54	488.14	329.13	243.22	248.62
G patch domain containing 4	Gpatch4	100753809	40.17	40.55	39.27	41.37	37.76	38.41	2252.03	2252.81	2182.35	2277.28	2057.55	2128.24
G patch domain containing 8	Gpatch8	100757325	21.98	21.91	16.28	17.92	16.66	16.78	4322.54	4340.92	3264.20	3548.61	3291.26	3310.60
G protein-coupled bile acid receptor 1	Gpbar1	100774722	0.15	0.18	0.13	0.10	0.07	0.23	8.35	13.73	11.18	8.11	10.35	14.02
GC-rich promoter binding protein 1	Gpbp1	100766572	48.12	49.21	50.68	57.29	54.29	52.48	2786.75	2886.02	2963.62	3268.15	3105.99	3006.83
GC-rich promoter binding protein 1-like 1	Gpbp1l1	100750460	37.34	38.90	33.88	36.59	40.56	39.54	3683.01	3877.91	3374.75	3547.45	3905.00	3829.34
glypican 1	Gpc1	100751141	45.73	43.02	42.22	30.53	19.51	23.02	4431.91	4232.03	4146.09	2934.39	1865.05	2213.30

glypican 2	Gpc2	100772009	0.00	0.00	0.00	0.00	0.00	0.00	0.76	0.00	0.00	0.00	0.00	0.00
glypican 3	Gpc3	100768919	0.00	0.00	0.00	0.00	0.00	0.00	0.76	0.00	0.00	0.00	0.00	0.93
glypican 4	Gpc4	100768625	0.00	0.00	0.00	0.00	0.00	0.00	0.00	0.00	0.00	0.00	0.00	0.00
glypican 5	Gpc5	100768469	0.11	0.07	0.15	0.17	0.03	0.07	10.63	9.15	12.42	10.43	5.17	3.74
glypican 6	Gpc6	100767906	9.60	10.06	13.32	13.96	14.55	13.55	1492.49	1581.18	2122.73	2176.45	2306.98	2165.63
glycerophosphocholine phosphodiesterase GDE1 homolog (S. cerevisiae)	Gpcpd1	100772527	5.18	5.60	6.50	7.50	8.35	7.45	459.52	502.35	573.84	653.63	746.23	668.29
glycerol-3-phosphate dehydrogenase 1 (soluble)	Gpd1	100772179	0.04	0.07	0.03	0.06	0.00	0.04	12.15	11.90	8.69	10.43	8.28	9.35
glycerol-3-phosphate dehydrogenase 1-like	Gpd1l	100773663	5.74	5.31	6.29	6.00	4.49	5.97	208.11	199.48	236.00	215.56	173.88	214.04
glycerol-3-phosphate dehydrogenase 2 (mitochondrial)	Gpd2	100766128	17.64	18.32	20.29	22.33	27.68	26.66	2841.44	2983.92	3296.50	3581.06	4417.32	4254.62
G protein-coupled estrogen receptor 1	Gper1	100767871	0.00	0.00	0.00	0.00	0.00	0.00	0.00	0.00	0.00	1.16	0.00	0.00
glycoprotein hormone alpha 2	Gpha2	100759178	0.19	0.14	0.06	0.09	0.13	0.12	13.67	14.64	6.21	11.59	10.35	9.35
glycoprotein hormone beta 5	Gphb5	100762212	0.00	0.00	0.00	0.00	0.00	0.00	0.00	0.00	0.00	0.00	0.00	0.00
golgi pH regulator	Gphr	100689387	31.83	31.62	37.84	38.15	37.49	39.39	2057.59	2109.15	2458.09	2456.91	2424.97	2509.59
glucose-6-phosphate isomerase	Gpi	100689468	28.02	28.12	37.79	31.32	23.94	26.67	1357.30	1428.37	1835.80	1549.48	1262.68	1313.21
glycosylphosphatidylinositol anchored high density lipoprotein binding protein 1	Gpihbp1	100759460	0.00	0.00	0.00	0.00	0.00	0.00	0.00	0.92	0.00	0.00	0.00	0.00
G patch domain and KOW motifs	Gpkow	100759434	14.48	13.86	16.85	16.73	15.69	16.74	1264.63	1237.13	1491.75	1477.62	1386.88	1465.56
glycosylphosphatidylinositol specific phospholipase D1	Gpld1	100759513	0.94	1.12	0.91	1.21	1.20	1.24	78.23	88.76	74.53	84.60	94.18	100.01
glycoprotein M6A	Gpm6a	100767618	0.00	0.00	0.00	0.00	0.00	0.00	0.00	0.92	0.00	0.00	0.00	0.00
glycoprotein M6B	Gpm6b	100765850	0.00	0.00	0.00	0.00	0.00	0.00	1.52	0.92	0.00	1.16	1.03	0.00
GPN-loop GTPase 1	Gpn1	100757373	30.40	29.26	35.00	37.50	36.25	36.70	2056.07	1983.79	2309.04	2473.14	2398.06	2414.25
GPN-loop GTPase 2	Gpn2	100769599	24.22	23.84	29.60	28.28	26.75	26.83	931.95	917.78	1115.39	1078.95	992.55	999.16
GPN-loop GTPase 3	Gpn3	100764448	19.37	19.51	26.09	27.57	29.46	28.99	851.44	843.66	1089.31	1116.04	1169.53	1173.01
glycoprotein (transmembrane) nmb	Gpnmb	100761867	13.38	13.63	14.90	14.62	17.18	16.96	1042.85	1059.61	1163.84	1134.58	1341.34	1326.30
G protein-coupled receptor 1	Gpr1	100757115	0.00	0.03	0.00	0.00	0.00	0.00	0.00	0.92	0.00	0.00	0.00	0.00
G protein-coupled receptor 101	Gpr101	100762214	0.00	0.00	0.00	0.00	0.00	0.00	0.00	0.00	0.00	0.00	0.00	0.00
G protein-coupled receptor 107	Gpr107	100766180	13.32	12.67	13.61	13.41	12.48	13.73	2295.33	2180.52	2358.72	2309.73	2130.00	2361.91
G protein-coupled receptor 108	Gpr108	100764254	17.51	16.87	22.37	24.13	20.31	22.56	1169.69	1124.58	1504.17	1623.65	1340.31	1474.91
G protein-coupled receptor 110	Gpr110	100768933	0.67	0.70	0.33	0.36	0.62	0.47	86.59	89.67	43.47	39.40	75.55	58.88
G protein-coupled receptor 111	Gpr111	100770071	0.00	0.00	0.02	0.02	0.00	0.00	0.00	0.00	1.24	1.16	0.00	0.00
G protein-coupled receptor 112	Gpr112	100767655	0.00	0.00	0.00	0.00	0.00	0.00	0.00	0.00	0.00	0.00	0.00	0.00
G protein-coupled receptor 113	Gpr113	100758712	0.22	0.18	0.10	0.03	0.11	0.09	41.02	32.03	14.91	11.59	17.59	24.30
G protein-coupled receptor 114	Gpr114	100765170	0.02	0.02	0.09	0.03	0.00	0.00	2.28	1.83	3.73	1.16	1.03	0.93
G protein-coupled receptor 115	Gpr115	100770639	0.00	0.00	0.00	0.00	0.00	0.00	0.00	0.00	0.00	0.00	0.00	0.00
G protein-coupled receptor 116	Gpr116	100769211	0.04	0.00	0.00	0.00	0.00	0.00	6.08	0.92	0.00	0.00	0.00	0.00
G protein-coupled receptor 119	Gpr119	100770518	0.00	0.00	0.00	0.00	0.00	0.00	0.00	0.00	0.00	0.00	0.00	0.00
G protein-coupled receptor 12	Gpr12	100764513	0.00	0.00	0.00	0.00	0.00	0.00	0.00	0.00	0.00	0.00	0.00	0.00
G protein-coupled receptor 123	Gpr123	100772388	0.00	0.00	0.00	0.00	0.00	0.00	0.00	0.00	0.00	0.00	0.00	0.00

G protein-coupled receptor 124	Gpr124	100751137	32.99	32.11	25.23	18.33	12.90	15.13	4636.23	4620.00	3758.55	2839.35	2117.58	2410.51
G protein-coupled receptor 125	Gpr125	100753965	4.21	3.84	3.31	3.68	3.79	3.63	540.79	495.03	429.76	465.89	484.37	473.88
G protein-coupled receptor 126	Gpr126	100772057	0.04	0.07	0.03	0.02	0.04	0.04	11.39	15.56	6.21	3.48	9.31	11.22
G protein-coupled receptor 128	Gpr128	100759621	0.00	0.00	0.00	0.00	0.00	0.00	0.00	0.00	0.00	0.00	0.00	0.00
G protein-coupled receptor 132	Gpr132	100753002	0.56	0.61	0.54	0.27	0.39	0.09	27.34	25.62	21.12	24.34	15.52	7.48
G protein-coupled receptor 133	Gpr133	100753766	33.23	32.78	5.22	5.63	1.99	2.04	4888.39	4832.29	794.94	826.31	290.83	297.22
G protein-coupled receptor 135	Gpr135	100764113	0.05	0.00	0.09	0.00	0.02	0.00	2.28	0.00	3.73	0.00	2.07	0.00
G protein-coupled receptor 137	Gpr137	100757640	2.36	2.45	2.13	1.47	1.70	1.44	139.76	139.09	141.60	112.42	97.29	91.60
G protein-coupled receptor 137B	Gpr137<br>b	100764875	12.44	12.16	12.89	13.64	14.22	13.31	685.10	662.48	731.59	752.14	796.94	771.10
G protein-coupled receptor 137C	Gpr137c	100763431	0.00	0.06	0.08	0.07	0.13	0.09	0.00	1.83	1.24	4.64	4.14	2.80
G protein-coupled receptor 139	Gpr139	100766538	0.00	0.00	0.00	0.00	0.00	0.00	0.00	0.00	0.00	0.00	0.00	0.00
G protein-coupled receptor 142	Gpr142	100761120	0.00	0.00	0.00	0.00	0.00	0.00	0.00	0.92	0.00	0.00	0.00	0.00
G protein-coupled receptor 143	Gpr143	100773343	0.00	0.00	0.00	0.00	0.00	0.00	0.00	0.00	0.00	0.00	0.00	0.00
G protein-coupled receptor 144	Gpr144	100772777	0.00	0.00	0.00	0.00	0.00	0.00	0.00	0.00	0.00	0.00	0.00	0.00
G protein-coupled receptor 146	Gpr146	100768440	0.18	0.18	0.07	0.07	0.05	0.15	19.75	20.13	7.45	8.11	5.17	16.82
G protein-coupled receptor 149	Gpr149	100771902	0.01	0.00	0.00	0.00	0.00	0.00	0.76	0.00	0.00	0.00	0.00	0.00
G protein-coupled receptor 15	Gpr15	100770469	0.04	0.03	0.02	0.02	0.06	0.10	3.80	2.75	1.24	2.32	3.10	5.61
G protein-coupled receptor 151	Gpr151	100765241	0.00	0.00	0.00	0.00	0.00	0.00	0.00	0.00	0.00	0.00	0.00	0.00
G protein-coupled receptor 152	Gpr152	100759644	0.28	0.31	0.09	0.15	0.19	0.14	15.19	17.39	6.21	12.75	15.52	11.22
G protein-coupled receptor 153	Gpr153	100755439	0.00	0.00	0.00	0.00	0.00	0.00	0.00	0.92	0.00	0.00	1.03	0.00
G protein-coupled receptor 155	Gpr155	100766852	0.01	0.00	0.00	0.00	0.00	0.00	1.52	0.00	0.00	1.16	0.00	0.93
G protein-coupled receptor 156	Gpr156	100761559	1.36	1.68	2.11	2.51	2.52	2.45	139.00	172.03	216.12	251.49	229.77	249.56
G protein-coupled receptor 157	Gpr157	100756989	0.00	0.00	0.00	0.00	0.00	0.00	0.00	0.00	0.00	0.00	0.00	0.00
G protein-coupled receptor 158	Gpr158	100757301	0.01	0.00	0.02	0.00	0.00	0.01	0.76	0.00	1.24	0.00	0.00	0.93
G protein-coupled receptor 160	Gpr160	100766599	0.15	0.05	0.08	0.17	0.07	0.08	6.84	2.75	4.97	9.27	2.07	1.87
G protein-coupled receptor 161	Gpr161	100757968	7.01	6.55	8.26	8.76	8.36	8.98	792.20	765.88	915.42	953.79	1003.94	1041.22
G protein-coupled receptor 162	Gpr162	103163106	1.85	1.37	1.57	1.33	1.28	1.41	165.58	130.85	151.53	118.21	111.78	127.12
G protein-coupled receptor 17	Gpr17	103163592	0.00	0.00	0.00	0.00	0.00	0.00	0.00	0.00	0.00	0.00	0.00	0.00

G protein-coupled receptor 173	Gpr173	100751517	0.00	0.00	0.00	0.00	0.00	0.00	0.00	0.00	0.00	0.00	0.00	0.00
G protein-coupled receptor 174	Gpr174	100756360	0.52	0.34	0.21	0.46	0.02	0.02	48.61	43.01	26.08	47.52	2.07	0.93
G protein-coupled receptor 176	Gpr176	100763678	11.58	11.37	19.09	17.88	15.79	16.21	1124.12	1102.62	1878.04	1741.86	1523.50	1582.40
G protein-coupled receptor 179	Gpr179	100763472	0.02	0.01	0.06	0.07	0.01	0.02	4.56	1.83	13.66	18.54	4.14	5.61
G protein-coupled receptor 18	Gpr18	100764954	0.34	0.38	0.19	0.24	0.34	0.29	26.58	32.94	16.15	20.86	30.01	25.24
G protein-coupled receptor 180	Gpr180	100767331	16.49	16.42	13.80	17.75	16.82	16.39	830.18	845.49	701.78	885.41	833.16	806.62
G protein-coupled receptor 182	Gpr182	100752156	0.14	0.17	0.06	0.05	0.08	0.01	9.11	10.98	3.73	4.64	5.17	0.93
G protein-coupled receptor 183	Gpr183	103161487	0.00	0.00	0.00	0.00	0.00	0.00	0.00	0.00	0.00	0.00	0.00	0.00
G protein-coupled receptor 19	Gpr19	100750479	4.29	3.91	4.00	4.22	4.09	3.57	273.43	258.04	268.29	289.73	276.34	255.16
G protein-coupled receptor 20	Gpr20	100772615	0.00	0.00	0.00	0.00	0.00	0.00	0.00	0.00	0.00	0.00	0.00	0.00
G protein-coupled receptor 21	Gpr21	100770264	0.09	0.17	0.09	0.13	0.08	0.11	3.04	6.41	3.73	5.79	3.10	5.61
G protein-coupled receptor 22	Gpr22	100753963	0.00	0.00	0.00	0.03	0.00	0.00	0.00	0.00	0.00	1.16	0.00	0.00
G protein-coupled receptor 25	Gpr25	100766966	0.00	0.00	0.00	0.00	0.00	0.00	0.00	0.00	0.00	0.00	0.00	0.00
G protein-coupled receptor 3	Gpr3	100759546	0.43	0.28	0.35	0.14	0.35	0.28	24.31	15.56	19.87	8.11	19.66	15.89
G protein-coupled receptor 31	Gpr31	100773323	0.03	0.00	0.00	0.00	0.00	0.00	0.76	0.00	0.00	0.00	0.00	0.00
G protein-coupled receptor 33	Gpr33	100752683	0.00	0.00	0.00	0.00	0.00	0.00	0.00	0.00	0.00	0.00	0.00	0.00
G protein-coupled receptor 34	Gpr34	100764606	0.00	0.00	0.00	0.00	0.00	0.00	0.00	0.00	0.00	0.00	0.00	0.00
G protein-coupled receptor 35	Gpr35	100770330	0.00	0.00	0.00	0.00	0.00	0.00	0.00	0.00	0.00	0.00	0.00	0.00
G protein-coupled receptor 37 (endothelin receptor type B-like)	Gpr37	100758560	0.00	0.00	0.00	0.00	0.00	0.00	0.00	0.00	0.00	0.00	0.00	0.00
G protein-coupled receptor 37 like 1	Gpr37l1	100754383	0.02	0.02	0.03	0.00	0.00	0.00	0.76	0.92	1.24	0.00	0.00	0.00
G protein-coupled receptor 39	Gpr39	100759577	2.51	2.65	1.99	1.58	1.59	1.37	136.72	140.92	104.34	84.60	93.15	74.77
G protein-coupled receptor 45	Gpr45	100761727	0.00	0.00	0.00	0.00	0.00	0.00	0.00	0.00	0.00	0.00	0.00	0.00
G protein-coupled receptor 50	Gpr50	100772960	0.00	0.00	0.00	0.00	0.00	0.00	0.00	0.00	0.00	0.00	0.00	0.00
G protein-coupled receptor 52	Gpr52	100752204	0.26	0.24	0.22	0.16	0.34	0.15	22.79	21.05	19.87	15.07	30.01	13.09
G protein-coupled receptor 55	Gpr55	100750740	0.00	0.00	0.00	0.00	0.00	0.00	0.00	0.00	0.00	0.00	0.00	0.00
G protein-coupled receptor 56	Gpr56	100764884	27.37	26.80	3.77	3.53	3.80	3.82	2876.38	2877.78	414.86	373.17	417.10	403.78
G protein-coupled receptor 6	Gpr6	100772414	0.00	0.00	0.00	0.00	0.00	0.00	0.00	0.00	0.00	0.00	0.00	0.00
G protein-coupled receptor 61	Gpr61	100772674	0.02	0.00	0.04	0.00	0.06	0.00	0.76	0.00	1.24	0.00	2.07	0.00
G protein-coupled receptor 62	Gpr62	103160720	0.00	0.06	0.13	0.12	0.07	0.06	1.52	2.75	6.21	4.64	3.10	3.74
G protein-coupled receptor 63	Gpr63	100767198	0.00	0.00	0.00	0.00	0.00	0.00	0.00	0.00	0.00	0.00	0.00	0.00
G protein-coupled receptor 64	Gpr64	100754781	0.02	0.05	0.00	0.00	0.00	0.00	3.80	7.32	1.24	0.00	0.00	0.00
G protein-coupled receptor 65	Gpr65	100751031	0.00	0.00	0.00	0.00	0.00	0.00	0.00	0.00	0.00	0.00	0.00	0.00
G protein-coupled receptor 68	Gpr68	100752950	0.00	0.03	0.00	0.00	0.00	0.00	0.76	1.83	0.00	0.00	0.00	0.00
G protein-coupled receptor 75	Gpr75	100766988	0.01	0.02	0.00	0.00	0.00	0.02	0.76	1.83	0.00	0.00	1.03	1.87
G protein-coupled receptor 82	Gpr82	100765377	0.00	0.00	0.00	0.00	0.00	0.00	0.00	0.00	0.00	0.00	0.00	0.00
G protein-coupled receptor 83	Gpr83	100751464	0.00	0.03	0.00	0.00	0.03	0.00	0.00	0.92	1.24	0.00	1.03	0.00
G protein-coupled receptor 84	Gpr84	100771143	0.03	0.03	0.00	0.00	0.04	0.03	0.76	0.92	0.00	0.00	1.03	0.93
G protein-coupled receptor 85	Gpr85	100763062	0.00	0.02	0.00	0.00	0.00	0.00	0.00	0.92	0.00	0.00	0.00	0.00
G protein-coupled receptor 87	Gpr87	100770345	0.00	0.00	0.00	0.00	0.00	0.00	0.00	0.00	0.00	0.00	0.00	0.00
G protein-coupled receptor 97	Gpr97	100764587	0.00	0.00	0.00	0.00	0.00	0.00	0.00	0.00	0.00	0.00	0.00	0.00

G protein-coupled receptor 98	Gpr98	100766602	0.07	0.11	0.01	0.05	0.01	0.15	7.60	10.07	3.73	3.48	6.21	5.61
G protein-coupled receptor associated sorting protein 1	Gprasp1	100769500	6.21	6.31	7.87	8.08	9.24	9.11	800.55	802.48	1016.03	1046.50	1178.85	1178.62
G protein-coupled receptor associated sorting protein 2	Gprasp2	100767299	1.07	0.96	0.87	1.05	0.88	1.22	116.21	90.59	104.34	106.62	86.94	129.92
G protein-coupled receptor, class C, group 5, member A	Gprc5a	100751438	13.80	12.64	11.94	10.13	10.75	10.79	969.93	891.24	865.73	730.12	766.92	779.51
G protein-coupled receptor, class C, group 5, member B	Gprc5b	100762762	0.01	0.00	0.00	0.00	0.00	0.00	0.76	0.00	0.00	0.00	0.00	0.00
G protein-coupled receptor, class C, group 5, member C	Gprc5c	100750919	0.00	0.00	0.00	0.00	0.00	0.00	0.00	0.00	0.00	0.00	0.00	0.00
G protein-coupled receptor, class C, group 5, member D	Gprc5d	100751724	0.00	0.00	0.00	0.00	0.00	0.00	0.76	0.00	0.00	0.00	0.00	0.93
G protein-coupled receptor, class C, group 6, member A	Gprc6a	100773740	0.00	0.00	0.00	0.00	0.00	0.00	0.00	0.00	0.00	0.00	0.00	0.00
G protein regulated inducer of neurite outgrowth 1	Gprin1	100767353	0.00	0.00	0.00	0.00	0.00	0.00	0.00	0.00	0.00	0.00	0.00	0.00
G protein regulated inducer of neurite outgrowth 2	Gprin2	100773657	0.00	0.00	0.00	0.00	0.00	0.00	0.00	0.00	0.00	0.00	0.00	0.00
GPRIN family member 3	Gprin3	100769431	0.00	0.00	0.00	0.00	0.00	0.00	0.00	0.00	0.00	0.00	0.00	0.00
G protein pathway suppressor 1	Gps1	100770684	119.78	117.81	50.70	41.08	26.87	27.68	7069.03	6843.54	2966.10	2365.36	1570.07	1606.70
G protein pathway suppressor 2	Gps2	100766072	27.28	25.03	29.08	26.47	24.31	26.55	957.02	884.84	1023.48	909.75	846.62	918.78
G-protein signaling modulator 1	Gpsm1	100758972	4.02	4.25	4.27	2.95	1.96	2.15	377.49	386.14	406.16	281.62	184.23	216.84
G-protein signaling modulator 2	Gpsm2	100766054	40.60	40.16	32.56	38.15	40.08	38.15	3175.63	3141.31	2528.89	2908.89	3080.12	2958.23
G-protein signaling modulator 3	Gpsm3	100766237	20.92	20.53	20.15	13.40	8.35	10.74	567.38	560.00	537.82	349.99	214.24	271.05
glutamic-pyruvate transaminase (alanine aminotransferase)	Gpt	100760506	0.09	0.11	0.09	0.03	0.05	0.05	8.35	8.24	6.21	4.64	3.10	4.67
glutamic pyruvate transaminase (alanine aminotransferase) 2	Gpt2	100768176	19.50	18.22	18.56	20.26	18.56	19.21	1999.87	1888.63	1935.17	2080.26	1909.55	1998.32
glutathione peroxidase 1	Gpx1	100771188	166.29	166.71	147.68	127.71	95.45	105.41	3567.56	3587.85	3148.69	2668.99	2036.85	2256.29
glutathione peroxidase 2 (gastrointestinal)	Gpx2	100751356	0.00	0.00	0.00	0.00	0.04	0.00	0.00	0.00	0.00	0.00	1.03	0.93
glutathione peroxidase 3 (plasma)	Gpx3	100764638	0.44	0.49	0.47	0.44	0.58	0.59	60.76	55.82	81.98	77.65	87.97	84.12
glutathione peroxidase 4	Gpx4	100765235	227.36	219.16	271.90	237.19	205.29	229.97	5441.34	5291.64	6560.70	5547.75	4915.15	5410.80
glutathione peroxidase 5 (epididymal androgen-related protein)	Gpx5	100766810	0.00	0.00	0.00	0.00	0.00	0.00	0.00	0.00	0.00	0.00	0.00	0.00
glutathione peroxidase 6 (olfactory)	Gpx6	100766519	0.00	0.00	0.00	0.00	0.00	0.00	0.00	0.00	0.00	0.00	1.03	0.93
glutathione peroxidase 7	Gpx7	100756109	0.03	0.04	0.00	0.00	0.00	0.00	0.76	0.92	0.00	0.00	0.00	0.00
glutathione peroxidase 8 (putative)	Gpx8	100775000	88.46	93.64	80.31	88.04	99.99	91.04	3061.70	3228.24	2759.92	3003.92	3400.97	3148.90
GRAM domain containing 1A	Gramd1a	100758316	10.57	9.82	12.40	9.14	6.07	5.96	635.73	592.03	742.77	534.26	352.93	357.98
GRAM domain containing 1B	Gramd1b	100760809	0.14	0.15	0.00	0.00	0.00	0.00	10.63	12.81	0.00	0.00	0.00	0.00
GRAM domain containing 1C	Gramd1c	100773582	0.99	0.91	0.42	0.93	0.83	0.85	93.42	88.76	47.20	64.90	74.52	61.69
GRAM domain containing 2	Gramd2	100755941	0.51	0.41	0.33	0.43	0.55	0.52	46.33	36.60	33.54	35.93	42.43	42.99
GRAM domain containing 3	Gramd3	100752099	6.97	6.55	8.12	8.25	10.16	9.12	488.38	457.52	573.84	572.51	707.93	635.58
GRAM domain containing 4	Gramd4	100763433	14.05	14.95	12.78	13.32	12.36	13.39	1282.86	1356.08	1177.50	1218.02	1147.80	1233.76
GRB2-related adaptor protein 2	Grap2	100769885	0.04	0.03	0.05	0.00	0.00	0.00	2.28	1.83	2.48	0.00	0.00	0.00

GRP1 (general receptor for phosphoinositides 1)-associated scaffold protein	Grasp	100764514	2.21	2.42	2.34	1.37	1.69	1.56	101.02	108.89	81.98	74.17	62.10	54.21
growth factor receptor-bound protein 10	Grb10	100760784	0.01	0.02	0.05	0.08	0.14	0.08	0.76	2.75	8.69	13.91	18.63	10.28
growth factor receptor-bound protein 14	Grb14	100752932	14.79	15.00	18.90	21.62	22.99	22.63	830.94	840.92	1064.47	1134.58	1243.02	1228.16
growth factor receptor-bound protein 2	Grb2	100769627	53.85	52.65	53.75	53.25	51.24	52.33	3832.63	3770.85	3836.81	3765.33	3601.75	3721.85
growth factor receptor-bound protein 7	Grb7	100760569	0.02	0.00	0.00	0.00	0.00	0.00	0.76	0.00	0.00	0.00	0.00	0.00
growth regulation by estrogen in breast cancer 1	Greb1	100764943	0.02	0.02	0.04	0.06	0.08	0.09	3.80	3.66	11.18	9.27	15.52	16.82
growth regulation by estrogen in breast cancer-like	Greb1l	100765529	0.02	0.01	0.02	0.00	0.03	0.05	3.80	2.75	3.73	0.00	6.21	12.15
gremlin 1, DAN family BMP antagonist	Grem1	100758320	0.03	0.00	0.00	0.00	0.00	0.00	0.76	0.00	0.00	0.00	0.00	0.00
gremlin 2, DAN family BMP antagonist	Grem2	100773984	0.00	0.00	0.00	0.00	0.00	0.00	0.00	0.00	0.00	0.00	0.00	0.00
grainyhead-like 1 (Drosophila)	Grhl1	100753443	1.74	1.69	0.94	0.89	0.96	0.91	274.95	270.85	154.02	151.82	154.21	143.94
grainyhead-like 2 (Drosophila)	Grhl2	100768148	0.06	0.11	0.04	0.02	0.02	0.00	4.56	7.32	2.48	2.32	1.03	0.93
grainyhead-like 3 (Drosophila)	Grhl3	100757124	0.32	0.41	0.25	0.31	0.42	0.36	29.62	35.69	22.36	24.34	38.29	38.32
glyoxylate reductase/hydroxypyruvate reductase	Grhpr	100752744	8.59	8.76	9.95	9.35	6.43	7.19	348.63	360.52	416.10	372.01	259.78	292.55
glutamate receptor, ionotropic, AMPA 1	Gria1	100769392	0.00	0.00	0.00	0.00	0.00	0.00	0.00	0.00	0.00	0.00	0.00	0.00
glutamate receptor, ionotropic, AMPA 2	Gria2	100764344	0.00	0.00	0.00	0.00	0.00	0.00	0.00	0.00	0.00	0.00	0.00	0.00
glutamate receptor, ionotropic, AMPA 3	Gria3	100770349	0.00	0.00	0.00	0.00	0.00	0.00	0.00	0.00	0.00	0.00	0.00	0.00
glutamate receptor, ionotropic, AMPA 4	Gria4	100755681	0.12	0.11	0.10	0.11	0.15	0.28	22.03	20.13	27.33	27.81	42.43	53.28
glutamate receptor, ionotropic, delta 1	Grid1	100760535	0.00	0.00	0.00	0.00	0.00	0.00	0.00	0.00	0.00	0.00	0.00	0.00
glutamate receptor, ionotropic, delta 2	Grid2	100769746	0.00	0.04	0.00	0.01	0.00	0.00	0.00	2.75	0.00	1.16	0.00	0.00
glutamate receptor, ionotropic, delta 2 (Grid2) interacting protein	Grid2ip	100766186	0.03	0.01	0.00	0.00	0.00	0.01	3.80	0.92	0.00	1.16	1.03	0.93
glutamate receptor, ionotropic, kainate 1	Grik1	100770303	0.00	0.00	0.00	0.00	0.00	0.00	0.00	0.00	0.00	0.00	0.00	0.00
glutamate receptor, ionotropic, kainate 2	Grik2	100754248	0.00	0.01	0.00	0.00	0.00	0.00	0.00	0.92	0.00	0.00	0.00	0.00
glutamate receptor, ionotropic, kainate 3	Grik3	100755668	0.00	0.00	0.00	0.00	0.00	0.00	0.00	0.00	0.00	0.00	0.00	0.00
glutamate receptor, ionotropic, kainate 4	Grik4	100767895	0.00	0.00	0.00	0.00	0.00	0.00	0.00	0.00	0.00	0.00	0.00	0.00
glutamate receptor, ionotropic, kainate 5	Grik5	100757915	8.42	7.40	8.88	7.35	5.22	6.73	698.78	604.84	730.35	602.64	419.17	538.37
glutamate receptor, ionotropic, N-methyl D-aspartate 1	Grin1	100770880	0.00	0.01	0.02	0.01	0.00	0.00	0.00	0.92	1.24	1.16	1.03	0.00
glutamate receptor, ionotropic, N-methyl D-aspartate 2A	Grin2a	100764315	0.00	0.00	0.00	0.00	0.00	0.00	0.00	0.00	0.00	0.00	0.00	0.00
glutamate receptor, ionotropic, N-methyl D-aspartate 2B	Grin2b	100753338	0.00	0.00	0.00	0.00	0.00	0.00	0.00	0.00	0.00	0.00	0.00	0.00

glutamate receptor, ionotropic, N-methyl D-aspartate 2C	Grin2c	100758804	0.06	0.07	0.12	0.07	0.10	0.11	12.15	19.22	40.99	31.29	18.63	28.97
glutamate receptor, ionotropic, N-methyl D-aspartate 2D	Grin2d	100750615	4.23	4.16	2.62	2.49	2.18	2.10	304.58	289.15	183.83	166.88	146.97	156.09
glutamate receptor, ionotropic, N-methyl-D-aspartate 3A	Grin3a	100758947	0.00	0.00	0.00	0.00	0.00	0.00	0.00	0.00	0.00	0.00	0.00	0.93
glutamate receptor, ionotropic, N-methyl-D-aspartate 3B	Grin3b	100753630	2.99	2.77	2.61	1.71	1.15	1.20	640.29	611.24	542.79	337.25	193.54	230.86
glutamate receptor, ionotropic, N-methyl D-aspartate-associated protein 1 (glutamate binding)	Grina	100753055	38.64	36.39	48.86	48.58	40.08	43.83	1717.32	1618.69	2148.81	2126.62	1767.76	1915.14
glutamate receptor interacting protein 1	Grip1	100767728	0.00	0.00	0.00	0.00	0.00	0.00	0.00	0.00	0.00	0.00	0.00	0.00
glutamate receptor interacting protein 2	Grip2	100774140	0.00	0.00	0.00	0.00	0.00	0.00	0.76	0.00	0.00	0.00	0.00	0.00
GRIP1 associated protein 1	Gripap1	100768253	16.78	15.39	15.78	14.74	13.86	16.26	1331.47	1266.41	1293.01	1200.64	1141.59	1288.91
G protein-coupled receptor kinase 1	Grk1	100750718	0.02	0.00	0.00	0.00	0.00	0.00	0.76	0.92	0.00	0.00	0.00	0.00
G protein-coupled receptor kinase 4	Grk4	100767022	4.25	4.22	4.16	4.34	5.48	6.07	279.51	272.68	252.14	285.09	368.46	370.13
G protein-coupled receptor kinase 5	Grk5	100771151	2.27	2.21	2.44	2.93	3.14	3.30	287.87	281.83	321.70	383.60	401.57	412.19
G protein-coupled receptor kinase 6	Grk6	103161260	36.77	35.68	33.59	29.65	23.47	25.53	3177.91	3172.42	3022.00	2668.99	2085.50	2246.95
glutamate receptor, metabotropic 1	Grm1	100751598	0.00	0.00	0.00	0.00	0.00	0.00	0.00	0.00	0.00	0.00	0.00	0.00
glutamate receptor, metabotropic 2	Grm2	100772929	0.00	0.00	0.00	0.00	0.00	0.00	0.00	0.00	1.24	0.00	1.03	0.00
glutamate receptor, metabotropic 3	Grm3	100760036	0.00	0.00	0.00	0.00	0.00	0.00	0.00	0.92	0.00	0.00	0.00	0.00
glutamate receptor, metabotropic 4	Grm4	100757229	0.00	0.00	0.00	0.02	0.00	0.00	0.00	0.00	0.00	1.16	0.00	0.00
glutamate receptor, metabotropic 5	Grm5	100763614	0.00	0.00	0.00	0.00	0.00	0.00	0.00	0.00	0.00	0.00	0.00	0.00
glutamate receptor, metabotropic 6	Grm6	100768692	0.00	0.00	0.00	0.00	0.00	0.00	0.00	0.00	0.00	0.00	0.00	0.00
glutamate receptor, metabotropic 7	Grm7	100774073	0.00	0.00	0.00	0.00	0.00	0.00	0.00	0.00	0.00	0.00	0.00	0.00
glutamate receptor, metabotropic 8	Grm8	100758817	0.00	0.06	0.00	0.00	0.00	0.00	0.00	0.92	0.00	0.00	0.00	0.00
granulin	Grn	100756647	162.04	154.33	133.43	125.47	103.83	118.33	10283.40	9899.75	8649.89	8076.51	6781.23	7738.13
gastrin-releasing peptide	Grp	100769410	0.00	0.00	0.00	0.00	0.00	0.00	0.00	0.00	0.00	0.00	0.00	0.00
GrpE-like 1, mitochondrial (E. coli)	Grpel1	100768386	122.35	117.01	137.64	136.18	124.32	127.81	3256.14	3120.26	3728.74	3576.43	3275.73	3388.18
GrpE-like 2, mitochondrial (E. coli)	Grpel2	100758874	16.17	16.81	15.34	15.67	15.89	16.61	104.06	94.25	88.19	103.14	97.29	99.07
gastrin-releasing peptide receptor	Grpr	100758098	0.03	0.00	0.00	0.00	0.00	0.00	1.52	0.00	0.00	0.00	0.00	0.00
G-rich RNA sequence binding factor 1	Grsf1	100751594	28.10	28.51	33.61	36.13	39.16	33.89	1680.86	1724.84	2015.91	2138.21	2322.51	2030.10
growth hormone regulated TBC protein 1	Grtp1	100760593	0.03	0.00	0.00	0.00	0.00	0.00	0.76	0.00	3.73	1.16	1.03	0.93
glutamate-rich WD repeat containing 1	Grwd1	100774235	28.88	27.11	31.42	25.47	16.94	19.20	1840.36	1755.95	2023.36	1664.21	1099.16	1230.03
glutaredoxin, cysteine rich 2	Grxcr2	100764658	0.00	0.00	0.00	0.00	0.00	0.00	0.76	0.00	0.00	0.00	0.00	0.00
gamma-secretase activating protein	Gsap	100760803	1.61	1.56	1.78	1.88	2.67	2.24	177.73	170.20	199.98	199.33	274.27	235.54
goosecoid homeobox	Gsc	100760478	0.00	0.00	0.00	0.00	0.00	0.00	0.00	0.00	0.00	0.00	0.00	0.00
goosecoid homeobox 2	Gsc2	103160525	0.00	0.00	0.00	0.00	0.00	0.00	0.00	0.00	0.00	0.00	0.00	0.00
gasdermin D	Gsdmd	100756673	4.20	3.70	4.41	4.01	3.55	3.93	300.78	271.76	308.04	309.43	276.34	287.88
Gse1 coiled-coil protein	Gse1	100771241	1.62	1.65	1.76	1.47	1.30	1.68	334.96	349.54	365.17	297.84	255.64	306.57
germ cell associated 1	Gsg1	100752816	0.00	0.03	0.00	0.00	0.00	0.00	0.76	1.83	1.24	0.00	0.00	0.00
GSG1-like	Gsg1l	100763426	0.00	0.00	0.00	0.31	0.27	0.00	0.00	0.00	1.24	4.64	3.10	1.87
glycogen synthase kinase 3 alpha	Gsk3a	100757049	35.17	35.77	35.92	31.68	26.84	29.10	1401.35	1423.79	1434.61	1224.98	1040.16	1133.75
glycogen synthase kinase 3 beta	Gsk3b	100760865	12.56	12.09	14.69	16.93	20.36	19.76	1317.04	1303.01	1530.25	1752.29	2118.62	2016.08

GSK3B interacting protein	Gskip	100770423	26.13	26.09	29.38	28.47	32.12	32.62	1139.31	1184.05	1345.18	1300.31	1457.26	1465.56
gelsolin	Gsn	100753350	8.78	9.28	6.84	5.63	5.24	5.00	310.65	340.39	188.80	201.65	170.77	175.72
G1 to S phase transition 1	Gspt1	100753280	91.18	96.29	97.51	107.53	107.74	102.51	6023.91	6472.95	6556.98	7138.95	7062.75	6721.21
G1 to S phase transition 2	Gspt2	100767578	0.00	0.00	0.00	0.00	0.00	0.00	0.00	0.00	0.00	0.00	0.00	0.00
glutathione reductase	Gsr	100757970	9.63	9.24	8.55	10.55	11.43	9.82	540.03	523.40	476.96	574.82	633.41	542.11
glutathione synthetase	Gss	100753200	189.41	182.39	216.57	215.85	211.99	236.60	10913.06	10726.02	12673.01	12457.23	12285.29	13425.60
glutathione S-transferase kappa 1	Gstk1	100750404	2.14	1.57	2.32	2.05	1.63	2.19	101.78	97.91	137.87	117.05	95.22	109.36
glutathione S-transferase mu 3 (brain)	Gstm3	100750357	173.39	168.69	131.31	131.89	127.74	141.16	3837.95	3698.57	2817.05	2780.25	2717.87	2992.81
glutathione S-transferase omega 1	Gsto1	100758427	29.28	31.07	35.39	36.89	36.58	37.21	469.40	480.39	583.78	628.13	652.04	647.73
glutathione S-transferase, pi 1	Gstp1	100689311	218.76	212.29	365.93	352.77	271.86	317.89	3632.12	3550.33	6092.44	5641.62	4310.72	4961.23
glutathione S-transferase zeta 1	Gstz1	100764455	4.80	5.36	3.30	4.77	4.33	5.11	176.97	176.60	127.93	159.93	159.39	167.31
GS homeobox 1	Gsx1	100762769	0.00	0.00	0.00	0.00	0.00	0.00	0.00	0.00	0.00	0.00	0.00	0.00
GS homeobox 2	Gsx2	100765381	0.00	0.00	0.00	0.00	0.00	0.00	0.00	0.00	0.00	0.00	0.00	0.00
glycosyltransferase-like domain containing 1	Gtdc1	100752161	7.61	6.49	7.98	9.43	9.07	9.02	437.49	419.09	447.15	507.61	497.83	511.26
general transcription factor IIA, 1, 19/37kDa	Gtf2a1	100750765	17.53	17.58	15.59	17.33	17.18	16.29	1895.81	1918.83	1732.71	1905.26	1861.94	1810.46
general transcription factor IIA, 1-like	Gtf2a1l	100769468	0.00	0.00	0.00	0.00	0.03	0.00	0.00	0.00	0.00	0.00	1.03	0.00
general transcription factor IIA, 2, 12kDa	Gtf2a2	100750331	113.11	116.57	115.54	112.47	129.05	129.54	1863.91	1957.26	1933.93	1836.89	2120.69	2121.70
general transcription factor IIB	Gtf2b	100754725	22.58	23.18	26.11	29.24	29.63	29.52	921.32	931.50	1035.90	1184.42	1148.83	1119.73
general transcription factor IIE, polypeptide 1, alpha 56kDa	Gtf2e1	100763009	24.28	25.28	23.97	24.86	23.94	25.03	2055.31	2132.94	2037.02	2091.85	2013.05	2116.09
general transcription factor IIE, polypeptide 2, beta 34kDa	Gtf2e2	100754209	20.95	17.85	17.27	19.42	20.98	20.76	773.97	682.62	637.19	706.94	802.11	776.71
general transcription factor IIF, polypeptide 1, 74kDa	Gtf2f1	100762222	59.97	58.46	68.64	57.56	46.52	52.16	3123.98	3130.33	3670.37	3035.21	2486.04	2731.10
general transcription factor IIF, polypeptide 2, 30kDa	Gtf2f2	100768332	50.34	50.96	55.44	54.52	51.59	52.85	2168.48	2206.15	2361.21	2225.13	2183.82	2202.08
general transcription factor IIH, polypeptide 1, 62kDa	Gtf2h1	100751383	65.02	66.25	60.89	65.57	67.02	68.48	5118.53	5230.33	4831.72	5140.97	5249.45	5352.85
general transcription factor IIH, polypeptide 3, 34kDa	Gtf2h3	100768069	28.83	28.42	25.32	30.36	31.23	31.13	1734.79	1726.67	1545.16	1829.93	1890.92	1869.34
general transcription factor IIH, polypeptide 4, 52kDa	Gtf2h4	103160241	23.50	22.73	29.10	23.79	15.14	17.79	1319.32	1267.32	1666.88	1369.84	942.87	1030.94
general transcription factor IIH, polypeptide 5	Gtf2h5	100772736	146.19	149.62	166.89	168.03	169.05	175.72	2533.06	2677.39	2957.41	2892.66	2861.74	2936.73
general transcription factor IIi	Gtf2i	100771695	16.63	16.75	17.99	18.03	18.96	18.53	2179.12	2230.85	2397.23	2359.56	2509.84	2375.00
GTF2I repeat domain containing 1	Gtf2ird1	100769489	11.02	9.26	11.35	11.15	9.56	10.61	1111.97	1045.88	1254.51	1253.95	1055.69	1144.97
general transcription factor IIIA	Gtf3a	100763921	74.27	70.27	70.48	71.50	64.64	66.60	2720.67	2652.68	2751.22	2631.91	2382.54	2460.05
general transcription factor IIIC, polypeptide 1, alpha 220kDa	Gtf3c1	100763706	18.38	18.23	18.64	17.93	17.17	18.14	3853.90	3893.47	4000.76	3807.05	3655.57	3831.21
general transcription factor IIIC, polypeptide 2, beta 110kDa	Gtf3c2	100766655	32.53	33.30	35.40	36.17	32.65	35.10	3486.29	3511.90	3838.05	3791.99	3352.32	3591.00
general transcription factor IIIC, polypeptide 3, 102kDa	Gtf3c3	100759578	40.54	41.88	39.03	45.46	46.74	45.36	4155.44	4344.58	4036.78	4612.50	4770.25	4646.24
general transcription factor IIIC, polypeptide 4, 90kDa	Gtf3c4	100773467	9.15	8.48	10.58	11.61	10.85	11.28	833.97	795.16	978.76	1074.32	991.52	1055.24
general transcription factor IIIC, polypeptide 5, 63kDa	Gtf3c5	100770779	28.00	28.53	29.97	29.83	26.13	28.39	1986.20	2029.54	2153.78	2068.67	1818.47	1987.11

general transcription factor IIIC, polypeptide 6, alpha 35kDa	Gtf3c6	100770634	78.16	80.02	65.59	70.89	75.62	69.80	1693.77	1726.67	1430.88	1521.66	1605.26	1474.91
GTP binding protein 1	Gtpbp1	100773534	19.32	19.25	22.13	19.95	18.77	20.65	1865.43	1879.48	2188.56	1962.05	1818.47	1991.78
GTP-binding protein 10 (putative)	Gtpbp10	100768924	22.12	22.15	17.14	20.05	21.82	19.84	1394.51	1406.41	1085.58	1249.32	1354.80	1247.78
GTP binding protein 2	Gtpbp2	100758529	50.40	49.40	50.20	45.44	31.94	37.17	4322.54	4340.92	4315.01	3909.04	2777.90	3191.90
GTP binding protein 3 (mitochondrial)	Gtpbp3	100751349	13.11	12.28	12.61	8.20	5.61	6.36	771.69	724.71	744.01	462.41	317.74	354.24
GTP binding protein 4	Gtpbp4	100753267	113.20	111.68	94.86	108.00	106.60	100.61	9458.54	9386.41	7959.29	8928.32	8822.22	8248.46
GTP binding protein 6 (putative)	Gtpbp6	100761234	3.77	3.86	6.45	4.88	2.49	3.58	217.23	198.56	335.36	241.06	138.69	175.72
GTP-binding protein 8 (putative)	Gtpbp8	100766372	7.97	7.88	9.05	7.83	9.66	8.76	324.32	349.54	411.13	360.42	409.85	381.35
G-2 and S-phase expressed 1	Gtse1	100758123	33.84	34.45	27.23	27.72	25.27	26.06	2549.77	2640.79	2095.40	2109.23	1944.74	1979.63
gametocyte specific factor 1	Gtsf1	100769899	0.00	0.00	0.00	0.00	0.00	0.00	0.00	0.92	1.24	0.00	0.00	0.93
gametocyte specific factor 1-like	Gtsf1l	100772957	0.00	0.00	0.00	0.00	0.00	0.00	0.00	0.92	0.00	1.16	2.07	2.80
guanylate cyclase activator 1A (retina)	Guca1a	100766612	0.00	0.00	0.00	0.00	0.00	0.00	0.00	0.00	0.00	0.00	0.00	0.00
guanylate cyclase activator 1B (retina)	Guca1b	100766902	0.00	0.00	0.00	0.00	0.00	0.00	0.00	0.00	0.00	0.00	0.00	0.00
guanylate cyclase activator 2A (guanylin)	Guca2a	100755664	0.26	0.21	0.39	0.18	0.19	0.23	9.87	9.15	12.42	5.79	7.24	7.48
guanylate cyclase activator 2B (uroguanylin)	Guca2b	100758844	0.00	0.11	0.00	0.00	0.13	0.00	0.00	0.00	0.00	0.00	0.00	0.00
guanylyl cyclase domain containing 1	Gucd1	100767981	15.44	15.81	18.94	18.62	16.71	16.90	1160.58	1192.29	1440.82	1405.77	1257.51	1274.89
guanylate cyclase 1, soluble, alpha 2	Gucy1a 2	100769238	0.00	0.05	0.00	0.03	0.00	0.03	0.76	0.92	0.00	1.16	0.00	0.93
guanylate cyclase 1, soluble, alpha 3	Gucy1a 3	100768699	0.00	0.01	0.00	0.00	0.00	0.00	0.00	0.92	0.00	1.16	0.00	0.00
guanylate cyclase 1, soluble, beta 3	Gucy1b 3	100772822	0.00	0.00	0.00	0.00	0.00	0.00	0.00	0.00	0.00	0.00	0.00	0.00
guanylate cyclase 2C (heat stable enterotoxin receptor)	Gucy2c	100757969	0.12	0.07	0.25	0.25	0.10	0.13	13.67	8.24	28.57	33.61	11.38	19.63
guanylate cyclase 2D, membrane (retina-specific)	Gucy2d	100754759	0.00	0.00	0.00	0.00	0.00	0.00	0.00	0.00	0.00	0.00	0.00	0.00
GUF1 GTPase homolog (S. cerevisiae)	Guf1	100756221	16.38	16.67	16.48	18.48	19.52	19.80	972.21	1012.03	1021.00	1103.29	1171.60	1180.49
guanylate kinase 1	Guk1	100770439	78.66	69.30	75.49	69.05	57.79	64.27	2235.32	1998.43	2208.43	1965.53	1652.87	1800.17
GULP, engulfment adaptor PTB domain containing 1	Gulp1	100759296	33.13	32.36	37.90	36.83	40.55	39.10	864.36	872.94	1011.06	1009.42	1213.00	1186.10
glucuronidase, beta	Gusb	100757362	44.75	46.41	81.09	84.48	72.71	72.16	2868.78	2993.99	5159.63	5305.54	4593.27	4570.53
glucoside xylosyltransferase 1	Gxylt1	100760543	4.75	4.31	3.41	4.39	3.81	3.66	893.22	819.87	652.10	829.79	716.21	701.00
glucoside xylosyltransferase 2	Gxylt2	100758512	0.02	0.00	0.00	0.03	0.00	0.00	0.76	0.00	0.00	1.16	0.00	0.00
glycogenin 1	Gyg1	100751671	46.81	49.37	76.00	77.74	67.39	67.27	2271.78	2381.83	3685.27	3724.77	3233.30	3254.52
glycosyltransferase-like 1B	Gyltl1b	100750932	0.00	0.00	0.00	0.00	0.00	0.00	0.00	0.00	0.00	0.00	0.00	0.00
glycophorin C (Gerbich blood group)	Gypc	100773140	26.87	25.01	31.31	33.19	30.90	30.14	1422.62	1320.39	1656.94	1738.38	1602.16	1576.79
glycogen synthase 1 (muscle)	Gys1	100769788	11.81	12.10	12.76	11.48	9.86	10.24	1210.71	1240.79	1355.12	1194.85	1060.86	1075.80
glycogen synthase 2 (liver)	Gys2	100770628	0.00	0.00	0.00	0.00	0.00	0.00	0.00	0.00	0.00	0.00	0.00	0.00
GDNF-inducible zinc finger protein 1	Gzf1	100763705	8.62	8.70	10.13	10.21	10.60	10.28	1000.31	1012.03	1181.22	1192.53	1223.35	1197.31
granzyme K (granzyme 3; tryptase II)	Gzmk	100774132	0.00	0.00	0.00	0.00	0.00	0.00	0.00	0.00	0.00	0.00	0.00	0.00
granzyme M (lymphocyte met-ase 1)	Gzmm	100764748	2.27	2.29	1.65	1.76	1.37	1.18	95.70	88.76	80.74	69.54	63.13	57.01
H3 histone, family 3A	H3f3a	100753137	377.59	379.26	364.86	367.68	393.35	397.78	5982.13	5849.81	5929.72	6065.79	6372.41	6559.51
H3 histone, family 3B (H3.3B)	H3f3b	100757430	287.10	289.45	346.44	370.82	370.00	384.85	7877.94	8270.07	9924.27	10643.52	10426.45	10730.94

hexose-6-phosphate dehydrogenase (glucose 1-dehydrogenase)	H6pd	100770090	7.08	6.60	9.06	7.60	6.07	6.69	1124.12	1045.88	1459.45	1214.55	956.33	1062.72
3-hydroxyanthranilate 3,4-dioxygenase	Haao	100759566	0.00	0.00	0.00	0.00	0.00	0.04	0.00	0.00	0.00	0.00	0.00	0.93
hyaluronan binding protein 2	Habp2	100774748	0.00	0.00	0.00	0.00	0.00	0.00	0.00	0.00	1.24	0.00	0.00	0.00
hyaluronan binding protein 4	Habp4	100760637	10.67	11.15	14.21	13.75	12.42	12.94	994.24	1034.90	1286.80	1213.39	1134.34	1151.51
HECT domain and ankyrin repeat containing E3 ubiquitin protein ligase 1	Hace1	100769328	44.97	45.64	20.91	22.71	25.15	26.23	2304.44	2341.57	1084.34	1193.69	1314.43	1320.69
2-hydroxyacyl-CoA lyase 1	Hacl1	100767514	84.92	81.82	86.44	90.03	99.30	101.46	6324.68	6203.01	6414.14	6648.72	7385.66	7599.80
hydroxyacyl-CoA dehydrogenase	Hadh	100761491	127.23	136.13	178.20	198.30	182.39	203.09	2796.62	2952.81	4047.96	4151.25	4017.82	4269.57
hydroxyacyl-CoA dehydrogenase/3-ketoacyl-CoA thiolase/enoyl-CoA hydratase (trifunctional protein), alpha subunit	Hadha	100757947	45.01	44.17	45.27	47.00	47.89	46.13	3856.18	3864.19	3970.95	4036.52	4146.16	3989.17
hydroxyacyl-CoA dehydrogenase/3-ketoacyl-CoA thiolase/enoyl-CoA hydratase (trifunctional protein), beta subunit	Hadhb	100758239	43.19	43.19	41.39	43.88	50.62	54.86	2516.35	2551.11	2415.86	2494.00	2895.89	3151.71
hydroxyacylglutathione hydrolase	Hagh	100765715	15.26	13.74	12.12	12.03	14.32	14.82	859.80	782.35	691.84	668.70	787.62	804.75
hydroxyacylglutathione hydrolase-like	Haghl	100772931	0.05	0.06	0.12	0.00	0.07	0.09	4.56	4.58	3.73	1.16	3.10	2.80
histidine ammonia-lyase	Hal	100753802	0.00	0.00	0.00	0.00	0.00	0.00	0.00	0.00	0.00	0.00	0.00	0.00
hepcidin antimicrobial peptide	Hamp	100757672	0.00	0.59	0.00	0.00	0.00	0.21	0.76	4.58	2.48	1.16	0.00	2.80
heart and neural crest derivatives expressed 1	Hand1	100767952	0.00	0.00	0.00	0.00	0.00	0.00	0.00	0.00	0.00	0.00	0.00	0.00
heart and neural crest derivatives expressed 2	Hand2	100763212	0.00	0.00	0.00	0.00	0.00	0.00	0.00	0.00	0.00	0.00	0.00	0.00
hydroxyacid oxidase (glycolate oxidase) 1	Hao1	100771827	0.18	0.19	0.19	0.43	0.56	0.54	9.11	11.90	14.91	28.97	36.22	36.45
hydroxyacid oxidase 2 (long chain)	Hao2	100773170	0.00	0.00	0.00	0.00	0.00	0.00	1.52	0.00	0.00	0.00	0.00	0.00
huntingtin-associated protein 1	Hap1	100757326	0.11	0.16	0.00	0.02	0.00	0.01	19.75	16.47	2.48	1.16	0.00	0.93
hyaluronan and proteoglycan link protein 1	Hapln1	100766601	0.00	0.00	0.00	0.00	0.00	0.00	0.00	0.00	0.00	0.00	0.00	0.00
hyaluronan and proteoglycan link protein 3	Hapln3	100763907	0.00	0.00	0.00	0.00	0.00	0.03	0.00	0.00	0.00	3.48	0.00	0.93
hyaluronan and proteoglycan link protein 4	Hapln4	100764042	11.14	11.08	12.20	10.76	8.76	10.06	1064.11	1063.27	1177.50	1022.17	827.99	963.64
harbinger transposase derived 1	Harbi1	100753396	8.78	8.17	8.70	9.43	10.13	9.45	424.58	390.72	412.37	448.50	492.65	453.31
histidyl-tRNA synthetase	Hars	100757635	26.83	26.79	28.13	30.00	27.05	28.15	1490.98	1518.04	1576.21	1661.89	1520.39	1547.81
histidyl-tRNA synthetase 2, mitochondrial	Hars2	100757923	9.48	10.04	10.83	12.57	10.73	10.50	506.61	573.73	607.38	698.83	618.92	601.93
hyaluronan synthase 2	Has2	100751055	0.00	0.00	0.00	0.00	0.00	0.00	0.00	0.00	0.00	0.00	0.00	0.00
hyaluronan synthase 3	Has3	100757895	0.49	0.63	0.72	0.61	0.61	0.78	25.82	32.03	42.23	38.24	36.22	44.86
histone acetyltransferase 1	Hat1	100768382	75.00	76.59	82.78	83.06	94.04	95.68	3140.69	3221.83	3431.89	3386.36	3862.57	3920.94
HAUS augmin-like complex, subunit 1	Haus1	100770145	9.45	9.61	9.37	10.41	13.29	11.99	432.18	455.69	435.97	483.27	615.82	556.13
HAUS augmin-like complex, subunit 2	Haus2	100757195	29.57	31.11	33.46	34.71	37.14	36.08	1538.07	1619.61	1750.10	1816.03	1937.49	1854.38
HAUS augmin-like complex, subunit 3	Haus3	100770464	10.90	11.30	14.17	14.62	16.54	16.28	1096.02	1143.79	1415.98	1452.13	1637.35	1631.00
HAUS augmin-like complex, subunit 4	Haus4	100769299	38.95	39.28	37.90	41.55	45.33	44.12	1534.27	1551.90	1488.02	1638.71	1751.20	1732.88

HAUS augmin-like complex, subunit 5	Haus5	100751456	1.79	1.82	2.15	1.84	1.39	1.71	179.25	183.92	218.61	180.79	134.55	167.31
HAUS augmin-like complex, subunit 6	Haus6	100761416	34.41	32.97	38.56	46.53	46.35	44.24	890.18	855.56	1049.56	1229.61	1238.88	1211.33
HAUS augmin-like complex, subunit 7	Haus7	100753533	17.72	16.94	20.39	20.73	21.65	19.89	802.83	753.07	878.16	909.75	966.68	883.26
HAUS augmin-like complex, subunit 8	Haus8	100754778	9.26	9.86	10.65	9.82	9.28	9.34	559.02	597.52	642.16	596.84	569.24	571.08
hepatitis A virus cellular receptor 2	Havcr2	100769794	0.00	0.00	0.00	0.00	0.00	0.00	0.00	0.00	0.00	0.00	0.00	0.00
HCLS1 associated protein X-1	Hax1	100761361	114.43	111.20	113.59	121.01	117.85	122.54	3519.70	3467.98	3562.31	3734.04	3663.85	3715.31
hemoglobin, epsilon 1	Hbe1	100768374	0.00	0.00	0.00	0.00	0.00	0.00	0.00	0.00	0.00	0.00	0.00	0.00
heparin-binding EGF-like growth factor	Hbegf	100689423	6.27	6.81	5.73	6.39	6.41	6.87	322.80	344.97	305.55	322.18	330.16	348.63
HMG-box transcription factor 1	Hbp1	100766157	56.27	57.21	61.25	62.31	65.15	64.94	3149.05	3141.31	3419.47	3428.09	3594.51	3572.31
HBS1-like (S. cerevisiae)	Hbs1l	100750806	48.66	49.28	54.19	56.85	57.68	55.33	3805.29	3887.98	4315.01	4440.98	4499.09	4371.45
hemoglobin, zeta	Hbz	100758314	0.00	0.00	0.00	0.00	0.00	0.00	0.76	0.00	0.00	0.00	0.00	0.00
hydroxycarboxylic acid receptor 1	Hcar1	100766347	0.00	0.00	0.00	0.00	0.00	0.00	0.00	0.00	0.00	0.00	0.00	0.00
hydroxycarboxylic acid receptor 2	Hcar2	100765472	0.00	0.00	0.00	0.00	0.00	0.00	0.00	0.00	0.00	0.00	0.00	0.00
host cell factor C1 (VP16-accessory protein)	Hcfc1	100774489	46.59	44.22	46.14	42.99	32.62	34.98	8671.66	8208.77	8653.62	7946.71	6082.61	6524.93
host cell factor C1 regulator 1 (XPO1 dependent)	Hcfc1r1	100768272	186.25	187.90	163.05	122.69	99.96	113.42	3063.22	3101.05	2655.58	1987.55	1650.80	1833.82
host cell factor C2	Hcfc2	100774776	6.06	6.24	5.24	6.43	7.36	7.23	985.88	1021.18	870.70	1033.76	1185.06	1152.45
hemopoietic cell kinase	Hck	100773315	0.00	0.02	0.00	0.00	0.00	0.04	0.00	1.83	1.24	0.00	1.03	1.87
hematopoietic cell-specific Lyn substrate 1	Hcls1	100764646	0.00	0.00	0.00	0.00	0.00	0.03	0.00	0.00	0.00	0.00	0.00	0.93
hyperpolarization activated cyclic nucleotide-gated potassium channel 1	Hcn1	100753109	0.00	0.00	0.00	0.00	0.00	0.01	0.00	0.00	0.00	0.00	0.00	0.93
hyperpolarization activated cyclic nucleotide-gated potassium channel 2	Hcn2	100751563	0.01	0.02	0.00	0.00	0.00	0.00	0.76	0.92	0.00	0.00	0.00	0.00
hyperpolarization activated cyclic nucleotide-gated potassium channel 3	Hcn3	100772247	0.83	0.67	0.63	0.69	0.66	0.65	87.35	69.54	64.59	70.69	67.27	68.23
hyperpolarization activated cyclic nucleotide-gated potassium channel 4	Hcn4	100758754	0.00	0.00	0.00	0.00	0.00	0.00	0.00	0.00	0.00	0.00	0.00	0.00
hypocretin (orexin) neuropeptide precursor	Hcrt	100754701	0.00	0.00	0.00	0.00	0.00	0.00	0.00	0.00	0.00	0.00	0.00	0.00
hypocretin (orexin) receptor 1	Hcrtr1	100773491	0.14	0.07	0.07	0.07	0.10	0.04	8.35	7.32	6.21	8.11	7.24	3.74
hypocretin (orexin) receptor 2	Hcrtr2	100762569	0.02	0.00	0.00	0.00	0.03	0.00	1.52	2.75	0.00	0.00	1.03	0.00
histone deacetylase 1	Hdac1	100752721	83.05	83.39	77.71	85.31	85.55	87.91	4986.37	5115.04	4733.59	5152.56	5166.65	5258.45
histone deacetylase 10	Hdac10	100758596	0.01	0.00	0.00	0.00	0.00	0.00	2.28	0.00	0.00	0.00	0.00	0.00
histone deacetylase 11	Hdac11	100772691	0.00	0.00	0.00	0.00	0.00	0.00	0.00	0.00	0.00	0.00	0.00	0.00
histone deacetylase 2	Hdac2	100764557	89.81	91.82	84.73	96.74	100.45	95.38	4610.40	4811.25	4452.88	4932.36	5157.34	4839.72
histone deacetylase 3	Hdac3	100752639	22.63	22.81	22.95	24.60	26.17	28.24	1315.52	1323.14	1329.03	1384.91	1447.95	1577.72
histone deacetylase 4	Hdac4	100767877	14.00	13.02	16.00	13.72	11.26	11.99	1767.45	1680.92	2075.53	1777.78	1486.24	1553.42
histone deacetylase 5	Hdac5	100689350	19.88	18.94	14.96	10.95	8.19	9.60	2179.12	2100.00	1679.30	1218.02	913.89	1044.03

histone deacetylase 6	Hdac6	100763688	27.71	26.27	28.40	28.15	27.62	28.67	3349.57	3252.03	3511.38	3407.22	3350.25	3445.19
histone deacetylase 7	Hdac7	100766171	6.81	6.40	5.52	5.11	4.17	4.10	785.36	743.01	642.16	586.41	483.34	483.22
histone deacetylase 9	Hdac9	100757696	0.21	0.23	0.14	0.11	0.33	0.27	27.34	31.11	16.15	16.22	36.22	30.84
histidine decarboxylase	Hdc	100753889	0.05	0.06	0.04	0.02	0.00	0.02	4.56	3.66	2.48	1.16	0.00	0.93
HD domain containing 2	Hddc2	100753668	62.26	55.84	26.18	27.54	25.95	26.80	1314.00	1235.30	561.42	584.10	547.51	562.67
HD domain containing 3	Hddc3	100764720	67.02	64.68	79.79	72.13	66.97	75.35	1576.80	1542.75	1873.07	1644.51	1547.30	1706.71
hepatoma-derived growth factor	Hdgf	100761454	269.04	276.83	310.86	317.99	305.13	321.14	8756.73	8981.97	9761.56	9864.73	8977.47	10042.09
haloacid dehalogenase-like hydrolase domain containing 1	Hdhd1	100774390	10.15	10.21	10.89	10.78	10.19	10.14	672.95	682.62	726.62	720.85	668.60	675.77
haloacid dehalogenase-like hydrolase domain containing 2	Hdhd2	100772344	14.22	14.73	15.64	15.23	16.75	17.27	793.72	823.53	871.94	846.01	944.94	969.25
haloacid dehalogenase-like hydrolase domain containing 3	Hdhd3	100752613	3.87	2.85	4.37	4.12	3.93	3.79	120.77	87.84	135.39	124.00	116.95	114.03
high density lipoprotein binding protein	Hdlbp	100772142	93.53	93.30	92.90	101.23	99.79	100.57	17408.64	17600.67	17647.57	18986.58	18609.05	18663.48
highly divergent homeobox	Hdx	100754145	0.00	0.02	0.00	0.00	0.00	0.00	0.00	0.92	0.00	0.00	0.00	0.00
HEAT repeat containing 1	Heatr1	100763985	35.85	34.60	34.43	37.64	38.50	39.24	7374.37	7156.48	7127.09	7665.10	7827.60	8026.01
HEAT repeat containing 2	Heatr2	100771212	10.72	9.90	10.46	9.59	7.64	8.74	947.15	883.01	948.95	854.12	677.92	762.69
HEAT repeat containing 3	Heatr3	100756422	22.37	22.42	21.64	22.81	24.75	22.62	2081.90	2121.05	2022.12	2080.26	2240.75	2082.44
HEAT repeat containing 4	Heatr4	100752922	3.35	3.71	4.01	3.73	4.13	4.45	519.52	584.71	591.23	574.82	651.01	705.68
HEAT repeat containing 5A	Heatr5a	100764339	11.46	11.60	10.74	12.11	15.16	14.69	1677.82	1668.11	1622.17	1784.74	2010.98	2031.04
HEAT repeat containing 5B	Heatr5b	100764477	7.12	6.72	6.33	6.96	7.19	7.13	1522.12	1473.20	1396.11	1520.50	1556.62	1534.73
HEAT repeat containing 6	Heatr6	100773538	4.60	4.41	2.06	2.03	1.97	2.11	602.31	591.11	276.99	270.03	256.68	288.81
HEAT repeat containing 9	Heatr9	100751967	0.00	0.00	0.00	0.00	0.00	0.00	1.52	2.75	0.00	0.00	0.00	0.00
heme binding protein 1	Hebp1	100752514	32.97	31.56	35.96	34.20	32.68	37.94	883.34	839.09	951.44	893.53	863.18	999.16
heme binding protein 2	Hebp2	100771345	0.00	0.00	0.00	0.00	0.00	0.00	0.00	0.00	0.00	0.00	0.00	0.00
headcase homolog (Drosophila)	Heca	100774249	9.84	9.98	14.05	15.30	14.46	14.55	1336.03	1346.01	1923.99	2074.47	1950.95	1979.63
HECT domain containing E3 ubiquitin protein ligase 1	Hectd1	100762999	30.32	29.06	30.42	36.18	35.08	32.45	7829.33	7482.23	7848.75	9227.32	8900.88	8340.06
HECT domain containing E3 ubiquitin protein ligase 2	Hectd2	100769372	3.11	3.15	3.53	3.87	4.80	4.39	424.58	422.75	464.54	504.13	652.04	595.38
HECT domain containing E3 ubiquitin protein ligase 3	Hectd3	100762194	29.74	29.62	35.14	32.99	31.70	32.15	2790.55	2810.07	3343.70	3072.30	2965.24	3014.31
HECT domain containing E3 ubiquitin protein ligase 4	Hectd4	100771362	5.27	5.13	3.93	3.87	3.94	3.89	2503.44	2448.63	1910.33	1857.75	1899.20	1863.73
HECT, C2 and WW domain containing E3 ubiquitin protein ligase 1	Hecw1	100752038	0.01	0.01	0.00	0.00	0.00	0.00	1.52	1.83	0.00	0.00	0.00	0.00
HECT, C2 and WW domain containing E3 ubiquitin protein ligase 2	Hecw2	100767617	0.04	0.06	0.01	0.02	0.05	0.03	9.11	14.64	3.73	5.79	11.38	6.54
heart development protein with EGF-like domains 1	Heg1	100764801	17.39	16.73	11.57	11.82	8.10	8.00	3102.72	3035.17	2109.06	2132.41	1453.12	1452.48
helicase (DNA) B	Helb	100771259	4.77	4.95	4.67	5.57	6.42	6.82	692.70	707.32	681.91	800.81	918.03	983.27
helicase, lymphoid-specific	Hells	100754676	28.04	30.34	34.31	39.67	41.21	36.15	2254.31	2421.18	2725.14	3107.06	3282.98	2878.78
helicase, POLQ-like	Helq	100767150	7.20	7.52	7.23	7.45	8.86	8.44	747.39	774.12	782.51	781.11	926.31	898.22
helt bHLH transcription factor	Helt	100751200	0.00	0.00	0.00	0.00	0.00	0.00	0.00	0.00	0.00	0.00	0.00	0.00
helicase with zinc finger	Helz	100766882	4.90	4.82	4.24	4.53	4.53	4.24	937.27	927.84	819.78	879.62	876.63	829.05

helicase with zinc finger 2, transcriptional coactivator	Helz2	100767179	6.22	5.43	5.00	4.76	4.13	4.65	1866.95	1649.81	1527.77	1456.76	1259.58	1406.68
hemogen	Hemgn	100770706	0.04	0.00	0.09	0.03	0.00	0.00	1.52	0.92	3.73	3.48	0.00	0.93
HemK methyltransferase family member 1	Hemk1	100750477	6.62	6.31	5.71	5.44	5.71	5.89	364.58	351.37	319.22	327.97	341.55	357.98
HEPACAM family member 2	Hepacam2	100765391	0.00	0.00	0.00	0.00	0.00	0.00	0.00	0.00	0.00	0.00	0.00	0.00
hephaestin	Heph	100753439	0.01	0.01	0.01	0.03	0.02	0.00	1.52	1.83	2.48	4.64	2.07	0.00
hephaestin-like 1	Hephl1	100752039	0.02	0.02	0.03	0.01	0.04	0.04	8.35	10.07	8.69	12.75	14.49	11.22
HECT and RLD domain containing E3 ubiquitin protein ligase family member 1	Herc1	100772726	6.39	6.34	5.93	6.45	6.81	6.82	3013.85	3047.98	2865.49	3095.48	3268.49	3262.93
HECT and RLD domain containing E3 ubiquitin protein ligase 3	Herc3	100752767	2.39	2.69	2.73	3.49	3.73	3.41	188.37	213.20	212.40	268.87	287.73	268.25
HECT and RLD domain containing E3 ubiquitin protein ligase 4	Herc4	100758073	21.50	22.26	22.00	25.54	29.53	27.64	2492.05	2591.37	2542.55	2932.07	3375.09	3178.81
HECT and RLD domain containing E3 ubiquitin protein ligase family member 6	Herc6	100767771	0.36	1.09	0.28	0.04	0.00	0.00	24.31	18.30	2.48	3.48	1.03	0.93
homocysteine-inducible, endoplasmic reticulum stress-inducible, ubiquitin-like domain member 1	Herpud1	100758367	47.37	45.88	50.48	51.73	45.45	48.35	2533.06	2444.05	2716.44	2740.85	2410.48	2591.84
HERPUD family member 2	Herpud2	100772076	16.80	16.34	22.47	25.16	24.96	24.55	1144.63	1146.54	1543.91	1711.72	1710.83	1674.93
hes family bHLH transcription factor 1	Hes1	100752756	6.69	8.08	4.07	4.84	7.62	6.97	94.94	109.80	55.89	64.90	101.43	89.73
hes family bHLH transcription factor 2	Hes2	100756407	0.00	0.00	0.00	0.00	0.00	0.00	0.00	0.00	0.00	0.00	0.00	0.00
hes family bHLH transcription factor 3	Hes3	100754865	0.18	0.00	0.00	0.00	0.00	0.00	3.04	0.92	1.24	1.16	0.00	0.00
hes family bHLH transcription factor 6	Hes6	100772285	11.26	11.75	11.76	11.48	9.32	10.18	395.72	400.78	403.68	381.28	309.46	341.15
HESX homeobox 1	Hesx1	100770203	0.00	0.00	0.00	0.00	0.00	0.00	0.00	0.00	0.00	0.00	0.00	0.00
hexosaminidase A (alpha polypeptide)	Hexa	100750552	16.21	15.01	25.46	24.63	27.95	26.86	912.21	859.22	1438.34	1386.07	1554.55	1514.16
hexosaminidase (glycosyl hydrolase family 20, catalytic domain) containing	Hexdc	100773287	17.72	16.36	6.00	5.25	4.38	4.95	978.29	907.71	317.97	289.73	246.33	263.58
hexamethylene bis-acetamide inducible 1	Hexim1	100760203	18.93	18.04	10.40	7.69	6.46	6.17	1199.31	1150.20	660.79	491.38	415.03	395.37
hexamethylene bis-acetamide inducible 2	Hexim2	100760493	2.93	2.89	1.85	1.16	1.05	1.48	177.73	184.84	106.82	76.49	69.34	91.60
hes-related family bHLH transcription factor with YRPW motif 1	Hey1	100752534	0.00	0.00	0.00	0.00	0.00	0.00	0.00	0.00	0.00	0.00	0.00	0.00
hes-related family bHLH transcription factor with YRPW motif 2	Hey2	100755611	0.00	0.00	0.00	0.00	0.00	0.00	0.00	0.00	0.00	0.00	0.00	0.00
hes-related family bHLH transcription factor with YRPW motif-like	Heyl	100771786	0.00	0.00	0.00	0.00	0.00	0.00	0.00	0.00	0.00	0.00	0.00	0.00
hemochromatosis	Hfe	100762132	5.37	5.53	6.23	6.73	7.39	7.10	374.45	398.95	434.73	467.04	521.63	517.81
hemochromatosis type 2 (juvenile)	Hfe2	100773682	0.03	0.04	0.00	0.05	0.00	0.00	0.00	0.00	0.00	0.00	0.00	0.00
HFM1, ATP-dependent DNA helicase homolog (S. cerevisiae)	Hfm1	100761873	0.00	0.00	0.01	0.00	0.00	0.00	0.76	0.00	1.24	0.00	0.00	0.93
homogentisate 1,2-dioxygenase	Hgd	100762426	0.00	0.05	0.00	0.00	0.00	0.00	0.76	1.83	0.00	0.00	0.00	0.00
hepatocyte growth factor (hepapoietin A; scatter factor)	Hgf	100764472	2.49	2.65	2.20	2.78	4.06	3.39	458.76	490.46	407.40	511.08	725.53	622.49
HGF activator	Hgfac	100765604	0.00	0.00	0.02	0.02	0.00	0.02	3.04	0.00	2.48	5.79	1.03	0.93

hepatocyte growth factor-regulated tyrosine kinase substrate	Hgs	100765681	79.80	76.57	44.37	36.50	26.04	29.40	5896.30	5676.87	3408.29	2777.93	1915.76	2110.48
heparan-alpha-glucosaminide N-acetyltransferase	Hgsnat	100752593	11.99	12.36	14.11	15.76	16.75	15.89	1044.37	1098.04	1234.63	1340.87	1424.14	1352.47
hedgehog acyltransferase	Hhat	100761049	0.03	0.02	0.00	0.00	0.00	0.00	2.28	1.83	0.00	0.00	0.00	0.00
hedgehog acyltransferase-like	Hhatl	100771282	0.00	0.00	0.03	0.00	0.00	0.00	0.00	0.00	1.24	0.00	0.00	0.93
hematopoietically expressed homeobox	Hhex	100751343	0.00	0.00	0.00	0.00	0.00	0.00	0.00	0.00	0.00	0.00	0.00	0.00
hedgehog interacting protein	Hhip	100771283	0.00	0.00	0.00	0.00	0.00	0.00	0.00	0.00	0.00	0.00	0.00	0.00
HHIP-like 1	Hhipl1	100769577	2.94	2.65	3.10	3.11	3.26	3.41	448.89	414.51	475.72	469.36	481.27	510.33
HHIP-like 2	Hhipl2	100755908	0.34	0.19	0.43	0.58	0.35	0.39	31.14	19.22	45.96	56.79	45.54	44.86
HERV-H LTR-associating 2	Hhla2	103161012	0.00	0.00	0.00	0.00	0.00	0.00	0.00	0.00	0.00	0.00	0.00	0.00
hippocampus abundant transcript 1	Hiat1	100772600	25.68	24.15	26.76	27.96	28.44	27.46	2048.48	1946.28	2119.00	2233.24	2290.42	2214.23
hippocampus abundant transcript-like 1	Hiatl1	100752082	13.72	14.69	14.51	15.73	18.85	17.73	1323.88	1413.73	1404.80	1513.55	1787.42	1692.69
3-hydroxyisobutyrate dehydrogenase	Hibadh	100760581	38.29	39.84	46.41	46.81	52.05	52.20	1828.97	1929.81	2239.48	2213.54	2428.08	2454.44
3-hydroxyisobutyryl-CoA hydrolase	Hibch	100759780	18.00	17.97	17.31	17.90	20.08	19.04	1128.68	1171.24	1096.76	1095.18	1256.47	1189.83
hypermethylated in cancer 1	Hic1	100767977	5.85	5.69	5.34	2.58	1.62	1.91	532.44	522.48	490.62	236.42	149.04	176.65
hypermethylated in cancer 2	Hic2	100751153	1.67	1.80	1.40	1.23	1.16	1.34	214.95	229.67	182.59	158.77	153.18	178.52
HID1 domain containing	Hid1	100772114	1.33	1.30	1.04	1.22	1.17	1.21	132.16	129.93	108.06	113.57	108.67	116.83
hypoxia inducible factor 1, alpha subunit (basic helix-loop-helix transcription factor)	Hif1a	100751293	27.23	28.31	29.50	32.21	33.53	31.57	2928.02	3040.66	3173.53	3429.24	3567.60	3365.75
hypoxia inducible factor 1, alpha subunit inhibitor	Hif1an	100752342	12.81	12.01	11.89	12.07	12.79	13.35	556.74	490.46	494.35	507.61	523.70	544.91
hypoxia inducible factor 3, alpha subunit	Hif3a	100761594	0.00	0.02	0.00	0.00	0.00	0.00	0.00	0.00	0.00	0.00	0.00	0.00
HIG1 hypoxia inducible domain family, member 1A	Higd1a	100766684	80.35	73.54	68.93	72.83	72.92	83.74	660.04	616.73	580.05	578.30	599.26	657.07
HIG1 hypoxia inducible domain family, member 1B	Higd1b	100764860	0.32	0.27	0.22	0.17	0.25	0.17	29.62	32.03	19.87	17.38	21.73	19.63
HIG1 hypoxia inducible domain family, member 2A	Higd2a	100750940	22.82	21.50	20.29	14.55	12.28	13.00	352.43	321.18	317.97	223.67	182.16	192.54
hypoxia inducible lipid droplet-associated	Hilpda	100750353	23.63	21.94	17.58	19.04	17.21	18.31	588.64	549.02	440.94	469.36	422.27	453.31
histone H4 transcription factor	Hinfp	100756963	23.50	23.55	22.87	24.66	21.78	23.18	1840.36	1863.92	1824.63	1966.69	1716.01	1822.61
histidine triad nucleotide binding protein 1	Hint1	100764340	98.03	96.78	101.69	96.09	95.23	92.87	1182.60	1159.35	1227.18	1179.78	1166.43	1146.84
histidine triad nucleotide binding protein 2	Hint2	100754892	17.01	15.65	16.42	15.79	14.19	13.74	235.46	217.78	227.30	199.33	180.09	181.33
histidine triad nucleotide binding protein 3	Hint3	100774127	5.75	6.43	2.32	3.46	3.67	3.28	502.81	506.93	219.85	280.46	300.15	284.14
huntingtin interacting protein 1	Hip1	100757209	28.38	27.41	33.66	36.02	49.57	51.48	6652.80	6492.16	7979.17	8403.33	11476.96	11976.86
huntingtin interacting protein 1 related	Hip1r	100766930	9.44	8.97	10.45	8.40	6.05	7.02	1343.62	1292.94	1485.54	1198.32	890.09	1003.84
homeodomain interacting protein kinase 1	Hipk1	100759237	9.17	10.08	10.03	10.04	11.11	10.93	1222.10	1315.82	1352.63	1320.01	1430.35	1436.59
homeodomain interacting protein kinase 2	Hipk2	100767468	5.58	5.35	5.38	5.29	5.64	5.81	2323.43	2226.28	2251.90	2197.31	2338.03	2414.25
homeodomain interacting protein kinase 3	Hipk3	100772418	18.38	18.12	19.86	22.58	22.81	21.69	3765.04	3738.83	4098.89	4642.63	4649.16	4469.59

homeodomain interacting protein kinase 4	Hipk4	100763406	0.02	0.00	0.00	0.00	0.00	0.00	3.04	0.00	1.24	0.00	0.00	2.80
HIRA interacting protein 3	Hirip3	100773932	83.04	82.81	76.89	78.96	81.24	82.95	5495.27	5438.04	5087.59	5196.60	5317.76	5598.67
human immunodeficiency virus type I enhancer binding protein 1	Hivep1	100768788	7.10	7.11	5.24	6.28	6.14	5.71	1825.93	1837.39	1366.30	1609.74	1562.83	1475.84
human immunodeficiency virus type I enhancer binding protein 2	Hivep2	100760180	7.23	7.45	6.43	7.22	7.30	7.22	2012.02	2078.04	1808.48	2025.79	2019.26	2018.89
human immunodeficiency virus type I enhancer binding protein 3	Hivep3	100755083	2.75	2.42	1.39	1.43	1.26	1.17	919.80	814.38	471.99	484.43	421.24	401.91
Holliday junction recognition protein	Hjurp	100755715	13.98	14.31	13.13	15.53	15.98	15.93	1420.34	1474.12	1325.31	1515.87	1574.21	1616.98
hexokinase 1	Hk1	100765413	51.60	52.73	46.91	45.16	41.87	43.71	5549.19	5719.87	5177.02	4896.44	4615.00	4739.71
hexokinase 2	Hk2	100772205	16.11	15.98	12.44	12.25	12.61	12.70	2444.20	2393.73	1871.82	1858.91	1901.27	1951.59
hexokinase 3 (white cell)	Hk3	100765901	0.03	0.01	0.00	0.03	0.03	0.04	2.28	1.83	0.00	2.32	2.07	2.80
hexokinase domain containing 1	Hkdc1	100765703	0.08	0.04	0.02	0.11	0.01	0.01	6.08	2.75	1.24	8.11	1.03	0.93
holocarboxylase synthetase (biotin-(proprionyl-CoA-carboxylase (ATP-hydrolysing)) ligase)	Hlcs	100752840	5.32	5.50	5.16	4.55	3.85	4.31	394.20	409.94	362.69	340.72	293.94	341.15
hepatic leukemia factor	Hlf	100759113	0.00	0.00	0.00	0.00	0.00	0.00	0.76	0.00	0.00	0.00	0.00	0.00
helicase-like transcription factor	Hltf	100751958	20.10	18.90	25.27	31.34	28.33	25.43	2096.33	2035.03	2745.01	3324.94	3007.67	2678.76
H2.0-like homeobox	Hlx	100756486	3.27	2.79	2.30	1.89	1.58	1.39	142.03	122.61	104.34	85.76	68.31	65.43
histocompatibility (minor) 13	Hm13	100757312	70.17	69.61	69.61	68.87	60.57	62.09	3113.35	3119.35	3082.86	2965.68	2694.07	2668.48
homeobox containing 1	Hmbox1	103160896	9.76	10.47	12.27	12.87	10.64	12.49	793.72	825.36	983.73	1046.50	920.10	986.08
hydroxymethylbilane synthase	Hmbs	100757819	29.00	27.90	33.52	35.41	35.93	37.51	1326.16	1313.99	1533.98	1629.44	1671.50	1709.51
5-hydroxymethylcytosine (hmC) binding, ES cell-specific	Hmces	100763397	22.63	20.64	23.38	22.59	20.40	21.06	938.03	880.26	1016.03	953.79	860.07	881.39
hemicentin 1	Hmcn1	100756726	0.01	0.01	0.00	0.00	0.00	0.00	3.04	1.83	0.00	0.00	0.00	0.93
hemicentin 2	Hmcn2	100758433	0.00	0.00	0.00	0.00	0.00	0.00	1.52	0.00	0.00	0.00	0.00	0.00
high mobility group 20A	Hmg20a	100759297	17.16	17.00	17.10	17.75	17.00	18.36	1732.51	1725.75	1733.95	1803.28	1705.66	1839.43
high mobility group 20B	Hmg20b	100771597	27.00	25.38	32.88	24.95	18.04	19.38	1362.61	1294.77	1635.83	1289.88	980.13	1016.92
high mobility group AT-hook 1	Hmga1	100689040	402.22	390.07	373.34	270.74	198.21	197.13	17950.19	17483.55	17392.94	12537.20	9282.79	9258.84
high mobility group AT-hook 2	Hmga2	103158819	100.19	88.73	114.22	126.42	132.90	132.34	536.23	497.78	575.09	564.39	607.54	625.29
high mobility group box 1	Hmgb1	100754755	386.18	414.44	375.09	390.96	410.49	384.65	7673.62	8203.28	7455.01	7514.44	8030.46	7567.09
high mobility group box 2	Hmgb2	100762338	127.34	131.24	143.76	147.31	143.50	134.53	3131.58	3278.57	3549.88	3626.26	3565.53	3321.82
high mobility group box 3	Hmgb3	100772377	0.00	0.00	0.00	0.00	0.00	0.05	0.00	0.00	0.00	0.00	0.00	0.93
high mobility group box 4	Hmgb4	100773406	0.00	0.00	0.00	0.00	0.00	0.00	0.00	0.00	0.00	0.00	0.00	0.00
3-hydroxymethyl-3-methylglutaryl-CoA lyase	Hmgcl	100755385	18.87	18.61	19.06	17.66	16.36	19.00	528.64	517.91	515.47	440.39	404.68	472.94
3-hydroxymethyl-3-methylglutaryl-CoA lyase-like 1	Hmgcll1	100763147	0.03	0.00	0.00	0.00	0.00	0.02	1.52	0.00	0.00	0.00	1.03	0.93
3-hydroxy-3-methylglutaryl-CoA reductase	Hmgcr	100756363	12.22	11.81	12.87	13.14	12.80	13.13	1591.99	1550.07	1679.30	1715.20	1654.94	1698.29
3-hydroxy-3-methylglutaryl-CoA synthase 1 (soluble)	Hmgcs1	100762235	10.98	10.15	14.08	15.57	14.09	14.80	1059.56	978.17	1363.81	1483.42	1338.24	1416.02
3-hydroxy-3-methylglutaryl-CoA synthase 2 (mitochondrial)	Hmgcs2	100774684	3.75	3.80	0.73	0.90	0.56	0.33	208.11	226.93	40.99	50.99	30.01	18.69
high mobility group nucleosome binding domain 1	Hmgn1	100754560	123.60	141.29	141.42	128.22	140.51	105.44	1041.33	1238.96	1098.00	995.51	1117.79	870.18

high mobility group nucleosomal binding domain 3	Hmgn3	100758486	58.64	63.64	42.19	44.45	52.07	45.37	1643.64	1766.01	1207.31	1301.47	1485.21	1322.56
high mobility group nucleosome binding domain 5	Hmgn5	100774904	140.44	138.00	128.06	143.37	130.26	121.15	6142.39	5979.74	5557.10	6209.49	5566.16	5208.91
HMG box domain containing 3	Hmgxb3	100764075	9.72	9.91	10.68	10.92	10.42	11.39	1350.46	1371.64	1491.75	1497.32	1442.77	1576.79
HMG box domain containing 4	Hmgxb4	100757455	13.05	12.51	12.94	14.55	15.65	16.48	1339.83	1331.37	1343.94	1497.32	1585.60	1675.86
histocompatibility (minor) HA-1	Hmha1	100764760	0.15	0.14	0.16	0.10	0.12	0.14	20.51	16.47	19.87	12.75	12.42	14.95
hyaluronan-mediated motility receptor (RHAMM)	Hmmr	100772538	12.08	12.25	10.35	14.02	15.24	13.60	1224.38	1224.31	1075.65	1408.09	1535.92	1358.08
heme oxygenase (decycling) 1	Hmox1	100756872	71.31	71.19	84.77	76.33	72.58	82.11	3440.71	3465.23	4116.28	3655.23	3480.66	3925.61
heme oxygenase (decycling) 2	Hmox2	100773727	32.03	32.03	38.99	38.31	41.20	43.86	1186.40	1189.54	1453.24	1464.87	1536.95	1621.65
H6 family homeobox 2	Hmx2	100768285	0.00	0.00	0.00	0.00	0.00	0.00	0.00	0.00	0.00	0.00	0.00	0.00
H6 family homeobox 3	Hmx3	100767995	0.00	0.00	0.00	0.00	0.00	0.00	0.00	0.00	0.00	0.00	0.00	0.00
hematological and neurological expressed 1	Hn1	100756788	107.27	105.20	120.88	104.98	76.38	84.20	3236.40	3186.15	3675.33	3127.92	2297.67	2530.15
hematological and neurological expressed 1-like	Hn1l	100765136	119.34	125.01	136.13	136.38	142.79	146.78	9200.30	9710.34	10624.81	10516.04	10911.86	11324.46
HNF1 homeobox A	Hnf1a	100774677	3.15	3.14	3.76	3.53	3.58	3.37	306.85	295.56	339.09	314.07	309.46	299.09
HNF1 homeobox B	Hnf1b	100771619	0.00	0.00	0.00	0.00	0.00	0.02	1.52	0.00	0.00	0.00	0.00	1.87
hepatocyte nuclear factor 4, alpha	Hnf4a	100770083	0.85	0.69	0.16	0.17	0.06	0.04	113.17	94.25	21.12	22.02	8.28	4.67
hepatocyte nuclear factor 4, gamma	Hnf4g	100751997	0.00	0.00	0.00	0.00	0.00	0.00	0.00	0.00	0.00	0.00	0.00	0.00
histamine N-methyltransferase	Hnmt	100761300	0.00	0.04	0.00	0.00	0.00	0.00	0.00	0.92	0.00	0.00	0.00	0.00
heterogeneous nuclear ribonucleoprotein A1	Hnrnpa1	100769923	493.77	504.99	456.17	493.57	498.18	470.50	25512.92	26216.63	23217.09	24929.53	25525.87	24089.24
heterogeneous nuclear ribonucleoprotein A2/B1	Hnrnpa2 b1	100759302	372.61	384.55	333.42	346.15	395.37	364.29	18752.26	19366.68	16718.49	17273.70	19713.38	18136.33
heterogeneous nuclear ribonucleoprotein A3	Hnrnpa3	100757096	607.64	600.84	571.53	629.73	602.62	569.49	23546.48	23897.02	22863.09	24681.52	23678.42	21952.59
heterogeneous nuclear ribonucleoprotein A/B	Hnrnpab	100767538	335.56	368.39	393.47	401.30	408.74	432.45	6704.45	7285.50	7779.19	7283.81	7055.50	7349.31
heterogeneous nuclear ribonucleoprotein C (C1/C2)	Hnrnpc	100751290	239.48	247.81	268.46	269.57	269.56	263.62	12176.93	12655.83	13607.06	13552.41	13564.53	13243.33
heterogeneous nuclear ribonucleoprotein D (AU-rich element RNA binding protein 1, 37kDa)	Hnrnpd	100767887	328.45	356.60	351.32	364.53	388.65	374.33	11561.71	12951.38	12425.84	12552.26	13416.53	12547.01
heterogeneous nuclear ribonucleoprotein D-like	Hnrnpdl	100767030	79.88	82.57	79.36	84.92	80.05	75.44	5637.30	5944.06	5708.63	6232.67	5848.71	5446.32
heterogeneous nuclear ribonucleoprotein F	Hnrnpf	100771400	384.60	375.66	377.40	374.24	358.88	368.32	23315.58	22836.49	23118.96	22743.80	21747.13	22447.03
heterogeneous nuclear ribonucleoprotein H1 (H)	Hnrnph1	100769493	127.83	125.14	90.19	96.78	99.20	93.70	7814.90	7653.34	5518.59	5975.39	6110.56	5800.56
heterogeneous nuclear ribonucleoprotein H2 (H')	Hnrnph2	100773216	58.22	58.12	57.05	61.22	67.25	63.42	3933.65	3872.42	3803.27	4064.33	4399.73	4253.68
heterogeneous nuclear ribonucleoprotein H3 (2H9)	Hnrnph3	100756529	25.66	24.20	20.76	23.32	22.60	22.59	2318.87	2253.73	1946.35	2184.56	2153.81	2106.75
heterogeneous nuclear ribonucleoprotein K	Hnrnpk	100762670	292.21	298.44	292.52	291.75	296.15	294.80	19391.04	19999.89	19791.41	19413.07	19993.87	19874.82
heterogeneous nuclear ribonucleoprotein L	Hnrnpl	100768344	180.68	170.61	163.82	161.66	174.44	189.57	8439.24	8256.35	8041.27	7862.11	8359.58	8884.04
heterogeneous nuclear ribonucleoprotein L-like	Hnrnpll	100758670	68.35	66.02	63.23	67.40	74.44	73.23	4390.90	4214.64	4080.26	4270.62	4704.01	4630.35

heterogeneous nuclear ribonucleoprotein M	Hnrnpm	100753958	70.97	64.32	50.70	48.88	48.08	46.82	4184.30	3992.29	3112.67	3097.79	3032.51	2846.07
heterogeneous nuclear ribonucleoprotein R	Hnrnpr	100767855	72.71	75.66	82.34	85.60	88.74	83.91	5451.21	5712.55	6286.20	6425.05	6592.86	6269.76
heterogeneous nuclear ribonucleoprotein U (scaffold attachment factor A)	Hnrnpu	100767942	343.25	356.79	330.92	350.30	345.69	341.24	26066.63	27441.86	25488.86	26580.99	26208.96	25658.55
heterogeneous nuclear ribonucleoprotein U-like 1	Hnrnpul 1	100772778	60.95	64.07	58.90	58.17	52.69	53.06	2750.29	2938.17	2704.02	2634.22	2390.82	2428.27
heterogeneous nuclear ribonucleoprotein U-like 2	Hnrnpul 2	100756306	72.96	74.04	75.41	84.22	80.70	75.30	4525.33	4609.94	4706.27	5136.33	4962.76	4621.01
4-hydroxy-2-oxoglutarate aldolase 1	Hoga1	100759109	6.19	6.91	6.60	6.87	5.83	6.20	754.98	852.81	814.81	825.15	692.41	740.26
homer homolog 1 (Drosophila)	Homer1	100760219	8.79	9.36	8.90	9.10	8.86	8.68	1004.87	1079.74	1022.24	1038.39	1016.36	998.23
homer homolog 2 (Drosophila)	Homer2	100771332	0.00	0.05	0.00	0.00	0.11	0.00	0.00	0.92	0.00	0.00	2.07	0.00
homer homolog 3 (Drosophila)	Homer3	100764933	5.01	4.90	5.81	4.19	3.60	3.85	288.62	284.58	305.55	239.90	216.31	202.82
homeobox and leucine zipper encoding	Homez	100766522	2.00	1.91	1.87	2.14	2.25	2.17	169.38	161.96	158.99	181.95	188.37	185.06
hook microtubule-tethering protein 1	Hook1	100766023	0.95	1.02	0.15	0.14	0.24	0.21	151.91	160.13	23.60	19.70	38.29	32.71
hook microtubule-tethering protein 2	Hook2	100762520	6.46	6.15	6.72	4.91	4.19	4.63	672.19	660.65	737.80	515.72	469.88	478.55
hook microtubule-tethering protein 3	Hook3	100770791	6.70	6.89	7.41	8.10	9.56	8.99	1093.74	1116.34	1189.92	1288.72	1512.11	1419.76
HOP homeobox	Hopx	100757669	0.00	0.00	0.00	0.00	0.00	0.00	0.00	0.00	0.00	0.00	0.00	0.00
HORMA domain containing 1	Hormad 1	100765118	0.00	0.00	0.00	0.00	0.00	0.00	0.00	0.00	0.00	0.00	0.00	0.00
HORMA domain containing 2	Hormad 2	100753726	0.00	0.00	0.00	0.00	0.00	0.03	0.76	0.00	0.00	0.00	0.00	0.93
homeobox A1	Hoxa1	100759424	1.35	1.53	1.20	1.69	1.69	1.64	75.19	86.01	72.04	98.51	100.39	89.73
homeobox A10	Hoxa10	100762719	4.83	5.75	5.59	4.28	6.98	6.23	255.21	299.22	295.62	225.99	358.11	317.79
homeobox A11	Hoxa11	100763010	0.00	0.00	0.00	0.00	0.00	0.00	0.00	0.00	0.00	1.16	1.03	1.87
homeobox A2	Hoxa2	100761561	0.50	0.67	0.87	0.74	0.99	0.97	31.90	41.18	55.89	45.20	63.13	63.56
homeobox A3	Hoxa3	100761845	0.60	0.64	0.70	0.65	0.57	0.44	49.37	53.99	60.86	55.63	45.54	36.45
homeobox A4	Hoxa4	100762131	1.19	0.94	1.00	1.07	0.88	1.26	31.14	24.71	26.08	27.81	22.77	32.71
homeobox A5	Hoxa5	100762428	2.58	2.17	2.30	2.43	4.23	3.13	78.99	70.46	88.19	76.49	132.48	100.94
homeobox A6	Hoxa6	100764757	2.15	2.25	2.94	2.84	2.40	2.38	177.73	185.75	208.67	203.97	189.40	178.52
homeobox A7	Hoxa7	100759709	4.22	4.30	5.47	4.55	5.17	5.25	541.55	553.60	704.26	582.94	639.62	671.09
homeobox A9	Hoxa9	100759995	5.20	5.14	5.82	5.71	8.17	7.50	296.22	295.56	334.12	329.13	451.25	425.27
homeobox B1	Hoxb1	100751249	0.00	0.00	0.00	0.00	0.00	0.00	0.00	0.00	0.00	0.00	0.00	0.00
homeobox B13	Hoxb13	100773981	0.00	0.00	0.00	0.00	0.00	0.00	0.00	0.00	0.00	0.00	0.00	0.00
homeobox B2	Hoxb2	100750955	1.53	1.24	0.78	0.53	0.47	0.29	69.12	63.14	57.14	39.40	42.43	31.78
homeobox B3	Hoxb3	100750662	0.91	0.63	0.73	0.49	0.42	0.20	54.69	39.35	44.72	30.13	23.80	13.09
homeobox B4	Hoxb4	100750374	3.40	3.64	2.61	1.74	1.49	1.13	66.84	72.29	52.17	33.61	28.98	24.30
homeobox B5	Hoxb5	100774854	0.57	0.52	0.44	0.47	0.52	0.42	61.52	55.82	48.44	49.83	56.92	47.67
homeobox B6	Hoxb6	100774559	4.88	4.53	4.69	4.41	4.04	4.15	419.27	407.19	383.80	382.44	377.77	372.93
homeobox B7	Hoxb7	100774273	21.78	22.35	16.30	16.21	16.84	16.52	729.16	744.84	551.49	540.06	553.72	549.59
homeobox B8	Hoxb8	100761150	0.98	1.08	1.20	1.19	0.86	1.27	63.80	68.63	81.98	70.69	52.78	76.64

homeobox C10	Hoxc10	100764281	12.69	12.35	18.38	16.17	15.72	15.97	600.80	585.62	873.19	768.36	732.77	748.67
homeobox C11	Hoxc11	100771213	0.00	0.00	0.00	0.00	0.00	0.00	0.76	0.00	0.00	0.00	0.00	0.00
homeobox C12	Hoxc12	100771504	0.00	0.00	0.00	0.00	0.00	0.00	0.00	0.00	0.00	0.00	0.00	0.00
homeobox C13	Hoxc13	103159695	0.00	0.00	0.00	0.00	0.00	0.00	0.00	0.00	0.00	0.00	0.00	0.00
homeobox C4	Hoxc4	103158598	18.19	19.03	23.05	21.81	20.38	20.26	614.47	630.46	768.85	734.76	683.09	687.92
homeobox C5	Hoxc5	100763415	0.85	0.94	1.06	0.96	0.77	0.77	85.83	95.16	110.55	98.51	77.62	75.71
homeobox C6	Hoxc6	100763696	6.87	5.81	11.11	11.14	7.06	8.23	229.38	193.99	356.48	363.90	231.84	280.40
homeobox C8	Hoxc8	100770928	5.09	5.68	10.13	9.83	6.86	6.98	275.71	299.22	545.28	515.72	367.42	372.00
homeobox C9	Hoxc9	100763984	3.78	3.68	5.96	4.79	4.81	4.12	214.19	204.97	344.06	267.71	267.03	230.86
homeobox D1	Hoxd1	100757676	0.00	0.00	0.00	0.00	0.00	0.00	0.00	0.00	0.00	0.00	0.00	0.00
homeobox D10	Hoxd10	100752695	0.00	0.00	0.00	0.00	0.04	0.00	0.00	0.00	0.00	0.00	1.03	0.00
homeobox D12	Hoxd12	100752995	0.00	0.00	0.00	0.00	0.00	0.00	0.00	0.00	0.00	0.00	0.00	0.00
homeobox D13	Hoxd13	100753293	0.00	0.00	0.00	0.00	0.00	0.00	0.00	0.00	0.00	0.00	0.00	0.00
homeobox D3	Hoxd3	100757961	0.85	0.69	1.39	0.98	0.74	0.82	35.70	28.37	40.99	38.24	18.63	28.97
homeobox D4	Hoxd4	103161712	1.25	0.75	0.80	1.30	1.46	1.31	37.22	24.71	29.81	47.52	47.61	46.73
homeobox D8	Hoxd8	100758537	35.49	38.04	40.79	41.81	46.92	47.83	1576.04	1698.30	1834.56	1847.32	2077.22	2114.22
homeobox D9	Hoxd9	100758825	16.13	17.90	20.18	16.94	17.83	16.74	439.01	478.56	547.76	438.07	456.43	432.75
haptoglobin	Hp	100756921	0.03	0.02	0.05	0.00	0.06	0.02	2.28	0.92	6.21	1.16	5.17	4.67
heterochromatin protein 1, binding protein 3	Hp1bp3	100765369	86.25	88.92	104.35	106.95	113.77	117.67	8120.99	8485.11	9982.65	10120.85	10749.37	11056.21
hippocalcin	Hpca	100750673	0.00	0.00	0.00	0.00	0.00	0.00	0.76	0.00	0.00	1.16	0.00	0.00
hippocalcin-like 1	Hpcal1	100752247	9.41	9.63	11.55	9.44	6.69	8.56	379.01	382.48	453.36	372.01	262.89	334.61
hippocalcin like 4	Hpcal4	100772357	0.00	0.00	0.00	0.00	0.00	0.00	0.00	0.00	0.00	0.00	0.00	0.00
4-hydroxyphenylpyruvate dioxygenase	Hpd	100768220	0.03	0.04	0.01	0.00	0.00	0.00	1.52	1.83	1.24	0.00	0.00	0.93
4-hydroxyphenylpyruvate dioxygenase-like	Hpdl	100752686	0.00	0.00	0.00	0.00	0.00	0.00	0.00	0.00	0.00	0.00	0.00	0.00
hydroxyprostaglandin dehydrogenase 15-(NAD)	Hpgd	100755116	0.00	0.00	0.00	0.00	0.00	0.00	0.00	0.00	0.00	0.00	0.00	0.00
hematopoietic prostaglandin D synthase	Hpgds	100768035	0.00	0.00	0.00	0.00	0.00	0.00	0.76	0.92	0.00	1.16	0.00	0.00
hepsin	Hpn	100757163	0.00	0.00	0.00	0.00	0.00	0.00	0.00	0.00	0.00	0.00	0.00	0.00
hypoxanthine phosphoribosyltransferase 1	Hprt1	100769768	38.94	42.43	41.28	42.14	48.15	49.27	1277.54	1398.17	1366.30	1337.39	1545.23	1561.83
Hermansky-Pudlak syndrome 1	Hps1	100761713	8.47	7.68	8.73	7.71	5.71	6.14	811.95	760.39	858.28	774.16	584.77	614.08
Hermansky-Pudlak syndrome 3	Hps3	100752245	3.48	3.44	4.99	5.82	6.08	5.36	392.68	384.31	563.91	659.43	665.50	594.45
Hermansky-Pudlak syndrome 4	Hps4	100774207	8.62	7.53	10.33	9.15	7.69	8.09	862.08	794.25	1075.65	939.88	791.76	835.59
Hermansky-Pudlak syndrome 5	Hps5	100750896	11.59	11.44	13.69	14.61	16.57	15.90	1497.05	1496.08	1767.49	1884.40	2121.72	2025.43
Hermansky-Pudlak syndrome 6	Hps6	100773245	5.61	6.00	6.74	5.17	3.02	3.64	398.76	428.24	480.69	368.54	212.17	260.77
heparanase	Hpse	100755610	14.77	15.13	12.46	14.15	20.08	21.15	761.82	772.29	648.37	719.69	1057.76	1112.26
heparanase 2	Hpse2	100762007	0.00	0.00	0.00	0.00	0.00	0.00	0.00	0.92	0.00	0.00	0.00	0.00
hemopexin	Hpx	100753194	0.18	0.02	0.09	0.14	0.07	0.17	12.15	0.92	3.73	6.95	3.10	8.41
hair growth associated	Hr	100765886	0.95	1.04	0.14	0.19	0.14	0.16	143.55	158.30	22.36	31.29	22.77	22.43

Harvey rat sarcoma viral oncogene homolog	Hras	100773737	37.30	37.24	40.00	34.74	28.95	33.60	1193.24	1217.91	1326.55	1112.56	919.07	1065.52
HRAS-like suppressor	Hrasls	100750510	0.00	0.00	0.00	0.00	0.00	0.00	0.00	0.00	0.00	0.00	0.00	0.00
HRAS-like suppressor family, member 5	Hrasls5	100773487	0.00	0.00	0.00	0.00	0.00	0.00	0.00	0.00	0.00	0.00	0.00	0.00
histidine rich calcium binding protein	Hrc	100771220	0.05	0.06	0.06	0.06	0.09	0.09	4.56	3.66	4.97	4.64	6.21	6.54
histidine rich carboxyl terminus 1	Hrct1	100758508	0.00	0.00	0.00	0.00	0.00	0.00	0.00	0.00	0.00	0.00	0.00	0.00
histidine-rich glycoprotein	Hrg	100764582	0.00	0.00	0.00	0.00	0.00	0.00	0.00	0.00	0.00	0.00	0.00	0.00
histamine receptor H1	Hrh1	100760663	0.06	0.00	0.00	0.02	0.00	0.00	3.80	0.00	0.00	1.16	0.00	0.00
histamine receptor H2	Hrh2	100760378	0.00	0.00	0.00	0.00	0.00	0.00	0.00	0.00	0.00	0.00	0.00	0.00
histamine receptor H3	Hrh3	100764853	0.00	0.00	0.00	0.00	0.00	0.00	0.00	0.00	0.00	0.00	0.00	0.00
histamine receptor H4	Hrh4	100774770	0.00	0.00	0.00	0.00	0.00	0.00	0.00	0.00	0.00	0.00	0.00	0.00
heat-responsive protein 12	Hrsp12	100754697	19.30	17.79	22.29	22.23	23.10	22.67	437.49	405.36	481.93	484.43	492.65	500.98
HCLS1 binding protein 3	Hs1bp3	100774467	8.73	8.45	9.71	8.55	8.28	9.91	782.33	748.50	854.56	762.57	733.81	872.98
heparan sulfate 2-O-sulfotransferase 1	Hs2st1	100760748	37.72	41.76	30.25	40.39	40.40	37.01	1788.71	1891.37	1706.63	1856.59	2019.26	1946.92
heparan sulfate (glucosamine) 3-O-sulfotransferase 1	Hs3st1	100759378	0.00	0.00	0.00	0.00	0.00	0.00	0.00	0.00	0.00	0.00	0.00	0.00
heparan sulfate (glucosamine) 3-O-sulfotransferase 2	Hs3st2	100765580	0.00	0.00	0.00	0.00	0.00	0.00	0.00	0.00	0.00	0.00	0.00	0.00
heparan sulfate (glucosamine) 3-O-sulfotransferase 4	Hs3st4	100770573	0.00	0.00	0.00	0.00	0.00	0.00	0.00	0.00	0.00	0.00	0.00	0.00
heparan sulfate (glucosamine) 3-O-sulfotransferase 5	Hs3st5	100763965	0.00	0.00	0.00	0.00	0.00	0.00	0.00	0.00	0.00	0.00	1.03	0.00
heparan sulfate (glucosamine) 3-O-sulfotransferase 6	Hs3st6	100766299	0.00	0.00	0.00	0.00	0.00	0.00	0.00	0.00	0.00	0.00	0.00	0.00
heparan sulfate 6-O-sulfotransferase 1	Hs6st1	100689173	22.80	22.09	19.10	16.59	15.63	16.40	2005.94	1948.11	1691.72	1461.40	1366.18	1448.74
heparan sulfate 6-O-sulfotransferase 2	Hs6st2	100763313	0.00	0.00	0.00	0.00	0.00	0.00	0.00	0.00	0.00	0.00	0.00	0.00
heparan sulfate 6-O-sulfotransferase 3	Hs6st3	100765877	0.09	0.34	0.00	0.00	0.00	0.00	5.32	6.41	0.00	0.00	0.00	0.00
heat shock factor binding protein 1	Hsbp1	100751023	115.67	115.79	126.65	123.07	140.72	142.98	3742.25	3740.66	4136.15	3925.26	4443.20	4509.78
heat shock factor binding protein 1-like 1	Hsbp1l1	100763672	0.00	0.00	0.00	0.00	0.00	0.00	0.00	0.00	0.00	0.00	0.00	0.00
HscB mitochondrial iron-sulfur cluster co-chaperone	Hscb	100769569	33.60	33.02	28.27	26.89	27.28	28.57	647.13	635.03	571.36	523.83	538.19	544.91
hydroxysteroid (11-beta) dehydrogenase 1	Hsd11b 1	100762502	0.00	0.00	0.00	0.00	0.00	0.00	0.76	0.92	0.00	1.16	0.00	0.93
hydroxysteroid (11-beta) dehydrogenase 2	Hsd11b 2	100766725	0.00	0.04	0.00	0.00	0.00	0.04	0.00	1.83	1.24	0.00	0.00	0.93
hydroxysteroid (17-beta) dehydrogenase 1	Hsd17b 1	100750866	0.68	0.49	0.71	0.55	0.16	0.35	36.46	28.37	24.84	24.34	12.42	18.69
hydroxysteroid (17-beta) dehydrogenase 10	Hsd17b 10	100757689	255.14	249.26	226.01	209.47	188.34	194.05	6223.66	6085.89	5483.81	4954.38	4479.42	4642.50
hydroxysteroid (17-beta) dehydrogenase 11	Hsd17b 11	100767058	8.43	7.95	7.73	7.99	10.33	9.31	327.36	315.69	299.34	307.11	395.36	362.65
hydroxysteroid (17-beta) dehydrogenase 12	Hsd17b 12	100751291	37.69	39.00	42.07	45.50	53.02	50.54	2125.19	2177.78	2346.30	2489.36	2899.00	2777.84
hydroxysteroid (17-beta) dehydrogenase 14	Hsd17b 14	100752644	0.00	0.00	0.00	0.00	0.00	0.00	0.00	0.00	0.00	0.00	0.00	0.00

hydroxysteroid (17-beta) dehydrogenase 2	Hsd17b 2	100751897	0.00	0.00	0.00	0.00	0.00	0.00	0.00	0.00	0.00	0.00	0.00	0.00
hydroxysteroid (17-beta) dehydrogenase 3	Hsd17b 3	100767644	0.00	0.00	0.00	0.00	0.00	0.00	0.00	0.00	0.00	0.00	0.00	0.00
hydroxysteroid (17-beta) dehydrogenase 4	Hsd17b 4	103161104	13.53	14.37	16.25	17.24	19.50	18.98	1040.57	1130.98	1271.90	1329.28	1490.38	1445.93
hydroxysteroid (17-beta) dehydrogenase 6	Hsd17b 6	100762147	0.03	0.00	0.00	0.00	0.00	0.08	1.52	0.00	0.00	1.16	0.00	1.87
hydroxysteroid (17-beta) dehydrogenase 7	Hsd17b 7	100767580	3.44	3.28	4.00	4.74	5.52	5.64	347.87	333.07	409.89	478.63	554.75	572.95
hydroxysteroid (17-beta) dehydrogenase 8	Hsd17b 8	100766230	28.44	26.45	36.80	31.06	26.51	28.39	1087.66	1066.01	1335.24	1176.30	994.62	1035.61
hydroxy-delta-5-steroid dehydrogenase, 3 beta- and steroid delta-isomerase 7	Hsd3b7	100754260	10.50	11.60	9.89	8.31	7.63	7.84	548.39	608.50	530.37	438.07	393.29	412.19
hydroxysteroid dehydrogenase like 1	Hsdl1	100774919	10.32	9.82	11.86	11.40	12.58	14.03	934.23	947.06	1131.54	1083.59	1249.23	1397.33
hydroxysteroid dehydrogenase like 2	Hsdl2	100769693	28.90	29.71	21.59	21.93	23.25	23.40	1487.94	1568.37	1150.17	1186.73	1282.35	1268.35
heat shock transcription factor 1	Hsf1	100762942	55.21	50.97	52.26	42.96	31.73	34.36	3496.92	3263.92	3333.76	2709.55	1984.07	2144.13
heat shock transcription factor 2	Hsf2	100752741	18.93	17.75	9.26	10.29	10.36	10.41	1424.90	1385.36	700.54	760.25	762.78	790.73
heat shock transcription factor 2 binding protein	Hsf2bp	100751781	5.07	4.62	4.93	4.66	4.65	3.96	322.80	324.84	317.97	267.71	312.57	271.99
heat shock transcription factor 4	Hsf4	100758969	0.48	0.41	0.31	0.35	0.20	0.37	28.86	37.52	23.60	20.86	16.56	21.50
heat shock transcription factor family member 5	Hsf5	100758083	0.05	0.04	0.00	0.05	0.04	0.00	3.04	1.83	0.00	2.32	3.10	0.93
hematopoietic SH2 domain containing	Hsh2d	100760566	0.25	0.23	0.25	0.06	0.16	0.25	17.47	21.05	22.36	8.11	13.45	17.76
heat shock protein 90kDa alpha (cytosolic), class A member 1	Hsp90a a1	100689397	206.33	203.67	194.69	227.58	204.86	186.83	15751.32	15669.95	15139.80	17497.37	15595.17	14308.86
heat shock protein 90kDa alpha (cytosolic), class B member 1	Hsp90a b1	100754509	1173.80	1190.86	1214.87	1228.64	1177.49	1279.05	80250.64	82170.93	84219.70	84532.78	80558.16	86815.86
heat shock protein 90kDa beta (Grp94), member 1	Hsp90b 1	100773331	618.74	624.20	701.08	774.19	793.00	774.65	51690.44	53224.76	59852.44	64976.00	66724.53	63950.10
heat shock 70kDa protein 12A	Hspa12 a	100758140	0.64	0.65	0.63	0.84	0.62	0.60	76.71	79.61	95.64	122.85	93.15	84.12
heat shock 70kD protein 12B	Hspa12 b	100766967	0.13	0.10	0.28	0.18	0.08	0.19	17.47	13.73	26.08	18.54	8.28	15.89
heat shock protein 70kDa family, member 13	Hspa13	100766580	19.29	20.28	21.26	23.50	26.91	26.42	2025.69	2114.64	2255.63	2497.47	2822.41	2790.92
heat shock 70kDa protein 14	Hspa14	100751911	19.68	20.44	17.09	19.21	19.99	17.67	1012.47	1032.16	929.08	1005.94	1009.11	918.78
heat shock 70kDa protein 1A	Hspa1a	100760510	0.03	0.05	0.02	0.00	0.01	0.01	0.00	0.00	0.00	0.00	0.00	0.00
heat shock 70kDa protein 1-like	Hspa1l	100760804	0.45	0.38	0.32	0.33	0.54	0.56	41.77	32.03	29.81	30.13	49.68	52.34
heat shock 70kDa protein 2	Hspa2	100764750	7.21	8.09	7.13	5.50	4.96	5.42	516.49	581.05	516.71	395.19	356.04	391.63
heat shock 70kDa protein 4	Hspa4	100764628	55.13	57.71	59.12	63.67	58.89	58.20	7003.71	7303.80	7545.68	7982.64	7385.66	7264.25
heat shock 70kDa protein 4-like	Hspa4l	100774014	0.03	0.02	0.00	0.00	0.01	0.00	3.04	2.75	0.00	0.00	2.07	0.00
heat shock 70kDa protein 5 (glucose-regulated protein, 78kDa)	Hspa5	100689305	687.89	721.10	896.48	881.00	813.29	820.99	47211.44	49679.92	62007.46	60346.12	55741.26	56070.83
heat shock 70kDa protein 8	Hspa8	100689472	304.08	302.98	362.78	373.63	325.37	338.25	13846.40	13909.42	16713.52	17033.80	14778.57	15448.22
heat shock 70kDa protein 9 (mortalin)	Hspa9	100689405	314.14	315.85	351.78	362.89	353.12	358.77	23867.76	24069.96	26745.86	27227.66	26402.50	27024.10
heat shock 27kDa protein 1	Hspb1	100763481	75.54	77.01	72.21	45.84	32.32	29.04	678.27	667.06	618.56	397.51	280.48	252.36
heat shock protein family B (small), member 11	Hspb11	100764905	31.83	30.05	29.84	30.93	31.10	32.31	450.41	447.45	385.05	391.71	399.50	402.84

heat shock 27kDa protein 2	Hspb2	100772331	0.00	0.00	0.00	0.00	0.00	0.00	0.00	0.92	0.00	0.00	0.00	0.93
heat shock 27kDa protein 3	Hspb3	100774495	0.00	0.07	0.00	0.00	0.00	0.00	0.00	0.92	0.00	0.00	0.00	0.00
heat shock protein, alpha-crystallin-related, B6	Hspb6	100774774	5.19	4.03	3.76	2.37	1.51	1.76	75.95	75.03	78.25	81.12	65.20	53.28
heat shock 27kDa protein family, member 7 (cardiovascular)	Hspb7	100751681	0.00	0.00	0.00	0.00	0.00	0.00	0.00	0.92	0.00	0.00	0.00	0.00
heat shock 22kDa protein 8	Hspb8	100754642	94.81	96.91	111.69	108.47	96.73	104.15	5009.16	5099.48	5932.21	5690.30	5053.84	5493.05
heat shock protein, alpha-crystallin-related, B9	Hspb9	100774771	1.24	1.22	0.89	0.79	0.71	0.81	196.72	224.18	134.15	143.71	139.72	158.89
HSPB (heat shock 27kDa) associated protein 1	Hspbap1	100767418	6.12	5.65	6.39	6.01	7.02	7.11	297.74	258.95	301.83	285.09	315.67	334.61
heat shock 60kDa protein 1 (chaperonin)	Hspd1	100689473	654.15	676.52	800.24	822.03	836.16	855.20	41064.49	42639.65	50252.35	50701.59	51440.89	52560.22
heparan sulfate proteoglycan 2	Hspg2	100689041	17.06	17.36	16.47	11.57	8.30	8.53	7395.63	7612.16	7252.55	5129.38	3728.02	3812.52
heat shock 105kDa/110kDa protein 1	Hsph1	100689462	60.10	61.22	71.76	72.77	75.92	74.18	5588.69	5738.18	6699.82	6740.28	7002.72	6821.22
HIV-1 Tat interactive protein 2, 30kDa	Htatip2	100759325	41.08	41.05	46.89	44.83	45.90	44.03	1130.19	1178.56	1324.06	1277.13	1255.44	1248.72
HIV-1 Tat specific factor 1	Htatsf1	100767363	51.97	51.20	47.13	51.77	53.75	51.63	3642.75	3683.01	3374.75	3688.84	3870.85	3631.19
5-hydroxytryptamine (serotonin) receptor 1A, G protein-coupled	Htr1a	100753851	0.00	0.00	0.00	0.00	0.00	0.00	0.00	0.00	0.00	0.00	0.00	0.00
5-hydroxytryptamine (serotonin) receptor 1B, G protein-coupled	Htr1b	100689321	2.45	2.34	2.08	2.36	3.55	2.82	334.96	320.26	291.89	324.50	483.34	386.02
5-hydroxytryptamine (serotonin) receptor 1D, G protein-coupled	Htr1d	100767570	0.00	0.00	0.00	0.00	0.00	0.00	0.00	0.00	0.00	1.16	0.00	0.00
5-hydroxytryptamine (serotonin) receptor 1F, G protein-coupled	Htr1f	100766950	0.03	0.00	0.00	0.00	0.00	0.00	0.76	0.00	0.00	0.00	0.00	0.00
5-hydroxytryptamine (serotonin) receptor 2A, G protein-coupled	Htr2a	100689471	0.00	0.00	0.00	0.00	0.00	0.00	0.00	0.00	0.00	0.00	0.00	0.00
5-hydroxytryptamine (serotonin) receptor 2B, G protein-coupled	Htr2b	103161688	0.00	0.02	0.00	0.00	0.00	0.00	2.28	0.92	1.24	0.00	2.07	0.93
5-hydroxytryptamine (serotonin) receptor 2C, G protein-coupled	Htr2c	100754331	0.00	0.00	0.00	0.00	0.00	0.00	0.00	0.00	0.00	0.00	0.00	0.00
5-hydroxytryptamine (serotonin) receptor 3A, ionotropic	Htr3a	100763929	0.00	0.00	0.00	0.00	0.00	0.00	0.00	0.00	0.00	0.00	0.00	0.00
5-hydroxytryptamine (serotonin) receptor 3B, ionotropic	Htr3b	100764440	0.00	0.03	0.00	0.00	0.00	0.00	0.00	1.83	0.00	0.00	0.00	0.00
5-hydroxytryptamine (serotonin) receptor 4, G protein-coupled	Htr4	100763863	0.00	0.00	0.00	0.00	0.00	0.00	0.00	0.00	0.00	0.00	0.00	0.00
5-hydroxytryptamine (serotonin) receptor 5A, G protein-coupled	Htr5a	100772532	0.00	0.00	0.00	0.00	0.00	0.00	0.00	0.00	0.00	0.00	0.00	0.00
5-hydroxytryptamine (serotonin) receptor 6, G protein-coupled	Htr6	100752934	0.11	0.09	0.17	0.07	0.08	0.13	9.87	8.24	14.91	5.79	7.24	12.15
5-hydroxytryptamine (serotonin) receptor 7, adenylate cyclase-coupled	Htr7	100764536	0.79	0.57	0.69	0.74	1.03	1.08	60.00	42.09	50.93	56.79	76.59	83.19
HtrA serine peptidase 1	Htra1	100762962	98.15	93.35	100.92	97.61	112.43	110.49	4935.49	4764.58	5065.23	4874.42	5597.21	5515.49
HtrA serine peptidase 2	Htra2	100760629	27.80	26.30	37.41	32.69	31.88	34.08	1492.49	1421.96	2048.20	1747.65	1787.42	1855.32
HtrA serine peptidase 3	Htra3	100774250	0.07	0.00	0.00	0.00	0.00	0.00	6.08	0.00	0.00	2.32	0.00	0.00
HtrA serine peptidase 4	Htra4	100754379	0.04	0.00	0.00	0.00	0.00	0.03	1.52	0.00	0.00	0.00	4.14	1.87
huntingtin	Htt	100764729	9.43	8.99	8.69	9.94	10.59	10.36	3777.95	3640.92	3518.83	3947.28	4222.74	4127.50
hormonally up-regulated Neu-associated kinase	Hunk	100765960	3.70	3.69	4.66	4.36	4.18	3.80	311.41	301.05	383.80	366.22	345.69	326.20
HUS1 checkpoint homolog (S. pombe)	Hus1	100754140	16.10	16.71	15.80	16.66	17.25	17.14	1622.38	1725.75	1648.25	1722.15	1750.16	1783.35

HUS1 checkpoint homolog b (S. pombe)	Hus1b	100756111	0.03	0.00	0.00	0.00	0.06	0.06	1.52	1.83	0.00	1.16	1.03	1.87
HECT, UBA and WWE domain containing 1, E3 ubiquitin protein ligase	Huwe1	100757403	46.15	45.16	41.82	46.43	43.44	43.58	19427.49	19046.42	17761.84	19444.36	18166.08	18292.42
hydrogen voltage-gated channel 1	Hvcn1	100774842	2.40	1.92	3.07	3.31	3.16	2.69	172.42	176.60	216.12	225.99	193.54	205.63
hyaluronoglucosaminidase 1	Hyal1	100752718	0.88	0.75	1.24	1.26	1.21	1.17	77.47	61.31	105.58	113.57	101.43	100.94
hyaluronoglucosaminidase 2	Hyal2	100752414	22.25	23.00	23.53	17.41	11.14	12.95	1239.57	1271.90	1320.34	967.70	625.13	718.76
hyaluronoglucosaminidase 3	Hyal3	100753318	1.66	2.26	2.49	2.35	2.53	2.54	108.61	143.66	135.39	139.07	142.83	151.42
hyaluronoglucosaminidase 4	Hyal4	100758272	0.00	0.00	0.00	0.00	0.00	0.00	0.00	0.00	0.00	0.00	0.00	0.00
HYDIN, axonemal central pair apparatus protein	Hydin	100758841	0.01	0.01	0.00	0.02	0.01	0.01	2.28	2.75	2.48	6.95	6.21	5.61
hydroxypyruvate isomerase (putative)	Hyi	100774763	73.42	71.35	84.36	71.78	61.64	67.71	1143.11	1122.75	1310.40	1089.38	968.75	1032.81
hydroxylysine kinase	Hykk	100756410	4.47	4.89	3.52	3.81	4.96	4.76	293.18	323.92	224.82	253.80	319.81	318.72
hydrolethalus syndrome 1	Hyls1	100759998	17.11	17.68	18.32	17.57	18.32	19.42	647.13	676.21	694.33	661.74	681.02	731.85
hypoxia up-regulated 1	Hyou1	100689308	205.97	208.32	253.12	257.61	254.24	271.37	23416.59	23704.86	28881.00	29065.71	28711.56	30724.46
huntingtin interacting protein K	Hypk	100753787	185.88	172.38	170.41	145.54	129.56	123.91	2069.74	1920.66	1845.74	1571.50	1459.33	1352.47
isoamyl acetate-hydrolyzing esterase 1 homolog (S. cerevisiae)	Iah1	100758521	47.88	45.98	50.02	50.08	49.95	57.52	1165.89	1137.39	1209.79	1238.89	1198.51	1371.16
islet amyloid polypeptide	Iapp	100771196	0.00	0.00	0.00	0.00	0.00	0.00	0.00	0.00	0.00	0.00	0.00	1.87
isoleucyl-tRNA synthetase	Iars	100754316	143.63	137.64	137.64	147.51	158.21	157.64	19448.76	18863.42	18826.31	19780.44	21225.50	20902.95
isoleucyl-tRNA synthetase 2, mitochondrial	Iars2	100758227	86.93	84.20	91.66	97.04	100.80	104.17	8144.54	8069.68	8800.19	9186.76	9531.19	9756.08
IBA57, iron-sulfur cluster assembly homolog (S. cerevisiae)	Iba57	100763588	0.72	0.87	0.72	0.68	0.28	0.55	18.99	21.05	24.84	22.02	11.38	14.95
integrin-binding sialoprotein	Ibsp	100752803	0.00	0.04	0.00	0.00	0.00	0.00	0.76	0.92	0.00	0.00	0.00	0.00
islet cell autoantigen 1, 69kDa	Ica1	100759520	0.00	0.00	0.00	0.00	0.00	0.00	0.00	0.00	0.00	0.00	0.00	0.00
islet cell autoantigen 1,69kDa-like	Ica1l	100764818	0.32	0.25	0.14	0.35	0.33	0.31	25.82	19.22	14.91	28.97	32.08	27.11
intercellular adhesion molecule 1	Icam1	100689428	96.09	94.61	108.55	99.02	84.40	85.41	7455.64	7388.90	8489.66	7717.25	6541.11	6667.00
intercellular adhesion molecule 2	Icam2	100750973	0.03	0.15	0.05	0.05	0.04	0.11	1.52	4.58	3.73	1.16	1.03	3.74
intercellular adhesion molecule 4 (Landsteiner-Wiener blood group)	Icam4	100761834	4.25	3.67	3.87	2.91	1.85	2.46	178.49	153.73	149.05	111.26	78.66	114.03
intercellular adhesion molecule 5, telencephalin	Icam5	100772237	1.26	0.95	1.21	0.89	0.66	0.95	157.98	126.27	161.47	120.53	89.01	123.38
intestinal cell (MAK-like) kinase	Ick	100752955	10.89	11.02	15.44	16.90	17.73	17.04	2073.54	2118.30	2981.01	3200.94	3339.90	3236.76
isoprenylcysteine carboxyl methyltransferase	Icmt	100765763	23.71	22.40	26.14	23.49	21.90	24.16	1491.74	1394.51	1635.83	1504.28	1389.99	1520.71
inducible T-cell co-stimulator	Icos	100770410	0.00	0.00	0.00	0.00	0.00	0.00	0.00	0.00	0.00	0.00	0.00	0.00
inducible T-cell co-stimulator ligand	Icoslg	100768618	0.00	0.00	0.00	0.00	0.00	0.00	0.00	0.00	0.00	0.00	0.00	0.93
immature colon carcinoma transcript 1	Ict1	100757939	17.99	19.01	21.31	20.57	19.34	19.93	543.07	593.86	633.46	622.34	578.56	589.78
inhibitor of DNA binding 1, dominant negative helix-loop-helix protein	Id1	100774183	3.25	3.46	2.43	2.36	2.24	2.57	72.92	77.78	54.65	53.31	49.68	56.08
inhibitor of DNA binding 2, dominant negative helix-loop-helix protein	Id2	100769110	5.12	5.63	2.19	2.93	4.62	4.84	149.63	162.88	69.56	89.24	137.65	138.33
inhibitor of DNA binding 3, dominant negative helix-loop-helix protein	Id3	100751577	34.28	36.57	28.83	23.10	22.33	23.90	823.34	869.28	700.54	548.17	545.44	562.67
insulin-degrading enzyme	Ide	100763752	19.13	18.55	19.20	20.50	21.51	20.94	2997.90	2929.02	3059.26	3267.00	3364.74	3276.02
isocitrate dehydrogenase 1 (NADP+), soluble	Idh1	100758958	16.11	16.85	14.46	15.29	19.18	16.96	960.82	997.39	849.59	903.96	1126.07	993.55

isocitrate dehydrogenase 2 (NADP+), mitochondrial	Idh2	103158535	9.55	8.85	11.94	11.55	9.42	10.22	397.24	366.01	510.50	474.00	386.05	411.25
isocitrate dehydrogenase 3 (NAD+) alpha	Idh3a	100754480	117.49	121.25	115.90	122.98	111.01	116.83	4844.34	4982.36	4866.50	5020.44	4717.47	4802.33
isocitrate dehydrogenase 3 (NAD+) beta	Idh3b	100771661	62.30	63.98	60.19	61.33	62.00	60.99	2784.47	2839.35	2685.39	2834.72	2779.97	2634.83
isocitrate dehydrogenase 3 (NAD+) gamma	Idh3g	100751753	67.43	66.05	71.08	73.52	67.35	71.46	2321.15	2320.53	2471.75	2483.57	2323.54	2465.66
isopentenyl-diphosphate delta isomerase 1	Idi1	100752366	10.33	10.19	12.25	13.22	13.77	12.78	758.78	767.71	921.63	966.54	1020.50	937.47
isopentenyl-diphosphate delta isomerase 2	Idi2	100752668	0.00	0.00	0.08	0.00	0.00	0.00	0.00	0.00	1.24	0.00	0.00	0.00
idnK, gluconokinase homolog (E. coli)	Idnk	100763818	29.82	31.55	32.68	36.43	25.26	26.74	819.54	848.24	886.85	1011.74	708.97	749.61
indoleamine 2,3-dioxygenase 1	Ido1	100754402	0.00	0.00	0.00	0.00	0.00	0.00	0.00	0.00	0.00	0.00	0.00	0.00
indoleamine 2,3-dioxygenase 2	Ido2	100754111	0.00	0.00	0.00	0.00	0.00	0.00	0.00	0.00	0.00	0.00	0.00	0.00
iduronate 2-sulfatase	Ids	100763345	0.30	0.33	0.15	0.26	0.26	0.31	25.06	25.62	13.66	26.66	20.70	24.30
iduronidase, alpha-L-	Idua	100767663	20.92	20.30	20.16	17.23	13.09	15.94	1586.68	1578.43	1557.58	1326.96	1040.16	1244.05
immediate early response 2	Ier2	103161436	3.68	3.10	3.58	2.76	1.97	2.39	525.60	442.88	500.56	360.42	249.43	303.77
immediate early response 3	Ier3	100763219	26.95	24.46	16.18	11.00	10.36	11.96	820.30	746.67	488.14	331.45	317.74	363.59
immediate early response 3 interacting protein 1	Ier3ip1	100772056	21.09	22.80	22.06	22.69	25.38	24.46	818.02	861.96	844.62	858.76	963.57	922.52
immediate early response 5	Ier5	103163864	16.20	16.44	14.83	13.20	12.84	12.76	1032.21	1044.97	951.44	841.38	811.43	818.77
immediate early response 5-like	Ier5l	100770969	0.41	0.37	0.34	0.05	0.00	0.13	12.15	9.15	7.45	1.16	0.00	2.80
intermediate filament family orphan 1	Iffo1	100753382	5.07	5.09	5.68	4.44	3.58	3.80	429.14	445.62	464.54	363.90	289.80	312.18
intermediate filament family orphan 2	Iffo2	100764698	4.86	5.08	4.04	3.22	2.86	3.50	154.19	167.45	120.48	106.62	85.90	102.81
interferon, gamma-inducible protein 30	Ifi30	100759310	33.74	37.59	34.67	35.04	29.58	28.67	1200.83	1322.22	1244.57	1198.32	1012.22	972.99
interferon-induced protein 35	Ifi35	100770156	106.26	103.41	85.37	74.53	66.51	72.88	8259.99	7941.58	6496.11	5380.87	4596.37	5126.66
interferon-induced protein 44	Ifi44	100760250	0.57	0.47	0.00	0.00	0.00	0.00	27.34	24.71	0.00	0.00	0.00	0.00
interferon-induced protein 44-like	Ifi44l	100760542	0.04	0.00	0.00	0.00	0.00	0.00	3.04	0.00	0.00	0.00	0.00	0.00
interferon induced with helicase C domain 1	Ifih1	100767593	1.71	1.73	2.12	2.32	2.42	2.46	238.50	240.65	293.13	316.39	334.30	336.48
interferon-induced protein with tetratricopeptide repeats 2	Ifit2	100763118	0.02	0.05	0.01	0.01	0.01	0.04	0.00	1.83	0.00	0.00	0.00	1.87
interferon-induced protein with tetratricopeptide repeats 3	Ifit3	100763410	0.00	0.02	0.00	0.00	0.01	0.03	0.00	0.92	0.00	0.00	0.00	0.93
interferon induced transmembrane protein 10	Ifitm10	100770183	0.00	0.00	0.00	0.00	0.00	0.00	0.00	0.00	0.00	0.00	0.00	0.00
interferon induced transmembrane protein 5	Ifitm5	100756384	0.50	0.18	0.16	0.47	0.42	0.38	8.35	5.49	3.73	10.43	6.21	5.61
intermediate filament tail domain containing 1	Ifltd1	100764631	0.00	0.00	0.00	0.00	0.00	0.00	0.00	0.00	0.00	0.00	0.00	0.00
interferon (alpha, beta and omega) receptor 1	Ifnar1	100768581	20.72	21.04	25.67	25.42	24.45	25.89	2312.80	2365.36	2881.64	2831.24	2723.05	2894.67
interferon, epsilon	Ifne	100761194	0.00	0.00	0.00	0.00	0.00	0.00	0.00	0.00	0.00	0.00	0.00	0.00
interferon, gamma	Ifng	100768486	0.07	0.03	0.00	0.04	0.00	0.00	2.28	0.92	0.00	1.16	0.00	0.00
interferon gamma receptor 1	Ifngr1	100772793	16.02	16.00	15.96	16.63	17.32	16.44	811.95	811.63	811.08	850.65	898.37	840.27
interferon gamma receptor 2 (interferon gamma transducer 1)	Ifngr2	100768877	11.72	11.01	10.79	11.17	11.12	10.08	603.07	570.98	570.12	579.46	579.59	534.63
interferon, kappa	Ifnk	100766116	0.00	0.00	0.00	0.00	0.00	0.00	0.00	0.00	0.00	0.00	0.00	0.00

interferon, lambda receptor 1	Ifnlr1	100756839	0.04	0.00	0.00	0.03	0.02	0.00	3.80	4.58	6.21	2.32	3.10	2.80
interferon-related developmental regulator 1	Ifrd1	100772061	176.27	182.40	183.26	200.32	217.55	225.78	8450.63	8737.66	8775.34	9391.89	10328.13	10644.95
interferon-related developmental regulator 2	Ifrd2	100753618	82.80	78.54	87.13	63.65	45.82	52.08	5258.29	5048.24	5625.41	4087.51	3003.53	3358.27
intraflagellar transport 122 homolog (Chlamydomonas)	Ift122	100771127	2.41	2.45	2.97	2.71	2.96	2.99	308.37	316.60	370.14	341.88	369.49	371.06
intraflagellar transport 140 homolog (Chlamydomonas)	Ift140	100774958	8.08	8.25	9.03	9.57	9.78	10.53	1402.87	1453.99	1615.95	1674.64	1748.09	1821.67
intraflagellar transport 172 homolog (Chlamydomonas)	Ift172	100757945	13.42	13.27	12.29	12.74	13.88	14.52	2250.51	2260.13	2065.59	2119.66	2323.54	2432.95
intraflagellar transport 20 homolog (Chlamydomonas)	Ift20	100759277	24.51	24.48	26.45	25.62	26.53	25.69	745.87	739.35	798.66	755.62	804.18	768.30
intraflagellar transport 22 homolog (Chlamydomonas)	Ift22	100768415	4.19	3.09	5.15	4.82	3.65	4.48	65.32	57.65	70.80	71.85	71.41	77.58
intraflagellar transport 27 homolog (Chlamydomonas)	Ift27	100772135	38.44	36.93	47.86	46.21	43.93	43.82	1175.77	1160.26	1521.56	1438.22	1401.37	1372.10
intraflagellar transport 43 homolog (Chlamydomonas)	Ift43	100757117	18.33	18.26	17.31	17.56	18.03	19.92	405.59	392.55	375.11	369.70	380.87	422.47
intraflagellar transport 46 homolog (Chlamydomonas)	Ift46	100758681	56.98	54.19	61.65	58.46	60.02	63.25	2776.12	2674.64	3056.78	2888.03	2979.73	3057.30
intraflagellar transport 52 homolog (Chlamydomonas)	Ift52	100772375	89.35	88.38	81.89	87.10	96.40	98.01	5618.31	5675.04	5244.09	5466.63	6133.33	6133.30
	Ift57	100758071	24.87	26.15	25.28	29.46	31.14	26.73	612.95	638.69	634.71	699.99	768.99	653.33
intraflagellar transport 74 homolog (Chlamydomonas)	Ift74	100765142	22.49	20.00	20.65	24.56	26.75	24.32	1454.52	1335.03	1332.76	1581.93	1681.85	1532.86
intraflagellar transport 80 homolog (Chlamydomonas)	Ift80	100767321	13.26	13.50	12.83	15.64	16.34	16.05	1158.30	1220.65	1147.69	1396.50	1448.98	1414.16
intraflagellar transport 81 homolog (Chlamydomonas)	Ift81	100765323	10.81	11.23	11.54	13.84	15.34	13.80	854.48	893.07	921.63	1068.52	1163.32	1044.03
intraflagellar transport 88 homolog (Chlamydomonas)	Ift88	100761553	4.41	4.63	4.11	5.29	5.77	5.33	421.54	448.37	404.92	504.13	545.44	520.61
immunoglobulin (CD79A) binding protein 1	Igbp1	100768558	40.73	44.14	49.70	49.24	51.98	51.48	1477.30	1580.26	1809.72	1744.17	1851.59	1837.56
immunoglobulin superfamily, DCC subclass, member 3	Igdcc3	100753053	0.00	0.00	0.00	0.00	0.00	0.00	0.00	0.00	0.00	1.16	0.00	0.00
immunoglobulin superfamily, DCC subclass, member 4	Igdcc4	100752753	2.62	3.08	3.08	2.68	2.28	2.66	491.42	581.96	608.62	513.40	431.59	502.85
insulin-like growth factor 1 (somatomedin C)	Igf1	100751642	0.00	0.00	0.00	0.00	0.00	0.00	0.00	0.00	0.00	0.00	0.00	0.00
insulin-like growth factor 1 receptor	Igf1r	100768531	2.74	2.58	3.79	3.63	4.00	4.53	387.37	359.61	524.16	502.97	562.00	638.38
insulin-like growth factor 2 (somatomedin A)	Igf2	100762359	0.00	0.00	0.00	0.00	0.00	0.00	0.00	0.00	0.00	0.00	0.00	0.00
insulin-like growth factor 2 mRNA binding protein 1	Igf2bp1	100754118	0.00	0.00	0.00	0.00	0.00	0.03	0.00	0.00	0.00	0.00	0.00	0.93
insulin-like growth factor 2 mRNA binding protein 2	Igf2bp2	100755568	25.36	26.40	25.97	29.15	30.99	29.46	2801.94	2893.34	2808.36	3218.32	3368.88	3198.44
insulin-like growth factor 2 mRNA binding protein 3	Igf2bp3	100764384	3.56	3.40	2.85	3.54	2.91	3.18	330.40	325.75	269.53	325.66	273.24	277.60
insulin-like growth factor 2 receptor	Igf2r	100762916	39.90	38.63	23.23	24.57	23.84	24.76	10299.35	9971.12	6015.43	6313.80	6120.91	6373.51
insulin-like growth factor binding protein, acid labile subunit	Igfals	100753202	0.24	0.24	0.24	0.31	0.22	0.26	22.03	21.96	23.60	27.81	18.63	20.56
insulin-like growth factor binding protein 1	Igfbp1	100763682	0.00	0.00	0.00	0.00	0.00	0.00	0.00	0.00	0.00	0.00	0.00	0.00

insulin-like growth factor binding protein 2, 36kDa	Igfbp2	100757494	0.00	0.00	0.00	0.00	0.00	0.00	0.00	0.00	0.00	0.00	0.00	0.00
insulin-like growth factor binding protein 3	Igfbp3	100764858	0.00	0.00	0.00	0.00	0.00	0.00	0.00	0.00	0.00	0.00	0.00	0.00
insulin-like growth factor binding protein 4	Igfbp4	100772587	999.60	986.83	255.02	289.66	425.53	425.98	57465.98	56848.29	14762.20	16591.10	24303.55	24499.56
insulin-like growth factor binding protein 5	Igfbp5	100754876	0.00	0.00	0.00	0.00	0.00	0.00	0.00	0.00	0.00	0.00	0.00	0.00
insulin-like growth factor binding protein 6	Igfbp6	100751810	0.17	0.12	0.45	0.16	0.05	0.13	5.32	4.58	13.66	4.64	5.17	5.61
insulin-like growth factor binding protein 7	Igfbp7	100757355	0.38	0.46	0.18	0.26	0.08	0.00	6.08	7.32	2.48	2.32	1.03	0.93
insulin-like growth factor binding protein-like 1	Igfbpl1	100769562	0.00	0.00	0.00	0.00	0.00	0.00	0.00	0.00	0.00	0.00	0.00	0.00
IGF-like family receptor 1	Igflr1	100775057	0.00	0.00	0.00	0.00	0.00	0.00	0.00	0.00	0.00	2.32	0.00	0.00
immunoglobulin-like and fibronectin type III domain containing 1	Igfn1	100765216	0.01	0.00	0.00	0.00	0.00	0.00	4.56	0.92	0.00	0.00	0.00	0.00
immunoglobulin mu binding protein 2	Ighmbp 2	100754637	13.47	12.71	13.54	10.70	8.15	8.88	1481.10	1371.64	1520.31	1163.56	909.75	991.68
IgA-inducing protein	Igip	100757565	1.80	1.47	1.27	2.37	2.36	2.13	113.17	92.42	85.70	146.02	141.79	130.85
immunoglobulin J polypeptide, linker protein for immunoglobulin alpha and mu polypeptides	Igj	100774033	5.18	4.87	3.55	4.61	5.42	4.54	132.16	158.30	108.06	112.42	146.97	123.38
immunoglobulin lambda-like polypeptide 5	Igll5	100754596	0.00	0.00	0.00	0.00	0.00	0.00	0.00	0.00	0.00	0.00	0.00	0.00
IgLON family member 5	Iglon5	100754556	0.00	0.00	0.00	0.00	0.00	0.00	0.00	0.00	0.00	0.00	0.00	0.00
immunoglobulin superfamily, member 1	Igsf1	100768646	0.00	0.01	0.00	0.00	0.00	0.00	0.00	0.92	0.00	0.00	0.00	0.00
immunoglobulin superfamily, member 10	Igsf10	100770822	0.00	0.00	0.00	0.00	0.00	0.00	0.00	0.00	0.00	0.00	0.00	0.00
immunoglobulin superfamily, member 11	Igsf11	100751379	0.00	0.00	0.00	0.00	0.00	0.00	0.00	0.00	0.00	0.00	0.00	0.00
immunoglobin superfamily, member 21	Igsf21	100770461	0.00	0.00	0.00	0.00	0.00	0.00	0.00	0.00	0.00	0.00	0.00	0.00
immunoglobulin superfamily, member 22	Igsf22	103164228	0.00	0.00	0.00	0.00	0.00	0.00	0.00	0.00	0.00	0.00	0.00	0.00
immunoglobulin superfamily, member 3	Igsf3	100769189	0.00	0.00	0.00	0.00	0.00	0.00	0.00	0.00	0.00	0.00	0.00	0.00
immunoglobulin superfamily, member 5	Igsf5	100765526	0.00	0.00	0.00	0.00	0.00	0.00	0.00	0.00	0.00	0.00	0.00	0.00
immunoglobulin superfamily, member 6	Igsf6	103159815	0.05	0.06	0.00	0.00	0.00	0.00	4.56	7.32	11.18	8.11	13.45	3.74
immunoglobulin superfamily, member 8	Igsf8	100763950	14.77	14.67	7.44	5.77	3.69	3.84	959.30	947.97	517.95	396.35	254.61	269.18
immunoglobulin superfamily, member 9	Igsf9	100755364	0.13	0.19	0.04	0.01	0.03	0.02	18.23	25.62	4.97	1.16	4.14	1.87
immunoglobulin superfamily, member 9B	Igsf9b	100752572	0.01	0.01	0.00	0.00	0.00	0.00	0.76	1.83	0.00	0.00	0.00	0.00
indian hedgehog	Ihh	100774896	0.00	0.00	0.00	0.00	0.00	0.00	0.00	0.00	0.00	0.00	0.00	0.00
IK cytokine, down-regulator of HLA II	Ik	100754728	29.17	29.45	31.60	34.27	36.16	35.89	1761.37	1778.83	1910.33	2035.06	2164.16	2123.57
IKBKB interacting protein	Ikbip	100757532	17.14	17.14	17.79	18.93	22.23	21.38	1336.03	1354.25	1414.74	1519.34	1758.44	1739.42
inhibitor of kappa light polypeptide gene enhancer in B-cells, kinase complex-associated protein	Ikbkap	100767755	58.49	56.60	57.98	60.04	57.02	60.51	8746.09	8734.91	8923.15	9055.80	8515.87	8906.47

inhibitor of kappa light polypeptide gene enhancer in B-cells, kinase beta	Ikbkb	100750488	14.68	15.25	15.86	15.99	14.76	16.18	1618.58	1627.84	1758.79	1761.56	1608.37	1770.26
inhibitor of kappa light polypeptide gene enhancer in B-cells, kinase epsilon	Ikbke	100761646	2.01	2.37	2.76	2.47	2.04	2.40	213.43	238.82	270.77	243.37	207.00	257.97
inhibitor of kappa light polypeptide gene enhancer in B-cells, kinase gamma	Ikbkg	100759762	10.54	10.91	10.82	11.12	11.64	12.34	2082.66	2158.56	2157.50	2203.11	2264.55	2432.95
IKAROS family zinc finger 1 (Ikaros)	Ikzf1	100759224	0.00	0.00	0.00	0.00	0.00	0.07	1.52	0.00	0.00	2.32	1.03	2.80
IKAROS family zinc finger 2 (Helios)	Ikzf2	100760184	0.58	0.53	0.25	0.20	0.27	0.29	53.17	53.07	24.84	22.02	32.08	27.11
IKAROS family zinc finger 3 (Aiolos)	Ikzf3	100768701	0.08	0.09	0.04	0.00	0.09	0.06	5.32	6.41	2.48	1.16	5.17	3.74
IKAROS family zinc finger 4 (Eos)	Ikzf4	100768259	0.29	0.19	0.39	0.43	0.32	0.27	38.74	26.54	52.17	59.10	45.54	35.52
IKAROS family zinc finger 5 (Pegasus)	Ikzf5	100766537	17.08	16.57	14.32	16.14	15.67	15.20	1342.87	1321.31	1125.33	1278.29	1235.77	1206.66
interleukin 10	Il10	100759320	0.00	0.00	0.00	0.00	0.00	0.00	0.00	0.00	0.00	0.00	0.00	0.00
interleukin 10 receptor, alpha	Il10ra	100762009	0.00	0.00	0.00	0.00	0.00	0.00	0.00	0.00	0.00	0.00	0.00	0.00
interleukin 10 receptor, beta	Il10rb	100768006	13.95	14.75	13.78	13.90	16.52	14.58	1181.08	1230.72	1119.12	1138.06	1326.85	1206.66
interleukin 11	Il11	100773527	3.63	2.86	0.50	0.79	0.42	0.44	85.83	64.05	19.87	20.86	13.45	14.02
interleukin 11 receptor, alpha	Il11ra	100752228	10.02	9.60	11.21	10.34	7.64	8.72	485.35	473.99	541.55	512.24	362.25	414.06
interleukin 12A	Il12a	100760602	0.00	0.00	0.00	0.00	0.00	0.00	0.00	0.00	0.00	0.00	0.00	0.00
interleukin 12B	Il12b	100764758	0.00	0.00	0.00	0.00	0.00	0.00	0.00	0.00	0.00	0.00	0.00	0.00
interleukin 12 receptor, beta 1	Il12rb1	100763378	0.02	0.04	0.00	0.01	0.00	0.00	6.08	5.49	1.24	3.48	0.00	0.00
interleukin 12 receptor, beta 2	Il12rb2	100758602	0.02	0.08	0.06	0.07	0.15	0.09	3.04	7.32	4.97	10.43	17.59	15.89
interleukin 13	Il13	100762597	0.00	0.00	0.00	0.18	0.00	0.00	0.00	1.83	0.00	1.16	1.03	0.93
interleukin 13 receptor, alpha 1	Il13ra1	100771375	0.02	0.00	0.00	0.00	0.00	0.00	1.52	0.00	0.00	0.00	1.03	0.00
interleukin 13 receptor, alpha 2	Il13ra2	100754617	19.15	16.83	6.62	8.22	9.41	9.29	828.66	720.13	296.86	348.83	397.43	405.65
interleukin 15	Il15	100772160	7.09	8.44	16.65	17.49	20.08	19.36	235.46	259.87	516.71	516.88	563.03	520.61
interleukin 15 receptor, alpha	Il15ra	100755321	3.49	4.71	3.26	3.38	2.87	2.52	161.02	199.48	139.11	141.39	132.48	125.25
interleukin 16	Il16	100767901	0.00	0.00	0.00	0.00	0.00	0.00	0.00	0.00	0.00	0.00	0.00	0.00
interleukin 17A	Il17a	100750422	0.00	0.00	0.00	0.00	0.00	0.00	0.00	0.00	0.00	0.00	0.00	0.00
interleukin 17B	Il17b	100757725	0.00	0.00	0.00	0.00	0.00	0.00	0.00	0.00	0.00	0.00	0.00	0.00
interleukin 17C	Il17c	100767127	0.66	0.49	0.32	0.38	0.31	0.36	36.46	30.20	18.63	23.18	17.59	24.30
interleukin 17D	Il17d	100763580	5.49	6.26	5.82	5.93	6.65	4.91	208.87	235.16	229.79	225.99	247.36	189.74
interleukin 17F	Il17f	100751004	0.68	0.61	0.33	0.31	0.37	0.33	22.03	17.39	8.69	8.11	12.42	10.28
interleukin 17 receptor A	Il17ra	100754214	7.07	7.10	7.13	6.86	5.29	5.68	871.95	870.20	869.46	846.01	638.59	704.74
interleukin 17 receptor B	Il17rb	100753686	0.00	0.00	0.03	0.03	0.00	0.02	0.00	0.00	2.48	1.16	1.03	0.93
interleukin 17 receptor C	Il17rc	100751334	1.94	1.72	3.48	3.14	2.42	3.15	197.48	180.26	334.12	304.80	267.03	311.25
interleukin 17 receptor D	Il17rd	100751618	0.00	0.00	0.00	0.00	0.00	0.00	0.00	0.00	0.00	0.00	0.00	0.00
interleukin 17 receptor E	Il17re	100751042	0.23	0.30	0.21	0.18	0.25	0.31	41.02	39.35	34.78	41.72	30.01	42.06
interleukin 18	Il18	100750469	25.10	22.89	22.10	26.15	29.49	29.96	903.09	868.37	809.84	932.93	1053.62	1070.20
interleukin 18 receptor 1	Il18r1	100758160	0.00	0.00	0.00	0.00	0.00	0.00	0.00	0.00	0.00	0.00	0.00	0.00
interleukin 18 receptor accessory protein	Il18rap	100757868	0.00	0.04	0.00	0.00	0.00	0.00	0.00	1.83	0.00	0.00	0.00	0.00
interleukin 19	Il19	100774163	0.08	0.04	0.26	0.20	0.39	0.13	2.28	0.92	6.21	8.11	10.35	4.67
interleukin 1, alpha	Il1a	100769260	0.00	0.00	0.00	0.00	0.00	0.00	0.00	0.00	0.00	0.00	0.00	0.00
interleukin 1, beta	Il1b	100775079	0.00	0.00	0.00	0.00	0.00	0.00	0.00	0.00	0.00	0.00	1.03	0.93
interleukin 1 family, member 10 (theta)	Il1f10	100752197	0.00	0.00	0.00	0.00	0.00	0.00	0.00	0.00	0.00	0.00	0.00	0.00

interleukin 1 receptor, type I	Il1r1	100758740	10.42	10.16	11.88	11.08	12.56	12.22	1456.04	1432.03	1671.85	1543.68	1735.67	1689.88
interleukin 1 receptor, type II	Il1r2	100759030	0.00	0.00	0.00	0.00	0.00	0.00	0.76	0.00	0.00	0.00	0.00	0.00
interleukin 1 receptor accessory protein	Il1rap	100771447	15.10	15.59	15.73	16.95	19.37	18.80	1734.79	1792.55	1809.72	1987.55	2247.99	2191.80
interleukin 1 receptor accessory protein-like 1	Il1rapl1	100767870	0.00	0.00	0.00	0.00	0.00	0.00	0.00	0.00	0.00	0.00	0.00	0.00
interleukin 1 receptor accessory protein-like 2	Il1rapl2	100771606	0.00	0.00	0.03	0.14	0.10	0.05	1.52	0.00	1.24	5.79	6.21	1.87
interleukin 1 receptor-like 1	Il1rl1	100760768	1.55	1.80	0.40	0.59	0.75	0.70	180.01	206.80	43.47	67.22	87.97	83.19
interleukin 1 receptor-like 2	Il1rl2	100758449	0.11	0.14	0.03	0.03	0.19	0.03	8.35	14.64	7.45	2.32	14.49	6.54
interleukin 1 receptor antagonist	Il1rn	100751912	0.00	0.00	0.00	0.00	0.00	0.00	0.00	0.00	0.00	0.00	0.00	0.00
interleukin 2	Il2	100755773	0.00	0.00	0.00	0.00	0.00	0.00	0.00	0.00	0.00	0.00	0.00	0.00
interleukin 20	Il20	100770693	0.04	0.00	0.00	0.00	0.00	0.00	0.76	0.00	0.00	0.00	1.03	0.00
interleukin 20 receptor, alpha	Il20ra	100751800	0.00	0.00	0.00	0.00	0.00	0.00	0.00	0.00	0.00	0.00	0.00	0.00
interleukin 20 receptor beta	Il20rb	100772546	0.00	0.04	0.00	0.00	0.00	0.00	0.76	2.75	0.00	0.00	1.03	1.87
interleukin 21	Il21	100755479	0.00	0.00	0.00	0.00	0.00	0.00	0.00	0.00	0.00	0.00	0.00	0.00
interleukin 21 receptor	Il21r	100763133	0.34	0.23	0.15	0.10	0.08	0.03	29.62	26.54	11.18	9.27	5.17	4.67
interleukin 22	Il22	100768781	0.00	0.00	0.00	0.00	0.00	0.00	0.00	0.00	0.00	0.00	0.00	0.00
interleukin 22 receptor, alpha 1	Il22ra1	100756546	3.22	3.48	3.14	3.36	2.84	2.95	412.43	450.20	402.44	418.37	366.39	400.97
interleukin 22 receptor, alpha 2	Il22ra2	100751510	0.00	0.00	0.00	0.00	0.00	0.00	0.00	0.00	0.00	0.00	0.00	0.00
interleukin 23, alpha subunit p19	Il23a	100765061	0.14	0.06	0.07	0.04	0.06	0.03	7.60	7.32	3.73	3.48	4.14	1.87
interleukin 23 receptor	Il23r	100758310	0.00	0.00	0.00	0.00	0.00	0.00	0.00	0.00	0.00	0.00	0.00	0.00
interleukin 24	Il24	100773869	0.00	0.00	0.00	0.00	0.00	0.00	0.00	0.00	0.00	0.00	0.00	0.00
interleukin 25	Il25	100766813	1.94	1.77	1.03	1.20	1.01	0.90	85.07	80.52	49.68	64.90	48.64	54.21
interleukin 27	Il27	100753480	0.00	0.00	0.00	0.00	0.00	0.00	0.00	0.00	0.00	0.00	0.00	0.93
interleukin 27 receptor, alpha	Il27ra	100760191	0.06	0.07	0.02	0.02	0.04	0.02	3.80	6.41	2.48	1.16	3.10	2.80
interleukin 2 receptor, alpha	Il2ra	100764797	0.00	0.00	0.00	0.00	0.00	0.00	0.00	0.00	0.00	0.00	0.00	0.00
interleukin 2 receptor, beta	Il2rb	100769948	0.09	0.03	0.04	0.00	0.00	0.03	3.04	2.75	1.24	0.00	0.00	0.93
interleukin 2 receptor, gamma	Il2rg	100756284	0.20	0.18	0.21	0.28	0.04	0.19	6.08	10.98	6.21	9.27	1.03	5.61
interleukin 31	Il31	100754195	0.00	0.00	0.00	0.00	0.00	0.00	0.76	0.00	1.24	0.00	0.00	1.87
interleukin 31 receptor A	Il31ra	100770824	0.07	0.06	0.14	0.10	0.15	0.15	9.87	10.98	21.12	13.91	20.70	22.43
interleukin 33	Il33	100761971	0.20	0.13	0.03	0.09	0.19	0.16	15.95	10.07	2.48	6.95	14.49	12.15
interleukin 34	Il34	100753324	0.08	0.07	0.09	0.00	0.08	0.00	3.80	1.83	2.48	0.00	2.07	0.00
interleukin 36, alpha	Il36a	100757088	0.08	0.00	0.55	0.13	0.34	0.63	0.76	2.75	11.18	3.48	8.28	9.35
interleukin 36, beta	Il36b	100773970	0.00	0.00	0.00	0.00	0.00	0.00	0.00	0.00	1.24	0.00	0.00	0.00
interleukin 36, gamma	Il36g	100757380	1.10	0.99	1.64	1.65	1.80	1.75	90.39	84.18	139.11	141.39	149.04	147.68
interleukin 36 receptor antagonist	Il36rn	100752490	0.00	0.00	0.00	0.00	0.06	0.00	0.00	0.00	0.00	0.00	0.00	0.00
interleukin 4	Il4	100755642	0.00	0.00	0.00	0.00	0.00	0.00	0.00	0.00	0.00	0.00	0.00	0.00
interleukin 4 induced 1	Il4i1	100773063	0.67	0.59	0.57	0.36	0.17	0.32	60.00	61.31	63.35	47.52	34.15	46.73
interleukin 4 receptor	Il4r	100762840	11.61	11.16	11.45	10.08	7.37	8.06	1090.70	1044.97	1098.00	972.33	706.90	771.10
interleukin 5	Il5	100755060	0.00	0.00	0.00	0.00	0.00	0.00	0.00	0.00	0.00	0.00	0.00	0.00
interleukin 5 receptor, alpha	Il5ra	100762117	0.01	0.05	0.00	0.00	0.06	0.07	1.52	3.66	0.00	2.32	3.10	5.61
interleukin 6	Il6	100689186	5.81	5.08	3.27	3.76	4.48	3.85	163.30	149.15	98.12	111.26	128.34	107.49
interleukin 6 receptor	Il6r	100750971	0.55	0.67	0.57	0.65	0.61	0.49	41.02	49.41	38.50	49.83	40.36	37.39
interleukin 6 signal transducer	Il6st	100767181	55.67	55.79	57.16	65.25	69.01	65.98	8390.63	8398.18	8684.67	9752.31	10271.20	9928.06
interleukin 7	Il7	100756443	0.29	0.17	0.30	0.20	0.35	0.33	21.27	11.90	21.12	12.75	24.84	21.50
interleukin 7 receptor	Il7r	100757700	0.00	0.00	0.03	0.00	0.00	0.00	0.00	0.00	1.24	0.00	0.00	0.00

interleukin 9	Il9	100755564	0.00	0.00	0.00	0.00	0.00	0.00	0.00	0.00	0.00	0.00	0.00	0.00
interleukin 9 receptor	Il9r	100759467	0.22	0.23	0.09	0.08	0.13	0.07	16.71	15.56	6.21	8.11	12.42	4.67
immunoglobulin-like domain containing receptor 1	Ildr1	100766502	0.00	0.00	0.00	0.00	0.00	0.00	0.00	0.00	0.00	0.00	0.00	0.00
immunoglobulin-like domain containing receptor 2	Ildr2	100772155	9.77	9.82	10.16	10.75	9.03	8.47	442.81	440.13	447.15	461.25	390.19	386.95
interleukin enhancer binding factor 2	Ilf2	100768916	41.18	43.23	47.34	52.32	52.21	51.62	1935.31	2016.73	2176.14	2410.55	2391.85	2375.93
interleukin enhancer binding factor 3, 90kDa	Ilf3	100757497	79.19	79.69	85.66	77.28	62.21	65.01	7791.35	8021.18	8740.57	7827.34	6441.75	6532.41
integrin-linked kinase	Ilk	100689388	68.01	69.52	91.40	98.74	99.43	103.65	3327.54	3457.00	4439.22	4680.88	4651.23	4829.44
integrin-linked kinase-associated serine/threonine phosphatase	Ilkap	100773641	50.44	52.10	50.80	50.08	47.08	47.49	1766.69	1834.64	1791.09	1714.04	1641.49	1678.67
ilvB (bacterial acetolactate synthase)-like	Ilvbl	100752439	10.46	10.73	12.02	9.64	8.47	9.87	739.03	753.99	883.12	696.51	598.22	686.05
IMP1 inner mitochondrial membrane peptidase-like (S. cerevisiae)	Immp1l	100766368	25.54	26.91	20.32	23.55	25.90	23.83	603.07	634.12	455.85	528.47	597.19	586.04
inner membrane protein, mitochondrial	Immt	100762629	70.54	72.22	74.32	79.67	82.78	81.81	6240.37	6410.72	6632.74	6994.08	7239.73	7210.98
IMP3, U3 small nucleolar ribonucleoprotein	Imp3	100760961	60.09	58.07	63.93	46.97	38.86	44.09	1479.58	1428.37	1609.74	1182.10	965.64	1101.04
IMP4, U3 small nucleolar ribonucleoprotein	Imp4	100773345	67.56	65.27	65.96	55.36	41.69	44.91	2847.51	2833.86	2867.98	2382.74	1815.37	1905.79
inositol(myo)-1(or 4)-monophosphatase 1	Impa1	100760520	23.28	21.38	18.91	19.75	23.51	21.97	1279.06	1296.60	1161.35	1185.58	1407.58	1341.25
inositol(myo)-1(or 4)-monophosphatase 2	Impa2	100763112	3.14	3.99	2.73	2.30	2.00	2.46	149.63	187.58	135.39	105.46	102.46	112.16
impact RWD domain protein	Impact	100774193	0.00	0.00	0.00	0.00	0.00	0.00	0.00	0.00	0.00	0.00	0.00	0.00
inositol monophosphatase domain containing 1	Impad1	100756245	26.09	26.07	23.49	26.27	29.28	28.56	2409.26	2408.37	2196.01	2403.60	2686.82	2669.42
IMP (inosine 5'-monophosphate) dehydrogenase 1	Impdh1	100760550	37.07	36.06	31.61	28.60	21.17	22.74	2479.90	2429.41	2183.59	1919.17	1446.91	1527.25
IMP (inosine 5'-monophosphate) dehydrogenase 2	Impdh2	100689398	178.30	179.54	209.76	222.48	219.11	214.67	8319.99	8486.94	9771.50	10109.26	10053.86	9787.86
interphotoreceptor matrix proteoglycan 1	Impg1	100766332	0.00	0.00	0.00	0.00	0.00	0.00	0.00	0.00	0.00	0.00	0.00	0.00
interphotoreceptor matrix proteoglycan 2	Impg2	100756357	0.01	0.01	0.01	0.00	0.00	0.00	0.76	1.83	1.24	0.00	0.00	0.00
internexin neuronal intermediate filament protein, alpha	Ina	100763646	0.00	0.00	0.00	0.00	0.00	0.00	0.00	0.00	0.00	0.00	0.00	0.00
InaD-like (Drosophila)	Inadl	100755532	0.79	0.87	0.44	0.65	0.73	0.74	150.39	167.45	88.19	114.73	141.79	141.14
inhibitor of CDK, cyclin A1 interacting protein 1	Inca1	100772801	0.15	0.04	0.00	0.05	0.04	0.11	6.84	2.75	2.48	4.64	6.21	7.48
inner centromere protein antigens 135/155kDa	Incenp	100770889	21.12	21.04	21.08	20.54	19.76	19.33	3245.51	3243.79	3214.52	3075.77	2960.06	2898.41
inhibitor of growth family, member 1	Ing1	100767785	38.33	37.24	50.31	48.70	47.49	49.44	1626.17	1584.84	2126.45	2020.00	1943.70	2006.74
inhibitor of growth family, member 2	Ing2	100755912	20.73	20.57	23.28	25.50	26.73	22.68	625.86	620.39	711.72	783.43	808.32	711.28
inhibitor of growth family, member 3	Ing3	100766428	26.20	26.43	30.37	32.70	36.13	35.57	1354.26	1425.62	1629.62	1698.98	1889.88	1857.19
inhibitor of growth family, member 4	Ing4	100754938	21.21	25.45	21.67	21.34	26.16	26.30	837.01	935.16	955.16	907.43	951.15	989.82
inhibitor of growth family, member 5	Ing5	100755543	12.19	12.97	14.28	13.41	13.54	13.08	394.20	405.36	429.76	445.03	457.46	412.19
inhibin, alpha	Inha	100766733	2.08	2.21	2.07	1.87	1.14	1.43	85.07	87.84	79.49	74.17	44.50	54.21
inhibin, beta A	Inhba	100762446	0.00	0.02	0.00	0.03	0.00	0.00	0.76	0.92	0.00	1.16	0.00	0.00
inhibin, beta B	Inhbb	100759312	10.07	10.36	7.43	7.21	6.95	7.79	755.74	777.78	557.70	537.74	507.14	582.30

inhibin, beta C	Inhbc	100755878	0.11	0.03	0.04	0.08	0.04	0.10	3.80	2.75	1.24	2.32	0.00	2.80
inhibin, beta E	Inhbe	100756167	0.03	0.06	0.03	0.00	0.00	0.06	1.52	6.41	2.48	2.32	0.00	2.80
INTS3 and NABP interacting protein	Inip	100770269	19.71	20.58	22.17	25.58	23.93	22.43	2014.30	2097.26	2265.57	2605.25	2419.80	2282.46
indolethylamine N-methyltransferase	Inmt	100773805	0.21	0.15	0.04	0.06	0.06	0.05	26.58	25.62	3.73	9.27	13.45	5.61
INO80 complex subunit	Ino80	100762469	27.42	26.92	23.15	24.82	23.35	23.75	4987.13	5028.11	4329.91	4592.80	4306.58	4351.82
INO80 complex subunit B	Ino80b	100764793	20.76	19.02	25.23	21.04	17.59	18.28	591.68	560.00	731.59	579.46	482.30	509.39
INO80 complex subunit C	Ino80c	100766878	9.31	10.12	7.96	9.20	9.59	9.83	679.03	737.52	583.78	676.81	695.51	718.76
INO80 complex subunit D	Ino80d	100754682	2.52	2.48	2.36	2.86	2.89	3.08	723.08	705.49	696.81	829.79	836.27	884.20
INO80 complex subunit E	Ino80e	100773645	27.99	28.74	35.97	31.47	23.63	26.36	1065.63	1106.28	1355.12	1153.13	907.68	979.53
inositol polyphosphate-1-phosphatase	Inpp1	100755639	14.64	12.27	12.04	13.36	12.79	12.91	521.80	454.77	452.12	480.95	464.71	475.75
inositol polyphosphate-4-phosphatase, type I, 107kDa	Inpp4a	100767245	7.35	7.39	7.47	7.58	7.61	8.41	1253.24	1256.34	1294.25	1310.74	1297.87	1427.24
inositol polyphosphate-4-phosphatase, type II, 105kDa	Inpp4b	100771589	0.00	0.01	0.00	0.00	0.00	0.00	0.76	0.92	0.00	0.00	0.00	0.00
inositol polyphosphate-5-phosphatase, 40kDa	Inpp5a	100760037	11.60	10.97	13.27	13.14	12.77	13.24	543.83	514.25	608.62	596.84	537.16	561.74
inositol polyphosphate-5-phosphatase, 75kDa	Inpp5b	100751850	24.47	24.81	28.09	30.45	33.42	33.85	2509.52	2571.24	2845.62	3066.50	3425.80	3485.38
inositol polyphosphate-5-phosphatase, 145kDa	Inpp5d	100764735	0.26	0.28	0.21	0.24	0.28	0.27	36.46	38.43	29.81	32.45	38.29	38.32
inositol polyphosphate-5-phosphatase, 72 kDa	Inpp5e	100759837	11.16	11.20	12.92	12.59	11.43	11.84	1276.03	1281.96	1495.47	1466.03	1317.54	1375.83
inositol polyphosphate-5-phosphatase F	Inpp5f	100753756	7.17	6.86	7.29	8.13	8.41	8.12	965.37	926.01	984.97	1094.02	1146.76	1088.89
inositol polyphosphate-5-phosphatase J	Inpp5j	100761777	2.70	2.33	2.06	1.58	1.17	1.33	306.85	255.29	219.85	177.31	130.41	146.74
inositol polyphosphate-5-phosphatase K	Inpp5k	100764873	14.49	15.17	20.56	20.85	20.08	20.19	1209.95	1271.90	1733.95	1755.76	1710.83	1702.97
inositol polyphosphate phosphatase-like 1	Inppl1	100772102	30.65	29.26	37.15	33.92	29.65	32.24	4137.21	3979.48	5065.23	4685.51	4210.32	4503.24
insulin	Ins	100760716	0.00	0.00	0.00	0.00	0.00	0.00	0.00	0.00	0.00	0.00	0.00	0.00
insulin induced gene 1	Insig1	100689080	3.69	3.45	4.19	4.53	3.86	4.36	230.14	220.52	272.02	287.41	244.26	272.92
insulin induced gene 2	Insig2	100689079	12.92	13.50	12.19	13.90	17.61	16.49	862.08	904.97	832.20	958.43	1158.15	1115.06
insulin-like 3 (Leydig cell)	Insl3	100754203	0.00	0.00	0.09	0.00	0.00	0.00	0.00	0.00	1.24	0.00	0.00	0.93
insulin-like 5	Insl5	100752144	0.00	0.00	0.00	0.00	0.00	0.00	0.00	0.00	0.00	0.00	1.03	0.00
insulin-like 6	Insl6	100769626	0.00	0.00	0.00	0.00	0.00	0.00	0.00	0.00	0.00	0.00	0.00	0.00
insulin receptor	Insr	100766818	7.90	7.57	7.45	7.39	6.50	6.88	1183.36	1162.09	1155.14	1150.81	994.62	1065.52
insulin receptor-related receptor	Insrr	100750978	0.01	0.00	0.00	0.01	0.00	0.01	0.76	0.00	0.00	0.00	0.00	0.93
integrator complex subunit 1	Ints1	100764130	12.39	11.85	11.77	8.66	6.63	7.49	2924.99	2828.37	2776.06	2062.88	1588.70	1801.11
integrator complex subunit 10	Ints10	100755256	28.84	29.01	29.23	31.13	34.36	31.81	2049.24	2104.58	2114.03	2210.06	2453.95	2284.33
integrator complex subunit 12	Ints12	100755340	29.12	27.56	23.16	22.18	22.79	24.03	1241.09	1162.09	1017.27	952.63	962.54	1024.40
integrator complex subunit 2	Ints2	100774909	13.17	13.06	6.77	7.51	8.07	7.89	1630.73	1618.69	844.62	927.14	973.92	944.95
integrator complex subunit 3	Ints3	100768339	22.22	20.90	24.07	24.97	22.66	24.37	2844.47	2704.84	3092.80	3142.99	2880.37	2993.75
integrator complex subunit 4	Ints4	100767027	25.32	26.04	28.66	28.65	28.78	31.25	2407.74	2478.83	2753.71	2706.08	2750.99	2986.27
integrator complex subunit 5	Ints5	100751877	17.14	16.42	19.04	16.96	12.62	13.87	1564.65	1505.23	1758.79	1559.91	1155.04	1276.76
integrator complex subunit 6	Ints6	100761628	22.83	24.06	25.15	28.11	27.56	25.40	1835.05	1918.83	1992.31	2235.56	2177.61	2009.54
integrator complex subunit 7	Ints7	100750715	9.98	9.63	9.77	10.46	9.48	9.51	1142.35	1118.17	1116.64	1199.48	1088.81	1106.65
integrator complex subunit 8	Ints8	100757889	6.87	6.65	9.37	9.54	10.89	8.99	743.59	708.24	996.15	1001.31	1125.03	955.23

integrator complex subunit 9	Ints9	100752291	6.20	6.47	7.55	7.54	6.96	7.24	486.86	531.63	618.56	606.12	576.49	590.71
inturned planar cell polarity protein	Intu	100771987	9.73	9.54	11.85	13.29	11.45	11.57	1032.21	1017.52	1264.44	1408.09	1216.11	1220.68
inversin	Invs	100750579	7.59	7.57	8.24	8.97	10.03	11.06	879.55	873.86	920.39	1021.01	1106.40	1215.07
inositol hexakisphosphate kinase 1	Ip6k1	100760280	39.87	39.20	50.86	47.45	37.89	43.80	5226.39	5147.98	6694.85	6203.70	4939.99	5731.39
inositol hexakisphosphate kinase 2	Ip6k2	100759816	14.44	13.15	17.36	16.16	14.07	15.08	860.56	788.76	965.10	879.62	779.34	828.12
inositol hexakisphosphate kinase 3	Ip6k3	100772883	0.06	0.12	0.05	0.04	0.10	0.09	4.56	6.41	2.48	3.48	6.21	5.61
interaction protein for cytohesin exchange factors 1	Ipcef1	100774344	0.00	0.00	0.00	0.00	0.03	0.00	0.00	0.00	0.00	0.00	1.03	0.93
inositol polyphosphate multikinase	Ipmk	100772629	6.28	6.50	7.08	7.79	7.76	8.04	927.40	972.68	1059.50	1155.44	1147.80	1193.57
importin 11	Ipo11	100756698	28.34	28.16	27.62	32.08	35.15	33.47	3635.15	3646.41	3607.02	4104.89	4499.09	4313.50
importin 13	Ipo13	100754981	15.27	14.22	17.80	16.55	13.15	14.35	1765.93	1680.92	2074.29	1901.79	1523.50	1676.80
importin 4	Ipo4	100760098	62.41	58.90	61.78	55.22	42.86	48.45	6822.94	6471.12	6712.24	6014.79	4705.05	5304.25
importin 5	Ipo5	100764714	198.08	201.48	200.46	210.79	215.88	224.04	23283.67	24199.89	24202.06	25119.59	25616.95	26186.64
importin 7	Ipo7	100752357	89.42	89.56	77.31	82.75	83.02	78.10	13379.28	13452.82	11658.23	12319.32	12272.87	11691.78
importin 8	Ipo8	100762731	5.81	5.63	5.69	6.47	6.04	6.84	590.16	580.13	566.39	661.74	592.01	668.29
importin 9	Ipo9	100756041	45.19	45.35	42.23	42.50	42.15	44.53	5620.59	5711.64	5309.92	5329.87	5234.96	5481.84
intracisternal A particle-promoted polypeptide	Ipp	100774648	4.20	4.56	4.61	4.64	6.35	5.58	423.06	443.79	467.02	461.25	627.20	569.21
inositol 1,3,4,5,6-pentakisphosphate 2-kinase	Ippk	100755765	9.37	9.17	7.41	8.15	8.63	8.26	669.91	639.61	511.74	567.87	617.89	552.39
IQ motif containing with AAA domain 1	Iqca1	100768381	0.00	0.00	0.00	0.00	0.00	0.00	0.00	0.00	0.00	0.00	0.00	0.00
IQ motif containing B1	Iqcb1	100765421	9.93	9.63	14.69	14.40	16.16	17.00	664.60	646.01	967.59	973.49	1053.62	1108.52
IQ motif containing C	Iqcc	100760183	8.02	8.37	9.55	9.92	12.73	11.74	372.93	387.06	439.70	451.98	572.35	535.57
IQ motif containing D	Iqcd	100771431	0.75	1.07	1.36	1.15	1.21	1.05	50.89	56.73	80.74	59.10	62.10	57.95
IQ motif containing E	Iqce	100761808	0.82	0.99	0.77	0.74	0.70	0.96	157.22	188.50	150.29	141.39	140.76	183.20
IQ motif containing F1	Iqcf1	100766292	0.42	0.19	0.59	0.16	0.43	0.32	24.31	21.96	14.91	16.22	22.77	24.30
IQ motif containing F2	Iqcf2	100767170	0.00	0.00	0.00	0.00	0.00	0.00	0.00	0.00	0.00	0.00	0.00	0.00
IQ motif containing F3	Iqcf3	100767457	0.00	0.00	0.00	0.00	0.00	0.00	0.00	0.00	0.00	0.00	0.00	0.00
IQ motif containing F5	Iqcf5	100766585	0.00	0.00	0.00	0.00	0.00	0.00	0.00	0.00	0.00	0.00	0.00	0.00
IQ motif containing G	Iqcg	100764504	0.69	0.72	0.62	0.49	0.92	0.74	45.57	47.58	36.02	32.45	54.85	45.80
IQ motif containing H	Iqch	100759510	0.11	0.15	0.11	0.15	0.20	0.07	12.91	18.30	12.42	17.38	18.63	12.15
IQ motif containing J	Iqcj	103160084	0.00	0.00	0.00	0.00	0.00	0.00	0.00	0.00	0.00	0.00	0.00	0.00
IQ motif containing K	Iqck	100763057	2.56	2.39	3.61	3.77	3.51	3.86	149.63	120.78	191.28	210.92	198.72	213.10
IQ motif containing GTPase activating protein 1	Iqgap1	100757264	68.75	69.18	72.32	76.98	48.01	48.81	13142.31	13376.88	13941.18	14547.92	9088.21	9200.89
IQ motif containing GTPase activating protein 2	Iqgap2	100761545	8.51	8.59	8.40	10.50	10.27	9.21	1430.21	1461.31	1429.64	1778.94	1717.04	1525.38
IQ motif containing GTPase activating protein 3	Iqgap3	100754110	14.81	15.30	16.44	16.18	14.65	15.46	2642.44	2737.78	2951.20	2888.03	2660.95	2761.01
IQ motif and Sec7 domain 1	Iqsec1	100763049	8.93	8.07	8.49	6.55	5.00	5.61	854.48	794.25	840.89	657.11	496.79	572.02
IQ motif and Sec7 domain 2	Iqsec2	100762612	0.87	0.94	1.00	0.93	0.81	0.71	91.90	101.57	116.76	97.35	84.87	75.71
IQ motif and Sec7 domain 3	Iqsec3	100756354	0.00	0.00	0.00	0.00	0.00	0.00	0.00	0.00	0.00	0.00	0.00	0.00
IQ motif and ubiquitin domain containing	Iqub	100759138	0.00	0.01	0.00	0.00	0.00	0.00	0.00	0.92	0.00	0.00	0.00	0.00
interleukin-1 receptor-associated kinase 1	Irak1	100773909	31.94	32.03	27.00	26.88	26.95	27.98	3048.79	3050.72	2634.47	2546.15	2544.00	2681.57
interleukin-1 receptor-associated kinase 1 binding protein 1	Irak1bp1	100758003	27.91	30.28	30.35	32.31	34.25	29.86	823.34	883.92	875.67	907.43	982.20	851.48

interleukin-1 receptor-associated kinase 2	Irak2	100758629	9.39	9.54	10.21	10.63	12.32	11.43	803.59	819.87	873.19	893.53	1037.06	975.80
interleukin-1 receptor-associated kinase 3	Irak3	100767434	0.00	0.00	0.00	0.00	0.00	0.00	0.00	0.00	0.00	0.00	0.00	1.87
interleukin-1 receptor-associated kinase 4	Irak4	100751891	6.52	5.98	7.45	8.08	8.32	7.44	490.66	441.96	563.91	601.48	620.99	575.76
iron-responsive element binding protein 2	Ireb2	100756122	20.27	19.61	18.96	21.50	21.81	20.69	3219.69	3144.06	3051.81	3400.27	3477.55	3327.42
interferon regulatory factor 1	Irf1	100756092	23.53	21.65	13.88	16.09	18.90	22.94	439.01	403.53	279.47	300.16	371.56	439.29
interferon regulatory factor 2	Irf2	100753270	11.54	12.57	13.97	14.50	14.32	13.96	772.45	850.07	939.02	945.68	956.33	926.26
interferon regulatory factor 2 binding protein 2	Irf2bp2	100770683	9.97	9.63	10.05	10.52	9.94	10.36	1177.29	1127.32	1191.16	1234.25	1157.11	1216.01
interferon regulatory factor 2 binding protein-like	Irf2bpl	100762712	6.77	6.91	5.24	4.58	4.36	4.02	629.66	645.10	495.59	426.48	404.68	378.54
interferon regulatory factor 3	Irf3	100765676	9.73	9.74	12.54	10.65	9.79	10.37	561.30	543.53	727.86	593.37	517.49	546.78
interferon regulatory factor 4	Irf4	100755524	0.00	0.00	0.00	0.00	0.00	0.00	0.00	0.00	0.00	0.00	0.00	0.00
interferon regulatory factor 5	Irf5	100753482	0.00	0.00	0.00	0.00	0.00	0.00	0.00	0.00	0.00	0.00	0.00	2.80
interferon regulatory factor 6	Irf6	100761633	0.00	0.00	0.00	0.00	0.00	0.00	0.76	0.00	0.00	0.00	0.00	0.00
interferon regulatory factor 7	Irf7	100755889	1.04	1.29	1.39	1.63	1.06	1.30	53.93	61.31	65.83	69.54	55.89	59.82
interferon regulatory factor 8	Irf8	100770390	0.00	0.00	0.00	0.00	0.00	0.00	0.00	0.00	0.00	0.00	0.00	0.00
interferon regulatory factor 9	Irf9	100760392	7.90	8.77	7.86	8.53	7.80	8.01	485.35	517.91	470.75	527.31	469.88	491.64
immunoresponsive 1 homolog (mouse)	Irg1	100764165	0.00	0.00	0.00	0.00	0.00	0.00	0.76	0.00	0.00	0.00	0.00	0.00
insulin receptor substrate 1	Irs1	100755710	6.68	6.63	6.99	6.43	4.60	4.59	935.75	944.31	1007.33	949.16	689.30	700.07
insulin receptor substrate 2	Irs2	100771848	8.20	8.51	5.34	4.63	4.93	4.92	1146.14	1183.14	741.53	635.09	667.57	672.96
insulin receptor substrate 4	Irs4	100773159	0.00	0.00	0.00	0.00	0.00	0.00	0.00	0.00	0.00	0.00	0.00	0.00
iroquois homeobox 1	Irx1	100750610	0.00	0.03	0.00	0.00	0.03	0.00	0.00	0.00	0.00	0.00	1.03	0.00
iroquois homeobox 3	Irx3	100773584	9.46	9.43	15.42	11.24	8.78	7.92	413.19	401.70	660.79	487.91	401.57	356.11
iroquois homeobox 4	Irx4	100770031	0.00	0.00	0.00	0.00	0.00	0.00	0.00	0.00	0.00	0.00	0.00	0.00
iroquois homeobox 5	Irx5	100773873	2.27	2.27	2.56	1.77	2.63	1.65	105.58	113.46	120.48	84.60	125.23	78.51
iroquois homeobox 6	Irx6	100774452	0.11	0.08	0.13	0.04	0.03	0.04	0.00	0.00	0.00	0.00	0.00	0.00
iron-sulfur cluster assembly 2	Isca2	100771623	15.38	18.53	18.41	16.40	20.35	19.04	339.51	404.44	399.95	349.99	430.55	429.01
iron-sulfur cluster assembly enzyme	Iscu	100773802	38.22	37.99	41.23	41.00	33.55	34.33	1149.18	1172.16	1247.06	1215.71	1006.01	1014.12
ISG15 ubiquitin-like modifier	Isg15	100772582	1.26	1.40	1.67	0.83	0.80	1.15	40.26	44.84	45.96	28.97	35.19	30.84
interferon stimulated exonuclease gene 20kDa	Isg20	100765291	0.31	0.37	0.33	0.26	0.42	0.42	8.35	9.15	8.69	5.79	12.42	11.22
interferon stimulated exonuclease gene 20kDa-like 2	Isg20l2	100752909	40.47	42.66	44.18	47.33	50.69	46.38	3116.39	3275.82	3418.22	3635.53	3870.85	3572.31
ISL LIM homeobox 1	Isl1	100774367	0.00	0.00	0.00	0.00	0.00	0.02	0.00	0.00	0.00	0.00	0.00	0.93
ISL LIM homeobox 2	Isl2	100764443	0.00	0.00	0.00	0.00	0.00	0.00	0.00	0.00	0.00	0.00	0.00	0.00
immunoglobulin superfamily containing leucine-rich repeat	Islr	100752905	0.06	0.00	0.00	0.00	0.00	0.00	2.28	0.00	0.00	1.16	0.00	0.00
immunoglobulin superfamily containing leucine-rich repeat 2	Islr2	100757313	0.04	0.01	0.06	0.06	0.00	0.00	3.04	0.92	3.73	4.64	0.00	0.00
isthmin 1, angiogenesis inhibitor	Ism1	100770831	0.00	0.00	0.00	0.00	0.00	0.00	0.00	0.00	0.00	1.16	0.00	0.00
isthmin 2	Ism2	100763293	0.00	0.00	0.00	0.00	0.00	0.00	0.00	0.00	0.00	0.00	0.00	0.00
isochorismatase domain containing 1	Isoc1	100773735	6.80	7.06	6.04	6.41	7.00	6.60	639.53	672.55	572.60	606.12	652.04	621.56
isochorismatase domain containing 2	Isoc2	100774695	1.50	1.20	1.23	0.78	0.69	1.24	44.05	37.52	32.29	24.34	22.77	32.71
isoprenoid synthase domain containing	Ispd	100759830	0.67	0.46	1.06	0.87	0.75	0.77	53.93	42.09	79.49	69.54	61.06	61.69

increased sodium tolerance 1 homolog (yeast)	Ist1	100756334	38.01	37.28	36.80	36.45	38.04	39.32	1759.09	1749.54	1733.95	1625.96	1606.30	1642.21
intestine-specific homeobox	Isx	100756409	0.00	0.00	0.00	0.00	0.00	0.00	0.00	0.00	0.00	0.00	0.00	0.00
inositol-3-phosphate synthase 1	Isyna1	100767831	42.48	38.64	35.12	27.05	16.32	18.48	1734.79	1581.18	1411.01	1065.05	652.04	729.98
itchy E3 ubiquitin protein ligase	Itch	100751263	22.29	23.43	22.13	26.29	27.85	25.68	3468.82	3688.50	3497.72	4114.17	4298.30	4050.86
integrin alpha FG-GAP repeat containing 1	Itfg1	100755490	42.94	44.29	55.10	59.13	65.65	65.26	3698.20	3874.25	4788.24	5101.57	5633.43	5500.53
integrin alpha FG-GAP repeat containing 2	Itfg2	100768769	13.44	13.52	12.55	10.85	9.16	9.24	884.86	898.56	886.85	779.95	629.27	658.94
integrin alpha FG-GAP repeat containing 3	Itfg3	100771773	34.55	33.49	39.92	39.36	31.30	33.28	2947.01	2848.50	3443.06	3355.07	2682.68	2840.46
integrin, alpha 1	Itga1	100750393	1.42	1.55	1.54	1.96	2.12	2.02	325.08	355.95	363.93	457.77	489.55	455.18
integrin, alpha 10	Itga10	100754198	0.05	0.00	0.02	0.02	0.03	0.00	7.60	3.66	3.73	11.59	8.28	3.74
integrin, alpha 11	Itga11	100761554	0.00	0.00	0.00	0.00	0.00	0.00	0.00	0.00	0.00	0.00	0.00	0.00
integrin, alpha 2 (CD49B, alpha 2 subunit of VLA-2 receptor)	Itga2	100774579	0.01	0.03	0.00	0.00	0.00	0.01	4.56	5.49	4.97	3.48	6.21	9.35
integrin, alpha 2b (platelet glycoprotein IIb of IIb/IIIa complex, antigen CD41)	Itga2b	100762814	1.92	1.88	2.13	1.32	1.05	1.11	195.20	203.14	197.49	128.64	94.18	107.49
integrin, alpha 3 (antigen CD49C, alpha 3 subunit of VLA-3 receptor)	Itga3	100758471	28.16	27.64	22.61	19.61	17.54	17.99	3858.46	3812.03	3127.57	2711.87	2430.15	2515.20
integrin, alpha 4 (antigen CD49D, alpha 4 subunit of VLA-4 receptor)	Itga4	100762323	0.00	0.01	0.00	0.00	0.00	0.00	0.76	0.92	0.00	1.16	1.03	0.00
integrin, alpha 5 (fibronectin receptor, alpha polypeptide)	Itga5	100768174	51.33	50.64	62.50	60.34	45.43	50.96	6783.44	6763.01	8382.84	7958.30	6079.51	6726.82
integrin, alpha 6	Itga6	100769534	0.02	0.01	0.00	0.00	0.00	0.00	1.52	0.92	0.00	0.00	0.00	0.00
integrin, alpha 7	Itga7	100773038	2.07	1.99	0.97	0.92	0.98	1.00	269.64	256.21	137.87	122.85	137.65	128.98
integrin, alpha 8	Itga8	100769007	0.00	0.00	0.00	0.00	0.00	0.00	0.00	0.00	0.00	0.00	0.00	0.00
integrin, alpha 9	Itga9	100754465	0.32	0.36	0.03	0.02	0.05	0.06	48.61	53.99	3.73	2.32	7.24	11.22
integrin, alpha E (antigen CD103, human mucosal lymphocyte antigen 1; alpha polypeptide)	Itgae	100772578	0.09	0.11	0.11	0.06	0.05	0.07	17.47	18.30	18.63	20.86	13.45	14.95
integrin, alpha L (antigen CD11A (p180), lymphocyte function-associated antigen 1; alpha polypeptide)	Itgal	100770691	0.00	0.00	0.00	0.03	0.02	0.00	0.00	0.00	0.00	2.32	1.03	0.00
integrin, alpha M (complement component 3 receptor 3 subunit)	Itgam	100758889	0.01	0.03	0.01	0.01	0.00	0.00	0.76	2.75	1.24	1.16	0.00	0.00
integrin, alpha V	Itgav	100752313	23.13	22.65	24.51	26.36	29.07	28.28	4510.14	4476.34	4859.04	5150.24	5672.76	5464.08
integrin, alpha X (complement component 3 receptor 4 subunit)	Itgax	100757452	0.01	0.00	0.00	0.00	0.00	0.00	0.76	0.00	0.00	0.00	0.00	0.00
integrin, beta 1 (fibronectin receptor, beta polypeptide, antigen CD29 includes MDF2, MSK12)	Itgb1	100763033	387.02	390.59	375.22	395.12	459.17	460.43	37585.04	38460.69	36955.81	38615.21	45163.70	44890.32
integrin beta 1 binding protein 1	Itgb1bp 1	100759099	41.57	41.18	45.25	47.04	50.18	51.46	1175.01	1164.84	1252.02	1310.74	1404.48	1430.04
integrin beta 1 binding protein (melusin) 2	Itgb1bp 2	100753749	0.03	0.10	0.00	0.00	0.00	0.00	5.32	6.41	6.21	1.16	7.24	7.48
integrin, beta 2 (complement component 3 receptor 3 and 4 subunit)	Itgb2	100756135	0.02	0.03	0.02	0.02	0.02	0.03	0.76	0.00	0.00	0.00	0.00	0.93
integrin, beta 3 (platelet glycoprotein IIIa, antigen CD61)	Itgb3	100775048	0.00	0.00	0.00	0.00	0.00	0.00	0.00	0.00	0.00	0.00	0.00	0.93

integrin beta 3 binding protein (beta3-endonexin)	Itgb3bp	100757281	3.07	3.23	4.24	4.39	6.30	5.39	156.47	161.96	226.06	222.51	242.19	234.60
integrin, beta 4	Itgb4	100756849	3.67	3.66	5.40	4.41	3.35	3.53	819.54	826.28	1252.02	994.35	745.19	799.14
integrin, beta 5	Itgb5	100765378	123.76	121.92	139.58	137.99	129.78	138.69	10581.90	10473.47	12038.31	11794.33	11069.18	11867.50
integrin, beta 6	Itgb6	100768511	0.04	0.05	0.02	0.02	0.04	0.07	4.56	3.66	1.24	1.16	2.07	3.74
integrin, beta 7	Itgb7	100770075	0.10	0.01	0.00	0.00	0.05	0.01	4.56	0.92	1.24	0.00	1.03	1.87
integrin, beta 8	Itgb8	100769713	0.00	0.00	0.00	0.00	0.00	0.00	0.00	0.00	0.00	0.00	0.00	0.00
integrin, beta-like 1 (with EGF-like repeat domains)	Itgbl1	100761751	0.00	0.00	0.00	0.00	0.00	0.00	0.00	0.00	0.00	0.00	0.00	0.00
inter-alpha-trypsin inhibitor heavy chain 1	Itih1	100761604	0.00	0.00	0.00	0.00	0.00	0.00	0.00	0.00	0.00	0.00	0.00	0.00
inter-alpha-trypsin inhibitor heavy chain 2	Itih2	100761405	0.00	0.01	0.02	0.03	0.00	0.01	0.00	0.92	1.24	2.32	0.00	0.93
inter-alpha-trypsin inhibitor heavy chain 3	Itih3	100761315	0.00	0.00	0.00	0.00	0.00	0.00	0.00	0.00	0.00	0.00	0.00	0.00
inter-alpha-trypsin inhibitor heavy chain family, member 4	Itih4	100753872	0.01	0.01	0.00	0.00	0.00	0.00	0.76	0.92	0.00	2.32	0.00	0.00
inter-alpha-trypsin inhibitor heavy chain family, member 5	Itih5	100761693	13.22	13.03	16.89	17.86	16.71	16.86	2295.33	2253.73	2949.96	3094.32	2887.61	2946.08
inter-alpha-trypsin inhibitor heavy chain family, member 6	Itih6	100755168	0.00	0.00	0.00	0.00	0.00	0.00	0.76	0.00	0.00	0.00	0.00	0.00
IL2-inducible T-cell kinase	Itk	100770087	0.00	0.00	0.00	0.00	0.00	0.00	0.00	0.00	0.00	0.00	0.00	0.00
integral membrane protein 2A	Itm2a	100755879	0.00	0.00	0.00	0.00	0.00	0.00	0.00	0.00	0.00	0.00	0.00	0.00
integral membrane protein 2B	Itm2b	100751459	40.55	40.94	37.97	39.44	40.96	40.45	1831.25	1826.41	1706.63	1747.65	1820.54	1808.59
integral membrane protein 2C	Itm2c	100750451	263.89	253.16	284.43	260.00	226.49	253.51	11261.69	10874.26	12148.85	11109.41	9219.66	10423.44
inosine triphosphatase (nucleoside triphosphate pyrophosphatase)	Itpa	100768122	82.11	77.61	86.88	80.01	75.83	80.16	2588.51	2462.36	2715.20	2503.27	2338.03	2506.78
inositol-tetrakisphosphate 1-kinase	Itpk1	100768438	7.89	6.96	8.95	7.59	5.86	6.42	639.53	558.17	721.65	603.80	468.85	522.48
inositol-trisphosphate 3-kinase A	Itpka	100772914	4.19	3.69	6.64	4.92	4.31	3.45	164.82	137.26	258.35	194.70	165.60	135.53
inositol-trisphosphate 3-kinase B	Itpkb	100774983	2.43	2.33	3.25	3.48	2.37	2.85	272.67	258.95	360.21	398.67	279.45	322.46
inositol-trisphosphate 3-kinase C	Itpkc	100770355	1.91	1.96	1.66	1.64	0.78	1.10	123.05	121.70	110.55	106.62	55.89	66.36
inositol 1,4,5-trisphosphate receptor, type 1	Itpr1	100764450	8.20	8.12	9.46	10.43	9.96	9.56	2361.41	2342.49	2727.62	2967.99	2840.00	2734.84
inositol 1,4,5-trisphosphate receptor, type 2	Itpr2	100758965	9.35	9.49	11.55	11.72	11.10	10.59	3228.04	3274.91	3997.04	4000.59	3811.85	3648.02
inositol 1,4,5-trisphosphate receptor, type 3	Itpr3	100751775	6.94	6.76	7.45	6.30	5.05	5.39	1939.10	1912.42	2143.84	1806.76	1474.86	1506.69
inositol 1,4,5-trisphosphate receptor interacting protein	Itprip	100762684	8.15	8.01	8.15	8.26	7.49	8.01	871.95	853.73	880.64	883.10	795.90	861.77
inositol 1,4,5-trisphosphate receptor interacting protein-like 1	Itpripl1	100759110	9.25	9.46	9.84	9.44	8.29	8.41	638.77	655.16	686.87	664.06	585.80	598.19
inositol 1,4,5-trisphosphate receptor interacting protein-like 2	Itpripl2	100756036	15.20	15.00	15.65	16.70	15.96	16.58	2633.32	2602.36	2737.56	2903.09	2746.85	2899.35
intersectin 1 (SH3 domain protein)	Itsn1	100758619	13.16	13.45	13.57	14.60	13.91	14.81	1553.26	1531.77	1665.64	1734.90	1682.89	1795.50
intersectin 2	Itsn2	100754648	19.42	18.41	15.90	19.45	20.67	18.96	3712.63	3588.76	3096.52	3740.99	3955.72	3621.85
isovaleryl-CoA dehydrogenase	Ivd	100759847	37.20	35.10	35.82	35.18	32.96	37.03	1688.46	1597.65	1642.04	1546.00	1411.72	1564.64
involucrin	Ivl	100761237	0.00	0.00	0.00	0.00	0.00	0.00	0.00	0.00	0.00	0.00	0.00	0.00
influenza virus NS1A binding protein	Ivns1abp	100753413	105.55	105.45	111.46	128.96	145.32	143.32	9516.27	9592.30	10223.62	11695.82	13162.96	12874.14
IWS1 homolog (S. cerevisiae)	Iws1	100771981	8.87	9.29	9.64	11.33	9.93	9.46	729.92	758.56	791.21	925.98	810.39	775.78
iodotyrosine deiodinase	Iyd	100766759	0.18	0.16	0.00	0.00	0.00	0.00	6.84	7.32	0.00	0.00	0.00	0.00

izumo sperm-egg fusion 1	Izumo1	100752050	0.92	1.47	0.38	0.25	0.50	0.29	51.65	52.16	11.18	13.91	13.45	9.35
IZUMO family member 2	Izumo2	100770186	0.00	0.00	0.00	0.00	0.00	0.00	0.00	0.00	0.00	0.00	0.00	0.00
IZUMO family member 3	Izumo3	100761208	0.00	0.00	0.00	0.00	0.00	0.00	0.00	0.00	0.00	0.00	0.00	0.00
IZUMO family member 4	Izumo4	100759426	2.04	2.51	2.49	2.46	2.71	2.26	75.19	96.08	116.76	104.30	110.74	97.21
jade family PHD finger 1	Jade1	100772857	16.87	17.63	14.61	16.98	15.21	15.27	2782.95	2915.30	2445.67	2803.43	2495.35	2523.61
jade family PHD finger 2	Jade2	100758538	5.69	4.85	5.37	3.90	3.03	2.97	445.09	380.65	421.07	317.54	255.64	246.75
jade family PHD finger 3	Jade3	100774923	5.41	5.67	4.13	5.38	5.72	5.68	321.29	334.90	268.29	341.88	327.06	343.96
jagged 1	Jag1	100750525	1.44	1.42	2.05	2.06	1.91	1.63	217.23	216.86	301.83	308.27	292.90	249.56
jagged 2	Jag2	100753300	0.01	0.00	0.00	0.00	0.00	0.00	0.76	0.00	0.00	0.00	0.00	0.00
jagunal homolog 1 (Drosophila)	Jagn1	100756602	64.76	61.03	77.26	73.81	70.77	71.33	1634.53	1534.51	1896.67	1785.90	1693.24	1730.07
Janus kinase 1	Jak1	100754824	48.20	48.45	52.78	58.24	59.30	60.44	5619.07	5780.27	6402.96	6893.26	7045.15	7056.76
Janus kinase 2	Jak2	100769337	10.46	11.09	13.11	15.82	15.92	13.62	1485.66	1573.86	1878.04	2257.58	2251.10	1945.05
Janus kinase 3	Jak3	100773497	1.25	1.29	1.34	1.13	0.88	0.88	236.98	245.23	248.42	184.27	172.84	158.89
janus kinase and microtubule interacting protein 1	Jakmip1	100771253	0.00	0.00	0.00	0.00	0.00	0.00	0.00	0.00	0.00	0.00	0.00	0.00
janus kinase and microtubule interacting protein 2	Jakmip2	100754908	0.00	0.00	0.00	0.00	0.00	0.00	0.00	0.00	0.00	0.00	0.00	0.00
Janus kinase and microtubule interacting protein 3	Jakmip3	100750449	0.02	0.00	0.00	0.00	0.04	0.01	4.56	0.00	0.00	0.00	3.10	0.93
junctional adhesion molecule 2	Jam2	100757794	0.00	0.06	0.00	0.00	0.00	0.00	0.00	1.83	0.00	0.00	0.00	0.00
junctional adhesion molecule 3	Jam3	100758045	0.00	0.00	0.00	0.00	0.00	0.00	0.00	0.00	0.00	1.16	0.00	0.00
jumonji, AT rich interactive domain 2	Jarid2	100765016	1.79	1.80	1.16	1.31	1.12	1.32	262.04	261.70	172.65	199.33	162.49	204.69
JAZF zinc finger 1	Jazf1	100763585	0.07	0.00	0.00	0.00	0.00	0.08	1.52	0.00	0.00	0.00	3.10	0.93
Jun dimerization protein 2	Jdp2	100759902	29.96	31.81	27.89	23.81	18.38	20.58	1207.67	1280.13	1120.36	953.79	735.88	819.71
JNK1/MAPK8-associated membrane protein	Jkamp	100763824	17.39	15.71	15.77	16.71	19.73	18.35	798.28	724.71	735.32	767.20	882.84	826.25
jumonji domain containing 1C	Jmjd1c	100773596	21.20	19.67	18.67	24.33	24.94	22.40	5250.69	4895.43	4677.70	5997.41	6136.43	5533.24
jumonji domain containing 4	Jmjd4	100765049	3.28	3.31	3.50	3.84	4.06	4.00	388.88	388.89	416.10	448.50	468.85	486.03
jumonji domain containing 6	Jmjd6	100774658	58.78	59.57	64.96	56.24	45.20	45.05	2626.49	2635.30	2864.25	2459.23	1926.11	1985.24
jumonji domain containing 7	Jmjd7	100760377	22.17	21.57	24.61	21.31	17.80	20.30	813.47	797.91	921.63	803.13	670.67	737.45
jumonji domain containing 8	Jmjd8	100771483	13.33	13.20	13.79	12.86	10.64	11.12	384.33	365.10	375.11	351.15	308.43	301.90
junction mediating and regulatory protein, p53 cofactor	Jmy	100761093	17.20	17.27	19.10	20.23	20.54	21.89	1828.97	1847.45	2029.57	2142.84	2187.96	2361.91
Josephin domain containing 1	Josd1	100773825	18.47	17.91	17.90	19.31	18.69	19.20	1574.52	1529.94	1531.49	1642.19	1573.18	1631.00
Josephin domain containing 2	Josd2	100755892	31.26	28.99	28.76	15.83	7.76	7.62	650.93	588.37	581.30	319.86	168.70	166.37
junctophilin 1	Jph1	100765834	0.00	0.00	0.00	0.00	0.00	0.00	0.00	0.00	0.00	0.00	0.00	0.00
junctophilin 2	Jph2	100771806	0.13	0.09	0.04	0.05	0.05	0.04	12.91	9.15	3.73	5.79	5.17	3.74
junctophilin 3	Jph3	100755711	0.01	0.00	0.00	0.02	0.00	0.00	3.04	0.00	0.00	1.16	0.00	0.00
junctophilin 4	Jph4	100772444	0.02	0.00	0.00	0.00	0.00	0.01	1.52	0.00	0.00	0.00	0.00	0.93
Jrk homolog (mouse)	Jrk	100765280	5.60	5.21	4.78	4.67	3.37	4.04	508.89	475.82	453.36	428.80	318.78	379.48
JRK-like	Jrkl	100763189	10.35	10.37	11.07	10.86	11.52	10.43	874.23	877.52	943.99	917.86	966.68	885.13
junctional sarcoplasmic reticulum protein 1	Jsrp1	100760291	0.08	0.05	0.00	0.00	0.00	0.00	1.52	0.92	1.24	0.00	0.00	0.00
jumping translocation breakpoint	Jtb	100766005	87.47	87.60	100.70	97.67	93.04	95.33	2261.15	2258.30	2608.38	2482.41	2369.08	2455.38
jun proto-oncogene	Jun	100766306	29.79	32.40	25.19	18.90	14.69	13.42	1408.19	1508.89	1196.13	880.78	689.30	656.14
jun B proto-oncogene	Junb	100756141	22.06	22.04	12.98	6.28	5.41	4.17	695.74	680.78	402.44	191.22	167.67	128.98
junction plakoglobin	Jup	100760007	0.21	0.27	0.09	0.03	0.04	0.07	21.27	32.03	8.69	4.64	4.14	8.41
kidney associated antigen 1	Kaag1	103163943	1.16	1.28	0.88	0.85	0.59	0.54	19.75	13.73	9.94	11.59	7.24	6.54

kalirin, RhoGEF kinase	Kalrn	100757073	0.00	0.00	0.00	0.00	0.00	0.00	0.00	0.92	0.00	0.00	0.00	0.00
KN motif and ankyrin repeat domains 1	Kank1	100759671	5.05	5.11	5.65	5.87	5.62	5.37	760.30	768.63	847.10	877.30	831.09	802.88
KN motif and ankyrin repeat domains 2	Kank2	100756045	8.91	9.27	8.02	6.42	5.86	6.39	1045.13	1076.99	953.92	755.62	710.00	781.38
KN motif and ankyrin repeat domains 3	Kank3	103160001	3.44	3.51	0.84	0.93	0.00	0.00	167.10	166.54	39.75	46.36	2.07	0.00
KN motif and ankyrin repeat domains 4	Kank4	100755240	0.00	0.00	0.00	0.00	0.00	0.00	0.76	0.00	0.00	0.00	0.00	0.00
KAT8 regulatory NSL complex subunit 1-like	Kansl1l	100761258	5.69	5.70	8.72	9.46	10.12	9.33	754.22	744.84	1136.51	1206.44	1299.94	1206.66
KAT8 regulatory NSL complex subunit 2	Kansl2	100758344	24.83	24.15	27.73	27.53	29.09	29.01	2603.70	2544.71	2921.39	2862.53	2989.04	3034.87
KAT8 regulatory NSL complex subunit 3	Kansl3	100770226	33.60	33.92	34.81	33.70	30.25	32.18	4153.16	4150.59	4372.15	4121.12	3818.06	4058.34
lysyl-tRNA synthetase	Kars	100766627	244.82	241.45	258.13	275.47	277.32	283.64	14328.71	14203.15	15107.50	15762.47	15835.29	16300.64
K(lysine) acetyltransferase 2A	Kat2a	100774478	38.40	35.90	28.88	27.22	19.68	20.85	3379.19	3161.44	2542.55	2371.15	1713.94	1800.17
K(lysine) acetyltransferase 2B	Kat2b	100772054	9.24	9.61	10.49	10.98	12.10	11.73	1140.83	1209.67	1299.22	1383.75	1483.14	1445.93
K(lysine) acetyltransferase 5	Kat5	100761603	26.91	27.58	29.09	29.43	25.45	27.73	1565.41	1615.03	1688.00	1676.96	1466.58	1583.33
K(lysine) acetyltransferase 6A	Kat6a	100751361	14.49	14.65	12.05	13.56	14.05	13.96	3827.32	3877.00	3213.28	3591.49	3703.18	3732.14
K(lysine) acetyltransferase 6B	Kat6b	100760447	7.23	7.29	6.49	7.44	8.08	7.91	1368.69	1396.34	1248.30	1412.72	1505.91	1487.99
K(lysine) acetyltransferase 7	Kat7	100757223	40.19	41.32	30.95	31.16	34.86	34.89	4080.24	4161.57	3116.40	3108.22	3444.43	3462.95
K(lysine) acetyltransferase 8	Kat8	100756283	19.12	18.99	18.47	19.85	20.84	20.31	911.45	877.52	860.77	898.16	951.15	940.28
katanin p60 (ATPase containing) subunit A 1	Katna1	100759022	75.22	74.52	77.73	82.97	92.27	84.90	3515.15	3502.75	3634.35	3808.21	4212.39	3901.31
katanin p60 subunit A-like 1	Katnal1	100755046	1.36	1.68	1.21	1.49	2.16	1.70	105.58	135.42	94.40	107.78	162.49	128.05
katanin p60 subunit A-like 2	Katnal2	100772637	0.26	0.19	0.00	0.04	0.10	0.09	16.71	13.73	0.00	4.64	7.24	6.54
katanin p80 (WD repeat containing) subunit B 1	Katnb1	100763993	18.90	17.90	20.98	17.68	11.25	14.74	1512.24	1485.10	1700.42	1442.86	933.56	1195.44
katanin p80 subunit B-like 1	Katnbl1	100772848	15.73	16.94	14.49	16.95	17.20	16.07	1261.59	1367.98	1168.80	1344.35	1366.18	1285.17
Kazal-type serine peptidase inhibitor domain 1	Kazald1	100750725	4.92	5.28	3.81	3.93	3.94	3.48	341.79	374.25	278.23	273.51	269.10	253.30
kelch repeat and BTB (POZ) domain containing 12	Kbtbd12	100766790	0.00	0.00	0.00	0.00	0.00	0.00	0.00	0.00	0.00	0.00	0.00	0.00
kelch repeat and BTB (POZ) domain containing 13	Kbtbd13	100754530	0.00	0.00	0.00	0.00	0.00	0.00	0.00	0.00	0.00	0.00	0.00	0.00
kelch repeat and BTB (POZ) domain containing 2	Kbtbd2	100768350	26.06	25.16	29.60	32.19	32.55	34.03	2378.12	2330.59	2737.56	2979.58	2981.80	3133.95
kelch repeat and BTB (POZ) domain containing 3	Kbtbd3	100755102	0.32	0.30	0.40	0.43	0.54	0.58	60.76	59.48	74.53	76.49	96.25	102.81
kelch repeat and BTB (POZ) domain containing 4	Kbtbd4	100760838	11.43	10.86	12.57	12.73	12.52	14.04	762.58	733.86	854.56	856.44	848.69	956.17
kelch repeat and BTB (POZ) domain containing 8	Kbtbd8	100751978	2.29	2.50	1.68	2.21	2.10	2.13	303.82	333.07	224.82	293.21	278.41	290.68
potassium channel modulatory factor 1	Kcmf1	100753827	42.45	44.95	46.71	44.78	42.41	40.22	3762.00	4033.47	4247.94	4024.93	3804.61	3615.30
potassium voltage-gated channel, shaker-related subfamily, member 1 (episodic ataxia with myokymia)	Kcna1	100774150	0.00	0.00	0.00	0.00	0.00	0.00	0.00	0.00	0.00	0.00	0.00	0.00

potassium voltage-gated channel, shaker-related subfamily, member 10	Kcna10	100759462	0.00	0.00	0.00	0.00	0.00	0.00	0.00	0.00	0.00	0.00	0.00	0.00
potassium voltage-gated channel, shaker-related subfamily, member 2	Kcna2	100759751	0.00	0.00	0.00	0.00	0.00	0.00	0.00	0.00	0.00	0.00	0.00	0.00
potassium voltage-gated channel, shaker-related subfamily, member 3	Kcna3	100760038	0.00	0.00	0.00	0.00	0.00	0.00	0.00	0.00	0.00	0.00	0.00	0.00
potassium voltage-gated channel, shaker-related subfamily, member 4	Kcna4	100764918	0.00	0.00	0.00	0.00	0.00	0.00	0.00	0.00	0.00	0.00	0.00	0.00
potassium voltage-gated channel, shaker-related subfamily, member 5	Kcna5	100774436	0.00	0.00	0.00	0.00	0.00	0.00	0.00	0.00	0.00	0.00	0.00	0.00
potassium voltage-gated channel, shaker-related subfamily, member 6	Kcna6	100773853	0.00	0.00	0.00	0.00	0.00	0.00	0.00	0.00	0.00	0.00	0.00	0.00
potassium voltage-gated channel, shaker-related subfamily, member 7	Kcna7	100754146	0.00	0.00	0.00	0.00	0.00	0.00	0.00	0.00	0.00	1.16	0.00	0.00
potassium voltage-gated channel, shaker-related subfamily, beta member 1	Kcnab1	100755538	0.20	0.17	0.09	0.08	0.17	0.10	18.23	16.47	9.94	11.59	15.52	9.35
potassium voltage-gated channel, shaker-related subfamily, beta member 2	Kcnab2	100765085	73.66	74.72	72.81	61.60	50.54	53.75	4551.16	4596.21	4565.91	3858.04	3286.08	3382.57
potassium voltage-gated channel, shaker-related subfamily, beta member 3	Kcnab3	100755337	0.22	0.18	0.06	0.14	0.18	0.11	15.95	16.47	6.21	9.27	9.31	12.15
potassium voltage-gated channel, Shab-related subfamily, member 1	Kcnb1	100759046	0.00	0.02	0.00	0.02	0.00	0.00	0.76	1.83	0.00	0.00	0.00	0.00
potassium voltage-gated channel, Shab-related subfamily, member 2	Kcnb2	100764283	0.01	0.00	0.00	0.00	0.00	0.00	0.76	0.00	0.00	0.00	0.00	0.00
potassium voltage-gated channel, Shaw-related subfamily, member 1	Kcnc1	100763523	0.00	0.00	0.00	0.00	0.00	0.00	0.00	0.00	0.00	0.00	0.00	0.00
potassium voltage-gated channel, Shaw-related subfamily, member 2	Kcnc2	100759610	0.02	0.02	0.00	0.00	0.00	0.00	0.76	0.92	0.00	0.00	0.00	0.00
potassium voltage-gated channel, Shaw-related subfamily, member 3	Kcnc3	100773356	0.00	0.00	0.00	0.00	0.00	0.00	0.00	0.00	0.00	0.00	0.00	0.00
potassium voltage-gated channel, Shaw-related subfamily, member 4	Kcnc4	100761530	0.00	0.00	0.00	0.00	0.00	0.00	0.00	0.00	0.00	0.00	0.00	0.00
potassium voltage-gated channel, Shal-related subfamily, member 1	Kcnd1	100761662	0.01	0.02	0.00	0.02	0.00	0.00	0.76	0.92	0.00	1.16	1.03	0.00
potassium voltage-gated channel, Shal-related subfamily, member 2	Kcnd2	100767012	0.00	0.00	0.00	0.00	0.00	0.00	0.00	0.00	0.00	0.00	0.00	0.00
potassium voltage-gated channel, Shal-related subfamily, member 3	Kcnd3	100764602	0.00	0.00	0.00	0.00	0.00	0.00	0.00	0.00	0.00	0.00	0.00	0.00
potassium voltage-gated channel, Isk-related family, member 1	Kcne1	100760439	0.00	0.00	0.00	0.20	0.00	0.00	0.00	0.00	0.00	1.16	0.00	0.00
potassium voltage-gated channel, Isk-related family, member 2	Kcne2	100759561	0.00	0.00	0.00	0.00	0.00	0.00	0.00	0.92	0.00	0.00	0.00	0.00
potassium voltage-gated channel, Isk-related family, member 3	Kcne3	100755499	0.00	0.00	0.00	0.00	0.00	0.00	0.00	0.00	0.00	0.00	0.00	0.00
potassium voltage-gated channel, Isk-related family, member 4	Kcne4	100773476	0.00	0.00	0.00	0.00	0.00	0.00	0.00	0.00	0.00	0.00	0.00	0.00
potassium voltage-gated channel, subfamily F, member 1	Kcnf1	100766980	0.00	0.00	0.00	0.00	0.00	0.00	0.00	0.00	0.00	0.00	0.00	0.00
potassium voltage-gated channel, subfamily G, member 1	Kcng1	100758175	0.00	0.00	0.00	0.00	0.00	0.00	0.00	0.00	0.00	0.00	0.00	0.00
potassium voltage-gated channel, subfamily G, member 2	Kcng2	100763959	1.41	1.29	1.62	1.80	1.65	2.17	85.83	79.61	88.19	91.55	75.55	99.07

potassium voltage-gated channel, subfamily G, member 3	Kcng3	100765018	0.00	0.00	0.00	0.00	0.00	0.00	0.00	0.00	0.00	0.00	0.00	0.00
potassium voltage-gated channel, subfamily G, member 4	Kcng4	100773553	0.00	0.00	0.00	0.00	0.00	0.00	0.00	0.00	0.00	0.00	0.00	0.00
potassium voltage-gated channel, subfamily H (eag-related), member 1	Kcnh1	100764830	0.00	0.00	0.00	0.01	0.00	0.01	0.00	0.00	0.00	0.00	0.00	0.00
potassium voltage-gated channel, subfamily H (eag-related), member 2	Kcnh2	100770245	0.00	0.00	0.00	0.01	0.00	0.00	0.00	0.00	0.00	0.00	0.00	0.00
potassium voltage-gated channel, subfamily H (eag-related), member 3	Kcnh3	100762492	0.00	0.00	0.01	0.00	0.00	0.00	0.00	0.92	2.48	0.00	0.00	0.00
potassium voltage-gated channel, subfamily H (eag-related), member 4	Kcnh4	100775053	0.00	0.00	0.00	0.00	0.00	0.00	0.00	0.00	0.00	0.00	0.00	0.00
potassium voltage-gated channel, subfamily H (eag-related), member 5	Kcnh5	100767166	0.00	0.00	0.00	0.00	0.00	0.00	0.00	0.00	0.00	0.00	0.00	0.00
potassium voltage-gated channel, subfamily H (eag-related), member 6	Kcnh6	100773317	0.00	0.01	0.00	0.01	0.00	0.00	0.00	0.92	0.00	1.16	0.00	0.00
potassium voltage-gated channel, subfamily H (eag-related), member 7	Kcnh7	100764883	0.00	0.00	0.00	0.00	0.00	0.00	0.00	0.00	0.00	0.00	0.00	0.00
potassium voltage-gated channel, subfamily H (eag-related), member 8	Kcnh8	100773504	0.00	0.00	0.00	0.00	0.00	0.00	0.00	0.00	0.00	0.00	0.00	0.00
Kv channel interacting protein 1	Kcnip1	100765158	0.00	0.00	0.00	0.00	0.00	0.00	0.00	0.00	0.00	0.00	0.00	0.00
Kv channel interacting protein 2	Kcnip2	100753351	0.13	0.12	0.27	0.18	0.21	0.22	15.19	19.22	28.57	23.18	21.73	21.50
Kv channel interacting protein 3, calsenilin	Kcnip3	100774362	1.37	1.62	1.32	1.16	1.07	1.23	120.01	147.32	115.51	112.42	97.29	104.68
potassium inwardly-rectifying channel, subfamily J, member 1	Kcnj1	100766584	0.00	0.03	0.00	0.00	0.00	0.00	0.00	0.00	0.00	0.00	0.00	0.00
potassium inwardly-rectifying channel, subfamily J, member 10	Kcnj10	100764542	0.00	0.00	0.00	0.00	0.00	0.00	0.00	0.00	0.00	0.00	0.00	0.00
potassium inwardly-rectifying channel, subfamily J, member 11	Kcnj11	100764690	0.00	0.00	0.00	0.00	0.00	0.00	0.00	0.00	0.00	0.00	0.00	0.00
potassium inwardly-rectifying channel, subfamily J, member 12	Kcnj12	100773954	0.00	0.02	0.00	0.03	0.02	0.00	0.00	5.49	1.24	2.32	1.03	0.00
potassium inwardly-rectifying channel, subfamily J, member 13	Kcnj13	100774709	0.05	0.08	0.07	0.03	0.00	0.03	0.76	2.75	3.73	1.16	0.00	0.93
potassium inwardly-rectifying channel, subfamily J, member 14	Kcnj14	100775100	1.00	0.61	1.13	1.11	1.14	1.06	54.69	38.43	58.38	61.42	65.20	61.69
potassium inwardly-rectifying channel, subfamily J, member 15	Kcnj15	100763028	0.00	0.00	0.00	0.00	0.00	0.00	0.00	0.00	0.00	0.00	0.00	0.00
potassium inwardly-rectifying channel, subfamily J, member 16	Kcnj16	100754748	0.00	0.00	0.00	0.00	0.00	0.00	0.00	0.00	0.00	0.00	0.00	0.00
potassium inwardly-rectifying channel, subfamily J, member 2	Kcnj2	100754461	0.00	0.00	0.00	0.00	0.00	0.00	0.00	0.00	0.00	0.00	0.00	0.00
potassium inwardly-rectifying channel, subfamily J, member 3	Kcnj3	100768536	0.02	0.02	0.00	0.00	0.00	0.00	0.76	0.92	0.00	0.00	1.03	0.00
potassium inwardly-rectifying channel, subfamily J, member 4	Kcnj4	100766934	0.02	0.00	0.00	0.00	0.00	0.00	0.76	0.00	0.00	0.00	0.00	0.00
potassium inwardly-rectifying channel, subfamily J, member 5	Kcnj5	100766874	0.05	0.00	0.00	0.00	0.00	0.00	1.52	0.00	0.00	0.00	0.00	0.00
potassium inwardly-rectifying channel, subfamily J, member 6	Kcnj6	100763599	0.00	0.03	0.00	0.00	0.00	0.00	0.00	0.00	0.00	0.00	0.00	0.00
potassium inwardly-rectifying channel, subfamily J, member 8	Kcnj8	100770058	0.00	0.00	0.00	0.00	0.00	0.00	0.00	0.00	0.00	0.00	0.00	0.00
potassium inwardly-rectifying channel, subfamily J, member 9	Kcnj9	100764245	0.00	0.00	0.00	0.00	0.00	0.00	0.00	0.00	0.00	0.00	0.00	0.00

potassium channel, subfamily K, member 1	Kcnk1	100762483	0.00	0.00	0.00	0.00	0.00	0.00	0.00	0.00	0.00	0.00	0.00	0.00
potassium channel, subfamily K, member 10	Kcnk10	100750735	0.00	0.00	0.00	0.00	0.00	0.00	0.00	0.00	0.00	0.00	0.00	0.00
potassium channel, subfamily K, member 12	Kcnk12	100770804	0.00	0.00	0.00	0.00	0.00	0.00	0.00	3.66	0.00	0.00	0.00	0.00
potassium channel, subfamily K, member 13	Kcnk13	100769761	0.00	0.00	0.00	0.00	0.00	0.00	0.00	0.00	0.00	0.00	0.00	0.93
potassium channel, subfamily K, member 15	Kcnk15	100756837	0.02	0.06	0.08	0.06	0.02	0.01	1.52	4.58	3.73	3.48	1.03	0.93
potassium channel, subfamily K, member 16	Kcnk16	100760808	0.00	0.00	0.00	0.00	0.00	0.00	0.76	0.00	0.00	0.00	0.00	0.00
potassium channel, subfamily K, member 17	Kcnk17	100770528	0.00	0.00	0.00	0.00	0.00	0.00	0.00	0.92	0.00	0.00	0.00	0.00
potassium channel, subfamily K, member 18	Kcnk18	100774565	0.00	0.00	0.00	0.00	0.00	0.00	0.00	0.00	0.00	0.00	0.00	0.00
potassium channel, subfamily K, member 2	Kcnk2	100751502	0.03	0.03	0.00	0.10	0.00	0.04	0.76	1.83	0.00	2.32	0.00	0.93
potassium channel, subfamily K, member 3	Kcnk3	100771641	0.00	0.00	0.00	0.00	0.00	0.00	0.00	0.00	0.00	0.00	0.00	0.00
potassium channel, subfamily K, member 4	Kcnk4	100760723	0.00	0.00	0.00	0.00	0.00	0.00	0.76	1.83	0.00	0.00	0.00	0.00
potassium channel, subfamily K, member 5	Kcnk5	100760223	2.23	2.27	1.89	1.69	1.44	1.63	230.14	235.16	188.80	177.31	144.90	167.31
potassium channel, subfamily K, member 6	Kcnk6	100764262	0.00	0.00	0.00	0.00	0.00	0.00	0.00	0.00	0.00	0.00	0.00	0.00
potassium channel, subfamily K, member 7	Kcnk7	100763826	0.00	0.00	0.00	0.00	0.00	0.00	0.00	0.00	0.00	0.00	0.00	0.00
potassium channel, subfamily K, member 9	Kcnk9	100756282	0.02	0.00	0.00	0.00	0.00	0.00	0.76	0.00	0.00	0.00	0.00	0.00
potassium large conductance calcium-activated channel, subfamily M, alpha member 1	Kcnma1	100764731	0.00	0.00	0.00	0.00	0.00	0.00	0.00	0.00	0.00	0.00	0.00	0.00
potassium large conductance calcium-activated channel, subfamily M, beta member 1	Kcnmb1	100765448	0.00	0.00	0.00	0.00	0.00	0.00	0.00	0.00	0.00	0.00	0.00	0.00
potassium large conductance calcium-activated channel, subfamily M, beta member 2	Kcnmb2	100761612	0.00	0.00	0.00	0.00	0.00	0.00	0.00	0.00	0.00	0.00	0.00	0.00
potassium large conductance calcium-activated channel, subfamily M beta member 3	Kcnmb3	100758210	0.10	0.06	0.17	0.08	0.12	0.24	8.35	10.98	11.18	5.79	6.21	12.15
potassium large conductance calcium-activated channel, subfamily M, beta member 4	Kcnmb4	100754855	0.00	0.00	0.00	0.00	0.00	0.00	0.00	0.00	0.00	0.00	0.00	0.00
potassium intermediate/small conductance calcium-activated channel, subfamily N, member 1	Kcnn1	100772046	0.01	0.04	0.07	0.01	0.01	0.03	3.80	10.07	11.18	8.11	5.17	6.54
potassium intermediate/small conductance calcium-activated channel, subfamily N, member 3	Kcnn3	100760285	0.00	0.00	0.00	0.00	0.00	0.00	0.00	0.00	0.00	0.00	0.00	0.00
potassium intermediate/small conductance calcium-activated channel, subfamily N, member 4	Kcnn4	100768359	4.18	4.00	1.23	1.59	1.31	0.83	96.46	91.50	29.81	33.61	26.91	16.82

potassium voltage-gated channel, KQT-like subfamily, member 1	Kcnq1	100761481	0.00	0.00	0.00	0.00	0.00	0.00	0.00	0.00	0.00	0.00	0.00	0.00
potassium voltage-gated channel, KQT-like subfamily, member 2	Kcnq2	100766596	0.00	0.00	0.00	0.00	0.00	0.00	0.00	0.00	0.00	0.00	0.00	0.00
potassium voltage-gated channel, KQT-like subfamily, member 4	Kcnq4	100757113	0.00	0.00	0.00	0.00	0.00	0.00	0.00	0.00	0.00	1.16	0.00	0.00
potassium voltage-gated channel, KQT-like subfamily, member 5	Kcnq5	100773342	6.84	7.06	7.17	8.15	7.60	7.20	745.87	781.44	807.36	914.39	859.04	795.40
potassium channel regulator	Kcnrg	100753429	0.47	0.77	0.42	0.79	0.53	0.63	17.47	17.39	16.15	22.02	19.66	26.17
potassium voltage-gated channel, delayed-rectifier, subfamily S, member 1	Kcns1	100762339	0.00	0.00	0.00	0.00	0.00	0.00	0.76	0.00	1.24	0.00	0.00	0.00
potassium voltage-gated channel, delayed-rectifier, subfamily S, member 2	Kcns2	100755567	0.00	0.06	0.04	0.04	0.03	0.00	0.00	1.83	1.24	1.16	1.03	0.00
potassium voltage-gated channel, delayed-rectifier, subfamily S, member 3	Kcns3	100755672	0.00	0.00	0.00	0.00	0.00	0.00	0.00	0.00	0.00	0.00	0.00	0.00
potassium channel, subfamily T, member 1	Kcnt1	100758898	0.00	0.00	0.00	0.00	0.00	0.00	0.00	0.92	0.00	0.00	1.03	0.00
potassium channel, subfamily T, member 2	Kcnt2	100768557	0.01	0.00	0.03	0.00	0.00	0.00	0.76	0.00	2.48	0.00	0.00	0.00
potassium channel, subfamily U, member 1	Kcnu1	100752893	0.03	0.02	0.00	0.08	0.04	0.02	7.60	2.75	2.48	9.27	5.17	2.80
potassium channel, subfamily V, member 2	Kcnv2	100757476	0.00	0.02	0.06	0.00	0.02	0.00	0.00	1.83	2.48	0.00	2.07	0.00
kielin/chordin-like protein	Kcp	100761131	0.27	0.21	0.18	0.08	0.07	0.09	41.77	36.60	27.33	18.54	14.49	16.82
potassium channel tetramerization domain containing 1	Kctd1	100770054	3.91	3.64	4.63	4.50	4.99	4.90	113.93	109.80	127.93	130.96	146.97	145.81
potassium channel tetramerization domain containing 10	Kctd10	100752520	17.08	16.83	17.09	16.93	14.38	15.22	1623.89	1626.01	1642.04	1583.08	1345.48	1429.11
potassium channel tetramerization domain containing 11	Kctd11	100760546	3.03	3.05	3.12	2.95	2.74	2.84	230.14	235.16	240.96	221.35	202.86	214.97
potassium channel tetramerization domain containing 12	Kctd12	100753862	2.63	2.68	3.14	3.18	3.76	4.25	178.49	174.77	217.37	215.56	258.75	277.60
potassium channel tetramerization domain containing 13	Kctd13	100753748	18.83	17.79	19.30	20.83	15.18	15.63	897.78	868.37	916.66	986.24	734.84	749.61
potassium channel tetramerization domain containing 14	Kctd14	100768863	5.73	5.20	5.77	6.15	4.16	5.39	182.29	165.62	183.83	192.38	132.48	171.98
potassium channel tetramerization domain containing 15	Kctd15	100765553	10.40	8.44	11.09	5.14	5.48	7.19	275.71	236.08	301.83	142.55	145.93	187.87
potassium channel tetramerization domain containing 16	Kctd16	100755887	0.00	0.00	0.00	0.00	0.03	0.00	0.00	0.00	0.00	1.16	1.03	0.00
potassium channel tetramerization domain containing 17	Kctd17	100763063	11.85	11.33	14.49	10.83	8.41	12.23	745.11	707.32	929.08	686.08	521.63	648.66
potassium channel tetramerization domain containing 18	Kctd18	100751064	7.15	6.95	7.99	8.66	9.12	8.89	587.12	573.73	675.70	718.53	750.37	740.26
potassium channel tetramerization domain containing 19	Kctd19	100761191	0.00	0.00	0.00	0.00	0.00	0.00	1.52	0.00	1.24	0.00	0.00	0.93
potassium channel tetramerization domain containing 2	Kctd2	100757654	5.09	4.41	5.75	4.67	4.18	4.46	530.92	470.33	609.86	497.18	442.97	471.07
potassium channel tetramerization domain containing 20	Kctd20	100752436	33.06	32.27	39.30	39.45	36.27	39.30	3818.96	3717.78	4573.36	4562.67	4169.96	4541.56
potassium channel tetramerization domain containing 21	Kctd21	100765855	1.79	1.78	2.27	1.84	2.38	2.13	151.91	150.98	195.01	159.93	203.89	180.39

potassium channel tetramerization domain containing 3	Kctd3	100760825	15.85	16.55	17.09	17.66	15.66	16.90	1832.01	1935.30	1989.82	2051.29	1811.23	1952.52
potassium channel tetramerization domain containing 4	Kctd4	100768043	0.00	0.00	0.00	0.00	0.06	0.00	0.76	0.00	0.00	0.00	1.03	0.00
potassium channel tetramerization domain containing 5	Kctd5	100772768	45.13	44.69	48.10	43.26	42.06	44.95	3013.85	2994.90	3230.67	2874.12	2804.81	3003.09
potassium channel tetramerization domain containing 6	Kctd6	100765751	4.73	4.04	4.76	4.90	5.48	4.86	205.84	179.35	212.40	227.15	239.08	220.58
potassium channel tetramerization domain containing 7	Kctd7	100757850	11.74	12.47	13.81	14.09	16.84	17.44	1260.08	1324.05	1505.41	1447.49	1760.51	1823.54
potassium channel tetramerization domain containing 8	Kctd8	100754636	0.00	0.00	0.00	0.00	0.00	0.00	0.00	0.00	0.00	0.00	0.00	0.00
potassium channel tetramerization domain containing 9	Kctd9	100753176	23.71	22.42	18.25	20.32	21.84	21.75	2058.35	1970.07	1609.74	1785.90	1929.21	1905.79
KDEL (Lys-Asp-Glu-Leu) containing 1	Kdelc1	100763723	20.58	22.13	24.80	24.48	25.81	26.07	1265.39	1381.70	1538.95	1528.62	1613.54	1608.57
KDEL (Lys-Asp-Glu-Leu) containing 2	Kdelc2	100753631	50.51	50.76	62.50	68.33	74.21	70.92	6050.49	6099.61	7535.74	8205.15	8822.22	8503.62
KDEL (Lys-Asp-Glu-Leu) endoplasmic reticulum protein retention receptor 1	Kdelr1	100750912	20.49	19.05	19.68	19.40	22.14	19.99	638.77	629.54	696.81	711.58	726.56	757.08
KDEL (Lys-Asp-Glu-Leu) endoplasmic reticulum protein retention receptor 2	Kdelr2	100765889	59.96	62.44	66.16	65.88	69.35	69.48	2657.63	2778.96	2942.50	2936.70	3089.43	3093.76
KDEL (Lys-Asp-Glu-Leu) endoplasmic reticulum protein retention receptor 3	Kdelr3	100750636	41.86	41.24	44.04	43.85	41.09	39.35	2006.70	1918.83	2127.69	2135.89	2003.73	2043.19
lysine (K)-specific demethylase 1A	Kdm1a	100770721	42.06	40.48	40.93	45.47	46.88	45.02	3779.47	3632.68	3690.24	4024.93	4131.67	4007.86
lysine (K)-specific demethylase 1B	Kdm1b	100767337	10.65	10.89	10.43	10.99	13.18	11.84	1347.42	1377.13	1315.37	1388.39	1651.84	1485.19
lysine (K)-specific demethylase 2A	Kdm2a	100763039	22.46	22.28	24.12	24.14	22.09	22.89	3310.07	3343.53	3650.49	3622.78	3405.10	3484.45
lysine (K)-specific demethylase 2B	Kdm2b	100767158	8.25	7.53	8.54	8.70	7.91	8.53	1276.03	1191.37	1355.12	1374.48	1237.84	1338.45
lysine (K)-specific demethylase 3A	Kdm3a	100771108	5.53	6.00	4.48	4.81	5.46	4.90	770.17	829.02	619.80	659.43	746.23	686.05
lysine (K)-specific demethylase 3B	Kdm3b	100752012	8.85	9.00	9.54	9.42	9.35	9.77	1332.99	1378.04	1460.69	1439.38	1435.53	1478.65
lysine (K)-specific demethylase 4A	Kdm4a	100756054	55.83	54.20	56.09	51.32	50.97	53.96	6171.26	6072.16	6358.24	5766.79	5706.91	5956.65
lysine (K)-specific demethylase 4B	Kdm4b	100770247	5.39	5.27	5.14	4.30	2.69	2.98	964.61	961.70	943.99	786.91	480.23	532.76
lysine (K)-specific demethylase 4C	Kdm4c	100771667	8.25	8.17	6.90	8.44	9.49	9.41	990.44	1000.13	835.92	1015.21	1121.93	1129.08
lysine (K)-specific demethylase 4D	Kdm4d	100753944	0.07	0.10	0.03	0.11	0.09	0.13	3.04	4.58	1.24	4.64	3.10	6.54
lysine (K)-specific demethylase 5A	Kdm5a	100757111	13.40	13.53	14.33	16.39	16.07	16.21	2367.48	2401.96	2546.28	2927.43	2846.21	2832.98
lysine (K)-specific demethylase 5B	Kdm5b	100761435	10.94	11.25	9.85	10.22	9.58	9.69	2198.87	2266.54	1993.55	2055.92	1913.69	1955.33
lysine (K)-specific demethylase 5C	Kdm5c	100758549	46.08	45.00	26.33	24.78	21.82	22.64	8841.04	8667.20	5092.56	4717.96	4187.55	4338.74
lysine (K)-specific demethylase 6A	Kdm6a	100758834	12.56	12.48	7.05	8.10	7.94	7.76	1815.30	1839.22	1034.66	1179.78	1139.52	1137.49
lysine (K)-specific demethylase 6B	Kdm6b	100756212	7.68	7.31	6.37	5.90	4.40	5.23	630.42	588.37	537.82	491.38	365.35	430.88
lysine (K)-specific demethylase 7A	Kdm7a	100773228	2.93	2.78	2.12	2.43	2.78	2.48	774.73	751.24	567.63	654.79	730.70	660.81
lysine (K)-specific demethylase 8	Kdm8	100762777	10.43	8.83	9.65	9.50	6.89	7.97	452.69	392.55	412.37	420.69	281.52	339.29
kinase insert domain receptor (a type III receptor tyrosine kinase)	Kdr	100756294	0.00	0.00	0.00	0.00	0.00	0.00	0.00	0.00	0.00	0.00	0.00	0.00
3-ketodihydrosphingosine reductase	Kdsr	100774578	4.08	4.26	4.76	4.84	5.62	5.49	653.96	690.85	766.37	776.48	890.09	890.74
kelch-like ECH-associated protein 1	Keap1	100759410	12.18	10.75	13.82	13.20	11.52	12.00	1100.57	978.17	1255.75	1201.80	1032.92	1090.76
Kell blood group, metallo-endopeptidase	Kel	100758474	0.01	0.00	0.00	0.00	0.00	0.00	0.76	0.00	0.00	0.00	0.00	0.00
keratocan	Kera	100755565	0.00	0.00	0.00	0.00	0.00	0.00	0.00	0.00	0.00	0.00	0.00	0.00

KH domain containing 3-like, subcortical maternal complex member	Khdc3l	103164222	0.05	0.00	0.00	0.00	0.00	0.00	0.76	0.00	0.00	0.00	0.00	0.93
KH domain containing, RNA binding, signal transduction associated 1	Khdrbs1	100754970	73.00	79.31	75.84	72.90	76.51	76.03	5525.65	6043.80	5815.45	5510.66	5808.34	5730.46
KH domain containing, RNA binding, signal transduction associated 2	Khdrbs2	100774778	0.03	0.00	0.00	0.00	0.00	0.00	0.76	0.00	0.00	0.00	0.00	0.93
ketohexokinase (fructokinase)	Khk	100763359	6.29	6.26	6.62	6.21	3.94	5.56	236.98	228.76	249.66	230.63	159.39	218.71
KH and NYN domain containing	Khnyn	100756338	5.38	5.46	7.65	6.29	5.21	5.62	418.51	420.92	592.48	518.04	410.89	459.86
KH-type splicing regulatory protein	Khsrp	100774871	65.02	62.58	54.94	43.33	36.57	38.18	7153.34	7039.35	6188.08	4868.62	4076.81	4236.86
KIAA0020 ortholog	Kiaa0020	100764311	73.46	70.95	64.69	68.58	69.83	65.13	4638.51	4581.57	4190.80	4354.06	4401.80	4083.57
KIAA0040 ortholog	Kiaa0040	100753399	15.36	14.92	12.46	13.50	15.38	13.78	806.63	777.78	690.60	715.05	774.17	750.54
KIAA0100 ortholog	Kiaa0100	100761624	240.90	238.60	237.36	254.09	251.86	265.97	22404.13	22583.03	22564.99	23786.83	23236.48	24855.67
KIAA0101 ortholog	Kiaa0101	100755621	50.93	55.36	39.88	41.01	43.97	40.32	1146.14	1247.19	885.61	908.59	997.73	901.96
KIAA0141 ortholog	Kiaa0141	100774899	23.47	23.92	19.04	19.03	17.09	18.36	1621.62	1642.49	1320.34	1303.78	1129.17	1215.07
KIAA0195 ortholog	Kiaa0195	100769338	6.03	5.47	6.60	5.52	3.94	4.53	937.27	858.30	1006.09	848.33	619.96	715.02
KIAA0196 ortholog	Kiaa0196	103158844	206.90	208.65	169.61	162.80	168.41	170.18	2907.52	2952.81	2327.67	2197.31	2246.96	2275.92
KIAA0226 ortholog	Kiaa0226	100754353	10.69	10.47	13.23	13.40	13.30	13.42	1696.05	1678.17	2158.75	2168.34	2121.72	2163.76
KIAA0226-like ortholog	Kiaa0226l	100760044	0.00	0.00	0.00	0.02	0.00	0.00	0.00	0.00	0.00	1.16	1.03	0.00
KIAA0232 ortholog	Kiaa0232	100769248	4.06	4.14	3.79	4.28	4.61	4.66	796.00	816.21	746.49	854.12	896.30	922.52
KIAA0247 ortholog	Kiaa0247	100757573	23.44	21.33	17.53	17.02	15.58	17.94	2438.12	2267.45	1895.42	1813.71	1650.80	1908.60
KIAA0319 ortholog	Kiaa0319	100759222	0.00	0.02	0.00	0.01	0.02	0.01	0.76	2.75	0.00	1.16	2.07	0.93
KIAA0319-like ortholog	Kiaa0319l	100766589	20.28	19.05	20.61	20.24	17.82	19.15	2644.72	2533.73	2728.86	2622.64	2348.38	2469.40
KIAA0355 ortholog	Kiaa0355	100764974	0.05	0.04	0.00	0.00	0.00	0.01	5.32	3.66	0.00	0.00	1.03	0.93
KIAA0368 ortholog	Kiaa0368	100757449	43.52	44.32	44.27	50.66	50.18	48.17	7653.88	7817.13	7827.63	8814.74	8709.41	8423.24
KIAA0391 ortholog	Kiaa0391	100757198	25.26	22.89	25.17	26.28	25.71	26.78	454.96	397.12	433.49	491.38	491.62	485.09
KIAA0408 ortholog	Kiaa0408	100765589	0.03	0.03	0.00	0.00	0.00	0.00	3.80	1.83	0.00	0.00	0.00	0.00
KIAA0430 ortholog	Kiaa0430	100771777	7.34	7.70	8.02	8.86	10.45	10.17	1614.78	1711.11	1793.57	1938.87	2305.95	2249.75
KIAA0513 ortholog	Kiaa0513	100771533	2.17	1.95	1.89	2.05	1.71	1.95	151.15	145.49	127.93	106.62	105.57	113.10
KIAA0556 ortholog	Kiaa0556	100763995	4.70	4.61	5.73	5.24	5.55	5.49	773.21	769.54	950.20	877.30	919.07	901.96
KIAA0586 ortholog	Kiaa0586	100753699	4.53	4.29	7.16	6.27	7.18	5.73	530.16	525.23	603.65	680.29	736.91	674.83
KIAA0753 ortholog	Kiaa0753	100755478	2.21	2.04	1.98	2.51	2.64	2.80	282.55	255.29	268.29	310.59	315.67	343.02

KIAA0825 ortholog	Kiaa0825	100754810	2.42	2.33	2.76	3.15	3.77	3.70	297.74	278.17	329.15	354.63	421.24	429.01
KIAA0895 ortholog	Kiaa0895	100768942	2.41	2.68	2.61	2.90	3.59	2.93	148.11	169.28	162.71	172.68	225.63	183.20
KIAA0895-like ortholog	Kiaa0895l	100759739	3.21	2.93	2.92	2.41	3.08	3.55	271.92	244.31	238.48	199.33	269.10	290.68
KIAA0907 ortholog	Kiaa0907	100764357	26.76	27.61	28.69	29.55	30.82	31.37	2020.37	2101.83	2230.79	2208.90	2334.93	2401.17
KIAA0922 ortholog	Kiaa0922	100771764	5.94	6.29	6.79	7.52	7.45	7.43	985.12	1077.91	1148.93	1258.59	1260.61	1231.89
KIAA0930 ortholog	Kiaa0930	100759763	16.28	16.82	16.44	13.57	8.80	10.28	1120.32	1184.97	1150.17	944.52	611.68	720.63
KIAA0947 ortholog	Kiaa0947	100758843	15.45	15.68	15.94	15.83	15.71	16.63	3710.35	3801.05	3847.99	3775.76	3795.29	4019.08
KIAA1024 ortholog	Kiaa1024	100758044	2.36	2.25	1.44	1.56	1.92	1.76	520.28	493.20	320.46	349.99	418.13	386.95
KIAA1024-like ortholog	Kiaa1024l	100772872	0.00	0.00	0.00	0.00	0.00	0.00	0.00	0.00	0.00	0.00	0.00	0.00
KIAA1033 ortholog	Kiaa1033	100772083	7.19	6.84	6.52	7.54	9.26	8.75	1238.05	1200.52	1155.14	1324.65	1631.14	1502.95
KIAA1045 ortholog	Kiaa1045	100770157	0.00	0.00	0.00	0.00	0.00	0.00	0.00	0.00	0.00	0.00	0.00	0.00
KIAA1107 ortholog	Kiaa1107	100769097	3.04	3.06	3.00	3.55	3.61	3.21	518.77	509.67	511.74	602.64	597.19	538.37
KIAA1109 ortholog	Kiaa1109	100756071	12.49	11.63	11.61	14.39	16.04	14.93	1510.72	1393.60	1413.49	1749.97	1933.35	1781.48
KIAA1143 ortholog	Kiaa1143	100764346	9.69	11.28	12.33	13.02	16.71	12.07	216.47	271.76	295.62	327.97	431.59	321.53
KIAA1147 ortholog	Kiaa1147	100750485	7.27	7.52	7.30	7.34	7.25	7.40	1357.30	1410.07	1376.23	1376.80	1349.62	1390.79
KIAA1161 ortholog	Kiaa1161	103164296	1.20	1.60	1.06	1.08	0.97	1.09	37.22	51.24	32.29	35.93	32.08	38.32
KIAA1191 ortholog	Kiaa1191	100751525	28.10	27.56	29.60	29.52	29.05	30.22	1807.70	1780.66	1928.96	1862.38	1802.95	1902.05
KIAA1210 ortholog	Kiaa1210	103159675	0.00	0.00	0.00	0.00	0.00	0.00	0.76	0.00	0.00	0.00	1.03	0.00
KIAA1211 ortholog	Kiaa1211	100770019	0.04	0.03	0.01	0.00	0.00	0.01	5.32	2.75	1.24	1.16	0.00	0.93
KIAA1211-like ortholog	Kiaa1211l	100764496	0.02	0.05	0.02	0.01	0.01	0.02	3.04	8.24	2.48	2.32	1.03	1.87
KIAA1217 ortholog	Kiaa1217	100767111	0.01	0.01	0.00	0.00	0.00	0.00	0.76	1.83	0.00	0.00	0.00	0.00
KIAA1244 ortholog	Kiaa1244	100771638	0.00	0.00	0.00	0.00	0.00	0.01	0.76	0.00	0.00	0.00	0.00	1.87
KIAA1279 ortholog	Kiaa1279	100770903	16.74	18.86	17.77	20.17	21.97	22.27	1152.22	1301.18	1235.88	1381.43	1503.84	1530.99
KIAA1324 ortholog	Kiaa1324	100761426	0.05	0.06	0.06	0.02	0.00	0.00	6.08	2.75	3.73	1.16	0.00	0.00
KIAA1324-like ortholog	Kiaa1324l	100760915	0.02	0.02	0.00	0.01	0.00	0.00	2.28	1.83	0.00	1.16	0.00	0.00
KIAA1328 ortholog	Kiaa1328	100763806	3.76	4.21	3.49	4.15	4.65	4.13	406.35	441.96	352.75	434.59	491.62	423.41
KIAA1377 ortholog	Kiaa1377	100762281	1.67	1.31	1.16	1.56	1.90	1.68	180.77	137.26	130.42	168.04	195.61	179.46

KIAA1407 ortholog	Kiaa1407	100773871	2.48	1.99	1.54	1.62	1.93	1.70	251.41	221.44	163.96	169.20	198.72	198.15
KIAA1429 ortholog	Kiaa1429	100759227	8.23	8.74	8.94	9.62	10.69	10.36	1407.43	1497.91	1561.30	1659.57	1842.28	1791.76
KIAA1430 ortholog	Kiaa1430	100752065	11.17	11.31	9.90	10.95	13.11	12.11	1272.23	1261.83	1140.24	1250.47	1471.75	1373.03
KIAA1432 ortholog	Kiaa1432	100760041	5.95	5.66	6.01	6.88	7.14	6.39	1192.48	1154.77	1247.06	1395.34	1442.77	1306.67
KIAA1456 ortholog	Kiaa1456	100771357	0.00	0.00	0.00	0.00	0.00	0.00	0.00	0.00	0.00	0.00	0.00	0.00
KIAA1462 ortholog	Kiaa1462	100762341	0.00	0.02	0.00	0.00	0.00	0.00	0.00	1.83	0.00	0.00	0.00	0.00
KIAA1467 ortholog	Kiaa1467	100773310	1.89	1.97	1.98	2.15	1.91	1.97	175.45	188.50	186.31	203.97	174.91	181.33
KIAA1468 ortholog	Kiaa1468	100750853	10.23	10.04	8.68	9.94	10.97	10.50	1699.85	1697.39	1484.29	1651.46	1839.17	1722.60
KIAA1522 ortholog	Kiaa1522	100751553	18.01	16.55	17.39	16.80	13.78	13.95	2653.83	2450.46	2587.27	2484.72	2022.36	2059.08
KIAA1524 ortholog	Kiaa1524	100760685	8.13	7.79	7.94	9.70	10.69	10.01	1300.33	1280.13	1291.77	1533.25	1699.45	1583.33
KIAA1549 ortholog	Kiaa1549	100771392	2.20	2.20	2.94	2.77	2.37	2.26	532.44	530.72	701.78	662.90	564.07	537.44
KIAA1549-like ortholog	Kiaa1549l	100755155	0.00	0.00	0.00	0.00	0.00	0.00	0.00	0.92	0.00	0.00	0.00	0.00
KIAA1551 ortholog	Kiaa1551	100762086	9.30	8.64	7.89	10.53	14.32	13.24	1481.86	1396.34	1276.87	1702.45	2292.49	2141.33
KIAA1598 ortholog	Kiaa1598	100774279	3.64	3.57	2.50	2.44	2.81	2.49	427.62	449.28	319.22	289.73	327.06	302.83
KIAA1614 ortholog	Kiaa1614	100754212	1.24	1.21	1.37	0.99	0.75	0.86	181.53	180.26	198.73	143.71	108.67	126.18
KIAA1644 ortholog	Kiaa1644	100765500	0.00	0.00	0.00	0.00	0.00	0.00	0.00	0.00	0.00	0.00	0.00	0.00
KIAA1683 ortholog	Kiaa1683	100769749	0.00	0.01	0.01	0.01	0.00	0.00	0.00	0.92	1.24	1.16	0.00	0.00
KIAA1715 ortholog	Kiaa1715	100753894	17.05	16.72	20.82	21.20	22.15	22.76	1173.49	1168.50	1459.45	1532.09	1648.73	1658.10
KIAA1731 ortholog	Kiaa1731	100755683	8.62	8.37	7.29	9.42	11.24	9.79	2037.84	2009.41	1776.18	2285.39	2694.07	2348.82
KIAA1751 ortholog	Kiaa1751	100756275	0.00	0.00	0.00	0.00	0.00	0.00	0.00	0.00	0.00	0.00	0.00	0.00
KIAA1755 ortholog	Kiaa1755	100764914	0.02	0.03	0.01	0.01	0.00	0.00	2.28	4.58	1.24	1.16	0.00	1.87
KIAA1841 ortholog	Kiaa1841	100755497	7.73	7.86	6.47	7.86	9.74	8.98	1111.21	1132.81	960.13	1088.23	1370.32	1300.13
KIAA1919 ortholog	Kiaa1919	100771202	4.43	5.15	5.17	5.89	6.88	6.27	382.81	436.47	448.39	501.81	567.17	547.72
KIAA1958 ortholog	Kiaa1958	100769984	1.43	1.37	1.21	2.18	2.14	1.74	151.15	121.70	125.45	181.95	161.46	156.09
KIAA2013 ortholog	Kiaa2013	100761634	23.15	23.06	25.55	18.45	13.47	14.72	1287.42	1273.73	1415.98	1014.05	734.84	805.69
KIAA2018 ortholog	Kiaa2018	100768025	2.21	1.94	1.64	2.24	2.43	2.13	548.39	484.05	419.83	558.60	604.43	532.76
KIAA2022 ortholog	Kiaa2022	100760705	0.01	0.00	0.00	0.00	0.00	0.01	0.76	0.00	0.00	0.00	0.00	0.93

KIAA2026 ortholog	Kiaa2026	100763428	3.88	3.86	3.63	5.03	5.00	4.62	792.96	786.93	750.22	1024.49	1019.46	951.49
kinase D-interacting substrate, 220kDa	Kidins220	100768633	14.07	13.94	13.28	14.76	16.26	16.12	2994.10	2982.09	2829.47	3140.67	3429.94	3409.68
kinesin family member 12	Kif12	100755270	0.00	0.00	0.00	0.00	0.00	0.00	0.00	0.00	0.00	0.00	0.00	0.00
kinesin family member 13A	Kif13a	100762096	4.23	4.41	5.07	4.98	4.75	5.12	910.69	932.42	1089.31	1074.32	1022.57	1096.37
kinesin family member 13B	Kif13b	100759580	2.52	2.46	2.39	2.61	2.72	2.56	698.78	684.45	678.18	724.32	756.57	722.50
kinesin family member 14	Kif14	100759691	10.50	9.80	10.18	12.71	12.64	11.81	2193.55	2045.10	2133.91	2640.02	2608.17	2463.79
kinesin family member 15	Kif15	100762343	30.83	31.25	26.81	32.29	35.23	31.95	4464.57	4565.10	3912.57	4644.95	4995.88	4592.97
kinesin family member 16B	Kif16b	100758004	11.89	11.52	12.29	12.50	12.83	13.20	1834.29	1743.14	1866.86	1840.36	1945.77	1994.59
kinesin family member 17	Kif17	100757590	0.01	0.07	0.00	0.03	0.00	0.00	1.52	5.49	1.24	3.48	0.00	0.00
kinesin family member 18A	Kif18a	100770187	9.78	9.97	9.63	11.83	12.69	10.58	960.82	998.30	955.16	1165.87	1229.56	1026.27
kinesin family member 18B	Kif18b	100765148	31.65	31.09	20.23	19.90	17.20	17.75	2801.94	2754.25	1796.06	1758.08	1502.80	1576.79
kinesin family member 19	Kif19	100751211	0.00	0.00	0.00	0.00	0.00	0.00	0.00	0.00	0.00	0.00	0.00	0.00
kinesin family member 1A	Kif1a	100770614	0.00	0.00	0.00	0.00	0.00	0.00	0.00	0.00	0.00	1.16	0.00	0.00
kinesin family member 1C	Kif1c	100753779	28.03	30.44	29.05	28.45	24.12	26.29	2489.77	2703.01	2557.46	2481.25	2089.64	2290.88
kinesin family member 20A	Kif20a	100773707	26.52	28.52	30.79	34.19	35.99	34.88	2381.16	2577.65	2838.17	3069.98	3218.81	3018.05
kinesin family member 20B	Kif20b	100764040	13.97	13.08	12.23	16.26	16.86	14.70	2344.70	2188.76	2035.78	2684.06	2782.04	2418.93
kinesin family member 21A	Kif21a	100766958	28.00	29.10	29.95	38.64	45.67	36.49	1443.88	1407.32	1697.93	2006.09	2143.46	1868.40
kinesin family member 21B	Kif21b	100766089	0.08	0.12	0.06	0.04	0.03	0.04	28.10	29.28	18.63	12.75	7.24	14.02
kinesin family member 22	Kif22	100751676	59.53	61.09	62.77	62.28	61.92	66.73	3736.17	3904.45	3977.16	3866.16	3868.78	4080.77
kinesin family member 23	Kif23	100682531	18.68	18.42	16.91	19.43	21.55	19.32	1780.36	1783.40	1637.07	1857.75	2103.09	1828.21
kinesin family member 24	Kif24	100770944	0.99	1.03	1.38	1.33	1.55	1.38	161.78	162.88	231.03	227.15	248.40	224.32
kinesin family member 25	Kif25	100750864	0.00	0.00	0.00	0.00	0.00	0.00	0.00	0.00	0.00	0.00	0.00	0.93
kinesin family member 26A	Kif26a	100750948	0.00	0.01	0.00	0.00	0.00	0.00	0.00	0.92	1.24	1.16	0.00	0.00
kinesin family member 26B	Kif26b	100764244	0.04	0.02	0.00	0.00	0.00	0.00	6.84	3.66	0.00	0.00	0.00	0.00
kinesin family member 27	Kif27	100764407	0.10	0.11	0.10	0.11	0.11	0.07	31.14	33.86	29.81	27.81	25.87	19.63
kinesin family member 28, pseudogene	Kif28p	103161207	1.94	1.64	1.64	1.63	1.62	1.33	0.76	0.92	0.00	0.00	1.03	2.80
kinesin heavy chain member 2A	Kif2a	100756108	14.65	14.40	13.40	16.13	16.27	15.98	1717.32	1695.56	1543.91	1858.91	1903.34	1794.57
kinesin family member 2B	Kif2b	100760507	0.00	0.00	0.00	0.00	0.00	0.00	0.00	0.00	0.00	0.00	0.00	0.00
kinesin family member 2C	Kif2c	100689309	24.24	23.73	21.93	24.60	26.46	25.89	2094.81	2105.49	1932.69	2110.39	2334.93	2202.08
kinesin family member 3A	Kif3a	100755936	4.83	4.92	5.46	5.65	6.65	6.48	770.93	827.19	878.16	912.07	1080.53	1048.70
kinesin family member 3B	Kif3b	100753120	8.83	7.95	9.34	10.07	11.74	11.99	852.20	759.48	920.39	970.02	1114.68	1139.36
kinesin family member 3C	Kif3c	100757377	4.69	4.78	6.48	6.10	5.80	5.10	573.45	586.54	804.87	747.50	713.11	632.77
kinesin family member 4A	Kif4a	100769995	12.40	11.93	10.31	12.35	13.19	12.11	1697.57	1642.49	1430.88	1672.32	1793.63	1644.08
kinesin family member 5A	Kif5a	100763800	0.14	0.20	0.17	0.14	0.19	0.20	20.51	23.79	17.39	16.22	24.84	24.30
kinesin family member 5B	Kif5b	100774173	62.16	59.24	54.41	64.10	67.40	62.19	9987.18	9559.36	8800.19	10226.31	10680.02	9964.51
kinesin family member 5C	Kif5c	100773258	0.01	0.01	0.00	0.00	0.00	0.00	1.52	0.92	0.00	0.00	0.00	0.00
kinesin family member 6	Kif6	100770243	0.00	0.00	0.00	0.00	0.00	0.00	0.00	0.92	0.00	0.00	0.00	0.00
kinesin family member 7	Kif7	100762172	7.84	7.89	7.18	5.04	3.83	4.23	1058.80	1062.35	961.38	708.10	550.61	615.01
kinesin family member 9	Kif9	100768828	0.33	0.43	0.45	0.50	0.59	0.65	38.74	49.41	57.14	59.10	65.20	75.71
kinesin-associated protein 3	Kifap3	100754239	21.92	22.81	19.73	23.00	25.40	24.39	1662.63	1708.37	1470.63	1688.55	1865.05	1840.36
kinesin family member C1	Kifc1	100752355	42.11	40.25	38.44	31.02	22.29	24.06	2710.04	2612.42	2543.79	2026.95	1498.66	1607.63
kinesin family member C2	Kifc2	100754821	0.81	0.82	0.86	0.58	0.78	0.80	91.14	100.65	96.88	64.90	77.62	85.05
kinesin family member C3	Kifc3	100763525	2.67	2.36	1.79	1.40	1.33	1.13	276.47	254.38	185.07	150.66	134.55	109.36

KIN, antigenic determinant of recA protein homolog (mouse)	Kin	100761113	6.75	6.82	4.98	7.32	8.43	7.06	243.81	241.57	199.98	257.28	309.46	263.58
kin of IRRE like (Drosophila)	Kirrel	100774583	46.04	43.88	41.93	42.92	42.93	43.23	8556.21	8209.68	7867.38	8061.45	7996.30	8040.03
kin of IRRE like 2 (Drosophila)	Kirrel2	100773329	0.00	0.00	0.00	0.00	0.00	0.00	0.00	0.00	0.00	0.00	0.00	0.00
kin of IRRE like 3 (Drosophila)	Kirrel3	100762032	0.00	0.00	0.00	0.00	0.00	0.00	0.00	0.00	0.00	0.00	0.00	0.00
KiSS-1 metastasis-suppressor	Kiss1	100762823	0.00	0.00	0.00	0.00	0.00	0.00	0.00	0.00	0.00	0.00	0.00	0.00
KISS1 receptor	Kiss1r	100753032	0.68	0.51	0.51	0.32	0.40	0.65	25.82	21.96	17.39	9.27	12.42	19.63
v-kit Hardy-Zuckerman 4 feline sarcoma viral oncogene homolog	Kit	100751027	0.00	0.00	0.00	0.00	0.00	0.00	0.00	0.00	0.00	0.00	0.00	0.00
KIT ligand	Kitlg	100752139	10.39	10.65	8.60	9.90	11.18	10.10	1547.94	1606.80	1295.50	1491.53	1671.50	1524.45
kizuna centrosomal protein	Kiz	100770594	13.34	12.32	14.14	16.32	18.36	16.07	994.24	930.59	1081.86	1226.14	1362.04	1210.40
klotho	Kl	100758189	0.00	0.01	0.00	0.00	0.00	0.00	0.00	1.83	0.00	0.00	0.00	0.00
klotho beta	Klb	100758821	0.02	0.05	0.06	0.07	0.05	0.04	3.04	4.58	3.73	10.43	4.14	6.54
kinesin light chain 1	Klc1	100689219	97.55	94.98	82.09	79.13	79.00	84.03	6313.29	6206.67	5249.06	4991.47	4994.84	5322.94
kinesin light chain 2	Klc2	100764190	32.67	30.60	33.52	28.54	21.71	24.35	3007.78	2821.05	3108.94	2620.32	2000.63	2215.17
kinesin light chain 4	Klc4	100774201	16.03	16.27	16.22	13.52	11.04	11.15	1187.92	1203.27	1219.73	1012.90	821.78	854.29
Kruppel-like factor 1 (erythroid)	Klf1	100763392	0.15	0.16	0.12	0.15	0.18	0.12	6.08	8.24	4.97	5.79	8.28	4.67
Kruppel-like factor 10	Klf10	100775085	13.92	14.65	10.49	9.77	8.56	8.36	733.71	737.52	556.45	497.18	429.52	416.86
Kruppel-like factor 11	Klf11	100753145	10.17	9.77	9.27	9.67	9.85	9.01	1443.88	1398.17	1329.03	1377.96	1396.20	1283.30
Kruppel-like factor 12	Klf12	100751247	0.02	0.00	0.00	0.00	0.00	0.00	0.76	0.00	0.00	0.00	0.00	1.87
Kruppel-like factor 14	Klf14	100764325	0.00	0.04	0.00	0.00	0.00	0.00	0.00	0.92	0.00	0.00	0.00	0.00
Kruppel-like factor 15	Klf15	100761798	0.00	0.00	0.00	0.00	0.00	0.00	0.00	0.00	0.00	0.00	0.00	0.00
Kruppel-like factor 16	Klf16	100758273	9.61	9.62	8.85	7.92	6.24	6.90	1133.23	1132.81	1040.87	934.09	726.56	814.10
Kruppel-like factor 17	Klf17	100774184	0.00	0.00	0.00	0.00	0.00	0.00	0.00	0.00	0.00	0.00	0.00	0.00
Kruppel-like factor 3 (basic)	Klf3	100762113	6.85	7.74	7.08	8.66	5.49	5.31	951.70	1055.95	966.34	1191.37	736.91	725.30
Kruppel-like factor 4 (gut)	Klf4	100763533	30.77	30.79	24.57	24.25	23.29	22.08	2384.19	2395.56	1923.99	1872.81	1793.63	1725.40
Kruppel-like factor 5 (intestinal)	Klf5	100774271	35.86	35.94	35.85	37.36	44.15	40.26	2713.83	2733.21	2737.56	2831.24	3329.55	3081.61
Kruppel-like factor 6	Klf6	100750724	69.77	68.18	58.76	62.62	72.86	71.53	3073.10	3060.79	2723.90	2845.15	3387.51	3259.19
Kruppel-like factor 7 (ubiquitous)	Klf7	100755667	6.21	6.20	2.95	4.08	5.50	5.36	737.51	744.84	358.96	490.22	653.08	645.86
Kruppel-like factor 8	Klf8	100765939	3.41	3.22	2.14	2.38	3.07	2.97	739.03	706.41	470.75	519.20	652.04	643.05
Kruppel-like factor 9	Klf9	100771438	18.51	18.16	14.47	16.26	15.12	13.93	1276.03	1278.30	1003.61	1131.11	1047.41	979.53
kelch domain containing 1	Klhdc1	100753687	2.56	2.80	2.97	3.65	4.54	3.83	124.56	135.42	134.15	161.09	192.51	178.52
kelch domain containing 10	Klhdc10	100764028	14.14	13.97	16.26	15.72	17.44	18.28	897.78	909.54	1079.37	1017.53	1118.82	1190.77
kelch domain containing 2	Klhdc2	100755535	48.75	50.13	57.66	58.99	63.36	59.71	1948.98	2050.59	2301.59	2360.72	2562.63	2305.83
kelch domain containing 3	Klhdc3	100770840	32.35	30.73	38.44	39.19	33.33	35.94	1593.51	1537.26	1971.19	1920.33	1619.75	1717.92
kelch domain containing 4	Klhdc4	100768960	36.92	37.81	35.82	31.37	26.39	26.39	2173.80	2210.72	2138.87	1824.14	1546.27	1565.57
kelch domain containing 7A	Klhdc7a	100769888	0.00	0.00	0.00	0.00	0.00	0.00	0.00	0.00	0.00	0.00	0.00	0.00
kelch domain containing 7B	Klhdc7b	100769456	0.00	0.00	0.00	0.00	0.00	0.00	0.00	0.00	0.00	0.00	0.00	0.00
kelch domain containing 8A	Klhdc8a	100754881	0.00	0.00	0.00	0.00	0.00	0.00	0.76	0.00	0.00	1.16	1.03	0.00
kelch domain containing 8B	Klhdc8b	100764543	0.46	0.37	0.31	0.32	0.30	0.27	38.74	29.28	26.08	26.66	34.15	32.71
kelch domain containing 9	Klhdc9	100766323	5.85	5.07	5.36	5.15	4.29	5.03	320.53	314.77	342.82	304.80	229.77	256.10
kelch-like family member 1	Klhl1	100772180	0.31	0.24	0.12	0.12	0.04	0.14	35.70	22.88	12.42	13.91	8.28	14.02
kelch-like family member 10	Klhl10	100755541	1.96	1.10	1.30	1.33	1.12	1.17	44.05	43.01	26.08	27.81	32.08	41.13

kelch-like family member 11	Klhl11	100750471	18.35	19.57	18.06	19.53	21.38	21.57	1121.08	1197.78	1137.75	1202.96	1321.68	1394.53
kelch-like family member 12	Klhl12	100752808	17.06	16.08	17.51	18.73	21.11	23.11	1106.65	1053.20	1142.72	1216.87	1385.85	1502.95
kelch-like family member 13	Klhl13	100764640	0.03	0.05	0.03	0.06	0.01	0.06	2.28	4.58	2.48	4.64	1.03	4.67
kelch-like family member 14	Klhl14	100773910	0.00	0.00	0.00	0.00	0.00	0.00	0.00	0.00	0.00	0.00	0.00	0.00
kelch-like family member 15	Klhl15	100768724	1.08	1.11	1.78	1.55	1.95	2.13	191.40	200.39	314.25	281.62	344.65	376.67
kelch-like family member 17	Klhl17	100772708	6.23	6.00	8.00	7.98	7.22	7.59	555.22	543.53	705.51	688.40	618.92	676.70
kelch-like family member 18	Klhl18	100765054	6.50	6.26	5.83	5.21	3.85	4.07	486.86	464.84	438.46	395.19	293.94	305.64
kelch-like family member 2	Klhl2	100757106	19.73	19.58	22.46	23.93	26.11	26.75	1629.97	1628.76	1861.89	1944.67	2137.25	2200.21
kelch-like family member 20	Klhl20	100774070	12.18	12.04	13.04	13.63	14.98	13.99	1389.20	1386.28	1502.93	1539.05	1683.92	1605.76
kelch-like family member 21	Klhl21	100760231	19.57	19.53	28.03	22.59	17.96	21.02	502.06	527.97	714.20	571.35	494.72	498.18
kelch-like family member 22	Klhl22	100752906	34.91	33.44	37.66	35.66	30.80	34.66	2516.35	2442.22	2733.83	2561.21	2199.35	2513.33
kelch-like family member 23	Klhl23	100765014	4.66	5.12	6.19	7.15	7.48	7.56	397.24	450.20	557.70	630.45	642.73	659.88
kelch-like family member 24	Klhl24	100758788	8.58	9.54	12.31	12.95	12.93	11.74	592.44	651.50	845.86	909.75	890.09	826.25
kelch-like family member 25	Klhl25	100767883	5.31	5.76	4.80	4.21	3.40	3.85	498.26	536.21	453.36	395.19	322.92	373.87
kelch-like family member 26	Klhl26	100756815	2.91	2.59	3.28	2.91	2.34	2.25	266.60	245.23	314.25	264.23	217.35	224.32
kelch-like family member 28	Klhl28	100755905	2.99	3.03	3.08	2.99	3.87	3.58	338.75	346.80	353.99	348.83	430.55	414.99
kelch-like family member 29	Klhl29	100761820	0.00	0.00	0.00	0.00	0.00	0.00	0.00	0.00	0.00	0.00	0.00	0.00
kelch-like family member 3	Klhl3	100753683	0.23	0.17	0.07	0.09	0.23	0.14	15.95	15.56	6.21	6.95	17.59	7.48
kelch-like family member 30	Klhl30	100772873	0.00	0.00	0.00	0.05	0.00	0.00	0.00	0.00	0.00	2.32	0.00	0.00
kelch-like family member 31	Klhl31	100752315	0.00	0.02	0.00	0.00	0.00	0.00	0.00	0.92	0.00	0.00	0.00	0.00
kelch-like family member 32	Klhl32	100768357	0.07	0.04	0.00	0.00	0.00	0.02	3.80	3.66	1.24	1.16	1.03	0.93
kelch-like family member 33	Klhl33	100754250	0.09	0.02	0.12	0.03	0.10	0.05	6.84	1.83	9.94	2.32	7.24	5.61
kelch-like family member 34	Klhl34	100752600	0.89	0.99	0.86	1.04	0.84	0.97	101.78	109.80	96.88	118.21	95.22	113.10
kelch-like family member 35	Klhl35	100757747	0.00	0.00	0.00	0.00	0.03	0.03	1.52	0.92	1.24	1.16	2.07	2.80
kelch-like family member 36	Klhl36	100772106	3.83	4.38	3.74	3.69	2.29	2.65	237.74	272.68	237.24	236.42	151.11	177.59
kelch-like family member 38	Klhl38	100764038	0.23	0.14	0.06	0.04	0.03	0.00	3.04	0.92	1.24	1.16	0.00	0.00
kelch-like family member 4	Klhl4	100764135	2.11	2.32	2.57	2.46	3.03	3.30	221.03	248.89	278.23	271.19	310.50	357.04
kelch-like family member 40	Klhl40	100771575	0.02	0.00	0.00	0.00	0.00	0.00	0.76	0.00	0.00	0.00	0.00	0.00
kelch-like family member 41	Klhl41	100763060	0.00	0.00	0.00	0.00	0.00	0.00	0.00	0.00	0.00	0.00	0.00	0.00
kelch-like family member 42	Klhl42	100757442	16.92	16.84	20.01	20.09	22.66	21.74	1421.10	1431.11	1697.93	1693.18	1862.98	1841.30
kelch-like family member 6	Klhl6	100758501	0.00	0.00	0.00	0.00	0.00	0.00	0.00	0.00	0.00	0.00	0.00	0.00
kelch-like family member 7	Klhl7	100763513	45.66	48.67	56.07	59.62	63.67	62.37	2334.82	2483.40	2865.49	3051.44	3202.25	3196.57
kelch-like family member 8	Klhl8	100751625	4.56	4.39	2.59	3.29	3.55	3.48	387.37	376.08	221.09	279.30	301.18	293.49
kelch-like family member 9	Klhl9	100765563	33.40	34.08	35.46	37.97	43.74	41.51	3765.04	3846.80	4033.06	4293.80	4912.05	4720.08
kallikrein 1	Klk1	100754155	0.00	0.00	0.00	0.00	0.00	0.00	0.00	0.00	0.00	0.00	0.00	0.00
kallikrein-related peptidase 10	Klk10	100752352	0.00	0.00	0.00	0.00	0.00	0.00	0.00	0.00	0.00	0.00	0.00	0.00
kallikrein-related peptidase 11	Klk11	100756472	0.00	0.00	0.00	0.00	0.00	0.00	0.00	0.00	0.00	0.00	0.00	0.00
kallikrein-related peptidase 12	Klk12	100752058	0.00	0.00	0.00	0.00	0.00	0.00	0.00	0.00	0.00	0.00	0.00	0.00
kallikrein-related peptidase 13	Klk13	100761429	0.00	0.00	0.00	0.00	0.00	0.00	0.00	0.00	0.00	0.00	0.00	0.00
kallikrein-related peptidase 14	Klk14	100764613	0.00	0.00	0.00	0.00	0.00	0.00	0.00	0.00	0.00	0.00	0.00	0.93
kallikrein-related peptidase 15	Klk15	100753855	0.00	0.00	0.00	0.00	0.00	0.00	0.00	0.00	0.00	0.00	0.00	0.00
kallikrein-related peptidase 4	Klk4	100757055	0.00	0.00	0.00	0.00	0.00	0.00	0.00	0.00	0.00	0.00	0.00	0.00
kallikrein-related peptidase 5	Klk5	100756768	0.00	0.00	0.00	0.00	0.00	0.00	0.00	0.00	0.00	0.00	0.00	0.00
kallikrein-related peptidase 6	Klk6	100753252	0.00	0.00	0.00	0.00	0.00	0.00	0.00	0.00	0.00	0.00	0.00	0.00
kallikrein-related peptidase 7	Klk7	100753553	0.00	0.00	0.00	0.00	0.00	0.00	0.00	0.00	0.00	0.00	0.00	0.00
kallikrein-related peptidase 8	Klk8	100752953	0.00	0.00	0.00	0.00	0.00	0.00	0.00	0.00	0.00	0.00	0.00	0.00

kallikrein-related peptidase 9	Klk9	100752653	0.00	0.00	0.00	0.00	0.00	0.00	0.00	0.00	0.00	0.00	0.00	0.00
kallikrein B, plasma (Fletcher factor) 1	Klkb1	100767873	0.02	0.00	0.00	0.00	0.02	0.00	0.76	0.00	1.24	0.00	1.03	1.87
killin, p53-regulated DNA replication inhibitor	Klln	103162910	0.39	0.45	0.16	0.24	0.28	0.42	28.10	33.86	11.18	18.54	19.66	28.97
killer cell lectin-like receptor subfamily G, member 1	Klrg1	100761776	0.00	0.00	0.00	0.00	0.00	0.00	0.00	0.00	0.00	0.00	0.00	0.00
killer cell lectin-like receptor subfamily G, member 2	Klrg2	100771105	7.42	7.36	10.66	9.47	7.57	8.33	366.86	376.08	535.34	475.16	369.49	413.12
kynurenine 3-monooxygenase (kynurenine 3-hydroxylase)	Kmo	100772925	0.04	0.00	0.06	0.00	0.02	0.02	2.28	1.83	2.48	0.00	2.07	0.93
lysine (K)-specific methyltransferase 2A	Kmt2a	100760424	12.24	12.25	12.01	13.44	13.62	13.48	4801.05	4828.63	4777.07	5339.14	5367.44	5340.70
lysine (K)-specific methyltransferase 2B	Kmt2b	100750575	2.82	2.82	2.33	1.56	0.90	0.91	447.37	460.26	383.80	270.03	165.60	170.11
lysine (K)-specific methyltransferase 2C	Kmt2c	100767367	4.70	4.63	4.68	5.19	5.52	5.69	2407.74	2404.71	2435.73	2693.33	2841.04	2947.95
lysine (K)-specific methyltransferase 2D	Kmt2d	100765465	6.31	5.95	6.37	6.11	4.88	5.06	3791.62	3575.04	3844.26	3721.29	2956.96	3091.89
lysine (K)-specific methyltransferase 2E	Kmt2e	100756731	9.60	9.59	9.64	10.38	10.57	9.94	1940.62	1919.74	1958.77	2118.51	2124.83	2045.99
kinocilin	Kncn	100760160	0.09	0.00	0.00	0.00	0.00	0.00	0.76	0.00	0.00	1.16	1.03	0.00
kinase non-catalytic C-lobe domain (KIND) containing 1	Kndc1	100770095	0.00	0.00	0.00	0.00	0.00	0.00	0.00	0.00	0.00	0.00	0.00	0.00
kininogen 1	Kng1	100764285	0.00	0.00	0.00	0.00	0.00	0.03	0.00	0.00	0.00	0.00	0.00	0.93
lysine-rich nucleolar protein 1	Knop1	100751019	36.54	37.72	37.25	39.34	38.48	41.31	5165.63	5335.56	5298.74	5568.61	5410.91	5850.10
kinetochore-localized astrin/SPAG5 binding protein	Knstrn	100759555	44.68	48.29	44.66	46.33	47.61	49.40	1917.08	2074.38	1874.31	1936.56	2034.78	2072.16
karyopherin alpha 1 (importin alpha 5)	Kpna1	103158558	4.54	3.09	3.55	4.47	4.48	4.52	3.04	0.92	1.24	2.32	2.07	4.67
karyopherin alpha 2 (RAG cohort 1, importin alpha 1)	Kpna2	100768235	103.46	106.85	100.69	113.17	115.10	112.90	5557.55	5708.89	5422.95	6000.89	6050.53	5996.84
karyopherin alpha 3 (importin alpha 4)	Kpna3	100761838	36.75	37.01	31.50	34.88	35.80	35.91	4258.74	4318.96	3665.40	4051.58	4113.04	4149.00
karyopherin alpha 4 (importin alpha 3)	Kpna4	100757318	66.50	67.17	73.07	80.97	79.31	76.76	6614.07	6773.08	7349.43	8033.63	7841.06	7701.68
karyopherin alpha 5 (importin alpha 6)	Kpna5	100771427	2.20	2.05	1.03	1.23	1.35	1.30	320.53	299.22	152.78	184.27	198.72	193.48
karyopherin alpha 6 (importin alpha 7)	Kpna6	100754505	42.53	41.87	46.08	44.56	44.90	50.88	7163.97	7121.71	7876.07	7574.70	7583.34	8567.18
karyopherin alpha 7 (importin alpha 8)	Kpna7	100750897	0.00	0.00	0.00	0.03	0.00	0.00	0.00	0.00	0.00	2.32	0.00	0.00
karyopherin (importin) beta 1	Kpnb1	100762896	396.04	389.03	283.62	308.00	301.39	300.48	46419.24	46138.74	33787.25	36224.36	35313.73	35064.13
keratinocyte proline-rich protein	Kprp	100756266	0.00	0.00	0.00	0.00	0.00	0.00	0.00	0.00	0.00	0.00	0.00	0.00
kaptin (actin binding protein)	Kptn	100765362	3.75	3.35	1.74	1.40	0.79	0.93	165.58	145.49	74.53	70.69	38.29	47.67
Kirsten rat sarcoma viral oncogene homolog	Kras	100763178	20.80	21.26	22.01	23.63	26.63	25.11	1712.00	1769.67	1824.63	1974.80	2231.43	2170.30
KRAB-A domain containing 1	Krba1	100774574	0.00	0.00	0.00	0.00	0.00	0.00	0.00	0.00	0.00	0.00	0.00	0.00
lysine-rich coiled-coil 1	Krcc1	100769396	35.87	39.28	47.57	57.21	58.20	54.19	1444.64	1590.33	1935.17	2299.30	2351.49	2188.06
kringle containing transmembrane protein 2	Kremen 2	100771248	0.13	0.05	0.05	0.00	0.04	0.04	9.11	6.41	3.73	1.16	3.10	4.67
KRI1 homolog (S. cerevisiae)	Kri1	100771958	50.64	48.97	52.76	49.66	44.79	44.99	3309.31	3223.66	3490.26	3273.95	2983.87	2944.21
KRIT1, ankyrin repeat containing	Krit1	100758813	15.38	15.59	14.47	16.34	18.72	16.92	1653.52	1710.20	1620.92	1762.72	2018.22	1821.67

KRR1, small subunit (SSU) processome component, homolog (yeast)	Krr1	100760872	55.69	57.07	51.24	53.35	55.17	52.10	2572.56	2681.96	2382.32	2425.62	2504.67	2375.00
keratin 1	Krt1	100765316	0.00	0.00	0.00	0.00	0.00	0.00	0.00	0.00	0.00	0.00	0.00	0.00
keratin 13	Krt13	100758976	0.00	0.00	0.00	0.00	0.00	0.00	0.00	0.00	0.00	0.00	0.00	0.00
keratin 14	Krt14	100760043	0.00	0.00	0.00	0.00	0.00	0.00	0.00	0.00	0.00	0.00	0.00	0.00
keratin 15	Krt15	100759259	0.00	0.00	0.00	0.00	0.00	0.00	0.00	0.00	0.00	0.00	0.00	0.00
keratin 16	Krt16	100760336	0.00	0.00	0.00	0.00	0.00	0.00	0.00	0.00	0.00	0.00	0.00	0.00
keratin 17	Krt17	100760921	0.00	0.00	0.00	0.00	0.00	0.00	0.00	0.00	0.00	0.00	0.00	0.00
keratin 18	Krt18	100759201	0.00	0.00	0.00	0.00	0.00	0.00	0.00	0.00	0.00	0.00	0.00	0.00
keratin 19	Krt19	100759756	0.00	0.00	0.00	0.00	0.00	0.00	0.00	0.00	0.00	0.00	0.00	0.00
keratin 2	Krt2	100752858	0.00	0.00	0.00	0.00	0.00	0.00	0.00	0.00	0.00	0.00	0.00	0.00
keratin 222	Krt222	100762604	0.12	0.00	0.00	0.00	0.00	0.00	1.52	0.92	0.00	1.16	0.00	0.00
keratin 28	Krt28	100769462	0.00	0.00	0.00	0.00	0.00	0.00	0.00	0.00	0.00	0.00	0.00	0.00
keratin 32	Krt32	100758110	0.00	0.00	0.00	0.00	0.00	0.00	0.00	0.00	0.00	0.00	0.00	0.00
keratin 34	Krt34	100757817	0.00	0.00	0.00	0.00	0.00	0.00	0.00	0.00	0.00	0.00	0.00	0.00
keratin 35	Krt35	100758394	0.00	0.00	0.00	0.00	0.00	0.00	0.00	0.00	0.00	0.00	0.00	0.00
keratin 36	Krt36	100758686	0.00	0.00	0.00	0.00	0.00	0.00	0.00	0.00	0.00	0.00	0.00	0.00
keratin 4	Krt4	100774447	0.00	0.00	0.00	0.00	0.00	0.00	0.00	0.00	0.00	0.00	0.00	0.00
keratin 40	Krt40	100758909	0.00	0.00	0.00	0.00	0.00	0.00	0.00	0.00	0.00	0.00	0.00	0.00
keratin 5	Krt5	100754346	0.04	0.00	0.00	0.00	0.00	0.00	1.52	0.00	0.00	0.00	0.00	0.00
keratin 7	Krt7	100771838	0.22	0.09	0.27	0.15	0.13	0.15	11.39	5.49	9.94	6.95	8.28	6.54
keratin 71	Krt71	100754630	0.00	0.00	0.00	0.00	0.00	0.00	0.00	0.00	0.00	0.00	0.00	0.00
keratin 72	Krt72	100752072	0.00	0.00	0.00	0.00	0.00	0.00	0.00	0.00	0.00	0.00	0.00	0.00
keratin 73	Krt73	100752555	0.00	0.00	0.00	0.00	0.00	0.00	0.00	0.00	0.00	0.00	0.00	0.00
keratin 74	Krt74	100754923	0.00	0.00	0.00	0.00	0.00	0.00	0.00	0.00	0.00	0.00	0.00	0.00
keratin 77	Krt77	100765610	0.00	0.00	0.00	0.00	0.00	0.00	0.76	0.00	0.00	0.00	0.00	0.00
keratin 78	Krt78	100774159	0.49	0.40	0.17	0.28	0.09	0.22	44.05	38.43	13.66	24.34	9.31	16.82
keratin 79	Krt79	100773865	0.00	0.00	0.00	0.00	0.00	0.00	0.00	0.00	0.00	0.00	0.00	0.00
keratin 8	Krt8	100759488	0.00	0.00	0.03	0.03	0.00	0.00	0.76	0.00	1.24	1.16	0.00	0.00
keratin 80	Krt80	100771547	4.07	4.29	1.67	1.73	2.36	2.16	407.87	443.79	178.86	173.84	238.05	218.71
keratin 81	Krt81	100766653	0.00	0.00	0.00	0.00	0.00	0.00	0.00	0.00	0.00	0.00	0.00	0.00
keratin 82	Krt82	100765776	0.00	0.00	0.00	0.00	0.00	0.00	0.00	0.00	0.00	0.00	0.00	0.00
keratin 83	Krt83	100764732	0.00	0.00	0.00	0.00	0.00	0.00	0.00	0.00	0.00	0.00	0.00	0.00
keratin 84	Krt84	100765488	0.00	0.00	0.00	0.00	0.00	0.00	0.00	0.00	0.00	0.00	0.00	0.00
keratin 86	Krt86	100764435	0.00	0.00	0.00	0.00	0.00	0.00	0.00	0.00	0.00	0.00	0.00	0.00
keratin 9	Krt9	100762074	0.00	0.00	0.00	0.00	0.00	0.00	0.00	0.00	0.00	0.00	0.00	0.00
keratinocyte associated protein 2	Krtcap2	100757504	94.94	92.99	114.73	100.58	85.79	89.92	1212.98	1144.71	1337.73	997.83	834.20	897.28
keratinocyte associated protein 3	Krtcap3	100773669	0.30	0.24	0.37	0.32	0.21	0.22	28.10	31.11	26.08	15.07	16.56	19.63
keratinocyte differentiation-associated protein	Krtdap	103164079	0.00	0.00	0.00	0.00	0.00	0.00	0.00	0.00	0.00	0.00	0.00	0.00
kinase suppressor of ras 1	Ksr1	100769629	1.23	1.22	1.63	1.04	0.65	0.85	104.06	102.48	141.60	92.71	62.10	72.90
kinase suppressor of ras 2	Ksr2	100758412	0.00	0.01	0.00	0.00	0.00	0.00	0.00	0.92	0.00	0.00	0.00	0.00
KTI12 homolog, chromatin associated (S. cerevisiae)	Kti12	100753691	54.82	53.47	51.22	39.99	29.36	31.42	2305.20	2261.96	2224.58	1863.54	1482.10	1540.34

kinectin 1 (kinesin receptor)	Ktn1	100752812	43.75	39.74	35.47	44.87	44.25	40.58	6292.78	5878.18	5265.21	6476.04	6450.03	5784.67
kyphoscoliosis peptidase	Ky	100770249	0.00	0.00	0.00	0.00	0.00	0.00	0.00	0.00	0.00	0.00	0.00	0.00
kynureninase	Kynu	100771448	0.26	0.07	0.10	0.05	0.19	0.16	6.84	2.75	2.48	1.16	2.07	3.74
L1 cell adhesion molecule	L1cam	100769119	0.00	0.00	0.00	0.00	0.00	0.00	0.00	0.00	0.00	0.00	0.00	0.00
LINE-1 type transposase domain containing 1	L1td1	100758719	0.51	0.58	0.05	0.03	0.03	0.00	39.50	43.01	3.73	3.48	2.07	0.00
L-2-hydroxyglutarate dehydrogenase	L2hgdh	100768751	4.18	3.79	4.89	4.63	5.63	5.86	391.16	370.59	464.54	462.41	538.19	571.08
L-3-hydroxyproline dehydratase (trans-)	L3hypdh	100764709	2.27	2.51	2.79	3.54	3.87	3.67	79.75	86.93	105.58	110.10	126.27	118.70
l(3)mbt-like 1 (Drosophila)	L3mbtl1	100772152	0.03	0.05	0.02	0.04	0.05	0.05	3.80	7.32	6.21	6.95	5.17	7.48
l(3)mbt-like 2 (Drosophila)	L3mbtl2	100751536	11.35	12.41	12.25	12.14	14.29	15.56	615.99	639.61	635.95	628.13	648.94	684.18
l(3)mbt-like 3 (Drosophila)	L3mbtl3	100767229	8.19	8.33	6.96	6.06	6.86	5.34	558.26	565.49	475.72	409.10	456.43	363.59
l(3)mbt-like 4 (Drosophila)	L3mbtl4	100766135	0.00	0.03	0.00	0.00	0.00	0.00	0.00	0.92	0.00	0.00	0.00	0.00
MHC class I antigen Hm1-C5	LOC100689008	100689008	75.48	76.77	81.69	68.80	51.82	57.25	1524.40	1474.12	1546.40	1273.65	1033.95	1178.62
MHC class I antigen Hm1-C4	LOC100689009	100689009	21.05	19.53	23.23	18.05	12.04	14.94	429.90	382.48	520.43	506.45	319.81	430.88
MHC class I antigen Hm1-C2	LOC100689010	100689010	31.04	29.61	29.37	21.88	14.57	19.23	710.93	647.84	688.12	553.96	362.25	491.64
MHC class I antigen Hm1-C3	LOC100689011	100689011	32.65	30.38	40.25	37.86	32.47	32.89	1385.40	1349.67	1796.06	1733.74	1554.55	1510.43
U14 snoRNA	LOC100689269	100689269	0.00	0.00	0.00	0.00	0.00	0.00	3.04	0.92	3.73	0.00	2.07	0.93
U14 snoRNA	LOC100689270	100689270	0.00	0.00	0.00	0.00	0.00	0.00	6.84	3.66	4.97	10.43	14.49	9.35
beta major globin chain	LOC100689319	100689319	0.00	0.00	0.00	0.00	0.00	0.00	0.00	0.00	0.00	0.00	0.00	0.00
chromosome unknown open reading frame, human C7orf49	LOC100689364	100689364	10.92	10.28	9.38	7.17	8.53	9.37	281.03	306.54	327.91	285.09	301.18	274.79
caspase-9	LOC100689370	100689370	4.96	5.12	5.10	4.58	3.82	5.09	316.73	299.22	306.80	283.94	232.87	314.98
keratin-associated protein 14-like	LOC100750334	100750334	0.00	0.00	0.00	0.00	0.00	0.00	0.00	0.00	0.00	0.00	0.00	0.00
NADP-dependent malic enzyme, mitochondrial pseudogene	LOC100750335	100750335	0.00	0.00	0.00	0.00	0.00	0.00	0.00	0.00	0.00	0.00	0.00	0.00
60S ribosomal protein L12-like	LOC100750337	100750337	142.97	136.48	123.54	62.30	27.80	33.23	1733.27	1665.36	1449.52	677.97	309.46	365.46

speckle-type POZ protein-like	LOC100 750339	100750339	0.00	0.00	0.00	0.00	0.00	0.00	0.00	0.00	0.00	0.00	0.00	0.00
UDP-glucuronosyltransferase 2B1	LOC100 750340	100750340	0.00	0.00	0.00	0.00	0.00	0.00	0.00	0.00	0.00	0.00	0.00	0.00
sorbitol dehydrogenase pseudogene	LOC100 750341	100750341	0.03	0.10	0.00	0.04	0.08	0.00	0.76	2.75	0.00	1.16	2.07	0.00
heterogeneous nuclear ribonucleoprotein A3 pseudogene	LOC100 750345	100750345	0.04	0.02	0.00	0.00	0.03	0.00	0.76	0.00	0.00	0.00	0.00	0.00
alpha-enolase pseudogene	LOC100 750346	100750346	0.09	0.09	0.11	0.04	0.13	0.01	3.80	4.58	4.97	2.32	4.14	0.00
nuclear autoantigen Sp-100-like	LOC100 750349	100750349	6.19	6.72	5.71	5.77	6.45	6.59	281.03	305.62	250.90	251.49	263.92	292.55
chromosome unknown open reading frame, human C11orf94	LOC100 750351	100750351	1.05	0.80	0.70	0.37	0.20	0.00	90.39	63.14	95.64	68.38	58.99	42.06
ribosomal protein S6 kinase alpha-4	LOC100 750354	100750354	31.95	32.55	29.06	19.15	14.79	17.82	1185.64	1225.23	1179.98	886.57	785.55	940.28
interferon-induced guanylate-binding protein 1-like	LOC100 750355	100750355	0.03	0.02	0.00	0.00	0.00	0.00	0.76	0.00	0.00	0.00	0.00	0.00
eukaryotic translation initiation factor 3 subunit J-like	LOC100 750360	100750360	0.44	0.45	0.00	0.00	0.51	0.00	5.32	4.58	0.00	0.00	5.17	0.00
protein B-Myc	LOC100 750363	100750363	0.00	0.00	0.00	0.00	0.00	0.00	0.00	0.00	0.00	0.00	0.00	0.00
solute carrier family 7 member 13-like	LOC100 750364	100750364	0.00	0.00	0.00	0.00	0.00	0.00	0.00	0.00	0.00	0.00	0.00	0.00
zinc finger protein 82 pseudogene	LOC100 750366	100750366	0.17	0.13	0.18	0.15	0.13	0.21	9.87	6.41	4.97	6.95	6.21	8.41
oxytocin-neurophysin 1	LOC100 750370	100750370	0.00	0.00	0.00	0.00	0.00	0.00	0.00	0.00	0.00	0.00	0.00	0.00
oxytocin-neurophysin 1-like	LOC100 750371	100750371	0.00	0.00	0.00	0.00	0.00	0.00	0.00	0.00	0.00	0.00	0.00	0.00
zinc finger protein 883-like	LOC100 750376	100750376	4.34	4.87	3.88	4.74	5.63	5.18	458.00	518.82	404.92	496.02	579.59	519.68
olfactory receptor 10Z1	LOC100 750377	100750377	0.00	0.00	0.00	0.00	0.00	0.00	0.00	0.00	0.00	0.00	0.00	0.00
transcription elongation factor B polypeptide 1 pseudogene	LOC100 750378	100750378	0.22	0.27	0.00	0.00	0.32	0.29	25.82	24.71	17.39	12.75	20.70	19.63

interferon-inducible protein AIM2	LOC100750381	100750381	29.97	30.08	29.88	32.83	38.80	32.76	2783.71	2813.73	2824.51	3064.18	3621.42	3083.48
ubiquitin-60S ribosomal protein L40-like	LOC100750383	100750383	0.00	0.00	0.00	0.00	0.00	0.00	0.00	0.00	0.00	0.00	0.00	0.00
putative olfactory receptor GPCRLTM7	LOC100750400	100750400	0.00	0.00	0.00	0.00	0.00	0.00	0.00	0.00	0.00	0.00	0.00	0.00
zinc finger protein OZF-like	LOC100750406	100750406	2.86	2.77	3.63	3.36	5.01	4.16	268.12	262.61	347.78	327.97	470.92	400.04
actin-related protein 3B	LOC100750418	100750418	0.00	0.00	0.00	0.00	0.00	0.00	0.00	0.92	0.00	0.00	0.00	0.00
selenoprotein K pseudogene	LOC100750421	100750421	0.00	0.00	0.00	0.00	0.00	0.00	0.00	0.92	2.48	0.00	0.00	2.80
uncharacterized protein CXorf21-like	LOC100750423	100750423	0.00	0.00	0.00	0.00	0.00	0.00	0.00	0.00	0.00	0.00	0.00	0.00
zinc finger protein 260-like	LOC100750424	100750424	2.40	2.61	2.53	2.97	2.87	3.21	164.82	179.35	170.17	198.18	195.61	219.65
eukaryotic translation initiation factor 1 pseudogene	LOC100750428	100750428	29.83	31.26	23.55	29.28	24.87	25.25	314.45	358.69	258.35	268.87	265.99	232.73
60S acidic ribosomal protein P0 pseudogene	LOC100750433	100750433	0.00	0.00	0.00	0.00	0.00	0.00	0.00	0.00	0.00	0.00	0.00	0.00
protocadherin beta-14-like	LOC100750434	100750434	0.10	0.12	0.15	0.02	0.06	0.07	3.04	1.83	7.45	1.16	4.14	5.61
leukocyte immunoglobulin-like receptor subfamily A member 6	LOC100750435	100750435	0.00	0.00	0.00	0.00	0.00	0.00	0.00	0.00	0.00	0.00	0.00	0.00
uncharacterized LOC100750436	LOC100750436	100750436	0.00	0.00	0.00	0.00	0.00	0.00	0.00	0.00	0.00	0.00	0.00	0.00
60S ribosomal protein L36a-like	LOC100750438	100750438	0.19	0.00	0.00	0.33	0.28	0.00	0.76	0.00	0.00	1.16	1.03	0.00
olfactory receptor 2C1-like	LOC100750442	100750442	0.00	0.00	0.00	0.00	0.00	0.00	0.00	0.00	0.00	0.00	0.00	0.00
sepiapterin reductase-like	LOC100750443	100750443	0.00	0.00	0.00	0.00	0.00	0.00	0.76	0.00	0.00	0.00	0.00	0.00
dnaJ homolog subfamily C member 8 pseudogene	LOC100750452	100750452	0.00	0.00	0.00	0.00	0.00	0.00	0.00	0.00	0.00	0.00	0.00	0.00
secreted seminal-vesicle Ly-6 protein 1-like	LOC100750453	100750453	0.00	0.00	0.00	0.00	0.00	0.00	0.00	0.00	0.00	0.00	0.00	0.00

uncharacterized LOC100750455	LOC100 750455	100750455	0.00	0.00	0.00	0.00	0.00	0.00	0.00	0.00	0.00	0.00	0.00	0.00
glyceraldehyde-3-phosphate dehydrogenase-like	LOC100 750456	100750456	0.03	0.08	0.00	0.05	0.09	0.01	0.76	1.83	0.00	1.16	1.03	0.00
vomeronasal type-2 receptor 116-like	LOC100 750458	100750458	0.00	0.00	0.00	0.00	0.00	0.00	0.00	0.00	0.00	0.00	0.00	0.00
60S ribosomal protein L17 pseudogene	LOC100 750459	100750459	0.63	0.62	0.18	0.24	0.17	0.00	7.60	15.56	7.45	3.48	3.10	3.74
serine/arginine-rich splicing factor 3 pseudogene	LOC100 750467	100750467	0.00	0.00	0.00	0.00	0.00	0.00	0.00	1.83	0.00	2.32	2.07	0.93
PRAME family member 9/15-like	LOC100 750468	100750468	0.00	0.04	0.00	0.00	0.00	0.00	0.00	0.92	0.00	0.00	0.00	0.00
ruvB-like 2 pseudogene	LOC100 750474	100750474	0.20	0.13	0.32	0.10	0.09	0.13	6.84	4.58	11.18	3.48	3.10	4.67
cytochrome c, somatic pseudogene	LOC100 750475	100750475	0.19	0.00	0.00	0.00	0.00	0.00	8.35	0.00	1.24	1.16	0.00	2.80
protein-S-isoprenylcysteine O-methyltransferase-like	LOC100 750476	100750476	0.00	0.00	0.00	0.00	0.00	0.00	0.00	0.00	0.00	0.00	0.00	0.00
60S ribosomal protein L6 pseudogene	LOC100 750478	100750478	0.40	0.18	0.42	0.17	0.15	0.00	7.60	4.58	7.45	3.48	3.10	0.00
chromosome unknown open reading frame, human C2orf69	LOC100 750484	100750484	4.83	4.79	5.06	4.81	6.05	4.82	374.45	378.82	398.71	383.60	477.13	383.21
olfactory receptor 4D1	LOC100 750490	100750490	0.00	0.00	0.00	0.00	0.00	0.00	0.00	0.00	0.00	0.00	0.00	0.00
14-3-3 protein theta pseudogene	LOC100 750496	100750496	0.00	0.00	0.00	0.00	0.00	0.00	0.00	0.00	0.00	0.00	0.00	0.00
olfactory receptor 5M11-like	LOC100 750497	100750497	0.00	0.00	0.00	0.00	0.00	0.00	0.00	0.00	0.00	0.00	0.00	0.00
potassium voltage-gated channel subfamily D member 2	LOC100 750498	100750498	0.23	0.29	0.07	0.21	0.10	0.15	11.39	16.47	4.97	13.91	6.21	7.48
zinc finger protein 883	LOC100 750500	100750500	4.12	3.83	4.09	4.96	5.50	5.74	331.92	316.60	330.40	404.46	454.36	474.81
olfactory guanylyl cyclase GC-D	LOC100 750501	100750501	0.00	0.00	0.00	0.00	0.00	0.00	0.76	0.00	0.00	2.32	0.00	0.00
olfactory receptor 2H1-like	LOC100 750504	100750504	0.00	0.01	0.00	0.00	0.11	0.00	0.00	0.00	0.00	0.00	2.07	0.00

zinc finger protein 451	LOC100 750506	100750506	5.73	5.54	5.64	6.12	6.46	6.39	578.01	565.49	562.67	629.29	649.97	645.86
cytochrome P450 2D4	LOC100 750508	100750508	4.49	4.42	5.88	5.11	4.79	5.78	319.01	301.96	416.10	365.06	357.07	422.47
killer cell lectin-like receptor 2	LOC100 750511	100750511	0.00	0.00	0.00	0.00	0.00	0.00	0.00	0.00	0.00	0.00	0.00	0.00
H-2 class II histocompatibility antigen, E-S beta chain-like	LOC100 750512	100750512	0.00	0.00	0.00	0.00	0.00	0.00	0.00	0.00	0.00	0.00	0.00	0.00
uncharacterized LOC100750514	LOC100 750514	100750514	0.00	0.00	0.00	0.00	0.00	0.00	0.00	0.00	0.00	0.00	0.00	0.00
leukocyte immunoglobulin-like receptor subfamily A member 6	LOC100 750515	100750515	0.00	0.00	0.00	0.00	0.00	0.00	0.00	0.00	0.00	0.00	0.00	0.00
olfactory receptor 8B3-like	LOC100 750516	100750516	0.00	0.00	0.00	0.00	0.00	0.00	0.00	0.00	0.00	0.00	0.00	0.00
vomeronasal type-1 receptor 2-like	LOC100 750527	100750527	0.00	0.00	0.00	0.00	0.00	0.00	0.00	0.00	0.00	0.00	0.00	0.00
outer dense fiber protein 1-like	LOC100 750528	100750528	0.04	0.00	0.00	0.00	0.00	0.00	0.76	0.00	0.00	0.00	0.00	0.00
olfactory receptor 5B3-like	LOC100 750529	100750529	0.00	0.00	0.00	0.00	0.00	0.00	0.00	0.00	0.00	0.00	0.00	0.00
GRB2-related adapter protein	LOC100 750533	100750533	1.09	1.19	0.40	0.22	0.44	0.35	33.42	36.60	14.91	9.27	12.42	10.28
zinc finger protein 431-like	LOC100 750536	100750536	0.00	0.00	0.00	0.00	0.00	0.00	0.00	0.00	0.00	0.00	0.00	0.00
NADH dehydrogenase [ubiquinone] 1 beta subcomplex subunit 8, mitochondrial pseudogene	LOC100 750539	100750539	0.00	0.00	0.00	0.00	0.00	0.00	1.52	3.66	0.00	0.00	2.07	0.00
disintegrin and metalloproteinase domain-containing protein 25-like	LOC100 750541	100750541	0.00	0.00	0.00	0.00	0.00	0.00	0.00	0.00	0.00	0.00	0.00	0.00
SRA stem-loop-interacting RNA-binding protein, mitochondrial pseudogene	LOC100 750546	100750546	1.76	1.32	0.85	2.11	1.92	1.44	4.56	2.75	1.24	6.95	6.21	5.61
lysozyme C-1	LOC100 750548	100750548	0.00	0.19	0.07	0.00	0.06	0.05	0.76	3.66	1.24	2.32	2.07	1.87
UDP-glucuronosyltransferase 1-8 pseudogene	LOC100 750549	100750549	0.04	0.00	0.00	0.02	0.01	0.00	0.00	0.00	0.00	0.00	0.00	0.00
histone H2A type 1-F	LOC100 750550	100750550	0.15	0.09	0.15	0.05	0.08	0.08	4.56	2.75	3.73	1.16	2.07	3.74

Ig heavy chain V-III region VH26-like	LOC100750551	100750551	0.00	0.00	0.00	0.00	0.00	0.15						
olfactory receptor 12-like	LOC100750553	100750553	0.00	0.00	0.00	0.00	0.00	0.00	0.00	0.00	0.00	0.00	0.00	0.00
60S ribosomal protein L37a-like	LOC100750557	100750557	0.42	1.03	0.90	0.00	0.30	0.57	1.52	3.66	2.48	0.00	1.03	0.93
histone H4	LOC100750560	100750560	0.15	0.00	0.00	0.00	0.00	0.18	3.04	0.92	0.00	0.00	1.03	5.61
uncharacterized LOC100750562	LOC100750562	100750562	0.00	0.00	0.00	0.00	0.00	0.00	0.00	0.92	0.00	0.00	0.00	0.00
olfactory receptor 2G3-like	LOC100750564	100750564	0.00	0.00	0.00	0.00	0.00	0.00	0.00	0.00	0.00	0.00	0.00	0.00
60S ribosomal protein L34 pseudogene	LOC100750567	100750567	0.00	0.00	0.00	0.00	0.00	0.00	0.00	0.00	0.00	0.00	0.00	0.00
60S ribosomal protein L10-like	LOC100750572	100750572	11.87	11.67	11.04	8.90	9.61	10.58	82.79	92.42	89.43	64.90	68.31	82.25
olfactory receptor 5W2-like	LOC100750574	100750574	0.00	0.00	0.00	0.00	0.00	0.00	0.00	0.00	0.00	0.00	0.00	0.00
chromosome unknown open reading frame, human C7orf25	LOC100750581	100750581	11.72	10.91	11.17	13.42	13.35	13.10	649.41	609.41	621.04	744.03	722.42	721.57
histone-lysine N-methyltransferase EZH2-like	LOC100750584	100750584	0.00	0.00	0.00	0.02	0.02	0.00	1.52	0.00	0.00	1.16	1.03	0.93
N-lysine methyltransferase SETD8 pseudogene	LOC100750585	100750585	0.00	0.00	0.00	0.00	0.00	0.00	0.00	0.00	0.00	0.00	0.00	0.00
anionic trypsin-like	LOC100750587	100750587	0.00	0.00	0.00	0.00	0.00	0.00	0.00	0.00	0.00	0.00	0.00	0.00
uncharacterized LOC100750590	LOC100750590	100750590	0.00	0.00	0.00	0.00	0.00	0.00	0.00	0.00	0.00	0.00	0.00	0.00
60S ribosomal protein L37 pseudogene	LOC100750592	100750592	0.98	1.05	0.73	0.86	0.75	0.69	17.47	19.22	6.21	5.79	11.38	4.67
acyl-CoA desaturase 1	LOC100750593	100750593	50.91	49.62	83.85	88.05	96.15	98.78	7230.05	7038.44	11972.48	12452.59	13462.07	14002.29
olfactory receptor 5AC1-like	LOC100750595	100750595	0.00	0.00	0.00	0.00	0.00	0.00	0.00	0.00	0.00	0.00	0.00	0.00
killer cell lectin-like receptor 2	LOC100750597	100750597	0.00	0.00	0.00	0.00	0.00	0.00	0.00	0.00	0.00	0.00	0.00	0.00

serum amyloid A-5 protein	LOC100 750598	100750598	0.00	0.00	0.00	0.00	0.00	0.00	0.00	0.00	0.00	0.00	0.00	0.00
serine protease 30	LOC100 750599	100750599	0.00	0.00	0.00	0.00	0.00	0.00	0.00	0.00	0.00	0.00	0.00	0.00
uncharacterized LOC100750600	LOC100 750600	100750600	0.67	0.47	1.04	0.31	0.73	0.91	20.51	16.47	33.54	18.54	39.33	31.78
prolactin	LOC100 750601	100750601	0.00	0.00	0.00	0.00	0.00	0.00	0.00	0.00	0.00	0.00	0.00	0.00
vomeronasal type-2 receptor 26-like	LOC100 750604	100750604	0.00	0.00	0.00	0.00	0.00	0.00	0.00	0.00	0.00	0.00	0.00	0.00
alkaline phosphatase, placental-like	LOC100 750606	100750606	0.00	0.00	0.00	0.00	0.00	0.00	0.76	0.00	0.00	0.00	0.00	0.00
transmembrane epididymal protein 1	LOC100 750611	100750611	0.00	0.00	0.00	0.00	0.00	0.00	0.00	0.00	0.00	0.00	0.00	0.00
voltage-dependent anion-selective channel protein 1 pseudogene	LOC100 750613	100750613	27.17	25.43	23.12	22.39	24.71	29.78	724.60	664.31	640.92	600.32	662.39	831.86
ATP-dependent RNA helicase DDX18 pseudogene	LOC100 750617	100750617	1.42	1.36	1.21	1.25	1.24	1.17	31.90	33.86	19.87	25.50	28.98	25.24
chromosome unknown open reading frame, human C18orf63	LOC100 750618	100750618	0.00	0.02	0.00	0.00	0.00	0.00	0.00	0.92	0.00	0.00	0.00	0.00
keratin-associated protein 19-3-like	LOC100 750623	100750623	0.00	0.00	0.00	0.00	0.00	0.00	0.00	0.00	0.00	0.00	0.00	0.00
partner of Y14 and mago	LOC100 750626	100750626	0.06	0.00	0.00	0.00	0.00	0.00	9.87	4.58	2.48	2.32	0.00	0.00
schlafen family member 13-like	LOC100 750627	100750627	0.00	0.00	0.00	0.00	0.00	0.00	0.76	0.00	0.00	0.00	0.00	0.00
speckle-type POZ protein pseudogene	LOC100 750628	100750628	0.00	0.00	0.00	0.00	0.00	0.00	0.00	0.00	1.24	0.00	0.00	0.00
sperm motility kinase Z-like	LOC100 750629	100750629	0.00	0.00	0.00	0.00	0.00	0.00	0.00	0.00	0.00	0.00	0.00	0.00
protein SET-like	LOC100 750634	100750634	2.10	2.87	2.78	1.29	2.36	0.97	42.53	63.14	59.62	25.50	49.68	16.82
interferon alpha-12	LOC100 750635	100750635	0.00	0.00	0.00	0.00	0.00	0.00	0.00	0.00	0.00	0.00	0.00	0.00
nuclear autoantigen Sp-100-like	LOC100 750637	100750637	0.35	0.22	0.48	0.27	0.97	0.82	27.34	17.39	42.23	27.81	71.41	72.90

putative protein FAM47D	LOC100 750638	100750638	0.00	0.00	0.00	0.00	0.00	0.00	0.00	0.00	0.00	0.00	0.00	0.00
glutathione S-transferase Mu 7	LOC100 750644	100750644	39.75	42.57	34.51	36.29	35.39	37.84	1427.17	1549.15	1242.09	1288.72	1267.86	1340.32
reticulon-1	LOC100 750648	100750648	0.00	0.00	0.00	0.00	0.00	0.00	0.00	0.00	0.00	0.00	0.00	0.00
60S ribosomal protein L7 pseudogene	LOC100 750652	100750652	6.25	7.57	5.08	4.00	5.34	4.05	47.85	64.97	37.26	24.34	34.15	20.56
histone H2A type 2-B	LOC100 750653	100750653	0.31	0.17	0.24	0.02	0.00	0.00	1.52	2.75	2.48	2.32	4.14	1.87
olfactory receptor 2Y1-like	LOC100 750654	100750654	0.00	0.00	0.00	0.00	0.00	0.00	0.00	0.00	0.00	0.00	0.00	0.00
60S acidic ribosomal protein P0 pseudogene	LOC100 750656	100750656	0.00	0.00	0.00	0.00	0.00	0.00	0.00	0.00	0.00	0.00	0.00	0.00
interferon-induced protein with tetratricopeptide repeats 3-like	LOC100 750657	100750657	0.00	0.00	0.00	0.00	0.01	0.02	0.00	0.00	0.00	0.00	0.00	0.00
chromosome unknown open reading frame, human C20orf141	LOC100 750658	100750658	0.00	0.13	0.00	0.00	0.00	0.00	0.00	0.92	0.00	1.16	0.00	0.00
serpin B6 pseudogene	LOC100 750660	100750660	0.00	0.00	0.00	0.00	0.00	0.00	0.00	0.00	0.00	0.00	0.00	0.00
pyruvate kinase PKM pseudogene	LOC100 750663	100750663	0.00	0.00	0.00	0.00	0.00	0.00	3.80	0.00	1.24	0.00	0.00	0.93
olfactory receptor 7E24-like	LOC100 750664	100750664	0.00	0.00	0.00	0.00	0.00	0.00	0.00	0.00	0.00	0.00	0.00	0.00
40S ribosomal protein S19 pseudogene	LOC100 750666	100750666	0.00	0.00	0.00	0.00	0.00	0.00	0.00	0.00	0.00	0.00	0.00	0.00
interferon-activable protein 203-like	LOC100 750669	100750669	0.35	0.15	1.85	2.37	0.62	0.86	14.43	8.24	88.19	121.69	24.84	31.78
transmembrane protein 230 pseudogene	LOC100 750671	100750671	0.00	0.00	0.00	0.00	0.06	0.00	6.08	5.49	8.69	4.64	1.03	3.74
PRELI domain-containing protein 1, mitochondrial-like	LOC100 750687	100750687	0.00	0.00	0.00	0.00	0.00	0.00	5.32	5.49	2.48	2.32	5.17	2.80
elongation factor 1-alpha 1-like	LOC100 750688	100750688	0.02	0.00	0.00	0.00	0.00	0.00	0.00	0.00	0.00	0.00	0.00	0.00
voltage-dependent anion-selective channel protein 1 pseudogene	LOC100 750690	100750690	0.00	0.09	0.13	0.00	0.11	0.00	0.76	3.66	2.48	2.32	2.07	0.00

glutathione S-transferase kappa 1-like	LOC100 750691	100750691	3.65	3.38	4.53	4.75	5.81	4.19	88.87	86.01	108.06	115.89	137.65	102.81
regenerating islet-derived protein 3-alpha-like	LOC100 750692	100750692	0.00	0.00	0.00	0.00	0.00	0.00	0.00	0.00	0.00	0.00	0.00	0.00
olfactory receptor 8	LOC100 750693	100750693	0.00	0.00	0.00	0.00	0.00	0.00	0.00	0.00	0.00	0.00	0.00	0.00
T-cell ecto-ADP-ribosyltransferase 1-like	LOC100 750708	100750708	0.00	0.00	0.00	0.00	0.00	0.00	0.00	0.00	0.00	0.00	0.00	0.00
glutathione S-transferase A2-like	LOC100 750710	100750710	0.00	0.00	0.00	0.00	0.00	0.00	0.00	0.00	0.00	0.00	0.00	0.00
ubiquitin-conjugating enzyme E2 B-like	LOC100 750711	100750711	7.15	8.08	6.99	6.46	7.50	6.24	6.08	8.24	14.91	3.48	9.31	8.41
NEDD8 pseudogene	LOC100 750714	100750714	1.09	0.53	2.61	0.00	1.26	0.59	3.80	1.83	8.69	0.00	4.14	1.87
zinc finger protein 11	LOC100 750719	100750719	1.71	1.80	1.87	2.29	2.68	2.40	512.69	514.25	561.42	664.06	756.57	715.96
protocadherin beta-14-like	LOC100 750720	100750720	0.04	0.00	0.00	0.01	0.00	0.03	1.52	0.00	0.00	0.00	1.03	0.93
uncharacterized LOC100750722	LOC100 750722	100750722	0.00	0.00	0.00	0.00	0.00	0.00	0.00	0.00	0.00	0.00	0.00	0.00
nuclear body protein SP140-like	LOC100 750728	100750728	6.80	9.00	7.98	6.77	7.05	6.03	223.30	286.41	265.81	265.39	244.26	246.75
sperm motility kinase X-like	LOC100 750729	100750729	0.02	0.07	0.10	0.08	0.02	0.08	3.80	5.49	1.24	2.32	3.10	6.54
40S ribosomal protein S25	LOC100 750731	100750731	6.86	9.57	7.77	3.11	7.00	4.16	57.72	81.44	68.31	25.50	56.92	27.11
olfactory receptor 491	LOC100 750739	100750739	0.00	0.03	0.00	0.00	0.00	0.00	0.00	0.92	0.00	0.00	0.00	0.00
glyceraldehyde-3-phosphate dehydrogenase-like	LOC100 750741	100750741	0.58	0.65	0.46	0.37	0.31	0.42	14.43	12.81	9.94	9.27	7.24	8.41
pre-mRNA-splicing factor SPF27 pseudogene	LOC100 750742	100750742	0.08	0.10	0.06	0.00	0.00	0.15	1.52	1.83	1.24	0.00	0.00	2.80
cytochrome P450 4V2	LOC100 750743	100750743	5.46	6.76	5.98	5.54	6.81	7.47	198.24	235.16	193.77	181.95	275.31	233.67
Ig lambda chain V-VI region SUT-like	LOC100 750744	100750744	0.00	0.00	0.00	0.00	0.00	0.00						

olfactory receptor 1444	LOC100750745	100750745	0.00	0.00	0.00	0.00	0.00	0.00	0.00	0.00	0.00	0.00	0.00	0.00
60S ribosomal protein L18a pseudogene	LOC100750746	100750746	0.10	0.17	0.11	0.32	0.00	0.00	0.00	1.83	0.00	1.16	0.00	0.00
40S ribosomal protein S15a pseudogene	LOC100750754	100750754	0.00	0.31	0.00	0.14	0.00	0.00	0.00	1.83	0.00	0.00	0.00	0.00
chromatin target of PRMT1 protein pseudogene	LOC100750760	100750760	0.05	0.06	0.00	0.00	0.00	0.00	0.76	0.92	0.00	1.16	0.00	0.00
zinc finger protein 431-like	LOC100750763	100750763	0.00	0.00	0.00	0.00	0.00	0.00	0.00	0.00	0.00	0.00	0.00	0.00
chromosome unknown open reading frame, human C3orf18	LOC100750764	100750764	2.61	2.98	2.22	1.74	1.21	1.60	96.46	111.63	85.70	68.38	51.75	61.69
serine protease 29-like	LOC100750768	100750768	0.00	0.00	0.00	0.00	0.00	0.00	0.00	0.00	0.00	0.00	0.00	0.00
spermine synthase pseudogene	LOC100750770	100750770	0.00	0.00	0.00	0.00	0.00	0.00	0.76	0.00	0.00	0.00	0.00	0.00
olfactory receptor 4D2	LOC100750777	100750777	0.00	0.00	0.00	0.00	0.00	0.00	0.00	0.00	0.00	0.00	0.00	0.00
transmembrane protein ENSP00000340100 homolog	LOC100750780	100750780	0.00	0.01	0.01	0.01	0.01	0.00	224.06	225.10	212.40	113.57	36.22	42.06
olfactory receptor 5M11-like	LOC100750785	100750785	0.00	0.00	0.00	0.00	0.00	0.00	0.00	0.00	0.00	0.00	0.00	0.00
golgin subfamily A member 7 pseudogene	LOC100750787	100750787	0.14	0.46	0.16	0.15	0.66	0.12	0.76	2.75	1.24	1.16	5.17	0.93
olfactory receptor 10K2	LOC100750789	100750789	0.00	0.00	0.00	0.00	0.00	0.00	0.00	0.00	0.00	0.00	0.00	0.00
charged multivesicular body protein 6 pseudogene	LOC100750796	100750796	0.68	0.98	1.00	0.85	0.47	0.34	44.81	48.50	34.78	26.66	19.66	14.95
killer cell lectin-like receptor 2	LOC100750803	100750803	0.00	0.00	0.00	0.00	0.00	0.00	0.00	0.00	0.00	0.00	0.00	0.00
39S ribosomal protein L51, mitochondrial pseudogene	LOC100750805	100750805	0.00	0.00	0.00	0.00	0.00	0.00	0.00	0.00	0.00	0.00	0.00	0.00
leukocyte immunoglobulin-like receptor subfamily A member 6	LOC100750807	100750807	0.00	0.00	0.00	0.00	0.00	0.00	0.00	0.92	0.00	0.00	0.00	0.00
60S ribosomal protein L37 pseudogene	LOC100750808	100750808	175.27	168.29	176.65	186.88	184.42	179.77	309.13	303.79	276.99	259.60	271.17	252.36

60S ribosomal protein L27 pseudogene	LOC100750813	100750813	0.00	0.00	0.00	0.00	0.00	0.00	0.00	0.00	1.24	0.00	0.00	0.00
outer dense fiber protein 1-like	LOC100750820	100750820	0.00	0.00	0.00	0.00	0.00	0.00	1.52	0.00	1.24	1.16	1.03	1.87
aphrodisin-like	LOC100750821	100750821	0.00	0.00	0.00	0.00	0.00	0.00	0.00	0.00	0.00	0.00	0.00	0.00
cytochrome b-c1 complex subunit 2, mitochondrial	LOC100750822	100750822	67.40	70.42	71.61	76.75	83.27	86.46	3228.04	3378.30	3479.09	3679.57	3971.24	4045.25
60S ribosomal protein L32 pseudogene	LOC100750823	100750823	4.03	2.57	2.66	1.72	2.09	0.95	28.86	18.30	18.63	11.59	15.52	6.54
monocarboxylate transporter 1-like	LOC100750832	100750832	0.00	0.00	0.00	0.00	0.00	0.00	0.00	0.00	0.00	0.00	0.00	0.00
disintegrin and metalloproteinase domain-containing protein 24-like	LOC100750833	100750833	0.00	0.00	0.00	0.00	0.00	0.00	0.00	0.00	0.00	0.00	0.00	0.00
PEST proteolytic signal-containing nuclear protein pseudogene	LOC100750835	100750835	0.00	0.00	0.00	0.00	0.00	0.00	0.00	0.00	0.00	0.00	0.00	0.00
40S ribosomal protein S27-like pseudogene	LOC100750836	100750836	51.52	61.75	57.97	51.52	49.23	54.60	426.86	486.80	403.68	347.68	289.80	336.48
testis-specific serine kinase substrate-like	LOC100750839	100750839	0.00	0.04	0.00	0.00	0.00	0.00	0.00	0.92	0.00	0.00	0.00	0.93
upstream-binding factor 1-like protein 1	LOC100750841	100750841	0.00	0.00	0.00	0.00	0.00	0.00	0.00	0.92	0.00	0.00	0.00	0.00
UDP-glucuronosyltransferase 1-8-like	LOC100750842	100750842	0.07	0.20	0.22	0.21	0.38	0.21	0.76	4.58	6.21	4.64	7.24	4.67
histone H2B type 1	LOC100750843	100750843	6.77	6.15	7.09	3.33	1.98	2.95	0.00	0.00	0.00	0.00	0.00	0.00
3-hydroxybutyrate dehydrogenase type 2	LOC100750844	100750844	25.23	25.94	19.93	31.99	27.91	23.04	135.96	136.34	88.19	141.39	141.79	117.77
olfactory receptor 52Z1-like	LOC100750846	100750846	0.00	0.00	0.00	0.00	0.00	0.00	0.00	0.00	0.00	0.00	0.00	0.00
olfactory receptor 12	LOC100750847	100750847	0.00	0.00	0.00	0.00	0.00	0.00	2.28	0.92	3.73	2.32	1.03	1.87
histone H2A type 1	LOC100750854	100750854	0.06	0.11	0.03	0.00	0.03	0.03	2.28	2.75	1.24	0.00	1.03	0.93
chromosome unknown open reading frame, human C20orf96	LOC100750855	100750855	0.15	0.19	0.05	0.05	0.21	0.08	6.84	4.58	1.24	2.32	8.28	3.74

thioredoxin domain-containing protein 17 pseudogene	LOC100750857	100750857	0.00	0.00	0.00	0.00	0.00	0.00	0.00	0.00	0.00	0.00	0.00	0.00
olfactory receptor 2A12-like	LOC100750858	100750858	0.00	0.00	0.00	0.00	0.00	0.00	0.00	0.00	0.00	0.00	0.00	0.00
myosin light polypeptide 6-like	LOC100750860	100750860	0.00	0.00	0.00	0.00	0.00	0.00	0.00	0.00	0.00	0.00	0.00	0.00
uncharacterized LOC100750862	LOC100750862	100750862	0.00	0.00	0.00	0.00	0.00	0.00	0.00	0.00	0.00	0.00	0.00	0.00
olfactory receptor 10AG1-like	LOC100750869	100750869	0.00	0.00	0.00	0.00	0.00	0.00	0.00	0.00	0.00	0.00	0.00	0.00
40S ribosomal protein S20 pseudogene	LOC100750878	100750878	0.00	0.00	0.00	0.00	0.00	0.00	0.76	0.00	0.00	0.00	0.00	0.00
anionic trypsin-2-like	LOC100750882	100750882	0.00	0.00	0.00	0.00	0.00	0.00	0.00	0.00	0.00	0.00	0.00	0.00
ATP-binding cassette sub-family A member 12	LOC100750884	100750884	0.09	0.08	0.01	0.01	0.00	0.00	15.95	16.47	1.24	1.16	0.00	0.00
uncharacterized LOC100750885	LOC100750885	100750885	0.00	0.00	0.00	0.00	0.00	0.00	0.00	0.00	0.00	0.00	0.00	0.00
acyl-CoA desaturase 2	LOC100750890	100750890	12.11	13.00	27.78	26.61	34.86	36.00	1611.74	1733.07	3804.51	3654.07	4782.67	4977.12
olfactory receptor 5AC1-like	LOC100750892	100750892	0.00	0.00	0.00	0.00	0.00	0.00	0.00	0.00	0.00	0.00	0.00	0.00
prolactin-3B1-like	LOC100750898	100750898	0.00	0.00	0.00	0.00	0.00	0.00	0.00	0.00	0.00	0.00	0.00	0.00
SAP30-binding protein pseudogene	LOC100750900	100750900	0.06	0.04	0.05	0.05	0.00	0.04	2.28	1.83	1.24	1.16	0.00	0.93
alkaline phosphatase, placental-like	LOC100750903	100750903	0.00	0.02	0.00	0.00	0.00	0.00	0.00	0.92	0.00	0.00	0.00	0.00
trace amine-associated receptor 3	LOC100750905	100750905	0.00	0.00	0.00	0.00	0.00	0.00	0.00	0.00	0.00	0.00	0.00	0.00
60S ribosomal protein L23a pseudogene	LOC100750906	100750906	6.86	8.34	6.01	4.42	6.53	4.16	2.28	0.92	2.48	2.32	4.14	0.93
60S ribosomal protein L19 pseudogene	LOC100750907	100750907	6.67	7.12	3.80	2.82	3.39	3.00	89.63	93.33	47.20	34.77	47.61	39.26
zinc finger protein 42 homolog	LOC100750909	100750909	0.00	0.00	0.00	0.00	0.00	0.00	0.00	0.00	0.00	0.00	0.00	0.00

transcription elongation factor B polypeptide 1 pseudogene	LOC100 750911	100750911	0.00	0.00	0.00	0.00	0.00	0.00	0.00	0.00	0.00	0.00	0.00	0.00
fibrous sheath-interacting protein 2-like	LOC100 750913	100750913	0.00	0.00	0.00	0.00	0.00	0.00	0.00	0.00	0.00	0.00	0.00	0.00
NACHT, LRR and PYD domains-containing protein 9B-like	LOC100 750914	100750914	0.00	0.00	0.00	0.00	0.00	0.00	0.00	0.00	0.00	0.00	0.00	0.00
schlafen family member 12-like	LOC100 750921	100750921	0.79	0.82	0.51	0.39	0.55	0.47	12.15	7.32	8.69	4.64	7.24	5.61
speckle-type POZ protein pseudogene	LOC100 750922	100750922	0.00	0.00	0.00	0.04	0.00	0.00	0.00	0.92	0.00	1.16	0.00	0.00
phosphatidylinositol 4-phosphate 5-kinase type-1 beta	LOC100 750926	100750926	0.00	0.00	0.00	0.00	0.00	0.00	0.00	0.00	0.00	0.00	0.00	0.00
interferon alpha-2-like	LOC100 750928	100750928	0.00	0.00	0.00	0.00	0.00	0.00	0.00	0.00	0.00	0.00	0.00	0.00
olfactory receptor 5V1	LOC100 750929	100750929	0.00	0.00	0.00	0.00	0.00	0.00	0.00	0.00	0.00	0.00	0.00	0.00
actin-related protein 2 pseudogene	LOC100 750930	100750930	0.16	0.05	0.11	0.00	0.00	0.13	4.56	0.92	2.48	0.00	0.00	3.74
uncharacterized LOC100750931	LOC100 750931	100750931	0.00	0.06	0.00	0.07	0.00	0.00	0.00	0.92	0.00	0.00	0.00	0.93
chromosome unknown open reading frame, human C8orf33	LOC100 750934	100750934	19.80	17.95	20.76	17.68	17.43	17.90	976.01	882.09	1011.06	840.22	801.08	832.79
anaphase-promoting complex subunit 10-like	LOC100 750936	100750936	0.06	0.15	0.00	0.00	0.00	0.08	0.76	1.83	0.00	0.00	0.00	0.93
glutathione S-transferase Mu 2	LOC100 750937	100750937	0.00	0.01	0.00	0.00	0.00	0.00	0.00	0.00	0.00	0.00	0.00	0.00
60S ribosomal protein L15 pseudogene	LOC100 750939	100750939	0.00	0.00	0.00	0.00	0.00	0.00	0.00	0.00	0.00	0.00	0.00	0.00
serine protease 40	LOC100 750943	100750943	0.00	0.00	0.00	0.00	0.00	0.00	0.00	0.00	0.00	0.00	0.00	0.00
V-type proton ATPase subunit G 1 pseudogene	LOC100 750944	100750944	0.00	0.00	0.00	0.00	0.00	0.15	0.76	0.92	0.00	1.16	0.00	1.87
leukocyte immunoglobulin-like receptor subfamily A member 1	LOC100 750945	100750945	0.00	0.01	0.00	0.00	0.00	0.00	0.00	1.83	0.00	0.00	0.00	0.00
non-histone chromosomal protein HMG-17 pseudogene	LOC100 750947	100750947	0.00	0.46	0.00	0.00	0.00	0.16	0.00	2.75	0.00	0.00	0.00	0.93

probable E3 ubiquitin-protein ligase TRIML2 pseudogene	LOC100750949	100750949	0.00	0.00	0.00	0.00	0.00	0.00	0.00	0.00	0.00	0.00	0.00	0.00
vomeronasal type-1 receptor 4-like	LOC100750950	100750950	0.00	0.00	0.00	0.00	0.00	0.00	0.00	0.00	0.00	0.00	0.00	0.00
protein FAM127A-like	LOC100750952	100750952	46.45	41.80	40.81	26.49	14.00	17.90	313.69	293.73	252.14	172.68	84.87	100.01
heterogeneous nuclear ribonucleoprotein A3 pseudogene	LOC100750953	100750953	0.88	1.39	0.98	0.69	0.99	0.29	17.47	27.45	17.39	15.07	17.59	5.61
putative gustatory receptor clone PTE01	LOC100750957	100750957	0.00	0.00	0.00	0.00	0.00	0.00	0.00	0.00	0.00	0.00	0.00	0.00
olfactory receptor 6N1	LOC100750962	100750962	0.00	0.00	0.00	0.00	0.00	0.00	0.00	0.00	0.00	0.00	0.00	0.00
chromosome unknown open reading frame, human C5orf47	LOC100750967	100750967	0.00	0.00	0.00	0.00	0.00	0.00	0.00	0.00	0.00	0.00	0.00	0.00
60S ribosomal protein L9 pseudogene	LOC100750970	100750970	2.51	3.62	2.93	1.43	3.31	1.71	42.53	66.80	53.41	24.34	60.03	29.91
chromosome unknown open reading frame, human C1orf141	LOC100750986	100750986	0.00	0.00	0.00	0.00	0.00	0.00	0.00	0.00	0.00	0.00	1.03	0.00
yrdC domain-containing protein, mitochondrial pseudogene	LOC100750988	100750988	0.40	0.28	0.09	0.09	0.16	0.21	5.32	3.66	2.48	1.16	2.07	2.80
transmembrane protein 11, mitochondrial pseudogene	LOC100750996	100750996	5.56	5.16	5.11	3.53	2.01	2.78	82.03	70.46	74.53	48.67	26.91	33.65
40S ribosomal protein S27-like	LOC100750998	100750998	0.00	1.27	0.45	0.46	0.57	0.00	2.28	4.58	2.48	2.32	1.03	0.93
zinc finger protein 420-like	LOC100751006	100751006	3.05	3.36	3.19	4.05	4.01	3.87	221.03	219.61	212.40	259.60	272.20	266.38
dnaJ homolog subfamily C member 14-like	LOC100751007	100751007	0.04	0.07	0.08	0.02	0.04	0.02	2.28	4.58	3.73	1.16	3.10	1.87
cdc42 effector protein 4-like	LOC100751014	100751014	0.00	0.00	0.00	0.00	0.00	0.00	0.00	0.00	0.00	0.00	0.00	0.00
RE1-silencing transcription factor pseudogene	LOC100751015	100751015	0.00	0.00	0.00	0.00	0.00	0.00	0.00	0.00	0.00	0.00	0.00	0.00
ADP-ribosylation factor-like protein 15	LOC100751017	100751017	0.06	0.07	0.00	0.00	0.17	0.07	0.76	1.83	0.00	0.00	2.07	0.93
sp110 nuclear body protein-like	LOC100751025	100751025	5.02	5.03	5.56	5.06	4.56	4.71	214.95	213.20	252.14	220.19	207.00	217.78

60S ribosomal protein L18 pseudogene	LOC100 751034	100751034	0.00	0.00	0.00	0.00	0.00	0.00	0.76	0.00	0.00	1.16	0.00	0.00
olfactory receptor 10T2	LOC100 751035	100751035	0.00	0.00	0.00	0.00	0.00	0.00	0.00	0.00	0.00	0.00	0.00	0.00
putative DNA repair and recombination protein RAD26-like	LOC100 751043	100751043	0.00	0.00	0.00	0.01	0.00	0.00	0.00	0.00	0.00	1.16	0.00	0.00
MLV-related proviral Env polyprotein-like	LOC100 751046	100751046	0.99	1.35	1.25	0.47	1.08	0.90	23.55	29.28	26.08	11.59	22.77	17.76
60S ribosomal protein L37a-like	LOC100 751047	100751047	0.00	0.00	0.00	0.00	0.00	0.00	0.00	0.00	0.00	1.16	0.00	0.93
keratin-associated protein 4-2-like	LOC100 751049	100751049	0.00	0.00	0.00	0.00	0.00	0.00	0.00	0.00	0.00	0.00	0.00	0.00
olfactory receptor 9I1-like	LOC100 751050	100751050	0.00	0.00	0.00	0.00	0.00	0.00	0.00	0.00	0.00	0.00	0.00	0.00
proto-oncogene FRAT1-like	LOC100 751057	100751057	0.04	0.00	0.16	0.00	0.03	0.02	0.76	0.00	1.24	0.00	0.00	0.00
zinc finger protein 431-like	LOC100 751059	100751059	0.03	0.00	0.00	0.04	0.00	0.03	0.00	0.00	0.00	1.16	0.00	0.00
thyroid transcription factor 1-associated protein 26-like	LOC100 751067	100751067	18.68	19.18	16.86	19.33	19.07	19.09	571.17	590.20	547.76	582.94	584.77	563.61
putative olfactory receptor 5AK3	LOC100 751079	100751079	0.00	0.00	0.00	0.00	0.00	0.00	0.00	0.00	0.00	0.00	0.00	0.00
alpha-enolase-like	LOC100 751081	100751081	0.00	0.00	0.00	0.00	0.00	0.00	0.00	0.00	0.00	0.00	0.00	0.00
probable cation-transporting ATPase 13A5	LOC100 751095	100751095	0.00	0.00	0.00	0.00	0.00	0.00	0.00	0.00	0.00	0.00	0.00	0.00
N-terminal EF-hand calcium-binding protein 1	LOC100 751097	100751097	0.04	0.02	0.00	0.00	0.03	0.00	3.80	1.83	0.00	0.00	1.03	1.87
leukocyte immunoglobulin-like receptor subfamily A member 5	LOC100 751100	100751100	0.00	0.00	0.00	0.00	0.00	0.00	0.00	0.92	0.00	0.00	0.00	0.00
olfactory receptor 145-like	LOC100 751102	100751102	0.00	0.00	0.00	0.00	0.00	0.00	0.00	0.00	0.00	0.00	0.00	0.00
acidic leucine-rich nuclear phosphoprotein 32 family member E pseudogene	LOC100 751105	100751105	0.00	0.00	0.00	0.00	0.00	0.00	0.00	0.00	0.00	0.00	0.00	0.00
serine/arginine-rich splicing factor 3 pseudogene	LOC100 751106	100751106	1.45	1.19	0.68	0.53	1.26	0.97	20.51	15.56	12.42	5.79	15.52	14.02

proteasome subunit beta type-3 pseudogene	LOC100751111	100751111	0.00	0.00	0.00	0.00	0.00	0.00	0.00	0.00	0.00	0.00	0.00	0.00
keratin-associated protein 19-4-like	LOC100751114	100751114	0.00	0.00	0.00	0.00	0.00	0.00	0.00	0.00	0.00	0.00	0.00	0.00
aphrodisin-like	LOC100751116	100751116	0.00	0.00	0.00	0.00	0.00	0.00	0.00	0.00	0.00	0.00	0.00	0.00
Ig heavy chain V-III region VH26-like	LOC100751117	100751117	0.00	0.00	0.00	0.00	0.00	0.00	0.00	0.00	0.00	0.00	0.00	0.00
chromosome unknown open reading frame, human C16orf52	LOC100751118	100751118	10.38	10.80	11.30	11.74	13.07	12.12	410.15	418.17	434.73	429.96	460.57	430.88
40S ribosomal protein S27-like	LOC100751121	100751121	0.08	0.00	0.26	0.00	0.00	0.00	0.00	0.92	0.00	0.00	0.00	0.00
transmembrane O-methyltransferase	LOC100751123	100751123	0.13	0.23	0.16	0.23	0.11	0.19	10.63	13.73	9.94	13.91	7.24	13.09
kelch-like protein 7 pseudogene	LOC100751125	100751125	2.28	2.89	2.12	2.35	2.51	2.63	185.33	178.43	176.38	206.29	195.61	235.54
sal-like protein 1 pseudogene	LOC100751128	100751128	1.12	0.98	0.50	0.35	0.22	0.13	108.61	93.33	45.96	34.77	20.70	14.02
tudor and KH domain-containing protein pseudogene	LOC100751129	100751129	0.00	0.00	0.00	0.00	0.00	0.00	0.00	0.00	0.00	0.00	0.00	0.00
disintegrin and metalloproteinase domain-containing protein 25-like	LOC100751130	100751130	0.00	0.00	0.00	0.00	0.00	0.00	0.00	0.00	0.00	0.00	0.00	0.00
60S ribosomal protein L31 pseudogene	LOC100751132	100751132	0.00	0.00	0.00	0.00	0.00	0.00	0.00	0.00	0.00	0.00	0.00	0.00
proto-oncogene FRAT1-like	LOC100751138	100751138	0.00	0.00	0.00	0.00	0.00	0.00	0.00	0.00	0.00	0.00	0.00	0.00
[Pyruvate dehydrogenase (acetyl-transferring)] kinase isozyme 1, mitochondrial pseudogene	LOC100751145	100751145	0.30	0.36	0.36	0.28	0.49	0.35	9.11	11.90	11.18	9.27	15.52	9.35
synaptobrevin homolog YKT6-like	LOC100751147	100751147	0.13	0.00	0.00	0.10	0.09	0.20	2.28	0.92	0.00	2.32	3.10	3.74
histone H2B type 1	LOC100751148	100751148	6.97	6.09	6.93	3.40	2.22	2.83	2.28	0.00	0.00	0.00	0.00	0.00
immunoglobulin omega chain-like	LOC100751150	100751150	0.00	0.00	0.00	0.00	0.00	0.00	0.00	0.00	0.00	0.00	0.00	0.00
olfactory receptor 2A12-like	LOC100751152	100751152	0.00	0.00	0.00	0.00	0.00	0.00	0.00	0.00	0.00	0.00	0.00	0.00

serine/threonine-protein phosphatase 2A 65 kDa regulatory subunit A alpha isoform pseudogene	LOC100751160	100751160	0.66	0.53	0.25	0.42	0.58	0.46	28.86	23.79	11.18	16.22	25.87	19.63
olfactory receptor 10AG1-like	LOC100751163	100751163	0.00	0.00	0.00	0.00	0.00	0.00	0.00	0.00	0.00	0.00	0.00	0.00
60S acidic ribosomal protein P1 pseudogene	LOC100751165	100751165	0.00	0.00	0.00	0.00	0.00	0.00	0.00	0.00	0.00	0.00	0.00	0.00
60S ribosomal protein L18a pseudogene	LOC100751167	100751167	0.08	0.00	0.00	0.00	0.00	0.00	0.76	0.00	0.00	0.00	0.00	0.00
prohibitin pseudogene	LOC100751168	100751168	2.57	1.21	0.92	0.59	0.89	0.80	47.85	24.71	16.15	10.43	16.56	15.89
60S acidic ribosomal protein P1	LOC100751171	100751171	31.00	52.15	42.50	31.33	21.04	16.27	186.85	250.72	223.58	129.80	47.61	55.15
aflatoxin B1 aldehyde reductase member 3-like	LOC100751175	100751175	0.10	0.03	0.08	0.11	0.00	0.09	3.04	0.92	2.48	3.48	0.00	2.80
DBH-like monooxygenase protein 2	LOC100751176	100751176	0.00	0.00	0.00	0.00	0.00	0.00	0.00	0.00	0.00	0.00	0.00	0.00
prohibitin pseudogene	LOC100751177	100751177	0.00	0.00	0.00	0.00	0.00	0.00	0.00	0.00	0.00	0.00	0.00	0.00
zinc finger CCCH domain-containing protein 15 pseudogene	LOC100751179	100751179	9.36	9.48	7.51	7.82	7.67	6.56	34.94	39.35	33.54	20.86	27.94	15.89
mitochondrial import inner membrane translocase subunit Tim23-like	LOC100751182	100751182	0.00	0.00	0.00	0.00	0.00	0.00	0.00	0.00	0.00	0.00	0.00	0.00
acyl-CoA desaturase 1	LOC100751184	100751184	4.06	4.41	5.89	5.16	5.46	5.20	37.98	50.33	44.72	32.45	50.71	31.78
olfactory receptor 5AC1-like	LOC100751186	100751186	0.00	0.00	0.00	0.00	0.00	0.00	0.00	0.00	0.00	0.00	0.00	0.00
ADP-ribosylation factor-binding protein GGA2-like	LOC100751188	100751188	0.00	0.00	0.03	0.00	0.00	0.00	0.00	0.00	1.24	0.00	0.00	0.00
ral guanine nucleotide dissociation stimulator-like	LOC100751191	100751191	0.00	0.00	0.00	0.00	0.00	0.00	0.00	0.00	0.00	0.00	0.00	0.93
eukaryotic translation elongation factor 1 epsilon-1 pseudogene	LOC100751195	100751195	23.41	25.97	25.16	23.39	24.70	22.59	353.19	402.61	390.02	349.99	374.67	339.29
intestinal-type alkaline phosphatase	LOC100751196	100751196	0.00	0.00	0.00	0.00	0.00	0.00	0.00	0.00	0.00	0.00	0.00	0.00
trace amine-associated receptor 4	LOC100751198	100751198	0.00	0.00	0.00	0.00	0.00	0.00	0.00	0.00	0.00	0.00	0.00	0.00

PRAME family member 5-like	LOC100 751199	100751199	0.00	0.00	0.00	0.00	0.00	0.00	0.00	0.00	0.00	0.00	0.00	0.00
zinc finger protein 157-like	LOC100 751201	100751201	0.01	0.01	0.00	0.01	0.01	0.01	0.00	0.00	0.00	0.00	0.00	0.00
pyridoxal kinase-like	LOC100 751202	100751202	11.04	10.79	7.79	6.50	5.33	6.28	12.91	13.73	9.94	9.27	3.10	5.61
chromosome unknown open reading frame, human C16orf93	LOC100 751203	100751203	1.57	2.02	2.28	1.91	1.46	1.93	73.68	91.50	101.85	83.44	69.34	83.19
uncharacterized protein C2orf78 homolog	LOC100 751207	100751207	0.00	0.00	0.00	0.00	0.05	0.00	0.00	0.92	0.00	0.00	2.07	0.00
chromosome unknown open reading frame, human C1orf177	LOC100 751212	100751212	0.00	0.00	0.00	0.04	0.07	0.00	0.00	0.00	0.00	1.16	2.07	0.00
speckle-type POZ protein-like	LOC100 751214	100751214	0.00	0.00	0.00	0.00	0.00	0.00	0.00	0.00	0.00	0.00	0.00	0.00
uncharacterized LOC100751217	LOC100 751217	100751217	0.00	0.00	0.00	0.00	0.00	0.00	0.00	0.00	0.00	0.00	0.00	0.00
suppressor of cytokine signaling 1-like	LOC100 751218	100751218	2.64	2.36	2.58	1.64	1.31	1.04	75.95	70.46	74.53	46.36	36.22	28.97
chromosome unknown open reading frame, human C1orf158	LOC100 751219	100751219	0.00	0.00	0.00	0.00	0.00	0.00	0.00	0.00	0.00	0.00	0.00	0.00
interferon alpha-12	LOC100 751221	100751221	0.00	0.00	0.00	0.00	0.00	0.00	0.00	0.00	0.00	0.00	0.00	0.00
olfactory receptor 5V1	LOC100 751222	100751222	0.00	0.00	0.00	0.00	0.00	0.00	0.00	0.00	0.00	0.00	0.00	0.00
thiosulfate sulfurtransferase/rhodanese-like domain-containing protein 3 pseudogene	LOC100 751223	100751223	0.14	0.04	0.12	0.00	0.15	0.18	0.76	0.92	1.24	0.00	2.07	1.87
pancreatic triacylglycerol lipase-like	LOC100 751227	100751227	0.00	0.00	0.00	0.00	0.00	0.00	2.28	3.66	8.69	8.11	5.17	3.74
prostatic steroid-binding protein C1-like	LOC100 751230	100751230	0.00	0.00	0.00	0.00	0.00	0.00	0.76	0.00	0.00	0.00	1.03	0.00
multifunctional protein ADE2 pseudogene	LOC100 751231	100751231	1.48	1.27	1.31	1.07	1.03	0.68	22.79	18.30	26.08	20.86	15.52	24.30
speckle-type POZ protein pseudogene	LOC100 751233	100751233	0.00	0.00	0.00	0.00	0.00	0.00	0.00	0.00	0.00	0.00	0.00	0.00
tetratricopeptide repeat protein 30A1	LOC100 751235	100751235	0.92	0.89	0.94	0.87	0.98	0.97	9.11	7.32	12.42	11.59	8.28	6.54

nucleoporin SEH1 pseudogene	LOC100 751237	100751237	0.22	0.32	0.31	0.28	0.25	0.25	1.52	3.66	2.48	3.48	4.14	2.80
bile salt sulfotransferase 1-like	LOC100 751240	100751240	0.00	0.00	0.00	0.00	0.00	0.00	0.00	0.00	0.00	0.00	0.00	0.00
chromosome unknown open reading frame, human C14orf180	LOC100 751241	100751241	0.00	0.00	0.00	0.00	0.00	0.00	0.00	0.00	0.00	0.00	0.00	0.00
60S ribosomal protein L3-like	LOC100 751242	100751242	0.00	0.00	0.00	0.00	0.00	0.00	0.00	0.00	0.00	0.00	0.00	0.00
26S protease regulatory subunit 4 pseudogene	LOC100 751245	100751245	0.00	0.00	0.00	0.00	0.00	0.00	0.00	0.00	0.00	0.00	0.00	0.00
olfactory receptor 7D4-like	LOC100 751251	100751251	0.00	0.00	0.00	0.00	0.00	0.00	0.00	0.00	0.00	0.00	0.00	0.00
beclin-2-like	LOC100 751252	100751252	0.05	0.03	0.11	0.03	0.16	0.11	3.80	2.75	4.97	2.32	7.24	5.61
adenylate kinase 4, mitochondrial pseudogene	LOC100 751253	100751253	0.34	0.09	0.13	0.12	0.11	0.33	4.56	3.66	1.24	1.16	1.03	4.67
olfactory receptor 6N2	LOC100 751255	100751255	0.00	0.00	0.00	0.00	0.00	0.00	0.00	0.00	0.00	0.00	0.00	0.00
zinc finger protein 248-like	LOC100 751259	100751259	0.00	0.00	0.00	0.00	0.00	0.00	0.00	0.00	0.00	1.16	0.00	0.00
cytochrome b5 type B-like	LOC100 751261	100751261	23.12	21.72	16.12	16.89	15.01	20.46	176.21	162.88	121.72	124.00	112.81	151.42
nucleophosmin pseudogene	LOC100 751264	100751264	0.22	0.07	0.05	0.14	0.18	0.26	6.08	0.92	1.24	2.32	5.17	4.67
intercellular adhesion molecule 2-like	LOC100 751267	100751267	0.46	0.47	0.19	0.27	0.21	0.19	35.70	37.52	16.15	16.22	16.56	14.95
cytochrome P450 1A1	LOC100 751269	100751269	0.00	0.00	0.00	0.00	0.00	0.00	0.00	0.00	0.00	0.00	0.00	0.00
1,25-dihydroxyvitamin D(3) 24-hydroxylase, mitochondrial	LOC100 751280	100751280	0.04	0.02	0.00	0.02	0.00	0.03	3.04	1.83	1.24	1.16	0.00	1.87
olfactory receptor 7C2	LOC100 751281	100751281	0.00	0.00	0.00	0.00	0.00	0.00	0.00	0.00	0.00	0.00	0.00	0.00
putative dimethylaniline monooxygenase [N-oxide-forming] 6	LOC100 751286	100751286	0.00	0.00	0.00	0.00	0.00	0.02	0.00	0.00	0.00	0.00	0.00	0.93
poly [ADP-ribose] polymerase 2 pseudogene	LOC100 751288	100751288	0.06	0.19	0.11	0.08	0.09	0.02	2.28	6.41	4.97	3.48	4.14	0.93

T-cell ecto-ADP-ribosyltransferase 1-like	LOC100751294	100751294	0.00	0.00	0.00	0.00	0.00	0.00	0.00	0.00	0.00	0.00	0.00	0.00
zinc finger protein 383-like	LOC100751297	100751297	0.00	0.00	0.00	0.00	0.02	0.01	0.00	0.00	0.00	0.00	0.00	0.00
tudor and KH domain-containing protein pseudogene	LOC100751298	100751298	0.00	0.00	0.00	0.05	0.00	0.00	0.76	0.00	0.00	1.16	0.00	0.00
zinc finger protein 58-like	LOC100751304	100751304	3.27	2.91	3.82	4.44	4.81	4.75	331.92	332.16	392.50	445.03	492.65	491.64
40S ribosomal protein S14 pseudogene	LOC100751306	100751306	0.00	0.00	0.00	0.00	0.00	0.00	0.00	0.00	0.00	0.00	0.00	0.00
barrier-to-autointegration factor-like	LOC100751307	100751307	18.03	13.65	11.68	10.74	9.49	11.84	253.69	298.30	224.82	200.49	114.88	157.02
uncharacterized LOC100751308	LOC100751308	100751308	0.00	0.00	0.00	0.00	0.00	0.00	0.00	0.00	0.00	0.00	0.00	0.00
PDZ domain-containing protein 7-like	LOC100751311	100751311	0.18	0.25	0.27	0.28	0.20	0.23	15.19	11.90	14.91	12.75	13.45	11.22
nuclear body protein SP140-like protein	LOC100751314	100751314	0.06	0.03	0.03	0.03	0.00	0.02	3.04	0.92	1.24	1.16	0.00	0.93
probable hydrolase PNKD	LOC100751315	100751315	32.29	30.70	34.60	31.48	25.50	31.11	559.78	541.70	611.11	520.36	385.01	469.20
sperm motility kinase X-like	LOC100751316	100751316	0.04	0.10	0.00	0.00	0.07	0.05	0.00	0.92	0.00	0.00	1.03	0.00
annexin A7 pseudogene	LOC100751323	100751323	0.06	0.08	0.00	0.07	0.03	0.00	2.28	2.75	0.00	2.32	1.03	0.00
UDP-glucuronosyltransferase 2B4-like	LOC100751325	100751325	0.00	0.00	0.00	0.00	0.00	0.00	0.00	0.00	0.00	0.00	0.00	0.00
cofilin-1-like	LOC100751327	100751327	0.00	0.00	0.00	0.00	0.00	0.00	0.00	0.00	0.00	0.00	0.00	0.00
bile salt sulfotransferase 1-like	LOC100751331	100751331	0.00	0.00	0.00	0.00	0.00	0.00	0.00	0.00	0.00	0.00	0.00	0.00
PRAME family member 5-like	LOC100751333	100751333	0.00	0.00	0.00	0.00	0.00	0.00	0.00	0.00	0.00	0.00	0.00	0.00
taste receptor type 2 member 134-like	LOC100751335	100751335	0.00	0.00	0.00	0.00	0.00	0.00	0.00	0.00	0.00	0.00	0.00	0.00
uncharacterized LOC100751337	LOC100751337	100751337	2.54	1.93	1.80	2.41	3.13	3.53	126.84	96.08	103.09	127.48	155.25	170.11

keratin-associated protein 10-7-like	LOC100751340	100751340	0.00	0.00	0.00	0.00	0.00	0.00	0.00	0.00	0.00	0.00	0.00	0.00
complement factor H-related protein 2-like	LOC100751341	100751341	0.00	0.00	0.00	0.00	0.10	0.00	0.00	0.00	0.00	0.00	1.03	0.00
FUN14 domain-containing protein 1 pseudogene	LOC100751342	100751342	0.18	0.00	0.15	0.00	0.13	0.06	1.52	0.00	1.24	0.00	1.03	0.00
melanoma-associated antigen 10-like	LOC100751350	100751350	0.49	0.72	0.40	0.45	0.37	0.22	0.00	0.00	0.00	0.00	0.00	0.00
nebulette-like	LOC100751354	100751354	0.00	0.00	0.00	0.00	0.00	0.00	0.00	0.00	0.00	0.00	0.00	0.00
olfactory receptor 4D2	LOC100751363	100751363	0.00	0.00	0.00	0.00	0.00	0.00	0.00	0.00	0.00	0.00	0.00	0.00
bile acid receptor-like	LOC100751364	100751364	0.11	0.19	0.58	0.71	1.14	1.08	9.87	12.81	47.20	53.31	94.18	79.45
etoposide-induced protein 2.4 homolog pseudogene	LOC100751366	100751366	0.00	0.00	0.00	0.00	0.00	0.06	0.00	0.92	0.00	0.00	0.00	1.87
glyceraldehyde-3-phosphate dehydrogenase pseudogene	LOC100751368	100751368	0.00	0.00	0.00	0.00	0.00	0.00	0.00	0.00	0.00	0.00	0.00	0.00
olfactory receptor 5G3-like	LOC100751371	100751371	0.00	0.00	0.00	0.00	0.00	0.00	0.00	0.00	0.00	0.00	0.00	0.00
52 kDa repressor of the inhibitor of the protein kinase-like	LOC100751373	100751373	0.87	1.15	1.23	0.92	0.87	1.00	43.29	51.24	63.35	59.10	53.82	56.08
left-right determination factor 1	LOC100751377	100751377	0.18	0.17	0.17	0.14	0.10	0.09	22.79	17.39	17.39	15.07	8.28	10.28
popy Class I histocompatibility antigen, A-1 alpha chain-like	LOC100751378	100751378	0.00	0.00	0.00	0.00	0.00	0.00	0.00	0.00	0.00	0.00	0.00	0.00
ATP synthase F(0) complex subunit B1, mitochondrial pseudogene	LOC100751380	100751380	0.00	0.00	0.00	0.00	0.00	0.00	0.00	0.00	0.00	0.00	0.00	0.00
prolactin-3B1-like	LOC100751386	100751386	0.00	0.00	0.00	0.00	0.00	0.00	0.00	0.00	0.00	0.00	0.00	0.00
elongation factor 1-gamma pseudogene	LOC100751389	100751389	1.57	1.68	1.21	0.87	1.17	1.30	51.65	55.82	40.99	27.81	39.33	41.13
Insulin receptor substrate 1	LOC100751390	100751390	0.34	0.26	0.40	0.45	0.45	0.48	25.82	24.71	37.26	49.83	39.33	47.67
schlafen family member 12-like	LOC100751393	100751393	53.07	52.53	31.78	34.68	35.63	38.62	3520.46	3488.11	2130.18	2296.98	2341.14	2557.26

importin subunit alpha-8-like	LOC100751395	100751395	0.00	0.00	0.00	0.00	0.00	0.00	0.00	0.00	0.00	0.00	0.00	0.00
granzyme A-like	LOC100751397	100751397	0.00	0.00	0.00	0.00	0.00	0.00	0.00	0.00	0.00	0.00	0.00	0.00
proteasome subunit alpha type-3 pseudogene	LOC100751403	100751403	0.00	0.00	0.00	0.00	0.00	0.00	3.80	0.92	1.24	0.00	0.00	0.93
kxDL motif-containing protein 1 pseudogene	LOC100751404	100751404	0.11	0.13	0.32	0.04	0.15	0.14	0.76	1.83	3.73	0.00	2.07	1.87
outer dense fiber protein 1-like	LOC100751407	100751407	0.11	0.05	0.00	0.00	0.00	0.00	0.00	0.00	0.00	1.16	0.00	0.00
Ig heavy chain V region 5A-like	LOC100751409	100751409	0.00	0.00	0.00	0.00	0.00	0.00						
60S ribosomal protein L9 pseudogene	LOC100751413	100751413	0.79	0.81	0.65	0.00	0.74	0.70	11.39	10.07	8.69	0.00	8.28	10.28
40S ribosomal protein SA pseudogene	LOC100751424	100751424	0.00	0.00	0.20	0.06	0.00	0.00	0.00	0.00	4.97	1.16	0.00	0.00
dolichyl-diphosphooligosaccharide--protein glycosyltransferase subunit 4-like	LOC100751425	100751425	0.00	0.00	0.00	0.00	0.00	0.00	0.00	0.00	0.00	0.00	0.00	0.00
S-adenosyl-L-methionine-dependent tRNA 4-demethylwyosine synthase-like	LOC100751426	100751426	9.42	8.40	11.47	13.23	10.21	11.51	202.04	172.94	243.45	268.87	211.14	237.41
uncharacterized LOC100751430	LOC100751430	100751430	0.20	0.12	0.00	0.00	0.18	0.20	6.84	2.75	2.48	1.16	5.17	5.61
olfactory receptor 2AG1	LOC100751431	100751431	0.00	0.00	0.00	0.00	0.00	0.00	0.00	0.00	0.00	0.00	0.00	0.00
microtubule-associated protein 4 pseudogene	LOC100751433	100751433	27.32	26.54	23.51	25.45	35.43	31.36	0.00	0.00	0.00	0.00	0.00	0.00
meiotic nuclear division protein 1 homolog pseudogene	LOC100751437	100751437	0.00	0.00	0.00	0.00	0.00	0.00	0.00	0.00	0.00	0.00	1.03	1.87
histone H2B type 1	LOC100751440	100751440	1.92	3.13	1.61	0.64	0.77	0.68	0.00	4.58	0.00	0.00	0.00	0.00
60S ribosomal protein L27 pseudogene	LOC100751443	100751443	0.00	0.00	0.00	0.00	0.00	0.00	0.00	0.00	0.00	0.00	0.00	0.00
olfactory receptor 2A12-like	LOC100751444	100751444	0.00	0.00	0.00	0.00	0.00	0.00	0.00	0.00	0.00	0.00	0.00	0.00
uncharacterized LOC100751446	LOC100751446	100751446	0.00	0.00	0.00	0.00	0.00	0.00	0.00	0.00	0.00	0.00	0.00	0.00

sperm motility kinase 2B-like	LOC100 751450	100751450	0.00	0.00	0.00	0.00	0.00	0.00	0.00	0.00	1.24	0.00	0.00	0.00
tubulin gamma-2 chain	LOC100 751452	100751452	7.88	8.02	6.07	4.40	2.05	2.22	47.85	41.18	38.50	28.97	1.03	1.87
olfactory receptor 10AG1-like	LOC100 751455	100751455	0.00	0.00	0.00	0.00	0.00	0.00	0.00	0.00	0.00	0.00	0.00	0.00
10 kDa heat shock protein, mitochondrial pseudogene	LOC100 751460	100751460	0.00	0.00	0.00	0.00	0.00	0.00	0.76	0.00	0.00	0.00	0.00	0.00
aflatoxin B1 aldehyde reductase member 3-like	LOC100 751467	100751467	0.48	0.49	0.80	0.75	0.40	0.36	25.06	21.96	38.50	31.29	20.70	26.17
glyceraldehyde-3-phosphate dehydrogenase-like	LOC100 751473	100751473	0.09	0.11	0.15	0.14	0.25	0.34	2.28	0.92	1.24	1.16	2.07	6.54
olfactory receptor 5AC1-like	LOC100 751478	100751478	0.00	0.00	0.00	0.00	0.00	0.00	0.00	0.00	0.00	0.00	0.00	0.00
glyceraldehyde-3-phosphate dehydrogenase pseudogene	LOC100 751481	100751481	0.00	0.00	0.00	0.00	0.00	0.00	0.00	0.00	0.00	0.00	0.00	0.00
60S ribosomal protein L7a pseudogene	LOC100 751485	100751485	2.37	2.51	2.22	2.03	2.39	1.94	27.34	15.56	19.87	18.54	21.73	10.28
paired immunoglobulin-like type 2 receptor beta	LOC100 751486	100751486	0.00	0.00	0.00	0.00	0.00	0.00	0.00	0.00	0.00	0.00	0.00	0.00
uncharacterized LOC100751490	LOC100 751490	100751490	0.00	0.00	0.00	0.00	0.00	0.07	1.52	0.92	0.00	0.00	0.00	1.87
cytochrome P450 2C26-like	LOC100 751491	100751491	0.00	0.00	0.00	0.00	0.00	0.00	0.00	0.00	0.00	0.00	0.00	0.00
uncharacterized LOC100751494	LOC100 751494	100751494	5.23	4.79	5.40	4.75	4.56	5.85	185.33	170.20	187.56	171.52	158.35	201.89
cation channel sperm-associated protein subunit beta-like	LOC100 751495	100751495	0.00	0.00	0.00	0.00	0.00	0.00	0.00	0.00	0.00	0.00	0.00	0.00
STAM-binding protein-like	LOC100 751498	100751498	0.00	0.00	0.00	0.00	0.00	0.00	0.00	0.92	0.00	0.00	0.00	0.00
cytochrome c oxidase assembly protein COX15 homolog	LOC100 751499	100751499	60.65	58.90	61.89	58.56	60.86	61.04	2748.01	2710.33	2823.26	2711.87	2834.83	2892.80
protein-lysine methyltransferase METTL21C	LOC100 751500	100751500	0.00	0.00	0.00	0.00	0.00	0.00	0.00	0.00	0.00	0.00	0.00	0.00
speckle-type POZ protein pseudogene	LOC100 751508	100751508	0.00	0.00	0.00	0.00	0.00	0.00	0.00	0.00	0.00	0.00	0.00	0.00

PRAME family member 20/21-like	LOC100751513	100751513	0.00	0.00	0.00	0.00	0.00	0.00	0.00	0.00	0.00	0.00	0.00	0.00
tRNA wybutosine-synthesizing protein 5-like	LOC100751516	100751516	0.11	0.00	0.00	0.00	0.07	0.00	1.52	0.00	0.00	0.00	1.03	0.00
cysteine sulfinic acid decarboxylase	LOC100751520	100751520	22.45	22.52	29.54	28.77	25.90	29.12	682.07	700.00	884.37	855.28	782.45	851.48
chromosome unknown open reading frame, human C10orf120	LOC100751521	100751521	0.00	0.00	0.00	0.00	0.00	0.00	0.00	0.00	0.00	0.00	0.00	0.00
speckle-type POZ protein pseudogene	LOC100751526	100751526	0.00	0.00	0.00	0.00	0.00	0.00	0.00	0.00	0.00	0.00	0.00	0.00
leukocyte immunoglobulin-like receptor subfamily B member 3	LOC100751527	100751527	0.00	0.00	0.00	0.00	0.00	0.00	0.00	0.00	0.00	0.00	0.00	0.00
tetratricopeptide repeat protein 30A2	LOC100751528	100751528	1.62	1.73	1.98	1.83	2.01	2.05	12.91	16.47	17.39	19.70	17.59	18.69
olfactory receptor 688	LOC100751530	100751530	0.00	0.00	0.00	0.00	0.00	0.00	0.00	0.00	0.00	0.00	0.00	0.00
uncharacterized protein C2orf78 homolog	LOC100751533	100751533	0.10	0.15	0.07	0.09	0.12	0.04	6.08	10.07	4.97	4.64	7.24	8.41
inverted formin-2	LOC100751534	100751534	19.19	19.80	17.28	14.77	13.11	14.25	1705.17	1752.29	1537.70	1303.78	1182.99	1270.22
U3 small nucleolar RNA-associated protein 14 homolog A-like	LOC100751535	100751535	5.35	5.67	4.78	4.62	3.98	4.09	0.00	0.00	0.00	0.00	0.00	0.00
40S ribosomal protein S2-like	LOC100751538	100751538	0.05	0.00	0.00	0.14	0.00	0.00	0.76	0.00	0.00	2.32	0.00	0.00
60S ribosomal protein L30 pseudogene	LOC100751543	100751543	0.00	0.00	0.00	0.00	0.00	0.00	0.00	0.00	0.00	0.00	2.07	0.00
olfactory receptor 7E24-like	LOC100751544	100751544	0.00	0.00	0.00	0.00	0.00	0.00	0.00	0.00	0.00	0.00	0.00	0.00
inhibitor of Bruton tyrosine kinase	LOC100751546	100751546	25.67	26.44	14.61	16.51	19.36	17.30	2995.62	3074.51	1750.10	1953.94	2224.19	2049.73
olfactory receptor 6N1	LOC100751548	100751548	0.00	0.00	0.00	0.00	0.00	0.00	0.00	0.00	0.00	0.00	0.00	0.00
cytochrome b561 domain-containing protein 2	LOC100751550	100751550	6.35	5.86	7.04	5.36	4.62	4.73	293.18	278.17	319.22	245.69	204.93	225.26
60S ribosomal protein L30 pseudogene	LOC100751552	100751552	0.00	0.00	0.00	0.00	0.00	0.00	0.00	0.00	0.00	0.00	0.00	0.00

chromosome unknown open reading frame, human C1orf21	LOC100751554	100751554	10.35	11.01	11.09	11.45	15.58	14.09	851.44	916.86	906.72	930.61	1243.02	1126.28
SNRPN upstream reading frame protein pseudogene	LOC100751555	100751555	0.00	0.00	0.00	0.00	0.00	0.00	0.76	1.83	0.00	1.16	0.00	0.00
cytochrome c oxidase subunit 7B, mitochondrial pseudogene	LOC100751557	100751557	0.00	0.00	0.00	0.00	0.00	0.00	0.76	0.92	1.24	1.16	1.03	1.87
coiled-coil domain-containing protein 47	LOC100751560	100751560	87.75	86.07	91.88	102.32	99.99	95.55	2724.47	2688.37	2876.67	3159.22	3141.18	2976.92
coiled-coil domain-containing protein 47 pseudogene	LOC100751567	100751567	0.00	0.00	0.00	0.00	0.00	0.00	0.00	0.00	0.00	0.00	0.00	0.00
olfactory receptor 2T6	LOC100751570	100751570	0.00	0.00	0.00	0.00	0.00	0.00	0.00	0.00	0.00	0.00	0.00	0.00
chromosome unknown open reading frame, human C17orf105	LOC100751574	100751574	0.12	0.07	0.00	0.00	0.00	0.07	2.28	2.75	0.00	0.00	1.03	4.67
dimethylaniline monooxygenase [N-oxide-forming] 3	LOC100751580	100751580	0.00	0.00	0.00	0.00	0.00	0.00	0.00	0.00	1.24	0.00	0.00	0.00
beta-galactosidase-1-like protein 2	LOC100751583	100751583	0.00	0.00	0.00	0.00	0.00	0.00	0.00	0.00	0.00	0.00	0.00	0.00
sterol 26-hydroxylase, mitochondrial	LOC100751584	100751584	0.02	0.00	0.00	0.00	0.02	0.00	2.28	0.92	0.00	0.00	1.03	0.00
chromosome unknown open reading frame, human C11orf83	LOC100751590	100751590	31.06	29.97	26.20	11.87	4.98	4.87	391.92	381.57	339.09	180.79	89.01	84.12
vomeronasal type-1 receptor 4-like	LOC100751592	100751592	0.00	0.00	0.00	0.00	0.00	0.00	0.00	0.00	0.00	0.00	0.00	0.00
cytochrome P450 2J5-like	LOC100751593	100751593	0.00	0.00	0.00	0.00	0.00	0.00	0.00	0.00	0.00	0.00	0.00	0.00
zinc finger protein 58-like	LOC100751600	100751600	2.27	1.83	2.49	2.99	3.27	3.17	152.67	127.19	168.92	206.29	215.28	214.97
eukaryotic translation initiation factor 1 pseudogene	LOC100751604	100751604	1.84	0.35	1.21	0.47	0.41	1.13	8.35	0.92	6.21	1.16	2.07	5.61
uncharacterized LOC100751607	LOC100751607	100751607	2.47	2.10	0.85	0.86	3.22	3.71	41.02	37.52	34.78	41.72	49.68	48.60
nuclear body protein SP140-like protein	LOC100751610	100751610	0.16	0.25	0.24	0.07	0.07	0.03	3.80	8.24	6.21	4.64	2.07	0.93
induced myeloid leukemia cell differentiation protein Mcl-1 homolog	LOC100751612	100751612	5.78	4.91	4.16	2.61	2.92	2.78	72.16	59.48	63.35	33.61	32.08	30.84

60S ribosomal protein L7 pseudogene	LOC100 751613	100751613	2.15	3.01	3.30	2.01	2.87	1.57	28.86	41.18	42.23	23.18	35.19	18.69
cell division cycle 5-like protein pseudogene	LOC100 751614	100751614	0.04	0.05	0.11	0.05	0.11	0.11	1.52	1.83	6.21	2.32	4.14	6.54
40S ribosomal protein S28 pseudogene	LOC100 751615	100751615	0.00	0.00	0.00	0.00	0.00	0.00	0.00	0.00	0.00	0.00	0.00	0.00
spermatogenesis-associated protein 7 homolog	LOC100 751616	100751616	5.57	5.59	4.68	5.51	5.56	4.59	205.08	199.48	173.89	208.61	215.28	169.18
protein SET-like	LOC100 751617	100751617	3.03	2.93	2.71	2.35	2.21	1.97	65.32	59.48	63.35	63.74	47.61	56.08
secreted seminal-vesicle Ly-6 protein 1-like	LOC100 751623	100751623	0.00	0.00	0.00	0.00	0.00	0.00	0.00	0.00	0.00	0.00	0.00	0.00
bile salt sulfotransferase 1-like	LOC100 751624	100751624	0.00	0.05	0.00	0.00	0.00	0.00	0.00	0.92	0.00	0.00	0.00	0.00
RNA-binding protein PNO1 pseudogene	LOC100 751626	100751626	0.19	0.20	0.16	0.00	0.02	0.08	4.56	4.58	3.73	0.00	0.00	1.87
high mobility group protein B1 pseudogene	LOC100 751631	100751631	14.78	18.03	13.63	14.98	14.77	14.98	246.09	300.13	234.75	282.78	223.56	257.03
40S ribosomal protein SA pseudogene	LOC100 751634	100751634	118.42	129.58	138.42	129.86	73.24	90.38	6232.78	6763.01	7507.17	6699.72	5381.93	5529.51
C-type lectin domain family 2 member H-like	LOC100 751635	100751635	0.00	0.00	0.00	0.00	0.00	0.00	0.00	0.00	0.00	0.00	0.00	0.00
olfactory receptor 183	LOC100 751637	100751637	0.00	0.00	0.00	0.00	0.00	0.00	0.00	0.00	0.00	0.00	0.00	0.00
UDP-glucuronosyltransferase 2B17-like	LOC100 751639	100751639	0.00	0.00	0.00	0.00	0.00	0.00	0.00	0.00	0.00	0.00	0.00	0.00
zinc finger MYM-type protein 5 pseudogene	LOC100 751643	100751643	0.13	0.22	0.20	0.07	0.20	0.16	6.08	7.32	4.97	3.48	4.14	2.80
60S ribosomal protein L9 pseudogene	LOC100 751651	100751651	1.34	1.14	1.00	0.65	1.31	0.93	3.80	3.66	2.48	3.48	6.21	2.80
protein FAM205A-like	LOC100 751656	100751656	0.00	0.00	0.00	0.00	0.00	0.00	0.00	0.00	0.00	0.00	0.00	0.00
60S acidic ribosomal protein P0 pseudogene	LOC100 751657	100751657	0.00	0.00	0.00	0.00	0.00	0.00	0.00	0.00	0.00	0.00	0.00	0.93
60S ribosomal protein L23a-like	LOC100 751658	100751658	0.00	0.36	0.00	0.00	0.00	0.00	0.00	2.75	0.00	0.00	0.00	0.93

olfactory receptor 1038-like	LOC100 751660	100751660	0.00	0.00	0.00	0.00	0.00	0.00	0.00	0.00	0.00	0.00	0.00	0.00
saoe class I histocompatibility antigen, A alpha chain-like	LOC100 751667	100751667	0.00	0.00	0.00	0.00	0.00	0.00	0.00	0.00	0.00	0.00	0.00	0.00
L-lactate dehydrogenase C chain-like	LOC100 751672	100751672	3.11	3.54	0.36	0.60	0.10	0.12	114.69	118.95	12.42	20.86	4.14	4.67
proteasome subunit beta type-3 pseudogene	LOC100 751673	100751673	0.47	1.00	0.39	0.00	0.17	0.15	3.04	6.41	2.48	0.00	1.03	0.93
prolactin-like	LOC100 751675	100751675	0.00	0.00	0.00	0.00	0.00	0.00	0.00	0.00	0.00	0.00	0.00	0.00
olfactory receptor 1537-like	LOC100 751683	100751683	0.00	0.00	0.00	0.00	0.00	0.00	0.00	0.00	0.00	0.00	0.00	0.00
leucine-rich repeat-containing protein 9-like	LOC100 751685	100751685	0.00	0.00	0.00	0.00	0.00	0.00	0.00	0.00	0.00	0.00	0.00	0.00
ferritin light chain 1 pseudogene	LOC100 751687	100751687	0.00	0.00	0.00	0.00	0.00	0.00	0.00	0.00	0.00	0.00	0.00	0.00
60S ribosomal protein L5 pseudogene	LOC100 751690	100751690	0.24	0.12	0.17	0.16	0.05	0.05	6.08	2.75	3.73	3.48	1.03	0.93
caspase-3-like	LOC100 751691	100751691	0.04	0.00	0.00	0.00	0.00	0.00	0.76	0.00	0.00	1.16	1.03	0.00
60S ribosomal protein L17 pseudogene	LOC100 751693	100751693	0.46	0.49	0.24	0.50	0.51	0.03	3.80	5.49	2.48	3.48	5.17	0.00
histone-binding protein RBBP4 pseudogene	LOC100 751697	100751697	0.23	0.38	0.06	0.14	0.23	0.20	12.91	17.39	3.73	8.11	12.42	12.15
secreted and transmembrane protein 1b-like	LOC100 751701	100751701	0.00	0.00	0.00	0.00	0.00	0.00	0.00	0.00	0.00	0.00	0.00	0.00
coiled-coil domain-containing protein 162	LOC100 751702	100751702	0.11	0.07	0.02	0.06	0.02	0.08	18.99	20.13	17.39	10.43	17.59	13.09
guanine nucleotide-binding protein G(I)/G(S)/G(T) subunit beta-1 pseudogene	LOC100 751704	100751704	0.00	0.00	0.00	0.00	0.00	0.00	0.00	0.00	0.00	0.00	0.00	0.00
uncharacterized LOC100751705	LOC100 751705	100751705	0.00	0.03	0.00	0.00	0.00	0.00	37.98	30.20	12.42	3.48	1.03	5.61
paired mesoderm homeobox protein 2B	LOC100 751707	100751707	0.00	0.00	0.00	0.00	0.00	0.00	0.00	0.00	0.00	0.00	0.00	0.00
vomeronasal type-1 receptor 4-like	LOC100 751709	100751709	0.00	0.00	0.00	0.00	0.00	0.00	0.00	0.00	0.00	0.00	0.00	0.00

tetratricopeptide repeat protein 1-like	LOC100 751710	100751710	0.14	0.11	0.12	0.05	0.10	0.13	3.04	1.83	3.73	1.16	1.03	3.74
chromosome unknown open reading frame, human C15orf43	LOC100 751713	100751713	0.00	0.00	0.00	0.00	0.00	0.00	0.00	0.00	1.24	0.00	0.00	0.00
nuclear receptor subfamily 1 group D member 1-like	LOC100 751718	100751718	18.11	17.84	16.50	15.79	11.68	12.90	194.44	227.84	195.01	193.54	120.06	134.59
histone H2A type 1	LOC100 751726	100751726	0.31	0.08	0.04	0.00	0.07	0.00	4.56	0.00	0.00	0.00	2.07	0.00
DNA (cytosine-5)-methyltransferase 3B	LOC100 751727	100751727	0.00	0.00	0.00	0.00	0.00	0.00	0.00	0.00	0.00	0.00	0.00	0.00
olfactory receptor 2A12-like	LOC100 751730	100751730	0.00	0.00	0.00	0.00	0.00	0.00	0.00	0.00	0.00	0.00	0.00	0.00
60S acidic ribosomal protein P1 pseudogene	LOC100 751731	100751731	3.42	4.54	3.41	2.42	4.35	1.73	4.56	2.75	2.48	0.00	1.03	0.00
taste receptor type 2 member 16	LOC100 751741	100751741	0.00	0.00	0.00	0.00	0.00	0.00	0.00	0.00	0.00	0.00	0.00	0.00
RING finger protein 113A	LOC100 751742	100751742	15.22	15.83	17.28	14.31	14.01	14.55	577.25	602.09	663.27	541.22	531.98	555.19
olfactory receptor 5I1	LOC100 751743	100751743	0.00	0.00	0.00	0.00	0.00	0.00	0.00	0.00	0.00	0.00	0.00	0.00
uncharacterized LOC100751747	LOC100 751747	100751747	0.16	0.07	0.16	0.18	0.06	0.00	3.80	3.66	3.73	6.95	1.03	1.87
ADP/ATP translocase 1 pseudogene	LOC100 751751	100751751	0.00	0.00	0.00	0.00	0.00	0.00	0.00	0.00	0.00	0.00	0.00	0.00
dynactin subunit 5 pseudogene	LOC100 751754	100751754	0.28	0.77	0.23	0.45	0.39	0.98	3.04	8.24	2.48	4.64	4.14	10.28
aflatoxin B1 aldehyde reductase member 2	LOC100 751755	100751755	24.77	24.37	30.44	25.57	13.04	13.89	847.65	867.45	1085.58	892.37	468.85	475.75
probable inactive serine protease 58	LOC100 751756	100751756	0.00	0.00	0.00	0.00	0.00	0.00	0.00	0.00	0.00	0.00	0.00	0.00
uncharacterized LOC100751758	LOC100 751758	100751758	0.00	0.00	0.00	0.00	0.00	0.00	0.00	0.00	0.00	0.00	0.00	0.00
cytochrome P450 2C23-like	LOC100 751762	100751762	0.00	0.00	0.00	0.00	0.00	0.00	0.00	0.00	0.00	0.00	0.00	0.00
olfactory receptor 187-like	LOC100 751765	100751765	0.00	0.00	0.00	0.00	0.00	0.00	0.00	0.00	0.00	0.00	0.00	0.00

cytochrome P450 3A31-like	LOC100751769	100751769	0.03	0.00	0.05	0.14	0.21	0.11	2.28	1.83	1.24	11.59	13.45	9.35
paired immunoglobulin-like type 2 receptor beta	LOC100751773	100751773	0.00	0.00	0.00	0.00	0.00	0.00	0.00	0.00	0.00	0.00	0.00	0.00
intestinal-type alkaline phosphatase 1	LOC100751774	100751774	0.00	0.00	0.00	0.00	0.00	0.00	0.00	0.00	0.00	0.00	0.00	0.00
cytochrome P450 2C26	LOC100751778	100751778	0.00	0.00	0.00	0.00	0.00	0.00	0.00	0.00	0.00	0.00	0.00	0.00
keratin, type II cytoskeletal 6A	LOC100751786	100751786	0.02	0.04	0.00	0.00	0.00	0.00	0.76	1.83	0.00	0.00	0.00	0.00
uncharacterized LOC100751789	LOC100751789	100751789	2.75	2.77	1.26	1.09	1.49	1.84	45.57	42.09	19.87	11.59	26.91	23.37
chromosome unknown open reading frame, human C12orf49	LOC100751796	100751796	12.39	12.16	12.90	12.65	13.69	14.52	1822.89	1798.96	1922.75	1854.27	1988.21	2140.39
UDP-glucuronosyltransferase 2B31-like	LOC100751798	100751798	0.00	0.00	0.00	0.00	0.00	0.00	0.00	0.00	0.00	0.00	0.00	0.00
putative uncharacterized protein	LOC100751802	100751802	0.02	0.02	0.00	0.00	0.00	0.00	3.04	1.83	0.00	0.00	0.00	0.00
chromosome unknown open reading frame, human C1orf27	LOC100751804	100751804	10.91	11.11	10.74	13.19	13.73	13.02	988.92	1024.84	958.89	1222.66	1258.54	1162.73
olfactory receptor 5V1	LOC100751806	100751806	0.00	0.00	0.00	0.00	0.00	0.00	0.00	0.00	0.00	0.00	0.00	0.00
calmodulin-like	LOC100751814	100751814	22.42	21.14	19.57	21.31	16.31	22.28	0.00	0.00	0.00	0.00	0.00	0.00
speckle-type POZ protein-like	LOC100751817	100751817	0.00	0.00	0.00	0.00	0.00	0.00	0.00	0.00	0.00	0.00	0.00	0.00
leukocyte immunoglobulin-like receptor subfamily B member 3	LOC100751818	100751818	0.00	0.00	0.00	0.00	0.00	0.00	0.00	0.92	0.00	0.00	0.00	0.00
tetratricopeptide repeat protein 30B	LOC100751819	100751819	3.85	4.33	4.02	4.09	4.48	4.64	234.70	261.70	243.45	244.53	259.78	278.53
olfactory receptor 2J3-like	LOC100751826	100751826	0.00	0.00	0.00	0.00	0.00	0.00	0.00	0.00	0.00	0.00	0.00	0.00
olfactory receptor 7E24-like	LOC100751834	100751834	0.00	0.00	0.00	0.00	0.00	0.00	0.00	0.00	0.00	0.00	0.00	0.00
uncharacterized LOC100751837	LOC100751837	100751837	12.66	12.83	13.24	13.14	15.39	15.44	781.57	789.67	817.29	813.56	948.05	958.04

olfactory receptor 6K3	LOC100 751839	100751839	0.00	0.00	0.00	0.00	0.00	0.00	0.00	0.00	0.00	0.00	0.00	0.00
polyadenylate-binding protein 1	LOC100 751841	100751841	0.00	0.00	0.00	0.00	0.00	0.02	0.00	0.00	0.00	0.00	0.00	0.93
small nuclear ribonucleoprotein-associated protein N-like	LOC100 751844	100751844	0.00	0.00	0.00	0.00	0.00	0.00	0.00	0.00	0.00	1.16	0.00	0.00
SUMO-conjugating enzyme UBC9-B pseudogene	LOC100 751845	100751845	35.15	33.63	45.74	48.86	55.94	57.04	395.72	384.31	495.59	489.06	571.31	576.69
cytochrome c, somatic pseudogene	LOC100 751857	100751857	140.88	139.58	141.07	123.68	126.93	124.75	751.18	732.94	725.38	622.34	639.62	611.27
olfactory receptor 1I1	LOC100 751862	100751862	0.00	0.00	0.00	0.00	0.00	0.00	0.00	0.00	0.00	0.00	0.00	0.00
60S ribosomal protein L9 pseudogene	LOC100 751869	100751869	7.52	5.69	4.86	3.59	4.59	4.78	164.06	124.44	108.06	71.85	90.04	97.21
olfactory receptor 11G2	LOC100 751879	100751879	0.00	0.00	0.00	0.00	0.00	0.00	0.00	0.00	0.00	0.00	0.00	0.00
vomeronasal type-1 receptor 4-like	LOC100 751881	100751881	0.00	0.00	0.00	0.00	0.00	0.00	0.00	0.00	0.00	0.00	0.00	0.00
mortality factor 4-like protein 1 pseudogene	LOC100 751883	100751883	4.96	6.32	3.71	2.50	2.81	1.62	69.12	85.10	43.47	32.45	35.19	21.50
androglobin-like	LOC100 751887	100751887	0.00	0.00	0.00	0.00	0.00	0.00	0.76	0.00	0.00	0.00	0.00	0.93
eukaryotic initiation factor 4A-III-like	LOC100 751889	100751889	0.00	0.00	0.00	0.00	0.00	0.00	0.00	0.00	0.00	0.00	0.00	0.00
putative RNA exonuclease NEF-sp	LOC100 751890	100751890	4.31	4.23	5.20	5.18	4.95	4.77	369.90	361.44	428.52	439.23	424.34	421.54
60S ribosomal protein L10-like	LOC100 751893	100751893	0.00	0.00	0.00	0.00	0.00	0.00	0.00	0.00	0.00	0.00	0.00	0.00
nicotinamide riboside kinase 1	LOC100 751894	100751894	8.32	8.75	8.07	8.05	11.56	10.83	231.66	237.91	208.67	194.70	260.82	236.47
olfactory receptor 4K1	LOC100 751895	100751895	0.00	0.00	0.00	0.00	0.00	0.00	0.00	0.00	0.00	0.00	0.00	0.00
sialic acid-binding Ig-like lectin 5	LOC100 751896	100751896	0.00	0.00	0.00	0.00	0.00	0.00	0.00	0.00	0.00	0.00	0.00	0.00
olfactory receptor 52B2	LOC100 751899	100751899	0.00	0.00	0.00	0.05	0.05	0.08	0.00	0.00	0.00	1.16	1.03	1.87

sepiapterin reductase-like	LOC100751901	100751901	2.35	2.00	2.62	0.87	1.63	2.61	24.31	27.45	42.23	25.50	34.15	42.06
olfactory receptor 14J1-like	LOC100751903	100751903	0.00	0.00	0.00	0.00	0.00	0.00	0.00	0.00	0.00	0.00	0.00	0.00
alcohol sulfotransferase A-like	LOC100751913	100751913	0.00	0.00	0.00	0.00	0.00	0.00	0.00	0.00	0.00	0.00	1.03	0.00
zinc finger protein 431-like	LOC100751918	100751918	0.00	0.00	0.00	0.00	0.00	0.00	0.00	0.00	0.00	0.00	0.00	0.00
60S ribosomal protein L23a pseudogene	LOC100751923	100751923	10.01	13.19	11.57	7.84	9.78	5.54	80.51	95.16	91.91	59.10	70.38	43.93
granzyme-like protein 2	LOC100751926	100751926	0.00	0.00	0.00	0.00	0.00	0.00	0.00	0.00	0.00	0.00	0.00	0.00
olfactory receptor 7E24-like	LOC100751931	100751931	0.00	0.00	0.00	0.00	0.00	0.00	0.00	0.00	0.00	0.00	0.00	0.00
40S ribosomal protein S18 pseudogene	LOC100751935	100751935	0.65	1.23	0.73	0.85	0.62	0.17	31.14	46.67	32.29	11.59	5.17	0.93
serpin B4-like	LOC100751936	100751936	0.00	0.00	0.00	0.00	0.00	0.00	0.00	1.83	0.00	0.00	0.00	0.00
olfactory receptor 1044	LOC100751946	100751946	0.00	0.00	0.00	0.00	0.00	0.00	0.00	0.00	0.00	0.00	0.00	0.00
probable G-protein coupled receptor 83	LOC100751949	100751949	0.02	0.00	0.00	0.00	0.00	0.00	5.32	1.83	0.00	1.16	1.03	2.80
translationally-controlled tumor protein pseudogene	LOC100751950	100751950	3.47	2.33	2.68	2.18	1.87	1.60	46.33	37.52	38.50	28.97	22.77	17.76
left-right determination factor 2	LOC100751951	100751951	0.08	0.02	0.00	0.00	0.00	0.00	2.28	0.00	0.00	0.00	1.03	0.00
DNA-directed RNA polymerase III subunit RPC10 pseudogene	LOC100751952	100751952	0.65	0.59	0.46	0.27	0.38	0.28	2.28	2.75	0.00	1.16	1.03	0.93
HLA class I histocompatibility antigen, A-36 alpha chain-like	LOC100751953	100751953	0.04	0.00	0.02	0.00	0.00	0.00	0.76	0.92	0.00	0.00	0.00	0.93
60S ribosomal protein L11-like	LOC100751955	100751955	0.13	0.16	0.33	0.11	0.05	0.00	2.28	1.83	3.73	1.16	0.00	0.00
prolactin-6A1-like	LOC100751961	100751961	0.00	0.00	0.00	0.00	0.00	0.00	0.00	0.00	0.00	0.00	0.00	0.00
60S ribosomal protein L30-like	LOC100751966	100751966	14.20	17.19	17.83	23.23	17.92	16.92	80.51	81.44	79.49	107.78	84.87	84.12

olfactory receptor 150-like	LOC100 751968	100751968	0.00	0.00	0.00	0.00	0.00	0.00	0.00	0.00	0.00	0.00	0.00	0.00
non-structural maintenance of chromosomes element 1 homolog pseudogene	LOC100 751970	100751970	0.00	0.00	0.00	0.00	0.00	0.00	0.00	0.00	0.00	0.00	0.00	0.00
olfactory receptor 2AG1	LOC100 751973	100751973	0.00	0.00	0.00	0.00	0.00	0.00	0.00	0.00	0.00	0.00	0.00	0.00
tudor and KH domain-containing protein-like	LOC100 751975	100751975	0.00	0.02	0.00	0.09	0.00	0.02	0.00	0.92	0.00	3.48	0.00	0.93
eukaryotic translation initiation factor 4E pseudogene	LOC100 751976	100751976	0.19	0.47	0.00	0.26	0.15	0.10	2.28	5.49	0.00	3.48	2.07	1.87
60 kDa heat shock protein, mitochondrial-like	LOC100 751980	100751980	0.33	0.40	0.22	0.21	0.81	0.11	8.35	9.15	2.48	4.64	13.45	6.54
keratin-associated protein 6-3-like	LOC100 751981	100751981	0.00	0.00	0.00	0.00	0.00	0.00	0.00	0.00	0.00	0.00	0.00	0.00
mortality factor 4-like protein 2	LOC100 751982	100751982	0.00	0.00	0.00	0.00	0.00	0.00	0.00	0.00	0.00	0.00	0.00	0.00
secreted and transmembrane protein 1A-like	LOC100 751987	100751987	0.00	0.00	0.00	0.00	0.00	0.00	0.00	0.00	0.00	0.00	0.00	0.00
vomeronasal type-1 receptor 4-like	LOC100 751996	100751996	0.00	0.00	0.00	0.00	0.00	0.00	0.00	0.00	0.00	0.00	0.00	0.00
60S ribosomal protein L30-like	LOC100 752001	100752001	0.61	0.13	0.09	0.17	0.00	0.13	3.80	0.92	0.00	1.16	0.00	0.93
histone H2B subacrosomal variant	LOC100 752003	100752003	0.00	0.00	0.00	0.00	0.00	0.00	0.00	0.00	0.00	0.00	0.00	0.00
PI-PLC X domain-containing protein 1-like	LOC100 752008	100752008	0.00	0.00	0.00	0.00	0.00	0.00	0.00	1.83	0.00	0.00	1.03	0.93
histone H2B type 1	LOC100 752010	100752010	54.85	54.31	54.55	32.72	39.53	37.56	1019.30	1007.45	1012.30	616.55	770.03	736.52
olfactory receptor 2A12-like	LOC100 752013	100752013	0.00	0.00	0.00	0.00	0.00	0.00	0.00	0.00	0.00	0.00	0.00	0.00
protein slowmo homolog 2 pseudogene	LOC100 752025	100752025	0.00	0.00	0.00	0.00	0.00	0.00	0.76	0.00	1.24	0.00	0.00	0.00
olfactory receptor 5W2-like	LOC100 752029	100752029	0.00	0.00	0.00	0.00	0.00	0.00	0.00	0.00	0.00	0.00	0.00	0.00
trypsin V-A-like	LOC100 752043	100752043	0.00	0.00	0.00	0.00	0.00	0.00	0.00	0.00	0.00	0.00	0.00	0.00

uncharacterized LOC100752045	LOC100 752045	100752045	0.00	0.00	0.00	0.00	0.00	0.00	0.00	0.00	0.00	0.00	0.00	0.00
ubiquitin carboxyl-terminal hydrolase 21-like	LOC100 752049	100752049	0.00	0.00	0.03	0.00	0.02	0.02	3.80	0.00	3.73	0.00	1.03	0.93
olfactory receptor 5K3-like	LOC100 752051	100752051	0.00	0.00	0.00	0.00	0.00	0.00	0.00	0.00	0.00	0.00	0.00	0.00
peptidyl-prolyl cis-trans isomerase A-like	LOC100 752053	100752053	84.96	84.91	73.69	59.64	66.67	54.00	386.61	270.85	218.61	134.43	139.72	170.11
trace amine-associated receptor 7a	LOC100 752062	100752062	0.00	0.00	0.00	0.00	0.00	0.00	0.00	0.00	0.00	0.00	0.00	0.00
cytochrome P450 2C26	LOC100 752064	100752064	0.00	0.00	0.00	0.00	0.00	0.00	0.00	0.00	0.00	0.00	0.00	0.00
40S ribosomal protein S29	LOC100 752071	100752071	2515.49	2608.66	2430.73	1315.30	962.71	936.74	8592.67	8731.25	7815.21	3906.72	3035.61	2913.37
heterogeneous nuclear ribonucleoproteins A2/B1 pseudogene	LOC100 752073	100752073	0.00	0.00	0.00	0.00	0.00	0.00	0.00	0.00	0.00	0.00	0.00	0.00
opioid growth factor receptor pseudogene	LOC100 752086	100752086	0.41	0.70	0.33	0.09	0.08	0.25	6.08	11.90	4.97	1.16	1.03	3.74
60S ribosomal protein L27 pseudogene	LOC100 752087	100752087	0.00	0.00	0.00	0.00	0.00	0.00	0.00	0.00	0.00	0.00	0.00	0.00
Ig heavy chain V-II region SESS-like	LOC100 752095	100752095	0.00	0.00	0.00	0.00	0.00	0.00						
olfactory receptor 2G3	LOC100 752097	100752097	0.00	0.00	0.00	0.00	0.00	0.00	0.00	0.00	0.00	0.00	0.00	0.00
TD and POZ domain-containing protein 5-like	LOC100 752100	100752100	0.00	0.00	0.00	0.00	0.00	0.00	0.00	0.00	0.00	0.00	0.00	0.00
LIM domain transcription factor LMO4	LOC100 752101	100752101	12.93	11.82	4.91	3.39	2.41	2.21	93.42	78.69	34.78	25.50	20.70	14.95
Krueppel-like factor 10 pseudogene	LOC100 752104	100752104	0.03	0.03	0.00	0.07	0.06	0.00	0.76	0.92	0.00	2.32	2.07	0.00
uncharacterized LOC100752107	LOC100 752107	100752107	1.18	1.08	0.00	0.13	0.32	0.30	36.46	32.03	0.00	3.48	9.31	9.35
apolipoprotein L6-like	LOC100 752109	100752109	0.00	0.00	0.00	0.00	0.00	0.00	0.00	0.00	0.00	0.00	0.00	0.00
non-histone chromosomal protein HMG-14 pseudogene	LOC100 752110	100752110	0.00	0.00	0.00	0.00	0.00	0.00	0.00	0.00	0.00	0.00	0.00	0.00

probable UDP-sugar transporter protein SLC35A4	LOC100752111	100752111	4.46	4.60	5.68	4.44	4.01	3.67	65.32	61.31	85.70	59.10	57.96	53.28
chromosome unknown open reading frame, human CXorf23	LOC100752113	100752113	11.36	11.51	12.76	13.12	15.27	14.01	811.19	841.83	907.97	936.41	1114.68	1043.09
olfactory receptor 7E24-like	LOC100752114	100752114	0.00	0.00	0.06	0.00	0.00	0.00	0.00	0.00	1.24	0.00	0.00	0.00
histone H3.3C-like	LOC100752116	100752116	0.61	0.97	1.01	0.74	1.07	0.71	12.15	11.90	14.91	9.27	9.31	4.67
chromosome unknown open reading frame, human C6orf118	LOC100752122	100752122	0.23	0.12	0.13	0.15	0.18	0.12	11.39	7.32	7.45	6.95	10.35	9.35
anaphase-promoting complex subunit 15-like	LOC100752128	100752128	0.00	0.00	0.00	0.00	0.00	0.00	0.00	0.00	0.00	0.00	0.00	0.00
glutathione S-transferase A2	LOC100752137	100752137	0.44	0.92	0.20	0.19	0.39	0.40	9.11	21.05	4.97	4.64	7.24	10.28
olfactory receptor 2T1	LOC100752140	100752140	0.00	0.00	0.00	0.00	0.00	0.00	0.00	0.00	0.00	0.00	0.00	0.00
60S ribosomal protein L15 pseudogene	LOC100752142	100752142	73.25	73.59	67.43	58.93	68.82	56.95	50.13	68.63	45.96	35.93	58.99	28.97
ATP synthase subunit g, mitochondrial pseudogene	LOC100752151	100752151	0.00	0.00	0.00	0.00	0.00	0.26	0.00	0.00	0.00	0.00	0.00	0.93
chromosome unknown open reading frame, human C11orf82	LOC100752154	100752154	1.45	1.62	1.39	1.50	1.80	1.58	225.58	252.55	217.37	231.78	276.34	247.69
olfactory receptor 11G2	LOC100752163	100752163	0.00	0.00	0.00	0.00	0.00	0.00	0.00	0.00	0.00	0.00	0.00	0.00
vomeronasal type-1 receptor 4-like	LOC100752165	100752165	0.00	0.00	0.00	0.00	0.00	0.00	0.00	0.00	0.00	0.00	0.00	0.00
peroxisomal biogenesis factor 3-like	LOC100752166	100752166	0.21	0.40	0.32	0.30	0.44	0.26	5.32	10.07	4.97	5.79	10.35	7.48
chromosome unknown open reading frame, human C19orf12	LOC100752167	100752167	0.00	0.00	0.00	0.00	0.00	0.00	0.00	0.00	0.00	0.00	0.00	0.00
ubiquitin-conjugating enzyme E2 D4 pseudogene	LOC100752171	100752171	0.67	0.57	0.41	0.31	0.23	0.14	5.32	7.32	2.48	5.79	4.14	2.80
zinc finger protein 728-like	LOC100752174	100752174	0.08	0.13	0.11	0.13	0.23	0.44	6.84	9.15	7.45	6.95	16.56	29.91
UV radiation resistance-associated gene protein pseudogene	LOC100752177	100752177	0.06	0.10	0.11	0.02	0.04	0.08	3.04	5.49	4.97	1.16	2.07	3.74

olfactory receptor 4K15	LOC100752180	100752180	0.00	0.00	0.00	0.00	0.00	0.00	0.00	0.00	0.00	0.00	0.00	0.00
olfactory receptor 52W1	LOC100752183	100752183	0.00	0.04	0.00	0.00	0.04	0.04	0.00	0.92	0.00	0.00	1.03	0.93
sperm motility kinase X-like	LOC100752185	100752185	0.08	0.05	0.14	0.17	0.22	0.08	2.28	0.92	3.73	3.48	7.24	1.87
putative zinc finger protein 724	LOC100752187	100752187	0.77	0.85	1.00	0.81	1.22	0.71	44.05	49.41	55.89	42.88	77.62	45.80
ferritin heavy chain pseudogene	LOC100752189	100752189	0.44	0.45	0.49	0.00	0.10	0.56	4.56	4.58	4.97	0.00	1.03	5.61
kelch repeat and BTB domain-containing protein 6-like	LOC100752191	100752191	3.94	4.61	5.97	5.74	9.08	7.88	167.10	205.88	247.18	232.94	367.42	313.11
protein archease pseudogene	LOC100752194	100752194	0.08	0.00	0.00	0.00	0.11	0.00	1.52	0.00	0.00	0.00	1.03	0.00
	LOC100752199	100752199	37.19	37.41	38.59	38.02	36.28	40.10	1840.36	1873.07	1925.23	1887.88	1796.74	1976.83
small RNA 2'-O-methyltransferase	LOC100752202	100752202	1.42	1.07	1.13	1.75	1.73	1.59	41.02	40.26	38.50	49.83	47.61	49.54
60S ribosomal protein L21 pseudogene	LOC100752203	100752203	0.53	0.40	0.50	0.11	0.56	0.25	3.04	2.75	2.48	0.00	2.07	0.93
phospholipase A2-like	LOC100752205	100752205	0.00	0.00	0.00	0.00	0.00	0.00	0.00	0.00	0.00	0.00	0.00	0.00
60S ribosomal protein L15-like	LOC100752208	100752208	1.09	0.74	0.46	0.26	0.54	0.49	12.91	10.98	7.45	4.64	10.35	7.48
TP53-regulated inhibitor of apoptosis 1 pseudogene	LOC100752209	100752209	0.00	0.00	0.00	0.00	0.00	0.00	0.00	0.00	0.00	0.00	0.00	0.00
chromatin target of PRMT1 protein pseudogene	LOC100752210	100752210	0.07	0.09	0.00	0.00	0.00	0.00	0.76	0.92	0.00	0.00	0.00	0.00
NADH-cytochrome b5 reductase 1	LOC100752211	100752211	152.22	149.70	175.12	173.07	187.30	190.60	6706.73	6570.86	7707.15	7487.78	8116.36	8308.28
olfactory receptor 8B3-like	LOC100752212	100752212	0.00	0.00	0.00	0.00	0.00	0.00	0.00	0.00	0.00	0.00	0.00	0.00
vomeronasal type-2 receptor 116-like	LOC100752215	100752215	0.00	0.00	0.00	0.00	0.00	0.00	0.00	0.00	0.00	0.00	0.00	0.00
zinc finger and BTB domain-containing protein 6-like	LOC100752220	100752220	0.53	0.62	0.65	0.54	0.78	0.49	25.82	27.45	37.26	34.77	36.22	27.11

olfactory receptor 8K5-like	LOC100752232	100752232	0.00	0.00	0.00	0.00	0.00	0.00	0.00	0.00	0.00	0.00	0.00	0.00
HLA class I histocompatibility antigen, A-36 alpha chain-like	LOC100752239	100752239	0.00	0.00	0.00	0.00	0.00	0.00	0.00	0.00	0.00	0.00	0.00	0.00
N-acetyllactosaminide alpha-1,3-galactosyltransferase-like	LOC100752240	100752240	0.00	0.00	0.00	0.00	0.00	0.00	0.00	0.00	0.00	0.00	0.00	0.00
glyceraldehyde-3-phosphate dehydrogenase pseudogene	LOC100752241	100752241	1.82	1.70	1.61	0.73	1.10	0.78	28.10	25.62	26.08	5.79	18.63	8.41
heat shock factor protein 3	LOC100752243	100752243	0.14	0.12	0.05	0.09	0.09	0.37	15.19	9.15	7.45	11.59	9.31	12.15
serine hydrolase-like protein	LOC100752244	100752244	12.85	14.06	24.01	26.10	25.15	24.25	452.69	525.23	838.41	878.46	829.02	829.99
prolactin-3D4-like	LOC100752249	100752249	0.00	0.00	0.00	0.00	0.00	0.00	0.00	0.00	0.00	0.00	0.00	0.00
chromosome unknown open reading frame, human C16orf54	LOC100752250	100752250	0.00	0.00	0.00	0.00	0.00	0.00	0.00	0.00	0.00	0.00	0.00	0.00
olfactory receptor 150-like	LOC100752255	100752255	0.00	0.00	0.00	0.00	0.00	0.00	0.00	0.00	0.00	0.00	0.00	0.00
dehydrogenase/reductase SDR family member 7-like	LOC100752257	100752257	0.30	0.15	0.28	0.07	0.35	0.18	0.00	0.00	0.00	0.00	0.00	0.00
ubiquitin-associated protein 2-like pseudogene	LOC100752259	100752259	2.12	1.87	1.82	1.69	1.58	1.77	168.62	150.07	141.60	135.59	126.27	143.94
olfactory receptor 2AG1-like	LOC100752260	100752260	0.00	0.00	0.00	0.00	0.00	0.00	0.00	0.00	0.00	0.00	0.00	0.00
40S ribosomal protein S21 pseudogene	LOC100752263	100752263	0.00	0.00	0.00	0.00	0.00	0.00	0.00	0.00	0.00	0.00	0.00	0.00
olfactory receptor 12	LOC100752264	100752264	0.00	0.00	0.00	0.00	0.00	0.00	0.00	0.00	0.00	0.00	0.00	0.00
calcium/calmodulin-dependent protein kinase type II delta chain	LOC100752268	100752268	21.47	23.54	32.97	37.54	43.40	41.10	877.27	920.52	1315.37	1471.83	1696.34	1540.34
carbonyl reductase [NADPH] 1-like	LOC100752270	100752270	0.23	0.33	0.23	0.13	0.30	0.17	0.00	0.00	0.00	0.00	0.00	0.00
protein PBDC1 pseudogene	LOC100752274	100752274	0.32	0.39	0.16	0.00	0.27	0.12	3.04	4.58	1.24	0.00	3.10	0.93
epithelial chloride channel protein-like	LOC100752277	100752277	0.02	0.03	0.01	0.01	0.02	0.01	1.52	2.75	0.00	1.16	2.07	0.93

testis-expressed protein 19.2-like	LOC100752279	100752279	0.00	0.00	0.00	0.00	0.04	0.00	0.00	0.00	0.00	0.00	1.03	0.00
ADP-ribosylation factor-like protein 6-interacting protein 1	LOC100752286	100752286	64.35	62.98	60.53	67.43	67.29	68.67	4087.08	4029.81	3906.36	4284.53	4244.48	4366.78
vomeronasal type-1 receptor 4-like	LOC100752288	100752288	0.00	0.00	0.00	0.00	0.00	0.00	0.00	0.00	0.00	0.00	0.00	0.00
adenosylhomocysteinase pseudogene	LOC100752289	100752289	0.00	0.03	0.00	0.00	0.00	0.00	1.52	2.75	0.00	0.00	0.00	0.00
tyrosinase	LOC100752294	100752294	0.00	0.00	0.00	0.00	0.00	0.00	0.00	0.00	0.00	0.00	0.00	0.00
PRAME family member 12-like	LOC100752297	100752297	0.00	0.00	0.00	0.00	0.00	0.00	0.00	0.00	0.00	0.00	0.00	0.00
histone H1t	LOC100752306	100752306	0.00	0.00	0.00	0.00	0.00	0.00	0.00	0.00	0.00	0.00	0.00	0.00
olfactory receptor 2A12-like	LOC100752309	100752309	0.00	0.00	0.00	0.00	0.00	0.00	0.00	0.00	0.00	0.00	0.00	0.00
60S ribosomal protein L23a	LOC100752312	100752312	6.38	6.20	6.12	4.24	4.98	3.87	10.63	13.73	18.63	10.43	8.28	4.67
sperm motility kinase Z	LOC100752318	100752318	0.00	0.00	0.00	0.00	0.00	0.00	0.00	0.92	0.00	0.00	0.00	0.00
cytochrome c oxidase subunit 7C, mitochondrial pseudogene	LOC100752319	100752319	0.00	0.00	0.00	0.38	0.64	0.00	1.52	2.75	1.24	3.48	3.10	0.00
taste receptor type 2 member 143	LOC100752320	100752320	0.07	0.00	0.00	0.00	0.00	0.00	2.28	1.83	0.00	0.00	0.00	0.00
alanyl-tRNA editing protein Aarsd1	LOC100752322	100752322	56.90	51.16	52.99	47.68	41.02	45.21	2292.29	2141.18	2169.93	1922.65	1677.71	1786.15
olfactory receptor 5W2-like	LOC100752325	100752325	0.00	0.00	0.00	0.00	0.00	0.00	0.00	0.00	0.00	0.00	0.00	0.00
H-2 class II histocompatibility antigen, I-E alpha chain-like	LOC100752328	100752328	0.00	0.00	0.00	0.00	0.00	0.00	0.00	0.00	0.00	0.00	0.00	0.00
NADH dehydrogenase [ubiquinone] 1 alpha subcomplex assembly factor 4 pseudogene	LOC100752329	100752329	0.00	0.00	0.00	0.00	0.00	0.00	0.00	0.00	0.00	0.00	0.00	0.00
mediator of RNA polymerase II transcription subunit 28 pseudogene	LOC100752338	100752338	0.29	0.26	0.12	0.00	0.00	0.00	2.28	1.83	0.00	0.00	0.00	0.00
ubiquitin-related modifier 1 pseudogene	LOC100752339	100752339	0.83	1.13	0.71	1.06	0.59	0.83	3.04	3.66	2.48	3.48	2.07	2.80

ephrin type-A receptor 6	LOC100 752345	100752345	0.00	0.00	0.00	0.00	0.04	0.00	0.00	0.00	0.00	0.00	1.03	0.00
cleavage and polyadenylation specificity factor subunit 5-like	LOC100 752346	100752346	0.04	0.20	0.20	0.06	0.17	0.15	0.76	3.66	3.73	1.16	3.10	2.80
heterogeneous nuclear ribonucleoprotein A3 pseudogene	LOC100 752347	100752347	0.32	0.58	0.28	0.42	0.42	0.21	6.84	12.81	6.21	10.43	10.35	4.67
trace amine-associated receptor 7e-like	LOC100 752356	100752356	0.00	0.00	0.00	0.00	0.00	0.00	0.00	0.00	0.00	0.00	0.00	0.00
cytochrome P450 2C26-like	LOC100 752358	100752358	0.00	0.00	0.00	0.00	0.00	0.00	0.00	0.00	0.00	0.00	0.00	0.00
ribosomal RNA-processing protein 7 homolog A pseudogene	LOC100 752359	100752359	0.00	0.00	0.03	0.00	0.00	0.00	0.00	0.00	0.00	0.00	0.00	0.00
nodal modulator 1	LOC100 752363	100752363	156.62	149.62	164.83	157.94	145.39	163.13	9640.83	9319.62	10186.35	9609.76	8793.24	9828.99
uncharacterized LOC100752373	LOC100 752373	100752373	0.91	1.25	0.80	0.59	0.81	0.98	71.40	87.84	54.65	49.83	61.06	69.17
bile salt sulfotransferase 1-like	LOC100 752375	100752375	0.00	0.00	0.00	0.00	0.00	0.00	0.00	0.00	0.00	0.00	0.00	0.00
vomeronasal type-2 receptor 116-like	LOC100 752377	100752377	0.00	0.00	0.00	0.00	0.00	0.00	0.76	1.83	2.48	2.32	1.03	3.74
zinc finger protein 37-like	LOC100 752382	100752382	0.37	0.50	0.40	0.50	0.44	0.49	0.00	0.00	1.24	0.00	1.03	0.93
calmodulin-alpha-like	LOC100 752387	100752387	0.28	0.20	0.28	0.37	0.52	0.20	4.56	6.41	4.97	12.75	7.24	9.35
olfactory receptor 14J1-like	LOC100 752388	100752388	0.00	0.00	0.00	0.00	0.00	0.00	0.00	0.00	0.00	0.00	0.00	0.00
NACHT, LRR and PYD domains-containing protein 5-like	LOC100 752389	100752389	0.02	0.00	0.02	0.01	0.01	0.02	12.15	9.15	16.15	12.75	1.03	10.28
guanine nucleotide-binding protein G(I)/G(S)/G(O) subunit gamma-5 pseudogene	LOC100 752390	100752390	1.22	2.95	3.24	1.17	1.84	0.91	3.04	3.66	3.73	1.16	3.10	0.93
GRB2-associated-binding protein 2 pseudogene	LOC100 752391	100752391	0.00	0.00	0.00	0.00	0.00	0.00	0.00	0.00	0.00	0.00	0.00	0.00
40S ribosomal protein S15a pseudogene	LOC100 752393	100752393	4.35	2.70	2.96	0.86	2.07	2.20	26.58	14.64	17.39	4.64	11.38	14.02
methylcytosine dioxygenase TET2-like	LOC100 752395	100752395	0.00	0.00	0.01	0.00	0.00	0.00	0.00	0.00	1.24	0.00	0.00	0.00

40S ribosomal protein S28 pseudogene	LOC100752396	100752396	0.00	0.00	0.00	0.00	0.00	0.00	0.00	0.00	0.00	0.00	0.00	0.00
interferon-induced transmembrane protein 2	LOC100752397	100752397	115.36	103.46	113.73	119.92	94.60	113.88	1099.05	995.56	1105.46	1175.14	883.88	1123.47
[Pyruvate dehydrogenase [acetyl-transferring]]-phosphatase 2, mitochondrial-like	LOC100752399	100752399	0.76	0.46	0.75	0.39	0.75	0.61	2.28	0.92	0.00	0.00	2.07	0.93
hereditary hemochromatosis protein homolog	LOC100752402	100752402	0.00	0.00	0.00	0.00	0.00	0.00	0.00	0.00	0.00	0.00	0.00	0.00
osteoclast-stimulating factor 1 pseudogene	LOC100752406	100752406	0.00	0.00	0.00	0.00	0.00	0.00	0.00	0.00	0.00	0.00	0.00	0.00
putative gustatory receptor clone PTE01	LOC100752407	100752407	0.00	0.00	0.00	0.00	0.00	0.00	0.00	0.00	0.00	0.00	0.00	0.00
olfactory receptor 6K3	LOC100752412	100752412	0.00	0.00	0.00	0.00	0.00	0.00	0.00	0.00	0.00	0.00	0.00	0.00
quinone oxidoreductase-like protein 2	LOC100752419	100752419	11.84	11.10	9.41	11.06	10.67	10.35	968.41	910.46	780.03	890.05	852.83	834.66
golgin subfamily A member 7 pseudogene	LOC100752426	100752426	0.67	0.94	0.47	0.33	0.64	0.36	6.84	10.07	3.73	3.48	6.21	2.80
myeloblastin	LOC100752428	100752428	0.00	0.00	0.00	0.00	0.00	0.00	0.00	0.92	0.00	0.00	0.00	0.00
chromosome unknown open reading frame, human C11orf87	LOC100752429	100752429	0.00	0.00	0.00	0.00	0.00	0.00	0.76	0.00	0.00	0.00	0.00	0.00
liprin-alpha-2	LOC100752430	100752430	1.02	1.21	1.07	1.41	1.83	1.72	107.85	134.51	118.00	140.23	196.65	168.24
arginine--tRNA ligase, cytoplasmic-like	LOC100752442	100752442	0.08	0.04	0.00	0.07	0.00	0.02	3.80	1.83	0.00	3.48	0.00	0.93
actin-related protein 2/3 complex subunit 1A pseudogene	LOC100752447	100752447	0.09	0.14	0.09	0.13	0.04	0.04	4.56	3.66	3.73	3.48	1.03	0.93
olfactory receptor 11H4	LOC100752456	100752456	0.00	0.00	0.00	0.00	0.00	0.00	0.00	0.00	0.00	0.00	0.00	0.00
zinc finger protein 160-like	LOC100752458	100752458	0.00	0.00	0.00	0.00	0.00	0.00	0.00	0.00	0.00	0.00	0.00	0.00
peroxiredoxin-1 pseudogene	LOC100752460	100752460	0.88	0.37	0.41	0.68	0.60	0.54	10.63	3.66	4.97	8.11	7.24	6.54
60S ribosomal protein L11-like	LOC100752467	100752467	6.61	5.72	4.66	3.21	3.43	2.65	97.98	81.44	64.59	39.40	46.57	33.65

zinc finger protein 728-like	LOC100752468	100752468	0.97	1.05	0.89	1.11	1.27	1.18	195.20	222.35	172.65	230.63	251.50	247.69
olfactory receptor 4N5	LOC100752473	100752473	0.00	0.00	0.00	0.00	0.00	0.00	0.00	0.00	0.00	0.00	0.00	0.00
chromosome unknown open reading frame, human C11orf42	LOC100752476	100752476	0.09	0.00	0.00	0.10	0.13	0.12	3.80	2.75	2.48	4.64	5.17	2.80
protein transport protein Sec61 subunit gamma-like	LOC100752477	100752477	0.00	0.00	0.00	0.00	0.00	0.13	0.00	0.00	0.00	0.00	1.03	0.93
sperm motility kinase X-like	LOC100752478	100752478	0.03	0.02	0.05	0.08	0.05	0.05	0.76	0.00	3.73	4.64	5.17	2.80
carcinoembryonic antigen-related cell adhesion molecule 5-like	LOC100752480	100752480	0.00	0.00	0.00	0.00	0.00	0.00	0.00	0.00	0.00	0.00	0.00	0.00
acidic leucine-rich nuclear phosphoprotein 32 family member B pseudogene	LOC100752482	100752482	17.28	18.80	22.25	18.32	23.19	19.20	376.73	378.82	438.46	317.54	303.25	276.66
G/T mismatch-specific thymine DNA glycosylase pseudogene	LOC100752484	100752484	0.05	0.12	0.06	0.00	0.00	0.00	0.76	1.83	0.00	0.00	0.00	0.00
claudin-3-like	LOC100752491	100752491	0.00	0.00	0.00	0.00	0.00	0.00	0.00	0.00	0.00	0.00	0.00	0.00
eukaryotic translation initiation factor 3 subunit E pseudogene	LOC100752494	100752494	0.00	0.00	0.00	0.00	0.00	0.00	0.00	0.00	0.00	0.00	0.00	0.00
pre-mRNA-splicing factor 38B-like	LOC100752495	100752495	69.22	68.77	65.06	69.64	64.18	61.77	6149.99	6177.39	5877.55	6204.86	5720.37	5518.29
actin-like protein 9	LOC100752497	100752497	0.00	0.00	0.00	0.00	0.00	0.00	0.00	0.00	0.00	0.00	0.00	0.00
phospholipase A2, minor isoenzyme-like	LOC100752499	100752499	0.00	0.00	0.00	0.00	0.00	0.00	0.00	0.00	0.00	0.00	0.00	0.00
coiled-coil domain-containing protein 11 pseudogene	LOC100752501	100752501	0.00	0.00	0.00	0.00	0.00	0.00	0.00	0.00	0.00	0.00	0.00	0.00
prostatic spermine-binding protein-like	LOC100752503	100752503	0.00	0.00	0.00	0.00	0.00	0.00	0.00	0.00	0.00	0.00	0.00	0.00
spliceosome RNA helicase DDX39B-like	LOC100752504	100752504	42.06	41.33	55.30	52.05	44.33	46.03	1070.95	1072.42	1422.19	1372.16	1152.97	1217.87
vomeronasal type-2 receptor 116-like	LOC100752509	100752509	0.00	0.00	0.00	0.00	0.00	0.00	0.00	0.00	0.00	0.00	0.00	0.00
syntaxin-binding protein 1-like	LOC100752510	100752510	0.00	0.00	0.00	0.00	0.00	0.00	0.00	0.00	0.00	0.00	0.00	0.00

oxidation resistance protein 1 pseudogene	LOC100752518	100752518	0.00	0.00	0.00	0.00	0.00	0.00	0.00	0.00	0.00	0.00	0.00	0.00
olfactory receptor 1052	LOC100752526	100752526	0.00	0.00	0.00	0.00	0.00	0.00	0.00	0.00	0.00	0.00	0.00	0.00
putative heat shock protein HSP 90-alpha A2	LOC100752530	100752530	0.05	0.00	0.00	0.00	0.00	0.00	0.76	1.83	0.00	0.00	0.00	0.00
ubiquitin-like protein FUBI pseudogene	LOC100752532	100752532	0.00	0.00	0.00	0.00	0.00	0.00	0.00	0.00	0.00	0.00	0.00	0.00
H-2 class I histocompatibility antigen, Q10 alpha chain-like	LOC100752535	100752535	0.04	0.00	0.00	0.00	0.00	0.00	0.00	0.00	0.00	0.00	1.03	0.00
probable G-protein coupled receptor 83	LOC100752539	100752539	0.00	0.00	0.00	0.00	0.00	0.00	0.00	0.00	0.00	0.00	0.00	0.00
serine hydrolase-like protein	LOC100752540	100752540	1.47	1.27	2.89	2.48	2.74	2.16	40.26	42.09	78.25	67.22	87.97	71.03
prolactin-3D1-like	LOC100752545	100752545	0.00	0.00	0.00	0.00	0.00	0.00	0.00	0.00	0.00	0.00	0.00	0.00
histone H1.0	LOC100752547	100752547	396.94	430.34	433.82	413.15	358.54	362.16	14098.57	14989.16	15221.77	14537.49	12076.22	12660.10
olfactory receptor 1537-like	LOC100752550	100752550	0.00	0.00	0.00	0.00	0.00	0.00	0.00	0.00	0.00	0.00	0.00	0.00
60 kDa heat shock protein, mitochondrial-like	LOC100752551	100752551	0.23	0.13	0.03	0.06	0.11	0.09	8.35	4.58	1.24	2.32	3.10	2.80
dehydrogenase/reductase SDR family member 7	LOC100752553	100752553	29.55	30.18	29.37	30.38	38.02	37.85	982.08	1029.41	998.64	1024.49	1281.31	1252.46
60S ribosomal protein L23a pseudogene	LOC100752554	100752554	0.15	0.11	0.03	0.03	0.05	0.09	0.76	0.92	0.00	0.00	0.00	0.00
olfactory receptor 2D3	LOC100752557	100752557	0.00	0.00	0.00	0.00	0.00	0.00	0.00	0.00	0.00	0.00	0.00	0.00
olfactory receptor 12	LOC100752561	100752561	0.03	0.00	0.00	0.00	0.00	0.00	0.76	0.00	0.00	0.00	0.00	0.00
ATP-binding cassette sub-family F member 2	LOC100752562	100752562	0.89	0.99	1.06	0.79	0.60	0.84	9.87	13.73	17.39	10.43	12.42	7.48
keratin-associated protein 20-2-like	LOC100752567	100752567	0.00	0.00	0.00	0.00	0.00	0.00	0.00	0.00	0.00	0.00	0.00	0.00
serine/threonine-protein phosphatase 2B catalytic subunit alpha isoform	LOC100752571	100752571	68.35	68.01	73.10	85.09	102.73	94.99	3098.16	3188.89	3409.53	3903.24	4547.73	4387.34

epithelial chloride channel protein-like	LOC100 752576	100752576	0.03	0.00	0.03	0.00	0.00	0.02	2.28	2.75	2.48	2.32	1.03	2.80
transcription initiation factor TFIID subunit 9-like	LOC100 752577	100752577	0.59	0.14	0.13	0.12	0.44	0.30	5.32	0.92	1.24	1.16	4.14	2.80
F-box only protein 28 pseudogene	LOC100 752585	100752585	0.61	0.65	0.56	0.42	0.57	0.43	25.82	28.37	24.84	18.54	24.84	17.76
vomeronasal type-1 receptor 4-like	LOC100 752586	100752586	0.00	0.00	0.00	0.00	0.00	0.00	0.00	0.00	0.00	0.00	0.00	0.00
beclin-2-like	LOC100 752587	100752587	0.00	0.00	0.00	0.00	0.00	0.00	0.00	0.00	0.00	0.00	0.00	0.00
ribonuclease P protein subunit p20-like	LOC100 752588	100752588	0.00	0.00	0.00	0.00	0.00	0.00	0.00	0.00	0.00	0.00	0.00	0.00
nuclear transport factor 2 pseudogene	LOC100 752590	100752590	1.37	1.08	1.16	0.55	0.53	0.64	15.95	13.73	14.91	6.95	8.28	9.35
uncharacterized LOC100752592	LOC100 752592	100752592	1.38	0.50	1.86	1.82	2.15	1.44	20.51	11.90	24.84	23.18	28.98	13.09
vomeronasal type-1 receptor 3-like	LOC100 752594	100752594	0.00	0.00	0.00	0.00	0.00	0.00	0.00	0.00	0.00	0.00	0.00	0.00
endoplasmic reticulum-Golgi intermediate compartment protein 2	LOC100 752596	100752596	76.52	75.87	62.33	72.66	72.37	71.00	811.19	798.82	684.39	763.73	783.48	729.04
aquaporin-12	LOC100 752599	100752599	0.00	0.00	0.00	0.00	0.00	0.00	0.00	0.00	0.00	0.00	0.00	0.00
transcription initiation factor TFIID subunit 13-like	LOC100 752601	100752601	0.00	0.00	0.00	0.00	0.00	0.00	0.00	0.00	0.00	0.00	0.00	0.00
histone H4	LOC100 752605	100752605	0.57	0.14	0.00	0.27	0.08	0.21	7.60	4.58	1.24	3.48	2.07	2.80
cholesterol side-chain cleavage enzyme, mitochondrial	LOC100 752606	100752606	0.20	0.12	0.08	0.05	0.23	0.38	12.15	10.07	4.97	4.64	13.45	23.37
carbonic anhydrase 15	LOC100 752607	100752607	0.00	0.08	0.00	0.05	0.00	0.00	1.52	2.75	1.24	5.79	1.03	0.93
olfactory receptor 2A12-like	LOC100 752609	100752609	0.03	0.00	0.00	0.00	0.00	0.00	0.76	0.00	0.00	0.00	0.00	0.00
enhancer of rudimentary homolog pseudogene	LOC100 752612	100752612	4.36	4.81	4.07	2.54	2.96	3.21	9.87	9.15	7.45	4.64	2.07	9.35
HIG1 domain family member 1A, mitochondrial pseudogene	LOC100 752615	100752615	0.00	0.27	0.00	0.00	0.00	0.00	0.00	1.83	0.00	0.00	0.00	0.00

actin-related protein 2/3 complex subunit 1A-like	LOC100752617	100752617	0.49	0.32	1.10	1.44	0.03	0.00	22.79	15.56	73.28	84.60	1.03	0.00
taste receptor type 2 member 41	LOC100752619	100752619	0.00	0.00	0.00	0.00	0.00	0.00	0.00	0.00	0.00	0.00	0.00	0.00
ATP-dependent RNA helicase DDX50 pseudogene	LOC100752622	100752622	0.00	0.00	0.00	0.00	0.00	0.00	0.00	0.00	0.00	0.00	0.00	0.00
protein SET-like	LOC100752623	100752623	36.74	37.82	41.49	36.78	35.00	36.05	750.42	831.77	922.87	909.75	832.13	741.19
olfactory receptor 5W2-like	LOC100752624	100752624	0.00	0.00	0.00	0.00	0.00	0.00	0.00	0.00	0.00	0.00	0.00	0.00
H-2 class I histocompatibility antigen, Q9 alpha chain-like	LOC100752627	100752627	1.62	1.94	2.18	1.55	1.12	1.65	18.23	17.39	31.05	16.22	10.35	19.63
AKT-interacting protein pseudogene	LOC100752629	100752629	0.00	0.00	0.00	0.00	0.00	0.04	0.00	0.00	0.00	0.00	0.00	0.00
histone-lysine N-methyltransferase SUV39H1-like	LOC100752631	100752631	0.04	0.10	0.00	0.06	0.04	0.00	1.52	4.58	0.00	2.32	1.03	0.00
neuroblastoma suppressor of tumorigenicity 1	LOC100752634	100752634	60.56	59.59	42.67	38.01	37.27	40.23	2811.05	2778.04	1992.31	1756.92	1706.69	1865.60
trypsin-4-like	LOC100752635	100752635	0.00	0.00	0.00	0.00	0.00	0.00	0.00	0.00	0.00	0.00	0.00	0.00
protein Shroom2-like	LOC100752637	100752637	0.00	0.00	0.00	0.00	0.00	0.00	0.00	0.00	0.00	0.00	0.00	0.00
olfactory receptor 5AU1	LOC100752638	100752638	0.00	0.00	0.00	0.00	0.00	0.00	0.00	0.00	0.00	0.00	0.00	0.00
hsc70-interacting protein pseudogene	LOC100752641	100752641	11.41	10.65	11.66	11.48	12.91	11.36	365.34	337.65	380.08	366.22	406.75	389.76
spindlin-2-like	LOC100752647	100752647	0.00	0.00	0.00	0.00	0.00	0.00	0.00	0.00	0.00	0.00	0.00	0.00
chromosome unknown open reading frame, human C11orf16	LOC100752651	100752651	0.00	0.00	0.00	0.00	0.00	0.00	0.00	0.00	0.00	0.00	0.00	0.00
transient receptor potential cation channel subfamily M member 7 pseudogene	LOC100752654	100752654	0.00	0.00	0.00	0.00	0.00	0.00	9.11	6.41	0.00	3.48	4.14	5.61
trace amine-associated receptor 7a-like	LOC100752657	100752657	0.00	0.00	0.00	0.00	0.00	0.00	0.00	0.00	0.00	0.00	0.00	0.00
ribosomal RNA-processing protein 7 homolog A pseudogene	LOC100752660	100752660	0.00	0.00	0.04	0.00	0.00	0.00	0.00	0.00	0.00	0.00	0.00	0.00

40S ribosomal protein S12 pseudogene	LOC100 752662	100752662	0.00	0.00	0.00	0.00	0.00	0.00	0.00	0.00	0.00	0.00	0.00	0.00
heat shock protein HSP 90-alpha-like	LOC100 752664	100752664	0.29	0.26	0.22	0.07	0.23	0.08	16.71	13.73	12.42	3.48	13.45	4.67
coiled-coil-helix-coiled-coil-helix domain-containing protein 2, mitochondrial-like	LOC100 752670	100752670	0.77	0.06	0.53	0.22	0.16	0.29	4.56	0.00	3.73	1.16	0.00	1.87
60S ribosomal protein L36a-like	LOC100 752672	100752672	0.00	0.00	0.00	0.00	0.00	0.00	0.00	0.00	0.00	0.00	0.00	0.00
zinc finger protein 431-like	LOC100 752674	100752674	9.91	10.60	10.79	12.82	14.42	12.12	715.49	754.90	772.58	914.39	1024.64	867.37
serine/threonine-protein kinase VRK1 pseudogene	LOC100 752676	100752676	0.00	0.00	0.00	0.00	0.00	0.00	0.00	0.00	0.00	0.00	0.00	0.00
protein FAM84A-like	LOC100 752679	100752679	0.00	0.00	0.00	0.00	0.00	0.00	0.00	0.00	0.00	0.00	0.00	0.00
zinc finger CCCH domain-containing protein 15-like	LOC100 752681	100752681	0.00	0.00	0.00	0.04	0.00	0.00	0.76	1.83	3.73	2.32	1.03	0.00
chromosome unknown open reading frame, human C10orf118	LOC100 752682	100752682	13.73	13.67	15.02	16.89	15.83	15.09	1292.74	1260.00	1417.22	1588.88	1461.40	1421.63
major urinary protein 5-like	LOC100 752684	100752684	0.00	0.00	0.00	0.00	0.00	0.00	0.00	0.00	0.00	0.00	0.00	0.00
40S ribosomal protein S20 pseudogene	LOC100 752690	100752690	0.00	0.00	0.00	0.00	0.00	0.00	0.00	0.00	0.00	0.00	0.00	0.00
tripartite motif-containing protein 30A-like	LOC100 752691	100752691	32.37	35.21	41.97	44.89	51.06	47.88	873.47	921.44	1102.97	1184.42	1382.74	1221.61
tripartite motif-containing protein 6 pseudogene	LOC100 752697	100752697	0.01	0.00	0.08	0.00	0.05	0.02	0.00	0.00	0.00	0.00	0.00	0.00
heterogeneous nuclear ribonucleoprotein A3 pseudogene	LOC100 752698	100752698	0.36	0.75	0.51	0.47	0.42	0.26	6.84	18.30	12.42	9.27	8.28	8.41
chromosome unknown open reading frame, human C1orf56	LOC100 752700	100752700	0.05	0.00	0.04	0.07	0.00	0.03	2.28	0.92	1.24	3.48	1.03	2.80
chromosome unknown open reading frame, human C14orf79	LOC100 752702	100752702	1.97	1.97	1.90	2.03	1.90	2.23	84.31	79.61	78.25	84.60	76.59	90.66
eukaryotic translation initiation factor 3 subunit J-like	LOC100 752704	100752704	0.00	0.05	0.00	0.00	0.00	0.00	0.76	0.92	0.00	0.00	0.00	0.00
microtubule-associated serine/threonine-protein kinase 4	LOC100 752705	100752705	1.02	0.96	0.87	0.93	1.06	1.07	269.64	252.55	239.72	242.21	289.80	283.20

60S ribosomal protein L35a pseudogene	LOC100752707	100752707	0.10	0.00	0.00	0.00	0.07	0.13	3.04	0.00	2.48	0.00	2.07	2.80
putative gustatory receptor clone PTE01	LOC100752711	100752711	0.00	0.00	0.00	0.00	0.00	0.00	0.00	0.00	0.00	0.00	0.00	0.00
ATP synthase subunit d, mitochondrial pseudogene	LOC100752712	100752712	0.00	0.00	0.00	0.00	0.00	0.00	0.00	0.00	0.00	0.00	0.00	0.00
60S ribosomal protein L5 pseudogene	LOC100752713	100752713	6.39	4.28	3.44	1.81	3.50	2.25	129.88	86.01	70.80	34.77	66.24	41.13
L-aminoadipate-semialdehyde dehydrogenase-phosphopantetheinyl transferase-like	LOC100752715	100752715	0.44	0.63	0.44	0.19	0.54	0.43	6.84	10.98	6.21	2.32	10.35	6.54
olfactory receptor 6K2	LOC100752716	100752716	0.00	0.00	0.00	0.00	0.00	0.00	0.00	0.00	0.00	0.00	0.00	0.00
cyclin-D-binding Myb-like transcription factor 1 pseudogene	LOC100752730	100752730	0.03	0.00	0.00	0.00	0.04	0.00	1.52	0.00	0.00	0.00	2.07	0.00
chromosome unknown open reading frame, human C12orf29	LOC100752737	100752737	13.55	13.49	13.06	14.74	17.96	16.78	752.70	752.16	716.68	775.32	907.68	842.14
olfactory receptor 10T2-like	LOC100752743	100752743	0.00	0.00	0.00	0.00	0.00	0.00	0.00	0.00	0.00	0.00	0.00	0.00
histone-lysine N-methyltransferase PRDM9-like	LOC100752747	100752747	0.42	0.37	0.20	0.43	0.37	0.20	24.31	24.71	12.42	28.97	21.73	15.89
60S ribosomal protein L26 pseudogene	LOC100752748	100752748	0.00	0.00	0.00	0.00	0.00	0.00	0.76	1.83	1.24	1.16	1.03	0.00
nucleolin-like	LOC100752758	100752758	9.88	10.54	8.50	9.77	9.34	7.91	951.70	1002.88	827.23	773.00	629.27	567.34
proteasome subunit alpha type-7 pseudogene	LOC100752759	100752759	0.00	0.00	0.00	0.00	0.00	0.00	0.00	0.00	0.00	0.00	0.00	0.00
olfactory receptor 11H7	LOC100752760	100752760	0.00	0.00	0.00	0.00	0.00	0.00	0.00	0.00	0.00	0.00	0.00	0.00
serine/threonine-protein kinase MARK2 pseudogene	LOC100752762	100752762	0.00	0.02	0.03	0.00	0.00	0.02	0.00	0.92	1.24	0.00	0.00	0.93
zinc finger protein 420-like	LOC100752763	100752763	0.48	0.59	0.52	0.95	0.96	0.67	28.86	35.69	29.81	50.99	50.71	40.19
WD repeat domain phosphoinositide-interacting protein 2 pseudogene	LOC100752764	100752764	1.22	1.39	1.59	1.16	1.21	0.89	17.47	14.64	22.36	11.59	18.63	12.15
eukaryotic initiation factor 4A-III pseudogene	LOC100752770	100752770	0.06	0.03	0.00	0.00	0.04	0.03	1.52	0.92	0.00	0.00	1.03	0.93

olfactory receptor 4K5-like	LOC100752775	100752775	0.00	0.00	0.00	0.00	0.00	0.00	0.00	0.00	0.00	0.00	0.00	0.00
dystrophin	LOC100752779	100752779	3.88	4.34	5.52	6.03	6.74	6.93	104.06	101.57	126.69	139.07	157.32	152.35
sepiapterin reductase-like	LOC100752780	100752780	0.07	0.17	0.08	0.19	0.23	0.39	2.28	4.58	3.73	4.64	6.21	8.41
carcinoembryonic antigen-related cell adhesion molecule 5-like	LOC100752782	100752782	0.00	0.00	0.00	0.00	0.00	0.00	0.00	0.00	0.00	0.00	0.00	0.00
mortality factor 4-like protein 1 pseudogene	LOC100752784	100752784	0.17	0.04	0.08	0.05	0.19	0.08	6.08	0.92	1.24	1.16	4.14	2.80
Krueppel-like factor 17	LOC100752787	100752787	0.00	0.00	0.00	0.00	0.00	0.00	0.00	0.00	0.00	0.00	0.00	0.00
histone-binding protein RBBP4 pseudogene	LOC100752790	100752790	0.03	0.13	0.07	0.03	0.12	0.03	0.76	3.66	1.24	0.00	4.14	0.93
zinc finger CCCH domain-containing protein 15 pseudogene	LOC100752793	100752793	0.86	0.87	0.43	0.36	0.88	0.69	14.43	10.98	6.21	4.64	11.38	8.41
60S acidic ribosomal protein P2 pseudogene	LOC100752794	100752794	0.00	0.00	0.00	0.00	0.00	0.00	1.52	0.00	0.00	1.16	0.00	0.00
high mobility group protein B1 pseudogene	LOC100752796	100752796	0.00	0.00	0.00	0.00	0.00	0.00	0.00	0.00	0.00	0.00	0.00	0.00
zinc finger protein 420-like	LOC100752799	100752799	1.83	1.71	1.88	2.43	2.50	2.36	170.14	161.05	181.34	221.35	227.70	220.58
IQ and ubiquitin-like domain-containing protein	LOC100752802	100752802	0.00	0.00	0.00	0.00	0.00	0.00	0.00	0.00	0.00	0.00	0.00	0.00
interferon-induced guanylate-binding protein 1-like	LOC100752806	100752806	0.00	0.01	0.00	0.00	0.00	0.00	0.00	0.00	0.00	0.00	0.00	0.00
olfactory receptor 143-like	LOC100752809	100752809	0.00	0.00	0.00	0.00	0.00	0.00	0.00	0.00	0.00	0.00	0.00	0.00
mitochondrial pyruvate carrier 1 pseudogene	LOC100752810	100752810	0.00	0.00	0.00	0.72	0.37	0.00	3.04	0.92	0.00	2.32	1.03	0.00
zinc finger protein 354B-like	LOC100752811	100752811	0.08	0.00	0.00	0.00	0.16	0.00	6.08	10.07	8.69	4.64	4.14	2.80
BBSome-interacting protein 1-like	LOC100752818	100752818	1.17	0.00	0.51	0.00	0.43	0.41	6.84	1.83	4.97	6.95	1.03	3.74
40S ribosomal protein S15a-like	LOC100752821	100752821	0.00	0.00	0.00	0.00	0.00	0.00	0.76	0.00	0.00	0.00	0.00	0.00

1-acyl-sn-glycerol-3-phosphate acyltransferase alpha-like	LOC100752827	100752827	0.00	0.00	0.00	0.00	0.00	0.17	0.00	0.00	0.00	0.00	0.00	1.87
olfactory receptor 8J3	LOC100752829	100752829	0.00	0.00	0.00	0.00	0.00	0.00	0.00	0.00	0.00	0.00	0.00	0.00
guanylate cyclase soluble subunit beta-2	LOC100752832	100752832	0.03	0.02	0.00	0.00	0.00	0.00	1.52	1.83	0.00	0.00	0.00	0.00
putative uncharacterized protein ZNRD1-AS1 homolog	LOC100752838	100752838	1.77	2.00	1.52	1.57	2.28	2.33	69.88	82.35	67.07	61.42	100.39	87.86
eukaryotic translation initiation factor 4B pseudogene	LOC100752841	100752841	0.00	0.00	0.00	0.00	0.00	0.00	0.00	0.00	0.00	0.00	0.00	0.00
40S ribosomal protein S28 pseudogene	LOC100752843	100752843	0.00	0.00	0.00	0.00	0.00	0.00	0.00	0.00	0.00	0.00	0.00	0.00
prolactin-3C1-like	LOC100752847	100752847	0.00	0.00	0.00	0.00	0.00	0.00	0.00	0.00	0.00	0.00	0.00	0.00
schlafen family member 12-like	LOC100752852	100752852	9.35	9.38	6.07	7.18	7.70	6.93	1276.79	1313.07	821.02	963.06	1023.60	974.86
olfactory receptor 146-like	LOC100752853	100752853	0.00	0.00	0.00	0.00	0.00	0.00	0.00	0.00	0.00	0.00	0.00	0.00
NADH dehydrogenase [ubiquinone] 1 alpha subcomplex subunit 13 pseudogene	LOC100752855	100752855	0.00	0.00	0.00	0.00	0.00	0.00	0.00	0.00	0.00	0.00	0.00	0.00
uncharacterized LOC100752857	LOC100752857	100752857	3.78	3.59	6.31	7.47	8.03	7.89	177.73	173.86	294.37	349.99	367.42	369.19
olfactory receptor 10C1-like	LOC100752862	100752862	0.00	0.00	0.00	0.00	0.00	0.00	0.00	0.00	0.00	0.00	0.00	0.00
alcohol sulfotransferase A-like	LOC100752864	100752864	0.00	0.00	0.00	0.00	0.00	0.00	0.00	0.00	0.00	0.00	0.00	0.00
39S ribosomal protein L15, mitochondrial pseudogene	LOC100752865	100752865	0.00	0.00	0.00	0.00	0.00	0.00	0.00	0.00	0.00	0.00	0.00	0.00
chromosome unknown open reading frame, human C3orf43	LOC100752866	100752866	0.00	0.00	0.27	0.00	0.00	0.07	0.00	0.92	3.73	1.16	0.00	0.93
keratin-associated protein 20-2-like	LOC100752868	100752868	0.00	0.00	0.00	0.00	0.00	0.00	0.00	0.00	0.00	0.00	0.00	0.00
carbonyl reductase [NADPH] 1-like	LOC100752869	100752869	7.54	6.50	6.41	4.82	5.18	5.35	145.07	110.72	113.03	96.19	104.53	96.27
chromosome unknown open reading frame, human C17orf80	LOC100752871	100752871	11.78	11.53	11.58	11.93	11.77	11.90	690.42	667.97	696.81	709.26	699.65	697.26

UPF0762 protein C6orf58-like	LOC100752877	100752877	0.00	0.00	0.00	0.00	0.00	0.00	0.00	0.00	0.00	0.00	0.00	0.00
mitogen-activated protein kinase 6-like	LOC100752878	100752878	0.00	0.00	0.00	0.02	0.02	0.00	0.00	0.00	0.00	1.16	2.07	0.00
glyceraldehyde-3-phosphate dehydrogenase-like	LOC100752882	100752882	0.00	0.00	0.00	0.00	0.00	0.00	0.00	0.00	0.00	0.00	0.00	0.00
39S ribosomal protein L15, mitochondrial pseudogene	LOC100752885	100752885	0.21	0.28	0.06	0.06	0.05	0.05	5.32	5.49	2.48	1.16	1.03	0.93
vomeronasal type-1 receptor 4-like	LOC100752886	100752886	0.00	0.00	0.00	0.00	0.00	0.00	0.00	0.00	0.00	0.00	0.00	0.00
serine protease 55-like	LOC100752889	100752889	0.00	0.00	0.00	0.00	0.00	0.00	0.00	0.00	0.00	0.00	0.00	0.00
protein slowmo homolog 2-like	LOC100752892	100752892	1.08	1.02	0.85	0.76	0.62	0.76	2.28	0.92	0.00	1.16	1.03	0.00
FGFR1 oncogene partner 2 homolog	LOC100752894	100752894	0.29	0.29	0.16	0.00	0.13	0.31	6.08	6.41	7.45	3.48	3.10	8.41
probable hydrolase PNKD	LOC100752898	100752898	21.47	21.34	21.75	21.17	16.54	18.57	2.28	2.75	3.73	2.32	6.21	2.80
40S ribosomal protein S27	LOC100752901	100752901	14.77	13.83	12.03	7.84	14.88	12.19	35.70	40.26	24.84	20.86	31.05	36.45
histone H1.2	LOC100752904	100752904	8.73	9.48	7.64	7.25	5.02	6.09	324.32	327.58	265.81	249.17	181.12	218.71
olfactory receptor 2A12-like	LOC100752908	100752908	0.00	0.00	0.00	0.00	0.00	0.00	0.00	0.00	0.00	0.00	0.00	0.00
oligosaccharyltransferase complex subunit OSTC pseudogene	LOC100752915	100752915	0.00	0.00	0.17	0.00	0.00	0.00	0.00	0.00	2.48	0.00	0.00	0.00
60S ribosomal protein L32 pseudogene	LOC100752916	100752916	0.00	0.00	0.00	0.00	0.00	0.00	0.00	0.00	0.00	0.00	0.00	0.00
putative olfactory receptor 10AC1	LOC100752918	100752918	0.00	0.00	0.00	0.00	0.00	0.00	0.00	0.00	0.00	0.00	0.00	0.00
olfactory receptor 5W2-like	LOC100752923	100752923	0.00	0.00	0.00	0.00	0.00	0.00	0.00	0.00	0.00	0.00	0.00	0.00
elongation of very long chain fatty acids protein 6-like	LOC100752924	100752924	0.04	0.00	0.00	0.06	0.00	0.00	1.52	0.92	0.00	2.32	0.00	0.00
H-2 class I histocompatibility antigen, Q10 alpha chain-like	LOC100752927	100752927	0.61	0.34	0.30	0.09	0.08	0.04	15.95	9.15	8.69	3.48	1.03	1.87

60S acidic ribosomal protein P1-like	LOC100752929	100752929	0.00	0.00	0.00	0.00	0.00	0.00	0.00	0.00	0.00	0.00	0.00	0.00
uncharacterized LOC100752930	LOC100752930	100752930	1535.83	1470.60	1398.48	870.05	639.95	656.57	15799.93	15679.10	14946.03	9300.33	7665.11	7295.10
zinc finger protein 431-like	LOC100752931	100752931	0.00	0.00	0.00	0.00	0.00	0.00	0.00	0.00	0.00	0.00	0.00	0.00
cationic trypsin-3-like	LOC100752935	100752935	0.00	0.00	0.00	0.00	0.00	0.00	0.00	0.00	0.00	0.00	0.00	0.00
uncharacterized LOC100752936	LOC100752936	100752936	0.00	0.00	0.00	0.00	0.00	0.00	0.76	0.00	0.00	0.00	0.00	0.00
chromosome unknown open reading frame, human C15orf57	LOC100752939	100752939	16.60	16.36	21.34	21.49	20.54	21.82	655.48	642.35	814.81	815.88	805.22	845.88
proteasome subunit beta type-5 pseudogene	LOC100752941	100752941	4.97	5.42	4.83	2.83	3.16	3.08	21.27	21.05	18.63	9.27	12.42	7.48
zinc finger protein 25-like	LOC100752942	100752942	2.14	2.34	2.31	3.35	3.55	3.02	148.11	171.11	171.41	235.26	250.47	218.71
zinc finger protein 431-like	LOC100752946	100752946	2.87	2.80	3.33	3.80	4.35	3.51	263.56	260.78	314.25	368.54	402.61	355.17
spindlin-2-like	LOC100752947	100752947	0.00	0.00	0.00	0.00	0.00	0.00	0.00	0.00	0.00	0.00	0.00	0.00
cytochrome c1, heme protein, mitochondrial pseudogene	LOC100752952	100752952	0.00	0.00	0.00	0.00	0.00	0.00	0.00	0.00	0.00	0.00	0.00	0.00
trace amine-associated receptor 7e	LOC100752958	100752958	0.00	0.00	0.00	0.00	0.00	0.00	0.00	0.00	0.00	0.00	0.00	0.00
chromosome unknown open reading frame, human C7orf57	LOC100752964	100752964	0.00	0.00	0.00	0.00	0.00	0.00	0.76	0.92	0.00	0.00	0.00	0.00
uncharacterized protein KIAA1109	LOC100752965	100752965	8.39	8.39	7.79	10.16	10.87	10.24	3060.94	3066.28	2838.17	3716.66	3925.70	3715.31
chromosome unknown open reading frame, human C11orf95	LOC100752967	100752967	2.92	2.52	2.09	1.32	0.47	0.73	152.67	124.44	106.82	77.65	31.05	38.32
synaptosomal-associated protein 23 pseudogene	LOC100752969	100752969	0.91	0.68	0.41	0.41	0.85	0.39	3.04	3.66	0.00	0.00	2.07	0.00
UPF0568 protein C14orf166 homolog pseudogene	LOC100752973	100752973	0.00	0.00	0.00	0.00	0.00	0.00	0.00	0.00	0.00	0.00	0.00	0.00
olfactory receptor 51A7-like	LOC100752976	100752976	0.00	0.00	0.00	0.00	0.00	0.00	0.00	0.00	0.00	0.00	0.00	0.00

transmembrane protein 126A pseudogene	LOC100752977	100752977	0.00	0.06	0.00	0.00	0.00	0.00	0.00	0.92	0.00	0.00	1.03	0.00
regulator of nonsense transcripts 3B pseudogene	LOC100752978	100752978	1.24	1.87	1.29	1.03	1.30	0.63	11.39	14.64	6.21	5.79	9.31	4.67
vomeronasal type-2 receptor 116-like	LOC100752980	100752980	0.00	0.00	0.00	0.00	0.00	0.00	0.00	0.00	0.00	0.00	0.00	0.00
cathepsin 7	LOC100752983	100752983	0.00	0.00	0.00	0.00	0.00	0.00	0.00	0.00	0.00	0.00	0.00	0.00
high mobility group protein B3 pseudogene	LOC100752984	100752984	0.00	0.00	0.00	0.00	0.00	0.00	0.00	0.00	0.00	0.00	0.00	0.00
carbonic anhydrase 7 pseudogene	LOC100752987	100752987	0.00	0.00	0.00	0.00	0.00	0.00	0.00	0.00	0.00	0.00	0.00	0.00
60S ribosomal protein L8 pseudogene	LOC100752989	100752989	8.73	8.82	8.03	5.91	8.99	6.28	125.32	134.51	121.72	86.92	131.44	86.92
slit homolog 2 protein	LOC100752994	100752994	0.00	0.03	0.00	0.00	0.00	0.00	0.76	1.83	0.00	0.00	0.00	0.00
ubiquitin-conjugating enzyme E2 variant 1 pseudogene	LOC100752996	100752996	0.00	0.00	0.00	0.00	0.00	0.00	0.00	0.00	0.00	0.00	0.00	0.93
gamma-aminobutyric acid receptor-associated protein pseudogene	LOC100752998	100752998	0.10	0.00	0.00	0.00	0.27	0.24	0.76	0.00	0.00	0.00	2.07	1.87
proto-oncogene FRAT1-like	LOC100753006	100753006	0.05	0.06	0.08	0.08	0.00	0.00	4.56	2.75	3.73	4.64	5.17	0.93
lamina-associated polypeptide 2, isoforms beta/gamma pseudogene	LOC100753009	100753009	0.00	0.00	0.00	0.00	0.00	0.00	0.00	0.00	0.00	0.00	0.00	0.00
olfactory receptor 867-like	LOC100753011	100753011	0.00	0.00	0.00	0.00	0.00	0.04	0.00	0.00	0.00	0.00	0.00	0.93
uncharacterized LOC100753012	LOC100753012	100753012	0.58	0.55	0.63	0.77	0.09	0.00	21.27	22.88	27.33	27.81	5.17	0.93
chromosome unknown open reading frame, human C21orf91	LOC100753013	100753013	16.16	16.23	15.72	18.08	19.27	17.61	1014.74	1053.20	997.40	1134.58	1189.20	1073.94
SOSS complex subunit B2 pseudogene	LOC100753014	100753014	0.00	0.00	0.00	0.00	0.00	0.15	0.00	0.00	0.00	0.00	0.00	1.87
protein FAM156A/FAM156B	LOC100753015	100753015	0.00	0.00	0.00	0.00	0.00	0.00	0.00	0.00	0.00	0.00	0.00	0.00
intelectin-1a-like	LOC100753016	100753016	0.02	0.00	0.00	0.00	0.00	0.00	0.00	0.00	0.00	0.00	0.00	0.00

heterogeneous nuclear ribonucleoproteins C1/C2-like	LOC100 753025	100753025	0.96	1.31	0.76	0.91	1.18	0.40	18.99	27.45	14.91	19.70	24.84	7.48
40S ribosomal protein S3a pseudogene	LOC100 753035	100753035	0.00	0.00	0.00	0.00	0.00	0.06	0.00	0.00	0.00	0.00	0.00	0.93
peptidyl-prolyl cis-trans isomerase A-like	LOC100 753042	100753042	0.16	0.19	0.00	0.00	0.00	0.20	1.52	1.83	0.00	0.00	0.00	1.87
olfactory receptor 10T2-like	LOC100 753043	100753043	0.00	0.00	0.00	0.00	0.00	0.00	0.76	0.00	0.00	0.00	0.00	0.00
glyceraldehyde-3-phosphate dehydrogenase-like	LOC100 753044	100753044	0.00	0.00	0.00	0.00	0.00	0.00	0.00	0.00	0.00	0.00	0.00	0.00
chromosome unknown open reading frame, human C18orf42	LOC100 753051	100753051	0.00	0.11	0.00	0.00	0.00	0.00	0.00	0.92	1.24	1.16	0.00	1.87
serologically defined colon cancer antigen 8 homolog	LOC100 753059	100753059	15.89	15.22	14.53	16.18	16.60	15.78	1621.62	1556.47	1565.03	1678.12	1704.62	1629.13
elongation factor 1-alpha 1-like	LOC100 753063	100753063	5.78	5.30	4.84	4.28	6.54	5.38	143.55	164.71	149.05	154.14	225.63	161.70
peptidyl-tRNA hydrolase 2, mitochondrial pseudogene	LOC100 753070	100753070	0.05	0.12	0.08	0.00	0.07	0.00	0.76	1.83	1.24	0.00	1.03	0.00
zinc finger protein 728-like	LOC100 753071	100753071	1.10	1.10	1.23	1.33	1.41	1.32	192.16	188.50	209.91	238.74	234.94	233.67
serine/threonine-protein phosphatase 2A 56 kDa regulatory subunit gamma isoform pseudogene	LOC100 753076	100753076	0.53	0.74	0.48	0.56	0.51	0.76	18.23	26.54	13.66	22.02	15.52	23.37
olfactory receptor 4K15-like	LOC100 753077	100753077	0.00	0.00	0.00	0.00	0.00	0.00	0.00	0.00	0.00	0.00	0.00	0.00
T-complex protein 1 subunit eta pseudogene	LOC100 753082	100753082	23.64	21.59	21.88	21.26	18.01	21.50	801.31	740.26	747.74	701.15	567.17	684.18
carcinoembryonic antigen-related cell adhesion molecule 3-like	LOC100 753084	100753084	0.00	0.00	0.00	0.00	0.00	0.00	0.00	0.00	0.00	0.00	0.00	0.00
uncharacterized protein C2orf78 homolog	LOC100 753093	100753093	0.00	0.00	0.00	0.00	0.00	0.00	0.00	0.00	0.00	0.00	0.00	1.87
60S ribosomal protein L7a-like	LOC100 753097	100753097	0.16	0.09	0.16	0.04	0.03	0.00	6.84	3.66	4.97	3.48	1.03	1.87
induced myeloid leukemia cell differentiation protein Mcl-1 homolog	LOC100 753098	100753098	0.87	1.23	0.76	0.49	0.92	0.31	31.90	44.84	19.87	13.91	18.63	6.54
actin-related protein 2/3 complex subunit 5-like protein pseudogene	LOC100 753099	100753099	0.16	0.20	0.14	0.00	0.11	0.10	7.60	5.49	3.73	1.16	1.03	1.87

chromosome unknown open reading frame, human C15orf38	LOC100753100	100753100	24.18	28.81	27.59	28.33	28.55	27.93	309.13	346.80	346.54	353.47	358.11	343.02
TGF-beta-activated kinase 1 and MAP3K7-binding protein 2 pseudogene	LOC100753102	100753102	0.04	0.02	0.00	0.03	0.03	0.00	1.52	0.00	0.00	0.00	1.03	0.00
hereditary hemochromatosis protein homolog	LOC100753110	100753110	0.00	0.00	0.00	0.00	0.00	0.00	0.00	0.00	0.00	0.00	0.00	0.00
uncharacterized LOC100753111	LOC100753111	100753111	0.00	0.00	0.00	0.00	0.00	0.06	0.00	0.00	0.00	0.00	0.00	0.00
zinc finger protein 525-like	LOC100753112	100753112	7.30	7.74	3.86	4.26	4.30	3.95	268.12	276.34	150.29	157.61	152.14	143.00
WW domain binding protein 1-like	LOC100753114	100753114	0.00	0.00	0.00	0.00	0.03	0.00	0.76	0.00	0.00	0.00	2.07	0.93
phosphatidylethanolamine-binding protein 1-like	LOC100753117	100753117	0.00	0.00	0.00	0.00	0.00	0.00	0.00	0.00	0.00	0.00	0.00	0.00
eukaryotic translation initiation factor 2 subunit 1 pseudogene	LOC100753118	100753118	0.00	0.00	0.00	0.05	0.04	0.00	0.00	0.00	0.00	1.16	1.03	0.00
uncharacterized LOC100753122	LOC100753122	100753122	0.00	0.00	0.00	0.00	0.00	0.00	0.76	0.92	2.48	2.32	2.07	0.93
phosphatidylinositol 3,4,5-trisphosphate 3-phosphatase TPTE2-like	LOC100753123	100753123	0.00	0.00	0.00	0.00	0.00	0.00	0.00	0.00	0.00	0.00	0.00	0.93
olfactory receptor 8K3	LOC100753130	100753130	0.00	0.00	0.00	0.00	0.00	0.00	0.00	0.00	0.00	0.00	0.00	0.00
chromosome unknown open reading frame, human C11orf54	LOC100753132	100753132	2.15	2.10	2.00	2.54	2.77	2.68	121.53	112.55	103.09	132.12	145.93	141.14
40S ribosomal protein S6 pseudogene	LOC100753134	100753134	0.00	0.00	0.00	0.11	0.00	0.00	0.76	0.00	0.00	1.16	0.00	0.00
10 kDa heat shock protein, mitochondrial	LOC100753142	100753142	129.18	125.74	116.35	104.27	97.76	104.91	1070.95	994.64	927.84	760.25	594.08	683.24
cytochrome c oxidase subunit 6C-2 pseudogene	LOC100753143	100753143	0.00	0.00	0.00	0.00	0.00	0.00	0.00	0.00	0.00	0.00	0.00	0.00
schlafen family member 12-like	LOC100753152	100753152	0.06	0.05	0.02	0.00	0.02	0.00	5.32	3.66	1.24	0.00	1.03	0.00
olfactory receptor 8D2-like	LOC100753153	100753153	0.00	0.00	0.00	0.00	0.00	0.00	0.00	0.00	0.00	0.00	0.00	0.00
aconitate hydratase, mitochondrial pseudogene	LOC100753154	100753154	3.32	3.49	3.41	2.94	3.19	3.35	50.89	40.26	28.57	32.45	31.05	42.06

cardiotrophin-2	LOC100 753155	100753155	0.02	0.04	0.02	0.05	0.02	0.04	1.52	1.83	2.48	3.48	1.03	1.87
60S ribosomal protein L7a pseudogene	LOC100 753156	100753156	0.00	0.00	0.07	0.00	0.06	0.00	0.00	2.75	1.24	0.00	2.07	0.00
olfactory receptor 2AP1-like	LOC100 753162	100753162	0.00	0.00	0.00	0.00	0.00	0.00	0.00	0.00	0.00	0.00	0.00	0.00
bile salt sulfotransferase 1-like	LOC100 753164	100753164	0.00	0.00	0.00	0.00	0.00	0.00	0.00	0.00	0.00	0.00	0.00	0.00
thiosulfate sulfurtransferase/rhodanese-like domain-containing protein 3 pseudogene	LOC100 753167	100753167	0.07	0.09	0.00	0.12	0.10	0.09	2.28	1.83	0.00	2.32	1.03	0.93
histone deacetylase 1 pseudogene	LOC100 753168	100753168	0.14	0.12	0.01	0.10	0.07	0.06	1.52	2.75	0.00	2.32	2.07	0.93
POU domain, class 5, transcription factor 1 pseudogene	LOC100 753170	100753170	0.00	0.00	0.00	0.00	0.00	0.00	0.00	0.00	0.00	0.00	0.00	0.00
glutathione S-transferase alpha-5	LOC100 753172	100753172	0.06	0.07	0.00	0.00	0.04	0.05	1.52	2.75	0.00	0.00	2.07	0.93
40S ribosomal protein S18-like	LOC100 753180	100753180	0.00	0.12	0.16	0.00	0.00	0.00	0.00	0.92	1.24	0.00	0.00	0.00
olfactory receptor 287	LOC100 753182	100753182	0.00	0.00	0.00	0.00	0.00	0.00	0.00	0.00	0.00	0.00	1.03	0.00
uncharacterized LOC100753183	LOC100 753183	100753183	0.54	0.75	0.62	0.48	0.67	0.60	34.18	40.26	42.23	33.61	40.36	37.39
prohibitin pseudogene	LOC100 753185	100753185	0.79	0.87	0.08	0.30	0.20	0.12	12.15	11.90	1.24	3.48	3.10	1.87
N-acylglucosamine 2-epimerase-like	LOC100 753186	100753186	0.00	0.00	0.00	0.00	0.00	0.00	0.00	0.00	0.00	0.00	0.00	0.00
serine protease 55	LOC100 753188	100753188	0.00	0.00	0.00	0.00	0.00	0.00	0.00	0.00	0.00	0.00	0.00	0.00
multifunctional protein ADE2-like	LOC100 753190	100753190	2.61	2.58	1.61	1.52	2.21	1.78	66.84	72.29	38.50	38.24	56.92	43.93
LIM zinc-binding domain-containing Nebulette	LOC100 753199	100753199	0.00	0.00	0.00	0.00	0.00	0.00	0.00	0.00	0.00	0.00	0.00	0.00
histone H3	LOC100 753203	100753203	0.08	0.28	0.00	0.00	0.00	0.00	1.52	3.66	0.00	0.00	0.00	0.00
keratin, type II cytoskeletal 8 pseudogene	LOC100 753205	100753205	0.00	0.00	0.00	0.00	0.00	0.00	0.00	0.00	0.00	0.00	0.00	0.00

L-amino-acid oxidase-like	LOC100753207	100753207	0.00	0.00	0.00	0.00	0.00	0.00	0.00	0.00	0.00	0.00	0.00	0.00
protein FAM115E-like	LOC100753218	100753218	0.00	0.00	0.00	0.00	0.00	0.00	0.00	0.00	0.00	0.00	0.00	0.00
acyl-coenzyme A thioesterase 1-like	LOC100753222	100753222	2.11	1.89	2.23	2.77	1.29	1.43	47.85	53.07	62.10	84.60	34.15	31.78
olfactory receptor 5W2-like	LOC100753223	100753223	0.00	0.00	0.00	0.00	0.00	0.00	0.00	0.00	0.00	0.00	0.00	0.00
ubiquitin-conjugating enzyme E2 L3 pseudogene	LOC100753227	100753227	22.13	25.38	20.55	21.38	21.79	20.92	26.58	22.88	9.94	13.91	14.49	13.09
methionine aminopeptidase 1 pseudogene	LOC100753231	100753231	1.11	1.11	0.93	1.18	1.18	1.26	24.31	27.45	22.36	26.66	27.94	28.97
anionic trypsin-2	LOC100753235	100753235	0.09	0.00	0.00	0.00	0.00	0.00	2.28	0.92	0.00	1.16	0.00	0.00
uncharacterized LOC100753237	LOC100753237	100753237	0.00	0.00	0.00	0.00	0.00	0.00	0.00	0.00	0.00	0.00	0.00	0.00
acyl-CoA desaturase 1-like	LOC100753241	100753241	0.20	0.16	0.11	0.18	0.29	0.22	6.84	9.15	6.21	3.48	7.24	7.48
zinc finger protein 709-like	LOC100753242	100753242	4.89	4.66	5.69	6.59	6.79	6.19	593.20	573.73	700.54	813.56	821.78	760.82
60S ribosomal protein L35	LOC100753244	100753244	30.73	32.98	40.08	27.13	29.31	29.69	209.63	225.10	259.60	170.36	194.58	198.15
olfactory receptor 5AC1-like	LOC100753245	100753245	0.00	0.00	0.00	0.00	0.00	0.00	0.00	0.00	0.00	0.00	0.00	0.00
disintegrin and metalloproteinase domain-containing protein 26A-like	LOC100753247	100753247	0.00	0.00	0.00	0.00	0.00	0.00	0.00	0.00	0.00	0.00	0.00	0.00
5'-nucleotidase-like	LOC100753248	100753248	0.13	0.08	0.00	0.02	0.10	0.02	7.60	4.58	0.00	1.16	5.17	0.93
glutathione S-transferase A4	LOC100753254	100753254	98.97	99.70	119.22	122.98	120.76	136.14	2450.27	2498.96	2918.90	2934.39	2905.21	3217.13
vacuolar protein sorting-associated protein 52 homolog	LOC100753256	100753256	20.63	19.24	17.63	15.17	11.60	13.69	1920.87	1777.91	1678.06	1493.85	1156.08	1363.68
trace amine-associated receptor 7c	LOC100753257	100753257	0.00	0.00	0.00	0.00	0.00	0.00	0.00	0.00	0.00	0.00	0.00	0.00
chromosome unknown open reading frame, human C9orf16	LOC100753264	100753264	9.18	8.86	12.62	9.07	5.93	6.99	171.66	159.22	232.27	191.22	128.34	156.09

transcription factor BTF3 pseudogene	LOC100753265	100753265	1.13	0.91	0.69	0.54	0.42	0.77	12.15	10.07	7.45	5.79	3.10	10.28
olfactory receptor 4F21	LOC100753266	100753266	0.00	0.00	0.00	0.00	0.00	0.00	0.00	0.00	0.00	0.00	0.00	0.00
transmembrane protein 110	LOC100753269	100753269	15.72	15.14	16.81	16.86	15.50	16.15	690.42	679.87	767.61	748.66	720.35	728.11
40S ribosomal protein SA pseudogene	LOC100753271	100753271	2.14	1.68	1.94	1.07	1.19	0.91	79.75	69.54	81.98	45.20	42.43	34.58
NADH-cytochrome b5 reductase-like	LOC100753272	100753272	2.18	1.82	1.89	1.71	1.77	1.98	214.95	181.18	180.10	168.04	175.95	191.61
olfactory receptor 52R1-like	LOC100753273	100753273	0.00	0.00	0.00	0.00	0.00	0.00	0.00	0.00	0.00	0.00	0.00	0.00
membrane magnesium transporter 2	LOC100753275	100753275	4.36	4.96	4.86	4.20	3.51	3.68	202.04	226.01	199.98	228.31	205.96	227.12
complement C5-like	LOC100753276	100753276	0.00	0.00	0.00	0.05	0.00	0.00	0.00	0.00	0.00	1.16	0.00	0.00
vomeronasal type-2 receptor 116-like	LOC100753278	100753278	0.00	0.00	0.00	0.00	0.00	0.00	0.00	0.00	0.00	0.00	0.00	0.00
cathepsin 7	LOC100753281	100753281	0.00	0.00	0.00	0.00	0.00	0.00	0.00	0.00	0.00	0.00	0.00	0.00
uncharacterized LOC100753285	LOC100753285	100753285	1.61	1.42	1.28	1.13	1.41	1.55	290.90	268.10	245.93	225.99	292.90	287.88
glycogen phosphorylase, liver form	LOC100753288	100753288	8.41	8.45	6.47	6.34	7.09	7.63	428.38	445.62	335.36	336.09	375.70	392.56
eukaryotic translation initiation factor 3 subunit J-like	LOC100753289	100753289	0.21	0.10	0.00	0.00	0.00	0.00	3.04	0.92	0.00	0.00	0.00	0.00
chromosome unknown open reading frame, human C5orf63	LOC100753290	100753290	23.21	23.88	18.34	19.32	25.15	22.66	556.74	564.58	439.70	477.47	590.98	534.63
hepatocyte cell adhesion molecule	LOC100753291	100753291	0.00	0.00	0.00	0.00	0.00	0.00	0.00	0.00	0.00	0.00	0.00	0.00
developmentally-regulated GTP-binding protein 1 pseudogene	LOC100753296	100753296	2.59	1.93	1.84	1.93	1.97	2.32	48.61	38.43	43.47	40.56	37.26	55.15
carcinoembryonic antigen-related cell adhesion molecule 3-like	LOC100753299	100753299	0.00	0.00	0.00	0.00	0.00	0.00	0.00	0.00	0.00	0.00	0.00	0.00
40S ribosomal protein S3 pseudogene	LOC100753302	100753302	56.07	61.61	58.13	50.36	60.76	51.32	741.31	848.24	770.09	638.56	813.50	629.97

heat shock protein HSP 90-beta pseudogene	LOC100753307	100753307	0.00	0.00	0.00	0.00	0.00	0.03	0.00	0.00	0.00	0.00	0.00	1.87
olfactory receptor 7E24-like	LOC100753311	100753311	0.00	0.00	0.00	0.00	0.00	0.00	0.00	0.00	0.00	0.00	0.00	0.00
probable rRNA-processing protein EBP2 pseudogene	LOC100753313	100753313	0.22	0.13	0.18	0.11	0.10	0.09	5.32	3.66	3.73	2.32	2.07	2.80
peptidyl-prolyl cis-trans isomerase A-like	LOC100753323	100753323	1.87	1.57	0.17	0.45	0.90	0.89	15.19	11.90	1.24	2.32	7.24	6.54
60S ribosomal protein L23a pseudogene	LOC100753325	100753325	0.16	0.19	0.00	0.00	0.33	0.20	1.52	1.83	0.00	0.00	3.10	1.87
chromosome unknown open reading frame, human C1orf109	LOC100753330	100753330	9.10	8.86	8.18	8.83	7.29	8.92	468.64	466.67	447.15	460.09	391.22	465.47
peptidyl-prolyl cis-trans isomerase D pseudogene	LOC100753334	100753334	0.00	0.00	0.00	0.00	0.00	0.00	0.00	0.00	0.00	0.00	0.00	0.00
chromosome unknown open reading frame, human C12orf50	LOC100753335	100753335	0.02	0.00	0.00	0.00	0.00	0.00	0.76	0.00	0.00	0.00	0.00	0.00
pleiotropic regulator 1 pseudogene	LOC100753344	100753344	0.09	0.14	0.05	0.07	0.13	0.15	3.80	7.32	2.48	3.48	6.21	7.48
ribosomal L1 domain-containing protein 1-like	LOC100753349	100753349	0.93	0.86	0.81	0.78	0.75	0.37	33.42	28.37	26.08	25.50	24.84	16.82
eukaryotic initiation factor 4A-III pseudogene	LOC100753355	100753355	6.07	6.50	5.70	3.50	1.58	1.63	214.19	233.33	206.19	105.46	46.57	49.54
zinc finger protein 420-like	LOC100753357	100753357	0.21	0.19	0.34	0.37	0.70	0.36	15.19	13.73	26.08	26.66	54.85	29.91
olfactory receptor 5B3-like	LOC100753369	100753369	0.00	0.00	0.00	0.00	0.00	0.00	0.00	0.00	0.00	0.00	0.00	0.00
olfactory receptor 2D3-like	LOC100753371	100753371	0.00	0.00	0.00	0.00	0.00	0.00	0.00	0.00	0.00	0.00	0.00	0.00
developmental pluripotency-associated protein 4-like	LOC100753372	100753372	0.00	0.00	0.00	0.00	0.00	0.00	0.00	0.00	0.00	0.00	0.00	0.00
sialic acid-binding Ig-like lectin 9	LOC100753373	100753373	0.00	0.00	0.00	0.00	0.00	0.00	0.00	0.00	0.00	0.00	0.00	0.00
sulfotransferase family cytosolic 2B member 1-like	LOC100753375	100753375	0.00	0.08	0.00	0.05	0.00	0.00	0.00	2.75	0.00	2.32	0.00	0.00
60S ribosomal protein L34 pseudogene	LOC100753380	100753380	0.16	0.00	0.79	0.00	0.00	0.07	0.76	0.00	1.24	0.00	0.00	0.00

protein SET-like	LOC100753387	100753387	0.05	0.04	0.07	0.00	0.05	0.06	0.76	0.00	1.24	0.00	1.03	1.87
ATM interactor pseudogene	LOC100753388	100753388	0.85	0.81	0.96	0.62	0.62	0.91	37.98	42.09	40.99	23.18	25.87	44.86
zinc finger protein 251	LOC100753390	100753390	2.92	2.80	4.09	3.41	3.64	3.68	244.57	225.10	327.91	295.52	303.25	311.25
sepiapterin reductase-like	LOC100753393	100753393	0.13	0.13	0.00	0.06	0.33	0.41	2.28	1.83	0.00	4.64	6.21	9.35
ATP synthase subunit e, mitochondrial-like	LOC100753395	100753395	0.00	0.00	0.00	0.00	0.00	0.00	0.00	0.00	0.00	0.00	0.00	0.00
vomeronasal type-2 receptor 116-like	LOC100753397	100753397	0.00	0.01	0.00	0.01	0.00	0.00	0.00	0.92	0.00	1.16	0.00	1.87
signal-regulatory protein beta-1 isoform 3-like	LOC100753401	100753401	0.00	0.00	0.00	0.00	0.00	0.00	0.00	0.00	0.00	0.00	0.00	0.00
membrane-spanning 4-domains subfamily A member 12-like	LOC100753402	100753402	0.00	0.00	0.00	0.00	0.00	0.00	0.00	0.00	0.00	0.00	0.00	0.00
COMM domain-containing protein 5	LOC100753405	100753405	33.34	31.26	36.53	25.46	22.02	23.79	962.34	896.73	1055.77	723.17	617.89	700.07
thyroid hormone receptor alpha	LOC100753407	100753407	37.43	37.35	32.43	29.03	22.92	23.45	6.84	3.66	8.69	2.32	9.31	2.80
60S ribosomal protein L32 pseudogene	LOC100753408	100753408	51.95	56.29	52.91	50.61	56.00	47.61	85.07	85.10	79.49	81.12	116.95	96.27
zinc finger protein 120-like	LOC100753410	100753410	0.00	0.00	0.00	0.00	0.00	0.00	0.00	0.00	0.00	0.00	0.00	0.00
proline-rich proteoglycan 2-like	LOC100753414	100753414	0.00	0.00	0.00	0.00	0.00	0.00	0.00	0.00	0.00	0.00	0.00	0.00
mesoderm induction early response protein 1-like	LOC100753415	100753415	0.17	0.03	0.08	0.08	0.14	0.06	5.32	0.92	2.48	2.32	4.14	1.87
ATP synthase F(0) complex subunit C2, mitochondrial pseudogene	LOC100753420	100753420	1.29	1.07	0.56	1.04	0.91	0.85	38.74	32.94	14.91	30.13	30.01	28.04
olfactory receptor 8K3-like	LOC100753427	100753427	0.00	0.00	0.00	0.00	0.00	0.00	0.00	0.00	0.00	0.00	0.00	0.00
peptidyl-prolyl cis-trans isomerase H pseudogene	LOC100753428	100753428	0.75	0.87	0.48	0.73	1.17	0.69	10.63	10.98	4.97	10.43	12.42	12.15
60S ribosomal protein L7a pseudogene	LOC100753430	100753430	0.00	0.00	0.00	0.00	0.00	0.00	0.00	0.00	0.00	0.00	0.00	0.00

EKC/KEOPS complex subunit LAGE3-like	LOC100753432	100753432	0.00	0.00	0.00	0.00	0.00	0.00	0.00	0.00	0.00	0.00	0.00	0.00
chromosome unknown open reading frame, human CXorf21	LOC100753437	100753437	0.00	0.00	0.00	0.00	0.00	0.00	0.00	0.00	0.00	0.00	0.00	0.00
NADH dehydrogenase [ubiquinone] 1 alpha subcomplex subunit 12 pseudogene	LOC100753440	100753440	0.17	0.42	0.43	0.55	0.24	0.22	1.52	3.66	7.45	4.64	2.07	3.74
prolactin-6A1-like	LOC100753446	100753446	0.00	0.00	0.00	0.00	0.00	0.00	0.00	0.00	0.00	0.00	0.00	0.00
chloride channel protein ClC-Ka	LOC100753449	100753449	0.01	0.00	0.00	0.00	0.00	0.00	0.76	0.00	0.00	0.00	0.00	0.00
olfactory receptor 149-like	LOC100753451	100753451	0.00	0.00	0.00	0.00	0.00	0.00	0.00	0.00	0.00	0.00	0.00	0.00
keratin, type II cytoskeletal 6B-like	LOC100753455	100753455	0.00	0.00	0.00	0.00	0.00	0.00	0.00	0.00	0.00	0.00	0.00	0.00
uncharacterized LOC100753456	LOC100753456	100753456	6.81	6.38	7.27	7.11	8.64	7.34	214.95	220.52	228.54	243.37	317.74	243.01
olfactory receptor 6C6-like	LOC100753459	100753459	0.00	0.00	0.00	0.00	0.00	0.00	0.00	0.00	0.00	0.00	0.00	0.00
10 kDa heat shock protein, mitochondrial pseudogene	LOC100753460	100753460	0.00	0.25	0.35	0.00	0.29	0.00	1.52	1.83	2.48	0.00	1.03	0.00
keratin-associated protein 20-2-like	LOC100753465	100753465	0.00	0.00	0.00	0.00	0.00	0.00	0.00	0.00	0.00	0.00	0.00	0.00
carbonyl reductase [NADPH] 1-like	LOC100753466	100753466	3.25	3.62	3.15	3.58	3.80	3.59	57.72	55.82	52.17	56.79	62.10	57.95
glutathione S-transferase alpha-3	LOC100753467	100753467	0.50	0.20	0.71	0.83	0.09	0.50	13.67	8.24	18.63	23.18	2.07	16.82
mitochondrial inner membrane protein pseudogene	LOC100753474	100753474	0.00	0.00	0.00	0.00	0.00	0.00	0.00	0.00	0.00	0.00	0.00	0.00
40S ribosomal protein S2 pseudogene	LOC100753476	100753476	0.00	0.00	0.08	0.00	0.00	0.00	0.00	0.00	1.24	0.00	0.00	0.00
olfactory receptor 226	LOC100753481	100753481	0.00	0.00	0.00	0.00	0.00	0.00	0.00	0.00	0.00	0.00	0.00	0.00
vomeronasal type-1 receptor 4-like	LOC100753484	100753484	0.00	0.00	0.00	0.00	0.00	0.00	0.00	0.00	0.00	0.00	0.00	0.00
L-lactate dehydrogenase A chain-like	LOC100753485	100753485	0.00	0.00	0.00	0.00	0.00	0.00	0.00	0.00	0.00	0.00	0.00	0.00

LETM1 domain-containing protein LETM2, mitochondrial	LOC100 753490	100753490	1.43	1.41	1.02	1.52	1.68	1.28	53.93	57.65	43.47	55.63	67.27	62.62
chromosome unknown open reading frame, human C10orf32	LOC100 753492	100753492	0.94	1.24	0.98	0.43	0.27	0.48	32.66	44.84	34.78	15.07	10.35	16.82
EKC/KEOPS complex subunit LAGE3-like	LOC100 753493	100753493	0.00	0.00	0.00	0.00	0.00	0.00	0.00	0.00	0.00	0.00	0.00	0.00
zinc finger protein 709-like	LOC100 753494	100753494	0.00	0.00	0.00	0.00	0.00	0.06	0.00	0.00	0.00	0.00	0.00	0.93
histone H2B type 1-B	LOC100 753502	100753502	2.59	3.04	2.22	0.75	1.01	0.84	0.00	0.00	0.00	0.00	0.00	0.00
transcription factor BTF3 pseudogene	LOC100 753503	100753503	1.83	1.87	1.98	1.45	0.76	1.39	14.43	15.56	16.15	15.07	6.21	12.15
heterogeneous nuclear ribonucleoprotein A1-like	LOC100 753504	100753504	0.28	0.47	0.25	0.25	0.21	0.40	8.35	15.56	7.45	6.95	9.31	14.02
disks large homolog 5-like	LOC100 753509	100753509	0.19	0.04	0.16	0.23	0.50	0.16	3.04	1.83	1.24	4.64	7.24	1.87
fructose-bisphosphate aldolase A pseudogene	LOC100 753510	100753510	0.00	0.00	0.00	0.00	0.00	0.00	0.00	0.00	0.00	0.00	0.00	0.00
centrin-2 pseudogene	LOC100 753513	100753513	3.82	3.46	3.35	2.66	5.99	4.55	96.46	96.08	85.70	77.65	146.97	108.42
cytochrome c-like	LOC100 753519	100753519	10.02	13.80	15.71	10.07	13.63	9.32	102.54	121.70	151.53	78.81	100.39	69.17
chromosome unknown open reading frame, human C4orf45	LOC100 753521	100753521	0.00	0.00	0.00	0.00	0.00	0.00	0.76	0.00	2.48	1.16	1.03	0.00
acyl-coenzyme A thioesterase 1-like	LOC100 753522	100753522	0.03	0.09	0.04	0.04	0.00	0.00	0.00	0.00	0.00	0.00	0.00	0.00
olfactory receptor 1019-like	LOC100 753523	100753523	0.00	0.00	0.00	0.00	0.00	0.00	0.00	0.00	0.00	0.00	0.00	0.00
high mobility group protein B1 pseudogene	LOC100 753524	100753524	0.00	0.07	0.09	0.00	0.08	0.00	1.52	2.75	1.24	1.16	1.03	1.87
H-2 class I histocompatibility antigen, Q10 alpha chain-like	LOC100 753528	100753528	1.23	2.01	1.45	1.26	1.00	0.94	22.03	35.69	33.54	31.29	30.01	27.11
GRB2-associated-binding protein 2 pseudogene	LOC100 753530	100753530	0.03	0.02	0.00	0.05	0.00	0.02	1.52	1.83	0.00	2.32	0.00	1.87
anionic trypsin-2-like	LOC100 753535	100753535	0.00	0.00	0.00	0.00	0.00	0.00	0.00	0.00	0.00	0.00	0.00	0.00

lys-63-specific deubiquitinase BRCC36	LOC100753541	100753541	0.00	0.00	0.00	0.00	0.00	0.00	0.00	0.00	0.00	0.00	0.00	0.00
acyl-CoA desaturase 2-like	LOC100753542	100753542	0.17	0.13	0.40	0.20	0.28	0.16	5.32	0.00	7.45	3.48	2.07	4.67
40S ribosomal protein S8 pseudogene	LOC100753543	100753543	0.00	0.00	0.00	0.00	0.00	0.00	0.00	0.00	0.00	0.00	0.00	0.00
olfactory receptor 5AC1-like	LOC100753546	100753546	0.00	0.00	0.00	0.00	0.00	0.00	0.00	0.00	0.00	0.00	0.00	0.00
olfactory receptor 9K2	LOC100753547	100753547	0.00	0.00	0.00	0.00	0.00	0.00	0.00	0.00	0.00	0.00	0.00	0.00
chromosome unknown open reading frame, human C14orf159	LOC100753550	100753550	0.05	0.08	0.03	0.04	0.07	0.02	6.84	10.98	2.48	5.79	7.24	2.80
GRB2-associated-binding protein 2 pseudogene	LOC100753556	100753556	0.00	0.00	0.00	0.00	0.00	0.00	0.00	0.00	0.00	0.00	0.00	0.00
H-2 class I histocompatibility antigen, L-D alpha chain-like	LOC100753557	100753557	106.76	97.80	122.86	115.15	89.50	105.44	1254.76	1131.90	1413.49	1287.56	1052.58	1233.76
trace amine-associated receptor 8a	LOC100753558	100753558	0.00	0.00	0.00	0.00	0.00	0.00	0.00	0.00	0.00	0.00	0.00	0.00
chromosome unknown open reading frame, human C11orf84	LOC100753566	100753566	18.64	17.79	21.60	18.67	13.14	15.16	887.90	839.09	1001.12	879.62	632.38	722.50
olfactory receptor 4K3	LOC100753567	100753567	0.00	0.00	0.00	0.00	0.00	0.00	0.00	0.00	0.00	0.00	0.00	0.00
F-box/WD repeat-containing protein 11-like	LOC100753568	100753568	0.91	0.87	0.50	0.78	0.62	0.70	47.09	46.67	29.81	46.36	33.12	32.71
probable G-protein coupled receptor 160	LOC100753572	100753572	0.00	0.00	0.00	0.00	0.00	0.00	0.00	0.00	0.00	0.00	0.00	0.00
olfactory receptor 51F1	LOC100753574	100753574	0.00	0.00	0.00	0.00	0.00	0.00	0.00	0.00	0.00	0.00	0.00	0.00
complement C5-like	LOC100753577	100753577	0.00	0.00	0.00	0.00	0.00	0.00	0.00	0.00	0.00	0.00	0.00	0.00
zinc finger MYND domain-containing protein 19 pseudogene	LOC100753585	100753585	0.00	0.06	0.00	0.00	0.00	0.00	0.00	0.92	0.00	0.00	0.00	0.00
Ig heavy chain V region 108A-like	LOC100753586	100753586	0.00	0.00	0.00	0.00	0.00	0.00	0.00	0.00	0.00	0.00	0.00	0.00
leukocyte immunoglobulin-like receptor subfamily B member 3	LOC100753588	100753588	0.00	0.00	0.00	0.00	0.00	0.00	0.00	0.00	0.00	0.00	0.00	0.00

chromatin target of PRMT1 protein pseudogene	LOC100 753592	100753592	0.00	0.00	0.00	0.00	0.00	0.00	0.00	0.00	0.00	0.00	0.00	0.00
60S ribosomal protein L21	LOC100 753593	100753593	16.79	18.32	14.54	10.76	14.24	11.48	136.72	146.41	109.30	92.71	111.78	89.73
LIM and senescent cell antigen-like-containing domain protein 1 pseudogene	LOC100 753595	100753595	0.12	0.07	0.10	0.05	0.17	0.00	2.28	2.75	3.73	1.16	3.10	0.00
acyl-CoA-binding protein pseudogene	LOC100 753598	100753598	1.75	1.77	0.25	0.24	1.03	0.76	12.91	10.07	2.48	2.32	6.21	3.74
14-3-3 protein epsilon	LOC100 753603	100753603	498.75	477.88	501.80	507.19	547.53	539.45	9265.62	8590.33	9519.35	9705.95	10751.44	10907.60
proteasome assembly chaperone 2 pseudogene	LOC100 753610	100753610	0.00	0.05	0.00	0.00	0.03	0.00	0.76	0.92	0.00	0.00	0.00	0.93
olfactory receptor 7D4-like	LOC100 753612	100753612	0.00	0.00	0.00	0.00	0.00	0.00	0.00	0.00	0.00	0.00	0.00	0.00
major histocompatibility complex class I-related gene protein	LOC100 753615	100753615	0.10	0.07	0.14	0.04	0.08	0.07	9.11	7.32	13.66	6.95	10.35	9.35
40S ribosomal protein S10 pseudogene	LOC100 753617	100753617	0.44	0.36	0.24	1.00	0.41	0.61	13.67	13.73	7.45	23.18	13.45	12.15
40S ribosomal protein S6-like	LOC100 753620	100753620	0.00	0.37	0.22	0.14	0.27	0.11	0.76	10.98	6.21	3.48	6.21	1.87
uncharacterized LOC100753628	LOC100 753628	100753628	4.26	4.08	3.13	3.76	1.74	1.84	1396.03	1337.78	1033.42	1248.16	575.45	607.54
paired mesoderm homeobox protein 2 pseudogene	LOC100 753633	100753633	0.00	0.00	0.00	0.00	0.00	0.00	0.00	0.00	0.00	0.00	0.00	0.00
transcription initiation factor IIA subunit 2 pseudogene	LOC100 753634	100753634	1.46	1.10	0.94	1.27	1.88	1.00	6.08	3.66	2.48	3.48	7.24	5.61
chromosome unknown open reading frame, human C10orf99	LOC100 753638	100753638	0.11	0.00	0.00	0.00	0.08	0.00	1.52	0.00	0.00	0.00	1.03	0.00
40S ribosomal protein S12 pseudogene	LOC100 753639	100753639	0.00	0.00	0.00	0.00	0.00	0.00	0.00	0.00	0.00	0.00	0.00	0.00
uncharacterized protein C9orf85 homolog pseudogene	LOC100 753643	100753643	0.13	0.08	0.00	0.00	0.00	0.00	1.52	0.92	0.00	0.00	0.00	0.00
lysosomal Pro-X carboxypeptidase-like	LOC100 753648	100753648	0.06	0.09	0.02	0.00	0.13	0.02	3.80	5.49	1.24	0.00	3.10	0.93
serine/threonine-protein phosphatase 2A 55 kDa regulatory subunit B alpha isoform pseudogene	LOC100 753649	100753649	0.07	0.00	0.00	0.00	0.00	0.00	1.52	0.00	0.00	0.00	1.03	0.93

60S ribosomal protein L37 pseudogene	LOC100753650	100753650	0.58	1.53	1.34	2.26	2.46	0.27	0.00	0.00	0.00	0.00	0.00	0.00
chromosome unknown open reading frame, human C22orf23	LOC100753660	100753660	5.97	7.57	5.69	5.54	3.83	4.47	632.70	639.61	604.90	557.44	464.71	512.20
heterogeneous nuclear ribonucleoprotein A3 pseudogene	LOC100753666	100753666	0.03	0.00	0.00	0.04	0.00	0.03	0.76	0.00	0.00	1.16	0.00	0.93
zinc finger protein 728-like	LOC100753667	100753667	4.01	3.98	5.01	5.65	5.43	5.29	281.79	272.68	353.99	400.99	387.08	371.06
olfactory receptor 5B3	LOC100753670	100753670	0.00	0.00	0.00	0.00	0.00	0.00	0.00	0.00	0.00	0.00	0.00	0.00
PRAME family member 5-like	LOC100753671	100753671	0.00	0.00	0.00	0.00	0.00	0.00	0.00	0.00	0.00	0.00	0.00	0.00
olfactory receptor 2D3-like	LOC100753672	100753672	0.00	0.00	0.00	0.00	0.00	0.00	0.00	0.00	0.00	0.00	0.00	0.00
olfactory receptor 11G2-like	LOC100753673	100753673	0.00	0.00	0.00	0.00	0.00	0.00	0.00	0.00	0.00	0.00	0.00	0.00
rRNA-processing protein FCF1 homolog	LOC100753675	100753675	0.06	0.00	0.00	0.10	0.00	0.00	2.28	2.75	0.00	1.16	2.07	0.93
putative vomeronasal receptor-like protein 4	LOC100753678	100753678	0.00	0.00	0.00	0.00	0.00	0.00	0.00	0.00	0.00	0.00	0.00	0.00
cytochrome P450 2C18-like	LOC100753681	100753681	0.02	0.00	0.00	0.00	0.00	0.00	0.76	0.92	0.00	1.16	0.00	0.00
HERV-K_11q22.1 provirus ancestral Pol protein-like	LOC100753682	100753682	0.00	0.00	0.00	0.00	0.00	0.00	0.00	0.00	0.00	0.00	0.00	0.00
sperm motility kinase X-like	LOC100753695	100753695	0.19	0.26	0.24	0.26	0.42	0.30	9.11	12.81	9.94	9.27	17.59	13.09
serine/threonine-protein phosphatase PP1-gamma catalytic subunit pseudogene	LOC100753696	100753696	3.36	2.84	3.06	2.22	2.18	2.78	119.25	108.89	116.76	91.55	77.62	91.60
tudor and KH domain-containing protein pseudogene	LOC100753697	100753697	0.00	0.00	0.00	0.07	0.00	0.05	0.00	0.00	0.00	1.16	0.00	0.93
nucleoporin NUP188 homolog	LOC100753702	100753702	26.37	24.92	27.56	27.11	24.79	27.60	2448.75	2325.10	2515.23	2417.51	2192.10	2363.78
cytochrome P450 2C26-like	LOC100753704	100753704	0.00	0.00	0.00	0.00	0.00	0.00	0.00	0.00	0.00	0.00	0.00	0.00
rho guanine nucleotide exchange factor 3 pseudogene	LOC100753706	100753706	0.00	0.00	0.00	0.00	0.00	0.00	0.00	0.00	0.00	0.00	0.00	0.00

40S ribosomal protein S6	LOC100 753707	100753707	891.15	860.18	918.29	956.23	935.06	979.64	4387.86	4598.96	4877.68	4863.99	4832.35	4807.01
40S ribosomal protein S11-like	LOC100 753710	100753710	0.00	0.00	0.00	0.00	0.00	0.00	0.00	0.00	0.00	0.00	0.00	0.93
olfactory receptor 2AE1-like	LOC100 753711	100753711	0.00	0.00	0.00	0.00	0.00	0.00	0.00	0.00	0.00	0.00	0.00	0.00
zinc finger protein 120-like	LOC100 753714	100753714	4.26	3.80	4.05	4.77	5.85	4.88	350.15	312.94	339.09	399.83	484.37	407.52
taste receptor type 2 member 14-like	LOC100 753718	100753718	0.00	0.00	0.00	0.00	0.00	0.00	0.00	0.00	0.00	0.00	0.00	0.00
poly(rC)-binding protein 2-like	LOC100 753720	100753720	12.06	12.77	12.07	11.15	11.29	8.82	410.15	441.96	423.55	359.27	351.90	271.05
uncharacterized LOC100753723	LOC100 753723	100753723	0.33	0.49	0.63	1.16	0.81	0.70	19.75	21.96	33.54	59.10	40.36	36.45
olfactory receptor 8K3-like	LOC100 753729	100753729	0.00	0.00	0.00	0.00	0.00	0.00	0.00	0.00	0.00	0.00	0.00	0.00
histone H3.3-like	LOC100 753731	100753731	0.29	0.51	1.19	1.17	1.34	0.93	6.84	10.98	9.94	9.27	8.28	5.61
olfactory receptor 7E24-like	LOC100 753737	100753737	0.00	0.00	0.00	0.00	0.00	0.00	0.00	0.00	0.00	0.00	0.00	0.00
uncharacterized LOC100753738	LOC100 753738	100753738	0.00	0.00	0.00	0.00	0.00	0.00	0.00	0.00	0.00	0.00	0.00	0.00
glyceraldehyde-3-phosphate dehydrogenase pseudogene	LOC100 753740	100753740	0.00	0.00	0.00	0.00	0.00	0.00	0.00	0.00	0.00	0.00	0.00	0.00
cytochrome P450 2D28-like	LOC100 753741	100753741	0.02	0.05	0.03	0.06	0.00	0.00	0.76	0.92	1.24	2.32	0.00	0.00
60S ribosomal protein L39 pseudogene	LOC100 753742	100753742	0.00	0.00	0.00	0.00	0.00	0.00	0.00	0.00	0.00	0.00	0.00	0.93
ribosome production factor 2 homolog pseudogene	LOC100 753743	100753743	0.69	0.88	0.55	0.27	0.32	0.44	11.39	11.90	7.45	3.48	3.10	5.61
SCAN domain-containing protein 3-like	LOC100 753744	100753744	4.69	4.47	4.74	4.72	4.39	4.27	218.75	215.03	269.53	321.02	300.15	314.05
prolactin-3D1-like	LOC100 753747	100753747	0.00	0.00	0.00	0.00	0.00	0.00	0.00	0.00	0.00	0.00	0.00	0.00
chloride channel protein ClC-Kb	LOC100 753750	100753750	0.00	0.00	0.00	0.00	0.00	0.00	0.00	0.00	0.00	1.16	0.00	0.00

olfactory receptor 8D1-like	LOC100 753752	100753752	0.00	0.04	0.00	0.00	0.00	0.00	0.00	0.92	0.00	0.00	0.00	0.00
lipoma HMGIC fusion partner	LOC100 753753	100753753	12.49	13.14	16.49	17.57	20.30	19.88	406.35	427.32	544.03	573.67	638.59	633.71
keratin, type II cytoskeletal 6A	LOC100 753757	100753757	0.02	0.05	0.00	0.00	0.00	0.00	0.76	1.83	0.00	0.00	0.00	0.00
actin-related protein 2 pseudogene	LOC100 753758	100753758	0.18	0.12	0.00	0.08	0.14	0.16	5.32	6.41	0.00	2.32	5.17	6.54
CD177 antigen	LOC100 753760	100753760	0.56	0.48	0.23	0.20	0.15	0.09	50.13	42.09	18.63	23.18	10.35	5.61
eukaryotic translation initiation factor 1	LOC100 753761	100753761	3.72	2.86	2.05	1.20	3.10	2.37	26.58	17.39	14.91	11.59	18.63	15.89
forkhead box protein I3-like	LOC100 753769	100753769	0.00	0.00	0.00	0.00	0.00	0.00	0.00	0.00	0.00	0.00	0.00	0.00
glutathione S-transferase A6	LOC100 753770	100753770	22.97	22.77	21.16	23.83	16.76	17.75	571.93	581.96	535.34	598.00	423.31	430.88
60S ribosomal protein L29 pseudogene	LOC100 753771	100753771	0.67	0.41	0.78	0.37	0.11	0.20	4.56	2.75	6.21	2.32	1.03	0.93
uncharacterized LOC100753780	LOC100 753780	100753780	213.42	195.53	222.42	198.47	153.11	172.20	9757.04	9438.57	10629.78	9656.12	7475.71	8382.12
vomeronasal type-1 receptor 4-like	LOC100 753784	100753784	0.00	0.00	0.00	0.00	0.00	0.00	0.00	0.00	0.00	0.00	0.00	0.00
chromosome unknown open reading frame, human C8orf74	LOC100 753786	100753786	0.00	0.00	0.00	0.00	0.00	0.00	0.00	0.00	0.00	0.00	0.00	0.00
solute carrier family 25 member 51-like	LOC100 753788	100753788	0.78	0.72	0.59	0.41	0.77	0.45	10.63	2.75	9.94	3.48	7.24	3.74
phosphatidylethanolamine-binding protein 1-like	LOC100 753789	100753789	0.33	0.25	0.15	0.09	0.17	0.22	3.80	2.75	1.24	1.16	2.07	2.80
GTPase KRas pseudogene	LOC100 753790	100753790	0.68	0.50	0.62	0.22	0.52	0.43	1.52	0.92	0.00	0.00	1.03	0.00
40S ribosomal protein S11-like	LOC100 753792	100753792	0.00	0.00	0.00	0.00	0.13	0.00	0.00	0.00	0.00	1.16	2.07	0.00
olfactory receptor 8S1	LOC100 753793	100753793	0.00	0.00	0.00	0.00	0.00	0.00	0.00	0.00	0.00	0.00	0.00	0.00
histone H2A type 1	LOC100 753804	100753804	0.03	0.04	0.00	0.00	0.00	0.00	1.52	0.92	0.00	0.00	0.00	0.00

immunoglobulin iota chain-like	LOC100 753806	100753806	0.00	0.00	0.00	0.00	0.00	0.00						
developmentally-regulated GTP-binding protein 1 pseudogene	LOC100 753810	100753810	0.63	0.76	1.03	1.35	0.46	0.42	6.08	10.98	9.94	8.11	5.17	1.87
26S proteasome non-ATPase regulatory subunit 4 pseudogene	LOC100 753812	100753812	0.12	0.00	0.13	0.00	0.00	0.00	3.04	0.00	2.48	0.00	1.03	0.00
olfactory receptor-like protein OLF3	LOC100 753820	100753820	0.00	0.00	0.00	0.00	0.00	0.00	0.00	0.00	0.00	0.00	0.00	0.00
40S ribosomal protein S15 pseudogene	LOC100 753823	100753823	0.00	0.00	0.00	0.00	0.00	0.00	0.00	0.00	0.00	0.00	0.00	0.00
acyl-coenzyme A thioesterase 5-like	LOC100 753824	100753824	0.02	0.03	0.00	0.01	0.01	0.00	0.00	0.00	0.00	0.00	0.00	0.00
olfactory receptor 10AG1-like	LOC100 753825	100753825	0.00	0.00	0.00	0.00	0.00	0.00	0.00	0.00	0.00	0.00	0.00	0.00
mitochondrial intermembrane space import and assembly protein 40 pseudogene	LOC100 753829	100753829	1.60	1.64	1.76	1.68	1.33	1.37	11.39	9.15	14.91	15.07	7.24	11.22
UPF0378 protein KIAA0100-like	LOC100 753831	100753831	0.03	0.03	0.01	0.02	0.04	0.02	6.08	5.49	1.24	3.48	7.24	3.74
olfactory receptor 9A4	LOC100 753836	100753836	0.00	0.00	0.00	0.00	0.00	0.00	0.00	0.00	0.00	0.00	0.00	0.00
ras-related protein Rab-5B pseudogene	LOC100 753839	100753839	0.00	0.06	0.00	0.00	0.07	0.00	0.76	0.92	0.00	0.00	1.03	0.00
chromosome unknown open reading frame, human C10orf76	LOC100 753840	100753840	8.14	8.40	8.75	9.95	11.61	11.88	636.49	646.93	716.68	744.03	893.19	896.35
RRP15-like protein pseudogene	LOC100 753845	100753845	0.00	0.00	0.00	0.00	0.00	0.00	0.00	0.00	0.00	0.00	0.00	0.00
olfactory receptor 187-like	LOC100 753848	100753848	0.00	0.00	0.00	0.00	0.00	0.00	0.00	0.00	0.00	0.00	0.00	0.00
dynein light chain 1, cytoplasmic-like	LOC100 753850	100753850	0.00	0.00	0.00	0.00	0.00	0.00	0.00	0.00	0.00	0.00	0.00	0.00
fructose-bisphosphate aldolase A pseudogene	LOC100 753853	100753853	0.69	0.44	0.37	0.17	0.29	0.23	17.47	11.90	9.94	4.64	7.24	5.61
centrin-2 pseudogene	LOC100 753854	100753854	0.09	0.00	0.00	0.14	0.00	0.00	3.04	1.83	1.24	1.16	1.03	0.00
uncharacterized LOC100753856	LOC100 753856	100753856	0.02	0.00	0.00	0.03	0.00	0.00	6.08	1.83	8.69	6.95	6.21	9.35

glutathione S-transferase A2	LOC100753857	100753857	0.06	0.00	0.00	0.00	0.00	0.00	1.52	0.00	0.00	0.00	0.00	0.00
small ubiquitin-related modifier 2	LOC100753859	100753859	0.18	0.60	0.24	0.58	0.60	0.37	18.23	21.96	19.87	19.70	7.24	9.35
enhancer of rudimentary homolog pseudogene	LOC100753861	100753861	0.49	0.12	0.66	0.64	0.55	0.25	6.08	3.66	8.69	5.79	6.21	4.67
mitochondrial thiamine pyrophosphate carrier pseudogene	LOC100753863	100753863	0.04	0.03	0.00	0.00	0.06	0.00	0.00	0.00	0.00	0.00	1.03	0.00
putative Dol-P-Glc:Glc(2)Man(9)GlcNAc(2)-PP-Dol alpha-1,2-glucosyltransferase	LOC100753866	100753866	5.15	5.27	6.27	6.95	7.20	7.10	687.38	708.24	845.86	927.14	960.47	958.97
calcium-binding mitochondrial carrier protein SCaMC-1-like	LOC100753868	100753868	0.00	0.00	0.03	0.00	0.00	0.00	0.76	1.83	1.24	0.00	1.03	0.00
olfactory receptor 4K3	LOC100753869	100753869	0.00	0.00	0.00	0.00	0.00	0.04	0.00	0.00	0.00	0.00	0.00	0.93
transforming growth factor beta regulator 1 pseudogene	LOC100753871	100753871	0.00	0.00	0.00	0.00	0.00	0.00	0.00	0.00	0.00	0.00	0.00	0.00
mitochondrial glutamate carrier 1 pseudogene	LOC100753875	100753875	0.00	0.00	0.00	0.00	0.00	0.00	0.00	0.00	0.00	0.00	0.00	0.00
olfactory receptor 51F2	LOC100753877	100753877	0.00	0.00	0.00	0.00	0.00	0.00	0.00	0.00	0.00	0.00	0.00	0.00
	LOC100753880	100753880	0.04	0.01	0.04	0.05	0.05	0.04	5.32	4.58	13.66	27.81	31.05	42.99
mRNA turnover protein 4 homolog pseudogene	LOC100753885	100753885	6.79	7.48	5.20	4.31	4.12	4.99	85.07	97.91	63.35	53.31	46.57	64.49
chromatin target of PRMT1 protein pseudogene	LOC100753888	100753888	0.00	0.00	0.00	0.00	0.00	0.00	0.00	0.00	0.00	0.00	0.00	0.00
leukocyte immunoglobulin-like receptor subfamily B member 3	LOC100753890	100753890	0.00	0.00	0.00	0.00	0.00	0.00	0.00	0.00	0.00	0.00	0.00	0.00
actin-related protein 2 pseudogene	LOC100753893	100753893	0.01	0.00	0.00	0.04	0.01	0.03	0.00	0.00	0.00	0.00	0.00	0.93
mortality factor 4-like protein 2	LOC100753896	100753896	0.00	0.00	0.00	0.00	0.00	0.00	0.00	0.00	0.00	0.00	0.00	0.00
chromosome unknown open reading frame, human C11orf53	LOC100753897	100753897	0.09	0.00	0.00	0.00	0.00	0.00	2.28	1.83	1.24	1.16	0.00	0.00
olfactory receptor 14A2-like	LOC100753900	100753900	0.00	0.00	0.00	0.00	0.00	0.00	0.00	0.00	0.00	0.00	0.00	0.00

transmembrane protein 198 pseudogene	LOC100753902	100753902	0.06	0.21	0.24	0.00	0.00	0.00	0.76	2.75	1.24	0.00	0.00	0.00
post-GPI attachment to proteins factor 2 pseudogene	LOC100753912	100753912	0.03	0.24	0.08	0.03	0.02	0.04	0.00	2.75	1.24	0.00	0.00	0.93
metal-response element-binding transcription factor 2 pseudogene	LOC100753919	100753919	0.00	0.00	0.00	0.00	0.00	0.00	0.00	0.00	0.00	0.00	0.00	0.00
dnaJ homolog subfamily B member 6-like	LOC100753920	100753920	0.41	0.11	0.15	0.00	0.06	0.23	9.87	3.66	3.73	0.00	1.03	5.61
chromosome unknown open reading frame, human C11orf65	LOC100753928	100753928	0.02	0.08	0.14	0.10	0.14	0.10	5.32	5.49	9.94	6.95	12.42	10.28
T-cell surface glycoprotein CD3 zeta chain-like	LOC100753930	100753930	0.00	0.00	0.00	0.00	0.00	0.00	0.00	0.00	0.00	0.00	0.00	0.00
olfactory receptor 2T2	LOC100753931	100753931	0.00	0.00	0.00	0.00	0.00	0.00	0.00	0.00	0.00	0.00	0.00	0.00
chromosome unknown open reading frame, human C6orf89	LOC100753933	100753933	62.44	61.19	83.23	88.06	80.19	85.11	3528.06	3446.02	4686.39	4839.65	4397.66	4679.89
RCC1 and BTB domain-containing protein 1 pseudogene	LOC100753934	100753934	2.73	2.59	2.58	2.69	2.87	2.90	97.98	84.18	91.91	86.92	79.69	82.25
uncharacterized LOC100753938	LOC100753938	100753938	0.00	0.00	0.00	0.00	0.00	0.11	0.76	0.00	0.00	0.00	0.00	2.80
reactive oxygen species modulator 1 pseudogene	LOC100753939	100753939	0.00	0.00	0.00	0.00	0.00	0.00	0.00	0.00	0.00	0.00	0.00	0.00
3-ketoacyl-CoA thiolase B, peroxisomal-like	LOC100753943	100753943	4.37	3.79	4.40	3.32	1.79	1.96	3.80	3.66	4.97	2.32	7.24	2.80
ferritin light chain 1 pseudogene	LOC100753945	100753945	0.00	0.00	0.00	0.00	0.00	0.00	0.00	0.00	0.00	0.00	0.00	0.00
60S ribosomal protein L37 pseudogene	LOC100753948	100753948	0.60	1.58	1.38	1.81	2.33	0.28	0.00	0.00	0.00	0.00	0.00	0.00
60S ribosomal protein L23a	LOC100753949	100753949	0.65	0.75	0.47	0.30	0.00	0.36	4.56	5.49	3.73	2.32	0.00	2.80
glycosyltransferase 6 domain-containing protein 1-like	LOC100753950	100753950	0.00	0.00	0.00	0.00	0.00	0.00	0.00	0.00	0.00	0.00	0.00	0.00
high mobility group protein B1 pseudogene	LOC100753951	100753951	0.17	0.36	0.18	0.04	0.20	0.09	2.28	5.49	3.73	0.00	6.21	3.74
deoxycytidine kinase pseudogene	LOC100753952	100753952	0.00	0.00	0.21	0.00	0.00	0.00	0.00	0.00	3.73	0.00	0.00	0.00

zinc finger protein 345-like	LOC100 753956	100753956	4.85	4.84	5.01	5.57	5.16	4.87	352.43	344.05	373.87	413.73	373.63	357.98
pre-mRNA-splicing factor SLU7 pseudogene	LOC100 753957	100753957	0.08	0.07	0.00	0.00	0.04	0.03	3.04	2.75	0.00	0.00	3.10	0.93
sepiapterin reductase-like	LOC100 753961	100753961	0.78	0.54	0.71	1.16	0.98	1.28	32.66	29.28	36.02	48.67	45.54	53.28
zinc finger protein 58-like	LOC100 753964	100753964	0.18	0.17	0.36	0.43	0.39	0.32	12.15	13.73	24.84	17.38	22.77	16.82
olfactory receptor 5B3	LOC100 753967	100753967	0.00	0.00	0.00	0.00	0.00	0.00	0.00	0.00	0.00	0.00	0.00	0.00
2-oxoisovalerate dehydrogenase subunit beta, mitochondrial pseudogene	LOC100 753968	100753968	0.07	0.39	0.00	0.06	0.05	0.09	1.52	10.07	0.00	1.16	2.07	1.87
olfactory receptor 6	LOC100 753969	100753969	0.00	0.00	0.00	0.00	0.00	0.00	0.00	0.00	0.00	0.00	0.00	0.00
olfactory receptor 11G2-like	LOC100 753970	100753970	0.00	0.00	0.00	0.00	0.00	0.00	0.00	0.00	0.00	0.00	0.00	0.00
sialic acid-binding Ig-like lectin 10	LOC100 753971	100753971	0.00	0.00	0.00	0.00	0.00	0.00	0.00	0.00	0.00	0.00	0.00	0.00
seminal plasma protein BSP-30 kDa-like	LOC100 753974	100753974	0.00	0.00	0.00	0.00	0.00	0.00	0.00	0.00	0.00	0.00	0.00	0.00
protein FAM156A/FAM156B-like	LOC100 753978	100753978	0.00	0.00	0.00	0.00	0.00	0.00	0.00	0.00	0.00	0.00	0.00	0.00
RNA-binding protein FUS pseudogene	LOC100 753979	100753979	0.00	0.00	0.00	0.00	0.00	0.00	0.00	0.00	0.00	0.00	0.00	0.00
uncharacterized LOC100753980	LOC100 753980	100753980	6.37	5.77	7.13	6.16	8.01	7.41	347.11	398.95	411.13	416.05	392.26	409.39
uncharacterized LOC100753984	LOC100 753984	100753984	0.00	0.00	0.00	0.00	0.00	0.00	0.00	0.00	1.24	0.00	0.00	0.00
dolichyl-diphosphooligosaccharide--protein glycosyltransferase subunit 4-like	LOC100 753988	100753988	10.04	0.00	0.00	0.00	0.00	4.38	3.04	0.00	0.00	0.00	0.00	0.93
polyadenylate-binding protein 1-like 2	LOC100 753990	100753990	0.00	0.00	0.00	0.00	0.00	0.00	0.00	0.00	0.00	0.00	0.00	0.00
speckle-type POZ protein pseudogene	LOC100 753991	100753991	0.04	0.00	0.00	0.00	0.00	0.00	0.76	0.00	0.00	0.00	0.00	0.00
40S ribosomal protein S12 pseudogene	LOC100 753993	100753993	0.00	0.00	0.00	0.00	0.00	0.00	0.00	0.00	0.00	0.00	0.00	0.00

zinc finger protein OZF-like	LOC100753996	100753996	5.19	5.09	5.57	6.81	6.41	6.49	543.83	528.89	616.08	749.82	704.83	708.48
transmembrane protein 127 pseudogene	LOC100754000	100754000	0.07	0.15	0.00	0.07	0.19	0.00	0.00	0.00	0.00	0.00	0.00	0.00
kelch-like protein 38	LOC100754001	100754001	0.15	0.11	0.04	0.04	0.01	0.01	0.00	0.00	0.00	0.00	0.00	0.00
pre-mRNA-processing-splicing factor 8	LOC100754005	100754005	78.74	85.52	86.42	88.82	86.61	87.49	0.00	0.00	0.00	0.00	0.00	0.00
taste receptor type 2 member 102-like	LOC100754012	100754012	0.00	0.00	0.00	0.00	0.00	0.00	0.00	0.00	0.00	0.00	0.00	0.00
uncharacterized protein C14orf53-like	LOC100754013	100754013	0.00	0.00	0.00	0.00	0.00	0.00	0.00	0.00	0.00	0.00	0.00	0.00
granzyme C-like	LOC100754015	100754015	0.00	0.00	0.00	0.00	0.00	0.00	0.00	0.00	0.00	0.00	0.00	0.00
ankyrin-1	LOC100754018	100754018	0.46	0.53	0.05	0.05	0.14	0.16	81.27	81.44	6.21	9.27	21.73	23.37
NUT family member Gm806-like	LOC100754022	100754022	0.00	0.00	0.00	0.00	0.00	0.00	0.00	0.00	0.00	0.00	0.00	0.00
olfactory receptor 1086	LOC100754026	100754026	0.00	0.00	0.00	0.00	0.00	0.00	0.00	0.00	0.00	0.00	0.00	0.00
histone-binding protein RBBP4 pseudogene	LOC100754035	100754035	0.51	0.49	0.34	0.47	0.30	0.43	12.15	8.24	7.45	10.43	8.28	10.28
uncharacterized LOC100754036	LOC100754036	100754036	0.00	0.00	0.00	0.00	0.00	0.00	0.00	0.00	0.00	0.00	0.00	0.00
chromosome unknown open reading frame, human C5orf46	LOC100754037	100754037	0.00	0.00	0.00	0.00	0.00	0.00	0.00	0.00	0.00	0.00	0.00	0.00
mas-related G-protein coupled receptor member A	LOC100754040	100754040	0.00	0.00	0.00	0.00	0.00	0.00	0.00	0.00	0.00	0.00	0.00	0.00
cytochrome c, somatic	LOC100754043	100754043	0.66	0.92	0.32	0.00	0.00	0.25	2.28	3.66	1.24	0.00	0.00	0.93
putative uncharacterized protein	LOC100754044	100754044	1.96	2.03	2.43	1.50	2.23	2.81	50.89	53.99	48.44	38.24	58.99	61.69
TGF-beta-activated kinase 1 and MAP3K7-binding protein 1 pseudogene	LOC100754048	100754048	0.00	0.02	0.00	0.03	0.00	0.00	0.00	0.92	0.00	0.00	0.00	0.00
transmembrane protein 11, mitochondrial pseudogene	LOC100754049	100754049	0.00	0.00	0.00	0.00	0.08	0.00	0.00	0.00	0.00	1.16	1.03	0.00

BPI fold-containing family A member 1	LOC100754051	100754051	0.00	0.00	0.00	0.00	0.00	0.00	0.00	0.00	0.00	0.00	0.00	0.00
keratin, type II cytoskeletal 6A-like	LOC100754054	100754054	0.07	0.08	0.00	0.00	0.00	0.00	2.28	3.66	0.00	0.00	0.00	0.00
NUT family member Gm806-like	LOC100754055	100754055	0.00	0.00	0.00	0.00	0.00	0.00	0.00	0.00	0.00	0.00	0.00	0.00
olfactory receptor 6C74	LOC100754058	100754058	0.00	0.00	0.00	0.00	0.00	0.00	0.00	0.00	0.00	0.00	0.00	0.00
MLV-related proviral Env polyprotein-like	LOC100754059	100754059	6.59	7.26	6.63	6.65	6.56	6.27	260.52	301.05	267.05	271.19	238.05	242.08
keratin-associated protein 20-2-like	LOC100754063	100754063	0.00	0.00	0.00	0.00	0.00	0.00	0.00	0.00	0.00	0.00	0.00	0.00
polymerase delta-interacting protein 3 pseudogene	LOC100754064	100754064	2.09	1.82	1.95	1.27	2.23	1.83	13.67	12.81	9.94	4.64	11.38	13.09
lipid phosphate phosphatase-related protein type 4	LOC100754065	100754065	0.00	0.00	0.00	0.00	0.00	0.00	0.00	0.00	1.24	0.00	0.00	0.00
glutathione S-transferase alpha-3-like	LOC100754066	100754066	0.00	0.15	0.00	0.00	0.00	0.00	0.00	0.92	0.00	0.00	0.00	0.00
eukaryotic translation initiation factor 3 subunit C	LOC100754077	100754077	319.67	308.58	325.62	319.24	305.32	309.80	14017.30	14155.57	14495.15	14190.97	13892.62	13971.44
60S ribosomal protein L35a pseudogene	LOC100754079	100754079	1.45	1.51	0.58	0.53	0.98	3.06	7.60	7.32	2.48	2.32	5.17	15.89
vomeronasal type-1 receptor 4-like	LOC100754080	100754080	0.00	0.00	0.00	0.00	0.00	0.00	0.00	0.00	0.00	0.00	0.00	0.00
phosphatidate phosphatase PPAPDC1A	LOC100754084	100754084	0.00	0.00	0.00	0.00	0.00	0.00	0.00	0.00	0.00	0.00	0.00	0.00
periphilin-1-like	LOC100754094	100754094	0.00	0.00	0.00	0.00	0.00	0.00	0.00	0.00	0.00	0.00	0.00	0.00
histone H3	LOC100754104	100754104	0.16	0.11	0.03	0.00	0.00	0.00	5.32	3.66	1.24	0.00	1.03	0.00
chromosome unknown open reading frame, human C15orf59	LOC100754105	100754105	0.04	0.00	0.00	0.00	0.00	0.00	2.28	0.00	0.00	0.00	0.00	0.00
Ig lambda chain V-I region BL2-like	LOC100754106	100754106	0.00	0.00	0.00	0.00	0.00	0.00						
chromosome unknown open reading frame, human C1orf50	LOC100754108	100754108	21.36	17.72	19.27	18.67	21.51	21.09	444.33	378.82	393.74	396.35	442.97	452.38

ragulator complex protein LAMTOR2-like	LOC100754114	100754114	0.00	0.00	0.00	0.00	0.00	0.00	0.00	0.00	0.00	0.00	0.00	1.87
peptidyl-prolyl cis-trans isomerase-like 2	LOC100754115	100754115	0.03	0.00	0.04	0.00	0.02	0.08	1.52	0.00	1.24	0.00	1.03	3.74
olfactory receptor-like protein OLF3	LOC100754121	100754121	0.00	0.00	0.00	0.00	0.00	0.00	0.00	0.00	0.00	0.00	0.00	0.00
olfactory receptor 10AG1-like	LOC100754126	100754126	0.00	0.00	0.00	0.00	0.00	0.00	0.00	0.00	0.00	0.00	0.00	0.00
pre-mRNA-splicing factor SLU7-like	LOC100754137	100754137	0.00	0.02	0.00	0.00	0.00	0.00	0.00	0.92	0.00	0.00	0.00	0.00
14-3-3 protein sigma	LOC100754138	100754138	0.04	0.03	0.00	0.00	0.00	0.03	0.00	0.00	0.00	1.16	0.00	0.00
Testis-expressed sequence 13A protein	LOC100754139	100754139	0.00	0.00	0.00	0.00	0.00	0.00	0.00	0.00	0.00	0.00	0.00	0.00
protocadherin gamma-B1-like	LOC100754141	100754141	0.52	0.58	0.58	0.66	0.56	0.67	60.00	69.54	70.80	89.24	68.31	86.92
histone H2A.Z pseudogene	LOC100754143	100754143	0.57	0.43	0.54	0.00	0.08	0.00	6.84	5.49	6.21	0.00	1.03	0.00
olfactory receptor 5K1-like	LOC100754148	100754148	0.00	0.00	0.00	0.00	0.00	0.00	0.00	0.00	0.00	0.00	0.00	0.00
potassium voltage-gated channel subfamily D member 2 pseudogene	LOC100754151	100754151	0.00	0.00	0.00	0.00	0.00	0.00	0.00	0.00	0.00	0.00	0.00	0.00
glutamyl-tRNA(Gln) amidotransferase subunit C, mitochondrial pseudogene	LOC100754157	100754157	0.04	0.22	0.00	0.00	0.13	0.23	0.00	2.75	0.00	0.00	3.10	1.87
PHD finger-like domain-containing protein 5A pseudogene	LOC100754158	100754158	0.39	0.19	0.26	0.00	0.22	0.25	3.80	1.83	2.48	0.00	2.07	2.80
vascular non-inflammatory molecule 3-like	LOC100754161	100754161	0.00	0.00	0.00	0.00	0.00	0.00	0.00	0.92	0.00	0.00	0.00	0.00
fatty acid-binding protein, intestinal	LOC100754164	100754164	0.00	0.00	0.00	0.00	0.00	0.00	0.00	0.00	0.00	0.00	0.00	0.00
zinc finger Ran-binding domain-containing protein 2-like	LOC100754166	100754166	8.52	8.06	7.85	7.43	8.37	8.31	259.76	230.59	207.43	152.98	144.90	153.29
calcium-binding mitochondrial carrier protein SCaMC-1	LOC100754168	100754168	19.96	20.31	18.79	18.96	20.95	19.67	1258.56	1298.43	1239.60	1226.14	1374.46	1297.32
olfactory receptor 4K14	LOC100754169	100754169	0.00	0.00	0.00	0.00	0.00	0.00	0.00	0.00	0.00	0.00	0.00	0.00

mitochondrial fission factor-like	LOC100 754170	100754170	0.34	0.21	0.28	0.21	0.23	0.21	8.35	4.58	6.21	5.79	6.21	7.48
myosin light polypeptide 6-like	LOC100 754174	100754174	0.00	0.00	0.00	0.00	0.00	0.00	0.00	0.00	0.00	0.00	0.00	0.00
uncharacterized LOC100754175	LOC100 754175	100754175	2.81	4.22	2.03	4.18	2.46	3.31	66.08	71.37	65.83	84.60	68.31	84.12
cytochrome P450 2C19-like	LOC100 754177	100754177	0.00	0.00	0.00	0.00	0.00	0.00	0.00	0.00	0.00	0.00	0.00	0.00
olfactory receptor 51F2-like	LOC100 754179	100754179	0.00	0.00	0.00	0.00	0.00	0.00	0.00	0.00	0.00	0.00	0.00	0.00
small EDRK-rich factor 2 pseudogene	LOC100 754183	100754183	0.00	0.00	0.00	0.00	0.00	0.00	0.00	0.00	0.00	0.00	0.00	0.00
olfactory receptor 8B12	LOC100 754186	100754186	0.00	0.00	0.00	0.00	0.00	0.00	0.00	0.00	0.00	0.00	0.00	0.00
vomeronasal type-1 receptor 2-like	LOC100 754200	100754200	0.00	0.00	0.00	0.00	0.00	0.00	0.00	0.00	0.00	0.00	0.00	0.00
polypyrimidine tract-binding protein 1 pseudogene	LOC100 754213	100754213	0.15	0.15	0.10	0.00	0.06	0.00	4.56	5.49	3.73	0.00	2.07	0.00
glyceraldehyde-3-phosphate dehydrogenase-like	LOC100 754219	100754219	0.00	0.00	0.00	0.00	0.00	0.00	0.00	0.92	0.00	0.00	0.00	0.93
60S ribosomal protein L35 pseudogene	LOC100 754220	100754220	0.00	0.00	0.00	0.00	0.00	0.00	0.00	0.00	0.00	0.00	0.00	0.00
actin-85C-like	LOC100 754243	100754243	0.00	0.00	0.00	0.00	0.00	0.04	0.00	0.00	0.00	0.00	0.00	0.93
protein MCM10 homolog	LOC100 754247	100754247	0.00	0.00	0.00	0.00	0.00	0.00	0.00	0.00	0.00	0.00	0.00	0.00
histone deacetylase 2 pseudogene	LOC100 754249	100754249	0.05	0.17	0.26	0.11	0.13	0.18	3.80	9.15	14.91	8.11	5.17	10.28
vomeronasal type-2 receptor 116-like	LOC100 754253	100754253	0.00	0.00	0.00	0.00	0.00	0.00	0.00	0.00	0.00	0.00	0.00	0.00
60S ribosomal protein L18 pseudogene	LOC100 754255	100754255	4.71	5.09	4.66	5.07	4.12	5.90	127.60	142.75	151.53	135.59	78.66	115.90
zinc finger protein 709 pseudogene	LOC100 754263	100754263	0.20	0.23	0.30	0.27	0.32	0.31	0.76	0.00	1.24	0.00	0.00	1.87
olfactory receptor 5B2	LOC100 754265	100754265	0.00	0.00	0.00	0.00	0.00	0.00	0.00	0.00	0.00	0.00	0.00	0.00

olfactory receptor 6	LOC100754267	100754267	0.00	0.00	0.00	0.00	0.00	0.00	0.00	0.00	0.00	0.00	0.00	0.00
olfactory receptor 4L1	LOC100754268	100754268	0.00	0.00	0.00	0.00	0.00	0.00	0.00	0.00	0.00	0.00	0.00	0.00
small ubiquitin-related modifier 2 pseudogene	LOC100754272	100754272	0.00	0.00	0.00	0.00	0.00	0.00	0.00	0.00	0.00	0.00	0.00	0.00
UDP-N-acetylglucosamine/UDP-glucose/GDP-mannose transporter-like	LOC100754273	100754273	0.59	0.63	0.38	0.41	0.18	0.45	12.91	14.64	8.69	9.27	3.10	10.28
U1 small nuclear ribonucleoprotein C pseudogene	LOC100754274	100754274	0.00	0.00	0.00	0.00	0.00	0.00	0.00	0.00	0.00	0.00	0.00	0.00
protein FAM156A/FAM156B-like	LOC100754277	100754277	0.00	0.00	0.00	0.00	0.00	0.00	0.00	0.00	0.00	0.00	0.00	0.00
vomeromodulin	LOC100754283	100754283	0.00	0.03	0.00	0.00	0.00	0.00	0.00	0.92	0.00	0.00	0.00	0.00
ATP synthase F(0) complex subunit C2, mitochondrial pseudogene	LOC100754287	100754287	0.00	0.00	0.23	0.11	0.00	0.09	3.04	1.83	6.21	2.32	2.07	2.80
E3 ubiquitin-protein ligase RNF216 pseudogene	LOC100754291	100754291	0.01	0.00	0.00	0.00	0.00	0.00	0.76	0.00	0.00	0.00	0.00	0.00
serine/threonine-protein kinase PLK4-like	LOC100754293	100754293	0.34	0.36	0.24	0.18	0.27	0.19	9.87	14.64	6.21	2.32	5.17	5.61
zinc finger protein 271-like	LOC100754294	100754294	0.29	0.44	0.60	0.78	0.88	0.90	9.87	20.13	18.63	24.34	32.08	30.84
nuclear speckle splicing regulatory protein 1 pseudogene	LOC100754295	100754295	0.39	0.29	0.12	0.42	0.14	0.49	8.35	8.24	2.48	11.59	4.14	11.22
olfactory receptor 52B4	LOC100754296	100754296	0.00	0.00	0.00	0.00	0.00	0.00	0.00	0.00	0.00	0.00	0.00	0.00
G-protein coupled receptor 26	LOC100754297	100754297	0.00	0.00	0.00	0.00	0.00	0.00	0.00	0.00	0.00	0.00	0.00	0.00
AP-3 complex subunit sigma-2	LOC100754298	100754298	82.66	90.01	89.83	96.62	102.82	111.00	2147.22	2220.79	2338.85	2373.47	2368.05	2560.99
melanoma-associated antigen 10-like	LOC100754300	100754300	0.24	0.21	0.06	0.25	0.19	0.10	0.00	0.00	0.00	0.00	0.00	0.00
taste receptor type 2 member 140-like	LOC100754307	100754307	0.00	0.00	0.00	0.00	0.00	0.00	0.00	0.00	0.00	0.00	0.00	0.00
granzyme B-like	LOC100754310	100754310	0.00	0.00	0.00	0.00	0.00	0.00	0.00	0.00	0.00	0.00	0.00	0.00

olfactory receptor 13J1	LOC100754315	100754315	0.00	0.00	0.00	0.00	0.00	0.00	0.00	0.00	0.00	0.00	0.00	0.00
olfactory receptor 8K3	LOC100754320	100754320	0.00	0.00	0.00	0.00	0.00	0.00	0.00	0.00	0.00	0.00	0.00	0.00
40S ribosomal protein S15 pseudogene	LOC100754323	100754323	0.00	0.00	0.00	0.00	0.00	0.00	0.00	0.00	0.00	0.00	0.00	0.00
olfactory receptor 7E24-like	LOC100754327	100754327	0.00	0.00	0.00	0.00	0.00	0.00	0.00	0.00	0.00	0.00	0.00	0.00
myeloid-associated differentiation marker	LOC100754329	100754329	0.00	0.00	0.00	0.00	0.00	0.00	0.00	0.00	0.00	0.00	0.00	0.00
AP-5 complex subunit mu-1	LOC100754336	100754336	8.54	8.13	9.57	8.90	11.71	11.36	298.50	287.32	341.57	323.34	403.64	403.78
olfactory receptor 8D2-like	LOC100754340	100754340	0.00	0.00	0.00	0.00	0.00	0.00	0.00	0.00	0.00	0.00	0.00	0.00
interleukin enhancer-binding factor 2 pseudogene	LOC100754341	100754341	0.35	0.35	0.21	0.12	0.25	0.15	10.63	8.24	4.97	2.32	8.28	3.74
golgin subfamily A member 7 pseudogene	LOC100754347	100754347	0.00	0.00	0.00	0.00	0.00	0.00	0.00	0.00	0.00	0.00	0.00	0.00
zinc finger protein 398	LOC100754349	100754349	3.73	3.73	4.12	4.06	3.98	3.68	435.98	450.20	505.53	487.91	471.95	457.05
olfactory receptor 6C65	LOC100754350	100754350	0.00	0.00	0.00	0.00	0.00	0.00	0.00	0.00	0.00	0.00	0.00	0.00
keratin-associated protein 20-2-like	LOC100754355	100754355	0.00	0.00	0.00	0.00	0.00	0.00	0.00	0.00	0.00	0.00	0.00	0.00
glutathione S-transferase-like	LOC100754357	100754357	0.00	0.00	0.00	0.00	0.00	0.00	0.00	0.00	0.00	0.00	0.00	0.00
chromosome unknown open reading frame, human C6orf57	LOC100754360	100754360	11.28	13.59	16.46	14.95	15.94	14.81	197.48	242.48	303.07	278.14	287.73	266.38
small proline-rich protein 2F-like	LOC100754363	100754363	0.00	0.00	0.00	0.00	0.00	0.00	0.00	0.00	0.00	0.00	0.00	0.00
protein NDRG3	LOC100754366	100754366	20.54	19.14	19.22	19.49	24.86	24.00	1374.77	1323.14	1320.34	1323.49	1669.43	1621.65
vomeronasal type-1 receptor 4-like	LOC100754373	100754373	0.00	0.00	0.00	0.00	0.00	0.00	0.00	0.00	0.00	0.00	0.00	0.00
putative deoxyribose-phosphate aldolase pseudogene	LOC100754374	100754374	0.00	0.00	0.00	0.00	0.00	0.00	0.00	0.00	0.00	0.00	0.00	0.00

egl nine homolog 3	LOC100 754377	100754377	0.00	0.00	0.00	0.00	0.00	0.00	0.00	0.00	0.00	0.00	0.00	0.00
actin-related protein 10 pseudogene	LOC100 754378	100754378	0.02	0.00	0.00	0.00	0.00	0.00	0.76	0.00	0.00	0.00	0.00	0.00
F-box/WD repeat-containing protein 2-like	LOC100 754385	100754385	14.65	14.62	17.61	20.03	20.27	18.85	0.00	0.00	0.00	0.00	0.00	0.00
uncharacterized LOC100754387	LOC100 754387	100754387	0.00	0.00	0.00	0.00	0.00	0.00	0.00	0.00	0.00	0.00	0.00	0.00
peptidyl-prolyl cis-trans isomerase A pseudogene	LOC100 754388	100754388	0.10	0.08	0.00	0.10	0.00	0.00	0.00	0.00	0.00	1.16	0.00	0.00
60S ribosomal protein L7a pseudogene	LOC100 754391	100754391	0.00	0.00	0.00	0.00	0.00	0.00	0.00	0.00	0.00	0.00	0.00	0.00
histone H4	LOC100 754396	100754396	0.27	0.14	0.41	0.10	0.07	0.07	3.04	1.83	4.97	1.16	2.07	0.93
Ig lambda-2 chain V region-like	LOC100 754398	100754398	0.00	0.00	0.00	0.00	0.00	0.00						
keratin, type I cytoskeletal 18 pseudogene	LOC100 754405	100754405	0.00	0.00	0.00	0.00	0.00	0.00	0.00	0.00	0.00	0.00	0.00	0.00
olfactory receptor-like protein OLF3	LOC100 754411	100754411	0.00	0.00	0.00	0.00	0.00	0.00	0.00	0.00	0.00	0.00	0.00	0.00
olfactory receptor 10AG1-like	LOC100 754416	100754416	0.00	0.00	0.00	0.00	0.00	0.00	0.00	0.00	0.00	0.00	0.00	0.00
39S ribosomal protein L37, mitochondrial pseudogene	LOC100 754428	100754428	0.04	0.00	0.00	0.06	0.05	0.00	0.76	0.00	0.00	1.16	1.03	0.00
uncharacterized LOC100754429	LOC100 754429	100754429	0.07	0.05	0.04	0.06	0.02	0.04	5.32	5.49	3.73	5.79	2.07	3.74
dihydrolipoyllysine-residue succinyltransferase component of 2-oxoglutarate dehydrogenase complex, mitochondrial pseudogene	LOC100 754435	100754435	2.49	2.15	2.27	1.34	1.59	1.45	148.87	114.38	109.30	68.38	62.10	62.62
voltage-dependent anion-selective channel protein 1 pseudogene	LOC100 754438	100754438	0.23	0.40	0.35	0.33	0.96	0.62	11.39	19.22	23.60	16.22	41.40	26.17
chromosome-associated kinesin KIF4A	LOC100 754439	100754439	0.00	0.00	0.00	0.00	0.00	0.00	0.00	0.00	0.00	0.00	0.00	0.00
60S ribosomal protein L23-like	LOC100 754445	100754445	4.97	3.56	1.99	2.04	2.71	1.84	25.06	18.30	9.94	6.95	12.42	8.41
60S ribosomal protein L23a pseudogene	LOC100 754447	100754447	0.07	0.17	0.00	0.00	0.20	0.09	1.52	1.83	0.00	0.00	3.10	1.87

60S ribosomal protein L37 pseudogene	LOC100754450	100754450	1.50	0.52	1.45	0.16	0.84	1.55	6.84	1.83	6.21	0.00	3.10	6.54
fatty acid-binding protein, intestinal	LOC100754451	100754451	2.94	3.23	0.90	1.45	1.96	1.76	69.88	81.44	23.60	35.93	48.64	42.99
60S acidic ribosomal protein P1-like	LOC100754453	100754453	1478.70	1514.30	1535.80	897.71	432.67	510.43	7880.98	7616.74	7760.56	4642.63	2723.05	3153.58
olfactory receptor 4K3-like	LOC100754455	100754455	0.00	0.13	0.23	0.22	0.34	0.30	0.00	2.75	6.21	6.95	12.42	11.22
SOSS complex subunit B2 pseudogene	LOC100754456	100754456	0.00	0.00	0.00	0.00	0.00	0.00	0.00	0.00	0.00	0.00	0.00	0.00
guanine nucleotide-binding protein G(s) subunit alpha-like	LOC100754457	100754457	1.26	1.15	0.37	0.72	0.65	0.39	80.51	71.37	33.54	42.88	41.40	24.30
cytochrome P450 2C28	LOC100754462	100754462	0.06	0.05	0.03	0.00	0.03	0.00	2.28	1.83	3.73	0.00	1.03	0.00
olfactory receptor 51F2	LOC100754464	100754464	0.00	0.00	0.00	0.00	0.00	0.00	0.00	0.00	0.00	0.00	0.00	0.00
olfactory receptor 145-like	LOC100754472	100754472	0.00	0.00	0.00	0.00	0.00	0.00	0.00	0.00	0.00	0.00	0.00	0.00
histone H2B type F-M-like	LOC100754482	100754482	0.00	0.00	0.00	0.00	0.00	0.00	0.00	0.00	0.00	0.00	0.00	0.00
nicotinamide phosphoribosyltransferase pseudogene	LOC100754483	100754483	0.00	0.00	0.00	0.00	0.00	0.00	0.00	0.00	0.00	0.00	0.00	0.00
vomeronasal type-2 receptor 116-like	LOC100754485	100754485	0.00	0.00	0.00	0.00	0.00	0.00	0.00	0.00	0.00	0.00	0.00	0.00
chromosome unknown open reading frame, human C11orf88	LOC100754486	100754486	0.12	0.12	0.04	0.19	0.27	0.00	7.60	4.58	1.24	6.95	11.38	1.87
chromatin target of PRMT1 protein pseudogene	LOC100754495	100754495	0.00	0.00	0.00	0.00	0.00	0.09	0.00	0.00	0.00	0.00	0.00	0.00
60S ribosomal protein L38 pseudogene	LOC100754503	100754503	0.00	0.00	0.00	0.00	0.00	0.00	0.00	0.00	0.00	0.00	0.00	0.00
eukaryotic translation initiation factor 5A-1-like	LOC100754508	100754508	0.00	0.00	0.00	0.00	0.00	0.00	0.00	0.00	0.00	0.00	0.00	0.00
cyclic GMP-AMP synthase-like	LOC100754516	100754516	0.81	0.78	0.49	0.46	0.53	0.40	58.48	64.97	43.47	40.56	41.40	28.97
thymosin beta-10 pseudogene	LOC100754517	100754517	0.00	0.00	0.00	0.00	0.00	0.00	0.00	0.92	0.00	0.00	0.00	0.00

histone-binding protein RBBP7 pseudogene	LOC100 754518	100754518	35.89	36.33	34.86	38.67	36.91	39.88	195.20	176.60	157.75	185.43	155.25	176.65
solute carrier family 25 member 51	LOC100 754521	100754521	15.52	16.00	15.81	16.99	20.63	20.21	965.37	1001.96	1001.12	1065.05	1300.98	1289.84
uncharacterized LOC100754524	LOC100 754524	100754524	0.00	0.00	0.00	0.00	0.08	0.07	0.00	0.00	0.00	0.00	0.00	0.00
actin, cytoplasmic 1-like	LOC100 754525	100754525	0.14	0.03	0.09	0.00	0.16	0.04	3.80	0.92	2.48	0.00	5.17	1.87
FERM domain-containing protein 8 pseudogene	LOC100 754526	100754526	0.43	0.27	0.27	0.58	0.37	0.45	15.19	7.32	8.69	19.70	12.42	14.95
N-acetyllactosaminide alpha-1,3-galactosyltransferase	LOC100 754535	100754535	0.04	0.02	0.00	0.00	0.00	0.00	2.28	0.92	0.00	1.16	0.00	0.00
protocadherin-11 X-linked	LOC100 754537	100754537	0.00	0.00	0.00	0.00	0.00	0.00	0.00	0.00	0.00	0.00	0.00	0.00
vomeronasal type-2 receptor 116-like	LOC100 754540	100754540	0.00	0.00	0.00	0.00	0.00	0.00	0.00	0.00	0.00	0.00	0.00	0.00
melanoma-associated antigen 10	LOC100 754546	100754546	0.00	0.00	0.00	0.00	0.00	0.00	0.00	0.00	0.00	0.00	0.00	0.00
ATPase ASNA1 pseudogene	LOC100 754550	100754550	0.34	0.39	0.27	0.27	0.36	0.17	9.11	10.98	7.45	9.27	9.31	4.67
olfactory receptor 5B3	LOC100 754552	100754552	0.00	0.00	0.00	0.00	0.00	0.00	0.00	0.00	0.00	0.00	0.00	0.00
poly [ADP-ribose] polymerase 14-like	LOC100 754553	100754553	0.07	0.00	0.00	0.05	0.05	0.04	0.76	0.00	1.24	2.32	3.10	0.00
olfactory receptor 226	LOC100 754554	100754554	0.00	0.00	0.00	0.00	0.00	0.00	0.00	0.00	0.00	0.00	0.00	0.00
olfactory receptor 4L1	LOC100 754555	100754555	0.00	0.00	0.00	0.00	0.00	0.00	0.00	0.00	0.00	0.00	0.00	0.00
uncharacterized LOC100754558	LOC100 754558	100754558	0.00	0.00	0.00	0.00	0.00	0.00	0.00	0.00	0.00	0.00	0.00	0.00
uncharacterized LOC100754561	LOC100 754561	100754561	0.00	0.00	0.00	0.00	0.00	0.00	0.00	0.00	0.00	0.00	0.00	0.00
ferritin heavy chain	LOC100 754563	100754563	0.00	0.00	0.00	0.00	0.00	0.00	0.00	0.00	0.00	0.00	0.00	0.00
stromelysin-1-like	LOC100 754565	100754565	0.29	0.15	0.00	0.00	0.00	0.00	16.71	8.24	0.00	0.00	0.00	0.00

3-hydroxyisobutyrate dehydrogenase, mitochondrial-like	LOC100754568	100754568	0.03	0.00	0.00	0.00	0.00	0.00	0.76	0.92	0.00	0.00	0.00	0.00
protein Mis18-beta-like	LOC100754569	100754569	0.26	0.19	0.17	0.00	0.07	0.30	3.80	2.75	2.48	0.00	1.03	3.74
transforming acidic coiled-coil-containing protein 2-like	LOC100754572	100754572	17.23	17.01	17.86	17.55	14.18	14.17	1364.89	1338.69	1383.68	1394.18	1124.00	1143.10
chromosome unknown open reading frame, human C17orf70	LOC100754573	100754573	23.70	23.80	22.87	17.29	11.40	12.55	2198.87	2218.96	2164.96	1641.03	1078.46	1211.33
gastrula zinc finger protein XlCGF26.1-like	LOC100754579	100754579	0.04	0.09	0.05	0.11	0.08	0.14	3.04	4.58	1.24	8.11	7.24	9.35
olfactory receptor 52B4	LOC100754583	100754583	0.00	0.00	0.00	0.00	0.00	0.00	0.00	0.00	0.00	0.00	0.00	0.00
zinc finger protein 91-like	LOC100754584	100754584	0.00	0.00	0.00	0.00	0.00	0.00	0.00	0.00	0.00	0.00	0.00	0.00
non-histone chromosomal protein HMG-17 pseudogene	LOC100754587	100754587	0.00	0.00	0.00	0.00	0.00	0.00	0.00	0.00	0.00	0.00	0.00	0.00
ATP-dependent RNA helicase DDX19A-like	LOC100754589	100754589	25.70	24.59	26.04	27.37	25.57	25.04	0.00	0.00	0.00	0.00	0.00	0.00
taste receptor type 2 member 136-like	LOC100754594	100754594	0.00	0.00	0.00	0.00	0.00	0.00	0.00	0.00	0.00	0.00	0.00	0.00
granzyme C-like	LOC100754595	100754595	0.00	0.00	0.00	0.00	0.00	0.00	0.00	0.00	0.00	0.00	0.00	0.00
60S ribosomal protein L24 pseudogene	LOC100754597	100754597	0.43	0.52	0.47	0.00	0.70	0.27	4.56	5.49	4.97	0.00	7.24	2.80
olfactory receptor 13C2	LOC100754601	100754601	0.00	0.00	0.00	0.00	0.00	0.00	0.00	0.00	0.00	0.00	0.00	0.00
olfactory receptor 5T3-like	LOC100754607	100754607	0.00	0.00	0.00	0.00	0.00	0.00	0.00	0.00	0.00	0.00	0.00	0.00
olfactory receptor 18-like	LOC100754612	100754612	0.00	0.00	0.00	0.00	0.00	0.00	0.00	0.00	0.00	0.00	0.00	0.00
UBA-like domain-containing protein 2 pseudogene	LOC100754613	100754613	0.00	0.00	0.00	0.00	0.00	0.00	0.00	0.00	0.00	0.00	0.00	0.00
phospholipid hydroperoxide glutathione peroxidase, mitochondrial-like	LOC100754616	100754616	0.00	0.00	0.00	0.05	0.00	0.00	0.76	0.92	0.00	2.32	0.00	0.00
short coiled-coil protein pseudogene	LOC100754623	100754623	0.12	0.43	0.00	0.19	0.17	0.08	0.76	2.75	0.00	1.16	1.03	0.00

olfactory receptor 8D2-like	LOC100754624	100754624	0.00	0.00	0.00	0.00	0.00	0.00	0.00	0.00	0.00	0.00	0.00	0.00
N-alpha-acetyltransferase 35, NatC auxiliary subunit pseudogene	LOC100754627	100754627	0.03	0.00	0.08	0.06	0.00	0.03	1.52	0.00	4.97	3.48	0.00	1.87
zinc finger protein 282-like	LOC100754633	100754633	20.24	20.68	23.06	18.24	16.69	14.78	1127.16	1161.18	1324.06	1073.16	998.76	936.54
olfactory receptor 6C74	LOC100754634	100754634	0.00	0.00	0.00	0.00	0.00	0.00	0.00	0.00	0.00	0.00	0.00	0.00
actin-related protein 2/3 complex subunit 1B pseudogene	LOC100754635	100754635	1.86	2.39	2.79	1.52	2.64	1.81	132.16	116.21	142.84	85.76	78.66	88.79
keratin-associated protein 20-2-like	LOC100754638	100754638	0.00	0.00	0.00	0.00	0.00	0.00	0.00	0.00	0.00	0.00	0.00	0.00
vomeronasal type-2 receptor 26-like	LOC100754639	100754639	0.01	0.00	0.00	0.00	0.00	0.00	0.00	0.00	0.00	0.00	0.00	0.00
small proline-rich protein 2H	LOC100754646	100754646	1.11	0.82	0.90	1.09	1.56	1.54	25.06	21.05	21.12	23.18	42.43	42.99
multifunctional protein ADE2 pseudogene	LOC100754657	100754657	0.00	0.00	0.00	0.00	0.00	0.00	0.00	0.00	0.00	0.00	0.00	0.00
olfactory receptor 3A1	LOC100754660	100754660	0.00	0.05	0.05	0.05	0.00	0.00	0.76	1.83	1.24	1.16	0.00	0.00
histo-blood group ABO system transferase 1-like	LOC100754666	100754666	0.13	0.14	0.36	0.56	0.02	0.01	6.08	6.41	17.39	26.66	1.03	0.00
histone H2A.Z	LOC100754667	100754667	46.76	50.56	39.62	23.70	18.31	12.78	319.01	316.60	234.75	152.98	111.78	80.38
non-histone chromosomal protein HMG-17 pseudogene	LOC100754668	100754668	0.00	0.00	0.00	0.00	0.00	0.00	0.00	0.00	0.00	0.00	0.00	0.00
spindlin-2-like	LOC100754669	100754669	0.00	0.00	0.00	0.00	0.00	0.00	0.00	0.00	0.00	0.00	0.00	0.00
1,2-dihydroxy-3-keto-5-methylthiopentene dioxygenase	LOC100754671	100754671	0.07	0.00	0.12	0.11	0.00	0.00	0.76	0.00	1.24	1.16	0.00	0.00
ubiquinol-cytochrome-c reductase complex assembly factor 1	LOC100754679	100754679	45.01	43.37	47.28	47.70	55.68	56.82	3270.58	3189.81	3428.16	3437.36	3989.87	4037.77
elongation factor 1-gamma-like	LOC100754680	100754680	6.93	8.01	7.72	4.30	3.87	3.70	89.63	106.14	105.58	70.69	76.59	60.75
histone H3.1	LOC100754683	100754683	1.16	1.14	0.23	0.52	0.46	0.18	18.23	21.96	6.21	8.11	8.28	2.80

60S ribosomal protein L18 pseudogene	LOC100 754684	100754684	0.00	0.00	0.00	0.00	0.00	0.00	0.00	0.00	0.00	0.00	0.00	0.00
uncharacterized LOC100754685	LOC100 754685	100754685	0.00	0.00	0.00	0.00	0.00	0.00	0.00	0.00	0.00	0.00	0.00	0.00
disintegrin and metalloproteinase domain-containing protein 18-like	LOC100 754689	100754689	0.00	0.00	0.00	0.00	0.00	0.00	0.00	0.00	0.00	0.00	0.00	0.00
mediator of RNA polymerase II transcription subunit 28 pseudogene	LOC100 754691	100754691	0.00	0.00	0.00	0.00	0.00	0.00	0.00	0.00	0.00	0.00	0.00	0.93
uncharacterized LOC100754693	LOC100 754693	100754693	0.14	0.06	0.08	0.00	0.07	0.06	5.32	1.83	1.24	1.16	4.14	4.67
actin, cytoplasmic 1-like	LOC100 754694	100754694	0.00	0.00	0.00	0.00	0.00	0.00	0.00	0.00	0.00	0.00	0.00	0.00
olfactory receptor 6B1	LOC100 754700	100754700	0.00	0.00	0.00	0.00	0.00	0.00	0.00	0.00	0.00	0.00	0.00	0.00
olfactory receptor 10AG1-like	LOC100 754704	100754704	0.00	0.00	0.00	0.00	0.00	0.00	0.00	0.00	0.00	0.00	0.00	0.00
chromosome unknown open reading frame, human C6orf15	LOC100 754705	100754705	0.62	0.40	0.14	0.45	0.34	0.26	7.60	7.32	4.97	5.79	10.35	6.54
chromosome unknown open reading frame, human C11orf31	LOC100 754707	100754707	50.42	54.91	44.82	34.10	31.23	27.21	888.66	967.19	758.92	573.67	535.09	457.05
40S ribosomal protein S11-like	LOC100 754710	100754710	0.00	0.00	0.00	0.00	0.00	0.00	0.00	0.00	0.00	0.00	0.00	0.00
protocadherin gamma-B6-like	LOC100 754716	100754716	0.02	0.00	0.00	0.00	0.00	0.00	0.76	0.00	0.00	0.00	0.00	0.00
uncharacterized LOC100754720	LOC100 754720	100754720	11.41	11.04	11.07	13.01	12.89	13.97	250.65	240.65	240.96	273.51	272.20	293.49
60S ribosomal protein L17 pseudogene	LOC100 754721	100754721	0.14	0.06	0.15	0.16	0.00	0.17	0.76	0.00	1.24	1.16	0.00	0.93
protein disulfide-isomerase TMX3 pseudogene	LOC100 754724	100754724	0.00	0.00	0.00	0.00	0.00	0.00	0.00	0.00	0.00	0.00	0.00	0.00
Friend virus susceptibility protein 1	LOC100 754726	100754726	37.96	34.39	47.67	46.68	38.93	40.51	988.92	943.40	1204.82	1160.08	1078.46	1149.64
60S ribosomal protein L26-like	LOC100 754729	100754729	0.21	0.27	0.06	0.27	0.15	0.30	4.56	6.41	1.24	5.79	4.14	7.48
60S ribosomal protein L7a pseudogene	LOC100 754732	100754732	0.98	1.06	0.77	0.77	0.76	0.52	4.56	2.75	1.24	2.32	1.03	2.80

25-hydroxycholesterol 7-alpha-hydroxylase	LOC100754734	100754734	7.42	7.29	5.76	5.48	8.91	8.45	726.88	696.34	571.36	521.51	837.30	818.77
olfactory receptor 4K14	LOC100754741	100754741	0.00	0.00	0.00	0.00	0.00	0.00	0.00	0.00	0.00	1.16	0.00	0.93
suppressor of G2 allele of SKP1 homolog pseudogene	LOC100754742	100754742	1.42	1.06	0.72	0.92	1.00	1.40	38.74	28.37	18.63	26.66	26.91	38.32
low-density lipoprotein receptor-related protein 10 pseudogene	LOC100754745	100754745	0.00	0.00	0.00	0.00	0.00	0.00	0.00	0.00	0.00	0.00	0.00	0.00
C-Myc-binding protein-like	LOC100754746	100754746	0.54	0.60	0.75	0.40	0.98	0.84	6.08	9.15	4.97	11.59	8.28	6.54
olfactory receptor 51S1	LOC100754750	100754750	0.00	0.00	0.00	0.00	0.00	0.00	0.00	0.00	0.00	0.00	0.00	0.00
hexokinase-2 pseudogene	LOC100754756	100754756	0.01	0.00	0.04	0.02	0.00	0.02	0.76	0.00	2.48	1.16	0.00	0.93
olfactory receptor 8B12-like	LOC100754758	100754758	0.00	0.00	0.00	0.00	0.00	0.00	0.00	0.00	0.00	0.00	0.00	0.00
phosphoglycerate kinase 1 pseudogene	LOC100754762	100754762	0.21	0.20	0.25	0.15	0.27	0.24	14.43	11.90	14.91	10.43	17.59	14.95
histone H2A-Bbd type 1	LOC100754763	100754763	0.00	0.00	0.00	0.00	0.00	0.00	0.00	0.00	0.00	0.00	0.00	0.00
Ig heavy chain V-II region SESS-like	LOC100754764	100754764	0.00	0.00	0.00	0.00	0.00	0.00						
complement factor H-related protein 4-like	LOC100754768	100754768	5.33	4.81	4.40	3.31	4.46	4.65	186.85	174.77	152.78	122.85	156.28	171.04
olfactory receptor 145-like	LOC100754776	100754776	0.00	0.00	0.00	0.00	0.00	0.00	0.00	0.00	0.00	0.00	0.00	0.00
40S ribosomal protein SA pseudogene	LOC100754787	100754787	0.00	0.00	0.00	0.00	0.00	0.00	0.00	0.00	0.00	0.00	0.00	0.00
signal transducer CD24 pseudogene	LOC100754790	100754790	0.00	0.00	0.00	0.00	0.00	0.00	0.00	0.00	0.00	0.00	0.00	0.00
zinc finger protein 300-like	LOC100754794	100754794	2.22	1.91	1.70	1.74	2.10	1.94	201.28	183.01	163.96	156.45	190.44	175.72
60S ribosomal protein L31-like	LOC100754795	100754795	0.00	0.00	0.00	0.00	0.00	0.00	0.00	0.00	0.00	0.00	0.00	0.00
chromosome unknown open reading frame, human C19orf26	LOC100754798	100754798	2.03	1.89	1.63	1.03	0.48	0.43	260.52	256.21	209.91	147.18	82.80	98.14

transmembrane protein 217	LOC100754804	100754804	0.68	0.62	0.45	0.68	0.81	0.57	13.67	12.81	9.94	18.54	18.63	12.15
BMP/retinoic acid-inducible neural-specific protein 1 pseudogene	LOC100754805	100754805	0.00	0.00	0.00	0.00	0.00	0.00	0.00	0.00	0.00	0.00	0.00	0.00
cytochrome P450 4F6	LOC100754807	100754807	0.70	0.90	0.34	0.25	0.07	0.05	74.43	82.35	34.78	31.29	19.66	22.43
3-ketoacyl-CoA thiolase B, peroxisomal-like	LOC100754813	100754813	12.28	11.56	14.08	12.70	9.24	10.74	392.68	381.57	462.06	441.55	352.93	405.65
uncharacterized LOC100754820	LOC100754820	100754820	0.05	0.08	0.03	0.00	0.10	0.04	2.28	2.75	1.24	0.00	4.14	1.87
uncharacterized LOC100754823	LOC100754823	100754823	0.00	0.00	0.00	0.00	0.00	0.00	0.76	0.92	0.00	1.16	0.00	0.00
vomeronasal type-1 receptor 4-like	LOC100754829	100754829	0.00	0.00	0.00	0.00	0.00	0.00	0.00	0.00	0.00	0.00	0.00	0.00
conserved oligomeric Golgi complex subunit 5	LOC100754837	100754837	14.33	14.86	15.76	16.55	20.14	19.03	761.06	810.72	852.07	876.14	1092.95	992.62
olfactory receptor 5B2	LOC100754840	100754840	0.00	0.00	0.00	0.00	0.00	0.00	0.00	0.00	0.00	0.00	0.00	0.00
poly [ADP-ribose] polymerase 9-like	LOC100754841	100754841	0.23	0.22	0.35	0.35	0.50	0.49	12.91	10.98	24.84	17.38	24.84	27.11
olfactory receptor 10A5-like	LOC100754842	100754842	0.00	0.00	0.00	0.00	0.00	0.00	0.00	0.00	0.00	0.00	0.00	0.00
SIGLEC family-like protein 1	LOC100754844	100754844	0.00	0.00	0.00	0.00	0.00	0.00	0.00	0.00	0.00	0.00	0.00	0.00
heterogeneous nuclear ribonucleoprotein C-like	LOC100754847	100754847	0.00	0.00	0.00	0.00	0.05	0.00	0.00	0.00	0.00	0.00	1.03	0.00
60S ribosomal protein L13 pseudogene	LOC100754850	100754850	0.00	0.00	0.00	0.00	0.00	0.00	0.00	0.00	0.00	0.00	0.00	0.00
	LOC100754853	100754853	58.24	57.30	74.52	71.99	71.56	73.80	2226.21	2212.55	2836.93	2701.44	2689.93	2738.58
polypyrimidine tract-binding protein 1 pseudogene	LOC100754857	100754857	0.00	0.00	0.00	0.00	0.00	0.00	0.00	0.00	0.00	0.00	0.00	0.00
vomeromodulin-like	LOC100754858	100754858	0.00	0.01	0.00	0.00	0.00	0.00	0.00	0.00	0.00	0.00	0.00	0.00
eukaryotic translation initiation factor 4B pseudogene	LOC100754862	100754862	0.08	0.17	0.07	0.12	0.10	0.19	3.80	7.32	2.48	4.64	5.17	9.35

chromosome unknown open reading frame, human C11orf74	LOC100754866	100754866	17.43	21.55	16.80	18.21	21.49	19.43	683.59	822.62	617.32	694.19	787.62	736.52
olfactory receptor 52B4	LOC100754870	100754870	0.00	0.00	0.00	0.00	0.00	0.00	0.00	0.00	0.00	0.00	0.00	0.00
vomeronasal type-1 receptor 4-like	LOC100754871	100754871	0.00	0.00	0.00	0.00	0.00	0.00	0.00	0.00	0.00	0.00	0.00	0.00
uncharacterized LOC100754872	LOC100754872	100754872	16.43	15.20	31.40	40.98	80.84	88.21	244.57	235.16	455.85	582.94	1184.02	1210.40
non-histone chromosomal protein HMG-17 pseudogene	LOC100754873	100754873	0.00	0.00	0.00	0.00	0.00	0.00	0.00	0.00	0.00	0.00	0.00	0.00
proteasome subunit alpha type-3 pseudogene	LOC100754878	100754878	0.00	0.00	0.00	0.00	0.00	0.00	0.00	0.00	0.00	0.00	0.00	0.00
melanoma-associated antigen D2	LOC100754879	100754879	9.11	9.58	12.19	10.87	9.03	10.12	552.94	590.20	763.88	667.54	566.14	621.56
KH domain-containing, RNA-binding, signal transduction-associated protein 1 pseudogene	LOC100754880	100754880	0.18	0.43	0.04	0.16	0.21	0.16	4.56	12.81	1.24	4.64	6.21	4.67
taste receptor type 2 member 109-like	LOC100754882	100754882	0.00	0.00	0.00	0.00	0.00	0.00	0.00	0.00	0.00	0.00	0.00	0.00
granzyme-like protein 2	LOC100754885	100754885	0.00	0.00	0.00	0.00	0.00	0.00	0.00	0.00	0.00	0.00	0.00	0.00
60S ribosomal protein L36a	LOC100754891	100754891	138.99	141.47	128.77	133.11	141.35	157.49	120.01	92.42	89.43	62.58	90.04	93.47
olfactory receptor 4C5-like	LOC100754894	100754894	0.00	0.00	0.00	0.00	0.00	0.00	0.00	0.00	0.00	0.00	0.00	0.00
60 kDa heat shock protein, mitochondrial pseudogene	LOC100754896	100754896	0.00	0.00	0.00	0.00	0.00	0.00	0.00	0.00	0.00	0.00	0.00	0.00
bcl-2-like protein 13	LOC100754898	100754898	57.42	58.28	66.45	64.30	60.54	64.14	4361.27	4395.82	5158.39	5136.33	4840.63	5099.56
olfactory receptor 998	LOC100754899	100754899	0.00	0.00	0.00	0.00	0.00	0.00	0.00	0.00	0.00	0.00	0.00	0.00
ubiquitin-like modifier-activating enzyme ATG7	LOC100754902	100754902	31.92	28.99	38.98	34.54	32.85	36.05	1222.86	1113.60	1486.78	1281.77	1277.17	1413.22
olfactory receptor 7E24-like	LOC100754904	100754904	0.00	0.00	0.00	0.00	0.00	0.00	0.00	0.00	0.00	0.00	0.00	0.00
glycylpeptide N-tetradecanoyltransferase 1-like	LOC100754906	100754906	0.46	0.24	0.45	0.47	0.48	0.49	27.34	14.64	23.60	28.97	27.94	30.84

40S ribosomal protein S2 pseudogene	LOC100754909	100754909	0.00	0.00	0.00	0.00	0.00	0.00	0.00	0.00	0.00	0.00	0.00	0.00
60S ribosomal protein L7a pseudogene	LOC100754910	100754910	1.03	1.92	1.35	0.70	1.58	0.39	4.56	12.81	8.69	3.48	11.38	2.80
40S ribosomal protein SA-like	LOC100754912	100754912	0.00	0.06	0.00	0.00	0.00	0.00	0.00	0.92	0.00	0.00	0.00	0.00
chromosome unknown open reading frame, human C14orf105	LOC100754915	100754915	0.00	0.00	0.00	0.00	0.00	0.00	1.52	0.00	0.00	2.32	1.03	0.93
zinc finger protein 120-like	LOC100754918	100754918	0.05	0.06	0.00	0.00	0.00	0.00	2.28	2.75	0.00	0.00	0.00	0.00
disintegrin and metalloproteinase domain-containing protein 29-like	LOC100754921	100754921	0.00	0.00	0.00	0.00	0.00	0.00	0.00	0.00	0.00	0.00	0.00	0.00
spermatogenesis-associated protein 4 pseudogene	LOC100754922	100754922	0.00	0.00	0.00	0.00	0.00	0.00	0.00	0.00	0.00	0.00	0.00	0.00
olfactory receptor 9K2-like	LOC100754927	100754927	0.00	0.00	0.00	0.00	0.00	0.00	0.00	0.00	0.00	0.00	0.00	0.00
keratin-associated protein 20-2-like	LOC100754931	100754931	0.00	0.00	0.00	0.00	0.00	0.00	0.00	0.00	0.00	0.00	0.00	0.00
claudin-3-like	LOC100754933	100754933	0.00	0.00	0.00	0.00	0.00	0.00	0.00	0.00	0.00	0.00	0.00	0.00
placenta-specific gene 8 protein-like	LOC100754936	100754936	0.00	0.00	0.00	0.00	0.33	0.46	0.00	0.00	0.00	0.00	2.07	2.80
cyclin-dependent kinases regulatory subunit 2 pseudogene	LOC100754937	100754937	0.77	2.25	3.39	1.80	2.63	1.42	3.80	10.98	16.15	8.11	12.42	6.54
immunity-related GTPase family M protein 1-like	LOC100754939	100754939	0.01	0.02	0.00	0.00	0.04	0.05	0.76	0.92	0.00	0.00	2.07	1.87
galectin-2-like	LOC100754948	100754948	0.00	0.00	0.00	0.00	0.00	0.00	0.00	0.00	0.00	0.00	0.00	0.00
olfactory receptor 3A1	LOC100754954	100754954	0.03	0.14	0.11	0.10	0.09	0.08	2.28	2.75	2.48	3.48	4.14	2.80
spermine synthase pseudogene	LOC100754960	100754960	0.66	0.73	0.77	0.31	0.54	0.43	13.67	15.56	14.91	4.64	9.31	7.48
keratin, type I cytoskeletal 12	LOC100754962	100754962	0.00	0.00	0.00	0.00	0.00	0.00	0.00	0.00	0.00	0.00	0.00	0.00
AKT-interacting protein pseudogene	LOC100754965	100754965	0.00	0.04	0.00	0.00	0.00	0.00	0.00	0.92	2.48	0.00	1.03	0.00

lipid phosphate phosphatase-related protein type 2	LOC100754967	100754967	0.04	0.09	0.14	0.05	0.05	0.04	6.84	10.07	13.66	8.11	5.17	7.48
60S ribosomal protein L17 pseudogene	LOC100754971	100754971	0.16	0.11	0.22	0.03	0.13	0.12	0.76	0.00	1.24	0.00	0.00	0.93
histone H1.1	LOC100754977	100754977	0.00	0.00	0.00	0.00	0.00	0.00	0.00	0.00	0.00	0.00	0.00	0.00
immunoglobulin iota chain-like	LOC100754979	100754979	0.11	0.00	0.00	0.00	0.00	0.00	0.00	0.00	0.00	0.00	0.00	0.00
60S ribosomal protein L26-like 1 pseudogene	LOC100754980	100754980	0.00	0.00	0.00	0.00	0.00	0.00	0.00	0.00	0.00	0.00	0.00	0.00
chromobox protein homolog 3-like	LOC100754983	100754983	0.54	0.30	0.59	0.51	0.59	0.52	0.00	3.66	3.73	3.48	2.07	2.80
periphilin-1-like	LOC100754987	100754987	0.18	0.42	0.15	0.13	0.47	0.43	121.53	129.93	115.51	147.18	138.69	128.05
transaldolase pseudogene	LOC100754988	100754988	0.00	0.00	0.00	0.00	0.00	0.00	0.00	0.00	0.00	0.00	0.00	0.00
olfactory receptor 2A5	LOC100754993	100754993	0.00	0.00	0.00	0.00	0.00	0.00	0.00	0.00	0.00	0.00	0.00	0.00
olfactory receptor 10AG1-like	LOC100754997	100754997	0.00	0.00	0.00	0.00	0.00	0.00	0.00	0.00	0.00	0.00	0.00	0.00
zinc finger protein 120-like	LOC100755006	100755006	3.81	3.43	4.23	4.51	4.85	4.60	462.56	434.64	535.34	588.73	627.20	575.76
actin-related protein 2/3 complex subunit 1A pseudogene	LOC100755008	100755008	0.00	0.00	0.00	0.00	0.00	0.00	0.00	0.00	0.00	0.00	0.00	0.00
orphan sodium- and chloride-dependent neurotransmitter transporter NTT5-like	LOC100755013	100755013	0.00	0.00	0.00	0.00	0.00	0.00	0.00	0.00	0.00	0.00	0.00	0.00
U6 snRNA-associated Sm-like protein LSm5 pseudogene	LOC100755017	100755017	0.00	0.00	0.00	0.00	0.00	0.00	0.00	0.00	0.00	0.00	0.00	0.00
ubiquitin-like protein FUBI	LOC100755019	100755019	6.45	6.78	6.11	4.78	3.08	2.95	76.71	66.80	60.86	40.56	37.26	42.99
glutathione S-transferase A1-like	LOC100755023	100755023	0.00	0.00	0.00	0.00	0.00	0.00	0.00	0.00	0.00	0.00	0.00	0.00
trace amine-associated receptor 2	LOC100755026	100755026	0.00	0.00	0.00	0.00	0.00	0.00	0.00	0.00	0.00	0.00	0.00	0.00
TIP41-like protein pseudogene	LOC100755027	100755027	1.08	1.45	1.21	1.40	1.30	1.76	58.48	56.73	45.96	41.72	32.08	42.06

DNA-directed RNA polymerases I, II, and III subunit RPABC3-like	LOC100755028	100755028	0.00	0.00	0.00	0.00	0.00	0.00	0.00	0.00	0.00	0.00	0.00	0.00
olfactory receptor 4F21-like	LOC100755034	100755034	0.03	0.00	0.00	0.00	0.00	0.00	0.76	0.00	0.00	0.00	0.00	0.00
ATP synthase subunit gamma, mitochondrial pseudogene	LOC100755035	100755035	0.00	0.00	0.00	0.00	0.00	0.00	0.00	0.00	0.00	0.00	0.00	0.00
40S ribosomal protein S10 pseudogene	LOC100755038	100755038	0.00	0.00	0.00	0.00	0.00	0.00	0.00	0.00	0.00	0.00	0.00	0.00
ornithine decarboxylase pseudogene	LOC100755039	100755039	0.00	0.00	0.00	0.00	0.00	0.03	0.00	0.00	0.00	0.00	0.00	0.93
ATP-dependent DNA helicase Q5	LOC100755041	100755041	10.52	9.32	11.55	9.70	8.61	9.42	603.83	549.94	658.31	549.33	477.13	528.09
G1/S-specific cyclin-D3 pseudogene	LOC100755042	100755042	0.00	0.04	0.00	0.06	0.00	0.05	0.00	0.92	0.00	1.16	0.00	0.93
olfactory receptor 51F2	LOC100755043	100755043	0.00	0.00	0.00	0.00	0.00	0.00	0.00	0.00	0.00	0.00	0.00	0.00
chromosome unknown open reading frame, human C2orf71	LOC100755045	100755045	0.00	0.00	0.00	0.00	0.00	0.00	0.00	0.00	0.00	0.00	0.00	0.00
40S ribosomal protein S29 pseudogene	LOC100755047	100755047	0.68	1.24	0.59	0.61	1.25	0.00	1.52	6.41	2.48	3.48	3.10	0.00
60 kDa heat shock protein, mitochondrial pseudogene	LOC100755049	100755049	0.16	0.19	0.25	0.15	0.17	0.11	6.08	6.41	9.94	6.95	6.21	2.80
selenoprotein W pseudogene	LOC100755052	100755052	10.64	7.68	8.35	7.42	5.11	9.72	38.74	25.62	32.29	23.18	17.59	26.17
acyl-coenzyme A synthetase ACSM4, mitochondrial	LOC100755056	100755056	0.00	0.00	0.00	0.00	0.00	0.00	1.52	3.66	2.48	3.48	1.03	4.67
40S ribosomal protein S15a-like	LOC100755058	100755058	0.00	0.00	0.00	0.00	0.00	0.00	0.00	0.00	0.00	0.00	0.00	0.00
up-regulator of cell proliferation pseudogene	LOC100755059	100755059	0.01	0.02	0.02	0.03	0.03	0.01	0.76	1.83	0.00	2.32	2.07	0.93
swi5-dependent recombination DNA repair protein 1 homolog	LOC100755061	100755061	45.36	43.04	44.62	47.31	56.92	55.82	2186.71	2059.74	2179.86	2269.17	2704.42	2667.55
uncharacterized LOC100755064	LOC100755064	100755064	0.00	0.00	0.00	0.00	0.00	0.00	0.00	0.00	0.00	0.00	0.00	0.00
mitochondrial thiamine pyrophosphate carrier pseudogene	LOC100755065	100755065	0.05	0.04	0.00	0.00	0.07	0.00	0.00	0.00	1.24	0.00	0.00	0.00

GSK-3-binding protein FRAT2-like	LOC100 755069	100755069	0.00	0.08	0.00	0.00	0.00	0.00	0.00	0.00	0.00	0.00	0.00	0.00
torsin-1A-interacting protein 2	LOC100 755074	100755074	8.03	7.40	6.89	7.41	6.06	6.99	603.07	551.77	535.34	552.80	465.74	522.48
guanine nucleotide-binding protein subunit beta-2-like 1 pseudogene	LOC100 755080	100755080	15.58	17.01	18.32	13.21	11.43	11.30	1233.49	1358.82	1428.40	952.63	576.49	692.59
40S ribosomal protein S19 pseudogene	LOC100 755081	100755081	0.00	0.00	0.00	0.00	0.00	0.00	0.00	0.00	0.00	0.00	0.00	0.00
nucleoporin Nup43 pseudogene	LOC100 755090	100755090	0.19	0.18	0.09	0.09	0.17	0.10	5.32	5.49	2.48	2.32	6.21	2.80
PCNA-associated factor pseudogene	LOC100 755094	100755094	0.17	0.00	0.00	0.00	0.00	0.00	0.76	0.00	0.00	0.00	0.00	0.00
serine/arginine-rich splicing factor 3 pseudogene	LOC100 755096	100755096	0.00	0.00	0.00	0.00	0.00	0.00	6.08	5.49	7.45	4.64	7.24	0.00
olfactory receptor 10H2	LOC100 755097	100755097	0.00	0.00	0.00	0.00	0.00	0.00	0.00	0.00	0.00	0.00	0.00	0.00
triosephosphate isomerase pseudogene	LOC100 755101	100755101	24.90	23.86	23.62	9.92	6.26	6.29	234.70	256.21	217.37	96.19	52.78	68.23
chromosome unknown open reading frame, human C11orf97	LOC100 755104	100755104	0.00	0.00	0.00	0.00	0.00	0.00	0.00	0.00	0.00	0.00	0.00	0.00
polyadenylate-binding protein 4 pseudogene	LOC100 755107	100755107	3.41	3.13	2.31	3.40	2.43	2.80	85.83	75.03	58.38	82.28	67.27	80.38
carbonyl reductase [NADPH] 1 pseudogene	LOC100 755108	100755108	0.00	0.00	0.00	0.00	0.00	0.00	0.00	0.00	0.00	0.00	0.00	0.00
thymidylate synthase pseudogene	LOC100 755111	100755111	0.14	0.07	0.04	0.21	0.07	0.03	3.80	1.83	1.24	5.79	2.07	0.93
ribosome biogenesis protein NSA2 homolog pseudogene	LOC100 755115	100755115	15.83	14.64	12.87	13.08	14.25	13.77	129.12	109.80	86.95	66.06	86.94	71.03
vomeronasal type-2 receptor 116-like	LOC100 755119	100755119	0.00	0.00	0.00	0.00	0.00	0.00	0.00	0.00	0.00	0.00	0.00	0.00
DNA-directed RNA polymerases I, II, and III subunit RPABC5	LOC100 755123	100755123	0.00	0.00	0.00	0.00	0.00	0.00	3.04	2.75	2.48	3.48	0.00	0.00
melanoma-associated antigen 10-like	LOC100 755124	100755124	0.00	0.00	0.00	0.00	0.00	0.00	0.76	0.00	0.00	0.00	0.00	0.00
zinc finger protein 58-like	LOC100 755127	100755127	1.16	0.86	1.03	0.91	1.44	1.45	34.94	32.94	31.05	27.81	39.33	33.65

guanine nucleotide-binding protein G(I)/G(S)/G(O) subunit gamma-2	LOC100755128	100755128	0.19	0.00	0.00	0.32	0.00	0.00	2.28	0.92	0.00	1.16	0.00	0.00
olfactory receptor 5B12-like	LOC100755130	100755130	0.00	0.00	0.00	0.00	0.00	0.00	0.00	0.00	0.00	0.00	0.00	0.00
poly [ADP-ribose] polymerase 14-like	LOC100755131	100755131	0.00	0.00	0.00	0.00	0.00	0.00	0.00	0.00	0.00	0.00	0.00	0.00
olfactory receptor 10A5	LOC100755132	100755132	0.00	0.00	0.00	0.00	0.00	0.00	0.00	0.00	0.00	0.00	0.00	0.00
ribosomal RNA-processing protein 7 homolog A pseudogene	LOC100755134	100755134	0.05	0.04	0.06	0.14	0.00	0.10	0.76	0.00	1.24	1.16	0.00	1.87
putative protein FAM47D	LOC100755136	100755136	0.00	0.00	0.00	0.08	0.00	0.00	0.00	0.00	0.00	2.32	0.00	0.00
Krueppel-like factor 10 pseudogene	LOC100755140	100755140	0.21	0.05	0.11	0.03	0.03	0.08	6.84	1.83	3.73	1.16	1.03	2.80
CCR4-NOT transcription complex subunit 2	LOC100755143	100755143	80.21	79.04	70.47	84.59	84.72	77.83	3149.05	3037.00	2803.39	3153.42	3351.29	3002.16
cyclin-G1 pseudogene	LOC100755148	100755148	0.17	0.08	0.08	0.00	0.00	0.08	18.23	19.22	13.66	4.64	9.31	8.41
olfactory receptor 10AD1-like	LOC100755150	100755150	0.00	0.00	0.00	0.00	0.00	0.00	0.00	0.00	0.00	0.00	0.00	0.00
adrenodoxin-like	LOC100755157	100755157	0.00	0.15	0.00	0.09	0.21	0.08	0.00	1.83	0.00	1.16	2.07	0.93
olfactory receptor 10T2	LOC100755158	100755158	0.00	0.00	0.00	0.00	0.00	0.00	0.00	0.00	0.00	0.00	0.00	0.00
vomeronasal type-1 receptor 4-like	LOC100755160	100755160	0.00	0.00	0.00	0.00	0.00	0.00	0.00	0.00	0.00	0.00	0.00	0.00
PHD finger protein 20-like protein 1	LOC100755163	100755163	4.10	4.10	2.89	2.57	3.39	3.59	508.13	473.99	345.30	316.39	406.75	430.88
R-spondin-3	LOC100755164	100755164	0.00	0.00	0.00	0.00	0.00	0.00	0.00	0.00	0.00	0.00	0.00	0.00
taste receptor type 2 member 129-like	LOC100755171	100755171	0.00	0.00	0.00	0.00	0.00	0.00	0.00	0.00	0.00	0.00	0.00	0.00
40S ribosomal protein S9 pseudogene	LOC100755172	100755172	0.77	0.31	0.21	0.10	0.18	0.19	9.87	7.32	2.48	2.32	2.07	1.87
granzyme-like protein 2	LOC100755174	100755174	0.00	0.00	0.00	0.00	0.00	0.00	0.00	0.00	0.00	0.00	0.00	0.00

eukaryotic translation initiation factor 1 pseudogene	LOC100 755183	100755183	0.00	0.00	0.00	0.00	0.00	0.00	0.00	0.00	0.00	0.00	0.00	0.00
tubulin beta-4B chain pseudogene	LOC100 755185	100755185	0.00	0.03	0.04	0.04	0.00	0.00	0.76	0.92	2.48	1.16	0.00	0.00
olfactory receptor 1002	LOC100 755188	100755188	5.64	5.14	2.49	3.00	1.50	1.38	799.04	730.20	357.72	456.61	215.28	199.08
class I histocompatibility antigen, Gogo-A*0101 alpha chain-like	LOC100 755192	100755192	0.00	0.00	0.00	0.00	0.00	0.00	0.00	0.00	0.00	0.00	0.00	0.00
olfactory receptor 18-like	LOC100 755193	100755193	0.00	0.00	0.00	0.00	0.00	0.00	0.00	0.00	0.00	0.00	0.00	0.00
polyhomeotic-like protein 2 pseudogene	LOC100 755194	100755194	0.00	0.00	0.00	0.00	0.00	0.00	0.00	0.00	1.24	0.00	0.00	0.00
eukaryotic translation initiation factor 5A-1-like	LOC100 755196	100755196	90.73	85.55	87.68	68.24	54.55	60.77	1964.17	1794.38	1849.47	1389.54	1097.09	1243.11
chromosome unknown open reading frame, human C12orf71	LOC100 755199	100755199	0.02	0.03	0.08	0.00	0.13	0.11	0.76	0.92	2.48	0.00	4.14	3.74
thioredoxin domain-containing protein 17 pseudogene	LOC100 755206	100755206	0.00	0.00	0.00	0.00	0.00	0.00	0.00	0.00	0.00	0.00	0.00	0.00
cytochrome c oxidase subunit 8A, mitochondrial	LOC100 755207	100755207	183.84	177.67	179.48	156.12	132.33	133.57	2090.25	2003.01	1988.58	1698.98	1468.65	1480.52
solute carrier family 22 member 21	LOC100 755213	100755213	0.07	0.11	0.07	0.10	0.16	0.02	0.00	0.00	0.00	0.00	0.00	0.00
calcium-binding and coiled-coil domain-containing protein 2 pseudogene	LOC100 755214	100755214	0.00	0.00	0.00	0.00	0.00	0.00	0.00	0.00	0.00	0.00	0.00	0.00
60S ribosomal protein L7-like	LOC100 755218	100755218	0.00	0.00	0.00	0.00	0.00	0.00	0.00	0.00	0.00	0.00	0.00	0.00
olfactory receptor 9K2	LOC100 755219	100755219	0.00	0.00	0.00	0.00	0.00	0.00	0.00	0.00	0.00	0.00	0.00	0.00
keratin, type II cytoskeletal 8 pseudogene	LOC100 755220	100755220	0.00	0.00	0.00	0.00	0.00	0.00	0.00	0.00	0.00	0.00	0.00	0.00
keratin-associated protein 8-1	LOC100 755222	100755222	0.00	0.00	0.00	0.00	0.00	0.00	0.00	0.00	0.00	0.00	0.00	0.00
mitochondrial inner membrane protease ATP23 homolog pseudogene	LOC100 755224	100755224	0.00	0.00	0.00	0.00	0.00	0.00	0.00	0.00	0.00	0.00	0.00	0.00
immunity-related GTPase family M protein 1-like	LOC100 755228	100755228	0.00	0.00	0.00	0.00	0.00	0.01	0.00	0.92	0.00	0.00	0.00	0.00

uncharacterized LOC100755231	LOC100755231	100755231	22.30	23.36	26.66	24.44	26.90	22.76	4641.54	4625.49	5339.73	5062.16	5558.91	5426.69
small proline-rich protein 3	LOC100755232	100755232	0.05	0.03	0.21	0.12	0.07	0.13	1.52	0.92	6.21	3.48	2.07	5.61
type-1A angiotensin II receptor	LOC100755233	100755233	0.19	0.20	0.13	0.34	0.25	0.15	11.39	13.73	11.18	13.91	16.56	9.35
sperm motility kinase-like	LOC100755238	100755238	0.00	0.00	0.04	0.00	0.00	0.00	0.76	0.00	2.48	1.16	0.00	0.00
THO complex subunit 2	LOC100755241	100755241	11.14	10.56	5.77	9.22	11.97	10.08	1316.28	1251.77	981.25	1211.07	1465.54	1258.07
olfactory receptor 1D2	LOC100755243	100755243	0.00	0.00	0.00	0.00	0.00	0.00	0.00	0.00	1.24	0.00	1.03	0.93
disintegrin and metalloproteinase domain-containing protein 5	LOC100755245	100755245	0.00	0.00	0.00	0.00	0.00	0.00	0.00	0.00	0.00	0.00	0.00	0.00
PRAME family member 12-like	LOC100755247	100755247	0.01	0.00	0.00	0.00	0.00	0.00	0.00	0.00	0.00	0.00	0.00	0.00
thyroglobulin-like	LOC100755249	100755249	0.07	0.00	0.00	0.00	0.00	0.00	1.52	0.00	0.00	0.00	0.00	0.00
developmentally-regulated GTP-binding protein 1-like	LOC100755253	100755253	0.97	1.12	0.76	1.04	1.21	1.26	22.03	23.79	13.66	19.70	23.80	24.30
cytochrome P450 26C1	LOC100755258	100755258	0.00	0.00	0.00	0.00	0.00	0.00	0.00	0.92	0.00	1.16	0.00	0.00
double homeobox protein 4C-like	LOC100755269	100755269	0.00	0.00	0.00	0.00	0.00	0.00	0.00	0.00	0.00	0.00	0.00	0.00
olfactory receptor 2A14	LOC100755277	100755277	0.00	0.00	0.00	0.00	0.00	0.00	0.00	0.00	0.00	0.00	0.00	0.00
phosphoglycerate kinase 1 pseudogene	LOC100755281	100755281	0.07	0.06	0.04	0.07	0.04	0.00	0.76	0.92	0.00	0.00	0.00	0.00
40S ribosomal protein S27-like	LOC100755284	100755284	9.23	7.17	4.68	4.44	7.46	7.81	4.56	7.32	2.48	2.32	4.14	6.54
CD209 antigen-like protein 2	LOC100755286	100755286	0.00	0.00	0.00	0.00	0.00	0.00	0.00	0.00	0.00	0.00	0.00	0.00
chromosome unknown open reading frame, human C9orf152	LOC100755291	100755291	0.00	0.00	0.00	0.00	0.00	0.06	0.00	0.00	0.00	0.00	0.00	0.93
protocadherin gamma-B5-like	LOC100755294	100755294	0.01	0.01	0.00	0.00	0.00	0.00	0.76	0.00	0.00	0.00	0.00	0.00

14-3-3 protein epsilon pseudogene	LOC100755297	100755297	118.01	106.41	95.93	88.34	116.76	115.59	1823.65	1732.16	1597.32	1512.39	1848.49	1889.90
protein FAM49B pseudogene	LOC100755301	100755301	4.40	4.12	2.53	2.31	2.44	2.49	114.69	107.06	60.86	54.47	63.13	67.30
40S ribosomal protein SA pseudogene	LOC100755302	100755302	10.57	11.24	11.68	7.16	6.75	7.21	211.15	224.18	243.45	151.82	138.69	147.68
F-box-like/WD repeat-containing protein TBL1X	LOC100755304	100755304	46.41	46.79	43.26	48.03	49.27	50.89	7237.65	7352.29	6896.07	7507.48	7694.09	7938.15
C-type lectin domain family 4 member K-like	LOC100755310	100755310	0.00	0.00	0.00	0.00	0.00	0.02	0.00	0.00	0.00	0.00	0.00	0.00
trace amine-associated receptor 7e-like	LOC100755312	100755312	0.00	0.00	0.00	0.00	0.00	0.00	0.00	0.00	0.00	0.00	0.00	0.00
chromosome unknown open reading frame, human C4orf26	LOC100755317	100755317	0.00	0.00	0.00	0.53	0.00	0.00	0.00	0.00	0.00	1.16	0.00	0.00
putative olfactory receptor GPCRLTM7	LOC100755320	100755320	0.00	0.00	0.00	0.00	0.00	0.00	0.00	0.00	0.00	0.00	0.00	0.00
unconventional myosin-XVB-like	LOC100755327	100755327	0.08	0.07	0.07	0.04	0.04	0.03	29.62	22.88	22.36	19.70	12.42	14.02
olfactory receptor 52R1-like	LOC100755329	100755329	0.00	0.00	0.00	0.00	0.00	0.00	0.00	0.00	0.00	0.00	0.00	0.00
sperm motility kinase 2B-like	LOC100755333	100755333	0.00	0.00	0.00	0.00	0.00	0.00	0.00	0.00	0.00	0.00	0.00	0.00
arylamine N-acetyltransferase 1	LOC100755335	100755335	0.00	0.00	0.00	0.00	0.00	0.00	0.00	0.00	0.00	0.00	0.00	0.00
olfactory receptor 52K1-like	LOC100755336	100755336	0.00	0.00	0.00	0.00	0.00	0.00	0.00	0.00	0.00	0.00	0.00	0.00
uncharacterized protein C1orf228-like	LOC100755338	100755338	0.08	0.09	0.00	0.06	0.04	0.05	4.56	3.66	0.00	2.32	2.07	2.80
vomeronasal type-2 receptor 116-like	LOC100755343	100755343	0.00	0.00	0.00	0.00	0.00	0.00	0.00	0.00	0.00	0.00	0.00	0.00
uncharacterized LOC100755357	LOC100755357	100755357	0.31	0.46	0.21	0.17	0.10	0.11	15.95	21.96	7.45	8.11	8.28	3.74
olfactory receptor 2B6-like	LOC100755362	100755362	0.00	0.00	0.00	0.00	0.00	0.00	0.00	0.00	0.00	0.00	0.00	0.00
zinc finger protein 420-like	LOC100755363	100755363	1.76	1.79	1.98	2.53	2.73	2.40	144.31	148.24	165.20	203.97	221.49	196.28

alpha-defensin 3-like	LOC100 755370	100755370	0.00	0.00	0.00	0.00	0.00	0.00	0.00	0.00	0.00	0.00	0.00	0.00
60S ribosomal protein L7a pseudogene	LOC100 755372	100755372	0.51	0.85	0.51	0.24	0.19	0.20	10.63	18.30	11.18	4.64	3.10	6.54
E3 ubiquitin-protein ligase Hakai pseudogene	LOC100 755373	100755373	0.12	0.05	0.00	0.00	0.00	0.10	0.76	0.00	0.00	0.00	0.00	0.00
chromosome unknown open reading frame, human C19orf24	LOC100 755377	100755377	23.95	24.21	27.26	22.29	14.87	17.10	301.54	317.52	344.06	295.52	210.10	222.45
cytochrome P450 4F4	LOC100 755384	100755384	0.01	0.00	0.00	0.01	0.00	0.00	0.00	0.00	0.00	0.00	0.00	0.00
coiled-coil domain-containing protein 43-like	LOC100 755399	100755399	0.00	0.29	0.00	0.15	0.14	0.06	1.52	6.41	1.24	2.32	2.07	0.93
vomeronasal type-1 receptor 4-like	LOC100 755405	100755405	0.00	0.00	0.00	0.00	0.00	0.00	0.00	0.00	0.00	0.00	0.00	0.00
40S ribosomal protein S16 pseudogene	LOC100 755408	100755408	22.02	17.31	19.05	9.83	14.62	22.92	135.96	106.14	111.79	57.95	91.08	143.94
olfactory receptor 5B12-like	LOC100 755414	100755414	0.00	0.00	0.00	0.00	0.00	0.00	0.00	0.00	0.00	0.00	0.00	0.00
E3 ubiquitin-protein ligase DTX3L-like	LOC100 755415	100755415	13.85	13.83	8.91	9.70	11.14	11.45	1358.06	1407.32	921.63	1015.21	1168.50	1164.60
olfactory receptor 10A4	LOC100 755416	100755416	0.00	0.00	0.00	0.00	0.00	0.00	0.00	0.00	0.00	0.00	0.00	0.00
heat shock cognate 71 kDa protein pseudogene	LOC100 755418	100755418	115.58	113.30	137.53	140.04	114.83	121.77	4949.92	4843.27	5993.07	6167.77	5033.14	5383.70
uncharacterized LOC100755420	LOC100 755420	100755420	0.14	0.07	0.09	0.30	0.08	0.10	3.80	1.83	3.73	9.27	2.07	3.74
glyceraldehyde-3-phosphate dehydrogenase pseudogene	LOC100 755422	100755422	0.15	0.25	0.09	0.14	0.13	0.11	0.76	5.49	0.00	2.32	1.03	1.87
UDP-glucuronosyltransferase 1-6	LOC100 755423	100755423	21.66	22.45	14.98	14.31	19.18	20.47	1576.80	1646.15	1045.84	1008.26	1329.96	1434.72
zinc finger FYVE domain-containing protein 1 pseudogene	LOC100 755425	100755425	0.00	0.00	0.00	0.00	0.00	0.02	0.00	0.00	0.00	0.00	0.00	0.00
keratin-associated protein 4-12-like	LOC100 755426	100755426	0.00	0.00	0.00	0.00	0.00	0.00	0.00	0.00	0.00	0.00	0.00	0.00
interferon-induced very large GTPase 1	LOC100 755428	100755428	0.00	0.00	0.00	0.00	0.00	0.00	0.00	0.92	0.00	0.00	0.00	0.00

nucleosome assembly protein 1-like 1 pseudogene	LOC100755430	100755430	0.16	0.28	0.24	0.15	0.45	0.20	4.56	6.41	6.21	4.64	10.35	6.54
T-complex protein 1 subunit gamma pseudogene	LOC100755434	100755434	0.00	0.02	0.00	0.03	0.00	0.00	0.00	0.92	0.00	1.16	0.00	0.00
olfactory receptor 10AD1-like	LOC100755435	100755435	0.00	0.00	0.00	0.00	0.00	0.00	0.00	0.00	0.00	0.00	0.00	0.00
ferritin light chain 1 pseudogene	LOC100755438	100755438	0.00	0.00	0.00	0.00	0.00	0.00	0.00	0.00	0.00	0.00	0.00	0.00
speckle-type POZ protein pseudogene	LOC100755443	100755443	0.00	0.00	0.11	0.00	0.00	0.00	0.00	0.00	0.00	0.00	0.00	0.00
olfactory receptor 502	LOC100755444	100755444	0.00	0.00	0.00	0.00	0.00	0.00	0.00	0.00	0.00	0.00	0.00	0.00
olfactory receptor 2H2-like	LOC100755448	100755448	0.00	0.00	0.00	0.00	0.00	0.00	0.00	0.00	0.00	0.00	0.00	0.00
40S ribosomal protein S4, X isoform-like	LOC100755449	100755449	1.48	1.48	1.39	0.83	1.07	0.79	73.68	63.14	55.89	39.40	57.96	41.13
forkhead box protein P4 pseudogene	LOC100755450	100755450	0.00	0.00	0.01	0.00	0.00	0.00	0.00	0.00	0.00	0.00	0.00	0.00
uncharacterized LOC100755453	LOC100755453	100755453	0.00	0.00	0.00	0.00	0.00	0.00	0.00	0.00	0.00	0.00	0.00	0.00
ras-related protein Rab-3D	LOC100755455	100755455	8.62	8.10	9.97	8.36	7.50	7.95	825.62	792.42	940.26	762.57	662.39	727.17
cytochrome c oxidase subunit 5A, mitochondrial pseudogene	LOC100755457	100755457	0.00	0.00	0.00	0.00	0.00	0.00	0.00	0.00	0.00	0.00	0.00	0.00
taste receptor type 2 member 109-like	LOC100755460	100755460	0.00	0.00	0.00	0.00	0.00	0.00	0.00	0.00	0.00	0.00	0.00	0.00
mast cell protease 8-like	LOC100755462	100755462	0.00	0.00	0.00	0.00	0.00	0.00	0.00	0.00	0.00	0.00	0.00	0.00
RIMS-binding protein 3	LOC100755463	100755463	0.04	0.09	0.04	0.07	0.06	0.05	7.60	11.90	4.97	10.43	9.31	9.35
60S ribosomal protein L27a pseudogene	LOC100755465	100755465	0.00	0.00	0.00	0.00	0.00	0.00	0.00	0.00	0.00	0.00	0.00	0.00
olfactory receptor 4C46-like	LOC100755472	100755472	0.00	0.00	0.00	0.00	0.00	0.00	0.00	0.00	0.00	0.00	0.00	0.00
cysteine-rich secretory protein 1-like	LOC100755475	100755475	0.00	0.00	0.00	0.00	0.00	0.00	0.00	0.00	0.00	0.00	0.00	0.00

murinoglobulin-1-like	LOC100 755476	100755476	0.00	0.00	0.00	0.00	0.00	0.00	0.00	0.00	0.00	0.00	0.00	0.00
olfactory receptor 9G4	LOC100 755477	100755477	0.00	0.00	0.00	0.00	0.00	0.00	0.00	0.00	0.00	0.00	0.00	0.00
HLA class I histocompatibility antigen, A-32 alpha chain-like	LOC100 755481	100755481	0.00	0.00	0.00	0.00	0.00	0.00	0.00	0.00	0.00	0.00	0.00	0.00
putative gustatory receptor clone PTE01	LOC100 755482	100755482	0.00	0.00	0.00	0.00	0.00	0.00	0.00	0.00	0.00	0.00	0.00	0.00
melanoma-associated antigen B2	LOC100 755483	100755483	0.00	0.00	0.00	0.00	0.00	0.00	0.00	0.00	0.00	0.00	0.00	0.00
snRNA-activating protein complex subunit 2-like	LOC100 755484	100755484	0.05	0.07	0.06	0.06	0.10	0.07	3.04	3.66	2.48	5.79	5.17	4.67
coiled-coil-helix-coiled-coil-helix domain-containing protein 2, mitochondrial pseudogene	LOC100 755494	100755494	0.00	0.00	0.00	0.00	0.00	0.00	0.00	0.00	0.00	0.00	0.00	0.00
cyclin-dependent kinase 4 pseudogene	LOC100 755502	100755502	0.00	0.00	0.00	0.00	0.00	0.00	0.00	0.00	0.00	0.00	0.00	0.00
retinal guanylyl cyclase 2-like	LOC100 755503	100755503	0.02	0.02	0.00	0.00	0.00	0.00	1.52	1.83	0.00	0.00	1.03	0.00
60S acidic ribosomal protein P1-like	LOC100 755504	100755504	0.00	0.00	0.00	0.00	0.00	0.00	0.00	0.00	0.00	0.00	0.00	0.00
ER membrane protein complex subunit 7 pseudogene	LOC100 755505	100755505	0.00	0.00	0.00	0.00	0.00	0.00	0.00	0.00	0.00	0.00	0.00	0.00
olfactory receptor 10A7	LOC100 755509	100755509	0.00	0.00	0.00	0.00	0.00	0.00	0.00	0.00	0.00	0.00	0.00	0.00
60S ribosomal protein L9 pseudogene	LOC100 755510	100755510	0.48	0.84	0.60	0.29	0.42	0.49	6.08	10.07	7.45	3.48	5.17	5.61
keratin-associated protein 7-1	LOC100 755511	100755511	0.00	0.00	0.00	0.00	0.00	0.00	0.00	0.00	0.00	0.00	0.00	0.00
peptidyl-prolyl cis-trans isomerase A-like	LOC100 755515	100755515	1.31	0.70	0.43	0.56	0.69	0.60	9.11	1.83	1.24	2.32	6.21	3.74
40S ribosomal protein S21 pseudogene	LOC100 755516	100755516	0.00	0.00	0.00	0.00	0.00	0.64	0.00	0.92	1.24	0.00	0.00	0.93
small proline-rich protein 2E-like	LOC100 755523	100755523	0.00	0.00	0.00	0.00	0.00	0.00	0.00	0.00	0.00	0.00	0.00	0.00
disintegrin and metalloproteinase domain-containing protein 18-like	LOC100 755537	100755537	0.00	0.00	0.00	0.00	0.00	0.00	0.00	0.92	0.00	0.00	0.00	0.00

E3 ubiquitin-protein ligase RNF216-like	LOC100755539	100755539	0.00	0.00	0.00	0.00	0.00	0.00	0.00	0.00	0.00	0.00	0.00	0.00
olfactory receptor 2W1-like	LOC100755542	100755542	0.00	0.00	0.00	0.00	0.00	0.00	0.00	0.00	0.00	0.00	0.00	0.00
cytochrome P450 26A1	LOC100755549	100755549	0.08	0.03	0.07	0.06	0.06	0.08	3.80	0.92	2.48	2.32	3.10	2.80
40S ribosomal protein S29 pseudogene	LOC100755557	100755557	0.00	0.00	0.00	0.00	0.00	0.00	0.00	0.00	0.00	0.00	0.00	0.00
chromosome unknown open reading frame, human C1orf85	LOC100755558	100755558	91.39	92.10	103.80	102.56	88.97	94.64	3893.40	3923.66	4445.43	4328.57	3766.32	4023.75
E3 ubiquitin-protein ligase TRAF7 pseudogene	LOC100755561	100755561	1.08	1.10	1.19	1.07	0.88	1.18	45.57	43.92	48.44	42.88	37.26	44.86
olfactory receptor 2A2-like	LOC100755570	100755570	0.00	0.00	0.00	0.00	0.00	0.00	0.00	0.00	0.00	0.00	0.00	0.00
olfactory receptor 1052-like	LOC100755574	100755574	0.00	0.00	0.00	0.00	0.00	0.00	0.00	0.00	0.00	0.00	0.00	0.00
CD209 antigen-like protein C	LOC100755579	100755579	0.00	0.00	0.00	0.00	0.00	0.00	0.00	0.92	0.00	0.00	0.00	0.00
60S ribosomal protein L23 pseudogene	LOC100755584	100755584	0.09	0.00	0.00	0.00	0.25	0.12	6.84	2.75	1.24	1.16	6.21	2.80
SH3 and cysteine-rich domain-containing protein 3	LOC100755585	100755585	39.13	39.09	42.01	39.52	33.64	32.40	4957.51	5109.55	5344.70	5120.11	4362.47	4201.34
charged multivesicular body protein 1b-2	LOC100755586	100755586	35.04	36.13	33.31	36.49	41.04	39.85	1294.25	1356.08	1201.10	1315.37	1480.03	1428.18
glyceraldehyde-3-phosphate dehydrogenase pseudogene	LOC100755589	100755589	0.19	0.27	0.10	0.20	0.13	0.04	4.56	6.41	2.48	4.64	3.10	0.93
hypothetical protein	LOC100755591	100755591	31.54	31.10	32.99	26.95	25.85	27.20	922.84	900.39	942.74	746.34	748.30	750.54
hemK methyltransferase family member 2 pseudogene	LOC100755595	100755595	0.00	0.00	0.00	0.00	0.00	0.00	0.00	0.00	0.00	0.00	0.00	0.00
aldehyde dehydrogenase family 3 member B1	LOC100755596	100755596	0.07	0.07	0.06	0.11	0.10	0.04	2.28	6.41	3.73	6.95	5.17	1.87
alpha-enolase pseudogene	LOC100755603	100755603	0.00	0.00	0.00	0.00	0.00	0.00	0.00	0.00	0.00	0.00	0.00	0.00
trace amine-associated receptor 9	LOC100755605	100755605	0.00	0.00	0.00	0.00	0.00	0.00	0.00	0.00	0.00	0.00	0.00	0.00

putative uncharacterized protein C14orf25	LOC100755606	100755606	0.00	0.00	0.00	0.00	0.06	0.00	0.00	0.92	1.24	0.00	2.07	0.93
spindlin-2B-like	LOC100755607	100755607	0.00	0.00	0.00	0.00	0.00	0.00	0.00	0.00	0.00	0.00	0.00	0.00
chromosome unknown open reading frame, human C4orf3	LOC100755608	100755608	31.59	37.41	28.51	24.40	35.43	27.22	635.73	737.52	535.34	515.72	724.49	587.91
chromosome unknown open reading frame, human C14orf28	LOC100755612	100755612	2.28	2.38	2.18	2.35	2.36	2.75	82.03	81.44	78.25	84.60	91.08	103.75
olfactory receptor 4K3-like	LOC100755613	100755613	0.00	0.00	0.00	0.00	0.00	0.00	0.00	0.00	0.00	0.00	0.00	0.00
nucleolar complex protein 3 homolog pseudogene	LOC100755617	100755617	0.00	0.00	0.02	0.00	0.00	0.00	0.00	0.00	1.24	0.00	0.00	0.00
3-hydroxyacyl-CoA dehydrogenase type-2 pseudogene	LOC100755628	100755628	0.00	0.00	0.00	0.00	0.00	0.00	0.00	0.00	0.00	0.00	0.00	0.00
olfactory receptor 56A4	LOC100755629	100755629	0.00	0.00	0.00	0.00	0.00	0.00	0.00	0.00	0.00	0.00	0.00	0.00
rho guanine nucleotide exchange factor 38	LOC100755633	100755633	0.00	0.00	0.00	0.00	0.00	0.00	0.00	0.00	0.00	0.00	0.00	0.00
melanoma-associated antigen B4-like	LOC100755634	100755634	0.00	0.00	0.00	0.00	0.00	0.00	0.00	0.00	0.00	0.00	0.00	0.00
iron-sulfur cluster assembly 1 homolog, mitochondrial pseudogene	LOC100755638	100755638	1.14	0.84	1.70	1.14	1.25	0.49	9.11	7.32	13.66	10.43	7.24	8.41
peptidyl-prolyl cis-trans isomerase D-like	LOC100755641	100755641	20.43	20.80	16.71	16.12	17.88	16.92	558.26	570.07	458.33	439.23	491.62	472.01
carcinoembryonic antigen-related cell adhesion molecule 3-like	LOC100755643	100755643	0.00	0.00	0.00	0.00	0.00	0.00	0.00	0.00	0.00	0.00	0.00	0.00
dynein light chain 1, cytoplasmic-like	LOC100755644	100755644	4.51	5.26	5.67	3.80	5.68	2.40	9.87	10.07	17.39	9.27	15.52	8.41
uncharacterized LOC100755646	LOC100755646	100755646	146.99	143.46	128.59	132.40	163.07	163.10	1099.05	1068.76	971.31	956.11	1210.93	1232.83
non-histone chromosomal protein HMG-17 pseudogene	LOC100755649	100755649	0.00	0.00	0.00	0.00	0.00	0.00	0.00	0.00	0.00	0.00	0.00	0.00
transmembrane protein 258 pseudogene	LOC100755652	100755652	0.00	0.46	0.00	0.00	0.00	0.54	13.67	12.81	9.94	9.27	5.17	12.15
chromosome unknown open reading frame, human CXorf36	LOC100755653	100755653	0.02	0.03	0.00	0.00	0.00	0.00	0.76	0.92	0.00	1.16	0.00	0.00

olfactory receptor 2J3-like	LOC100 755654	100755654	0.00	0.00	0.00	0.00	0.00	0.00	0.00	0.00	0.00	0.00	0.00	0.00
zinc finger protein 728-like	LOC100 755655	100755655	2.61	2.59	3.09	3.39	3.86	3.23	274.95	263.53	329.15	376.65	408.82	350.50
serine/threonine-protein kinase WNK1	LOC100 755657	100755657	44.15	44.13	37.56	43.45	45.14	41.40	11716.65	11751.78	10306.84	11728.27	12105.20	11119.77
pyruvate kinase PKM pseudogene	LOC100 755661	100755661	3.19	3.11	2.64	1.69	2.07	2.00	107.85	104.31	95.64	55.63	70.38	72.90
U3 small nucleolar RNA-interacting protein 2 pseudogene	LOC100 755675	100755675	0.03	0.02	0.00	0.04	0.02	0.05	1.52	0.92	0.00	2.32	1.03	2.80
zinc finger protein 878-like	LOC100 755677	100755677	3.90	3.95	3.61	4.47	5.14	4.92	1219.06	1242.62	1135.27	1408.09	1601.12	1546.88
late cornified envelope protein 1C-like	LOC100 755687	100755687	1.03	0.99	0.94	0.96	2.04	1.97	110.13	104.31	106.82	106.62	214.24	216.84
ubiquitin carboxyl-terminal hydrolase 12 pseudogene	LOC100 755689	100755689	1.03	0.91	0.37	1.21	1.20	0.71	33.42	31.11	16.15	35.93	39.33	26.17
glyceraldehyde-3-phosphate dehydrogenase pseudogene	LOC100 755694	100755694	73.27	87.58	72.08	52.95	36.82	33.05	41.02	48.50	42.23	28.97	32.08	22.43
vomeronasal type-1 receptor 4-like	LOC100 755698	100755698	0.00	0.00	0.00	0.00	0.00	0.00	0.00	0.00	0.00	0.00	0.00	0.00
uncharacterized LOC100755699	LOC100 755699	100755699	0.04	0.00	0.00	0.00	0.00	0.00	1.52	0.00	0.00	0.00	0.00	0.00
thioredoxin-like protein 4A	LOC100 755704	100755704	9.83	8.99	6.01	2.61	1.41	1.04	48.61	41.18	31.05	11.59	4.14	3.74
olfactory receptor 5B3-like	LOC100 755706	100755706	0.00	0.00	0.00	0.00	0.00	0.00	0.00	0.00	0.00	0.00	0.00	0.00
olfactory receptor 2D2	LOC100 755708	100755708	0.00	0.00	0.00	0.00	0.00	0.00	0.00	0.00	0.00	0.00	0.00	0.00
zinc finger protein 761-like	LOC100 755709	100755709	0.00	0.07	0.02	0.04	0.08	0.00	0.76	3.66	1.24	2.32	4.14	0.93
7,8-dihydro-8-oxoguanine triphosphatase pseudogene	LOC100 755712	100755712	0.00	0.00	0.00	0.00	0.00	0.00	0.00	0.00	0.00	0.00	0.00	0.00
ribosome biogenesis regulatory protein homolog	LOC100 755718	100755718	0.00	0.00	0.00	0.00	0.00	0.00	0.00	0.00	0.00	0.00	1.03	0.00
olfactory receptor 2AG1-like	LOC100 755721	100755721	0.00	0.00	0.00	0.00	0.00	0.00	0.00	0.00	0.00	0.00	0.00	0.00

olfactory receptor 10AD1-like	LOC100 755728	100755728	0.00	0.00	0.00	0.00	0.00	0.00	0.00	0.00	0.00	0.00	0.00	0.00
TBC1 domain family member 1	LOC100 755731	100755731	34.89	36.44	39.18	43.01	43.34	42.19	2058.35	2173.20	2378.60	2572.80	2591.61	2520.80
adenosine receptor A3	LOC100 755732	100755732	0.00	0.00	0.00	0.00	0.00	0.04	0.00	0.00	0.00	0.00	0.00	0.93
ATP synthase F(0) complex subunit C2, mitochondrial pseudogene	LOC100 755733	100755733	1.16	1.81	2.06	0.93	1.13	0.75	31.90	32.94	39.75	12.75	28.98	15.89
uncharacterized LOC100755734	LOC100 755734	100755734	1.37	1.46	1.56	1.28	1.36	1.26	30.38	35.69	38.50	27.81	33.12	28.97
protein maternal effect lethal 26-like	LOC100 755736	100755736	0.00	0.00	0.00	0.00	0.00	0.00	0.00	0.00	0.00	0.00	0.00	0.00
olfactory receptor 502-like	LOC100 755737	100755737	0.00	0.00	0.00	0.00	0.00	0.00	0.00	0.00	0.00	0.00	0.00	0.00
leukocyte immunoglobulin-like receptor subfamily B member 3	LOC100 755740	100755740	0.00	0.00	0.00	0.00	0.00	0.00	0.00	0.00	0.00	0.00	0.00	0.00
olfactory receptor 2B6-like	LOC100 755741	100755741	0.00	0.00	0.00	0.00	0.00	0.00	0.00	0.00	0.00	0.00	0.00	0.00
mortality factor 4-like protein 2	LOC100 755747	100755747	0.00	0.00	0.00	0.00	0.00	0.00	0.00	0.00	0.00	0.00	0.00	0.00
60S ribosomal protein L32 pseudogene	LOC100 755748	100755748	16.19	18.91	17.81	12.33	17.23	14.68	106.34	129.93	114.27	84.60	111.78	73.84
microtubule-associated proteins 1A/1B light chain 3B pseudogene	LOC100 755757	100755757	1.37	1.50	1.54	0.74	1.55	0.72	13.67	19.22	17.39	6.95	19.66	9.35
60S ribosomal protein L26-like	LOC100 755759	100755759	0.49	0.04	0.38	0.32	0.28	0.19	3.80	0.92	4.97	2.32	1.03	1.87
olfactory receptor 4C13	LOC100 755766	100755766	0.00	0.00	0.00	0.00	0.00	0.00	0.00	0.00	0.00	0.00	0.00	0.00
alpha-2-macroglobulin-like	LOC100 755770	100755770	0.00	0.00	0.00	0.00	0.00	0.00	0.00	0.00	0.00	0.00	0.00	0.00
class I histocompatibility antigen, Gogo-B*0101 alpha chain-like	LOC100 755775	100755775	0.00	0.00	0.00	0.00	0.00	0.00	0.00	0.00	0.00	0.00	0.00	0.00
alpha-enolase pseudogene	LOC100 755776	100755776	0.00	0.00	0.00	0.00	0.00	0.00	0.00	0.00	0.00	0.00	0.00	0.00
40S ribosomal protein S4, X isoform-like	LOC100 755788	100755788	0.00	0.00	0.00	0.00	0.00	0.00	0.00	0.00	0.00	0.00	0.00	0.00

chromosome unknown open reading frame, human C2orf74	LOC100755791	100755791	0.00	0.00	0.00	0.00	0.00	0.00	0.00	0.00	0.00	0.00	0.00	0.00
dynein light chain 1, cytoplasmic	LOC100755794	100755794	220.53	220.26	255.68	230.94	213.29	198.50	1316.28	1368.89	1535.22	1350.14	1216.11	1084.22
hypothetical protein	LOC100755802	100755802	0.00	0.05	0.00	0.00	0.00	0.05	0.00	0.92	0.00	1.16	0.00	1.87
olfactory receptor 6C74	LOC100755803	100755803	0.00	0.00	0.00	0.00	0.00	0.00	0.00	0.00	0.00	0.00	0.00	0.00
60S ribosomal protein L5-like	LOC100755804	100755804	0.03	0.08	0.06	0.05	0.19	0.04	0.76	1.83	1.24	1.16	4.14	0.93
keratin-associated protein 11-1	LOC100755805	100755805	0.00	0.00	0.00	0.00	0.00	0.00	0.00	0.00	0.00	0.00	0.00	0.00
40S ribosomal protein S7 pseudogene	LOC100755806	100755806	0.00	0.00	0.00	0.00	0.00	0.00	0.00	0.00	0.00	0.00	0.00	0.00
60S ribosomal protein L31 pseudogene	LOC100755807	100755807	0.00	0.00	0.00	0.00	0.00	0.00	0.00	0.00	0.00	0.00	0.00	0.00
thymidine kinase, cytosolic pseudogene	LOC100755810	100755810	1.24	0.73	0.89	0.55	0.58	0.56	0.00	0.92	1.24	0.00	0.00	0.00
60S ribosomal protein L18 pseudogene	LOC100755814	100755814	11.89	12.31	11.45	8.77	7.01	6.82	157.98	171.11	173.89	134.43	85.90	88.79
chromosome unknown open reading frame, human C11orf49	LOC100755821	100755821	21.97	23.12	27.77	26.61	25.61	25.48	1102.09	1130.07	1335.24	1240.04	1166.43	1204.79
GRB2-associated-binding protein 2-like	LOC100755822	100755822	0.00	0.00	0.00	0.00	0.00	0.00	0.00	0.00	0.00	0.00	0.00	0.00
vomeronasal type-2 receptor 116-like	LOC100755826	100755826	0.00	0.00	0.00	0.00	0.00	0.00	0.00	0.00	0.00	0.00	0.00	0.00
vomeronasal type-2 receptor 1-like	LOC100755832	100755832	0.00	0.00	0.00	0.00	0.00	0.00	0.00	0.00	0.00	0.00	0.00	0.00
zinc finger protein 239-like	LOC100755833	100755833	0.00	0.00	0.00	0.00	0.00	0.00	0.00	0.00	0.00	0.00	0.00	0.00
WNT1-inducible-signaling pathway protein 1-like	LOC100755834	100755834	0.00	0.03	0.00	0.00	0.00	0.03	0.00	2.75	0.00	0.00	0.00	0.93
nucleoporin SEH1 pseudogene	LOC100755835	100755835	0.00	0.00	0.00	0.13	0.04	0.03	0.00	0.00	0.00	2.32	1.03	0.93
chromosome unknown open reading frame, human C10orf67	LOC100755839	100755839	0.00	0.00	0.00	0.00	0.00	0.00	0.00	0.00	0.00	0.00	0.00	0.00

neuroligin-1-like	LOC100755841	100755841	1.40	1.44	1.38	1.63	1.85	1.41	66.08	70.46	68.31	82.28	84.87	66.36
toll-like receptor 12	LOC100755844	100755844	0.02	0.01	0.02	0.00	0.00	0.00	2.28	0.92	1.24	2.32	0.00	0.00
fer-1-like protein 4	LOC100755848	100755848	0.17	0.15	0.08	0.09	0.04	0.07	40.26	32.03	18.63	19.70	10.35	14.02
cytochrome P450 1A2	LOC100755851	100755851	0.00	0.00	0.00	0.00	0.00	0.00	0.00	0.00	0.00	0.00	0.00	0.00
chromosome unknown open reading frame, human C9orf43	LOC100755857	100755857	0.11	0.23	0.18	0.21	0.13	0.03	29.62	37.52	27.33	23.18	21.73	18.69
chromosome unknown open reading frame, human C10orf107	LOC100755859	100755859	0.00	0.00	0.00	0.00	0.00	0.00	0.00	0.00	0.00	0.00	0.00	0.00
dipeptidase 2-like	LOC100755863	100755863	0.00	0.00	0.00	0.00	0.00	0.00	0.00	0.00	0.00	0.00	0.00	0.00
cell cycle control protein 50C-like	LOC100755866	100755866	0.03	0.03	0.04	0.04	0.03	0.00	0.76	0.92	1.24	1.16	2.07	0.00
olfactory receptor 1052-like	LOC100755868	100755868	0.00	0.00	0.00	0.00	0.00	0.00	0.00	0.00	0.00	0.00	0.00	0.00
40S ribosomal protein S26-like	LOC100755871	100755871	0.31	0.37	0.00	0.00	0.22	0.40	1.52	1.83	0.00	0.00	1.03	1.87
GRB2-associated-binding protein 2 pseudogene	LOC100755873	100755873	0.00	0.02	0.00	0.00	0.00	0.00	0.00	0.92	0.00	0.00	0.00	0.00
chromosome unknown open reading frame, human C18orf8	LOC100755875	100755875	11.01	10.78	11.46	12.36	13.35	13.99	765.62	758.56	765.13	850.65	926.31	952.43
acidic leucine-rich nuclear phosphoprotein 32 family member E-like	LOC100755891	100755891	1.05	0.16	0.14	0.35	0.18	0.72	9.11	0.92	2.48	4.64	2.07	6.54
elongation factor 1-delta	LOC100755894	100755894	335.33	347.47	343.22	327.69	280.28	308.55	10875.84	11271.38	11079.41	10408.26	9005.42	9848.61
protein cornichon homolog 1-like	LOC100755897	100755897	54.15	57.30	58.93	48.53	43.61	50.80	544.59	590.20	589.99	446.18	397.43	468.27
spindlin-2B-like	LOC100755900	100755900	0.00	0.07	0.00	0.00	0.08	0.00	0.00	0.92	0.00	0.00	1.03	0.00
60S ribosomal protein L15 pseudogene	LOC100755902	100755902	0.00	0.00	0.00	0.00	0.00	0.00	3.80	9.15	12.42	4.64	9.31	2.80
olfactory receptor 4F15	LOC100755906	100755906	0.00	0.00	0.00	0.00	0.09	0.00	0.00	0.00	0.00	0.00	2.07	0.93

olfactory receptor 51F1-like	LOC100 755916	100755916	0.00	0.00	0.00	0.00	0.00	0.00	0.00	0.00	0.00	0.00	0.00	0.00
uncharacterized LOC100755921	LOC100 755921	100755921	1.40	1.41	1.35	0.63	0.30	0.30	41.02	36.60	36.02	19.70	12.42	14.95
olfactory receptor 52L1	LOC100 755924	100755924	0.00	0.00	0.00	0.00	0.00	0.00	0.00	0.00	0.00	0.00	0.00	0.00
cytochrome b5 domain-containing protein 1	LOC100 755925	100755925	5.07	5.69	6.29	6.43	8.91	7.91	255.21	267.19	295.62	322.18	420.20	390.69
Kv channel-interacting protein 4	LOC100 755926	100755926	0.00	0.00	0.00	0.00	0.00	0.00	0.00	0.92	0.00	0.00	0.00	0.00
glutathione S-transferase C-terminal domain-containing protein	LOC100 755927	100755927	15.24	17.18	17.65	19.98	21.91	21.15	586.36	649.67	679.42	746.34	824.88	799.14
actin-related protein 2 pseudogene	LOC100 755931	100755931	3.45	4.79	3.76	3.89	4.29	3.46	3.80	5.49	4.97	3.48	9.31	3.74
single-stranded DNA-binding protein, mitochondrial-like	LOC100 755932	100755932	0.06	0.27	0.10	0.03	0.24	0.13	3.80	3.66	2.48	1.16	4.14	0.93
regucalcin-like	LOC100 755933	100755933	0.00	0.00	0.00	0.00	0.00	0.00	0.00	0.00	0.00	0.00	0.00	0.00
carcinoembryonic antigen-related cell adhesion molecule 3-like	LOC100 755937	100755937	0.00	0.00	0.00	0.00	0.00	0.00	0.00	0.00	0.00	0.00	0.00	0.00
eukaryotic translation initiation factor 3 subunit J-like	LOC100 755940	100755940	0.00	0.00	0.00	0.00	0.00	0.00	0.00	0.00	0.00	0.00	0.00	0.93
olfactory receptor 14A2-like	LOC100 755942	100755942	0.00	0.00	0.00	0.00	0.00	0.00	0.00	0.00	0.00	0.00	0.00	0.00
tudor and KH domain-containing protein pseudogene	LOC100 755945	100755945	0.00	0.00	0.00	0.00	0.00	0.05	0.00	0.00	0.00	0.00	0.00	1.87
60S ribosomal protein L21	LOC100 755948	100755948	14.25	14.06	8.08	6.37	9.29	9.39	92.66	71.37	39.75	26.66	43.47	54.21
UBA-like domain-containing protein 1 pseudogene	LOC100 755949	100755949	0.00	0.00	0.00	0.00	0.00	0.00	0.00	0.00	0.00	0.00	0.00	0.00
60S ribosomal protein L31	LOC100 755951	100755951	22.31	20.75	16.74	12.94	16.34	10.36	111.65	93.33	81.98	53.31	81.76	48.60
prefoldin subunit 3 pseudogene	LOC100 755959	100755959	0.36	1.00	0.14	0.39	0.46	0.28	4.56	10.98	1.24	4.64	8.28	6.54
autophagy protein 5 pseudogene	LOC100 755960	100755960	0.00	0.00	0.00	0.00	0.00	0.00	0.00	0.00	0.00	0.00	0.00	0.00

BRCA1-associated RING domain protein 1 pseudogene	LOC100 755970	100755970	0.00	0.00	0.00	0.00	0.00	0.00	0.00	0.00	0.00	0.00	0.00	0.00
zinc finger protein 124-like	LOC100 755972	100755972	0.65	0.69	1.55	0.83	1.02	0.86	51.65	57.65	40.99	69.54	83.83	71.03
cell division cycle 5-like protein pseudogene	LOC100 755975	100755975	0.06	0.03	0.00	0.02	0.06	0.03	3.04	1.83	0.00	1.16	3.10	1.87
olfactory receptor 1009	LOC100 755977	100755977	0.02	0.03	0.00	0.03	0.03	0.00	1.52	0.92	0.00	1.16	2.07	0.00
heat shock protein HSP 90-alpha pseudogene	LOC100 755978	100755978	11.24	11.16	9.68	8.93	9.87	7.45	259.76	231.50	223.58	216.72	209.07	165.44
60S ribosomal protein L23-like	LOC100 755984	100755984	2.32	3.27	1.11	1.22	1.17	0.12	18.23	25.62	8.69	9.27	8.28	0.93
actin-related protein 2/3 complex subunit 1A pseudogene	LOC100 755986	100755986	0.22	0.51	0.24	0.04	0.27	0.40	5.32	13.73	6.21	1.16	7.24	11.22
vomeronasal type-1 receptor 4-like	LOC100 755993	100755993	0.00	0.00	0.00	0.00	0.00	0.00	0.00	0.00	0.00	0.00	0.00	0.00
high mobility group protein B1 pseudogene	LOC100 755997	100755997	0.00	0.00	0.00	0.00	0.00	0.00	0.00	0.00	0.00	0.00	0.00	0.00
olfactory receptor 5B3-like	LOC100 756002	100756002	0.00	0.00	0.00	0.00	0.00	0.00	0.00	0.00	0.00	0.00	0.00	0.00
olfactory receptor 2D3	LOC100 756004	100756004	0.00	0.00	0.00	0.00	0.00	0.00	0.00	0.00	0.00	0.00	0.00	0.00
zinc finger protein 420-like	LOC100 756005	100756005	0.00	0.00	0.00	0.00	0.00	0.00	0.00	0.00	0.00	0.00	0.00	0.00
collagen alpha-4(IV) chain	LOC100 756006	100756006	0.02	0.00	0.00	0.00	0.00	0.00	0.76	0.00	0.00	0.00	0.00	0.00
26S protease regulatory subunit 8 pseudogene	LOC100 756012	100756012	0.00	0.00	0.00	0.00	0.00	0.00	0.00	0.00	0.00	0.00	0.00	0.00
NUT family member Gm806-like	LOC100 756013	100756013	0.00	0.00	0.00	0.00	0.00	0.00	0.00	0.00	0.00	0.00	0.00	0.00
PRAME family member 12-like	LOC100 756014	100756014	0.00	0.00	0.04	0.00	0.00	0.00	0.00	0.00	1.24	0.00	0.00	0.00
keratin-associated protein 2-1	LOC100 756015	100756015	0.00	0.00	0.00	0.00	0.00	0.00	0.00	0.00	0.00	0.00	0.00	0.00
cytochrome c, somatic pseudogene	LOC100 756016	100756016	0.37	0.11	0.00	0.00	0.00	0.00	1.52	0.00	0.00	0.00	0.00	0.00

olfactory receptor 2D3-like	LOC100756017	100756017	0.00	0.00	0.00	0.00	0.00	0.00	0.00	0.00	0.00	0.00	0.00	0.00
transmembrane protein 92-like	LOC100756018	100756018	0.00	0.00	0.00	0.00	0.00	0.00	0.00	0.00	0.00	0.00	0.00	0.00
A-kinase anchor protein 9-like	LOC100756022	100756022	4.17	3.74	4.16	5.14	4.52	4.81	165.58	146.41	152.78	195.86	183.19	183.20
Testis-specific H1 histone	LOC100756024	100756024	0.00	0.00	0.00	0.00	0.00	0.00	0.00	0.00	0.00	0.00	0.00	0.00
killer cell lectin-like receptor subfamily B member 1F	LOC100756028	100756028	0.00	0.00	0.00	0.00	0.00	0.00	0.00	0.00	0.00	0.00	0.00	0.00
speckle-type POZ protein pseudogene	LOC100756032	100756032	0.04	0.00	0.00	0.00	0.00	0.00	0.00	0.00	0.00	0.00	0.00	0.00
rab GDP dissociation inhibitor beta pseudogene	LOC100756033	100756033	2.37	2.61	2.23	1.59	1.52	1.39	47.85	58.56	45.96	27.81	27.94	24.30
aphrodisin-like	LOC100756037	100756037	0.00	0.00	0.00	0.00	0.00	0.00	0.00	0.00	0.00	0.00	0.00	0.00
STE20-related kinase adapter protein alpha	LOC100756042	100756042	7.31	7.21	6.60	5.69	4.74	4.83	0.00	0.00	0.00	0.00	0.00	0.00
mortality factor 4-like protein 2	LOC100756043	100756043	0.00	0.00	0.00	0.00	0.00	0.00	0.00	0.00	0.00	0.00	0.00	0.00
mast cell protease 1-like	LOC100756053	100756053	0.00	0.00	0.00	0.00	0.00	0.00	0.00	0.00	0.00	0.00	0.00	0.00
protein SET-like	LOC100756057	100756057	1.65	3.23	3.11	1.57	2.24	1.41	12.15	43.01	37.26	18.54	23.80	13.09
chromosome unknown open reading frame, human C12orf43	LOC100756058	100756058	29.30	28.95	30.19	24.01	19.54	20.29	808.91	787.84	803.63	599.16	430.55	436.49
chromosome unknown open reading frame, human C11orf58	LOC100756062	100756062	124.89	123.90	126.65	121.41	122.23	129.53	5218.03	5201.97	5336.01	5065.64	5059.01	5355.66
olfactory receptor 4C45-like	LOC100756064	100756064	0.00	0.00	0.00	0.00	0.00	0.00	0.00	0.00	0.00	0.00	0.00	0.00
voltage-dependent anion-selective channel protein 2 pseudogene	LOC100756067	100756067	0.00	0.00	0.00	0.00	0.00	0.00	0.00	0.00	0.00	0.00	0.00	0.00
selenoprotein K pseudogene	LOC100756068	100756068	0.00	0.00	0.00	0.00	0.00	0.00	0.00	0.00	0.00	0.00	0.00	0.00
28S ribosomal protein L42, mitochondrial-like	LOC100756070	100756070	0.75	1.41	0.62	0.13	0.64	0.26	3.80	11.90	4.97	1.16	5.17	3.74

olfactory receptor 18-like	LOC100756074	100756074	0.00	0.00	0.00	0.00	0.00	0.00	0.00	0.00	0.00	0.00	0.00	0.00
melanoma-associated antigen B3	LOC100756075	100756075	0.00	0.00	0.00	0.00	0.00	0.00	0.00	0.00	0.00	0.00	0.00	0.00
tRNA-splicing endonuclease subunit Sen15 pseudogene	LOC100756077	100756077	26.43	22.11	14.33	2.80	8.25	0.00	36.46	27.45	21.12	5.79	9.31	0.93
ATP synthase subunit g, mitochondrial pseudogene	LOC100756081	100756081	1.34	1.10	1.33	0.65	1.80	0.98	3.04	0.92	3.73	0.00	2.07	0.00
60S ribosomal protein L9 pseudogene	LOC100756082	100756082	0.07	0.24	0.22	0.21	0.09	0.00	0.76	2.75	2.48	2.32	1.03	0.00
peptidyl-prolyl cis-trans isomerase A-like	LOC100756085	100756085	0.00	0.00	0.16	0.15	0.13	0.00	0.00	0.00	1.24	1.16	1.03	0.00
olfactory receptor 6C74-like	LOC100756098	100756098	0.00	0.00	0.00	0.00	0.00	0.00	0.00	0.00	0.00	0.00	0.00	0.00
cyclin-dependent kinase 4 pseudogene	LOC100756101	100756101	0.00	0.00	0.00	0.00	0.00	0.00	0.00	0.00	0.00	0.00	0.00	0.00
alpha-enolase pseudogene	LOC100756105	100756105	15.87	14.74	17.00	17.75	11.51	13.40	557.50	508.76	583.78	593.37	410.89	476.68
phosphoglycerate mutase 1 pseudogene	LOC100756106	100756106	0.00	0.00	0.00	0.00	0.00	0.00	0.00	0.00	0.00	0.00	0.00	0.00
ubiquitin carboxyl-terminal hydrolase isozyme L3-like	LOC100756112	100756112	0.05	0.00	0.00	0.00	0.08	0.00	0.76	0.00	0.00	0.00	1.03	0.00
NACHT, LRR and PYD domains-containing protein 1-like	LOC100756113	100756113	0.11	0.13	0.04	0.07	0.31	0.23	12.91	18.30	6.21	9.27	35.19	31.78
protein FAM111A-like	LOC100756114	100756114	0.00	0.00	0.00	0.00	0.00	0.00	0.00	0.92	0.00	0.00	0.00	0.00
melanoma-associated antigen 10-like	LOC100756116	100756116	0.00	0.00	0.00	0.00	0.00	0.00	0.00	0.00	0.00	0.00	0.00	0.00
cytochrome c oxidase subunit 7A-related protein, mitochondrial	LOC100756118	100756118	0.00	0.00	0.00	0.28	0.00	0.00	0.00	0.00	0.00	1.16	0.00	0.00
39S ribosomal protein L42, mitochondrial	LOC100756124	100756124	0.48	0.23	0.44	0.08	0.26	0.07	6.08	3.66	4.97	2.32	5.17	0.00
chromatin target of PRMT1 protein pseudogene	LOC100756127	100756127	0.09	0.14	0.00	0.00	0.00	0.15	0.76	0.92	0.00	0.00	0.00	0.93
polyhomeotic-like protein 2	LOC100756136	100756136	29.58	29.12	32.08	28.58	22.25	23.24	1999.87	1954.51	2166.20	1913.38	1482.10	1566.51

putative ATP-dependent RNA helicase TDRD12	LOC100756144	100756144	0.00	0.00	0.00	0.00	0.00	0.00	0.00	0.00	0.00	0.00	0.00	0.00
olfactory receptor 2A1/2A42-like	LOC100756155	100756155	0.04	0.06	0.10	0.07	1.00	0.86	3.80	5.49	8.69	5.79	95.22	83.19
SERTA domain-containing protein 2-like	LOC100756164	100756164	0.14	0.10	0.18	0.00	0.04	0.10	3.80	6.41	7.45	0.00	1.03	4.67
mimitin, mitochondrial pseudogene	LOC100756166	100756166	7.37	6.60	6.80	5.66	7.77	7.41	6.84	9.15	12.42	3.48	10.35	2.80
probasin-like	LOC100756179	100756179	0.00	0.00	0.00	0.00	0.00	0.00	0.00	0.00	0.00	0.00	0.00	0.00
40S ribosomal protein S26-like	LOC100756182	100756182	0.46	1.12	0.26	0.00	0.87	0.00	2.28	5.49	1.24	0.00	4.14	0.00
40S ribosomal protein S3 pseudogene	LOC100756184	100756184	0.00	0.00	0.08	0.00	0.00	0.06	0.00	0.00	2.48	1.16	0.00	0.93
TAR DNA-binding protein 43 pseudogene	LOC100756187	100756187	0.00	0.00	0.00	0.00	0.00	0.00	0.00	0.00	0.00	1.16	0.00	0.00
protein S-Myc	LOC100756188	100756188	0.00	0.00	0.00	0.00	0.00	0.00	0.00	0.00	0.00	0.00	0.00	0.00
chromosome unknown open reading frame, human C18orf54	LOC100756190	100756190	5.10	5.13	6.23	6.39	8.00	7.00	328.12	330.33	378.84	397.51	505.07	428.08
60S ribosomal protein L32 pseudogene	LOC100756191	100756191	1.81	1.75	1.51	1.74	1.14	1.27	15.19	15.56	12.42	13.91	9.31	9.35
olfactory receptor 4F15-like	LOC100756192	100756192	0.00	0.04	0.11	0.00	0.05	0.04	0.00	0.92	3.73	0.00	2.07	0.93
aldo-keto reductase family 1 member C13-like	LOC100756193	100756193	0.00	0.00	0.00	0.00	0.00	0.00	0.00	0.00	0.00	0.00	0.00	0.00
protein BEX4-like	LOC100756194	100756194	0.00	0.00	0.00	0.00	0.00	0.00	0.00	0.00	0.00	0.00	0.00	0.00
60S ribosomal protein L27a	LOC100756207	100756207	5.37	4.76	3.62	2.02	3.67	2.76	39.50	37.52	28.57	13.91	26.91	19.63
mitochondrial import receptor subunit TOM20 homolog	LOC100756210	100756210	0.00	0.00	0.00	0.00	0.00	0.00	0.00	0.00	0.00	0.00	0.00	0.00
olfactory receptor 56A3	LOC100756211	100756211	0.00	0.00	0.00	0.00	0.00	0.00	0.00	0.00	0.00	0.00	0.00	0.00
protein CutA homolog	LOC100756214	100756214	0.05	0.00	0.00	0.00	0.03	0.03	3.04	0.00	1.24	2.32	2.07	1.87

speedy protein E4-like	LOC100 756216	100756216	0.03	0.00	0.00	0.00	0.00	0.00	2.28	0.00	0.00	0.00	1.03	1.87
cofilin-1 pseudogene	LOC100 756219	100756219	0.00	0.00	0.00	0.00	0.00	0.00	0.00	0.00	0.00	0.00	0.00	0.00
DCN1-like protein 1 pseudogene	LOC100 756220	100756220	4.68	4.17	5.18	5.39	5.37	4.62	78.99	84.18	104.34	105.46	97.29	86.92
transcription elongation factor B polypeptide 2 pseudogene	LOC100 756224	100756224	0.00	0.00	0.00	0.00	0.00	0.00	0.00	0.00	0.00	0.00	0.00	0.00
olfactory receptor 9	LOC100 756225	100756225	0.00	0.00	0.00	0.00	0.00	0.00	0.00	0.00	0.00	0.00	0.00	0.00
olfactory receptor 1361	LOC100 756229	100756229	0.00	0.00	0.00	0.00	0.00	0.00	0.00	0.00	0.00	0.00	0.00	0.00
tripartite motif-containing protein 60-like	LOC100 756234	100756234	0.00	0.00	0.00	0.00	0.00	0.00	0.00	0.00	0.00	0.00	0.00	0.00
isocitrate dehydrogenase [NAD] subunit alpha, mitochondrial pseudogene	LOC100 756236	100756236	0.02	0.20	0.10	0.09	0.15	0.06	0.00	4.58	2.48	2.32	3.10	0.93
high affinity immunoglobulin epsilon receptor subunit alpha-like	LOC100 756237	100756237	0.00	0.00	0.00	0.00	0.00	0.00	0.00	0.00	0.00	0.00	0.00	0.00
U6 snRNA-associated Sm-like protein LSm3 pseudogene	LOC100 756239	100756239	0.55	0.33	0.61	0.30	0.39	0.35	4.56	2.75	4.97	2.32	3.10	2.80
calcium-binding and coiled-coil domain-containing protein 2-like	LOC100 756241	100756241	0.04	0.00	0.00	0.00	0.00	0.00	1.52	0.00	0.00	0.00	0.00	0.00
chloride intracellular channel protein 5	LOC100 756242	100756242	0.04	0.05	0.00	0.00	0.00	0.00	0.76	0.92	0.00	0.00	1.03	0.00
chromosome unknown open reading frame, human C14orf1	LOC100 756249	100756249	17.72	16.76	19.55	18.51	18.20	20.86	600.04	560.92	654.58	603.80	601.33	688.85
high mobility group protein HMG-I/HMG-Y-like	LOC100 756250	100756250	0.00	0.00	0.00	0.00	0.00	0.00	0.00	0.00	0.00	0.00	0.00	0.00
bile acid-CoA:amino acid N-acyltransferase	LOC100 756252	100756252	0.27	0.35	0.34	0.60	0.48	0.60	25.82	34.77	39.75	57.95	45.54	71.97
zinc finger protein 20-like	LOC100 756255	100756255	0.00	0.00	0.00	0.00	0.00	0.01	1.52	0.00	0.00	0.00	1.03	0.93
olfactory receptor 1013-like	LOC100 756262	100756262	0.00	0.02	0.00	0.02	0.00	0.00	0.00	0.92	0.00	1.16	0.00	0.00
60S ribosomal protein L7a pseudogene	LOC100 756263	100756263	0.08	0.00	0.00	0.00	0.00	0.00	1.52	0.00	0.00	1.16	0.00	0.00

40S ribosomal protein SA pseudogene	LOC100756269	100756269	0.00	0.00	0.00	0.00	0.00	0.00	0.00	0.00	0.00	0.00	0.00	0.00
high mobility group protein B1 pseudogene	LOC100756271	100756271	0.40	0.41	0.33	0.43	0.32	0.20	3.80	4.58	3.73	4.64	6.21	9.35
zinc finger protein 431-like	LOC100756273	100756273	0.00	0.00	0.00	0.00	0.00	0.00	0.00	0.00	0.00	0.00	0.00	0.00
zinc finger protein 383-like	LOC100756278	100756278	1.60	1.52	1.73	2.58	2.39	2.25	154.19	150.07	161.47	244.53	223.56	213.10
olfactory receptor 5B3-like	LOC100756288	100756288	0.00	0.00	0.00	0.00	0.00	0.00	0.00	0.00	0.00	0.00	0.00	0.00
elongation factor 1-alpha 1-like	LOC100756289	100756289	677.74	723.21	739.84	722.72	776.15	665.71	8343.54	8751.38	8993.95	8541.24	9380.08	8124.15
olfactory receptor 2AG2-like	LOC100756290	100756290	0.00	0.00	0.00	0.00	0.00	0.00	0.00	0.00	0.00	0.00	0.00	0.00
zinc finger protein 883-like	LOC100756291	100756291	0.00	0.00	0.00	0.00	0.00	0.00	0.00	0.00	0.00	0.00	0.00	0.00
rhomboid-related protein 4	LOC100756292	100756292	54.50	47.48	49.81	47.94	51.08	52.60	1137.03	1045.88	1063.23	1046.50	1052.58	1111.32
chromosome unknown open reading frame, human C16orf95	LOC100756293	100756293	0.00	0.00	0.00	0.00	0.00	0.04	0.00	0.00	1.24	1.16	0.00	0.93
UDP-glucuronosyltransferase 1-3-like	LOC100756297	100756297	0.00	0.00	0.00	0.00	0.00	0.00	0.00	0.00	0.00	0.00	0.00	0.00
melanoma-associated antigen B3-like	LOC100756298	100756298	0.00	0.00	0.00	0.00	0.00	0.00	0.00	0.00	0.00	0.00	0.00	0.00
putative PRAME family member 25	LOC100756300	100756300	0.00	0.00	0.00	0.00	0.00	0.00	0.00	0.00	0.00	1.16	0.00	0.00
keratin-associated protein 2-1-like	LOC100756301	100756301	0.00	0.00	0.00	0.00	0.00	0.00	0.00	0.00	0.00	0.00	0.00	0.00
transcription elongation factor B polypeptide 1 pseudogene	LOC100756302	100756302	0.72	0.43	0.20	0.39	0.68	0.16	18.23	18.30	13.66	4.64	14.49	14.95
transmembrane protein 92-like	LOC100756304	100756304	0.00	0.00	0.00	0.00	0.00	0.00	0.00	0.00	0.00	0.00	0.00	0.00
40S ribosomal protein S15 pseudogene	LOC100756308	100756308	0.00	0.00	0.00	0.00	0.00	0.00	0.00	0.00	0.00	0.00	0.00	0.00
killer cell lectin-like receptor subfamily B member 1	LOC100756313	100756313	0.00	0.00	0.00	0.00	0.00	0.00	0.00	0.00	0.00	0.00	0.00	0.00

60S acidic ribosomal protein P2 pseudogene	LOC100756316	100756316	4.24	4.50	6.43	5.93	6.48	7.36	132.16	129.93	196.25	195.86	209.07	228.99
speckle-type POZ protein pseudogene	LOC100756317	100756317	0.00	0.00	0.00	0.00	0.00	0.00	0.00	0.00	0.00	0.00	0.00	0.00
carcinoembryonic antigen-related cell adhesion molecule 6-like	LOC100756319	100756319	0.00	0.00	0.00	0.00	0.00	0.00	0.00	0.00	0.00	0.00	0.00	0.00
olfactory receptor 10-like	LOC100756320	100756320	0.00	0.00	0.00	0.00	0.00	0.04	0.00	0.00	0.00	0.00	0.00	0.93
hemojuvelin	LOC100756322	100756322	0.01	0.01	0.00	0.02	0.00	0.00	0.00	0.00	0.00	0.00	0.00	0.00
focadhesin	LOC100756324	100756324	20.12	19.58	15.09	15.26	16.24	17.72	1548.70	1527.19	1193.65	1258.59	1283.38	1292.65
UBX domain-containing protein 1	LOC100756326	100756326	387.08	391.67	458.76	466.99	379.82	415.99	1997.59	2095.43	2353.75	2265.69	2043.06	2137.59
resistin-like beta	LOC100756330	100756330	0.00	0.00	0.00	0.00	0.00	0.00	0.00	0.00	0.00	0.00	0.00	0.00
coiled-coil domain-containing protein 171	LOC100756343	100756343	0.43	0.44	0.42	0.28	0.24	0.52	39.50	37.52	38.50	20.86	25.87	32.71
putative nmrA-like family domain-containing protein ENSP00000305954	LOC100756347	100756347	0.00	0.00	0.00	0.00	0.00	0.00	0.00	0.00	0.00	0.00	1.03	0.00
olfactory receptor 4A47	LOC100756351	100756351	0.00	0.00	0.00	0.00	0.00	0.00	0.00	0.00	0.00	0.00	0.00	0.00
alpha-enolase-like	LOC100756361	100756361	0.00	0.00	0.00	0.00	0.00	0.00	0.00	0.00	0.00	0.00	0.00	0.00
melanoma-associated antigen B2-like	LOC100756362	100756362	0.05	0.06	0.00	0.00	0.00	0.04	2.28	0.92	0.00	0.00	0.00	0.93
ankyrin repeat family A protein 2 pseudogene	LOC100756364	100756364	0.00	0.00	0.05	0.00	0.00	0.00	0.00	1.83	2.48	0.00	0.00	0.00
heterogeneous nuclear ribonucleoprotein D-like pseudogene	LOC100756371	100756371	0.00	0.00	0.00	0.00	0.00	0.00	0.00	0.00	0.00	0.00	0.00	0.00
putative vomeronasal receptor-like protein 4	LOC100756373	100756373	0.04	0.00	0.00	0.00	0.05	0.00	0.76	0.00	0.00	0.00	0.00	0.00
olfactory receptor 6C70	LOC100756387	100756387	0.00	0.00	0.00	0.00	0.00	0.00	0.00	0.00	0.00	0.00	0.00	0.00
28S ribosomal protein S21, mitochondrial pseudogene	LOC100756388	100756388	0.00	0.00	0.00	0.00	0.00	0.00	0.00	0.00	0.00	0.00	1.03	0.00

rho guanine nucleotide exchange factor 10-like protein	LOC100 756389	100756389	8.52	8.17	11.21	9.11	5.59	6.82	573.45	555.43	741.53	609.59	369.49	436.49
Ig heavy chain V-III region VH26-like	LOC100 756391	100756391	0.00	0.00	0.00	0.00	0.00	0.00	0.00	0.00	0.00	0.00	0.00	0.00
zinc finger protein Pegasus-like	LOC100 756393	100756393	0.00	0.00	0.00	0.00	0.00	0.00	0.00	0.00	0.00	0.00	0.00	0.00
cytochrome c oxidase assembly factor 7	LOC100 756398	100756398	9.98	10.15	10.20	11.05	9.08	9.43	977.53	1002.88	1022.24	1106.77	901.47	930.00
U6 snRNA-associated Sm-like protein LSm5 pseudogene	LOC100 756402	100756402	0.00	0.00	0.00	0.00	0.00	0.00	0.00	0.00	0.00	0.00	0.00	0.00
mothers against decapentaplegic homolog 1-like	LOC100 756412	100756412	0.00	0.00	0.00	0.00	0.00	0.00	0.00	0.00	0.00	0.00	0.00	0.00
condensin-2 complex subunit H2 pseudogene	LOC100 756414	100756414	0.11	0.22	0.08	0.07	0.15	0.17	5.32	10.98	3.73	4.64	7.24	8.41
40S ribosomal protein S26-like	LOC100 756416	100756416	0.00	0.00	0.00	0.00	0.00	0.00	0.00	0.00	0.00	0.00	0.00	0.00
interferon-induced guanylate-binding protein 2-like	LOC100 756417	100756417	0.01	0.01	0.00	0.00	0.00	0.03	0.00	0.00	1.24	0.00	0.00	0.93
LIM homeobox transcription factor 1-beta	LOC100 756418	100756418	9.16	7.32	14.28	10.37	7.67	8.24	235.46	203.14	392.50	256.12	201.82	208.43
heat shock protein HSP 90-alpha pseudogene	LOC100 756421	100756421	0.00	0.00	0.00	0.00	0.00	0.00	0.00	0.00	0.00	0.00	0.00	0.00
60S ribosomal protein L3-like	LOC100 756423	100756423	0.02	0.01	0.08	0.04	0.17	0.00	0.76	0.00	2.48	1.16	5.17	0.00
histone H2B type 1-A	LOC100 756432	100756432	0.04	0.05	0.05	0.00	0.04	0.00	0.00	0.00	0.00	0.00	0.00	0.00
polyamine-modulated factor 1	LOC100 756436	100756436	63.71	61.45	61.78	54.57	51.70	54.64	1684.66	1657.13	1642.04	1438.22	1397.23	1446.87
survival motor neuron protein	LOC100 756440	100756440	86.06	87.01	86.91	83.48	85.01	86.84	2918.15	2988.50	2962.38	2817.33	2849.32	2898.41
olfactory receptor 141-like	LOC100 756448	100756448	0.00	0.00	0.00	0.00	0.00	0.00	0.00	0.00	0.00	0.00	0.00	0.00
ankyrin-2-like	LOC100 756453	100756453	0.00	0.00	0.00	0.00	0.00	0.00	0.00	0.00	0.00	0.00	0.00	0.00
sodium-dependent phosphate transporter 1 pseudogene	LOC100 756454	100756454	0.05	0.06	0.00	0.02	0.02	0.06	3.04	3.66	0.00	1.16	2.07	3.74

major facilitator superfamily domain-containing protein 9-like	LOC100756467	100756467	0.00	0.00	0.00	0.00	0.00	0.00	0.00	0.00	0.00	0.00	0.00	0.00
aldehyde dehydrogenase family 3 member B1-like	LOC100756470	100756470	0.04	0.02	0.00	0.08	0.05	0.02	3.04	1.83	0.00	4.64	2.07	1.87
T-complex protein 1 subunit beta pseudogene	LOC100756476	100756476	0.00	0.00	0.00	0.00	0.00	0.00	0.00	0.00	0.00	0.00	0.00	0.00
arylacetamide deacetylase-like 4	LOC100756477	100756477	0.00	0.00	0.00	0.00	0.00	0.00	0.00	0.00	0.00	0.00	0.00	0.00
olfactory receptor 4K3	LOC100756478	100756478	0.00	0.00	0.00	0.00	0.00	0.04	0.00	0.00	0.00	0.00	0.00	0.93
uncharacterized LOC100756479	LOC100756479	100756479	9.24	8.73	8.73	8.37	10.47	10.72	312.93	290.98	286.92	270.03	360.18	357.04
40S ribosomal protein S6 pseudogene	LOC100756482	100756482	0.47	0.46	0.36	0.30	0.30	0.78	14.43	14.64	9.94	12.75	9.31	22.43
olfactory receptor 4F6-like	LOC100756484	100756484	0.00	0.00	0.17	0.11	0.12	0.15	0.76	0.00	3.73	3.48	3.10	4.67
aldo-keto reductase family 1 member C21-like	LOC100756485	100756485	0.00	0.00	0.00	0.00	0.00	0.08	1.52	0.00	1.24	1.16	0.00	1.87
glyceraldehyde-3-phosphate dehydrogenase pseudogene	LOC100756490	100756490	244.71	241.31	188.87	94.44	45.71	48.09	2698.64	2732.29	2092.92	929.45	465.74	462.66
zinc finger protein DZIP1 pseudogene	LOC100756492	100756492	0.00	0.00	0.00	0.00	0.00	0.00	0.00	0.00	0.00	0.00	0.00	0.00
protein CTLA-2-beta-like	LOC100756494	100756494	1.41	2.20	1.62	2.02	1.92	2.24	33.42	47.58	43.47	40.56	45.54	57.01
chromosome unknown open reading frame, human C17orf103	LOC100756496	100756496	9.01	9.12	9.72	10.36	9.77	10.21	802.07	815.29	875.67	925.98	868.35	911.30
olfactory receptor 52L1	LOC100756502	100756502	0.00	0.00	0.00	0.00	0.00	0.00	0.00	0.00	0.00	0.00	0.00	0.00
aryl hydrocarbon receptor repressor	LOC100756505	100756505	15.71	15.42	18.28	19.22	20.01	19.85	2079.62	2046.93	2446.91	2558.90	2639.22	2644.18
speedy protein E4-like	LOC100756506	100756506	0.03	0.00	0.00	0.00	0.00	0.00	2.28	0.00	2.48	0.00	1.03	0.93
bystin-like	LOC100756507	100756507	3.54	2.31	2.64	2.08	2.05	2.06	67.60	39.35	55.89	39.40	34.15	42.06
cryptic protein-like	LOC100756508	100756508	0.00	0.00	0.00	0.00	0.00	0.00	0.00	0.00	0.00	0.00	0.00	0.00

dual 3',5'-cyclic-AMP and -GMP phosphodiesterase 11A	LOC100 756511	100756511	0.00	0.00	0.00	0.00	0.00	0.00	0.00	0.00	0.00	0.00	0.00	0.00
endoplasmic reticulum mannosyl-oligosaccharide 1,2-alpha-mannosidase	LOC100 756512	100756512	47.60	47.51	47.61	47.26	44.67	45.69	3529.58	3508.24	3638.07	3533.55	3362.67	3617.17
ubiquitin-60S ribosomal protein L40-like	LOC100 756515	100756515	18.33	23.98	16.50	11.78	15.78	12.09	62.28	67.71	40.99	39.40	43.47	31.78
26S proteasome non-ATPase regulatory subunit 13-like	LOC100 756516	100756516	9.40	7.54	7.85	8.44	9.29	7.52	262.80	214.12	224.82	239.90	261.85	214.04
zinc finger protein 709-like	LOC100 756518	100756518	8.82	8.86	12.37	14.04	12.63	12.79	518.01	518.82	731.59	828.63	740.02	755.21
peptidyl-prolyl cis-trans isomerase A pseudogene	LOC100 756519	100756519	0.00	0.00	0.00	0.00	0.00	0.03	0.00	0.00	0.00	0.00	0.00	0.93
keratin-associated protein 2-1	LOC100 756520	100756520	0.00	0.00	0.00	0.00	0.00	0.00	0.00	0.00	0.00	0.00	0.00	0.00
olfactory receptor 10J1	LOC100 756527	100756527	0.00	0.00	0.00	0.00	0.00	0.00	0.00	0.00	0.00	0.00	0.00	0.00
60S acidic ribosomal protein P1 pseudogene	LOC100 756532	100756532	0.00	0.00	0.00	0.00	0.00	0.00	0.76	1.83	1.24	0.00	0.00	0.93
probable ribosome biogenesis protein RLP24 pseudogene	LOC100 756539	100756539	0.07	0.00	0.00	0.00	0.10	0.00	0.76	0.00	0.00	0.00	1.03	0.00
cytochrome c oxidase subunit 7A2, mitochondrial	LOC100 756540	100756540	278.97	295.30	230.98	232.01	243.36	236.00	2685.73	2831.11	2141.36	2082.58	2215.91	2105.81
acyl-coenzyme A amino acid N-acyltransferase 2-like	LOC100 756541	100756541	0.00	0.00	0.04	0.00	0.00	0.03	0.00	0.00	1.24	0.00	0.00	0.93
ribosome biogenesis regulatory protein homolog	LOC100 756542	100756542	0.05	0.00	0.09	0.08	0.00	0.00	0.76	0.00	2.48	1.16	0.00	0.00
zinc finger protein 878-like	LOC100 756545	100756545	1.53	1.50	2.01	2.27	2.31	2.00	109.37	110.72	150.29	163.41	164.56	143.94
glycine cleavage system H protein, mitochondrial-like	LOC100 756550	100756550	1.97	2.19	1.23	1.90	1.93	1.35	18.99	13.73	4.97	11.59	10.35	9.35
olfactory receptor 1013-like	LOC100 756552	100756552	0.00	0.00	0.00	0.00	0.00	0.00	0.00	0.00	0.00	0.00	0.00	0.00
tubulin alpha-1B chain-like	LOC100 756553	100756553	0.01	0.00	0.00	0.01	0.00	0.00	0.00	0.00	0.00	0.00	0.00	0.00
acyl-CoA-binding protein	LOC100 756555	100756555	0.00	0.00	0.00	0.51	0.00	0.00	0.00	0.00	0.00	0.00	0.00	0.00

chromosome unknown open reading frame, human C1orf68	LOC100756558	100756558	0.00	0.00	0.00	0.00	0.00	0.00	0.00	0.00	0.00	0.00	0.00	0.00
testis-specific serine/threonine-protein kinase 5-like	LOC100756562	100756562	0.00	0.04	0.00	0.00	0.00	0.00	2.28	7.32	2.48	8.11	2.07	6.54
trans-1,2-dihydrobenzene-1,2-diol dehydrogenase pseudogene	LOC100756563	100756563	0.00	0.00	0.00	0.00	0.00	0.00	0.00	0.00	0.00	0.00	0.00	0.00
olfactory receptor 11G2-like	LOC100756567	100756567	0.00	0.00	0.00	0.00	0.00	0.00	0.00	0.00	0.00	0.00	0.00	0.00
glucosamine 6-phosphate N-acetyltransferase pseudogene	LOC100756573	100756573	0.28	0.37	0.15	0.10	0.17	0.08	3.80	6.41	2.48	1.16	2.07	0.93
trafficking protein particle complex subunit 9-like	LOC100756575	100756575	7.58	7.27	8.71	5.86	5.92	6.56	201.28	185.75	233.51	152.98	173.88	171.98
olfactory receptor 5B3-like	LOC100756581	100756581	0.00	0.00	0.00	0.00	0.00	0.00	0.00	0.00	0.00	0.00	0.00	0.00
60S ribosomal protein L7 pseudogene	LOC100756583	100756583	2.12	2.06	2.11	1.23	1.68	0.96	29.62	27.45	31.05	15.07	20.70	11.22
zinc finger protein 595-like	LOC100756584	100756584	0.00	0.00	0.00	0.00	0.00	0.00	0.00	0.00	0.00	0.00	0.00	0.00
UDP-glucuronosyltransferase 1-5-like	LOC100756590	100756590	0.02	0.04	0.00	0.03	0.02	0.04	0.00	0.00	0.00	0.00	0.00	0.00
melanoma-associated antigen B4-like	LOC100756591	100756591	0.00	0.00	0.00	0.00	0.00	0.00	0.00	0.00	0.00	0.00	0.00	0.00
selenoprotein W pseudogene	LOC100756592	100756592	4.65	2.40	2.67	5.20	1.84	4.85	75.95	52.16	38.50	61.42	25.87	57.01
ribosome biogenesis regulatory protein homolog	LOC100756594	100756594	0.00	0.03	0.00	0.00	0.00	0.00	1.52	1.83	1.24	0.00	1.03	0.00
keratin-associated protein 9-3	LOC100756595	100756595	0.00	0.00	0.00	0.00	0.00	0.00	0.00	0.00	0.00	0.00	0.00	0.00
interferon-induced very large GTPase 1	LOC100756597	100756597	0.00	0.00	0.00	0.00	0.00	0.00	0.00	0.00	0.00	0.00	0.00	0.00
vomeronasal type-1 receptor 4-like	LOC100756598	100756598	0.00	0.00	0.00	0.00	0.00	0.00	0.00	0.00	0.00	0.00	0.00	0.00
protein SAAL1-like	LOC100756600	100756600	2.83	2.80	3.24	3.21	4.43	3.80	229.38	236.08	247.18	260.76	301.18	302.83
oocyte-secreted protein 1-like	LOC100756603	100756603	0.00	0.00	0.00	0.00	0.00	0.00	0.00	0.00	0.00	0.00	0.00	0.93

alcohol dehydrogenase [NADP(+)] pseudogene	LOC100 756608	100756608	0.00	0.00	0.00	0.00	0.00	0.00	0.00	0.00	0.00	0.00	0.00	0.00
uncharacterized protein C17orf105 homolog	LOC100 756610	100756610	0.00	0.00	0.00	0.00	0.00	0.00	0.00	0.00	0.00	0.00	0.00	0.00
ligand-dependent corepressor	LOC100 756611	100756611	8.11	7.80	6.58	6.63	7.57	7.49	1156.02	1149.28	956.41	983.92	1134.34	1116.00
ADAMTS-like protein 3	LOC100 756612	100756612	0.03	0.03	0.01	0.04	0.08	0.01	2.28	3.66	1.24	3.48	5.17	0.93
olfactory receptor 10-like	LOC100 756616	100756616	0.00	0.00	0.00	0.00	0.00	0.00	0.00	0.00	0.00	0.00	0.00	0.00
alcohol dehydrogenase 1	LOC100 756617	100756617	0.00	0.01	0.00	0.00	0.00	0.00	0.00	0.00	0.00	0.00	0.00	0.93
60S ribosomal protein L32-like	LOC100 756619	100756619	5.47	4.56	4.51	3.10	4.70	3.85	107.10	120.78	173.89	197.02	183.19	171.04
interferon-induced very large GTPase 1	LOC100 756621	100756621	0.02	0.01	0.01	0.01	0.00	0.00	3.80	2.75	1.24	2.32	0.00	0.00
zinc finger protein 431-like	LOC100 756623	100756623	0.00	0.03	0.00	0.02	0.03	0.03	0.00	0.92	0.00	0.00	1.03	0.93
resistin-like beta	LOC100 756627	100756627	0.12	0.00	0.19	0.00	0.16	0.00	1.52	0.92	2.48	0.00	1.03	0.93
60S ribosomal protein L17 pseudogene	LOC100 756629	100756629	0.00	0.08	0.00	0.00	0.00	0.09	0.00	0.92	0.00	0.00	0.00	0.00
60S ribosomal protein L21 pseudogene	LOC100 756634	100756634	4.64	4.27	4.29	3.20	4.09	2.45	21.27	20.13	32.29	23.18	26.91	12.15
chromosome unknown open reading frame, human C8orf4	LOC100 756639	100756639	0.00	0.00	0.00	0.00	0.00	0.00	0.00	0.00	0.00	0.00	0.00	0.00
U6 snRNA-associated Sm-like protein LSm5 pseudogene	LOC100 756645	100756645	0.00	0.00	0.00	0.00	0.00	0.00	0.00	0.00	0.00	0.00	0.00	0.00
olfactory receptor 4A5-like	LOC100 756650	100756650	0.00	0.00	0.00	0.00	0.00	0.00	0.00	0.00	0.00	0.00	0.00	0.00
tetratricopeptide repeat protein 39B-like	LOC100 756657	100756657	0.16	0.12	0.07	0.11	0.10	0.12	9.11	8.24	3.73	5.79	5.17	7.48
Wiskott-Aldrich syndrome protein family member 1	LOC100 756660	100756660	0.01	0.02	0.02	0.02	0.00	0.00	0.76	0.92	1.24	1.16	0.00	0.00
olfactory receptor 7E24	LOC100 756661	100756661	0.00	0.00	0.00	0.00	0.00	0.00	0.00	0.00	0.00	0.00	0.00	0.00

beta-hexosaminidase subunit beta-like	LOC100756663	100756663	7.48	7.82	10.23	11.28	13.06	11.92	265.08	280.00	399.95	433.44	527.84	477.62
proteasome subunit alpha type-7 pseudogene	LOC100756664	100756664	0.00	0.00	0.00	0.00	0.00	0.00	0.00	0.00	0.00	0.00	0.00	0.00
sperm motility kinase W	LOC100756665	100756665	0.00	0.00	0.00	0.00	0.00	0.00	0.00	0.00	0.00	0.00	0.00	0.00
cocaine esterase-like	LOC100756666	100756666	0.00	0.00	0.00	0.00	0.00	0.00	0.00	0.00	0.00	0.00	0.00	0.00
60 kDa heat shock protein, mitochondrial pseudogene	LOC100756669	100756669	0.61	0.83	0.80	0.48	0.31	0.58	28.10	38.43	36.02	20.86	14.49	26.17
alpha-actinin-3	LOC100756672	100756672	10.15	10.21	12.46	11.52	8.32	9.50	1104.37	1128.24	1368.78	1241.20	911.82	1032.81
putative vomeronasal receptor-like protein 4	LOC100756674	100756674	0.00	0.00	0.00	0.00	0.00	0.00	0.00	0.00	0.00	0.00	0.00	0.00
interferon-induced transmembrane protein 2	LOC100756685	100756685	115.51	107.24	118.98	122.31	112.58	105.70	2467.74	2278.43	2584.78	2637.70	2398.06	2263.77
vacuolar protein sorting-associated protein 26A-like	LOC100756686	100756686	0.00	0.00	0.00	0.00	0.00	0.00	0.00	0.00	0.00	0.00	0.00	0.00
olfactory receptor 6C76	LOC100756688	100756688	0.00	0.00	0.00	0.00	0.00	0.00	0.00	0.00	0.00	0.00	0.00	0.00
serine/arginine-rich splicing factor 3 pseudogene	LOC100756689	100756689	0.85	0.95	0.91	0.38	0.91	0.48	22.03	21.05	23.60	16.22	20.70	15.89
Ig heavy chain V-II region SESS-like	LOC100756692	100756692	0.00	0.00	0.00	0.00	0.00	0.00						
olfactory receptor 14A16-like	LOC100756694	100756694	0.00	0.00	0.00	0.00	0.00	0.00	0.00	0.00	0.00	0.00	0.00	0.00
cytochrome b-c1 complex subunit 7-like	LOC100756699	100756699	0.24	0.30	0.82	0.00	0.00	0.47	4.56	2.75	4.97	0.00	0.00	3.74
60S ribosomal protein L15 pseudogene	LOC100756700	100756700	7.26	5.12	5.40	2.77	3.29	3.26	113.93	85.10	80.74	42.88	50.71	51.41
C-type lectin domain family 2 member H-like	LOC100756701	100756701	0.00	0.00	0.14	0.04	0.02	0.00	0.00	0.00	0.00	0.00	1.03	0.00
NACHT, LRR and PYD domains-containing protein 1-like	LOC100756703	100756703	0.00	0.00	0.00	0.00	0.00	0.00	0.00	0.00	0.00	0.00	0.00	0.00
selection and upkeep of intraepithelial T-cells protein 2-like	LOC100756707	100756707	0.00	0.00	0.00	0.00	0.00	0.00	0.00	0.92	0.00	0.00	0.00	0.00

14-3-3 protein eta-like	LOC100756708	100756708	0.00	0.00	0.00	0.00	0.00	0.00	0.00	0.00	0.00	0.00	0.00	0.00
chromosome unknown open reading frame, human C9orf116	LOC100756710	100756710	1.54	1.74	1.84	1.39	1.55	1.47	44.05	50.33	45.96	42.88	39.33	42.99
piwi-like protein 1	LOC100756712	100756712	0.00	0.00	0.00	0.00	0.00	0.00	0.00	0.92	0.00	0.00	0.00	0.93
[3-methyl-2-oxobutanoate dehydrogenase [lipoamide]] kinase, mitochondrial pseudogene	LOC100756715	100756715	0.12	0.09	0.13	0.20	0.20	0.16	2.28	2.75	3.73	5.79	5.17	3.74
ATPase family AAA domain-containing protein 2	LOC100756717	100756717	206.11	207.51	139.19	175.23	182.88	165.80	1974.04	1980.13	1326.55	1565.70	1744.99	1563.70
olfactory receptor 14A2-like	LOC100756718	100756718	0.00	0.00	0.00	0.00	0.00	0.00	0.00	0.00	0.00	0.00	0.00	0.00
reactive oxygen species modulator 1	LOC100756722	100756722	3.70	1.66	2.82	0.66	2.09	1.95	6.08	3.66	7.45	0.00	3.10	1.87
sentrin-specific protease 2-like	LOC100756725	100756725	0.00	0.00	0.00	0.06	0.00	0.00	0.00	0.00	0.00	2.32	1.03	0.00
histone H2A type 4	LOC100756730	100756730	0.00	0.15	0.20	0.00	0.00	0.00	0.00	0.00	1.24	0.00	0.00	0.00
GTPase ERas-like	LOC100756735	100756735	0.00	0.00	0.00	0.00	0.07	0.00	0.00	0.00	0.00	0.00	1.03	0.00
prothymosin alpha-like	LOC100756745	100756745	0.83	0.65	0.66	0.68	0.33	0.31	19.75	14.64	14.91	16.22	21.73	10.28
olfactory receptor 8H3-like	LOC100756747	100756747	0.00	0.00	0.00	0.00	0.00	0.00	0.00	0.00	0.00	0.00	0.00	0.00
cytochrome P450 26B1	LOC100756748	100756748	0.03	0.10	0.15	0.02	0.03	0.03	2.28	6.41	9.94	1.16	2.07	1.87
40S ribosomal protein S27-like	LOC100756750	100756750	22.07	22.21	13.49	11.66	19.93	15.59	47.85	45.75	21.12	19.70	38.29	34.58
vomeronasal type-2 receptor 116	LOC100756752	100756752	0.00	0.00	0.00	0.00	0.00	0.00	0.00	0.00	0.00	0.00	0.00	0.00
cytochrome P450 2C70-like	LOC100756757	100756757	0.00	0.02	0.00	0.00	0.03	0.00	0.00	0.92	0.00	0.00	2.07	0.00
protocadherin gamma-A5-like	LOC100756758	100756758	0.00	0.01	0.00	0.02	0.01	0.02	0.76	0.92	0.00	2.32	1.03	4.67
secretoglobin family 2A member 2-like	LOC100756762	100756762	0.17	0.31	0.00	0.40	0.12	0.21	3.04	3.66	1.24	4.64	3.10	5.61

ETS domain-containing transcription factor ERF	LOC100 756763	100756763	5.42	5.11	5.93	4.19	1.87	1.44	49.37	45.75	58.38	32.45	14.49	14.02
ras-related protein Rap-1A-like	LOC100 756765	100756765	2.33	1.39	0.99	0.55	1.14	1.13	42.53	27.45	18.63	13.91	17.59	21.50
ubiquitin-60S ribosomal protein L40 pseudogene	LOC100 756766	100756766	0.00	0.00	0.00	0.00	0.00	0.00	2.28	0.92	0.00	0.00	1.03	0.00
odorant-binding protein-like	LOC100 756767	100756767	0.00	0.00	0.00	0.00	0.00	0.00	0.00	0.00	0.00	0.00	0.00	0.00
motile sperm domain-containing protein 1 pseudogene	LOC100 756773	100756773	1.02	1.48	1.34	1.40	2.07	1.61	25.06	37.52	22.36	19.70	41.40	35.52
olfactory receptor 4F3/4F16/4F29-like	LOC100 756775	100756775	0.00	0.00	0.03	0.00	0.00	0.00	0.00	0.00	0.00	0.00	0.00	0.00
olfactory receptor 4K2-like	LOC100 756781	100756781	0.00	0.00	0.00	0.00	0.00	0.00	0.00	0.00	0.00	0.00	0.00	0.00
60S ribosomal protein L27a pseudogene	LOC100 756782	100756782	0.11	0.23	0.05	0.05	0.20	0.10	0.00	0.92	0.00	0.00	1.03	0.00
protein CTLA-2-beta	LOC100 756791	100756791	114.29	111.78	64.06	77.74	87.16	87.72	2024.93	1994.77	1181.22	1299.15	1524.53	1506.69
olfactory receptor 56A4-like	LOC100 756799	100756799	0.00	0.00	0.00	0.00	0.00	0.00	0.00	0.00	0.00	0.00	0.00	0.00
protein Dr1 pseudogene	LOC100 756801	100756801	0.42	0.25	0.23	0.11	0.20	0.09	4.56	1.83	2.48	0.00	2.07	0.93
inorganic pyrophosphatase 2, mitochondrial-like	LOC100 756805	100756805	129.09	131.06	105.00	109.45	124.70	128.96	1926.19	2065.23	1649.49	1663.05	1905.41	1876.82
protein FAM187B	LOC100 756806	100756806	0.00	0.00	0.00	0.00	0.00	0.00	0.00	0.00	0.00	0.00	0.00	0.00
vomeronasal type-2 receptor 116-like	LOC100 756808	100756808	0.00	0.00	0.00	0.00	0.00	0.00	0.00	0.00	0.00	0.00	0.00	0.00
vomeronasal type-1 receptor 4-like	LOC100 756810	100756810	0.00	0.00	0.00	0.00	0.00	0.00	0.00	0.00	0.00	0.00	0.00	0.00
chromosome unknown open reading frame, human C7orf31	LOC100 756811	100756811	6.05	6.36	7.29	8.24	9.85	9.15	343.31	355.03	412.37	450.82	566.14	513.13
DCN1-like protein 5 pseudogene	LOC100 756812	100756812	0.00	0.10	0.13	0.13	0.00	0.00	0.00	1.83	3.73	2.32	0.00	0.00
zinc finger protein 112 pseudogene	LOC100 756814	100756814	4.91	4.44	5.03	4.94	4.93	5.29	22.03	11.90	17.39	16.22	6.21	15.89

tripartite motif-containing protein 75-like	LOC100756820	100756820	0.06	0.08	0.02	0.05	0.06	0.04	3.80	5.49	2.48	3.48	6.21	2.80
regulator of chromosome condensation-like	LOC100756821	100756821	0.03	0.00	0.00	0.00	0.00	0.06	0.00	0.00	0.00	0.00	0.00	0.00
taste receptor type 2 member 119	LOC100756822	100756822	0.00	0.00	0.00	0.00	0.00	0.00	0.00	0.00	0.00	0.00	0.00	0.00
high affinity immunoglobulin epsilon receptor subunit alpha-like	LOC100756823	100756823	0.00	0.00	0.00	0.00	0.00	0.00	0.00	0.00	0.00	0.00	0.00	0.00
phenylalanine--tRNA ligase alpha subunit pseudogene	LOC100756824	100756824	0.00	0.00	0.00	0.00	0.00	0.00	0.00	0.00	0.00	0.00	0.00	0.00
zinc finger protein ZFP69-like	LOC100756827	100756827	3.81	3.45	4.14	4.11	4.53	4.15	185.33	173.86	207.43	215.56	226.66	218.71
glyceraldehyde-3-phosphate dehydrogenase-like	LOC100756828	100756828	0.00	0.00	0.00	0.00	0.00	0.00	0.00	0.00	0.00	0.00	0.00	0.00
eukaryotic translation initiation factor 3 subunit G pseudogene	LOC100756832	100756832	0.04	0.17	0.00	0.05	0.17	0.09	0.76	3.66	0.00	1.16	3.10	1.87
60S ribosomal protein L36a-like	LOC100756833	100756833	0.96	0.31	0.76	0.23	0.21	0.09	0.00	0.00	0.00	0.00	0.00	0.00
lipid phosphate phosphatase-related protein type 1	LOC100756834	100756834	0.00	0.00	0.00	0.00	0.00	0.00	0.00	0.00	0.00	0.00	0.00	0.00
zinc finger protein 14-like	LOC100756838	100756838	2.23	2.08	1.53	2.16	2.69	2.68	119.25	110.72	86.95	118.21	150.07	147.68
ubiquitin-like domain-containing CTD phosphatase 1 pseudogene	LOC100756841	100756841	0.00	0.00	0.00	0.00	0.00	0.00	0.00	0.00	0.00	0.00	0.00	0.00
40S ribosomal protein S29 pseudogene	LOC100756842	100756842	0.00	0.00	0.00	0.00	0.00	0.00	3.04	0.00	0.00	0.00	0.00	0.00
selenoprotein K pseudogene	LOC100756843	100756843	0.06	0.12	0.00	0.00	0.00	0.00	0.76	0.92	0.00	1.16	0.00	0.93
60S ribosomal protein L6 pseudogene	LOC100756844	100756844	0.00	0.00	0.00	0.00	0.00	0.00	0.00	0.00	0.00	0.00	0.00	0.00
olfactory receptor 1013	LOC100756845	100756845	0.59	0.77	0.40	0.39	0.32	0.29	46.33	54.90	28.57	30.13	23.80	22.43
60S ribosomal protein L26 pseudogene	LOC100756848	100756848	0.00	0.00	0.00	0.00	0.00	0.00	0.00	0.00	0.00	0.00	0.00	0.00
GTPase ERas-like	LOC100756851	100756851	0.00	0.00	0.00	0.00	0.00	0.00	0.00	0.00	0.00	0.00	0.00	0.00

sphingomyelin phosphodiesterase 3	LOC100756855	100756855	0.02	0.00	0.00	0.00	0.03	0.00	3.04	0.92	1.24	0.00	2.07	1.87
mitochondrial inner membrane protease ATP23 homolog	LOC100756856	100756856	0.00	0.00	0.00	0.00	0.00	0.00	0.00	0.00	0.00	0.00	0.00	0.00
PRAME family member 6-like	LOC100756857	100756857	0.00	0.00	0.00	0.00	0.00	0.00	0.00	0.00	0.00	0.00	0.00	0.00
olfactory receptor 11H6	LOC100756859	100756859	0.00	0.00	0.00	0.00	0.00	0.00	0.00	0.00	0.00	0.00	0.00	0.00
protein kinase C zeta type	LOC100756860	100756860	1.33	1.13	0.59	0.46	0.46	0.21	101.78	106.14	69.56	49.83	55.89	34.58
60S ribosomal protein L22 pseudogene	LOC100756861	100756861	5.57	9.50	8.74	7.51	9.61	4.53	329.64	299.22	276.99	319.86	333.27	344.89
zinc finger protein 2 homolog	LOC100756863	100756863	1.56	1.41	1.77	1.88	2.00	1.72	212.67	196.73	245.93	265.39	277.38	245.82
non-histone chromosomal protein HMG-17 pseudogene	LOC100756866	100756866	0.00	0.00	0.00	0.00	0.00	0.00	0.00	0.00	0.00	0.00	0.00	0.00
olfactory receptor 5B2-like	LOC100756873	100756873	0.00	0.00	0.00	0.00	0.00	0.00	0.00	0.00	0.00	0.00	0.00	0.00
adenylosuccinate lyase-like	LOC100756874	100756874	0.00	0.00	0.00	0.00	0.03	0.00	0.00	0.00	0.00	0.00	1.03	0.00
zinc finger protein 678-like	LOC100756876	100756876	0.00	0.00	0.00	0.00	0.00	0.00	0.00	0.00	0.00	0.00	0.00	0.00
UDP-glucuronosyltransferase 1-9-like	LOC100756880	100756880	0.12	0.21	0.03	0.05	0.06	0.00	7.60	7.32	0.00	3.48	3.10	1.87
phosphatidylinositol N-acetylglucosaminyltransferase subunit Y-like	LOC100756881	100756881	0.00	0.00	0.00	0.00	0.00	0.00	0.00	0.00	0.00	0.00	0.00	0.00
chromosome unknown open reading frame, human C21orf59	LOC100756882	100756882	28.92	30.84	35.14	35.84	26.71	27.57	835.49	906.80	1022.24	1008.26	781.41	781.38
zinc finger FYVE domain-containing protein 1 pseudogene	LOC100756885	100756885	0.00	0.00	0.00	0.00	0.00	0.07	0.00	0.00	0.00	0.00	0.00	0.93
keratin-associated protein 2-1	LOC100756886	100756886	0.00	0.00	0.00	0.00	0.00	0.00	0.00	0.00	0.00	0.00	0.00	0.00
chromosome unknown open reading frame, human C11orf70	LOC100756887	100756887	0.13	0.16	0.16	0.20	0.09	0.29	4.56	6.41	4.97	4.64	2.07	6.54
60S ribosomal protein L5 pseudogene	LOC100756888	100756888	0.00	0.00	0.00	0.00	0.00	0.00	0.76	0.00	1.24	0.00	0.00	0.00

vomeronasal type-1 receptor 4-like	LOC100756889	100756889	0.00	0.00	0.00	0.00	0.00	0.00	0.00	0.00	0.00	0.00	0.00	0.00
chromosome unknown open reading frame, human C10orf2	LOC100756896	100756896	39.27	39.06	48.56	44.86	38.26	39.74	3303.24	3300.53	4183.35	3922.94	3337.83	3493.80
small ubiquitin-related modifier 2 pseudogene	LOC100756901	100756901	3.70	5.53	3.65	2.40	3.64	2.46	30.38	46.67	32.29	20.86	24.84	17.76
CD209 antigen-like protein C	LOC100756903	100756903	0.00	0.00	0.00	0.00	0.00	0.00	0.00	0.00	0.00	0.00	0.00	0.00
olfactory receptor 14A2-like	LOC100756904	100756904	0.00	0.00	0.00	0.00	0.00	0.00	0.00	0.00	0.00	0.00	0.00	0.00
up-regulated during skeletal muscle growth protein 5	LOC100756907	100756907	2.72	1.86	1.77	0.61	2.00	1.43	26.58	34.77	24.84	25.50	31.05	31.78
guanylate-binding protein 4-like	LOC100756912	100756912	0.00	0.00	0.00	0.00	0.00	0.00	0.00	0.00	0.00	0.00	0.00	0.00
chromosome unknown open reading frame, human C19orf38	LOC100756916	100756916	0.00	0.00	0.00	0.00	0.00	0.07	1.52	0.92	1.24	1.16	0.00	2.80
resistin-like beta	LOC100756917	100756917	0.00	0.00	0.00	0.00	0.00	0.00	0.00	0.00	0.00	0.00	0.00	0.00
UBA-like domain-containing protein 2	LOC100756918	100756918	3.94	3.64	2.64	2.81	2.23	0.64	37.22	40.26	23.60	24.34	20.70	5.61
chromosome unknown open reading frame, human C15orf61	LOC100756919	100756919	1.83	1.76	2.74	1.65	1.58	1.98	77.47	74.12	114.27	71.85	69.34	78.51
putative MAGE domain-containing protein MAGEA13P	LOC100756923	100756923	0.00	0.00	0.00	0.06	0.00	0.00	0.00	0.00	0.00	1.16	0.00	0.00
leucine-rich repeat-containing protein homolog	LOC100756927	100756927	0.00	0.00	0.00	0.00	0.00	0.00	0.76	0.00	1.24	2.32	1.03	0.00
disintegrin and metalloproteinase domain-containing protein 18	LOC100756928	100756928	0.00	0.00	0.00	0.00	0.00	0.00	0.00	0.00	0.00	0.00	0.00	0.00
metallothionein-4	LOC100756932	100756932	0.00	0.00	0.00	0.00	0.00	0.00	0.00	0.00	0.00	0.00	0.00	0.00
40S ribosomal protein S6-like	LOC100756935	100756935	0.00	0.00	0.00	0.00	0.00	0.00	0.00	0.92	7.45	0.00	3.10	1.87
olfactory receptor 4A5-like	LOC100756937	100756937	0.00	0.00	0.00	0.00	0.00	0.00	0.00	0.00	0.00	0.00	0.00	0.00
voltage-dependent anion-selective channel protein 1 pseudogene	LOC100756939	100756939	0.08	0.00	0.00	0.00	0.00	0.00	3.04	0.00	0.00	0.00	0.00	0.93

40S ribosomal protein SA pseudogene	LOC100756940	100756940	0.18	0.62	0.06	0.28	0.20	0.09	4.56	12.81	2.48	5.79	4.14	1.87
Uncharacterized protein C3orf18	LOC100756942	100756942	11.14	11.67	11.60	7.17	5.66	6.52	429.90	427.32	426.04	250.33	210.10	231.80
neuferricin	LOC100756943	100756943	1.12	1.08	1.06	1.13	1.51	1.26	56.21	46.67	50.93	52.15	66.24	56.08
60S ribosomal protein L5	LOC100756946	100756946	232.05	224.65	227.58	226.66	222.06	215.97	2637.12	2509.02	2495.35	2315.52	2351.49	2215.17
H-2 class I histocompatibility antigen, alpha chain-like	LOC100756947	100756947	0.00	0.00	0.01	0.00	0.00	0.00	0.00	0.00	0.00	0.00	0.00	0.00
olfactory receptor 4E1	LOC100756949	100756949	0.00	0.00	0.00	0.00	0.00	0.00	0.00	0.00	0.00	0.00	0.00	0.00
beta-hexosaminidase subunit beta-like	LOC100756951	100756951	28.35	28.17	36.56	37.17	43.55	43.37	1446.92	1443.01	1868.10	1854.27	2236.61	2222.64
zinc finger protein 11	LOC100756955	100756955	1.44	1.38	1.95	2.12	2.16	1.92	126.84	124.44	173.89	188.90	188.37	172.91
malate dehydrogenase, cytoplasmic pseudogene	LOC100756956	100756956	0.00	0.00	0.00	0.00	0.00	0.00	0.00	0.00	0.00	0.00	0.00	0.00
double C2-like domain-containing protein gamma	LOC100756957	100756957	0.09	0.08	0.07	0.00	0.03	0.08	3.80	4.58	6.21	4.64	3.10	4.67
vomeronasal type-1 receptor 90-like	LOC100756960	100756960	0.00	0.00	0.00	0.00	0.00	0.00	0.00	0.00	0.00	0.00	0.00	0.00
high mobility group protein B1 pseudogene	LOC100756961	100756961	0.44	0.20	0.12	0.05	0.09	0.14	9.11	7.32	2.48	5.79	5.17	8.41
chromosome unknown open reading frame, human C14orf80	LOC100756967	100756967	5.76	5.10	7.17	5.28	4.29	4.74	344.83	316.60	416.10	332.61	274.27	307.51
uncharacterized LOC100756969	LOC100756969	100756969	0.00	0.00	0.00	0.00	0.00	0.00	0.00	0.00	0.00	0.00	0.00	0.00
60S ribosomal protein L36-like	LOC100756971	100756971	0.87	1.49	1.22	0.60	0.51	0.36	117.73	129.02	78.25	23.18	16.56	11.22
ran guanine nucleotide release factor pseudogene	LOC100756972	100756972	0.10	0.30	0.48	0.31	0.27	0.08	2.28	6.41	3.73	3.48	2.07	3.74
ubiquitin-like	LOC100756974	100756974	0.31	0.47	0.00	0.00	0.00	0.00	2.28	1.83	0.00	0.00	1.03	0.00
60S ribosomal protein L10-like	LOC100756977	100756977	0.00	0.00	0.00	0.00	0.00	0.00	0.00	0.00	0.00	0.00	0.00	0.00

nucleophosmin pseudogene	LOC100 756978	100756978	33.51	31.91	30.43	22.71	26.35	25.57	909.17	879.35	845.86	620.02	719.32	685.11
methyl-CpG-binding domain protein 5-like	LOC100 756979	100756979	0.02	0.01	0.00	0.02	0.02	0.03	0.00	0.92	0.00	0.00	1.03	0.93
60S ribosomal protein L6 pseudogene	LOC100 756982	100756982	0.74	0.10	0.00	0.00	0.23	0.10	6.84	0.92	0.00	0.00	2.07	0.93
filaggrin-2-like	LOC100 756984	100756984	31.77	31.92	23.01	26.10	27.65	26.16	2789.03	2843.92	2022.12	2343.34	2449.81	2310.50
poly(rC)-binding protein 2 pseudogene	LOC100 756985	100756985	0.53	0.70	0.32	0.22	0.26	0.19	15.95	14.64	4.97	5.79	5.17	1.87
NACHT, LRR and PYD domains-containing protein 1-like	LOC100 756987	100756987	0.00	0.01	0.00	0.00	0.01	0.00	0.00	0.92	1.24	0.00	1.03	0.00
selection and upkeep of intraepithelial T-cells protein 1-like	LOC100 756990	100756990	0.00	0.00	0.00	0.00	0.00	0.00	0.00	0.00	0.00	0.00	0.00	0.00
olfactory receptor 490	LOC100 756996	100756996	0.00	0.00	0.00	0.00	0.00	0.00	0.00	0.00	0.00	0.00	0.00	0.00
speckle-type POZ protein-like	LOC100 757001	100757001	0.00	0.00	0.00	0.00	0.00	0.00	0.00	0.00	0.00	0.00	0.00	0.00
potassium voltage-gated channel subfamily D member 2 pseudogene	LOC100 757003	100757003	0.00	0.00	0.00	0.00	0.00	0.00	0.00	0.00	0.00	0.00	0.00	0.00
olfactory receptor 477	LOC100 757005	100757005	0.00	0.00	0.00	0.00	0.00	0.00	0.00	0.00	0.00	0.00	0.00	0.00
keratin-associated protein 10-1-like	LOC100 757010	100757010	0.00	0.00	0.00	0.00	0.00	0.00	0.00	0.00	0.00	0.00	0.00	0.00
cytochrome P450 2C18-like	LOC100 757012	100757012	0.00	0.00	0.00	0.00	0.00	0.00	0.00	0.00	0.00	0.00	0.00	0.93
chromosome unknown open reading frame, human C12orf56	LOC100 757013	100757013	0.02	0.02	0.00	0.00	0.02	0.00	0.76	0.92	0.00	0.00	1.03	0.00
protein transport protein Sec61 subunit gamma-like	LOC100 757015	100757015	0.00	0.00	0.00	0.00	0.00	0.00	0.76	0.00	0.00	0.00	1.03	0.93
neutrophil antibiotic peptide NP-1-like	LOC100 757016	100757016	0.00	0.00	0.00	0.00	0.00	0.00	0.00	0.00	0.00	0.00	0.00	0.00
cytochrome c oxidase subunit 4 isoform 2, mitochondrial	LOC100 757022	100757022	0.08	0.00	0.00	0.00	0.00	0.10	0.76	0.00	0.00	0.00	0.00	1.87
probable G-protein coupled receptor 160	LOC100 757034	100757034	0.00	0.00	0.00	0.00	0.00	0.00	0.00	0.00	0.00	0.00	0.00	0.00

60S ribosomal protein L5	LOC100757036	100757036	606.97	596.45	603.28	665.13	616.21	619.90	7890.09	7802.49	7848.75	8971.20	7833.81	8118.54
olfactory receptor 8H1	LOC100757037	100757037	0.00	0.00	0.00	0.00	0.00	0.00	0.00	0.00	0.00	0.00	0.00	0.00
transforming protein RhoA	LOC100757042	100757042	0.00	0.00	0.00	0.00	0.00	0.00	0.00	0.00	0.00	0.00	0.00	0.00
uncharacterized LOC100757044	LOC100757044	100757044	112.57	117.32	114.70	112.25	144.60	133.24	781.57	904.97	835.92	786.91	986.34	870.18
protocadherin gamma-A4	LOC100757045	100757045	0.00	0.01	0.00	0.00	0.00	0.00	0.00	0.92	0.00	0.00	0.00	0.00
nogo-B receptor pseudogene	LOC100757052	100757052	0.20	0.20	0.22	0.05	0.09	0.04	4.56	4.58	4.97	1.16	2.07	0.93
lethal(3)malignant brain tumor-like protein 3	LOC100757058	100757058	4.71	4.87	3.65	3.84	3.78	3.62	448.13	463.01	380.08	358.11	362.25	338.35
ADP-ribosylation factor-like protein 1	LOC100757060	100757060	26.10	28.34	29.79	29.05	32.34	28.81	428.38	451.11	558.94	610.75	625.13	590.71
arylacetamide deacetylase-like 4	LOC100757061	100757061	0.00	0.00	0.00	0.00	0.00	0.00	0.00	0.00	0.00	0.00	0.00	0.00
olfactory receptor 4F6	LOC100757062	100757062	0.00	0.00	0.03	0.00	0.00	0.00	0.00	0.00	0.00	0.00	0.00	0.00
L-lactate dehydrogenase A chain pseudogene	LOC100757063	100757063	0.00	0.00	0.00	0.00	0.00	0.00	0.00	0.00	0.00	0.00	0.00	0.00
high mobility group nucleosome-binding domain-containing protein 5 pseudogene	LOC100757064	100757064	0.84	1.09	0.96	0.71	0.63	0.32	10.63	13.73	11.18	8.11	8.28	3.74
ankyrin repeat domain-containing protein 7-like	LOC100757068	100757068	0.00	0.00	0.00	0.00	0.06	0.00	0.00	0.00	1.24	0.00	4.14	0.93
chromosome unknown open reading frame, human C10orf128	LOC100757072	100757072	0.00	0.00	0.00	0.00	0.00	0.00	0.00	0.00	0.00	0.00	0.00	0.00
protein CTLA-2-beta	LOC100757077	100757077	29.38	26.67	13.07	15.22	18.42	20.90	396.48	387.06	176.38	197.02	243.22	283.20
multifunctional protein ADE2 pseudogene	LOC100757079	100757079	0.00	0.00	0.00	0.00	0.00	0.00	0.00	0.00	0.00	1.16	0.00	0.00
PRAME family member 12	LOC100757084	100757084	0.00	0.00	0.00	0.00	0.00	0.00	0.00	0.00	0.00	0.00	0.00	0.00
olfactory receptor 56A4-like	LOC100757086	100757086	0.00	0.00	0.00	0.00	0.00	0.00	0.00	0.00	0.00	0.00	0.00	0.00

guanylate-binding protein 4-like	LOC100757089	100757089	0.00	0.00	0.00	0.00	0.00	0.00	0.00	0.00	0.00	0.00	0.00	0.00
zinc finger protein 2-like	LOC100757091	100757091	5.57	6.13	6.00	5.93	7.17	6.90	606.87	652.42	628.50	635.09	744.16	686.98
hepatitis A virus cellular receptor 1 homolog	LOC100757092	100757092	0.00	0.00	0.00	0.00	0.00	0.00	0.00	0.00	0.00	0.00	0.00	0.00
40S ribosomal protein S3 pseudogene	LOC100757094	100757094	0.00	0.00	0.00	0.00	0.07	0.00	0.00	0.00	0.00	0.00	1.03	0.00
olfactory receptor 478	LOC100757095	100757095	0.00	0.00	0.00	0.00	0.00	0.00	0.00	0.00	0.00	0.00	0.00	0.00
vomeronasal type-1 receptor 4-like	LOC100757097	100757097	0.00	0.00	0.00	0.00	0.00	0.00	0.00	0.00	0.00	0.00	0.00	0.00
cytochrome c, somatic	LOC100757098	100757098	233.62	232.31	248.86	245.08	260.03	264.65	5248.42	5254.12	5645.28	5445.76	5689.32	5887.48
ubiquitin-conjugating enzyme E2 variant 1 pseudogene	LOC100757099	100757099	0.85	1.12	0.74	0.96	0.96	0.20	6.08	8.24	6.21	8.11	6.21	2.80
cytochrome c oxidase subunit 6A2, mitochondrial	LOC100757101	100757101	0.18	0.32	0.00	0.00	0.00	0.00	2.28	4.58	1.24	1.16	0.00	0.00
chromosome unknown open reading frame, human C19orf60	LOC100757102	100757102	17.61	16.54	17.54	10.91	7.64	7.85	328.88	325.75	317.97	198.18	157.32	147.68
regulator of chromosome condensation	LOC100757107	100757107	0.13	0.08	0.23	0.04	0.06	0.13	3.80	2.75	7.45	1.16	3.10	2.80
ATPase inhibitor, mitochondrial pseudogene	LOC100757109	100757109	0.00	0.00	0.00	0.00	0.00	0.24	0.00	0.00	0.00	0.00	0.00	0.93
protein tyrosine phosphatase type IVA 2 pseudogene	LOC100757114	100757114	0.00	0.00	0.00	0.00	0.00	0.00	0.00	0.00	0.00	0.00	0.00	0.00
myosin light polypeptide 6-like	LOC100757119	100757119	0.10	0.00	0.00	0.00	0.00	0.00	0.76	0.00	0.00	0.00	0.00	0.00
40S ribosomal protein S15a pseudogene	LOC100757123	100757123	0.13	0.00	0.00	0.00	0.00	0.17	0.76	0.00	0.00	0.00	0.00	3.74
60S ribosomal protein L37a	LOC100757126	100757126	0.00	0.31	0.00	0.44	0.00	0.00	0.00	0.92	0.00	1.16	0.00	0.00
glyceraldehyde-3-phosphate dehydrogenase pseudogene	LOC100757127	100757127	1.15	1.68	1.16	0.88	0.73	0.52	31.14	42.09	29.81	24.34	17.59	14.95
sperm motility kinase X-like	LOC100757128	100757128	0.00	0.00	0.00	0.00	0.00	0.00	0.00	0.00	0.00	0.00	0.00	0.00

ATP synthase F(0) complex subunit B1, mitochondrial pseudogene	LOC100757129	100757129	0.08	0.18	0.00	0.18	0.00	0.09	1.52	5.49	0.00	3.48	0.00	1.87
olfactory receptor 5AK2-like	LOC100757131	100757131	0.00	0.00	0.00	0.00	0.00	0.00	0.00	0.00	0.00	0.00	0.00	0.00
nucleoporin SEH1	LOC100757132	100757132	20.79	21.22	24.07	25.45	25.16	23.71	1436.29	1475.95	1659.43	1740.70	1724.29	1637.54
late cornified envelope protein 3D-like	LOC100757137	100757137	0.00	0.00	0.00	0.00	0.00	0.00	0.00	0.00	0.00	0.00	0.00	0.00
ubiquitin-60S ribosomal protein L40 pseudogene	LOC100757139	100757139	0.77	0.72	0.80	0.00	0.34	0.62	4.56	3.66	4.97	0.00	2.07	3.74
S-adenosylmethionine decarboxylase proenzyme-like	LOC100757144	100757144	3.44	3.21	3.65	3.43	3.54	3.42	82.03	86.01	126.69	111.26	113.85	134.59
uncharacterized LOC100757145	LOC100757145	100757145	3.10	2.85	2.28	2.25	2.09	1.65	88.11	81.44	68.31	64.90	61.06	55.15
40S ribosomal protein S2 pseudogene	LOC100757146	100757146	119.87	126.08	120.26	93.05	107.34	93.79	408.63	443.79	401.19	290.89	367.42	303.77
chromosome unknown open reading frame, human C1orf86	LOC100757150	100757150	24.82	23.06	21.97	21.50	19.30	22.34	993.48	929.67	893.06	857.60	775.20	886.07
suppressor of cytokine signaling 5-like	LOC100757153	100757153	0.07	0.00	0.24	0.17	0.08	0.12	3.04	0.00	9.94	8.11	4.14	6.54
tyrosine-protein kinase RYK-like	LOC100757154	100757154	1.47	1.08	0.83	0.94	1.01	0.60	44.81	27.45	22.36	22.02	21.73	10.28
40S ribosomal protein S9 pseudogene	LOC100757156	100757156	0.00	0.00	0.00	0.00	0.00	0.00	0.00	0.00	0.00	0.00	0.00	0.00
apoptosis-associated speck-like protein containing a CARD	LOC100757158	100757158	26.79	27.26	34.92	26.15	24.61	25.18	471.67	489.54	604.90	457.77	430.55	443.03
oocyte-secreted protein 1 pseudogene	LOC100757159	100757159	0.00	0.00	0.00	0.00	0.00	0.00	0.00	0.92	0.00	3.48	0.00	0.00
ephrin type-A receptor 6-like	LOC100757161	100757161	0.00	0.00	0.00	0.00	0.00	0.00	0.76	0.00	0.00	0.00	0.00	0.00
olfactory receptor 5B3-like	LOC100757164	100757164	0.00	0.00	0.00	0.00	0.04	0.00	0.00	0.00	0.00	0.00	1.03	0.00
eukaryotic translation initiation factor 4 gamma 1 pseudogene	LOC100757166	100757166	2.10	1.74	1.62	1.12	1.39	1.28	96.46	89.67	81.98	50.99	68.31	57.95
zinc finger protein 420-like	LOC100757167	100757167	0.01	0.02	0.00	0.00	0.00	0.00	0.00	0.00	0.00	0.00	0.00	0.00

NUT family member Gm806-like	LOC100757170	100757170	0.00	0.00	0.00	0.00	0.00	0.00	0.00	0.00	0.00	0.00	0.00	0.00
melanoma-associated antigen B4-like	LOC100757173	100757173	0.00	0.00	0.00	0.00	0.00	0.00	0.00	0.00	0.00	0.00	0.00	0.00
keratin-associated protein 3-1	LOC100757178	100757178	0.00	0.00	0.00	0.00	0.00	0.00	0.00	0.00	0.00	0.00	0.00	0.00
uncharacterized LOC100757181	LOC100757181	100757181	0.12	0.19	0.00	0.09	0.00	0.22	4.56	8.24	2.48	1.16	6.21	11.22
zinc finger protein 883-like	LOC100757182	100757182	0.00	0.00	0.00	0.00	0.00	0.03	0.00	0.00	0.00	0.00	0.00	1.87
serine protease inhibitor Kazal-type 12-like	LOC100757183	100757183	0.00	0.00	0.00	0.00	0.00	0.00	0.00	0.00	0.00	0.00	0.00	0.00
60S ribosomal protein L23a pseudogene	LOC100757189	100757189	2.66	2.91	1.62	1.16	2.26	0.79	22.03	25.62	12.42	9.27	17.59	7.48
CD209 antigen-like protein E	LOC100757193	100757193	0.00	0.00	0.00	0.00	0.00	0.00	0.00	0.00	0.00	0.00	0.00	0.00
olfactory receptor 14A2-like	LOC100757194	100757194	0.00	0.00	0.00	0.00	0.00	0.00	0.00	0.00	0.00	0.00	0.00	0.00
60S ribosomal protein L5 pseudogene	LOC100757197	100757197	0.00	0.00	0.00	0.00	0.00	0.00	0.00	0.00	0.00	0.00	0.00	0.00
histone H4	LOC100757199	100757199	0.00	0.00	0.00	0.30	0.00	0.00	0.00	0.00	0.00	0.00	0.00	0.00
GRB2-associated-binding protein 2 pseudogene	LOC100757201	100757201	0.03	0.09	0.00	0.09	0.08	0.17	0.00	0.92	0.00	2.32	2.07	3.74
G/T mismatch-specific thymine DNA glycosylase-like	LOC100757203	100757203	0.03	0.09	0.05	0.00	0.00	0.00	0.00	0.00	0.00	0.00	0.00	0.00
guanine nucleotide-binding protein G(I)/G(S)/G(O) subunit gamma-5	LOC100757206	100757206	96.07	84.61	79.83	67.64	44.61	50.30	561.30	521.57	504.29	562.08	487.48	530.89
transforming protein RhoA pseudogene	LOC100757217	100757217	2.26	1.44	2.52	1.33	1.38	2.43	13.67	9.15	19.87	11.59	6.21	19.63
heterogeneous nuclear ribonucleoprotein A3 pseudogene	LOC100757232	100757232	0.00	0.00	0.00	0.00	0.00	0.00	0.00	0.00	0.00	0.00	0.00	0.00
HLA class I histocompatibility antigen, A-25 alpha chain-like	LOC100757234	100757234	0.04	0.00	0.01	0.00	0.00	0.00	0.00	0.00	1.24	0.00	0.00	0.00
S-methyl-5'-thioadenosine phosphorylase pseudogene	LOC100757236	100757236	0.50	0.28	0.19	0.18	0.11	0.05	9.87	5.49	3.73	3.48	2.07	0.93

olfactory receptor 10G2	LOC100757237	100757237	0.00	0.00	0.00	0.00	0.00	0.00	0.00	0.00	0.00	0.00	0.00	0.00
microtubule-associated proteins 1A/1B light chain 3B pseudogene	LOC100757238	100757238	0.00	0.00	0.00	0.00	0.00	0.00	1.52	1.83	0.00	0.00	1.03	0.93
cytochrome P450 2C29	LOC100757240	100757240	0.00	0.00	0.00	0.00	0.00	0.00	0.00	0.00	0.00	0.00	0.00	0.00
immunoglobulin-binding protein 1b-like	LOC100757243	100757243	8.60	8.45	7.97	8.61	8.54	8.53	596.24	588.37	558.94	610.75	609.61	586.04
vomeronasal type-1 receptor 90-like	LOC100757248	100757248	0.00	0.00	0.00	0.00	0.00	0.00	0.00	0.00	0.00	0.00	0.00	0.00
keratin, type I cuticular Ha3-I	LOC100757249	100757249	0.00	0.00	0.00	0.00	0.00	0.00	0.00	0.00	0.00	0.00	0.00	0.00
uncharacterized LOC100757256	LOC100757256	100757256	0.07	0.24	0.11	0.15	0.18	0.24	3.04	6.41	3.73	4.64	4.14	5.61
uncharacterized LOC100757257	LOC100757257	100757257	0.00	0.00	0.00	0.00	0.00	0.00	0.00	0.00	0.00	0.00	0.00	0.00
C-type lectin domain family 4 member K-like	LOC100757258	100757258	0.00	0.03	0.00	0.00	0.00	0.02	0.76	0.92	1.24	0.00	0.00	2.80
40S ribosomal protein S29	LOC100757259	100757259	1.20	1.05	0.91	0.76	1.05	1.78	1.52	3.66	1.24	0.00	2.07	3.74
aldose reductase-related protein 1	LOC100757262	100757262	0.59	0.71	0.36	0.59	0.61	0.42	24.31	32.03	14.91	24.34	24.84	17.76
zinc finger protein 431-like	LOC100757265	100757265	1.91	1.88	1.92	2.31	2.84	2.19	216.47	208.63	211.15	253.80	315.67	245.82
cytochrome c, somatic pseudogene	LOC100757274	100757274	1.37	0.17	0.46	0.23	0.19	0.36	8.35	0.92	3.73	1.16	1.03	3.74
killer cell lectin-like receptor subfamily B member 1F	LOC100757275	100757275	0.00	0.00	0.00	0.00	0.00	0.00	0.00	0.00	0.00	0.00	0.00	0.00
39S ribosomal protein L33, mitochondrial pseudogene	LOC100757276	100757276	1.26	0.76	0.00	0.00	0.95	0.94	15.95	16.47	6.21	3.48	7.24	4.67
vesicle-associated membrane protein-associated protein A-like	LOC100757277	100757277	13.64	17.32	15.33	13.21	12.65	10.88	224.82	285.49	243.45	207.45	204.93	172.91
selection and upkeep of intraepithelial T-cells protein 10-like	LOC100757279	100757279	0.00	0.00	0.00	0.00	0.00	0.00	0.00	0.00	0.00	1.16	0.00	0.00
olfactory receptor 494	LOC100757285	100757285	0.00	0.00	0.00	0.00	0.00	0.00	0.00	0.00	0.00	0.00	0.00	0.00

nascent polypeptide-associated complex subunit alpha pseudogene	LOC100757286	100757286	1.33	1.31	0.80	0.48	0.55	0.53	16.71	16.47	9.94	5.79	6.21	6.54
non-histone chromosomal protein HMG-17	LOC100757288	100757288	5.45	5.51	5.02	5.22	6.98	3.94	61.52	54.90	67.07	49.83	52.78	55.15
nucleoporin SEH1 pseudogene	LOC100757289	100757289	0.00	0.00	0.00	0.00	0.00	0.00	0.00	0.00	0.00	0.00	0.00	0.00
vomeronasal type-2 receptor 116-like	LOC100757290	100757290	0.00	0.00	0.00	0.00	0.00	0.00	0.00	0.00	0.00	0.00	0.00	0.00
60S ribosomal protein L15 pseudogene	LOC100757292	100757292	0.40	0.08	0.00	0.11	0.00	0.25	4.56	0.00	0.00	1.16	0.00	3.74
ribonuclease K6-like	LOC100757294	100757294	0.10	0.00	0.16	0.00	0.06	0.17	0.00	0.00	0.00	0.00	0.00	0.00
gamma-crystallin E	LOC100757295	100757295	0.00	0.00	0.00	0.00	0.00	0.00	0.00	0.00	0.00	0.00	0.00	0.00
neutrophil antibiotic peptide NP-1	LOC100757305	100757305	0.00	0.00	0.00	0.00	0.00	0.00	0.00	0.00	0.00	0.00	0.00	0.00
chromodomain Y-like protein	LOC100757319	100757319	0.40	0.36	0.38	0.12	0.22	0.20	14.43	10.07	16.15	5.79	9.31	11.22
la-related protein 4 pseudogene	LOC100757320	100757320	0.00	0.02	0.03	0.06	0.00	0.00	1.52	0.92	1.24	2.32	0.00	0.00
histone H2B type 1	LOC100757322	100757322	0.72	0.58	0.20	0.29	0.42	0.00	1.52	4.58	3.73	0.00	0.00	0.93
transmembrane protein 45A-like	LOC100757327	100757327	0.00	0.00	0.00	0.03	0.00	0.00	0.00	0.00	0.00	1.16	0.00	0.00
olfactory receptor 1094-like	LOC100757329	100757329	0.00	0.00	0.00	0.00	0.00	0.00	0.00	0.00	0.00	0.00	0.00	0.00
cationic amino acid transporter 3-like	LOC100757335	100757335	0.00	0.00	0.00	0.00	0.00	0.00	0.00	0.00	0.00	1.16	0.00	0.00
protocadherin gamma-B1-like	LOC100757337	100757337	0.04	0.03	0.04	0.12	0.00	0.00	1.52	1.83	2.48	4.64	0.00	0.00
small ubiquitin-related modifier 2-A-like	LOC100757351	100757351	0.00	0.00	0.00	0.00	0.00	0.00	0.00	0.00	0.00	0.00	0.00	0.00
olfactory receptor 4F3/4F16/4F29-like	LOC100757353	100757353	0.00	0.00	0.00	0.00	0.00	0.00	0.00	0.00	0.00	0.00	0.00	0.00
coiled-coil-helix-coiled-coil-helix domain-containing protein 2, mitochondrial pseudogene	LOC100757358	100757358	0.72	0.72	0.60	0.32	0.76	0.91	6.08	7.32	6.21	3.48	4.14	10.28

kalirin-like	LOC100757365	100757365	0.00	0.00	0.00	0.00	0.00	0.00	0.00	0.00	0.00	0.00	0.00	0.00
WD repeat-containing protein 92 pseudogene	LOC100757366	100757366	1.55	1.32	0.80	0.77	0.86	0.96	50.89	42.09	24.84	23.18	24.84	26.17
protein CTLA-2-beta-like	LOC100757369	100757369	0.00	0.00	0.00	0.00	0.00	0.00	0.00	0.00	0.00	0.00	0.00	0.00
rho GTPase-activating protein 20-like	LOC100757375	100757375	0.00	0.00	0.00	0.00	0.00	0.00	0.76	0.00	0.00	0.00	1.03	0.00
nuclear fragile X mental retardation-interacting protein 2-like	LOC100757378	100757378	16.86	17.49	13.57	15.53	15.69	15.86	143.55	129.02	91.91	63.74	42.43	32.71
interferon-induced guanylate-binding protein 2-like	LOC100757382	100757382	0.72	0.94	0.28	0.19	0.44	0.32	41.77	51.24	19.87	10.43	24.84	16.82
TAF5-like RNA polymerase II p300/CBP-associated factor-associated factor 65 kDa subunit 5L pseudogene	LOC100757385	100757385	5.65	5.06	5.29	4.85	4.52	4.64	241.53	210.46	213.64	209.76	183.19	187.87
olfactory receptor 10T2-like	LOC100757388	100757388	0.00	0.00	0.00	0.00	0.00	0.00	0.00	0.00	0.00	0.00	0.00	0.00
protein FAM127A-like	LOC100757391	100757391	17.90	14.65	18.54	9.64	6.59	10.72	34.94	42.09	27.33	19.70	9.31	10.28
inhibitor of growth protein 1 pseudogene	LOC100757394	100757394	10.00	9.91	15.14	7.27	10.50	11.11	0.00	2.75	0.00	1.16	0.00	0.00
guanine nucleotide-binding protein subunit beta-2-like 1 pseudogene	LOC100757395	100757395	0.00	0.00	0.00	0.00	0.00	0.00	0.00	0.00	0.00	0.00	0.00	0.00
olfactory receptor 6F1	LOC100757396	100757396	0.00	0.00	0.00	0.00	0.00	0.00	0.00	0.00	0.00	0.00	0.00	0.00
chromosome unknown open reading frame, human C12orf60	LOC100757398	100757398	0.00	0.00	0.00	0.15	0.00	0.00	0.00	0.92	0.00	2.32	0.00	0.00
myosin light polypeptide 6-like	LOC100757407	100757407	0.00	0.00	0.00	0.00	0.07	0.00	3.80	3.66	4.97	3.48	7.24	6.54
glutathione S-transferase omega-1 pseudogene	LOC100757416	100757416	0.00	0.00	0.00	0.00	0.00	0.00	0.00	0.00	0.00	0.00	0.00	0.00
securin pseudogene	LOC100757418	100757418	0.48	0.52	0.29	0.45	0.53	0.59	16.71	18.30	11.18	16.22	19.66	21.50
F-box/WD repeat-containing protein 12-like	LOC100757422	100757422	0.00	0.00	0.00	0.00	0.00	0.00	0.00	0.00	0.00	0.00	0.00	0.00
putative olfactory receptor 4A8	LOC100757423	100757423	0.00	0.00	0.00	0.00	0.00	0.00	0.00	0.00	0.00	0.00	0.00	0.00

cofilin-1 pseudogene	LOC100 757424	100757424	0.00	0.00	0.00	0.00	0.00	0.00	0.00	0.00	0.00	0.00	0.00	0.00
gastric triacylglycerol lipase-like	LOC100 757425	100757425	0.00	0.06	0.00	0.00	0.00	0.00	0.00	0.92	1.24	0.00	0.00	0.00
olfactory receptor 1013-like	LOC100 757426	100757426	0.29	0.33	0.21	0.17	0.11	0.10	1.52	2.75	2.48	2.32	1.03	1.87
late cornified envelope protein 3D-like	LOC100 757432	100757432	0.00	0.00	0.00	0.00	0.00	0.00	0.00	0.00	0.00	0.00	0.00	0.00
zinc finger protein 664-like	LOC100 757435	100757435	5.11	5.10	6.24	7.10	8.07	7.38	531.68	527.06	658.31	738.23	817.64	768.30
Growth-arrest-specific protein 2 domain containing protein	LOC100 757436	100757436	0.00	0.00	0.00	0.00	0.00	0.00	0.76	0.00	0.00	0.00	1.03	0.00
suppressor of cytokine signaling 5-like	LOC100 757448	100757448	0.29	0.46	0.43	0.18	0.40	0.38	10.63	16.47	13.66	5.79	15.52	17.76
tomoregulin-2-like	LOC100 757450	100757450	4.76	4.71	6.20	3.33	3.50	2.67	102.54	102.48	129.18	71.85	80.73	56.08
olfactory receptor 5B3-like	LOC100 757457	100757457	0.00	0.00	0.00	0.00	0.00	0.00	0.00	0.00	0.00	0.00	0.00	0.00
40S ribosomal protein S10 pseudogene	LOC100 757459	100757459	0.00	0.00	0.00	0.00	0.00	0.00	0.00	0.00	0.00	0.00	0.00	0.00
zinc finger protein 420-like	LOC100 757460	100757460	1.07	1.45	1.46	1.51	1.60	1.52	75.95	99.74	104.34	104.30	103.50	101.88
proteasome subunit beta type-6 pseudogene	LOC100 757462	100757462	0.00	0.00	0.00	0.00	0.00	0.00	0.00	0.00	0.00	0.00	0.00	0.00
uncharacterized LOC100757465	LOC100 757465	100757465	0.97	0.99	0.43	0.57	0.56	0.54	6.08	0.92	0.00	0.00	2.07	1.87
UDP-glucuronosyltransferase 1-8-like	LOC100 757466	100757466	0.03	0.08	0.00	0.00	0.05	0.00	0.00	0.92	0.00	0.00	0.00	0.00
melanoma-associated antigen B3	LOC100 757467	100757467	0.00	0.00	0.00	0.00	0.00	0.00	0.00	0.00	0.00	0.00	0.00	0.00
Krueppel-like factor 10 pseudogene	LOC100 757470	100757470	0.02	0.00	0.00	0.00	0.03	0.05	0.76	0.00	0.00	0.00	1.03	1.87
keratin-associated protein 3-2	LOC100 757471	100757471	0.00	0.00	0.00	0.00	0.00	0.00	0.00	0.00	0.00	0.00	0.00	0.00
60S ribosomal protein L5 pseudogene	LOC100 757473	100757473	0.00	0.00	0.00	0.00	0.00	0.00	0.76	0.00	0.00	0.00	0.00	0.93

zinc finger protein 883-like	LOC100757474	100757474	0.00	0.00	0.00	0.00	0.00	0.00	0.00	0.00	2.48	0.00	1.03	0.00
poly(A) polymerase alpha pseudogene	LOC100757475	100757475	0.59	0.87	0.42	0.48	0.53	0.31	18.23	23.79	16.15	11.59	16.56	14.95
X-linked lymphocyte-regulated protein 3A-like	LOC100757478	100757478	0.05	0.00	0.00	0.00	0.00	0.00	2.28	0.00	1.24	0.00	1.03	0.00
protein-arginine deiminase type-4	LOC100757481	100757481	0.12	0.05	0.00	0.00	0.00	0.00	2.28	0.92	1.24	0.00	0.00	0.00
40S ribosomal protein S23 pseudogene	LOC100757482	100757482	0.09	0.00	0.16	0.00	0.00	0.00	0.76	0.00	1.24	0.00	1.03	0.00
leukocyte immunoglobulin-like receptor subfamily B member 3	LOC100757485	100757485	0.00	0.00	0.00	0.00	0.00	0.00	0.00	0.00	0.00	0.00	0.00	0.00
cyclin-D-binding Myb-like transcription factor 1 pseudogene	LOC100757486	100757486	0.00	0.00	0.00	0.00	0.00	0.00	0.00	0.00	0.00	0.00	0.00	0.00
glutamyl aminopeptidase-like	LOC100757489	100757489	0.58	0.92	0.37	0.49	0.90	0.67	18.99	32.03	11.18	17.38	28.98	21.50
ferritin heavy polypeptide-like 17	LOC100757490	100757490	0.00	0.00	0.00	0.00	0.00	0.00	0.00	0.00	0.00	0.00	0.00	0.00
uncharacterized LOC100757491	LOC100757491	100757491	0.19	0.22	0.17	0.05	0.02	0.02	28.86	38.43	17.39	12.75	4.14	6.54
uncharacterized LOC100757493	LOC100757493	100757493	23.13	21.08	16.19	14.74	14.14	14.85	34.18	29.28	17.39	18.54	17.59	6.54
60S ribosomal protein L18a pseudogene	LOC100757496	100757496	0.00	0.00	0.00	0.00	0.00	0.00	0.00	0.00	0.00	0.00	0.00	0.00
zinc finger CCHC domain-containing protein 9 pseudogene	LOC100757498	100757498	0.16	0.17	0.05	0.05	0.04	0.04	4.56	3.66	1.24	0.00	1.03	0.93
E3 SUMO-protein ligase NSE2-like	LOC100757511	100757511	6.15	4.89	4.19	4.58	4.61	5.56	160.26	149.15	126.69	114.73	106.60	129.92
chromosome unknown open reading frame, human C3orf70	LOC100757514	100757514	1.17	0.96	1.24	1.15	1.25	1.01	97.22	82.35	103.09	98.51	109.71	88.79
28S ribosomal protein S21, mitochondrial pseudogene	LOC100757516	100757516	0.00	0.00	0.00	0.00	0.00	0.00	0.00	0.00	1.24	0.00	0.00	0.00
mannose-binding protein A	LOC100757517	100757517	0.00	0.00	0.00	0.00	0.00	0.00	0.00	0.00	0.00	0.00	0.00	0.00
60S ribosomal protein L19-like	LOC100757518	100757518	0.05	0.00	0.00	0.08	0.00	0.00	0.76	0.00	0.00	1.16	0.00	0.00

popy Class I histocompatibility antigen, A-1 alpha chain-like	LOC100 757524	100757524	0.00	0.00	0.00	0.00	0.00	0.06	0.00	0.00	0.00	0.00	0.00	0.00
putative P2Y purinoceptor 10	LOC100 757526	100757526	0.44	0.50	1.02	1.05	1.38	1.08	34.18	38.43	79.49	81.12	103.50	80.38
pirin pseudogene	LOC100 757528	100757528	0.00	0.00	0.00	0.00	0.00	0.00	0.00	0.00	0.00	0.00	0.00	0.00
acyl-CoA-binding protein pseudogene	LOC100 757529	100757529	0.00	0.00	0.00	0.00	0.00	0.00	0.00	0.00	0.00	0.00	0.00	0.00
60S ribosomal protein L5 pseudogene	LOC100 757533	100757533	0.00	0.00	0.00	0.00	0.00	0.00	0.00	0.00	0.00	0.00	0.00	0.00
glutathione S-transferase P 2	LOC100 757534	100757534	1.44	1.01	3.90	2.69	2.39	2.67	33.42	21.96	94.40	61.42	48.64	57.95
	LOC100 757535	100757535	39.19	39.03	33.30	28.53	23.23	25.83	2094.05	2094.51	1767.49	1510.07	1191.27	1263.67
keratin, type I cuticular Ha3-II	LOC100 757537	100757537	0.00	0.00	0.00	0.00	0.00	0.00	0.00	0.00	0.00	0.00	0.00	0.00
histone H2AX	LOC100 757539	100757539	76.85	75.51	82.57	73.26	72.92	75.15	2714.59	2657.26	2926.36	2567.01	2567.80	2656.33
natural cytotoxicity triggering receptor 2-like	LOC100 757541	100757541	0.00	0.00	0.00	0.00	0.00	0.00	0.00	0.00	0.00	0.00	0.00	0.00
olfactory receptor 2T2-like	LOC100 757545	100757545	0.00	0.00	0.00	0.00	0.00	0.00	0.00	0.00	0.00	0.00	0.00	0.00
acid trehalase-like protein 1	LOC100 757546	100757546	13.11	11.76	12.71	13.08	10.74	11.17	734.47	674.38	724.14	704.62	628.24	686.98
thymosin beta-10 pseudogene	LOC100 757549	100757549	69.41	70.05	55.44	42.79	50.71	48.71	394.96	412.68	329.15	231.78	274.27	261.71
Ig heavy chain V region 6.96-like	LOC100 757551	100757551	0.00	0.00	0.00	0.00	0.00	0.00						
heterogeneous nuclear ribonucleoprotein A1-like	LOC100 757559	100757559	0.90	1.50	1.09	0.61	0.70	0.64	20.51	42.09	28.57	18.54	17.59	17.76
serpin B9-like	LOC100 757562	100757562	1.20	1.38	0.33	0.57	0.75	0.92	68.36	82.35	18.63	28.97	44.50	54.21
U6 snRNA-associated Sm-like protein LSm5 pseudogene	LOC100 757563	100757563	0.00	0.00	0.00	0.00	0.00	0.00	0.00	0.00	0.00	0.00	0.00	0.00
plasminogen activator inhibitor 1 RNA-binding protein pseudogene	LOC100 757569	100757569	0.00	0.00	0.00	0.00	0.00	0.00	0.00	0.00	0.00	0.00	0.00	0.00

olfactory receptor 507	LOC100757571	100757571	0.00	0.00	0.00	0.00	0.00	0.00	0.00	0.00	0.00	0.00	0.00	0.00
60S acidic ribosomal protein P2-like	LOC100757574	100757574	0.00	0.00	0.00	0.00	0.00	0.00	0.00	0.00	0.00	0.00	0.00	0.00
leukocyte immunoglobulin-like receptor subfamily A member 6	LOC100757575	100757575	0.03	0.02	0.00	0.00	0.00	0.00	1.52	0.92	0.00	0.00	0.00	0.00
zinc finger protein 431-like	LOC100757578	100757578	1.75	1.44	2.07	1.94	3.02	1.94	44.81	40.26	62.10	53.31	77.62	61.69
60S ribosomal protein L10a-like	LOC100757581	100757581	2.11	2.77	1.15	0.71	0.12	0.63	0.00	1.83	1.24	3.48	1.03	0.93
transcription elongation factor B polypeptide 1 pseudogene	LOC100757582	100757582	0.49	0.59	0.28	0.00	0.70	0.00	3.04	2.75	1.24	0.00	4.14	0.00
GRB2-associated-binding protein 2-like	LOC100757583	100757583	0.00	0.00	0.00	0.00	0.00	0.00	0.00	0.00	0.00	0.00	0.00	0.00
40S ribosomal protein S26	LOC100757586	100757586	9.02	8.13	6.99	3.65	3.61	4.65	61.52	54.90	43.47	17.38	20.70	28.97
chromosome unknown open reading frame, human C12orf66	LOC100757589	100757589	5.37	5.05	4.73	4.93	5.43	5.20	739.79	701.83	644.64	682.60	734.84	713.15
heterogeneous nuclear ribonucleoprotein F-like	LOC100757591	100757591	68.54	61.23	58.15	64.15	56.38	69.27	2542.18	2258.30	2157.50	2357.24	2056.52	2576.88
chromosome unknown open reading frame, human C19orf10	LOC100757594	100757594	75.31	74.41	82.64	64.48	57.10	62.79	2072.02	2065.23	2253.15	1829.93	1652.87	1753.44
malignant T-cell-amplified sequence 2	LOC100757597	100757597	10.39	11.90	9.32	8.42	10.05	10.05	217.99	250.72	196.25	176.16	207.00	213.10
chromosome unknown open reading frame, human C19orf40	LOC100757600	100757600	4.86	4.28	5.90	4.94	3.89	4.81	189.88	160.13	227.30	190.06	155.25	188.80
eukaryotic translation initiation factor 1 pseudogene	LOC100757603	100757603	11.40	13.31	5.64	6.64	7.53	7.06	520.28	495.03	392.50	418.37	387.08	414.06
la-related protein 4 pseudogene	LOC100757604	100757604	0.02	0.02	0.03	0.04	0.00	0.00	1.52	4.58	4.97	3.48	0.00	1.87
transcription factor BTF3 pseudogene	LOC100757605	100757605	2.48	2.50	1.60	1.02	1.76	0.93	21.27	17.39	6.21	3.48	7.24	8.41
T-cell receptor beta-2 chain C region-like	LOC100757609	100757609	0.00	0.00	0.00	0.00	0.00	0.00	0.76	0.00	0.00	0.00	0.00	0.00
ATP synthase subunit f, mitochondrial-like	LOC100757610	100757610	0.04	0.05	0.07	0.00	0.00	0.00	1.52	0.92	1.24	0.00	0.00	0.00

GDP-fucose transporter 1 pseudogene	LOC100757612	100757612	0.00	0.00	0.00	0.00	0.00	0.00	0.00	0.00	0.00	0.00	0.00	0.00
olfactory receptor 141-like	LOC100757613	100757613	0.00	0.00	0.00	0.00	0.00	0.00	0.00	0.00	0.00	0.00	0.00	0.00
H-2 class I histocompatibility antigen, Q10 alpha chain-like	LOC100757615	100757615	0.85	0.96	0.59	0.79	0.78	0.68	25.82	32.03	24.84	26.66	25.87	27.11
inversin-B-like	LOC100757618	100757618	0.00	0.00	0.00	0.00	0.00	0.00	0.00	0.00	0.00	0.00	0.00	0.00
protein PF14_0175-like	LOC100757620	100757620	0.00	0.00	0.00	0.00	0.00	0.00	0.76	0.00	0.00	0.00	0.00	0.00
RNA-binding protein 7 pseudogene	LOC100757621	100757621	0.51	0.51	0.69	0.23	0.23	0.37	7.60	6.41	11.18	3.48	3.10	6.54
protocadherin gamma-A3-like	LOC100757622	100757622	0.03	0.01	0.05	0.00	0.03	0.03	2.28	0.92	3.73	1.16	2.07	1.87
secretoglobin family 2A member 2-like	LOC100757626	100757626	0.00	0.00	0.00	0.00	0.00	0.00	0.00	0.00	0.00	0.00	0.00	0.00
DCN1-like protein 3 pseudogene	LOC100757630	100757630	0.79	0.87	0.73	0.87	0.68	0.55	10.63	18.30	18.63	20.86	10.35	11.22
60S acidic ribosomal protein P1 pseudogene	LOC100757637	100757637	0.00	0.00	0.00	0.00	0.00	0.00	0.00	0.00	0.00	0.00	0.00	0.00
putative olfactory receptor GPCRLTM7	LOC100757639	100757639	0.00	0.00	0.00	0.00	0.00	0.00	0.00	0.00	0.00	0.00	0.00	0.00
hemoglobin subunit alpha	LOC100757644	100757644	0.00	0.00	0.00	0.00	0.00	0.00	0.76	0.00	0.00	0.00	0.00	0.00
putative olfactory receptor ENSP00000348552	LOC100757645	100757645	0.00	0.01	0.00	0.00	0.00	0.00	0.00	0.00	0.00	0.00	0.00	0.00
60S ribosomal protein L26-like	LOC100757647	100757647	2.78	3.09	3.06	2.10	2.57	1.73	24.31	22.88	27.33	18.54	31.05	15.89
uncharacterized protein C3orf38 homolog pseudogene	LOC100757653	100757653	0.00	0.00	0.00	0.00	0.00	0.00	0.00	0.92	0.00	0.00	0.00	0.00
40S ribosomal protein S26	LOC100757655	100757655	0.00	0.00	0.00	0.00	0.00	0.00	0.00	0.00	0.00	0.00	0.00	0.00
protein CTLA-2-beta-like	LOC100757657	100757657	0.00	0.00	0.00	0.00	0.00	0.00	0.00	0.00	0.00	0.00	0.00	0.00
anoctamin-6	LOC100757664	100757664	38.01	40.32	48.51	55.21	59.58	58.37	1937.58	2049.68	2439.46	2711.87	2953.85	2852.61

zinc finger protein 709	LOC100757671	100757671	6.37	6.66	7.26	8.18	8.42	8.42	481.55	484.97	530.37	607.27	611.68	629.03
40S ribosomal protein S10 pseudogene	LOC100757673	100757673	0.00	0.00	0.00	0.00	0.00	0.00	0.00	0.00	0.00	0.00	0.00	0.00
nucleoporin SEH1 pseudogene	LOC100757678	100757678	0.07	0.08	0.00	0.22	0.08	0.06	0.00	0.00	0.00	3.48	0.00	0.93
heterogeneous nuclear ribonucleoproteins A2/B1 pseudogene	LOC100757680	100757680	0.00	0.00	0.00	0.00	0.00	0.00	0.00	0.00	0.00	0.00	0.00	0.00
olfactory receptor 8B3-like	LOC100757681	100757681	0.00	0.00	0.00	0.00	0.00	0.00	0.00	0.00	0.00	0.00	0.00	0.00
histone H2A.J	LOC100757684	100757684	9.13	9.06	9.14	2.48	1.35	2.22	86.59	77.78	78.25	23.18	19.66	18.69
pyrin and HIN domain-containing protein 1-like	LOC100757690	100757690	0.00	0.00	0.00	0.00	0.00	0.00	0.00	0.00	0.00	0.00	0.00	0.00
keratin-associated protein 10-8-like	LOC100757692	100757692	0.00	0.00	0.00	0.00	0.00	0.00	0.00	0.00	0.00	0.00	0.00	0.00
40S ribosomal protein S4, X isoform-like	LOC100757701	100757701	0.60	0.66	0.71	0.68	0.78	0.51	18.99	24.71	28.57	31.29	30.01	17.76
sperm motility kinase-like	LOC100757707	100757707	0.00	0.00	0.00	0.00	0.00	0.00	0.76	0.00	0.00	0.00	0.00	0.00
putative olfactory receptor 4A8	LOC100757708	100757708	0.00	0.00	0.00	0.00	0.00	0.00	0.00	0.00	0.00	0.00	0.00	0.00
60S ribosomal protein L5-like	LOC100757709	100757709	1.09	1.31	0.95	0.35	0.78	0.61	31.14	33.86	24.84	6.95	18.63	14.95
gastric triacylglycerol lipase-like	LOC100757710	100757710	0.03	0.00	0.00	0.00	0.00	0.00	0.76	0.00	0.00	0.00	0.00	0.00
olfactory receptor 1013	LOC100757711	100757711	0.00	0.00	0.00	0.00	0.00	0.00	0.76	0.00	0.00	0.00	0.00	0.00
sodium-independent sulfate anion transporter	LOC100757714	100757714	10.53	10.29	11.75	9.83	8.14	9.61	490.66	463.92	531.61	428.80	360.18	436.49
late cornified envelope protein 3D-like	LOC100757718	100757718	0.00	0.00	0.00	0.00	0.00	0.00	0.00	0.00	0.00	0.00	0.00	0.00
putative protein ZNF720	LOC100757721	100757721	0.00	0.00	0.00	0.00	0.00	0.00	0.00	0.00	0.00	0.00	0.00	0.00
cytochrome P450 2C29-like	LOC100757723	100757723	0.00	0.00	0.00	0.00	0.00	0.00	0.00	0.92	0.00	0.00	0.00	0.00

ras-related protein Rab-5B pseudogene	LOC100757726	100757726	0.04	0.00	0.00	0.00	0.06	0.05	2.28	2.75	0.00	1.16	1.03	0.93
zinc finger protein 639 pseudogene	LOC100757728	100757728	1.47	1.59	2.09	1.89	1.63	1.54	45.57	53.07	67.07	53.31	51.75	45.80
zinc finger protein 345-like	LOC100757733	100757733	3.60	3.31	4.24	5.17	5.60	4.42	382.81	350.46	453.36	543.53	579.59	465.47
olfactory receptor 2K2	LOC100757736	100757736	0.00	0.00	0.00	0.00	0.00	0.00	0.00	0.00	0.00	0.00	0.00	0.00
histone-binding protein RBBP4 pseudogene	LOC100757737	100757737	0.00	0.00	0.00	0.00	0.00	0.00	0.00	0.00	0.00	0.00	0.00	0.00
glyceraldehyde-3-phosphate dehydrogenase-like	LOC100757738	100757738	1.65	1.19	1.08	0.61	0.76	0.52	28.86	20.13	16.15	13.91	11.38	11.22
histo-blood group ABO system transferase 2	LOC100757742	100757742	0.00	0.00	0.00	0.00	0.00	0.00	0.00	0.00	0.00	0.00	0.00	0.00
60S ribosomal protein L6 pseudogene	LOC100757744	100757744	4.75	4.66	3.46	2.09	3.23	2.31	82.03	78.69	60.86	38.24	54.85	42.99
chromosome unknown open reading frame, human C16orf74	LOC100757748	100757748	0.11	0.53	0.27	0.09	0.00	0.00	3.80	10.07	3.73	1.16	2.07	0.93
NUT family member Gm806-like	LOC100757750	100757750	0.00	0.00	0.00	0.00	0.00	0.00	0.00	0.00	0.00	0.00	0.00	0.00
UDP-glucuronosyltransferase 1-8-like	LOC100757751	100757751	0.01	0.00	0.02	0.00	0.01	0.00	0.00	0.00	0.00	0.00	0.00	0.00
keratin-associated protein 4-12-like	LOC100757756	100757756	0.00	0.00	0.00	0.00	0.00	0.00	0.00	0.00	0.00	0.00	0.00	0.00
nuclear transport factor 2	LOC100757758	100757758	3.10	3.10	2.28	2.31	2.56	2.33	5.32	6.41	6.21	6.95	6.21	0.93
zinc finger protein 501-like	LOC100757759	100757759	0.66	0.59	0.63	0.50	0.74	0.65	50.89	47.58	53.41	38.24	55.89	45.80
X-linked lymphocyte-regulated protein 3A-like	LOC100757762	100757762	1.15	1.14	0.43	0.34	0.36	0.27	30.38	33.86	14.91	13.91	12.42	9.35
leukocyte immunoglobulin-like receptor subfamily B member 3	LOC100757768	100757768	0.00	0.00	0.00	0.00	0.00	0.00	0.00	0.00	0.00	0.00	0.00	0.00
vomeronasal type-2 receptor 116-like	LOC100757769	100757769	0.00	0.00	0.00	0.00	0.00	0.00	0.00	0.00	0.00	0.00	0.00	0.00
olfactory receptor 10A5-like	LOC100757770	100757770	0.00	0.00	0.00	0.00	0.00	0.00	0.00	0.00	0.00	0.00	0.00	0.00

E3 ubiquitin-protein ligase RNF216-like	LOC100757771	100757771	0.00	0.00	0.00	0.00	0.00	0.00	0.00	0.00	0.00	0.00	0.00	0.00
histone H2A type 2-A	LOC100757772	100757772	0.98	0.81	0.54	0.06	0.03	0.00	18.23	17.39	2.48	4.64	2.07	3.74
nuclear fragile X mental retardation-interacting protein 2	LOC100757773	100757773	19.55	20.59	16.12	19.51	20.01	20.34	0.00	0.00	0.00	0.00	0.00	0.00
L-lactate dehydrogenase B chain pseudogene	LOC100757774	100757774	0.00	0.00	0.00	0.00	0.00	0.00	0.00	0.00	0.00	0.00	0.00	0.00
40S ribosomal protein S26-like	LOC100757778	100757778	0.00	0.00	0.00	0.00	0.00	0.00	0.00	0.00	0.00	0.00	0.00	0.00
60S ribosomal protein L10-like	LOC100757781	100757781	22.12	18.15	18.70	12.20	13.73	16.98	226.34	192.16	192.52	126.32	135.58	171.98
chromosome unknown open reading frame, human CXorf66	LOC100757782	100757782	0.00	0.00	0.00	0.00	0.00	0.00	0.00	0.00	0.00	0.00	0.00	0.00
60S ribosomal protein L4 pseudogene	LOC100757785	100757785	2.00	1.50	1.77	1.64	1.42	1.43	62.28	42.09	50.93	41.72	41.40	42.06
PRELI domain-containing protein 1, mitochondrial pseudogene	LOC100757787	100757787	0.00	0.00	0.00	0.00	0.00	0.00	0.76	0.00	0.00	0.00	0.00	0.00
myosin regulatory light chain 2, smooth muscle major isoform pseudogene	LOC100757788	100757788	0.00	0.00	0.00	0.00	0.00	0.00	0.00	0.00	0.00	0.00	0.00	0.00
myeloperoxidase-like	LOC100757792	100757792	0.00	0.00	0.02	0.00	0.00	0.00	0.00	0.00	1.24	0.00	0.00	0.00
cTAGE family member 5-like	LOC100757793	100757793	7.06	6.92	8.87	8.96	10.09	9.90	973.73	951.63	1228.42	1231.93	1363.08	1357.14
pulmonary surfactant-associated protein A	LOC100757798	100757798	0.00	0.00	0.00	0.00	0.00	0.00	0.00	0.00	0.00	0.00	0.00	0.00
heterogeneous nuclear ribonucleoprotein M pseudogene	LOC100757799	100757799	0.08	0.10	0.02	0.06	0.08	0.08	5.32	6.41	1.24	3.48	4.14	4.67
HLA class I histocompatibility antigen, A-1 alpha chain-like	LOC100757804	100757804	0.00	0.00	0.00	0.00	0.00	0.00	0.00	0.00	0.00	0.00	0.00	0.00
putative P2Y purinoceptor 10	LOC100757805	100757805	0.09	0.08	0.23	0.10	0.07	0.14	1.52	0.92	1.24	0.00	0.00	0.93
60S ribosomal protein L27a pseudogene	LOC100757806	100757806	0.00	0.00	0.00	0.00	0.00	0.00	0.00	0.00	0.00	1.16	0.00	0.93
protein Red pseudogene	LOC100757807	100757807	0.02	0.02	0.00	0.03	0.00	0.02	0.76	1.83	0.00	1.16	0.00	0.93

elongation factor 1-alpha 1 pseudogene	LOC100757816	100757816	0.00	0.00	0.00	0.00	0.00	0.00	12.91	14.64	7.45	3.48	6.21	20.56
cytosolic beta-glucosidase	LOC100757820	100757820	0.00	0.00	0.00	0.00	0.00	0.00	0.00	0.00	0.00	0.00	0.00	0.00
vomeronasal type-2 receptor 116-like	LOC100757822	100757822	0.00	0.01	0.00	0.00	0.00	0.01	0.00	0.92	0.00	0.00	0.00	0.93
tubulin alpha-1C chain pseudogene	LOC100757824	100757824	9.92	9.21	8.11	6.09	6.02	5.66	52.41	59.48	43.47	38.24	45.54	37.39
interferon-induced transmembrane protein 3	LOC100757825	100757825	126.73	112.33	132.33	134.54	110.63	121.53	1996.83	1798.04	2101.61	2069.83	1712.90	1867.47
serine/threonine-protein kinase RIO2 pseudogene	LOC100757827	100757827	0.02	0.02	0.00	0.00	0.03	0.11	0.76	0.92	0.00	0.00	1.03	3.74
glyceraldehyde-3-phosphate dehydrogenase pseudogene	LOC100757828	100757828	103.21	102.26	80.05	59.62	67.14	61.88	610.67	605.75	438.46	326.82	392.26	363.59
zinc finger protein 120-like	LOC100757831	100757831	3.40	3.73	4.01	4.17	4.96	4.61	155.71	177.52	175.13	197.02	231.84	218.71
Ig heavy chain V-III region VH26-like	LOC100757836	100757836	0.00	0.00	0.00	0.00	0.00	0.00	0.00	0.00	0.00	0.00	0.00	0.00
speedy protein B-like	LOC100757839	100757839	0.00	0.00	0.00	0.00	0.00	0.00	0.00	0.00	0.00	0.00	0.00	0.00
selection and upkeep of intraepithelial T-cells protein 2-like	LOC100757840	100757840	0.00	0.00	0.00	0.00	0.00	0.00	0.00	0.00	0.00	0.00	0.00	0.00
DNA-directed RNA polymerases I and III subunit RPAC2	LOC100757843	100757843	171.58	176.89	195.36	191.02	192.72	190.47	2787.51	2903.40	3142.48	3043.32	3042.86	2980.66
histone H3.3-like	LOC100757845	100757845	0.22	0.00	0.00	0.06	0.00	0.00	2.28	0.00	0.00	0.00	0.00	0.00
chromosome unknown open reading frame, human C15orf27	LOC100757853	100757853	0.00	0.00	0.00	0.00	0.00	0.00	0.00	0.92	1.24	0.00	0.00	0.93
beta-defensin 130	LOC100757854	100757854	0.00	0.00	0.00	0.00	0.00	0.00	0.00	0.00	1.24	0.00	1.03	0.00
olfactory receptor 494-like	LOC100757855	100757855	0.00	0.00	0.00	0.00	0.00	0.00	0.00	0.00	0.00	0.00	0.00	0.00
ferritin light chain 1 pseudogene	LOC100757857	100757857	0.14	0.00	0.00	0.00	0.00	0.00	1.52	0.00	0.00	0.00	0.00	0.00
glutathione S-transferase Mu 1	LOC100757858	100757858	107.31	110.33	112.61	111.93	104.19	116.10	5041.06	5177.26	5286.32	5178.05	4865.47	5411.74

Ig heavy chain V region 3-like	LOC100757859	100757859	0.00	0.00	0.00	0.00	0.00	0.00						
zinc finger protein 26	LOC100757861	100757861	2.94	2.90	2.72	3.28	3.55	3.44	2.28	1.83	3.73	1.16	2.07	0.00
ubiquitin-60S ribosomal protein L40	LOC100757863	100757863	921.84	875.15	825.23	664.57	472.55	533.30	22032.71	21967.21	20744.09	17003.67	12900.07	13648.98
receptor tyrosine-protein kinase erbB-4	LOC100757865	100757865	0.04	0.05	0.03	0.09	0.13	0.02	1.52	1.83	1.24	3.48	5.17	0.93
heat shock factor-binding protein 1 pseudogene	LOC100757870	100757870	0.00	0.00	0.00	0.00	0.00	0.00	0.00	0.00	0.00	0.00	0.00	0.00
PEST proteolytic signal-containing nuclear protein pseudogene	LOC100757880	100757880	0.08	0.00	0.13	0.00	0.00	0.00	0.76	0.92	1.24	1.16	0.00	0.00
proteasome subunit beta type-5 pseudogene	LOC100757888	100757888	0.00	0.05	0.00	0.00	0.00	0.00	0.00	0.92	0.00	0.00	0.00	0.00
olfactory receptor 4P4-like	LOC100757900	100757900	0.00	0.00	0.00	0.00	0.00	0.00	0.00	0.00	0.00	0.00	0.00	0.00
vomeronasal type-2 receptor 116-like	LOC100757905	100757905	0.00	0.00	0.00	0.00	0.00	0.00	0.00	0.00	0.00	0.00	0.00	0.00
chromosome unknown open reading frame, human C19orf81	LOC100757921	100757921	0.42	0.66	0.00	0.00	0.00	0.00	15.19	19.22	1.24	0.00	1.03	0.00
olfactory receptor 4K14-like	LOC100757926	100757926	0.00	0.00	0.00	0.00	0.00	0.00	0.00	0.00	0.00	0.00	0.00	0.00
putative olfactory receptor ENSP00000348552	LOC100757930	100757930	0.00	0.01	0.00	0.00	0.00	0.00	0.00	0.00	0.00	0.00	0.00	0.00
cellular nucleic acid-binding protein pseudogene	LOC100757938	100757938	0.00	0.00	0.00	0.00	0.00	0.00	0.00	0.00	0.00	0.00	0.00	0.00
60S ribosomal protein L5-like	LOC100757942	100757942	0.02	0.05	0.04	0.03	0.03	0.08	3.04	1.83	2.48	1.16	1.03	7.48
60S ribosomal protein L6-like	LOC100757946	100757946	0.00	0.00	0.00	0.00	0.00	0.00	0.00	0.00	0.00	0.00	0.00	0.00
zinc finger protein 844-like	LOC100757955	100757955	0.65	0.52	0.41	0.54	0.81	0.57	67.60	52.16	53.41	70.69	72.45	60.75
60S ribosomal protein L15 pseudogene	LOC100757960	100757960	0.06	0.00	0.00	0.00	0.00	0.08	0.76	0.00	0.00	0.00	0.00	0.93
ubiquitin-conjugating enzyme E2 variant 1-like	LOC100757965	100757965	0.11	0.00	0.00	0.00	0.00	0.00	1.52	0.92	0.00	3.48	1.03	0.00

protein LSM14 homolog B-like	LOC100 757966	100757966	13.77	14.04	13.08	11.21	12.24	11.64	258.24	265.36	207.43	163.41	171.81	156.09
retinoic acid early-inducible protein 1-alpha-like	LOC100 757967	100757967	3.93	4.56	2.82	3.66	4.43	3.64	413.19	463.01	298.10	372.01	438.83	370.13
pyrin and HIN domain-containing protein 1-like	LOC100 757974	100757974	0.09	0.06	0.13	0.22	0.14	0.14	5.32	3.66	8.69	13.91	9.31	11.22
40S ribosomal protein SA pseudogene	LOC100 757975	100757975	15.47	12.71	14.30	11.67	4.79	8.46	299.26	244.31	281.95	223.67	86.94	159.83
keratin-associated protein 12-1	LOC100 757976	100757976	0.00	0.00	0.00	0.00	0.00	0.00	0.00	0.00	0.00	0.00	0.00	0.00
short coiled-coil protein-like	LOC100 757977	100757977	0.20	0.00	0.00	0.00	0.00	0.00	0.76	0.00	0.00	0.00	0.00	0.00
bromodomain and WD repeat-containing protein 3-like	LOC100 757981	100757981	0.00	0.00	0.00	0.03	0.00	0.00	0.00	0.00	0.00	1.16	0.00	0.00
olfactory receptor 4A5-like	LOC100 757997	100757997	0.00	0.00	0.00	0.00	0.00	0.00	0.00	0.00	0.00	0.00	0.00	0.00
60S ribosomal protein L23a pseudogene	LOC100 757998	100757998	0.09	0.00	0.00	0.00	0.00	0.00	0.76	0.00	0.00	0.00	0.00	0.00
olfactory receptor 9G4-like	LOC100 758000	100758000	0.00	0.00	0.00	0.05	0.00	0.04	0.00	0.00	0.00	1.16	0.00	0.93
late cornified envelope protein 3D-like	LOC100 758008	100758008	0.00	0.00	0.00	0.00	0.00	0.00	0.00	0.00	0.00	0.00	0.00	0.00
60S ribosomal protein L34 pseudogene	LOC100 758010	100758010	4.67	3.16	4.05	2.15	3.21	1.70	25.82	11.90	17.39	15.07	19.66	16.82
alpha-enolase pseudogene	LOC100 758015	100758015	3.26	3.59	3.88	3.08	2.61	2.27	0.00	0.00	0.00	0.00	0.00	0.00
ran guanine nucleotide release factor pseudogene	LOC100 758019	100758019	1.18	1.19	1.74	0.68	1.03	0.51	23.55	22.88	24.84	9.27	12.42	13.09
zinc finger protein 660-like	LOC100 758022	100758022	0.07	0.02	0.04	0.05	0.03	0.01	1.52	2.75	1.24	0.00	1.03	0.00
chromosome unknown open reading frame, human C9orf84	LOC100 758025	100758025	0.01	0.01	0.00	0.01	0.01	0.00	1.52	1.83	0.00	2.32	2.07	0.93
40S ribosomal protein S17 pseudogene	LOC100 758027	100758027	4.73	4.94	4.76	2.38	2.66	2.53	28.86	22.88	26.08	13.91	17.59	17.76
transcription elongation factor B polypeptide 1-like	LOC100 758029	100758029	0.00	0.00	0.00	0.00	0.00	0.00	0.00	0.00	0.00	0.00	0.00	0.00

histo-blood group ABO system transferase	LOC100758032	100758032	0.00	0.00	0.00	0.00	0.00	0.00	0.00	0.00	0.00	0.00	0.00	0.00
olfactory receptor 5B2	LOC100758033	100758033	0.00	0.00	0.00	0.00	0.00	0.00	0.00	0.00	0.00	0.00	0.00	0.00
zinc finger protein 431-like	LOC100758039	100758039	0.00	0.00	0.00	0.00	0.00	0.00	0.00	0.00	0.00	0.00	0.00	0.00
UDP-glucuronosyltransferase 1-8-like	LOC100758042	100758042	0.10	0.07	0.00	0.02	0.04	0.05	0.00	0.00	0.00	0.00	0.00	0.00
bystin-like	LOC100758046	100758046	0.80	0.74	0.51	0.78	0.86	0.27	13.67	10.07	12.42	15.07	11.38	4.67
transmembrane protease serine 11D-like	LOC100758048	100758048	0.00	0.00	0.00	0.00	0.00	0.00	0.00	0.00	0.00	0.00	0.00	0.00
sal-like protein 4	LOC100758049	100758049	0.00	0.00	0.00	0.00	0.00	0.00	0.00	0.00	0.00	0.00	0.00	0.00
zinc finger protein 773-like	LOC100758050	100758050	0.00	0.00	0.00	0.00	0.00	0.00	0.00	0.00	0.00	1.16	0.00	0.00
RING-box protein 2 pseudogene	LOC100758051	100758051	13.26	14.07	7.78	4.51	2.82	3.18	129.12	136.34	78.25	45.20	27.94	30.84
grpE protein homolog 2, mitochondrial pseudogene	LOC100758052	100758052	0.11	0.09	0.00	0.00	0.05	0.09	2.28	4.58	2.48	0.00	4.14	1.87
PWWP domain-containing protein MUM1L1	LOC100758054	100758054	0.48	0.49	0.24	0.33	0.35	0.41	56.21	55.82	33.54	41.72	35.19	44.86
JNK1/MAPK8-associated membrane protein pseudogene	LOC100758057	100758057	0.00	0.00	0.00	0.00	0.00	0.00	0.00	0.00	0.00	0.00	0.00	0.00
zinc finger protein 420-like	LOC100758060	100758060	0.00	0.00	0.03	0.00	0.00	0.00	0.00	0.00	0.00	0.00	0.00	0.00
proto-oncogene FRAT1-like	LOC100758062	100758062	0.00	0.00	0.00	0.00	0.00	0.00	0.00	0.00	0.00	0.00	0.00	0.00
olfactory receptor 2A1/2A42-like	LOC100758063	100758063	0.00	0.00	0.00	0.00	0.00	0.00	0.00	0.00	0.00	0.00	0.00	0.00
60 kDa heat shock protein, mitochondrial-like	LOC100758064	100758064	0.05	0.03	0.03	0.03	0.00	0.05	2.28	0.92	1.24	1.16	0.00	1.87
histone H2A type 2-A	LOC100758065	100758065	1.72	1.31	0.55	0.14	0.17	0.18	34.18	29.28	14.91	3.48	4.14	1.87
cyclin-dependent kinase 12-like	LOC100758066	100758066	25.63	27.56	19.99	22.30	26.22	21.16	0.00	0.00	0.00	0.00	0.00	0.00

proto-oncogene FRAT1-like	LOC100758069	100758069	0.00	0.00	0.05	0.00	0.00	0.00	0.00	0.00	0.00	0.00	0.00	0.00
nucleophosmin pseudogene	LOC100758076	100758076	8.29	8.27	7.88	7.30	6.87	5.41	290.90	293.73	268.29	310.59	272.20	241.14
ubiquitin-conjugating enzyme E2 variant 1 pseudogene	LOC100758077	100758077	0.00	0.00	0.00	0.00	0.00	0.00	0.00	0.00	0.00	0.00	0.00	0.00
nucleolar RNA helicase 2 pseudogene	LOC100758078	100758078	0.00	0.00	0.00	0.00	0.00	0.00	0.00	0.00	0.00	0.00	0.00	0.00
40S ribosomal protein S26-like	LOC100758080	100758080	0.00	0.00	0.00	0.00	0.00	0.00	0.00	0.00	0.00	0.00	0.00	0.00
pyruvate kinase PKM pseudogene	LOC100758090	100758090	0.06	0.30	0.23	0.12	0.21	0.07	2.28	11.90	8.69	4.64	8.28	2.80
endothelial cell-specific chemotaxis regulator-like	LOC100758094	100758094	0.00	0.00	0.00	0.00	0.13	0.00	0.00	0.00	0.00	0.00	1.03	0.00
patr class I histocompatibility antigen, A-126 alpha chain-like	LOC100758095	100758095	0.00	0.03	0.01	0.00	0.00	0.00	0.76	3.66	0.00	1.16	0.00	0.00
NACHT, LRR and PYD domains-containing protein 9A-like	LOC100758096	100758096	0.00	0.01	0.01	0.00	0.00	0.00	0.00	0.92	1.24	0.00	0.00	0.00
chromosome unknown open reading frame, human C16orf70	LOC100758102	100758102	13.89	13.19	14.51	14.00	14.13	15.32	985.12	967.19	1065.71	1046.50	1039.13	1097.30
RNA-binding protein 4B	LOC100758105	100758105	9.16	8.54	8.26	7.50	7.03	7.97	205.08	193.99	199.98	157.61	171.81	197.22
non-histone chromosomal protein HMG-17	LOC100758108	100758108	0.21	1.80	0.21	1.29	0.18	0.67	7.60	10.98	1.24	6.95	0.00	2.80
apolipoprotein L3-like	LOC100758111	100758111	0.00	0.02	0.00	0.00	0.02	0.00	0.00	0.92	0.00	0.00	1.03	0.00
NUT family member Gm806-like	LOC100758113	100758113	0.00	0.00	0.00	0.00	0.00	0.00	0.00	0.00	0.00	0.00	0.00	0.00
interferon-induced transmembrane protein 2	LOC100758119	100758119	0.09	0.12	0.13	0.21	0.28	0.28	7.60	8.24	12.42	18.54	21.73	22.43
aldose reductase-related protein 2	LOC100758120	100758120	0.00	0.04	0.00	0.00	0.05	0.04	0.00	0.00	0.00	0.00	0.00	0.00
6-phosphofructokinase type C pseudogene	LOC100758124	100758124	0.02	0.02	0.00	0.05	0.00	0.02	0.76	0.92	0.00	2.32	0.00	0.00
glyceraldehyde-3-phosphate dehydrogenase pseudogene	LOC100758128	100758128	0.00	0.00	0.00	0.00	0.00	0.00	0.00	0.00	0.00	0.00	0.00	0.00

tudor and KH domain-containing protein pseudogene	LOC100758130	100758130	0.00	0.00	0.00	0.00	0.00	0.00	0.00	0.00	0.00	0.00	0.00	0.00
selection and upkeep of intraepithelial T-cells protein 8-like	LOC100758132	100758132	0.00	0.00	0.00	0.00	0.00	0.00	0.00	0.00	0.00	0.00	0.00	0.00
serpin B9	LOC100758135	100758135	0.07	0.06	0.00	0.03	0.04	0.02	0.00	0.00	0.00	0.00	0.00	0.00
NACHT, LRR and PYD domains-containing protein 1-like	LOC100758136	100758136	0.00	0.00	0.00	0.00	0.00	0.00	0.00	0.00	0.00	0.00	0.00	0.00
GRB2-associated-binding protein 2-like	LOC100758138	100758138	0.00	0.00	0.00	0.00	0.00	0.00	0.00	0.00	0.00	0.00	0.00	0.00
40S ribosomal protein S12 pseudogene	LOC100758141	100758141	0.00	0.00	0.00	0.00	0.00	0.00	0.00	0.00	0.00	0.00	0.00	0.00
olfactory receptor 10V1-like	LOC100758143	100758143	0.15	0.00	0.10	0.00	0.08	0.11	4.56	4.58	3.73	0.00	6.21	2.80
beta-defensin 109-like	LOC100758145	100758145	0.00	0.00	0.00	0.00	0.00	0.00	0.00	0.00	1.24	0.00	1.03	0.00
mesencephalic astrocyte-derived neurotrophic factor pseudogene	LOC100758146	100758146	0.07	0.24	0.11	0.00	0.14	0.25	0.76	0.92	1.24	0.00	0.00	0.93
AP-1 complex-associated regulatory protein	LOC100758150	100758150	23.39	23.46	17.95	22.83	25.03	24.34	1315.52	1315.82	1011.06	1291.04	1408.62	1376.77
transmembrane protein 101	LOC100758152	100758152	33.55	30.62	38.52	30.23	17.06	21.11	9.87	10.98	7.45	4.64	1.03	5.61
vomeronasal type-1 receptor 4-like	LOC100758153	100758153	0.00	0.00	0.00	0.00	0.00	0.00	0.00	0.00	0.00	0.00	0.00	0.00
interferon-induced transmembrane protein 2-like	LOC100758155	100758155	56.82	50.72	56.00	58.09	49.12	52.88	10.63	10.98	9.94	4.64	4.14	6.54
RNMT-activating mini protein pseudogene	LOC100758156	100758156	0.00	0.00	0.00	0.00	0.00	0.00	0.00	0.00	0.00	0.00	0.00	0.00
DPH3 homolog	LOC100758158	100758158	0.00	0.00	0.00	0.00	0.00	0.00	0.00	0.00	0.00	0.00	0.00	0.00
histone H2A.Z pseudogene	LOC100758162	100758162	0.00	0.00	0.00	0.00	0.00	0.00	0.00	0.00	0.00	0.00	0.00	0.00
protein arginine N-methyltransferase 5-like	LOC100758180	100758180	0.06	0.17	0.05	0.02	0.02	0.00	4.56	10.07	3.73	1.16	1.03	0.00
glyceraldehyde-3-phosphate dehydrogenase pseudogene	LOC100758182	100758182	0.00	0.00	0.06	0.00	0.00	0.00	0.00	0.00	1.24	0.00	0.00	0.00

chromosome unknown open reading frame, human C7orf34	LOC100758186	100758186	0.00	0.00	0.00	0.00	0.00	0.00	0.00	0.00	0.00	0.00	0.00	0.00
olfactory receptor 4C16-like	LOC100758191	100758191	0.00	0.00	0.00	0.00	0.00	0.00	0.00	0.00	0.00	0.00	0.00	0.00
adenylate kinase 4, mitochondrial pseudogene	LOC100758193	100758193	0.05	0.00	0.00	0.00	0.07	0.07	0.76	0.00	0.00	1.16	1.03	0.93
caspase-6 pseudogene	LOC100758198	100758198	0.00	0.00	0.00	0.00	0.00	0.00	0.00	0.00	0.00	0.00	0.00	0.00
heterogeneous nuclear ribonucleoproteins C1/C2-like	LOC100758202	100758202	1.12	1.06	1.13	1.08	1.17	0.86	20.51	14.64	21.12	18.54	20.70	14.02
60S ribosomal protein L30-like	LOC100758208	100758208	253.54	237.48	272.23	237.13	151.38	169.90	1848.72	1715.69	1930.20	1569.18	998.76	1110.39
60S ribosomal protein L7a pseudogene	LOC100758213	100758213	3.10	2.67	2.21	1.96	1.47	1.76	25.06	13.73	16.15	17.38	8.28	13.09
platelet factor 4-like	LOC100758214	100758214	0.00	0.00	0.00	0.00	0.00	0.00	0.00	0.00	0.00	0.00	0.00	0.00
olfactory receptor 5AN1	LOC100758221	100758221	0.00	0.00	0.00	0.00	0.00	0.00	0.00	0.00	0.00	0.00	0.00	0.00
EF-hand calcium-binding domain-containing protein 10	LOC100758222	100758222	0.00	0.00	0.00	0.00	0.00	0.00	0.00	0.00	0.00	0.00	0.00	0.00
putative olfactory receptor ENSP00000348552	LOC100758224	100758224	0.00	0.01	0.00	0.00	0.00	0.00	0.00	0.00	0.00	0.00	0.00	0.00
brachyury protein-like	LOC100758225	100758225	0.02	0.00	0.00	0.00	0.00	0.00	1.52	0.00	0.00	0.00	0.00	0.00
FERM and PDZ domain-containing protein 2-like	LOC100758229	100758229	0.01	0.00	0.00	0.00	0.00	0.00	0.76	0.00	0.00	0.00	0.00	0.00
5'-AMP-activated protein kinase subunit gamma-1 pseudogene	LOC100758233	100758233	0.00	0.00	0.10	0.00	0.00	0.00	0.00	0.00	2.48	0.00	0.00	0.00
arylacetamide deacetylase-like 4	LOC100758234	100758234	0.00	0.00	0.00	0.00	0.00	0.00	0.00	0.00	0.00	0.00	0.00	0.00
poly(A)-specific ribonuclease PARN-like domain-containing protein 1 pseudogene	LOC100758238	100758238	0.04	0.02	0.00	0.00	0.00	0.00	1.52	0.92	0.00	0.00	0.00	0.00
Ig heavy chain V-III region VH26-like	LOC100758242	100758242	0.00	0.00	0.00	0.00	0.00	0.00						
serine/threonine-protein phosphatase 4 regulatory subunit 1-like	LOC100758248	100758248	0.00	0.00	0.00	0.00	0.00	0.00	0.00	0.00	0.00	0.00	0.00	0.00

disintegrin and metalloproteinase domain-containing protein 21-like	LOC100 758249	100758249	0.00	0.00	0.00	0.02	0.00	0.00	0.00	0.00	0.00	1.16	0.00	0.00
odorant-binding protein 2a-like	LOC100 758255	100758255	0.00	0.00	0.00	0.00	0.00	0.00	0.00	0.00	0.00	0.00	0.00	0.00
nuclear pore complex protein Nup85-like	LOC100 758257	100758257	27.34	21.54	28.89	20.48	17.01	18.26	259.00	215.95	285.68	190.06	155.25	172.91
40S ribosomal protein S15 pseudogene	LOC100 758261	100758261	24.53	23.67	12.88	3.43	2.33	3.19	171.66	160.13	86.95	23.18	15.52	21.50
interferon-activable protein 203-like	LOC100 758264	100758264	0.02	0.05	0.00	0.03	0.06	0.00	0.76	1.83	2.48	1.16	1.03	0.00
keratin-associated protein 12-1-like	LOC100 758265	100758265	0.00	0.00	0.00	0.00	0.00	0.00	0.00	0.00	0.00	0.00	0.00	0.00
60S ribosomal protein L39 pseudogene	LOC100 758276	100758276	6.44	5.82	3.66	4.60	3.39	7.41	7.60	5.49	3.73	3.48	5.17	7.48
alpha-methylacyl-CoA racemase	LOC100 758278	100758278	38.94	45.42	47.58	45.01	45.94	42.43	865.87	990.98	1017.27	1014.05	992.55	951.49
pre-mRNA-splicing regulator WTAP pseudogene	LOC100 758281	100758281	3.09	3.03	3.19	2.85	3.29	3.16	36.46	45.75	40.99	40.56	66.24	60.75
olfactory receptor 1013-like	LOC100 758287	100758287	0.00	0.07	0.00	0.00	0.00	0.00	0.76	1.83	0.00	1.16	0.00	0.00
thioredoxin	LOC100 758292	100758292	366.95	352.52	283.34	303.26	297.24	293.07	5240.82	5241.31	4066.59	4387.67	4398.69	4272.38
mitochondrial import inner membrane translocase subunit Tim23 pseudogene	LOC100 758295	100758295	1.80	1.16	1.02	0.37	0.48	0.58	22.03	12.81	11.18	10.43	8.28	9.35
chromosome unknown open reading frame, human C10orf54	LOC100 758303	100758303	0.03	0.00	0.00	0.00	0.00	0.00	0.76	0.00	0.00	0.00	0.00	0.00
male-enhanced antigen 1 pseudogene	LOC100 758304	100758304	6.04	5.27	5.74	4.56	5.02	4.42	7.60	9.15	6.21	8.11	8.28	6.54
zinc finger protein 420-like	LOC100 758306	100758306	0.60	0.58	0.80	0.88	0.99	1.02	34.94	31.11	44.72	55.63	58.99	63.56
lymphocyte antigen 6H	LOC100 758308	100758308	0.00	0.00	0.00	0.00	0.00	0.00	0.00	0.00	0.00	0.00	0.00	0.00
prorelaxin-like	LOC100 758313	100758313	0.00	0.00	0.00	0.00	0.00	0.00	0.00	0.00	0.00	0.00	0.00	0.00
tubulin alpha-1C chain-like	LOC100 758315	100758315	0.00	0.03	0.00	0.00	0.00	0.03	0.00	0.92	0.00	0.00	0.00	0.93

olfactory receptor 5B2	LOC100758317	100758317	0.00	0.00	0.00	0.00	0.00	0.00	0.00	0.00	0.00	0.00	0.00	0.00
solute carrier family 7 member 13	LOC100758319	100758319	0.02	0.02	0.03	0.02	0.04	0.06	0.76	0.92	2.48	1.16	4.14	2.80
low-density lipoprotein receptor-related protein 10 pseudogene	LOC100758321	100758321	0.00	0.00	0.00	0.00	0.00	0.00	0.00	0.00	0.00	0.00	0.00	0.00
ankyrin repeat domain-containing protein 26-like	LOC100758324	100758324	0.00	0.02	0.00	0.00	0.01	0.00	0.00	1.83	0.00	0.00	1.03	0.93
zinc finger protein 30 homolog	LOC100758327	100758327	2.28	1.90	2.12	2.31	2.18	2.28	175.45	152.81	161.47	180.79	166.63	172.91
zinc finger protein 773-like	LOC100758333	100758333	0.98	0.78	0.57	0.74	0.79	0.61	73.68	72.29	54.65	56.79	69.34	57.01
ficolin-2	LOC100758341	100758341	0.00	0.00	0.00	0.00	0.00	0.00	0.00	0.00	0.00	0.00	0.00	0.00
mitochondrial import receptor subunit TOM20 homolog pseudogene	LOC100758342	100758342	0.00	0.00	0.00	0.00	0.00	0.00	0.00	0.00	0.00	0.00	0.00	0.93
tyrosine-protein phosphatase non-receptor type substrate 1-like	LOC100758347	100758347	0.00	0.00	0.00	0.00	0.00	0.00	0.00	0.00	0.00	0.00	0.00	0.00
eukaryotic translation initiation factor 5 pseudogene	LOC100758349	100758349	0.00	0.00	0.00	0.00	0.00	0.00	0.00	0.00	0.00	0.00	0.00	0.00
histone H2A type 2-A	LOC100758350	100758350	0.55	0.76	0.76	0.07	0.04	0.00	0.76	1.83	3.73	0.00	0.00	0.93
myc proto-oncogene protein-like	LOC100758352	100758352	569.25	572.52	536.23	515.50	475.44	467.99	14967.48	15090.73	14195.81	13508.37	12479.86	12379.70
chromosome unknown open reading frame, human C6orf62	LOC100758362	100758362	22.57	21.13	22.60	25.71	28.24	25.74	2108.48	1970.98	2130.18	2408.24	2612.31	2403.97
sperm-associated antigen 6	LOC100758363	100758363	0.00	0.00	0.00	0.00	0.00	0.00	0.00	0.00	0.00	0.00	0.00	0.00
bcl-2-associated transcription factor 1 pseudogene	LOC100758364	100758364	1.96	2.00	1.40	1.43	1.62	1.72	11.39	23.79	8.69	8.11	9.31	7.48
40S ribosomal protein S11-like	LOC100758366	100758366	0.32	0.43	0.44	0.52	0.48	0.25	13.67	12.81	14.91	9.27	11.38	9.35
chromosome unknown open reading frame, human C17orf47	LOC100758370	100758370	0.00	0.00	0.00	0.00	0.00	0.00	0.00	0.00	0.00	0.00	0.00	0.00
60S ribosomal protein L23a pseudogene	LOC100758371	100758371	4.44	2.50	2.38	2.00	2.35	1.47	31.90	18.30	17.39	13.91	16.56	9.35

60S ribosomal protein L23a pseudogene	LOC100758373	100758373	0.00	0.00	0.00	0.32	0.00	0.00	0.00	0.00	0.00	2.32	0.00	0.00
40S ribosomal protein S12 pseudogene	LOC100758375	100758375	2.89	2.80	2.51	2.31	4.78	2.56	84.31	84.18	94.40	96.19	145.93	102.81
activated RNA polymerase II transcriptional coactivator p15	LOC100758384	100758384	0.06	0.07	0.00	0.20	0.00	0.16	0.00	0.00	0.00	0.00	1.03	0.00
diphosphoinositol polyphosphate phosphohydrolase 3-alpha	LOC100758392	100758392	0.00	0.00	0.14	0.00	0.00	0.00	0.00	0.00	1.24	0.00	0.00	0.00
NUT family member Gm806-like	LOC100758397	100758397	0.00	0.00	0.00	0.00	0.00	0.00	0.00	0.00	0.00	0.00	0.00	0.00
vesicle-associated membrane protein 8 pseudogene	LOC100758398	100758398	3.64	3.18	3.00	3.48	3.59	4.03	11.39	9.15	6.21	13.91	12.42	12.15
putative V-set and immunoglobulin domain-containing-like protein IGHV4OR15-8	LOC100758408	100758408	0.00	0.00	0.00	0.00	0.00	0.00						
MAM domain-containing protein 2	LOC100758409	100758409	37.73	39.19	39.21	44.15	46.63	44.45	1893.53	1992.94	1988.58	2264.53	2363.91	2210.49
60S ribosomal protein L22-like 1	LOC100758411	100758411	0.00	0.00	0.00	0.00	0.00	0.00	0.00	0.00	0.00	1.16	0.00	0.93
selection and upkeep of intraepithelial T-cells protein 1-like	LOC100758417	100758417	0.04	0.00	0.00	0.00	0.00	0.00	3.80	0.00	0.00	0.00	0.00	0.00
Ig lambda chain V-VI region EB4-like	LOC100758418	100758418	0.00	0.00	0.00	0.00	0.00	0.00						
serpin B9-like	LOC100758420	100758420	0.00	0.06	0.00	0.00	0.00	0.00	0.00	0.92	0.00	0.00	0.00	0.00
60S ribosomal protein L22 pseudogene	LOC100758421	100758421	0.00	0.00	0.00	0.00	0.00	0.00	0.00	0.00	0.00	0.00	0.00	0.00
histone H2A.Z pseudogene	LOC100758422	100758422	0.00	0.00	0.00	0.00	0.00	0.00	0.00	0.00	0.00	0.00	0.00	0.00
40S ribosomal protein S6 pseudogene	LOC100758423	100758423	2.21	2.02	1.67	1.17	2.01	1.45	30.38	22.88	17.39	16.22	30.01	20.56
histone H3.3 pseudogene	LOC100758425	100758425	0.00	0.00	0.00	0.00	0.00	0.00	0.00	0.00	0.00	0.00	0.00	0.00
chromosome unknown open reading frame, human C10orf82	LOC100758426	100758426	0.74	0.76	0.37	0.53	0.50	0.51	60.00	64.05	42.23	46.36	37.26	43.93
rho GDP-dissociation inhibitor 1 pseudogene	LOC100758428	100758428	0.00	0.00	0.00	0.00	0.00	0.00	0.00	0.00	0.00	0.00	0.00	0.00

olfactory receptor 10V1	LOC100758429	100758429	0.00	0.00	0.00	0.00	0.00	0.00	0.00	0.00	0.00	0.00	0.00	0.00
kinetochore protein Spc25 pseudogene	LOC100758435	100758435	0.00	0.00	0.00	0.00	0.00	0.00	0.00	0.00	0.00	0.00	0.00	0.00
transmembrane protein 184C pseudogene	LOC100758437	100758437	0.00	0.06	0.00	0.00	0.03	0.00	0.00	1.83	0.00	1.16	1.03	0.00
olfactory receptor 1468	LOC100758441	100758441	0.00	0.00	0.00	0.00	0.00	0.00	0.00	0.00	0.00	0.00	0.00	0.00
CDC42 small effector protein 2	LOC100758446	100758446	10.22	10.50	10.31	12.41	11.72	12.19	166.34	165.62	190.04	205.13	182.16	197.22
THO complex subunit 1-like	LOC100758451	100758451	0.33	0.39	0.10	0.08	0.36	0.21	7.60	10.98	1.24	1.16	7.24	5.61
zinc finger protein SNAI1 pseudogene	LOC100758454	100758454	0.00	0.00	0.00	0.00	0.00	0.00	2.28	0.00	0.00	0.00	0.00	0.00
chromosome unknown open reading frame, human C15orf60	LOC100758463	100758463	0.19	0.08	0.05	0.05	0.22	0.08	0.00	0.92	1.24	1.16	2.07	0.93
glyceraldehyde-3-phosphate dehydrogenase pseudogene	LOC100758468	100758468	0.00	0.00	0.00	0.00	0.00	0.00	0.00	0.00	0.00	0.00	0.00	0.00
nucleophosmin-like	LOC100758469	100758469	0.00	0.00	0.00	0.00	0.00	0.00	0.00	0.00	0.00	0.00	0.00	0.00
grpE protein homolog 1, mitochondrial pseudogene	LOC100758470	100758470	0.00	0.00	0.00	0.00	0.00	0.00	0.00	0.00	0.00	0.00	0.00	0.00
Ig-like V-type domain-containing protein FAM187A	LOC100758476	100758476	4.34	4.30	3.22	3.01	3.38	3.60	291.66	283.66	214.88	202.81	227.70	244.88
glyceraldehyde-3-phosphate dehydrogenase pseudogene	LOC100758478	100758478	0.78	0.48	0.42	0.19	0.40	0.22	8.35	5.49	3.73	3.48	6.21	3.74
olfactory receptor 4C11-like	LOC100758479	100758479	0.00	0.00	0.00	0.00	0.00	0.00	0.00	0.00	0.00	0.00	0.00	0.00
olfactory receptor 4A15-like	LOC100758480	100758480	0.00	0.00	0.00	0.00	0.00	0.00	0.00	0.00	0.00	0.00	0.00	0.00
H-2 class I histocompatibility antigen, Q10 alpha chain-like	LOC100758481	100758481	7.23	6.02	6.28	4.24	3.20	5.56	40.26	36.60	29.81	22.02	14.49	26.17
tetratricopeptide repeat protein 9C pseudogene	LOC100758495	100758495	0.00	0.12	0.08	0.07	0.00	0.06	0.00	1.83	1.24	1.16	0.00	0.93
ETS translocation variant 3-like protein	LOC100758497	100758497	8.82	8.32	6.42	8.11	5.85	3.34	41.02	29.28	27.33	32.45	23.80	14.02

40S ribosomal protein S7 pseudogene	LOC100 758499	100758499	0.07	0.00	0.00	0.00	0.00	0.00	0.76	0.00	0.00	0.00	0.00	0.00
lysophosphatidic acid receptor 4-like	LOC100 758504	100758504	0.00	0.00	0.00	0.00	0.00	0.00	0.00	0.00	0.00	0.00	0.00	0.00
olfactory receptor 4K3-like	LOC100 758505	100758505	0.00	0.00	0.00	0.00	0.00	0.00	0.00	0.00	0.00	0.00	0.00	0.00
olfactory receptor 5A2	LOC100 758506	100758506	0.00	0.00	0.00	0.00	0.00	0.00	0.00	0.00	0.00	0.00	0.00	0.00
uncharacterized LOC100758509	LOC100 758509	100758509	0.06	0.08	0.06	0.07	0.14	0.10	12.91	16.47	8.69	9.27	14.49	12.15
protein FAM98A pseudogene	LOC100 758510	100758510	9.44	9.92	10.47	9.74	10.38	11.09	375.21	398.95	411.13	394.03	404.68	443.97
peptidyl-prolyl cis-trans isomerase A-like	LOC100 758511	100758511	16.03	20.02	11.75	5.65	17.15	8.29	66.08	83.27	47.20	17.38	71.41	30.84
chromosome unknown open reading frame, human C12orf40	LOC100 758516	100758516	0.24	0.23	0.13	0.14	0.26	0.22	20.51	21.05	12.42	11.59	21.73	18.69
eukaryotic translation initiation factor 4E pseudogene	LOC100 758517	100758517	0.00	0.00	0.00	0.00	0.00	0.00	0.76	1.83	0.00	0.00	0.00	0.00
arylacetamide deacetylase-like 4	LOC100 758519	100758519	0.00	0.00	0.00	0.00	0.00	0.00	0.00	0.00	0.00	0.00	0.00	0.00
adenosine 3'-phospho 5'-phosphosulfate transporter 1 pseudogene	LOC100 758522	100758522	0.50	0.31	0.62	0.28	0.30	0.59	12.91	9.15	16.15	6.95	6.21	13.09
tubulin alpha-1B chain-like	LOC100 758525	100758525	0.02	0.00	0.00	0.07	0.06	0.03	0.76	0.00	0.00	2.32	2.07	0.93
eukaryotic translation initiation factor 2 subunit 2 pseudogene	LOC100 758535	100758535	8.91	7.82	6.66	6.04	6.31	4.99	136.72	111.63	100.61	71.85	76.59	69.17
60S ribosomal protein L29 pseudogene	LOC100 758536	100758536	0.00	0.00	0.00	0.00	0.00	0.00	0.00	0.00	0.00	0.00	0.00	0.00
U3 small nucleolar RNA-associated protein 14 homolog B	LOC100 758539	100758539	1.36	1.31	2.20	2.37	2.34	2.16	163.30	156.47	263.32	288.57	282.55	263.58
lysozyme C-2-like	LOC100 758541	100758541	0.98	1.21	1.26	1.57	1.48	1.54	30.38	42.09	42.23	48.67	38.29	51.41
sialidase-3 pseudogene	LOC100 758542	100758542	1.60	1.48	1.18	1.74	1.35	1.64	1.52	0.00	0.00	2.32	0.00	1.87
uncharacterized LOC100758547	LOC100 758547	100758547	0.01	0.00	0.00	0.00	0.00	0.00	0.76	0.00	0.00	0.00	0.00	0.00

interferon-activable protein 204	LOC100 758550	100758550	5.96	5.42	8.10	9.52	8.91	8.20	574.97	516.99	792.45	924.82	847.65	794.47
keratin-associated protein 12-1	LOC100 758552	100758552	0.00	0.00	0.00	0.00	0.00	0.00	0.00	0.00	0.00	0.00	0.00	0.00
40S ribosomal protein S29 pseudogene	LOC100 758567	100758567	12.79	15.08	16.63	12.94	13.02	14.18	18.99	15.56	16.15	9.27	10.35	9.35
60S ribosomal protein L36a-like	LOC100 758569	100758569	21.81	23.12	19.73	16.45	22.29	17.44	46.33	63.14	57.14	41.72	51.75	25.24
40S ribosomal protein S27-like pseudogene	LOC100 758571	100758571	3.37	5.77	3.40	2.41	3.93	2.10	28.10	30.20	24.84	15.07	16.56	13.09
olfactory receptor 2G3	LOC100 758576	100758576	0.06	0.00	0.05	0.00	0.00	0.00	1.52	0.00	1.24	0.00	0.00	0.00
eukaryotic translation initiation factor 1A	LOC100 758579	100758579	90.52	90.03	81.17	90.29	101.40	94.81	2760.17	2722.22	2451.88	2751.28	3020.09	2936.73
mitochondrial import inner membrane translocase subunit Tim17-A-like	LOC100 758581	100758581	0.00	0.00	0.00	0.00	0.00	0.00	0.00	0.00	0.00	0.00	0.00	0.00
protein sprouty homolog 4-like	LOC100 758585	100758585	0.00	0.00	0.00	0.00	0.00	0.00	0.00	0.00	0.00	0.00	0.00	0.00
epoxide hydrolase 1	LOC100 758590	100758590	131.01	125.38	179.07	165.69	124.16	147.91	3304.76	3196.21	4552.25	4489.66	3522.06	3925.61
olfactory receptor 6S1	LOC100 758593	100758593	0.00	0.04	0.00	0.00	0.00	0.00	0.00	0.92	0.00	0.00	0.00	0.00
chromosome unknown open reading frame, human C10orf105	LOC100 758594	100758594	0.00	0.00	0.00	0.00	0.00	0.00	0.00	0.00	0.00	0.00	0.00	0.00
zinc finger protein 420-like	LOC100 758597	100758597	2.00	2.03	2.20	2.79	2.80	2.44	196.72	204.97	218.61	280.46	282.55	242.08
olfactory receptor 5B2	LOC100 758609	100758609	0.00	0.00	0.00	0.00	0.00	0.00	0.00	0.00	0.00	0.00	0.00	0.00
chromosome unknown open reading frame, human C10orf10	LOC100 758614	100758614	0.00	0.00	0.00	0.00	0.00	0.00	0.00	0.00	0.00	0.00	0.00	0.00
60S ribosomal protein L6 pseudogene	LOC100 758615	100758615	2.26	2.49	1.69	1.16	2.38	1.24	48.61	53.07	34.78	24.34	49.68	26.17
chromosome unknown open reading frame, human C4orf36	LOC100 758616	100758616	0.76	0.59	0.48	0.60	0.13	0.42	30.38	22.88	16.15	22.02	9.31	15.89
zinc finger protein 30-like	LOC100 758618	100758618	0.03	0.04	0.00	0.10	0.00	0.00	0.76	1.83	0.00	3.48	2.07	2.80

bcl-2-related protein A1-like	LOC100 758620	100758620	0.95	0.81	0.00	0.00	0.00	0.00	18.23	15.56	0.00	0.00	0.00	0.00
60S ribosomal protein L31 pseudogene	LOC100 758621	100758621	2.44	1.38	0.87	0.67	1.83	0.80	15.95	10.07	7.45	5.79	12.42	5.61
olfactory receptor 5D16	LOC100 758622	100758622	0.00	0.00	0.00	0.00	0.00	0.00	0.00	0.00	0.00	0.00	0.00	0.00
keratin-associated protein 9-3-like	LOC100 758623	100758623	0.00	0.00	0.00	0.00	0.00	0.00	0.00	0.00	0.00	0.00	0.00	0.00
UDP-glucuronosyltransferase 2B17-like	LOC100 758624	100758624	0.00	0.00	0.00	0.00	0.00	0.00	0.00	0.00	0.00	0.00	0.00	0.00
olfactory receptor 10G7	LOC100 758628	100758628	0.00	0.00	0.00	0.00	0.00	0.00	0.00	0.00	0.00	0.00	0.00	0.00
vomeronasal type-2 receptor 116-like	LOC100 758630	100758630	0.00	0.00	0.00	0.00	0.00	0.00	0.00	0.00	0.00	0.00	0.00	0.00
zinc finger protein 2-like	LOC100 758631	100758631	0.00	0.00	0.00	0.00	0.00	0.00	0.00	0.00	0.00	0.00	0.00	0.00
tubulin beta-2B chain-like	LOC100 758633	100758633	0.00	0.01	0.00	0.00	0.01	0.00	0.00	0.00	0.00	0.00	1.03	0.00
Ig heavy chain V-III region VH26-like	LOC100 758635	100758635	0.00	0.00	0.00	0.00	0.00	0.00	0.00	0.00	0.00	0.00	0.00	0.00
14-3-3 protein theta	LOC100 758637	100758637	0.09	0.03	0.08	0.00	0.00	0.03	2.28	0.00	2.48	0.00	0.00	0.00
phosphatidylinositide phosphatase SAC2-like	LOC100 758638	100758638	1.17	1.30	1.19	0.84	1.07	1.84	44.05	58.56	39.75	33.61	40.36	41.13
eukaryotic translation initiation factor 5 pseudogene	LOC100 758640	100758640	0.00	0.00	0.00	0.00	0.00	0.06	0.00	0.00	0.00	0.00	0.00	0.93
histone H2B type 1-like	LOC100 758641	100758641	13.17	13.89	20.96	20.02	20.56	19.33	650.17	698.17	1049.56	1005.94	1020.50	973.93
zinc finger protein ZFAT pseudogene	LOC100 758643	100758643	1.61	1.35	1.25	0.80	0.59	0.57	28.86	25.62	22.36	18.54	8.28	14.95
glyceraldehyde-3-phosphate dehydrogenase-like	LOC100 758654	100758654	0.00	0.00	0.00	0.00	0.00	0.04	0.00	0.00	0.00	0.00	0.00	1.87
60S ribosomal protein L29 pseudogene	LOC100 758655	100758655	0.07	0.09	0.59	0.22	0.30	0.09	0.76	2.75	9.94	2.32	3.10	5.61
baculoviral IAP repeat-containing protein 1b	LOC100 758657	100758657	0.28	0.23	0.06	0.11	0.08	0.05	47.09	42.09	17.39	23.18	7.24	9.35

triosephosphate isomerase-like	LOC100 758660	100758660	0.00	0.00	0.00	0.00	0.00	0.00	0.00	0.00	0.00	0.00	0.00	0.00
protein SET-like	LOC100 758661	100758661	4.30	4.53	3.67	2.88	3.34	3.35	32.66	33.86	23.60	22.02	15.52	26.17
H-2 class I histocompatibility antigen, Q10 alpha chain-like	LOC100 758672	100758672	0.00	0.00	0.00	0.00	0.00	0.00	0.76	0.00	0.00	0.00	1.03	0.00
60S ribosomal protein L32 pseudogene	LOC100 758682	100758682	0.00	0.00	0.00	0.00	0.00	0.00	0.00	0.00	0.00	0.00	0.00	0.00
steroid 17-alpha-hydroxylase/17,20 lyase	LOC100 758683	100758683	0.00	0.00	0.03	0.00	0.00	0.00	0.76	0.00	1.24	2.32	1.03	2.80
polypyrimidine tract-binding protein 1	LOC100 758684	100758684	0.00	0.00	0.03	0.00	0.00	0.00	0.00	0.00	1.24	0.00	1.03	0.00
phosphoglycerate mutase 1-like	LOC100 758688	100758688	0.00	0.00	0.00	0.00	0.00	0.00	0.00	0.00	0.00	0.00	0.00	0.00
chromosome unknown open reading frame, human C2orf73	LOC100 758691	100758691	0.00	0.18	0.26	0.25	0.00	0.00	0.76	2.75	3.73	1.16	1.03	0.00
fatty acyl-CoA reductase 2	LOC100 758702	100758702	0.33	0.26	0.33	0.46	0.48	0.25	22.79	19.22	22.36	28.97	32.08	17.76
chromosome unknown open reading frame, human C12orf57	LOC100 758705	100758705	20.68	20.00	17.45	11.19	7.59	7.94	653.20	633.20	544.03	353.47	246.33	252.36
selection and upkeep of intraepithelial T-cells protein 8-like	LOC100 758708	100758708	0.00	0.00	0.00	0.00	0.00	0.00	0.00	0.00	0.00	0.00	0.00	0.00
NACHT, LRR and PYD domains-containing protein 1-like	LOC100 758714	100758714	0.00	0.00	0.00	0.00	0.00	0.00	0.00	0.00	0.00	0.00	0.00	0.00
olfactory receptor 10V1-like	LOC100 758720	100758720	0.12	0.17	0.05	0.22	0.15	0.08	0.76	0.00	0.00	0.00	0.00	0.00
cytochrome c oxidase assembly protein COX16 homolog, mitochondrial	LOC100 758725	100758725	6.81	6.99	6.68	5.12	6.27	6.89	257.48	267.19	236.00	213.24	230.80	269.18
uncharacterized LOC100758727	LOC100 758727	100758727	0.00	0.00	0.00	0.00	0.12	0.00	0.00	0.00	0.00	0.00	2.07	0.00
	LOC100 758728	100758728	56.15	55.80	52.78	55.26	54.35	55.39	2216.34	2256.47	2167.44	2222.81	2164.16	2238.53
PRAME family member 12-like	LOC100 758729	100758729	0.00	0.00	0.00	0.00	0.00	0.00	0.00	0.00	0.00	0.00	0.00	0.00
60S ribosomal protein L30 pseudogene	LOC100 758730	100758730	476.20	443.95	513.14	488.35	434.64	443.55	2491.29	2338.83	2604.66	2337.54	2138.28	2136.65

cyclin-dependent kinase 11B	LOC100758733	100758733	25.51	21.66	22.34	27.42	29.74	27.85	0.00	0.00	0.00	0.00	0.00	0.00
40S ribosomal protein S29 pseudogene	LOC100758735	100758735	0.00	0.00	0.00	0.00	0.00	0.00	0.00	0.00	0.00	0.00	0.00	0.00
histidine--tRNA ligase, cytoplasmic pseudogene	LOC100758736	100758736	0.00	0.00	0.00	0.00	0.00	0.00	0.00	0.00	0.00	0.00	0.00	0.00
phosphatidylethanolamine-binding protein 1-like	LOC100758742	100758742	0.42	0.25	0.17	0.05	0.00	0.18	3.80	2.75	1.24	0.00	0.00	1.87
beta-defensin 19-like	LOC100758753	100758753	0.00	0.00	0.00	0.00	0.00	0.00	0.00	0.00	0.00	0.00	0.00	0.00
vesicle-associated membrane protein-associated protein B pseudogene	LOC100758759	100758759	1.54	2.46	1.31	1.27	1.99	1.22	135.20	153.73	110.55	101.98	170.77	117.77
N-alpha-acetyltransferase 40 pseudogene	LOC100758760	100758760	0.00	0.00	0.00	0.00	0.00	0.00	0.00	0.00	2.48	0.00	0.00	0.00
transmembrane protein 101-like	LOC100758764	100758764	0.00	0.00	0.00	0.00	0.00	0.00	0.00	0.00	0.00	0.00	0.00	0.00
transmembrane protein 45A-like	LOC100758768	100758768	0.00	0.00	0.00	0.00	0.00	0.00	0.00	0.00	0.00	0.00	0.00	0.00
olfactory receptor 4P4	LOC100758769	100758769	0.00	0.00	0.00	0.00	0.00	0.00	0.00	0.00	0.00	0.00	0.00	0.00
olfactory receptor 4A16-like	LOC100758770	100758770	0.00	0.00	0.00	0.00	0.00	0.00	0.00	0.00	0.00	0.00	0.00	0.00
protein DEK pseudogene	LOC100758771	100758771	0.27	0.21	0.07	0.00	0.06	0.11	4.56	2.75	1.24	0.00	1.03	1.87
cytochrome P450 2C29-like	LOC100758779	100758779	0.00	0.00	0.00	0.00	0.00	0.00	0.00	0.00	0.00	0.00	0.00	0.00
ubiquitin carboxyl-terminal hydrolase 5 pseudogene	LOC100758782	100758782	0.00	0.00	0.00	0.00	0.02	0.00	0.00	0.00	0.00	0.00	1.03	0.00
26S protease regulatory subunit 4 pseudogene	LOC100758785	100758785	0.29	0.11	0.09	0.23	0.06	0.08	10.63	5.49	2.48	9.27	4.14	2.80
C-X-C motif chemokine 2-like	LOC100758790	100758790	0.00	0.00	0.00	0.00	0.00	0.00	0.00	0.00	0.00	0.00	0.00	0.00
olfactory receptor 4K3-like	LOC100758794	100758794	0.00	0.00	0.00	0.00	0.00	0.00	0.00	0.00	0.00	0.00	0.00	0.00
olfactory receptor 5AN1	LOC100758795	100758795	0.00	0.00	0.00	0.00	0.00	0.00	0.00	0.00	0.00	0.00	0.00	0.00

vasculin-like protein 1 pseudogene	LOC100758798	100758798	0.14	0.33	0.00	0.11	0.14	0.13	3.04	8.24	1.24	2.32	3.10	2.80
C-terminal-binding protein 2 pseudogene	LOC100758799	100758799	0.05	0.24	0.05	0.16	0.09	0.04	0.76	5.49	1.24	3.48	2.07	0.93
40S ribosomal protein S11-like	LOC100758800	100758800	0.00	0.00	0.00	0.00	0.00	0.00	0.00	0.00	0.00	0.00	0.00	0.00
uncharacterized LOC100758805	LOC100758805	100758805	3.54	4.42	4.67	4.17	4.99	6.77	71.40	86.01	91.91	90.40	81.76	113.10
transmembrane protease serine 11D-like	LOC100758807	100758807	0.00	0.00	0.00	0.00	0.00	0.00	0.00	0.00	0.00	0.00	0.00	0.00
transcription elongation factor B polypeptide 2	LOC100758808	100758808	0.07	0.00	0.00	0.00	0.00	0.00	0.00	0.00	0.00	0.00	0.00	0.00
lipid phosphate phosphatase-related protein type 5	LOC100758814	100758814	0.00	0.00	0.00	0.00	0.00	0.00	0.00	0.00	0.00	1.16	0.00	0.00
chromosome unknown open reading frame, human C10orf62	LOC100758819	100758819	0.35	0.32	0.28	0.14	0.18	0.23	19.75	18.30	16.15	9.27	10.35	14.02
olfactory receptor 14A2-like	LOC100758824	100758824	0.00	0.00	0.00	0.00	0.00	0.00	0.00	0.00	0.00	0.00	0.00	0.00
putative short-chain dehydrogenase/reductase family 42E member 2	LOC100758827	100758827	1.89	2.09	0.89	0.72	1.32	1.79	685.86	681.70	311.76	272.35	459.53	542.11
malignant fibrous histiocytoma-amplified sequence 1 homolog pseudogene	LOC100758829	100758829	0.02	0.00	0.00	0.00	0.02	0.02	0.76	0.00	0.00	0.00	1.03	0.93
olfactory receptor 6N2	LOC100758837	100758837	0.00	0.00	0.00	0.00	0.00	0.00	0.00	0.00	0.00	0.00	0.00	0.00
Actin-like containing protein	LOC100758838	100758838	0.00	0.00	0.00	0.00	0.00	0.00	0.00	0.00	0.00	0.00	0.00	0.00
kinesin-like protein KIF18A pseudogene	LOC100758842	100758842	6.27	5.79	6.64	6.96	10.15	9.32	341.79	324.84	397.47	419.53	557.86	524.35
zinc finger protein 14-like	LOC100758856	100758856	0.00	0.00	0.00	0.00	0.00	0.00	0.00	0.00	0.00	0.00	0.00	0.00
serine/threonine-protein kinase MARK2-like	LOC100758857	100758857	0.01	0.00	0.02	0.00	0.00	0.02	0.76	0.00	3.73	0.00	0.00	0.00
splicing factor U2AF 35 kDa subunit pseudogene	LOC100758860	100758860	39.74	41.36	35.16	36.15	38.68	37.96	1180.32	1206.01	1104.22	1021.01	1006.01	1038.42
chromosome unknown open reading frame, human C9orf89	LOC100758861	100758861	36.47	38.36	17.94	15.65	18.06	17.45	706.37	711.90	342.82	288.57	334.30	315.92

histone H2B type 1-like	LOC100 758862	100758862	0.13	0.00	0.00	0.00	0.03	0.02	0.76	0.00	0.00	0.00	0.00	0.00
proteasome maturation protein pseudogene	LOC100 758867	100758867	0.00	0.00	0.00	0.00	0.00	0.00	0.00	0.00	0.00	0.00	1.03	0.00
olfactory receptor 2Z1-like	LOC100 758870	100758870	0.03	0.03	0.04	0.23	0.13	0.05	1.52	1.83	2.48	13.91	7.24	2.80
solute carrier family 25 member 39 pseudogene	LOC100 758875	100758875	0.43	0.52	0.36	0.18	0.42	0.04	12.15	14.64	7.45	4.64	11.38	0.93
sphingolipid delta(4)-desaturase DES1	LOC100 758876	100758876	0.10	0.16	0.11	0.00	0.09	0.00	3.04	3.66	2.48	0.00	2.07	0.00
cytochrome c oxidase copper chaperone	LOC100 758878	100758878	48.94	57.98	53.56	36.21	40.05	31.31	514.21	605.75	521.68	351.15	386.05	309.38
40S ribosomal protein S2 pseudogene	LOC100 758883	100758883	0.00	0.08	0.06	0.10	0.05	0.04	1.52	2.75	1.24	3.48	1.03	0.93
heterogeneous nuclear ribonucleoprotein F-like	LOC100 758884	100758884	1.95	2.78	1.95	1.66	2.01	1.69	14.43	23.79	11.18	13.91	13.45	14.95
40S ribosomal protein S26-like	LOC100 758886	100758886	0.00	0.00	0.00	0.00	0.00	0.00	0.00	0.00	0.00	0.00	0.00	0.00
probable E3 ubiquitin-protein ligase makorin-2 pseudogene	LOC100 758890	100758890	0.11	0.14	0.02	0.08	0.07	0.00	10.63	10.07	3.73	2.32	5.17	1.87
heat shock protein HSP 90-beta pseudogene	LOC100 758894	100758894	0.00	0.00	0.00	0.00	0.00	0.00	0.00	0.00	0.00	0.00	0.00	0.00
zinc finger protein 420-like	LOC100 758905	100758905	2.08	1.90	2.25	2.66	2.75	2.48	398.00	374.25	424.79	512.24	526.81	496.31
60S ribosomal protein L23a pseudogene	LOC100 758907	100758907	0.05	0.00	0.00	0.00	0.14	0.00	0.00	0.00	0.00	0.00	1.03	0.00
olfactory receptor 5D18-like	LOC100 758908	100758908	0.00	0.00	0.00	0.00	0.00	0.00	0.00	0.00	0.00	0.00	0.00	0.00
olfactory receptor 10G9-like	LOC100 758915	100758915	0.00	0.00	0.00	0.00	0.00	0.00	0.00	0.00	0.00	0.00	0.00	0.00
vomeronasal type-2 receptor 116-like	LOC100 758916	100758916	0.00	0.00	0.00	0.00	0.00	0.00	0.00	0.00	0.00	0.00	0.00	0.00
nucleoporin SEH1 pseudogene	LOC100 758918	100758918	1.08	0.80	1.04	0.86	0.50	0.93	12.91	10.98	12.42	10.43	6.21	10.28
survival motor neuron protein pseudogene	LOC100 758922	100758922	0.00	0.00	0.00	0.00	0.00	0.00	0.00	0.00	0.00	0.00	0.00	0.00

olfactory receptor 1537-like	LOC100 758923	100758923	0.00	0.00	0.00	0.00	0.00	0.00	0.00	0.00	0.00	0.00	0.00	0.00
nucleoporin SEH1 pseudogene	LOC100 758924	100758924	0.00	0.00	0.00	0.00	0.00	0.00	0.00	0.00	0.00	0.00	0.00	0.00
zinc finger MYND domain-containing protein 19 pseudogene	LOC100 758925	100758925	0.00	0.00	0.00	0.00	0.00	0.00	0.00	0.00	0.00	0.00	0.00	0.00
lysine-specific demethylase 4D-like	LOC100 758929	100758929	0.00	0.00	0.00	0.00	0.00	0.00	0.00	0.00	0.00	0.00	0.00	0.00
chromosome unknown open reading frame, human C19orf44	LOC100 758930	100758930	6.38	5.67	6.68	5.50	5.87	5.99	431.42	376.99	469.51	380.13	387.08	405.65
resistin-like alpha	LOC100 758932	100758932	0.00	0.00	0.00	0.00	0.00	0.00	0.00	0.00	0.00	0.00	0.00	0.00
baculoviral IAP repeat-containing protein 2 pseudogene	LOC100 758933	100758933	0.00	0.00	0.00	0.00	0.00	0.00	0.00	1.83	0.00	0.00	0.00	0.00
cysteine-rich PDZ-binding protein pseudogene	LOC100 758938	100758938	0.00	0.00	0.00	0.28	0.00	0.00	0.00	0.92	0.00	1.16	0.00	0.00
small EDRK-rich factor 1	LOC100 758944	100758944	23.61	24.69	26.09	24.40	28.10	27.09	176.21	212.29	213.64	186.59	211.14	216.84
ubiquitin-40S ribosomal protein S27a pseudogene	LOC100 758945	100758945	29.90	24.11	23.01	15.91	19.91	24.98	330.40	396.21	372.63	271.19	323.95	257.03
60S ribosomal protein L21-like	LOC100 758946	100758946	0.00	0.00	0.00	0.00	0.00	0.00	0.00	0.00	0.00	0.00	0.00	0.00
60S acidic ribosomal protein P0 pseudogene	LOC100 758948	100758948	0.00	0.00	0.11	0.20	0.09	0.16	0.76	1.83	3.73	6.95	2.07	1.87
E3 ubiquitin-protein ligase HERC2	LOC100 758957	100758957	10.34	10.49	10.85	11.71	12.69	13.00	4943.84	5120.53	5282.60	5635.83	6143.68	6205.27
F-actin-capping protein subunit alpha-1 pseudogene	LOC100 758959	100758959	0.00	0.00	0.00	0.00	0.00	0.00	0.00	0.00	0.00	0.00	0.00	0.00
HLA class I histocompatibility antigen, alpha chain F-like	LOC100 758961	100758961	0.00	0.00	0.00	0.04	0.16	0.11	1.52	2.75	2.48	2.32	7.24	7.48
polycomb group RING finger protein 5 pseudogene	LOC100 758966	100758966	0.44	0.30	0.24	0.12	0.14	0.31	6.84	4.58	3.73	1.16	2.07	4.67
mitochondrial import inner membrane translocase subunit Tim23 pseudogene	LOC100 758967	100758967	0.45	0.30	0.12	0.08	0.03	0.16	5.32	0.92	1.24	1.16	0.00	1.87
GRB2-associated-binding protein 2 pseudogene	LOC100 758974	100758974	0.00	0.00	0.00	0.00	0.00	0.00	0.00	0.00	0.00	0.00	0.00	0.00

alpha-enolase pseudogene	LOC100 758979	100758979	0.61	0.89	0.76	1.15	0.40	0.37	26.58	21.96	26.08	28.97	18.63	14.95
bifunctional heparan sulfate N-deacetylase/N-sulfotransferase 3	LOC100 758983	100758983	0.00	0.00	0.00	0.00	0.00	0.00	0.00	0.00	0.00	0.00	0.00	0.00
AFG3-like protein 1	LOC100 758986	100758986	24.46	23.54	25.94	25.17	25.25	25.99	1977.08	1994.77	2126.45	2108.08	2061.69	2128.24
60S ribosomal protein L17 pseudogene	LOC100 758997	100758997	0.38	0.67	0.99	0.27	0.38	0.20	4.56	6.41	14.91	5.79	2.07	1.87
elongation factor 1-alpha 1 pseudogene	LOC100 759000	100759000	371.22	396.23	389.47	396.67	405.34	359.05	5331.97	5966.93	6425.32	7475.03	6611.49	6593.16
cytochrome b-c1 complex subunit Rieske, mitochondrial	LOC100 759002	100759002	112.32	116.37	133.83	136.41	143.40	141.09	2690.29	2769.81	3173.53	3196.30	3362.67	3301.25
solute carrier family 22 member 9-like	LOC100 759009	100759009	0.00	0.00	0.00	0.00	0.00	0.00	0.00	0.00	0.00	0.00	0.00	0.00
vesicle-associated membrane protein 3 pseudogene	LOC100 759012	100759012	0.00	0.00	0.00	0.00	0.00	0.00	0.76	0.00	0.00	0.00	0.00	0.00
alpha-enolase pseudogene	LOC100 759013	100759013	0.00	0.00	0.00	0.00	0.00	0.00	0.00	0.00	0.00	0.00	0.00	0.00
olfactory receptor 11A1	LOC100 759014	100759014	0.00	0.00	0.00	0.00	0.00	0.00	0.00	0.00	0.00	0.00	0.00	0.00
60S ribosomal protein L21 pseudogene	LOC100 759017	100759017	0.07	0.00	0.00	0.00	0.00	0.00	0.76	0.00	0.00	0.00	0.00	0.00
PRAME family member 12-like	LOC100 759019	100759019	0.00	0.00	0.00	0.00	0.00	0.00	0.00	0.00	0.00	0.00	0.00	0.00
triosephosphate isomerase pseudogene	LOC100 759023	100759023	0.00	0.00	0.00	0.00	0.00	0.00	0.00	0.00	0.00	0.00	0.00	0.00
60S acidic ribosomal protein P0 pseudogene	LOC100 759028	100759028	0.00	0.00	0.00	0.00	0.00	0.00	0.76	0.00	0.00	0.00	0.00	0.00
60S ribosomal protein L6-like	LOC100 759032	100759032	0.07	0.00	0.06	0.00	0.14	0.04	1.52	0.00	1.24	0.00	3.10	0.93
transcription factor BTF3 pseudogene	LOC100 759037	100759037	2.06	3.36	1.78	1.43	1.73	1.11	15.19	27.45	16.15	12.75	16.56	9.35
chromosome unknown open reading frame, human C19orf57	LOC100 759042	100759042	3.69	3.28	4.34	3.40	2.95	3.03	252.93	229.67	293.13	252.64	214.24	225.26
polypyrimidine tract-binding protein 1 pseudogene	LOC100 759056	100759056	0.00	0.00	0.00	0.00	0.00	0.00	0.00	0.00	0.00	0.00	0.00	0.00

olfactory receptor 4C6-like	LOC100 759057	100759057	0.00	0.00	0.00	0.00	0.00	0.00	0.00	0.00	0.00	0.00	0.00	0.00
olfactory receptor 4A15-like	LOC100 759058	100759058	0.00	0.00	0.00	0.00	0.00	0.00	0.00	0.00	0.00	0.00	0.00	0.00
olfactory receptor 1019	LOC100 759061	100759061	0.00	0.00	0.00	0.00	0.00	0.00	0.00	0.00	0.00	0.00	0.00	0.00
elongation factor 1-beta pseudogene	LOC100 759066	100759066	0.18	0.14	0.00	0.00	0.08	0.07	2.28	2.75	1.24	1.16	1.03	0.93
60S ribosomal protein L18a pseudogene	LOC100 759068	100759068	0.00	0.00	0.00	0.00	0.00	0.00	0.00	0.00	0.00	0.00	0.00	0.00
probable UDP-sugar transporter protein SLC35A4	LOC100 759071	100759071	1.96	2.14	2.25	1.92	1.63	2.02	47.09	44.84	50.93	39.40	39.33	51.41
taste receptor type 1 member 1	LOC100 759079	100759079	2.16	2.16	1.60	1.55	1.50	1.31	362.30	368.76	272.02	274.66	278.41	287.88
olfactory receptor 4F4-like	LOC100 759083	100759083	0.00	0.00	0.00	0.00	0.00	0.00	0.00	0.00	0.00	0.00	0.00	0.00
E3 ubiquitin-protein ligase RAD18	LOC100 759084	100759084	2.89	2.92	2.76	3.49	4.31	3.76	110.89	129.02	106.82	152.98	186.30	142.07
GRB2-associated-binding protein 2 pseudogene	LOC100 759089	100759089	0.00	0.00	0.00	0.00	0.05	0.00	0.76	0.00	0.00	0.00	0.00	0.00
acidic leucine-rich nuclear phosphoprotein 32 family member B pseudogene	LOC100 759093	100759093	10.99	12.09	9.72	9.33	10.24	9.08	243.05	278.17	217.37	208.61	224.59	202.82
uncharacterized LOC100759095	LOC100 759095	100759095	4.90	7.06	1.76	2.00	6.03	4.62	66.08	74.12	36.02	40.56	64.17	61.69
arylacetamide deacetylase-like 4	LOC100 759096	100759096	0.00	0.00	0.00	0.00	0.00	0.00	0.00	0.00	0.00	0.00	0.00	0.00
glyceraldehyde-3-phosphate dehydrogenase-like	LOC100 759100	100759100	1.12	1.25	1.18	0.45	0.75	0.62	89.63	75.95	72.04	45.20	44.50	46.73
N-alpha-acetyltransferase 35, NatC auxiliary subunit pseudogene	LOC100 759103	100759103	0.07	0.08	0.05	0.11	0.10	0.05	4.56	3.66	2.48	5.79	5.17	2.80
Ig heavy chain V-III region VH26-like	LOC100 759105	100759105	0.00	0.00	0.00	0.00	0.00	0.00						
nuclear receptor-binding factor 2 pseudogene	LOC100 759106	100759106	0.00	0.02	0.08	0.08	0.04	0.00	0.00	0.00	1.24	1.16	0.00	0.00
glutathione S-transferase omega-2	LOC100 759107	100759107	0.00	0.00	0.00	0.00	0.00	0.00	0.00	0.00	0.00	0.00	0.00	0.00

membrane-spanning 4-domains subfamily A member 4A-like	LOC100 759112	100759112	0.00	0.00	0.00	0.00	0.00	0.00	0.00	0.00	0.00	0.00	0.00	0.00
olfactory receptor 14A2-like	LOC100 759115	100759115	0.00	0.00	0.00	0.00	0.00	0.00	0.00	0.00	0.00	0.00	0.00	0.00
homeobox protein Hox-D11-like	LOC100 759116	100759116	0.00	0.00	0.00	0.00	0.00	0.00	0.00	0.00	0.00	0.00	0.00	0.00
olfactory receptor 2A1/2A42	LOC100 759118	100759118	0.00	0.00	0.00	0.00	0.00	0.00	0.00	0.00	0.00	0.00	0.00	0.00
	LOC100 759119	100759119	0.00	0.00	0.00	0.00	0.00	0.00	0.00	0.00	0.00	0.00	0.00	0.00
nucleoporin SEH1-like	LOC100 759121	100759121	0.13	0.10	0.05	0.02	0.08	0.07	0.00	0.00	0.00	0.00	0.00	0.00
mitochondrial import inner membrane translocase subunit Tim23 pseudogene	LOC100 759125	100759125	0.80	1.01	0.64	0.75	0.67	0.53	18.23	21.05	14.91	13.91	13.45	12.15
TATA box-binding protein-associated factor RNA polymerase I subunit B pseudogene	LOC100 759127	100759127	0.12	0.05	0.17	0.14	0.00	0.02	6.08	2.75	8.69	6.95	0.00	0.00
olfactory receptor 6K6	LOC100 759128	100759128	0.00	0.00	0.00	0.00	0.00	0.00	0.00	0.00	0.00	0.00	0.00	0.00
keratin-associated protein 10-4	LOC100 759130	100759130	0.00	0.00	0.00	0.00	0.00	0.00	0.00	0.00	0.00	0.00	0.00	0.00
eukaryotic translation elongation factor 1 epsilon-1 pseudogene	LOC100 759131	100759131	0.63	0.53	0.88	0.25	0.22	0.12	9.11	9.15	11.18	2.32	3.10	1.87
nucleophosmin-like	LOC100 759134	100759134	0.00	0.05	0.00	0.00	0.06	0.11	0.00	0.00	0.00	0.00	1.03	0.93
putative spermatogenesis-associated protein 31D3	LOC100 759146	100759146	0.00	0.00	0.00	0.00	0.00	0.00	0.00	0.00	0.00	0.00	0.00	0.00
nucleophosmin pseudogene	LOC100 759149	100759149	0.11	0.04	0.18	0.00	0.00	0.09	3.04	0.92	4.97	0.00	1.03	6.54
histone H2A type 1-F	LOC100 759151	100759151	0.00	0.00	0.00	0.00	0.00	0.00	0.00	0.00	0.00	0.00	0.00	0.00
60S ribosomal protein L21	LOC100 759156	100759156	16.19	12.37	11.58	11.19	13.35	10.57	130.64	94.25	81.98	79.97	98.32	80.38
aldehyde oxidase 2	LOC100 759157	100759157	0.01	0.03	0.01	0.00	0.00	0.01	3.04	5.49	1.24	0.00	1.03	3.74
40S ribosomal protein S19-like	LOC100 759160	100759160	0.20	0.12	0.16	0.00	0.55	0.25	1.52	0.92	1.24	0.00	4.14	1.87

40S ribosomal protein S2 pseudogene	LOC100759167	100759167	0.00	0.00	0.00	0.00	0.00	0.00	0.00	0.00	0.00	0.00	0.00	0.00
zinc finger protein 420-like	LOC100759173	100759173	3.10	2.94	4.17	3.88	4.56	3.65	189.88	178.43	249.66	250.33	285.66	234.60
contactin-1	LOC100759179	100759179	4.36	4.67	0.23	0.33	0.00	0.00	286.35	269.93	14.91	16.22	0.00	0.00
protein FAM187B-like	LOC100759185	100759185	0.00	0.00	0.00	0.00	0.00	0.00	0.00	0.00	0.00	0.00	0.00	0.00
glyceraldehyde-3-phosphate dehydrogenase pseudogene	LOC100759189	100759189	1.81	1.60	1.09	0.68	1.03	1.01	37.98	31.11	17.39	15.07	19.66	18.69
N-alpha-acetyltransferase 50 pseudogene	LOC100759192	100759192	0.31	0.66	0.26	0.12	0.43	0.15	3.04	6.41	2.48	1.16	4.14	0.93
olfactory receptor 143-like	LOC100759196	100759196	0.00	0.00	0.00	0.00	0.00	0.00	0.00	0.00	0.00	0.00	0.00	0.00
olfactory receptor 5L1	LOC100759198	100759198	0.00	0.00	0.00	0.00	0.00	0.00	0.00	0.00	0.00	0.00	0.00	0.00
sperm motility kinase	LOC100759199	100759199	0.00	0.00	0.00	0.00	0.00	0.00	0.76	0.00	0.00	0.00	0.00	0.00
glutamine synthetase-like	LOC100759200	100759200	0.00	0.00	0.00	0.04	0.00	0.00	0.00	0.00	0.00	1.16	0.00	0.00
proteasome subunit alpha type-6 pseudogene	LOC100759202	100759202	2.91	2.81	3.46	3.10	3.96	4.13	116.97	85.10	115.51	115.89	141.79	158.89
olfactory receptor 4D5	LOC100759204	100759204	0.00	0.00	0.00	0.00	0.00	0.00	0.00	0.00	0.00	0.00	0.00	0.00
Ig heavy chain V-III region VH26-like	LOC100759209	100759209	0.00	0.00	0.00	0.00	0.00	0.00	0.00	0.00	0.00	0.00	0.00	0.00
peptidyl-prolyl cis-trans isomerase A-like	LOC100759211	100759211	0.00	0.00	0.00	0.00	0.00	0.10	3.04	6.41	7.45	4.64	0.00	5.61
NUT family member Gm806-like	LOC100759212	100759212	0.00	0.00	0.00	0.00	0.00	0.00	0.00	0.00	0.00	0.00	0.00	0.00
TP53-regulated inhibitor of apoptosis 1 pseudogene	LOC100759213	100759213	0.00	0.00	0.00	0.00	0.00	0.00	0.00	0.00	0.00	0.00	0.00	0.00
mitochondrial carnitine/acylcarnitine carrier protein pseudogene	LOC100759215	100759215	0.00	0.08	0.21	0.00	0.00	0.00	0.00	0.92	2.48	0.00	0.00	0.00
keratin, type I cytoskeletal 27	LOC100759216	100759216	0.00	0.00	0.00	0.00	0.00	0.00	0.00	0.00	0.00	0.00	0.00	0.00

vomeronasal type-1 receptor 4-like	LOC100759218	100759218	0.00	0.00	0.00	0.00	0.00	0.00	0.00	0.00	0.00	0.00	0.00	0.00
resistin-like alpha	LOC100759219	100759219	0.00	0.00	0.00	0.00	0.00	0.00	0.00	0.00	0.00	0.00	0.00	0.00
60S ribosomal protein L12-like	LOC100759223	100759223	235.27	227.39	200.98	120.42	73.76	75.11	2928.78	2824.71	2492.87	1435.90	864.21	893.54
bromo adjacent homology domain-containing 1 protein-like	LOC100759233	100759233	1.43	1.40	1.02	0.78	0.63	0.51	3.80	7.32	4.97	3.48	2.07	2.80
mitochondrial import inner membrane translocase subunit Tim13 pseudogene	LOC100759234	100759234	97.74	116.41	95.98	31.47	9.10	10.58	139.00	163.79	121.72	34.77	11.38	12.15
cytochrome P450 1B1	LOC100759241	100759241	0.00	0.00	0.00	0.00	0.00	0.00	0.00	0.00	0.00	0.00	0.00	0.00
H-2 class I histocompatibility antigen, Q10 alpha chain-like	LOC100759246	100759246	5.55	5.55	7.22	6.26	6.31	6.41	311.41	330.33	419.83	360.42	378.80	381.35
putative 60S ribosomal protein L37a	LOC100759247	100759247	10.36	13.72	10.04	10.07	10.19	6.85	53.17	64.05	47.20	46.36	45.54	27.11
60S ribosomal protein L23a pseudogene	LOC100759249	100759249	0.00	0.00	0.00	0.00	0.00	0.00	0.00	0.00	0.00	0.00	0.00	0.00
ubiquitin-conjugating enzyme E2 J2 pseudogene	LOC100759251	100759251	2.98	2.76	2.36	2.36	1.93	2.18	23.55	21.05	16.15	5.79	8.28	10.28
60S ribosomal protein L10-like	LOC100759252	100759252	0.56	0.57	0.22	0.12	0.29	0.16	0.76	0.92	1.24	0.00	0.00	0.93
metal transporter CNNM2-like	LOC100759256	100759256	3.45	3.89	3.23	2.92	2.04	1.85	144.31	153.73	130.42	120.53	83.83	84.12
platelet-activating factor acetylhydrolase IB subunit beta-like	LOC100759264	100759264	0.52	0.77	0.53	0.62	0.41	0.57	26.58	33.86	26.08	27.81	23.80	24.30
nucleophosmin pseudogene	LOC100759270	100759270	0.04	0.07	0.14	0.07	0.00	0.00	0.76	0.92	2.48	2.32	0.00	0.00
uncharacterized LOC100759275	LOC100759275	100759275	0.00	0.00	0.00	0.00	0.00	0.00	0.00	0.00	0.00	0.00	0.00	0.00
cytochrome c oxidase subunit 7A-related protein, mitochondrial	LOC100759281	100759281	111.14	116.36	127.57	121.77	126.16	126.90	3875.93	4086.54	4474.00	4231.22	4363.50	4401.36
Ig lambda chain V-VI region SUT-like	LOC100759284	100759284	0.00	0.00	0.00	0.00	0.00	0.00						
vomeronasal type-2 receptor 1-like	LOC100759301	100759301	0.00	0.00	0.00	0.00	0.00	0.00	0.00	0.92	0.00	0.00	0.00	0.00

dnaJ homolog subfamily C member 14 pseudogene	LOC100759304	100759304	0.00	0.00	0.00	0.00	0.03	0.02	0.00	0.00	0.00	0.00	1.03	0.93
UDP-glucose 4-epimerase	LOC100759306	100759306	6.42	5.20	7.01	6.43	6.37	5.61	55.45	43.01	77.01	50.99	57.96	51.41
ran-specific GTPase-activating protein pseudogene	LOC100759308	100759308	0.00	0.00	0.00	0.00	0.00	0.00	0.00	0.00	0.00	0.00	0.00	0.00
histone H2A.Z pseudogene	LOC100759313	100759313	0.56	0.97	0.77	0.30	0.35	0.23	2.28	7.32	3.73	1.16	3.10	0.00
opioid growth factor receptor pseudogene	LOC100759317	100759317	0.09	0.10	0.12	0.06	0.02	0.09	3.80	2.75	4.97	2.32	1.03	3.74
BPI fold-containing family B member 3-like	LOC100759326	100759326	0.00	0.00	0.00	0.00	0.00	0.00	0.00	0.00	0.00	0.00	0.00	0.00
teashirt homolog 3-like	LOC100759329	100759329	0.00	0.00	0.00	0.00	0.00	0.00	0.00	0.00	0.00	0.00	0.00	0.00
beclin-2-like	LOC100759331	100759331	0.00	0.00	0.00	0.00	0.00	0.00	0.00	0.00	0.00	0.00	0.00	0.00
histone H3.3-like	LOC100759332	100759332	2.76	5.29	4.34	5.53	3.78	4.16	18.99	32.94	17.39	32.45	8.28	25.24
glyceraldehyde-3-phosphate dehydrogenase pseudogene	LOC100759335	100759335	0.06	0.00	0.00	0.00	0.04	0.00	1.52	0.00	0.00	0.00	1.03	0.00
transmembrane protein 45A-like	LOC100759339	100759339	0.00	0.00	0.00	0.00	0.00	0.00	0.00	0.00	0.00	0.00	0.00	0.00
olfactory receptor 4C6-like	LOC100759340	100759340	0.00	0.00	0.00	0.00	0.00	0.00	0.00	0.00	0.00	0.00	0.00	0.00
olfactory receptor 4A16	LOC100759341	100759341	0.00	0.00	0.00	0.00	0.00	0.00	0.00	0.00	0.00	0.00	0.00	0.00
HLA class II histocompatibility antigen, DP beta 1 chain-like	LOC100759342	100759342	0.00	0.00	0.00	0.00	0.00	0.00	0.76	0.92	0.00	0.00	0.00	0.00
olfactory receptor 1020	LOC100759344	100759344	0.03	0.00	0.00	0.00	0.00	0.00	0.76	0.00	0.00	0.00	0.00	0.00
60S ribosomal protein L15 pseudogene	LOC100759354	100759354	0.03	0.00	0.10	0.05	0.04	0.00	2.28	0.00	2.48	1.16	1.03	0.00
olfactory receptor 2L13-like	LOC100759355	100759355	0.00	0.00	0.00	0.00	0.00	0.00	0.00	0.00	0.00	0.00	0.00	0.00
24-hydroxycholesterol 7-alpha-hydroxylase	LOC100759356	100759356	2.74	2.66	2.96	3.38	4.21	3.94	210.39	213.20	232.27	257.28	314.64	303.77

immunoglobulin omega chain-like	LOC100759362	100759362	0.00	0.00	0.00	0.00	0.00	0.00	0.00	0.00	0.00	0.00	0.00	0.00
olfactory receptor 4K15-like	LOC100759363	100759363	0.00	0.00	0.00	0.00	0.00	0.00	0.00	0.00	0.00	0.00	0.00	0.00
olfactory receptor 5A1	LOC100759364	100759364	0.00	0.00	0.00	0.00	0.00	0.00	0.00	0.00	0.00	0.00	0.00	0.00
putative olfactory receptor ENSP00000348552	LOC100759366	100759366	0.00	0.00	0.00	0.00	0.00	0.00	0.00	0.00	0.00	0.00	0.00	0.00
60S ribosomal protein L23 pseudogene	LOC100759371	100759371	0.83	1.00	0.41	0.00	0.35	0.00	7.60	10.07	3.73	0.00	3.10	0.00
arylacetamide deacetylase-like 4	LOC100759379	100759379	0.00	0.00	0.00	0.00	0.00	0.00	0.00	0.00	0.00	0.00	0.00	0.00
putative SMEK homolog 3	LOC100759383	100759383	0.02	0.01	0.04	0.02	0.00	0.00	1.52	0.92	2.48	1.16	0.00	0.00
zinc finger protein 420-like	LOC100759388	100759388	2.69	2.38	3.17	3.12	3.66	3.49	230.14	213.20	264.56	263.07	296.01	297.22
40S ribosomal protein S14 pseudogene	LOC100759391	100759391	1.83	1.88	1.61	1.42	1.14	1.12	21.27	21.05	17.39	15.07	12.42	11.22
transmembrane protein 127 pseudogene	LOC100759396	100759396	0.14	0.12	0.00	0.07	0.31	0.03	1.52	0.00	0.00	0.00	2.07	0.00
membrane-spanning 4-domains subfamily A member 4D-like	LOC100759397	100759397	0.00	0.00	0.00	0.00	0.00	0.00	0.00	0.00	0.00	0.00	0.00	0.00
olfactory receptor 14A2-like	LOC100759400	100759400	0.00	0.00	0.00	0.00	0.00	0.00	0.00	0.00	0.00	0.00	0.00	0.00
olfactory receptor 10A7-like	LOC100759403	100759403	0.00	0.00	0.00	0.00	0.00	0.00	0.00	0.00	0.00	0.00	0.00	0.00
olfactory receptor 2T29-like	LOC100759404	100759404	0.00	0.00	0.00	0.00	0.00	0.00	0.00	0.00	0.00	0.00	0.00	0.00
dolichyl-diphosphooligosaccharide--protein glycosyltransferase subunit 4	LOC100759406	100759406	5.45	4.20	3.66	4.65	5.40	5.47	18.23	15.56	12.42	19.70	17.59	20.56
interferon alpha-12-like	LOC100759411	100759411	0.00	0.00	0.00	0.00	0.00	0.00	0.00	0.00	0.00	0.00	0.00	0.00
protein FAM104A-like	LOC100759413	100759413	8.48	7.65	4.48	4.34	4.63	4.32	426.10	432.81	250.90	249.17	274.27	265.45
chromosome unknown open reading frame, human C19orf70	LOC100759422	100759422	94.87	90.88	88.64	80.26	67.75	81.59	1483.38	1421.96	1408.53	1207.59	1074.32	1272.09

Fc receptor-like protein 2	LOC100759428	100759428	0.00	0.00	0.06	0.12	0.00	0.00	0.00	0.00	2.48	5.79	0.00	0.00
60S ribosomal protein L27a-like	LOC100759430	100759430	2.00	2.35	2.18	0.86	1.83	1.29	13.67	21.05	17.39	5.79	14.49	9.35
sperm motility kinase X-like	LOC100759433	100759433	0.00	0.00	0.00	0.00	0.00	0.00	0.00	0.00	0.00	0.00	0.00	0.00
galectin-1 pseudogene	LOC100759436	100759436	0.76	1.27	1.05	1.01	0.94	0.75	6.84	11.90	7.45	8.11	8.28	6.54
uncharacterized LOC100759437	LOC100759437	100759437	0.00	0.01	0.00	0.00	0.00	0.00	0.00	0.00	0.00	0.00	0.00	0.00
leucine-rich repeat and IQ domain-containing protein 1	LOC100759441	100759441	0.00	0.03	0.00	0.02	0.00	0.00	0.00	1.83	0.00	1.16	0.00	0.00
calcyclin-binding protein pseudogene	LOC100759444	100759444	0.63	1.13	0.58	0.24	0.46	0.32	26.58	31.11	28.57	15.07	17.59	14.95
39S ribosomal protein L1, mitochondrial-like	LOC100759447	100759447	0.74	0.42	0.31	0.45	0.36	0.31	10.63	9.15	4.97	8.11	5.17	3.74
SEC14-like protein 4	LOC100759451	100759451	0.00	0.00	0.00	0.00	0.00	0.00	0.00	0.00	0.00	0.00	0.00	0.00
zinc finger protein 420-like	LOC100759458	100759458	0.15	0.13	0.12	0.08	0.17	0.15	16.71	14.64	11.18	8.11	14.49	14.02
chromosome unknown open reading frame, human C11orf85	LOC100759463	100759463	0.00	0.00	0.00	0.00	0.00	0.00	0.76	0.92	0.00	0.00	1.03	0.00
eukaryotic translation initiation factor 4E pseudogene	LOC100759468	100759468	22.71	25.22	19.59	19.71	20.95	20.75	518.01	625.88	476.96	489.06	526.81	500.98
60S ribosomal protein L10-like	LOC100759472	100759472	7.47	8.63	6.12	4.94	5.96	4.03	98.74	115.29	74.53	62.58	74.52	54.21
nucleoplasmin-3 pseudogene	LOC100759480	100759480	0.06	0.00	0.19	0.00	0.00	0.15	0.76	0.92	4.97	1.16	1.03	2.80
olfactory receptor 145	LOC100759481	100759481	0.00	0.00	0.00	0.00	0.00	0.00	0.00	0.00	0.00	0.00	0.00	0.00
olfactory receptor 13F1	LOC100759482	100759482	0.00	0.00	0.00	0.00	0.00	0.00	0.00	0.00	0.00	0.00	0.00	0.00
olfactory receptor 5D18	LOC100759483	100759483	0.00	0.00	0.00	0.00	0.00	0.00	0.00	0.00	0.00	0.00	0.00	0.00
small ubiquitin-related modifier 2	LOC100759487	100759487	26.58	34.49	37.89	36.48	39.12	22.29	274.19	311.11	315.49	254.96	235.98	154.22

olfactory receptor 10G9	LOC100759491	100759491	0.00	0.00	0.00	0.00	0.00	0.00	0.00	0.00	0.00	0.00	0.00	0.00
cytochrome P450 2B1-like	LOC100759494	100759494	0.00	0.00	0.00	0.00	0.00	0.00	0.00	0.00	0.00	0.00	0.00	0.00
uncharacterized LOC100759496	LOC100759496	100759496	0.00	0.00	0.00	0.00	0.00	0.00	0.00	0.00	0.00	0.00	0.00	0.00
ribosomal RNA-processing protein 7 homolog A pseudogene	LOC100759497	100759497	0.00	0.00	0.00	0.00	0.00	0.00	0.00	0.00	0.00	0.00	0.00	0.00
ubiquitin-conjugating enzyme E2 S pseudogene	LOC100759498	100759498	0.00	0.00	0.00	0.00	0.00	0.00	0.00	0.00	0.00	0.00	0.00	0.00
ribosomal RNA-processing protein 7 homolog A pseudogene	LOC100759499	100759499	0.13	0.04	0.08	0.02	0.05	0.00	1.52	0.00	1.24	0.00	1.03	0.00
PRAME family member 12-like	LOC100759500	100759500	0.00	0.00	0.00	0.00	0.00	0.00	0.00	0.00	0.00	0.00	0.00	0.93
putative tripartite motif-containing protein 43C	LOC100759504	100759504	0.00	0.00	0.00	0.00	0.00	0.00	0.00	0.00	0.00	0.00	0.00	0.00
T-cell receptor-associated transmembrane adapter 1	LOC100759508	100759508	0.00	0.00	0.00	0.00	0.00	0.00	0.00	0.00	0.00	0.00	0.00	0.00
60S ribosomal protein L23a pseudogene	LOC100759523	100759523	0.00	0.00	0.00	0.00	0.00	0.00	0.00	0.00	0.00	0.00	0.00	0.00
prohibitin-2-like	LOC100759526	100759526	0.11	0.00	0.06	0.05	0.00	0.09	2.28	0.92	2.48	1.16	0.00	1.87
ribonuclease P protein subunit p21	LOC100759534	100759534	5.33	5.20	4.74	1.94	1.25	1.02	169.38	168.37	135.39	54.47	16.56	15.89
agmatinase, mitochondrial-like	LOC100759536	100759536	0.00	0.00	0.00	0.00	0.00	0.00	0.00	0.00	0.00	0.00	0.00	0.00
DNA-binding protein inhibitor ID-2 pseudogene	LOC100759537	100759537	0.00	0.00	0.00	0.00	0.00	0.00	0.00	0.00	0.00	0.00	0.00	0.00
40S ribosomal protein S6 pseudogene	LOC100759539	100759539	0.10	0.23	0.20	0.04	0.20	0.09	3.04	7.32	6.21	1.16	6.21	2.80
liver carboxylesterase-like	LOC100759544	100759544	0.00	0.00	0.00	0.00	0.00	0.00	0.00	0.00	0.00	0.00	0.00	0.00
cytochrome c oxidase assembly factor 4 homolog, mitochondrial	LOC100759549	100759549	24.44	26.77	23.64	19.41	20.73	18.97	627.38	696.34	627.25	521.51	570.28	521.55
Ig lambda chain V-1 region	LOC100759552	100759552	0.00	0.00	0.00	0.00	0.00	0.00	0.00	0.00	0.00	0.00	0.00	0.00

HD domain-containing protein 2 pseudogene	LOC100759553	100759553	0.00	0.00	0.00	0.00	0.00	0.00	0.00	0.00	0.00	0.00	0.00	0.00
contactin-associated protein-like 3	LOC100759554	100759554	0.00	0.00	0.00	0.00	0.00	0.00	0.00	0.00	0.00	0.00	0.00	0.00
heat shock cognate 71 kDa protein pseudogene	LOC100759556	100759556	15.11	15.38	17.24	16.30	13.10	14.49	330.40	296.47	295.62	308.27	219.42	329.94
putative olfactory receptor 51H1	LOC100759558	100759558	0.00	0.00	0.00	0.00	0.00	0.00	0.00	0.00	0.00	0.00	0.00	0.00
chromosome unknown open reading frame, human C4orf17	LOC100759564	100759564	0.00	0.00	0.00	0.00	0.00	0.00	0.76	0.00	0.00	1.16	1.03	0.93
serine/threonine-protein phosphatase 2A 55 kDa regulatory subunit B alpha isoform pseudogene	LOC100759568	100759568	0.00	0.00	0.00	0.00	0.00	0.00	0.00	0.00	0.00	0.00	0.00	0.00
COMM domain-containing protein 7 pseudogene	LOC100759581	100759581	2.00	1.52	1.83	1.26	0.75	0.96	9.87	6.41	7.45	2.32	3.10	4.67
vomeronasal type-2 receptor 1-like	LOC100759584	100759584	0.00	0.00	0.00	0.00	0.00	0.00	0.00	0.00	0.00	0.00	0.00	0.00
GRB2-associated-binding protein 2 pseudogene	LOC100759586	100759586	0.21	0.17	0.34	0.08	0.22	0.21	6.08	4.58	11.18	1.16	5.17	7.48
ubiquitin-conjugating enzyme E2 variant 1-like	LOC100759587	100759587	0.09	0.00	0.14	0.24	0.00	0.00	0.00	0.92	2.48	3.48	1.03	0.93
vomeronasal type-1 receptor 4-like	LOC100759589	100759589	0.00	0.00	0.00	0.00	0.00	0.00	0.00	0.00	0.00	0.00	0.00	0.00
40S ribosomal protein SA pseudogene	LOC100759591	100759591	0.44	0.41	0.36	0.05	0.00	0.08	9.87	9.15	8.69	1.16	0.00	2.80
calcium-binding and coiled-coil domain-containing protein 2 pseudogene	LOC100759592	100759592	0.00	0.00	0.00	0.00	0.00	0.00	0.00	0.00	0.00	0.00	0.00	0.00
alcohol dehydrogenase class-3 pseudogene	LOC100759594	100759594	0.00	0.00	0.00	0.00	0.00	0.00	0.00	0.00	0.00	0.00	0.00	0.00
eukaryotic translation initiation factor 3 subunit A pseudogene	LOC100759598	100759598	10.99	11.27	9.77	9.97	6.53	6.85	159.50	159.22	146.57	142.55	78.66	117.77
taste receptor type 2 member 135	LOC100759618	100759618	0.00	0.00	0.00	0.00	0.00	0.00	0.00	0.00	0.00	0.00	0.00	0.00
olfactory receptor 4C11-like	LOC100759622	100759622	0.00	0.00	0.00	0.00	0.00	0.00	0.00	0.92	0.00	0.00	0.00	0.00
olfactory receptor 140-like	LOC100759623	100759623	0.00	0.00	0.00	0.00	0.00	0.00	0.00	0.00	0.00	0.00	0.00	0.00

gastric triacylglycerol lipase-like	LOC100759625	100759625	0.00	0.00	0.00	0.00	0.00	0.00	0.00	0.00	0.00	0.00	0.00	0.00
olfactory receptor 5M10-like	LOC100759626	100759626	0.00	0.00	0.00	0.00	0.00	0.00	0.00	0.00	0.00	0.00	0.00	0.00
putative uncharacterized protein FLJ22184	LOC100759627	100759627	0.01	0.00	0.00	0.01	0.02	0.02	0.76	0.00	0.00	1.16	2.07	2.80
glyceraldehyde-3-phosphate dehydrogenase pseudogene	LOC100759637	100759637	0.00	0.00	0.00	0.00	0.00	0.00	0.00	0.00	0.00	0.00	0.00	0.00
high mobility group protein B1 pseudogene	LOC100759639	100759639	0.10	0.08	0.09	0.12	0.22	0.16	1.52	2.75	1.24	1.16	3.10	2.80
lamina-associated polypeptide 2, isoform beta	LOC100759640	100759640	125.47	129.53	145.51	149.52	160.11	151.77	2907.52	3050.72	3379.72	3477.92	3667.99	3502.21
olfactory receptor 2M3-like	LOC100759650	100759650	0.00	0.00	0.00	0.00	0.00	0.00	0.00	0.00	0.00	0.00	0.00	0.00
olfactory receptor 4D6	LOC100759652	100759652	0.00	0.00	0.00	0.00	0.00	0.00	0.00	0.00	0.00	0.00	0.00	0.00
putative olfactory receptor ENSP00000348552	LOC100759654	100759654	0.00	0.01	0.00	0.00	0.00	0.00	0.00	0.00	0.00	0.00	0.00	0.00
putative ATP-dependent RNA helicase PI10	LOC100759657	100759657	0.00	0.04	0.00	0.00	0.03	0.00	2.28	1.83	0.00	0.00	1.03	0.00
NADH dehydrogenase [ubiquinone] 1 subunit C2 pseudogene	LOC100759660	100759660	0.27	0.33	0.00	0.45	0.19	0.00	2.28	2.75	1.24	2.32	1.03	0.93
acyl-protein thioesterase 1 pseudogene	LOC100759661	100759661	0.00	0.06	0.00	0.00	0.00	0.00	0.00	0.92	0.00	0.00	0.00	0.00
transcription initiation factor TFIID subunit 9-like	LOC100759662	100759662	0.00	0.00	0.00	0.00	0.00	0.00	0.00	0.00	0.00	0.00	0.00	0.00
histone H3.3-like	LOC100759664	100759664	1.72	2.62	2.46	1.59	2.15	1.72	9.11	10.07	9.94	8.11	8.28	8.41
40S ribosomal protein S15a pseudogene	LOC100759669	100759669	5.01	3.49	3.57	3.44	2.60	3.70	28.10	16.47	18.63	13.91	16.56	22.43
olfactory receptor 51E2	LOC100759672	100759672	0.00	0.00	0.00	0.00	0.00	0.00	0.00	0.00	0.00	0.00	0.00	0.00
60S ribosomal protein L7-like	LOC100759675	100759675	10.25	6.81	6.38	6.08	7.23	7.24	175.45	118.95	89.43	75.33	97.29	73.84
poly(A) polymerase alpha-like	LOC100759677	100759677	0.57	0.48	0.37	0.23	0.35	0.31	17.47	14.64	12.42	9.27	14.49	6.54

60S ribosomal protein L23-like	LOC100759678	100759678	0.48	0.06	0.18	0.17	0.38	0.41	2.28	0.92	1.24	1.16	2.07	1.87
von Willebrand factor A domain-containing protein 5A-like	LOC100759681	100759681	13.61	15.04	11.38	12.96	23.53	23.06	994.24	1099.87	823.50	916.71	1670.47	1626.33
interferon-induced guanylate-binding protein 1-like	LOC100759682	100759682	0.03	0.05	0.04	0.00	0.00	0.00	1.52	3.66	2.48	0.00	0.00	0.00
microtubule-associated proteins 1A/1B light chain 3B pseudogene	LOC100759686	100759686	0.00	0.00	0.00	0.00	0.00	0.00	0.00	0.00	0.00	0.00	0.00	0.00
inhibitor of growth protein 1-like	LOC100759689	100759689	0.92	0.81	0.64	0.84	0.99	1.03	4.56	1.83	1.24	3.48	5.17	10.28
speckle-type POZ protein pseudogene	LOC100759690	100759690	0.04	0.00	0.05	0.22	0.08	0.14	2.28	0.00	1.24	5.79	2.07	2.80
26S proteasome non-ATPase regulatory subunit 3	LOC100759692	100759692	546.14	538.68	420.52	347.58	320.44	338.41	6071.76	6040.14	4750.98	4184.86	3773.56	3901.31
60S ribosomal protein L7a-like	LOC100759693	100759693	13.56	15.33	13.92	14.57	11.07	9.72	112.41	98.82	83.22	103.14	55.89	74.77
THO complex subunit 3-like	LOC100759697	100759697	0.21	0.11	0.23	0.00	0.22	0.04	4.56	1.83	3.73	0.00	4.14	1.87
intelectin-1a-like	LOC100759699	100759699	0.01	0.00	0.00	0.00	0.00	0.00	0.00	0.00	0.00	0.00	0.00	0.00
keratin-associated protein 10-2-like	LOC100759701	100759701	0.00	0.00	0.00	0.00	0.00	0.00	0.00	0.00	0.00	0.00	0.00	0.00
polyadenylate-binding protein 1 pseudogene	LOC100759704	100759704	4.08	3.78	3.54	3.46	3.92	3.08	43.29	51.24	48.44	40.56	46.57	28.97
protein FRG1 pseudogene	LOC100759708	100759708	1.45	1.45	1.21	1.47	2.38	2.59	40.26	37.52	33.54	39.40	64.17	68.23
hyaluronidase-like	LOC100759710	100759710	0.00	0.00	0.00	0.00	0.00	0.00	0.00	0.00	0.00	0.00	0.00	0.00
40S ribosomal protein S5 pseudogene	LOC100759715	100759715	1.09	1.28	1.12	0.28	0.25	0.22	476.99	344.97	360.21	114.73	66.24	68.23
tubulin alpha-1 chain-like	LOC100759721	100759721	0.58	0.42	0.38	0.35	0.28	0.33	5.32	1.83	3.73	3.48	2.07	1.87
vesicle-associated membrane protein-associated protein A-like	LOC100759732	100759732	0.00	0.00	0.00	0.00	0.00	0.00	0.00	0.00	0.00	0.00	0.00	0.00
basic leucine zipper and W2 domain-containing protein 2 pseudogene	LOC100759734	100759734	0.10	0.14	0.25	0.09	0.16	0.07	4.56	5.49	9.94	3.48	7.24	2.80

filaggrin-like	LOC100 759735	100759735	0.00	0.00	0.00	0.00	0.00	0.00	0.00	0.00	0.00	0.00	0.00	0.00
cation transport regulator-like protein 2 pseudogene	LOC100 759736	100759736	0.00	0.00	0.00	0.00	0.00	0.00	0.00	0.00	0.00	0.00	0.00	0.00
40S ribosomal protein S2-like	LOC100 759737	100759737	0.00	0.00	0.00	0.00	0.00	0.00	0.00	0.00	0.00	0.00	0.00	0.00
interferon alpha-12	LOC100 759741	100759741	0.00	0.00	0.00	0.00	0.00	0.00	0.00	0.00	0.00	0.00	0.00	0.00
60S ribosomal protein L8 pseudogene	LOC100 759742	100759742	0.75	1.06	0.72	0.34	0.55	0.00	12.91	18.30	12.42	5.79	9.31	0.00
60S ribosomal protein L36a-like	LOC100 759744	100759744	0.02	0.00	0.00	0.00	0.00	0.00	0.00	0.00	0.00	0.00	0.00	0.00
caspase-12-like	LOC100 759746	100759746	3.80	3.88	2.95	2.57	3.74	3.79	268.88	267.19	197.49	173.84	248.40	263.58
zinc finger protein 501-like	LOC100 759748	100759748	0.03	0.02	0.00	0.00	0.04	0.00	3.04	1.83	2.48	2.32	7.24	5.61
lymphocyte antigen 6A-2/6E-1-like	LOC100 759749	100759749	0.00	0.00	0.00	0.00	0.00	0.00	0.00	0.00	0.00	0.00	0.00	0.00
cytochrome c oxidase subunit 7B, mitochondrial pseudogene	LOC100 759754	100759754	0.00	0.44	0.00	0.00	0.00	0.00	0.00	1.83	0.00	0.00	4.14	0.00
interferon-inducible GTPase 1-like	LOC100 759759	100759759	0.00	0.00	0.00	0.00	0.00	0.00	0.00	0.00	0.00	0.00	0.00	0.00
undifferentiated embryonic cell transcription factor 1 pseudogene	LOC100 759768	100759768	0.00	0.00	0.00	0.00	0.00	0.00	0.00	0.00	0.00	0.00	0.00	0.00
transmembrane protein 180-like	LOC100 759771	100759771	0.02	0.00	0.00	0.00	0.00	0.00	0.76	0.00	0.00	1.16	0.00	0.00
olfactory receptor 8B3	LOC100 759772	100759772	0.00	0.00	0.00	0.00	0.00	0.00	0.00	0.00	0.00	0.00	0.00	0.00
serine/threonine-protein phosphatase 2A regulatory subunit B'' subunit gamma pseudogene	LOC100 759774	100759774	0.29	0.19	0.30	0.28	0.37	0.23	9.87	6.41	7.45	9.27	12.42	7.48
olfactory receptor 5D14	LOC100 759775	100759775	0.00	0.00	0.00	0.00	0.00	0.00	0.00	0.00	0.00	0.00	0.00	0.00
60S ribosomal protein L35a pseudogene	LOC100 759776	100759776	0.76	1.12	1.26	0.47	1.06	0.54	15.19	10.98	12.42	8.11	15.52	6.54
histamine N-methyltransferase	LOC100 759779	100759779	0.15	0.09	0.00	0.00	0.00	0.00	1.52	0.92	0.00	0.00	0.00	0.00

olfactory receptor 10G6	LOC100759783	100759783	0.00	0.00	0.00	0.00	0.00	0.00	0.00	0.00	0.00	0.00	0.00	0.00
meteorin-like protein pseudogene	LOC100759785	100759785	0.00	0.00	0.00	0.00	0.00	0.00	0.00	0.00	0.00	0.00	0.00	0.93
cytochrome P450 2B1-like	LOC100759787	100759787	0.00	0.00	0.00	0.00	0.00	0.00	0.76	1.83	0.00	0.00	0.00	0.00
40S ribosomal protein S13 pseudogene	LOC100759789	100759789	0.10	0.00	0.00	0.00	0.00	0.00	2.28	0.00	0.00	1.16	0.00	0.00
tripartite motif-containing protein 6-like	LOC100759791	100759791	0.04	0.02	0.07	0.02	0.06	0.02	0.00	0.00	0.00	0.00	0.00	0.00
WD repeat-containing protein 5B	LOC100759797	100759797	6.15	5.72	7.05	7.52	8.69	7.78	3.80	0.00	2.48	2.32	2.07	1.87
heterogeneous nuclear ribonucleoprotein A1-like	LOC100759802	100759802	7.16	6.95	5.75	5.53	7.02	5.13	303.06	306.54	255.87	243.37	292.90	227.12
40S ribosomal protein S15	LOC100759804	100759804	13.87	11.14	10.54	5.37	4.40	4.52	413.19	409.02	382.56	215.56	193.54	210.30
centromere protein O pseudogene	LOC100759807	100759807	0.07	0.04	0.00	0.00	0.15	0.04	1.52	0.92	0.00	0.00	4.14	0.93
40S ribosomal protein S18 pseudogene	LOC100759812	100759812	0.00	0.00	0.00	0.00	0.00	0.00	0.00	0.00	0.00	0.00	0.00	0.00
probable G-protein coupled receptor 141	LOC100759821	100759821	0.00	0.00	0.00	0.00	0.00	0.00	0.00	0.00	0.00	0.00	0.00	0.00
sodium- and chloride-dependent transporter XTRP3B	LOC100759829	100759829	0.09	0.07	0.02	0.01	0.09	0.08	13.67	6.41	2.48	4.64	11.38	11.22
poly(A) RNA polymerase, mitochondrial	LOC100759832	100759832	15.09	15.31	14.98	16.43	16.45	15.53	707.13	726.54	693.08	750.98	765.89	730.91
acylcarnitine hydrolase-like	LOC100759838	100759838	0.05	0.06	0.05	0.05	0.09	0.04	3.80	5.49	4.97	3.48	5.17	2.80
spindle and kinetochore-associated protein 3-like	LOC100759845	100759845	0.00	0.00	0.00	0.00	0.00	0.00	0.00	0.00	0.00	0.00	0.00	0.00
spindle pole body component 110-like	LOC100759848	100759848	0.00	0.00	0.00	0.00	0.00	0.00	0.00	0.00	0.00	0.00	0.00	0.00
putative olfactory receptor 51H1	LOC100759849	100759849	0.00	0.00	0.00	0.00	0.00	0.00	0.00	0.00	0.00	0.00	0.00	0.00
alpha-enolase pseudogene	LOC100759850	100759850	0.00	0.00	0.00	0.00	0.00	0.00	0.00	0.00	0.00	0.00	0.00	0.00

uncharacterized LOC100759851	LOC100 759851	100759851	8.25	8.25	6.23	6.51	7.89	7.69	1554.02	1570.20	1363.81	1369.84	1633.21	1668.39
Ig heavy chain V-I region V35-like	LOC100 759854	100759854	0.00	0.00	0.00	0.00	0.00	0.00						
uncharacterized LOC100759856	LOC100 759856	100759856	0.00	0.00	0.00	0.00	0.00	0.00	0.00	0.00	0.00	0.00	0.00	0.00
arylacetamide deacetylase-like 4	LOC100 759860	100759860	0.00	0.00	0.00	0.00	0.00	0.00	0.00	0.00	0.00	0.00	0.00	0.00
chromosome unknown open reading frame, human C1orf185	LOC100 759862	100759862	0.05	0.00	0.00	0.00	0.00	0.00	0.76	0.00	0.00	0.00	0.00	0.00
chromosome unknown open reading frame, human C2orf66	LOC100 759873	100759873	0.00	0.00	0.00	0.30	0.00	0.00	0.00	0.00	0.00	1.16	0.00	0.00
L-threonine 3-dehydrogenase, mitochondrial	LOC100 759874	100759874	0.00	0.00	0.00	0.00	0.00	0.00	0.00	0.00	0.00	0.00	0.00	0.00
vomeronasal type-2 receptor 1	LOC100 759878	100759878	0.00	0.00	0.00	0.00	0.00	0.00	0.00	0.00	0.00	0.00	0.00	0.00
olfactory receptor 8B4	LOC100 759880	100759880	0.00	0.00	0.00	0.00	0.00	0.00	0.00	0.00	0.00	0.00	0.00	0.00
alpha-1B-glycoprotein-like	LOC100 759881	100759881	0.00	0.00	0.00	0.00	0.00	0.00	0.00	0.00	0.00	0.00	0.00	0.00
N-acetylglucosaminyl-phosphatidylinositol biosynthetic protein pseudogene	LOC100 759884	100759884	0.06	0.08	0.13	0.12	0.08	0.09	0.00	0.00	0.00	0.00	0.00	0.00
5-hydroxytryptamine receptor 5B	LOC100 759887	100759887	0.00	0.00	0.00	0.00	0.00	0.00	0.00	0.00	0.00	0.00	0.00	0.00
chromosome unknown open reading frame, human C7orf72	LOC100 759903	100759903	0.00	0.00	0.00	0.00	0.00	0.00	0.00	0.00	0.00	0.00	0.00	0.00
pre-mRNA-splicing factor 38A pseudogene	LOC100 759904	100759904	0.00	0.00	0.00	0.00	0.00	0.00	0.00	0.00	0.00	0.00	0.00	0.00
protein NipSnap homolog 2 pseudogene	LOC100 759907	100759907	0.00	0.00	0.00	0.00	0.00	0.00	0.00	0.00	0.00	0.00	0.00	0.00
M-phase phosphoprotein 6 pseudogene	LOC100 759910	100759910	0.46	0.39	0.39	0.26	0.21	0.25	10.63	8.24	8.69	5.79	4.14	5.61
olfactory receptor 4P4	LOC100 759911	100759911	0.00	0.00	0.00	0.00	0.00	0.00	0.00	0.00	0.00	0.00	0.00	0.00
olfactory receptor 4C15-like	LOC100 759912	100759912	0.00	0.00	0.00	0.00	0.00	0.00	0.00	0.00	0.00	0.00	0.00	0.00

RLA class II histocompatibility antigen, DP alpha-1 chain	LOC100759913	100759913	0.00	0.00	0.00	0.00	0.00	0.00	0.76	0.00	0.00	1.16	0.00	0.00
olfactory receptor 5M10-like	LOC100759915	100759915	0.00	0.00	0.00	0.00	0.00	0.00	0.00	0.00	0.00	0.00	0.00	0.00
E3 ubiquitin-protein ligase RNF5 pseudogene	LOC100759916	100759916	0.00	0.00	0.00	0.00	0.00	0.00	0.00	0.00	0.00	0.00	0.00	0.00
transcription factor BTF3	LOC100759921	100759921	1.74	1.26	1.04	0.65	0.87	0.27	9.11	10.07	4.97	4.64	5.17	0.93
adenylate kinase 4, mitochondrial pseudogene	LOC100759922	100759922	0.44	0.33	0.32	0.26	0.00	0.34	6.08	4.58	3.73	3.48	0.00	4.67
programmed cell death protein 5 pseudogene	LOC100759924	100759924	5.61	5.13	5.43	4.11	2.87	2.48	45.57	45.75	42.23	24.34	17.59	16.82
60S ribosomal protein L23a pseudogene	LOC100759928	100759928	0.64	0.33	0.46	0.00	0.26	0.06	5.32	3.66	3.73	0.00	2.07	0.00
polyadenylate-binding protein 4 pseudogene	LOC100759930	100759930	0.00	0.00	0.00	0.00	0.00	0.00	0.00	0.00	0.00	0.00	0.00	0.00
chromosome unknown open reading frame, human C11orf86	LOC100759935	100759935	0.03	0.04	0.00	0.05	0.00	0.00	1.52	1.83	0.00	1.16	0.00	0.00
immunoglobulin omega chain	LOC100759939	100759939	0.00	0.00	0.00	0.00	0.00	0.00	0.00	0.00	0.00	0.00	0.00	0.00
olfactory receptor 4F3/4F16/4F29	LOC100759940	100759940	0.00	0.00	0.00	0.00	0.00	0.00	0.00	0.00	0.00	0.00	0.00	0.00
olfactory receptor 4D9-like	LOC100759941	100759941	0.00	0.00	0.00	0.00	0.00	0.00	0.00	0.00	0.00	0.00	0.00	0.00
uncharacterized LOC100759943	LOC100759943	100759943	0.00	0.00	0.00	0.00	0.00	0.00	0.00	0.00	0.00	0.00	0.00	0.00
CMRF35-like molecule 3	LOC100759951	100759951	0.00	0.00	0.00	0.00	0.00	0.00	0.00	0.00	0.00	0.00	0.00	0.00
coiled-coil-helix-coiled-coil-helix domain-containing protein 2, mitochondrial pseudogene	LOC100759955	100759955	0.00	0.00	0.00	0.00	0.11	0.00	0.00	0.00	0.00	0.00	1.03	0.00
	LOC100759959	100759959	0.00	0.00	0.00	0.00	0.00	0.00	0.00	0.00	0.00	0.00	0.00	0.00
olfactory receptor 51I2	LOC100759960	100759960	0.00	0.00	0.00	0.00	0.00	0.00	0.00	0.00	0.00	0.00	0.00	0.00
Ig heavy chain V-II region SESS-like	LOC100759962	100759962	0.00	0.00	0.00	0.00	0.00	0.00						

chromosome unknown open reading frame, human C17orf67	LOC100759971	100759971	0.00	0.00	0.00	0.00	0.00	0.00	0.00	0.00	0.00	0.00	0.00	0.00
ATP-binding cassette sub-family G member 3-like	LOC100759972	100759972	0.11	0.00	0.04	0.02	0.00	0.00	1.52	0.00	2.48	1.16	0.00	0.00
alpha-1B-glycoprotein-like	LOC100759976	100759976	0.00	0.00	0.00	0.00	0.00	0.00	0.00	0.00	0.00	0.00	0.00	0.00
vomeronasal type-1 receptor 4-like	LOC100759977	100759977	0.00	0.00	0.00	0.00	0.00	0.00	0.00	0.00	0.00	0.00	0.00	0.00
vomeronasal type-1 receptor 2-like	LOC100759978	100759978	0.00	0.00	0.00	0.00	0.00	0.00	0.00	0.00	0.00	0.00	0.00	0.00
60S ribosomal protein L23a pseudogene	LOC100759979	100759979	20.14	16.16	13.04	8.49	10.76	9.47	15.95	12.81	9.94	10.43	8.28	4.67
olfactory receptor 8B12-like	LOC100759980	100759980	0.00	0.00	0.00	0.00	0.00	0.00	0.00	0.00	0.00	0.00	0.00	0.00
chromosome unknown open reading frame, human C16orf78	LOC100759982	100759982	0.00	0.00	0.00	0.00	0.00	0.00	0.00	0.00	0.00	0.00	0.00	0.00
mortality factor 4-like protein 2	LOC100759984	100759984	0.00	0.00	0.00	0.00	0.00	0.00	0.00	0.00	0.00	0.00	0.00	0.00
olfactory receptor 2G3-like	LOC100759985	100759985	0.00	0.00	0.00	0.00	0.00	0.00	0.00	0.00	0.00	0.00	0.00	0.00
intelectin-1a-like	LOC100759987	100759987	0.01	0.00	0.00	0.00	0.00	0.00	0.00	0.00	0.00	0.00	0.00	0.00
MARVEL domain-containing protein 3-like	LOC100759990	100759990	0.00	0.00	0.00	0.00	0.00	0.00	0.00	0.00	0.00	0.00	0.00	0.00
histone-lysine N-methyltransferase SETDB2 pseudogene	LOC100759993	100759993	0.00	0.00	0.00	0.00	0.00	0.00	0.00	0.00	0.00	0.00	0.00	0.00
hyaluronidase PH-20	LOC100759996	100759996	0.00	0.00	0.00	0.00	0.00	0.00	0.00	0.00	0.00	0.00	0.00	0.00
Fc receptor-like protein 1	LOC100759999	100759999	0.00	0.00	0.00	0.00	0.00	0.00	0.00	0.00	0.00	0.00	1.03	0.00
40S ribosomal protein S4, X isoform-like	LOC100760008	100760008	47.55	50.45	45.97	37.40	45.64	39.47	149.63	181.18	154.02	115.89	158.35	96.27
dnaJ homolog subfamily A member 1 pseudogene	LOC100760009	100760009	0.00	0.00	0.00	0.00	0.00	0.00	0.00	0.00	0.00	0.00	0.00	0.00
60S ribosomal protein L27a pseudogene	LOC100760012	100760012	0.66	0.87	0.26	0.44	0.54	0.30	4.56	6.41	2.48	3.48	6.21	2.80

histone H1.5	LOC100760013	100760013	0.00	0.00	0.00	0.00	0.00	0.02	0.00	0.00	0.00	0.00	0.00	0.00
putative deoxyribose-phosphate aldolase pseudogene	LOC100760014	100760014	0.00	0.00	0.05	0.00	0.00	0.00	0.00	0.00	1.24	0.00	0.00	0.00
isopentenyl-diphosphate Delta-isomerase 1-like	LOC100760015	100760015	0.05	0.06	0.09	0.00	0.00	0.06	0.76	0.92	1.24	0.00	0.00	0.93
cytochrome c oxidase subunit 7C, mitochondrial pseudogene	LOC100760019	100760019	117.92	122.08	96.34	103.27	83.76	103.99	566.62	578.30	480.69	409.10	321.88	387.89
transmembrane emp24 domain-containing protein 7	LOC100760020	100760020	28.97	28.15	27.63	30.95	37.07	33.57	2618.13	2560.26	2521.44	2804.59	3330.59	3060.11
leucine-rich repeat-containing protein 70	LOC100760022	100760022	0.47	0.46	0.22	0.21	0.18	0.06	16.71	19.22	6.21	6.95	9.31	1.87
60S ribosomal protein L18a-like	LOC100760023	100760023	0.00	0.08	0.00	0.00	0.00	0.00	6.84	10.98	6.21	3.48	2.07	0.00
uncharacterized LOC100760024	LOC100760024	100760024	0.10	0.11	0.00	0.00	0.00	0.00	6.08	8.24	0.00	0.00	0.00	0.00
pre-mRNA-splicing regulator WTAP	LOC100760025	100760025	66.65	66.15	58.16	62.77	69.19	69.27	492.18	527.06	438.46	439.23	500.93	466.40
exocyst complex component 3-like protein	LOC100760026	100760026	0.19	0.08	0.14	0.04	0.05	0.03	15.19	6.41	9.94	2.32	5.17	4.67
interferon alpha-12-like	LOC100760028	100760028	0.00	0.00	0.00	0.00	0.00	0.00	0.00	0.00	0.00	0.00	0.00	0.00
40S ribosomal protein S2 pseudogene	LOC100760029	100760029	0.00	0.06	0.00	0.00	0.07	0.00	0.00	0.92	0.00	0.00	1.03	0.00
zinc finger protein 420-like	LOC100760035	100760035	2.14	1.76	2.13	2.27	2.45	2.44	102.54	83.27	110.55	118.21	112.81	120.57
40S ribosomal protein S2 pseudogene	LOC100760046	100760046	0.22	0.10	0.07	0.00	0.03	0.07	0.76	0.00	0.00	0.00	0.00	0.00
interferon-inducible GTPase 1	LOC100760047	100760047	0.00	0.00	0.00	0.00	0.00	0.00	0.00	0.00	0.00	0.00	0.00	0.00
ubiquitin-conjugating enzyme E2 D2 pseudogene	LOC100760048	100760048	0.00	0.00	0.00	0.00	0.00	0.00	0.00	0.00	0.00	0.00	0.00	0.00
cytochrome c-like	LOC100760052	100760052	0.00	0.00	0.27	0.00	0.00	0.00	0.00	0.00	2.48	1.16	0.00	0.00
small integral membrane protein 15-like	LOC100760057	100760057	0.00	0.00	0.00	0.00	0.00	0.00	0.00	0.00	0.00	0.00	0.00	0.00

olfactory receptor 8B3-like	LOC100760061	100760061	0.00	0.00	0.00	0.00	0.00	0.00	0.00	0.00	0.00	0.00	0.00	0.00
olfactory receptor 13C9	LOC100760063	100760063	0.00	0.00	0.00	0.00	0.00	0.00	0.00	0.00	0.00	0.00	0.00	0.00
olfactory receptor 5D18	LOC100760064	100760064	0.00	0.00	0.00	0.00	0.00	0.00	0.00	0.00	0.00	0.00	0.00	0.00
spermatogenesis-associated protein 31-like	LOC100760067	100760067	0.00	0.00	0.00	0.00	0.00	0.00	0.00	0.00	0.00	0.00	0.00	0.00
plasminogen activator inhibitor 1 RNA-binding protein pseudogene	LOC100760068	100760068	25.61	29.37	28.23	24.65	17.41	14.19	832.45	948.89	917.90	791.54	556.82	459.86
chromosome unknown open reading frame, human C2orf88	LOC100760069	100760069	0.00	0.00	0.00	0.00	0.00	0.14	0.76	0.00	0.00	1.16	1.03	0.93
olfactory receptor 10S1	LOC100760073	100760073	0.00	0.00	0.00	0.00	0.00	0.00	0.00	0.00	0.00	0.00	0.00	0.00
chromosome unknown open reading frame, human C2orf70	LOC100760075	100760075	0.12	0.10	0.10	0.25	0.26	0.18	8.35	7.32	6.21	16.22	12.42	11.22
short-chain dehydrogenase/reductase family 16C member 6	LOC100760076	100760076	0.02	0.00	0.00	0.00	0.00	0.00	1.52	0.00	0.00	0.00	0.00	0.00
olfactory receptor 51I2	LOC100760078	100760078	0.00	0.00	0.00	0.00	0.00	0.00	0.00	0.00	0.00	0.00	0.00	0.00
alcohol dehydrogenase 1	LOC100760081	100760081	0.03	0.01	0.00	0.00	0.00	0.00	0.76	0.00	0.00	0.00	0.00	0.00
60S ribosomal protein L27	LOC100760082	100760082	43.17	46.21	33.53	27.46	30.80	26.03	129.88	80.52	65.83	46.36	40.36	56.08
uncharacterized LOC100760083	LOC100760083	100760083	0.00	0.00	0.00	0.00	0.00	0.00	0.00	0.00	0.00	0.00	0.00	0.00
chromodomain Y-like protein	LOC100760087	100760087	0.07	0.27	0.08	0.18	0.15	0.23	3.04	10.98	4.97	13.91	7.24	8.41
proto-oncogene FRAT1-like	LOC100760088	100760088	0.00	0.00	0.00	0.00	0.00	0.00	0.00	0.00	1.24	0.00	0.00	0.00
non-histone chromosomal protein HMG-17 pseudogene	LOC100760090	100760090	0.00	0.00	0.00	0.00	0.00	0.00	0.00	0.00	0.00	0.00	0.00	0.00
chromobox protein homolog 3 pseudogene	LOC100760099	100760099	0.30	0.36	0.22	0.09	0.15	0.14	3.80	5.49	2.48	1.16	2.07	3.74
olfactory receptor 2B11	LOC100760102	100760102	0.00	0.00	0.00	0.00	0.00	0.00	0.00	0.00	0.00	0.00	0.00	0.00

tubulin polyglutamylase TTLL6-like	LOC100760107	100760107	0.39	0.38	0.10	0.08	0.15	0.09	25.06	20.13	4.97	4.64	10.35	6.54
nucleophosmin pseudogene	LOC100760108	100760108	0.00	0.00	0.00	0.00	0.00	0.00	0.76	0.00	0.00	0.00	0.00	0.00
RNA-binding protein FUS pseudogene	LOC100760114	100760114	0.00	0.00	0.00	0.00	0.00	0.00	0.00	0.00	0.00	0.00	0.00	0.00
gamma-crystallin D	LOC100760119	100760119	0.00	0.00	0.00	0.00	0.00	0.00	0.00	0.00	0.00	0.00	0.00	0.00
sodium- and chloride-dependent transporter XTRP3A	LOC100760123	100760123	0.00	0.04	0.00	0.06	0.02	0.02	0.76	2.75	0.00	4.64	2.07	0.93
lysozyme-like protein 1	LOC100760126	100760126	0.00	0.00	0.00	0.00	0.00	0.00	0.00	0.00	0.00	0.00	0.00	0.00
mortality factor 4-like protein 1	LOC100760128	100760128	2.89	2.76	1.68	1.80	1.22	1.56	81.27	77.78	45.96	50.99	42.43	43.93
ATP synthase F(0) complex subunit C2, mitochondrial pseudogene	LOC100760132	100760132	0.92	0.95	0.89	0.66	0.59	0.68	13.67	21.05	14.91	11.59	13.45	7.48
vomeronasal type-2 receptor 116-like	LOC100760141	100760141	0.00	0.04	0.02	0.02	0.00	0.04	0.00	3.66	1.24	1.16	1.03	3.74
GDNF family receptor alpha-1	LOC100760143	100760143	0.55	0.63	0.56	0.65	0.76	0.65	34.18	30.20	26.08	32.45	33.12	35.52
olfactory receptor 51G2	LOC100760144	100760144	0.00	0.00	0.00	0.00	0.00	0.00	0.00	0.00	0.00	0.00	0.00	0.00
chromosome unknown open reading frame, human C21orf140	LOC100760147	100760147	0.00	0.00	0.00	0.00	0.00	0.06	0.00	0.00	0.00	0.00	0.00	0.93
uncharacterized LOC100760150	LOC100760150	100760150	0.00	0.00	0.00	0.00	0.00	0.00	0.00	0.00	0.00	0.00	0.00	0.00
calreticulin-like	LOC100760153	100760153	0.83	0.86	0.47	0.46	0.57	0.67	50.89	52.16	34.78	28.97	34.15	43.93
tRNA (uracil(54)-C(5))-methyltransferase homolog pseudogene	LOC100760154	100760154	0.08	0.02	0.07	0.06	0.03	0.05	3.04	0.00	2.48	2.32	1.03	1.87
WD repeat-containing protein 49-like	LOC100760155	100760155	0.01	0.00	0.00	0.00	0.00	0.00	0.76	0.00	1.24	1.16	0.00	0.00
sterile alpha motif domain-containing protein 9-like	LOC100760157	100760157	0.46	0.38	0.34	0.35	0.41	0.32	3.80	8.24	2.48	5.79	7.24	7.48
N-acetyllactosaminide beta-1,6-N-acetylglucosaminyl-transferase, isoform C-like	LOC100760161	100760161	0.00	0.00	0.00	0.00	0.00	0.00	0.00	0.00	0.00	0.00	0.00	0.00

glyceraldehyde-3-phosphate dehydrogenase pseudogene	LOC100760164	100760164	0.00	0.00	0.00	0.00	0.00	0.04	0.00	0.00	0.00	0.00	0.00	0.93
ephrin-B2-like	LOC100760165	100760165	9.31	8.05	10.89	11.49	12.17	11.86	758.78	671.63	911.69	975.81	1036.02	1020.66
chromosome unknown open reading frame, human C3orf33	LOC100760167	100760167	13.68	13.48	11.58	12.73	13.14	12.36	364.58	356.86	311.76	338.40	342.58	328.07
DNA-dependent protein kinase catalytic subunit-like	LOC100760171	100760171	1.59	1.63	1.36	1.88	1.89	1.52	0.76	0.00	0.00	0.00	0.00	0.00
UBA-like domain-containing protein 1 pseudogene	LOC100760172	100760172	0.00	0.00	0.00	0.00	0.00	0.00	0.00	0.00	0.00	1.16	0.00	0.00
glycosyltransferase 6 domain-containing protein 1-like	LOC100760173	100760173	0.00	0.00	0.00	0.00	0.00	0.00	0.00	0.00	0.00	0.00	0.00	0.00
serine protease inhibitor A3N-like	LOC100760187	100760187	0.00	0.00	0.00	0.00	0.00	0.00	0.00	0.00	0.00	0.00	0.00	0.00
DNA (cytosine-5)-methyltransferase 3B-like	LOC100760192	100760192	0.00	0.00	0.00	0.00	0.00	0.00	0.76	0.92	2.48	2.32	3.10	0.93
olfactory receptor 1073	LOC100760194	100760194	0.00	0.00	0.00	0.00	0.00	0.00	0.00	0.00	0.00	0.00	0.00	0.00
peptidyl-prolyl cis-trans isomerase A-like	LOC100760197	100760197	57.49	64.77	54.83	45.12	52.27	38.06	207.35	228.76	150.29	126.32	144.90	69.17
olfactory receptor-like protein OLF3	LOC100760201	100760201	0.00	0.00	0.00	0.00	0.00	0.00	0.00	0.00	0.00	0.00	0.00	0.00
transcription elongation factor B polypeptide 2 pseudogene	LOC100760206	100760206	0.20	0.15	0.27	0.26	0.11	0.00	0.00	0.00	0.00	0.00	0.00	0.00
olfactory receptor 4C15-like	LOC100760207	100760207	0.00	0.00	0.00	0.00	0.00	0.00	0.00	0.00	0.00	0.00	0.00	0.00
HLA class II histocompatibility antigen, DO alpha chain	LOC100760208	100760208	0.10	0.04	0.00	0.00	0.00	0.00	2.28	1.83	0.00	0.00	0.00	0.00
gastric triacylglycerol lipase-like	LOC100760209	100760209	0.00	0.00	0.00	0.00	0.00	0.00	0.00	0.00	0.00	0.00	0.00	0.00
putative olfactory receptor 5AK3	LOC100760210	100760210	0.00	0.04	0.06	0.00	0.00	0.00	0.00	0.92	1.24	0.00	0.00	0.00
aldehyde oxidase 4	LOC100760212	100760212	0.00	0.00	0.00	0.00	0.00	0.00	0.00	0.00	0.00	0.00	0.00	0.00
60S ribosomal protein L27a-like	LOC100760216	100760216	0.00	0.00	0.00	0.00	0.00	0.00	0.00	0.00	0.00	0.00	0.00	0.00

heat shock 70 kDa protein 1A	LOC100760218	100760218	0.23	0.23	0.12	0.11	0.02	0.11	15.19	13.73	6.21	6.95	1.03	6.54
olfactory receptor 6X1	LOC100760226	100760226	0.00	0.00	0.00	0.00	0.00	0.00	0.00	0.00	0.00	0.00	0.00	0.00
sperm motility kinase W	LOC100760228	100760228	0.00	0.00	0.00	0.00	0.00	0.00	0.00	0.00	0.00	0.00	0.00	0.00
uncharacterized LOC100760232	LOC100760232	100760232	0.00	0.00	0.00	0.00	0.00	0.00	0.00	0.00	0.00	0.00	0.00	0.00
immunoglobulin omega chain	LOC100760234	100760234	0.00	0.00	0.00	0.00	0.00	0.00	0.00	0.00	0.00	0.00	0.00	0.00
olfactory receptor 4F3/4F16/4F29	LOC100760235	100760235	0.00	0.00	0.00	0.00	0.00	0.00	0.00	0.00	0.00	0.00	0.00	0.00
olfactory receptor 4D10-like	LOC100760236	100760236	0.00	0.00	0.00	0.00	0.00	0.00	0.00	0.00	0.00	0.00	0.00	0.00
protein LAP2-like	LOC100760237	100760237	0.82	0.82	0.72	0.80	0.81	0.69	25.06	26.54	23.60	17.38	16.56	21.50
uncharacterized LOC100760240	LOC100760240	100760240	0.00	0.00	0.00	0.00	0.00	0.00	0.00	0.00	0.00	0.00	0.00	0.00
60S ribosomal protein L5-like	LOC100760242	100760242	0.00	0.02	0.00	0.07	0.00	0.00	0.00	0.00	0.00	1.16	0.00	0.00
CMRF35-like molecule	LOC100760245	100760245	0.00	0.00	0.00	0.00	0.00	0.00	0.00	0.00	0.00	0.00	0.00	0.00
interferon-inducible double-stranded RNA-dependent protein kinase activator A pseudogene	LOC100760247	100760247	0.00	0.00	0.00	0.00	0.00	0.00	0.00	0.00	0.00	0.00	0.00	0.00
cytochrome P450 2B1-like	LOC100760248	100760248	0.00	0.00	0.00	0.00	0.00	0.00	0.00	0.00	0.00	0.00	0.00	0.00
YY1-associated factor 2	LOC100760251	100760251	28.39	30.36	29.13	28.07	32.63	33.90	1330.71	1378.96	1322.82	1255.11	1463.47	1544.07
olfactory receptor 51F2-like	LOC100760253	100760253	0.00	0.00	0.00	0.00	0.00	0.00	0.00	0.00	0.00	0.00	0.00	0.00
60S ribosomal protein L23 pseudogene	LOC100760254	100760254	0.20	0.37	0.17	0.16	0.14	0.39	1.52	2.75	1.24	1.16	1.03	2.80
olfactory receptor 2B11	LOC100760259	100760259	0.00	0.00	0.00	0.00	0.00	0.00	0.00	0.00	0.00	0.00	0.00	0.00
	LOC100760261	100760261	150.79	148.70	150.03	161.86	161.40	159.09	6996.88	7041.18	7019.03	7529.50	7599.90	7370.81

aphrodisin-like	LOC100760262	100760262	0.00	0.00	0.00	0.00	0.00	0.00	0.00	0.00	0.00	0.00	0.00	0.00
lupus La protein homolog pseudogene	LOC100760264	100760264	0.38	0.12	0.32	0.24	0.00	0.07	9.87	3.66	8.69	5.79	0.00	1.87
tripartite motif-containing protein 6 pseudogene	LOC100760265	100760265	0.00	0.04	0.05	0.00	0.00	0.00	0.00	0.92	1.24	0.00	0.00	0.00
olfactory receptor 1D2	LOC100760267	100760267	0.00	0.00	0.00	0.00	0.00	0.00	0.00	0.00	0.00	0.00	0.00	0.00
nucleoporin SEH1-like	LOC100760269	100760269	0.00	0.00	0.00	0.00	0.00	0.00	0.00	0.00	0.00	0.00	0.00	0.00
vomeronasal type-1 receptor 4-like	LOC100760270	100760270	0.00	0.00	0.00	0.00	0.00	0.00	0.00	0.00	0.00	0.00	0.00	0.00
olfactory receptor 10C1-like	LOC100760272	100760272	0.00	0.00	0.00	0.00	0.00	0.00	0.00	0.00	0.00	0.00	0.00	0.00
histone H3.3	LOC100760278	100760278	19.93	18.69	16.39	16.33	10.63	11.37	104.82	86.01	85.70	68.38	32.08	43.93
hyaluronidase PH-20-like	LOC100760289	100760289	0.00	0.00	0.00	0.00	0.00	0.00	0.00	0.00	0.00	0.00	0.00	0.00
60S ribosomal protein L7 pseudogene	LOC100760297	100760297	0.52	0.46	0.48	0.07	0.45	0.28	11.39	10.98	9.94	1.16	8.28	6.54
L-gulonolactone oxidase	LOC100760306	100760306	0.06	0.05	0.09	0.13	0.11	0.08	7.60	8.24	8.69	17.38	14.49	9.35
histone H2B type 1	LOC100760308	100760308	0.11	0.06	0.07	0.02	0.08	0.05	0.00	0.00	0.00	0.00	0.00	0.00
schwannomin-interacting protein 1	LOC100760309	100760309	7.40	8.14	8.93	8.50	9.02	8.44	388.12	441.05	468.27	441.55	476.09	448.64
neuronal acetylcholine receptor subunit alpha-7	LOC100760319	100760319	0.00	0.00	0.00	0.00	0.00	0.00	0.00	0.00	0.00	0.00	0.00	0.00
interferon alpha-12	LOC100760323	100760323	0.00	0.00	0.00	0.00	0.00	0.00	0.00	0.00	0.00	0.00	0.00	0.00
olfactory receptor 11A1	LOC100760332	100760332	0.00	0.00	0.00	0.05	0.00	0.00	0.00	0.00	0.00	1.16	0.00	0.00
leucine-rich repeat and calponin homology domain-containing protein 1	LOC100760337	100760337	9.44	9.15	9.37	10.64	11.39	11.55	1156.02	1139.22	1135.27	1303.78	1365.15	1394.53
glyceraldehyde-3-phosphate dehydrogenase pseudogene	LOC100760338	100760338	0.04	0.00	0.00	0.06	0.00	0.02	0.76	0.00	0.00	1.16	0.00	0.00

60S ribosomal protein L27 pseudogene	LOC100 760340	100760340	0.00	0.00	0.00	0.00	0.00	0.00	0.76	0.92	0.00	0.00	1.03	0.00
interferon-inducible GTPase 1-like	LOC100 760341	100760341	0.03	0.00	0.00	0.00	0.04	0.00	0.76	0.00	0.00	0.00	1.03	0.00
dentin sialophosphoprotein-like	LOC100 760342	100760342	0.01	0.03	0.01	0.01	0.03	0.02	2.28	2.75	1.24	1.16	3.10	1.87
prefoldin subunit 1 pseudogene	LOC100 760343	100760343	0.09	0.29	0.07	0.13	0.36	0.16	1.52	4.58	1.24	3.48	6.21	2.80
PIH1 domain-containing protein 1 pseudogene	LOC100 760347	100760347	0.05	0.00	0.09	0.08	0.00	0.00	0.76	0.92	1.24	2.32	0.00	0.00
serpin B6	LOC100 760352	100760352	80.33	82.53	51.31	52.14	68.35	75.41	2470.02	2632.55	1586.14	1627.12	2087.57	2258.16
olfactory receptor 8B3-like	LOC100 760355	100760355	0.00	0.00	0.00	0.00	0.00	0.00	0.00	0.00	0.00	0.00	0.00	0.00
olfactory receptor 13D1	LOC100 760357	100760357	0.00	0.00	0.00	0.00	0.00	0.00	0.00	0.00	0.00	0.00	0.00	0.00
olfactory receptor 10AG1-like	LOC100 760358	100760358	0.00	0.00	0.00	0.00	0.00	0.00	0.00	0.00	0.00	0.00	0.00	0.00
60S ribosomal protein L19 pseudogene	LOC100 760359	100760359	17.49	17.01	11.02	6.82	12.74	10.34	108.61	111.63	68.31	50.99	129.37	98.14
asparagine--tRNA ligase, cytoplasmic pseudogene	LOC100 760362	100760362	19.14	18.64	18.11	19.31	19.90	18.55	751.94	725.62	699.29	718.53	733.81	674.83
TATA box-binding protein-like protein 2	LOC100 760364	100760364	6.22	6.48	4.55	5.11	6.58	6.04	406.35	399.87	298.10	330.29	418.13	392.56
ribonucleoside-diphosphate reductase subunit M2 pseudogene	LOC100 760365	100760365	0.00	0.00	0.00	0.00	0.00	0.00	0.00	0.00	0.00	0.00	0.00	0.00
olfactory receptor 145	LOC100 760369	100760369	0.00	0.00	0.00	0.00	0.00	0.00	0.00	0.00	0.00	0.00	0.00	0.00
olfactory receptor 51E1	LOC100 760374	100760374	0.00	0.00	0.00	0.00	0.00	0.00	0.00	0.00	0.00	0.00	0.00	0.00
ATP-binding cassette sub-family A member 3-like	LOC100 760380	100760380	0.00	0.00	0.00	0.00	0.00	0.00	0.00	0.00	0.00	0.00	0.00	0.00
nucleoporin SEH1 pseudogene	LOC100 760384	100760384	0.02	0.08	0.00	0.00	0.06	0.00	0.00	0.00	0.00	0.00	0.00	0.00
acidic leucine-rich nuclear phosphoprotein 32 family member A pseudogene	LOC100 760391	100760391	0.11	0.00	0.00	0.00	0.00	0.00	1.52	0.00	0.00	0.00	0.00	0.00

uncharacterized LOC100760395	LOC100 760395	100760395	0.00	0.00	0.00	0.00	0.00	0.00	0.00	0.00	0.00	0.00	0.00	0.00
60S ribosomal protein L23-like	LOC100 760400	100760400	3.82	4.43	2.02	1.78	2.42	1.31	47.85	53.07	23.60	19.70	27.94	17.76
uncharacterized LOC100760401	LOC100 760401	100760401	0.06	0.57	0.09	0.19	0.32	0.07	3.04	9.15	2.48	3.48	8.28	2.80
prolactin-8A9-like	LOC100 760404	100760404	0.00	0.00	0.00	0.00	0.00	0.00	0.00	0.00	0.00	0.00	0.00	0.00
60S ribosomal protein L26-like	LOC100 760405	100760405	94.22	85.37	90.04	78.52	84.96	85.41	580.29	527.97	547.76	403.30	455.39	435.56
gamma-crystallin E	LOC100 760411	100760411	0.00	0.00	0.00	0.00	0.00	0.00	0.00	0.00	0.00	0.00	0.00	0.00
25-hydroxyvitamin D-1 alpha hydroxylase, mitochondrial	LOC100 760414	100760414	0.26	0.22	0.15	0.19	0.11	0.22	21.27	19.22	16.15	13.91	8.28	17.76
ferritin light chain pseudogene	LOC100 760418	100760418	0.00	0.00	0.00	0.00	0.00	0.00	0.00	0.00	0.00	0.00	0.00	0.00
1,2-dihydroxy-3-keto-5-methylthiopentene dioxygenase pseudogene	LOC100 760419	100760419	0.00	0.00	0.00	0.00	0.00	0.00	0.00	0.00	0.00	0.00	0.00	0.00
uncharacterized LOC100760420	LOC100 760420	100760420	0.00	0.42	0.60	1.24	1.52	0.48	56.21	47.58	58.38	75.33	61.06	69.17
cytochrome b5 type B	LOC100 760422	100760422	72.95	76.53	70.67	69.78	76.67	80.02	4026.32	4270.46	3869.10	3649.44	3938.12	4096.66
sphingolipid delta(4)-desaturase DES1-like	LOC100 760423	100760423	8.16	8.09	7.12	6.40	7.79	8.34	31.14	30.20	21.12	15.07	16.56	16.82
39S ribosomal protein L12, mitochondrial-like	LOC100 760430	100760430	0.12	0.00	0.00	0.10	0.00	0.00	4.56	0.92	0.00	3.48	1.03	0.00
probable rRNA-processing protein EBP2	LOC100 760432	100760432	0.03	0.00	0.00	0.00	0.09	0.00	0.76	0.00	0.00	0.00	2.07	0.00
zinc finger protein 420-like	LOC100 760433	100760433	2.43	2.17	2.68	2.58	2.94	2.55	141.27	131.76	158.99	156.45	168.70	151.42
olfactory receptor 51A7-like	LOC100 760436	100760436	0.00	0.00	0.00	0.00	0.00	0.00	0.00	0.00	0.00	0.00	0.00	0.00
uncharacterized LOC100760441	LOC100 760441	100760441	0.00	0.00	0.00	0.00	0.00	0.00	0.00	0.00	0.00	0.00	0.00	0.00
ADP-ribosylation factor-like protein 8B pseudogene	LOC100 760444	100760444	0.07	0.09	0.00	0.11	0.10	0.00	1.52	1.83	0.00	1.16	2.07	0.93

60S ribosomal protein L35-like	LOC100760445	100760445	0.71	0.65	1.04	0.71	0.50	0.80	6.08	5.49	8.69	5.79	4.14	7.48
putative serine/threonine-protein	LOC100760452	100760452	2.79	2.44	5.44	5.85	7.23	6.88	296.22	278.17	467.02	502.97	578.56	582.30
carcinoembryonic antigen-related cell adhesion molecule 3-like	LOC100760462	100760462	0.00	0.00	0.00	0.00	0.00	0.00	0.00	0.00	0.00	0.00	0.00	0.00
DDB1- and CUL4-associated factor 8 pseudogene	LOC100760463	100760463	0.27	0.16	0.13	0.13	0.07	0.03	18.23	15.56	9.94	6.95	4.14	3.74
40S ribosomal protein S16 pseudogene	LOC100760465	100760465	0.10	0.00	0.00	0.00	0.00	0.00	1.52	0.00	0.00	0.00	0.00	0.00
chromatin target of PRMT1 protein pseudogene	LOC100760466	100760466	0.06	0.16	0.11	0.10	0.00	0.00	0.76	1.83	1.24	1.16	1.03	0.00
60S ribosomal protein L23a-like	LOC100760472	100760472	8.03	9.16	8.13	3.77	6.95	3.82	34.94	33.86	32.29	13.91	26.91	14.95
keratin-associated protein 10-2-like	LOC100760473	100760473	0.00	0.00	0.00	0.00	0.00	0.00	0.00	0.00	0.00	0.00	0.00	0.00
receptor tyrosine-protein kinase erbB-4	LOC100760475	100760475	0.00	0.00	0.00	0.00	0.00	0.08	1.52	0.00	0.00	0.00	1.03	4.67
60S ribosomal protein L13a pseudogene	LOC100760483	100760483	2.06	1.64	1.57	1.65	1.16	0.70	24.31	18.30	18.63	15.07	12.42	8.41
transmembrane protein 189	LOC100760484	100760484	32.36	30.45	30.26	22.26	18.30	20.09	1010.95	970.85	948.95	703.46	577.52	636.51
60S ribosomal protein L3 pseudogene	LOC100760487	100760487	0.94	1.46	0.81	0.93	1.19	0.49	32.66	51.24	28.57	32.45	41.40	16.82
pyruvate dehydrogenase E1 component subunit alpha, somatic form, mitochondrial-like	LOC100760488	100760488	0.08	0.03	0.00	0.04	0.04	0.05	3.04	4.58	0.00	6.95	2.07	7.48
olfactory receptor-like protein OLF3	LOC100760491	100760491	0.00	0.00	0.00	0.00	0.00	0.00	0.00	0.00	0.00	0.00	0.00	0.00
40S ribosomal protein S13 pseudogene	LOC100760497	100760497	0.00	0.00	0.00	0.00	0.00	0.00	0.00	0.00	0.00	0.00	0.00	0.00
olfactory receptor 4C15-like	LOC100760498	100760498	0.00	0.00	0.00	0.00	0.00	0.00	0.00	0.00	0.00	0.00	0.00	0.00
gastric triacylglycerol lipase-like	LOC100760500	100760500	0.00	0.00	0.00	0.00	0.00	0.00	0.00	0.00	0.00	0.00	0.00	0.00
olfactory receptor 5M11-like	LOC100760501	100760501	0.00	0.00	0.00	0.00	0.00	0.00	0.00	0.00	0.00	0.00	0.00	0.00

calmodulin-like	LOC100760503	100760503	0.07	0.08	0.00	0.00	0.10	0.00	2.28	0.92	0.00	0.00	1.03	0.00
aldehyde oxidase 3	LOC100760504	100760504	0.00	0.00	0.00	0.00	0.00	0.00	0.00	0.00	0.00	0.00	0.00	0.00
cell division cycle protein 123 homolog pseudogene	LOC100760508	100760508	0.00	0.00	0.00	0.00	0.00	0.00	0.00	0.00	0.00	0.00	0.00	0.00
60S ribosomal protein L32 pseudogene	LOC100760511	100760511	0.52	0.24	0.00	0.24	0.14	0.32	8.35	1.83	2.48	1.16	1.03	2.80
6.8 kDa mitochondrial proteolipid	LOC100760515	100760515	13.42	14.39	9.17	11.73	13.31	10.44	378.25	311.11	322.94	305.95	360.18	437.43
ankyrin repeat family A protein 2-like	LOC100760519	100760519	0.00	0.00	0.00	0.00	0.00	0.00	0.00	0.00	0.00	0.00	0.00	0.00
mortality factor 4-like protein 2	LOC100760523	100760523	0.00	0.00	0.00	0.00	0.00	0.00	0.00	0.00	0.00	0.00	0.00	0.00
COMM domain-containing protein 9	LOC100760524	100760524	0.09	0.21	0.15	0.28	0.25	0.11	2.28	3.66	1.24	2.32	2.07	1.87
Ig lambda chain V-II region MGC	LOC100760526	100760526	0.00	0.00	0.00	0.00	0.00	0.00	0.00	0.00	0.00	0.00	0.00	0.00
olfactory receptor 4F3/4F16/4F29-like	LOC100760528	100760528	0.00	0.00	0.00	0.00	0.00	0.00	0.00	0.00	0.00	0.00	0.00	0.00
olfactory receptor 4D10	LOC100760529	100760529	0.00	0.00	0.00	0.00	0.00	0.00	0.00	0.00	0.00	0.00	0.00	0.00
uncharacterized LOC100760530	LOC100760530	100760530	0.00	0.00	0.00	0.00	0.00	0.00	0.00	0.00	0.00	0.00	0.00	0.00
fructose-bisphosphate aldolase A pseudogene	LOC100760534	100760534	1.02	1.10	0.62	0.34	1.24	0.57	29.62	30.20	16.15	9.27	35.19	14.02
cytochrome b5 reductase 4	LOC100760537	100760537	26.10	26.16	12.59	13.83	14.07	13.89	1773.52	1824.58	868.22	932.93	970.82	940.28
CMRF35-like molecule 3	LOC100760538	100760538	0.00	0.00	0.00	0.00	0.00	0.00	0.00	0.00	0.00	0.00	0.00	0.00
AP-1 complex-associated regulatory protein pseudogene	LOC100760544	100760544	0.07	0.00	0.23	0.00	0.00	0.00	0.76	0.00	2.48	0.00	0.00	0.00
olfactory receptor 51F2-like	LOC100760545	100760545	0.00	0.00	0.00	0.00	0.00	0.00	0.00	0.00	0.00	0.00	0.00	0.00
CD177 antigen-like	LOC100760551	100760551	0.17	0.19	0.04	0.02	0.12	0.08	1.52	0.00	0.00	1.16	1.03	1.87

nucleolar RNA helicase 2 pseudogene	LOC100760552	100760552	0.07	0.21	0.11	0.14	0.15	0.08	3.80	11.90	6.21	8.11	8.28	4.67
protein FAM47E-like	LOC100760554	100760554	0.00	0.00	0.00	0.00	0.00	0.00	0.00	0.00	0.00	0.00	0.00	0.00
ribosomal RNA-processing protein 7 homolog A pseudogene	LOC100760555	100760555	0.00	0.00	0.00	0.00	0.06	0.00	0.00	0.00	0.00	0.00	0.00	0.00
ornithine decarboxylase 2-like	LOC100760559	100760559	0.12	0.14	0.00	0.24	0.16	0.07	10.63	10.07	6.21	19.70	9.31	10.28
olfactory receptor 1361-like	LOC100760560	100760560	0.00	0.00	0.00	0.00	0.00	0.00	0.00	0.00	0.00	0.00	0.00	0.00
tRNA:m(4)X modification enzyme TRM13 homolog	LOC100760563	100760563	11.37	11.27	11.24	13.67	14.70	13.10	299.26	280.92	305.55	358.11	393.29	337.42
transmembrane protein 19	LOC100760567	100760567	18.62	18.86	16.84	18.01	21.01	21.03	1643.64	1673.60	1586.14	1687.39	1924.04	1902.05
geranylgeranyl transferase type-2 subunit beta-like	LOC100760579	100760579	0.00	0.00	0.00	0.06	0.10	0.04	0.00	0.92	0.00	3.48	4.14	2.80
60S ribosomal protein L21 pseudogene	LOC100760585	100760585	9.16	5.61	5.21	5.83	6.24	3.74	87.35	51.24	45.96	48.67	49.68	33.65
prefoldin subunit 2-like	LOC100760590	100760590	0.50	0.52	0.20	0.10	0.13	0.39	5.32	6.41	2.48	1.16	2.07	4.67
fragile X mental retardation 1 neighbor protein-like	LOC100760598	100760598	0.02	0.00	0.00	0.00	0.00	0.00	1.52	0.00	0.00	0.00	0.00	0.00
histone H2A type 1-like	LOC100760601	100760601	0.00	0.00	0.00	0.00	0.00	0.00	0.00	0.00	0.00	0.00	0.00	0.00
heat shock protein HSP 90-alpha pseudogene	LOC100760605	100760605	1.35	1.62	1.17	0.83	0.96	0.70	22.79	27.45	19.87	13.91	12.42	10.28
peptidyl-prolyl cis-trans isomerase FKBP3 pseudogene	LOC100760608	100760608	0.49	0.55	0.09	0.00	0.19	0.27	13.67	10.98	6.21	2.32	7.24	13.09
bax inhibitor 1 pseudogene	LOC100760613	100760613	0.75	0.57	0.67	1.23	0.50	0.87	15.19	10.07	12.42	24.34	10.35	19.63
interferon alpha-12	LOC100760616	100760616	0.03	0.00	0.00	0.00	0.00	0.00	0.76	0.00	0.00	0.00	0.00	0.00
chromosome unknown open reading frame, human C5orf52	LOC100760617	100760617	0.17	0.41	0.00	0.16	0.00	0.04	9.87	11.90	0.00	5.79	2.07	2.80
murinoglobulin-1-like	LOC100760619	100760619	0.00	0.00	0.00	0.00	0.00	0.00	0.00	0.00	0.00	0.00	0.00	0.00

high mobility group protein B1 pseudogene	LOC100760625	100760625	0.45	0.63	0.29	0.21	0.33	0.42	30.38	28.37	16.15	22.02	30.01	28.97
cell division control protein 42 homolog pseudogene	LOC100760628	100760628	0.00	0.00	0.00	0.00	0.00	0.00	0.00	0.00	0.00	0.00	0.00	0.00
keratin, type I cytoskeletal 42	LOC100760630	100760630	0.00	0.00	0.00	0.00	0.00	0.00	0.00	0.00	0.00	0.00	0.00	0.00
neuron-specific protein family member 2	LOC100760634	100760634	0.00	0.00	0.00	0.00	0.00	0.00	0.00	0.00	0.00	0.00	0.00	0.00
interferon-inducible GTPase 1-like	LOC100760635	100760635	0.00	0.01	0.05	0.01	0.00	0.00	0.00	0.00	0.00	0.00	0.00	0.00
uncharacterized LOC100760636	LOC100760636	100760636	0.00	0.00	0.07	0.06	0.00	0.03	0.76	0.00	2.48	2.32	2.07	0.93
olfactory receptor 4X2	LOC100760639	100760639	0.00	0.00	0.00	0.00	0.00	0.00	0.00	0.00	0.00	0.00	0.00	0.00
60S ribosomal protein L35a pseudogene	LOC100760640	100760640	0.82	0.75	0.69	0.23	0.19	0.27	5.32	3.66	2.48	1.16	1.03	1.87
chromodomain Y-like protein pseudogene	LOC100760645	100760645	0.00	0.00	0.00	0.00	0.00	0.00	0.00	0.00	0.00	0.00	0.00	0.00
olfactory receptor 145-like	LOC100760648	100760648	0.03	0.00	0.00	0.00	0.00	0.00	0.76	0.00	0.00	0.00	0.00	0.00
protein NipSnap homolog 3A	LOC100760650	100760650	0.00	0.00	0.00	0.00	0.00	0.00	0.00	0.00	0.00	0.00	0.00	0.00
olfactory receptor 5W2-like	LOC100760651	100760651	0.00	0.00	0.00	0.00	0.00	0.00	0.00	0.00	0.00	0.00	0.00	0.00
60S ribosomal protein L9 pseudogene	LOC100760652	100760652	0.00	0.00	0.00	0.00	0.08	0.30	0.00	0.00	0.00	1.16	4.14	8.41
hypothetical protein	LOC100760653	100760653	0.00	0.00	0.00	0.00	0.00	0.00	0.00	0.00	0.00	0.00	0.00	0.93
calcineurin B homologous protein 1 pseudogene	LOC100760654	100760654	0.09	0.04	0.00	0.09	0.12	0.04	0.76	0.00	0.00	1.16	1.03	0.00
40S ribosomal protein S13 pseudogene	LOC100760657	100760657	4.02	2.56	2.56	2.10	3.68	3.65	21.27	8.24	7.45	9.27	18.63	14.95
ficolin-1	LOC100760658	100760658	0.03	0.00	0.00	0.00	0.04	0.04	1.52	0.00	1.24	0.00	1.03	0.93
olfactory receptor 4D9-like	LOC100760660	100760660	0.00	0.00	0.00	0.00	0.00	0.00	0.00	0.00	0.00	0.00	0.00	0.00

acidic mammalian chitinase	LOC100760661	100760661	0.00	0.00	0.00	0.00	0.00	0.00	0.00	0.00	0.00	0.00	0.00	0.00
ubiquitin-conjugating enzyme E2 D3	LOC100760666	100760666	176.63	175.22	181.86	195.00	213.62	203.56	7266.51	7366.93	7631.38	8132.14	8864.66	8424.18
olfactory receptor 51D1	LOC100760668	100760668	0.00	0.00	0.00	0.00	0.00	0.00	0.00	0.00	0.00	0.00	0.00	0.00
glyceraldehyde-3-phosphate dehydrogenase pseudogene	LOC100760672	100760672	0.12	0.00	0.07	0.00	0.00	0.01	3.04	1.83	1.24	0.00	0.00	0.00
chromosome unknown open reading frame, human C6orf47	LOC100760681	100760681	29.48	32.09	35.10	27.88	21.71	19.67	720.04	771.37	804.87	642.04	504.04	445.84
multidrug resistance protein 1 pseudogene	LOC100760682	100760682	16.49	14.91	11.26	16.08	22.48	18.08	0.00	0.00	0.00	0.00	0.00	0.00
ADP-ribosylation factor 1 pseudogene	LOC100760687	100760687	26.00	25.90	22.71	22.23	21.51	22.10	296.98	277.26	249.66	236.42	209.07	232.73
60S acidic ribosomal protein P0 pseudogene	LOC100760688	100760688	107.67	115.50	112.00	87.27	59.76	58.78	822.58	1075.16	1084.34	902.80	548.54	515.94
histone H4	LOC100760690	100760690	0.09	0.02	0.14	0.19	0.00	0.00	0.00	0.00	0.00	1.16	2.07	0.00
hepatoma-derived growth factor-like protein 1	LOC100760697	100760697	0.00	0.00	0.00	0.00	0.00	0.00	0.00	0.00	0.00	0.00	0.00	0.00
prolactin-7A1-like	LOC100760700	100760700	0.00	0.00	0.00	0.00	0.00	0.00	0.00	0.00	0.00	0.00	0.00	0.00
zinc finger HIT domain-containing protein 1	LOC100760701	100760701	4.46	3.63	3.34	1.32	1.35	1.16	42.53	21.05	24.84	12.75	7.24	8.41
40S ribosomal protein S12-like	LOC100760717	100760717	0.00	0.00	0.00	0.00	0.06	0.00	0.00	0.00	0.00	0.00	1.03	0.00
septin-7 pseudogene	LOC100760721	100760721	3.55	3.09	2.88	2.70	2.89	2.14	164.82	144.58	125.45	124.00	124.20	114.03
uncharacterized LOC100760722	LOC100760722	100760722	0.00	0.00	0.00	0.00	0.00	0.00	1.52	0.00	0.00	0.00	0.00	0.00
guanylate cyclase 2G-like	LOC100760728	100760728	0.00	0.05	0.04	0.02	0.09	0.09	0.00	5.49	2.48	2.32	7.24	8.41
zinc finger protein 665-like	LOC100760730	100760730	1.69	1.92	2.53	2.87	3.09	2.99	239.25	264.44	356.48	402.15	438.83	421.54
olfactory receptor 51A4	LOC100760733	100760733	0.00	0.00	0.00	0.00	0.00	0.00	0.00	0.00	0.00	0.00	0.00	0.00

2-oxoisovalerate dehydrogenase subunit beta, mitochondrial pseudogene	LOC100760734	100760734	0.07	0.00	0.00	0.00	0.00	0.00	1.52	0.00	0.00	0.00	0.00	0.00
uncharacterized LOC100760739	LOC100760739	100760739	0.00	0.00	0.00	0.00	0.00	0.00	0.00	0.00	0.00	0.00	0.00	0.00
interferon-inducible GTPase 1-like	LOC100760741	100760741	0.01	0.00	0.00	0.00	0.00	0.01	0.00	0.00	0.00	1.16	0.00	0.00
ADP/ATP translocase 2 pseudogene	LOC100760743	100760743	0.26	0.52	0.80	0.13	0.47	0.21	4.56	9.15	13.66	2.32	8.28	3.74
cysteine-rich PDZ-binding protein pseudogene	LOC100760745	100760745	0.00	0.00	0.64	0.00	0.00	0.00	0.00	0.00	2.48	0.00	1.03	0.00
peptidyl-prolyl cis-trans isomerase A-like	LOC100760752	100760752	2.59	3.51	1.61	1.79	2.42	1.68	20.51	28.37	12.42	11.59	19.66	13.09
olfactory receptor 6F1-like	LOC100760753	100760753	0.00	0.00	0.00	0.00	0.00	0.00	0.00	0.00	0.00	0.00	0.00	0.00
dynein light chain 1, cytoplasmic-like	LOC100760754	100760754	0.00	0.00	0.00	0.00	0.00	0.00	0.00	0.00	0.00	0.00	0.00	0.00
arylacetamide deacetylase-like 4	LOC100760755	100760755	0.00	0.00	0.00	0.00	0.00	0.00	0.00	0.00	0.00	0.00	0.00	0.00
nucleophosmin pseudogene	LOC100760757	100760757	5.58	4.69	3.83	2.51	3.19	3.66	23.55	23.79	18.63	12.75	14.49	16.82
forkhead box protein I2-like	LOC100760759	100760759	0.00	0.00	0.00	0.00	0.00	0.00	0.00	0.00	0.00	0.00	0.00	0.00
microtubule-associated protein 6	LOC100760760	100760760	7.44	6.70	4.21	1.29	0.67	0.85	377.49	338.56	214.88	66.06	33.12	44.86
chromosome unknown open reading frame, human C2orf54	LOC100760762	100760762	0.00	0.00	0.00	0.00	0.00	0.00	0.00	0.00	0.00	0.00	0.00	0.00
pleckstrin homology domain-containing family F member 2	LOC100760765	100760765	3.42	8.52	6.10	5.46	5.62	4.99	37.22	50.33	36.02	40.56	34.15	35.52
keratin-associated protein 10-2-like	LOC100760771	100760771	0.00	0.00	0.00	0.00	0.00	0.00	0.00	0.00	0.00	0.00	0.00	0.00
PC-esterase domain-containing protein 1B-like	LOC100760773	100760773	0.15	0.03	0.04	0.00	0.00	0.00	6.08	0.92	1.24	0.00	1.03	0.93
chromosome unknown open reading frame, human C20orf112	LOC100760781	100760781	0.05	0.01	0.02	0.00	0.03	0.00	4.56	1.83	2.48	3.48	3.10	0.93
GRB2-associated-binding protein 2 pseudogene	LOC100760783	100760783	0.00	0.00	0.00	0.00	0.00	0.00	0.00	0.00	0.00	0.00	0.00	0.00

dachshund homolog 1	LOC100760786	100760786	0.00	0.00	0.00	0.03	0.00	0.00	0.00	0.00	0.00	1.16	0.00	0.00
protein FAM115C-like	LOC100760787	100760787	0.07	0.06	0.02	0.00	0.00	0.00	5.32	4.58	1.24	0.00	0.00	0.00
four and a half LIM domains protein 1	LOC100760792	100760792	0.40	0.40	0.41	0.51	0.34	0.63	21.27	19.22	18.63	25.50	16.56	29.91
polypyrimidine tract-binding protein 3 pseudogene	LOC100760793	100760793	0.68	0.88	0.38	0.49	0.61	0.36	50.13	38.43	19.87	19.70	23.80	14.02
class II histocompatibility antigen, M alpha chain	LOC100760794	100760794	2.90	2.94	2.07	2.00	1.17	1.40	129.12	127.19	88.19	84.60	51.75	56.08
olfactory receptor 1030	LOC100760796	100760796	0.00	0.00	0.00	0.00	0.00	0.00	0.00	0.00	0.00	0.00	0.00	0.00
4-trimethylaminobutyraldehyde dehydrogenase pseudogene	LOC100760798	100760798	4.25	3.92	2.82	2.72	3.56	3.56	202.04	193.99	141.60	125.16	167.67	171.04
chromosome unknown open reading frame, human C9orf117	LOC100760800	100760800	0.20	0.13	0.26	0.08	0.02	0.09	100.26	97.91	144.08	124.00	91.08	100.94
cytochrome b-245 heavy chain	LOC100760802	100760802	0.00	0.00	0.00	0.00	0.00	0.00	0.00	0.00	0.00	0.00	0.00	0.00
olfactory receptor 4F3/4F16/4F29-like	LOC100760819	100760819	0.00	0.00	0.00	0.00	0.00	0.00	0.00	0.00	0.00	0.00	0.00	0.00
macrophage-expressed gene 1 protein-like	LOC100760820	100760820	0.00	0.00	0.00	0.00	0.00	0.00	0.00	0.00	0.00	0.00	0.00	0.00
claudin-3-like	LOC100760821	100760821	0.00	0.00	0.00	0.00	0.00	0.00	0.00	0.00	0.00	0.00	0.00	0.00
actin-related protein T2	LOC100760823	100760823	0.00	0.00	0.00	0.00	0.00	0.00	0.00	0.00	0.00	0.00	0.00	0.00
ATP synthase subunit gamma, mitochondrial	LOC100760824	100760824	164.03	166.52	151.66	153.85	151.19	155.61	5249.18	5389.55	4849.11	4855.87	4860.30	4872.43
nucleophosmin pseudogene	LOC100760828	100760828	2.19	1.83	1.58	1.86	1.94	1.91	94.18	66.80	65.83	91.55	93.15	109.36
CMRF35-like molecule	LOC100760829	100760829	0.00	0.00	0.00	0.00	0.00	0.00	0.00	0.00	0.00	0.00	0.00	0.00
adenosylhomocysteinase pseudogene	LOC100760831	100760831	0.00	0.03	0.00	0.00	0.00	0.00	0.76	0.92	0.00	0.00	0.00	0.00
olfactory receptor 51F2-like	LOC100760836	100760836	0.00	0.00	0.00	0.00	0.00	0.00	0.00	0.00	0.00	0.00	0.00	0.00

uncharacterized LOC100760837	LOC100760837	100760837	1.07	1.16	1.62	1.73	1.55	2.26	67.60	86.01	94.40	115.89	104.53	148.61
uncharacterized LOC100760839	LOC100760839	100760839	0.00	0.00	0.00	0.00	0.00	0.00	0.00	0.00	0.00	0.00	0.00	0.00
olfactory receptor 52N2	LOC100760842	100760842	0.00	0.00	0.00	0.00	0.00	0.00	0.00	0.00	0.00	0.00	0.00	0.00
olfactory receptor 2B11	LOC100760843	100760843	0.00	0.00	0.00	0.00	0.00	0.00	0.00	0.00	0.00	0.00	0.00	0.00
aspartate--tRNA ligase, cytoplasmic pseudogene	LOC100760845	100760845	3.78	4.34	3.74	3.42	3.92	3.12	34.94	31.11	37.26	33.61	28.98	21.50
olfactory receptor 1361	LOC100760851	100760851	0.00	0.00	0.00	0.00	0.00	0.00	0.00	0.00	0.00	0.00	0.00	0.00
zinc finger protein 431-like	LOC100760852	100760852	0.05	0.00	0.04	0.04	0.11	0.03	2.28	1.83	1.24	2.32	5.17	0.93
alpha-1,3-mannosyl-glycoprotein 4-beta-N-acetylglucosaminyltransferase-like protein homolog	LOC100760854	100760854	0.00	0.00	0.03	0.00	0.00	0.00	0.00	0.00	1.24	1.16	0.00	0.00
olfactory receptor 4A15-like	LOC100760855	100760855	0.00	0.00	0.00	0.00	0.00	0.00	0.00	0.00	0.00	0.00	0.00	0.00
olfactory receptor 2H2-like	LOC100760861	100760861	0.00	0.00	0.00	0.00	0.00	0.00	0.00	0.00	0.00	0.00	0.00	0.00
proto-oncogene Mas	LOC100760876	100760876	0.03	0.00	0.00	0.00	0.00	0.00	0.76	0.00	0.00	0.00	0.00	0.00
uncharacterized LOC100760888	LOC100760888	100760888	0.00	0.00	0.00	0.00	0.00	0.00	0.00	0.00	0.00	0.00	0.00	0.00
cyclin-dependent kinases regulatory subunit 1	LOC100760892	100760892	2.19	2.54	2.21	2.03	2.71	1.47	2.28	2.75	1.24	2.32	4.14	0.93
T-complex protein 1 subunit beta pseudogene	LOC100760901	100760901	0.07	0.00	0.00	0.00	0.00	0.05	1.52	0.00	0.00	0.00	0.00	0.93
interferon alpha-9-like	LOC100760906	100760906	0.00	0.00	0.00	0.00	0.00	0.00	0.00	0.00	0.00	0.00	0.00	0.00
chromosome unknown open reading frame, human C3orf30	LOC100760908	100760908	0.00	0.00	0.00	0.00	0.00	0.00	0.00	0.00	0.00	0.00	0.00	0.00
alpha-1-inhibitor 3-like	LOC100760909	100760909	0.00	0.00	0.00	0.00	0.00	0.00	0.00	0.00	0.00	0.00	0.00	0.00
40S ribosomal protein S3 pseudogene	LOC100760916	100760916	2.56	3.26	1.97	1.94	2.51	1.00	46.33	59.48	34.78	33.61	43.47	17.76

ubiquitin carboxyl-terminal hydrolase isozyme L5 pseudogene	LOC100760919	100760919	0.16	0.53	0.36	0.19	0.28	0.20	3.04	9.15	4.97	3.48	5.17	3.74
60S ribosomal protein L29	LOC100760923	100760923	806.48	818.29	809.08	504.16	247.09	284.84	7045.49	6812.42	6860.05	4259.03	2030.64	2525.48
arylacetamide deacetylase-like 4	LOC100760924	100760924	0.00	0.00	0.00	0.00	0.00	0.00	0.00	0.00	0.00	0.00	0.00	0.00
interferon-inducible GTPase 1-like	LOC100760925	100760925	0.00	0.00	0.00	0.00	0.00	0.00	0.00	0.00	0.00	0.00	0.00	0.00
olfactory receptor 4B1	LOC100760928	100760928	0.00	0.00	0.00	0.00	0.00	0.00	0.00	0.00	0.00	0.00	0.00	0.00
tubulin alpha-1C chain-like	LOC100760929	100760929	2.18	2.09	2.30	1.46	1.98	1.09	39.50	37.52	44.72	31.29	37.26	20.56
GRB2-associated-binding protein 2 pseudogene	LOC100760936	100760936	0.00	0.00	0.03	0.03	0.00	0.00	0.00	0.00	1.24	1.16	0.00	0.00
protein NipSnap homolog 3B	LOC100760938	100760938	13.68	14.13	9.18	10.75	10.34	10.76	525.60	546.28	356.48	404.46	399.50	414.99
olfactory receptor 5W2-like	LOC100760939	100760939	0.00	0.00	0.00	0.00	0.00	0.00	0.00	0.00	0.00	0.00	0.00	0.00
killer cell lectin-like receptor subfamily B member 1B allele C	LOC100760941	100760941	0.00	0.05	0.07	0.00	0.00	0.00	0.76	1.83	1.24	0.00	0.00	0.00
PI-PLC X domain-containing protein 1-like	LOC100760948	100760948	0.00	0.00	0.00	0.00	0.00	0.00	0.76	0.92	0.00	0.00	0.00	1.87
chitinase-like protein 4	LOC100760950	100760950	0.00	0.00	0.00	0.00	0.00	0.00	0.76	0.00	0.00	0.00	0.00	0.00
40S ribosomal protein S27-like	LOC100760954	100760954	0.21	0.13	0.18	0.20	0.22	0.33	20.51	10.07	13.66	17.38	16.56	24.30
chromosome unknown open reading frame, human C2orf44	LOC100760958	100760958	7.15	6.72	6.98	8.90	8.61	8.44	572.69	536.21	552.73	705.78	681.02	673.90
60S ribosomal protein L7a pseudogene	LOC100760962	100760962	0.06	0.00	0.00	0.13	0.00	0.00	0.76	0.00	0.00	2.32	0.00	0.00
RNA-binding protein PNO1 pseudogene	LOC100760969	100760969	0.19	0.17	0.45	0.21	0.38	0.21	2.28	1.83	6.21	2.32	4.14	2.80
protein regulator of cytokinesis 1 pseudogene	LOC100760973	100760973	0.10	0.15	0.03	0.10	0.02	0.02	4.56	7.32	1.24	4.64	1.03	0.93
proteasome subunit beta type-3 pseudogene	LOC100760976	100760976	0.05	0.12	0.00	0.00	0.00	0.00	0.76	1.83	0.00	0.00	0.00	0.00

60S ribosomal protein L23-like	LOC100760980	100760980	1.10	2.89	0.37	0.55	1.50	0.99	6.84	18.30	2.48	3.48	9.31	5.61
chromosome unknown open reading frame, human C8orf37	LOC100760984	100760984	4.95	4.64	4.08	4.13	5.05	5.27	183.81	172.94	132.90	157.61	189.40	182.26
SOSS complex subunit B2 pseudogene	LOC100760985	100760985	0.06	0.00	0.10	0.00	0.00	0.00	0.76	0.00	1.24	0.00	0.00	0.00
40S ribosomal protein S15 pseudogene	LOC100760988	100760988	0.00	0.00	0.00	0.00	0.00	0.00	0.00	0.00	0.00	0.00	0.00	0.00
prolactin-2A1-like	LOC100760990	100760990	0.00	0.00	0.00	0.00	0.00	0.00	0.00	0.00	0.00	0.00	0.00	0.00
40S ribosomal protein S4, X isoform	LOC100760991	100760991	0.00	0.00	0.00	0.00	0.00	0.00	0.00	0.00	0.00	0.00	0.00	0.00
probable G-protein coupled receptor 141	LOC100760993	100760993	0.00	0.00	0.00	0.00	0.00	0.00	0.00	0.00	0.00	0.00	0.00	0.00
interferon lambda-2	LOC100760996	100760996	0.00	0.00	0.00	0.00	0.00	0.00	0.00	0.00	0.00	0.00	0.00	0.00
60S ribosomal protein L7a pseudogene	LOC100761000	100761000	6.52	7.18	6.47	6.01	5.18	4.50	34.94	36.60	39.75	30.13	17.59	24.30
T-complex protein 1 subunit delta pseudogene	LOC100761006	100761006	0.00	0.00	0.00	0.00	0.00	0.00	0.00	0.00	0.00	0.00	0.00	0.00
coiled-coil domain-containing protein 121-like	LOC100761009	100761009	0.00	0.00	0.00	0.00	0.00	0.00	0.00	0.00	0.00	0.00	0.00	0.00
60S ribosomal protein L18a pseudogene	LOC100761010	100761010	0.00	0.00	0.00	0.00	0.00	0.00	0.00	0.00	0.00	0.00	0.00	0.00
E3 ubiquitin-protein ligase RAD18-like	LOC100761012	100761012	0.00	0.00	0.00	0.00	0.00	0.00	0.00	0.00	0.00	0.00	0.00	0.00
mitochondrial carrier homolog 2 pseudogene	LOC100761021	100761021	3.02	3.49	4.18	3.19	3.61	3.29	60.00	71.37	81.98	60.26	71.41	68.23
chromosome unknown open reading frame, human C15orf52	LOC100761022	100761022	3.49	3.42	2.20	2.38	1.94	2.26	295.46	307.45	202.46	199.33	169.74	199.08
olfactory receptor 51L1	LOC100761024	100761024	0.00	0.00	0.00	0.00	0.00	0.00	0.00	0.00	0.00	0.00	0.00	0.00
40S ribosomal protein S5-like	LOC100761031	100761031	0.00	0.00	0.00	0.00	0.00	0.00	0.00	0.00	0.00	0.00	0.00	0.93
interferon-inducible GTPase 1-like	LOC100761033	100761033	0.00	0.00	0.00	0.00	0.00	0.00	0.00	0.00	0.00	0.00	0.00	0.00

insulin-induced gene 2 protein-like	LOC100761035	100761035	0.85	0.90	1.05	0.48	0.66	0.95	4.56	3.66	6.21	0.00	3.10	2.80
serpin B6-like	LOC100761037	100761037	0.16	0.12	0.05	0.15	0.04	0.08	19.75	20.13	7.45	11.59	8.28	3.74
eukaryotic translation initiation factor 1A, X-chromosomal-like	LOC100761040	100761040	1.03	0.38	0.00	1.06	1.19	0.83	6.08	6.41	1.24	3.48	7.24	2.80
tudor and KH domain-containing protein pseudogene	LOC100761043	100761043	0.87	0.26	1.84	1.86	2.52	1.85	13.67	1.83	34.78	34.77	47.61	37.39
olfactory receptor 14C36-like	LOC100761044	100761044	0.00	0.00	0.00	0.00	0.00	0.00	0.00	0.00	0.00	0.00	0.00	0.00
nogo-B receptor	LOC100761045	100761045	0.00	0.00	0.00	0.00	0.00	0.00	0.00	0.00	0.00	0.00	0.00	0.00
arylacetamide deacetylase-like 4	LOC100761046	100761046	0.00	0.00	0.00	0.00	0.00	0.00	0.00	0.00	0.00	0.00	0.00	0.00
2'-5'-oligoadenylate synthase 1A-like	LOC100761048	100761048	0.00	0.00	0.00	0.00	0.00	0.00	0.00	0.00	0.00	0.00	0.00	0.00
phospholipase A2-like	LOC100761051	100761051	0.00	0.00	0.00	0.00	0.00	0.00	0.00	0.00	0.00	0.00	0.00	0.00
chromosome unknown open reading frame, human C4orf27	LOC100761052	100761052	36.22	37.09	41.47	48.43	48.12	48.21	1061.08	1130.98	1227.18	1419.68	1400.34	1405.74
replication protein A 70 kDa DNA-binding subunit-like	LOC100761053	100761053	49.92	56.10	70.31	72.70	85.06	82.66	0.00	0.00	0.00	0.00	1.03	0.00
voltage-dependent anion-selective channel protein 1 pseudogene	LOC100761055	100761055	0.00	0.00	0.00	0.00	0.00	0.00	0.00	0.00	0.00	0.00	0.00	0.00
DNA-binding protein inhibitor ID-2 pseudogene	LOC100761056	100761056	0.00	0.00	0.00	0.00	0.00	0.00	0.00	0.00	0.00	0.00	0.00	0.00
40S ribosomal protein S18-like	LOC100761059	100761059	30.39	28.49	23.85	15.65	5.12	8.03	202.04	181.18	145.32	99.67	35.19	55.15
IQ and AAA domain-containing protein 1-like	LOC100761064	100761064	0.00	0.00	0.00	0.00	0.00	0.00	0.76	0.00	0.00	0.00	0.00	0.00
chromosome unknown open reading frame, human C19orf43	LOC100761069	100761069	55.81	51.02	45.39	39.30	37.12	40.43	1595.03	1479.61	1302.95	1126.47	1041.20	1133.75
putative germ cell-less protein-like 1-like	LOC100761074	100761074	0.00	0.00	0.00	0.00	0.00	0.00	0.00	0.00	0.00	0.00	0.00	0.00
olfactory receptor-like protein OLF3	LOC100761075	100761075	0.00	0.00	0.00	0.00	0.00	0.00	0.00	0.00	0.00	0.00	0.00	0.00

NTF2-related export protein 2-like	LOC100 761079	100761079	0.00	0.00	0.00	0.00	0.00	0.00	0.00	0.00	0.00	0.00	0.00	0.00
olfactory receptor 4C15-like	LOC100 761081	100761081	0.00	0.00	0.00	0.00	0.00	0.00	0.00	0.00	0.00	0.00	0.00	0.00
class II histocompatibility antigen, M beta 1 chain	LOC100 761082	100761082	1.02	1.04	0.52	0.43	0.27	0.30	41.02	40.26	22.36	17.38	10.35	12.15
gastric triacylglycerol lipase	LOC100 761083	100761083	0.00	0.00	0.00	0.00	0.00	0.00	0.00	0.00	0.00	0.00	0.00	0.00
olfactory receptor 1030-like	LOC100 761084	100761084	0.00	0.00	0.00	0.00	0.00	0.00	0.00	0.00	0.00	0.00	0.00	0.00
DNA primase large subunit pseudogene	LOC100 761095	100761095	0.09	0.05	0.08	0.18	0.13	0.12	3.04	1.83	3.73	6.95	5.17	4.67
isocitrate dehydrogenase [NADP] cytoplasmic-like	LOC100 761102	100761102	0.12	0.00	0.08	0.00	0.07	0.06	6.84	3.66	7.45	2.32	3.10	2.80
immunoglobulin omega chain	LOC100 761106	100761106	0.00	0.00	0.00	0.00	0.00	0.00	0.00	0.00	0.00	0.00	0.00	0.00
olfactory receptor 4F3/4F16/4F29	LOC100 761108	100761108	0.00	0.00	0.00	0.00	0.00	0.00	0.00	0.00	0.00	0.00	0.00	0.00
olfactory receptor 5A1	LOC100 761109	100761109	0.00	0.00	0.00	0.00	0.00	0.00	0.00	0.00	0.00	0.00	0.00	0.00
40S ribosomal protein S25 pseudogene	LOC100 761110	100761110	0.26	0.16	0.00	0.22	0.56	0.00	0.76	0.92	0.00	1.16	3.10	0.00
PR domain zinc finger protein 16-like	LOC100 761112	100761112	1.34	1.24	1.57	1.26	0.44	0.80	14.43	14.64	16.15	10.43	4.14	7.48
N-acylneuraminate-9-phosphatase-like	LOC100 761114	100761114	0.00	0.00	0.00	0.00	0.00	0.00	0.00	0.00	0.00	0.00	0.00	0.00
olfactory receptor 8B3-like	LOC100 761118	100761118	0.00	0.00	0.00	0.00	0.00	0.00	0.00	0.00	0.00	0.00	0.00	0.00
zinc finger protein 431-like	LOC100 761126	100761126	4.24	4.02	5.27	5.57	6.62	4.91	360.02	339.48	443.43	456.61	542.33	410.32
ubiquitin carboxyl-terminal hydrolase 17-like protein 6	LOC100 761132	100761132	0.07	0.06	0.03	0.03	0.04	0.05	3.04	1.83	1.24	1.16	2.07	2.80
zinc finger protein 420-like	LOC100 761135	100761135	0.49	0.26	0.50	0.25	1.09	1.00	23.55	11.90	21.12	16.22	60.03	47.67
	LOC100 761140	100761140	0.00	0.00	0.00	0.00	0.00	0.00	0.00	0.00	0.00	0.00	0.00	0.00

alpha-1,3-mannosyl-glycoprotein 4-beta-N-acetylglucosaminyltransferase-like protein homolog	LOC100761144	100761144	0.02	0.02	0.03	0.03	0.03	0.00	0.76	0.92	2.48	2.32	1.03	0.00
hemoglobin subunit alpha pseudogene	LOC100761145	100761145	0.00	0.00	0.00	0.00	0.00	0.00	0.00	0.00	0.00	0.00	0.00	0.00
transcription initiation factor TFIID subunit 7-like	LOC100761147	100761147	0.08	0.03	0.00	0.00	0.11	0.03	2.28	0.92	0.00	0.00	3.10	1.87
ran guanine nucleotide release factor pseudogene	LOC100761149	100761149	0.00	0.00	0.00	0.00	0.00	0.00	0.00	0.00	0.00	0.00	0.00	0.00
speckle-type POZ protein-like	LOC100761158	100761158	0.00	0.00	0.00	0.00	0.00	0.00	0.00	0.00	0.00	0.00	0.00	0.00
protein SET-like	LOC100761160	100761160	76.17	75.80	91.42	86.65	79.73	96.32	1780.36	1847.45	2356.24	2235.56	2043.06	2446.97
zinc finger protein 431-like	LOC100761161	100761161	0.97	0.88	0.86	1.19	1.26	1.25	141.27	130.85	139.11	178.47	201.82	186.93
chromosome unknown open reading frame, human C20orf195	LOC100761165	100761165	0.00	0.00	0.00	0.00	0.00	0.00	0.00	0.00	0.00	0.00	0.00	0.00
sentrin-specific protease 2 pseudogene	LOC100761167	100761167	0.00	0.00	0.00	0.00	0.00	0.00	0.00	0.00	0.00	0.00	0.00	0.00
40S ribosomal protein S28 pseudogene	LOC100761171	100761171	1.91	1.74	0.82	1.24	1.38	0.32	6.84	5.49	2.48	4.64	6.21	1.87
60S ribosomal protein L8 pseudogene	LOC100761175	100761175	18.97	18.84	17.84	12.25	15.91	13.08	273.43	277.26	265.81	169.20	231.84	186.93
olfactory receptor 4P4-like	LOC100761177	100761177	0.00	0.00	0.00	0.00	0.00	0.00	0.00	0.00	0.00	0.00	0.00	0.00
histone H3.1-like	LOC100761179	100761179	0.00	0.01	0.03	0.00	0.00	0.00	0.76	0.00	0.00	0.00	0.00	0.00
cytochrome c oxidase subunit 7A-related protein, mitochondrial pseudogene	LOC100761180	100761180	0.19	0.11	0.00	0.00	0.00	0.25	1.52	1.83	1.24	0.00	0.00	1.87
ribose-phosphate pyrophosphokinase 1-like	LOC100761185	100761185	0.16	0.22	0.11	0.10	0.16	0.04	3.04	4.58	2.48	2.32	3.10	0.93
LIM domain-containing protein ajuba pseudogene	LOC100761189	100761189	0.00	0.00	0.00	0.00	0.00	0.00	0.00	0.00	0.00	0.00	0.00	0.00
Krueppel-like factor 10 pseudogene	LOC100761192	100761192	0.18	0.00	0.04	0.00	0.06	0.00	9.11	1.83	1.24	2.32	2.07	0.93
pseudouridine-5'-monophosphatase-like	LOC100761202	100761202	0.00	0.00	0.00	0.00	0.00	0.00	0.00	0.00	0.00	0.00	0.00	0.00

putative zinc finger protein 724	LOC100 761204	100761204	3.52	3.29	3.47	3.76	3.19	3.10	466.36	434.64	459.57	499.49	418.13	406.58
cytochrome c oxidase subunit 4 isoform 1, mitochondrial pseudogene	LOC100 761210	100761210	0.00	0.00	0.00	0.00	0.00	0.00	0.00	0.00	0.00	0.00	0.00	0.00
olfactory receptor 52E1-like	LOC100 761211	100761211	0.00	0.00	0.00	0.00	0.00	0.00	0.00	0.00	0.00	0.00	0.00	0.00
egl nine homolog 3 pseudogene	LOC100 761212	100761212	0.00	0.00	0.00	0.00	0.00	0.00	0.00	0.00	0.00	0.00	0.00	0.00
GRB2-associated-binding protein 2 pseudogene	LOC100 761213	100761213	0.03	0.00	0.00	0.00	0.00	0.00	2.28	2.75	0.00	0.00	1.03	0.00
signal peptidase complex subunit 2 pseudogene	LOC100 761214	100761214	0.41	0.18	0.17	0.72	0.14	0.06	8.35	5.49	4.97	10.43	4.14	0.93
olfactory receptor 4B1-like	LOC100 761216	100761216	0.00	0.00	0.00	0.00	0.00	0.00	0.00	0.00	0.00	0.00	0.00	0.00
olfactory receptor 13A1-like	LOC100 761217	100761217	0.00	0.00	0.00	0.00	0.00	0.00	0.00	0.00	0.00	0.00	0.00	0.00
ubiquilin-3	LOC100 761223	100761223	0.00	0.00	0.00	0.00	0.00	0.00	0.00	0.92	0.00	0.00	0.00	0.00
olfactory receptor 1020-like	LOC100 761227	100761227	0.00	0.00	0.00	0.00	0.00	0.00	0.00	0.00	0.00	0.00	0.00	0.00
stathmin domain-containing protein 1-like	LOC100 761228	100761228	0.08	0.00	0.00	0.00	0.12	0.00	0.00	0.00	0.00	0.00	0.00	0.00
high mobility group protein B1 pseudogene	LOC100 761236	100761236	0.27	0.36	0.44	0.38	0.20	0.27	3.80	3.66	6.21	2.32	2.07	1.87
odorant-binding protein 2a-like	LOC100 761241	100761241	0.00	0.00	0.00	0.00	0.00	0.00	0.00	0.00	0.00	0.00	0.00	0.00
olfactory receptor 52I2	LOC100 761243	100761243	0.00	0.00	0.00	0.00	0.00	0.00	0.00	0.00	0.00	0.00	0.00	0.00
zinc finger HIT domain-containing protein 3 pseudogene	LOC100 761246	100761246	3.36	4.16	1.66	1.14	1.60	1.27	78.99	82.35	37.26	23.18	24.84	19.63
protein phosphatase 1 regulatory subunit 26-like	LOC100 761250	100761250	0.00	0.00	0.00	0.00	0.00	0.00	0.00	0.00	0.00	0.00	0.00	0.00
chromosome unknown open reading frame, human C1orf54	LOC100 761253	100761253	0.05	0.06	0.00	0.08	0.00	0.00	2.28	3.66	1.24	1.16	4.14	1.87
uncharacterized LOC100761255	LOC100 761255	100761255	63.86	52.04	53.39	40.27	66.45	77.31	142.03	107.97	122.97	93.87	141.79	142.07

probable ATP-dependent RNA helicase DDX5	LOC100761256	100761256	0.00	0.00	0.00	0.00	0.03	0.00	0.00	0.00	0.00	0.00	1.03	0.00
proto-oncogene FRAT1-like	LOC100761257	100761257	0.00	0.00	0.00	0.00	0.00	0.00	0.00	0.00	0.00	0.00	0.00	0.00
40S ribosomal protein S2 pseudogene	LOC100761261	100761261	0.00	0.00	0.00	0.00	0.00	0.00	0.00	0.00	0.00	0.00	0.00	0.00
acidic leucine-rich nuclear phosphoprotein 32 family member A pseudogene	LOC100761267	100761267	0.09	0.00	0.00	0.00	0.06	0.00	1.52	0.00	0.00	0.00	1.03	0.00
histone H2B type 1	LOC100761268	100761268	1.93	2.19	1.47	1.04	0.49	0.94	12.91	14.64	11.18	8.11	4.14	8.41
amelogenin, X isoform	LOC100761271	100761271	0.00	0.00	0.00	0.00	0.00	0.00	0.00	0.00	0.00	0.00	0.00	0.00
olfactory receptor 1019-like	LOC100761273	100761273	0.00	0.00	0.00	0.00	0.00	0.00	0.00	0.00	0.00	0.00	0.00	0.00
proteasome subunit beta type-6-like	LOC100761275	100761275	0.00	0.00	0.00	0.00	0.00	0.00	0.00	0.00	0.00	0.00	0.00	0.00
coiled-coil-helix-coiled-coil-helix domain-containing protein 2, mitochondrial pseudogene	LOC100761277	100761277	0.00	0.00	0.00	0.00	0.00	0.00	0.00	0.00	0.00	0.00	0.00	0.00
prolactin-2C2-like	LOC100761279	100761279	0.00	0.00	0.00	0.00	0.00	0.00	0.00	0.00	0.00	0.00	0.00	0.00
protein TSSC1-like	LOC100761280	100761280	0.00	0.00	0.00	0.00	0.00	0.00	0.00	0.00	0.00	0.00	0.00	0.00
DNA-directed RNA polymerases I and III subunit RPAC2-like	LOC100761281	100761281	0.00	0.00	0.00	0.00	0.00	0.00	0.76	0.00	0.00	1.16	1.03	0.00
L-lactate dehydrogenase A chain pseudogene	LOC100761286	100761286	0.00	0.00	0.00	0.00	0.00	0.00	0.00	0.00	0.00	0.00	0.00	0.00
beta-adrenergic receptor kinase 2	LOC100761290	100761290	0.00	0.00	0.00	0.00	0.00	0.00	0.00	0.00	0.00	0.00	0.00	0.00
40S ribosomal protein S27-like pseudogene	LOC100761291	100761291	4.79	5.13	5.67	5.06	7.01	1.74	15.19	18.30	12.42	12.75	14.49	10.28
40S ribosomal protein S6-like	LOC100761293	100761293	0.00	0.06	0.08	0.00	0.06	0.00	0.00	0.92	0.00	0.00	1.03	0.00
zinc finger protein 1 homolog pseudogene	LOC100761296	100761296	0.50	0.40	0.57	0.68	0.43	0.53	13.67	9.15	14.91	17.38	10.35	14.02
ribosome biogenesis protein NSA2 homolog	LOC100761299	100761299	0.91	0.48	0.11	0.47	0.24	0.38	21.27	10.98	2.48	11.59	6.21	11.22

carcinoembryonic antigen-related cell adhesion molecule 3-like	LOC100761302	100761302	0.00	0.00	0.00	0.00	0.00	0.00	0.00	0.00	0.00	0.00	0.00	0.00
selenoprotein K pseudogene	LOC100761305	100761305	0.22	0.26	0.00	0.00	0.00	0.29	0.76	0.92	0.00	0.00	0.00	0.93
zinc finger protein 501-like	LOC100761311	100761311	0.86	0.73	0.88	0.73	1.19	1.16	52.41	47.58	52.17	49.83	76.59	69.17
poly(A) polymerase alpha pseudogene	LOC100761312	100761312	0.94	0.82	0.61	0.33	0.74	0.42	68.36	67.71	44.72	39.40	60.03	59.82
olfactory receptor 52E2	LOC100761314	100761314	0.00	0.00	0.00	0.00	0.00	0.00	0.00	0.00	0.00	0.00	0.00	0.00
keratin-associated protein 26-1-like	LOC100761318	100761318	0.00	0.00	0.00	0.00	0.00	0.00	0.00	0.00	0.00	0.00	0.00	0.00
heterogeneous nuclear ribonucleoprotein A3 pseudogene	LOC100761320	100761320	0.03	0.00	0.08	0.02	0.26	0.06	0.76	0.00	0.00	0.00	4.14	0.93
E3 ubiquitin-protein ligase TRIM39-like	LOC100761321	100761321	0.97	0.83	1.10	0.97	1.17	0.95	2.28	0.92	3.73	3.48	3.10	6.54
hypothetical protein	LOC100761322	100761322	0.00	0.00	0.00	0.00	0.00	0.00	0.00	0.00	0.00	0.00	0.00	0.00
interferon-inducible GTPase 1	LOC100761326	100761326	0.00	0.00	0.00	0.00	0.00	0.00	0.00	0.00	0.00	0.00	0.00	0.93
zinc finger protein 805-like	LOC100761328	100761328	9.53	8.82	8.36	7.48	6.79	7.06	392.68	364.18	363.93	309.43	277.38	292.55
olfactory receptor 8S1	LOC100761332	100761332	0.00	0.00	0.00	0.00	0.00	0.00	0.00	0.00	0.00	0.00	0.00	0.00
beta-defensin 43-like	LOC100761338	100761338	0.00	0.00	0.00	0.00	0.00	0.00	0.00	0.00	0.00	0.00	0.00	0.00
L-aminoadipate-semialdehyde dehydrogenase-phosphopantetheinyl transferase pseudogene	LOC100761344	100761344	0.02	0.10	0.00	0.00	0.09	0.04	0.00	1.83	0.00	0.00	2.07	0.93
uncharacterized LOC100761345	LOC100761345	100761345	10.08	12.28	5.66	5.57	4.28	3.76	0.76	0.92	0.00	1.16	2.07	3.74
olfactory receptor 5B3-like	LOC100761347	100761347	0.00	0.00	0.00	0.00	0.00	0.00	0.00	0.00	0.00	0.00	0.00	0.00
keratin, type I cytoskeletal 24-like	LOC100761350	100761350	0.00	0.00	0.00	0.00	0.00	0.00	0.00	0.00	0.00	0.00	0.00	0.00
chromosome unknown open reading frame, human C2orf76	LOC100761351	100761351	3.10	2.39	2.25	2.64	3.08	2.39	56.21	51.24	33.54	42.88	42.43	42.06

ubiquitin-conjugating enzyme E2 variant 1 pseudogene	LOC100761366	100761366	0.00	0.00	0.00	0.00	0.00	0.00	0.00	0.00	0.00	0.00	0.00	0.00
olfactory receptor-like protein OLF3	LOC100761367	100761367	0.00	0.00	0.00	0.00	0.00	0.00	0.00	0.00	0.00	0.00	0.00	0.00
grpE protein homolog 2, mitochondrial	LOC100761370	100761370	13.63	14.49	13.53	13.23	12.88	12.26	75.95	86.01	80.74	86.92	63.13	65.43
olfactory receptor 4C15	LOC100761372	100761372	0.00	0.00	0.00	0.00	0.00	0.00	0.00	0.00	0.00	0.00	0.00	0.00
rano class II histocompatibility antigen, A beta chain-like	LOC100761373	100761373	0.00	0.08	0.00	0.00	0.00	0.00	0.00	0.92	0.00	0.00	0.00	0.00
olfactory receptor 1030	LOC100761375	100761375	0.00	0.00	0.00	0.00	0.00	0.00	0.00	0.00	0.00	0.00	0.00	0.00
olfactory receptor 4F15-like	LOC100761376	100761376	0.00	0.00	0.00	0.00	0.12	0.02	0.00	0.00	0.00	0.00	0.00	0.00
chromosome unknown open reading frame, human CXorf27	LOC100761382	100761382	0.00	0.00	0.00	0.00	0.00	0.00	0.00	0.00	0.00	0.00	0.00	0.00
alpha-1-antitrypsin	LOC100761387	100761387	0.00	0.00	0.00	0.00	0.00	0.00	0.00	0.00	0.00	0.00	0.00	0.00
olfactory receptor 8D4	LOC100761391	100761391	0.00	0.00	0.00	0.00	0.00	0.00	0.00	0.00	0.00	0.00	0.00	0.00
ubiquitin-40S ribosomal protein S27a pseudogene	LOC100761394	100761394	1.39	1.42	1.63	1.21	1.05	0.45	24.31	23.79	24.84	19.70	16.56	10.28
BTB/POZ domain-containing protein 10 pseudogene	LOC100761395	100761395	0.05	0.03	0.04	0.07	0.08	0.12	1.52	0.92	1.24	2.32	2.07	3.74
immunoglobulin omega chain-like	LOC100761398	100761398	0.00	0.00	0.00	0.00	0.00	0.00	0.00	0.00	0.00	0.00	0.00	0.00
pyrethroid hydrolase Ces2e-like	LOC100761399	100761399	0.02	0.00	0.00	0.00	0.00	0.00	0.00	0.00	0.00	0.00	0.00	0.00
putative olfactory receptor GPCRLTM7	LOC100761400	100761400	0.00	0.00	0.00	0.00	0.00	0.00	0.00	0.00	0.00	0.00	0.00	0.00
olfactory receptor 5AN1-like	LOC100761401	100761401	0.00	0.00	0.00	0.00	0.00	0.00	0.00	0.92	0.00	0.00	0.00	0.00
NADH dehydrogenase [ubiquinone] 1 beta subcomplex subunit 4 pseudogene	LOC100761403	100761403	0.08	0.10	0.00	0.00	0.23	0.00	0.76	2.75	1.24	1.16	5.17	0.00
C-type lectin domain family 2 member E-like	LOC100761404	100761404	0.28	0.35	0.29	0.34	0.66	0.79	15.95	20.13	16.15	19.70	41.40	44.86

olfactory receptor 143-like	LOC100 761408	100761408	0.00	0.00	0.00	0.00	0.00	0.00	0.00	0.00	0.00	0.00	0.00	0.00
chromosome unknown open reading frame, human C2orf57	LOC100 761409	100761409	0.00	0.00	0.00	0.00	0.00	0.00	0.00	0.00	0.00	0.00	0.00	0.00
zinc finger CCHC domain-containing protein 10 pseudogene	LOC100 761412	100761412	1.23	0.56	0.51	0.49	0.96	0.58	11.39	6.41	6.21	6.95	10.35	6.54
olfactory receptor 12-like	LOC100 761417	100761417	0.00	0.00	0.00	0.00	0.00	0.00	0.00	0.00	0.00	0.00	0.00	0.00
disks large homolog 5-like	LOC100 761422	100761422	0.00	0.00	0.00	0.00	0.00	0.00	0.00	0.00	0.00	0.00	0.00	0.00
olfactory receptor 1361-like	LOC100 761431	100761431	0.00	0.00	0.00	0.00	0.00	0.00	0.00	0.00	0.00	0.00	0.00	0.00
carbonic anhydrase 6-like	LOC100 761433	100761433	0.00	0.00	0.00	0.00	0.00	0.00	0.00	0.00	0.00	0.00	0.00	0.00
sphingomyelin phosphodiesterase 3	LOC100 761434	100761434	0.93	0.93	1.08	1.14	1.06	1.08	94.18	94.25	109.30	118.21	109.71	112.16
olfactory receptor 2Z1	LOC100 761437	100761437	0.03	0.00	0.00	0.00	0.00	0.00	0.76	0.00	0.00	0.00	0.00	0.00
40S ribosomal protein SA-like	LOC100 761442	100761442	0.00	0.00	0.06	0.06	0.05	0.09	0.76	0.00	1.24	1.16	1.03	3.74
E3 ubiquitin-protein ligase RNF168 pseudogene	LOC100 761443	100761443	0.01	0.01	0.00	0.00	0.00	0.05	0.00	0.00	0.00	1.16	0.00	2.80
pleckstrin homology-like domain family A member 1	LOC100 761451	100761451	61.97	53.20	36.74	33.89	46.90	43.27	836.25	725.62	481.93	433.44	593.05	560.80
acyl-coenzyme A thioesterase 8	LOC100 761464	100761464	32.65	29.56	28.03	20.59	15.71	17.13	1026.90	953.46	894.30	673.33	544.40	580.43
cytochrome c oxidase assembly factor 3 homolog, mitochondrial	LOC100 761465	100761465	128.49	122.60	84.86	75.67	65.34	70.15	2309.00	2208.89	1526.52	1353.62	1181.95	1254.33
olfactory receptor 4P4-like	LOC100 761468	100761468	0.00	0.00	0.00	0.00	0.00	0.00	0.00	0.00	0.00	0.00	0.00	0.00
speckle-type POZ protein pseudogene	LOC100 761478	100761478	0.00	0.00	0.00	0.00	0.00	0.00	0.00	0.00	0.00	0.00	0.00	0.00
phosphoglycerate mutase 1 pseudogene	LOC100 761479	100761479	0.00	0.00	0.00	0.00	0.00	0.00	5.32	4.58	2.48	0.00	0.00	0.93
methylcytosine dioxygenase TET2-like	LOC100 761483	100761483	0.01	0.00	0.00	0.00	0.01	0.00	0.00	0.00	0.00	0.00	0.00	0.00

programmed cell death protein 5 pseudogene	LOC100761487	100761487	9.50	11.52	7.55	6.16	4.08	4.45	47.09	56.73	37.26	24.34	19.66	23.37
DNA-binding protein inhibitor ID-2 pseudogene	LOC100761488	100761488	0.00	0.00	0.00	0.00	0.00	0.00	0.00	0.00	0.00	0.00	0.00	0.00
60S ribosomal protein L21 pseudogene	LOC100761493	100761493	12.52	10.92	10.17	7.74	8.27	8.15	100.26	78.69	75.77	55.63	60.03	66.36
keratin-associated protein 16-1	LOC100761497	100761497	0.00	0.00	0.00	0.00	0.00	0.00	0.00	0.00	0.00	0.00	0.00	0.00
gamma-tubulin complex component 4 pseudogene	LOC100761499	100761499	0.29	0.47	0.27	0.34	0.36	0.42	30.38	37.52	26.08	23.18	22.77	31.78
olfactory receptor 52J3-like	LOC100761501	100761501	0.00	0.00	0.00	0.00	0.00	0.00	0.00	0.00	0.00	0.00	0.00	0.00
interferon-inducible GTPase 1-like	LOC100761502	100761502	0.00	0.00	0.00	0.00	0.00	0.00	0.00	0.00	0.00	0.00	0.00	0.00
olfactory receptor 142	LOC100761506	100761506	0.00	0.00	0.00	0.00	0.00	0.00	0.00	0.00	0.00	0.00	0.00	0.00
olfactory receptor 13G1	LOC100761507	100761507	0.00	0.00	0.00	0.00	0.00	0.00	0.00	0.00	0.00	0.00	0.00	0.00
deoxycytidine kinase pseudogene	LOC100761509	100761509	0.04	0.00	0.00	0.00	0.00	0.05	0.76	0.00	0.00	0.00	0.00	1.87
olfactory receptor 52H1-like	LOC100761513	100761513	0.03	0.00	0.00	0.00	0.00	0.00	0.76	0.00	0.00	0.00	0.00	0.00
melanoma-associated antigen B18	LOC100761514	100761514	0.03	0.00	0.00	0.00	0.00	0.04	0.76	0.00	0.00	1.16	0.00	1.87
olfactory receptor 5L2-like	LOC100761517	100761517	0.00	0.00	0.00	0.00	0.00	0.00	0.00	0.00	0.00	0.00	0.00	0.00
killer cell lectin-like receptor subfamily B member 1A	LOC100761519	100761519	0.04	0.00	0.00	0.00	0.00	0.00	0.76	0.92	0.00	0.00	0.00	0.00
peptidyl-prolyl cis-trans isomerase A-like	LOC100761526	100761526	0.08	0.28	0.00	0.00	0.02	0.10	1.52	1.83	0.00	0.00	0.00	0.93
60S ribosomal protein L37 pseudogene	LOC100761527	100761527	0.00	0.00	0.00	0.00	0.00	0.00	0.00	0.00	0.00	1.16	0.00	0.00
olfactory receptor 5-like	LOC100761528	100761528	0.00	0.00	0.00	0.00	0.00	0.00	0.00	0.00	0.00	0.00	0.00	0.00
glycosyltransferase 6 domain-containing protein 1-like	LOC100761532	100761532	0.00	0.00	0.00	0.00	0.00	0.00	0.00	0.00	0.00	0.00	0.00	0.00

putative olfactory receptor 51H1	LOC100761534	100761534	0.00	0.00	0.00	0.00	0.00	0.00	0.00	0.00	0.00	0.00	0.00	0.00
60S ribosomal protein L15-like	LOC100761537	100761537	1.63	2.43	2.19	1.09	1.88	1.35	47.85	74.12	68.31	32.45	56.92	40.19
actin-related protein 2 pseudogene	LOC100761543	100761543	0.03	0.00	0.10	0.00	0.06	0.08	0.76	0.00	2.48	0.00	1.03	1.87
GRB2-associated-binding protein 2-like	LOC100761547	100761547	0.00	0.00	0.00	0.00	0.00	0.00	0.00	0.00	0.00	0.00	0.00	0.00
GSK-3-binding protein FRAT2-like	LOC100761548	100761548	0.00	0.00	0.00	0.00	0.00	0.00	0.00	0.00	0.00	0.00	0.00	0.00
histone deacetylase 1-like	LOC100761556	100761556	0.78	1.00	0.82	0.70	0.84	0.61	28.86	37.52	29.81	22.02	31.05	18.69
histone H3.1	LOC100761562	100761562	0.09	0.12	0.00	0.00	0.02	0.00	0.00	0.92	0.00	0.00	0.00	0.00
zinc finger protein 73-like	LOC100761566	100761566	1.92	1.68	1.99	2.55	2.99	2.82	236.98	209.54	248.42	309.43	372.60	350.50
non-POU domain-containing octamer-binding protein pseudogene	LOC100761570	100761570	0.00	0.00	0.00	0.00	0.00	0.00	0.00	0.00	0.00	0.00	0.00	0.00
uncharacterized LOC100761572	LOC100761572	100761572	10.74	11.37	13.17	15.32	17.90	16.57	572.69	597.52	706.75	812.40	911.82	852.42
60S ribosomal protein L6 pseudogene	LOC100761573	100761573	0.00	0.00	0.00	0.00	0.00	0.00	0.00	0.00	0.00	0.00	0.00	0.00
transcriptional adapter 3 pseudogene	LOC100761575	100761575	0.17	0.05	0.10	0.08	0.08	0.09	5.32	1.83	3.73	2.32	3.10	1.87
retinol dehydrogenase 7-like	LOC100761577	100761577	0.00	0.00	0.00	0.00	0.07	0.00	0.00	0.00	0.00	0.00	0.00	0.00
ras-related protein Rab-1A pseudogene	LOC100761579	100761579	0.88	1.02	1.31	0.60	0.38	0.65	10.63	12.81	16.15	9.27	9.31	7.48
malate dehydrogenase, mitochondrial pseudogene	LOC100761581	100761581	0.00	0.00	0.00	0.00	0.00	0.00	0.00	0.00	0.00	0.00	0.00	0.00
uncharacterized protein KIAA1671	LOC100761583	100761583	0.00	0.00	0.00	0.00	0.00	0.00	0.00	0.00	0.00	0.00	0.00	0.00
C-C chemokine receptor type 1	LOC100761586	100761586	0.00	0.00	0.00	0.00	0.00	0.00	0.00	0.00	0.00	0.00	0.00	0.00
small conductance calcium-activated potassium channel protein 2	LOC100761590	100761590	0.00	0.00	0.00	0.00	0.00	0.00	0.00	0.00	0.00	0.00	0.00	0.00

chromosome unknown open reading frame, human C11orf24	LOC100761593	100761593	51.98	50.44	91.99	90.55	74.83	77.96	2658.39	2614.25	4790.73	4682.04	3916.39	4098.53
transmembrane protein 128 pseudogene	LOC100761597	100761597	0.00	0.00	0.00	0.00	0.00	0.00	0.00	0.00	0.00	0.00	0.00	0.00
ATP synthase-coupling factor 6, mitochondrial-like	LOC100761601	100761601	0.07	0.33	0.00	0.22	0.19	0.26	11.39	10.98	12.42	10.43	19.66	20.56
protein slowmo homolog 2 pseudogene	LOC100761606	100761606	0.00	0.00	0.00	0.00	0.00	0.00	0.00	0.00	0.00	0.00	0.00	0.00
proline-rich protein 23-like	LOC100761608	100761608	0.00	0.00	0.00	0.00	0.00	0.00	0.00	0.00	0.00	0.00	0.00	0.00
keratin-associated protein 13-1-like	LOC100761609	100761609	0.00	0.00	0.00	0.00	0.00	0.00	0.00	0.00	0.00	0.00	0.00	0.00
mitochondrial import receptor subunit TOM20 homolog pseudogene	LOC100761610	100761610	1.20	0.62	0.71	0.75	0.84	0.22	17.47	8.24	18.63	6.95	10.35	3.74
E3 ubiquitin-protein ligase TRIM39-like	LOC100761611	100761611	3.22	3.10	4.19	4.43	4.19	4.06	94.18	86.01	121.72	119.37	121.09	114.96
Ig heavy chain Mem5-like	LOC100761614	100761614	0.00	0.00	0.00	0.00	0.00	0.00	0.00	0.00	0.00	0.00	0.00	0.00
ubiquitin carboxyl-terminal hydrolase 29-like	LOC100761617	100761617	0.00	0.00	0.00	0.00	0.00	0.00	0.00	0.00	0.00	0.00	0.00	0.00
uncharacterized LOC100761618	LOC100761618	100761618	0.89	1.17	0.96	1.18	1.12	1.60	56.97	62.22	70.80	74.17	75.55	86.92
glyceraldehyde-3-phosphate dehydrogenase pseudogene	LOC100761621	100761621	6.41	6.70	5.91	3.41	3.88	3.15	50.89	50.33	52.17	26.66	32.08	23.37
inhibitor of carbonic anhydrase	LOC100761626	100761626	0.03	0.00	0.02	0.02	0.00	0.00	2.28	0.00	1.24	1.16	0.00	0.00
zinc finger protein 120-like	LOC100761630	100761630	0.01	0.00	0.00	0.06	0.03	0.02	0.00	0.00	0.00	2.32	0.00	0.93
protein crumbs homolog 3 pseudogene	LOC100761635	100761635	0.00	0.00	0.00	0.00	0.00	0.00	0.00	0.00	0.00	0.00	0.00	0.00
elongation factor 1-alpha 1 pseudogene	LOC100761640	100761640	86.81	107.07	94.05	71.35	98.82	67.40	2488.25	3052.55	2636.95	2037.38	2753.06	1895.51
N-alpha-acetyltransferase 10-like	LOC100761642	100761642	0.07	0.26	0.12	0.06	0.05	0.13	3.80	6.41	2.48	5.79	2.07	3.74
ADP/ATP translocase 2-like	LOC100761654	100761654	98.84	94.43	83.35	48.39	24.87	29.78	2422.93	2350.72	2061.86	1176.30	594.08	695.39

olfactory receptor 13	LOC100761658	100761658	0.00	0.00	0.00	0.00	0.00	0.00	0.00	0.00	0.00	0.00	0.00	0.00
chromosome unknown open reading frame, human C5orf34	LOC100761661	100761661	2.79	3.09	3.61	4.44	4.90	4.99	205.84	237.91	264.56	337.25	373.63	362.65
olfactory receptor 4C15-like	LOC100761665	100761665	0.00	0.00	0.00	0.00	0.00	0.00	0.00	0.00	0.00	0.00	0.00	0.00
lipase member N	LOC100761667	100761667	0.05	0.03	0.00	0.00	0.00	0.00	2.28	1.83	0.00	0.00	1.03	2.80
olfactory receptor 5M8	LOC100761668	100761668	0.00	0.00	0.00	0.00	0.00	0.00	0.00	0.00	0.00	0.00	0.00	0.00
ankyrin repeat domain-containing protein 26-like	LOC100761669	100761669	0.00	0.00	0.00	0.00	0.01	0.02	0.76	0.00	2.48	1.16	3.10	8.41
ferritin heavy chain pseudogene	LOC100761670	100761670	0.93	1.25	0.97	0.57	0.97	0.37	18.99	25.62	19.87	11.59	22.77	7.48
U2 small nuclear ribonucleoprotein B'' pseudogene	LOC100761674	100761674	0.10	0.26	0.36	0.15	0.23	0.12	1.52	3.66	4.97	2.32	3.10	1.87
signal peptidase complex subunit 2-like	LOC100761677	100761677	0.00	0.00	0.00	0.00	0.00	0.00	0.00	0.00	0.00	0.00	0.00	0.00
chromosome unknown open reading frame, human C11orf80	LOC100761684	100761684	0.07	0.10	0.08	0.03	0.16	0.18	5.32	7.32	9.94	3.48	12.42	10.28
glyceraldehyde-3-phosphate dehydrogenase pseudogene	LOC100761686	100761686	0.09	0.11	0.04	0.04	0.13	0.08	3.04	3.66	1.24	1.16	4.14	2.80
liver carboxylesterase-like	LOC100761688	100761688	0.00	0.00	0.00	0.00	0.00	0.00	0.00	0.00	0.00	0.00	0.00	0.00
olfactory receptor 4F3/4F16/4F29	LOC100761689	100761689	0.00	0.00	0.00	0.00	0.00	0.00	0.00	0.00	0.00	0.00	0.00	0.00
olfactory receptor 5AN1-like	LOC100761690	100761690	0.00	0.00	0.00	0.00	0.00	0.00	0.00	0.00	0.00	0.00	0.00	0.00
transcription elongation factor A protein-like 8	LOC100761691	100761691	0.00	0.00	0.00	0.00	0.00	0.00	0.00	0.00	0.00	0.00	0.00	0.00
C-type lectin domain family 2 member D11-like	LOC100761692	100761692	1.75	1.76	2.08	2.07	1.83	1.95	191.40	197.65	222.33	250.33	222.52	222.45
heterogeneous nuclear ribonucleoprotein A3 pseudogene	LOC100761695	100761695	46.31	53.32	45.28	43.09	46.70	34.70	818.02	931.50	827.23	750.98	830.06	607.54
olfactory receptor 147	LOC100761696	100761696	0.00	0.00	0.00	0.00	0.00	0.00	0.00	0.00	0.00	0.00	0.00	0.00

olfactory receptor 14A16-like	LOC100 761705	100761705	0.00	0.00	0.00	0.00	0.00	0.00	0.00	0.00	0.00	0.00	0.00	0.00
retinal dehydrogenase 1-like	LOC100 761706	100761706	0.00	0.00	0.00	0.00	0.00	0.00	0.00	0.00	0.00	0.00	0.00	0.00
zinc transporter 9-like	LOC100 761715	100761715	2.28	2.41	2.17	1.89	2.96	2.45	90.39	92.42	86.95	74.17	114.88	93.47
tubulin alpha-1A chain	LOC100 761720	100761720	143.35	138.99	133.92	109.94	87.53	95.06	3285.01	3086.41	3005.85	2417.51	1729.46	2006.74
zinc finger protein 420-like	LOC100 761721	100761721	0.02	0.00	0.00	0.00	0.02	0.00	0.76	0.00	0.00	0.00	1.03	0.93
chromatin target of PRMT1 protein pseudogene	LOC100 761722	100761722	0.29	0.10	0.24	0.07	0.12	0.16	2.28	0.92	0.00	0.00	0.00	1.87
leukotriene-B(4) omega-hydroxylase 2	LOC100 761725	100761725	0.00	0.00	0.00	0.01	0.00	0.00	0.00	0.00	0.00	0.00	0.00	0.00
tubulin alpha-1C chain-like	LOC100 761726	100761726	0.47	0.36	0.28	0.05	0.48	0.26	69.88	53.99	42.23	32.45	52.78	73.84
E3 ubiquitin-protein ligase RNF168 pseudogene	LOC100 761731	100761731	0.01	0.01	0.00	0.00	0.00	0.00	0.00	0.00	0.00	0.00	0.00	0.00
39S ribosomal protein L24, mitochondrial pseudogene	LOC100 761739	100761739	0.77	0.78	0.63	0.96	0.71	0.93	12.91	14.64	9.94	17.38	12.42	13.09
high mobility group protein B1 pseudogene	LOC100 761755	100761755	9.05	11.07	7.89	6.32	5.96	5.06	62.28	85.10	54.65	40.56	35.19	39.26
olfactory receptor 4P4-like	LOC100 761756	100761756	0.02	0.00	0.04	0.03	0.03	0.03	1.52	0.00	1.24	1.16	1.03	0.93
histone H2A type 1-F	LOC100 761758	100761758	0.00	0.00	0.00	0.00	0.00	0.00	0.00	0.00	0.00	0.00	0.00	0.00
peroxiredoxin-1 pseudogene	LOC100 761768	100761768	0.00	0.00	0.00	0.00	0.00	0.00	1.52	2.75	0.00	0.00	1.03	0.00
PRAME family member 12-like	LOC100 761772	100761772	0.00	0.00	0.00	0.00	0.00	0.00	0.00	0.00	0.00	0.00	0.00	0.00
interferon alpha-12-like	LOC100 761773	100761773	0.00	0.00	0.00	0.00	0.00	0.00	0.00	0.00	0.00	0.00	0.00	0.00
NADH dehydrogenase [ubiquinone] iron-sulfur protein 8, mitochondrial pseudogene	LOC100 761775	100761775	0.00	0.00	0.00	0.00	0.00	0.00	0.00	0.00	0.00	0.00	0.00	0.00
cytochrome P450 2U1	LOC100 761779	100761779	16.08	17.02	23.99	25.12	23.93	22.37	448.89	468.50	650.85	689.56	647.90	613.14

keratin, type I cuticular Ha1-like	LOC100 761784	100761784	0.00	0.00	0.00	0.00	0.00	0.00	0.00	0.00	0.00	0.00	0.00	0.00
olfactory receptor 1496	LOC100 761786	100761786	0.00	0.00	0.00	0.00	0.00	0.00	0.00	0.00	0.00	0.00	0.00	0.00
chromosome unknown open reading frame, human CXorf30	LOC100 761787	100761787	0.00	0.02	0.00	0.00	0.00	0.00	0.00	0.92	0.00	0.00	0.00	0.00
olfactory receptor 52D1-like	LOC100 761788	100761788	0.00	0.00	0.00	0.00	0.00	0.00	0.00	0.00	0.00	0.00	0.00	0.00
interferon-inducible GTPase 1-like	LOC100 761789	100761789	0.00	0.00	0.00	0.00	0.00	0.00	0.00	0.00	0.00	0.00	0.00	0.00
probable E3 ubiquitin-protein ligase HERC3	LOC100 761790	100761790	1.69	1.71	1.58	2.04	2.43	2.16	120.01	127.19	120.48	148.34	165.60	153.29
olfactory receptor 4B1	LOC100 761793	100761793	0.00	0.00	0.00	0.00	0.00	0.00	0.00	0.00	0.00	0.00	0.00	0.00
olfactory receptor 226-like	LOC100 761794	100761794	0.00	0.00	0.00	0.00	0.00	0.00	0.00	0.00	0.00	0.00	0.00	0.00
dnaJ homolog subfamily C member 10 pseudogene	LOC100 761795	100761795	0.00	0.00	0.00	0.00	0.00	0.00	0.00	0.00	0.00	0.00	0.00	0.00
opioid growth factor receptor pseudogene	LOC100 761796	100761796	1.60	1.34	1.38	0.95	0.53	0.79	78.23	73.20	57.14	46.36	16.56	30.84
olfactory receptor 52E1-like	LOC100 761799	100761799	0.00	0.00	0.00	0.00	0.00	0.00	0.00	0.00	0.00	0.00	0.00	0.00
probable G-protein coupled receptor 160	LOC100 761800	100761800	0.02	0.00	0.03	0.02	0.11	0.06	0.00	0.00	0.00	0.00	2.07	0.00
melanoma-associated antigen B4	LOC100 761801	100761801	0.00	0.00	0.00	0.00	0.04	0.00	0.00	0.00	0.00	0.00	1.03	0.00
olfactory receptor 5D18	LOC100 761804	100761804	0.00	0.00	0.00	0.00	0.00	0.00	0.00	0.00	0.00	0.00	0.00	0.00
alpha-2-macroglobulin-like	LOC100 761806	100761806	0.01	0.00	0.00	0.01	0.01	0.00	8.35	5.49	3.73	2.32	11.38	6.54
tumor suppressor candidate 3	LOC100 761811	100761811	11.98	10.90	11.85	14.34	16.31	13.36	420.03	374.25	408.65	484.43	556.82	446.77
histone H3.3	LOC100 761813	100761813	4.53	3.88	2.48	3.65	3.01	3.92	248.37	266.27	243.45	244.53	203.89	228.06
olfactory receptor 5	LOC100 761814	100761814	0.00	0.00	0.00	0.00	0.00	0.00	0.00	0.00	0.00	0.00	0.00	0.00

sperm motility kinase-like	LOC100 761816	100761816	0.03	0.03	0.00	0.00	0.04	0.00	1.52	0.92	0.00	0.00	0.00	0.00
uncharacterized LOC100761818	LOC100 761818	100761818	0.00	0.00	0.00	0.00	0.00	0.00	0.00	0.00	0.00	0.00	0.00	0.00
olfactory receptor 12-like	LOC100 761821	100761821	0.05	0.00	0.00	0.00	0.00	0.00	0.76	0.00	0.00	0.00	0.00	0.00
eukaryotic translation initiation factor 3 subunit J-like	LOC100 761823	100761823	0.04	0.00	0.00	0.00	0.00	0.00	1.52	1.83	2.48	0.00	0.00	1.87
60S ribosomal protein L31 pseudogene	LOC100 761824	100761824	0.00	0.00	0.00	0.00	0.00	0.00	0.00	0.00	0.00	0.00	0.00	0.00
nebulin-related-anchoring protein	LOC100 761828	100761828	15.46	16.53	14.16	16.88	15.50	15.89	712.45	753.07	660.79	801.97	725.53	753.34
pre-mRNA-splicing factor SPF27 pseudogene	LOC100 761831	100761831	0.29	0.23	0.21	0.05	0.07	0.16	9.87	6.41	4.97	1.16	1.03	3.74
60S acidic ribosomal protein P1 pseudogene	LOC100 761837	100761837	29.17	26.51	25.49	15.44	7.11	7.36	224.82	256.21	244.69	105.46	50.71	52.34
chromosome unknown open reading frame, human C21orf2	LOC100 761839	100761839	6.88	7.53	8.60	7.14	5.88	6.36	302.30	331.24	377.59	305.95	257.71	277.60
histone H1.4	LOC100 761846	100761846	0.18	0.22	0.16	0.03	0.01	0.02	0.00	0.00	0.00	0.00	0.00	0.00
60S acidic ribosomal protein P1-like	LOC100 761847	100761847	3.33	4.38	6.07	2.98	0.98	0.70	15.95	17.39	27.33	8.11	7.24	8.41
pre-mRNA-splicing factor SPF27 pseudogene	LOC100 761848	100761848	0.29	0.38	0.12	0.00	0.27	0.21	8.35	6.41	1.24	2.32	7.24	5.61
protein FAM222B-like	LOC100 761849	100761849	0.02	0.03	0.02	0.02	0.02	0.02	0.76	0.92	1.24	1.16	0.00	0.93
zinc finger protein 39-like	LOC100 761850	100761850	1.32	1.10	1.47	1.64	2.17	1.38	95.70	79.61	95.64	107.78	146.97	97.21
prolactin-2B1-like	LOC100 761856	100761856	0.00	0.00	0.00	0.00	0.00	0.00	0.00	0.00	0.00	0.00	0.00	0.00
voltage-dependent anion-selective channel protein 3 pseudogene	LOC100 761857	100761857	0.71	0.67	0.57	0.51	0.48	0.28	17.47	14.64	12.42	12.75	11.38	5.61
mitogen-activated protein kinase 6 pseudogene	LOC100 761862	100761862	6.31	7.00	7.56	7.50	8.72	8.76	26.58	41.18	34.78	17.38	32.08	25.24
40S ribosomal protein SA pseudogene	LOC100 761864	100761864	0.00	0.00	0.00	0.00	0.00	0.00	0.00	0.00	0.00	0.00	0.00	0.00

C-C chemokine receptor 1-like protein 1	LOC100761868	100761868	0.00	0.00	0.00	0.00	0.00	0.00	0.00	0.00	0.00	0.00	0.00	0.00
coronin-1B pseudogene	LOC100761872	100761872	0.00	0.00	0.00	0.00	0.00	0.00	0.00	0.00	0.00	0.00	0.00	0.00
olfactory receptor 7G3-like	LOC100761874	100761874	0.00	0.00	0.00	0.00	0.00	0.00	0.00	0.00	0.00	0.00	0.00	0.00
olfactory receptor 52E8	LOC100761875	100761875	0.00	0.00	0.00	0.00	0.00	0.00	0.00	0.00	0.00	0.00	0.00	0.00
myosin light polypeptide 6-like	LOC100761877	100761877	0.00	0.00	0.00	0.00	0.00	0.00	0.00	0.00	0.00	0.00	0.00	0.00
serine/threonine-protein phosphatase 5	LOC100761879	100761879	19.58	19.98	21.40	17.01	14.70	15.34	726.12	745.75	747.74	577.14	506.11	515.94
ferritin light chain 1 pseudogene	LOC100761883	100761883	0.00	0.05	0.00	0.00	0.00	0.06	0.00	0.00	0.00	0.00	0.00	0.00
zinc finger protein 883-like	LOC100761886	100761886	2.56	2.62	3.09	2.25	2.99	2.17	182.29	174.77	196.25	173.84	207.00	153.29
tubulin beta-2A chain	LOC100761889	100761889	0.00	0.01	0.00	0.00	0.01	0.00	0.00	0.00	0.00	0.00	0.00	0.00
histone-lysine N-methyltransferase 2D-like	LOC100761892	100761892	0.00	0.00	0.00	0.00	0.00	0.00	0.00	0.00	0.00	0.00	0.00	0.00
keratin-associated protein 13-1	LOC100761893	100761893	0.00	0.00	0.00	0.00	0.00	0.00	0.00	0.00	0.00	0.00	0.00	0.00
tudor and KH domain-containing protein	LOC100761896	100761896	0.87	0.55	1.91	2.21	2.57	1.72	12.15	9.15	33.54	34.77	46.57	26.17
H-2 class I histocompatibility antigen, L-D alpha chain-like	LOC100761901	100761901	1.52	1.22	1.27	0.36	0.25	0.04	76.71	70.46	65.83	16.22	14.49	9.35
Uncharacterized protein C1orf31-like	LOC100761903	100761903	42.46	40.88	50.59	43.39	40.94	41.04	862.84	826.28	1021.00	858.76	796.94	808.49
60S ribosomal protein L9 pseudogene	LOC100761904	100761904	0.20	0.00	0.00	0.00	0.00	0.00	2.28	0.92	1.24	0.00	1.03	0.93
olfactory receptor 8S1-like	LOC100761906	100761906	0.00	0.00	0.00	0.00	0.00	0.00	0.00	0.00	0.00	0.00	0.00	0.00
TAF5-like RNA polymerase II p300/CBP-associated factor-associated factor 65 kDa subunit 5L pseudogene	LOC100761911	100761911	0.03	0.06	0.00	0.05	0.02	0.02	1.52	2.75	0.00	2.32	1.03	0.93
olfactory receptor 1468	LOC100761913	100761913	0.00	0.00	0.00	0.00	0.00	0.00	0.00	0.00	0.00	0.00	0.00	0.00

chromosome unknown open reading frame, human C1orf74	LOC100761917	100761917	0.02	0.00	0.00	0.00	0.00	0.02	1.52	0.00	0.00	0.00	0.00	0.93
olfactory receptor 958-like	LOC100761918	100761918	0.00	0.00	0.00	0.00	0.00	0.00	0.00	0.00	0.00	0.00	0.00	0.00
leukocyte immunoglobulin-like receptor subfamily A member 6	LOC100761919	100761919	0.00	0.00	0.00	0.00	0.00	0.00	0.00	0.00	0.00	0.00	0.00	0.00
olfactory receptor 5B3-like	LOC100761921	100761921	0.00	0.00	0.00	0.00	0.00	0.00	0.00	0.00	0.00	0.00	0.00	0.00
eukaryotic translation initiation factor 3 subunit K pseudogene	LOC100761924	100761924	0.00	0.00	0.00	0.00	0.00	0.00	0.00	0.00	0.00	0.00	0.00	0.00
40S ribosomal protein S8 pseudogene	LOC100761925	100761925	0.00	0.00	0.00	0.00	0.16	0.00	0.00	0.00	0.00	0.00	2.07	0.00
primary ciliary dyskinesia protein 1	LOC100761926	100761926	0.01	0.00	0.00	0.00	0.00	0.00	0.76	0.92	0.00	0.00	1.03	0.93
protein FAM118B pseudogene	LOC100761927	100761927	0.00	0.00	0.02	0.04	0.08	0.04	0.00	0.00	0.00	2.32	4.14	2.80
DNA-directed RNA polymerases I, II, and III subunit RPABC5	LOC100761930	100761930	0.57	0.36	0.55	0.42	0.56	0.30	3.80	0.00	2.48	0.00	2.07	0.93
GLIPR1-like protein 2	LOC100761938	100761938	0.00	0.00	0.05	0.00	0.00	0.00	0.00	0.00	3.73	0.00	1.03	0.00
40S ribosomal protein S2 pseudogene	LOC100761939	100761939	0.00	0.00	0.00	0.00	0.00	0.00	0.00	0.00	0.00	0.00	0.00	0.00
probable ribosome biogenesis protein RLP24 pseudogene	LOC100761940	100761940	0.33	0.40	0.28	0.40	0.35	0.21	3.04	3.66	2.48	3.48	3.10	1.87
protein transport protein Sec61 subunit gamma	LOC100761941	100761941	0.00	0.00	0.00	0.00	0.00	0.00	0.00	0.00	0.00	0.00	0.00	0.00
olfactory receptor 2A12-like	LOC100761942	100761942	0.00	0.00	0.00	0.00	0.00	0.00	0.00	0.00	0.00	0.00	0.00	0.00
putative vomeronasal receptor-like protein 4	LOC100761945	100761945	0.00	0.00	0.00	0.00	0.00	0.00	0.00	0.00	0.00	0.00	0.00	0.00
uncharacterized LOC100761946	LOC100761946	100761946	0.36	0.26	0.12	0.23	0.30	0.46	5.32	3.66	4.97	3.48	4.14	7.48
olfactory receptor 4C15	LOC100761950	100761950	0.00	0.00	0.00	0.00	0.00	0.00	0.00	0.00	0.00	0.00	0.00	0.00
HLA class II histocompatibility antigen, DRB1-10 beta chain-like	LOC100761951	100761951	0.00	0.00	0.00	0.00	0.00	0.00	0.00	0.00	0.00	0.00	0.00	0.00

olfactory receptor 5M3	LOC100 761953	100761953	0.00	0.00	0.00	0.00	0.00	0.00	0.00	0.00	0.00	0.00	0.00	0.00
myeloid cell surface antigen CD33	LOC100 761955	100761955	0.04	0.00	0.00	0.06	0.00	0.00	3.80	3.66	2.48	2.32	1.03	0.93
ferritin heavy chain	LOC100 761960	100761960	0.00	0.00	0.00	0.00	0.00	0.00	0.00	0.00	0.00	0.00	0.00	0.00
uncharacterized LOC100761961	LOC100 761961	100761961	0.00	0.00	0.00	0.00	0.00	0.00	0.76	1.83	0.00	0.00	0.00	0.93
60S ribosomal protein L36a-like	LOC100 761963	100761963	10.08	10.43	9.20	4.37	6.59	10.36	31.14	36.60	36.02	16.22	20.70	38.32
immunoglobulin omega chain	LOC100 761975	100761975	0.00	0.00	0.00	0.00	0.00	0.00	0.00	0.00	0.00	0.00	0.00	0.00
mothers against decapentaplegic homolog 1 pseudogene	LOC100 761976	100761976	0.11	0.16	0.07	0.14	0.03	0.06	0.00	0.00	0.00	0.00	0.00	0.00
olfactory receptor 4K3	LOC100 761978	100761978	0.00	0.00	0.00	0.00	0.00	0.00	0.00	0.00	0.00	0.00	0.00	0.00
olfactory receptor 5AN1-like	LOC100 761979	100761979	0.00	0.00	0.00	0.00	0.00	0.00	0.00	0.00	0.00	0.00	0.00	0.00
chromosome unknown open reading frame, human C22orf46	LOC100 761980	100761980	0.19	0.20	0.18	0.23	0.19	0.16	24.31	25.62	23.60	30.13	24.84	20.56
Ig lambda chain V-I region MEM-like	LOC100 761984	100761984	0.00	0.00	0.00	0.00	0.00	0.00						
olfactory receptor 147-like	LOC100 761987	100761987	0.00	0.00	0.00	0.00	0.00	0.00	0.00	0.00	0.00	0.00	0.00	0.00
forkhead box protein P4 pseudogene	LOC100 761998	100761998	0.04	0.05	0.02	0.00	0.04	0.04	1.52	0.00	0.00	0.00	0.00	0.00
S-formylglutathione hydrolase-like	LOC100 761999	100761999	0.05	0.30	0.06	0.00	0.10	0.13	12.15	15.56	6.21	2.32	11.38	7.48
olfactory receptor 52M1	LOC100 762002	100762002	0.00	0.00	0.00	0.00	0.00	0.00	0.00	0.00	0.00	0.00	0.00	0.00
tubulin beta chain	LOC100 762004	100762004	0.00	0.00	0.00	0.00	0.00	0.00	0.00	0.00	0.00	0.00	0.00	0.00
tudor and KH domain-containing protein pseudogene	LOC100 762006	100762006	0.08	0.12	0.05	0.20	0.27	0.31	1.52	0.92	2.48	3.48	5.17	6.54
chromatin target of PRMT1 protein pseudogene	LOC100 762017	100762017	0.00	0.09	0.00	0.00	0.11	0.00	1.52	0.92	0.00	0.00	1.03	0.00

zinc finger protein 709-like	LOC100762018	100762018	8.71	8.65	10.22	11.99	12.01	11.41	692.70	690.85	821.02	949.16	937.70	925.32
solute carrier family 22 member 4	LOC100762019	100762019	2.73	1.83	2.57	2.44	2.57	2.31	94.94	80.52	96.88	114.73	113.85	106.55
glyceraldehyde-3-phosphate dehydrogenase pseudogene	LOC100762023	100762023	0.20	0.18	0.16	0.07	0.13	0.13	7.60	2.75	6.21	1.16	2.07	4.67
nuclear envelope pore membrane protein POM 121	LOC100762026	100762026	17.32	17.21	17.47	14.95	11.08	12.45	2349.25	2376.34	2387.29	1974.80	1414.83	1597.35
GTP-binding nuclear protein Ran-like	LOC100762030	100762030	0.00	0.00	0.00	0.00	0.00	0.00	0.00	0.00	0.00	0.00	0.00	0.00
interferon-induced GTP-binding protein Mx2	LOC100762031	100762031	10.39	11.44	13.04	12.71	11.72	12.54	783.84	880.26	993.67	954.95	921.14	949.62
enhancer of rudimentary homolog pseudogene	LOC100762033	100762033	2.35	2.33	2.33	1.23	1.96	2.96	3.80	0.92	1.24	1.16	0.00	1.87
mortality factor 4-like protein 2	LOC100762034	100762034	1.71	1.12	1.03	1.41	1.34	1.44	29.62	20.13	19.87	26.66	24.84	24.30
pre-mRNA-splicing regulator WTAP	LOC100762035	100762035	24.94	25.26	26.37	26.85	27.57	25.41	1289.70	1296.60	1351.39	1425.47	1416.90	1315.08
protein SET-like	LOC100762039	100762039	0.02	0.00	0.07	0.00	0.00	0.03	0.76	0.00	2.48	0.00	0.00	0.93
elongation factor 1-alpha 1 pseudogene	LOC100762041	100762041	0.00	0.00	0.00	0.00	0.00	0.00	0.00	0.00	0.00	0.00	0.00	0.00
olfactory receptor 5D18-like	LOC100762047	100762047	0.00	0.00	0.00	0.00	0.00	0.00	0.00	0.00	0.00	0.00	0.00	0.00
histone H2B type 1	LOC100762049	100762049	1.23	1.07	0.83	0.42	0.24	0.33	0.76	0.00	0.00	0.00	0.00	0.00
chromosome unknown open reading frame, human C2orf80	LOC100762054	100762054	0.00	0.00	0.00	0.00	0.00	0.00	0.00	0.00	0.00	0.00	0.00	0.00
40S ribosomal protein S13	LOC100762057	100762057	1.79	1.99	2.59	1.70	2.35	1.88	20.51	31.11	29.81	17.38	31.05	17.76
retinol dehydrogenase 7-like	LOC100762059	100762059	0.00	0.00	0.00	0.00	0.00	0.00	0.00	0.00	0.00	0.00	0.00	0.00
endoplasmin-like	LOC100762060	100762060	0.04	0.01	0.02	0.04	0.00	0.00	2.28	0.92	1.24	2.32	0.00	0.00
tripartite motif-containing protein 6 pseudogene	LOC100762063	100762063	0.00	0.00	0.00	0.00	0.04	0.00	0.00	0.00	0.00	0.00	1.03	0.00

interferon alpha-12	LOC100 762064	100762064	0.00	0.00	0.00	0.00	0.00	0.00	0.00	0.00	0.00	0.00	0.00	0.00
60S ribosomal protein L24 pseudogene	LOC100 762066	100762066	0.00	0.11	0.00	0.00	0.00	0.00	0.00	0.92	0.00	0.00	0.00	0.00
Heterogeneous nuclear ribonucleoprotein D-like	LOC100 762069	100762069	0.00	0.00	0.00	0.00	0.00	0.00	0.00	0.00	0.00	0.00	0.00	0.00
ribose-phosphate pyrophosphokinase 1-like	LOC100 762070	100762070	0.21	0.17	0.22	0.17	0.23	0.25	3.80	3.66	4.97	2.32	1.03	5.61
GTPase ERas-like	LOC100 762076	100762076	0.00	0.00	0.00	0.00	0.00	0.00	0.00	0.00	0.00	0.00	0.00	0.00
interferon-inducible GTPase 1	LOC100 762078	100762078	0.01	0.00	0.00	0.00	0.00	0.01	0.00	0.00	0.00	0.00	0.00	0.00
ran-binding protein 17	LOC100 762081	100762081	2.35	2.71	2.62	2.82	3.41	3.76	233.18	268.10	257.11	279.30	336.37	356.11
high mobility group protein HMG-I/HMG-Y	LOC100 762083	100762083	0.24	0.15	0.00	0.00	0.12	0.11	9.11	12.81	16.15	8.11	10.35	4.67
olfactory receptor 226-like	LOC100 762084	100762084	0.00	0.00	0.00	0.00	0.00	0.00	0.00	0.00	0.00	0.00	0.00	0.00
olfactory receptor 51T1	LOC100 762090	100762090	0.00	0.00	0.00	0.00	0.00	0.00	0.00	0.00	0.00	0.00	0.00	0.00
melanoma-associated antigen B3-like	LOC100 762092	100762092	0.00	0.00	0.00	0.00	0.00	0.00	0.00	0.00	0.00	0.00	0.00	0.00
olfactory receptor 10AG1-like	LOC100 762095	100762095	0.00	0.00	0.00	0.00	0.00	0.00	0.00	0.00	0.00	0.00	0.00	0.00
partner of Y14 and mago-like	LOC100 762100	100762100	0.00	0.00	0.00	0.00	0.00	0.00	0.00	0.00	0.00	0.00	0.00	0.00
actin-related protein 2 pseudogene	LOC100 762101	100762101	0.07	0.09	0.08	0.12	0.14	0.11	0.76	0.92	0.00	3.48	3.10	0.93
olfactory receptor 5-like	LOC100 762104	100762104	0.00	0.00	0.00	0.00	0.00	0.00	0.00	0.00	0.00	0.00	0.00	0.00
vomeronasal secretory protein 1-like	LOC100 762106	100762106	0.00	0.00	0.00	0.00	0.00	0.00	0.00	0.00	0.00	0.00	0.00	0.00
kinesin-associated protein 3-like	LOC100 762107	100762107	0.07	0.11	0.08	0.11	0.07	0.04	4.56	7.32	4.97	6.95	4.14	2.80
bifunctional heparan sulfate N-deacetylase/N-sulfotransferase 4	LOC100 762108	100762108	0.00	0.00	0.00	0.00	0.00	0.00	0.00	0.00	0.00	0.00	0.00	0.00

PEST proteolytic signal-containing nuclear protein	LOC100 762114	100762114	10.02	10.02	12.39	13.82	14.53	13.27	205.84	189.41	253.39	285.09	288.76	273.86
pancreatic triacylglycerol lipase	LOC100 762115	100762115	0.00	0.00	0.00	0.00	0.00	0.00	0.00	1.83	1.24	1.16	1.03	0.93
cytochrome c oxidase subunit 7C, mitochondrial pseudogene	LOC100 762120	100762120	0.00	0.00	0.00	0.00	0.00	0.00	0.00	0.00	0.00	0.00	0.00	0.00
60S ribosomal protein L31 pseudogene	LOC100 762135	100762135	0.00	0.00	0.00	0.00	0.00	0.00	0.00	0.00	0.00	0.00	0.00	0.00
zinc finger protein 39-like	LOC100 762136	100762136	2.19	1.80	1.95	2.15	2.75	2.39	242.29	201.31	217.37	239.90	306.36	266.38
chloride intracellular channel protein 1 pseudogene	LOC100 762137	100762137	2.76	2.67	2.27	1.97	2.42	1.89	48.61	43.01	36.02	34.77	41.40	33.65
DAZ-associated protein 2-like	LOC100 762145	100762145	14.88	12.93	13.89	15.45	15.09	15.50	170.14	146.41	155.26	170.36	155.25	172.91
serine/threonine-protein kinase MST4	LOC100 762146	100762146	0.00	0.00	0.00	0.00	0.00	0.00	0.00	0.00	0.00	0.00	0.00	0.00
60S ribosomal protein L27 pseudogene	LOC100 762148	100762148	10.97	11.79	8.59	4.62	6.97	5.22	102.54	107.97	78.25	39.40	61.06	46.73
pleiotropic regulator 1 pseudogene	LOC100 762150	100762150	0.22	0.22	0.18	0.22	0.24	0.22	3.80	4.58	1.24	2.32	5.17	2.80
polyadenylate-binding protein 4 pseudogene	LOC100 762156	100762156	0.00	0.12	0.32	0.07	0.00	0.00	0.00	1.83	4.97	1.16	0.00	0.00
vacuolar protein sorting-associated protein 72 homolog pseudogene	LOC100 762158	100762158	0.06	0.03	0.18	0.04	0.00	0.00	1.52	0.92	4.97	1.16	0.00	0.00
chromosome unknown open reading frame, human C6orf25	LOC100 762159	100762159	0.00	0.00	0.00	0.00	0.00	0.00	0.00	0.92	0.00	0.00	0.00	0.00
olfactory receptor 7G2	LOC100 762161	100762161	0.00	0.00	0.00	0.00	0.00	0.00	0.00	0.00	0.00	0.00	0.00	0.00
olfactory receptor 52K1	LOC100 762163	100762163	0.00	0.00	0.00	0.00	0.00	0.00	0.00	0.00	0.00	0.00	0.00	0.00
40S ribosomal protein S25 pseudogene	LOC100 762164	100762164	3.49	1.68	1.33	0.37	0.65	1.38	34.94	18.30	12.42	3.48	8.28	13.09
rho-related GTP-binding protein RhoV pseudogene	LOC100 762165	100762165	0.00	0.00	0.00	0.00	0.00	0.00	0.00	0.00	0.00	0.00	0.00	0.00
PNMA-like protein 1	LOC100 762167	100762167	0.00	0.00	0.00	0.00	0.00	0.00	0.00	0.00	0.00	0.00	0.00	0.00

heat shock protein HSP 90-beta-like	LOC100 762168	100762168	0.03	0.03	0.03	0.01	0.01	0.01	1.52	1.83	1.24	1.16	1.03	0.00
zinc finger protein 2 homolog	LOC100 762173	100762173	1.06	0.98	1.46	1.74	1.76	1.52	205.08	193.07	294.37	355.79	343.62	299.09
serpin B6-like	LOC100 762176	100762176	8.43	6.91	4.85	3.92	6.80	7.16	0.00	0.92	0.00	0.00	0.00	0.00
60S ribosomal protein L6 pseudogene	LOC100 762179	100762179	1.29	0.84	0.94	0.41	0.89	0.51	21.27	13.73	17.39	8.11	15.52	9.35
keratin-associated protein 13-1-like	LOC100 762180	100762180	0.00	0.00	0.00	0.00	0.00	0.00	0.00	0.00	0.00	0.00	0.00	0.00
interferon-induced very large GTPase 1-like	LOC100 762181	100762181	0.00	0.00	0.00	0.00	0.00	0.00	0.00	0.00	0.00	0.00	0.00	0.00
H-2 class I histocompatibility antigen, Q10 alpha chain-like	LOC100 762182	100762182	3.44	3.68	3.14	0.94	0.60	0.87	73.68	77.78	57.14	22.02	9.31	12.15
zinc finger MYND domain-containing protein 19 pseudogene	LOC100 762183	100762183	0.00	0.00	0.00	0.00	0.00	0.00	0.00	0.00	0.00	0.00	0.00	0.00
citrate synthase, mitochondrial pseudogene	LOC100 762185	100762185	0.19	0.20	0.14	0.17	0.23	0.05	22.79	17.39	8.69	15.07	17.59	17.76
60 kDa heat shock protein, mitochondrial-like	LOC100 762188	100762188	0.02	0.04	0.00	0.00	0.05	0.00	0.76	1.83	0.00	0.00	2.07	0.00
lanosterol 14-alpha demethylase	LOC100 762189	100762189	32.16	35.64	51.08	54.30	49.57	47.01	2583.19	2874.12	4097.64	4328.57	3944.33	3751.76
protein AMN1 homolog pseudogene	LOC100 762190	100762190	0.00	0.00	0.00	0.00	0.00	0.00	0.00	0.00	0.00	0.00	0.00	0.00
heterogeneous nuclear ribonucleoprotein A3 pseudogene	LOC100 762191	100762191	6.37	5.87	4.22	4.51	6.09	3.26	60.00	51.24	36.02	40.56	48.64	28.97
forkhead box protein N1	LOC100 762193	100762193	0.00	0.00	0.00	0.00	0.00	0.00	0.00	0.92	0.00	0.00	0.00	0.93
la-related protein 4-like	LOC100 762197	100762197	0.00	0.00	0.00	0.01	0.00	0.00	0.00	0.00	0.00	0.00	0.00	0.00
uncharacterized protein C7orf73-like	LOC100 762200	100762200	2.82	0.00	2.59	13.05	0.00	0.00	107.85	113.46	125.45	89.24	50.71	64.49
olfactory receptor 1496	LOC100 762201	100762201	0.00	0.00	0.00	0.00	0.00	0.00	0.00	0.00	0.00	0.00	0.00	0.00
60S ribosomal protein L7-like	LOC100 762215	100762215	0.00	0.00	0.00	0.00	0.00	0.00	0.00	0.00	0.00	0.00	0.00	0.00

nucleosome assembly protein 1-like 1 pseudogene	LOC100762223	100762223	32.97	34.02	36.30	34.45	35.27	30.86	2318.11	2307.71	2323.94	2232.08	2255.24	2108.61
28S ribosomal protein S21, mitochondrial-like	LOC100762230	100762230	0.29	0.00	0.00	0.00	0.00	0.00	0.76	0.00	0.00	0.00	0.00	0.00
olfactory receptor 13	LOC100762231	100762231	0.00	0.00	0.00	0.00	0.00	0.00	0.00	0.00	0.00	0.00	0.00	0.00
chromosome unknown open reading frame, human C17orf53	LOC100762232	100762232	7.90	7.38	5.77	5.94	5.42	4.89	625.86	616.73	459.57	472.84	436.76	399.10
putative vomeronasal receptor-like protein 4	LOC100762234	100762234	0.00	0.00	0.00	0.00	0.00	0.00	0.00	0.00	0.00	0.00	0.00	0.00
olfactory receptor 4C15-like	LOC100762238	100762238	0.00	0.00	0.00	0.00	0.00	0.00	0.00	0.00	0.00	0.00	0.00	0.00
H-2 class II histocompatibility antigen, E-D beta chain-like	LOC100762240	100762240	0.00	0.00	0.00	0.00	0.00	0.00	0.00	0.00	0.00	0.00	0.00	0.00
olfactory receptor 5M3-like	LOC100762242	100762242	0.00	0.00	0.00	0.00	0.00	0.00	0.00	0.00	0.00	0.00	0.00	0.00
chromosome unknown open reading frame, human C11orf73	LOC100762248	100762248	82.75	80.69	78.08	73.64	78.74	71.84	2193.55	2117.39	2066.83	1933.08	2043.06	1884.29
zinc finger protein 431-like	LOC100762252	100762252	4.54	4.47	5.83	5.97	6.52	6.17	439.77	438.30	572.60	589.89	633.41	608.47
rab5 GDP/GTP exchange factor-like	LOC100762254	100762254	3.11	3.89	4.02	4.62	3.88	3.75	180.77	228.76	232.27	250.33	220.45	196.28
tubulin polyglutamylase complex subunit 2 pseudogene	LOC100762257	100762257	0.00	0.09	0.06	0.06	0.11	0.29	0.00	1.83	1.24	1.16	2.07	6.54
immunoglobulin iota chain-like	LOC100762263	100762263	0.00	0.00	0.00	0.00	0.00	0.00	0.00	0.00	0.00	0.00	0.00	0.00
uncharacterized LOC100762264	LOC100762264	100762264	0.03	0.05	0.08	0.08	0.09	0.04	0.76	1.83	37.26	31.29	35.19	34.58
olfactory receptor 4K13-like	LOC100762265	100762265	0.00	0.00	0.00	0.00	0.00	0.00	0.00	0.00	0.00	0.00	0.00	0.00
olfactory receptor 5AN1-like	LOC100762266	100762266	0.00	0.00	0.00	0.00	0.00	0.00	0.00	0.00	0.00	0.00	0.00	0.00
olfactory receptor 52E2-like	LOC100762267	100762267	0.00	0.00	0.00	0.00	0.00	0.00	0.00	0.00	0.00	0.00	0.00	0.00
killer cell lectin-like receptor subfamily B member 1F	LOC100762270	100762270	0.82	0.49	0.60	0.50	1.45	1.64	34.94	21.96	27.33	28.97	47.61	71.97

ras GTPase-activating protein-binding protein 1 pseudogene	LOC100 762271	100762271	5.02	4.58	4.12	3.14	3.46	3.43	178.49	162.88	157.75	113.57	117.99	130.85
olfactory receptor 7E24-like	LOC100 762272	100762272	0.00	0.00	0.00	0.00	0.00	0.00	0.00	0.00	0.00	0.00	0.00	0.00
olfactory receptor 8B3-like	LOC100 762275	100762275	0.00	0.00	0.00	0.00	0.00	0.00	0.00	0.00	0.00	0.00	0.00	0.00
golgin subfamily A member 6-like protein 2	LOC100 762276	100762276	0.00	0.02	0.00	0.00	0.00	0.00	0.76	0.92	1.24	2.32	0.00	0.00
proprotein convertase subtilisin/kexin type 5	LOC100 762283	100762283	0.00	0.00	0.00	0.00	0.00	0.00	0.00	0.00	0.00	0.00	0.00	0.00
polypyrimidine tract-binding protein 1-like	LOC100 762287	100762287	25.34	24.93	23.27	17.21	10.00	10.90	619.02	613.07	581.30	392.87	202.86	243.01
RNA-binding protein with serine-rich domain 1 pseudogene	LOC100 762289	100762289	0.21	0.15	0.27	0.13	0.11	0.05	3.80	2.75	4.97	2.32	2.07	0.93
olfactory receptor 52I2-like	LOC100 762292	100762292	0.00	0.00	0.00	0.00	0.00	0.00	0.00	0.00	0.00	0.00	0.00	0.00
paired box protein Pax-3	LOC100 762293	100762293	24.95	24.25	28.85	25.12	20.12	19.98	996.52	983.66	1172.53	1027.96	788.66	835.59
60S ribosomal protein L31 pseudogene	LOC100 762295	100762295	0.21	0.25	0.17	0.16	0.48	0.30	4.56	6.41	4.97	3.48	12.42	7.48
39S ribosomal protein L1, mitochondrial-like	LOC100 762296	100762296	0.00	0.00	0.00	0.00	0.00	0.00	0.00	0.00	0.00	0.00	0.00	0.00
high mobility group protein B1 pseudogene	LOC100 762298	100762298	20.62	16.76	13.50	13.62	16.27	20.11	217.99	179.35	136.63	143.71	170.77	221.52
KH homology domain-containing protein 1 pseudogene	LOC100 762305	100762305	0.00	0.00	0.00	0.00	0.00	0.00	0.00	0.00	0.00	0.00	0.00	0.00
receptor-type tyrosine-protein phosphatase V	LOC100 762307	100762307	0.08	0.12	0.02	0.02	0.05	0.01	14.43	21.96	3.73	2.32	9.31	1.87
zinc finger protein 670-like	LOC100 762310	100762310	5.97	6.18	6.36	8.13	8.67	7.12	564.34	561.83	556.45	690.72	734.84	668.29
chromosome unknown open reading frame, human C2orf49	LOC100 762312	100762312	6.51	6.00	5.47	5.81	6.70	6.91	833.21	770.46	711.72	748.66	848.69	892.61
mitochondrial import inner membrane translocase subunit Tim8 A pseudogene	LOC100 762327	100762327	93.69	76.28	66.99	60.06	39.26	45.78	410.15	326.67	281.95	216.72	140.76	166.37
uncharacterized LOC100762336	LOC100 762336	100762336	4.64	4.31	0.69	0.40	0.81	1.37	41.77	36.60	6.21	3.48	9.31	3.74

olfactory receptor 5D18-like	LOC100762342	100762342	0.00	0.00	0.00	0.00	0.00	0.00	0.00	0.00	0.00	0.00	0.00	0.00
olfactory receptor 2B2	LOC100762344	100762344	0.00	0.00	0.00	0.00	0.00	0.00	0.00	0.00	0.00	0.00	0.00	0.00
putative ankyrin repeat domain-containing protein ENSP00000383069	LOC100762352	100762352	0.00	0.00	0.00	0.00	0.00	0.00	0.00	0.00	0.00	0.00	0.00	0.00
phospholipid scramblase 2-like	LOC100762355	100762355	0.00	0.00	0.00	0.00	0.00	0.00	0.00	0.00	0.00	0.00	0.00	0.00
bystin-like	LOC100762361	100762361	0.52	0.67	0.64	0.50	0.59	0.60	18.99	23.79	37.26	27.81	19.66	26.17
interferon alpha-12-like	LOC100762362	100762362	0.00	0.00	0.00	0.00	0.00	0.09	0.00	0.00	0.00	0.00	0.00	0.93
chromosome unknown open reading frame, human CXorf57	LOC100762363	100762363	0.51	0.50	0.34	0.60	0.79	0.77	55.45	54.90	49.68	70.69	91.08	88.79
glyceraldehyde-3-phosphate dehydrogenase-like	LOC100762365	100762365	51.89	76.09	62.22	37.29	57.80	31.99	133.68	233.33	195.01	83.44	154.21	70.10
blood group Rh(D) polypeptide-like	LOC100762369	100762369	0.00	0.00	0.00	0.00	0.00	0.00	0.00	0.00	0.00	0.00	0.00	0.00
iroquois-class homeodomain protein IRX-2-like	LOC100762372	100762372	3.49	0.00	12.87	0.00	0.00	0.00	17.47	24.71	40.99	24.34	21.73	18.69
claudin-3-like	LOC100762373	100762373	0.07	0.17	0.58	0.22	0.24	0.22	1.52	4.58	13.66	5.79	5.17	6.54
cytochrome b-c1 complex subunit 7	LOC100762374	100762374	137.20	136.39	174.78	160.20	165.45	155.66	1434.01	1421.96	1783.64	1554.11	1520.39	1523.51
olfactory receptor 1468	LOC100762375	100762375	0.00	0.00	0.00	0.00	0.00	0.00	0.00	0.00	0.00	0.00	0.00	0.00
chromosome unknown open reading frame, human CXorf22	LOC100762376	100762376	0.00	0.00	0.00	0.00	0.00	0.00	0.00	0.00	0.00	0.00	0.00	0.00
interferon-inducible GTPase 1	LOC100762377	100762377	0.34	0.42	0.42	0.42	0.43	0.49	35.70	43.01	44.72	44.04	46.57	51.41
UDP-glucuronosyltransferase 2B17	LOC100762380	100762380	0.00	0.00	0.00	0.00	0.00	0.00	0.00	0.00	0.00	0.00	0.00	0.00
olfactory receptor 4X2-like	LOC100762381	100762381	0.00	0.00	0.00	0.00	0.00	0.00	0.00	0.00	0.00	0.00	0.00	0.00
olfactory receptor 13A1-like	LOC100762382	100762382	0.00	0.00	0.00	0.00	0.00	0.00	0.00	0.00	0.00	0.00	0.00	0.00

olfactory receptor 51A7	LOC100762388	100762388	0.00	0.00	0.00	0.00	0.00	0.00	0.00	0.00	0.00	0.00	0.00	0.00
tripartite motif-containing protein 30A-like	LOC100762389	100762389	0.21	0.24	0.55	0.52	0.34	0.36	14.43	16.47	38.50	38.24	23.80	28.04
phosphopantothenate--cysteine ligase pseudogene	LOC100762390	100762390	0.00	0.00	0.00	0.00	0.00	0.00	0.00	0.00	0.00	0.00	0.00	0.00
olfactory receptor 5-like	LOC100762403	100762403	0.00	0.00	0.00	0.00	0.00	0.00	0.00	0.00	0.00	0.00	0.00	0.00
uncharacterized protein C3orf38 homolog	LOC100762404	100762404	0.68	0.47	0.54	0.27	0.27	0.35	28.10	18.30	22.36	9.27	11.38	15.89
killer cell lectin-like receptor 2	LOC100762405	100762405	0.00	0.00	0.00	0.00	0.00	0.00	0.00	0.00	0.00	0.00	0.00	0.00
ATP synthase subunit epsilon, mitochondrial	LOC100762406	100762406	326.68	317.42	292.30	229.77	202.37	206.14	934.99	855.56	747.74	483.27	415.03	400.04
zinc finger MYND domain-containing protein 19 pseudogene	LOC100762412	100762412	0.00	0.00	0.00	0.00	0.00	0.00	0.00	0.00	0.00	0.00	0.00	0.00
eukaryotic translation initiation factor 5 pseudogene	LOC100762419	100762419	0.22	0.26	0.18	0.26	0.00	0.07	3.04	3.66	2.48	3.48	0.00	0.93
acidic leucine-rich nuclear phosphoprotein 32 family member E pseudogene	LOC100762421	100762421	0.00	0.00	0.00	0.00	0.00	0.00	0.00	0.00	0.00	0.00	0.00	0.00
chromosome unknown open reading frame, human C21orf33	LOC100762424	100762424	30.32	29.21	28.10	21.39	17.62	18.76	1147.66	1120.00	1101.73	841.38	692.41	748.67
phenazine biosynthesis-like domain-containing protein 2	LOC100762425	100762425	0.07	0.08	0.06	0.00	0.00	0.04	2.28	3.66	1.24	0.00	0.00	1.87
histone H2A type 1-F-like	LOC100762429	100762429	0.00	0.23	0.00	0.00	0.00	0.00	0.00	1.83	0.00	0.00	0.00	0.00
serine/Arginine-related protein 53-like	LOC100762434	100762434	2.92	2.50	2.65	3.51	2.70	2.25	65.32	58.56	62.10	81.12	60.03	49.54
eukaryotic translation initiation factor 4E pseudogene	LOC100762436	100762436	0.32	1.03	0.22	0.25	0.22	0.13	4.56	13.73	2.48	3.48	2.07	1.87
prolactin-7A1-like	LOC100762438	100762438	0.00	0.00	0.00	0.00	0.00	0.00	0.00	0.00	0.00	0.00	0.00	0.00
squamous cell carcinoma antigen recognized by T-cells 3	LOC100762447	100762447	34.75	33.55	31.71	29.35	25.93	29.63	3371.59	3268.50	3082.86	2846.31	2553.31	2880.65
L-aminoadipate-semialdehyde dehydrogenase-phosphopantetheinyl transferase pseudogene	LOC100762453	100762453	0.19	0.16	0.10	0.07	0.10	0.11	3.80	4.58	2.48	2.32	2.07	2.80

vomeronasal type-2 receptor 116-like	LOC100 762456	100762456	0.00	0.00	0.00	0.00	0.00	0.00	0.00	0.00	0.00	0.00	0.00	0.00
60S acidic ribosomal protein P1 pseudogene	LOC100 762462	100762462	0.51	0.25	0.51	0.00	0.00	0.46	2.28	0.92	2.48	0.00	0.00	1.87
trimeric intracellular cation channel type B pseudogene	LOC100 762466	100762466	0.23	0.09	0.18	0.12	0.29	0.12	4.56	1.83	3.73	2.32	5.17	1.87
zinc finger protein 2 homolog	LOC100 762468	100762468	0.00	0.02	0.02	0.00	0.09	0.03	0.00	0.92	4.97	2.32	5.17	2.80
chromosome unknown open reading frame, human CXorf40A	LOC100 762472	100762472	21.07	20.16	22.25	21.91	22.93	22.09	536.99	514.25	558.94	542.37	576.49	558.93
ADP-ribosylation factor 1 pseudogene	LOC100 762474	100762474	0.00	0.00	0.00	0.00	0.00	0.00	0.00	0.00	0.00	0.00	0.00	0.00
keratin-associated protein 13-1	LOC100 762476	100762476	0.00	0.00	0.00	0.00	0.00	0.00	0.00	0.00	0.00	0.00	0.00	0.00
interferon-induced very large GTPase 1-like	LOC100 762477	100762477	0.00	0.00	0.00	0.00	0.00	0.00	0.00	0.00	0.00	0.00	0.00	0.00
H-2 class I histocompatibility antigen, Q10 alpha chain-like	LOC100 762478	100762478	6.07	6.16	6.25	3.40	1.82	1.46	246.09	244.31	265.81	154.14	89.01	86.92
zinc finger protein 431-like	LOC100 762479	100762479	0.01	0.00	0.00	0.00	0.00	0.00	0.00	0.00	1.24	0.00	0.00	0.00
elongation factor 1-alpha 1 pseudogene	LOC100 762485	100762485	168.64	178.85	186.55	183.23	204.64	185.05	3180.95	3597.91	3859.16	3869.63	4220.67	3683.53
mitochondrial import inner membrane translocase subunit Tim23 pseudogene	LOC100 762487	100762487	6.64	6.87	7.47	7.47	7.01	6.67	128.36	131.76	155.26	141.39	132.48	129.92
60S ribosomal protein L17 pseudogene	LOC100 762489	100762489	0.41	0.53	0.18	0.23	0.09	0.12	3.04	4.58	1.24	2.32	0.00	0.93
	LOC100 762490	100762490	0.02	0.03	0.01	0.01	0.00	0.04	1.52	1.83	3.73	6.95	6.21	5.61
olfactory receptor 1468	LOC100 762498	100762498	0.00	0.00	0.00	0.00	0.00	0.00	0.00	0.00	0.00	0.00	0.00	0.00
protein atonal homolog 8-like	LOC100 762503	100762503	0.55	0.48	0.65	0.16	0.07	0.00	11.39	11.90	16.15	3.48	4.14	0.93
spliceosome RNA helicase DDX39B-like	LOC100 762504	100762504	41.78	40.78	54.94	53.95	50.86	49.65	53.17	42.09	52.17	45.20	69.34	38.32
melanoma-associated antigen B3-like	LOC100 762507	100762507	0.00	0.00	0.00	0.00	0.00	0.00	0.00	0.00	0.00	0.00	0.00	0.00

chromosome unknown open reading frame, human C3orf14	LOC100762512	100762512	12.17	12.68	9.44	9.85	13.49	12.55	815.75	838.17	624.77	630.45	861.11	815.97
small integral membrane protein 14 pseudogene	LOC100762522	100762522	0.10	0.12	0.11	0.00	0.14	0.04	0.76	0.00	2.48	1.16	2.07	0.93
ubiquitin-like	LOC100762524	100762524	0.51	0.62	0.47	0.84	0.36	0.17	3.04	2.75	2.48	5.79	2.07	2.80
glutamic acid-rich protein-like	LOC100762525	100762525	0.00	0.00	0.00	0.00	0.00	0.00	0.00	0.00	0.00	0.00	0.00	0.00
anionic trypsin-2	LOC100762526	100762526	0.00	0.00	0.00	0.00	0.00	0.00	0.00	0.00	0.00	0.00	0.00	0.00
putative vomeronasal receptor-like protein 4	LOC100762529	100762529	0.00	0.00	0.00	0.00	0.00	0.00	0.00	0.00	0.00	0.00	0.00	0.00
olfactory receptor 4C15	LOC100762534	100762534	0.00	0.00	0.00	0.00	0.00	0.00	0.00	0.00	0.00	0.00	0.00	0.00
glyceraldehyde-3-phosphate dehydrogenase pseudogene	LOC100762535	100762535	0.00	0.00	0.00	0.00	0.00	0.00	0.00	0.00	0.00	0.00	0.00	0.00
olfactory receptor 5M9	LOC100762538	100762538	0.00	0.00	0.00	0.00	0.00	0.00	0.00	0.00	0.00	0.00	0.00	0.00
small EDRK-rich factor 2 pseudogene	LOC100762543	100762543	12.30	10.61	11.79	8.44	11.24	7.90	6.08	4.58	3.73	2.32	8.28	3.74
histone H2B-like	LOC100762554	100762554	0.00	0.00	0.00	0.00	0.00	0.00	0.00	0.00	0.00	0.00	0.00	0.00
olfactory receptor 2M3	LOC100762559	100762559	0.00	0.00	0.00	0.00	0.00	0.00	0.00	0.00	0.00	0.00	0.00	0.00
probable RNA-binding protein EIF1AD pseudogene	LOC100762560	100762560	0.00	0.00	0.00	0.00	0.00	0.00	0.00	0.00	0.00	0.00	0.00	0.00
Ig heavy chain V-I region V35-like	LOC100762561	100762561	0.00	0.00	0.00	0.00	0.15	0.00						
olfactory receptor 4K3	LOC100762562	100762562	0.00	0.00	0.00	0.00	0.00	0.00	0.00	0.00	0.00	0.00	0.00	0.00
olfactory receptor 5AN1-like	LOC100762563	100762563	0.00	0.00	0.00	0.00	0.00	0.00	0.00	0.00	0.00	0.00	0.00	0.00
olfactory receptor 52J3	LOC100762564	100762564	0.00	0.00	0.00	0.00	0.00	0.00	0.00	0.00	0.00	0.00	0.00	0.00
C-type lectin domain family 2 member D11-like	LOC100762568	100762568	1.95	3.19	2.06	2.63	3.13	2.83	94.18	126.27	103.09	113.57	133.51	133.66

olfactory receptor 7E24-like	LOC100 762570	100762570	0.00	0.00	0.00	0.00	0.00	0.00	0.00	0.00	0.00	0.00	0.00	0.00
olfactory receptor 8B3-like	LOC100 762573	100762573	0.00	0.00	0.00	0.00	0.00	0.00	0.00	0.00	0.00	0.00	0.00	0.00
interferon-induced guanylate-binding protein 1-like	LOC100 762574	100762574	0.61	0.50	0.08	0.17	0.02	0.05	39.50	32.94	4.97	10.43	1.03	2.80
60S ribosomal protein L9 pseudogene	LOC100 762579	100762579	0.43	0.66	0.51	0.56	0.58	0.63	19.75	17.39	9.94	9.27	14.49	11.22
NADH dehydrogenase [ubiquinone] 1 alpha subcomplex subunit 2 pseudogene	LOC100 762584	100762584	0.00	0.00	0.00	0.00	0.00	0.00	0.00	0.00	0.00	0.00	0.00	0.00
serine/arginine-rich splicing factor 3 pseudogene	LOC100 762585	100762585	0.00	0.33	0.35	0.00	0.30	0.00	5.32	5.49	6.21	3.48	4.14	2.80
glyceraldehyde-3-phosphate dehydrogenase pseudogene	LOC100 762590	100762590	3.52	6.69	4.99	2.64	3.90	1.33	62.28	137.26	114.27	49.83	67.27	25.24
uncharacterized LOC100762592	LOC100 762592	100762592	0.00	0.00	0.00	0.00	0.00	0.00	0.00	0.00	0.00	0.00	0.00	0.00
tubulin alpha-1B chain-like	LOC100 762606	100762606	0.25	0.36	0.20	0.17	0.20	0.14	9.11	11.90	6.21	5.79	6.21	4.67
pre-mRNA-splicing factor SPF27 pseudogene	LOC100 762609	100762609	0.47	0.41	0.24	0.38	0.34	0.21	6.84	6.41	3.73	6.95	5.17	2.80
high mobility group protein B1-like	LOC100 762614	100762614	0.00	0.00	0.00	0.00	0.00	0.00	0.76	0.00	2.48	0.00	3.10	2.80
chromosome unknown open reading frame, human C20orf173	LOC100 762617	100762617	0.24	0.35	0.23	0.29	0.39	0.29	16.71	23.79	18.63	24.34	27.94	28.04
FGFR1 oncogene partner 2 homolog pseudogene	LOC100 762620	100762620	0.88	1.32	1.04	0.53	1.11	1.60	7.60	18.30	7.45	10.43	16.56	6.54
Microtubule-associated protein 1A	LOC100 762622	100762622	0.04	0.00	0.00	0.00	0.00	0.00	0.76	0.00	0.00	0.00	0.00	0.00
mas-related G-protein coupled receptor member H-like	LOC100 762623	100762623	0.03	0.00	0.00	0.00	0.00	0.00	0.76	0.92	0.00	0.00	0.00	0.00
60S acidic ribosomal protein P0-like	LOC100 762631	100762631	217.25	207.22	192.79	148.05	108.86	111.27	754.22	632.29	504.29	301.32	262.89	285.07
40S ribosomal protein S15 pseudogene	LOC100 762632	100762632	170.51	125.56	92.66	26.10	8.95	14.77	2826.25	2462.36	1707.87	504.13	167.67	223.39
olfactory receptor 5D18-like	LOC100 762637	100762637	0.00	0.00	0.00	0.00	0.00	0.00	0.00	0.00	0.00	0.00	0.00	0.00

chromosome unknown open reading frame, human C19orf71	LOC100762642	100762642	0.10	0.13	0.17	0.00	0.00	0.00	49.37	41.18	32.29	27.81	22.77	30.84
ribosome biogenesis regulatory protein homolog	LOC100762654	100762654	0.38	0.45	0.00	0.20	0.00	0.00	4.56	6.41	0.00	2.32	0.00	0.00
interferon alpha-9	LOC100762655	100762655	0.00	0.00	0.00	0.00	0.00	0.00	0.00	0.00	0.00	0.00	0.00	0.00
solute carrier family 23 member 2-like	LOC100762656	100762656	0.22	0.07	0.15	0.08	0.05	0.07	10.63	4.58	11.18	5.79	4.14	4.67
olfactory receptor 49-like	LOC100762660	100762660	0.00	0.00	0.00	0.00	0.00	0.00	0.00	0.00	0.00	0.00	0.00	0.00
centrosomal protein of 192 kDa-like	LOC100762665	100762665	0.07	0.07	0.07	0.06	0.05	0.04	4.56	3.66	3.73	4.64	4.14	2.80
zinc finger protein 239-like	LOC100762666	100762666	0.49	0.64	0.68	0.70	0.69	0.93	32.66	42.09	43.47	47.52	47.61	56.08
olfactory receptor 1496	LOC100762667	100762667	0.00	0.00	0.00	0.00	0.00	0.00	0.00	0.00	0.00	0.00	0.00	0.00
melanoma-associated antigen B16	LOC100762668	100762668	0.00	0.00	0.00	0.00	0.00	0.00	0.00	0.00	0.00	0.00	0.00	0.00
interferon-inducible GTPase 1-like	LOC100762669	100762669	0.00	0.00	0.00	0.00	0.00	0.00	0.00	0.00	0.00	0.00	0.00	0.00
UDP-glucuronosyltransferase 2A3	LOC100762673	100762673	0.04	0.04	0.00	0.00	0.00	0.00	1.52	1.83	0.00	0.00	0.00	0.00
olfactory receptor 13G1-like	LOC100762674	100762674	0.00	0.00	0.03	0.00	0.00	0.00	0.00	0.00	0.00	0.00	0.00	0.00
60S ribosome subunit biogenesis protein NIP7 homolog	LOC100762679	100762679	0.00	0.00	0.00	0.00	0.00	0.00	0.76	0.00	0.00	0.00	0.00	0.00
tripartite motif-containing protein 30A-like	LOC100762680	100762680	11.68	11.60	11.87	11.67	15.25	14.96	680.55	669.80	690.60	706.94	868.35	895.41
trimeric intracellular cation channel type B pseudogene	LOC100762685	100762685	0.10	0.09	0.08	0.00	0.12	0.02	3.04	4.58	2.48	0.00	3.10	0.00
uncharacterized protein ENSP00000372125 homolog	LOC100762686	100762686	0.11	0.05	0.06	0.17	0.10	0.16	6.08	2.75	4.97	9.27	9.31	9.35
interferon-induced guanylate-binding protein 2-like	LOC100762688	100762688	0.10	0.11	0.00	0.00	0.02	0.00	4.56	6.41	1.24	1.16	1.03	0.00
inactive ubiquitin carboxyl-terminal hydrolase 54	LOC100762693	100762693	8.23	8.15	8.92	9.69	11.14	11.18	950.18	948.89	1045.84	1118.36	1300.98	1303.86

serpin B8	LOC100762694	100762694	12.75	12.48	10.72	10.71	10.56	11.22	1353.50	1329.54	1134.03	1126.47	1095.02	1162.73
olfactory receptor 10A7	LOC100762695	100762695	0.00	0.00	0.00	0.00	0.00	0.00	0.00	0.00	0.00	0.00	0.00	0.00
acidic leucine-rich nuclear phosphoprotein 32 family member E-like	LOC100762696	100762696	0.00	0.12	0.33	0.00	0.00	0.00	3.80	2.75	9.94	1.16	3.10	4.67
peptidyl-prolyl cis-trans isomerase A-like	LOC100762697	100762697	0.00	0.00	0.00	0.00	0.00	0.00	0.00	0.00	0.00	0.00	0.00	0.00
cAMP-regulated phosphoprotein 19 pseudogene	LOC100762699	100762699	54.35	51.50	49.28	57.13	49.38	49.20	487.62	473.07	418.58	451.98	383.98	379.48
ubiquitin fusion degradation protein 1 homolog pseudogene	LOC100762700	100762700	0.83	0.97	0.72	0.75	1.15	0.60	15.95	20.13	16.15	13.91	20.70	8.41
cytochrome c oxidase subunit 8B, mitochondrial-like	LOC100762701	100762701	2.05	2.10	1.60	1.94	1.83	1.77	1.52	6.41	7.45	4.64	2.07	3.74
phenazine biosynthesis-like domain-containing protein	LOC100762717	100762717	0.19	0.40	0.23	0.04	0.07	0.03	6.84	14.64	4.97	2.32	1.03	0.00
histone H4	LOC100762720	100762720	0.04	0.19	0.20	0.00	0.00	0.00	1.52	1.83	2.48	0.00	0.00	0.93
serine/arginine repetitive matrix protein 1-like	LOC100762728	100762728	2.69	2.84	2.07	1.89	2.30	1.97	240.01	251.63	191.28	169.20	198.72	181.33
prolactin-7D1-like	LOC100762730	100762730	0.00	0.00	0.00	0.00	0.00	0.00	0.00	0.00	0.00	0.00	0.00	0.00
heat shock protein HSP 90-alpha-like	LOC100762732	100762732	0.43	0.48	0.22	0.17	0.10	0.12	25.06	28.37	12.42	10.43	6.21	7.48
ATP synthase subunit gamma, mitochondrial pseudogene	LOC100762734	100762734	0.00	0.00	0.00	0.00	0.00	0.00	0.00	0.00	0.00	0.00	0.00	0.00
nascent polypeptide-associated complex subunit alpha	LOC100762735	100762735	782.19	799.70	787.48	834.30	795.57	798.69	17856.77	18329.95	17833.88	18371.20	17804.87	17691.43
40S ribosomal protein S2 pseudogene	LOC100762736	100762736	0.33	1.14	0.46	0.59	0.72	0.24	5.32	18.30	7.45	9.27	11.38	3.74
nucleophosmin pseudogene	LOC100762737	100762737	4.91	4.88	4.51	3.27	3.71	3.02	48.61	57.65	60.86	44.04	50.71	35.52
cellular nucleic acid-binding protein pseudogene	LOC100762742	100762742	12.08	10.49	13.14	6.29	6.31	6.61	881.07	725.62	709.23	389.40	257.71	282.27
60S ribosomal protein L37 pseudogene	LOC100762744	100762744	9.70	11.48	10.04	6.73	11.98	5.55	41.02	47.58	36.02	20.86	44.50	21.50

putative gustatory receptor clone PTE03	LOC100762745	100762745	0.00	0.00	0.00	0.00	0.00	0.00	0.00	0.00	0.00	0.00	0.00	0.00
something about silencing protein 10-like	LOC100762748	100762748	0.06	0.04	0.05	0.00	0.00	0.00	2.28	2.75	1.24	1.16	0.00	0.00
60S ribosomal protein L31 pseudogene	LOC100762751	100762751	1.60	1.29	1.09	0.58	1.51	0.61	18.23	11.90	7.45	3.48	10.35	6.54
olfactory receptor 5B3-like	LOC100762752	100762752	0.00	0.00	0.00	0.00	0.00	0.00	0.00	0.00	0.00	0.00	0.00	0.00
phosphoglycerate kinase 1 pseudogene	LOC100762753	100762753	5.21	6.82	5.41	4.39	4.89	4.35	139.76	178.43	135.39	104.30	116.95	109.36
zinc finger protein 420-like	LOC100762754	100762754	3.01	3.30	3.95	4.31	5.19	3.53	242.29	267.19	314.25	339.56	402.61	287.88
heterogeneous nuclear ribonucleoprotein A3-like	LOC100762758	100762758	27.64	26.38	24.20	24.67	24.02	26.86	453.44	468.50	460.81	463.57	461.60	462.66
chromosome unknown open reading frame, human CXorf38	LOC100762759	100762759	13.30	13.85	14.04	14.12	13.73	15.52	1434.01	1512.55	1531.49	1518.19	1490.38	1665.58
40S ribosomal protein S8 pseudogene	LOC100762760	100762760	0.00	0.08	0.00	0.00	0.00	0.00	0.00	0.92	0.00	0.00	0.00	0.00
keratin-associated protein 13-1-like	LOC100762761	100762761	0.00	0.00	0.00	0.00	0.00	0.00	0.00	0.00	0.00	0.00	0.00	0.00
mamu class I histocompatibility antigen, alpha chain F-like	LOC100762763	100762763	4.12	3.02	4.20	3.45	2.21	3.11	14.43	21.96	21.12	10.43	10.35	11.22
olfactory receptor 2AT4	LOC100762766	100762766	0.00	0.00	0.00	0.00	0.00	0.00	0.00	0.00	0.00	0.00	0.00	0.00
protein lifeguard 2-like	LOC100762771	100762771	0.00	0.00	0.05	0.05	0.05	0.00	1.52	0.92	1.24	2.32	2.07	0.00
olfactory receptor 19	LOC100762779	100762779	0.00	0.00	0.00	0.00	0.00	0.00	0.00	0.00	0.00	0.00	0.00	0.00
acyl-protein thioesterase 1 pseudogene	LOC100762782	100762782	0.68	0.62	0.36	0.36	0.52	0.51	1.52	0.92	1.24	1.16	0.00	0.00
synaptonemal complex protein 3-like	LOC100762783	100762783	68.03	62.06	66.14	69.48	80.90	74.76	2200.39	2045.10	2131.42	2234.40	2585.40	2358.17
60 kDa heat shock protein, mitochondrial pseudogene	LOC100762784	100762784	3.11	3.16	3.18	2.06	2.64	2.48	186.09	198.56	173.89	104.30	108.67	97.21
gephyrin	LOC100762786	100762786	27.18	27.86	26.38	28.79	28.73	29.67	1190.96	1178.56	1155.14	1178.62	1216.11	1247.78

ephrin-B2-like	LOC100762789	100762789	13.62	11.75	11.69	13.18	14.01	15.52	280.27	276.34	269.53	311.75	350.86	375.74
olfactory receptor 2A1/2A42-like	LOC100762793	100762793	0.00	0.00	0.00	0.00	0.00	0.00	0.00	0.00	0.00	0.00	0.00	0.00
translationally-controlled tumor protein	LOC100762800	100762800	1.15	1.49	0.62	0.75	0.82	0.40	18.99	36.60	18.63	17.38	20.70	9.35
tryptase beta-2-like	LOC100762802	100762802	0.00	0.00	0.00	0.00	0.00	0.00	0.00	0.00	0.00	0.00	0.00	0.00
actin, cytoplasmic 2-like	LOC100762803	100762803	0.89	0.73	0.92	0.49	0.75	0.55	37.98	26.54	40.99	17.38	28.98	20.56
C-terminal-binding protein 2 pseudogene	LOC100762805	100762805	0.89	0.70	0.51	0.40	0.77	0.87	7.60	15.56	1.24	9.27	7.24	16.82
putative vomeronasal receptor-like protein 4	LOC100762816	100762816	0.00	0.00	0.00	0.00	0.00	0.00	0.00	0.00	0.00	0.00	0.00	0.00
zinc finger protein 780A-like	LOC100762819	100762819	0.77	0.58	0.77	0.69	1.16	0.87	34.18	28.37	36.02	34.77	54.85	40.19
olfactory receptor 4C16-like	LOC100762821	100762821	0.00	0.00	0.00	0.00	0.00	0.00	0.00	0.00	0.00	0.00	0.00	0.00
putative olfactory receptor 2W6	LOC100762822	100762822	0.00	0.00	0.00	0.00	0.00	0.00	0.00	0.00	0.00	0.00	0.00	0.00
olfactory receptor 1038	LOC100762825	100762825	0.00	0.00	0.00	0.00	0.00	0.00	0.00	0.00	0.00	0.00	0.00	0.00
60S ribosomal protein L31 pseudogene	LOC100762828	100762828	0.00	0.00	0.00	0.00	0.10	0.09	0.00	0.00	0.00	0.00	1.03	0.93
histone H2A-Bbd type 1	LOC100762832	100762832	0.00	0.00	0.00	0.00	0.00	0.00	0.00	0.00	0.00	0.00	0.00	0.00
interferon alpha-inducible protein 27-like protein 2A	LOC100762836	100762836	0.00	0.00	0.00	0.00	0.00	0.00	0.00	0.00	0.00	0.00	0.00	0.00
proteasome assembly chaperone 2 pseudogene	LOC100762848	100762848	0.00	0.00	0.00	0.00	0.03	0.00	0.00	0.00	0.00	0.00	0.00	0.00
olfactory receptor 5AN1-like	LOC100762849	100762849	0.00	0.00	0.00	0.00	0.00	0.00	0.00	0.00	0.00	0.00	0.00	0.00
olfactory receptor 52E4-like	LOC100762850	100762850	0.00	0.00	0.00	0.00	0.00	0.00	0.00	0.00	0.00	0.00	0.00	0.00
olfactory receptor 140	LOC100762854	100762854	0.00	0.00	0.00	0.00	0.00	0.00	0.00	0.00	0.00	0.00	0.00	0.00

60S ribosomal protein L23a pseudogene	LOC100762856	100762856	24.15	30.14	23.12	16.25	17.05	14.22	103.30	85.10	63.35	42.88	47.61	63.56
olfactory receptor 18-like	LOC100762857	100762857	0.00	0.00	0.00	0.00	0.00	0.00	0.00	0.00	0.00	0.00	0.00	0.00
olfactory receptor 145-like	LOC100762859	100762859	0.00	0.00	0.00	0.00	0.00	0.00	0.00	0.00	0.00	0.00	0.00	0.00
interferon-induced guanylate-binding protein 1	LOC100762860	100762860	0.03	0.12	0.00	0.02	0.00	0.01	2.28	8.24	0.00	1.16	0.00	0.93
inhibitor of growth protein 1 pseudogene	LOC100762870	100762870	11.08	9.64	12.07	10.22	10.21	10.56	183.81	161.05	217.37	198.18	171.81	214.97
cytochrome P450 2B1	LOC100762876	100762876	0.00	0.00	0.00	0.00	0.00	0.00	0.00	0.00	0.00	0.00	0.00	0.00
60S ribosomal protein L27-like	LOC100762878	100762878	0.63	0.00	0.00	0.21	0.36	0.16	5.32	0.00	0.00	3.48	3.10	0.93
chromosome unknown open reading frame, human C10orf131	LOC100762880	100762880	0.00	0.00	0.00	0.00	0.00	0.00	0.00	0.00	0.00	0.00	0.00	0.00
SNRPN upstream reading frame protein pseudogene	LOC100762881	100762881	0.00	0.00	0.00	0.00	0.00	0.00	0.00	0.00	0.00	0.00	0.00	0.00
carcinoembryonic antigen-related cell adhesion molecule 3-like	LOC100762882	100762882	0.00	0.00	0.00	0.00	0.00	0.00	0.00	0.00	0.00	0.00	0.00	0.00
olfactory receptor 7G2-like	LOC100762883	100762883	0.00	0.00	0.00	0.00	0.00	0.00	0.00	0.00	0.00	0.00	0.00	0.00
mitochondrial import inner membrane translocase subunit Tim21-like	LOC100762887	100762887	0.07	0.04	0.00	0.00	0.00	0.00	1.52	0.92	0.00	0.00	0.00	0.00
tubulin alpha-3 chain	LOC100762888	100762888	0.00	0.01	0.02	0.02	0.00	0.00	0.00	0.00	0.00	0.00	0.00	0.00
keratin, type I cytoskeletal 20	LOC100762889	100762889	0.00	0.00	0.00	0.00	0.00	0.00	0.00	0.00	0.00	0.00	0.00	0.00
taste receptor type 2 member 134-like	LOC100762892	100762892	0.00	0.00	0.00	0.00	0.00	0.00	0.00	0.00	0.00	0.00	0.00	0.00
uncharacterized LOC100762900	LOC100762900	100762900	18.38	19.49	12.96	14.47	14.62	11.59	363.06	387.06	264.56	273.51	276.34	241.14
chromosome unknown open reading frame, human C1orf43	LOC100762908	100762908	16.01	16.17	20.70	20.38	23.06	21.65	550.67	563.66	704.26	702.31	820.74	745.87
mas-related G-protein coupled receptor member X2-like	LOC100762911	100762911	0.00	0.00	0.00	0.00	0.00	0.00	0.00	0.00	0.00	0.00	0.00	0.00

39S ribosomal protein L35, mitochondrial pseudogene	LOC100762921	100762921	0.00	0.08	0.00	0.11	0.00	0.00	0.00	0.92	0.00	1.16	0.00	0.00
GRB2-associated-binding protein 2-like	LOC100762922	100762922	0.00	0.00	0.00	0.00	0.00	0.00	0.00	0.00	0.00	0.00	0.00	0.00
membrane primary amine oxidase	LOC100762926	100762926	0.00	0.01	0.00	0.00	0.00	0.01	0.00	0.92	0.00	0.00	0.00	0.93
sorting nexin-2 pseudogene	LOC100762927	100762927	2.76	2.63	2.33	1.68	1.24	1.25	123.05	111.63	94.40	71.85	49.68	52.34
olfactory receptor 5W2	LOC100762928	100762928	0.00	0.00	0.00	0.00	0.00	0.00	0.00	0.00	0.00	0.00	0.00	0.00
60S ribosomal protein L12 pseudogene	LOC100762931	100762931	0.00	0.09	0.00	0.00	0.00	0.00	0.00	0.92	0.00	0.00	1.03	0.93
isocitrate dehydrogenase [NADP] cytoplasmic pseudogene	LOC100762937	100762937	0.29	0.19	0.26	0.15	0.09	0.16	3.80	4.58	2.48	4.64	2.07	0.93
uncharacterized LOC100762940	LOC100762940	100762940	6.05	7.86	7.54	7.68	12.19	11.05	257.48	324.84	306.80	319.86	499.90	453.31
chromosome unknown open reading frame, human C6orf222	LOC100762947	100762947	0.00	0.00	0.00	0.00	0.00	0.00	0.00	0.92	0.00	0.00	0.00	0.00
claudin-3-like	LOC100762956	100762956	0.06	0.00	0.00	0.00	0.00	0.00	0.00	0.00	0.00	0.00	0.00	0.00
olfactory receptor 1496	LOC100762958	100762958	0.00	0.00	0.00	0.00	0.00	0.00	0.00	0.00	0.00	0.00	0.00	0.00
interferon-inducible GTPase 1	LOC100762960	100762960	0.00	0.00	0.00	0.00	0.00	0.00	0.00	0.00	0.00	0.00	0.00	0.00
olfactory receptor 13A1-like	LOC100762964	100762964	0.00	0.00	0.00	0.00	0.00	0.00	0.00	0.00	0.00	0.00	0.00	0.00
olfactory receptor 51G1	LOC100762969	100762969	0.00	0.00	0.00	0.00	0.00	0.00	0.00	0.00	0.00	0.00	0.00	0.00
actin, cytoplasmic 1-like	LOC100762971	100762971	0.08	0.10	0.00	0.09	0.04	0.00	2.28	2.75	0.00	2.32	1.03	0.00
60S ribosomal protein L22-like 1 pseudogene	LOC100762973	100762973	0.00	0.00	0.00	0.00	0.00	0.00	0.00	0.00	0.00	0.00	0.00	0.00
olfactory receptor 7E24-like	LOC100762974	100762974	0.00	0.00	0.00	0.00	0.00	0.00	0.00	0.00	0.00	0.00	0.00	0.00
guanylate-binding protein 4-like	LOC100762978	100762978	0.00	0.00	0.00	0.00	0.00	0.00	0.00	0.00	0.00	0.00	0.00	0.00

chromosome unknown open reading frame, human C9orf172	LOC100762980	100762980	0.03	0.03	0.00	0.02	0.00	0.01	4.56	2.75	4.97	2.32	0.00	0.93
olfactory receptor 5AR1-like	LOC100762985	100762985	0.00	0.00	0.00	0.00	0.00	0.00	0.00	0.00	0.00	0.00	0.00	0.00
nuclear factor of activated T-cells, cytoplasmic 2	LOC100762988	100762988	0.00	0.00	0.00	0.00	0.00	0.00	0.00	0.00	0.00	0.00	0.00	0.00
protein FAM228B	LOC100762989	100762989	3.45	2.97	4.10	4.77	4.61	4.75	142.03	143.66	167.68	191.22	185.26	199.08
chromosome unknown open reading frame, human C9orf142	LOC100762994	100762994	12.51	14.35	19.36	17.17	15.78	17.12	297.74	323.01	440.94	376.65	342.58	381.35
sperm motility kinase X-like	LOC100762996	100762996	0.05	0.03	0.06	0.08	0.05	0.05	3.80	0.92	4.97	4.64	4.14	3.74
serine protease inhibitor Kazal-type 7	LOC100763002	100763002	0.00	0.00	0.00	0.00	0.00	0.00	0.00	0.00	0.00	0.00	0.00	0.00
lipid phosphate phosphatase-related protein type 3	LOC100763012	100763012	0.03	0.02	0.02	0.02	0.02	0.00	3.04	1.83	1.24	1.16	1.03	0.93
histone H2A type 1-C	LOC100763014	100763014	0.00	0.00	0.00	0.00	0.00	0.00	0.00	0.00	0.00	0.00	0.00	0.00
doublesex- and mab-3-related transcription factor A1-like	LOC100763017	100763017	0.02	0.00	0.00	0.00	0.00	0.00	2.28	0.92	0.00	0.00	0.00	0.93
R3H domain-containing protein 2 pseudogene	LOC100763019	100763019	0.00	0.00	0.00	0.00	0.00	0.00	0.00	0.00	0.00	0.00	0.00	0.00
60S ribosomal protein L36a-like	LOC100763021	100763021	0.00	0.00	0.00	0.00	0.00	0.00	0.00	0.00	0.00	0.00	0.00	0.00
nuclear migration protein nudC pseudogene	LOC100763025	100763025	6.59	5.44	5.70	3.74	5.91	4.42	310.65	272.68	249.66	206.29	265.99	269.18
sperm motility kinase-like	LOC100763031	100763031	0.00	0.00	0.00	0.00	0.00	0.00	0.00	0.00	0.00	0.00	0.00	0.00
uncharacterized LOC100763035	LOC100763035	100763035	0.00	0.00	0.00	0.00	0.00	0.00	0.00	0.00	0.00	0.00	0.00	0.00
paired box protein Pax-3-like	LOC100763036	100763036	0.00	0.00	0.00	0.00	0.00	0.00	0.00	0.00	0.00	0.00	0.00	0.00
putative gustatory receptor clone PTE03	LOC100763037	100763037	0.00	0.00	0.00	0.00	0.00	0.00	0.00	0.00	0.00	0.00	0.00	0.00
enhancer of rudimentary homolog pseudogene	LOC100763040	100763040	0.00	0.00	0.00	0.00	0.00	0.00	0.00	0.00	0.00	0.00	0.00	0.00

olfactory receptor 5B3-like	LOC100 763045	100763045	0.00	0.00	0.00	0.00	0.00	0.00	0.00	0.00	0.00	0.00	0.00	0.00
transgelin-2 pseudogene	LOC100 763046	100763046	2.30	2.37	1.55	0.87	1.57	0.80	27.34	30.20	14.91	10.43	17.59	9.35
zinc finger protein 420-like	LOC100 763047	100763047	3.85	3.75	4.94	5.97	6.14	5.36	430.66	412.68	557.70	646.68	667.57	598.19
melanoma-associated antigen 11	LOC100 763051	100763051	0.79	0.99	0.72	0.60	0.62	0.53	16.71	20.13	13.66	11.59	13.45	15.89
glyceraldehyde-3-phosphate dehydrogenase-like	LOC100 763053	100763053	0.00	0.00	0.00	0.00	0.00	0.00	0.00	0.00	0.00	0.00	0.00	0.00
keratin-associated protein 13-1	LOC100 763055	100763055	0.00	0.00	0.00	0.00	0.00	0.00	0.00	0.00	0.00	0.00	0.00	0.00
tudor and KH domain-containing protein pseudogene	LOC100 763059	100763059	0.00	0.00	0.00	0.00	0.00	0.01	0.00	0.00	0.00	0.00	0.00	0.00
olfactory receptor 2AT4	LOC100 763061	100763061	0.00	0.00	0.00	0.00	0.00	0.00	0.00	0.00	0.00	0.00	0.00	0.00
ubiquitin-40S ribosomal protein S27a pseudogene	LOC100 763071	100763071	0.00	0.00	0.00	0.00	0.00	0.00	0.00	0.00	0.00	0.00	0.00	0.00
leucine-rich repeat-containing protein 3C-like	LOC100 763076	100763076	0.00	0.03	0.00	0.00	0.00	0.00	0.00	0.00	0.00	0.00	0.00	0.00
histone H2A-Bbd type 2/3	LOC100 763078	100763078	0.00	0.00	0.24	0.00	0.00	0.00	0.00	0.00	1.24	0.00	0.00	0.00
cytochrome c oxidase assembly protein COX11, mitochondrial	LOC100 763079	100763079	14.40	13.57	7.54	8.99	10.42	9.26	589.40	561.83	317.97	378.97	410.89	385.08
GTPase ERas-like	LOC100 763084	100763084	0.00	0.00	0.00	0.00	0.00	0.00	0.00	0.00	0.00	0.00	0.00	0.00
60S ribosomal protein L26-like 1 pseudogene	LOC100 763086	100763086	0.56	0.40	0.34	0.00	0.43	0.46	3.04	2.75	1.24	0.00	2.07	1.87
60S ribosomal protein L36 pseudogene	LOC100 763087	100763087	0.00	0.11	0.00	0.00	0.00	0.00	0.00	0.00	0.00	0.00	0.00	0.00
U6 snRNA-associated Sm-like protein LSm5 pseudogene	LOC100 763090	100763090	0.00	0.00	0.00	0.00	0.00	0.00	0.00	0.00	0.00	0.00	0.00	0.00
60S ribosomal protein L13a-like	LOC100 763092	100763092	0.03	0.00	0.00	0.00	0.00	0.00	0.00	0.00	0.00	0.00	0.00	0.00
transmembrane protein 92-like	LOC100 763094	100763094	0.00	0.00	0.00	0.00	0.00	0.00	0.00	0.00	0.00	0.00	0.00	0.00

serine/arginine-rich splicing factor 10-like	LOC100763095	100763095	0.54	0.56	0.89	0.59	0.80	0.47	0.00	0.00	0.00	0.00	0.00	0.00
rac GTPase-activating protein 1 pseudogene	LOC100763099	100763099	0.01	0.05	0.02	0.09	0.02	0.13	0.76	2.75	1.24	4.64	1.03	6.54
kinesin-like protein KIF18A pseudogene	LOC100763101	100763101	0.00	0.00	0.00	0.00	0.00	0.00	0.00	0.00	0.00	0.00	0.00	0.00
tudor and KH domain-containing protein pseudogene	LOC100763104	100763104	0.00	0.00	0.00	0.00	0.00	0.00	0.00	0.00	0.00	0.00	0.00	0.00
olfactory receptor 4C45-like	LOC100763116	100763116	0.00	0.00	0.00	0.00	0.00	0.00	0.00	0.00	0.00	0.00	0.00	0.00
olfactory receptor 5AL1	LOC100763119	100763119	0.00	0.00	0.00	0.00	0.00	0.00	0.00	0.00	0.00	0.00	0.00	0.00
chromosome unknown open reading frame, human C17orf97	LOC100763122	100763122	0.00	0.00	0.00	0.00	0.07	0.06	0.00	0.92	0.00	0.00	1.03	0.93
zinc finger protein 26-like	LOC100763123	100763123	1.92	1.67	2.36	2.38	2.41	2.34	0.00	0.00	0.00	0.00	1.03	0.00
putative small nuclear ribonucleoprotein polypeptide E-like protein 1	LOC100763125	100763125	0.00	0.00	0.00	0.00	0.00	0.00	0.00	0.00	0.00	0.00	0.00	0.00
interferon alpha-inducible protein 27-like protein 2	LOC100763130	100763130	0.61	0.68	0.61	0.47	0.23	0.61	0.76	2.75	3.73	3.48	1.03	3.74
chromosome unknown open reading frame, human C16orf80	LOC100763131	100763131	41.09	41.40	40.80	41.40	48.12	46.93	1153.74	1163.01	1172.53	1126.47	1323.75	1270.22
pyrethroid hydrolase Ces2e-like	LOC100763140	100763140	0.00	0.00	0.00	0.00	0.00	0.00	0.00	0.00	0.00	0.00	0.00	0.00
olfactory receptor 5AN1-like	LOC100763142	100763142	0.00	0.00	0.00	0.00	0.00	0.00	0.00	0.00	0.00	0.00	0.00	0.00
olfactory receptor 52A1	LOC100763143	100763143	0.00	0.00	0.00	0.00	0.00	0.00	0.00	0.00	0.00	0.00	0.00	0.00
olfactory receptor 4C3-like	LOC100763145	100763145	0.00	0.00	0.00	0.00	0.00	0.00	0.00	0.00	0.00	0.00	0.00	0.00
olfactory receptor 7E24-like	LOC100763148	100763148	0.00	0.00	0.00	0.00	0.00	0.00	0.00	0.00	0.00	0.00	0.00	0.00
olfactory receptor 145-like	LOC100763150	100763150	0.00	0.00	0.00	0.00	0.00	0.00	0.00	0.00	0.00	0.00	0.00	0.00
60S ribosomal protein L7a pseudogene	LOC100763152	100763152	1.95	1.35	1.63	1.16	0.88	0.92	6.08	4.58	6.21	4.64	1.03	3.74

ATP synthase subunit g, mitochondrial-like	LOC100763160	100763160	0.00	0.00	0.00	0.00	0.00	0.00	0.00	0.00	0.00	0.00	0.00	0.00
forkhead box protein P4-like	LOC100763161	100763161	0.02	0.09	0.03	0.00	0.05	0.05	0.00	0.92	0.00	0.00	0.00	0.00
uncharacterized protein C20orf24 homolog	LOC100763162	100763162	187.48	187.76	179.75	189.57	206.21	194.30	1906.44	2029.54	1812.20	1805.60	1924.04	1818.87
MRG/MORF4L-binding protein-like	LOC100763165	100763165	0.00	0.08	0.00	0.00	0.00	0.00	0.00	0.92	0.00	0.00	0.00	0.00
cytochrome P450 2B1-like	LOC100763166	100763166	0.00	0.00	0.02	0.00	0.00	0.00	0.00	0.00	0.00	0.00	0.00	0.00
chromosome unknown open reading frame, human C6orf201	LOC100763167	100763167	0.00	0.00	0.00	0.00	0.00	0.00	0.00	0.00	0.00	0.00	0.00	0.00
small nuclear ribonucleoprotein-associated protein N pseudogene	LOC100763171	100763171	0.00	0.00	0.00	0.00	0.00	0.00	0.00	0.00	0.00	0.00	0.00	0.00
carcinoembryonic antigen-related cell adhesion molecule 5-like	LOC100763172	100763172	0.00	0.00	0.00	0.00	0.00	0.00	0.00	0.00	0.00	0.00	0.00	0.00
phenylalanine--tRNA ligase alpha subunit pseudogene	LOC100763173	100763173	0.11	0.00	0.00	0.17	0.10	0.05	2.28	0.00	1.24	3.48	2.07	0.93
cytochrome b-c1 complex subunit 8	LOC100763175	100763175	128.39	139.39	148.77	121.82	153.21	124.09	441.29	421.83	396.23	271.19	338.44	271.05
olfactory receptor 51G2-like	LOC100763177	100763177	0.00	0.00	0.00	0.00	0.00	0.00	0.00	0.00	0.00	0.00	0.00	0.00
chromatin target of PRMT1 protein pseudogene	LOC100763179	100763179	0.07	0.00	0.02	0.00	0.00	0.02	1.52	0.92	0.00	0.00	0.00	0.00
sterile alpha motif domain-containing protein 5	LOC100763181	100763181	17.79	18.08	19.42	21.08	20.60	20.71	3674.65	3816.60	4154.78	4526.74	4365.57	4443.42
40S ribosomal protein S8 pseudogene	LOC100763182	100763182	0.00	0.00	0.00	0.00	0.00	0.00	0.00	0.00	0.00	0.00	0.00	0.00
probable GPI-anchored adhesin-like protein PGA55	LOC100763184	100763184	0.00	0.00	0.00	0.00	0.00	0.00	0.00	0.00	0.00	0.00	0.00	0.00
chromosome unknown open reading frame, human C2orf40	LOC100763185	100763185	0.09	0.00	0.00	0.00	0.00	0.00	0.76	0.00	0.00	2.32	1.03	0.00
chromosome unknown open reading frame, human C1orf189	LOC100763195	100763195	0.39	0.23	0.32	0.60	0.54	0.06	15.19	10.07	16.15	19.70	30.01	14.02
cell division cycle protein 27 homolog pseudogene	LOC100763196	100763196	0.75	0.81	0.54	0.60	0.62	0.73	18.99	28.37	16.15	12.75	22.77	17.76

mas-related G-protein coupled receptor member B2-like	LOC100763198	100763198	0.00	0.00	0.00	0.00	0.00	0.00	0.00	0.00	0.00	0.00	0.00	0.00
protein THEMIS3-like	LOC100763208	100763208	0.00	0.00	0.00	0.00	0.00	0.00	0.00	0.00	0.00	0.00	0.00	0.00
glyceraldehyde-3-phosphate dehydrogenase-like	LOC100763210	100763210	0.04	0.09	0.09	0.12	0.00	0.00	0.76	1.83	1.24	2.32	0.00	0.00
WAP four-disulfide core domain protein 15A-like	LOC100763213	100763213	0.00	0.00	0.00	0.00	0.00	0.00	0.00	0.00	0.00	0.00	0.00	0.00
olfactory receptor 5W2-like	LOC100763216	100763216	0.00	0.00	0.00	0.00	0.00	0.00	0.00	0.00	0.00	0.00	0.00	0.00
alpha-N-acetylgalactosaminide alpha-2,6-sialyltransferase 1	LOC100763224	100763224	0.00	0.00	0.00	0.02	0.00	0.01	0.00	0.00	0.00	0.00	0.00	0.00
solute carrier family 40 member 1-like	LOC100763228	100763228	0.00	0.00	0.00	0.00	0.00	0.00	0.00	0.00	0.00	0.00	0.00	0.00
interferon alpha-12-like	LOC100763235	100763235	0.00	0.00	0.00	0.00	0.00	0.00	0.00	0.00	0.00	0.00	0.00	0.00
60S ribosomal export protein NMD3 pseudogene	LOC100763236	100763236	0.00	0.00	0.00	0.05	0.00	0.00	0.00	0.00	0.00	1.16	0.00	0.00
cyclic nucleotide-gated cation channel beta-1-like	LOC100763240	100763240	0.00	0.00	0.00	0.00	0.00	0.00	0.00	0.00	0.00	0.00	0.00	0.00
protein MEMO1 pseudogene	LOC100763241	100763241	2.95	2.05	2.24	1.88	0.72	1.79	75.95	54.90	57.14	42.88	17.59	41.13
olfactory receptor 1496	LOC100763247	100763247	0.08	0.06	0.07	0.09	0.01	0.00	11.39	8.24	9.94	13.91	1.03	0.00
receptor-type tyrosine-protein phosphatase kappa	LOC100763249	100763249	10.15	11.06	0.03	0.00	0.00	0.00	429.90	410.85	1.24	0.00	0.00	0.00
sulfotransferase 1 family member D1	LOC100763252	100763252	0.11	0.07	0.00	0.00	0.00	0.00	7.60	4.58	0.00	0.00	0.00	0.00
olfactory receptor 13G1-like	LOC100763253	100763253	0.03	0.06	0.04	0.15	0.00	0.02	35.70	41.18	32.29	25.50	23.80	25.24
melanoma-associated antigen G1-like	LOC100763256	100763256	0.00	0.03	0.00	0.00	0.06	0.00	0.00	0.92	0.00	0.00	2.07	0.00
olfactory receptor 52R1-like	LOC100763258	100763258	0.00	0.00	0.00	0.00	0.00	0.00	0.00	0.00	0.00	0.00	0.00	0.00
olfactory receptor 52H1	LOC100763259	100763259	0.00	0.00	0.05	0.05	0.00	0.00	0.76	0.00	1.24	1.16	0.00	0.00

olfactory receptor 10-like	LOC100763260	100763260	0.00	0.00	0.00	0.00	0.00	0.00	0.00	0.00	0.00	0.00	0.00	0.00
C-C motif chemokine 7	LOC100763261	100763261	9.70	8.61	3.08	2.71	2.53	2.11	168.62	149.15	52.17	45.20	42.43	34.58
E3 ubiquitin-protein ligase RNF216 pseudogene	LOC100763262	100763262	0.00	0.00	0.00	0.00	0.00	0.00	0.00	0.00	0.00	0.00	0.00	0.00
olfactory receptor 7E24-like	LOC100763263	100763263	0.00	0.00	0.00	0.00	0.00	0.00	0.00	0.00	0.00	0.00	0.00	0.00
acidic leucine-rich nuclear phosphoprotein 32 family member E pseudogene	LOC100763264	100763264	4.79	7.25	5.07	3.99	5.13	3.54	87.35	129.93	91.91	67.22	94.18	66.36
high mobility group protein B1 pseudogene	LOC100763266	100763266	4.37	4.62	4.35	4.17	4.50	4.69	50.89	55.82	54.65	45.20	55.89	61.69
interferon-induced guanylate-binding protein 1-like	LOC100763267	100763267	0.00	0.00	0.00	0.00	0.00	0.00	0.00	0.00	0.00	0.00	0.00	0.00
chromosome unknown open reading frame, human C2orf16	LOC100763270	100763270	0.00	0.00	0.00	0.00	0.00	0.00	0.00	0.00	0.00	0.00	0.00	0.00
40S ribosomal protein S2 pseudogene	LOC100763271	100763271	1.19	0.89	0.70	0.15	0.26	0.55	25.06	18.30	13.66	2.32	4.14	11.22
olfactory receptor 8S1	LOC100763276	100763276	0.00	0.00	0.00	0.00	0.00	0.00	0.00	0.00	0.00	0.00	0.00	0.00
nucleoporin SEH1-like	LOC100763286	100763286	0.19	0.25	0.28	0.12	0.27	0.23	1.52	3.66	2.48	2.32	5.17	2.80
40S ribosomal protein S20 pseudogene	LOC100763287	100763287	0.00	0.00	0.00	0.00	0.00	0.00	0.76	0.00	0.00	0.00	0.00	0.00
signal peptidase complex subunit 3	LOC100763290	100763290	0.00	0.00	0.00	0.00	0.00	0.00	0.00	0.00	0.00	0.00	0.00	0.00
uncharacterized LOC100763301	LOC100763301	100763301	0.00	0.00	0.00	0.00	0.00	0.00	0.76	0.92	0.00	0.00	0.00	0.00
transmembrane epididymal protein 1-like	LOC100763303	100763303	0.00	0.00	0.00	0.00	0.00	0.00	0.00	0.00	0.00	0.00	0.00	0.00
speckle-type POZ protein-like	LOC100763306	100763306	0.00	0.03	0.00	0.00	0.00	0.00	0.00	0.92	0.00	0.00	0.00	0.00
prolactin-7C1-like	LOC100763308	100763308	0.00	0.00	0.00	0.00	0.00	0.00	0.00	0.00	0.00	0.00	0.00	0.00
splicing factor U2AF 65 kDa subunit	LOC100763310	100763310	44.49	44.03	37.35	29.96	21.07	23.20	1527.43	1517.13	1284.32	1032.60	724.49	798.21

olfactory receptor 11	LOC100 763311	100763311	0.00	0.00	0.00	0.00	0.00	0.00	0.00	0.00	0.00	0.00	0.00	0.00
high mobility group protein B1-like	LOC100 763315	100763315	0.04	0.05	0.00	0.00	0.05	0.08	0.76	0.92	0.00	0.00	1.03	0.93
40S ribosomal protein S12 pseudogene	LOC100 763318	100763318	0.00	0.24	0.00	0.00	0.00	0.00	4.56	7.32	2.48	3.48	0.00	0.00
uncharacterized LOC100763326	LOC100 763326	100763326	0.00	0.00	0.00	0.00	0.12	0.00	0.00	0.00	0.00	0.00	2.07	0.00
olfactory receptor 7G2-like	LOC100 763328	100763328	0.00	0.00	0.00	0.00	0.00	0.00	0.00	0.00	0.00	0.00	0.00	0.00
hepatitis A virus cellular receptor 1 homolog	LOC100 763331	100763331	0.00	0.00	0.00	0.00	0.00	0.00	0.00	0.00	0.00	0.00	0.00	0.00
transcription elongation factor B polypeptide 1 pseudogene	LOC100 763333	100763333	0.00	0.00	0.00	0.00	0.00	0.00	0.00	0.00	0.00	0.00	0.00	0.00
NADH dehydrogenase [ubiquinone] 1 alpha subcomplex subunit 3 pseudogene	LOC100 763334	100763334	0.00	0.00	0.00	0.00	0.00	0.00	0.00	0.00	0.00	0.00	0.00	0.00
olfactory receptor 5B2-like	LOC100 763338	100763338	0.00	0.00	0.00	0.00	0.00	0.00	0.00	0.00	0.00	0.00	0.00	0.00
ceramide synthase 2 pseudogene	LOC100 763341	100763341	0.05	0.15	0.00	0.00	0.00	0.09	3.04	4.58	0.00	1.16	0.00	3.74
dnaJ homolog subfamily A member 1 pseudogene	LOC100 763342	100763342	10.20	10.35	12.34	10.93	10.65	10.07	291.66	292.81	357.72	295.52	277.38	265.45
keratin-associated protein 13-1	LOC100 763349	100763349	0.00	0.00	0.00	0.00	0.00	0.00	0.00	0.00	0.00	0.00	0.00	0.00
maestro heat-like repeat family member 5	LOC100 763350	100763350	0.00	0.00	0.00	0.00	0.00	0.00	0.00	0.00	0.00	0.00	0.00	0.00
H-2 class I histocompatibility antigen, Q10 alpha chain-like	LOC100 763351	100763351	0.16	0.38	0.00	0.19	0.00	0.00	0.00	0.92	0.00	0.00	0.00	0.00
tudor and KH domain-containing protein pseudogene	LOC100 763352	100763352	0.07	0.04	0.00	0.05	0.04	0.04	0.76	0.00	0.00	0.00	0.00	0.00
olfactory receptor 2AT4	LOC100 763354	100763354	0.00	0.00	0.00	0.00	0.00	0.00	0.00	0.00	0.00	0.00	0.00	0.00
rRNA 2'-O-methyltransferase fibrillarin pseudogene	LOC100 763356	100763356	0.07	0.17	0.11	0.00	0.10	0.22	9.87	16.47	12.42	2.32	5.17	5.61
olfactory receptor 2AJ1-like	LOC100 763360	100763360	0.00	0.00	0.00	0.00	0.00	0.00	0.00	0.00	0.00	0.00	0.00	0.00

leukocyte immunoglobulin-like receptor subfamily B member 3	LOC100763364	100763364	0.00	0.01	0.00	0.00	0.00	0.00	0.00	0.92	0.00	0.00	0.00	0.00
40S ribosomal protein S21 pseudogene	LOC100763369	100763369	0.33	0.00	0.00	0.00	0.00	0.00	0.76	0.00	0.00	0.00	0.00	0.00
HMG domain-containing protein 4-like	LOC100763370	100763370	0.02	0.04	0.05	0.00	0.04	0.00	3.80	5.49	6.21	2.32	3.10	2.80
nucleophosmin pseudogene	LOC100763371	100763371	0.31	0.30	0.09	0.17	0.12	0.02	9.87	9.15	7.45	5.79	7.24	7.48
synaptojanin-2-binding protein	LOC100763373	100763373	14.93	14.34	13.12	13.91	15.80	14.99	1690.73	1625.10	1496.71	1583.08	1780.18	1712.31
Ig kappa chain V-I region HK102-like	LOC100763380	100763380	0.00	0.00	0.00	0.00	0.00	0.00						
U1 small nuclear ribonucleoprotein C pseudogene	LOC100763384	100763384	0.00	0.00	0.00	0.00	0.00	0.00	0.00	0.00	0.00	0.00	0.00	0.00
serine/arginine-rich splicing factor 10-like	LOC100763388	100763388	1.91	2.48	2.90	3.26	2.58	3.65	14.43	17.39	16.15	24.34	15.52	26.17
serine protease 29-like	LOC100763389	100763389	0.00	0.00	0.00	0.00	0.00	0.00	0.00	0.00	0.00	0.00	0.00	0.00
polyadenylate-binding protein 1 pseudogene	LOC100763393	100763393	0.54	0.74	0.51	0.14	0.08	0.15	34.18	40.26	34.78	8.11	4.14	6.54
heterogeneous nuclear ribonucleoproteins C1/C2 pseudogene	LOC100763396	100763396	0.04	0.10	0.00	0.00	0.00	0.00	3.80	10.07	6.21	0.00	5.17	1.87
olfactory receptor 9A2	LOC100763399	100763399	0.00	0.00	0.00	0.00	0.00	0.00	0.00	0.00	0.00	0.00	0.00	0.00
chromosome unknown open reading frame, human C17orf104	LOC100763401	100763401	0.00	0.01	0.00	0.00	0.00	0.01	0.00	1.83	0.00	0.00	0.00	0.93
olfactory receptor 4C45-like	LOC100763408	100763408	0.00	0.00	0.00	0.00	0.00	0.00	0.00	0.00	0.00	0.00	0.00	0.00
olfactory receptor 5AL1-like	LOC100763411	100763411	0.00	0.00	0.00	0.00	0.00	0.00	0.00	0.00	0.00	0.00	0.00	0.00
uncharacterized LOC100763417	LOC100763417	100763417	0.00	0.00	0.00	0.00	0.00	0.00	0.76	0.00	1.24	0.00	0.00	0.00
tropomyosin alpha-4 chain pseudogene	LOC100763420	100763420	1.81	2.03	1.38	0.95	1.92	1.15	39.50	41.18	24.84	20.86	40.36	23.37
interferon alpha-inducible protein 27-like protein 2A	LOC100763422	100763422	0.00	0.00	0.00	0.00	0.00	0.00	0.00	0.00	0.00	0.00	0.00	0.00

calponin-3 pseudogene	LOC100763427	100763427	6.44	6.44	5.98	4.17	3.09	3.10	245.33	236.08	208.67	120.53	84.87	81.32
UDP-glucuronosyltransferase 2B31-like	LOC100763430	100763430	0.00	0.02	0.00	0.00	0.03	0.00	0.00	0.92	0.00	0.00	1.03	0.00
phospholipase A2-like	LOC100763432	100763432	0.00	0.00	0.00	0.00	0.00	0.00	0.00	0.00	0.00	0.00	0.00	0.00
olfactory receptor 5A1	LOC100763434	100763434	0.00	0.00	0.00	0.00	0.00	0.00	0.00	0.00	0.00	0.00	0.00	0.00
olfactory receptor 52K1-like	LOC100763435	100763435	0.00	0.00	0.00	0.00	0.00	0.00	0.00	0.00	0.00	0.00	0.00	0.00
elongation of very long chain fatty acids protein 6	LOC100763438	100763438	7.18	7.49	7.58	8.76	8.85	7.58	628.90	647.84	671.97	763.73	762.78	669.22
olfactory receptor 8B3-like	LOC100763442	100763442	0.00	0.00	0.00	0.00	0.00	0.00	0.00	0.00	0.00	0.00	0.00	0.00
exocyst complex component 8-like	LOC100763446	100763446	0.00	0.00	0.00	0.00	0.00	0.00	0.00	0.00	0.00	0.00	0.00	0.00
zinc finger protein 260-like	LOC100763447	100763447	0.01	0.01	0.00	0.00	0.01	0.00	0.76	0.92	0.00	0.00	0.00	0.00
60S ribosomal protein L30-like	LOC100763448	100763448	0.77	2.42	3.76	1.78	1.31	0.91	16.71	30.20	42.23	34.77	14.49	20.56
protein NDRG3-like	LOC100763452	100763452	0.18	0.26	0.21	0.24	0.40	0.34	0.00	0.92	0.00	0.00	2.07	0.93
C-type lectin domain family 10 member A-like	LOC100763454	100763454	0.00	0.00	0.00	0.00	0.00	0.00	0.00	0.00	0.00	0.00	0.00	0.00
carcinoembryonic antigen-related cell adhesion molecule 3-like	LOC100763460	100763460	0.00	0.00	0.00	0.00	0.00	0.00	0.00	0.00	0.00	0.00	0.00	0.00
induced myeloid leukemia cell differentiation protein Mcl-1 homolog	LOC100763461	100763461	0.25	0.22	0.00	0.11	0.07	0.00	1.52	1.83	0.00	1.16	0.00	0.00
interleukin-36 receptor antagonist protein-like	LOC100763466	100763466	0.00	0.00	0.00	0.00	0.11	0.00	0.00	0.00	0.00	0.00	0.00	0.00
olfactory receptor 226-like	LOC100763468	100763468	0.00	0.00	0.00	0.00	0.00	0.00	0.00	0.00	0.00	0.00	0.00	0.00
dnaJ homolog subfamily B member 6-like	LOC100763476	100763476	0.47	0.57	0.62	0.37	0.52	0.41	8.35	10.07	9.94	5.79	8.28	6.54
chromosome unknown open reading frame, human C4orf46	LOC100763477	100763477	48.96	49.39	81.33	84.41	98.25	95.20	2809.54	2828.37	4705.02	4812.99	5625.15	5505.20

60S ribosomal protein L17 pseudogene	LOC100763483	100763483	0.16	0.13	0.09	0.00	0.00	0.00	1.52	1.83	1.24	4.64	2.07	2.80
mas-related G-protein coupled receptor member B2-like	LOC100763485	100763485	0.00	0.00	0.00	0.00	0.00	0.00	0.00	0.00	0.00	0.00	0.00	0.00
ubiquitin-conjugating enzyme E2 D2B	LOC100763486	100763486	0.00	0.00	0.00	0.00	0.00	0.00	0.00	0.00	0.00	0.00	0.00	0.00
N-acetyllactosaminide beta-1,6-N-acetylglucosaminyl-transferase	LOC100763488	100763488	0.00	0.00	0.00	0.00	0.00	0.00	0.00	0.00	0.00	0.00	0.00	0.00
high mobility group protein B1 pseudogene	LOC100763490	100763490	24.66	21.79	20.58	16.19	21.71	17.76	212.67	213.20	209.91	154.14	212.17	160.76
putative uncharacterized protein DDB_G0282133	LOC100763491	100763491	0.00	0.00	0.00	0.00	0.00	0.00	0.00	0.00	0.00	0.00	0.00	0.00
mitochondrial import inner membrane translocase subunit Tim23 pseudogene	LOC100763492	100763492	0.25	0.31	0.08	0.08	0.14	0.03	6.08	6.41	2.48	3.48	2.07	0.00
40S ribosomal protein S19-like	LOC100763493	100763493	0.00	0.00	0.00	0.00	0.00	0.00	0.76	0.92	1.24	0.00	0.00	0.93
40S ribosomal protein S16 pseudogene	LOC100763495	100763495	0.00	0.00	0.00	0.15	0.00	0.00	0.00	0.00	0.00	1.16	0.00	0.93
60S ribosomal protein L4 pseudogene	LOC100763496	100763496	0.24	0.31	0.08	0.27	0.19	0.07	7.60	10.07	2.48	8.11	5.17	1.87
antigen-presenting glycoprotein CD1d1-like	LOC100763497	100763497	3.46	3.24	3.14	3.18	2.85	3.08	167.86	153.73	144.08	158.77	152.14	156.09
multifunctional protein ADE2 pseudogene	LOC100763498	100763498	0.08	0.14	0.11	0.07	0.11	0.01	1.52	3.66	2.48	2.32	1.03	0.00
WAP four-disulfide core domain protein 15A-like	LOC100763499	100763499	0.00	0.00	0.00	0.00	0.00	0.00	0.00	0.00	0.00	0.00	0.00	0.00
olfactory receptor 5W2-like	LOC100763502	100763502	0.00	0.00	0.00	0.00	0.00	0.00	0.00	0.00	0.00	0.00	0.00	0.00
actin-related protein 2/3 complex subunit 5-like	LOC100763508	100763508	5.53	6.57	4.93	2.50	2.26	2.01	175.45	218.69	152.78	66.06	50.71	44.86
androgen-binding protein homolog	LOC100763511	100763511	0.00	0.00	0.00	0.00	0.00	0.00	0.00	0.00	0.00	0.00	0.00	0.00
protein SET-like	LOC100763515	100763515	0.22	0.23	0.17	0.05	0.14	0.12	3.80	4.58	2.48	1.16	3.10	1.87
interferon alpha-3	LOC100763520	100763520	0.00	0.00	0.00	0.00	0.00	0.00	0.00	0.00	0.00	0.00	0.00	0.00

uncharacterized LOC100763526	LOC100 763526	100763526	0.00	0.00	0.00	0.00	0.00	0.00	0.00	0.00	0.00	0.00	0.00	0.00
60S ribosomal protein L30-like	LOC100 763530	100763530	176.70	167.15	170.71	111.14	51.02	54.95	1276.79	1208.76	1179.98	717.37	335.34	339.29
olfactory receptor 1468-like	LOC100 763532	100763532	0.00	0.00	0.00	0.00	0.00	0.00	0.00	0.00	0.00	0.00	0.00	0.00
60S ribosomal protein L23-like	LOC100 763537	100763537	2.99	7.28	4.74	4.44	3.54	2.15	27.34	66.80	43.47	45.20	33.12	22.43
UDP-glucuronosyltransferase 2B7	LOC100 763538	100763538	0.00	0.02	0.00	0.00	0.00	0.00	0.00	0.92	0.00	0.00	0.00	0.00
olfactory receptor 13G1-like	LOC100 763539	100763539	0.00	0.00	0.00	0.00	0.00	0.00	0.00	0.00	0.00	0.00	0.00	0.00
cytochrome c-type heme lyase	LOC100 763541	100763541	19.09	19.36	19.62	19.44	22.39	20.88	1127.16	1134.64	1153.90	1134.58	1288.56	1206.66
olfactory receptor 51A4-like	LOC100 763543	100763543	0.00	0.00	0.00	0.00	0.00	0.00	0.00	0.00	0.00	0.00	0.00	0.00
alpha-enolase pseudogene	LOC100 763549	100763549	0.00	0.00	0.00	0.00	0.00	0.00	0.00	0.00	0.00	0.00	0.00	0.00
peptidyl-prolyl cis-trans isomerase A-like	LOC100 763550	100763550	2.30	2.87	1.84	1.28	1.36	1.51	28.10	30.20	17.39	13.91	15.52	16.82
interferon-induced guanylate-binding protein 1-like	LOC100 763553	100763553	0.00	0.00	0.00	0.00	0.00	0.00	0.00	0.00	0.00	0.00	0.00	0.00
butyrophilin-like protein 1	LOC100 763557	100763557	0.00	0.00	0.00	0.00	0.00	0.00	0.76	0.00	0.00	0.00	0.00	0.00
coiled-coil domain-containing protein 150	LOC100 763560	100763560	2.66	2.06	2.77	3.73	3.84	2.83	202.04	166.54	195.01	287.41	296.01	215.91
olfactory receptor 8S1	LOC100 763561	100763561	0.00	0.00	0.00	0.00	0.00	0.00	0.00	0.00	0.00	0.00	0.00	0.00
ATP-binding cassette sub-family G member 3-like	LOC100 763562	100763562	0.00	0.00	0.00	0.00	0.00	0.00	0.00	0.00	0.00	0.00	0.00	0.00
olfactory receptor 2T29	LOC100 763568	100763568	0.00	0.00	0.00	0.00	0.00	0.00	0.00	0.00	0.00	0.00	0.00	0.00
von Willebrand factor A domain-containing protein 5A-like	LOC100 763569	100763569	1.94	2.19	3.31	3.58	1.42	1.34	208.87	232.42	360.21	383.60	156.28	144.87
nucleoporin SEH1-like	LOC100 763571	100763571	0.07	0.04	0.12	0.04	0.05	0.04	0.00	0.00	1.24	0.00	0.00	0.00

ATP synthase subunit s, mitochondrial pseudogene	LOC100763572	100763572	0.00	0.00	0.00	0.00	0.00	0.00	0.00	0.00	0.00	0.00	0.00	0.00
olfactory receptor 51G2-like	LOC100763573	100763573	0.00	0.00	0.00	0.00	0.00	0.00	0.00	0.00	0.00	0.00	0.00	0.00
uncharacterized LOC100763576	LOC100763576	100763576	0.00	0.00	0.00	0.00	0.00	0.00	0.00	0.00	0.00	0.00	0.00	0.00
small nuclear ribonucleoprotein Sm D2 pseudogene	LOC100763579	100763579	3.84	6.15	4.21	4.85	3.46	1.81	135.96	158.30	110.55	121.69	93.15	96.27
transmembrane epididymal protein 1-like	LOC100763587	100763587	0.00	0.00	0.00	0.00	0.00	0.00	0.00	0.00	0.00	0.00	0.00	0.00
prolactin-4A1-like	LOC100763591	100763591	0.00	0.00	0.00	0.00	0.00	0.00	0.00	0.00	0.00	0.00	0.00	0.00
RNA-binding protein EWS pseudogene	LOC100763593	100763593	0.49	0.37	0.27	0.31	0.43	0.26	25.82	19.22	13.66	15.07	21.73	14.02
olfactory receptor 11-like	LOC100763594	100763594	0.00	0.00	0.00	0.00	0.00	0.00	0.00	0.00	0.00	0.00	0.00	0.00
polycomb group RING finger protein 6-like	LOC100763597	100763597	0.09	0.10	0.17	0.05	0.16	0.13	11.39	4.58	9.94	3.48	10.35	8.41
NADH dehydrogenase [ubiquinone] 1 beta subcomplex subunit 11, mitochondrial pseudogene	LOC100763602	100763602	0.00	0.00	0.00	0.00	0.00	0.00	0.76	0.00	0.00	0.00	0.00	0.00
uncharacterized LOC100763607	LOC100763607	100763607	0.00	0.00	0.00	0.00	0.00	0.00	0.00	0.00	0.00	0.00	0.00	0.00
hepatitis A virus cellular receptor 1 homolog	LOC100763611	100763611	0.00	0.00	0.00	0.00	0.00	0.00	0.00	0.00	0.00	0.00	0.00	0.00
acylcarnitine hydrolase	LOC100763617	100763617	0.04	0.00	0.00	0.00	0.00	0.00	1.52	0.00	0.00	0.00	0.00	0.00
chromosome unknown open reading frame, human C2orf81	LOC100763618	100763618	0.07	0.06	0.03	0.07	0.02	0.02	3.80	4.58	2.48	4.64	2.07	0.93
olfactory receptor 5B12-like	LOC100763619	100763619	0.00	0.00	0.00	0.00	0.00	0.00	0.00	0.00	0.00	0.00	0.00	0.00
pumilio domain-containing protein KIAA0020 pseudogene	LOC100763627	100763627	0.10	0.10	0.07	0.06	0.04	0.04	4.56	5.49	3.73	3.48	2.07	0.93
microtubule-associated protein futsch-like	LOC100763629	100763629	0.00	0.00	0.00	0.00	0.00	0.00	0.00	0.00	0.00	0.00	0.00	0.00
ribonuclease P protein subunit p21-like	LOC100763630	100763630	3.32	2.77	2.48	1.79	1.62	0.99	90.39	75.03	68.31	48.67	25.87	19.63

olfactory receptor 2L13-like	LOC100763638	100763638	0.00	0.00	0.00	0.00	0.00	0.00	0.00	0.00	0.00	0.00	0.00	0.00
cytochrome P450 2B1-like	LOC100763642	100763642	0.00	0.03	0.00	0.00	0.00	0.00	0.00	1.83	0.00	0.00	0.00	0.00
mitochondrial pyruvate carrier 1 pseudogene	LOC100763643	100763643	0.00	0.00	0.00	0.00	0.00	0.00	0.00	0.00	0.00	0.00	0.00	0.00
olfactory receptor 2AG2	LOC100763647	100763647	0.00	0.00	0.00	0.00	0.00	0.00	0.00	0.00	0.00	0.00	0.00	0.00
zinc finger X-linked protein ZXDB	LOC100763650	100763650	2.87	2.80	2.77	2.63	2.06	1.99	154.19	151.90	146.57	159.93	137.65	125.25
protein SET-like	LOC100763651	100763651	0.25	0.72	0.56	0.45	0.55	0.34	2.28	10.07	4.97	3.48	8.28	3.74
cytochrome P450 2B1-like	LOC100763652	100763652	0.00	0.00	0.00	0.00	0.00	0.00	0.00	0.00	0.00	0.00	0.00	0.00
high mobility group protein B1 pseudogene	LOC100763654	100763654	10.62	9.45	7.23	5.43	5.62	3.72	107.10	100.65	78.25	73.01	76.59	58.88
GTPase ERas-like	LOC100763655	100763655	0.00	0.00	0.00	0.00	0.00	0.00	0.00	0.00	0.00	0.00	0.00	0.00
putative 60S ribosomal protein L37a	LOC100763656	100763656	8.25	10.39	7.36	6.07	8.89	5.70	50.13	63.14	40.99	35.93	56.92	28.04
olfactory receptor 10V1	LOC100763657	100763657	0.42	0.51	0.58	0.65	0.65	0.61	25.82	33.86	40.99	45.20	54.85	42.06
Reprimo-like protein	LOC100763659	100763659	0.05	0.00	0.00	0.00	0.07	0.06	0.76	0.00	0.00	3.48	1.03	1.87
DPH3 homolog	LOC100763661	100763661	0.00	0.00	0.00	0.00	0.00	0.00	0.00	0.00	0.00	0.00	0.00	0.00
low affinity sodium-glucose cotransporter-like	LOC100763664	100763664	0.01	0.00	0.05	0.09	0.07	0.09	0.00	0.00	0.00	0.00	0.00	0.00
serine protease inhibitor A3N-like	LOC100763668	100763668	0.00	0.00	0.00	0.00	0.00	0.00	0.00	0.00	0.00	0.00	0.00	0.00
40S ribosomal protein S29 pseudogene	LOC100763669	100763669	0.00	0.00	0.00	0.00	0.00	0.00	0.00	0.00	0.00	0.00	0.00	0.00
serine protease 28-like	LOC100763670	100763670	0.00	0.00	0.00	0.00	0.00	0.00	0.00	0.00	0.00	0.00	0.00	0.00
protein SET-like	LOC100763674	100763674	8.78	8.47	8.11	7.27	7.80	7.23	24.31	33.86	36.02	32.45	31.05	30.84

olfactory receptor 5AL1-like	LOC100763692	100763692	0.00	0.00	0.00	0.00	0.00	0.00	0.00	0.00	0.00	0.00	0.00	0.00
40S ribosomal protein S24 pseudogene	LOC100763697	100763697	27.34	31.61	24.83	20.88	27.55	28.39	229.38	250.72	182.59	150.66	196.65	174.78
SAGA-associated factor 29 homolog pseudogene	LOC100763699	100763699	2.51	3.06	3.13	1.33	0.77	0.81	19.75	32.03	26.08	11.59	5.17	6.54
rhox homeobox family member 1-like	LOC100763700	100763700	0.00	0.00	0.00	0.00	0.00	0.00	0.00	0.00	0.00	0.00	0.00	0.00
40S ribosomal protein S26	LOC100763702	100763702	147.38	129.56	110.23	52.15	30.91	33.65	189.88	148.24	129.18	78.81	61.06	110.29
interferon alpha-inducible protein 27-like protein 2A	LOC100763703	100763703	0.00	0.00	0.00	0.00	0.00	0.00	0.00	0.00	0.00	0.00	0.00	0.00
Ig heavy chain V-III region VH26-like	LOC100763713	100763713	0.00	0.00	0.00	0.00	0.00	0.00	0.00	0.00	0.00	0.00	0.00	0.00
olfactory receptor 4D10-like	LOC100763716	100763716	0.00	0.00	0.00	0.00	0.00	0.00	0.00	0.00	0.00	0.00	0.00	0.00
olfactory receptor 52M1-like	LOC100763717	100763717	0.00	0.00	0.00	0.00	0.00	0.00	0.00	0.00	0.00	0.00	0.00	0.00
protein FAM76A pseudogene	LOC100763719	100763719	0.63	0.91	0.32	0.67	0.60	1.08	11.39	17.39	6.21	12.75	11.38	18.69
40S ribosomal protein S25 pseudogene	LOC100763720	100763720	0.26	0.16	0.67	0.44	0.28	0.35	1.52	0.00	2.48	0.00	1.03	0.00
olfactory receptor 10C1-like	LOC100763724	100763724	0.00	0.00	0.00	0.00	0.00	0.00	0.00	0.00	0.00	0.00	0.00	0.00
general transcription factor IIH subunit 5 pseudogene	LOC100763726	100763726	3.04	2.60	3.04	2.35	2.86	1.65	5.32	6.41	13.66	6.95	14.49	3.74
protein SET-like	LOC100763729	100763729	8.05	9.39	8.52	7.20	8.11	6.48	148.87	170.20	157.75	130.96	139.72	118.70
zinc finger protein 175	LOC100763730	100763730	0.00	0.00	0.00	0.00	0.09	0.00	0.00	0.00	0.00	0.00	0.00	0.00
proteasome inhibitor PI31 subunit-like	LOC100763731	100763731	0.24	0.23	0.27	0.27	0.18	0.20	3.04	2.75	4.97	3.48	2.07	1.87
developmental pluripotency-associated protein 2-like	LOC100763734	100763734	0.00	0.00	0.00	0.00	0.00	0.00	0.00	0.00	0.00	0.00	0.00	0.00
60S ribosomal protein L37 pseudogene	LOC100763735	100763735	0.00	0.00	0.00	0.00	0.00	0.00	0.00	0.00	0.00	0.00	0.00	0.00

aldose reductase pseudogene	LOC100 763739	100763739	0.00	0.00	0.00	0.00	0.00	0.00	0.00	0.00	0.00	0.00	0.00	0.00
carcinoembryonic antigen-related cell adhesion molecule 3-like	LOC100 763744	100763744	0.02	0.00	0.00	0.00	0.00	0.00	0.76	0.00	0.00	0.00	0.00	0.00
sperm motility kinase X-like	LOC100 763745	100763745	0.02	0.00	0.02	0.00	0.00	0.02	0.76	0.00	0.00	0.00	0.00	0.93
60S ribosomal protein L10-like	LOC100 763747	100763747	0.00	0.00	0.00	0.00	0.00	0.00	0.00	0.00	0.00	0.00	0.00	0.00
la-related protein 4 pseudogene	LOC100 763763	100763763	0.01	0.00	0.00	0.04	0.00	0.00	0.76	0.00	1.24	2.32	0.00	0.00
chromosome unknown open reading frame, human C17orf64	LOC100 763764	100763764	0.00	0.00	0.00	0.00	0.00	0.00	0.76	0.00	0.00	0.00	1.03	0.00
40S ribosomal protein S11-like	LOC100 763765	100763765	0.00	0.00	0.00	0.00	0.00	0.00	0.00	0.00	0.00	0.00	0.00	0.00
solute carrier family 2, facilitated glucose transporter member 3-like	LOC100 763766	100763766	0.00	0.00	0.00	0.00	0.00	0.00	0.00	0.00	0.00	0.00	0.00	0.00
dehydrogenase/reductase SDR family member 2, mitochondrial-like	LOC100 763767	100763767	0.00	0.00	0.00	0.00	0.00	0.00	0.00	0.00	0.00	0.00	0.00	0.00
ribose-5-phosphate isomerase-like	LOC100 763768	100763768	1.00	1.44	1.48	0.85	0.40	0.61	23.55	33.86	32.29	19.70	10.35	14.95
mas-related G-protein coupled receptor member X2-like	LOC100 763769	100763769	0.00	0.00	0.04	0.00	0.00	0.00	0.00	0.00	1.24	0.00	0.00	0.00
mitochondrial import receptor subunit TOM6 homolog pseudogene	LOC100 763770	100763770	1.64	0.33	1.89	0.00	0.00	0.00	5.32	3.66	4.97	1.16	0.00	0.00
uncharacterized LOC100763771	LOC100 763771	100763771	39.96	48.46	44.78	38.04	48.46	36.54	892.46	990.98	1069.44	986.24	1119.86	938.41
cytochrome c, somatic pseudogene	LOC100 763772	100763772	0.45	0.00	0.00	0.00	0.00	0.00	1.52	0.00	0.00	0.00	0.00	0.00
guanine nucleotide-binding protein G(I)/G(S)/G(T) subunit beta-1 pseudogene	LOC100 763777	100763777	0.00	0.00	0.00	0.00	0.00	0.04	0.00	0.00	0.00	0.00	0.00	0.93
40S ribosomal protein S2 pseudogene	LOC100 763781	100763781	0.04	0.00	0.00	0.00	0.00	0.00	0.76	0.00	0.00	0.00	0.00	0.00
40S ribosomal protein S15	LOC100 763784	100763784	0.00	0.00	0.00	0.00	0.00	0.00	0.00	0.00	0.00	0.00	0.00	0.00
olfactory receptor 5W2-like	LOC100 763785	100763785	0.00	0.00	0.00	0.00	0.00	0.00	0.00	0.00	0.00	0.00	0.00	0.00

olfactory receptor 2Y1-like	LOC100 763786	100763786	0.00	0.00	0.00	0.00	0.00	0.00	0.00	0.00	0.00	0.00	0.00	0.00
60S ribosomal protein L17	LOC100 763788	100763788	293.61	289.39	284.57	282.45	279.82	267.63	4912.70	5481.05	5177.02	4972.93	5141.81	4388.27
interferon-inducible GTPase 1	LOC100 763791	100763791	0.00	0.00	0.00	0.00	0.00	0.00	0.00	0.00	0.00	0.00	0.00	0.00
disintegrin and metalloproteinase domain-containing protein 21-like	LOC100 763794	100763794	0.01	0.02	0.00	0.02	0.00	0.03	0.76	0.92	0.00	1.16	1.03	1.87
major allergen I polypeptide chain 1-like	LOC100 763797	100763797	0.00	0.00	0.00	0.00	0.00	0.00	0.00	0.92	0.00	0.00	0.00	0.93
phospholipid scramblase 1-like	LOC100 763798	100763798	0.00	0.02	0.00	0.00	0.00	0.00	0.00	0.92	0.00	0.00	0.00	0.00
insulin-induced gene 1 protein pseudogene	LOC100 763805	100763805	0.00	0.00	0.00	0.00	0.00	0.00	0.00	0.00	0.00	0.00	0.00	0.00
barrier-to-autointegration factor pseudogene	LOC100 763811	100763811	2.21	2.02	1.91	1.94	0.80	0.76	6.08	6.41	6.21	4.64	4.14	1.87
chromosome unknown open reading frame, human C8orf46	LOC100 763812	100763812	0.00	0.00	0.00	0.00	0.00	0.00	0.00	0.00	0.00	0.00	0.00	0.00
taste receptor type 1 member 2	LOC100 763814	100763814	0.00	0.00	0.00	0.00	0.00	0.00	0.76	0.00	1.24	1.16	1.03	0.00
olfactory receptor 1L4-like	LOC100 763816	100763816	0.03	0.00	0.00	0.00	0.00	0.00	0.76	0.92	0.00	0.00	0.00	0.00
zinc finger protein OZF-like	LOC100 763819	100763819	3.23	3.28	3.50	3.36	2.94	3.08	394.96	402.61	439.70	394.03	370.53	394.43
protein arginine N-methyltransferase 1 pseudogene	LOC100 763821	100763821	0.06	0.07	0.00	0.09	0.00	0.30	3.04	2.75	1.24	2.32	3.10	6.54
UDP-glucuronosyltransferase 2B17-like	LOC100 763822	100763822	0.00	0.00	0.00	0.00	0.00	0.00	0.00	0.00	0.00	0.00	0.00	0.00
olfactory receptor 13A1	LOC100 763823	100763823	0.00	0.00	0.00	0.00	0.00	0.00	0.00	0.00	0.00	0.00	0.00	0.00
cytochrome c oxidase subunit 7C, mitochondrial pseudogene	LOC100 763827	100763827	0.88	0.89	0.00	0.52	0.85	0.81	3.80	2.75	3.73	1.16	5.17	1.87
olfactory receptor 51G2-like	LOC100 763828	100763828	0.00	0.00	0.00	0.00	0.00	0.00	0.00	0.00	0.00	0.00	0.00	0.00
tripartite motif-containing protein 6	LOC100 763830	100763830	3.34	3.71	4.34	4.78	4.79	4.20	454.20	502.35	572.60	665.22	643.76	580.43

glyceraldehyde-3-phosphate dehydrogenase pseudogene	LOC100763832	100763832	1.74	1.84	1.51	0.76	1.10	0.59	32.66	32.03	27.33	15.07	17.59	12.15
C-C motif chemokine 2	LOC100763833	100763833	129.15	157.63	81.22	99.66	131.30	114.03	2744.22	3359.09	1704.14	2062.88	2748.92	2389.95
hypothetical protein	LOC100763834	100763834	0.01	0.03	0.00	0.00	0.02	0.00	0.76	1.83	0.00	0.00	2.07	0.00
olfactory receptor 7E24-like	LOC100763835	100763835	0.00	0.00	0.00	0.00	0.00	0.00	0.00	0.00	0.00	0.00	0.00	0.00
CDGSH iron-sulfur domain-containing protein 1 pseudogene	LOC100763838	100763838	0.00	0.00	0.00	0.00	0.00	0.00	0.00	0.00	0.00	0.00	0.00	0.00
uncharacterized LOC100763842	LOC100763842	100763842	1.33	1.10	1.76	0.54	1.11	0.67	27.34	20.13	24.84	27.81	23.80	25.24
olfactory receptor 8S1	LOC100763846	100763846	0.00	0.00	0.00	0.00	0.00	0.00	0.00	0.00	0.00	0.00	0.00	0.00
actin-related protein 2 pseudogene	LOC100763848	100763848	6.71	6.25	6.85	6.26	7.07	6.97	173.17	159.22	168.92	156.45	174.91	169.18
histone H2A.Z	LOC100763852	100763852	351.63	360.55	354.10	333.16	340.09	355.82	4978.78	5093.08	5045.36	4622.93	4597.41	4733.17
olfactory receptor 2T29	LOC100763854	100763854	0.00	0.00	0.00	0.00	0.00	0.00	0.00	0.00	0.00	0.00	0.00	0.00
proteasome subunit alpha type-6 pseudogene	LOC100763858	100763858	0.00	0.00	0.00	0.00	0.00	0.00	0.00	0.00	0.00	0.00	0.00	0.00
KAT8 regulatory NSL complex subunit 1	LOC100763859	100763859	285.58	290.21	292.77	311.73	277.39	280.07	7219.42	7338.57	7468.67	7844.73	7287.34	7405.39
cytochrome c oxidase subunit 7A-related protein, mitochondrial pseudogene	LOC100763871	100763871	0.00	0.00	0.00	0.00	0.00	0.00	0.00	0.00	0.00	0.00	0.00	0.00
phosphatidylserine synthase 2-like	LOC100763873	100763873	2.65	2.96	2.68	3.05	2.47	3.10	91.90	104.31	88.19	110.10	85.90	110.29
transmembrane epididymal protein 1-like	LOC100763875	100763875	0.00	0.00	0.00	0.00	0.00	0.04	0.00	0.00	0.00	0.00	0.00	0.93
interferon-inducible GTPase 1-like	LOC100763878	100763878	0.00	0.03	0.08	0.00	0.10	0.06	1.52	0.92	2.48	0.00	3.10	1.87
prolactin-5A1-like	LOC100763880	100763880	0.00	0.00	0.00	0.00	0.00	0.00	0.00	0.00	0.00	0.00	0.00	0.00
NEDD8-conjugating enzyme UBE2F-like	LOC100763882	100763882	0.52	0.09	0.00	0.00	0.21	0.09	23.55	14.64	11.18	10.43	6.21	4.67

putative olfactory receptor 2W6	LOC100763883	100763883	0.00	0.04	0.00	0.00	0.00	0.00	0.00	0.92	0.00	0.00	0.00	0.00
zinc finger protein 431-like	LOC100763889	100763889	2.33	2.20	2.65	3.44	3.46	2.85	209.63	207.71	240.96	316.39	318.78	261.71
uncharacterized LOC100763894	LOC100763894	100763894	0.00	0.00	0.00	0.00	0.00	0.00	0.00	0.00	0.00	0.00	0.00	0.00
vomeronasal type-2 receptor 116-like	LOC100763896	100763896	0.00	0.00	0.00	0.00	0.00	0.00	0.00	0.00	0.00	0.00	0.00	0.00
40S ribosomal protein S15	LOC100763904	100763904	0.00	0.00	0.00	0.00	0.00	0.00	0.00	0.00	0.00	0.00	0.00	0.00
F-box only protein 34-like	LOC100763906	100763906	0.00	0.00	0.00	0.00	0.00	0.00	0.00	0.00	0.00	0.00	0.00	0.00
paternally-expressed gene 3 protein-like	LOC100763909	100763909	0.00	0.00	0.00	0.00	0.00	0.00	0.00	0.00	0.00	0.00	0.00	0.00
keratin-associated protein 13-1-like	LOC100763914	100763914	0.00	0.00	0.00	0.00	0.00	0.00	0.00	0.00	0.00	0.00	0.00	0.00
60S ribosomal protein L7a pseudogene	LOC100763918	100763918	0.17	0.21	0.16	0.02	0.23	0.21	5.32	6.41	4.97	2.32	8.28	1.87
60S ribosomal protein L9 pseudogene	LOC100763922	100763922	0.00	0.00	0.16	0.00	0.00	0.00	0.00	0.00	1.24	0.00	0.00	0.00
EF-hand calcium-binding domain-containing protein 1-like	LOC100763924	100763924	2.32	2.32	2.28	2.37	2.45	1.77	85.07	71.37	86.95	76.49	86.94	70.10
olfactory receptor 2L13	LOC100763925	100763925	0.00	0.00	0.00	0.00	0.00	0.00	0.00	0.00	0.00	0.00	0.00	0.00
leukocyte immunoglobulin-like receptor subfamily B member 3	LOC100763930	100763930	0.00	0.00	0.00	0.02	0.00	0.00	0.00	0.00	0.00	1.16	0.00	0.00
ATP synthase subunit g, mitochondrial pseudogene	LOC100763933	100763933	0.00	0.00	0.00	0.00	0.00	0.00	0.00	0.00	0.00	0.00	0.00	0.00
olfactory receptor 2AG2	LOC100763934	100763934	0.00	0.00	0.00	0.00	0.00	0.00	0.00	0.00	0.00	0.00	0.00	0.00
NUT family member Gm806-like	LOC100763938	100763938	0.00	0.00	0.00	0.00	0.00	0.00	0.00	0.00	0.00	0.00	0.00	0.00
zinc finger MYND domain-containing protein 19 pseudogene	LOC100763941	100763941	0.00	0.00	0.00	0.00	0.00	0.00	0.00	0.00	0.00	0.00	0.00	0.00
leucine-rich repeat-containing protein 3C-like	LOC100763945	100763945	0.00	0.03	0.00	0.00	0.00	0.00	0.00	0.00	0.00	0.00	0.00	0.00

pre-mRNA-splicing factor SLU7 pseudogene	LOC100763946	100763946	0.09	0.00	0.00	0.05	0.00	0.00	2.28	0.92	0.00	2.32	0.00	0.00
60S ribosomal protein L35a pseudogene	LOC100763948	100763948	4.12	6.08	3.37	3.46	3.55	2.77	25.82	35.69	19.87	18.54	21.73	14.02
39S ribosomal protein L27, mitochondrial pseudogene	LOC100763949	100763949	0.00	0.00	0.00	0.00	0.00	0.00	0.00	0.00	0.00	0.00	0.00	0.00
metal-response element-binding transcription factor 2-like	LOC100763952	100763952	0.06	0.00	0.03	0.03	0.00	0.00	2.28	0.00	1.24	1.16	1.03	0.00
amiloride-sensitive amine oxidase [copper-containing]	LOC100763954	100763954	6.04	6.95	3.43	4.24	4.64	5.09	431.42	500.52	248.42	302.48	330.16	359.85
serine protease inhibitor A3N-like	LOC100763955	100763955	0.00	0.00	0.00	0.00	0.00	0.00	0.00	0.00	0.00	0.00	0.00	0.00
protein FAM209A-like	LOC100763956	100763956	0.00	0.00	0.00	0.00	0.00	0.00	0.00	0.92	0.00	0.00	0.00	0.00
mastin-like	LOC100763957	100763957	0.00	0.00	0.00	0.00	0.05	0.00	0.00	0.00	0.00	0.00	1.03	0.00
40S ribosomal protein S11	LOC100763967	100763967	2.86	2.12	1.58	0.70	2.17	1.48	31.90	25.62	16.15	6.95	22.77	14.95
14-3-3 protein theta pseudogene	LOC100763969	100763969	0.77	0.96	0.53	0.43	0.58	0.38	22.79	24.71	16.15	8.11	12.42	9.35
60S ribosomal protein L7a pseudogene	LOC100763972	100763972	1339.56	1253.06	1273.62	1189.62	776.75	977.57	8282.78	7659.75	7635.11	6865.44	4678.14	5856.64
olfactory receptor 4C45-like	LOC100763977	100763977	0.00	0.00	0.00	0.00	0.00	0.00	0.00	0.00	0.00	0.00	0.00	0.00
olfactory receptor 1044	LOC100763980	100763980	0.00	0.00	0.00	0.00	0.00	0.00	0.00	0.00	0.00	0.00	0.00	0.00
diazepam-binding inhibitor-like 5	LOC100763983	100763983	13.75	15.47	13.21	18.27	19.16	13.69	165.58	183.92	163.96	195.86	204.93	175.72
60S ribosomal protein L34 pseudogene	LOC100763986	100763986	0.00	0.00	0.00	0.00	0.00	0.00	0.00	0.00	0.00	0.00	0.00	0.00
synaptosomal-associated protein 23-like	LOC100763992	100763992	0.00	0.00	0.00	0.00	0.00	0.00	0.00	0.00	0.00	0.00	0.00	0.00
mitochondrial import receptor subunit TOM20 homolog pseudogene	LOC100763997	100763997	4.84	5.52	4.76	3.40	5.16	3.30	57.72	75.03	59.62	40.56	69.34	39.26
transmembrane protease serine 11G	LOC100763999	100763999	0.00	0.00	0.00	0.00	0.00	0.00	0.00	0.00	0.00	0.00	0.00	0.00

cytochrome c oxidase subunit 6A1, mitochondrial	LOC100764001	100764001	248.82	237.22	233.74	178.39	146.86	161.75	3746.05	3575.95	3498.96	2576.28	2165.19	2355.37
olfactory receptor 4D11	LOC100764003	100764003	0.00	0.00	0.00	0.00	0.00	0.00	0.00	0.00	0.00	0.00	0.00	0.00
olfactory receptor 52M1-like	LOC100764004	100764004	0.00	0.00	0.00	0.00	0.00	0.00	0.00	0.00	0.00	0.00	0.00	0.00
mitochondrial import receptor subunit TOM7 homolog pseudogene	LOC100764005	100764005	0.00	0.00	0.00	0.00	0.00	0.00	0.00	0.00	0.00	0.00	0.00	0.00
olfactory receptor 4B1	LOC100764006	100764006	0.00	0.00	0.00	0.00	0.00	0.00	0.00	0.00	0.00	0.00	0.00	0.00
60S ribosomal protein L7 pseudogene	LOC100764007	100764007	0.62	0.96	0.66	0.14	0.43	0.20	4.56	5.49	6.21	0.00	2.07	0.93
RING-box protein 1-like	LOC100764010	100764010	0.47	0.19	0.00	0.00	0.22	0.00	6.84	5.49	2.48	0.00	2.07	0.93
olfactory receptor 10C1-like	LOC100764011	100764011	0.00	0.00	0.00	0.00	0.00	0.00	0.00	0.00	0.00	0.00	0.00	0.00
sialic acid-binding Ig-like lectin 12	LOC100764017	100764017	0.00	0.00	0.00	0.00	0.00	0.00	0.76	0.00	0.00	0.00	0.00	0.00
protoheme IX farnesyltransferase, mitochondrial	LOC100764018	100764018	7.82	7.98	8.79	9.05	6.29	7.52	434.46	444.71	493.11	487.91	373.63	449.58
pantothenate kinase 2, mitochondrial pseudogene	LOC100764019	100764019	0.00	0.00	0.00	0.00	0.17	0.00	3.04	3.66	8.69	3.48	10.35	3.74
apomucin-like	LOC100764026	100764026	0.00	0.00	0.00	0.00	0.00	0.00	0.00	0.00	0.00	0.00	0.00	0.00
Ig heavy chain V region PJ14-like	LOC100764029	100764029	0.00	0.00	0.00	0.00	0.00	0.00						
transmembrane 9 superfamily member 3-like	LOC100764032	100764032	0.51	0.47	0.28	0.07	0.32	0.23	21.27	21.05	14.91	2.32	13.45	8.41
heat shock 70 kDa protein 4 pseudogene	LOC100764039	100764039	0.14	0.09	0.00	0.04	0.10	0.09	5.32	4.58	0.00	1.16	5.17	2.80
hemoglobin subunit theta-1-like	LOC100764041	100764041	0.00	0.00	0.00	0.00	0.00	0.00	0.00	0.00	0.00	0.00	0.00	0.00
TAF5-like RNA polymerase II p300/CBP-associated factor-associated factor 65 kDa subunit 5L pseudogene	LOC100764049	100764049	0.06	0.05	0.05	0.04	0.08	0.03	1.52	0.92	0.00	0.00	1.03	0.93
chromosome unknown open reading frame, human C2orf50	LOC100764052	100764052	0.02	0.00	0.10	0.06	0.11	0.02	0.76	0.00	4.97	2.32	6.21	3.74

N-acetyllactosaminide beta-1,6-N-acetylglucosaminyl-transferase, isoform A-like	LOC100764057	100764057	0.00	0.00	0.00	0.00	0.00	0.00	0.00	0.00	0.00	0.00	0.00	0.00
E3 ubiquitin-protein ligase Praja-1-like	LOC100764065	100764065	0.25	0.22	0.13	0.12	0.14	0.18	6.84	5.49	3.73	2.32	4.14	2.80
seminal vesicle secretory protein 2-like	LOC100764067	100764067	0.00	0.00	0.00	0.00	0.00	0.00	0.00	0.00	0.00	0.00	0.00	0.00
60 kDa heat shock protein, mitochondrial pseudogene	LOC100764068	100764068	0.19	0.14	0.08	0.05	0.21	0.10	113.17	90.59	90.67	18.54	23.80	17.76
olfactory receptor 5W2-like	LOC100764069	100764069	0.00	0.00	0.00	0.00	0.00	0.00	0.00	0.00	0.00	0.00	0.00	0.00
olfactory receptor 2Y1	LOC100764070	100764070	0.00	0.00	0.00	0.00	0.00	0.00	0.00	0.00	0.00	0.00	0.00	0.00
eukaryotic translation initiation factor 1 pseudogene	LOC100764072	100764072	8.80	12.66	6.43	4.56	6.71	3.79	3130.82	3159.61	2331.40	2060.56	2276.97	2203.02
intelectin-1a	LOC100764079	100764079	0.13	0.14	0.00	0.11	0.00	0.03	6.08	6.41	0.00	4.64	0.00	0.93
Interferon alpha-inducible protein 27	LOC100764080	100764080	21.97	19.88	25.69	23.00	22.09	25.91	573.45	519.74	676.94	601.48	575.45	693.52
NHP2-like protein 1 pseudogene	LOC100764081	100764081	4.83	5.12	4.80	3.35	3.08	2.29	12.91	15.56	12.42	8.11	13.45	2.80
chromosome unknown open reading frame, human C1orf112	LOC100764089	100764089	13.99	14.00	15.52	17.29	20.42	20.32	1068.67	1098.96	1217.25	1308.42	1562.83	1508.56
hyaluronidase PH-20-like	LOC100764094	100764094	0.00	0.00	0.00	0.00	0.00	0.00	0.00	0.00	0.00	0.00	0.00	0.00
cytochrome c, somatic pseudogene	LOC100764096	100764096	4.93	5.15	5.07	3.09	4.82	3.97	37.98	40.26	37.26	19.70	37.26	28.04
metabotropic glutamate receptor 8	LOC100764098	100764098	0.00	0.00	0.00	0.00	0.00	0.00	0.00	0.00	0.00	0.00	0.00	0.00
acylcarnitine hydrolase-like	LOC100764100	100764100	0.01	0.00	0.00	0.00	0.00	0.00	0.00	0.00	0.00	1.16	0.00	0.00
putative olfactory receptor 3A4	LOC100764104	100764104	0.00	0.00	0.00	0.00	0.00	0.00	0.00	0.00	0.00	0.00	0.00	0.00
olfactory receptor 10W1-like	LOC100764105	100764105	0.00	0.00	0.00	0.00	0.00	0.00	0.00	0.00	0.00	0.00	0.00	0.00
thioredoxin domain-containing protein 9 pseudogene	LOC100764106	100764106	0.07	0.30	0.08	0.00	0.14	0.19	0.76	4.58	1.24	0.00	2.07	2.80

methylcytosine dioxygenase TET2-like	LOC100764108	100764108	0.00	0.00	0.00	0.00	0.00	0.00	0.00	0.00	0.00	0.00	0.00	0.00
UDP-glucuronosyltransferase 2B37-like	LOC100764111	100764111	0.00	0.00	0.00	0.00	0.00	0.00	0.00	0.00	0.00	0.00	0.00	0.00
olfactory receptor 13A1-like	LOC100764112	100764112	0.00	0.00	0.00	0.00	0.00	0.00	0.00	0.00	0.00	0.00	0.00	0.00
olfactory receptor 52D1	LOC100764117	100764117	0.00	0.00	0.00	0.00	0.00	0.00	0.00	0.00	0.00	0.00	0.00	0.00
olfactory receptor 52B6	LOC100764119	100764119	0.00	0.00	0.00	0.05	0.04	0.00	0.00	1.83	0.00	1.16	1.03	0.00
C-C motif chemokine 12-like	LOC100764122	100764122	0.00	0.00	0.00	0.00	0.00	0.00	0.00	0.00	0.00	0.00	0.00	0.00
transcription initiation factor IIA subunit 2 pseudogene	LOC100764124	100764124	12.07	11.15	10.96	6.15	3.68	5.02	157.98	146.41	137.87	74.17	48.64	62.62
alpha-enolase pseudogene	LOC100764125	100764125	0.00	0.00	0.00	0.00	0.00	0.00	0.00	0.00	0.00	0.00	0.00	0.00
glutaredoxin domain-containing cysteine-rich protein 1	LOC100764131	100764131	0.07	0.04	0.05	0.00	0.00	0.00	1.52	1.83	1.24	1.16	0.00	0.00
low molecular weight phosphotyrosine protein phosphatase pseudogene	LOC100764132	100764132	0.12	0.25	0.00	0.00	0.14	0.13	1.52	3.66	0.00	0.00	2.07	1.87
mitochondrial carrier homolog 2 pseudogene	LOC100764136	100764136	3.09	4.00	3.97	4.18	3.48	3.38	58.48	79.61	81.98	78.81	69.34	68.23
ATP-binding cassette sub-family G member 3-like	LOC100764139	100764139	0.00	0.00	0.00	0.00	0.00	0.00	0.00	0.00	0.00	0.00	0.00	0.00
60S ribosomal protein L15 pseudogene	LOC100764140	100764140	0.38	1.01	0.93	0.93	0.63	0.47	5.32	13.73	12.42	12.75	8.28	6.54
L-lactate dehydrogenase A chain-like	LOC100764142	100764142	12.39	12.16	10.18	6.70	10.37	8.23	201.28	173.86	145.32	77.65	139.72	103.75
olfactory receptor 2T29-like	LOC100764144	100764144	0.00	0.00	0.00	0.00	0.00	0.00	0.00	0.00	0.00	0.00	0.00	0.00
uncharacterized LOC100764147	LOC100764147	100764147	0.00	0.00	0.00	0.00	0.00	0.00	0.00	0.00	0.00	0.00	0.00	0.00
histone-lysine N-methyltransferase SETMAR	LOC100764148	100764148	0.67	0.66	0.39	0.58	0.52	0.57	61.52	58.56	34.78	52.15	46.57	53.28
GSK-3-binding protein FRAT2-like	LOC100764150	100764150	0.25	0.00	0.00	0.00	0.00	0.16	1.52	0.00	0.00	0.00	0.00	0.93

ferritin light chain 1	LOC100764157	100764157	0.00	0.15	0.00	0.00	0.00	0.00	0.76	1.83	1.24	0.00	0.00	0.00
cell differentiation protein RCD1 homolog	LOC100764166	100764166	0.00	0.00	0.00	0.00	0.00	0.05	0.76	3.66	1.24	0.00	0.00	0.93
Ig kappa chain V-IV region-like	LOC100764167	100764167	0.32	0.19	0.26	0.25	0.22	0.40						
olfactory receptor 56	LOC100764168	100764168	0.00	0.00	0.00	0.00	0.00	0.00	0.00	0.00	0.00	0.00	0.00	0.00
cytochrome P450 2J3	LOC100764171	100764171	13.18	14.53	14.03	16.03	18.13	17.33	782.33	888.50	848.35	943.36	1103.30	1050.57
chromobox protein homolog 3 pseudogene	LOC100764172	100764172	0.50	0.16	0.47	0.22	0.54	0.30	5.32	0.92	3.73	1.16	6.21	1.87
olfactory receptor 11-like	LOC100764173	100764173	0.00	0.00	0.00	0.00	0.00	0.00	0.00	0.00	0.00	0.00	0.00	0.00
mitochondrial inner membrane protein OXA1L	LOC100764174	100764174	36.35	36.36	33.25	32.38	31.67	34.09	1556.30	1577.52	1440.82	1382.59	1366.18	1466.50
transcription factor BTF3	LOC100764179	100764179	3.32	2.90	2.20	2.20	2.59	1.88	43.29	34.77	22.36	22.02	32.08	19.63
olfactory receptor 7G2-like	LOC100764188	100764188	0.00	0.00	0.00	0.00	0.00	0.00	0.00	0.00	0.00	0.00	0.00	0.00
ADP-ribosylation factor-related protein 1 pseudogene	LOC100764193	100764193	0.17	0.27	0.37	0.35	0.31	0.07	2.28	3.66	4.97	4.64	4.14	0.93
zinc finger protein 431-like	LOC100764206	100764206	7.30	7.02	7.54	8.40	8.63	8.62	518.77	493.20	537.82	587.57	587.87	600.99
keratin-associated protein 15-1	LOC100764209	100764209	0.00	0.00	0.00	0.00	0.00	0.00	0.00	0.00	0.00	0.00	0.00	0.00
elongation factor 1-alpha 1 pseudogene	LOC100764210	100764210	0.05	0.06	0.00	0.00	0.00	0.03	1.52	1.83	0.00	0.00	0.00	0.93
NUT family member Gm806-like	LOC100764212	100764212	0.00	0.00	0.00	0.00	0.00	0.00	0.00	0.00	0.00	0.00	0.00	0.00
membrane-spanning 4-domains subfamily A member 6D-like	LOC100764213	100764213	0.00	0.00	0.00	0.00	0.00	0.00	0.00	0.00	0.00	0.00	0.00	0.00
olfactory receptor 2L8-like	LOC100764220	100764220	0.00	0.00	0.00	0.00	0.00	0.00	0.00	0.00	0.00	0.00	0.00	0.00
40S ribosomal protein S3a pseudogene	LOC100764221	100764221	0.00	0.00	0.00	0.00	0.00	0.00	0.00	0.00	0.00	0.00	0.00	0.00

leukocyte immunoglobulin-like receptor subfamily B member 3	LOC100764224	100764224	0.00	0.00	0.00	0.00	0.00	0.00	0.00	0.00	0.00	0.00	0.00	0.00
PEST proteolytic signal-containing nuclear protein	LOC100764225	100764225	11.04	9.94	14.01	13.35	11.10	14.97	75.19	65.88	85.70	68.38	45.54	83.19
olfactory receptor 2AG1	LOC100764228	100764228	0.00	0.00	0.00	0.00	0.00	0.00	0.00	0.00	0.00	0.00	0.00	0.00
putative olfactory receptor GPCRLTM7	LOC100764233	100764233	0.00	0.00	0.00	0.00	0.00	0.00	0.00	0.00	0.00	0.00	0.00	0.00
chromatin target of PRMT1 protein pseudogene	LOC100764236	100764236	0.00	0.00	0.00	0.00	0.00	0.14	0.00	0.00	0.00	0.00	0.00	0.93
probable ribosome biogenesis protein RLP24 pseudogene	LOC100764238	100764238	0.00	0.10	0.07	0.00	0.06	0.16	0.00	0.00	0.00	0.00	0.00	0.93
tryptophan--tRNA ligase, cytoplasmic	LOC100764239	100764239	31.61	28.05	34.15	32.44	40.95	37.26	0.00	0.00	0.00	0.00	0.00	0.00
uncharacterized LOC100764240	LOC100764240	100764240	4.83	3.88	3.22	2.09	3.37	3.09	2.28	1.83	1.24	0.00	0.00	0.00
60S ribosomal protein L27 pseudogene	LOC100764241	100764241	0.00	0.00	0.00	0.00	0.08	0.00	0.00	0.00	0.00	0.00	1.03	0.00
core histone macro-H2A.2	LOC100764242	100764242	0.03	0.00	0.05	0.00	0.00	0.03	3.04	0.00	1.24	1.16	0.00	0.93
sperm motility kinase X-like	LOC100764243	100764243	0.00	0.00	0.00	0.00	0.00	0.02	0.00	0.00	0.00	0.00	0.00	0.93
chromosome unknown open reading frame, human C3orf62	LOC100764246	100764246	0.24	0.21	0.33	0.25	0.24	0.30	13.67	13.73	21.12	17.38	16.56	17.76
serine protease inhibitor A3N-like	LOC100764251	100764251	0.00	0.00	0.00	0.00	0.00	0.00	0.00	0.00	0.00	0.00	0.00	0.00
adenosine kinase pseudogene	LOC100764258	100764258	0.00	0.00	0.00	0.00	0.00	0.00	0.00	0.00	0.00	0.00	0.00	0.00
macrophage erythroblast attacher-like	LOC100764260	100764260	0.00	0.07	0.09	0.04	0.04	0.04	0.76	1.83	2.48	2.32	1.03	0.93
peptidyl-prolyl cis-trans isomerase A-like	LOC100764261	100764261	0.07	0.08	0.11	0.00	0.09	0.00	1.52	0.92	0.00	0.00	1.03	0.00
heterogeneous nuclear ribonucleoprotein H-like	LOC100764265	100764265	0.55	0.60	0.50	0.45	0.20	0.29	25.82	26.54	13.66	15.07	10.35	9.35
40S ribosomal protein S15a pseudogene	LOC100764272	100764272	0.49	2.12	1.49	1.14	0.90	0.16	3.04	11.90	9.94	4.64	5.17	0.93

olfactory receptor 4C12-like	LOC100764274	100764274	0.00	0.00	0.00	0.00	0.00	0.00	0.00	0.00	0.00	0.00	0.00	0.00
olfactory receptor 8J3	LOC100764277	100764277	0.00	0.00	0.00	0.00	0.00	0.00	0.00	0.00	0.00	0.00	0.00	0.00
E3 ubiquitin-protein ligase MARCH2 pseudogene	LOC100764279	100764279	0.00	0.00	0.00	0.00	0.00	0.00	0.00	0.00	0.00	0.00	0.00	0.00
arylsulfatase K pseudogene	LOC100764286	100764286	0.00	0.00	0.00	0.00	0.00	0.00	0.00	0.00	0.00	0.00	0.00	0.00
NADH dehydrogenase [ubiquinone] 1 beta subcomplex subunit 4 pseudogene	LOC100764292	100764292	0.00	0.00	0.00	0.00	0.00	0.00	0.00	0.00	1.24	2.32	1.03	0.00
ATP-binding cassette sub-family G member 3-like	LOC100764293	100764293	0.00	0.00	0.00	0.00	0.00	0.00	0.00	0.00	0.00	0.00	0.00	0.00
uncharacterized protein C8orf59 homolog pseudogene	LOC100764299	100764299	0.81	1.69	0.36	0.00	0.76	0.55	5.32	15.56	2.48	0.00	7.24	3.74
olfactory receptor 51G2	LOC100764300	100764300	0.00	0.00	0.00	0.00	0.00	0.00	0.00	0.00	0.00	0.00	0.00	0.00
olfactory receptor 4X1	LOC100764302	100764302	0.00	0.00	0.00	0.00	0.00	0.00	0.00	0.00	0.00	0.00	0.00	0.00
olfactory receptor 2B6	LOC100764308	100764308	0.00	0.00	0.00	0.00	0.00	0.00	0.00	0.00	0.00	0.00	0.00	0.00
chromosome unknown open reading frame, human C17orf74	LOC100764321	100764321	0.00	0.00	0.00	0.00	0.02	0.01	0.00	0.00	0.00	0.00	2.07	0.93
chromosome unknown open reading frame, human C17orf49	LOC100764322	100764322	15.28	14.23	15.98	16.02	13.66	17.46	470.15	454.77	480.69	482.11	388.12	470.14
U1 small nuclear ribonucleoprotein A pseudogene	LOC100764323	100764323	1.06	0.84	0.52	0.34	0.43	0.24	1.52	2.75	1.24	0.00	1.03	0.00
eukaryotic translation initiation factor 2 subunit 1 pseudogene	LOC100764326	100764326	0.03	0.00	0.05	0.00	0.04	0.00	0.76	0.00	2.48	0.00	1.03	0.00
attractin-like protein 1	LOC100764329	100764329	5.71	6.63	7.57	8.31	9.88	10.10	261.28	300.13	357.72	389.40	425.38	461.73
TBC1 domain family member 20 pseudogene	LOC100764330	100764330	0.00	0.00	0.00	0.00	0.00	0.00	0.00	0.00	0.00	0.00	0.00	0.00
U11/U12 small nuclear ribonucleoprotein 48 kDa protein pseudogene	LOC100764332	100764332	0.03	0.07	0.10	0.19	0.15	0.04	1.52	1.83	1.24	4.64	3.10	0.93
calcium-activated chloride channel regulator 4-like	LOC100764333	100764333	0.00	0.00	0.00	0.00	0.02	0.00	0.00	0.00	0.00	0.00	1.03	0.00

UBA-like domain-containing protein 1 pseudogene	LOC100764335	100764335	0.00	0.00	0.00	0.00	0.00	0.00	0.00	0.00	0.00	0.00	0.00	0.00
PRAME family member 10-like	LOC100764337	100764337	0.36	0.44	0.61	0.30	1.03	0.00	6.08	4.58	2.48	3.48	6.21	0.00
myocyte-specific enhancer factor 2B-like	LOC100764338	100764338	1.07	0.79	1.67	0.66	0.58	0.82	16.71	10.98	26.08	9.27	9.31	12.15
60S ribosomal protein L7a pseudogene	LOC100764343	100764343	1.86	1.34	1.31	1.08	1.22	1.00	7.60	4.58	3.73	2.32	3.10	8.41
60S ribosomal protein L24 pseudogene	LOC100764347	100764347	0.33	0.80	0.86	0.23	0.13	0.12	2.28	6.41	6.21	1.16	1.03	0.93
mas-related G-protein coupled receptor member B1-like	LOC100764353	100764353	0.00	0.00	0.00	0.00	0.00	0.00	0.00	0.00	0.00	0.00	0.00	0.00
protein tyrosine phosphatase type IVA 1	LOC100764354	100764354	0.70	0.65	0.94	0.60	1.54	0.96	7.60	5.49	13.66	8.11	15.52	13.09
protein transport protein Sec61 subunit gamma pseudogene	LOC100764356	100764356	1.43	1.00	0.18	0.18	0.62	0.00	9.87	6.41	1.24	1.16	3.10	0.00
guanine nucleotide-binding protein G(I)/G(S)/G(O) subunit gamma-4 pseudogene	LOC100764358	100764358	0.00	0.00	0.00	0.00	0.00	0.00	0.00	0.00	0.00	0.00	0.00	0.00
chromosome unknown open reading frame, human C9orf72	LOC100764363	100764363	7.30	6.27	5.38	5.51	6.52	6.78	717.76	616.73	529.13	540.06	636.52	664.55
seminal vesicle secretory protein 6-like	LOC100764366	100764366	0.00	0.00	0.00	0.00	0.00	0.00	0.00	0.00	0.00	0.00	0.00	0.00
olfactory receptor 10AG1-like	LOC100764368	100764368	0.00	0.00	0.00	0.00	0.00	0.00	0.00	0.00	0.00	0.00	0.00	0.00
chromosome unknown open reading frame, human C6orf136	LOC100764372	100764372	39.92	40.13	43.07	41.66	34.52	35.65	1245.64	1289.28	1374.99	1313.06	1120.89	1128.15
60S ribosomal protein L5 pseudogene	LOC100764377	100764377	0.00	0.00	0.00	0.00	0.00	0.00	0.00	0.00	0.00	0.00	0.00	0.00
protein slowmo homolog 2-like	LOC100764382	100764382	0.20	0.38	0.13	0.37	0.33	0.25	53.93	47.58	42.23	34.77	27.94	34.58
chromosome unknown open reading frame, human C16orf86	LOC100764389	100764389	0.21	0.18	0.16	0.00	0.14	0.10	7.60	5.49	4.97	2.32	4.14	4.67
mortality factor 4-like protein 1	LOC100764391	100764391	0.92	0.58	0.38	0.50	0.22	0.64	13.67	5.49	1.24	4.64	1.03	8.41
olfactory receptor 9I1	LOC100764398	100764398	0.00	0.00	0.00	0.00	0.00	0.00	0.00	0.00	0.00	0.00	0.00	0.00

zinc finger protein 431-like	LOC100764401	100764401	0.29	0.24	0.30	0.32	0.40	0.34	28.86	27.45	32.29	33.61	41.40	33.65
olfactory receptor 1D2	LOC100764404	100764404	0.00	0.00	0.00	0.00	0.00	0.00	0.00	0.00	0.00	0.00	0.00	0.00
olfactory receptor 10Q1	LOC100764405	100764405	0.00	0.00	0.00	0.00	0.00	0.00	0.00	0.00	0.00	0.00	0.00	0.00
vomeronasal type-2 receptor 116-like	LOC100764406	100764406	0.00	0.00	0.00	0.00	0.00	0.00	0.00	0.00	0.00	0.00	0.00	0.00
60S ribosomal protein L35a pseudogene	LOC100764410	100764410	0.28	0.00	0.00	0.00	0.00	0.00	5.32	3.66	1.24	2.32	4.14	3.74
scavenger receptor cysteine-rich domain-containing protein SCART1-like	LOC100764412	100764412	0.00	0.00	0.00	0.00	0.00	0.00	0.00	0.00	0.00	0.00	0.00	0.00
olfactory receptor 52J3-like	LOC100764417	100764417	0.00	0.00	0.00	0.00	0.00	0.00	0.00	0.00	0.00	0.00	0.00	0.00
ras GTPase-activating protein-binding protein 1 pseudogene	LOC100764418	100764418	14.06	13.50	11.65	8.54	5.92	7.26	666.12	622.22	603.65	438.07	279.45	338.35
olfactory receptor 52H1	LOC100764419	100764419	0.00	0.00	0.00	0.05	0.00	0.00	0.00	0.00	0.00	1.16	0.00	0.00
olfactory receptor 13C3-like	LOC100764423	100764423	0.00	0.00	0.00	0.00	0.00	0.00	0.00	0.00	0.00	0.00	0.00	0.00
olfactory receptor 7E24-like	LOC100764425	100764425	0.00	0.00	0.00	0.00	0.00	0.00	0.00	0.00	0.00	0.00	0.00	0.00
kinesin-like protein KIF11	LOC100764426	100764426	0.00	0.03	0.00	0.00	0.14	0.03	0.00	1.83	1.24	0.00	4.14	4.67
isocitrate dehydrogenase [NADP], mitochondrial	LOC100764431	100764431	8.91	9.44	11.54	12.26	8.36	11.53	32.66	40.26	45.96	45.20	17.59	38.32
60S ribosomal protein L18a pseudogene	LOC100764432	100764432	0.00	0.00	0.00	0.00	0.00	0.00	7.60	7.32	2.48	0.00	1.03	1.87
cytochrome b ascorbate-dependent protein 3	LOC100764433	100764433	10.28	11.15	10.07	10.52	10.55	11.95	509.65	515.16	478.20	506.45	533.02	640.25
chromosome unknown open reading frame, human C10orf11	LOC100764434	100764434	0.00	0.05	0.00	0.00	0.00	0.00	0.00	0.92	0.00	0.00	0.00	0.00
alpha-aminoadipic semialdehyde dehydrogenase pseudogene	LOC100764436	100764436	2.35	1.80	1.70	1.58	1.05	1.27	129.88	93.33	101.85	81.12	52.78	62.62
olfactory receptor 8S1	LOC100764437	100764437	0.00	0.00	0.00	0.00	0.00	0.00	0.00	0.00	0.00	0.00	0.00	0.00

polyadenylate-binding protein 4 pseudogene	LOC100764444	100764444	0.00	0.02	0.07	0.00	0.03	0.02	0.00	0.00	0.00	0.00	0.00	0.00
apolipoprotein L2-like	LOC100764446	100764446	0.00	0.00	0.00	0.00	0.00	0.00	0.00	0.00	0.00	0.00	0.00	0.00
olfactory receptor 958	LOC100764447	100764447	0.00	0.00	0.00	0.00	0.00	0.00	0.00	0.00	0.00	0.00	0.00	0.00
nucleoporin SEH1 pseudogene	LOC100764449	100764449	0.89	0.99	1.01	0.96	0.76	0.55	8.35	6.41	8.69	10.43	6.21	4.67
myeloid leukemia factor 2 pseudogene	LOC100764458	100764458	0.00	0.00	0.00	0.00	0.00	0.00	0.00	0.00	0.00	0.00	0.00	0.00
cytochrome c, testis-specific	LOC100764460	100764460	0.00	0.00	0.00	0.00	0.00	0.00	0.00	0.00	0.00	0.00	0.00	0.00
39S ribosomal protein L33, mitochondrial pseudogene	LOC100764467	100764467	0.00	0.00	0.00	0.00	0.00	0.00	0.00	0.00	0.00	0.00	0.00	0.00
Ig kappa chain V-III region MOPC 63-like	LOC100764468	100764468	0.00	0.00	0.00	0.00	0.00	0.00						
olfactory receptor 2M3	LOC100764469	100764469	0.00	0.00	0.00	0.00	0.00	0.00	0.00	0.00	0.00	0.00	0.00	0.00
cytochrome P450 2J3-like	LOC100764471	100764471	0.38	0.44	0.42	0.40	0.84	0.73	26.58	32.94	22.36	25.50	52.78	46.73
putative olfactory receptor 2W6	LOC100764473	100764473	0.00	0.00	0.00	0.00	0.00	0.00	0.00	0.00	0.00	0.00	0.00	0.00
uncharacterized LOC100764481	LOC100764481	100764481	11.31	11.71	7.60	7.71	8.64	8.57	521.80	545.36	351.51	348.83	397.43	393.50
olfactory receptor 7G2-like	LOC100764488	100764488	0.00	0.00	0.00	0.00	0.00	0.00	0.00	0.00	0.00	0.00	0.00	0.00
integral membrane protein 2C-like	LOC100764494	100764494	1.76	1.90	1.47	1.32	1.53	1.35	28.86	29.28	21.12	19.70	20.70	18.69
elongation factor 1-alpha 1-like	LOC100764499	100764499	0.00	0.00	0.00	0.00	0.00	0.00	0.00	0.00	0.00	0.00	0.00	0.00
26S protease regulatory subunit 10B pseudogene	LOC100764500	100764500	0.00	0.00	0.00	0.00	0.00	0.00	0.00	0.00	0.00	0.00	0.00	0.00
zinc finger protein 431-like	LOC100764503	100764503	3.02	3.05	3.54	3.78	4.50	1.47	53.17	48.50	39.75	52.15	60.03	38.32
Golgin subfamily A member 2	LOC100764507	100764507	0.13	0.08	0.05	0.16	0.00	0.17	0.00	0.00	0.00	0.00	0.00	0.00

NUT family member Gm806-like	LOC100 764509	100764509	0.00	0.00	0.00	0.00	0.00	0.00	0.00	0.00	0.00	0.00	0.00	0.00
olfactory receptor 2L13-like	LOC100 764516	100764516	0.00	0.00	0.00	0.00	0.00	0.00	0.00	0.00	0.00	0.00	0.00	0.00
U1 small nuclear ribonucleoprotein C-like	LOC100 764520	100764520	7.39	7.20	8.03	6.85	6.98	8.39	316.73	333.99	376.35	324.50	297.04	357.98
PEST proteolytic signal-containing nuclear protein-like	LOC100 764521	100764521	20.59	19.12	24.32	28.80	27.24	27.09	126.08	107.97	155.26	171.52	139.72	157.02
olfactory receptor 2D3-like	LOC100 764524	100764524	0.00	0.00	0.00	0.00	0.00	0.00	0.00	0.00	0.00	0.00	0.00	0.00
ornithine decarboxylase pseudogene	LOC100 764525	100764525	0.00	0.00	0.00	0.00	0.00	0.00	0.00	0.00	0.00	0.00	0.00	0.00
uncharacterized LOC100764528	LOC100 764528	100764528	0.00	0.00	0.00	0.00	0.00	0.00	0.00	0.00	0.00	0.00	0.00	0.00
olfactory receptor 4K3-like	LOC100 764529	100764529	0.02	0.00	0.00	0.00	0.00	0.00	0.76	0.00	0.00	0.00	0.00	0.00
60S ribosomal protein L23-like	LOC100 764530	100764530	0.19	0.34	0.21	0.00	0.00	0.00	2.28	4.58	1.24	0.00	1.03	0.00
nucleoporin SEH1 pseudogene	LOC100 764532	100764532	0.14	0.05	0.01	0.00	0.02	0.01	0.76	0.00	0.00	0.00	0.00	0.00
MORF4 family-associated protein 1-like	LOC100 764537	100764537	0.00	0.00	0.00	0.00	0.00	0.19	0.00	0.00	0.00	1.16	0.00	0.93
chromosome unknown open reading frame, human C10orf35	LOC100 764539	100764539	0.00	0.00	0.00	0.00	0.00	0.00	0.00	0.00	0.00	0.00	0.00	0.00
eukaryotic initiation factor 4A-I pseudogene	LOC100 764541	100764541	2.96	2.86	1.92	1.64	2.02	1.82	81.27	81.44	53.41	45.20	55.89	49.54
chromosome unknown open reading frame, human C21orf58	LOC100 764544	100764544	0.00	0.00	0.06	0.00	0.05	0.00	2.28	4.58	4.97	6.95	5.17	3.74
serine protease inhibitor A3N	LOC100 764548	100764548	0.00	0.00	0.00	0.00	0.00	0.00	0.00	0.00	0.00	0.00	0.00	0.00
mitochondrial fission factor pseudogene	LOC100 764551	100764551	0.98	0.84	0.68	0.36	0.61	0.48	20.51	21.05	13.66	16.22	14.49	16.82
protein SET-like	LOC100 764554	100764554	0.04	0.14	0.00	0.00	0.00	0.00	0.00	2.75	0.00	0.00	0.00	0.00
vomeronasal type-1 receptor 90-like	LOC100 764566	100764566	0.00	0.00	0.00	0.00	0.00	0.00	0.00	0.00	0.00	0.00	0.00	0.00

60 kDa heat shock protein, mitochondrial pseudogene	LOC100764571	100764571	1.84	2.09	2.02	1.18	1.38	1.10	77.47	86.01	83.22	49.83	58.99	42.99
phosphoglycerate kinase 1 pseudogene	LOC100764573	100764573	0.00	0.00	0.00	0.00	0.00	0.00	0.00	0.00	0.00	0.00	0.00	0.00
olfactory receptor 8K1	LOC100764574	100764574	0.00	0.00	0.00	0.00	0.00	0.00	0.00	0.00	0.00	0.00	0.00	0.00
40S ribosomal protein S21 pseudogene	LOC100764581	100764581	24.47	27.02	16.72	4.23	2.92	3.46	106.34	120.78	73.28	20.86	13.45	11.22
sperm motility kinase-like	LOC100764588	100764588	0.00	0.00	0.00	0.00	0.00	0.00	0.00	0.00	0.00	0.00	0.00	0.00
coiled-coil domain-containing protein 91	LOC100764589	100764589	23.89	23.90	26.89	26.40	27.92	27.31	554.46	567.32	633.46	617.70	657.22	641.18
guanylate-binding protein 6-like	LOC100764590	100764590	0.03	0.00	0.00	0.00	0.00	0.00	0.76	0.00	0.00	0.00	0.00	0.00
phospholipase A2	LOC100764593	100764593	0.00	0.00	0.00	0.00	0.00	0.00	0.00	0.00	0.00	0.00	0.00	0.00
liver carboxylesterase-like	LOC100764594	100764594	0.00	0.00	0.00	0.00	0.00	0.00	0.00	0.00	0.00	0.00	0.00	0.00
olfactory receptor 52K1-like	LOC100764599	100764599	0.00	0.00	0.00	0.00	0.00	0.00	0.00	0.00	0.00	0.00	0.00	0.00
glycerol kinase	LOC100764600	100764600	0.00	0.00	0.00	0.00	0.00	0.00	0.00	0.00	0.00	0.00	0.00	0.00
olfactory receptor 4C45-like	LOC100764601	100764601	0.00	0.00	0.00	0.00	0.00	0.00	0.00	0.00	0.00	0.00	0.00	0.00
acyl-protein thioesterase 1 pseudogene	LOC100764608	100764608	0.41	0.14	0.42	0.22	0.33	0.36	3.80	2.75	2.48	1.16	3.10	0.93
NADH dehydrogenase [ubiquinone] 1 beta subcomplex subunit 4 pseudogene	LOC100764614	100764614	0.00	0.00	0.00	0.00	0.00	0.06	0.76	0.00	0.00	0.00	0.00	0.00
guanine nucleotide-binding protein G(I)/G(S)/G(O) subunit gamma-5 pseudogene	LOC100764615	100764615	0.00	0.00	0.00	0.00	0.00	0.00	1.52	1.83	1.24	0.00	0.00	0.00
triosephosphate isomerase pseudogene	LOC100764617	100764617	0.08	0.20	0.00	0.00	0.12	0.00	0.76	1.83	0.00	0.00	1.03	0.00
saccharopine dehydrogenase-like oxidoreductase	LOC100764621	100764621	0.39	0.39	0.58	0.33	0.16	0.20	12.91	10.98	17.39	10.43	5.17	5.61
OX-2 membrane glycoprotein-like	LOC100764624	100764624	0.00	0.00	0.00	0.00	0.00	0.00	0.00	0.00	0.00	0.00	0.00	0.00

UDP-N-acetylglucosamine transporter pseudogene	LOC100 764626	100764626	0.02	0.00	0.00	0.04	0.07	0.06	0.76	0.00	0.00	1.16	2.07	2.80
protein FAM76A	LOC100 764627	100764627	18.76	19.80	22.25	23.10	26.57	28.22	789.16	876.60	945.23	1052.30	1162.29	1208.53
spermatogenesis-associated protein 31D1-like	LOC100 764630	100764630	0.00	0.00	0.00	0.00	0.00	0.00	0.00	0.00	0.00	0.00	0.00	0.00
U6 snRNA-associated Sm-like protein LSm2 pseudogene	LOC100 764632	100764632	0.00	0.00	0.00	0.00	0.00	0.00	0.00	0.00	0.00	0.00	0.00	0.00
60S ribosomal protein L36 pseudogene	LOC100 764635	100764635	0.00	0.00	0.00	0.00	0.00	0.25	0.00	0.00	0.00	0.00	0.00	0.93
eukaryotic translation initiation factor 1	LOC100 764637	100764637	9.70	11.22	6.53	4.06	5.76	4.38	34.18	33.86	24.84	11.59	18.63	16.82
chromosome unknown open reading frame, human C17orf96	LOC100 764639	100764639	0.49	0.60	0.54	0.33	0.36	0.39	9.87	13.73	13.66	6.95	9.31	6.54
biorientation of chromosomes in cell division protein 1 pseudogene	LOC100 764641	100764641	0.00	0.00	0.00	0.00	0.00	0.00	0.00	0.00	0.00	0.00	0.00	0.00
60S ribosomal protein L17 pseudogene	LOC100 764647	100764647	0.00	0.00	0.00	0.00	0.00	0.00	0.00	0.00	0.00	0.00	0.00	0.00
NF-kappa-B inhibitor alpha pseudogene	LOC100 764650	100764650	3.28	3.62	3.18	1.72	2.21	1.71	100.26	114.38	95.64	52.15	67.27	50.47
mas-related G-protein coupled receptor member B4-like	LOC100 764651	100764651	0.00	0.00	0.00	0.00	0.00	0.00	0.00	0.00	0.00	0.00	0.00	0.00
interferon-induced GTP-binding protein Mx1-like	LOC100 764653	100764653	0.45	0.34	0.56	0.37	0.22	0.36	26.58	17.39	29.81	22.02	14.49	17.76
putative PRAME family member 24	LOC100 764657	100764657	19.79	19.47	21.03	19.99	19.15	19.16	1490.22	1490.59	1603.53	1506.60	1440.70	1464.63
archaemetzincin-2-like	LOC100 764659	100764659	0.00	0.00	0.00	0.00	0.04	0.00	0.00	0.00	0.00	0.00	1.03	0.00
nuclear transport factor 2 pseudogene	LOC100 764660	100764660	0.25	0.46	1.70	0.42	0.72	0.49	1.52	2.75	9.94	2.32	3.10	2.80
uncharacterized protein C9orf114 homolog pseudogene	LOC100 764662	100764662	0.13	0.19	0.00	0.04	0.04	0.03	3.80	5.49	0.00	1.16	1.03	0.93
olfactory receptor 10AG1-like	LOC100 764665	100764665	0.00	0.00	0.00	0.00	0.00	0.00	0.00	0.00	0.00	0.00	0.00	0.00
40S ribosomal protein S10 pseudogene	LOC100 764666	100764666	0.00	0.00	0.00	0.00	0.00	0.00	0.00	0.00	0.00	0.00	0.00	0.00

death domain-associated protein 6 pseudogene	LOC100764667	100764667	1.58	1.28	1.48	1.00	0.78	0.92	184.57	198.56	177.62	147.18	91.08	106.55
tudor and KH domain-containing protein pseudogene	LOC100764670	100764670	0.00	0.00	0.00	0.00	0.00	0.00	0.00	0.00	0.00	0.00	0.00	0.00
vasculin-like protein 1 pseudogene	LOC100764674	100764674	0.00	0.00	0.00	0.00	0.03	0.00	0.00	0.92	0.00	0.00	1.03	0.00
forkhead box protein K2-like	LOC100764677	100764677	0.82	0.81	0.30	0.22	0.42	0.12	42.53	48.50	16.15	12.75	21.73	6.54
gap junction alpha-1 protein-like	LOC100764683	100764683	2.11	2.00	0.73	0.42	0.60	0.78	18.23	15.56	3.73	4.64	2.07	4.67
ATP synthase subunit g, mitochondrial pseudogene	LOC100764687	100764687	1.76	1.97	2.11	1.35	2.02	2.10	16.71	15.56	18.63	11.59	15.52	19.63
high mobility group protein B1 pseudogene	LOC100764688	100764688	1.09	1.17	1.59	1.08	0.85	0.82	3.04	4.58	11.18	5.79	7.24	1.87
melanoma-associated antigen G1	LOC100764696	100764696	25.34	26.16	27.42	25.17	22.15	23.11	1227.42	1265.49	1337.73	1216.87	1065.00	1122.54
60S ribosomal protein L6 pseudogene	LOC100764697	100764697	12.54	11.05	8.75	7.53	9.77	7.17	226.34	208.63	165.20	137.91	186.30	132.72
olfactory receptor 1P1-like	LOC100764700	100764700	0.00	0.02	0.00	0.03	0.05	0.02	0.00	0.00	0.00	0.00	0.00	0.00
olfactory receptor 1S1-like	LOC100764701	100764701	0.00	0.00	0.00	0.00	0.00	0.00	0.00	0.00	0.00	0.00	0.00	0.00
vomeronasal type-2 receptor 116-like	LOC100764702	100764702	0.00	0.00	0.00	0.00	0.00	0.00	0.00	0.00	0.00	0.00	0.00	0.00
chromosome unknown open reading frame, human C9orf64	LOC100764703	100764703	24.28	24.75	25.13	26.25	28.88	26.86	1315.52	1353.33	1386.17	1428.95	1563.86	1487.99
40S ribosomal protein S27-like	LOC100764707	100764707	1.63	1.63	2.89	1.75	2.21	0.98	4.56	5.49	8.69	3.48	4.14	3.74
olfactory receptor 52D1	LOC100764715	100764715	0.00	0.00	0.00	0.00	0.00	0.00	0.00	0.00	0.00	0.00	0.00	0.00
olfactory receptor 13C3-like	LOC100764719	100764719	0.00	0.00	0.00	0.00	0.00	0.00	0.00	0.92	0.00	0.00	0.00	0.00
olfactory receptor 18-like	LOC100764722	100764722	0.00	0.00	0.00	0.00	0.00	0.00	0.00	0.00	0.00	0.00	0.00	0.00
ribosome biogenesis regulatory protein homolog	LOC100764724	100764724	2.40	2.02	1.74	0.21	0.19	0.22	30.38	35.69	26.08	2.32	1.03	2.80

putative germ cell-less protein-like 1-like	LOC100764734	100764734	0.00	0.00	0.00	0.00	0.00	0.00	0.00	0.00	0.00	0.00	0.00	0.00
cell division cycle-associated 7-like protein pseudogene	LOC100764738	100764738	0.00	0.00	0.00	0.00	0.00	0.00	0.00	0.00	0.00	0.00	0.00	0.00
uncharacterized protein C9orf40 homolog	LOC100764740	100764740	15.35	16.51	18.60	18.61	21.05	18.99	972.21	1045.88	1194.89	1185.58	1329.96	1214.14
SAP domain-containing ribonucleoprotein-like	LOC100764744	100764744	1.06	0.45	0.10	0.83	0.35	0.16	12.91	5.49	1.24	9.27	4.14	0.93
zinc finger protein 426-like	LOC100764753	100764753	53.01	51.97	60.32	62.29	59.43	61.72	1353.50	1356.99	1532.74	1537.89	1476.93	1507.62
Ig kappa chain V-III region MOPC 321-like	LOC100764764	100764764	0.00	0.00	0.00	0.00	0.00	0.00	0.00	0.00	0.00	0.00	0.00	0.00
olfactory receptor 10	LOC100764765	100764765	0.00	0.00	0.00	0.00	0.00	0.00	0.00	0.00	0.00	0.00	0.00	0.00
15 kDa protein B-like	LOC100764766	100764766	0.00	0.00	0.00	0.00	0.00	0.00	0.00	0.00	0.00	1.16	0.00	0.00
cytochrome P450 2J6	LOC100764768	100764768	0.07	0.07	0.05	0.04	0.00	0.03	0.76	1.83	1.24	1.16	0.00	0.93
putative olfactory receptor 2B8	LOC100764770	100764770	0.03	0.04	0.00	0.00	0.00	0.00	1.52	1.83	1.24	0.00	1.03	0.00
protein orai-3 pseudogene	LOC100764773	100764773	0.00	0.00	0.00	0.00	0.00	0.00	0.00	0.00	0.00	0.00	0.00	0.00
melanoma-associated antigen H1	LOC100764776	100764776	16.05	14.40	14.35	12.80	12.36	12.23	698.78	630.46	623.53	549.33	534.05	531.83
nucleophosmin pseudogene	LOC100764778	100764778	0.00	0.00	0.00	0.00	0.00	0.00	0.00	0.00	0.00	0.00	0.00	0.00
chromosome unknown open reading frame, human C16orf90	LOC100764781	100764781	0.52	0.31	0.45	0.28	0.00	0.27	16.71	8.24	16.15	8.11	1.03	8.41
olfactory receptor 7G1-like	LOC100764784	100764784	0.00	0.00	0.00	0.00	0.00	0.00	0.00	0.00	0.00	0.00	0.00	0.00
protein FAM84A	LOC100764785	100764785	0.00	0.00	0.00	0.00	0.00	0.00	0.00	0.00	0.00	0.00	0.00	0.00
B9 domain-containing protein 1-like	LOC100764790	100764790	0.04	0.08	0.00	0.05	0.00	0.04	2.28	1.83	0.00	3.48	2.07	0.93
deoxycytidylate deaminase pseudogene	LOC100764791	100764791	0.00	0.00	0.00	0.00	0.00	0.00	0.00	0.00	0.00	0.00	0.00	0.00

histone H1.0-like	LOC100 764795	100764795	1.51	1.31	0.98	0.59	0.90	0.61	22.03	19.22	14.91	8.11	12.42	8.41
heat shock protein HSP 90-beta-like	LOC100 764796	100764796	0.00	0.00	0.00	0.00	0.00	0.00	0.00	0.00	0.00	0.00	0.00	0.00
uncharacterized LOC100764798	LOC100 764798	100764798	38.15	43.88	48.86	53.84	52.77	43.86	511.17	552.68	611.11	635.09	655.15	562.67
cyclin-G1 pseudogene	LOC100 764803	100764803	0.00	0.00	0.00	0.00	0.00	0.00	0.00	0.00	0.00	0.00	0.00	0.00
peptidyl-prolyl cis-trans isomerase A-like	LOC100 764805	100764805	2.41	1.08	1.54	0.82	0.74	1.81	4.56	6.41	4.97	3.48	3.10	4.67
sperm motility kinase 2B-like	LOC100 764806	100764806	0.01	0.02	0.02	0.00	0.00	0.00	0.00	0.92	1.24	0.00	0.00	0.00
membrane-spanning 4-domains subfamily A member 4A-like	LOC100 764807	100764807	0.00	0.00	0.00	0.00	0.00	0.00	0.00	0.00	0.00	0.00	0.00	0.00
proliferating cell nuclear antigen pseudogene	LOC100 764808	100764808	0.00	0.00	0.00	0.00	0.00	0.00	0.00	0.00	0.00	0.00	0.00	0.00
ral guanine nucleotide dissociation stimulator-like	LOC100 764810	100764810	0.00	0.00	0.00	0.00	0.00	0.00	0.00	0.00	0.00	0.00	0.00	0.00
ubiquitin-like	LOC100 764813	100764813	29.75	27.86	20.61	17.11	18.92	16.05	196.72	154.64	116.76	91.55	101.43	96.27
putative claudin-25	LOC100 764814	100764814	0.26	0.32	0.54	0.41	0.36	0.53	6.08	9.15	12.42	10.43	9.31	12.15
butyrophilin-like protein 1	LOC100 764815	100764815	0.00	0.00	0.00	0.00	0.00	0.00	0.00	0.00	0.00	0.00	0.00	0.00
uncharacterized LOC100764817	LOC100 764817	100764817	0.00	0.00	0.00	0.00	0.00	0.00	0.00	0.92	0.00	0.00	0.00	0.00
keratin, type II cytoskeletal 2 oral-like	LOC100 764819	100764819	0.00	0.00	0.00	0.00	0.00	0.00	0.00	0.00	0.00	0.00	0.00	0.00
eukaryotic translation initiation factor 5 pseudogene	LOC100 764821	100764821	0.00	0.00	0.00	0.00	0.00	0.00	0.00	0.00	0.00	0.00	0.00	0.00
NUT family member Gm806-like	LOC100 764822	100764822	0.00	0.00	0.00	0.00	0.00	0.00	0.00	0.00	0.00	0.00	0.00	0.00
bcl-2-associated transcription factor 1-like	LOC100 764824	100764824	11.49	11.69	10.59	12.05	12.43	11.67	168.62	170.20	135.39	151.82	152.14	145.81
olfactory receptor 4F4-like	LOC100 764828	100764828	0.00	0.00	0.00	0.00	0.00	0.00	0.00	0.00	0.00	0.00	0.00	0.00

cytochrome c oxidase subunit 7C, mitochondrial pseudogene	LOC100764831	100764831	0.00	0.00	0.00	0.00	0.00	0.00	0.00	0.00	0.00	0.00	0.00	0.00
zinc finger and BTB domain-containing protein 34	LOC100764833	100764833	4.59	4.64	8.18	8.28	6.60	6.54	0.00	0.00	0.00	0.00	0.00	0.00
squalene monooxygenase	LOC100764834	100764834	43.85	44.33	48.88	43.59	34.09	32.78	1676.30	1674.51	1861.89	1737.22	1416.90	1304.80
olfactory receptor 481-like	LOC100764836	100764836	0.00	0.00	0.00	0.00	0.00	0.00	0.00	0.00	0.00	0.00	0.00	0.00
forkhead box protein J1-like	LOC100764846	100764846	0.00	0.00	0.00	0.00	0.00	0.00	0.00	0.00	0.00	0.00	0.00	0.00
GTPase IMAP family member 1-like	LOC100764847	100764847	0.00	0.00	0.00	0.00	0.00	0.00	0.00	0.00	0.00	0.00	0.00	0.00
serine protease inhibitor A3N	LOC100764848	100764848	0.00	0.00	0.00	0.00	0.00	0.00	0.00	0.00	0.00	0.00	0.00	0.00
60S ribosomal protein L9 pseudogene	LOC100764849	100764849	0.00	0.00	0.00	0.00	0.00	0.00	0.00	0.00	0.00	0.00	0.00	0.00
peptidyl-prolyl cis-trans isomerase FKBP3 pseudogene	LOC100764855	100764855	0.23	0.35	0.48	0.21	0.25	0.00	6.84	2.75	7.45	3.48	2.07	0.93
uncharacterized LOC100764856	LOC100764856	100764856	0.07	0.19	0.00	0.00	0.00	0.00	2.28	5.49	0.00	0.00	0.00	0.00
putative vomeronasal receptor-like protein 4	LOC100764863	100764863	0.00	0.00	0.00	0.00	0.00	0.00	0.00	0.00	0.00	0.00	0.00	0.00
chromosome unknown open reading frame, human C5orf28	LOC100764864	100764864	2.05	1.70	1.91	1.85	2.46	1.84	72.92	62.22	72.04	66.06	87.97	67.30
cytochrome P450 2G1	LOC100764865	100764865	0.00	0.00	0.00	0.00	0.00	0.00	0.00	0.00	0.00	0.00	0.00	0.00
cytochrome c oxidase subunit 7B, mitochondrial	LOC100764866	100764866	231.38	230.69	178.92	181.80	188.16	178.64	2242.92	2207.06	1701.66	1668.84	1751.20	1668.39
putative olfactory receptor 4A4	LOC100764867	100764867	0.00	0.00	0.00	0.00	0.00	0.00	0.00	0.00	0.00	0.00	0.00	0.00
putative olfactory receptor 5AK3	LOC100764870	100764870	0.02	0.09	0.00	0.00	0.13	0.00	2.28	3.66	1.24	0.00	5.17	0.00
uncharacterized LOC100764871	LOC100764871	100764871	2.40	2.08	2.48	2.18	2.06	2.10	96.46	82.35	96.88	86.92	78.66	82.25
uncharacterized LOC100764872	LOC100764872	100764872	0.00	0.00	0.00	0.00	0.00	0.00	0.00	0.00	0.00	0.00	0.00	0.00

platelet-activating factor acetylhydrolase IB subunit beta pseudogene	LOC100764881	100764881	18.15	17.62	14.74	19.02	17.50	19.19	293.18	286.41	245.93	305.95	280.48	301.90
alpha-1-antiproteinase-like	LOC100764882	100764882	0.00	0.00	0.00	0.00	0.00	0.00	0.00	0.00	0.00	0.00	0.00	0.00
ubiquitin-conjugating enzyme E2 J2-like	LOC100764886	100764886	0.51	0.49	0.71	0.45	0.60	0.33	3.04	4.58	0.00	0.00	1.03	0.93
guanylate-binding protein 6-like	LOC100764887	100764887	0.00	0.00	0.00	0.00	0.00	0.00	0.00	0.00	0.00	0.00	0.00	0.00
taste receptor cell protein 1	LOC100764892	100764892	0.07	0.10	0.06	0.13	0.06	0.21	16.71	17.39	11.18	26.66	21.73	39.26
olfactory receptor 51G2	LOC100764894	100764894	0.00	0.00	0.00	0.00	0.00	0.00	0.00	0.00	0.00	0.00	0.00	0.00
zinc finger protein 474	LOC100764895	100764895	0.00	0.00	0.00	0.00	0.00	0.00	0.00	0.00	0.00	0.00	0.00	0.00
olfactory receptor 4C45-like	LOC100764897	100764897	0.00	0.00	0.00	0.00	0.00	0.00	0.00	0.00	0.00	0.00	0.00	0.00
T-cell leukemia/lymphoma protein 1A	LOC100764901	100764901	0.00	0.00	0.00	0.00	0.00	0.00	0.00	0.00	0.00	0.00	0.00	0.00
olfactory receptor 2H2	LOC100764903	100764903	0.00	0.03	0.00	0.00	0.00	0.00	0.00	0.92	0.00	0.00	0.00	0.00
hemoglobin subunit beta-1	LOC100764904	100764904	0.00	0.00	0.00	0.00	0.00	0.00	0.00	0.00	0.00	0.00	0.00	0.00
G2/mitotic-specific cyclin-B1 pseudogene	LOC100764909	100764909	0.10	0.29	0.04	0.00	0.07	0.06	3.04	9.15	1.24	0.00	2.07	1.87
histone H2A-Bbd type 2/3	LOC100764913	100764913	0.00	0.00	0.00	0.00	0.00	0.00	0.00	0.00	0.00	0.00	0.00	0.00
polyadenylate-binding protein 1 pseudogene	LOC100764917	100764917	0.15	0.09	0.25	0.00	0.11	0.19	1.52	0.92	2.48	0.00	1.03	1.87
OX-2 membrane glycoprotein	LOC100764921	100764921	0.00	0.00	0.00	0.00	0.00	0.00	0.00	0.00	0.00	0.00	0.00	0.00
uncharacterized protein C1orf43 homolog	LOC100764923	100764923	0.22	0.07	0.00	0.00	0.08	0.21	7.60	4.58	9.94	6.95	3.10	5.61
glycerol-3-phosphate dehydrogenase 1-like protein pseudogene	LOC100764929	100764929	0.00	0.00	0.00	0.00	0.00	0.00	0.00	0.00	0.00	0.00	0.00	0.00
tRNA (guanine(26)-N(2))-dimethyltransferase pseudogene	LOC100764934	100764934	0.28	0.46	0.23	0.29	0.19	0.15	12.91	24.71	13.66	18.54	10.35	10.28

actin-like protein 6A pseudogene	LOC100 764938	100764938	0.00	0.00	0.00	0.00	0.00	0.00	0.00	0.00	0.00	0.00	0.00	0.00
uracil-DNA glycosylase-like	LOC100 764939	100764939	0.05	0.07	0.08	0.08	0.04	0.07	3.80	7.32	4.97	4.64	4.14	6.54
mas-related G-protein coupled receptor member X1-like	LOC100 764948	100764948	0.00	0.00	0.00	0.00	0.00	0.00	0.00	0.00	0.00	0.00	0.00	0.00
GON-4-like protein	LOC100 764952	100764952	8.75	9.42	9.95	11.23	12.19	11.19	1094.50	1213.33	1291.77	1490.37	1607.33	1481.45
UPF0687 protein C20orf27 homolog pseudogene	LOC100 764960	100764960	0.00	0.00	0.00	0.00	0.00	0.00	0.00	0.00	0.00	0.00	0.00	0.00
casein kinase II subunit alpha pseudogene	LOC100 764962	100764962	0.39	0.50	0.20	0.49	0.20	0.36	12.15	15.56	7.45	15.07	5.17	12.15
olfactory receptor 10AG1-like	LOC100 764963	100764963	0.00	0.00	0.00	0.00	0.00	0.00	0.00	0.00	0.00	0.00	0.00	0.00
trafficking protein particle complex subunit 13-like	LOC100 764973	100764973	2.19	2.16	1.55	2.11	2.32	1.57	34.18	35.69	37.26	35.93	39.33	34.58
SCY1-like protein 2	LOC100 764975	100764975	9.09	8.54	8.20	10.20	9.55	9.61	231.66	215.03	238.48	267.71	231.84	244.88
60S ribosomal protein L8-like	LOC100 764977	100764977	0.00	0.00	0.00	0.00	0.00	0.00	0.00	0.00	0.00	0.00	0.00	0.00
mediator of RNA polymerase II transcription subunit 28 pseudogene	LOC100 764979	100764979	0.00	0.09	0.12	0.00	0.00	0.00	0.00	0.92	1.24	0.00	0.00	0.00
alpha-enolase pseudogene	LOC100 764980	100764980	0.00	0.00	0.00	0.00	0.00	0.00	0.00	0.00	0.00	0.00	0.00	0.00
cyclin-dependent kinase inhibitor 2A, isoforms 1/2/3-like	LOC100 764983	100764983	67.48	61.21	68.84	38.29	23.80	25.34	1386.92	1287.45	1408.53	770.68	497.83	510.33
olfactory receptor 1468	LOC100 764985	100764985	0.00	0.00	0.00	0.00	0.00	0.00	0.00	0.00	0.00	0.00	0.00	0.00
band 4.1-like protein 5 pseudogene	LOC100 764986	100764986	0.00	0.00	0.00	0.13	0.00	0.00	0.00	0.00	0.00	1.16	0.00	0.00
60S ribosomal protein L36a-like	LOC100 764989	100764989	8.83	9.53	7.76	5.61	8.62	7.19	1.52	0.92	0.00	1.16	4.14	0.00
sorting nexin-4 pseudogene	LOC100 764992	100764992	0.00	0.00	0.00	0.00	0.00	0.00	0.00	0.00	0.00	0.00	0.00	0.00
olfactory receptor 1A1	LOC100 764996	100764996	0.03	0.02	0.00	0.00	0.00	0.00	0.00	0.00	0.00	0.00	0.00	0.00

olfactory receptor 9Q2	LOC100764997	100764997	0.00	0.00	0.00	0.00	0.00	0.00	0.00	0.00	0.00	0.00	0.00	0.00
formyl peptide receptor-related sequence 3	LOC100764999	100764999	0.00	0.00	0.00	0.00	0.00	0.00	0.00	0.00	0.00	0.00	0.00	0.00
NUT family member Gm806-like	LOC100765002	100765002	0.00	0.00	0.00	0.00	0.00	0.00	0.00	0.00	0.00	0.00	0.00	0.00
elongation factor 1-gamma pseudogene	LOC100765007	100765007	30.83	31.52	32.09	20.37	19.76	19.84	576.49	618.56	580.05	381.28	433.66	409.39
olfactomedin-4	LOC100765008	100765008	0.10	0.05	0.06	0.03	0.13	0.05	3.80	1.83	1.24	3.48	6.21	2.80
olfactory receptor 13C8	LOC100765012	100765012	0.00	0.00	0.00	0.00	0.00	0.00	0.00	0.00	0.00	0.00	0.00	0.00
olfactory receptor 7E24	LOC100765015	100765015	0.00	0.00	0.00	0.00	0.00	0.00	0.00	0.00	0.00	0.00	0.00	0.00
melanin-concentrating hormone receptor 1	LOC100765019	100765019	0.00	0.00	0.00	0.00	0.00	0.00	0.00	0.00	0.00	0.00	0.00	0.00
pepsin F-like	LOC100765022	100765022	0.00	0.00	0.00	0.05	0.00	0.00	0.00	0.00	0.00	1.16	0.00	0.00
keratin, type II cuticular Hb5	LOC100765023	100765023	0.00	0.00	0.00	0.00	0.00	0.00	0.00	0.00	0.00	0.00	0.00	0.00
chromosome unknown open reading frame, human C16orf82	LOC100765025	100765025	0.00	0.00	0.00	0.00	0.00	0.00	0.00	0.00	0.00	0.00	0.00	0.00
uncharacterized protein C7orf73	LOC100765027	100765027	0.00	0.00	0.00	0.00	0.00	0.00	0.00	0.00	0.00	0.00	0.00	0.00
serine/arginine-rich splicing factor 3 pseudogene	LOC100765028	100765028	0.39	0.12	0.00	0.05	0.49	0.08	12.15	5.49	2.48	2.32	11.38	2.80
chromosome unknown open reading frame, human C9orf41	LOC100765030	100765030	9.37	9.48	10.67	12.06	12.57	11.65	309.13	335.82	357.72	398.67	403.64	396.30
C-type lectin domain family 2 member E-like	LOC100765032	100765032	0.00	0.00	0.00	0.00	0.00	0.00	0.00	0.00	0.00	0.00	0.00	0.00
up-regulated during skeletal muscle growth protein 5 pseudogene	LOC100765038	100765038	0.54	0.32	0.00	0.00	0.00	0.00	4.56	2.75	2.48	2.32	1.03	0.00
zinc finger protein OZF-like	LOC100765042	100765042	2.61	2.07	3.14	3.00	3.09	3.01	111.65	93.33	130.42	128.64	126.27	128.05
heat shock cognate 71 kDa protein pseudogene	LOC100765043	100765043	0.00	0.00	0.00	0.00	0.00	0.00	0.00	0.00	0.00	0.00	0.00	0.00

olfactory receptor 10	LOC100 765053	100765053	0.00	0.00	0.00	0.00	0.00	0.00	0.00	0.00	0.00	0.00	0.00	0.00
cytochrome P450 2J3	LOC100 765057	100765057	4.72	4.78	4.72	5.34	6.02	5.89	556.74	569.15	541.55	633.93	698.62	684.18
OTU domain-containing protein 6B pseudogene	LOC100 765058	100765058	0.04	0.00	0.06	0.00	0.00	0.00	0.76	0.00	1.24	0.00	0.00	0.00
olfactory receptor 1F12	LOC100 765059	100765059	0.00	0.00	0.00	0.00	0.00	0.00	0.00	0.00	0.00	0.00	0.00	0.00
histone H3.1-like	LOC100 765062	100765062	0.00	0.00	0.00	0.00	0.00	0.00	0.00	0.00	0.00	0.00	0.00	0.00
zinc finger protein OZF	LOC100 765065	100765065	1.14	1.11	1.12	1.43	1.23	1.29	144.31	136.34	131.66	173.84	149.04	153.29
alpha-soluble NSF attachment protein	LOC100 765071	100765071	31.57	33.56	35.61	36.13	31.58	31.58	308.37	324.84	353.99	302.48	257.71	272.92
olfactory receptor 7G2-like	LOC100 765072	100765072	0.00	0.00	0.00	0.00	0.00	0.00	0.00	0.00	0.00	0.00	0.00	0.00
carcinoembryonic antigen-related cell adhesion molecule 21-like	LOC100 765079	100765079	0.00	0.00	0.00	0.00	0.00	0.00	0.00	0.00	0.00	0.00	0.00	0.00
liver carboxylesterase-like	LOC100 765081	100765081	0.00	0.00	0.00	0.00	0.00	0.00	0.00	0.00	0.00	0.00	0.00	0.00
zinc finger protein 14-like	LOC100 765088	100765088	4.12	4.02	4.97	6.15	5.14	5.06	227.10	223.27	281.95	341.88	274.27	275.73
60S ribosomal protein L23-like	LOC100 765092	100765092	1.82	2.14	1.63	1.70	1.38	1.26	10.63	15.56	11.18	13.91	13.45	6.54
NADH-cytochrome b5 reductase 2	LOC100 765093	100765093	0.00	0.00	0.00	0.00	0.00	0.00	0.00	0.00	0.00	0.00	0.00	0.00
olfactory receptor 148	LOC100 765094	100765094	0.00	0.00	0.00	0.00	0.00	0.00	0.00	0.00	0.00	0.00	0.00	0.00
chromosome unknown open reading frame, human C7orf60	LOC100 765096	100765096	10.41	10.47	15.92	16.91	21.14	19.14	944.87	949.80	1454.48	1544.84	1896.09	1744.09
lithocholate 6-beta-hydroxylase-like	LOC100 765098	100765098	0.03	0.02	0.06	0.08	0.07	0.15	10.63	7.32	6.21	18.54	14.49	18.69
general transcription factor 3C polypeptide 3 pseudogene	LOC100 765101	100765101	0.00	0.00	0.00	0.00	0.00	0.00	0.00	0.00	0.00	0.00	0.00	0.00
non-histone chromosomal protein HMG-17	LOC100 765102	100765102	1.67	2.37	1.41	1.34	1.48	1.70	6.84	10.07	4.97	8.11	5.17	4.67

transcription elongation factor 1 homolog	LOC100 765103	100765103	2.30	0.40	0.57	1.18	2.41	0.46	5.32	1.83	1.24	2.32	5.17	0.93
Ig heavy chain V-III region VH26-like	LOC100 765105	100765105	0.00	0.00	0.00	0.00	0.00	0.00						
cell division cycle 5-like protein pseudogene	LOC100 765107	100765107	0.11	0.09	0.02	0.00	0.01	0.08	4.56	4.58	1.24	0.00	0.00	4.67
ribosomal RNA-processing protein 7 homolog A pseudogene	LOC100 765114	100765114	0.16	0.26	0.24	0.26	0.29	0.26	3.80	6.41	6.21	4.64	6.21	5.61
E3 ubiquitin-protein ligase RNF216 pseudogene	LOC100 765117	100765117	0.00	0.00	0.00	0.00	0.00	0.00	0.00	0.00	0.00	0.00	0.00	0.93
microtubule-associated protein tau	LOC100 765120	100765120	3.63	4.52	1.77	1.51	0.94	0.57	55.45	67.71	24.84	24.34	12.42	12.15
serine/arginine-rich splicing factor 3 pseudogene	LOC100 765121	100765121	57.69	57.49	56.77	54.62	59.69	55.78	1801.63	1786.15	1776.18	1645.67	1634.24	1528.18
60S ribosomal protein L5 pseudogene	LOC100 765123	100765123	3.88	5.33	3.35	2.39	4.01	1.77	66.08	90.59	52.17	39.40	62.10	28.97
transcription elongation factor B polypeptide 1 pseudogene	LOC100 765126	100765126	0.40	0.69	0.44	0.57	0.25	0.11	18.99	20.13	22.36	15.07	15.52	12.15
DCN1-like protein 2	LOC100 765127	100765127	0.00	0.00	0.00	0.00	0.00	0.00	0.00	0.00	0.00	1.16	1.03	0.00
POC1 centriolar protein homolog A-like	LOC100 765128	100765128	12.67	12.47	16.32	14.85	14.24	13.71	389.64	387.97	500.56	447.34	442.97	420.60
glyceraldehyde-3-phosphate dehydrogenase pseudogene	LOC100 765130	100765130	0.51	0.39	0.19	0.36	0.26	0.38	108.61	84.18	69.56	27.81	66.24	39.26
voltage-dependent anion-selective channel protein 1 pseudogene	LOC100 765132	100765132	0.19	0.28	0.16	0.02	0.11	0.19	3.04	5.49	2.48	0.00	1.03	3.74
chymotrypsin-like elastase family member 3B	LOC100 765144	100765144	0.00	0.00	0.00	0.00	0.00	0.00	0.00	0.92	0.00	0.00	0.00	0.00
putative vomeronasal receptor-like protein 4	LOC100 765150	100765150	0.00	0.00	0.00	0.00	0.00	0.00	0.00	0.00	0.00	0.00	0.00	0.00
cytochrome P450 2B1-like	LOC100 765152	100765152	0.00	0.00	0.00	0.00	0.00	0.00	0.00	0.00	0.00	0.00	0.00	0.00
olfactory receptor 4C12-like	LOC100 765153	100765153	0.00	0.00	0.00	0.00	0.00	0.00	0.00	0.00	0.00	0.00	0.00	0.00
olfactory receptor 8K3-like	LOC100 765155	100765155	0.00	0.00	0.00	0.00	0.00	0.00	0.00	0.00	0.00	0.00	0.00	0.00

paired mesoderm homeobox protein 1-like	LOC100765166	100765166	0.00	0.00	0.00	0.00	0.00	0.00	0.00	0.92	0.00	0.00	0.00	0.00
alpha-1-antiproteinase-like	LOC100765168	100765168	0.00	0.00	0.00	0.00	0.00	0.00	0.00	0.00	0.00	0.00	0.00	0.00
replication protein A 32 kDa subunit pseudogene	LOC100765171	100765171	0.00	0.00	0.00	0.00	0.00	0.00	0.00	0.00	0.00	0.00	0.00	0.00
carbonic anhydrase-related protein	LOC100765172	100765172	0.00	0.00	0.00	0.00	0.00	0.00	0.00	0.00	0.00	0.00	0.00	0.00
UDP-glucuronosyltransferase 2B4-like	LOC100765175	100765175	0.00	0.00	0.00	0.00	0.00	0.00	0.00	0.00	0.00	0.00	0.00	0.00
phospholipase A2-like	LOC100765177	100765177	0.00	0.00	0.00	0.00	0.00	0.00	0.00	0.00	0.00	0.00	0.00	0.00
chromosome unknown open reading frame, human C15orf39	LOC100765178	100765178	14.64	14.35	14.10	10.57	6.94	7.60	1557.81	1523.53	1524.04	1160.08	750.37	852.42
olfactory receptor 51G2-like	LOC100765180	100765180	0.00	0.00	0.00	0.00	0.00	0.00	0.00	0.00	0.00	0.00	0.00	0.00
olfactory receptor 4C5-like	LOC100765183	100765183	0.00	0.00	0.00	0.00	0.00	0.00	0.00	0.00	0.00	0.00	0.00	0.00
histone deacetylase 8	LOC100765184	100765184	9.30	9.30	11.15	10.55	13.30	11.55	160.26	171.11	198.73	184.27	207.00	219.65
60S ribosomal protein L4-like	LOC100765186	100765186	7.82	9.12	9.28	6.90	7.80	5.96	189.13	236.99	224.82	157.61	195.61	143.00
histone H3.3-like	LOC100765188	100765188	0.44	0.10	0.61	0.46	0.20	0.40	3.04	0.92	4.97	2.32	1.03	0.93
SH3 domain-binding protein 5-like pseudogene	LOC100765190	100765190	1.16	1.11	1.06	1.50	1.36	1.13	35.70	32.94	36.02	48.67	42.43	37.39
olfactory receptor 52A5-like	LOC100765191	100765191	0.00	0.00	0.00	0.00	0.00	0.00	0.00	0.00	0.00	0.00	0.00	0.00
HERV-K_6q14.1 provirus ancestral Gag-Pol polyprotein-like	LOC100765194	100765194	0.73	0.69	0.64	0.51	0.59	0.55	49.37	43.92	111.79	84.60	74.52	84.12
60S ribosomal protein L26-like	LOC100765195	100765195	0.00	0.00	0.00	0.00	0.00	0.00	0.00	0.00	0.00	0.00	0.00	0.00
40S ribosomal protein S10 pseudogene	LOC100765205	100765205	0.00	0.00	0.00	0.00	0.00	0.00	0.76	0.00	0.00	1.16	0.00	0.00
DET1- and DDB1-associated protein 1	LOC100765207	100765207	1.48	3.28	0.74	1.61	1.50	1.16	3.04	10.98	1.24	5.79	6.21	4.67

uncharacterized LOC100765209	LOC100765209	100765209	28.88	28.49	31.49	33.63	33.93	38.91	431.42	439.22	566.39	596.84	563.03	615.01
cathepsin L1-like	LOC100765210	100765210	0.00	0.00	0.00	0.00	0.00	0.00	0.00	0.00	0.00	0.00	0.00	0.00
60S ribosomal protein L39 pseudogene	LOC100765211	100765211	104.70	158.58	125.48	115.97	140.95	95.01	76.71	122.61	86.95	103.14	97.29	81.32
40S ribosomal protein SA pseudogene	LOC100765212	100765212	0.81	0.49	0.38	0.65	0.46	1.12	18.23	10.98	8.69	13.91	10.35	25.24
triggering receptor expressed on myeloid cells 1-like	LOC100765213	100765213	0.00	0.00	0.00	0.00	0.00	0.00	0.00	0.00	0.00	0.00	0.00	0.00
olfactory receptor 10A3-like	LOC100765215	100765215	0.00	0.00	0.00	0.00	0.00	0.00	0.00	0.00	0.00	0.00	0.00	0.00
solute carrier family 25 member 51	LOC100765224	100765224	18.56	18.66	17.48	18.15	20.97	20.50	477.75	482.22	455.85	475.16	528.88	529.02
60S ribosomal protein L38 pseudogene	LOC100765230	100765230	1.51	0.92	0.37	0.56	0.47	0.58	3.80	2.75	1.24	2.32	1.03	1.87
transcription factor BTF3 pseudogene	LOC100765233	100765233	0.95	1.18	0.55	0.69	0.56	0.21	9.11	13.73	6.21	8.11	5.17	0.93
synaptotagmin-11 pseudogene	LOC100765238	100765238	0.98	0.65	0.57	0.70	0.99	0.68	10.63	10.07	4.97	10.43	14.49	14.02
40S ribosomal protein S18-like	LOC100765240	100765240	0.00	0.00	0.00	0.00	0.00	0.00	0.00	0.00	0.00	0.00	0.00	0.00
wee1-like protein kinase 2 pseudogene	LOC100765243	100765243	0.00	0.00	0.00	0.00	0.00	0.00	0.00	0.00	0.00	0.00	0.00	0.00
chromosome unknown open reading frame, human C6orf165	LOC100765244	100765244	0.05	0.04	0.02	0.02	0.00	0.00	3.04	2.75	1.24	1.16	0.00	0.00
antigen-presenting glycoprotein CD1d1-like	LOC100765246	100765246	2.16	2.32	1.98	2.10	1.55	1.81	48.61	48.50	36.02	42.88	37.26	34.58
polypeptide N-acetylgalactosaminyltransferase 4	LOC100765247	100765247	2.10	2.37	2.14	2.02	2.33	2.55	205.84	230.59	209.91	194.70	226.66	251.43
olfactory receptor 10AG1-like	LOC100765250	100765250	0.00	0.00	0.00	0.00	0.00	0.00	0.00	0.00	0.00	0.00	0.00	0.00
coiled-coil-helix-coiled-coil-helix domain-containing protein 2, mitochondrial pseudogene	LOC100765254	100765254	0.19	0.64	0.00	0.00	0.40	0.12	1.52	5.49	0.00	0.00	3.10	0.93
60S ribosomal protein L4 pseudogene	LOC100765256	100765256	0.55	0.57	0.58	0.46	0.69	0.41	15.95	19.22	19.87	15.07	20.70	13.09

melanoma-associated antigen E1	LOC100765259	100765259	3.52	3.80	4.43	3.94	4.92	5.12	224.06	241.57	283.20	250.33	310.50	325.27
spermine synthase pseudogene	LOC100765261	100765261	0.63	0.46	0.91	0.54	0.34	0.23	13.67	15.56	23.60	11.59	6.21	3.74
EGF-like and EMI domain-containing protein 1	LOC100765263	100765263	0.00	0.00	0.00	0.00	0.00	0.00	0.76	0.92	0.00	0.00	0.00	0.00
protein BEX2	LOC100765265	100765265	0.06	0.00	0.00	0.29	0.25	0.08	0.00	0.00	0.00	2.32	1.03	0.00
nuclear receptor-binding factor 2-like	LOC100765266	100765266	0.04	0.03	0.06	0.05	0.10	0.03	0.00	0.00	1.24	0.00	1.03	0.00
interferon alpha-9	LOC100765269	100765269	0.00	0.00	0.00	0.00	0.00	0.00	0.00	0.00	0.00	0.00	0.00	0.00
olfactory receptor 1468	LOC100765271	100765271	0.00	0.00	0.00	0.00	0.00	0.00	0.00	0.00	0.00	0.00	0.00	0.00
NADH dehydrogenase [ubiquinone] 1 alpha subcomplex subunit 5 pseudogene	LOC100765275	100765275	0.00	0.00	0.00	0.00	0.00	0.00	0.00	0.00	0.00	0.00	0.00	0.00
olfactory receptor 1468-like	LOC100765282	100765282	0.00	0.00	0.00	0.00	0.00	0.00	0.00	0.00	0.00	0.00	0.00	0.00
olfactory receptor 9Q1	LOC100765283	100765283	0.00	0.00	0.00	0.00	0.00	0.00	0.00	0.00	0.00	0.00	0.00	0.00
protection of telomeres protein 1-like	LOC100765285	100765285	8.27	8.34	7.69	8.64	10.30	8.83	828.66	825.36	808.60	880.78	1031.88	904.76
zinc finger protein 431-like	LOC100765287	100765287	1.80	1.47	2.22	2.25	2.34	1.96	151.15	127.19	183.83	187.75	190.44	170.11
zinc finger protein 883-like	LOC100765289	100765289	2.72	2.46	4.03	3.95	3.86	4.09	166.34	149.15	255.87	239.90	226.66	246.75
oligosaccharyltransferase complex subunit OSTC	LOC100765294	100765294	0.07	0.09	0.18	0.06	0.20	0.09	0.76	0.92	1.24	0.00	2.07	0.93
chromosome unknown open reading frame, human C6orf58	LOC100765295	100765295	0.00	0.00	0.00	0.00	0.00	0.00	0.00	0.00	0.00	0.00	0.00	0.00
testin-2-like	LOC100765299	100765299	15.63	16.62	7.18	10.68	27.10	25.60	628.14	665.23	291.89	411.42	1072.25	1007.57
B-cell receptor-associated protein 31 pseudogene	LOC100765300	100765300	0.30	0.31	0.11	0.29	0.22	0.26	7.60	6.41	7.45	9.27	8.28	11.22
olfactory receptor 13C8-like	LOC100765301	100765301	0.00	0.00	0.00	0.00	0.00	0.00	0.00	0.00	0.00	0.00	0.00	0.00

olfactory receptor 18-like	LOC100765303	100765303	0.00	0.00	0.00	0.00	0.00	0.00	0.00	0.00	0.00	0.00	0.00	0.00
chromosome unknown open reading frame, human C1orf131	LOC100765311	100765311	30.23	33.63	37.06	41.62	37.84	38.10	1036.77	1163.01	1275.62	1391.86	1274.07	1281.43
transcriptional adapter 3 pseudogene	LOC100765315	100765315	3.35	3.29	3.03	2.76	2.64	2.72	47.09	43.92	37.26	42.88	37.26	33.65
A-kinase anchor protein 9-like	LOC100765321	100765321	0.00	0.02	0.00	0.00	0.05	0.04	0.00	0.00	1.24	0.00	2.07	1.87
40S ribosomal protein S2 pseudogene	LOC100765322	100765322	0.05	0.00	0.09	0.08	0.07	0.00	2.28	0.00	2.48	4.64	2.07	0.93
60 kDa heat shock protein, mitochondrial pseudogene	LOC100765326	100765326	0.09	0.08	0.16	0.07	0.10	0.12	0.00	0.00	0.00	0.00	0.00	0.00
zinc finger protein 120-like	LOC100765327	100765327	0.58	0.74	0.53	0.51	0.09	0.73	7.60	8.24	4.97	9.27	1.03	4.67
chromosome unknown open reading frame, human C15orf41	LOC100765329	100765329	14.81	13.68	14.72	17.09	16.72	16.85	985.12	950.72	999.88	1148.49	1138.48	1117.86
40S ribosomal protein S27-like pseudogene	LOC100765331	100765331	0.95	0.46	1.30	0.32	0.82	1.65	5.32	1.83	4.97	1.16	3.10	5.61
PRAME family member 12 pseudogene	LOC100765332	100765332	0.00	0.00	0.00	0.00	0.00	0.00	0.00	0.00	0.00	0.00	0.00	0.00
zinc finger protein 709-like	LOC100765341	100765341	0.00	0.00	0.00	0.02	0.00	0.00	0.76	0.00	0.00	1.16	0.00	0.00
olfactory receptor 10	LOC100765345	100765345	0.00	0.00	0.00	0.00	0.00	0.00	0.00	0.00	0.00	0.00	0.00	0.00
15 kDa protein B-like	LOC100765346	100765346	0.15	0.27	0.00	0.00	0.00	0.00	3.04	3.66	0.00	0.00	0.00	1.87
39S ribosomal protein L18, mitochondrial pseudogene	LOC100765347	100765347	0.18	0.17	0.47	0.00	0.30	0.49	2.28	1.83	4.97	0.00	3.10	5.61
RNA-binding protein EWS-like	LOC100765349	100765349	0.01	0.00	0.00	0.00	0.00	0.00	0.76	0.00	0.00	0.00	0.00	0.00
zinc finger protein with KRAB and SCAN domains 3	LOC100765350	100765350	8.98	8.96	10.30	11.07	11.05	11.27	688.14	700.00	789.97	828.63	844.55	862.70
LIM domain-containing protein ajuba	LOC100765353	100765353	15.12	15.19	7.87	6.08	4.41	5.87	412.43	427.32	197.49	147.18	105.57	106.55
cysteine-rich protein 2 pseudogene	LOC100765355	100765355	0.00	0.06	0.00	0.00	0.07	0.00	0.00	0.92	0.00	0.00	0.00	0.00

chromosome unknown open reading frame, human C16orf46	LOC100765361	100765361	3.68	4.10	3.09	3.01	2.43	2.48	251.41	285.49	218.61	209.76	165.60	171.98
olfactory receptor 7G2-like	LOC100765363	100765363	0.00	0.00	0.00	0.00	0.00	0.00	0.00	0.00	0.00	0.00	0.00	0.00
oligophrenin-1	LOC100765371	100765371	12.27	12.13	12.42	14.05	17.78	17.69	440.53	430.98	433.49	496.02	598.22	607.54
DNA-directed RNA polymerase III subunit RPC10	LOC100765374	100765374	29.43	28.22	25.25	26.15	33.69	34.18	1604.15	1558.30	1389.90	1437.06	1827.79	1847.84
leucine-rich repeat neuronal protein 4-like	LOC100765376	100765376	0.00	0.00	0.00	0.00	0.00	0.00	0.00	0.00	0.00	0.00	0.00	0.00
olfactory receptor 481-like	LOC100765382	100765382	0.00	0.00	0.00	0.00	0.00	0.00	0.00	0.00	0.00	0.00	0.00	0.00
putative olfactory receptor 10D4	LOC100765383	100765383	0.00	0.00	0.00	0.00	0.00	0.00	0.00	0.00	0.00	0.00	0.00	0.00
taste receptor type 2 member 9-like	LOC100765386	100765386	0.00	0.00	0.00	0.00	0.00	0.00	0.00	0.00	1.24	0.00	0.00	0.00
dynein assembly factor 3, axonemal-like	LOC100765387	100765387	0.00	0.00	0.00	0.00	0.00	0.00	0.00	0.00	0.00	0.00	0.00	0.00
uncharacterized LOC100765388	LOC100765388	100765388	0.37	0.48	0.73	0.61	0.70	0.45	16.71	25.62	39.75	32.45	35.19	25.24
COBW domain-containing protein 1	LOC100765393	100765393	28.08	27.82	25.88	28.00	29.72	31.26	668.39	646.01	555.21	609.59	725.53	708.48
Ig kappa chain V-I region HK102-like	LOC100765397	100765397	0.00	0.00	0.00	0.00	0.39	0.18						
N-lysine methyltransferase SETD8 pseudogene	LOC100765401	100765401	0.13	0.00	0.00	0.00	0.00	0.00	1.52	0.00	0.00	0.00	0.00	0.00
leukocyte immunoglobulin-like receptor subfamily B member 3	LOC100765406	100765406	0.00	0.00	0.00	0.00	0.00	0.00	0.00	0.00	0.00	0.00	0.00	0.00
GTPase ERas-like	LOC100765408	100765408	0.00	0.00	0.00	0.00	0.00	0.00	0.00	0.00	0.00	0.00	0.00	0.00
Uncharacterized protein ENSP00000382514-like	LOC100765409	100765409	0.00	0.00	0.00	0.00	0.00	0.00	0.00	0.00	0.00	0.00	0.00	0.00
elongation factor 2 pseudogene	LOC100765422	100765422	1.55	1.98	2.00	0.94	1.24	0.96	49.37	64.97	62.10	26.66	41.40	30.84
eukaryotic translation initiation factor 5A-1-like	LOC100765428	100765428	0.00	0.11	0.07	0.00	0.00	0.11	0.76	1.83	0.00	1.16	0.00	0.93

protein jagunal homolog 1 pseudogene	LOC100765429	100765429	0.11	0.07	0.09	0.00	0.00	0.00	2.28	1.83	3.73	1.16	0.00	0.00
protein FAM229B pseudogene	LOC100765432	100765432	0.00	0.00	0.00	0.00	0.00	0.00	0.00	0.00	0.00	0.00	0.00	0.00
mRNA turnover protein 4 homolog pseudogene	LOC100765435	100765435	0.17	0.15	0.23	0.12	0.11	0.09	3.04	2.75	4.97	2.32	3.10	1.87
putative vomeronasal receptor-like protein 4	LOC100765439	100765439	0.00	0.00	0.00	0.00	0.00	0.00	0.00	0.00	0.00	0.00	0.00	0.00
40S ribosomal protein S21 pseudogene	LOC100765440	100765440	0.00	0.00	0.00	0.00	0.00	0.00	0.00	0.00	0.00	0.00	0.00	0.00
cytochrome P450 2B1	LOC100765441	100765441	0.00	0.00	0.00	0.00	0.00	0.00	0.00	0.00	0.00	0.00	0.00	0.00
fibronectin type III domain containing protein 3C1	LOC100765442	100765442	0.00	0.02	0.00	0.00	0.00	0.00	2.28	2.75	0.00	0.00	0.00	0.00
olfactory receptor 4C12-like	LOC100765444	100765444	0.00	0.00	0.00	0.00	0.00	0.00	0.00	0.00	0.00	0.00	0.00	0.00
olfactory receptor 8K5-like	LOC100765446	100765446	0.00	0.00	0.00	0.00	0.00	0.00	0.00	0.00	0.00	0.00	0.00	0.00
keratin, type I cytoskeletal 18 pseudogene	LOC100765452	100765452	0.00	0.00	0.00	0.00	0.00	0.00	0.00	0.00	0.00	0.00	0.00	0.00
glutathione S-transferase theta-1	LOC100765453	100765453	0.00	0.00	0.00	0.00	0.00	0.00	1.52	0.00	0.00	0.00	0.00	0.00
rhox homeobox family member 1-like	LOC100765456	100765456	0.00	0.00	0.00	0.00	0.00	0.00	0.00	0.00	0.00	0.00	0.00	0.00
alpha-1-antiproteinase-like	LOC100765458	100765458	0.00	0.00	0.00	0.00	0.00	0.00	0.00	0.00	0.00	0.00	0.00	0.00
zinc finger CCCH domain-containing protein 15 pseudogene	LOC100765461	100765461	0.21	0.17	0.19	0.07	0.13	0.09	10.63	13.73	6.21	4.64	3.10	2.80
chromosome unknown open reading frame, human C3orf67	LOC100765462	100765462	36.11	33.00	35.80	35.25	51.11	40.73	1066.39	1114.51	987.46	1059.25	1332.03	1254.33
thyroid transcription factor 1-associated protein 26	LOC100765463	100765463	38.02	42.07	40.83	38.76	44.55	39.10	1418.82	1569.28	1560.06	1522.82	1668.40	1476.78
carboxylesterase 1C-like	LOC100765464	100765464	0.00	0.00	0.00	0.00	0.00	0.00	0.00	0.00	0.00	0.00	0.00	0.00
phospholipase A2-like	LOC100765466	100765466	0.00	0.00	0.00	0.00	0.00	0.00	0.00	0.00	0.00	0.00	0.00	0.00

NADH dehydrogenase [ubiquinone] iron-sulfur protein 3, mitochondrial pseudogene	LOC100765467	100765467	0.00	0.00	0.00	0.00	0.00	0.00	0.00	0.00	0.00	0.00	0.00	0.00
olfactory receptor 51G2	LOC100765470	100765470	0.00	0.00	0.00	0.00	0.00	0.00	0.00	0.00	0.00	0.00	0.00	0.00
olfactory receptor 140-like	LOC100765473	100765473	0.00	0.00	0.00	0.00	0.00	0.00	0.00	0.00	0.00	0.00	0.00	0.00
olfactory receptor 2G3-like	LOC100765479	100765479	0.00	0.00	0.00	0.00	0.00	0.00	0.00	0.00	0.00	0.00	0.00	0.00
olfactory receptor 52Z1	LOC100765480	100765480	0.00	0.00	0.00	0.00	0.00	0.00	0.00	0.00	0.00	0.00	0.00	0.00
metal transporter CNNM2	LOC100765482	100765482	7.66	7.28	7.49	8.01	7.82	8.14	406.35	395.29	416.10	421.85	432.62	454.25
aldehyde oxidase 1	LOC100765484	100765484	1.26	1.70	1.73	1.78	1.55	1.77	91.90	115.29	113.03	130.96	121.09	122.44
centromere protein S-like	LOC100765487	100765487	58.91	63.22	65.71	68.11	71.60	69.52	1081.58	1153.86	1242.09	1207.59	1258.54	1260.87
60S ribosomal protein L36 pseudogene	LOC100765491	100765491	66.88	67.45	63.36	19.65	9.57	9.71	279.51	290.98	250.90	69.54	36.22	37.39
thymosin beta-4 pseudogene	LOC100765498	100765498	364.65	350.95	383.81	396.93	418.85	421.47	3238.68	3403.01	3723.78	3511.53	4214.46	3652.69
nuclear factor erythroid 2-related factor 1-like	LOC100765501	100765501	0.16	0.07	0.05	0.04	0.04	0.14	3.80	1.83	1.24	1.16	1.03	3.74
chromosome unknown open reading frame, human C5orf15	LOC100765503	100765503	49.66	51.25	52.72	55.10	57.04	56.23	3076.13	3187.98	3297.74	3423.45	3506.53	3504.08
Ig kappa chain V-III region MOPC 63-like	LOC100765504	100765504	0.00	0.00	0.00	0.00	0.00	0.00						
vomeronasal type-1 receptor 4-like	LOC100765507	100765507	0.00	0.00	0.00	0.00	0.00	0.00	0.00	0.00	0.00	0.00	0.00	0.00
netrin-G2 pseudogene	LOC100765510	100765510	0.00	0.00	0.00	0.00	0.00	0.00	0.00	0.00	0.00	0.00	0.00	0.00
chromosome unknown open reading frame, human C17orf98	LOC100765515	100765515	0.00	0.09	0.12	0.00	0.00	0.00	1.52	4.58	1.24	1.16	2.07	0.00
uroplakin-3b-like protein	LOC100765519	100765519	0.00	0.00	0.00	0.00	0.00	0.00	0.00	0.00	0.00	0.00	0.00	0.00
60S ribosomal protein L11-like	LOC100765520	100765520	0.00	0.00	0.00	0.00	0.00	0.00	0.00	0.00	0.00	0.00	0.00	0.00

40S ribosomal protein S3 pseudogene	LOC100765523	100765523	0.00	0.00	0.00	0.00	0.00	0.00	0.00	0.00	0.00	0.00	0.00	0.00
60S ribosomal protein L5-like	LOC100765527	100765527	0.87	1.13	0.71	0.45	0.89	0.85	11.39	17.39	9.94	10.43	15.52	14.95
60S ribosomal protein L29 pseudogene	LOC100765528	100765528	0.00	0.00	0.00	0.00	0.00	0.00	0.00	0.00	0.00	0.00	0.00	0.00
60S ribosomal protein L32 pseudogene	LOC100765534	100765534	0.00	0.00	0.00	0.00	0.00	0.00	0.00	0.00	0.00	0.00	0.00	0.00
C-terminal-binding protein 2 pseudogene	LOC100765536	100765536	0.00	0.00	0.00	0.00	0.00	0.00	0.00	0.00	0.00	0.00	0.00	0.00
olfactory receptor 10AG1	LOC100765541	100765541	0.00	0.00	0.00	0.00	0.00	0.00	0.00	0.00	0.00	0.00	0.00	0.00
chromosome unknown open reading frame, human C1orf87	LOC100765542	100765542	0.18	0.15	0.05	0.00	0.00	0.00	23.55	20.13	7.45	2.32	1.03	0.00
cyclin-dependent kinase 4 pseudogene	LOC100765543	100765543	0.38	0.37	0.31	0.00	0.05	0.21	8.35	7.32	7.45	0.00	1.03	4.67
ribosome biogenesis protein NSA2 homolog pseudogene	LOC100765545	100765545	0.00	0.00	0.00	0.00	0.00	0.00	0.00	0.00	0.00	0.00	0.00	0.00
phosphoglycerate kinase 1 pseudogene	LOC100765548	100765548	20.00	20.98	19.81	18.24	20.88	19.16	260.52	269.02	249.66	177.31	229.77	207.50
trafficking protein particle complex subunit 13-like	LOC100765552	100765552	1.52	1.33	0.85	1.52	0.93	1.11	15.19	20.13	12.42	20.86	18.63	16.82
60S ribosomal protein L31 pseudogene	LOC100765554	100765554	0.00	0.00	0.00	0.00	0.15	0.14	0.00	0.00	0.00	0.00	1.03	0.93
olfactory receptor 1468	LOC100765565	100765565	0.00	0.00	0.00	0.00	0.00	0.00	0.00	0.00	0.00	0.00	0.00	0.00
protein inscuteable homolog	LOC100765569	100765569	0.04	0.00	0.00	0.00	0.00	0.00	0.76	0.00	0.00	0.00	0.00	0.00
liver carboxylesterase-like	LOC100765571	100765571	0.00	0.00	0.00	0.00	0.00	0.00	0.00	0.00	0.00	0.00	0.00	0.00
frizzled-3-like	LOC100765572	100765572	0.24	0.46	0.22	0.29	0.29	0.20	2.28	2.75	0.00	0.00	0.00	0.93
GDP-Man:Man(3)GlcNAc(2)-PP-Dol alpha-1,2-mannosyltransferase-like	LOC100765573	100765573	0.06	0.04	0.11	0.03	0.02	0.01	3.04	0.92	3.73	1.16	1.03	0.00
chromosome unknown open reading frame, human C2orf61	LOC100765575	100765575	0.00	0.00	0.00	0.00	0.00	0.00	0.76	0.00	0.00	0.00	1.03	0.00

olfactory receptor 1468-like	LOC100 765576	100765576	0.00	0.00	0.00	0.00	0.00	0.00	0.00	0.00	0.00	0.00	0.00	0.00
probable tRNA pseudouridine synthase 1 pseudogene	LOC100 765577	100765577	0.33	0.29	0.42	0.13	0.08	0.16	8.35	6.41	11.18	3.48	2.07	3.74
EGF-like module-containing mucin-like hormone receptor-like 4	LOC100 765579	100765579	0.00	0.02	0.00	0.00	0.00	0.00	0.00	0.92	0.00	1.16	0.00	0.00
ubiquitin-40S ribosomal protein S27a pseudogene	LOC100 765583	100765583	14.34	17.07	12.72	7.62	15.89	8.08	125.32	148.24	109.30	63.74	140.76	72.90
uncharacterized LOC100765584	LOC100 765584	100765584	0.00	0.00	0.00	0.00	0.00	0.00	0.76	0.00	0.00	0.00	0.00	0.00
40S ribosomal protein S25	LOC100 765587	100765587	3.28	3.93	2.48	2.71	2.75	2.47	25.06	28.37	18.63	20.86	20.70	26.17
cytochrome b-c1 complex subunit 6, mitochondrial	LOC100 765588	100765588	0.00	0.00	0.00	0.00	0.00	0.00	0.00	0.00	0.00	0.00	0.00	0.00
forkhead box protein B2	LOC100 765591	100765591	0.00	0.00	0.00	0.00	0.00	0.00	0.00	0.00	0.00	0.00	0.00	0.00
cathepsin L1-like	LOC100 765592	100765592	0.00	0.00	0.00	0.00	0.04	0.00	1.52	1.83	0.00	0.00	1.03	0.00
olfactory receptor 13C8-like	LOC100 765594	100765594	0.00	0.00	0.00	0.00	0.00	0.00	0.00	0.00	0.00	0.00	0.00	0.00
C-type lectin domain family 18 member A-like	LOC100 765595	100765595	0.04	0.02	0.00	0.00	0.03	0.02	2.28	1.83	0.00	1.16	4.14	3.74
olfactory receptor 7E24-like	LOC100 765597	100765597	0.00	0.00	0.00	0.00	0.00	0.00	0.00	0.00	0.00	0.00	0.00	0.00
tubulin alpha-3 chain	LOC100 765599	100765599	0.51	0.36	0.14	0.22	0.17	0.06	16.71	11.90	3.73	8.11	5.17	2.80
elongation factor Tu, mitochondrial-like	LOC100 765606	100765606	0.10	0.07	0.14	0.03	0.21	0.16	7.60	2.75	7.45	2.32	11.38	8.41
eukaryotic translation initiation factor 1A, X-chromosomal-like	LOC100 765607	100765607	0.00	0.04	0.12	0.00	0.00	0.05	0.76	0.00	1.24	1.16	1.03	0.93
nucleophosmin pseudogene	LOC100 765609	100765609	0.24	0.48	0.20	0.13	0.29	0.44	6.84	4.58	6.21	4.64	8.28	8.41
olfactory receptor 2AG1	LOC100 765613	100765613	0.00	0.00	0.00	0.00	0.00	0.00	0.00	0.00	0.00	0.00	0.00	0.00
Ig lambda chain V-VI region WLT-like	LOC100 765616	100765616	0.00	0.00	0.00	0.00	0.00	0.00						

zinc finger protein OZF-like	LOC100765623	100765623	14.88	14.83	16.48	16.99	20.09	18.61	1182.60	1207.84	1343.94	1369.84	1619.75	1481.45
rhox homeobox family member 2-like	LOC100765625	100765625	0.00	0.00	0.00	0.00	0.00	0.00	0.00	0.00	0.00	0.00	0.00	0.00
transcription elongation factor A protein 1 pseudogene	LOC100765626	100765626	30.65	31.73	39.96	40.02	40.35	37.78	664.60	662.48	822.26	791.54	780.38	732.78
nucleoside diphosphate kinase B	LOC100765627	100765627	972.94	973.56	454.36	411.15	369.58	391.22	15616.89	15913.35	7319.62	6398.40	5816.62	6056.66
olfactory receptor 2G3-like	LOC100765633	100765633	0.00	0.00	0.00	0.00	0.00	0.00	0.00	0.00	0.00	0.00	0.00	0.00
prolyl-tRNA synthetase associated domain-containing protein 1	LOC100765635	100765635	22.94	22.72	23.76	22.06	23.64	24.66	305.33	299.22	311.76	273.51	301.18	320.59
olfactory receptor 2Y1-like	LOC100765636	100765636	0.00	0.00	0.00	0.00	0.00	0.00	0.00	0.00	0.00	0.00	0.00	0.00
uncharacterized LOC100765638	LOC100765638	100765638	0.00	0.00	0.00	0.00	0.00	0.00	0.00	0.00	0.00	0.00	0.00	0.00
uncharacterized LOC100765640	LOC100765640	100765640	0.00	0.00	0.00	0.00	0.00	0.00	0.00	0.00	0.00	0.00	0.00	0.00
zinc finger protein with KRAB and SCAN domains 8	LOC100765641	100765641	8.00	7.99	9.90	10.57	10.61	9.88	501.30	495.03	594.96	665.22	683.09	621.56
chromosome unknown open reading frame, human C14orf93	LOC100765644	100765644	2.01	2.12	1.67	1.56	1.86	1.88	156.47	163.79	125.45	125.16	145.93	145.81
ribosomal RNA-processing protein 7 homolog A pseudogene	LOC100765649	100765649	0.00	0.00	0.00	0.00	0.00	0.00	0.00	0.00	0.00	0.00	0.00	0.00
butyrophilin subfamily 1 member A1-like	LOC100765650	100765650	0.00	0.00	0.00	0.00	0.00	0.00	0.00	0.00	0.00	0.00	0.00	0.00
60S acidic ribosomal protein P0 pseudogene	LOC100765654	100765654	70.13	66.55	62.07	52.25	46.35	49.19	2586.23	2429.41	2279.23	1401.13	1084.67	1088.89
triosephosphate isomerase pseudogene	LOC100765655	100765655	1.00	1.58	1.68	0.89	1.30	0.68	15.19	20.13	23.60	12.75	18.63	11.22
chromosome unknown open reading frame, human C1orf233	LOC100765659	100765659	3.70	3.33	5.22	4.05	4.11	5.28	231.66	206.80	325.43	250.33	251.50	330.87
protein FAM24A	LOC100765661	100765661	0.00	0.00	0.00	0.00	0.00	0.00	0.76	0.00	0.00	0.00	0.00	0.00
heat shock cognate protein HSP 90-beta pseudogene	LOC100765670	100765670	1.71	1.32	0.81	0.59	0.90	0.92	76.71	53.07	28.57	20.86	27.94	39.26

olfactory receptor 469	LOC100765672	100765672	0.00	0.00	0.00	0.00	0.00	0.00	0.00	0.00	0.00	0.00	0.00	0.00
putative olfactory receptor 10D4	LOC100765674	100765674	0.00	0.00	0.00	0.00	0.00	0.00	0.00	0.00	0.00	0.00	0.00	0.00
complement factor H-like	LOC100765684	100765684	0.03	0.03	0.02	0.02	0.00	0.00	0.76	2.75	0.00	2.32	2.07	0.00
transcriptional enhancer factor TEF-4 pseudogene	LOC100765685	100765685	0.00	0.00	0.00	0.00	0.00	0.00	0.00	0.00	0.00	0.00	0.00	0.00
importin subunit alpha-5	LOC100765686	100765686	22.25	22.06	21.23	25.33	25.42	25.46	1297.29	1267.32	1304.19	1493.85	1487.28	1504.82
Ig kappa chain V-I region HK102-like	LOC100765688	100765688	0.00	0.00	0.00	0.00	0.00	0.00						
RNA-binding protein PNO1 pseudogene	LOC100765689	100765689	0.61	0.45	0.29	0.24	0.25	0.35	16.71	11.90	8.69	6.95	6.21	13.09
26S proteasome non-ATPase regulatory subunit 10 pseudogene	LOC100765690	100765690	0.28	0.22	0.00	0.00	0.13	0.12	2.28	1.83	1.24	0.00	1.03	2.80
40S ribosomal protein S8 pseudogene	LOC100765692	100765692	48.86	48.70	45.02	34.48	36.16	32.31	193.68	164.71	162.71	117.05	134.55	123.38
60S ribosomal protein L29 pseudogene	LOC100765693	100765693	250.35	281.18	257.16	153.92	88.14	78.61	200.52	184.84	147.81	89.24	101.43	88.79
putative tripartite motif-containing protein 43C	LOC100765698	100765698	0.00	0.00	0.00	0.00	0.00	0.05	0.00	0.00	0.00	0.00	0.00	0.93
uncharacterized LOC100765709	LOC100765709	100765709	4.69	4.95	4.23	4.78	5.34	4.91	794.48	790.59	709.23	878.46	854.90	884.20
tubulin alpha-1C chain-like	LOC100765713	100765713	0.00	0.00	0.00	0.00	0.03	0.00	0.00	0.00	0.00	0.00	1.03	0.00
60S ribosomal protein L23a pseudogene	LOC100765714	100765714	25.14	24.56	19.89	14.30	17.68	13.87	37.98	57.65	53.41	39.40	33.12	17.76
pre-mRNA branch site protein p14-like	LOC100765717	100765717	1.13	1.60	0.63	0.41	0.89	0.49	6.84	9.15	3.73	2.32	4.14	2.80
opioid growth factor receptor-like	LOC100765718	100765718	16.26	16.55	15.14	13.31	9.53	10.85	605.35	585.62	541.55	494.86	349.83	422.47
poly(A) RNA polymerase, mitochondrial-like	LOC100765719	100765719	4.41	4.17	4.26	4.82	4.52	4.05	105.58	99.74	88.19	86.92	72.45	73.84
40S ribosomal protein S29 pseudogene	LOC100765721	100765721	0.00	0.00	0.00	0.00	0.00	0.00	0.00	0.00	0.00	1.16	0.00	0.00

vomeronasal type-1 receptor 90-like	LOC100765728	100765728	0.00	0.00	0.00	0.00	0.00	0.00	0.00	0.00	0.00	0.00	0.00	0.00
60S ribosomal protein L27a pseudogene	LOC100765731	100765731	11.52	12.44	7.72	6.80	7.95	7.47	53.17	51.24	34.78	26.66	41.40	45.80
olfactory receptor 4A5	LOC100765732	100765732	0.00	0.00	0.00	0.00	0.00	0.00	0.00	0.00	0.00	0.00	0.00	0.00
ferritin light chain 1 pseudogene	LOC100765736	100765736	0.00	0.00	0.00	0.00	0.00	0.00	0.00	0.00	0.00	0.00	0.00	0.00
glutathione S-transferase theta-4	LOC100765742	100765742	0.00	0.00	0.00	0.00	0.00	0.00	0.00	0.00	0.00	0.00	0.00	0.00
60S ribosomal protein L27 pseudogene	LOC100765743	100765743	0.00	0.00	0.00	0.00	0.00	0.00	0.00	0.00	0.00	0.00	0.00	0.00
protein BTG1-like	LOC100765745	100765745	1.87	2.58	2.37	0.57	1.00	0.45	15.95	21.96	19.87	4.64	8.28	3.74
histone H3.3-like	LOC100765746	100765746	0.05	0.11	0.15	0.19	0.06	0.35	0.00	0.00	1.24	0.00	0.00	2.80
ring finger protein 26-like	LOC100765749	100765749	0.05	0.16	0.02	0.04	0.05	0.07	1.52	4.58	0.00	1.16	1.03	1.87
ATP synthase F(0) complex subunit C1, mitochondrial pseudogene	LOC100765750	100765750	0.30	0.61	0.00	0.16	0.14	0.00	1.52	3.66	0.00	1.16	1.03	0.00
calcium-binding and coiled-coil domain-containing protein 2 pseudogene	LOC100765752	100765752	0.00	0.05	0.00	0.07	0.06	0.00	0.00	0.92	0.00	1.16	1.03	0.00
carboxylesterase 1D	LOC100765753	100765753	0.00	0.00	0.00	0.00	0.00	0.00	0.00	0.00	0.00	0.00	0.00	0.00
phospholipase A2	LOC100765755	100765755	0.00	0.00	0.00	0.00	0.00	0.00	0.00	0.00	0.00	0.00	0.00	0.00
cytochrome c oxidase subunit 5A, mitochondrial	LOC100765757	100765757	348.66	361.57	499.05	496.11	518.50	531.46	5151.95	5337.39	7292.29	7006.83	7499.51	7527.83
olfactory receptor 51G2-like	LOC100765759	100765759	0.00	0.00	0.00	0.00	0.00	0.00	0.00	0.00	0.00	0.00	0.00	0.00
mortality factor 4-like protein 1 pseudogene	LOC100765760	100765760	1.18	1.02	0.43	0.74	0.64	0.92	28.10	27.45	11.18	19.70	14.49	27.11
olfactory receptor 4C45-like	LOC100765762	100765762	0.00	0.00	0.00	0.00	0.00	0.00	0.00	0.00	0.00	0.00	0.00	0.00
nuclear RNA export factor 2-like	LOC100765765	100765765	0.00	0.00	0.00	0.00	0.02	0.00	0.00	0.00	0.00	0.00	1.03	0.00

probable ribosome biogenesis protein RLP24 pseudogene	LOC100765767	100765767	2.32	2.86	1.79	1.32	2.38	1.32	23.55	24.71	17.39	13.91	21.73	14.02
olfactory receptor 14J1-like	LOC100765770	100765770	0.00	0.00	0.00	0.00	0.00	0.00	0.00	0.00	0.00	0.00	0.00	0.00
olfactory receptor 52A5	LOC100765771	100765771	0.00	0.00	0.00	0.00	0.00	0.00	0.00	0.00	0.00	0.00	0.00	0.00
aldehyde oxidase 1	LOC100765774	100765774	0.50	0.39	0.62	0.42	0.59	0.33	6.84	4.58	9.94	5.79	6.21	3.74
caspase-3 pseudogene	LOC100765779	100765779	0.00	0.00	0.00	0.00	0.00	0.00	0.00	0.00	0.00	0.00	0.00	0.00
DNA-directed RNA polymerase II subunit RPB7	LOC100765781	100765781	0.42	0.54	0.45	0.33	0.54	0.53	4.56	9.15	8.69	4.64	5.17	5.61
E3 ubiquitin-protein ligase TRIM9	LOC100765785	100765785	0.00	0.00	0.00	0.00	0.00	0.00	0.00	0.00	0.00	0.00	0.00	0.00
non-histone chromosomal protein HMG-17 pseudogene	LOC100765794	100765794	0.00	0.00	0.00	0.00	0.00	0.00	0.00	0.00	0.00	0.00	0.00	0.00
guanylate cyclase activator 2B	LOC100765796	100765796	0.00	0.11	0.00	0.00	0.13	0.00	0.00	0.00	0.00	0.00	0.00	0.00
spermine synthase pseudogene	LOC100765797	100765797	0.54	0.62	0.70	0.44	0.68	0.54	4.56	8.24	8.69	4.64	7.24	5.61
peptidyl-prolyl cis-trans isomerase A-like	LOC100765799	100765799	5.22	5.76	4.44	2.76	3.89	2.66	15.19	10.07	11.18	4.64	7.24	3.74
60S ribosomal protein L6 pseudogene	LOC100765801	100765801	0.00	0.00	0.00	0.00	0.00	0.00	0.00	0.00	0.00	0.00	0.00	0.00
stimulated by retinoic acid gene 6 protein homolog	LOC100765804	100765804	0.00	0.02	0.00	0.00	0.00	0.00	0.00	0.92	0.00	0.00	0.00	0.00
male-specific lethal 3 homolog	LOC100765805	100765805	14.56	14.98	8.18	7.89	7.24	7.74	917.52	949.80	524.16	505.29	460.57	494.44
granzyme B	LOC100765811	100765811	0.00	0.00	0.00	0.00	0.00	0.00	0.00	0.00	0.00	0.00	0.00	0.00
lysine-rich nucleolar protein 1-like	LOC100765816	100765816	0.03	0.00	0.05	0.05	0.00	0.08	0.76	0.00	2.48	1.16	1.03	3.74
chromosome unknown open reading frame, human C5orf51	LOC100765818	100765818	19.80	20.56	17.16	18.79	22.79	22.63	1948.98	2024.05	1751.34	1875.13	2245.92	2202.08
tropomyosin alpha-3 chain pseudogene	LOC100765821	100765821	0.11	0.00	0.18	0.00	0.20	0.00	0.76	0.00	1.24	0.00	2.07	0.00

40S ribosomal protein S9 pseudogene	LOC100765824	100765824	0.00	0.00	0.00	0.00	0.00	0.00	0.00	0.00	0.00	0.00	0.00	0.00
olfactory receptor 10AG1-like	LOC100765827	100765827	0.00	0.00	0.00	0.00	0.00	0.00	0.00	0.00	0.00	0.00	0.00	0.00
chromosome unknown open reading frame, human CXorf58	LOC100765828	100765828	0.00	0.00	0.00	0.00	0.00	0.00	0.76	0.00	0.00	0.00	0.00	0.00
H/ACA ribonucleoprotein complex subunit 4-like	LOC100765830	100765830	0.02	0.00	0.03	0.00	0.00	0.02	0.76	0.00	1.24	0.00	0.00	0.93
zinc finger CCHC domain-containing protein 3-like	LOC100765833	100765833	16.01	15.23	15.00	13.17	11.12	11.81	1381.60	1305.75	1290.53	1127.63	968.75	1032.81
melanoma-associated antigen E2	LOC100765836	100765836	0.65	0.57	1.83	1.58	2.05	1.85	33.42	29.28	93.16	78.81	100.39	92.53
uncharacterized LOC100765838	LOC100765838	100765838	3.92	3.48	3.25	2.82	4.08	3.61	200.52	180.26	180.10	155.30	210.10	203.76
60S ribosomal protein L38-like	LOC100765839	100765839	31.10	15.46	23.53	12.44	20.93	18.23	95.70	56.73	72.04	37.09	49.68	44.86
7,8-dihydro-8-oxoguanine triphosphatase pseudogene	LOC100765843	100765843	0.00	0.00	0.00	0.00	0.00	0.00	0.00	0.00	0.00	0.00	0.00	0.00
60S ribosomal protein L15-like	LOC100765844	100765844	0.34	0.31	0.28	0.09	0.24	0.21	6.84	6.41	3.73	1.16	6.21	3.74
interferon alpha-12	LOC100765849	100765849	0.00	0.00	0.00	0.00	0.00	0.00	0.00	0.00	0.00	0.00	0.00	0.00
olfactory receptor 1468-like	LOC100765851	100765851	0.00	0.00	0.00	0.00	0.00	0.00	0.00	0.00	0.00	0.00	0.00	0.00
U1 small nuclear ribonucleoprotein C pseudogene	LOC100765852	100765852	0.67	0.43	0.26	0.18	0.27	0.59	18.23	10.07	16.15	4.64	10.35	20.56
vomeronasal type-1 receptor 1-like	LOC100765853	100765853	0.00	0.00	0.00	0.00	0.00	0.00	0.00	0.00	0.00	0.00	0.00	0.00
exocrine gland-secreted peptide 22-like	LOC100765857	100765857	0.00	0.00	0.00	0.00	0.00	0.00	0.00	0.00	0.00	0.00	0.00	0.00
cysteine-rich PDZ-binding protein pseudogene	LOC100765859	100765859	0.18	0.43	0.00	0.00	0.00	0.00	0.00	1.83	0.00	0.00	1.03	0.00
olfactory receptor-like protein DTMT	LOC100765863	100765863	0.00	0.00	0.00	0.00	0.00	0.00	0.00	0.00	0.00	0.00	0.00	0.00
olfactory receptor 5B12-like	LOC100765864	100765864	0.00	0.00	0.00	0.00	0.00	0.00	0.00	0.00	0.00	0.00	0.00	0.00

vomeronasal type-2 receptor 116-like	LOC100 765866	100765866	0.00	0.00	0.00	0.00	0.00	0.00	0.00	0.00	0.00	1.16	0.00	0.00
nucleolar and coiled-body phosphoprotein 1 pseudogene	LOC100 765867	100765867	1.57	1.54	1.37	1.12	1.27	1.28	54.69	53.99	49.68	37.09	43.47	42.06
zinc finger protein 850-like	LOC100 765869	100765869	1.01	0.90	0.95	1.23	1.57	1.30	162.54	146.41	163.96	198.18	264.96	216.84
beta-actin-like protein 2	LOC100 765872	100765872	0.00	0.00	0.00	0.00	0.00	0.00	0.00	0.00	0.00	0.00	0.00	0.00
N-acetyllactosaminide alpha-1,3-galactosyltransferase-like	LOC100 765880	100765880	0.00	0.00	0.00	0.00	0.04	0.03	0.00	0.00	0.00	0.00	1.03	0.93
olfactory receptor 7E24-like	LOC100 765883	100765883	0.00	0.00	0.00	0.00	0.00	0.00	0.00	0.00	0.00	0.00	0.00	0.00
stathmin domain-containing protein 1-like	LOC100 765884	100765884	0.02	0.09	0.00	0.00	0.03	0.00	1.52	0.92	0.00	0.00	0.00	0.93
cytochrome P450 2E1	LOC100 765891	100765891	0.00	0.00	0.00	0.00	0.00	0.00	0.00	0.00	0.00	0.00	0.00	0.00
malignant fibrous histiocytoma-amplified sequence 1 homolog	LOC100 765894	100765894	0.00	0.00	0.00	0.00	0.00	0.00	0.00	0.00	0.00	0.00	0.00	0.00
serum amyloid A-3 protein	LOC100 765897	100765897	0.00	0.00	0.00	0.00	0.00	0.09	0.00	0.00	0.00	0.00	1.03	0.93
signal-regulatory protein beta-1 isoform 3-like	LOC100 765898	100765898	0.00	0.00	0.00	0.00	0.00	0.00	0.00	0.00	0.00	0.00	0.00	0.00
olfactory receptor 2AG1-like	LOC100 765900	100765900	0.00	0.00	0.00	0.00	0.00	0.00	0.00	0.00	0.00	0.00	0.00	0.00
Ig lambda chain V-VI region WLT-like	LOC100 765903	100765903	0.00	0.00	0.00	0.00	0.00	0.00						
ras-related protein Rab-1A pseudogene	LOC100 765911	100765911	0.16	0.77	0.27	0.13	0.39	0.36	1.52	7.32	2.48	1.16	3.10	2.80
homeobox protein Rhox5-like	LOC100 765912	100765912	0.00	0.00	0.00	0.00	0.00	0.00	0.00	0.00	0.00	0.00	0.00	0.00
60S ribosomal protein L7a pseudogene	LOC100 765913	100765913	4.74	4.64	4.12	3.54	4.25	2.61	60.00	56.73	65.83	42.88	55.89	45.80
vomeronasal type-2 receptor 116-like	LOC100 765915	100765915	0.00	0.00	0.00	0.00	0.00	0.00	0.00	0.00	0.00	0.00	0.00	0.00
60S ribosomal protein L31 pseudogene	LOC100 765920	100765920	17.92	14.26	8.86	6.71	11.70	7.73	139.76	110.72	67.07	50.99	87.97	57.95

putative olfactory receptor 2W6	LOC100 765921	100765921	0.00	0.00	0.00	0.00	0.00	0.00	0.00	0.00	0.00	0.00	0.00	0.00
prefoldin subunit 3-like	LOC100 765923	100765923	0.25	0.25	0.10	0.00	0.20	0.10	3.80	3.66	1.24	0.00	3.10	1.87
olfactory receptor 10	LOC100 765925	100765925	0.00	0.00	0.00	0.00	0.00	0.00	0.00	0.00	0.00	0.00	0.00	0.00
death domain-containing membrane protein NRADD	LOC100 765926	100765926	0.17	0.30	0.13	0.51	0.08	0.07	8.35	12.81	8.69	22.02	6.21	3.74
type-1 angiotensin II receptor-like	LOC100 765929	100765929	0.00	0.00	0.00	0.00	0.00	0.00	0.00	0.92	0.00	0.00	0.00	0.00
ferritin heavy chain pseudogene	LOC100 765930	100765930	0.88	0.93	0.94	0.75	0.79	0.80	12.91	16.47	13.66	16.22	12.42	14.02
60S ribosomal protein L34 pseudogene	LOC100 765937	100765937	0.00	0.00	0.00	0.00	0.00	0.00	0.00	0.00	0.00	0.00	0.00	0.00
olfactory receptor 1361	LOC100 765941	100765941	0.00	0.00	0.00	0.00	0.00	0.00	0.00	0.00	0.00	0.00	1.03	0.00
putative gustatory receptor clone PTE38	LOC100 765944	100765944	0.00	0.00	0.00	0.00	0.00	0.00	0.00	0.00	0.00	0.00	0.00	0.00
protein tyrosine phosphatase type IVA 1 pseudogene	LOC100 765945	100765945	0.48	0.47	0.55	0.68	0.53	0.60	15.95	15.56	18.63	20.86	20.70	19.63
cystatin-14	LOC100 765948	100765948	0.00	0.00	0.00	0.00	0.00	0.00	0.00	0.00	0.00	0.00	0.00	0.00
chromosome unknown open reading frame, human C10orf88	LOC100 765951	100765951	6.09	6.23	6.70	6.84	7.44	7.32	576.49	597.52	655.82	664.06	714.14	706.61
olfactory receptor 481-like	LOC100 765962	100765962	0.00	0.00	0.00	0.00	0.00	0.00	0.00	0.00	0.00	0.00	0.00	0.00
olfactory receptor 149-like	LOC100 765963	100765963	0.04	0.03	0.03	0.00	0.03	0.00	2.28	0.92	1.24	0.00	2.07	0.00
MKI67 FHA domain-interacting nucleolar phosphoprotein pseudogene	LOC100 765964	100765964	2.14	2.27	1.48	0.94	1.78	1.39	39.50	40.26	28.57	18.54	33.12	22.43
interferon-induced guanylate-binding protein 2	LOC100 765968	100765968	15.79	15.28	9.93	11.95	14.80	14.96	645.61	654.25	421.07	496.02	615.82	622.49
cytochrome P450 3A25-like	LOC100 765971	100765971	0.00	0.00	0.00	0.00	0.00	0.00	0.00	0.00	0.00	0.00	0.00	0.00
complement factor H-like	LOC100 765974	100765974	5.37	4.89	4.83	5.46	5.45	5.17	282.55	263.53	262.08	286.25	292.90	286.01

vigilin pseudogene	LOC100 765975	100765975	0.00	0.00	0.00	0.00	0.00	0.00	0.00	0.00	0.00	0.00	0.00	0.00
putative ankyrin repeat domain-containing protein ENSP00000383069	LOC100 765981	100765981	0.00	0.00	0.00	0.00	0.03	0.02	0.00	0.00	1.24	1.16	1.03	1.87
E3 ubiquitin-protein ligase RNF216 pseudogene	LOC100 765983	100765983	0.00	0.00	0.00	0.00	0.00	0.00	0.00	0.00	0.00	0.00	0.00	0.00
prohibitin pseudogene	LOC100 765984	100765984	0.48	0.68	0.61	0.19	0.40	0.16	8.35	10.98	11.18	3.48	7.24	2.80
zinc finger protein 239-like	LOC100 765987	100765987	0.00	0.00	0.05	0.00	0.00	0.00	0.00	0.00	1.24	0.00	0.00	0.00
zinc finger and BTB domain-containing protein 34	LOC100 765988	100765988	4.59	4.64	8.18	8.28	6.60	6.54	0.00	0.00	0.00	0.00	0.00	0.00
prefoldin subunit 3	LOC100 765989	100765989	0.35	0.46	0.52	0.20	0.70	0.24	4.56	6.41	8.69	2.32	10.35	2.80
60S ribosomal protein L11-like	LOC100 765990	100765990	2.07	2.12	0.81	0.00	1.13	1.88	9.87	10.07	3.73	1.16	5.17	8.41
protein transport protein Sec61 subunit gamma-like	LOC100 765998	100765998	0.00	0.00	0.00	0.00	0.00	0.00	0.00	0.00	0.00	0.00	0.00	0.00
ribosome biogenesis protein NSA2 homolog pseudogene	LOC100 765999	100765999	0.04	0.20	0.07	0.13	0.06	0.00	0.76	3.66	1.24	2.32	1.03	0.93
interferon-inducible GTPase 1	LOC100 766009	100766009	0.07	0.00	0.04	0.00	0.00	0.00	3.04	0.00	2.48	0.00	0.00	0.00
E3 ubiquitin-protein ligase RNF14 pseudogene	LOC100 766012	100766012	1.05	0.98	0.81	1.68	1.07	1.37	21.27	21.05	16.15	33.61	22.77	28.97
phosphoglycerate mutase 1 pseudogene	LOC100 766014	100766014	1.39	1.18	0.84	0.29	0.32	0.29	22.79	19.22	13.66	4.64	5.17	4.67
histone H2A.V	LOC100 766018	100766018	86.04	87.64	89.59	88.07	97.04	104.43	1579.08	1616.86	1647.01	1570.34	1694.27	1822.61
putative vomeronasal receptor-like protein 4	LOC100 766019	100766019	0.00	0.00	0.00	0.00	0.00	0.00	0.00	0.00	0.00	0.00	0.00	0.00
IgGFc-binding protein-like	LOC100 766020	100766020	0.03	0.03	0.01	0.02	0.01	0.01	1.52	1.83	0.00	0.00	1.03	1.87
olfactory receptor 4A5-like	LOC100 766022	100766022	0.00	0.00	0.00	0.00	0.00	0.00	0.00	0.00	0.00	0.00	0.00	0.00
ran-binding protein 17	LOC100 766027	100766027	2.11	2.21	1.72	2.65	3.11	2.79	132.16	140.00	115.51	168.04	179.05	171.04

nidogen-1	LOC100766031	100766031	266.50	270.07	368.72	368.64	299.65	305.16	23425.71	23962.90	32922.75	32999.08	27066.96	27526.96
cytochrome c1, heme protein, mitochondrial	LOC100766032	100766032	183.92	172.66	185.99	175.15	156.92	173.62	5609.96	5321.84	5724.78	5300.90	4786.81	5211.72
39S ribosomal protein L51, mitochondrial pseudogene	LOC100766033	100766033	0.51	0.44	0.12	0.11	0.71	0.23	6.08	4.58	1.24	1.16	7.24	2.80
glutathione S-transferase theta-2	LOC100766034	100766034	6.61	5.54	7.97	6.92	5.28	6.03	255.21	231.50	332.88	304.80	241.15	252.36
10 kDa heat shock protein, mitochondrial	LOC100766036	100766036	35.00	51.10	38.01	39.16	34.88	22.69	101.02	143.66	100.61	91.55	87.97	56.08
60S ribosomal protein L19 pseudogene	LOC100766039	100766039	0.05	0.19	0.00	0.00	0.00	0.00	1.52	2.75	0.00	0.00	0.00	0.93
pleiotropic regulator 1 pseudogene	LOC100766041	100766041	0.12	0.02	0.05	0.04	0.05	0.07	3.80	0.92	1.24	1.16	1.03	1.87
serine/threonine-protein kinase 40 pseudogene	LOC100766042	100766042	0.00	0.03	0.02	0.07	0.03	0.00	0.00	0.92	0.00	2.32	1.03	0.00
GDP-Man:Man(3)GlcNAc(2)-PP-Dol alpha-1,2-mannosyltransferase-like	LOC100766044	100766044	0.21	0.26	0.25	0.21	0.22	0.24	2.28	7.32	4.97	2.32	4.14	4.67
carboxylesterase 1E	LOC100766045	100766045	0.00	0.00	0.00	0.00	0.00	0.00	0.00	0.00	0.00	0.00	0.00	0.00
phospholipase A2-like	LOC100766047	100766047	0.00	0.00	0.00	0.00	0.00	0.00	0.00	0.00	0.00	0.00	0.00	0.00
olfactory receptor 51I1-like	LOC100766051	100766051	0.00	0.00	0.00	0.00	0.00	0.00	0.00	0.00	0.00	0.00	0.00	0.00
kinetochore-associated protein 1	LOC100766053	100766053	36.18	38.58	33.15	37.39	50.56	46.59	3810.61	3952.03	3481.57	3865.00	4971.04	4594.84
ubiquitin-conjugating enzyme E2 C-like	LOC100766056	100766056	0.00	0.00	0.00	0.00	0.00	0.00	0.00	0.00	0.00	0.00	0.00	0.00
40S ribosomal protein S11-like	LOC100766060	100766060	0.00	0.00	0.00	0.00	0.00	0.00	0.76	0.92	2.48	0.00	0.00	0.00
cathepsin L1-like	LOC100766062	100766062	0.00	0.00	0.00	0.00	0.13	0.00	2.28	0.92	0.00	0.00	4.14	0.00
olfactory receptor 14J1-like	LOC100766063	100766063	0.00	0.00	0.00	0.00	0.00	0.00	0.00	0.00	0.00	0.00	0.00	0.00
olfactory receptor 51G2	LOC100766064	100766064	0.00	0.00	0.00	0.00	0.00	0.00	0.00	0.00	0.00	0.00	0.00	0.00

3-hydroxybutyrate dehydrogenase type 2	LOC100766065	100766065	5.38	4.61	6.26	5.92	5.53	5.10	205.08	204.05	193.77	203.97	197.68	180.39
keratin, type II cytoskeletal cochleal-like	LOC100766068	100766068	0.00	0.00	0.00	0.00	0.00	0.00	0.00	0.00	0.00	0.00	0.00	0.00
glyceraldehyde-3-phosphate dehydrogenase pseudogene	LOC100766073	100766073	188.32	199.45	177.01	152.52	125.46	130.67	397.24	423.66	402.44	326.82	205.96	329.94
60S ribosomal protein L29 pseudogene	LOC100766075	100766075	1.10	2.01	1.08	1.17	1.23	0.29	9.87	14.64	11.18	8.11	10.35	1.87
alpha-enolase pseudogene	LOC100766078	100766078	0.79	0.53	0.70	0.48	0.62	0.46	16.71	9.15	13.66	8.11	9.31	10.28
uncharacterized LOC100766081	LOC100766081	100766081	0.66	0.48	0.00	0.00	0.00	0.00	5.32	4.58	0.00	0.00	0.00	0.93
TP53-regulated inhibitor of apoptosis 1 pseudogene	LOC100766085	100766085	0.19	0.00	0.00	0.00	0.00	0.00	0.76	0.00	0.00	0.00	0.00	0.00
proto-oncogene FRAT1-like	LOC100766086	100766086	0.25	0.14	0.44	0.24	0.05	0.14	5.32	1.83	8.69	6.95	1.03	2.80
histone H2A.Z	LOC100766092	100766092	86.96	88.64	68.71	36.74	26.15	25.53	1424.14	1428.37	1042.11	441.55	214.24	239.28
proton-coupled amino acid transporter 1	LOC100766093	100766093	3.21	3.35	3.20	2.90	3.28	3.38	457.24	477.65	454.60	403.30	468.85	486.96
pre-mRNA-processing factor 17 pseudogene	LOC100766098	100766098	0.00	0.00	0.00	0.00	0.00	0.00	0.00	0.00	0.00	0.00	0.00	0.00
60S ribosomal protein L13 pseudogene	LOC100766103	100766103	0.00	0.00	0.00	0.00	0.00	0.00	0.00	0.92	0.00	0.00	0.00	0.00
granzyme C-like	LOC100766105	100766105	0.00	0.00	0.00	0.00	0.00	0.00	0.00	0.00	0.00	0.00	0.00	0.00
vacuole membrane protein 1-like	LOC100766106	100766106	0.00	0.00	0.00	0.00	0.00	0.00	0.00	0.00	0.00	0.00	0.00	0.00
chromosome unknown open reading frame, human C7orf62	LOC100766109	100766109	0.00	0.00	0.00	0.00	0.00	0.00	0.00	0.00	0.00	0.00	0.00	0.00
ADP/ATP translocase 2 pseudogene	LOC100766110	100766110	0.00	0.00	0.00	0.00	0.00	0.00	0.00	0.00	0.00	0.00	0.00	0.00
glycoprotein-N-acetylgalactosamine 3-beta-galactosyltransferase 1 pseudogene	LOC100766115	100766115	0.00	0.00	0.00	0.00	0.00	0.00	0.00	0.00	0.00	0.00	0.00	0.00
eppin-like	LOC100766120	100766120	0.00	0.00	0.00	0.00	0.00	0.00	0.00	0.00	0.00	0.00	0.00	0.00

olfactory receptor 10AG1-like	LOC100766122	100766122	0.00	0.00	0.00	0.00	0.00	0.00	0.00	0.00	0.00	0.00	0.00	0.00
transcription factor SPT20 homolog pseudogene	LOC100766123	100766123	0.00	0.00	0.00	0.00	0.00	0.00	0.00	0.92	0.00	0.00	0.00	0.00
60S ribosomal protein L23a pseudogene	LOC100766124	100766124	0.00	0.00	0.00	0.00	0.00	0.00	0.00	0.00	0.00	0.00	0.00	0.00
chromosome unknown open reading frame, human C8orf34	LOC100766125	100766125	0.00	0.00	0.00	0.00	0.00	0.00	0.00	0.92	0.00	0.00	0.00	0.00
enolase-like	LOC100766129	100766129	0.10	0.22	0.27	0.10	0.11	0.02	6.08	10.98	12.42	4.64	6.21	2.80
U1 small nuclear ribonucleoprotein A pseudogene	LOC100766133	100766133	0.00	0.00	0.00	0.00	0.00	0.00	0.00	0.00	0.00	0.00	0.00	0.00
40S ribosomal protein S8 pseudogene	LOC100766136	100766136	0.85	0.82	0.48	0.37	0.49	0.63	9.87	10.07	6.21	4.64	6.21	7.48
protein BEX2-like	LOC100766137	100766137	0.06	0.00	0.00	0.10	0.26	0.08	0.00	0.00	0.00	0.00	0.00	0.00
interferon alpha-12	LOC100766144	100766144	0.00	0.00	0.00	0.00	0.00	0.00	0.00	0.00	0.00	0.00	0.00	0.00
olfactory receptor-like protein I9	LOC100766145	100766145	0.00	0.00	0.00	0.00	0.00	0.00	0.00	0.00	0.00	0.00	0.00	0.00
vomeronasal type-1 receptor 4-like	LOC100766147	100766147	0.00	0.00	0.00	0.00	0.00	0.00	0.00	0.00	0.00	0.00	0.00	0.00
hemoglobin subunit alpha-like	LOC100766152	100766152	0.00	0.00	0.00	0.00	0.00	0.00	0.00	0.00	0.00	0.00	0.00	0.00
olfactory receptor 139	LOC100766158	100766158	0.00	0.00	0.00	0.00	0.00	0.00	0.00	0.00	0.00	0.00	0.00	0.00
olfactory receptor 5B12-like	LOC100766159	100766159	0.00	0.00	0.00	0.00	0.00	0.00	0.00	0.00	0.00	0.00	0.00	0.00
lamin-B receptor-like	LOC100766162	100766162	0.00	0.02	0.03	0.03	0.00	0.00	0.00	0.92	1.24	1.16	0.00	0.00
vomeronasal type-2 receptor 116-like	LOC100766163	100766163	0.00	0.00	0.00	0.00	0.00	0.00	0.00	0.00	0.00	0.00	0.00	0.00
nucleophosmin pseudogene	LOC100766165	100766165	0.00	0.00	0.00	0.00	0.00	0.00	0.00	0.00	0.00	0.00	0.00	0.00
beta-actin-like protein 2	LOC100766167	100766167	0.00	0.00	0.00	0.00	0.00	0.00	0.00	0.00	0.00	0.00	0.00	0.00

dnaJ homolog subfamily C member 14 pseudogene	LOC100766169	100766169	0.00	0.00	0.00	0.00	0.00	0.00	0.00	0.00	0.00	0.00	0.00	0.00
ribosome biogenesis protein NSA2 homolog pseudogene	LOC100766185	100766185	0.18	0.05	0.07	0.07	0.06	0.05	3.04	0.92	1.24	1.16	1.03	0.93
olfactory receptor 13A1	LOC100766188	100766188	0.00	0.00	0.00	0.00	0.00	0.00	0.00	0.00	0.00	0.00	0.00	0.00
synphilin-1-like	LOC100766193	100766193	0.00	0.00	0.00	0.00	0.00	0.00	0.00	0.00	0.00	0.00	0.00	0.00
tripartite motif-containing protein 43B-like	LOC100766194	100766194	0.00	0.00	0.00	0.00	0.00	0.00	0.00	0.00	0.00	0.00	0.00	0.00
RNA binding motif protein, X-linked-like-1	LOC100766196	100766196	0.12	0.06	0.08	0.11	0.03	0.00	4.56	4.58	7.45	4.64	3.10	2.80
olfactory receptor 2AG1-like	LOC100766198	100766198	0.00	0.00	0.00	0.00	0.00	0.00	0.00	0.00	0.00	0.00	0.00	0.00
coiled-coil-helix-coiled-coil-helix domain-containing protein 2, mitochondrial pseudogene	LOC100766201	100766201	0.62	0.98	0.68	0.09	0.73	0.15	9.11	11.90	8.69	1.16	8.28	3.74
carcinoembryonic antigen-related cell adhesion molecule 1-like	LOC100766202	100766202	0.00	0.00	0.00	0.00	0.00	0.00	0.00	0.00	0.00	0.00	0.00	0.00
immunoglobulin-binding protein 1-like	LOC100766203	100766203	0.00	0.00	0.00	0.00	0.00	0.00	0.00	0.00	0.00	0.00	0.00	0.00
low molecular weight phosphotyrosine protein phosphatase pseudogene	LOC100766206	100766206	0.79	1.18	0.80	1.01	1.31	1.38	20.51	31.11	21.12	26.66	31.05	36.45
zinc finger protein 26	LOC100766207	100766207	3.11	3.09	2.82	3.31	3.77	3.69	167.10	154.64	145.32	166.88	178.02	184.13
zinc finger and BTB domain-containing protein 14-like	LOC100766208	100766208	0.00	0.00	0.00	0.00	0.00	0.00	0.00	0.00	0.00	0.00	0.00	0.00
protein phosphatase 1 regulatory subunit 26-like	LOC100766209	100766209	0.00	0.00	0.00	0.00	0.00	0.00	0.00	0.00	0.00	0.00	0.00	0.00
zinc finger protein 431-like	LOC100766213	100766213	11.14	10.67	11.63	13.45	12.64	11.69	1001.83	975.43	1076.89	1294.51	1184.02	1100.11
chromosome unknown open reading frame, human C19orf53	LOC100766214	100766214	84.01	79.18	82.59	67.03	57.94	60.92	2380.40	2246.41	2343.82	1884.40	1619.75	1722.60
putative olfactory receptor 2W6	LOC100766219	100766219	0.00	0.00	0.05	0.00	0.00	0.00	0.00	0.00	1.24	0.00	0.00	0.00
60S ribosomal protein L37a-like	LOC100766222	100766222	0.00	0.00	0.00	0.00	0.00	0.00	0.00	0.00	0.00	0.00	0.00	0.00

olfactory receptor 10	LOC100766223	100766223	0.00	0.00	0.00	0.00	0.00	0.00	0.00	0.00	0.00	0.00	0.00	0.00
PRAME family member 12 pseudogene	LOC100766226	100766226	0.12	0.03	0.00	0.08	0.10	0.18	3.80	1.83	0.00	2.32	3.10	6.54
ribosomal L1 domain-containing protein 1 pseudogene	LOC100766227	100766227	1.20	0.74	0.66	0.65	0.66	0.65	61.52	46.67	39.75	30.13	37.26	34.58
olfactory receptor 2B11	LOC100766228	100766228	0.00	0.00	0.00	0.00	0.00	0.00	0.00	0.00	0.00	0.00	0.00	0.00
olfactory receptor 15	LOC100766238	100766238	0.00	0.00	0.00	0.00	0.00	0.00	0.00	0.00	0.00	0.00	0.00	0.00
olfactory receptor 7G2-like	LOC100766241	100766241	0.00	0.00	0.00	0.00	0.00	0.00	0.00	0.00	0.00	0.00	0.00	0.00
chromosome unknown open reading frame, human C11orf68	LOC100766243	100766243	163.45	157.01	147.13	102.26	86.74	97.19	5834.02	5609.16	5267.69	3634.37	3080.12	3492.86
14-3-3 protein zeta/delta pseudogene	LOC100766248	100766248	0.04	0.00	0.00	0.00	0.00	0.09	0.76	0.00	0.00	0.00	0.00	1.87
liver carboxylesterase	LOC100766249	100766249	0.00	0.00	0.00	0.00	0.00	0.00	0.00	0.00	0.00	0.00	0.00	0.00
uncharacterized LOC100766251	LOC100766251	100766251	0.00	0.00	0.00	0.00	0.00	0.00	0.00	0.00	0.00	0.00	0.00	0.00
ferritin light chain-like	LOC100766254	100766254	0.00	0.00	0.00	0.00	0.00	0.00	0.00	0.00	0.00	0.00	0.00	0.00
olfactory receptor 149	LOC100766259	100766259	0.00	0.00	0.00	0.00	0.00	0.00	0.00	0.00	0.00	0.00	0.00	0.00
hypothetical protein	LOC100766264	100766264	12.65	12.01	12.03	11.55	11.78	12.06	192.92	185.75	177.62	181.95	193.54	187.87
interferon-induced guanylate-binding protein 2-like	LOC100766265	100766265	0.00	0.00	0.04	0.00	0.04	0.00	0.76	0.00	1.24	1.16	4.14	8.41
alkylated DNA repair protein alkB homolog 8 pseudogene	LOC100766267	100766267	2.82	2.81	2.82	2.74	3.41	2.56	66.84	73.20	67.07	61.42	82.80	62.62
uncharacterized LOC100766272	LOC100766272	100766272	10.69	10.98	9.39	10.89	12.21	12.20	337.99	344.05	340.33	362.74	464.71	437.43
chromosome unknown open reading frame, human C15orf26	LOC100766273	100766273	0.00	0.04	0.00	0.00	0.00	0.00	0.00	0.92	0.00	0.00	0.00	0.00
transcription elongation factor B polypeptide 1 pseudogene	LOC100766274	100766274	0.14	0.67	0.47	0.46	0.20	0.18	0.76	3.66	2.48	3.48	1.03	0.93

GTPase ERas-like	LOC100 766275	100766275	0.00	0.00	0.00	0.00	0.00	0.00	0.00	0.00	0.00	0.00	0.00	0.00
high mobility group protein B2 pseudogene	LOC100 766278	100766278	1.47	1.38	3.15	2.71	1.95	1.80	16.71	13.73	36.02	28.97	20.70	17.76
zinc finger protein 120-like	LOC100 766281	100766281	0.09	0.11	0.00	0.14	0.00	0.00	0.76	0.00	0.00	2.32	2.07	0.93
leukocyte immunoglobulin-like receptor subfamily A member 6	LOC100 766283	100766283	0.00	0.00	0.00	0.00	0.00	0.00	0.00	0.00	0.00	0.00	0.00	0.00
keratin, type I cytoskeletal 26	LOC100 766284	100766284	0.00	0.00	0.00	0.00	0.00	0.00	0.00	0.00	0.00	0.00	0.00	0.00
40S ribosomal protein S26-like	LOC100 766285	100766285	0.13	0.00	0.00	0.00	0.00	0.00	0.76	0.00	0.00	0.00	0.00	0.00
TAR DNA-binding protein 43 pseudogene	LOC100 766288	100766288	0.71	0.79	0.32	0.37	0.70	0.18	41.02	42.09	34.78	17.38	24.84	10.28
dihydrofolate reductase pseudogene	LOC100 766291	100766291	0.00	0.01	0.00	0.00	0.00	0.00	0.76	1.83	0.00	0.00	0.00	0.00
keratin-associated protein 12-1-like	LOC100 766293	100766293	0.00	0.00	0.00	0.00	0.00	0.00	0.00	0.00	0.00	0.00	0.00	0.00
60S ribosomal protein L7a pseudogene	LOC100 766309	100766309	0.00	0.00	0.00	0.00	0.06	0.00	0.76	0.00	0.00	1.16	3.10	0.00
olfactory receptor 50	LOC100 766310	100766310	0.00	0.00	0.00	0.00	0.00	0.00	0.00	0.00	0.00	0.00	0.00	0.00
vomeronasal type-1 receptor 90-like	LOC100 766314	100766314	0.00	0.00	0.00	0.00	0.00	0.00	0.00	0.00	0.00	0.00	0.00	0.00
IgGFc-binding protein-like	LOC100 766315	100766315	0.58	0.48	0.12	0.19	0.12	0.14	161.78	132.68	38.50	49.83	31.05	42.06
olfactory receptor 4A5-like	LOC100 766317	100766317	0.00	0.00	0.00	0.00	0.00	0.00	0.00	0.00	0.00	0.00	0.00	0.00
pancreatic alpha-amylase	LOC100 766318	100766318	0.03	0.00	0.02	0.00	0.00	0.00	0.76	0.00	0.00	0.00	0.00	0.00
serine protease 42-like	LOC100 766321	100766321	0.00	0.00	0.00	0.00	0.00	0.00	0.00	0.00	0.00	0.00	0.00	0.00
vitamin K-dependent protein S-like	LOC100 766322	100766322	0.01	0.03	0.00	0.02	0.04	0.00	0.76	1.83	0.00	1.16	2.07	0.00
60S ribosomal protein L39-like	LOC100 766325	100766325	0.00	0.00	0.00	0.00	0.00	0.00	0.00	0.00	0.00	0.00	0.00	0.00

phosphoglycerate kinase 1 pseudogene	LOC100766327	100766327	1.13	0.90	0.93	0.45	1.58	0.99	21.27	19.22	19.87	10.43	24.84	16.82
serine incorporator 3-like	LOC100766329	100766329	0.47	0.38	0.14	0.12	0.52	0.37	24.31	16.47	7.45	8.11	21.73	12.15
L-lactate dehydrogenase A chain pseudogene	LOC100766330	100766330	0.00	0.00	0.00	0.00	0.00	0.00	0.00	0.00	0.00	0.00	0.00	0.00
liver carboxylesterase 4-like	LOC100766339	100766339	0.00	0.00	0.00	0.00	0.00	0.00	0.00	0.00	0.00	0.00	0.00	0.00
phospholipase A2-like	LOC100766341	100766341	0.00	0.00	0.00	0.00	0.00	0.00	0.00	0.00	0.00	0.00	0.00	0.00
eukaryotic translation initiation factor 1	LOC100766344	100766344	103.65	101.39	87.73	85.95	85.64	89.28	977.53	919.61	808.60	847.17	817.64	932.80
olfactory receptor 51G2-like	LOC100766345	100766345	0.00	0.00	0.00	0.00	0.00	0.00	0.00	0.00	0.00	0.00	0.00	0.00
olfactory receptor 51B6-like	LOC100766357	100766357	0.00	0.00	0.00	0.00	0.00	0.00	0.00	0.00	0.00	0.00	0.00	0.00
signal transducer CD24 pseudogene	LOC100766359	100766359	0.00	0.00	0.00	0.00	0.00	0.00	0.00	0.00	0.00	0.00	0.00	0.00
acidic leucine-rich nuclear phosphoprotein 32 family member B pseudogene	LOC100766360	100766360	0.00	0.00	0.00	0.03	0.00	0.00	0.76	0.92	0.00	0.00	0.00	0.00
keratin, type II cytoskeletal 8-like	LOC100766361	100766361	0.00	0.00	0.00	0.00	0.00	0.00	0.00	0.00	0.00	0.00	0.00	0.00
homeobox protein TGIF2 pseudogene	LOC100766365	100766365	0.31	0.27	0.14	0.09	0.08	0.10	8.35	7.32	3.73	2.32	2.07	2.80
programmed cell death protein 6 pseudogene	LOC100766367	100766367	0.19	0.35	0.26	0.00	0.45	0.00	5.32	8.24	2.48	0.00	6.21	2.80
olfactory receptor 181	LOC100766369	100766369	0.00	0.00	0.00	0.00	0.00	0.00	0.00	0.00	0.00	0.00	0.00	0.00
ubiquitin-like protein FUBI pseudogene	LOC100766377	100766377	0.00	0.00	0.00	0.00	0.00	0.00	0.76	0.00	0.00	0.00	1.03	0.93
ubiquitin-conjugating enzyme E2 variant 1 pseudogene	LOC100766378	100766378	1.04	0.96	0.65	0.96	1.28	1.31	28.86	31.11	18.63	27.81	48.64	43.93
H/ACA ribonucleoprotein complex subunit 4 pseudogene	LOC100766379	100766379	0.07	0.05	0.11	0.00	0.00	0.03	3.04	0.92	2.48	0.00	0.00	1.87
high mobility group protein B1 pseudogene	LOC100766385	100766385	0.00	0.00	0.00	0.00	0.00	0.02	0.00	0.00	0.00	0.00	0.00	0.00

heat shock protein HSP 90-beta pseudogene	LOC100766396	100766396	23.60	25.98	23.21	16.81	21.26	18.79	987.40	1039.48	948.95	690.72	585.80	606.60
chromosome unknown open reading frame, human C5orf22	LOC100766398	100766398	5.25	5.64	6.17	6.61	6.98	7.22	609.91	661.57	731.59	763.73	823.85	841.20
granzyme G-like	LOC100766399	100766399	0.00	0.00	0.00	0.00	0.00	0.00	0.00	0.00	0.00	0.00	0.00	0.00
selenoprotein K pseudogene	LOC100766400	100766400	0.00	0.00	0.00	0.00	0.00	0.00	1.52	0.92	0.00	1.16	0.00	0.93
PWWP domain-containing protein MUM1	LOC100766402	100766402	17.34	18.13	22.01	20.92	17.66	17.76	1548.70	1586.67	1943.87	1860.07	1521.43	1574.92
caspase-3-like	LOC100766404	100766404	0.10	0.17	0.00	0.00	0.07	0.17	2.28	3.66	0.00	0.00	1.03	3.74
heterogeneous nuclear ribonucleoproteins C1/C2 pseudogene	LOC100766410	100766410	1.27	1.00	0.75	0.32	0.64	0.73	12.91	7.32	4.97	3.48	5.17	4.67
single-stranded DNA-binding protein, mitochondrial pseudogene	LOC100766411	100766411	0.09	0.24	0.15	0.07	0.18	0.00	0.76	2.75	2.48	1.16	3.10	0.00
uncharacterized protein C2orf78 homolog	LOC100766415	100766415	0.00	0.00	0.00	0.08	0.00	0.00	0.00	0.00	0.00	1.16	1.03	0.00
cleavage and polyadenylation specificity factor subunit 4 pseudogene	LOC100766416	100766416	0.10	0.06	0.08	0.00	0.14	0.00	1.52	0.92	1.24	0.00	2.07	0.00
uncharacterized LOC100766417	LOC100766417	100766417	13.81	13.59	13.68	9.36	6.08	8.13	489.90	475.82	484.41	331.45	217.35	290.68
RNA-binding protein 8A pseudogene	LOC100766423	100766423	0.45	0.18	0.25	0.30	0.21	0.43	4.56	1.83	2.48	2.32	2.07	3.74
60S ribosomal protein L10-like	LOC100766425	100766425	0.00	0.00	0.00	0.00	0.00	0.00	0.76	0.92	0.00	0.00	0.00	0.00
ADP-ribosylation factor 1 pseudogene	LOC100766427	100766427	21.28	23.14	19.68	17.02	18.08	17.05	271.92	297.39	248.42	219.04	230.80	216.84
signal peptidase complex subunit 3	LOC100766431	100766431	0.00	0.00	0.00	0.00	0.00	0.00	0.00	0.00	0.00	0.00	0.00	0.00
uncharacterized LOC100766434	LOC100766434	100766434	0.51	0.55	0.00	0.44	0.31	0.42	9.87	7.32	0.00	6.95	5.17	8.41
interferon alpha-12-like	LOC100766437	100766437	0.00	0.00	0.00	0.00	0.00	0.00	0.00	0.00	0.00	0.00	0.00	0.00
olfactory receptor 1	LOC100766438	100766438	0.00	0.00	0.00	0.00	0.00	0.00	0.00	0.00	0.00	0.00	0.00	0.00

vomeronasal type-1 receptor 4-like	LOC100766439	100766439	0.00	0.00	0.00	0.00	0.00	0.00	0.00	0.00	0.00	0.00	0.00	0.00
cytochrome P450 2K4-like	LOC100766444	100766444	0.00	0.00	0.02	0.00	0.00	0.00	0.00	0.00	0.00	0.00	0.00	0.00
V-type proton ATPase subunit D pseudogene	LOC100766447	100766447	0.77	0.28	0.58	0.36	0.52	0.23	12.15	4.58	8.69	5.79	8.28	3.74
olfactory receptor 3A1-like	LOC100766450	100766450	0.00	0.01	0.00	0.00	0.00	0.00	0.00	0.00	0.00	0.00	0.00	0.00
olfactory receptor 10Q1-like	LOC100766451	100766451	0.00	0.00	0.00	0.00	0.00	0.00	0.00	0.00	0.00	0.00	0.00	0.00
serine/threonine-protein phosphatase 1 regulatory subunit 10-like	LOC100766453	100766453	0.01	0.01	0.02	0.03	0.02	0.00	0.00	0.92	1.24	2.32	1.03	0.00
WW domain-binding protein 2 pseudogene	LOC100766454	100766454	0.00	0.00	0.00	0.00	0.00	0.00	0.00	0.00	0.00	0.00	0.00	0.00
protein FAM84A-like	LOC100766456	100766456	0.00	0.00	0.00	0.00	0.00	0.00	0.00	0.00	0.00	0.00	0.00	0.00
zinc finger protein 420-like	LOC100766457	100766457	0.00	0.02	0.00	0.00	0.00	0.00	0.00	0.92	1.24	1.16	0.00	0.93
chromosome unknown open reading frame, human C7orf26	LOC100766477	100766477	19.37	18.59	18.68	16.11	11.60	13.22	944.11	921.44	917.90	788.07	567.17	650.53
zinc finger protein 120-like	LOC100766483	100766483	0.00	0.00	0.00	0.02	0.00	0.02	0.00	0.00	0.00	1.16	0.00	0.93
protein SAAL1-like	LOC100766485	100766485	31.28	30.37	36.80	37.41	38.21	37.84	1099.05	1067.84	1312.89	1295.67	1369.29	1322.56
poly [ADP-ribose] polymerase 9-like	LOC100766487	100766487	0.21	0.12	0.18	0.18	0.26	0.26	11.39	4.58	11.18	5.79	16.56	13.09
CDC42 small effector protein 1 pseudogene	LOC100766490	100766490	0.30	0.61	0.17	0.16	0.28	0.00	6.84	9.15	6.21	3.48	3.10	1.87
transcription elongation factor A protein-like 4	LOC100766491	100766491	34.67	36.37	29.25	31.75	31.03	32.44	810.43	851.90	700.54	744.03	724.49	766.43
vomeronasal type-2 receptor 116-like	LOC100766492	100766492	0.00	0.00	0.00	0.00	0.00	0.00	0.00	0.00	0.00	0.00	0.00	0.00
H-2 class I histocompatibility antigen, alpha chain-like	LOC100766493	100766493	0.00	0.00	0.00	0.00	0.00	0.00	0.00	0.00	0.00	0.00	0.00	0.00
putative gustatory receptor clone PTE01	LOC100766497	100766497	0.00	0.00	0.00	0.00	0.00	0.00	0.00	0.00	0.00	0.00	0.00	0.00

transmembrane 9 superfamily member 2-like	LOC100766498	100766498	0.04	0.02	0.00	0.00	0.00	0.00	2.28	0.92	0.00	0.00	0.00	0.00
putative deoxyribonuclease TATDN2 pseudogene	LOC100766499	100766499	1.90	1.71	2.25	2.10	2.04	1.86	8.35	10.98	13.66	9.27	12.42	5.61
chromosome unknown open reading frame, human C19orf68	LOC100766500	100766500	6.06	5.25	6.53	5.02	5.26	4.49	344.83	304.71	376.35	283.94	296.01	256.10
vesicle transport protein USE1 pseudogene	LOC100766501	100766501	0.00	0.13	0.00	0.00	0.00	0.00	0.00	1.83	0.00	0.00	0.00	0.00
40S ribosomal protein S18 pseudogene	LOC100766503	100766503	1.32	1.74	1.26	0.45	0.90	0.36	9.87	9.15	6.21	2.32	3.10	1.87
zinc finger protein 431-like	LOC100766504	100766504	2.05	2.06	2.10	2.47	2.42	2.00	122.29	111.63	103.09	122.85	117.99	96.27
myosin phosphatase Rho-interacting protein	LOC100766510	100766510	19.61	19.35	20.17	19.98	19.42	20.22	4829.15	4863.40	5240.36	5167.62	4962.76	5207.98
olfactory receptor 10-like	LOC100766514	100766514	0.00	0.00	0.00	0.00	0.00	0.00	0.00	0.00	0.00	0.00	0.00	0.00
60S ribosomal protein L7 pseudogene	LOC100766517	100766517	1.58	1.62	1.39	0.48	1.70	0.61	28.10	32.03	28.57	8.11	32.08	12.15
60S ribosomal protein L23a pseudogene	LOC100766523	100766523	8.42	9.05	7.60	6.34	6.94	3.97	24.31	47.58	29.81	35.93	33.12	11.22
cytochrome P450 3A9	LOC100766524	100766524	0.00	0.00	0.00	0.00	0.00	0.00	0.00	0.00	0.00	0.00	0.00	0.00
histidine triad nucleotide-binding protein 1 pseudogene	LOC100766525	100766525	32.18	43.36	37.15	32.73	33.37	25.80	194.44	261.70	214.88	183.11	195.61	149.55
ubiquitin carboxyl-terminal hydrolase 27-like	LOC100766526	100766526	0.31	0.32	0.19	0.18	0.12	0.22	0.76	0.00	0.00	0.00	0.00	0.93
olfactory receptor 1361-like	LOC100766528	100766528	0.00	0.00	0.00	0.00	0.00	0.00	0.00	0.00	0.00	0.00	0.00	0.00
olfactory receptor 7G2-like	LOC100766531	100766531	0.00	0.00	0.00	0.00	0.00	0.00	0.00	0.00	0.00	0.00	0.00	0.00
39S ribosomal protein L1, mitochondrial pseudogene	LOC100766535	100766535	0.98	0.81	0.73	0.83	0.96	0.96	25.06	18.30	17.39	25.50	23.80	23.37
thymosin beta-10 pseudogene	LOC100766540	100766540	0.00	0.00	0.00	0.00	0.00	0.00	0.00	0.00	0.00	0.00	0.00	0.00
olfactory receptor 6C4-like	LOC100766542	100766542	0.00	0.00	0.00	0.00	0.00	0.00	0.00	0.00	0.00	0.00	0.00	0.00

zinc finger protein 431-like	LOC100766543	100766543	0.00	0.00	0.07	0.00	0.00	0.00	0.76	0.00	0.00	0.00	0.00	0.00
olfactory receptor 473	LOC100766547	100766547	0.00	0.00	0.00	0.00	0.00	0.00	0.00	0.00	0.00	0.00	0.00	0.00
olfactory receptor 148	LOC100766548	100766548	0.00	0.00	0.00	0.00	0.00	0.00	0.00	0.00	0.00	0.00	0.00	0.00
40S ribosomal protein S2 pseudogene	LOC100766550	100766550	0.09	0.00	0.00	0.00	0.00	0.00	1.52	0.00	0.00	0.00	0.00	0.00
guanylate-binding protein 4-like	LOC100766554	100766554	0.01	0.02	0.02	0.00	0.00	0.05	0.76	0.92	1.24	0.00	0.00	2.80
U2 small nuclear ribonucleoprotein B'' pseudogene	LOC100766555	100766555	0.56	0.51	0.26	0.49	0.26	0.35	9.87	6.41	3.73	6.95	3.10	4.67
asparagine synthetase domain-containing protein 1-like	LOC100766556	100766556	0.00	0.00	0.00	0.00	0.00	0.00	0.00	0.00	0.00	0.00	0.00	0.00
E3 ubiquitin-protein ligase MARCH5-like	LOC100766557	100766557	0.02	0.04	0.00	0.06	0.00	0.05	0.00	0.92	0.00	1.16	1.03	0.93
NAD(P) transhydrogenase, mitochondrial	LOC100766566	100766566	46.47	47.06	43.01	45.29	46.96	47.78	1882.14	2025.88	1866.86	1922.65	2119.65	2082.44
actin-related protein 2 pseudogene	LOC100766567	100766567	0.90	0.64	0.53	0.45	0.76	0.49	6.08	4.58	4.97	3.48	5.17	1.87
pre-mRNA-splicing regulator WTAP-like	LOC100766571	100766571	2.93	3.04	2.93	3.34	3.62	2.38	57.72	69.54	60.86	54.47	54.85	42.06
polypyrimidine tract-binding protein 1 pseudogene	LOC100766574	100766574	4.75	5.27	4.89	2.79	1.44	1.54	47.85	54.90	45.96	35.93	16.56	18.69
Golgi SNAP receptor complex member 2	LOC100766577	100766577	235.59	227.77	226.25	223.68	205.34	233.43	3238.68	3109.28	3085.34	2856.74	2567.80	2823.64
protein LSM14 homolog A pseudogene	LOC100766579	100766579	0.00	0.00	0.00	0.00	0.00	0.00	0.00	0.00	0.00	0.00	0.00	0.00
cystatin-C	LOC100766583	100766583	0.00	0.00	0.00	0.00	0.00	0.00	0.00	0.00	0.00	0.00	0.00	0.00
40S ribosomal protein S8-like	LOC100766588	100766588	0.04	0.00	0.00	0.00	0.00	0.00	1.52	0.00	0.00	0.00	1.03	0.00
uncharacterized LOC100766590	LOC100766590	100766590	0.62	0.11	0.59	0.43	0.87	0.23	9.11	8.24	9.94	8.11	15.52	11.22
olfactory receptor 50	LOC100766603	100766603	0.00	0.00	0.00	0.00	0.00	0.00	0.00	0.00	0.00	0.00	0.00	0.00

uncharacterized LOC100766606	LOC100766606	100766606	17.99	16.36	19.51	21.43	19.65	19.91	1940.62	1880.39	2323.94	2322.48	2084.46	1971.22
putative vomeronasal receptor-like protein 4	LOC100766607	100766607	0.00	0.00	0.00	0.00	0.00	0.00	0.00	0.00	0.00	0.00	0.00	0.00
olfactory receptor 4A5-like	LOC100766610	100766610	0.00	0.00	0.00	0.00	0.00	0.00	0.00	0.00	0.00	0.00	0.00	0.00
glyceraldehyde-3-phosphate dehydrogenase pseudogene	LOC100766616	100766616	0.67	0.82	0.50	0.59	0.96	0.27	5.32	4.58	2.48	3.48	8.28	0.93
serine/arginine-rich splicing factor 3 pseudogene	LOC100766621	100766621	0.00	0.00	0.00	0.00	0.00	0.00	0.00	0.00	0.00	0.00	0.00	0.00
ADP/ATP translocase 2 pseudogene	LOC100766622	100766622	0.26	0.16	0.03	0.05	0.07	0.17	2.28	3.66	0.00	1.16	1.03	0.93
liver carboxylesterase 1-like	LOC100766630	100766630	0.00	0.00	0.00	0.00	0.00	0.00	0.00	0.00	0.00	0.00	0.00	0.00
phospholipase A2-like	LOC100766632	100766632	0.00	0.00	0.00	0.00	0.00	0.00	0.00	0.00	0.00	0.00	0.00	0.00
olfactory receptor 52Z1	LOC100766636	100766636	0.00	0.00	0.00	0.00	0.00	0.00	0.00	0.00	0.00	0.00	0.00	0.00
putative olfactory receptor 2W6	LOC100766647	100766647	0.00	0.00	0.00	0.00	0.00	0.00	0.00	0.00	0.00	0.00	0.00	0.00
putative olfactory receptor 52P1	LOC100766648	100766648	0.00	0.00	0.00	0.00	0.00	0.00	0.00	0.00	0.00	0.00	0.00	0.00
EKC/KEOPS complex subunit LAGE3-like	LOC100766651	100766651	0.00	0.00	0.00	0.00	0.00	0.00	0.00	0.00	0.00	0.00	0.00	0.00
vomeronasal type-2 receptor 116-like	LOC100766652	100766652	0.00	0.00	0.00	0.00	0.00	0.00	0.00	0.00	0.00	0.00	0.00	0.00
voltage-dependent anion-selective channel protein 1 pseudogene	LOC100766657	100766657	0.24	0.27	0.16	0.05	0.07	0.08	12.91	15.56	6.21	2.32	2.07	5.61
myosin-4	LOC100766659	100766659	0.00	0.00	0.00	0.00	0.00	0.00	0.00	0.00	0.00	0.00	0.00	0.00
olfactory receptor 5K1-like	LOC100766661	100766661	0.00	0.00	0.00	0.00	0.00	0.00	0.00	0.00	0.00	0.00	0.00	0.00
POC1 centriolar protein homolog A	LOC100766662	100766662	9.59	9.58	11.24	9.14	8.06	8.36	266.60	257.12	310.52	246.85	213.21	232.73
putative DMBT1-like protein	LOC100766665	100766665	0.00	0.00	0.00	0.00	0.00	0.00	0.00	0.00	0.00	0.00	0.00	0.00

signal-regulatory protein beta-1 isoform 3-like	LOC100 766667	100766667	0.00	0.00	0.00	0.00	0.00	0.00	0.00	0.00	0.00	0.00	0.00	0.00
epithelial chloride channel protein-like	LOC100 766670	100766670	0.00	0.00	0.00	0.00	0.00	0.00	0.00	0.00	0.00	0.00	0.00	0.00
ADP-ribosylation factor-like protein 4D	LOC100 766671	100766671	6.18	6.11	3.82	3.18	2.08	2.55	63.80	51.24	37.26	19.70	8.28	17.76
calcineurin-like phosphoesterase domain-containing protein 1 pseudogene	LOC100 766672	100766672	0.00	0.00	0.00	0.00	0.00	0.00	0.00	0.00	0.00	0.00	0.00	0.00
chromosome unknown open reading frame, human C1orf106	LOC100 766673	100766673	0.09	0.02	0.00	0.04	0.02	0.00	5.32	2.75	0.00	2.32	1.03	0.00
chromosome unknown open reading frame, human C20orf27	LOC100 766674	100766674	17.19	16.56	18.75	15.66	12.87	13.56	605.35	593.86	665.76	548.17	451.25	500.05
anaphase-promoting complex subunit CDC26 pseudogene	LOC100 766682	100766682	0.00	0.38	0.00	0.00	0.00	0.00	0.00	2.75	1.24	0.00	0.00	0.93
40S ribosomal protein S2-like	LOC100 766689	100766689	2.13	1.12	0.76	0.70	1.11	1.65	38.74	21.96	19.87	10.43	20.70	30.84
signal peptidase complex subunit 2 pseudogene	LOC100 766695	100766695	0.09	0.22	0.15	0.14	0.12	0.17	3.04	3.66	2.48	2.32	2.07	2.80
60S ribosomal protein L23 pseudogene	LOC100 766697	100766697	0.00	0.11	0.00	0.00	0.00	0.00	1.52	2.75	1.24	1.16	0.00	0.00
WAP four-disulfide core domain protein 6A-like	LOC100 766705	100766705	0.00	0.00	0.00	0.00	0.00	0.00	0.00	0.00	0.00	0.00	0.00	0.00
olfactory receptor 5AS1-like	LOC100 766707	100766707	0.00	0.00	0.00	0.00	0.00	0.00	0.00	0.00	0.00	0.00	0.00	0.00
transcription factor SPT20 homolog pseudogene	LOC100 766708	100766708	0.00	0.00	0.00	0.00	0.00	0.00	0.00	0.00	0.00	0.00	0.00	0.00
F-actin-capping protein subunit alpha-1-like	LOC100 766712	100766712	0.06	0.00	0.19	0.06	0.08	0.09	0.76	0.00	3.73	1.16	1.03	1.87
interferon alpha-inducible protein 6-like	LOC100 766713	100766713	0.72	0.67	0.27	0.09	0.39	0.14	18.23	18.30	7.45	2.32	8.28	5.61
signal peptidase complex subunit 3	LOC100 766720	100766720	0.02	0.00	0.00	0.00	0.00	0.00	0.76	0.00	0.00	0.00	0.00	0.00
cyclin-dependent kinase 1-like	LOC100 766722	100766722	0.04	0.00	0.00	0.00	0.00	0.00	0.76	0.00	0.00	0.00	0.00	0.00
prohibitin pseudogene	LOC100 766723	100766723	0.10	0.06	0.00	0.00	0.13	0.06	6.08	1.83	1.24	0.00	2.07	1.87

interferon alpha-12	LOC100766726	100766726	0.00	0.00	0.00	0.00	0.00	0.00	0.00	0.00	0.00	0.00	0.00	0.00
olfactory receptor 1468	LOC100766727	100766727	0.00	0.00	0.00	0.00	0.00	0.00	0.00	0.00	0.00	0.00	0.00	0.00
vomeronasal type-1 receptor 4-like	LOC100766728	100766728	0.00	0.00	0.00	0.00	0.00	0.00	0.00	0.00	0.00	0.00	0.00	0.00
chromosome unknown open reading frame, human C1orf159	LOC100766731	100766731	16.03	17.15	19.53	22.10	21.58	23.94	919.04	995.56	1156.38	1277.13	1285.45	1428.18
chromosome unknown open reading frame, human C6orf141	LOC100766734	100766734	2.09	2.02	2.27	2.44	2.30	1.98	87.35	86.93	96.88	86.92	82.80	60.75
liver carboxylesterase-like	LOC100766735	100766735	0.00	0.00	0.00	0.00	0.00	0.00	0.00	0.00	0.00	0.00	0.00	0.00
olfactory receptor 1A1	LOC100766740	100766740	0.03	0.02	0.00	0.00	0.00	0.00	0.00	0.00	0.00	0.00	0.00	0.00
olfactory receptor 10W1-like	LOC100766741	100766741	0.00	0.00	0.00	0.00	0.00	0.00	0.00	0.00	0.00	0.00	0.00	0.00
proliferation-associated protein 2G4 pseudogene	LOC100766743	100766743	2.29	2.47	1.83	1.31	1.77	1.33	56.21	56.73	45.96	28.97	39.33	28.97
vomeronasal type-1 receptor 2-like	LOC100766745	100766745	0.00	0.00	0.00	0.00	0.00	0.00	0.00	0.00	0.00	0.00	0.00	0.00
vomeronasal type-2 receptor 116-like	LOC100766746	100766746	0.00	0.00	0.00	0.00	0.00	0.00	0.00	0.00	0.00	0.00	0.00	0.00
suppressor of cytokine signaling 5-like	LOC100766747	100766747	0.00	0.00	0.00	0.00	0.00	0.00	0.00	0.00	0.00	0.00	0.00	0.00
tudor and KH domain-containing protein pseudogene	LOC100766748	100766748	0.00	0.00	0.00	0.03	0.00	0.02	0.00	0.00	0.00	1.16	0.00	0.93
chromosome unknown open reading frame, human C9orf9	LOC100766755	100766755	0.07	0.12	0.16	0.23	0.27	0.33	4.56	7.32	8.69	6.95	10.35	13.09
translationally-controlled tumor protein pseudogene	LOC100766760	100766760	300.23	314.10	308.45	281.57	220.07	207.33	3329.06	3465.23	3359.84	3012.03	2281.11	2175.91
uncharacterized LOC100766763	LOC100766763	100766763	0.00	0.00	0.00	0.00	0.00	0.00	0.00	0.00	0.00	0.00	0.00	0.00
alcohol dehydrogenase 1	LOC100766767	100766767	0.00	0.00	0.00	0.00	0.00	0.00	0.00	0.00	0.00	0.00	0.00	0.00
zinc finger protein 431-like	LOC100766771	100766771	0.00	0.00	0.00	0.00	0.03	0.00	0.00	0.00	0.00	0.00	1.03	0.00

glutathione S-transferase Y1	LOC100 766772	100766772	47.08	50.70	43.99	45.84	41.28	45.34	1431.73	1542.75	1319.10	1355.94	1208.86	1344.06
H-2 class II histocompatibility antigen, E-S beta chain-like	LOC100 766775	100766775	0.00	0.00	0.00	0.00	0.00	0.00	0.00	0.00	0.00	0.00	0.00	0.00
E3 ubiquitin-protein ligase RING2 pseudogene	LOC100 766779	100766779	0.00	0.00	0.00	0.00	0.00	0.00	0.00	0.00	0.00	0.00	0.00	0.00
draxin	LOC100 766781	100766781	0.00	0.00	0.00	0.10	0.00	0.00	0.00	0.92	0.00	1.16	0.00	0.00
eukaryotic translation initiation factor 3 subunit J-like	LOC100 766783	100766783	0.11	0.20	0.19	0.35	0.31	0.07	1.52	3.66	2.48	4.64	4.14	0.93
M-phase inducer phosphatase 3 pseudogene	LOC100 766784	100766784	0.04	0.11	0.11	0.07	0.03	0.08	0.00	0.00	0.00	0.00	0.00	1.87
olfactory receptor 7D4-like	LOC100 766786	100766786	0.00	0.00	0.00	0.00	0.00	0.00	0.00	0.00	0.00	0.00	0.00	0.00
histone H3.3 type 1-like	LOC100 766787	100766787	0.72	0.41	0.93	0.63	0.54	0.63	3.04	2.75	7.45	4.64	3.10	3.74
rhox homeobox family member 2	LOC100 766788	100766788	0.00	0.00	0.00	0.00	0.00	0.00	0.00	0.00	0.00	0.00	0.00	0.00
chromosome unknown open reading frame, human C1orf100	LOC100 766789	100766789	0.16	0.13	0.00	0.33	0.07	0.07	6.84	5.49	1.24	6.95	4.14	3.74
zinc finger protein OZF-like	LOC100 766794	100766794	2.77	2.81	2.96	4.15	4.14	3.82	174.69	164.71	191.28	245.69	253.57	249.56
fas apoptotic inhibitory molecule 1	LOC100 766798	100766798	35.62	36.09	19.96	19.16	21.88	22.55	866.63	883.92	480.69	453.14	518.53	513.13
cytochrome b-c1 complex subunit 1, mitochondrial	LOC100 766805	100766805	100.72	90.83	94.17	93.31	73.32	78.52	3968.59	3563.14	3696.45	3579.90	2836.90	3074.13
dnaJ homolog subfamily B member 6-like	LOC100 766806	100766806	0.00	0.00	0.00	0.00	0.00	0.00	0.76	2.75	1.24	0.00	2.07	0.93
mitogen-activated protein kinase 3 pseudogene	LOC100 766808	100766808	0.00	0.00	0.00	0.00	0.00	0.00	0.00	0.00	0.00	0.00	0.00	0.00
cytochrome c oxidase subunit 6B1	LOC100 766809	100766809	204.54	206.40	197.01	187.42	155.68	168.86	1988.47	2106.41	1920.27	1766.19	1546.27	1588.94
60S ribosomal protein L7 pseudogene	LOC100 766815	100766815	0.56	0.34	0.44	0.47	0.49	0.37	5.32	3.66	4.97	4.64	6.21	4.67
ATP synthase F(0) complex subunit C1, mitochondrial pseudogene	LOC100 766821	100766821	0.43	0.64	0.00	0.17	0.45	0.41	3.04	4.58	0.00	1.16	3.10	2.80

olfactory receptor 7G2-like	LOC100766823	100766823	0.00	0.00	0.00	0.00	0.00	0.00	0.00	0.00	0.00	0.00	0.00	0.00
asparagine--tRNA ligase, cytoplasmic pseudogene	LOC100766826	100766826	0.05	0.08	0.09	0.08	0.12	0.02	2.28	3.66	3.73	3.48	5.17	0.93
DNA replication complex GINS protein PSF2 pseudogene	LOC100766827	100766827	5.61	4.70	5.18	5.56	4.07	6.55	98.74	79.61	90.67	91.55	71.41	112.16
cytochrome b-245 light chain	LOC100766833	100766833	18.77	19.19	22.42	17.78	12.35	13.03	344.83	363.27	417.34	315.23	233.91	247.69
olfactory receptor 6C4-like	LOC100766834	100766834	0.00	0.00	0.00	0.00	0.00	0.00	0.00	0.00	0.00	0.00	0.00	0.00
nuclear speckle splicing regulatory protein 1 pseudogene	LOC100766836	100766836	0.65	0.90	0.81	0.69	0.83	0.47	28.10	38.43	38.50	28.97	36.22	18.69
olfactory receptor 473-like	LOC100766839	100766839	0.00	0.00	0.00	0.00	0.00	0.00	0.00	0.00	0.00	0.00	0.00	0.00
von Willebrand factor A domain-containing protein 5A-like	LOC100766840	100766840	8.45	8.79	4.92	4.78	5.72	5.23	890.94	930.59	509.26	519.20	589.94	551.46
ornithine decarboxylase pseudogene	LOC100766841	100766841	0.10	0.03	0.08	0.04	0.04	0.03	3.04	0.92	2.48	1.16	2.07	0.93
acyl-protein thioesterase 1 pseudogene	LOC100766845	100766845	0.00	0.00	0.00	0.00	0.00	0.00	0.00	0.00	0.00	0.00	0.00	0.00
60S ribosomal protein L39-like	LOC100766848	100766848	0.00	0.00	0.00	0.00	0.00	0.00	0.00	0.00	0.00	0.00	0.00	0.00
cytochrome P450 4X1	LOC100766854	100766854	2.23	1.92	0.29	0.37	0.04	0.08	206.59	183.01	28.57	37.09	3.10	5.61
BRCA1-A complex subunit Abraxas	LOC100766855	100766855	17.31	17.46	22.27	24.35	24.81	25.16	414.71	413.60	534.10	593.37	608.57	600.99
actin-related protein 2 pseudogene	LOC100766860	100766860	0.08	0.19	0.06	0.18	0.18	0.11	0.76	4.58	0.00	3.48	4.14	2.80
T-cell ecto-ADP-ribosyltransferase 2-like	LOC100766862	100766862	0.00	0.00	0.00	0.00	0.00	0.00	0.00	0.00	0.00	0.00	0.00	0.00
keratin-associated protein 10-1-like	LOC100766876	100766876	0.00	0.00	0.00	0.00	0.00	0.00	0.00	0.00	0.00	0.00	0.00	0.00
putative GTP cyclohydrolase 1 type 2 Nif3l1 pseudogene	LOC100766889	100766889	0.05	0.00	0.16	0.11	0.10	0.12	1.52	0.00	4.97	3.48	3.10	3.74
HAUS augmin-like complex subunit 7 pseudogene	LOC100766892	100766892	0.00	0.00	0.00	0.00	0.00	0.00	0.00	0.00	0.00	0.00	0.00	0.00

olfactory receptor 1N1	LOC100 766893	100766893	0.00	0.00	0.00	0.00	0.00	0.00	0.00	0.00	0.00	0.00	0.00	0.00
peptidyl-prolyl cis-trans isomerase A-like	LOC100 766894	100766894	5.78	6.76	4.66	3.25	4.24	3.51	40.26	43.01	27.33	17.38	23.80	20.56
putative vomeronasal receptor-like protein 4	LOC100 766897	100766897	0.00	0.00	0.00	0.00	0.00	0.00	0.00	0.00	0.00	0.00	0.00	0.00
zinc finger protein 60-like	LOC100 766898	100766898	3.73	3.55	5.18	5.79	6.59	6.24	334.96	312.03	460.81	522.67	581.66	564.54
putative olfactory receptor 4A8	LOC100 766900	100766900	0.00	0.00	0.00	0.00	0.00	0.00	0.00	0.00	0.00	0.00	0.00	0.00
3-oxo-5-alpha-steroid 4-dehydrogenase 2	LOC100 766917	100766917	0.05	0.04	0.05	0.00	0.02	0.02	3.04	1.83	2.48	0.00	1.03	0.93
arfaptin-2 pseudogene	LOC100 766919	100766919	0.04	0.10	0.14	0.00	0.00	0.00	0.76	1.83	2.48	0.00	0.00	0.00
proteasome activator complex subunit 3 pseudogene	LOC100 766920	100766920	0.64	0.76	0.50	0.28	0.23	0.19	17.47	19.22	12.42	6.95	6.21	4.67
nuclear receptor-binding factor 2-like	LOC100 766926	100766926	0.08	0.05	0.20	0.00	0.06	0.05	3.04	0.92	3.73	0.00	1.03	1.87
60S ribosomal protein L15 pseudogene	LOC100 766928	100766928	0.00	0.00	0.00	0.00	0.00	0.00	0.00	0.00	0.00	0.00	0.00	0.00
beta-defensin 33	LOC100 766932	100766932	0.00	0.00	0.00	0.00	0.00	0.00	0.00	0.00	0.00	0.00	0.00	0.00
protein ARMCX6-like	LOC100 766933	100766933	0.00	0.00	0.00	0.00	0.00	0.00	0.00	0.00	0.00	0.00	0.00	0.00
pumilio domain-containing protein KIAA0020 pseudogene	LOC100 766938	100766938	0.00	0.00	0.00	0.00	0.00	0.00	0.00	0.00	0.00	0.00	0.00	0.00
olfactory receptor 51V1-like	LOC100 766939	100766939	0.00	0.00	0.00	0.00	0.00	0.00	0.00	0.00	0.00	0.00	0.00	0.00
ATP-dependent RNA helicase DDX19A	LOC100 766941	100766941	45.50	47.13	47.99	50.61	48.55	50.16	2232.29	2268.37	2312.77	2461.55	2408.41	2618.94
EKC/KEOPS complex subunit LAGE3-like	LOC100 766943	100766943	0.00	0.00	0.00	0.00	0.00	0.00	0.00	0.00	0.00	0.00	0.00	0.00
olfactory receptor 1537-like	LOC100 766945	100766945	0.00	0.00	0.00	0.00	0.00	0.00	0.00	0.00	0.00	0.00	0.00	0.00
high mobility group protein HMG-I/HMG-Y-like	LOC100 766946	100766946	1.43	0.69	2.89	2.26	1.03	0.48	43.29	34.77	32.29	20.86	12.42	10.28

uncharacterized protein KIAA2012 homolog	LOC100766951	100766951	0.05	0.01	0.03	0.03	0.01	0.01	6.84	1.83	3.73	5.79	3.10	2.80
myosin-1	LOC100766952	100766952	0.00	0.00	0.00	0.01	0.00	0.00	0.00	0.00	0.00	0.00	0.00	0.00
olfactory receptor 187-like	LOC100766954	100766954	0.00	0.00	0.00	0.00	0.00	0.00	0.00	0.00	0.00	0.00	0.00	0.00
uncharacterized LOC100766956	LOC100766956	100766956	16.78	16.30	21.84	20.58	17.20	19.97	1044.37	1016.60	1376.23	1286.40	1071.21	1252.46
cytochrome c oxidase assembly factor 5	LOC100766959	100766959	17.10	17.47	17.88	19.78	21.52	23.25	984.36	1048.63	1066.95	1177.46	1296.84	1373.03
beta-hexosaminidase subunit beta-like	LOC100766960	100766960	5.28	5.28	5.43	5.54	6.84	7.31	219.51	227.84	257.11	235.26	311.53	312.18
trafficking protein particle complex subunit 2-like	LOC100766962	100766962	16.02	16.14	13.64	14.82	18.27	17.13	330.40	339.48	275.74	308.27	377.77	360.78
aminoacyl tRNA synthase complex-interacting multifunctional protein 2 pseudogene	LOC100766970	100766970	0.00	0.00	0.00	0.00	0.00	0.00	0.00	0.00	0.00	0.00	0.00	0.00
chromobox protein homolog 3 pseudogene	LOC100766974	100766974	0.18	0.28	0.10	0.32	0.45	0.59	15.95	17.39	16.15	22.02	13.45	6.54
acyl-protein thioesterase 1 pseudogene	LOC100766975	100766975	0.32	0.30	0.28	0.14	0.16	0.06	8.35	5.49	4.97	2.32	2.07	0.93
eukaryotic translation initiation factor 4B pseudogene	LOC100766976	100766976	0.00	0.00	0.00	0.00	0.00	0.00	0.00	0.00	0.00	0.00	0.00	0.00
granzyme G-like	LOC100766984	100766984	0.00	0.00	0.00	0.00	0.00	0.00	0.00	0.00	0.00	0.00	0.00	0.00
thioredoxin domain-containing protein 17 pseudogene	LOC100766985	100766985	0.00	0.00	0.00	0.00	0.00	0.19	0.00	0.00	0.00	1.16	0.00	0.93
40S ribosomal protein SA pseudogene	LOC100766992	100766992	89.67	68.67	79.24	70.45	73.65	86.47	1422.62	1062.35	1230.91	1209.91	1216.11	1466.50
uncharacterized LOC100766994	LOC100766994	100766994	0.21	0.04	0.11	0.00	0.00	0.04	4.56	0.92	2.48	0.00	0.00	0.93
putative E3 ubiquitin-protein ligase UBR7 pseudogene	LOC100766995	100766995	0.00	0.00	0.00	0.00	0.00	0.00	0.00	0.00	0.00	0.00	0.00	0.00
60S ribosomal protein L7 pseudogene	LOC100766997	100766997	0.24	0.61	0.55	0.52	0.60	0.88	6.08	15.56	13.66	12.75	17.59	22.43
olfactory receptor 1052	LOC100767000	100767000	0.00	0.00	0.00	0.00	0.00	0.00	0.00	0.00	0.00	0.00	0.00	0.00

pancreatic alpha-amylase-like	LOC100 767001	100767001	0.14	0.17	0.13	0.25	0.24	0.22	6.84	6.41	7.45	12.75	10.35	8.41
40S ribosomal protein S15a-like	LOC100 767002	100767002	0.00	0.23	0.00	0.21	0.06	0.00	0.00	0.92	0.00	0.00	0.00	0.00
V-type proton ATPase subunit d 1 pseudogene	LOC100 767009	100767009	0.03	0.00	0.05	0.00	0.00	0.00	0.76	0.92	2.48	0.00	0.00	0.00
protein kish-A pseudogene	LOC100 767013	100767013	0.54	0.65	0.94	0.00	0.80	0.00	0.76	0.92	1.24	0.00	1.03	0.00
protein SSX8-like	LOC100 767016	100767016	0.83	0.37	0.91	1.52	1.23	1.09	43.29	31.11	38.50	84.60	68.31	67.30
leukocyte immunoglobulin-like receptor subfamily A member 1	LOC100 767032	100767032	0.00	0.00	0.00	0.00	0.00	0.00	0.00	0.00	0.00	0.00	0.00	0.00
triosephosphate isomerase pseudogene	LOC100 767034	100767034	0.52	0.74	0.37	0.21	0.53	0.17	6.84	11.90	6.21	3.48	8.28	2.80
olfactory receptor 1P1-like	LOC100 767035	100767035	0.00	0.02	0.16	0.03	0.09	0.06	0.00	0.00	3.73	1.16	1.03	3.74
olfactory receptor 10W1-like	LOC100 767036	100767036	0.00	0.00	0.00	0.00	0.00	0.00	0.00	0.00	0.00	0.00	0.00	0.00
60S ribosomal protein L7a	LOC100 767038	100767038	36.47	38.34	37.17	30.51	35.02	29.33	130.64	113.46	109.30	89.24	96.25	123.38
mortality factor 4-like protein 2	LOC100 767041	100767041	0.00	0.00	0.00	0.00	0.00	0.00	0.00	0.00	0.00	0.00	0.00	0.00
ATP-binding cassette sub-family A member 3-like	LOC100 767045	100767045	0.00	0.00	0.00	0.00	0.00	0.00	0.00	0.00	0.00	0.00	0.00	0.00
PCNA-associated factor pseudogene	LOC100 767049	100767049	0.00	0.00	0.00	0.00	0.00	0.00	0.00	0.00	0.00	0.00	0.00	0.00
zinc finger protein 717-like	LOC100 767051	100767051	0.00	0.00	0.00	0.00	0.00	0.00	0.00	0.00	0.00	0.00	0.00	0.00
alcohol dehydrogenase 6	LOC100 767061	100767061	0.02	0.02	0.03	0.03	0.03	0.00	1.52	0.92	1.24	1.16	2.07	1.87
rhox homeobox family member 1-like	LOC100 767067	100767067	0.00	0.00	0.00	0.00	0.00	0.00	0.00	0.00	0.00	0.00	0.00	0.00
H-2 class II histocompatibility antigen, E-D beta chain-like	LOC100 767068	100767068	0.00	0.00	0.00	0.00	0.00	0.00	0.00	0.00	0.00	0.00	0.00	0.00
40S ribosomal protein S6	LOC100 767070	100767070	852.66	880.03	958.81	1039.74	1127.87	1035.75	12894.70	13113.35	14329.96	15534.16	16846.47	15732.36

olfactory receptor 187	LOC100767071	100767071	0.00	0.00	0.00	0.00	0.00	0.00	0.00	0.00	0.00	0.00	0.00	0.00
chromosome unknown open reading frame, human C5orf24	LOC100767072	100767072	42.76	42.80	41.37	42.33	47.81	47.19	2529.27	2612.42	2553.73	2584.39	2944.54	2871.31
40S ribosomal protein S15a pseudogene	LOC100767076	100767076	12.00	15.16	10.21	6.14	8.62	9.52	63.80	80.52	57.14	27.81	46.57	51.41
olfactory receptor 18-like	LOC100767080	100767080	0.00	0.00	0.00	0.00	0.00	0.00	0.00	0.00	0.00	0.00	0.00	0.00
zinc finger protein OZF-like	LOC100767086	100767086	0.17	0.08	0.14	0.10	0.09	0.03	9.87	2.75	6.21	5.79	3.10	4.67
40S ribosomal protein S2 pseudogene	LOC100767092	100767092	9.92	11.11	10.72	6.39	7.92	5.12	59.24	67.71	60.86	40.56	49.68	29.91
platelet-activating factor acetylhydrolase IB subunit beta pseudogene	LOC100767093	100767093	4.62	4.79	4.89	4.75	5.26	3.51	71.40	77.78	81.98	76.49	79.69	55.15
ameloblastin pseudogene	LOC100767095	100767095	0.03	0.00	0.00	0.00	0.00	0.00	0.00	0.00	0.00	0.00	0.00	0.00
transmembrane protein 233-like	LOC100767100	100767100	0.00	0.00	0.00	0.00	0.00	0.00	0.00	0.92	0.00	1.16	0.00	0.00
40S ribosomal protein S13 pseudogene	LOC100767101	100767101	0.00	0.00	0.00	0.00	0.00	0.00	0.00	0.00	0.00	0.00	0.00	0.00
heterogeneous nuclear ribonucleoprotein A3-like	LOC100767106	100767106	1.57	1.75	1.51	0.89	1.47	0.77	95.70	81.44	69.56	50.99	79.69	32.71
cell cycle control protein 50B-like	LOC100767107	100767107	0.00	0.00	0.00	0.00	0.00	0.00	0.00	0.00	0.00	0.00	0.00	0.00
olfactory receptor 1361	LOC100767113	100767113	0.00	0.00	0.00	0.00	0.00	0.00	0.00	0.00	0.00	0.00	0.00	0.00
putative gustatory receptor clone PTE38	LOC100767116	100767116	0.00	0.00	0.00	0.00	0.00	0.00	0.00	0.00	0.00	0.00	0.00	0.00
transmembrane protein 14C	LOC100767117	100767117	0.00	0.29	0.41	0.00	0.00	0.00	0.76	2.75	1.24	0.00	0.00	0.00
mortality factor 4-like protein 1 pseudogene	LOC100767119	100767119	1.69	1.63	0.75	0.65	0.99	0.32	37.22	38.43	16.15	16.22	21.73	7.48
60S ribosomal protein L26 pseudogene	LOC100767120	100767120	0.35	0.42	0.33	0.00	0.16	0.00	2.28	2.75	1.24	0.00	1.03	0.00
60S ribosomal protein L23 pseudogene	LOC100767128	100767128	0.11	0.13	0.00	0.00	0.00	0.00	0.76	0.92	0.00	0.00	0.00	0.00

ferritin heavy chain	LOC100 767129	100767129	0.00	0.00	0.00	0.00	0.00	0.00	0.00	0.00	0.00	0.00	0.00	0.00
ORM1-like protein 1	LOC100 767133	100767133	1.57	1.57	0.74	0.83	1.29	0.80	10.63	14.64	7.45	6.95	11.38	4.67
ubiquitin-conjugating enzyme E2 variant 1 pseudogene	LOC100 767134	100767134	0.00	0.00	0.00	0.00	0.00	0.00	3.80	5.49	4.97	2.32	6.21	4.67
guanylate-binding protein 4-like	LOC100 767140	100767140	0.03	0.01	0.00	0.00	0.01	0.01	2.28	0.92	0.00	0.00	1.03	0.93
GRB2-associated-binding protein 2 pseudogene	LOC100 767147	100767147	0.01	0.00	0.02	0.00	0.03	0.03	0.76	0.00	2.48	1.16	3.10	2.80
cytochrome P450 4A12	LOC100 767149	100767149	0.00	0.00	0.00	0.00	0.00	0.00	0.00	0.00	0.00	0.00	0.00	0.00
zinc finger protein 175-like	LOC100 767155	100767155	0.00	0.00	0.00	0.00	0.00	0.00	0.00	0.00	0.00	0.00	0.00	0.00
T-cell ecto-ADP-ribosyltransferase 2-like	LOC100 767157	100767157	0.00	0.00	0.00	0.00	0.00	0.00	0.00	0.00	0.00	0.00	0.00	0.00
transmembrane protein 184C pseudogene	LOC100 767160	100767160	0.02	0.05	0.01	0.00	0.03	0.01	0.00	0.00	1.24	0.00	0.00	0.00
Ig lambda-2 chain C region-like	LOC100 767162	100767162	0.00	0.00	0.00	0.00	0.00	0.00						
cystatin-8-like	LOC100 767168	100767168	0.00	0.00	0.00	0.00	0.00	0.00	0.00	0.00	0.00	0.00	0.00	0.00
keratin-associated protein 10-1-like	LOC100 767171	100767171	0.00	0.00	0.00	0.00	0.00	0.00	0.00	0.00	0.00	0.00	0.00	0.00
tubulin alpha chain-like	LOC100 767173	100767173	0.00	0.13	0.00	0.06	0.00	0.00	0.00	2.75	0.00	1.16	0.00	0.00
olfactory receptor 50	LOC100 767185	100767185	0.00	0.00	0.00	0.00	0.00	0.00	0.00	0.00	0.00	0.00	0.00	0.00
eukaryotic translation initiation factor 4B pseudogene	LOC100 767186	100767186	0.17	0.19	0.09	0.13	0.10	0.11	5.32	6.41	3.73	4.64	4.14	4.67
tudor and KH domain-containing protein pseudogene	LOC100 767189	100767189	0.06	0.02	0.07	0.03	0.03	0.07	2.28	0.92	2.48	1.16	1.03	2.80
zinc finger protein 60-like	LOC100 767190	100767190	2.83	2.72	2.95	3.42	3.69	3.36	293.94	305.62	305.55	330.29	369.49	324.33
olfactory receptor 4A5-like	LOC100 767192	100767192	0.00	0.00	0.00	0.00	0.00	0.00	0.00	0.00	0.00	0.00	0.00	0.00

60S ribosomal protein L39	LOC100767204	100767204	1250.74	1251.07	1058.60	1310.30	1237.79	1260.23	2166.97	2114.64	1684.27	1909.90	1949.91	1901.12
WD repeat domain-containing protein 83 pseudogene	LOC100767207	100767207	0.03	0.00	0.00	0.05	0.00	0.00	0.76	1.83	0.00	1.16	1.03	0.00
pre-mRNA-splicing factor SLU7 pseudogene	LOC100767210	100767210	0.00	0.00	0.00	0.00	0.00	0.00	0.00	0.00	0.00	0.00	0.00	0.00
carboxylesterase 1D-like	LOC100767211	100767211	0.00	0.00	0.00	0.00	0.00	0.00	0.00	0.00	0.00	0.00	0.00	0.00
poly(A) RNA polymerase GLD2 pseudogene	LOC100767215	100767215	0.00	0.00	0.00	0.00	0.00	0.00	0.00	0.00	0.00	0.00	0.00	0.00
condensin complex subunit 2-like	LOC100767216	100767216	0.05	0.08	0.06	0.03	0.04	0.03	2.28	4.58	3.73	1.16	2.07	1.87
olfactory receptor 52A5	LOC100767217	100767217	0.00	0.00	0.00	0.00	0.00	0.00	0.00	0.00	0.00	0.00	0.00	0.00
60S ribosomal protein L7 pseudogene	LOC100767220	100767220	0.00	0.00	0.00	0.00	0.00	0.00	0.00	0.00	0.00	0.00	0.00	0.00
beta-defensin 4-like	LOC100767221	100767221	0.00	0.00	0.00	0.00	0.00	0.00	0.00	0.00	0.00	0.00	0.00	0.00
60S ribosomal protein L23-like	LOC100767222	100767222	0.29	0.31	0.29	0.35	0.24	0.22	7.60	5.49	4.97	4.64	4.14	4.67
olfactory receptor 52A1-like	LOC100767227	100767227	0.00	0.00	0.00	0.00	0.00	0.00	0.00	0.00	0.00	0.00	0.00	0.00
serine/threonine-protein kinase OSR1 pseudogene	LOC100767231	100767231	2.32	2.48	2.12	1.69	1.64	1.58	117.73	126.27	104.34	81.12	82.80	78.51
zinc finger protein 551-like	LOC100767232	100767232	1.37	1.41	1.44	1.89	1.83	1.47	90.39	88.76	94.40	119.37	114.88	99.07
prolactin-7C1-like	LOC100767233	100767233	0.00	0.00	0.00	0.00	0.00	0.00	0.00	0.00	0.00	0.00	0.00	0.00
olfactory receptor 186-like	LOC100767240	100767240	0.00	0.00	0.00	0.00	0.00	0.00	0.00	0.00	0.00	0.00	0.00	0.00
serotransferrin-like	LOC100767241	100767241	14.16	13.71	15.33	14.53	14.91	15.13	1552.50	1514.38	1701.66	1601.63	1645.63	1667.45
speckle-type POZ protein pseudogene	LOC100767249	100767249	0.02	0.03	0.00	0.00	0.00	0.06	0.76	0.92	0.00	0.00	0.00	1.87
60S ribosomal protein L36a-like	LOC100767252	100767252	7.65	3.71	4.02	2.56	3.22	5.00	38.74	16.47	22.36	13.91	13.45	22.43

hyaluronidase PH-20-like	LOC100767253	100767253	0.00	0.00	0.00	0.00	0.00	0.00	0.00	0.00	0.00	0.00	0.00	0.00
high mobility group protein HMG-I/HMG-Y-like	LOC100767255	100767255	0.00	0.70	0.24	0.24	0.31	0.38	467.12	441.05	406.16	296.68	215.28	256.10
ribosome biogenesis protein NSA2 homolog pseudogene	LOC100767256	100767256	0.63	0.25	0.44	0.18	0.35	0.45	10.63	4.58	8.69	2.32	6.21	7.48
NADH dehydrogenase [ubiquinone] iron-sulfur protein 8, mitochondrial pseudogene	LOC100767257	100767257	0.58	0.31	0.53	0.20	0.45	0.32	6.84	3.66	6.21	2.32	5.17	3.74
chromosome unknown open reading frame, human C9orf156	LOC100767259	100767259	8.05	7.23	6.81	5.80	5.47	5.74	373.69	333.07	327.91	275.82	259.78	276.66
membrane cofactor protein	LOC100767260	100767260	0.00	0.00	0.00	0.00	0.00	0.00	0.00	0.00	0.00	0.00	0.00	0.00
cathepsin G	LOC100767270	100767270	0.00	0.00	0.00	0.00	0.00	0.00	0.00	0.00	0.00	0.00	0.00	0.00
U2 small nuclear ribonucleoprotein auxiliary factor 35 kDa subunit-related protein 1	LOC100767271	100767271	2.53	2.96	2.34	2.86	3.22	2.97	138.24	162.88	131.66	157.61	175.95	162.63
alpha-enolase pseudogene	LOC100767280	100767280	0.05	0.06	0.21	0.12	0.37	0.09	1.52	1.83	6.21	3.48	10.35	2.80
olfactory receptor 1052	LOC100767285	100767285	0.00	0.00	0.00	0.00	0.00	0.00	0.00	0.00	0.00	0.00	0.00	0.00
60S ribosomal protein L23a	LOC100767286	100767286	157.18	181.55	165.72	94.64	79.49	68.74	546.87	619.48	515.47	341.88	322.92	288.81
chromosome unknown open reading frame, human C1orf110	LOC100767287	100767287	0.00	0.00	0.00	0.00	0.00	0.00	2.28	0.00	0.00	0.00	0.00	0.00
zinc finger protein 431-like	LOC100767289	100767289	5.59	5.93	6.26	7.12	7.89	6.77	452.69	481.31	527.89	586.41	665.50	570.15
disks large-associated protein 1	LOC100767291	100767291	0.00	0.00	0.00	0.00	0.00	0.00	0.00	0.00	0.00	0.00	0.00	0.00
cytochrome c oxidase subunit 8C, mitochondrial	LOC100767292	100767292	0.00	0.00	0.00	0.00	0.00	0.00	0.00	0.00	0.00	0.00	0.00	0.00
synaptosomal-associated protein 23 pseudogene	LOC100767293	100767293	0.41	0.29	0.14	0.09	0.30	0.21	0.76	0.92	1.24	0.00	2.07	0.93
olfactory receptor 5V1	LOC100767296	100767296	0.00	0.00	0.00	0.00	0.00	0.00	0.00	0.00	0.00	0.00	0.00	0.00
cullin-4B-like	LOC100767300	100767300	0.65	0.53	0.54	0.54	0.39	0.55	41.02	33.86	34.78	33.61	24.84	34.58

elongation factor 1-gamma pseudogene	LOC100767302	100767302	0.00	0.16	0.14	0.12	0.08	0.05	0.00	4.58	4.97	1.16	2.07	1.87
homer protein homolog 2 pseudogene	LOC100767304	100767304	0.16	0.18	0.04	0.14	0.15	0.15	12.91	11.90	3.73	10.43	10.35	11.22
leukocyte immunoglobulin-like receptor subfamily B member 3	LOC100767307	100767307	0.00	0.03	0.00	0.00	0.00	0.00	0.00	0.92	0.00	0.00	0.00	0.00
prefoldin subunit 2-like	LOC100767309	100767309	0.09	0.00	0.00	0.00	0.12	0.00	0.76	0.00	1.24	1.16	1.03	0.00
synaptophysin-like protein 1 pseudogene	LOC100767311	100767311	1.75	1.67	1.66	1.52	1.19	1.67	42.53	41.18	40.99	34.77	28.98	42.99
cell division cycle-associated protein 3 pseudogene	LOC100767313	100767313	0.12	0.04	0.11	0.00	0.05	0.08	0.76	0.00	1.24	0.00	0.00	0.93
olfactory receptor 10Q1	LOC100767318	100767318	0.00	0.00	0.00	0.00	0.00	0.00	0.00	0.00	0.00	0.00	0.00	0.00
SUMO-conjugating enzyme UBC9 pseudogene	LOC100767320	100767320	0.05	0.33	0.00	0.00	0.13	0.18	0.00	1.83	0.00	0.00	1.03	0.93
zinc finger protein 615-like	LOC100767322	100767322	0.00	0.00	0.00	0.00	0.00	0.00	0.00	0.00	0.00	0.00	0.00	0.00
zinc finger protein OZF-like	LOC100767323	100767323	1.79	2.13	1.71	2.35	2.28	2.26	98.74	106.14	83.22	118.21	117.99	104.68
speedy protein B-like	LOC100767340	100767340	0.00	0.05	0.00	0.06	0.00	0.00	2.28	1.83	0.00	2.32	1.03	0.00
alcohol dehydrogenase 1-like	LOC100767342	100767342	0.00	0.00	0.00	0.00	0.00	0.00	0.00	0.00	0.00	0.00	0.00	0.93
annexin A7 pseudogene	LOC100767344	100767344	0.12	0.11	0.09	0.04	0.02	0.07	7.60	8.24	4.97	2.32	1.03	2.80
Ig kappa chain V-III region MOPC 63-like	LOC100767346	100767346	0.00	0.00	0.00	0.00	0.00	0.00						
60S ribosomal protein L5 pseudogene	LOC100767347	100767347	0.00	0.00	0.00	0.00	0.00	0.00	0.00	0.00	0.00	0.00	0.00	0.00
chromatin target of PRMT1 protein pseudogene	LOC100767349	100767349	0.00	0.00	0.00	0.00	0.00	0.00	0.00	0.00	0.00	0.00	0.00	0.00
cytochrome c oxidase subunit 7A2, mitochondrial	LOC100767351	100767351	39.59	41.32	35.09	34.79	38.12	38.96	29.62	20.13	24.84	17.38	15.52	16.82
olfactory receptor 186	LOC100767352	100767352	0.00	0.00	0.00	0.00	0.00	0.00	0.00	0.00	0.00	0.00	0.00	0.00

acyl-coenzyme A synthetase ACSM2, mitochondrial	LOC100767354	100767354	0.00	0.00	0.00	0.00	0.00	0.00	0.00	0.00	0.00	0.00	0.00	0.00
epithelial chloride channel protein-like	LOC100767355	100767355	0.00	0.00	0.00	0.00	0.00	0.00	0.00	0.00	0.00	0.00	0.00	0.00
interferon-induced protein with tetratricopeptide repeats 1-like	LOC100767358	100767358	0.00	0.00	0.00	0.00	0.00	0.00	0.00	0.00	0.00	0.00	0.00	0.00
proto-oncogene FRAT1-like	LOC100767360	100767360	0.00	0.00	0.10	0.00	0.00	0.00	0.76	0.92	0.00	0.00	0.00	0.00
ubiquitin-conjugating enzyme E2 variant 1 pseudogene	LOC100767362	100767362	0.41	0.12	0.34	0.25	0.14	0.26	6.84	2.75	4.97	6.95	2.07	4.67
60S ribosomal protein L7 pseudogene	LOC100767369	100767369	0.00	0.00	0.00	0.00	0.00	0.00	0.00	0.00	0.00	0.00	0.00	0.00
60S ribosomal protein L28-like	LOC100767372	100767372	0.00	0.00	0.00	0.00	0.00	0.00	0.00	0.00	1.24	0.00	0.00	0.00
60S ribosome subunit biogenesis protein NIP7 homolog pseudogene	LOC100767376	100767376	0.03	0.03	0.00	0.00	0.00	0.00	0.00	0.00	0.00	0.00	0.00	0.00
60S acidic ribosomal protein P1-like	LOC100767385	100767385	0.00	0.00	0.00	0.00	0.00	0.00	0.00	0.00	0.00	0.00	0.00	0.00
heat shock factor-binding protein 1 pseudogene	LOC100767389	100767389	0.00	0.00	0.00	0.00	0.00	0.00	0.00	0.00	0.00	0.00	0.00	0.00
cytochrome P450 3A9-like	LOC100767391	100767391	0.00	0.00	0.00	0.00	0.00	0.00	0.00	0.00	0.00	0.00	0.00	0.00
chromosome unknown open reading frame, human C19orf45	LOC100767394	100767394	0.00	0.00	0.03	0.03	0.03	0.10	0.76	0.92	2.48	2.32	4.14	4.67
tenascin-X-like	LOC100767397	100767397	0.49	0.52	0.11	0.07	0.04	0.07	145.07	151.90	34.78	17.38	12.42	25.24
olfactory receptor 7G2-like	LOC100767401	100767401	0.00	0.00	0.00	0.00	0.00	0.00	0.00	0.00	0.00	0.00	0.00	0.00
probable N-acetyltransferase 8B	LOC100767406	100767406	5.76	5.84	5.18	5.93	7.87	4.88	49.37	48.50	39.75	49.83	63.13	41.13
chromosome unknown open reading frame, human C1orf194	LOC100767413	100767413	1.50	1.61	0.35	0.34	1.04	0.60	22.03	26.54	6.21	11.59	16.56	11.22
olfactory receptor 6C3-like	LOC100767416	100767416	0.00	0.00	0.00	0.00	0.00	0.00	0.00	0.00	0.00	0.00	0.00	0.00
40S ribosomal protein S2 pseudogene	LOC100767417	100767417	0.54	0.41	0.21	0.43	0.36	0.59	9.11	7.32	3.73	6.95	5.17	10.28

septin-7 pseudogene	LOC100767425	100767425	0.00	0.00	0.00	0.00	0.00	0.00	0.00	0.00	0.00	0.00	0.00	0.00
rRNA/tRNA 2'-O-methyltransferase fibrillarin-like protein 1	LOC100767429	100767429	0.00	0.00	0.00	0.00	0.00	0.00	0.00	0.00	0.00	0.00	0.00	0.00
apolipoprotein L3-like	LOC100767439	100767439	0.00	0.00	0.00	0.00	0.00	0.00	0.00	0.00	0.00	0.00	0.00	0.00
zinc transporter ZIP8	LOC100767442	100767442	42.77	41.96	40.50	37.32	40.55	40.65	1544.14	1505.23	1382.44	1393.02	1489.35	1453.41
Ig lambda-1 chain C region-like	LOC100767448	100767448	0.00	0.00	0.00	0.00	0.00	0.00						
heterogeneous nuclear ribonucleoprotein H-like	LOC100767454	100767454	0.00	0.00	0.00	0.00	0.00	0.00	0.00	0.00	0.00	0.00	0.00	0.00
keratin-associated protein 10-3-like	LOC100767458	100767458	0.00	0.00	0.00	0.00	0.00	0.00	0.00	0.00	0.00	0.00	0.00	0.00
chromosome unknown open reading frame, human C18orf21	LOC100767460	100767460	30.21	32.18	33.46	33.35	33.43	34.55	783.84	831.77	876.91	852.97	867.32	875.79
40S ribosomal protein S27-like	LOC100767461	100767461	0.73	0.92	1.18	0.52	0.98	0.43	9.87	10.07	18.63	5.79	12.42	11.22
olfactory receptor 1N1	LOC100767473	100767473	0.00	0.00	0.00	0.00	0.00	0.00	0.00	0.00	0.00	0.00	0.00	0.00
histone H3.3-like	LOC100767474	100767474	0.04	0.05	0.00	0.14	0.00	0.06	0.00	0.00	0.00	1.16	1.03	0.00
protein FRG2-like	LOC100767476	100767476	0.00	0.00	0.06	0.11	0.00	0.00	8.35	4.58	4.97	2.32	1.03	8.41
putative olfactory receptor 4A8	LOC100767480	100767480	0.00	0.00	0.00	0.00	0.00	0.00	0.00	0.00	0.00	0.00	0.00	0.00
proliferation-associated protein 2G4 pseudogene	LOC100767481	100767481	0.09	0.05	0.00	0.03	0.03	0.08	3.04	0.92	0.00	1.16	0.00	1.87
tRNA (guanine(26)-N(2))-dimethyltransferase pseudogene	LOC100767486	100767486	0.00	0.00	0.00	0.00	0.00	0.00	0.00	0.00	0.00	0.00	0.00	0.00
isochorismatase domain-containing protein 2A, mitochondrial-like	LOC100767490	100767490	8.03	7.83	9.44	9.23	7.02	6.92	338.75	325.75	362.69	309.43	231.84	226.19
zinc finger protein 112-like	LOC100767491	100767491	1.99	2.92	2.13	2.36	1.60	1.82	20.51	28.37	17.39	22.02	11.38	12.15
MLV-related proviral Env polyprotein-like	LOC100767496	100767496	0.10	0.12	0.13	0.08	0.13	0.17	0.76	0.00	0.00	0.00	1.03	0.00

methyltransferase-like protein 25	LOC100767499	100767499	0.04	0.01	0.03	0.03	0.04	0.01	0.76	0.00	0.00	2.32	1.03	0.00
sentrin-specific protease 2 pseudogene	LOC100767500	100767500	0.00	0.00	0.00	0.00	0.00	0.00	0.00	0.00	0.00	0.00	0.00	0.00
olfactory receptor 52A1-like	LOC100767506	100767506	0.00	0.00	0.00	0.00	0.00	0.00	0.00	0.00	0.00	0.00	0.00	0.00
chromosome unknown open reading frame, human C17orf78	LOC100767508	100767508	0.12	0.11	0.03	0.05	0.05	0.05	13.67	12.81	2.48	4.64	5.17	5.61
partner of Y14 and mago pseudogene	LOC100767509	100767509	0.00	0.00	0.00	0.00	0.00	0.00	0.00	0.00	0.00	0.00	0.00	0.00
beta-defensin 13-like	LOC100767510	100767510	0.00	0.00	0.00	0.00	0.00	0.00	0.00	0.00	0.00	0.00	0.00	0.00
insulin-induced gene 1 protein pseudogene	LOC100767511	100767511	0.04	0.10	0.07	0.00	0.00	0.00	0.76	1.83	1.24	0.00	0.00	0.00
glyceraldehyde-3-phosphate dehydrogenase-like	LOC100767512	100767512	0.00	0.00	0.05	0.05	0.09	0.00	0.00	0.00	1.24	1.16	2.07	0.93
60S ribosomal protein L30-like	LOC100767516	100767516	0.26	0.00	0.22	0.21	0.37	0.17	1.52	0.92	1.24	1.16	2.07	0.93
olfactory receptor 52A5-like	LOC100767517	100767517	0.00	0.00	0.00	0.00	0.00	0.00	0.00	0.00	0.00	0.00	0.00	0.00
olfactory receptor 10K1-like	LOC100767521	100767521	0.00	0.00	0.00	0.00	0.00	0.00	0.00	0.00	0.00	0.00	0.00	0.00
TIP41-like protein pseudogene	LOC100767522	100767522	2.19	2.27	2.59	0.83	0.97	0.87	60.00	60.39	59.62	18.54	19.66	16.82
vomeronasal type-1 receptor 4-like	LOC100767523	100767523	0.00	0.00	0.00	0.00	0.00	0.00	0.00	0.00	0.00	0.00	0.00	0.00
prolactin-2A1-like	LOC100767524	100767524	0.00	0.00	0.00	0.00	0.00	0.00	0.00	0.00	0.00	0.00	0.00	0.00
60S acidic ribosomal protein P1-like	LOC100767529	100767529	606.01	601.22	573.12	312.19	131.72	156.03	3047.27	2905.23	2735.08	1450.97	618.92	740.26
glutathione S-transferase Mu 3	LOC100767530	100767530	3.47	3.47	9.37	8.98	13.74	12.06	105.58	99.74	272.02	282.78	404.68	352.37
olfactory receptor Olfr180	LOC100767532	100767532	0.00	0.00	0.00	0.00	0.00	0.00	0.00	0.00	0.00	0.00	0.00	0.00
sperm motility kinase X	LOC100767536	100767536	0.00	0.02	0.00	0.00	0.00	0.00	0.00	0.00	0.00	0.00	0.00	0.00

NADH dehydrogenase [ubiquinone] iron-sulfur protein 5 pseudogene	LOC100767544	100767544	0.00	0.16	0.23	0.00	0.19	0.00	0.00	0.92	1.24	0.00	1.03	0.00
60S ribosomal protein L18a pseudogene	LOC100767545	100767545	1.89	1.60	0.83	0.45	0.70	0.45	38.74	36.60	27.33	16.22	13.45	9.35
transmembrane protein 106A pseudogene	LOC100767549	100767549	0.00	0.00	0.00	0.00	0.00	0.00	0.00	0.00	0.00	0.00	0.00	0.00
deoxyuridine 5'-triphosphate nucleotidohydrolase pseudogene	LOC100767560	100767560	0.25	0.20	0.41	0.13	0.12	0.10	2.28	1.83	3.73	1.16	1.03	2.80
chymase-like	LOC100767563	100767563	0.00	0.00	0.00	0.00	0.00	0.00	0.00	0.00	0.00	0.00	0.00	0.00
chromosome unknown open reading frame, human C19orf25	LOC100767566	100767566	48.49	45.94	55.26	41.15	25.39	30.44	1133.23	1060.52	1259.48	822.83	419.17	538.37
olfactory receptor 1052-like	LOC100767577	100767577	0.00	0.00	0.00	0.00	0.00	0.00	0.00	0.00	0.00	0.00	0.00	0.00
nucleoside diphosphate kinase A 1 pseudogene	LOC100767579	100767579	0.00	0.00	0.00	0.00	0.00	0.00	0.00	0.00	0.00	0.00	0.00	0.00
melanoma-associated antigen B4-like	LOC100767584	100767584	0.51	0.34	0.00	0.03	0.00	0.00	22.03	12.81	0.00	1.16	0.00	0.00
neurogenic locus notch homolog protein 2 pseudogene	LOC100767588	100767588	0.07	0.12	0.01	0.06	0.08	0.04	3.04	8.24	1.24	2.32	3.10	0.93
aphrodisin-like	LOC100767590	100767590	0.00	0.00	0.00	0.00	0.00	0.00	0.00	0.00	0.00	0.00	0.00	0.00
60S ribosomal protein L18 pseudogene	LOC100767601	100767601	17.34	18.26	18.87	16.57	15.77	16.33	1986.20	1916.08	2178.62	2029.27	1416.90	1688.95
killer cell immunoglobulin-like receptor 3DL1	LOC100767604	100767604	0.00	0.00	0.00	0.00	0.00	0.00	0.00	0.00	0.00	0.00	0.00	0.00
ly-6/neurotoxin-like protein 1	LOC100767605	100767605	0.00	0.00	0.00	0.00	0.00	0.00	0.00	0.00	0.00	0.00	0.00	0.00
ATP-binding cassette sub-family A member 13-like	LOC100767606	100767606	0.00	0.01	0.00	0.00	0.00	0.00	0.00	0.92	0.00	0.00	0.00	0.00
olfactory receptor 9I1	LOC100767607	100767607	0.00	0.00	0.00	0.00	0.00	0.00	0.00	0.00	0.00	0.00	0.00	0.00
histone H4	LOC100767612	100767612	0.10	0.11	0.07	0.03	0.00	0.00	0.00	0.00	0.00	0.00	0.00	0.00
40S ribosomal protein S2 pseudogene	LOC100767613	100767613	1.96	2.49	2.29	1.86	2.38	1.18	22.03	23.79	23.60	18.54	25.87	11.22

chromosome unknown open reading frame, human C1orf168	LOC100767623	100767623	0.00	0.00	0.00	0.00	0.00	0.00	0.00	0.00	0.00	0.00	0.00	0.00
putative olfactory receptor 10AC1	LOC100767629	100767629	0.00	0.00	0.00	0.00	0.00	0.00	0.00	0.00	0.00	0.00	0.00	0.00
tubulin alpha chain-like	LOC100767630	100767630	0.00	0.00	0.00	0.00	0.00	0.00	1.52	2.75	0.00	1.16	0.00	0.00
alcohol dehydrogenase 4	LOC100767633	100767633	0.00	0.00	0.00	0.00	0.00	0.00	0.00	0.00	0.00	0.00	0.00	0.93
cytochrome P450 4A14-like	LOC100767636	100767636	0.00	0.00	0.00	0.00	0.00	0.00	0.00	0.00	0.00	0.00	0.00	0.00
cofilin-1 pseudogene	LOC100767637	100767637	0.00	0.00	0.00	0.00	0.00	0.00	0.00	0.00	0.00	0.00	0.00	0.00
	LOC100767638	100767638	0.20	0.30	0.20	0.08	0.17	0.19	83.55	99.74	70.80	77.65	109.71	114.03
H-2 class II histocompatibility antigen, E-U alpha chain	LOC100767639	100767639	0.00	0.00	0.00	0.00	0.00	0.00	0.00	0.00	0.00	0.00	0.00	0.00
olfactory receptor 5H2-like	LOC100767642	100767642	0.00	0.00	0.00	0.00	0.00	0.00	0.00	0.00	0.00	0.00	0.00	0.00
very low-density lipoprotein receptor	LOC100767645	100767645	28.65	27.08	30.03	33.57	32.72	31.58	2190.51	2147.58	2322.70	2678.26	2599.89	2489.03
histone RNA hairpin-binding protein pseudogene	LOC100767650	100767650	0.69	0.19	0.52	0.51	0.95	0.17	3.04	4.58	4.97	2.32	3.10	3.74
keratin, type I cytoskeletal 10	LOC100767651	100767651	0.00	0.00	0.00	0.00	0.00	0.00	0.00	0.00	0.00	0.00	0.00	0.00
olfactory receptor 867	LOC100767654	100767654	0.00	0.00	0.00	0.00	0.00	0.00	0.00	0.00	0.00	0.00	0.00	0.00
cytochrome c oxidase protein 20 homolog	LOC100767657	100767657	8.34	8.34	7.34	8.67	10.19	8.60	602.31	628.63	541.55	632.77	735.88	650.53
toll-like receptor 13	LOC100767676	100767676	0.00	0.01	0.00	0.00	0.00	0.02	0.00	0.92	0.00	0.00	0.00	1.87
nuclear pore complex protein Nup54 pseudogene	LOC100767677	100767677	0.00	0.00	0.00	0.00	0.00	0.00	0.00	0.00	0.00	0.00	0.00	0.00
putative olfactory receptor 2W6	LOC100767678	100767678	0.00	0.00	0.00	0.00	0.00	0.00	0.00	0.00	0.00	0.00	0.00	0.00
zinc finger protein OZF-like	LOC100767679	100767679	3.15	3.01	2.96	3.23	3.23	3.31	204.32	204.97	209.91	219.04	216.31	222.45

adiponectin receptor protein 1-like	LOC100767683	100767683	0.00	0.00	0.00	0.00	0.00	0.00	0.00	0.00	0.00	0.00	1.03	0.93
60S ribosomal protein L27 pseudogene	LOC100767689	100767689	0.36	0.66	0.40	0.39	0.34	0.16	2.28	3.66	2.48	2.32	2.07	0.93
probable N-acetyltransferase CML3	LOC100767690	100767690	34.37	34.75	42.27	41.76	39.45	39.14	1539.59	1507.98	1866.86	1816.03	1704.62	1786.15
steroid 21-hydroxylase	LOC100767691	100767691	0.02	0.02	0.00	0.00	0.00	0.03	1.52	0.92	0.00	0.00	0.00	0.93
olfactory receptor 7G2	LOC100767694	100767694	0.00	0.00	0.00	0.00	0.00	0.00	0.00	0.00	0.00	0.00	0.00	0.00
pre-mRNA-processing factor 6	LOC100767706	100767706	139.49	141.32	116.10	111.09	117.57	123.62	6773.57	6849.94	5621.69	5430.70	5801.10	6037.97
protein AKNAD1-like	LOC100767707	100767707	0.08	0.13	0.05	0.13	0.11	0.03	33.42	37.52	18.63	13.91	14.49	13.09
olfactory receptor 6C3-like	LOC100767709	100767709	0.00	0.00	0.00	0.00	0.00	0.00	0.00	0.00	0.00	0.00	0.00	0.00
chromosome unknown open reading frame, human C3orf58	LOC100767713	100767713	5.68	6.22	3.68	3.29	4.40	3.47	499.02	543.53	332.88	289.73	393.29	307.51
chromosome unknown open reading frame, human C21orf62	LOC100767714	100767714	0.00	0.00	0.00	0.00	0.00	0.00	0.00	0.00	0.00	0.00	0.00	0.00
probable tRNA N6-adenosine threonylcarbamoyltransferase, mitochondrial	LOC100767715	100767715	6.54	6.95	7.03	6.61	7.66	8.43	265.08	271.76	289.41	274.66	317.74	315.92
vesicle-associated membrane protein 4 pseudogene	LOC100767716	100767716	2.68	2.04	1.76	2.38	2.81	2.69	38.74	28.37	26.08	25.50	32.08	28.04
transcription factor Spi-C pseudogene	LOC100767718	100767718	0.00	0.00	0.00	0.07	0.00	0.00	0.00	0.00	0.00	1.16	0.00	0.00
apolipoprotein L2-like	LOC100767734	100767734	0.00	0.00	0.00	0.00	0.00	0.00	0.00	0.00	0.00	0.00	0.00	0.00
olfactory receptor 13G1	LOC100767735	100767735	0.00	0.00	0.00	0.00	0.00	0.00	0.00	0.00	0.00	0.00	0.00	0.00
lysine-specific demethylase 4D-like	LOC100767737	100767737	0.00	0.00	0.00	0.00	0.00	0.00	0.00	0.00	0.00	0.00	0.00	0.00
acyl-coenzyme A synthetase ACSM1, mitochondrial-like	LOC100767739	100767739	0.00	0.00	0.00	0.03	0.00	0.00	0.00	0.00	0.00	1.16	0.00	0.00
ADP-ribosylation factor-like protein 4D	LOC100767745	100767745	5.64	6.22	3.60	3.41	2.32	2.62	0.00	0.00	0.00	0.00	0.00	0.00

olfactory receptor 7G2-like	LOC100 767746	100767746	0.00	0.00	0.00	0.00	0.00	0.00	0.00	0.00	0.00	0.00	0.00	0.00
olfactory receptor 6B2	LOC100 767747	100767747	0.00	0.00	0.00	0.00	0.00	0.00	0.00	0.00	0.00	0.00	0.00	0.00
coiled-coil domain-containing protein 170-like	LOC100 767748	100767748	0.09	0.10	0.08	0.04	0.08	0.03	10.63	10.07	8.69	4.64	8.28	2.80
IQ domain-containing protein F5 pseudogene	LOC100 767753	100767753	0.00	0.00	0.00	0.00	0.00	0.00	0.00	0.00	0.00	0.00	0.00	0.00
chromosome unknown open reading frame, human C1orf216	LOC100 767757	100767757	4.91	4.83	6.63	6.06	4.37	5.31	164.82	181.18	234.75	227.15	208.03	237.41
alpha-enolase-like	LOC100 767766	100767766	0.52	0.52	0.61	0.26	0.42	0.40	6.84	6.41	6.21	1.16	5.17	6.54
olfactory receptor 1N2	LOC100 767768	100767768	0.00	0.00	0.00	0.00	0.00	0.00	0.00	0.00	0.00	0.00	0.00	0.00
10 kDa heat shock protein, mitochondrial pseudogene	LOC100 767769	100767769	5.26	2.63	2.36	1.49	2.02	2.09	31.90	22.88	18.63	9.27	10.35	14.02
zinc finger protein 60-like	LOC100 767772	100767772	2.58	2.70	3.39	3.65	3.82	3.80	281.79	298.30	388.77	406.78	423.31	424.34
olfactory receptor 4A5-like	LOC100 767774	100767774	0.07	0.00	0.00	0.00	0.00	0.00	1.52	0.92	0.00	0.00	0.00	0.00
ubiquitin-conjugating enzyme E2 variant 1-like	LOC100 767776	100767776	0.00	0.00	0.00	0.00	0.27	0.00	21.27	36.60	27.33	23.18	19.66	31.78
60S ribosomal protein L4 pseudogene	LOC100 767783	100767783	0.33	0.28	0.42	0.26	0.37	0.41	12.15	10.07	17.39	9.27	15.52	13.09
protein S100-A11 pseudogene	LOC100 767786	100767786	0.00	0.00	0.00	0.00	0.00	0.00	0.00	0.00	0.00	0.00	0.00	0.00
small ubiquitin-related modifier 2 pseudogene	LOC100 767791	100767791	24.09	31.12	35.34	22.89	14.89	14.39	142.79	168.37	162.71	92.71	53.82	59.82
protein Red pseudogene	LOC100 767793	100767793	0.00	0.00	0.00	0.00	0.00	0.02	0.00	0.00	0.00	0.00	0.00	0.93
hemoglobin subunit beta	LOC100 767796	100767796	0.00	0.00	0.00	0.00	0.00	0.00	0.76	0.00	0.00	0.00	0.00	0.00
sperm-associated antigen 7 pseudogene	LOC100 767800	100767800	0.00	0.00	0.00	0.00	0.00	0.00	0.76	0.00	0.00	0.00	0.00	0.00
60S ribosomal protein L7a pseudogene	LOC100 767801	100767801	0.05	0.04	0.00	0.08	0.03	0.16	0.76	0.00	0.00	1.16	0.00	2.80

uncharacterized LOC100767803	LOC100 767803	100767803	9.11	7.48	6.37	3.65	1.61	1.96	215.71	172.03	158.99	130.96	103.50	99.07
zona pellucida sperm-binding protein 4 pseudogene	LOC100 767805	100767805	0.00	0.00	0.00	0.00	0.00	0.00	0.00	0.00	0.00	0.00	0.00	0.00
40S ribosomal protein SA pseudogene	LOC100 767806	100767806	11.87	9.83	10.15	5.29	5.31	5.62	85.83	64.05	78.25	53.31	42.43	50.47
cytochrome b reductase 1	LOC100 767807	100767807	8.61	8.72	11.59	12.50	14.73	14.44	1110.45	1124.58	1505.41	1614.38	1886.78	1872.14
olfactory receptor 150-like	LOC100 767813	100767813	0.00	0.00	0.00	0.00	0.00	0.00	0.00	0.00	0.00	0.00	0.00	0.00
AT-rich interactive domain-containing protein 4A-like	LOC100 767815	100767815	0.05	0.06	0.00	0.29	0.06	0.06	3.80	2.75	3.73	6.95	1.03	2.80
uncharacterized LOC100767816	LOC100 767816	100767816	0.00	0.00	0.00	0.00	0.00	0.00	0.00	0.00	0.00	0.00	0.00	0.00
glutathione S-transferase Mu 6-like	LOC100 767818	100767818	0.08	0.06	0.04	0.00	0.00	0.03	5.32	7.32	1.24	2.32	2.07	5.61
olfactory receptor 187-like	LOC100 767820	100767820	0.00	0.00	0.00	0.00	0.00	0.00	0.00	0.00	0.00	0.00	0.00	0.00
actin-related protein 2/3 complex subunit 1B-like	LOC100 767822	100767822	0.00	0.00	0.00	0.00	0.00	0.00	0.00	0.00	0.00	0.00	0.00	0.00
calcium-activated chloride channel regulator 4-like	LOC100 767824	100767824	0.00	0.00	0.00	0.00	0.00	0.00	0.76	0.00	0.00	0.00	1.03	1.87
nucleophosmin pseudogene	LOC100 767826	100767826	0.11	0.13	0.06	0.00	0.00	0.04	2.28	2.75	1.24	0.00	0.00	0.93
UDP-glucose 6-dehydrogenase	LOC100 767827	100767827	132.69	138.11	116.34	152.56	161.10	161.20	1967.21	2081.70	1799.78	2203.11	2433.25	2328.26
protein DDC8	LOC100 767828	100767828	0.95	1.13	1.06	1.02	1.04	0.89	65.32	81.44	78.25	75.33	74.52	63.56
60S ribosomal protein L7 pseudogene	LOC100 767832	100767832	0.00	0.00	0.00	0.00	0.00	0.00	0.00	0.00	0.00	0.00	0.00	0.00
glutathione S-transferase omega-1 pseudogene	LOC100 767843	100767843	0.00	0.00	0.00	0.00	0.00	0.00	0.00	0.00	0.00	0.00	0.00	0.00
60S ribosomal protein L13a pseudogene	LOC100 767858	100767858	0.05	0.00	0.00	0.00	0.13	0.00	0.00	0.00	0.00	0.00	1.03	0.00
olfactory receptor 8H1-like	LOC100 767861	100767861	0.00	0.00	0.00	0.00	0.00	0.00	0.00	0.00	0.00	0.00	0.00	0.00

protein FAM49B-like	LOC100 767863	100767863	46.49	44.76	28.87	31.42	33.65	32.50	1197.79	1188.63	760.16	820.52	854.90	842.14
zinc finger protein 260-like	LOC100 767866	100767866	5.85	5.84	5.77	6.49	7.05	6.88	356.22	364.18	357.72	399.83	454.36	442.10
60S ribosomal protein L7a pseudogene	LOC100 767868	100767868	4.02	3.77	2.62	2.94	3.43	2.61	18.23	34.77	18.63	13.91	31.05	14.02
olfactory receptor 1G1-like	LOC100 767872	100767872	0.00	0.00	0.00	0.00	0.00	0.00	0.00	0.00	0.00	0.00	0.00	0.00
peptidyl-prolyl cis-trans isomerase A-like	LOC100 767874	100767874	0.00	0.13	0.24	0.23	0.10	0.00	0.76	1.83	2.48	2.32	1.03	0.00
leucine-rich repeat-containing protein PRAME-like	LOC100 767875	100767875	0.00	0.00	0.00	0.00	0.00	0.00	0.00	0.00	0.00	0.00	0.00	0.00
uncharacterized LOC100767876	LOC100 767876	100767876	0.14	0.06	0.00	0.07	0.00	0.00	2.28	1.83	1.24	1.16	0.00	0.00
D-3-phosphoglycerate dehydrogenase pseudogene	LOC100 767881	100767881	0.02	0.02	0.00	0.03	0.02	0.02	0.76	0.92	0.00	1.16	1.03	0.93
leukocyte immunoglobulin-like receptor subfamily B member 4	LOC100 767889	100767889	0.00	0.00	0.00	0.00	0.00	0.00	0.00	0.00	0.00	0.00	0.00	0.00
60S ribosomal protein L4 pseudogene	LOC100 767890	100767890	0.00	0.00	0.00	0.00	0.00	0.00	0.00	0.00	0.00	0.00	0.00	0.00
uncharacterized LOC100767896	LOC100 767896	100767896	0.00	0.00	0.00	0.00	0.00	0.00	0.00	0.00	0.00	0.00	0.00	0.00
zinc finger protein 420-like	LOC100 767898	100767898	4.78	4.17	5.77	6.78	6.85	5.87	272.67	243.40	336.61	388.24	381.91	346.76
vomeronasal type-1 receptor 4-like	LOC100 767899	100767899	0.03	0.00	0.00	0.00	0.00	0.00	0.76	0.00	0.00	0.00	0.00	0.00
olfactory receptor 6C6	LOC100 767900	100767900	0.00	0.00	0.00	0.00	0.00	0.00	0.00	0.00	0.00	0.00	0.00	0.00
mitotic spindle assembly checkpoint protein MAD2B pseudogene	LOC100 767902	100767902	0.00	0.00	0.00	0.05	0.00	0.00	0.00	0.00	0.00	0.00	0.00	0.00
disintegrin and metalloproteinase domain-containing protein 21	LOC100 767903	100767903	0.00	0.00	0.00	0.00	0.00	0.00	0.00	0.00	0.00	0.00	0.00	0.00
thyrotropin-releasing hormone receptor-like	LOC100 767904	100767904	0.06	0.04	0.02	0.00	0.06	0.00	4.56	1.83	1.24	0.00	3.10	2.80
2'-5'-oligoadenylate synthase 1A-like	LOC100 767905	100767905	0.00	0.00	0.00	0.00	0.00	0.00	0.00	0.00	0.00	0.00	0.00	0.00

chromosome unknown open reading frame, human C16orf72	LOC100767915	100767915	29.85	27.16	29.46	31.95	30.64	30.83	1236.53	1146.54	1230.91	1326.96	1274.07	1271.15
copine-8	LOC100767916	100767916	35.47	38.84	42.39	40.99	48.21	46.28	1664.15	1718.43	1823.38	1907.58	2143.46	2062.82
signal recognition particle subunit SRP72 pseudogene	LOC100767918	100767918	0.00	0.00	0.00	0.00	0.00	0.00	0.00	0.00	0.00	0.00	0.00	0.00
uncharacterized LOC100767920	LOC100767920	100767920	0.00	0.00	0.00	0.00	0.00	0.00	0.00	0.00	0.00	0.00	0.00	0.00
cytochrome P450 4A14	LOC100767921	100767921	0.07	0.02	0.00	0.01	0.00	0.00	0.76	0.00	0.00	0.00	0.00	0.00
doublesex- and mab-3-related transcription factor C1-like	LOC100767922	100767922	0.00	0.00	0.00	0.00	0.00	0.00	0.00	0.00	0.00	0.00	0.00	0.00
uncharacterized LOC100767926	LOC100767926	100767926	184.65	183.40	144.76	137.27	174.10	182.95	3068.54	3044.32	2398.47	2220.49	2797.57	2943.27
olfactory receptor 183-like	LOC100767927	100767927	0.03	0.00	0.00	0.00	0.00	0.00	0.76	0.00	0.00	0.00	0.00	0.00
hereditary hemochromatosis protein-like	LOC100767932	100767932	0.00	0.00	0.00	0.00	0.00	0.00	0.00	0.00	0.00	0.00	0.00	0.00
chromosome unknown open reading frame, human C14orf2	LOC100767934	100767934	398.54	350.38	361.35	385.74	400.31	446.45	2228.49	1994.77	2060.62	2014.20	2194.17	2388.08
heterogeneous nuclear ribonucleoprotein K pseudogene	LOC100767941	100767941	0.02	0.06	0.11	0.04	0.00	0.00	2.28	1.83	3.73	3.48	0.00	0.00
chromosome unknown open reading frame, human C17orf58	LOC100767946	100767946	0.00	0.00	0.00	0.00	0.00	0.00	0.00	1.83	0.00	0.00	0.00	0.00
zinc finger protein 431-like	LOC100767949	100767949	1.03	1.00	0.79	1.24	1.29	0.83	70.64	68.63	54.65	85.76	84.87	63.56
ATP synthase subunit e, mitochondrial-like	LOC100767951	100767951	0.46	0.37	0.00	0.00	0.00	0.20	2.28	1.83	1.24	1.16	0.00	0.93
putative RNA-binding protein Luc7-like 2	LOC100767953	100767953	154.40	157.31	145.90	149.98	161.00	158.76	10267.45	10548.51	9920.55	10237.90	10871.50	10719.73
uncharacterized protein FLJ43738-like	LOC100767956	100767956	0.51	0.56	0.32	0.28	0.47	0.57	94.18	105.23	67.07	66.06	90.04	92.53
importin subunit alpha-1-like	LOC100767960	100767960	0.04	0.03	0.09	0.04	0.02	0.07	3.04	1.83	4.97	2.32	1.03	3.74
protein SET-like	LOC100767966	100767966	0.03	0.17	0.13	0.05	0.05	0.04	0.76	2.75	2.48	1.16	0.00	0.00

olfactory receptor 2W1	LOC100767967	100767967	0.00	0.00	0.00	0.00	0.00	0.00	0.00	0.00	0.00	0.00	0.00	0.00
olfactory receptor 49	LOC100767970	100767970	0.00	0.00	0.00	0.00	0.00	0.00	0.00	0.00	0.00	0.00	0.00	0.00
glyceraldehyde-3-phosphate dehydrogenase-like	LOC100767974	100767974	0.00	0.00	0.00	0.00	0.00	0.00	0.00	0.00	0.00	0.00	0.00	0.00
N-acetyltransferase 8	LOC100767982	100767982	0.12	0.12	0.16	0.09	0.03	0.05	8.35	8.24	6.21	4.64	5.17	3.74
chondroitin sulfate synthase 3	LOC100767985	100767985	0.00	0.00	0.00	0.00	0.00	0.00	0.00	0.00	0.00	0.00	0.00	0.00
olfactory receptor 7G2-like	LOC100767986	100767986	0.00	0.00	0.00	0.00	0.00	0.00	0.00	0.00	0.00	0.00	0.00	0.00
chromosome unknown open reading frame, human C11orf71	LOC100767988	100767988	7.09	8.06	6.90	3.19	1.84	1.14	84.31	93.33	86.95	48.67	24.84	22.43
putative spermatogenesis-associated protein 31D3	LOC100767989	100767989	0.01	0.01	0.00	0.00	0.02	0.00	0.76	1.83	0.00	0.00	2.07	0.00
probable N-acetyltransferase CML1	LOC100767993	100767993	5.44	5.11	5.55	4.16	3.28	4.33	73.68	58.56	70.80	54.47	39.33	54.21
leydig cell tumor 10 kDa protein-like	LOC100767994	100767994	0.00	0.00	0.00	0.35	0.00	0.28	0.00	0.00	0.00	1.16	0.00	0.93
cTAGE family member 5-like	LOC100767997	100767997	0.52	0.94	0.91	1.13	1.05	1.12	42.53	57.65	62.10	76.49	68.31	71.03
olfactory receptor 6C2-like	LOC100768003	100768003	0.00	0.00	0.00	0.00	0.00	0.00	0.00	0.00	0.00	0.00	0.00	0.00
olfactory receptor 4M1	LOC100768007	100768007	0.00	0.00	0.00	0.00	0.00	0.00	0.00	0.00	0.00	0.00	0.00	0.00
eukaryotic translation initiation factor 2 subunit 3, Y-linked pseudogene	LOC100768010	100768010	0.00	0.00	0.00	0.00	0.00	0.00	0.00	0.00	0.00	0.00	0.00	0.00
lymphocyte antigen 6D-like	LOC100768011	100768011	0.89	0.85	0.30	0.29	0.72	1.02	28.10	21.96	6.21	9.27	21.73	29.91
protein KIBRA	LOC100768014	100768014	0.00	0.01	0.00	0.00	0.00	0.00	0.76	2.75	1.24	0.00	0.00	0.00
tubulin beta-6 chain pseudogene	LOC100768015	100768015	0.59	0.66	0.48	0.32	0.27	0.24	103.30	102.48	81.98	42.88	17.59	10.28
chromosome unknown open reading frame, human C6orf203	LOC100768017	100768017	17.55	18.17	19.93	19.26	19.62	19.19	542.31	562.75	612.35	591.05	602.36	584.17

protein FAM111A-like	LOC100768019	100768019	7.11	6.89	11.12	13.65	13.53	11.88	627.38	606.67	988.70	1209.91	1181.95	1061.78
60S ribosomal protein L32 pseudogene	LOC100768022	100768022	0.00	0.00	0.00	0.00	0.00	0.00	0.00	0.00	0.00	0.00	0.00	0.00
histone-binding protein RBBP4 pseudogene	LOC100768023	100768023	0.05	0.14	0.19	0.08	0.23	0.08	25.06	22.88	16.15	17.38	40.36	28.97
melanoma-associated antigen B4-like	LOC100768024	100768024	0.00	0.00	0.00	0.00	0.00	0.00	0.00	0.00	0.00	0.00	0.00	0.00
upstream-binding factor 1-like protein 1	LOC100768028	100768028	0.02	0.00	0.03	0.00	0.00	0.00	0.76	0.92	1.24	0.00	0.00	0.00
TP53-regulated inhibitor of apoptosis 1 pseudogene	LOC100768030	100768030	0.00	0.00	0.00	0.00	0.00	0.00	0.00	0.00	0.00	0.00	0.00	0.00
keratin, type I cytoskeletal 24-like	LOC100768034	100768034	0.00	0.00	0.00	0.00	0.00	0.00	0.00	0.00	0.00	0.00	0.00	0.00
uncharacterized LOC100768036	LOC100768036	100768036	14.26	14.33	15.36	16.23	13.94	15.46	1.52	3.66	2.48	1.16	5.17	0.00
olfactory receptor 6B2	LOC100768038	100768038	0.02	0.00	0.00	0.00	0.00	0.00	0.00	0.00	0.00	0.00	0.00	0.00
chromosome unknown open reading frame, human C20orf202	LOC100768041	100768041	0.00	0.16	0.00	0.00	0.00	0.00	0.00	0.92	0.00	0.00	0.00	0.00
ubiquitin-like protein FUBI pseudogene	LOC100768052	100768052	1.77	1.43	1.18	0.19	0.99	0.70	18.99	13.73	7.45	0.00	6.21	6.54
SLC2A4 regulator-like	LOC100768053	100768053	1.30	1.16	1.48	1.56	1.44	1.32	81.27	84.18	84.46	105.46	103.50	87.86
protein FAM60A pseudogene	LOC100768057	100768057	0.04	0.00	0.00	0.07	0.00	0.11	37.22	36.60	45.96	17.38	23.80	15.89
BRCA1-associated RING domain protein 1-like	LOC100768058	100768058	0.05	0.00	0.00	0.00	0.00	0.00	1.52	0.00	0.00	0.00	0.00	0.00
ankyrin repeat domain-containing protein 36A-like	LOC100768060	100768060	0.67	0.68	0.68	0.75	0.88	0.83	118.49	120.78	119.24	144.86	153.18	152.35
chromosome unknown open reading frame, human C5orf45	LOC100768063	100768063	12.64	12.69	15.21	12.47	9.66	10.35	432.18	441.96	517.95	428.80	329.13	372.00
olfactory receptor 4A5-like	LOC100768064	100768064	0.00	0.00	0.00	0.00	0.00	0.00	0.00	0.00	0.00	0.00	0.00	0.00
60S acidic ribosomal protein P0 pseudogene	LOC100768075	100768075	12.67	11.70	9.52	4.66	1.86	2.30	331.92	305.62	248.42	118.21	49.68	57.95

protein SPACA7-like	LOC100768076	100768076	0.00	0.00	0.00	0.00	0.00	0.00	0.00	0.00	0.00	0.00	0.00	0.93
dynein light chain Tctex-type 3 pseudogene	LOC100768077	100768077	0.00	0.00	0.00	0.00	0.00	0.00	0.00	0.00	0.00	0.00	0.00	0.00
60S acidic ribosomal protein P2 pseudogene	LOC100768083	100768083	0.00	0.14	0.00	0.00	0.00	0.00	0.76	2.75	1.24	1.16	0.00	0.00
olfactory receptor 52E4-like	LOC100768084	100768084	0.00	0.00	0.00	0.00	0.00	0.00	0.00	0.00	0.00	0.00	0.00	0.00
hemoglobin subunit beta-Y	LOC100768087	100768087	0.00	0.00	0.00	0.00	0.00	0.00	0.00	0.00	0.00	0.00	0.00	0.00
alpha-defensin 10-like	LOC100768088	100768088	0.00	0.00	0.00	0.00	0.00	0.00	0.00	0.00	0.00	0.00	0.00	0.00
NADH dehydrogenase [ubiquinone] 1 beta subcomplex subunit 7	LOC100768089	100768089	0.00	0.00	0.00	0.00	0.00	0.00	0.00	0.00	0.00	0.00	0.00	0.00
prostaglandin-E(2) 9-reductase-like	LOC100768090	100768090	0.00	0.00	0.00	0.00	0.00	0.00	0.00	0.00	0.00	0.00	1.03	0.00
40S ribosomal protein S7 pseudogene	LOC100768094	100768094	0.00	0.00	0.00	0.00	0.00	0.00	0.00	0.00	0.00	2.32	0.00	0.00
heterogeneous nuclear ribonucleoprotein A1-like	LOC100768095	100768095	0.00	0.00	0.00	0.00	0.00	0.00	0.00	0.00	0.00	0.00	0.00	0.00
SUMO-activating enzyme subunit 2-like	LOC100768101	100768101	2.82	2.93	3.13	3.63	3.36	3.27	147.35	151.90	162.71	179.63	173.88	175.72
T-complex protein 1 subunit zeta pseudogene	LOC100768103	100768103	0.65	0.77	0.50	0.50	0.43	0.47	15.19	10.98	9.94	10.43	9.31	8.41
60 kDa heat shock protein, mitochondrial pseudogene	LOC100768104	100768104	0.00	0.00	0.00	0.00	0.00	0.00	0.00	0.00	0.00	0.00	0.00	0.00
olfactory receptor 151	LOC100768108	100768108	0.00	0.00	0.00	0.00	0.00	0.00	0.00	0.00	0.00	0.00	0.00	0.00
glutathione S-transferase Mu 6	LOC100768110	100768110	95.42	96.95	107.59	115.15	90.43	100.50	3032.84	3083.66	3429.40	3569.47	2823.44	3134.88
olfactory receptor Olfr180-like	LOC100768112	100768112	0.00	0.00	0.00	0.00	0.00	0.00	0.00	0.00	0.00	0.00	0.00	0.00
GTPase ERas	LOC100768116	100768116	0.00	0.00	0.00	0.00	0.00	0.00	0.00	0.00	0.00	0.00	0.00	0.00
centrosomal protein C10orf90 homolog	LOC100768118	100768118	0.00	0.00	0.00	0.00	0.00	0.00	0.76	0.00	0.00	0.00	0.00	0.00

T-cell-interacting, activating receptor on myeloid cells protein 1-like	LOC100768119	100768119	0.00	0.00	0.00	0.00	0.00	0.00	0.00	0.00	0.00	0.00	0.00	0.00
eukaryotic translation initiation factor 1A, Y-chromosomal-like	LOC100768123	100768123	0.00	0.00	0.00	0.00	0.00	0.00	0.00	0.00	0.00	0.00	0.00	0.00
high mobility group protein B1 pseudogene	LOC100768125	100768125	0.00	0.00	0.00	0.00	0.00	0.02	0.00	0.00	0.00	0.00	0.00	0.00
zinc finger protein 431-like	LOC100768137	100768137	0.64	0.64	0.18	0.52	0.15	0.14	6.84	4.58	0.00	6.95	3.10	2.80
ubiquitin-40S ribosomal protein S27a pseudogene	LOC100768140	100768140	15.47	15.20	15.21	9.69	15.43	10.16	231.66	172.03	161.47	114.73	178.02	172.91
cytochrome b-c1 complex subunit 10	LOC100768142	100768142	238.11	202.79	185.41	116.47	85.08	101.54	1958.09	1669.94	1490.50	881.94	673.78	781.38
POU domain, class 5, transcription factor 1-like	LOC100768143	100768143	0.00	0.00	0.00	0.00	0.00	0.09	0.76	6.41	1.24	0.00	2.07	4.67
tripeptidyl-peptidase 1 pseudogene	LOC100768144	100768144	0.55	0.48	0.46	0.50	0.61	0.44	28.86	20.13	31.05	24.34	41.40	30.84
chromosome unknown open reading frame, human C6orf163	LOC100768147	100768147	0.32	0.27	0.26	0.20	0.13	0.16	9.87	8.24	9.94	4.64	5.17	3.74
olfactory receptor 8I2	LOC100768151	100768151	0.00	0.00	0.00	0.00	0.00	0.00	0.00	0.00	0.00	0.00	0.00	0.00
SUMO-conjugating enzyme UBC9-B pseudogene	LOC100768155	100768155	6.24	6.65	5.72	5.10	2.80	3.49	47.09	54.90	48.44	46.36	18.63	28.97
cytokine receptor-like factor 3	LOC100768156	100768156	0.43	0.34	0.27	0.23	0.39	0.31	10.63	8.24	3.73	4.64	7.24	6.54
protein SET-like	LOC100768157	100768157	1.11	1.28	1.07	0.79	0.88	0.32	18.99	27.45	19.87	18.54	21.73	7.48
V-type proton ATPase subunit G 1 pseudogene	LOC100768159	100768159	3.96	3.63	1.90	1.45	2.54	3.01	38.74	37.52	21.12	12.75	27.94	26.17
chromosome unknown open reading frame, human C7orf50	LOC100768160	100768160	24.90	26.02	29.35	27.19	19.76	22.59	1007.91	1076.08	1243.33	1110.25	811.43	920.65
olfactory receptor 12D2-like	LOC100768162	100768162	0.00	0.00	0.00	0.00	0.00	0.00	0.00	0.00	0.00	0.00	0.00	0.00
nuclear RNA export factor 2-like	LOC100768165	100768165	0.11	0.22	0.00	0.00	0.02	0.00	8.35	17.39	0.00	1.16	1.03	0.00
neurotrophin receptor-interacting factor 1-like	LOC100768170	100768170	8.62	8.46	7.72	8.16	8.55	8.03	506.61	488.63	460.81	504.13	524.74	500.05

E3 ubiquitin-protein ligase RNF138	LOC100768178	100768178	0.00	0.00	0.00	0.00	0.00	0.00	0.00	0.00	0.00	0.00	0.00	0.00
FANCD2 opposite strand protein-like	LOC100768181	100768181	0.00	0.00	0.00	0.00	0.00	0.00	0.00	0.00	0.00	0.00	0.00	0.00
[truncated: 391,380 more chars]
